# Transgelin: a new gene involved in LDL endocytosis identified by a genome-wide CRISPR-Cas9 screen

**DOI:** 10.1016/j.jlr.2021.100160

**Published:** 2021-12-10

**Authors:** Diego Lucero, Ozan Dikilitas, Michael M. Mendelson, Zahra Aligabi, Promotto Islam, Edward B. Neufeld, Aruna T. Bansal, Lita A. Freeman, Boris Vaisman, Jingrong Tang, Christian A. Combs, Yuesheng Li, Szilard Voros, Iftikhar J. Kullo, Alan T. Remaley

**Affiliations:** 1 Lipoprotein Metabolism Laboratory, Translational Vascular Medicine Branch, National Heart, Lung, and Blood Institute, National Institutes of Health, Bethesda, MD, USA; 2 Department of Internal Medicine, Mayo Clinic, Rochester, MN, USA; 3 Department of Cardiovascular Medicine, Mayo Clinic, Rochester, MN, USA; 4 Mayo Clinician-Investigator Training Program, Mayo Clinic, Rochester, MN, USA; 5 Department of Cardiology, Boston Children’s Hospital, Harvard Medical School, Boston, MA, USA; 6 Acclarogen Ltd, St John’s Innovation Centre, Cambridge, United Kingdom; 7 NHLBI Light Microscopy Facility, National Institutes of Health, Bethesda, MD, USA; 8 DNA Sequencing and Genomics Core, National Heart, Lung, and Blood Institute, National Institutes of Health, Bethesda, MD, USA; 9 Global Genomics Group, LLC, Midlothian, VA, USA; 10 Gonda Vascular Center, Mayo Clinic, Rochester, MN, USA

**Keywords:** transgelin, LDL, LDL receptor, endocytosis, whole-genome CRISPR-Cas9 screen, cellular LDL uptake, actin-binding protein, HepG2 cells, ASCVD, atherosclerotic cardiovascular disease, CAD, coronary artery disease, CME, clathrin-mediated endocytosis, CRISPR, clustered regularly interspaced short palindromic repeat, FACS, fluorescence-activated cell sorting, FH, familial hypercholesterolemia, GTEx, Genotype-Tissue Expression, GWAS, genome-wide association studies, LDLR, LDL receptor, LFC, log-fold change, mSREBP2, mature or active form of SREBP2, NHLBI, National Heart, Lung and Blood Institute, PCSK, proprotein convertase subtilisin/kexin, pSREBP2, precursor form of SREBP2, qPCR, quantitative PCR, sgRNA, single-guide RNA, SNV, single nucleotide variant, sTfR, soluble transferrin receptor, TAGLN, transgelin, TC, total cholesterol, TG, triglyceride, UKBB, UK Biobank, UTR, untranslated region

## Abstract

A significant proportion of patients with elevated LDL and a clinical presentation of familial hypercholesterolemia do not carry known genetic mutations associated with hypercholesterolemia, such as defects in the LDL receptor. To identify new genes involved in the cellular uptake of LDL, we developed a novel whole-genome clustered regularly interspaced short palindromic repeat-Cas9 KO screen in HepG2 cells. We identified transgelin (TAGLN), an actin-binding protein, as a potentially new gene involved in LDL endocytosis. In silico validation demonstrated that genetically predicted differences in expression of TAGLN in human populations were significantly associated with elevated plasma lipids (triglycerides, total cholesterol, and LDL-C) in the Global Lipids Genetics Consortium and lipid-related phenotypes in the UK Biobank. In biochemical studies, *TAGLN*-KO HepG2 cells showed a reduction in cellular LDL uptake, as measured by flow cytometry. In confocal microscopy imaging, *TAGLN*-KO cells had disrupted actin filaments as well as an accumulation of LDL receptor on their surface because of decreased receptor internalization. Furthermore, *TAGLN*-KO cells exhibited a reduction in total and free cholesterol content, activation of SREBP2, and a compensatory increase in cholesterol biosynthesis. TAGLN deficiency also disrupted the uptake of VLDL and transferrin, other known cargoes for receptors that depend upon clathrin-mediated endocytosis. Our data suggest that TAGLN is a novel factor involved in the actin-dependent phase of clathrin-mediated endocytosis of LDL. The identification of novel genes involved in the endocytic uptake of LDL may improve the diagnosis of hypercholesterolemia and provide future therapeutic targets for the prevention of cardiovascular disease.

Epidemiological and Mendelian randomization studies have clearly demonstrated a causal role of LDL-C in atherosclerotic cardiovascular disease (ASCVD) (1). Patients with genetically elevated LDL-C are at an especially high risk for ASCVD (1) because of their lifelong elevations.

Familial hypercholesterolemia (FH), affecting between 1:250 and 1:300 individuals worldwide, is caused mainly by loss-of-function mutations in LDL receptor (*LDLR*) and less commonly in *APOB* and by gain-of-function mutations in proprotein convertase subtilisin/kexin type 9 (PCSK9) (2). Other rare causative mutations in *STAP1* and *LDLRAP1* have also been described (2); however, a known genetic mutation is not identified in 20–40% of patients with clear clinical criteria for FH (3, 4, 5). In addition to monogenic FH, genome-wide association studies (GWAS) have not completely identified all the contributors to the heritability of circulating LDL-C in the general population, suggesting that additional genes could be involved in LDL plasma clearance.

Recent advances in genome-editing technologies, especially the application of clustered regularly interspaced short palindromic repeat (CRISPR)-Cas9 technology, have allowed great progress in our understanding of gene function in eukaryotic cells (6). A single-guide RNA (sgRNA) recognizes a specific genomic region, directing Cas9 nuclease to perform a double-stranded break in DNA that is then mainly repaired by the imperfect nonhomologous end-joining process, generating random insertions and deletions (indels) at the cleavage site, leading to disruption of gene function (7). Genome-wide pooled CRISPR-Cas9 KO screens have recently been developed to identify new genes involved in different metabolic cellular pathways, such as in cancer drug resistance (8), signal transduction (9), and cell fitness and viability (8, 10).

Our aim was to identify new genes involved in hepatic LDL uptake by applying a whole-genome CRISPR-Cas9 KO screen on a liver cell line (HepG2). In the present study, we identified transgelin (TAGLN), an actin-binding protein, as a novel gene involved in cellular LDL uptake. Furthermore, we found that single nucleotide variants (SNVs) in the human *TAGLN* locus as well as genetically predicted expression levels of *TAGLN* are associated with elevated plasma lipids, thus validating our approach by revealing an important role for TAGLN in human lipoprotein metabolism. Moreover, we go on to demonstrate the cellular mechanism underlying the role of TAGLN in LDL metabolism. Our results potentially have both diagnostic and therapeutic implications for the prevention of ASCVD.

## Materials and methods

### Reagents

Stably Cas9-expressing HepG2 cell line (catalog no.: T3256), lentiviral sgRNA targeting human LDLR (catalog no.: 264181110204), and nonviral plasmids containing sgRNA targeting individual candidate genes were purchased from ABM Goods, Inc (Canada). Whole-genome lentiviral pooled CRISPR-Cas9 KO library (catalog no.: 73178-LV) was obtained from Addgene (Cambridge, MA). Alexa Fluor™ 568 carboxylic acid succinyl ester, Alexa Fluor™ 488-Human Transferrin conjugate (catalog no.: T13342), and DiI-human LDL conjugate (catalog no.: L3482) were obtained from Molecular Probes (Eugene, OR). Primer kits and Master Mix for RT-quantitative PCR (qPCR) were purchased from Applied Biosystems (Foster City, CA). Monensin, sodium salt (catalog no.: M5273), was purchased from Sigma-Aldrich (St. Louis, MO). Anti-LDLR antibody (catalog no.: 3839) was purchased from BioVision (Milpitas, CA), anti-TAGLN antibody (catalog no.: GT336) from Invitrogen (Carlsbad, CA), and anti-PCSK7 antibody (catalog no.: 19346S) from Cell Signaling Technology (Danvers, MA).

### Cell culture

Stably Cas9-expressing HepG2 cells (ABM Goods, Canada; catalog no.: T3256) were cultured in growing media: DMEM supplemented with 100 IU/ml of penicillin G, 100 μg/ml streptomycin, and 10% (v/v) FBS. Cells were incubated in a humidified incubator with 5% CO_2_/95% air and split every 2 or 3 days.

### Preparation and fluorescent labeling of lipoproteins

VLDLs (*d*: 0.940–1.006 g/ml) and LDL (*d*: 1.019–1.064 g/ml) were isolated from healthy human plasma by preparative sequential potassium bromide density gradient ultracentrifugation (330,000 *g*) followed by extensive dialysis at 4°C against PBS to remove potassium bromide. Purified VLDL or LDL (3 mg protein) was then incubated with Alexa Fluor™ 568 carboxylic acid, succinyl ester (Molecular Probes, Eugene, OR), for 1 h at room temperature in the dark. Free unbound dye was separated from lipoprotein-bound dye by preparative fast protein liquid chromatography, using a HiTrap Desalting column (GE Healthcare, Chicago, IL). Labeled lipoproteins were collected in the void volume.

### Generation of LDLR-K HepG2 cells

Stably Cas9-expressing HepG2 cells were transduced at a multiplicity of infection of 1.5 with lentivirus containing an sgRNA targeting human *LDLR* (ABM Goods, Inc, Canada; catalog no.: 264181110204) and a neomycin selection marker. Transduced cells were selected with neomycin (1 mg/ml) for 14 days. After selection, cells were sorted into 96-well plates (one cell per well) in a BD FACSAria™ III Cell Sorter. Clones were expanded, and loss of LDLR was tested at the mRNA and protein levels by RT-qPCR and Western blotting, respectively (supplemental Fig. S1A). In addition, phenotype was confirmed by fluorescent LDL uptake, showing ∼75% reduction in LDL uptake (supplemental Fig. S1B and C).

### Pooled CRISPR-Cas9 KO screen

**Fig. 1:**
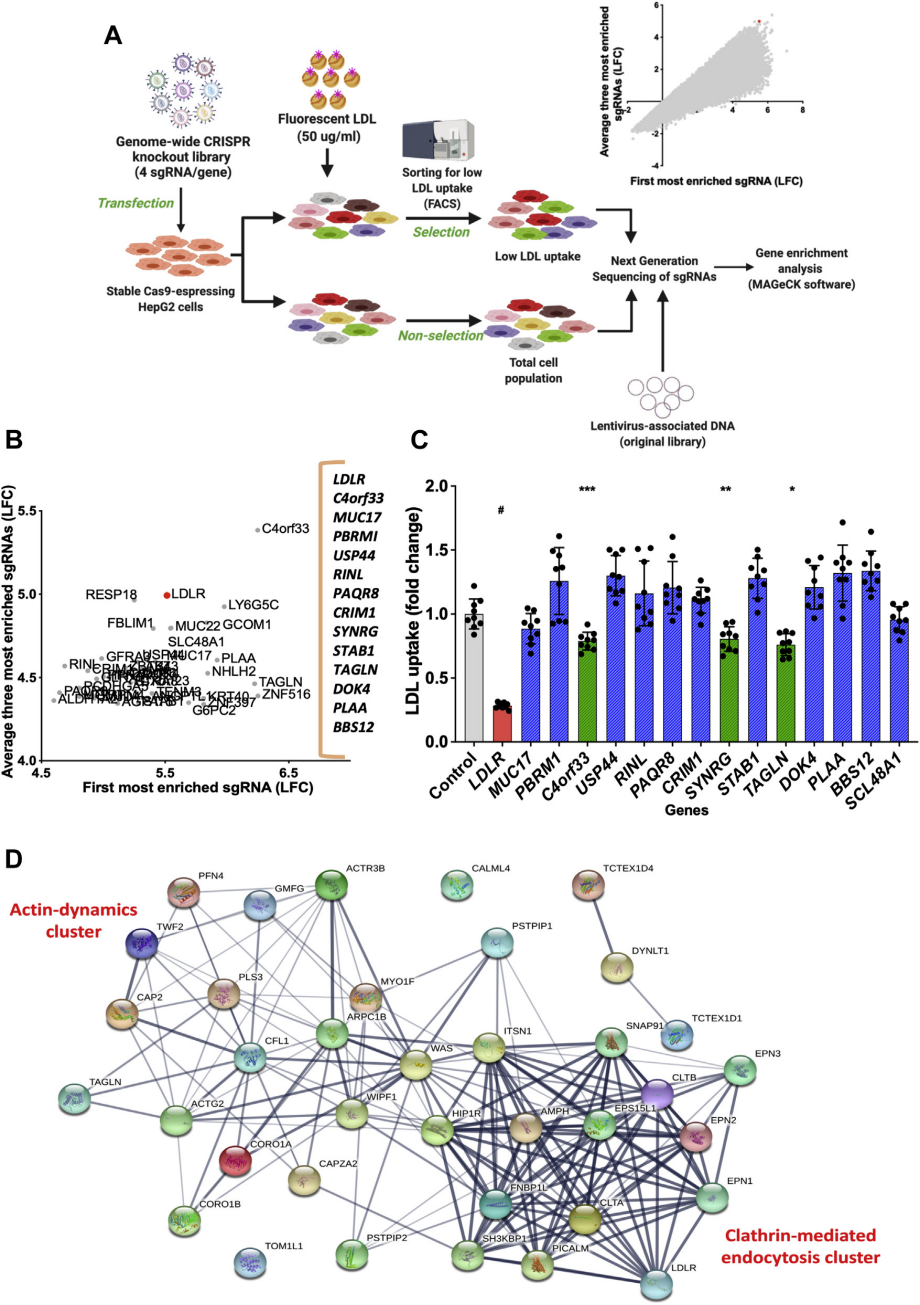
CRISPR-Cas9 screen. A: Schematic for genome-wide CRISPR screen. HepG2 cells with stable Cas9 expression were transformed with lentivirus containing a genome-wide sgRNA library (76,441 sgRNAs, targeting 19,114 genes; four sgRNAs/gene). A fraction of transformed cells were not selected and were frozen for further analysis. The remainder of transformed cells was incubated with 50 μg/ml fluorescent LDL for 4 h and sorted for low LDL uptake by flow cytometry. Next-generation sequencing was used to detect sgRNA in each sample. Enrichment of sgRNA in cells and library was determined with MAGeCK software (13). Genes were ranked by the average of LFC of the three most enriched sgRNA for each gene compared with the nonselected cell population (Image created with BioRender.com). B: Plot of LFC of average for the three best sgRNAs versus LFC of the most enriched single sgRNA of the top ranked 40 genes. Selected candidate genes indicated on the right. C: LDL uptake in CRISPR-generated stable KO clones of the 15 candidates in B. D: Interactome obtained with endocytosis genes that showed at least 1.5 LFC in the genome-wide CRISPR KO screen. Interactome was constructed using STRING database (http://string-db.org/) with moderate confidence (0.40). ^#^*P* < 0.001 versus control. ∗*P* = 0.001 versus control. ∗∗*P* = 0.013 versus control. ∗∗∗*P* = 0.007 versus control. One-way ANOVA.

Stably Cas9-expressing HepG2 cells were transduced with genome-wide lentiviral-pooled CRISPR-Cas9 KO library (Addgene; catalog no.: 73178) (Brunello; catalog no.: 73178-LV) (Addgene, Inc, Watertown, MA) containing 76,441 sgRNAs, targeting 19,114 (four sgRNAs/gene) (11). Approximately 10^8^ cells were transduced with lentiviral library, considering that each sgRNA is represented by 400 cells and at a low multiplicity of infection (∼0.3) to ensure that majority of cells were infected by a single viral particle (12). After overnight infection, media were changed for fresh growing media for 24 h. Transduced cells were then selected in puromycin (4 μg/ml) for 7 days. Finally, cells were incubated 4 h with fluorescent LDL (50 μg/ml) at 37°C, washed, and dissociated by trypsinization. One portion of unsorted cells was kept as nonselected cells, and the rest were sorted according to LDL uptake in a BD FACSAria™ III Cell Sorter (Fig. 1A). Fluorescent LDL signal associated with wild-type and *LDLR*-KO cells was used as reference to establish laser gates. Cells with 5% lower LDL uptake were collected as those with reduced LDL uptake.

After sorting, cells were cultured for 72 h, and genomic DNA was extracted using a Maxwell® RSC Cell DNA Purification Kit (Promega, Madison, WI), following manufacturer's instructions. sgRNAs integrated into the genome were PCR amplified using primers harboring the Illumina TruSeq adapters, P5 and P7. PCR products were separated by preparative gel electrophoresis (1% agarose). Specific bands with the expected amplicon size (354 nt) were purified from gel by Zymoclean Large Fragment DNA Recovery, following manufacturer's instructions. Purified fragments, containing sgRNA sequences, were sequenced in a HiSeq 2500 (Illumina, San Diego, CA). Fastq files were processed for sgRNA and gene enrichment using MAGeCK software (Dana-Farber Cancer Institute) (13). Genes were ranked according to the average log-fold change (LFC) of the best three sgRNAs targeting each gene (Fig. 1B).

### Validation of candidates

Each candidate was validated by generating individual stable KOs and retesting LDL uptake. Stably Cas9-expressing HepG2 cells were transfected with plasmids containing sgRNA sequence, targeting a different sequence than those used in the screen (supplemental Table S3) and GFP as a selection marker, using Lipofectamine 3000 (Thermo Fisher Scientific, Waltham, MA), following manufacturer's instructions. After 72 h, transfected cells (GFP positive) were sorted into 96-well plates, one cell per well. Clones were expanded, and loss of each gene expression was tested by RT-qPCR. Only those clones with significant reduction of mRNA levels were considered for further analysis. Clones for each gene were individually evaluated for LDL uptake as indicated later.

### Association of lipid traits with genetically predicted expression of *TAGLN*, *SYNRG*, and *C4orf33*

We performed association analysis between genetically predicted expression of *TAGLN*, *SYNRG* (synergin gamma), *C4orf33* (chromosome 4 open reading frame 33) and the lipid traits LDL-C, HDL-C, total cholesterol (TC), and triglyceride (TG). We used MASHR-based transcriptome prediction models trained on 49 tissues in the Genotype-Tissue Expression (GTEx), version 8 release data (14), which are available online (http://predictdb.org/). We obtained genome-wide association summary statistics from Global Lipids Genetics Consortium (15) for these lipid traits and imputed them to include all variants present within GTEx reference. We performed tissue-stratified associations using S-PrediXcan (16) for each gene-lipid trait pair and aggregated them in S-MultiXcan (17), which jointly fits the trait on predicted gene expression across multiple tissues to leverage substantial sharing of expression quantitative trait loci between them. Gene-lipid trait associations were deemed significant at a Bonferroni threshold of two-sided *P* value <4.2 × 10^−3^ (0.05/[number of genes tested × number of traits tested]). Harmonization, imputation of summary statistics, and association testing were all performed using the collection of tools available within MetaXcan following their recommended best practice guidelines (https://github.com/hakyimlab/MetaXcan).

### Search for SNV associations at candidate gene loci in previous GWAS

We interrogated previously reported GWAS for SNVs mapped to the three candidate genes identified in the CRISPR screen (*C4orf33*, *SYNRG*, and *TAGLN*) using the National Heart, Lung and Blood Institute (NHLBI) Genome-Wide Repository of Associations Between SNPs and Phenotypes (GRASP, version 2.0.0.0) (18). The GRASP version 2.0.0.0 catalog includes 8,872,472 genotype-phenotype results at a nominal *P* < 0.05 comprising 186,362 unique phenotypes from 177 broad phenotype categories as reported in 2,082 GWAS. Our primary search focused on lipid-related phenotypes and coronary artery disease (CAD). Our secondary search broadened to all reported phenotypes.

### Phenome-wide scan using genetically predicted expression of TAGLN

To evaluate potential pleiotropic effects of genetically predicted expression of *TAGLN*, we used PhenomeXcan database (19, 20), which synthesizes ∼8.8 million variants from GWAS on 4,091 traits with gene expression regulation data from GTEx, version 8 into a transcriptome-wide association resource including 22,255 genes. We filtered precomputed S-MultiXcan association statistics of *TAGLN* to include available clinical phenotypes ascertained through self-report, laboratory measurements, medication data, and electronic health record data in the UK Biobank (UKBB) cohort. We deemed associations significant at a Bonferroni threshold of two-sided *P* value <1 × 10^−5^.

### Cellular lipoprotein uptake

Control, *TAGLN*-KO, and *LDLR*-KO cells were plated in 96-well plates, at a density of 20,000 cells/well, and incubated for 48 h in DMEM supplemented with 100 IU/ml of penicillin G, 100 μg/ml streptomycin, and 10% (v/v) FBS to allow cells to recover the expression of surface receptors. Cells were then incubated with the indicated concentrations of fluorescently labeled LDL or VLDL in serum-free media containing 0.1% BSA (Sigma-Aldrich, St. Louis, MO) for 4 h at 37°C. Cells were then washed with PBS, dissociated by trypsinization, and resuspended in ice-cold PBS containing 0.5% BSA/2.5 μM EDTA. Uptake, proportional to mean fluorescence intensity, was then quantified by fluorescence-activated cell sorting (FACS) flow cytometry in a BD LSRFortessa (BD Biosciences, Franklin Lakes, NJ). Typically, the number of counted events was at least 10,000.

### Cellular transferrin uptake

Control, *TAGLN*-KO, and *LDLR*-KO cells were seeded onto 12-well plates and then incubated for 48 h to allow for recovery of transferrin receptors prior to the experiment. After washing with warm PBS, and incubation with DMEM, containing 0.1% BSA and 25 mM Hepes for 30 min, cells were then incubated with 25 μg/ml transferrin in DMEM, 0.1% BSA, and 25 mM Hepes for 45 min at 37°C. To avoid transferrin resecretion, cells were placed on ice, washed three times with ice-cold PBS, dissociated by brief trypsinization, and then fixed with 4% paraformaldehyde. Uptake, proportional to mean fluorescence intensity, was then quantified by FACS flow cytometry in a BD LSRFortessa. At least 10,000 events were counted.

### Phalloidin staining

Control and *TAGLN*-KO cells were plated in cell culture dishes containing collagen-coated glass coverslip bottoms. After 48 h, cells were fixed with 4% paraformaldehyde, permeabilized with 0.1% Triton X-100, and stained for 30 min with Alexa Fluor® 488 Phalloidin (Molecular Probes, Eugene, OR). Image stacks of cells labeled with Alexa Fluor® 488 Phalloidin were acquired on a Zeiss 880 confocal microscope (Jena, Germany) using a Zeiss 40× Plan-Apochromat objective lens (numerical aperture of 1.3) and 488 nm excitation, the emission bandwidth set to 490–555 nm, the lateral pixel sizes set to 90 nm, and the interslice thickness set to 440 nm. The maximum projection images as displayed in Fig. 3 were generated using the Zeiss Zen software.

### Immunoblot analysis

Protein lysates were prepared from control, *LDLR*-KO, and *TAGLN*-KO cells by incubation with RIPA buffer in the presence of Halt™ protease and Halt™ phosphatase inhibitors (Thermo Fisher Scientific). Prior to protein extraction for SREBP2 expression analysis, cells were incubated with 50 μg/ml of fresh human LDL for 72 h. Western blots were performed as previously described (21) using rabbit anti-human LDLR (1:500) (BioVision, Inc, Milpitas, CA), TAGLN (1:1,000), PCSK7 (1:1,000) (Cell Signaling Technology, Inc, Danvers, MA), SREBP2 (1:1,000) (Abcam, Cambridge, UK), and beta-actin (1:10,000) antibodies (GeneTex, Irvine, CA and Santa Cruz Biotechnology, Dallas, TX). The secondary antibody was goat antirabbit antibody conjugated to HRP (Abcam, Cambridge, UK).

### RNA isolation, expression analysis, and RNA-sequencing

Control, *TAGLN*-KO, and *LDLR*-KO cells were preincubated 72 h in DMEM supplemented with 10% lipoprotein-depleted serum, containing 50 μg/ml of fresh human LDL. Then RNA was extracted using a Qiagen RNeasy® Mini Kit (Qiagen, Hilden, Germany), according to the manufacturer's instructions. Total RNA concentration was measured using a Nanodrop (Thermo Fisher Scientific, St. Louis, MO).

To quantitate expression of selected genes by RT-qPCR, complementary DNA was first synthesized from 200 ng of total RNA, using the QuantiTect Reverse Transcription Kit (Qiagen, Hilden, Germany). RT-qPCR was carried out using TaqMan Gene Expression Assays and TaqMan Master Mix (Applied Biosystems, Foster City, CA). 18S (Hs03003631_g1) was used as an internal control. The primer kits used for RT-qPCR can be found in supplemental Table S4.

### Cellular cholesterol assay

For lipid extraction, 3 × 10^6^ cells were plated into a 10 mm dish. Following overnight incubation for cell adherence, cells were washed twice with warm PBS and preincubated for 72 h in DMEM supplemented with 10% lipoprotein-depleted serum, containing 50 μg/ml of LDL. Cells were then washed twice with ice-cold PBS, and total lipids were extracted into hexane:isopropanol (3:2) for 30 min at room temperature. The extraction procedure was repeated three times. The combined solvents containing extracted lipids were transferred to an Eppendorf tube and dried under nitrogen. The lipid residue was resuspended in 200 μl of 5% Triton X-100. TC was measured by enzymatic colorimetric method (Wako Cholesterol E; Wako, Japan), subtracting a 5% Triton X-100 blank to eliminate nonspecific absorbance. Protein residue, precipitated after lipid extraction, was solubilized with 0.1 N NaOH, and protein concentration was determined using Pierce™ BCA Protein Assay Kit (Thermo Fisher Scientific, Waltham, MA). Cellular cholesterol content data were expressed as microgram cholesterol/milligram of protein.

### Filipin staining

Control, *TAGLN*-KO, and *LDLR*-KO cells were plated in 12-well plates, at a density of 50,000 cells/well, in DMEM supplemented with 100 IU/ml of penicillin G, 100 μg/ml streptomycin, and 10% (v/v) FBS. After overnight incubation for cell adherence and PBS washes, cells were starved in DMEM supplemented with 10% lipoprotein-depleted serum for 24 h and then incubated for 48 h with 50 μg/ml of fresh human LDL in DMEM supplemented with 10% lipoprotein-depleted serum. Next, cells were trypsinized, resuspended in chilled 0.5% BSA in PBS, and fixed with 4% paraformaldehyde. After PBS washes, cells were incubated with 1.5 mg/ml glycine for 10 min at room temperature to reduce autofluorescence and then stained with 125 μg/ml filipin (Polysciences, Inc, Warrington, PA) for 2 h in the dark. After thorough washes with cold PBS, the amount of filipin associated with the cells was quantified by FACS flow cytometry in a BD LSRFortessa using a UV excitation laser (355 nm). The number of counted events was at least 10,000.

### Evaluation of LDL binding sites on cell surface

Control and *TAGLN*-KO cells were plated in glass coverslip-bottomed plastic dishes (Mat Tek Co, Ashland, MA) containing 2.5 × 10^5^ cells/dish and grown in complete growth media for 48 h to allow recovery of LDLR on the cell surface. Cells were then washed twice with ice-cold PBS and incubated for 4 h in prechilled DMEM supplemented with 0.1% BSA at 4°C with 50 μg/ml LDL containing 20% DiI-LDL (Molecular Probes, Eugene, OR). Next, cells were thoroughly washed with ice-cold PBS, and fluorescent LDL bound on the cell surface was imaged with a Zeiss 880 confocal microscope (Jena, Germany) using a Zeiss 40× Plan-Apochromat objective lens (numerical aperture of 1.3). DiI-LDL fluorescence in labeled cells was acquired using 561 nm excitation, an emission bandwidth of 565–700 nm, a pinhole set to 1 AU, a pixel format that varied with optical zoom to provide a lateral pixel size of 110 nm, and an interslice thickness of 640 nm (total slice number/stack varied with cell height). In addition, a portion of the cells, after thorough washing with cold PBS, was incubated at 37°C for 2 h in complete growth media to allow LDL internalization, and then imaged as described previously.

### Monensin treatment and analysis of LDL binding sites on the cell surface

Control and *TAGLN*-KO cells were plated in 96-well plates at a density of 20,000 cells/well and cultured for 48 h to allow expression of LDLR on cell surface to recover. Cells were treated with 30 μM monensin for 0, 15, 30, 60, 90, or 120 min and then treated with DiI-LDL in the cold, as described previously (Evaluation of LDL binding sites on cell surface). Cells were then dissociated with an enzyme-free cell dissociation buffer (Gibco, Gaithersburg, MD) and prepared for flow cytometry. Cell surface-associated fluorescence was quantified by FACS flow cytometry in a BD LSRFortessa. The number of counted events was at least 10,000. The amount of fluorescence associated with cell surface was expressed as percent of the fluorescence associated with nontreated cells (no monensin).

### TAGLN expression in starved and LDL-overloaded HepG2 cells

Wild-type HepG2 cells were plated in 12-well plates, at a density of 50,000 cells/well, in DMEM supplemented with 100 IU/ml of penicillin G, 100 μg/ml streptomycin, and 10% (v/v) FBS. After overnight incubation for cell adherence, cells were incubated for 72 h in DMEM supplemented with 10% lipoprotein-depleted serum either alone (starvation) or containing fresh human LDL: 20 μg/ml (low LDL) or 200 μg/ml (high LDL). Finally, total RNA was extracted from cells, and the mRNA expression levels of *TAGLN* and *LDLR* were determined by RT-qPCR as described above.

### Statistical analysis

Data are presented as mean ± SD. Data distribution was verified by means of Shapiro-Wilk test. All parameters followed a normal distribution, except for precursor form of SREBP2 (pSREBP2) expression levels. Unless otherwise indicated, differences between groups were tested by Student's *t*-test and one-way ANOVA, followed by Dunnett's test, depending on each experiment design, and two-sided *P* < 0.05 were considered statistically significant.

## Results

### Whole-genome CRISPR-Cas9 KO screen of hepatic cellular LDL uptake

To identify new genes involved in LDL uptake in liver cells, we applied a genome-wide KO CRISPR screen on HepG2 cells, using a lentiviral genome-scale CRISPR loss-of-function library (Brunello; Addgene, Watertown, MA), containing 76,441 sgRNAs, targeting 19,114 genes (four sgRNAs/gene), and 1,000 nontargeting control sgRNAs (11). Transduced cells were incubated for 4 h with fluorescent LDL. A fraction of unsorted cells was kept as nonselected cells, whereas the rest were sorted according to the degree of cellular LDL uptake. Cells with less than the fifth percentile of fluorescence LDL uptake were selected. Cut point for cell selection was determined based on the LDL fluorescence in wild-type HepG2 cells and HepG2 cells in which the LDLR was specifically knocked out with an sgRNA targeting *LDLR* (supplemental Fig. S1), which typically led to a ∼75% reduction in LDL uptake. Subsequently, deep sequencing was applied to determine the sgRNA representation in both cell groups (low LDL uptake selected cells and nonselected cell population) and in the original sgRNA library. An overall general outline of our screening approach can be found in Fig. 1A.

If a gene influences a phenotype, its sgRNAs should be among the most enriched in a CRISPR screening of any selected cell (22). Since LDLR clearly affects cellular LDL uptake, we examined the enrichment of its four sgRNAs that were designed in silico in the construction of the original library. Three of the four sgRNAs in the library were among the most enriched (above 95th percentile) in the group of cells with lower LDL uptake (supplemental Fig. S2A). In contrast, for the nonsorted control cells, sgRNAs targeting *LDLR* were randomly distributed among all sgRNAs (supplemental Fig. S2B).

Enriched genes from the screen were ranked by averaging the LFC of their three most enriched sgRNAs to minimize the possible influence of off-target effects on gene classification and because of the known variability in the effectiveness of the individual sgRNAs in the library for their target (supplemental Table S1). Figure 1B shows the plot of average of LFC of the three best sgRNAs for each gene versus the LFC of most enriched sgRNA. *LDLR* was on top of the list (supplemental Fig. S2C). Possible other candidate genes involved in LDL uptake were selected by searching for those genes previously associated with LDL metabolism or were biologically plausible based on their known function. Genes associated with cell growth or apoptosis could potentially be confounders and were not considered. A total of 15 candidate genes were selected for further validation (Fig. 1B) after confirmation of their enrichment in an independent replicate screen (supplemental Fig. S2, D and E).

Cellular uptake of fluorescent LDL was tested by flow cytometry in stable KO cells for each individual candidate gene (Fig. 1C). As expected, *LDLR* showed the most robust effect, with a reduction of approximately 80% in cellular LDL uptake (*P* = 0.0002) when compared with control cells (wild type). Although small reductions were observed for several of the candidate genes, consistent reductions in cellular LDL uptake were only observed for the following three candidate genes: *SYNRG* (*synergin gamma*) (−19%, *P* = 0.013), *C4orf33* (chromosome 4 open reading frame 33) (−21%, *P* = 0.007), and *TAGLN* (−25%, *P* = 0.001).

### In silico validation of lipid phenotypes in human populations

To further validate our in vitro findings, we used a computational approach where we performed an association analysis between genetically predicted expression of these three candidate genes, that is, genetic component of the expression levels predicted using common cis-expression quantitative trait loci, and lipid traits, namely LDL-C, HDL-C, TC, and TG. This was done using the Global Lipids Genetics Consortium GWAS summary statistics (15) and transcriptome prediction models trained on GTEx, version 8 (14) data (see Materials and methods section). Genetically predicted expression levels of *TAGLN* were negatively and significantly associated with TC and LDL-C and TGs at the Bonferroni threshold (*p*_*LDL-C*_ = 2.8 × 10^−5^, *p*_*TC*_ = 1.2 × 10^−19^, and *p*_*TG*_ = 2.0 × 10^−57^), and positively with HDL-C (*p*_*HDL-C*_ = 2.3 × 10^−14^). We did not detect significant associations between *SYNRG* and *C4orf33* for any of the lipid traits examined (*P* > 0.18) (Table 1). In addition, we examined the association of SNV at the three in vitro validated gene loci (*SYNRG*, *C4orf33*, and *TAGLN*) with plasma lipids in large human populations, using summary statistics from GWAS (Table 2). Of our three candidate genes, SNVs mapped in the *TAGLN* locus (rs17120434, rs2075547, rs2269397, rs508487, and rs641620) had significant associations with elevated TG, TC, and LDL-C (Table 2). In addition, three SNVs in the *TAGLN* locus also had reported associations with soluble transferrin receptor (sTfR): rs508487 (*P* = 3 × 10^−20^), rs641620 (*P* = 9.8 × 10^−15^), and rs664971 (*P* = 8.5 × 10^−17^), indicating that, in addition to lipoprotein metabolism, this locus might also be involved in transferrin/iron metabolism. No significant associations were observed for the other two candidate genes for any lipid variable.

**Table 1:** Association of lipid traits with genetically predicted expression of *TAGLN*, *SYNRG*, and *C4orf33*

Lipid Trait	Gene	Gene Band	N_tissues_	N_indep_	*z*-Score, Mean (SD)^a^	*P*^b^
LDL-C	*TAGLN*	11q23.3	47	5	−0.177 (1.844)	**2.8 × 10**^**−5**^
HDL-C	*TAGLN*	11q23.3	47	5	0.082 (4.590)	**2.3 × 10**^**−14**^
TC	*TAGLN*	11q23.3	47	5	−0.545 (3.476)	**1.2 × 10**^**−19**^
TGs	*TAGLN*	11q23.3	47	5	−1.814 (5.529)	**2.0 × 10**^**−57**^
LDL-C	*SYNRG*	17q12	47	3	0.610 (0.695)	0.328
HDL-C	*SYNRG*	17q12	47	3	0.862 (0.438)	0.691
TC	*SYNRG*	17q12	47	3	1.571 (0.284)	0.181
TGs	*SYNRG*	17q12	47	3	0.631 (0.420)	0.770
LDL-C	*C4orf33*	4q28.2	48	3	0.778 (0.685)	0.386
HDL-C	*C4orf33*	4q28.2	48	3	−0.098 (0.482)	0.456
TC	*C4orf33*	4q28.2	48	3	0.367 (0.351)	0.852
TGs	*C4orf33*	4q28.2	48	3	−0.306 (0.666)	0.408

N_tissues_, number of tissues available to S-MultiXcan when computing significance for a gene; N_indep_, number of independent components of variations among N_tissues_.

a Mean *z*-score and its SD among single-tissue S-PrediXcan associations.

b *P* value of S-MultiXcan association, significant associations are bolded.

**Table 2:** Associations of SNVs in *LDLR*, *TAGLN*, *C4orf33*, and *SYNRG* with plasma lipids, obtained in data analysis from previous GWAS

Gene in Screen	SNP	Trait	*P*	Mapped Gene	PMID
*TAGLN*	rs17120434	TGs	9E-11	*TAGLN*/*PCSK7*	23063622
		LDL-C	1.7E-07	*TAGLN*/*PCSK7*	23063622
	rs2075547	TGs	4.8E-09	*TAGLN*/*SIDT2*/*LOC100652768*	20686565
		TC	9E-07	*TAGLN*/*SIDT2*/*LOC100652768*	20686565
	rs2269397	TC	1.2E-06	*TAGLN*/*PCSK7*/*LOC100652768*	20686565
		TGs	0.0047	*TAGLN*/*PCSK7*/*LOC100652768*	20686565
		LDL-C	0.012	*TAGLN*/*PCSK7*/*LOC100652768*	20686565
		CAD	0.019	*TAGLN*/*PCSK7*/*LOC100652768*	23202125
	rs508487	TGs	9.7E-71	*TAGLN*/*PCSK7*	20686565
		TC	5.3E-23	*TAGLN*/*PCSK7*	20686565
		TGs	1.6E-14	*TAGLN*/*PCSK7*	19060906
		TGs	2.2E-10	*TAGLN*/*PCSK7*	21943158
		LDL-C	6.2E-10	*TAGLN*/*PCSK7*	20686565
		CAD	0.000067	*TAGLN*/*PCSK7*	23202125
	rs641620	LDL-C	0.0024	*TAGLN*/*PCSK7*/*LOC100652768*	19060906
		LDL-C	8.8E-07	*TAGLN*/*PCSK7*/*LOC100652768*	20686565
		TC	7.5E-19	*TAGLN*/*PCSK7*/*LOC100652768*	20686565
		TGs	1.8E-30	*TAGLN*/*PCSK7*/*LOC100652768*	20686565
		TGs	8E-10	*TAGLN*/*PCSK7*/*LOC100652768*	19060906
		TGs	5.9E-09	*TAGLN*/*PCSK7*/*LOC100652768*	23063622
*SYNRG*	rs12942520	LDL-C change with statins	0.015	*SYNRG*	20339536
		TC change with statins	0.017	*SYNRG*	20339536
	rs17138637	LDL-C	0.0024	*SYNRG*	20339536
		TC	0.025	*SYNRG*	20339536
	rs17696979	LDL-C	0.002	*SYNRG*	20339536
		TC	0.02	*SYNRG*	20339536
		TC change with statins	0.026	*SYNRG*	20339536
*C4orf33*	rs10518544	TC	0.03	*C4orf33*	20339536
	rs2271570	LDL-C	0.0068	*C4orf33*	20339536
		TC	0.032	*C4orf33*	20339536
	rs2655310	TC	0.032	*C4orf33*	20339536
		TGs	0.034	*C4orf33*	19060906
	rs337276	TC	0.032	*C4orf34*	20339536
		TC	0.033	*C4orf33*	20339536
	rs189236	TC	0.023	*C4orf33*	20339536
	rs13114398	LDL-C	0.022	*C4orf33*	20339536

It is important to note that the *TAGLN* sequence partially overlaps tail to tail with the antisense *PCSK7*, raising the possibility that some of the aforementioned SNVs may be related to *PCSK7*. We, therefore, analyzed whether the sgRNAs targeting *TAGLN* in the library also targeted *PCSK7*. The sgRNAs for *TAGLN* targeted its exons 2 and 3 as well as *PCSK7* on its terminal intron 19 in its 3′-untranslated region (UTR) region (supplemental Fig. S3). In contrast, none of the sgRNAs targeting *PCSK7* were mapped close to the coding region of *TAGLN*. Their targeting sequences are all about 10 kb downstream from the 3′-UTR of *TAGLN* (supplemental Fig. S3). Furthermore, none of these *PCSK7* sgRNAs showed enrichment in the screen (LFC = 3.59; −1.04; −2.50, and −3.078). To generate stable *TAGLN*-KO cells, a fifth independent sgRNA, different to those in the screen library, was used. Its targeted sequence (CACTTCGCGGCTCATGCCAT) maps to *TAGLN* on exon 2 and on *PCSK7* to its terminal intron 19 in the 3′-UTR region. Using this fifth *TAGLN* sgRNA, we found that PCSK7 protein expression was unchanged or, in fact, slightly increased in *TAGLN*-KO cells (supplemental Fig. S4), indicating that the observed reduction in cellular LDL uptake depends upon *TAGLN* and not on *PCSK7*.

Since *TAGLN* was the only one among the three candidates to show significant associations with plasma lipid levels at both the gene-level and SNV-level analyses, we further evaluated its association of genetically predicted expression of *TAGLN* at a phenome-wide scale in the UKBB, using PhenomeXcan database (see Materials and methods section). Below the Bonferroni significance threshold, predicted expression of *TAGLN* was associated with 18 phenotypes in the UKBB. Of the 18 associations, 10 were related to abnormal lipid metabolism and related phenotypes, such as high cholesterol, lipid-lowering medication use, waist circumference, body fat percentage, and body mass index (Table 3). On the other hand, the eight remaining associations were related to hematologic traits, such as platelet count, hematocrit, red blood cell and platelet distribution widths, and eosinophil count (Table 3). These associations suggest that this locus (11q23.3) could in addition affect erythrocytes, beyond just sTfR metabolism.

**Table 3:** Phenome-wide association of genetically predicted expression of *TAGLN* in the UKBB

Phenotype	N_tissues_	N_indep_	Best Sign	Consensus Sign	*P*^a^
Platelet count	47	5	+	+	3.5 × 10^−16^
Platelet distribution width	47	5	−	−	3.5 × 10^−13^
Eosinophil count	47	5	+	−	1.9 × 10^−10^
Self-reported high cholesterol	47	5	−	+	1.3 × 10^−9^
Red blood cell (erythrocyte) distribution width	47	5	+	−	2.8 × 10^−9^
Platelet crit	47	5	+	+	1.4 × 10^−8^
Leg fat percentage (right)	47	5	−	−	5.2 × 10^−8^
Mean platelet (thrombocyte) volume	47	5	−	−	5.9 × 10^−8^
Eosinophil percentage	47	5	+	−	6.5 × 10^−8^
Leg fat percentage (left)	47	5	−	−	1.6 × 10^−7^
Cholesterol-lowering medication use	47	5	−	−	1.0 × 10^−6^
Waist circumference	47	5	−	−	1.1 × 10^−6^
Body fat percentage	47	5	−	−	2.3 × 10^−6^
Leg fat mass (right)	47	5	−	−	2.7 × 10^−6^
Hematocrit percentage	47	5	+	+	3.0 × 10^−6^
Treatment/medication code: fenofibrate	47	5	−	−	3.4 × 10^−6^
BMI	47	5	−	−	4.2 × 10^−6^
Leg fat mass (left)	47	5	−	−	4.5 × 10^−6^

N_tissues_, number of tissues available to S-MultiXcan when computing significance for a gene; N_indep_, number of independent components of variations among N_tissues_; Best sign, the sign of effect of the most significant tissue where “+” (“−”) means higher (lower) expression is associated with higher risk or higher value of the phenotype; Consensus sign, the sign of effect of the consensus among those tissues with *P* value <1 × 10^−4^ where “+“ (“−”) means the same as in the “Best sign” column.

a Obtained using S-MultiXcan on GWAS summary statistics of UKBB phenotypes.

### Role of endocytosis-related genes in LDL uptake

Internalization of LDLR occurs through clathrin-mediated endocytosis (CME) (23), which is a complex process that comprises not only proteins associated with the formation of the clathrin-coated pit but also scission proteins and actin filament-associated proteins (24). We, therefore, investigated whether endocytosis-related genes, in general, were significantly enriched in our screen and consequently associated with reduced cellular LDL uptake in HepG2 cells. We first used the list of endocytosis-related genes compiled by Lacy *et al.* (25), including 105 genes related to each step in the endocytic pathway. Thirty-seven of these genes showed at least 1.5 LFCs in our screen (supplemental Table S2). Figure 1D shows the interactome between these genes and *LDLR*, showing two interconnected clusters: CME and actin dynamics. Numerous studies have demonstrated that the coordinated function of both clusters is essential to ensure LDL internalization (26, 27, 28). Among all endocytosis-related genes, *TAGLN*, however, showed the highest average enrichment (average LFC: 4.39).

### Validation of the functional role of TAGLN in LDL internalization

**Fig. 2:**
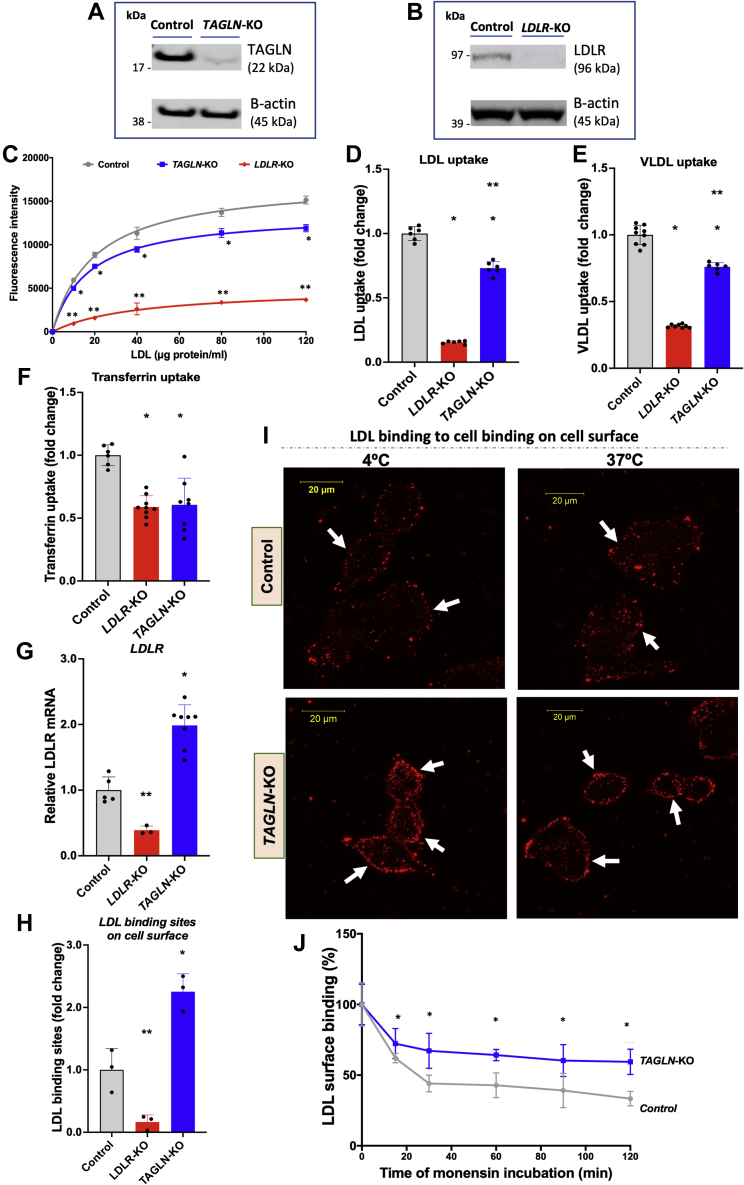
Reduced uptake of LDL, VLDL, and transferrin and reduced LDLR internalization in *TAGLN*-KO cells. A and B: Western blots for TAGLN and LDLR in HepG2 cells, respectively. Cells were transfected with scrambled sgRNA (control) and sgRNA targeting *TAGLN* and *LDLR*. C: Control, *TAGLN*-KO, and *LDLR*-KO HepG2 cells were incubated with increasing concentration of fluorescent LDL. Fluorescence intensity, proportional to LDL internalization, was determined by FACS. D–F, Control, *LDLR*-KO, and *TAGLN*-KO HepG2 cells were incubated with: (D) fluorescent LDL (40 μg/ml); (E) fluorescent VLDL (50 μg/ml); and (F) transferrin (25 μg/ml). Fluorescence intensity, proportional to probe internalization, was determined by FACS. G: mRNA expression levels of LDLR were determined in control, *TAGLN*-KO, and *LDLR*-KO HepG2 cells by RT-PCR. H: Cells were incubated with 10 μg/ml DiI-LDL for 4 h at 4°C, and cell-associated fluorescence was then quantified by FACS. I: Control and *TAGLN*-KO cells were incubated with 10 μg/ml DiI-LDL for 4 h at 4°C and then, after thorough washes with cold PBS, imaged by confocal microscopy (upper and lower left panels). After cold PBS washes, a batch of control and *TAGLN*-KO cells was allowed to warm up to 37°C for 2 h to allow LDL/LDLR internalization and then imaged by confocal microscopy (upper and lower right panels). Images represent the maximum intensity projection of the z-stack and are representative of three independent experiments. White arrows indicate LDL on plasma membrane. J: Control and *TAGLN*-KO cells were incubated with 30 μM monensin at the indicated time points to block LDLR recycling and were then incubated with 10 μg/ml DiI-LDL for 4 h at 4°C. After thorough washes with cold PBS, cells were then dissociated with nonenzymatic buffer, and cell-associated fluorescence was quantified by FACS. ∗*P* < 0.05 versus control. ∗∗*P* < 0.05 versus control and *TAGLN*-KO. Student's *t*-test or one-way ANOVA as appropriate.

To investigate the biochemical role of *TAGLN* in LDL metabolism, we utilized stable clones of *TAGLN*-KO HepG2 cells (Fig. 2A). Positive and negative control cells were also generated by transfecting HepG2 cells with an *LDLR*-targeting sgRNA and a nontargeting sgRNA, respectively (Fig. 2B). We evaluated LDL uptake using a broad concentration range of LDL (from 0 to 120 μg protein of LDL/ml). Cellular LDL uptake in *TAGLN*-KO cells saturated at ∼50 μg/ml of LDL, similar to control cells and to previously reported values for LDL uptake in fibroblasts (29). However, in marked contrast to control cells, LDL uptake in *TAGLN*-KO cells was significantly reduced by ∼30% (Fig. 2C).

Since prior GWAS and our computational work showed associations between *TAGLN* loci and TGs, sTfR, and hematologic traits, we extended our analysis to transferrin and VLDL, the main plasma lipoprotein carrier of TGs. Interestingly, uptake of VLDL and transferrin was also reduced compared with control cells (*P* < 0.04) and to a similar degree as LDL (*P* > 0.12) (Fig. 2D–F). This suggests that *TAGLN* deficiency affects a common mechanism involved in the internalization of these three different cargoes. In contrast, in *LDLR*-KO cells, the defect in uptake was specific for LDL.

To test whether reduced cellular LDL uptake in *TAGLN*-KO cells is due to alterations in *LDLR* expression, we assessed *LDLR* mRNA expression. Surprisingly, *LDLR* mRNA levels were significantly increased by 2-fold in *TAGLN*-KO cells when compared with controls (*P* = 0.0002) (Fig. 2G). This finding suggests that decreased LDL uptake in *TAGLN*-KO cells may be due to less efficient internalization of LDLR, rather than reduced LDLR expression and that the defect in LDL internalization in *TAGLN*-KO is partially compensated by upregulation of the receptor. This would imply that the amount of LDLR on cell surface of *TAGLN*-KO cells would be increased compared with control cells. To test this hypothesis, we incubated cells with fluorescent LDL at low temperature (4°C) to prevent LDL internalization in order to allow visualization of LDL binding sites on the cell surface. Quantification by flow cytometry indicated a 2-fold increase in LDL binding sites on the cell surface of *TAGLN*-KO cells compared with controls (*P* = 0.0001) (Fig. 2H), which correlated with LDLR mRNA levels (Fig. 2G).

Confocal microscopy (Fig. 2I, left panels) confirmed the aforementioned flow cytometry findings. The observed accumulation of fluorescent LDL on the surface of *TAGLN*-KO cells incubated at 4°C is consistent with increased LDLR expression on the plasma membrane. After low-temperature incubation, a portion of cells were warmed to 37°C to allow LDL internalization for 2 h (Fig. 2I, right panels). After this treatment, control cells had reduced LDL fluorescence on the cell surface and increased cytoplasmic DiI fluorescence (Fig. 2I; right, upper panel). In contrast, the amount of LDL fluorescence on the cell surface of *TAGLN*-KO cells (Fig. 2I; right, lower panel) appeared to be unchanged, consistent with impaired LDL internalization. To evaluate LDLR internalization rate, we incubated cells with monensin for different times and then evaluated the remaining LDL binding sites on cell surface by FACS (Fig. 2J). Monensin is known to block LDLR recycling in mammal cells, promoting LDLR disappearance from cell surface (30). Similar to others (31), we found, using FACS, that in control cells, monensin incubation decreased cell surface LDLR by 70%. In contrast, in *TAGLN*-KO cells, loss of surface LDLR was significantly decreased (*P* < 0.03) by the monensin treatment (Fig. 2J). Taken together, these findings indicate that loss of TAGLN expression reduces LDLR internalization, resulting in the retention of LDLR at the plasma membrane.

**Fig. 3:**
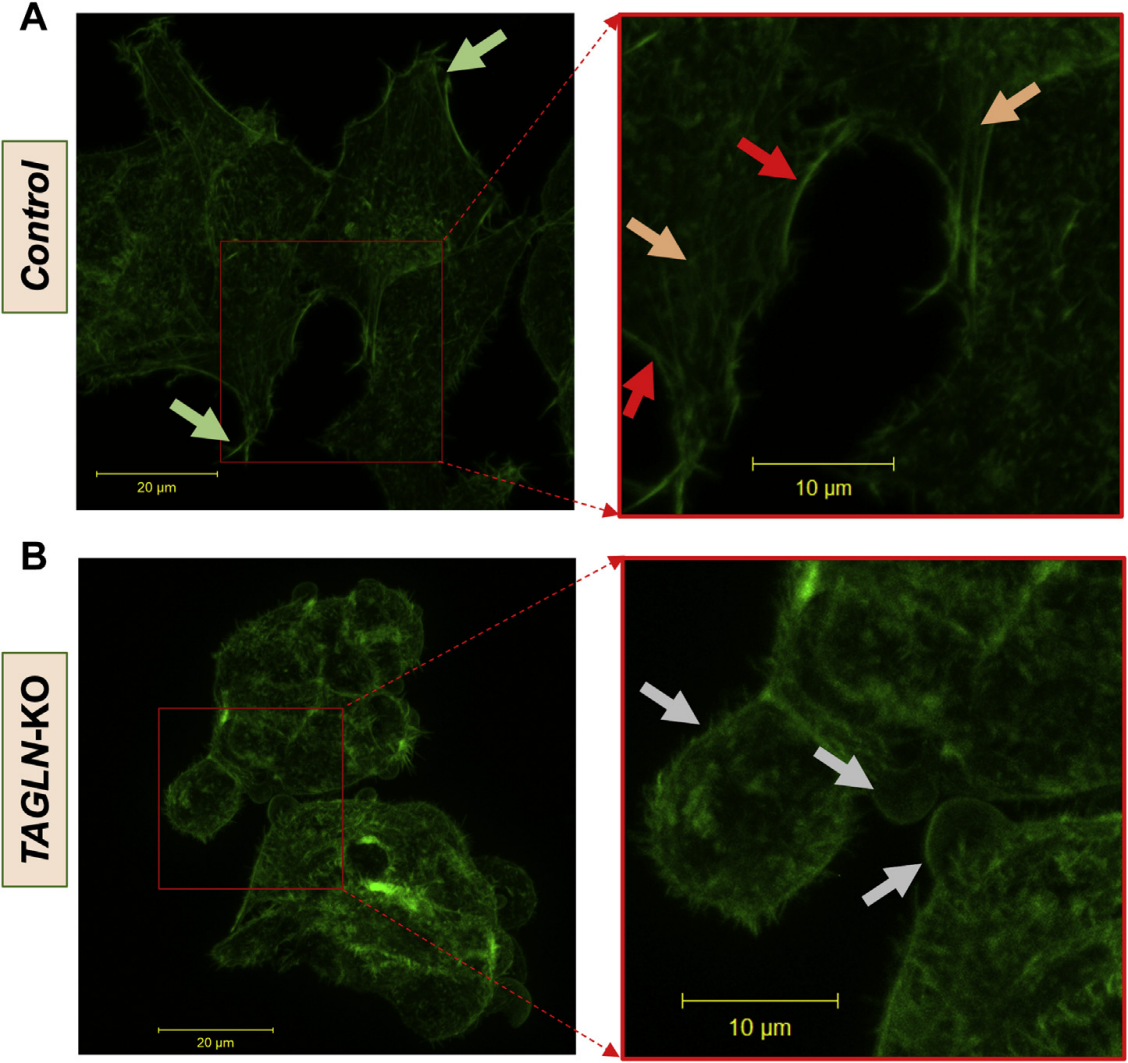
TAGLN deficiency is associated with morphological changes in HepG2 cells, associated with changes in actin cytoskeleton. Control and *TAGLN*-KO cells were plated in cell culture dishes containing collagen-coated glass coverslip bottoms. After 48 h, cells were fixed with 4% paraformaldehyde, permeabilized, and stained with Alexa Fluor® 488 Phalloidin (Molecular Probes, Eugene, OR). Confocal images were acquired as described in the Materials and methods section. Maximum projection images as displayed in the figures were generated using the Zeiss Zen software. A: Control cells, showing characteristic spread shape (green arrows), cortical actin filaments (red arrows), and intracellular actin filaments (orange arrows). B: *TAGLN*-KO cells were round with the presence of blebs (gray arrows). Images represent the maximum intensity projection of the z-stack. The scale bar represents 20 μm.

TAGLN is a cytoskeletal protein that binds to actin, stabilizing actin filament bundles that are critical during the invagination of the endosome (32). Therefore, we analyzed the arrangement of actin filaments in *TAGLN*-KO and control cells by staining cytoplasmic actin filaments with fluorescent phalloidin. Control cells exhibited characteristic cell spreading with linear cytoplasmic and subcortical actin filaments (Fig. 3A). In marked contrast, *TAGLN*-KO cells were rounded, with disorganization of intracellular actin filaments and the presence of blebs lacking subcortical actin (Fig. 3B). These studies demonstrate a pivotal role for TAGLN in stabilizing actin filaments during not only endocytosis but also in maintaining overall normal cell morphology.

**Fig. 4:**
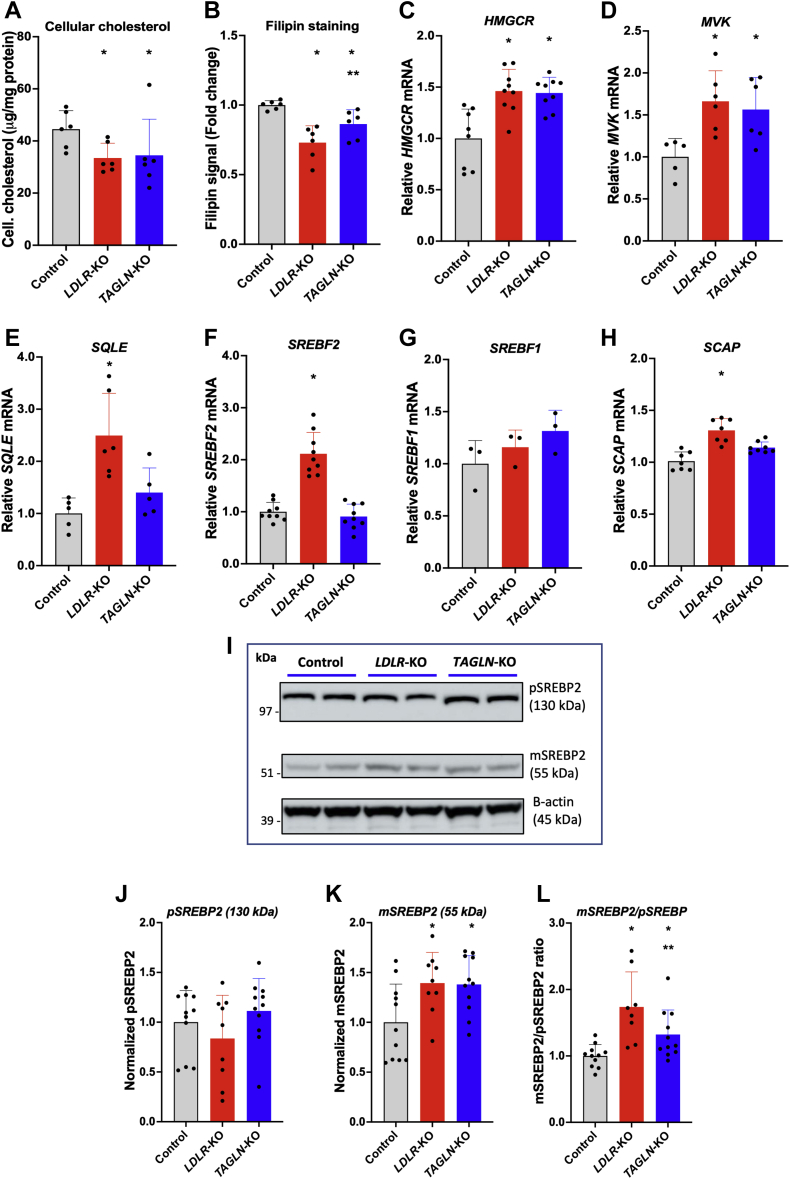
Reduction in the content of cellular total and free cholesterol in *TAGLN*-KO cells induces mRNA expression of cholesterol synthesis enzymes through the activation of SREBP2. A: Control, *LDLR*-KO, and *TAGLN*-KO cells were incubated in serum-free media containing 50 μg/ml LDL for 72 h. Total lipids were extracted with hexane:isopropanol (3:2), and cholesterol was measured by an enzymatic colorimetric assay. B: Control, *LDLR*-KO, and *TAGLN*-KO cells were starved for 24 h and then incubated in serum-free media containing 50 μg/ml LDL for 48 h. Next, cells were fixed and stained with 125 μg/ml Filipin. Cell-associated filipin fluorescence was measured by FACS. C–H: Control, *LDLR*-KO, and *TAGLN*-KO cells were incubated in serum-free media containing 50 μg/ml LDL for 72 h. mRNA expression levels of 3-hydroxy-3-methylglutaryl-CoA reductase (*HMGCR*), mevalonate kinase (*MVK*), squalene monooxygenase (also called squalene epoxidase [*SQLE*]), SREBF chaperone (*SCAP*), *SREBF2*, and *SREBF1* were determined by RT-PCR. Data are shown as fold change. I: Control, *LDLR*-KO, and *TAGLN*-KO cells were incubated in serum-free media containing 50 μg/ml LDL for 72 h. SREBP2 expression was determined by Western blot in cellular protein extracts using an anti-SREBP2 antibody. Beta actin was used as loading control. J: Normalized expression levels of precursor form of SREBP2 (pSREBP2) (126 kDa). Data are shown as fold change. K: Normalized expression levels of mature form of SREBP2 (mSREBP2) (55 kDa). Data are shown as fold change. L: Ratio between mSREBP2 and pSREBP2. Data are shown as fold change. ∗*P* < 0.05 versus control. ∗∗*P* < 0.05 versus *LDLR*-KO. One-way ANOVA. For pSREBP2, Kruskal-Wallis test is used.

Given the importance of LDL internalization in overall cholesterol cellular balance, we examined alterations in several known cholesterol homeostatic pathways in *TAGLN*-KO cells. Cells were first incubated in media containing LDL as the sole extracellular source of cholesterol. Cellular cholesterol content in *TAGLN*-KO cells was about 40% lower compared with controls (*P* < 0.05), which is comparable to the reduced cellular cholesterol content seen in *LDLR*-KO cells (*P* = 0.99) (Fig. 4A). The content of unesterified cholesterol in cellular membranes is a crucial regulator of cellular cholesterol homeostasis (33). Filipin is a naturally fluorescent antibiotic that specifically binds to free cholesterol and has been used to evaluate the cellular-free cholesterol pools and intracellular vesicle trafficking (34). We determined filipin staining fluorescence by flow cytometry to measure cellular-free cholesterol content after incubating cells with LDL as the sole source of cholesterol. As expected, *LDLR*-KO cells showed a decrease in filipin staining (*P* = 0.0004). Although more modest than *LDLR*-KO cells, *TAGLN*-KO cells also showed a reduction in filipin signal compared with control (*P* = 0.0112) (Fig. 4B), consistent with the reduction of LDL uptake. We next evaluated mRNA expression levels of several critical enzymes involved in cholesterol synthesis (Fig. 4). Compared with controls, *TAGLN*-KO cells showed increased expression of two cholesterol biosynthetic enzymes, namely 3-hydroxy-3-methylglutaryl-CoA reductase and mevalonate kinase (*P* < 0.05), comparable to the increased levels seen in *LDLR*-KO cells (*P* > 0.905). In addition, squalene monooxygenase (also called squalene epoxidase; encoded by *SQLE gene*) mRNA levels also showed an increase of 40% in *TAGLN*-KO (*P* = 0.147). Unlike in *LDLR*-KO cells, mRNA levels of *SREBF2* and *SCAP* were not altered in *TAGLN*-KO cells when compared with controls; however, *SREBF1* mRNA levels were increased (*P* = 0.044). Although *TAGLN*-KO cells did not show differences in *SREBF2* mRNA levels with control, TAGLN deficiency may still affect the protein expression and processing of SREBP2, the main regulator of cellular cholesterol homeostasis (35). In our immunoblot analysis, we observed a band of 126 kDa, corresponding to pSREBP2, and a band of 55 kDa, the size of the mature or active form of SREBP2 (mSREBP2) (Fig. 4I). After 72 h of incubation with LDL, no changes were observed in the expression levels of pSREBP2 (126 kDa) in *TAGLN*-KO or *LDLR*-KO cells in comparison to control cells (Fig. 4J); whereas, relative mSREBP2 expression and the mSREBP2/pSREBP2 ratio were increased in *TAGLN*-KO and *LDLR*-KO cells (Fig. 4K, L), suggesting a greater SREBP2 activation. Overall, these findings are consistent with compensatory activation of cholesterol biosynthesis in *TAGLN*-KO cells because of decreased cellular cholesterol.

**Fig. 5:**
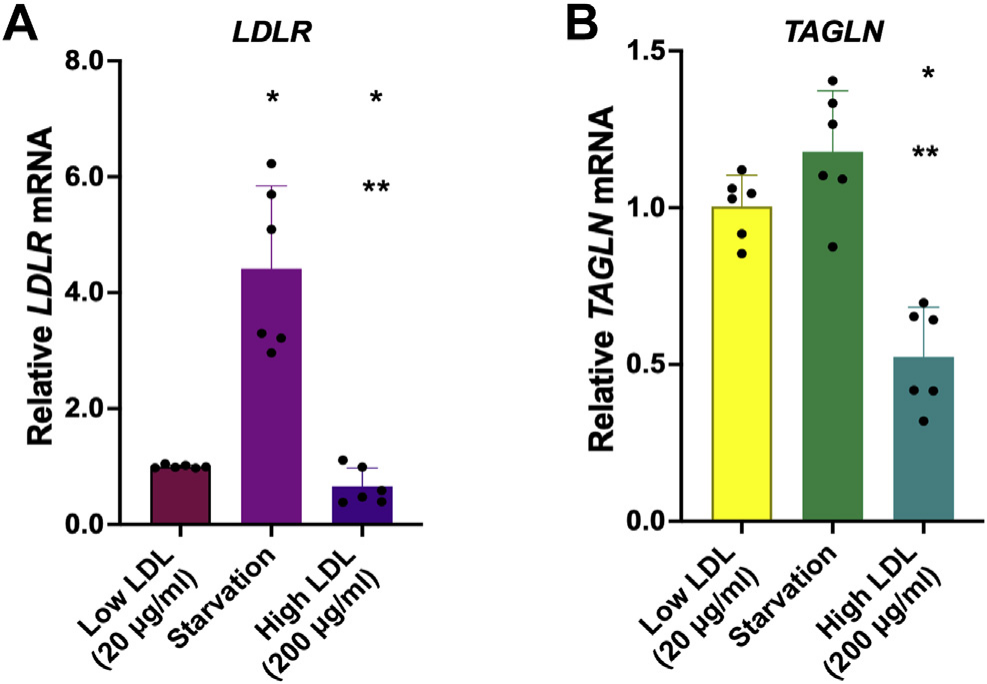
TAGLN expression is increased by starvation and suppressed by LDL overload. Wild-type HepG2 cells were incubated for 72 h in media supplemented with 10% lipoprotein-depleted serum, either alone (starvation) or containing 20 μg/ml of LDL (low LDL) or 200 μg/ml of LDL (high LDL). Next, total RNA was extracted, and *TAGLN* mRNA expression was measured by RT-qPCR. *LDLR* mRNA expression was used as an assay control. A: *LDLR* mRNA levels relative to 18S. B: *TAGLN* mRNA levels relative to 18S. Data are shown in fold change. One-way ANOVA. ∗*P* < 0.05 versus low LDL.∗∗*P* < 0.05 versus starvation.

To investigate whether there is a role of cholesterol homeostasis in regulating TAGLN expression, we evaluated *TAGLN* mRNA levels in wild-type HepG2 cells after starvation and LDL overload. *LDLR* mRNA levels functioned as an internal control of the experiment (Fig. 5A). Interestingly, compared with the cells incubated with low LDL concentrations, cells exposed to high LDL showed lower *TAGLN* mRNA levels (*P* < 0.001), whereas levels of *TAGLN* mRNA in starved cells tended to increase (*P* = 0.078) (Fig. 5B), suggesting that TAGLN expression may be directly or indirectly regulated by cellular cholesterol content.

## Discussion

In the present study, we implemented a new genome-wide CRISPR-Cas9 KO screen in a liver cell line, using cellular LDL uptake as the basis for selection, in order to identify and characterize new genes involved in LDL internalization. We identified *TAGLN*, an actin-binding protein, as a novel gene participating in LDL internalization in liver cells. Furthermore, using different genomic association approaches, we found that common variants at the *TAGLN* locus and genetically predicted expression of *TAGLN* are both associated with elevations in plasma total and LDL-C and TGs, as well as with lipid-related phenotypes, such as body mass index, waist circumference, body fat percentage, dyslipidemia, and lipid-lowering medication use. Finally, our biochemical and cell biology analyses suggest that TAGLN plays a vital role during LDLR internalization, most likely by binding to actin filaments during endocytosis, thereby facilitating LDL uptake and consequently affecting cellular cholesterol homeostasis.

TAGLN is a 22 KDa actin-binding protein, a member of the calponin family, known to stabilize actin filaments (32). In smooth muscle cells, TAGLN is strongly associated with actin filaments and represents one of their earliest differentiation markers (32). In addition, TAGLN dysregulation has been observed in different types of tumors, suggesting a role in tumor proliferation and invasiveness (36, 37). Our CRISPR-Cas9 screen of a liver cell line revealed that TAGLN also plays a role in LDL metabolism. We observed, however, only modest reduction in LDL uptake in *TAGLN*-KO cells, possibly because of compensatory changes. Cholesterol is a critical molecule for cell survival, and low cellular cholesterol content is sensed in the endoplasmic reticulum and induces the cleavage of SREBP2, which stimulates the expression of LDLR and cholesterol synthesis enzymes (33). In *TAGLN*-KO cells incubated in media containing LDL as the exclusive source of extracellular cholesterol, reduced LDL internalization depleted cellular total and free cholesterol content and induced the activation of SREBP2, and therefore, upregulated the expression of cholesterol synthesis enzymes and also LDLR. Nevertheless, increased expression of enzymes involved in cholesterol synthesis was not able to fully compensate for the reduction in cellular cholesterol levels. Moreover, TAGLN expression was influenced by cellular cholesterol load; however, the mechanisms of this regulation remain to be elucidated.

GWAS have identified many possible associations between SNVs and phenotypic traits in large-scale human genetic studies. The associations of SNVs at the *TAGLN* locus (11q23.3) and genetically predicted expression of *TAGLN* with plasma lipids support our in vitro findings of an important physiologic role for this locus in lipoprotein metabolism. Although at lower significance, SNVs at the *TAGLN* locus have also been associated with CAD (38). In a preliminary study, we also observed a reduction of circulating plasma TAGLN, as measured by mass spectrometry, in patients with CAD in comparison to controls (data not shown), but additional studies will be needed to further investigate this issue. Interestingly, TAGLN-deficient mice with an *Apoe*-KO background are reported to develop accelerated atherosclerosis compared with control *Apoe*-KO mice (39). This was attributed to a phenotypic modulation of smooth muscle cells, but no difference in plasma lipids was reported. Because apoE is a major ligand for LDLR, the absence of apoE in this mouse model may have masked the effect of TAGLN on lipid levels.

Cellular LDL uptake, mediated by LDLR, occurs via CME. CME is mediated by a complex network of endocytosis-related proteins that we found is also significantly altered in our screen (LFC > 1.5). The enrichment of the actin-dynamic cluster seen in our screen further highlights the significant role that actin filaments play during CME, particularly at the later stages, before scission of endocytic vesicles (40). Our data suggest that the coordinated function of the endocytic network is necessary for cellular LDL uptake to occur.

**Fig. 6:**
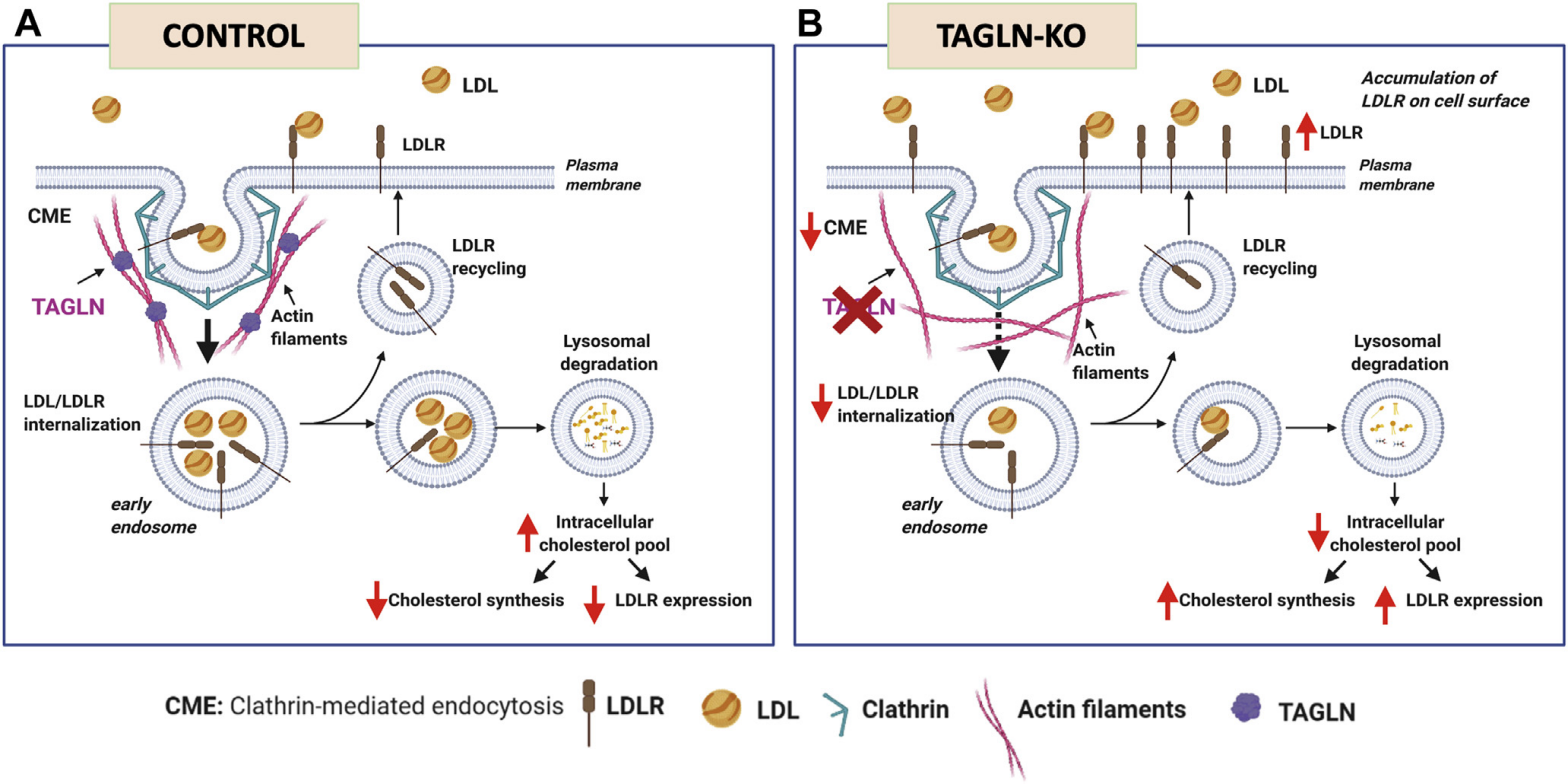
Proposed mechanism for the role of TAGLN during endocytosis of LDL. A: In control cells, LDL binds to its receptor (LDLR), and the complex is internalized by CME. During the actin-dependent phase of CME, TAGLN binds to actin filaments, creating actin filament bundles to support plasma membrane bending before endocytic vesicle scission. In early endosomes, LDLR is recycled to plasma membrane, whereas LDL is directed to lysosomes for degradation. Cholesterol in LDL increases intracellular cholesterol pools, negatively regulating LDLR transcription and suppressing intracellular cholesterol synthesis. B: In *TAGLN*-KO cells, a failure in the formation of actin bundles leads to inefficient CME, reducing the internalization of LDLR. Therefore, cellular LDL is decreased, and LDLR accumulates on plasma membrane. Lower cellular availability of LDL depletes intracellular cholesterol pools and thus induces expression of LDLR and cholesterol synthesis enzymes. The figure was created with BioRender.com.

Actin filaments are thought to generate the force to bend the plasma membrane during endocytosis (41). In this context, the yeast *TAGLN* ortholog, *SCP1*, plays a central role during CME, localizing with actin filaments and supporting membrane invagination (42). We, therefore, hypothesized that, if TAGLN is involved in the actin-dependent phase during endocytosis, internalization of LDLR would be impaired, and therefore, excess LDLR would be retained on the plasma membrane (Fig. 6). Our 4°C LDL binding experiments are consistent with this hypothesis. Furthermore, our monensin experiments demonstrated a decreased rate of internalization of LDLR in *TAGLN*-KO cells. It has previously been demonstrated that *LDLRAP1* deficiency completely blocks LDLR internalization, leading to receptor retention on the cell surface (31). The partial blockage of receptor internalization observed in TAGLN deficiency may be due to functional compensation by other actin-binding proteins, which requires additional investigation. In addition to its importance in CME, TAGLN may have other roles (32). For example, the stabilization of actin filaments of the cytoskeleton by TAGLN likely explains the morphological changes we observed in *TAGLN*-KO cells. Nonetheless, further structural studies will be needed to determine the exact interaction of TAGLN with the LDLR endocytic machinery.

Like LDLR, LRP1, an important hepatic receptor for VLDL, is also internalized by CME (43, 44). In *TAGLN*-KO cells, uptake of LDL and VLDL is equally affected, suggesting that CME is affected at a common step, presumably downstream of the interaction with adaptor proteins (45), likely during the actin-dependent phase of endocytosis. Based on prior GWAS findings, carriers of variants in the *TAGLN* locus would be expected to have not only increased LDL-C but also TGs, which is consistent with our cell culture findings. Patients with this lipid phenotype are often diagnosed as having the familial combined hyperlipidemia. These patients are known to be at a marked increased risk of CAD, but a clear monogenic basis for this disorder is yet to be identified (46). Hypermethylation of *TAGLN* promoter has been shown to decrease TAGLN expression in tumor cells (47). Besides mutations and polymorphisms in *TAGLN*, epigenetic *TAGLN* modifications related to metabolic disorders could, therefore, also be linked to lipid levels.

Another ligand known to undergo CME and showed decreased uptake in our *TAGLN*-KO cells is transferrin. The transferrin receptor is cleaved from the plasma membrane, and its plasma soluble form (sTfR) serves as a marker of iron deficiency (48). Associations reported in previous GWAS showed that different SNVs in *TAGLN* locus were associated with sTfR (49). This finding could also further explain the associations we found between predicted expression levels of TAGLN and various erythrocyte indices in the UKBB. Alterations in TAGLN function leading to destabilization of actin filaments could possibly also be related to red blood cell fragility (50).

Some limitations of the present study should be noted. The large number of cells needed for the whole-genome CRISPR KO screen and the clonal expansion of stable KOs require the use of immortalized cell lines, such as HepG2, which may not always behave the same as primary cells and have a hyperdiploid karyotype (51). HepG2 cells, however, exhibited in our study the expected response to the deletion of LDLR and starvation. HepG2 cells are also one of the most commonly used liver cells in several areas of biomedical research. Nevertheless, further studies with primary liver cells or induced pluripotent stem cells will be needed to further define the role of TAGLN in LDL metabolism and other cell systems. It is also important to note that some of the genes we identified in our KO screen did not show any effect on LDL uptake when tested in individual stable KO cell lines, suggesting that they may be false positives. Applying sequential selections to the sorted cells, also based on LDL internalization, might be helpful to enrich for true positive hits. Our studies with filipin staining measured by flow cytometry only accounted for the total free cholesterol levels in the cells and are insensitive to changes in the free cholesterol distribution in different cellular compartments. Further studies will be needed to elucidate whether TAGLN has a role in vesicle trafficking or in intracellular cholesterol distribution. Finally, because of the broad role of TAGLN in cell function, its association with CAD may be multifactorial, which will likely require future animal studies to sort out.

In summary, using a genome-wide CRISPR-Cas9 KO screen, we identified *TAGLN* as novel gene involved in LDL endocytosis. The human relevance of our in vitro findings was supported by population genetic analysis, demonstrating that SNVs at the *TAGLN* locus and genetically predicted expression of *TAGLN* were associated with plasma lipids and related phenotypes. Furthermore, our biochemical and cell biology data suggest that TAGLN stabilizes actin filaments during the actin-dependent phase of CME, affecting internalization of not only LDLR but also possibly other cargo. We also observed an enrichment of other genes in our screen related to endocytosis, which raises the possibility that polymorphisms in other endocytosis-related genes that may only slightly affect function and are still compatible with life, may also affect plasma LDL levels. The identification of new genetic factors involved in lipoprotein metabolism could eventually reveal new diagnostic markers and therapeutic targets for reducing plasma LDL-C for the prevention of ASCVD.

## Data availability

The authors declare that all the biochemical data supporting the findings of this study are available within the article and its supplemental data files. Data from UKBB is available upon subscription at https://www.ukbiobank.ac.uk/. Transcriptome prediction models in the GTEx, version 8 release data are available online (http://predictdb.org/).

## Supplemental data

This article contains supplemental data.

## Conflict of interest

S. V. salary and significant ownership in Global Genomics Group. M. M. M. is now an employee of Novartis Institutes of Biomedical Research, but he was not an employee when his contribution to the work was conducted. All other authors declare that they have no conflicts of interest with the contents of this article.
