# Supplementary material for: Transgelin: a new gene involved in LDL endocytosis identified by a genome-wide CRISPR-Cas9 screen
Source: J Lipid Res. 2021 Dec 10;63(1):100160. doi: 10.1016/j.jlr.2021.100160 (PMC8953622; doi:10.1016/j.jlr.2021.100160)
Supplement: Supplemental Tables S1–S4 [file mmc1.pdf]

Supplementary Table 1. Whole list of targeted genes (n=19,114) by Brunello genome-wide CRISPR/Cas9 knockout library (Doench, J.G., et al. Nat Biotechnol, 2016). Each gene was targeted by four single guiding RNA (sgRNA). Genes were ranked by the average of the three most enriched sgRNA.

Rank	Gene	Log. Fold Change of sgRNAs				
		1st	2nd	3rd	4th	Average of 3 most enriched
1	*C4orf33*	6.235	5.3319	4.5448	0.17582	5.37
2	*LDLR*	5.5014	4.8066	4.6289	0.86656	4.98
3	*RESP18*	5.2364	5.0818	4.5366	4.4184	4.95
4	*LY6G5C*	5.9644	4.5194	4.2512	-0.13231	4.91
5	*GCOM1*	5.8228	4.5281	4.0937	-1.0997	4.81
6	*MUC22*	5.5336	4.7189	4.0977	4.0116	4.78
7	*SLC48A1*	5.5297	4.5023	4.3131	0.16173	4.78
8	*FBLIM1*	5.3896	4.7012	4.2504	0.58258	4.78
9	*GFRA3*	4.9731	4.8064	4.0208	2.7129	4.60
10	*PLAA*	5.9071	4.3885	3.4763	1.6061	4.59
11	*MUC17*	5.4071	4.3603	3.9755	-1.2817	4.58
12	*USP44*	5.2643	4.2724	4.1975	-0.68741	4.58
13	*RINL*	4.6738	4.5229	4.4673	0.42034	4.55
14	*ZBTB43*	5.1629	4.441	4.0296	-0.31075	4.54
15	*AK7*	5.2913	4.9282	3.4104	-0.99059	4.54
16	*CRIM1*	4.8607	4.573	4.1477	0.062547	4.53
17	*NHLH2*	5.8302	4.0292	3.6763	-1.9259	4.51
18	*C1orf86*	5.1582	4.7753	3.5778	-1.9874	4.50
19	*DOK4*	5.2721	4.2456	3.9867	-0.05054	4.50
20	*PPP1R3D*	4.9881	4.7152	3.7704	3.1948	4.49
21	*IFNA7*	5.0347	4.2577	4.176	0.9264	4.49
22	*GLRX2*	4.9321	4.3587	4.1456	-0.44661	4.48
23	*CXorf23*	5.2504	4.5961	3.5244	-0.09731	4.46
24	*STRA8*	5.2058	4.1191	4.0362	0.14017	4.45
25	*ZNF516*	6.2097	5.7961	1.3418	-2.7736	4.45
26	*PCDHGA5*	4.7926	4.6757	3.8012	0.14337	4.42
27	*PAQR8*	4.6322	4.5894	3.9656	-3.3058	4.40
28	*TENM3*	5.3796	4.223	3.5706	1.4592	4.39
29	*TAGLN*	6.237	3.6013	3.2889	-0.27744	4.38
30	*MGMT*	4.791	4.5744	3.7511	-0.72666	4.37
31	*TRPM4*	4.9192	4.6103	3.5859	-0.72973	4.37
32	*ANGPTL1*	5.3419	4.5496	3.2004	-0.87328	4.36
33	*JMJD4*	4.9126	4.8843	3.2905	-0.99694	4.36
34	*KRT40*	5.7973	4.2595	3.0288	1.1047	4.36
35	*ALDH1A2*	4.5863	4.4708	3.987	0.18682	4.35
36	*G6PC2*	5.6759	4.6681	2.6616	-0.88877	4.34
37	*STAB1*	5.2723	4.6737	3.0566	2.273	4.33
38	*AGPAT5*	5.1024	4.6991	3.1897	2.8237	4.33
39	*ZNF397*	5.798	4.3352	2.844	1.2626	4.33

40	*PNLDC1*	4.4094	4.2965	4.2362	-1.8761	4.31
41	*CYP11B1*	5.1665	4.8214	2.946	-0.64186	4.31
42	*AP1S1*	4.9854	4.4895	3.4433	1.3216	4.31
43	*AHNAK*	5.065	4.2752	3.5771	2.1505	4.31
44	*PPTC7*	5.289	4.1202	3.4983	1.0694	4.30
45	*ALDH6A1*	5.2942	4.3043	3.296	0.37781	4.30
46	*CTRL*	4.4165	4.3068	4.151	-1.0121	4.29
47	*PSPH*	5.28	3.8804	3.6686	3.1005	4.28
48	*PRRC1*	5.0229	4.187	3.6123	2.4157	4.27
49	*TPPP3*	4.365	4.2829	4.1723	-1.8735	4.27
50	*COX19*	5.362	4.157	3.2847	-1.2208	4.27
51	*MYLPF*	4.9827	4.3594	3.459	-1.4473	4.27
52	*FAAH2*	4.923	4.5789	3.2939	2.4812	4.27
53	*SLC9A9*	4.4306	4.3575	4.0066	-1.1439	4.26
54	*TNIP1*	6.1302	5.2052	1.4539	-1.1991	4.26
55	*FUT9*	4.9041	4.7301	3.1435	-0.45029	4.26
56	*CBY3*	4.7575	4.1568	3.8169	-0.56851	4.24
57	*ADAM2*	5.4378	3.912	3.3745	-0.10686	4.24
58	*PLEKHA4*	4.923	4.2332	3.5478	-0.568	4.23
59	*TANC1*	5.084	4.7594	2.8449	-0.75192	4.23
60	*KRTAP10-1*	5.1425	4.102	3.414	0.002584	4.22
61	*FAM228A*	5.2795	4.1275	3.2414	1.2437	4.22
62	*BBS12*	5.5819	4.5587	2.4599	-0.49115	4.20
63	*NPY4R*	5.2821	3.7334	3.5737	3.0819	4.20
64	*TMCC2*	5.0495	4.1247	3.4089	-0.59622	4.19
65	*GALNT11*	5.2184	4.8919	2.4595	0.74648	4.19
66	*PBRM1*	4.6018	4.1477	3.8097	-1.7068	4.19
67	*GDF7*	5.11	4.3092	3.0924	-1.1442	4.17
68	*CNTNAP1*	4.5666	4.5029	3.4359	-1.2505	4.17
69	*NME5*	4.5095	4.2548	3.7389	1.856	4.17
70	*RPL22L1*	5.0493	4.3215	3.1184	0.2484	4.16
71	*KIAA0196*	5.2804	4.6454	2.5203	-2.0952	4.15
72	*IFITM1*	5.8601	4.9036	1.678	-1.8341	4.15
73	*IL13RA1*	4.8932	4.1375	3.4089	-0.83963	4.15
74	*ASB7*	4.8313	3.8641	3.7186	2.4343	4.14
75	*HBE1*	4.8198	4.8095	2.7596	1.7425	4.13
76	*PLIN2*	4.8434	4.295	3.2483	0.12893	4.13
77	*LRRCC1*	4.275	4.1979	3.9093	0.4622	4.13
78	*SAA2*	5.1925	4.3382	2.8332	-0.85194	4.12
79	*CEP70*	4.6155	3.9294	3.8113	3.042	4.12
80	*KRTAP5-2*	5.0238	4.6927	2.6276	-0.79596	4.11
81	*RNF219*	5.104	3.9672	3.2699	0.081756	4.11
82	*TXNL1*	5.0798	3.9237	3.2966	-0.87739	4.10
83	*HSPA1B*	5.5324	4.8918	1.8623	-0.39924	4.10
84	*IGLL5*	5.2724	3.9211	3.0916	-0.67149	4.10
85	*CDH11*	4.4266	4.2785	3.5774	-0.35774	4.09

86	*FERD3L*	4.5885	4.3652	3.3234	0.85755	4.09
87	*MYH2*	4.8283	4.1251	3.3236	-0.91862	4.09
88	*IGDCC4*	4.9772	3.9224	3.3636	1.3734	4.09
89	*CAPN6*	4.78	4.0612	3.4166	2.9261	4.09
90	*SSBP3*	5.7751	4.792	1.6586	-0.86459	4.08
91	*SUMO3*	4.4642	4.4223	3.3347	2.8821	4.07
92	*DIP2C*	4.9133	4.1297	3.1678	2.7009	4.07
93	*DPP6*	4.6649	4.6281	2.8934	0.95594	4.06
94	*RIC1*	4.6196	3.8528	3.705	-0.5204	4.06
95	*VEGFB*	5.8439	4.5779	1.7266	0.77351	4.05
96	*METTL24*	5.5065	3.8359	2.8032	-1.0761	4.05
97	*MAP3K1*	5.3953	4.1643	2.5748	0.94811	4.04
98	*CABLES2*	5.3805	5.1354	1.605	-1.7971	4.04
99	*COL16A1*	5.5757	4.4375	2.1047	-1.0205	4.04
100	*ARMC8*	4.8313	4.7447	2.5174	0.96941	4.03
101	*SOSTDC1*	4.6844	4.4416	2.9632	-0.72975	4.03
102	*STRADB*	4.9394	4.658	2.4819	-1.5552	4.03
103	*HEATR5B*	4.6382	4.2913	3.1445	-0.53356	4.02
104	*SPATA31D1*	4.5179	3.9469	3.6051	-0.10857	4.02
105	*ZNF136*	4.5762	4.0287	3.4192	1.272	4.01
106	*CLEC2D*	4.4325	4.1176	3.4727	0.74176	4.01
107	*CT45A1*	4.018	3.9906			4.00
108	*USF2*	5.0679	4.6309	2.2964	-1.3268	4.00
109	*CXorf38*	5.1174	4.4278	2.4418	-1.1426	4.00
110	*ARFGAP2*	4.701	4.1956	3.0768	-0.67791	3.99
111	*AHSA1*	4.1657	3.9415	3.8603	-0.84501	3.99
112	*AXL*	4.6662	4.4846	2.8061	1.5157	3.99
113	*TMPRSS15*	4.3654	4.273	3.3175	0.14105	3.99
114	*FAM101A*	5.7959	4.7555	1.3953	0.11747	3.98
115	*AASDH*	5.0993	4.1903	2.6394	-2.3805	3.98
116	*NSUN7*	5.2859	5.2711	1.3665	0.42638	3.97
117	*MMP26*	5.0287	4.7725	2.1061	-1.8297	3.97
118	*ZNF248*	4.4461	4.2346	3.2098	2.7641	3.96
119	*AP1S2*	4.3999	4.3914	3.0987	-1.4909	3.96
120	*GTPBP1*	4.9794	4.124	2.7841	-0.78443	3.96
121	*SPSB2*	4.8504	4.3342	2.6717	-1.8275	3.95
122	*OCRL*	5.2781	3.8237	2.7182	-0.83784	3.94
123	*PPP1R3F*	4.6303	4.0558	3.1321	-0.99508	3.94
124	*SEC63*	5.3842	3.572	2.8606	-1.1576	3.94
125	*MREG*	5.4869	3.3486	2.9801	-1.8404	3.94
126	*CYP4A22*	4.5208	3.9189	3.3729	-1.2114	3.94
127	*KCND1*	5.4908	4.6852	1.6078	-2.8003	3.93
128	*AHR*	4.4197	3.8891	3.4736	2.8619	3.93
129	*OR5H2*	4.9978	4.0713	2.7131	2.4183	3.93
130	*UCK2*	4.1184	4.0061	3.6492	-1.4873	3.92
131	*NOS1*	4.0834	3.9341	3.7502	-0.05655	3.92

132	*ZFR*	4.6188	4.4014	2.7454	-1.7963	3.92
133	*FAM216B*	4.2992	4.0052	3.4435	3.42	3.92
134	*HIST1H1C*	5.7892	4.9451	1.0131	-1.344	3.92
135	*SLC45A1*	5.6736	3.4294	2.6299	0.12004	3.91
136	*AMDHD1*	5.293	3.226	3.2135	-0.33363	3.91
137	*KCNA1*	4.3689	4.3376	3.0146	-2.1807	3.91
138	*TM6SF2*	4.9793	4.1965	2.5354	-0.17591	3.90
139	*MAD2L1BP*	4.4302	4.0635	3.2022	0.95622	3.90
140	*RSPO4*	4.4567	4.0237	3.2144	2.396	3.90
141	*CASP4*	4.9336	3.8257	2.9318	2.0211	3.90
142	*MFAP2*	4.4461	4.0781	3.1603	-1.1361	3.89
143	*ISL1*	5.7569	3.4745	2.4393	1.7808	3.89
144	*CLEC5A*	4.4479	3.6258	3.5949	-0.07487	3.89
145	*THY1*	5.0076	4.0083	2.6419	-0.24912	3.89
146	*LOC101927844*	5.4512	3.9484	2.2578	-0.19834	3.89
147	*RBM3*	4.2465	4.0488	3.3606	-1.4234	3.89
148	*NKX6-2*	4.3644	3.7146	3.5738	-0.68299	3.88
149	*TRIM38*	4.1516	4.0803	3.4139	3.2508	3.88
150	*CCT6B*	4.2109	3.7778	3.6524	-0.14448	3.88
151	*MYOM1*	5.5102	3.7391	2.3786	-0.42567	3.88
152	*CPA5*	4.4892	3.9364	3.2005	-0.21175	3.88
153	*EHMT1*	4.3348	3.7943	3.4818	2.5072	3.87
154	*TMEM71*	4.4579	4.1817	2.9647	-1.9713	3.87
155	*FAM58A*	4.0679	4.0608	3.4473	-0.12333	3.86
156	*ATP6V1G3*	4.6434	4.3525	2.58	-1.5817	3.86
157	*ZNF391*	4.7901	3.3956	3.3877	-1.1945	3.86
158	*ALG8*	3.9836	3.9737	3.6145	-0.72391	3.86
159	*SLC38A9*	4.4199	3.7376	3.3995	-0.44318	3.85
160	*OR4F6*	5.094	4.1351	2.3263	1.0816	3.85
161	*UIMC1*	4.5458	3.7623	3.2464	0.40295	3.85
162	*CDYL*	5.4713	5.29	0.78595	-2.6062	3.85
163	*BOD1L2*	4.4663	3.5464	3.5338	-0.05622	3.85
164	*CLUL1*	4.4669	4.3251	2.7544	-1.7024	3.85
165	*ARHGEF12*	4.9361	3.5427	3.0669	-0.45569	3.85
166	*C20orf62*	4.423	4.2043	2.9021	-2.0198	3.84
167	*UBE2E2*	5.2963	4.6647	1.5677	-1.7908	3.84
168	*SULT1A2*	4.3832	3.8205	3.3235	1.6898	3.84
169	*RECQL*	5.2763	3.8791	2.3699	-2.2223	3.84
170	*SON*	4.3024	4.0271	3.1834	-1.695	3.84
171	*ZNF729*	4.168	3.892	3.4315	-1.5343	3.83
172	*FAM156B*	4.9578	4.4239	2.1077	0.58702	3.83
173	*ADAMTS4*	4.1236	3.9162	3.4493	-1.1492	3.83
174	*MLLT4*	5.5172	2.9989	2.9656	0.47288	3.83
175	*SPRN*	5.0432	4.0957	2.3399	2.3384	3.83
176	*RAP2A*	4.1536	3.9169	3.3811	1.0712	3.82
177	*SLC32A1*	5.1432	4.3006	2.0075	0.035082	3.82

178	*ADAMTS8*	4.1335	3.8093	3.4675	2.8758	3.80
179	*RHOH*	5.6095	4.1861	1.6102	-1.5268	3.80
180	*BBOX1*	3.9436	3.9193	3.5388	3.3045	3.80
181	*ANKS1A*	4.3916	4.0973	2.8972	1.5482	3.80
182	*C1orf158*	4.3761	3.8949	3.1098	1.2217	3.79
183	*TESC*	4.3804	3.8566	3.1384	0.60831	3.79
184	*SENP5*	5.0025	4.9347	1.4368	-0.3797	3.79
185	*STAC*	4.2766	4.2572	2.8283	2.1212	3.79
186	*FEM1A*	4.7649	4.3984	2.1879	0.89897	3.78
187	*GSTM1*	4.4145	4.1794	2.7196	1.812	3.77
188	*LILRA1*	4.1735	4.0122	3.117	-0.20462	3.77
189	*DCD*	4.298	3.7879	3.2083	-1.6	3.76
190	*CELSR1*	4.2185	3.7462	3.317	-1.5118	3.76
191	*ARL1*	5.4235	4.7955	1.056	-1.9203	3.76
192	*OR4X1*	4.3538	4.1593	2.759	-1.6187	3.76
193	*ATP6V1G2*	4.8136	4.3466	2.1101	-1.3542	3.76
194	*MRC1*	4.4094	3.9597	2.8946	1.9157	3.75
195	*SMAD2*	4.0393	3.959	3.2221	0.71975	3.74
196	*ZNF529*	5.4944	4.8033	0.92106	0.63291	3.74
197	*RAB8B*	4.1232	4.0052	3.09	0.3977	3.74
198	*NAA20*	4.2891	3.6187	3.3063	-0.49045	3.74
199	*OTOA*	5.44	2.9777	2.7866	-1.2975	3.73
200	*PIDD1*	4.6579	3.3479	3.1883	1.5642	3.73
201	*CREB3L3*	4.3092	3.6519	3.2262	-1.267	3.73
202	*C3orf36*	3.9935	3.87	3.3111	-0.83436	3.72
203	*C1orf115*	4.3825	4.1988	2.5899	1.9494	3.72
204	*MANBA*	5.0396	3.0953	3.0308	-1.3526	3.72
205	*LAPTM4A*	5.1802	4.9113	1.0692	-0.02799	3.72
206	*COL9A1*	5.3821	4.0285	1.7477	0.71692	3.72
207	*RAB39B*	5.1753	4.2734	1.7078	0.063013	3.72
208	*FASTKD1*	4.335	3.6384	3.1682	1.2526	3.71
209	*CDKN2B*	5.423	3.2153	2.5005	1.6142	3.71
210	*FNBP1L*	4.7845	4.4528	1.9014	-1.3291	3.71
211	*FOXA3*	4.1915	3.6112	3.3321	2.1941	3.71
212	*CLEC10A*	5.2276	3.257	2.6471	0.39232	3.71
213	*ZSCAN21*	3.8366	3.7805	3.5098	3.0648	3.71
214	*ZNF382*	4.8677	3.8527	2.4057	0.25548	3.71
215	*H2AFV*	4.352	3.813	2.9518	-0.5961	3.71
216	*ZC3H8*	5.3525	3.0794	2.6805	-0.55074	3.70
217	*PCSK6*	3.9129	3.6088	3.589	-0.00112	3.70
218	*KLHL31*	5.0027	4.0619	2.0352	-0.30887	3.70
219	*GAL3ST3*	4.6246	3.3763	3.0901	-2.3118	3.70
220	*ERICH4*	5.0037	4.1723	1.9112	0.87484	3.70
221	*GCH1*	4.7787	4.1711	2.1356	-1.8238	3.70
222	*ADI1*	5.389	4.6667	1.028	-0.57304	3.69
223	*LRWD1*	3.9191	3.6535	3.5056	1.6397	3.69

224	*PCDH9*	5.1805	3.5709	2.323	-0.84337	3.69
225	*MIA3*	5.9246	4.3478	0.79784	0.64504	3.69
226	*GABRB1*	4.0898	3.9709	3.0058	2.1828	3.69
227	*CHRM1*	4.6908	4.4163	1.9553	-0.32327	3.69
228	*ASCL5*	3.7728	3.7511	3.5125	0.11917	3.68
229	*CHRM2*	4.461	4.4196	2.1556	0.25134	3.68
230	*MED12L*	4.1437	3.6606	3.2281	0.039331	3.68
231	*ZBTB46*	3.8267	3.6601	3.5449	-1.5719	3.68
232	*ZBTB16*	5.0189	4.084	1.9159	-0.86412	3.67
233	*HIST2H4A*	3.8861	3.6594	3.4722	1.7769	3.67
234	*KLF5*	4.4647	3.5953	2.9534	-0.89822	3.67
235	*OR2Y1*	4.6846	3.3511	2.959	0.036582	3.66
236	*HIST1H2BF*	5.0862	3.4436	2.4432	-0.39171	3.66
237	*DYNC1I2*	5.2307	3.0128	2.724	0.13241	3.66
238	*ZNF276*	4.0256	3.8674	3.0729	-0.43305	3.66
239	*DNM3*	3.9728	3.7264	3.2612	-0.28961	3.65
240	*SPRED3*	4.2299	3.4113	3.318	-1.9519	3.65
241	*H1FOO*	4.7805	4.1854	1.9932	-1.0419	3.65
242	*ZNF554*	4.7511	4.0173	2.1899	-1.8106	3.65
243	*CYR61*	4.2363	3.8182	2.9024	0.51492	3.65
244	*SULT1E1*	4.8399	4.0104	2.106	-2.1321	3.65
245	*PROCA1*	4.0883	3.6076	3.2362	0.83433	3.64
246	*PPIP5K2*	4.7936	3.6488	2.4825	-0.39192	3.64
247	*LRCH2*	4.9332	4.5919	1.3994	0.81245	3.64
248	*ROBO1*	4.5119	3.4547	2.9576	-2.3487	3.64
249	*TRIM73*	4.2895	4.1971	2.4359	2.0096	3.64
250	*TMEM2*	4.9873	3.1194	2.8112	-1.0898	3.64
251	*SPHK1*	3.799	3.7064	3.4112	-1.4064	3.64
252	*ZAR1L*	4.3078	3.8102	2.7961	-0.76621	3.64
253	*SYNRG*	5.6145	5.3887	-0.09054	-1.3114	3.64
254	*TMTC2*	4.367	3.6014	2.944	-1.6302	3.64
255	*MYO1G*	4.6967	3.397	2.8173	0.38387	3.64
256	*MPRIP*	4.6676	3.3311	2.9095	0.018783	3.64
257	*LMOD2*	4.4455	3.2719	3.1848	-0.06266	3.63
258	*IL1RAPL1*	4.475	3.2449	3.1819	1.3901	3.63
259	*SAMSN1*	5.3784	4.4674	1.0462	-1.165	3.63
260	*GNS*	5.1842	3.1029	2.6005	-2.0725	3.63
261	*SLC7A1*	4.5507	3.4575	2.8704	-1.0702	3.63
262	*IGFL2*	5.4422	4.8722	0.55279	-1.0582	3.62
263	*MDH1B*	4.5141	3.2375	3.1125	1.5365	3.62
264	*BTNL3*	4.5465	3.576	2.7413	2.6827	3.62
265	*KNOP1*	5.0373	3.0412	2.7814	-0.73565	3.62
266	*PGM1*	5.7068	4.614	0.53673	0.43624	3.62
267	*PCDHGB2*	3.9716	3.8068	3.0783	-0.42697	3.62
268	*TMEM128*	4.9902	4.7318	1.1321	0.87572	3.62
269	*KCNC3*	4.3413	3.9014	2.6035	0.14329	3.62

270	*ELOVL4*	5.4319	3.6107	1.8024	-0.89866	3.62
271	*ICAM2*	4.6549	4.3302	1.8562	-1.5382	3.61
272	*LPPR2*	5.3461	3.6675	1.8267	-0.93489	3.61
273	*RHOXF2*	4.4241	3.2598	3.148	-0.69626	3.61
274	*HES1*	5.7656	2.862	2.204	-0.90229	3.61
275	*TBX1*	5.186	3.1215	2.5213	2.4144	3.61
276	*DEFB126*	4.5878	4.4239	1.8139	-0.13094	3.61
277	*SEC14L1*	4.1372	3.3929	3.2857	-0.2889	3.61
278	*PTN*	4.9353	2.9729	2.8993	2.6056	3.60
279	*LBP*	3.844	3.5308	3.4316	-1.7134	3.60
280	*HIST1H1T*	5.7922	5.5653	-0.55554	-0.96661	3.60
281	*EFHB*	4.5488	3.6679	2.5799	0.15458	3.60
282	*TAS2R41*	4.8047	3.3964	2.5809	-1.2202	3.59
283	*OVCH1*	4.4431	3.197	3.1383	2.0964	3.59
284	*BSPH1*	5.183	4.2216	1.3728	0.19797	3.59
285	*TMCO6*	4.5116	3.6107	2.6474	-1.3242	3.59
286	*LUC7L2*	3.8557	3.46	3.4525	1.106	3.59
287	*TCEAL1*	4.1337	3.5769	3.0565	1.5708	3.59
288	*MPHOSPH6*	4.9843	2.8934	2.8759	1.4317	3.58
289	*ARVCF*	4.6714	4.0747	2.0064	-1.6711	3.58
290	*SYTL1*	4.2648	3.5297	2.9502	1.2424	3.58
291	*RCBTB1*	4.2788	3.6181	2.8445	-0.9703	3.58
292	*GOLGA7B*	5.0016	4.2615	1.475	-0.33174	3.58
293	*TSSC1*	4.8976	3.3711	2.4632	-1.1585	3.58
294	*TNFAIP6*	5.5855	3.1045	2.0334	0.15554	3.57
295	*SPARCL1*	5.491	3.0741	2.1558	-0.56769	3.57
296	*TUBE1*	4.3943	3.8627	2.4574	0.53549	3.57
297	*LIPM*	4.1984	3.7047	2.8089	2.5867	3.57
298	*BEX5*	4.3026	3.6585	2.7465	1.0399	3.57
299	*PPP4R4*	4.8962	3.7814	2.0278	1.7767	3.57
300	*MMACHC*	4.0969	4.0409	2.5612	-0.03924	3.57
301	*CABLES1*	5.4833	4.2573	0.95819	-0.07166	3.57
302	*C9orf172*	4.8349	4.4789	1.3831	-0.45786	3.57
303	*MUC12*	5.2903	3.0405	2.3622	-3.3136	3.56
304	*HOXB6*	4.2078	3.6954	2.7859	-0.40396	3.56
305	*LGALS12*	5.4889	2.6488	2.5486	-0.93727	3.56
306	*SCN8A*	4.3176	4.1588	2.2096	0.064672	3.56
307	*SRI*	4.6347	4.3797	1.6714	0.3432	3.56
308	*MAATS1*	4.7805	4.3135	1.5802	1.059	3.56
309	*IFNA4*	3.7072	3.4927	3.4699	0.00091	3.56
310	*GPR75-ASB3*	5.4004	3.009	2.2593	0.16815	3.56
311	*DEFB1*	4.4269	3.8116	2.43	-1.5059	3.56
312	*SCG3*	4.1382	3.4448	3.0824	-0.39494	3.56
313	*ESPNL*	4.4426	3.3266	2.8864	-0.28752	3.55
314	*SEPHS1*	4.4256	3.8935	2.3315	-1.4171	3.55
315	*ARHGEF10L*	4.2295	3.8801	2.54	0.6301	3.55

316	*SERPINA9*	3.9031	3.5319	3.2091	0.39144	3.55
317	*GNG7*	4.9265	3.9092	1.8058	-0.84409	3.55
318	*TBC1D24*	4.4056	4.2296	2.0037	-0.22016	3.55
319	*HTR1A*	4.6258	4.1067	1.9024	0.31525	3.54
320	*DAZL*	5.0951	4.1225	1.4081	0.4969	3.54
321	*GAN*	5.2823	4.1078	1.2332	-1.8478	3.54
322	*TCTN2*	4.0188	3.4492	3.1446	-0.21159	3.54
323	*IGSF11*	3.9609	3.6296	3.015	0.27793	3.54
324	*TMEM14B*	4.8303	3.5921	2.1817	-0.20928	3.53
325	*RNF183*	5.2532	4.9204	0.4304	-1.5369	3.53
326	*C4orf51*	4.8541	2.8851	2.8644	0.15299	3.53
327	*TGFB3*	5.5284	3.4284	1.6434	-2.5153	3.53
328	*MAGIX*	4.6406	3.2931	2.6604	2.5944	3.53
329	*SLC9A5*	4.334	3.3023	2.9545	-2.817	3.53
330	*TMEM192*	4.4851	3.5966	2.5083	0.80493	3.53
331	*SPAG11A*	4.9042	4.7886	0.89563	0.52995	3.53
332	*PGF*	4.2615	3.2359	3.0814	1.7287	3.53
333	*VSIG10*	3.6207	3.4832	3.4738	-0.93506	3.53
334	*CC2D1A*	4.14	4.0393	2.3906	-1.3766	3.52
335	*TCEAL2*	5.241	5.2207	0.10802	-1.6075	3.52
336	*RWDD3*	5.0796	3.0775	2.4112	0.11365	3.52
337	*SLC25A31*	5.6173	2.8497	2.0999	-1.3443	3.52
338	*ZNF577*	5.7169	4.1666	0.68288	-1.1046	3.52
339	*ONECUT1*	4.8599	2.9245	2.7796	0.31049	3.52
340	*IRX4*	5.465	4.9693	0.12418	-0.40692	3.52
341	*CA2*	4.2584	4.1241	2.1746	2.1307	3.52
342	*DYNLT1*	4.0375	3.9498	2.5672	-0.51673	3.52
343	*C17orf112*	3.666	3.6207	3.2676	2.9027	3.52
344	*LECT1*	4.2856	3.6107	2.6565	-0.45973	3.52
345	*ASAP1*	4.9689	3.8067	1.7754	0.11212	3.52
346	*SLFN12L*	5.5471	4.1127	0.88736	-1.5128	3.52
347	*PPP1R3C*	5.2374	3.2357	2.0712	0.40775	3.51
348	*ZNF821*	5.5966	5.3648	-0.4191	-1.9305	3.51
349	*TSTD2*	4.1312	3.491	2.9114	-1.7931	3.51
350	*TPSG1*	4.6074	4.2194	1.7054	-1.0599	3.51
351	*OPALIN*	4.6143	4.519	1.3985	-0.4082	3.51
352	*XAGE1B*	4.1691	3.6874	2.6737	-0.0401	3.51
353	*NDFIP2*	5.025	3.9994	1.4987	-0.781	3.51
354	*ATAD3A*	5.3535	5.3378	-0.17042	-1.916	3.51
355	*HTN1*	4.4866	4.1614	1.8721	1.3968	3.51
356	*TGM3*	4.7319	3.2125	2.5724	-2.0375	3.51
357	*OAZ2*	3.6803	3.6654	3.1599	0.93382	3.50
358	*CRTC1*	4.4597	3.1697	2.8706	-2.2078	3.50
359	*ZNF202*	5.2272	3.8658	1.4017	-1.3287	3.50
360	*PAPSS2*	3.8417	3.4444	3.2021	-2.3517	3.50
361	*TECPR2*	4.5128	3.3211	2.6542	-1.072	3.50

362	*CLEC18B*	4.5869	3.3354	2.563	-1.0901	3.50
363	*C18orf42*	4.5756	3.1114	2.7965	1.7997	3.49
364	*ERVMER34-1*	5.382	3.8141	1.2837	-0.53713	3.49
365	*NOMO1*	4.8256	4.386	1.2617	-0.8715	3.49
366	*PDP1*	4.7326	3.0177	2.7217	2.1548	3.49
367	*PARPBP*	3.8042	3.6469	3.0196	-1.6917	3.49
368	*MPZ*	4.1294	3.2878	3.0487	0.28201	3.49
369	*WAS*	3.9411	3.684	2.8318	-1.8157	3.49
370	*GABBR2*	4.5656	4.0415	1.8477	-1.536	3.48
371	*FAM198B*	4.5412	3.5444	2.3691	0.14895	3.48
372	*RCN2*	4.8834	3.0374	2.5337	0.033945	3.48
373	*DNAH10*	5.1493	4.4813	0.81863	-2.6476	3.48
374	*CCNG2*	4.87	2.8532	2.7256	-2.7274	3.48
375	*BLOC1S2*	5.4031	3.0709	1.9674	0.26985	3.48
376	*ARPC1B*	4.9398	4.5636	0.9361	0.67857	3.48
377	*TRIM39*	4.8098	4.1512	1.4756	-0.72877	3.48
378	*G3BP2*	4.8203	4.5125	1.0997	-1.2411	3.48
379	*ZNF383*	4.526	3.4762	2.4296	-1.6033	3.48
380	*HACD4*	4.6296	4.4224	1.3782	-2.0593	3.48
381	*MAPK14*	3.8394	3.5603	3.027	0.94996	3.48
382	*KMT2C*	3.9948	3.7761	2.6518	-0.71919	3.47
383	*ASGR1*	4.3733	3.1633	2.8828	-0.93727	3.47
384	*PCDHGB4*	4.5118	4.178	1.727	0.24364	3.47
385	*LCE2C*	4.4069	4.2412	1.7679	-0.60604	3.47
386	*CNTROB*	4.8781	4.8066	0.73023	0.035999	3.47
387	*UBB*	4.6628	4.3789	1.3663	1.0461	3.47
388	*CPNE8*	4.3609	3.2918	2.7534	1.2786	3.47
389	*TRIL*	5.4459	3.3632	1.5937	-0.82366	3.47
390	*CASQ2*	5.4479	3.3253	1.6268	-0.16451	3.47
391	*B9D2*	3.971	3.2944	3.1323	0.92068	3.47
392	*OPN1MW*	4.6396	4.2761	1.4705	-0.8202	3.46
393	*FAM78A*	4.0901	3.2149	3.0805	0.078506	3.46
394	*ANKRD9*	4.7555	3.2698	2.3533	0.90985	3.46
395	*PDCL2*	3.8122	3.8116	2.7543	-0.22609	3.46
396	*CCR7*	4.167	3.232	2.9789	0.17412	3.46
397	*MATN2*	4.7509	3.7009	1.9241	-0.51539	3.46
398	*SCIMP*	4.2289	3.7073	2.437	0.62977	3.46
399	*DMRTC2*	4.5366	2.9572	2.8786	0.30333	3.46
400	*SNX1*	5.2903	5.0169	0.06449	-1.3275	3.46
401	*UBP1*	4.8801	2.9887	2.5028	-1.1325	3.46
402	*ADSSL1*	4.7243	3.1956	2.4508	-1.1904	3.46
403	*TMEM64*	4.7121	3.9613	1.6907	0.57975	3.45
404	*NMNAT3*	4.6235	3.6173	2.1189	1.6763	3.45
405	*WFDC3*	4.6284	3.1583	2.572	0.54388	3.45
406	*ERICH6B*	3.9651	3.3815	3.0116	0.060481	3.45
407	*KRTAP9-8*	4.3765	3.5604	2.4204	0.18521	3.45

408	*GYPA*	5.1039	3.868	1.383	-0.86517	3.45
409	*PLCD3*	4.9021	4.3707	1.0767	-0.29602	3.45
410	*DIABLO*	4.7667	4.401	1.1801	-0.08298	3.45
411	*GFRAL*	4.6572	4.331	1.3531	-0.45965	3.45
412	*OR56A3*	4.4217	4.1164	1.7937	-1.8366	3.44
413	*TSACC*	5.7141	2.8503	1.7668	-0.01483	3.44
414	*CNN2*	4.1443	3.7894	2.3959	0.57904	3.44
415	*GLIS1*	4.0373	3.5865	2.7007	0.088476	3.44
416	*OR4C6*	4.4219	3.2883	2.6115	2.0609	3.44
417	*TMEM114*	4.1892	3.6122	2.5185	-0.0994	3.44
418	*GPR151*	4.6739	3.3977	2.2425	0.58233	3.44
419	*CD28*	4.6614	3.8878	1.7601	-2.0218	3.44
420	*OR2W1*	4.3767	3.0244	2.9062	2.1939	3.44
421	*SORCS1*	4.9176	3.1063	2.2786	1.4651	3.43
422	*RNF165*	4.8326	3.7422	1.7229	-2.0483	3.43
423	*MAB21L2*	4.982	4.9344	0.37779	-1.5611	3.43
424	*TXNIP*	4.2879	3.2374	2.7616	1.2519	3.43
425	*IRS4*	4.4587	3.5932	2.2348	-2.4386	3.43
426	*PDE11A*	5.0447	2.9212	2.3196	-0.96407	3.43
427	*FXYD6-FXYD2*	5.0169	4.1943	1.0686	-0.41131	3.43
428	*PRR18*	5.3369	4.0594	0.87366	-0.46956	3.42
429	*RLBP1*	5.6371	3.2202	1.4107	0.028035	3.42
430	*SNAI2*	3.9655	3.6686	2.626	-1.0216	3.42
431	*PPP1R2*	5.1235	4.5649	0.57144	-2.5478	3.42
432	*CRCT1*	4.763	2.9658	2.5235	0.67866	3.42
433	*PRSS33*	4.9082	3.611	1.7296	0.41418	3.42
434	*CERS6*	4.8599	3.6508	1.737	-1.8212	3.42
435	*ZNF107*	4.1654	3.7886	2.2926	-0.64519	3.42
436	*MOB3A*	4.9509	2.7863	2.5076	1.1202	3.41
437	*C1orf112*	3.988	3.3943	2.861	-0.32552	3.41
438	*TMEM72*	4.1953	3.637	2.4095	-1.3955	3.41
439	*OR10W1*	4.5597	4.515	1.1657	0.1562	3.41
440	*RSBN1L*	4.3282	3.0782	2.8327	-0.85586	3.41
441	*CARD17*	4.8431	4.358	1.0267	0.27203	3.41
442	*NPFFR1*	3.6895	3.3285	3.2075	0.091234	3.41
443	*KPNA5*	4.1496	3.0925	2.9817	-0.78313	3.41
444	*TDRD7*	4.913	4.8798	0.42976	0.42427	3.41
445	*ZNF230*	4.2575	3.224	2.7345	-0.30038	3.41
446	*TTC12*	4.364	4.0653	1.781	-0.49034	3.40
447	*GCNT7*	5.5777	4.675	-0.04444	-1.7188	3.40
448	*UBQLNL*	4.9301	3.9683	1.3018	0.74561	3.40
449	*PBDC1*	4.0363	3.2874	2.8708	0.88384	3.40
450	*ZNF845*	4.8514	3.1987	2.1383	1.5933	3.40
451	*DCLRE1C*	4.1249	3.8065	2.2551	-2.5478	3.40
452	*ZNF787*	4.3755	3.3253	2.4828	-1.9305	3.39
453	*KIF20B*	4.5971	3.3886	2.1931	-1.8963	3.39

454	*C4orf45*	5.2605	4.2683	0.64613	-0.86793	3.39
455	*LRRC75A*	4.0025	3.1671	3.0021	-0.46711	3.39
456	*GAMT*	4.3099	2.9835	2.8774	-1.3324	3.39
457	*A3GALT2*	4.5561	3.2834	2.33	-0.87642	3.39
458	*HMG20A*	4.6504	3.1866	2.3316	-0.45484	3.39
459	*ZNF184*	4.7927	2.938	2.437	1.9554	3.39
460	*YWHAQ*	3.7551	3.4334	2.9785	-0.31091	3.39
461	*AMFR*	3.9535	3.3416	2.8716	1.3708	3.39
462	*KRTAP25-1*	5.3975	4.2581	0.51092	0.091323	3.39
463	*OXTR*	3.7562	3.3206	3.0843	0.62615	3.39
464	*SDR16C5*	5.9577	4.4047	-0.2075	-1.5869	3.38
465	*DGKZ*	4.1375	3.0879	2.9287	0.60294	3.38
466	*TNF*	4.8549	3.6501	1.649	-0.87837	3.38
467	*CELF2*	3.8446	3.5552	2.7539	-0.05571	3.38
468	*STEAP2*	4.6743	3.0299	2.4472	1.4592	3.38
469	*MPP4*	4.3579	3.6616	2.1307	-1.7876	3.38
470	*GFOD1*	4.3358	3.3435	2.4703	-2.7705	3.38
471	*ASIC3*	4.7748	4.2708	1.1012	0.12142	3.38
472	*ABCB10*	3.98	3.4024	2.7595	0.91511	3.38
473	*TNS2*	4.3989	3.8706	1.8701	0.659	3.38
474	*SPINK13*	4.9793	4.0475	1.1118	-2.075	3.38
475	*KDELC1*	4.3553	3.8651	1.9162	-1.066	3.38
476	*KIAA1671*	3.6139	3.5007	3.0089	0.70341	3.37
477	*DUSP10*	3.8784	3.3466	2.8978	-1.2246	3.37
478	*CLEC19A*	4.3877	3.9243	1.81	-1.4078	3.37
479	*DMTN*	4.333	2.9399	2.8465	-1.4894	3.37
480	*EVC2*	4.9032	3.7159	1.4974	0.012816	3.37
481	*TRIM72*	5.0085	3.9247	1.1803	-0.30611	3.37
482	*C3orf56*	4.9806	4.3031	0.82809	-0.59634	3.37
483	*RAP1GDS1*	4.3923	3.3654	2.3435	-0.06975	3.37
484	*SPI1*	4.5384	3.833	1.7288	0.45098	3.37
485	*SLC25A35*	4.1665	3.5818	2.3501	-1.0315	3.37
486	*CMA1*	4.5607	3.2519	2.2836	0.20268	3.37
487	*COG5*	5.7012	3.534	0.85953	0.8042	3.36
488	*APBA3*	4.2746	3.8926	1.9262	-0.63643	3.36
489	*PPP1R14D*	4.2865	3.2395	2.5647	-2.2443	3.36
490	*ATXN3L*	4.6471	4.162	1.2802	0.62467	3.36
491	*SHD*	3.9758	3.2267	2.8772	-0.79108	3.36
492	*CACNA1I*	5.02	4.1806	0.87562	-1.3496	3.36
493	*OR5T2*	4.8094	3.6908	1.5757	0.2573	3.36
494	*ASRGL1*	5.7501	4.6803	-0.3579	-0.73668	3.36
495	*WFDC5*	4.5484	3.0884	2.4353	-1.0564	3.36
496	*COX7B2*	4.1424	3.8727	2.0544	-1.274	3.36
497	*HSPA4*	3.7336	3.1775	3.1576	2.8072	3.36
498	*TLR1*	4.125	3.2235	2.7131	-1.1442	3.35
499	*COX8C*	4.1417	3.086	2.8338	0.37321	3.35

500	*OLFML1*	3.9896	3.8161	2.2492	-1.1704	3.35
501	*TMEM107*	4.6486	3.8119	1.5906	0.093927	3.35
502	*DAZ4*	5.5221	3.9229	0.60282	-0.85018	3.35
503	*RAB5B*	4.3906	3.9136	1.7421	-0.39079	3.35
504	*ZNF793*	4.2827	3.9447	1.8173	0.25544	3.35
505	*TPRG1*	4.4291	3.8882	1.7254	-0.59195	3.35
506	*AFAP1L2*	3.9265	3.2234	2.8902	-1.1178	3.35
507	*KLHL13*	3.9574	3.1534	2.9219	-1.0088	3.34
508	*LELP1*	4.2185	3.5843	2.2209	-0.62792	3.34
509	*HSFX2*	5.2203	4.6666	0.13639	-0.45989	3.34
510	*NPTX2*	4.9264	3.3902	1.7046	0.026096	3.34
511	*TMPO*	3.814	3.4658	2.7367	1.2343	3.34
512	*ATP11AUN*	4.2564	4.1914	1.5628	0.42428	3.34
513	*TIA1*	4.5357	4.0745	1.3997	0.45049	3.34
514	*KIAA0408*	4.5899	3.1701	2.2416	1.5006	3.33
515	*FKBP3*	3.9381	3.4222	2.6404	-1.6731	3.33
516	*NKD1*	5.3084	4.453	0.23777	-0.37913	3.33
517	*SEMG1*	3.8335	3.1662	2.9991	1.5464	3.33
518	*APCS*	4.1429	4.1266	1.7222	-0.89438	3.33
519	*CDCA7L*	4.4685	4.4277	1.0949	-0.95747	3.33
520	*HTR1E*	4.1004	3.2762	2.611	0.47111	3.33
521	*GGA1*	4.8399	3.2619	1.8838	-2.0351	3.33
522	*RANBP3*	5.8102	5.0392	-0.87056	-2.2094	3.33
523	*C22orf31*	5.4644	4.7075	-0.19735	-1.127	3.32
524	*MFSD4*	5.0982	4.7578	0.11456	-2.3222	3.32
525	*KRT76*	5.016	3.7102	1.2372	-1.5908	3.32
526	*PTPRM*	4.1377	2.9762	2.8491	2.1758	3.32
527	*FAM50B*	4.3775	3.683	1.9024	-1.2864	3.32
528	*DYRK2*	5.2386	4.4146	0.3084	0.13194	3.32
529	*PSMB10*	4.5592	4.1688	1.2241	-0.44353	3.32
530	*UPK3BL*	4.8198	2.9035	2.2222	0.009975	3.32
531	*NKX3-1*	4.4489	2.8255	2.671	-1.1012	3.32
532	*ZNF613*	4.8253	3.7249	1.3938	-1.2719	3.31
533	*ZNF428*	4.011	3.4784	2.4525	-1.4983	3.31
534	*ANGPTL6*	4.9233	4.4037	0.61081	-1.0997	3.31
535	*FEZF1*	5.2374	4.2681	0.4314	0.37368	3.31
536	*MPPED2*	3.8424	3.84	2.2498	-1.265	3.31
537	*CACYBP*	4.8715	4.3186	0.7417	0.22581	3.31
538	*PITHD1*	5.165	4.2773	0.48089	-0.59272	3.31
539	*TMEM189-UBE2V1*	5.0669	4.8015	0.05283	-1.0196	3.31
540	*TRIB3*	4.0959	3.4799	2.3421	-0.24661	3.31
541	*ANKAR*	5.0443	4.069	0.80282	-2.2811	3.31
542	*LOC728392*	4.8604	3.8314	1.2226	-0.63643	3.30
543	*DCAF8L2*	4.2346	3.8883	1.7899	-0.688	3.30
544	*PEX5*	4.5729	2.7409	2.596	-1.984	3.30
545	*LIMS1*	4.2568	4.1034	1.5444	-0.80727	3.30

546	*AP4B1*	4.9076	3.4323	1.5625	-0.38408	3.30
547	*C11orf70*	4.0095	3.2264	2.6664	-0.15263	3.30
548	*SLC35A1*	4.0941	3.4657	2.337	1.0432	3.30
549	*KLRK1*	4.8391	4.7262	0.32263	-0.1693	3.30
550	*ZP1*	4.3732	4.2485	1.2651	-0.27982	3.30
551	*RASA1*	5.8683	2.9668	1.0516	-1.1333	3.30
552	*FAM46A*	5.1808	4.6675	0.03698	-0.54934	3.30
553	*GAST*	3.8258	3.1351	2.9218	-0.3952	3.29
554	*CHL1*	5.5151	3.4005	0.96163	-0.46724	3.29
555	*MEF2BNB*	4.3904	2.9164	2.5698	-1.245	3.29
556	*MIER3*	4.5912	3.229	2.0507	1.115	3.29
557	*TMEM115*	3.7939	3.5932	2.4817	2.0159	3.29
558	*EDEM3*	3.6995	3.2694	2.8988	2.7863	3.29
559	*TINAGL1*	5.7078	2.921	1.2369	-1.843	3.29
560	*C18orf54*	3.7974	3.6777	2.39	0.60022	3.29
561	*MARCH1*	5.2726	2.8595	1.7325	-1.8714	3.29
562	*GJA10*	3.906	3.3301	2.6195	0.17066	3.29
563	*ARSB*	5.3191	3.7608	0.77552	0.20444	3.29
564	*ZNF782*	4.5716	4.0396	1.2421	0.57978	3.28
565	*DNAJC24*	3.4565	3.2579	3.1369	-2.2886	3.28
566	*RAB33A*	3.6235	3.4513	2.7742	0.10609	3.28
567	*OR3A1*	5.042	3.63	1.1743	-0.9304	3.28
568	*OR10K1*	5.095	4.069	0.6814	-0.59793	3.28
569	*CEP57L1*	3.9672	3.6915	2.1863	-1.3604	3.28
570	*FCRL3*	4.6302	3.2367	1.9779	1.4641	3.28
571	*BICD1*	3.6054	3.4529	2.7864	-1.4241	3.28
572	*OR2H1*	4.9022	4.2807	0.66176	-1.6711	3.28
573	*KLRG2*	4.5627	3.3939	1.8841	0.073736	3.28
574	*FXYD2*	4.257	2.9635	2.6179	2.1489	3.28
575	*B3GALT4*	4.6916	4.2893	0.85669	-0.85059	3.28
576	*YIPF4*	3.6153	3.2059	3.0128	-0.67352	3.28
577	*MRPL16*	4.8699	2.7984	2.163	0.31617	3.28
578	*CFDP1*	4.2156	4.0723	1.538	0.19565	3.28
579	*UGT2A3*	4.2256	3.6261	1.9701	-0.15279	3.27
580	*F2RL2*	5.2369	3.5203	1.064	-1.0212	3.27
581	*TSHZ3*	3.6564	3.4497	2.7138	0.94387	3.27
582	*XKRY2*	4.6635	3.5162	1.6348	-0.43473	3.27
583	*MACC1*	3.516	3.3543	2.9436	-1.8686	3.27
584	*CLIP4*	4.8399	3.0531	1.9205	0.33336	3.27
585	*SLC25A36*	3.625	3.2768	2.9114	0.17861	3.27
586	*CRIPT*	5.4516	4.612	-0.2505	-5.5233	3.27
587	*TCEAL5*	4.6371	3.2702	1.8981	-0.2697	3.27
588	*TBX19*	4.7427	4.7321	0.32923	-0.72102	3.27
589	*COG2*	5.235	4.9924	-0.42353	-0.90386	3.27
590	*FAM103A1*	5.265	2.7754	1.7625	-2.0249	3.27
591	*LOC643802*	5.1526	3.262	1.3877	-0.59696	3.27

592	*RELN*	4.2261	3.5155	2.0581	0.37386	3.27
593	*NLRC4*	4.5434	4.3368	0.91849	-1.4112	3.27
594	*ECHS1*	4.0681	3.1444	2.5855	-2.2943	3.27
595	*PYROXD2*	5.0498	2.9665	1.7799	0.66509	3.27
596	*KCNK5*	4.3411	3.4486	2.0044	0.52216	3.26
597	*CXCL9*	4.7472	3.5447	1.502	0.49055	3.26
598	*PABPC4*	4.4492	3.2985	2.046	-1.5908	3.26
599	*PTH*	4.2495	3.6688	1.8752	-0.20695	3.26
600	*PSG9*	5.0204	4.4042	0.36759	-1.2908	3.26
601	*FAM168B*	4.5195	3.0559	2.2163	-1.8909	3.26
602	*MBD1*	3.6889	3.1418	2.9585	-0.71493	3.26
603	*USP16*	3.668	3.3712	2.7475	0.78458	3.26
604	*C11orf53*	5.2056	3.3247	1.2549	-0.71753	3.26
605	*RPA4*	4.7354	4.6407	0.40878	-0.23528	3.26
606	*ZXDC*	4.1688	3.6094	2.0021	-1.5707	3.26
607	*ANXA1*	3.939	3.7584	2.0808	-2.3293	3.26
608	*SFMBT2*	5.2888	4.3049	0.1829	-2.0134	3.26
609	*MCOLN1*	4.9319	4.2268	0.61587	-1.8792	3.26
610	*USP50*	3.9653	3.1511	2.6486	-0.66425	3.26
611	*ADAM20*	4.4558	3.6495	1.657	-0.52276	3.25
612	*DEFB106A*	4.8338	3.7535	1.1742	0.45681	3.25
613	*GCSAML*	4.5032	3.5192	1.7383	0.93904	3.25
614	*GNG4*	4.1943	3.434	2.1306	-0.23517	3.25
615	*PLEKHG1*	3.3598	3.2048	3.1894	-2.007	3.25
616	*WFDC10A*	3.6785	3.133	2.9418	-0.12627	3.25
617	*SESTD1*	4.7012	4.5229	0.52602	-0.22492	3.25
618	*TLL2*	4.1475	3.3247	2.2755	0.31574	3.25
619	*PPP1R14A*	5.3828	5.0104	-0.64748	-2.0384	3.25
620	*CLHC1*	5.1397	2.8828	1.7232	-0.34456	3.25
621	*SECISBP2L*	4.8752	3.7588	1.1097	-2.4018	3.25
622	*NLRP10*	5.1445	2.3828	2.2133	1.4508	3.25
623	*DUS4L*	3.5056	3.2519	2.9803	-1.1737	3.25
624	*CUEDC1*	3.3937	3.2104	3.1336	-0.67378	3.25
625	*HAUS6*	3.7566	3.2171	2.7628	-1.7171	3.25
626	*TFAP2A*	5.3167	3.5999	0.8183	-0.72085	3.24
627	*MEIS1*	4.1329	3.2881	2.3135	1.674	3.24
628	*FARP2*	5.0048	3.7213	1.0043	-1.0704	3.24
629	*VCX3B*	4.6184	4.1636	0.94507	-1.2569	3.24
630	*CTIF*	5.8259	2.3531	1.5398	-1.8587	3.24
631	*KIAA1033*	3.5931	3.2696	2.8512	-0.32101	3.24
632	*ARL11*	4.3787	4.0364	1.2984	0.4084	3.24
633	*HIST1H2AC*	4.5233	3.229	1.9587	-2.5663	3.24
634	*DPYSL4*	3.3283	3.2014	3.1796	-1.5687	3.24
635	*CMSS1*	4.1129	3.6502	1.9451	0.29285	3.24
636	*AKR7A3*	4.0068	3.3047	2.3946	0.39969	3.24
637	*ARHGEF15*	3.8049	3.6821	2.2163	-1.4969	3.23

638	*FAM167A*	3.8337	3.1033	2.7662	1.0791	3.23
639	*CD200*	4.4508	3.1684	2.0812	1.9874	3.23
640	*NDUFA3*	5.2263	2.484	1.9878	-1.3097	3.23
641	*BCAR1*	3.5161	3.2622	2.9194	-2.6024	3.23
642	*DNMT3L*	5.0187	2.5613	2.1129	-1.1186	3.23
643	*MPG*	3.6044	3.2254	2.8617	-0.03472	3.23
644	*TTC9C*	4.4477	3.1581	2.0856	-1.1955	3.23
645	*F2R*	3.9146	3.4933	2.2834	-0.72157	3.23
646	*SPATS1*	4.4356	3.3842	1.8713	0.15281	3.23
647	*IL4*	3.7415	3.091	2.8581	2.6592	3.23
648	*PPIAL4A*	4.9499	4.4487	0.29117	-3.5396	3.23
649	*FAM221B*	4.4917	3.4668	1.7249	-1.9164	3.23
650	*TGFB2*	5.8245	2.8866	0.97199	0.53842	3.23
651	*OR52A1*	4.0758	4.0022	1.6015	-2.3776	3.23
652	*ZNF667*	4.5688	4.1543	0.9554	0.36987	3.23
653	*PIP5K1B*	4.3844	3.0879	2.1956	-0.05066	3.22
654	*SAFB*	4.7629	3.7033	1.2007	0.8235	3.22
655	*GTF2E1*	4.9004	3.3794	1.3796	0.24871	3.22
656	*TRIP12*	4.654	4.0422	0.95827	-0.72771	3.22
657	*PKIA*	4.7039	3.7472	1.2031	0.65454	3.22
658	*ZC3HAV1L*	3.7425	3.637	2.2722	1.2152	3.22
659	*PGP*	4.7481	2.9876	1.9159	0.41821	3.22
660	*G6PC*	5.0118	4.1718	0.46635	-2.6675	3.22
661	*M6PR*	4.7172	3.4416	1.4903	-1.6275	3.22
662	*CPE*	4.4813	2.7938	2.3723	-1.8583	3.22
663	*RNF182*	5.159	3.4285	1.0582	-1.8642	3.22
664	*UAP1*	3.913	3.5978	2.1265	-0.34255	3.21
665	*MIEF2*	3.778	2.9951	2.8639	0.67989	3.21
666	*TRAPPC5*	5.038	3.7989	0.79432	-1.6682	3.21
667	*NLRP6*	4.7896	2.976	1.8651	1.3407	3.21
668	*PLEC*	5.9129	2.6943	1.0215	0.35076	3.21
669	*GSTZ1*	4.2137	3.6178	1.7925	1.2124	3.21
670	*OR10G3*	4.1855	4.1237	1.3107	-1.4926	3.21
671	*LY6K*	4.1176	2.8174	2.6844	-0.06015	3.21
672	*CARF*	4.5064	4.2721	0.84074	-1.2987	3.21
673	*CALML6*	5.2246	4.7857	-0.39318	-0.92217	3.21
674	*PPP2CA*	3.4228	3.1749	3.0173	2.9739	3.21
675	*CHDH*	4.8674	2.8365	1.9104	-1.3203	3.20
676	*TMEM255A*	5.7505	3.7382	0.12492	-0.11186	3.20
677	*SEMA3A*	3.9311	3.0961	2.5862	0.23008	3.20
678	*DOCK9*	5.3199	3.3548	0.93869	-1.4173	3.20
679	*SEC62*	4.4738	3.2881	1.8475	-0.88834	3.20
680	*UNCX*	4.855	2.5722	2.1821	-1.7364	3.20
681	*MYH13*	4.4669	3.4457	1.6936	-2.0141	3.20
682	*ARC*	3.9569	2.9279	2.7181	0.93991	3.20
683	*GDAP1*	3.6336	3.3859	2.5831	-3.873	3.20

684	*CENPE*	3.7345	3.4783	2.3893	-0.17714	3.20
685	*STAG3*	5.1496	4.5107	-0.06139	-2.4094	3.20
686	*DAZ1*	5.4041	4.2087	-0.01439	-1.9065	3.20
687	*SUGP1*	5.4876	4.4909	-0.39916	-1.5744	3.19
688	*STYXL1*	4.9725	3.2433	1.3602	0.28279	3.19
689	*TRIM51*	4.5877	3.0442	1.9411	1.3397	3.19
690	*THAP2*	5.122	3.8177	0.63277	-0.59187	3.19
691	*RNF24*	4.8493	3.6209	1.102	-1.2919	3.19
692	*PRR16*	5.5125	2.4514	1.5982	-0.26128	3.19
693	*INPPL1*	4.9161	3.8074	0.83829	-0.62694	3.19
694	*UROS*	4.2065	3.1162	2.2383	-1.2342	3.19
695	*MDGA1*	3.9908	2.7857	2.7842	1.3341	3.19
696	*SLAIN1*	4.9889	3.0932	1.4766	1.3887	3.19
697	*ARSH*	4.5396	3.2989	1.7196	-0.41291	3.19
698	*DSCAM*	3.3381	3.1528	3.0654	-0.72916	3.19
699	*CDC42EP2*	4.8804	4.3416	0.33426	0.16186	3.19
700	*HORMAD1*	4.1582	3.9178	1.4782	-2.1312	3.18
701	*IGSF21*	4.0191	2.995	2.5384	0.861	3.18
702	*KIAA0226L*	4.8219	4.5167	0.21027	-0.4521	3.18
703	*CLTB*	4.7656	4.4864	0.29654	0.11372	3.18
704	*DST*	4.4192	4.3549	0.77215	-1.7595	3.18
705	*SRPK3*	3.8639	2.9472	2.7351	0.3289	3.18
706	*C19orf54*	4.2217	3.5495	1.772	1.0009	3.18
707	*CDKL3*	5.3959	3.6204	0.52665	-3.0687	3.18
708	*SLC39A4*	3.8926	3.6505	1.9954	-0.83566	3.18
709	*NPSR1*	5.4579	3.2244	0.85067	0.56815	3.18
710	*APOA5*	4.2399	2.6839	2.609	1.0231	3.18
711	*TMEM121*	3.7475	3.2054	2.5793	-1.7322	3.18
712	*SPINT1*	4.4246	3.5967	1.5101	-0.99059	3.18
713	*C16orf74*	4.5231	2.9235	2.0815	-0.65705	3.18
714	*SMCO2*	4.566	3.6434	1.3186	-0.1672	3.18
715	*ADRB3*	5.7677	2.3855	1.3745	-0.25832	3.18
716	*ZMYND10*	5.5989	3.9975	-0.07032	-0.71032	3.18
717	*ODC1*	4.2246	3.4522	1.8492	-1.2639	3.18
718	*ZBTB8B*	4.2297	3.3979	1.8949	0.051568	3.17
719	*MESDC2*	3.3955	3.1769	2.9487	-0.691	3.17
720	*LEPROTL1*	4.1258	4.1179	1.2753	-0.2099	3.17
721	*FLRT2*	3.811	2.8854	2.8211	1.1673	3.17
722	*ANKRD34C*	4.8642	3.1609	1.4916	-0.72488	3.17
723	*SLX4IP*	4.3167	3.1947	2.0038	-0.08458	3.17
724	*DOCK5*	3.9681	3.3311	2.2156	-1.2649	3.17
725	*HS3ST1*	4.3089	3.8906	1.3116	-0.95184	3.17
726	*RAD54L2*	3.3659	3.2473	2.8971	0.10278	3.17
727	*CORO1A*	4.7969	3.9305	0.78273	-1.3153	3.17
728	*SPIN2B*	4.1337	3.0296	2.3458	-0.49941	3.17
729	*SH3RF3*	4.3257	3.0707	2.1121	-1.0497	3.17

730	*PLCH1*	4.9159	4.1834	0.40817	-1.6604	3.17
731	*SRSF5*	4.7171	3.9294	0.86	0.84005	3.17
732	*ASB17*	4.6805	4.2805	0.54055	-0.96878	3.17
733	*ARF3*	3.8628	3.8211	1.8109	0.69118	3.16
734	*CCDC134*	4.2594	2.8242	2.4106	-1.059	3.16
735	*CCBL2*	4.596	3.7898	1.1075	-0.76358	3.16
736	*C1orf122*	4.9423	4.001	0.54907	0.082627	3.16
737	*CD109*	5.3696	4.1534	-0.03148	-0.27796	3.16
738	*ZC3H14*	4.6849	3.7769	1.0265	-1.632	3.16
739	*AKR1C3*	5.1286	3.7571	0.60079	-2.5865	3.16
740	*C14orf132*	6.0317	2.1613	1.2932	-1.8033	3.16
741	*MAGEA12*	4.7215	4.5903	0.1739	-0.44353	3.16
742	*GNAS*	4.4487	3.8568	1.1784	-0.30355	3.16
743	*SULT1A1*	3.687	3.1808	2.6152	-1.0643	3.16
744	*GAS8*	4.1545	3.7855	1.5416	-1.4339	3.16
745	*RASSF4*	4.0468	3.0157	2.4191	0.50787	3.16
746	*GNAI2*	3.7868	3.3248	2.3694	1.0419	3.16
747	*MANF*	4.945	4.6577	-0.12565	-2.1018	3.16
748	*GYS2*	5.1997	3.5553	0.72185	0.5297	3.16
749	*NADK*	4.9226	4.2538	0.29321	-5.1224	3.16
750	*KPNA1*	4.89	4.0043	0.57295	0.030847	3.16
751	*GDPD2*	3.9589	3.1701	2.3376	1.5728	3.16
752	*OTULIN*	4.6613	4.6399	0.16234	-1.3694	3.15
753	*PECR*	4.1674	2.9127	2.3797	1.0417	3.15
754	*CLCA4*	5.4851	3.0381	0.93577	-0.17318	3.15
755	*FGGY*	4.6971	2.9377	1.8147	-2.6157	3.15
756	*SLC26A5*	4.6247	4.098	0.72264	-1.571	3.15
757	*GPR61*	5.8813	4.6447	-1.0826	-1.9581	3.15
758	*PHLDA1*	4.1637	2.6637	2.6069	1.9934	3.14
759	*PLOD3*	3.5759	3.0336	2.8227	-0.32894	3.14
760	*ULK4*	5.0465	3.354	1.0299	-1.0858	3.14
761	*PPIAL4C*	4.027	3.1957	2.2045	-0.20924	3.14
762	*CSNK1G2*	4.0106	2.8199	2.5961	-2.2167	3.14
763	*TRIM23*	4.7651	4.5012	0.15492	-0.56382	3.14
764	*CHPF2*	4.0655	3.1018	2.2536	0.053863	3.14
765	*SURF2*	5.9923	1.9177	1.5097	-1.6876	3.14
766	*ORMDL1*	4.5025	3.2059	1.7108	-1.153	3.14
767	*MX2*	5.3561	4.8824	-0.82	-2.071	3.14
768	*KCNJ8*	4.6885	2.572	2.1551	1.2948	3.14
769	*PRSS27*	4.8397	4.0038	0.56964	0.13197	3.14
770	*PRM3*	3.9554	3.3783	2.0787	0.74505	3.14
771	*WNT16*	3.6667	3.5251	2.2176	0.78047	3.14
772	*CCDC8*	5.7511	4.3688	-0.71051	-1.4183	3.14
773	*ANXA2*	5.3569	4.7231	-0.67413	-0.92046	3.14
774	*ZNF77*	3.4673	3.2653	2.6682	1.2514	3.13
775	*MBOAT1*	4.2865	3.8229	1.2892	-1.0145	3.13

776	*MCCC2*	3.8336	3.5617	2.0026	-1.0757	3.13
777	*MICA*	5.4906	4.2794	-0.37461	-1.525	3.13
778	*KITLG*	4.5731	2.8467	1.9716	-0.10888	3.13
779	*RBM11*	4.5401	2.5854	2.2657	0.45315	3.13
780	*ALG6*	3.786	3.6644	1.9373	-0.75053	3.13
781	*DCUN1D2*	3.2927	3.2304	2.8595	-0.85686	3.13
782	*C1QTNF9*	4.9773	2.8626	1.5399	-1.7526	3.13
783	*QRFPR*	4.2149	2.7203	2.4441	1.1126	3.13
784	*LAMTOR3*	5.5723	2.0439	1.7624	-1.5082	3.13
785	*KCNJ11*	3.9539	3.5222	1.9019	-0.53417	3.13
786	*TDRD12*	7.0726	1.7051	0.59944	-1.927	3.13
787	*WWC2*	4.6166	3.0309	1.7295	-0.80771	3.13
788	*HPS6*	4.04	2.7584	2.5786	-0.70417	3.13
789	*FGFR1OP2*	5.1578	2.8795	1.3385	-1.2666	3.13
790	*HIBADH*	4.3975	3.3702	1.6056	-1.5579	3.12
791	*NTMT1*	3.9878	3.9646	1.4205	-0.57896	3.12
792	*SHBG*	5.0877	3.2797	1.0044	0.096888	3.12
793	*LPAR4*	3.9107	3.8757	1.5823	-2.1162	3.12
794	*FUK*	4.6196	4.1209	0.62701	0.049057	3.12
795	*ZNF606*	3.9094	3.4892	1.9627	-0.89699	3.12
796	*GRK6*	4.9573	3.2532	1.1487	-0.14543	3.12
797	*OR8D2*	4.2815	4.2185	0.85826	-1.5176	3.12
798	*CCDC66*	4.5097	4.2617	0.58665	-1.2328	3.12
799	*ACOT12*	3.9819	3.6498	1.725	-2.5647	3.12
800	*GOLGA1*	5.344	3.2279	0.78169	-0.16122	3.12
801	*TSPAN8*	4.9528	4.0061	0.39457	-0.50246	3.12
802	*MED1*	4.1259	3.4569	1.7643	-1.38	3.12
803	*ZNF37A*	5.6403	2.2003	1.5061	-0.42014	3.12
804	*XRRA1*	4.5446	3.3091	1.4924	-0.20142	3.12
805	*LMX1B*	5.3732	3.1546	0.81339	0.12611	3.11
806	*NYX*	3.9999	3.944	1.3915	-0.12742	3.11
807	*IFFO1*	5.0933	4.3653	-0.12428	-0.15596	3.11
808	*SLIRP*	4.5968	3.8252	0.90715	-1.507	3.11
809	*KSR2*	4.7439	4.1135	0.47073	-2.8044	3.11
810	*NPRL2*	5.0855	4.8957	-0.6548	-1.8402	3.11
811	*CUX1*	4.4082	2.9331	1.984	1.2744	3.11
812	*S100A1*	4.4666	4.0112	0.84658	-0.27982	3.11
813	*ZNF700*	4.1924	2.692	2.4353	-1.009	3.11
814	*AASS*	4.2526	3.3259	1.7402	1.7	3.11
815	*LURAP1L*	4.7229	3.4036	1.1897	-0.71132	3.11
816	*CDR1*	4.619	3.4937	1.2025	-0.43264	3.11
817	*PSD*	3.8632	2.9874	2.462	-0.17025	3.10
818	*DAPP1*	3.6831	3.2667	2.3626	-1.2027	3.10
819	*MEGF6*	3.7689	2.8383	2.7051	-0.74086	3.10
820	*NRG1*	3.6967	3.3823	2.2315	-1.729	3.10
821	*GPX3*	4.5253	4.2732	0.50891	-1.5538	3.10

822	*KRTAP10-8*	4.0098	2.6691	2.6275	0.44775	3.10
823	*C2orf40*	4.3887	3.2474	1.6682	1.4073	3.10
824	*SCGN*	4.3981	4.3788	0.51957	0.39116	3.10
825	*PPAPDC1B*	3.6444	3.392	2.2598	0.31761	3.10
826	*MYADML2*	4.4047	2.9479	1.9428	-2.2951	3.10
827	*LAMP1*	3.5752	3.462	2.2581	1.2512	3.10
828	*POLK*	4.8341	4.2939	0.16383	-0.31475	3.10
829	*ZNF449*	4.2956	4.2263	0.76881	0.29782	3.10
830	*OTUD4*	5.0264	4.5138	-0.25004	-0.93057	3.10
831	*KPNA3*	4.9721	4.001	0.31294	-1.0313	3.10
832	*OR2T2*	4.2355	4.1781	0.87135	-0.58404	3.09
833	*AGTR2*	3.6967	3.2966	2.2909	0.28143	3.09
834	*FYB*	5.8263	3.3842	0.07224	-0.07903	3.09
835	*CAPRIN1*	5.3791	4.1644	-0.26203	-0.84419	3.09
836	*GALNS*	5.9109	2.2126	1.1576	0.009703	3.09
837	*VCY1B*	4.4537	2.5061	2.3205	-2.3949	3.09
838	*FAM57B*	3.6831	3.5645	2.0311	-2.5743	3.09
839	*ZNRF1*	5.1603	3.1957	0.91407	0.4966	3.09
840	*ZNF732*	4.8359	2.9929	1.4387	-0.41762	3.09
841	*TEX37*	4.6944	2.4207	2.152	-0.34819	3.09
842	*NCAPD2*	4.689	4.0369	0.54017	-0.99387	3.09
843	*FAM179A*	4.3498	3.1088	1.8063	-1.3393	3.09
844	*PLA2G2A*	4.848	3.4959	0.91085	-1.8253	3.08
845	*GNA13*	3.8521	2.7663	2.6302	1.2351	3.08
846	*HCAR2*	4.0118	3.3485	1.8869	0.080928	3.08
847	*MAGEA10-MAGEA*	4.995	2.9263	1.3235	-0.26207	3.08
848	*HIF1AN*	4.3314	3.1314	1.7773	0.12471	3.08
849	*VHLL*	4.2258	3.6724	1.341	-0.44884	3.08
850	*EHD2*	4.3164	4.0063	0.91429	-1.6288	3.08
851	*ZCCHC5*	4.8836	3.8485	0.50078	-0.37215	3.08
852	*ANKHD1*	3.8327	3.1364	2.2626	-1.7945	3.08
853	*C1orf100*	4.705	2.8323	1.6934	0.14603	3.08
854	*EEFSEC*	3.185	3.0529	2.9906	-0.78793	3.08
855	*KCTD6*	4.6776	3.442	1.1081	-0.61119	3.08
856	*CLLU1OS*	5.4441	3.2766	0.50492	0.38838	3.08
857	*DYRK4*	4.3435	2.8997	1.9811	-0.54663	3.07
858	*ESAM*	3.5847	3.1187	2.5154	1.5361	3.07
859	*FANK1*	4.4184	3.9859	0.81204	0.62542	3.07
860	*CCDC6*	4.7996	2.3583	2.0579	0.020029	3.07
861	*SH2D3C*	4.0568	4.0416	1.1162	0.049826	3.07
862	*FAM83C*	4.9449	4.4549	-0.18898	-1.8851	3.07
863	*IL7*	3.4972	2.8899	2.8222	0.01556	3.07
864	*C11orf1*	4.955	2.5151	1.7389	-0.32446	3.07
865	*LCNL1*	3.6162	2.8641	2.7278	0.85583	3.07
866	*TGM6*	3.3126	3.1493	2.7462	1.2105	3.07
867	*AVPI1*	4.222	3.4708	1.5141	0.17324	3.07

868	*KLRF1*	3.6468	2.904	2.6556	-1.547	3.07
869	*RNASE1*	3.8501	2.7177	2.6381	1.6948	3.07
870	*ENG*	4.6833	4.0906	0.42787	-0.51752	3.07
871	*CDSN*	5.1466	2.2781	1.7768	-1.5971	3.07
872	*B3GNT2*	4.9536	3.4098	0.83536	-1.0095	3.07
873	*ZNF844*	3.7339	3.0372	2.4245	0.36126	3.07
874	*NEFM*	4.8244	2.789	1.5812	-1.9326	3.06
875	*OR4F3*	4.3592	3.7779	1.0546	-0.82053	3.06
876	*BTNL8*	3.8878	2.8824	2.4203	-0.03648	3.06
877	*FAM167B*	4.3915	3.7452	1.0528	-0.37495	3.06
878	*KCNE3*	4.0719	2.8149	2.3017	0.84712	3.06
879	*AOAH*	4.6698	3.4097	1.1086	-0.95565	3.06
880	*DYNC2H1*	4.376	2.7917	2.0159	-1.1733	3.06
881	*NUP37*	4.5294	4.0333	0.62018	-0.23116	3.06
882	*SLC4A8*	3.7551	3.2074	2.2197	-0.69286	3.06
883	*KRTAP19-4*	4.7089	2.5419	1.9303	-0.05	3.06
884	*PHPT1*	3.4469	3.3965	2.3376	-1.3407	3.06
885	*OAS3*	3.9044	3.5192	1.7539	-2.3294	3.06
886	*LOC100130370*	4.9562	4.048	0.1727	-0.4264	3.06
887	*HERC4*	4.0947	3.8166	1.2597	0.30815	3.06
888	*PSEN2*	4.9159	4.644	-0.38945	-1.3696	3.06
889	*DDC*	3.9913	3.135	2.0435	-1.3208	3.06
890	*SLC24A2*	5.5315	4.4241	-0.78588	-1.5121	3.06
891	*IPMK*	4.8758	4.3591	-0.0665	-0.36196	3.06
892	*DIAPH3*	3.8697	3.4148	1.8832	0.14641	3.06
893	*ARHGEF1*	3.9446	3.1118	2.111	-2.6257	3.06
894	*UHMK1*	4.5716	4.1558	0.43849	-0.55047	3.06
895	*SYCE1*	3.5242	3.4561	2.1847	0.81769	3.06
896	*C14orf180*	4.3192	4.1375	0.70551	-0.05804	3.05
897	*SH3BGR*	4.6024	3.4848	1.0747	0.43719	3.05
898	*MYOF*	3.8458	3.4991	1.8138	1.2541	3.05
899	*C16orf87*	3.6759	3.0023	2.479	-1.6617	3.05
900	*SP7*	4.4457	2.7893	1.9214	1.7623	3.05
901	*METRN*	4.9691	3.781	0.40322	-1.5752	3.05
902	*RERGL*	5.4132	4.1536	-0.41469	-2.0476	3.05
903	*ZNF548*	4.8858	3.3679	0.89741	-1.0564	3.05
904	*TMEM176B*	5.2063	3.4025	0.54228	-0.93764	3.05
905	*VWF*	4.069	2.7211	2.3598	0.72362	3.05
906	*TULP4*	3.7709	3.1226	2.2542	1.5469	3.05
907	*SLC15A4*	5.5275	3.3999	0.2169	-0.82959	3.05
908	*HIST1H1D*	4.4895	4.0775	0.57685	-1.7549	3.05
909	*ROCK1*	4.8016	4.2547	0.08598	-2.1217	3.05
910	*CYP7A1*	3.9875	3.7627	1.3875	-0.46384	3.05
911	*WDR31*	3.7043	2.8285	2.6047	-1.7061	3.05
912	*ACER2*	4.5383	3.1615	1.4344	0.40931	3.04
913	*PTDSS2*	5.1545	2.8626	1.1098	-0.33174	3.04

914	*ASMTL*	4.9635	3.7366	0.42619	-1.8583	3.04
915	*ZNF316*	4.4984	3.9545	0.6695	-0.10928	3.04
916	*PEX19*	3.8114	3.3287	1.9822	-1.2955	3.04
917	*CCDC22*	3.8468	3.1426	2.1244	-1.8816	3.04
918	*ITFG3*	3.9603	3.7904	1.3566	-1.0117	3.04
919	*ADCY2*	4.4018	3.5456	1.1597	-0.09235	3.04
920	*PON3*	4.8316	3.6027	0.67277	-0.81205	3.04
921	*RAP2C*	5.383	4.127	-0.40319	-1.8123	3.04
922	*KLF1*	4.297	3.0909	1.7167	0.26476	3.03
923	*PLIN1*	4.4303	2.4241	2.246	0.73119	3.03
924	*OR4N5*	3.6283	3.2866	2.1835	-1.8076	3.03
925	*ARHGAP19*	4.8579	3.9939	0.24633	-1.2867	3.03
926	*ZSCAN32*	4.7328	4.3655	-0.00066	-3.1229	3.03
927	*DUS3L*	4.2455	2.5617	2.288	1.25	3.03
928	*PPP1R3A*	4.1418	4.0174	0.93548	-2.3849	3.03
929	*FAM132A*	3.9052	2.7243	2.4647	-0.11906	3.03
930	*TXNDC15*	3.6207	2.7616	2.7097	-1.1247	3.03
931	*HNRNPUL1*	3.4594	3.1746	2.4546	-0.51259	3.03
932	*IL22*	4.4264	3.1653	1.4952	-0.39856	3.03
933	*CALY*	5.1745	3.3725	0.53888	-1.0221	3.03
934	*TRIM37*	3.4001	2.8829	2.8011	-0.06671	3.03
935	*AANAT*	3.7291	3.3899	1.9629	-1.1313	3.03
936	*OR4A5*	3.4268	2.8769	2.7747	-0.15733	3.03
937	*NFIC*	4.5909	3.3292	1.1567	-1.1338	3.03
938	*MRPS9*	4.0872	2.6565	2.3321	0.4688	3.03
939	*RNF152*	4.4977	2.4912	2.0866	1.2117	3.03
940	*PIWIL1*	4.2867	2.7237	2.0611	-1.3713	3.02
941	*TEX11*	3.7475	3.1943	2.1254	-1.0063	3.02
942	*SFRP4*	5.2362	3.7208	0.10924	-1.2234	3.02
943	*RBMY1B*	4.2014	3.7717	1.0887	-0.61092	3.02
944	*PALLD*	5.7063	3.4285	-0.07335	-3.0444	3.02
945	*TAPBP*	4.3817	3.4804	1.1989	0.001973	3.02
946	*THAP11*	4.9398	2.5991	1.522	0.56658	3.02
947	*EBF3*	5.2987	2.0728	1.683	1.363	3.02
948	*MMP15*	4.0063	2.5653	2.4801	-1.138	3.02
949	*PSG8*	5.0845	3.1545	0.81141	-0.86821	3.02
950	*LOC100505549*	3.3143	3.0985	2.6346	1.2898	3.02
951	*GSTA1*	4.8385	4.1545	0.05428	-0.198	3.02
952	*AK4*	4.3883	4.1481	0.50936	-0.42675	3.02
953	*NBPF9*	4.7507	4.0336	0.25952	-1.449	3.01
954	*SDR42E1*	3.3902	3.1335	2.518	1.4349	3.01
955	*PITPNM3*	3.8883	3.3315	1.8194	-1.3781	3.01
956	*OTUD5*	3.1772	3.1619	2.6961	-1.0089	3.01
957	*EBF1*	4.1578	3.5487	1.3277	0.16456	3.01
958	*PRR5-ARHGAP8*	3.6877	2.7245	2.6202	-0.72969	3.01
959	*FAM91A1*	4.3102	4.2891	0.43054	0.31699	3.01

960	*GPR84*	5.6612	3.0098	0.35707	-0.58736	3.01
961	*TBX10*	5.8211	3.9839	-0.77895	-1.3523	3.01
962	*ZNF200*	5.3335	2.2676	1.4244	0.038662	3.01
963	*GPR143*	4.0785	2.8732	2.0726	-1.0444	3.01
964	*WDFY3*	4.2598	2.7475	2.0168	-2.3496	3.01
965	*GPNMB*	4.8169	2.4621	1.745	-1.0528	3.01
966	*VCL*	4.5982	2.4265	1.9993	0.50522	3.01
967	*CLEC17A*	4.4605	4.2392	0.32334	0.19285	3.01
968	*OR51L1*	5.1934	3.5184	0.31065	-1.3098	3.01
969	*EMID1*	5.0621	2.559	1.4013	0.69052	3.01
970	*BHLHE40*	3.7367	3.5311	1.7514	0.55151	3.01
971	*EEPD1*	4.0296	2.5007	2.4885	0.6291	3.01
972	*LYNX1*	4.4295	2.463	2.1259	-0.36148	3.01
973	*METTL21B*	4.2331	3.3939	1.3892	0.76725	3.01
974	*PHKB*	4.5709	2.2821	2.1623	-0.714	3.01
975	*TIGD3*	4.985	3.8919	0.13796	-0.09541	3.00
976	*GLP1R*	4.3962	4.3133	0.30472	-1.707	3.00
977	*CEMIP*	5.2751	2.8104	0.92641	-0.61164	3.00
978	*FLNC*	3.8173	3.1064	2.0855	1.0684	3.00
979	*F13A1*	4.53	3.1447	1.3337	-0.47183	3.00
980	*TNRC18*	4.3078	2.7608	1.9391	0.29241	3.00
981	*VLDLR*	5.5502	1.8964	1.5589	-1.7652	3.00
982	*OSR2*	4.6328	4.159	0.21256	-1.4935	3.00
983	*OR14C36*	4.0925	4.0796	0.82971	-1.3035	3.00
984	*GSK3A*	4.6661	4.6035	-0.26854	-1.6663	3.00
985	*CEP85L*	3.3404	2.9581	2.6994	-1.3457	3.00
986	*TIMP4*	4.1422	3.9017	0.95279	0.74742	3.00
987	*TVP23B*	3.0889	3.0422	2.8633	-0.80771	3.00
988	*FADS3*	4.308	3.4152	1.2674	0.37779	3.00
989	*DKK2*	4.5766	2.496	1.912	-1.9821	2.99
990	*DHX29*	4.3134	2.9681	1.7008	-1.8946	2.99
991	*ARRDC5*	4.2483	4.2074	0.52335	-1.1573	2.99
992	*CCDC172*	3.9622	3.8347	1.1817	-0.57824	2.99
993	*CDC25A*	4.7498	4.6054	-0.37898	-1.1421	2.99
994	*DYDC2*	3.6318	3.1148	2.2244	0.28506	2.99
995	*IFT80*	3.9593	2.9829	2.0277	-0.25892	2.99
996	*CD19*	4.9432	4.5032	-0.47777	-1.0626	2.99
997	*C12orf50*	4.0539	3.751	1.1637	-0.55779	2.99
998	*ART1*	5.4914	4.5256	-1.0564	-1.3358	2.99
999	*ASZ1*	4.5816	2.6415	1.736	-0.49989	2.99
1000	*CD47*	4.0529	3.6732	1.2327	-1.1927	2.99
1001	*LIN7A*	5.3074	4.0794	-0.43077	-1.7493	2.99
1002	*ZNF292*	3.7714	3.3871	1.7966	-0.47023	2.99
1003	*PRORY*	4.6632	3.4573	0.83398	-0.5395	2.98
1004	*ZSWIM4*	5.0844	4.2459	-0.3791	-1.0324	2.98
1005	*CDKN1A*	3.9051	2.5486	2.4952	-2.6072	2.98

1006	*PRAF2*	4.204	3.2615	1.4825	0.33089	2.98
1007	*RPH3AL*	3.5064	3.0244	2.414	-1.8889	2.98
1008	*FAM98A*	3.9243	3.908	1.1098	-0.12837	2.98
1009	*NR2F6*	3.621	3.3008	2.0187	-0.10911	2.98
1010	*OR4A16*	4.7041	3.3679	0.86615	0.70665	2.98
1011	*RWDD2B*	3.3402	3.008	2.5897	-0.32495	2.98
1012	*SERPINB2*	4.0723	2.6513	2.2134	-1.6526	2.98
1013	*TC2N*	4.4602	4.2863	0.19018	-3.2582	2.98
1014	*GMPR*	3.768	2.8664	2.3017	0.06209	2.98
1015	*YWHAG*	3.4364	2.7709	2.7287	-0.8903	2.98
1016	*PPP1R12C*	5.1792	3.1227	0.63103	-0.54501	2.98
1017	*MAN2B1*	4.5562	2.5958	1.7805	0.69304	2.98
1018	*GCSAM*	4.0758	3.1622	1.6943	-0.43203	2.98
1019	*RPL3L*	4.3553	3.4934	1.0821	-2.0164	2.98
1020	*GALNT1*	3.2604	2.862	2.8081	0.5143	2.98
1021	*BTN3A1*	3.3194	3.2457	2.364	0.7829	2.98
1022	*ZNF627*	3.8564	2.9785	2.0914	1.3754	2.98
1023	*USP17L1*	4.2616	4.2601	0.4045	-1.4241	2.98
1024	*RASGRF2*	5.4734	3.4232	0.02935	-1.4376	2.98
1025	*LEUTX*	3.8871	3.1416	1.8945	-0.46749	2.97
1026	*SVIP*	3.9851	2.7407	2.1942	-1.8342	2.97
1027	*ZNF418*	4.016	3.9994	0.90157	0.32028	2.97
1028	*TIPARP*	3.427	3.226	2.2635	-0.57921	2.97
1029	*METTL6*	4.3596	4.3406	0.21608	0.10942	2.97
1030	*FBLN1*	4.5847	4.1543	0.17591	-0.92581	2.97
1031	*UNC80*	4.7912	3.348	0.77541	-0.01594	2.97
1032	*CLCN5*	4.7276	2.9645	1.2223	0.57821	2.97
1033	*OR6Q1*	3.6787	2.9792	2.2553	-0.35089	2.97
1034	*FIS1*	5.2375	2.8052	0.8702	-0.96072	2.97
1035	*BCKDHA*	5.2346	3.6763	0.00116	-0.3859	2.97
1036	*KIAA1324L*	5.2128	3.723	-0.02429	-1.8282	2.97
1037	*EPS15L1*	4.764	2.7912	1.3561	1.1738	2.97
1038	*ZXDA*	5.1578	3.9317	-0.18153	-0.2728	2.97
1039	*KLF10*	4.0168	3.9075	0.98354	0.38526	2.97
1040	*OR1C1*	4.7927	2.5767	1.537	1.4043	2.97
1041	*BRPF1*	3.6324	2.9033	2.3705	0.13294	2.97
1042	*ITM2A*	4.4695	3.5613	0.87508	-0.83101	2.97
1043	*VIL1*	4.7078	4.2752	-0.07731	-0.99033	2.97
1044	*EPHA3*	5.0056	3.2165	0.68229	-1.677	2.97
1045	*MAST3*	4.806	2.3118	1.7853	-1.894	2.97
1046	*KCNAB2*	4.518	3.5759	0.80876	0.43849	2.97
1047	*RANBP3L*	5.048	2.0945	1.7598	0.50421	2.97
1048	*LPAR3*	3.5447	3.1093	2.248	-0.86229	2.97
1049	*ARAP1*	5.2021	4.7045	-1.0093	-1.3282	2.97
1050	*MRPL24*	4.2721	2.561	2.0628	-1.4375	2.97
1051	*ZSWIM7*	4.96	3.1804	0.74827	0.19567	2.96

1052	*RAP2B*	4.8822	3.7376	0.26815	-0.76593	2.96
1053	*SOCS1*	4.9904	2.9019	0.99547	-1.6524	2.96
1054	*ZNF536*	4.8972	4.606	-0.61687	-1.0185	2.96
1055	*LRPAP1*	4.4375	3.1643	1.2806	-0.86403	2.96
1056	*PCDHA12*	4.0796	3.6654	1.1357	-0.53657	2.96
1057	*DEGS1*	4.2238	3.9985	0.65767	-1.1927	2.96
1058	*RGS12*	4.8019	4.4354	-0.35924	-1.2329	2.96
1059	*TCP11*	4.5644	4.1437	0.16896	-0.05159	2.96
1060	*DNAJB9*	4.6955	3.4227	0.75872	-1.3617	2.96
1061	*FAM53C*	5.3849	3.0131	0.47848	-0.03775	2.96
1062	*C1orf95*	4.9006	4.0868	-0.11666	-0.44353	2.96
1063	*ZNF774*	4.5268	3.9719	0.37026	-0.73362	2.96
1064	*CRTC3*	3.3617	3.0305	2.4761	1.0958	2.96
1065	*PCIF1*	4.7788	3.0881	1.0013	-0.09893	2.96
1066	*HIST1H3F*	4.8404	3.5337	0.49389	-2.0476	2.96
1067	*HMCN2*	3.7218	2.8116	2.333	1.1733	2.96
1068	*GZMH*	3.6475	3.3091	1.9093	1.2681	2.96
1069	*GAA*	5.068	3.0119	0.77904	-1.2576	2.95
1070	*ZDHHC15*	3.2882	3.1886	2.3819	-0.63139	2.95
1071	*ZNF705A*	4.5096	2.494	1.8533	-0.23286	2.95
1072	*REC114*	4.2929	3.7384	0.82264	0.5635	2.95
1073	*KRT72*	3.8711	3.0127	1.9694	0.64312	2.95
1074	*ADAD1*	4.5265	3.3665	0.95926	-0.5468	2.95
1075	*SETMAR*	4.1541	2.3563	2.3418	-0.46205	2.95
1076	*TDRD1*	4.4534	4.1634	0.23329	-0.20142	2.95
1077	*MCC*	3.5508	3.5431	1.7561	0.82785	2.95
1078	*HAND2*	4.3961	3.7181	0.73398	-0.76405	2.95
1079	*DHCR7*	3.9341	2.6976	2.2122	-0.47199	2.95
1080	*RPUSD3*	4.3414	2.5784	1.9229	-2.2352	2.95
1081	*ZNF79*	5.27	2.8724	0.69967	-0.443	2.95
1082	*PRAMEF4*	4.1471	3.4171	1.2763	-2.0016	2.95
1083	*AGO3*	5.5504	3.7461	-0.45867	-1.3683	2.95
1084	*NUCKS1*	5.0009	2.9873	0.84087	-1.0296	2.94
1085	*MMAB*	3.4684	2.9129	2.4449	-0.82654	2.94
1086	*CTSG*	4.4699	3.2102	1.146	-2.3282	2.94
1087	*BRIP1*	5.741	2.8288	0.25593	-0.86712	2.94
1088	*STT3A*	3.9241	2.7018	2.1981	-0.70665	2.94
1089	*NOTCH2NL*	4.8051	3.2884	0.72968	-0.44353	2.94
1090	*ABCD2*	4.6386	3.6591	0.52504	-1.3951	2.94
1091	*CTSW*	4.9754	3.2011	0.64535	-1.349	2.94
1092	*SPRR2G*	4.1826	3.1833	1.4556	-0.35182	2.94
1093	*SSX2B*	6.1791	3.5233	-0.88159	-1.2567	2.94
1094	*LINC00452*	4.7856	4.1109	-0.07659	-0.47481	2.94
1095	*TNFSF18*	3.7726	3.7624	1.2836	0.78283	2.94
1096	*KRTAP12-4*	4.3989	2.9671	1.452	-0.23203	2.94
1097	*RP1*	4.7309	3.1755	0.9111	0.84999	2.94

1098	*TNPO2*	3.6145	3.2661	1.9368	-0.82056	2.94
1099	*EPOR*	5.4961	2.2852	1.0352	-0.55288	2.94
1100	*UGT1A1*	3.79	3.1636	1.8622	-0.26519	2.94
1101	*TYR*	3.7193	2.6991	2.3968	0.062078	2.94
1102	*OR10P1*	3.0758	2.8727	2.8659	1.3206	2.94
1103	*MTRR*	5.1734	2.6217	1.0183	-1.7766	2.94
1104	*OR5H15*	3.7923	2.954	2.0654	-2.3372	2.94
1105	*MYOCD*	3.9387	3.0625	1.8082	0.58461	2.94
1106	*TAOK2*	4.5718	2.5023	1.7331	1.1449	2.94
1107	*EPPIN-WFDC6*	4.6188	2.5458	1.6424	-1.7493	2.94
1108	*PRELID2*	4.0016	3.4987	1.3057	-0.27982	2.94
1109	*CEP112*	4.1141	3.85	0.8389	-1.2867	2.93
1110	*SENP1*	4.2278	3.6037	0.97037	-1.3117	2.93
1111	*RABL3*	3.3878	3.2089	2.2046	-1.0453	2.93
1112	*FTH1P18*	3.0823	3.0363	2.6821	0.30568	2.93
1113	*ANAPC13*	4.4564	4.2447	0.0995	-0.58959	2.93
1114	*TJP1*	5.2753	4.7364	-1.2114	-3.0595	2.93
1115	*FBXL5*	3.5885	3.2524	1.9588	1.5088	2.93
1116	*IDH1*	4.135	3.7324	0.93217	-0.57042	2.93
1117	*PODN*	4.5425	2.9266	1.3303	-0.83883	2.93
1118	*LRRC40*	4.0816	3.4724	1.2446	-0.02523	2.93
1119	*TMEM38B*	5.4802	2.1784	1.1372	-0.53929	2.93
1120	*LOC100129520*	4.1716	3.5212	1.1024	-0.26315	2.93
1121	*GID8*	4.364	4.001	0.42975	-2.2456	2.93
1122	*NLRP5*	4.3029	2.7724	1.7175	-1.2246	2.93
1123	*PRAMEF17*	4.8436	4.0498	-0.1042	-0.44137	2.93
1124	*GUSB*	4.0694	2.4131	2.304	0.19372	2.93
1125	*GRID1*	5.0541	3.7062	0.0251	-0.97138	2.93
1126	*SLC35F2*	3.9631	3.3919	1.4289	-1.535	2.93
1127	*GP6*	3.91	3.8118	1.0613	-1.4864	2.93
1128	*BTBD16*	5.1672	3.4878	0.12703	-1.4684	2.93
1129	*EPGN*	4.7327	3.8423	0.20381	-0.98062	2.93
1130	*DRC7*	4.6641	4.4112	-0.29752	-1.3664	2.93
1131	*RNF130*	4.9795	3.556	0.23983	-0.22048	2.93
1132	*PUM1*	4.9991	3.3556	0.41853	-1.5244	2.92
1133	*HIST1H3G*	4.4766	3.9776	0.31771	0.24995	2.92
1134	*TMEM167B*	5.0909	3.8581	-0.18006	-0.8952	2.92
1135	*GABRQ*	4.1085	3.0268	1.6321	0.86523	2.92
1136	*SIPA1*	3.9524	2.5444	2.2672	-0.08614	2.92
1137	*C1orf174*	4.4265	3.9783	0.3582	-1.9054	2.92
1138	*MARCH3*	5.3339	3.2207	0.20193	-0.80636	2.92
1139	*GALNT4*	5.0701	3.9388	-0.25253	-1.1169	2.92
1140	*BST2*	4.1854	3.4182	1.1523	-0.85794	2.92
1141	*HAAO*	4.8631	2.2106	1.6811	0.83672	2.92
1142	*TECTA*	4.7847	2.9491	1.0205	-0.7512	2.92
1143	*CASP8*	4.0207	2.7323	1.9964	-0.40112	2.92

1144	*MUC4*	4.2394	3.3048	1.2034	0.9368	2.92
1145	*DNAH14*	4.0239	3.7823	0.9403	-2.1342	2.92
1146	*ADNP2*	4.2209	3.5921	0.93283	-1.5631	2.92
1147	*TOP3B*	3.579	3.4002	1.7658	0.011438	2.92
1148	*CD209*	5.7087	3.5215	-0.48756	-0.5101	2.91
1149	*IFT88*	3.5045	2.6296	2.6034	1.1922	2.91
1150	*SRR*	4.3387	4.1324	0.26451	-1.4962	2.91
1151	*ADAM7*	4.045	4.0323	0.65411	0.24553	2.91
1152	*GSE1*	4.6714	4.3716	-0.31191	-0.31232	2.91
1153	*CHD9*	4.2157	3.1526	1.3599	-0.24191	2.91
1154	*FLT3LG*	5.1714	2.7314	0.82421	-1.2569	2.91
1155	*C1orf186*	4.1236	2.6243	1.9781	1.0849	2.91
1156	*TREX2*	5.1681	3.1939	0.36019	0.20959	2.91
1157	*RASSF7*	4.6482	2.1542	1.9181	0.68157	2.91
1158	*TNIP3*	3.9534	3.0639	1.7012	-0.04425	2.91
1159	*BCL11B*	5.0381	2.7461	0.93044	-0.27541	2.90
1160	*GAP43*	3.7123	3.0737	1.9268	-2.0059	2.90
1161	*PACSIN3*	3.5093	2.7181	2.4849	0.47147	2.90
1162	*ZNF726*	4.423	4.3931	-0.10676	-0.58957	2.90
1163	*C9orf173*	4.075	3.1419	1.4916	0.91604	2.90
1164	*CES5A*	3.5822	3.4474	1.6778	-1.5533	2.90
1165	*TRAF5*	3.7426	3.7098	1.255	-1.5552	2.90
1166	*PAGE2*	3.6673	2.9186	2.1184	0.94917	2.90
1167	*TFDP3*	5.6809	3.418	-0.39506	-2.4394	2.90
1168	*ZNF26*	4.5689	2.8056	1.3283	-1.2509	2.90
1169	*LIF*	5.3015	2.8296	0.57159	-2.2165	2.90
1170	*SLC2A11*	5.5485	3.644	-0.49247	-1.5765	2.90
1171	*TRH*	4.4138	3.7811	0.50496	-0.82326	2.90
1172	*TARBP2*	3.2086	3.0846	2.4062	-0.74913	2.90
1173	*FAM214A*	4.2329	2.8656	1.5994	0.34505	2.90
1174	*LGI4*	4.0663	3.8502	0.78012	-0.81512	2.90
1175	*RAB20*	3.5257	2.6628	2.5081	0.9037	2.90
1176	*UMAD1*	5.6086	4.5717	-1.4841	-1.8157	2.90
1177	*RASAL3*	4.0655	3.3435	1.287	-1.7365	2.90
1178	*FBXL6*	3.876	3.7664	1.0484	0.63235	2.90
1179	*MFGE8*	5.0259	4.2057	-0.54391	-1.8851	2.90
1180	*CTCF*	3.9009	2.6273	2.1583	0.030912	2.90
1181	*ATXN7*	5.2068	2.0996	1.3796	0.14155	2.90
1182	*FNIP2*	4.8164	3.401	0.46662	-0.275	2.89
1183	*KCNJ18*	3.9283	2.6443	2.1111	2.0277	2.89
1184	*SMKR1*	3.5192	2.9383	2.2254	-4.8437	2.89
1185	*GALE*	4.6078	3.5179	0.55475	-0.44353	2.89
1186	*NOL8*	3.3045	2.7793	2.5929	-1.7528	2.89
1187	*RFX3*	5.1885	2.2917	1.1952	-1.7277	2.89
1188	*ZDBF2*	4.8695	4.0574	-0.25605	-1.6885	2.89
1189	*HDGFL1*	4.8364	4.2593	-0.42615	-1.9919	2.89

1190	*ZRANB3*	3.3219	2.7865	2.56	0.072941	2.89
1191	*SPINK2*	4.1119	3.4657	1.0892	-0.67853	2.89
1192	*PHACTR4*	3.7822	3.2151	1.6667	-1.42	2.89
1193	*ITGAD*	5.1368	4.0171	-0.49127	-0.85565	2.89
1194	*SMIM3*	4.2499	3.1964	1.2163	0.061214	2.89
1195	*FAM214B*	4.5384	3.4723	0.65175	0.41875	2.89
1196	*FEM1B*	4.6708	3.9851	0.00589	-0.60113	2.89
1197	*OPRL1*	5.3667	3.1156	0.17807	-0.89699	2.89
1198	*CCND2*	3.8015	2.5993	2.2565	-1.3186	2.89
1199	*LDHD*	4.0835	2.7488	1.8192	-0.01493	2.88
1200	*PCSK4*	4.3427	2.4198	1.8885	-1.3028	2.88
1201	*SORT1*	4.8838	2.6705	1.0964	-1.9381	2.88
1202	*GID4*	3.3905	2.7353	2.5224	-0.68869	2.88
1203	*CCNT2*	4.5132	3.7629	0.37208	-1.7229	2.88
1204	*PCDHB12*	4.3416	3.1542	1.1509	-2.2075	2.88
1205	*NFIL3*	3.994	3.897	0.75464	-0.87655	2.88
1206	*PIN1*	3.2757	3.2454	2.1208	0.2288	2.88
1207	*SRD5A2*	4.0375	2.3342	2.2702	0.89431	2.88
1208	*STH*	4.6276	2.8879	1.1236	-0.51767	2.88
1209	*HOXD9*	3.8217	2.7078	2.1087	-0.17238	2.88
1210	*CCDC122*	3.5945	3.5298	1.5139	-0.19718	2.88
1211	*FASTKD2*	3.2988	3.0667	2.2686	-0.14091	2.88
1212	*NRK*	3.9294	3.8579	0.84579	-0.45757	2.88
1213	*PCCA*	3.7561	3.2357	1.6401	0.44709	2.88
1214	*MTAP*	4.0849	3.2223	1.3244	-1.2716	2.88
1215	*IRAK1*	3.4585	3.08	2.0924	-0.64967	2.88
1216	*SMIM8*	4.2484	3.6562	0.72413	-0.61818	2.88
1217	*RHCG*	3.2744	3.1176	2.2366	-1.6735	2.88
1218	*TBX22*	3.6303	3.2497	1.7468	-0.1536	2.88
1219	*AGBL2*	4.2784	3.715	0.62861	0.060969	2.87
1220	*CHST8*	3.6988	2.7624	2.1607	-1.5996	2.87
1221	*PGPEP1*	4.3938	2.7655	1.4617	-2.3358	2.87
1222	*ACTL8*	4.416	2.7603	1.4439	-1.8972	2.87
1223	*KIAA1217*	4.8495	3.5308	0.23715	-1.4996	2.87
1224	*MRGPRG*	3.5111	2.7983	2.3062	0.65057	2.87
1225	*SDR9C7*	4.9411	2.5212	1.1528	-1.8623	2.87
1226	*NAALADL2*	4.75	3.47	0.39313	0.22925	2.87
1227	*CDC42EP1*	4.7866	3.6596	0.16456	-2.1155	2.87
1228	*HNRNPDL*	4.363	4.3616	-0.12092	-0.35328	2.87
1229	*MBD4*	4.5859	2.2304	1.7845	-0.93112	2.87
1230	*USP49*	4.7424	3.46	0.39528	-0.03976	2.87
1231	*KCNJ5*	3.8815	3.5064	1.2069	-0.93892	2.86
1232	*OR56B4*	3.8056	3.0258	1.7628	-1.9548	2.86
1233	*GRM5*	4.2259	4.0409	0.31613	-1.5073	2.86
1234	*PLEKHH2*	5.0142	2.8408	0.72458	-2.1669	2.86
1235	*ZCCHC17*	5.1517	2.7591	0.66646	-1.4782	2.86

1236	*TMEM60*	3.389	2.8146	2.3717	0.54766	2.86
1237	*CALCR*	4.0385	2.5096	2.0257	0.18696	2.86
1238	*MAST2*	4.1309	2.9834	1.459	-0.73145	2.86
1239	*USP43*	3.5652	2.6289	2.3774	-1.1858	2.86
1240	*TAF7*	4.0101	3.9714	0.58878	0.49222	2.86
1241	*CSF2RB*	4.513	3.5121	0.54515	0.22531	2.86
1242	*RAD18*	3.6179	3.4508	1.5003	0.60507	2.86
1243	*COL8A2*	3.5079	3.3123	1.748	-0.45373	2.86
1244	*IRAK1BP1*	4.4106	4.3294	-0.17696	-2.4333	2.85
1245	*AOC2*	5.047	2.1162	1.3983	-1.0733	2.85
1246	*TMEM117*	4.4458	3.8287	0.28648	0.25825	2.85
1247	*SNX2*	3.7512	3.4984	1.3104	0.92154	2.85
1248	*ZNF277*	4.6095	3.3117	0.63682	0.32367	2.85
1249	*PDE7A*	5.4921	2.3174	0.74748	-0.69399	2.85
1250	*CEACAM4*	5.0818	2.6774	0.79458	0.001501	2.85
1251	*CAB39L*	4.8941	3.7669	-0.10857	-1.2399	2.85
1252	*SLC9A7*	5.2914	1.8285	1.4319	-0.03501	2.85
1253	*NRG4*	4.4828	4.2616	-0.19364	-1.7766	2.85
1254	*SLC5A9*	4.9581	3.4007	0.18807	-0.11483	2.85
1255	*ZNF800*	3.6462	3.1437	1.7568	-1.1523	2.85
1256	*C17orf96*	5.1686	4.2416	-0.86358	-1.1263	2.85
1257	*VAX1*	5.353	1.7374	1.4561	-0.27023	2.85
1258	*CAPN13*	5.2067	2.3913	0.94832	-0.03648	2.85
1259	*PDGFD*	4.7947	2.9079	0.8417	-0.80432	2.85
1260	*TGDS*	4.8199	4.4643	-0.74279	-1.3272	2.85
1261	*PRKRIR*	5.4539	3.3195	-0.23264	-1.2797	2.85
1262	*B3GNT5*	4.7656	2.7517	1.022	-1.9062	2.85
1263	*ZKSCAN1*	4.4728	2.4454	1.6205	-1.439	2.85
1264	*FAM171A1*	3.6915	3.1351	1.7113	1.6846	2.85
1265	*CELSR3*	4.1713	3.9408	0.42579	-0.98099	2.85
1266	*TMEM155*	4.451	3.7448	0.34011	-1.1692	2.85
1267	*ODF3L1*	4.4334	2.6782	1.4233	-1.237	2.84
1268	*CPO*	5.0489	2.4395	1.0447	0.32798	2.84
1269	*OSCP1*	4.2926	3.4548	0.78479	-0.27771	2.84
1270	*SMLR1*	5.4296	1.9785	1.1229	0.82871	2.84
1271	*PCCB*	4.6524	2.6731	1.2025	-1.146	2.84
1272	*GCM2*	4.9558	3.5614	0.0091	-0.69961	2.84
1273	*TXNRD3*	5.7086	2.7338	0.08159	-0.62125	2.84
1274	*FBXO25*	4.4386	3.5576	0.52708	-0.81963	2.84
1275	*MSRB3*	4.6591	1.9853	1.8778	0.78652	2.84
1276	*WIPF1*	5.3792	3.8415	-0.70193	-1.9099	2.84
1277	*SLC1A1*	4.8539	3.9534	-0.28901	-0.85345	2.84
1278	*ZSCAN18*	4.5094	3.0808	0.92731	0.91889	2.84
1279	*NIPAL1*	4.2196	2.9623	1.3352	-4.897	2.84
1280	*COL4A1*	4.3944	2.1502	1.9694	0.46873	2.84
1281	*SBSPON*	4.6662	3.3753	0.47145	-0.80213	2.84

1282	*CCDC132*	3.4168	2.9147	2.1752	0.50266	2.84
1283	*P2RX6*	4.7916	3.0261	0.68874	-0.38638	2.84
1284	*C5AR1*	4.1166	2.7594	1.6296	0.40518	2.84
1285	*CYSTM1*	4.566	2.9097	1.0298	-1.6594	2.84
1286	*CLPTM1*	3.0272	2.982	2.4924	2.3659	2.83
1287	*SOX2*	4.0187	3.0091	1.4691	0.031644	2.83
1288	*ERCC5*	5.3576	2.246	0.88995	0.65005	2.83
1289	*HOSPHO2-KLHL2*	5.4069	3.2135	-0.12728	-1.0021	2.83
1290	*CYB5D1*	3.357	2.9614	2.1725	-0.88667	2.83
1291	*IQCF2*	3.5237	2.8517	2.112	-0.27877	2.83
1292	*C9orf106*	4.5164	3.0074	0.96042	-1.928	2.83
1293	*CARM1*	3.5831	2.7119	2.1881	-1.4864	2.83
1294	*TSPO2*	3.8161	2.502	2.162	0.81991	2.83
1295	*KDM5B*	5.1148	3.7088	-0.34553	-0.67417	2.83
1296	*NID2*	3.3151	2.8767	2.2859	0.1789	2.83
1297	*DCLK3*	4.1385	3.2044	1.1347	-1.7235	2.83
1298	*CDC20B*	5.184	3.7698	-0.47777	-2.1921	2.83
1299	*GRIA1*	5.0919	2.31	1.0735	-0.08301	2.83
1300	*ZFAT*	5.2738	3.3665	-0.16622	-1.9693	2.82
1301	*LIPC*	4.8323	2.4576	1.184	-1.2864	2.82
1302	*NEK2*	4.3853	3.0063	1.0821	-0.65417	2.82
1303	*IZUMO3*	5.4185	1.9225	1.1322	1.0872	2.82
1304	*EPHA7*	4.113	3.7221	0.63521	-1.622	2.82
1305	*CTRB2*	4.351	3.5716	0.54694	-0.58349	2.82
1306	*COMMD9*	2.9833	2.9114	2.5748	-1.9295	2.82
1307	*CLEC9A*	3.4066	3.0792	1.9836	-0.67458	2.82
1308	*HERPUD1*	4.8498	3.8271	-0.2098	-0.57133	2.82
1309	*MAPT*	3.6966	2.6792	2.0907	-1.1629	2.82
1310	*CEP63*	3.0465	2.8278	2.592	-2.372	2.82
1311	*MCM3AP*	4.8698	3.3412	0.25501	-0.95579	2.82
1312	*TPRA1*	4.2317	3.8951	0.33384	-0.19793	2.82
1313	*PHKA2*	4.9085	4.0662	-0.5144	-1.7994	2.82
1314	*PEX7*	4.5146	2.9018	1.0428	-0.71421	2.82
1315	*ZNF677*	4.4579	4.0742	-0.07436	-1.4936	2.82
1316	*RAB27B*	4.4394	2.6883	1.3298	-1.2342	2.82
1317	*TMBIM6*	4.5156	2.5037	1.4356	-1.8357	2.82
1318	*NECAB3*	3.2286	2.8672	2.3585	-0.51713	2.82
1319	*P3H1*	5.3196	2.229	0.90514	-0.66488	2.82
1320	*ALDH3B1*	5.394	3.9479	-0.8895	-1.7696	2.82
1321	*EPB41L2*	4.1973	3.8499	0.40435	-1.8842	2.82
1322	*ENDOG*	4.6211	4.0178	-0.18783	-2.7616	2.82
1323	*GSPT2*	3.4412	3.167	1.8424	-1.773	2.82
1324	*PPP2R2A*	4.3302	2.5245	1.5933	1.2979	2.82
1325	*NR2F1*	4.4606	3.6647	0.32236	-1.4607	2.82
1326	*PNMA3*	3.8525	3.7211	0.87394	-0.79678	2.82
1327	*CLEC12A*	3.5417	2.6411	2.2643	1.9213	2.82

1328	*KLRC4*	4.9605	3.1974	0.28605	-1.683	2.81
1329	*APPL2*	4.0112	2.4863	1.9461	-0.72819	2.81
1330	*ANAPC7*	3.1499	2.9025	2.3901	0.50427	2.81
1331	*HCRTR2*	3.4579	2.7287	2.2559	-0.26575	2.81
1332	*RIPK4*	3.9511	2.8588	1.6319	-0.42356	2.81
1333	*ZAR1*	4.0651	3.6147	0.76029	-1.2981	2.81
1334	*STEAP1*	3.4538	2.8569	2.1256	-0.42276	2.81
1335	*PRR4*	4.6279	3.4663	0.34044	0.22469	2.81
1336	*SEC14L2*	5.0485	3.0477	0.33838	-1.6926	2.81
1337	*DPP10*	3.9781	2.6682	1.7882	0.68145	2.81
1338	*CD24*	4.4206	2.7263	1.2862	0.38204	2.81
1339	*LAMA1*	5.2083	4.3321	-1.1075	-1.9558	2.81
1340	*KIAA1586*	4.3785	3.1965	0.85386	-0.8472	2.81
1341	*PCYOX1L*	4.2162	2.4085	1.8021	1.4331	2.81
1342	*GNRH2*	4.8808	3.2895	0.24705	-0.12998	2.81
1343	*SASS6*	5.166	2.3808	0.8688	-0.88818	2.81
1344	*CXCR6*	4.3343	2.4717	1.6091	-0.42181	2.81
1345	*TRIM56*	4.5218	3.0098	0.88094	-0.71093	2.80
1346	*RAC1*	4.014	3.4441	0.95209	-0.77418	2.80
1347	*FGD3*	4.4766	3.1629	0.77066	-1.2571	2.80
1348	*ZNF573*	4.0419	3.642	0.72381	-1.1953	2.80
1349	*OR5M10*	4.6001	2.9024	0.90518	-1.2662	2.80
1350	*EPYC*	3.877	3.3796	1.1509	0.28289	2.80
1351	*TDRD5*	5.2106	4.0887	-0.89219	-0.89988	2.80
1352	*WFDC8*	4.14	3.3624	0.90312	0.37605	2.80
1353	*FUT3*	4.8411	4.512	-0.94903	-1.6374	2.80
1354	*PDHA2*	3.953	2.5872	1.8638	-0.52042	2.80
1355	*ELAVL4*	4.8626	4.2346	-0.69678	-1.0388	2.80
1356	*CCNE2*	5.4593	3.4115	-0.47135	-0.85194	2.80
1357	*RNF103*	5.2594	2.4022	0.73769	-0.58597	2.80
1358	*ZFP82*	5.0217	3.0565	0.31975	-0.26383	2.80
1359	*PDZRN3*	3.7257	3.588	1.0839	0.1409	2.80
1360	*PCYOX1*	6.0076	2.5931	-0.20648	-0.52732	2.80
1361	*PCBD2*	3.0564	2.967	2.3705	0.45236	2.80
1362	*NXPH2*	4.2397	3.4905	0.66302	0.5806	2.80
1363	*HSPBP1*	3.8365	3.6208	0.93487	0.86057	2.80
1364	*CD6*	4.8978	4.2082	-0.71467	-1.7753	2.80
1365	*S100G*	4.6102	2.1729	1.6036	0.94601	2.80
1366	*ERBB2IP*	5.3468	2.1246	0.91528	-1.8961	2.80
1367	*OR2T8*	4.3014	2.6993	1.384	0.98094	2.79
1368	*BPY2C*	5.1925	4.1826	-0.99059	-1.3038	2.79
1369	*OGFRL1*	5.5437	3.0388	-0.19943	-0.42153	2.79
1370	*CNGB1*	4.9353	3.9479	-0.50053	-1.5485	2.79
1371	*FCAMR*	2.8954	2.8111	2.6749	2.0581	2.79
1372	*NXF5*	4.2545	3.8788	0.24698	-1.1591	2.79
1373	*CRYGB*	4.26	2.4257	1.6921	-2.3778	2.79

1374	*TPBG*	5.6164	2.8756	-0.11452	-0.43251	2.79
1375	*NXPE3*	4.8797	2.2295	1.2642	0.35003	2.79
1376	*RAI1*	3.9016	3.8814	0.58992	-0.0937	2.79
1377	*OLA1*	3.4264	3.018	1.9276	-1.0147	2.79
1378	*POMC*	4.2731	2.1651	1.9318	1.8788	2.79
1379	*NTS*	5.5041	1.869	0.9942	-1.1598	2.79
1380	*USF1*	4.3312	2.4149	1.6211	-0.07266	2.79
1381	*FXYD7*	3.8342	3.8256	0.70739	-1.3844	2.79
1382	*SCAMP3*	4.2334	3.2108	0.92165	-1.988	2.79
1383	*ROGDI*	4.6624	3.3687	0.33301	-1.5744	2.79
1384	*THAP5*	3.6323	3.0806	1.6496	1.0586	2.79
1385	*CCDC60*	4.2551	3.5873	0.51984	-2.2165	2.79
1386	*SKA1*	3.6656	3.5467	1.1478	-1.6352	2.79
1387	*ENOPH1*	3.2524	2.6192	2.4876	-0.72936	2.79
1388	*LRRC8C*	4.6322	2.7106	1.0159	-0.03346	2.79
1389	*SLC35F6*	5.0765	3.7351	-0.45296	-0.61222	2.79
1390	*GALNT8*	4.182	2.3117	1.8597	0.13609	2.78
1391	*SPINK14*	4.0886	2.2766	1.9875	-0.78959	2.78
1392	*PDZRN4*	3.4594	2.61	2.282	-0.7763	2.78
1393	*TLR10*	4.6269	3.2775	0.44601	-1.4123	2.78
1394	*TMUB2*	5.0108	2.3063	1.0311	-2.0258	2.78
1395	*FAM89B*	4.4146	4.2645	-0.33098	-2.0871	2.78
1396	*PCED1A*	5.4351	2.699	0.21153	-1.2917	2.78
1397	*CENPQ*	3.0037	2.7553	2.585	0.052809	2.78
1398	*BEX2*	4.5575	3.5197	0.26607	0.006223	2.78
1399	*ZNF84*	3.4105	3.0534	1.8793	0.65684	2.78
1400	*PRKAA1*	4.4865	3.2193	0.63592	-0.55492	2.78
1401	*MEF2BNB-MEF2B*	4.6151	2.0874	1.6372	-0.34051	2.78
1402	*SUCNR1*	4.1469	2.3184	1.8733	-0.42899	2.78
1403	*SOX14*	4.1246	3.8289	0.38458	-1.2676	2.78
1404	*CYP46A1*	3.3407	3.1724	1.8227	0.37505	2.78
1405	*SLC6A5*	5.5583	1.4878	1.2853	-1.4681	2.78
1406	*SYNE1*	4.4157	3.8159	0.09878	-0.90092	2.78
1407	*EPCAM*	3.8522	3.4337	1.0426	-0.03524	2.78
1408	*SPOCK3*	4.0412	3.4591	0.82643	-0.00546	2.78
1409	*SPRR2D*	4.4736	3.8793	-0.02643	-1.28	2.78
1410	*SVOP*	4.424	2.6917	1.21	-0.29562	2.78
1411	*CR1*	2.9317	2.8692	2.5242	1.145	2.78
1412	*TMEM247*	3.7351	3.5033	1.0866	-1.5671	2.78
1413	*MLLT11*	5.539	3.1512	-0.36569	-1.1825	2.77
1414	*FOXL2*	4.2046	2.7024	1.4171	1.0621	2.77
1415	*ARL6*	3.1736	2.7699	2.3795	-1.1699	2.77
1416	*GADD45B*	4.6501	2.3365	1.3354	-0.31317	2.77
1417	*FAM208B*	4.1013	2.6519	1.5657	-1.3952	2.77
1418	*ALDH3A1*	5.1778	3.7609	-0.62005	-0.62833	2.77
1419	*GABRB2*	5.4508	2.1427	0.72495	0.14269	2.77

1420	*TRABD*	5.0215	2.4368	0.85932	-1.4541	2.77
1421	*RAD51AP1*	3.2822	2.6131	2.4223	-2.3958	2.77
1422	*CDC25C*	3.6867	3.1337	1.4949	0.10279	2.77
1423	*C9orf64*	4.3322	2.9621	1.0184	-0.71988	2.77
1424	*SPATA7*	4.6121	2.3532	1.3457	-0.7722	2.77
1425	*VSIG2*	4.3328	2.1091	1.8652	0.63802	2.77
1426	*LHX6*	4.0632	3.4664	0.77367	-1.0516	2.77
1427	*GM2A*	4.3299	2.5805	1.3901	0.51513	2.77
1428	*TBC1D28*	4.4214	3.6466	0.23173	-0.59816	2.77
1429	*PTBP2*	3.5308	3.416	1.352	-0.84248	2.77
1430	*OLIG1*	3.7047	3.5762	1.0163	-0.158	2.77
1431	*RHBDD3*	3.4976	2.591	2.2066	-0.37215	2.77
1432	*OR1N2*	3.7794	3.6225	0.89246	-0.90296	2.76
1433	*EPHB6*	4.2718	3.3441	0.67678	-1.2086	2.76
1434	*PRCC*	4.6693	2.0919	1.5285	0.52501	2.76
1435	*ZMYM6NB*	4.6058	3.0984	0.58544	-0.3091	2.76
1436	*OR2Z1*	5.1908	1.6001	1.4976	-1.8547	2.76
1437	*BHLHA15*	2.9692	2.8096	2.5079	-0.6714	2.76
1438	*KCNN1*	3.6702	3.2059	1.409	-0.67913	2.76
1439	*DNASE1L3*	4.0443	3.7027	0.53765	-1.8775	2.76
1440	*ZNF878*	3.6511	3.0912	1.5421	-0.68757	2.76
1441	*E2F4*	4.5022	3.614	0.1674	-0.63845	2.76
1442	*C3orf58*	5.256	1.6273	1.398	-1.2267	2.76
1443	*GLRA1*	4.2667	3.5244	0.48998	-1.9675	2.76
1444	*GPM6B*	4.4201	4.2234	-0.36315	-0.61663	2.76
1445	*TNR*	4.1678	3.7344	0.37721	-0.22962	2.76
1446	*SMIM21*	4.7128	3.4943	0.07209	-1.2208	2.76
1447	*PDILT*	5.0654	4.0896	-0.87817	-1.7662	2.76
1448	*OR2T5*	3.9799	3.2483	1.0461	0.10959	2.76
1449	*OXR1*	4.3467	3.1132	0.81408	-0.13823	2.76
1450	*FAXC*	4.1946	2.8641	1.2147	0.24958	2.76
1451	*DNAJC4*	3.3418	2.9007	2.0261	-2.1151	2.76
1452	*ZNF500*	4.1709	3.4386	0.65887	-1.1209	2.76
1453	*CTSV*	3.8667	3.7265	0.67455	0.40785	2.76
1454	*TAZ*	3.0226	2.7064	2.533	1.9525	2.75
1455	*KIAA1257*	2.9372	2.8613	2.4622	-0.09762	2.75
1456	*AMZ2*	3.6711	3.3007	1.2875	-0.44134	2.75
1457	*ACKR4*	4.9048	3.2031	0.14969	0.071915	2.75
1458	*PLA2G4A*	3.713	2.8545	1.6896	0.35324	2.75
1459	*MFSD7*	5.3812	2.8146	0.05963	-0.78429	2.75
1460	*ENTPD2*	4.9631	3.1317	0.15808	-0.5206	2.75
1461	*PUF60*	3.2192	2.7291	2.3045	-0.23653	2.75
1462	*TSGA10IP*	4.0561	2.2631	1.9332	-0.42349	2.75
1463	*SPTLC3*	3.2415	2.962	2.0481	1.0363	2.75
1464	*CKLF*	4.7547	3.5675	-0.07138	-0.46372	2.75
1465	*OC90*	4.9374	2.9385	0.37296	-1.9748	2.75

1466	*GRIA3*	4.1808	2.6983	1.3674	0.34861	2.75
1467	*KCNN2*	3.3913	3.109	1.7433	-1.2426	2.75
1468	*SEMA4B*	3.3535	2.7446	2.1454	1.9195	2.75
1469	*UGT1A4*	3.523	3.1532	1.5637	-1.4749	2.75
1470	*VPS4B*	4.3854	3.6583	0.19594	-2.2951	2.75
1471	*DIDO1*	5.2027	2.3244	0.71144	-0.40097	2.75
1472	*IRF8*	4.4528	3.6245	0.16049	-1.7552	2.75
1473	*SRM*	4.7595	3.6755	-0.19943	-0.94113	2.75
1474	*NAGPA*	4.5644	2.486	1.1842	0.64483	2.74
1475	*UBE2W*	3.131	3.1078	1.9953	0.70365	2.74
1476	*PDRG1*	3.6291	3.5344	1.0705	-0.33152	2.74
1477	*PNLIP*	3.7207	2.9862	1.5237	0.30216	2.74
1478	*DNMT3B*	4.684	3.5634	-0.01699	-0.91269	2.74
1479	*ZIC1*	3.9849	2.1641	2.0814	0.18044	2.74
1480	*SPATA6*	4.3547	2.3832	1.4919	1.2313	2.74
1481	*TRMT5*	3.2543	2.5314	2.4421	2.3176	2.74
1482	*TCTEX1D4*	5.4376	2.7664	0.02334	-1.3139	2.74
1483	*SAT2*	4.4782	3.4213	0.32523	-1.5116	2.74
1484	*TAAR1*	3.5476	2.8525	1.8245	-0.32122	2.74
1485	*A4GNT*	5.3949	3.9912	-1.162	-2.0337	2.74
1486	*ALPK2*	4.6706	4.4304	-0.87898	-1.917	2.74
1487	*EXD3*	4.5957	2.2215	1.4019	-0.56634	2.74
1488	*FOXL2NB*	5.0109	3.2225	-0.01548	-2.6188	2.74
1489	*HHLA3*	4.054	2.0978	2.0661	-0.96463	2.74
1490	*FOXRED2*	5.2565	2.2307	0.73007	0.25632	2.74
1491	*TMEM196*	3.99	3.6065	0.61939	-0.32537	2.74
1492	*MRPL15*	3.8726	3.403	0.94	-1.7592	2.74
1493	*EGFR*	3.8422	3.6305	0.74126	-0.26711	2.74
1494	*REEP5*	4.2373	2.186	1.7897	0.37134	2.74
1495	*CYS1*	2.9117	2.7733	2.526	0.40961	2.74
1496	*TECPR1*	3.9643	3.1865	1.06	0.30312	2.74
1497	*LOXL1*	4.1872	4.1767	-0.15385	-0.35694	2.74
1498	*OGG1*	3.9137	3.7554	0.54046	-0.66604	2.74
1499	*BBC3*	5.0448	2.0652	1.0983	-0.89593	2.74
1500	*TMEM74B*	5.0654	2.6504	0.49197	-1.4529	2.74
1501	*KIF13A*	3.7945	2.9378	1.475	-0.59025	2.74
1502	*CRYGD*	5.5225	4.3463	-1.6635	-2.4157	2.74
1503	*CCR1*	3.4802	3.4542	1.2691	-1.5876	2.73
1504	*SRF*	4.42	2.4434	1.3392	-1.9252	2.73
1505	*PRSS36*	3.563	3.4453	1.1936	-0.95573	2.73
1506	*QSOX1*	4.2678	3.1164	0.81744	-1.638	2.73
1507	*CEP192*	3.8976	2.8897	1.4143	-2.1445	2.73
1508	*DPCD*	4.4906	2.5808	1.1294	-0.1855	2.73
1509	*FCRL2*	4.3651	3.8576	-0.0249	-0.09906	2.73
1510	*MTMR14*	4.3312	2.5675	1.2956	-0.56098	2.73
1511	*DPPA5*	5.1715	2.2434	0.77879	-0.60065	2.73

1512	*ANP32E*	3.8983	2.9283	1.3666	-1.7049	2.73
1513	*CLEC16A*	5.445	1.4434	1.3036	-0.85551	2.73
1514	*BSN*	3.9011	3.3599	0.9297	0.65767	2.73
1515	*TTLL3*	5.185	2.7836	0.22099	-0.85739	2.73
1516	*ZNF799*	3.6319	2.4586	2.0975	-1.8484	2.73
1517	*NMRK1*	4.2095	4.0311	-0.05387	-0.55359	2.73
1518	*CHRNA7*	4.285	2.482	1.4192	0.80859	2.73
1519	*MTO1*	4.1626	3.4753	0.5469	-1.6711	2.73
1520	*SUV39H2*	4.9112	2.3641	0.90894	-1.4597	2.73
1521	*PCOLCE2*	3.7377	2.7833	1.6605	-1.0028	2.73
1522	*SUMO4*	4.9533	1.8204	1.4074	-1.0932	2.73
1523	*TRAPPC13*	4.4319	3.0784	0.66958	0.27172	2.73
1524	*OR11L1*	3.3798	3.271	1.5239	-1.0373	2.72
1525	*RIOK1*	5.1222	2.9546	0.09624	-1.7268	2.72
1526	*TRIM24*	4.5346	2.4808	1.1569	0.85373	2.72
1527	*ESX1*	4.0221	4.0174	0.12923	-1.3387	2.72
1528	*HK1*	4.695	2.0095	1.4591	-1.0529	2.72
1529	*LPPR5*	3.7576	2.8793	1.5244	-1.1709	2.72
1530	*ALPI*	5.2302	3.4634	-0.53332	-1.1545	2.72
1531	*PTH1R*	4.5869	3.8687	-0.29638	-1.2277	2.72
1532	*RHOBTB3*	5.1454	2.3289	0.6828	0.35637	2.72
1533	*CCDC106*	5.2636	3.2218	-0.32849	-0.95036	2.72
1534	*STK17B*	4.9495	2.8009	0.40276	-0.53254	2.72
1535	*FMN1*	3.7811	3.0507	1.3191	0.49269	2.72
1536	*RAB23*	4.2238	4.0326	-0.10619	-0.42361	2.72
1537	*OR1G1*	4.1745	3.0766	0.89846	-0.73259	2.72
1538	*KRTAP5-6*	4.4308	3.7045	0.01374	-0.26302	2.72
1539	*GSTO2*	3.5282	3.1812	1.4388	0.89403	2.72
1540	*CNN1*	3.539	2.8849	1.722	-1.5672	2.72
1541	*KCNK16*	5.9073	2.7392	-0.50062	-0.72168	2.72
1542	*FBXO32*	4.103	2.0764	1.9655	0.26709	2.71
1543	*CEACAM8*	3.6445	3.4803	1.02	0.87156	2.71
1544	*C12orf57*	2.9718	2.6216	2.5493	2.2731	2.71
1545	*AMPH*	4.8977	1.9093	1.3351	-1.5095	2.71
1546	*OSBP*	4.7544	2.7001	0.68723	-0.54515	2.71
1547	*STIL*	3.9181	2.4127	1.8103	-1.9563	2.71
1548	*AGR2*	4.8379	3.4284	-0.12651	-0.79149	2.71
1549	*C8orf4*	3.9493	2.6492	1.5411	-2.1983	2.71
1550	*TOR2A*	5.3211	1.892	0.92478	-1.6741	2.71
1551	*VCX3A*	4.2343	3.7514	0.1517	-0.89474	2.71
1552	*TSPYL1*	4.8135	3.2261	0.09568	-0.19842	2.71
1553	*GJB1*	4.4758	3.0234	0.6356	-1.4473	2.71
1554	*AGAP3*	3.5723	2.5575	2.0049	-1.1964	2.71
1555	*GOLGA6L2*	3.612	2.551	1.9699	1.848	2.71
1556	*PCSK1*	3.1594	3.0362	1.9372	0.88166	2.71
1557	*SPDYE4*	4.033	3.2424	0.85729	-0.71393	2.71

1558	*RER1*	3.039	2.8069	2.283	-0.49155	2.71
1559	*OR8K1*	3.7137	3.275	1.1385	-1.5984	2.71
1560	*MYO3B*	4.0381	3.0398	1.0431	0.28497	2.71
1561	*SLC17A6*	4.5082	2.2162	1.3946	-0.58274	2.71
1562	*SEC24B*	3.8517	2.6013	1.6659	-0.79149	2.71
1563	*CPLX2*	3.6799	2.9398	1.4983	1.1743	2.71
1564	*TMEM106A*	4.1249	3.7567	0.23424	-1.7725	2.71
1565	*CDC169-SOHLH2*	4.727	3.4397	-0.05133	-1.4142	2.71
1566	*NPB*	3.5152	2.9454	1.6492	1.2316	2.70
1567	*C8orf59*	3.7162	2.8708	1.522	-0.87997	2.70
1568	*NLGN1*	4.2287	3.1332	0.74684	-2.7442	2.70
1569	*CBS*	3.897	2.216	1.9952	-1.6796	2.70
1570	*NIF3L1*	2.9967	2.6109	2.4979	-0.95505	2.70
1571	*MORF4L1*	3.6808	2.6065	1.8169	-0.63097	2.70
1572	*TBC1D19*	3.3486	2.8889	1.8658	-2.0267	2.70
1573	*TSTD1*	5.2369	3.1699	-0.30424	-1.8154	2.70
1574	*ESPL1*	3.1387	2.5246	2.4385	2.0758	2.70
1575	*WDR38*	4.9502	4.4774	-1.3288	-2.0574	2.70
1576	*CP*	5.0152	2.4516	0.63111	0.57403	2.70
1577	*SLC25A11*	3.9664	3.7502	0.38081	-0.99892	2.70
1578	*FMN2*	3.3374	3.3361	1.4226	-0.57766	2.70
1579	*NUTM2G*	3.4662	3.4467	1.1821	-1.4747	2.70
1580	*TCEAL3*	3.7576	2.2081	2.129	-0.32811	2.70
1581	*PDCL*	3.8831	3.5477	0.66088	-1.1346	2.70
1582	*SLAMF6*	4.471	3.3615	0.25714	-0.69972	2.70
1583	*ZNF33A*	3.7272	3.4188	0.94361	0.65601	2.70
1584	*GCLC*	4.1087	4.034	-0.05369	-1.1501	2.70
1585	*IFT20*	4.2822	3.1235	0.68273	-0.25605	2.70
1586	*DMD*	3.3774	2.4249	2.2834	0.58032	2.70
1587	*FGFR1OP*	2.9978	2.7283	2.3586	0.33236	2.69
1588	*B3GNT9*	4.6023	2.7449	0.73377	-0.02412	2.69
1589	*FAM19A1*	4.1094	3.4239	0.5469	-1.2437	2.69
1590	*MLKL*	4.5947	3.4841	-0.00032	-1.0832	2.69
1591	*SOCS3*	4.4225	2.8245	0.83078	-0.71645	2.69
1592	*CNTNAP2*	5.1814	1.4899	1.4028	-1.2455	2.69
1593	*OR5H6*	5.1603	4.0801	-1.1665	-1.843	2.69
1594	*ZDHHC24*	4.285	4.0031	-0.21434	-0.23105	2.69
1595	*CORO1B*	4.453	3.9732	-0.35367	-1.3161	2.69
1596	*CST4*	4.1266	3.3093	0.63448	0.15099	2.69
1597	*UST*	3.7505	2.5884	1.7292	-1.714	2.69
1598	*OR4C15*	3.7677	3.007	1.2927	-1.7438	2.69
1599	*ERAP1*	3.6035	3.5743	0.88735	-0.3791	2.69
1600	*LRRC37A3*	4.1083	3.4173	0.53898	-1.7994	2.69
1601	*HNRNPH2*	4.4353	2.3267	1.3021	-0.47407	2.69
1602	*SRSF9*	3.6116	3.1241	1.3251	0.75933	2.69
1603	*CYBRD1*	4.3609	1.8669	1.8323	-0.25493	2.69

1604	*LY6G5B*	4.8234	1.6592	1.5761	-0.30692	2.69
1605	*MS4A3*	5.0366	3.8677	-0.84652	-1.0914	2.69
1606	*KANSL1L*	4.058	2.241	1.7573	0.2293	2.69
1607	*HIST4H4*	4.1547	2.4565	1.4421	-0.22269	2.68
1608	*GPRASP2*	4.2502	3.4073	0.39196	-1.3784	2.68
1609	*HES3*	4.3011	2.3552	1.392	0.29116	2.68
1610	*ITM2B*	4.7955	3.6117	-0.36057	-0.4139	2.68
1611	*SMPDL3A*	3.9434	2.9168	1.1858	0.1919	2.68
1612	*CCDC144A*	5.3128	2.0264	0.70596	-1.5635	2.68
1613	*SHMT2*	4.1292	3.188	0.7269	-0.61132	2.68
1614	*CCDC14*	4.5502	4.4718	-0.98062	-1.8016	2.68
1615	*SEPT14*	4.2525	3.8011	-0.01245	-0.95722	2.68
1616	*ITGA5*	4.1672	3.0995	0.7744	-1.3533	2.68
1617	*KIF5B*	4.5299	2.6159	0.89528	-1.1077	2.68
1618	*DDX21*	5.0098	3.6902	-0.66008	-1.3795	2.68
1619	*ZDHHC1*	5.7836	1.6347	0.6216	-1.7991	2.68
1620	*SYCE3*	3.9501	3.1578	0.9318	-1.671	2.68
1621	*SULT1B1*	4.7505	2.9398	0.34851	-0.80754	2.68
1622	*ARL5B*	5.4221	4.406	-1.7945	-2.4322	2.68
1623	*CHP2*	3.8619	3.6458	0.52471	-2.9417	2.68
1624	*HDHD1*	3.5105	2.3061	2.2143	2.0325	2.68
1625	*CERS3*	5.3852	3.5162	-0.87208	-1.2193	2.68
1626	*C7orf65*	3.3816	2.5119	2.1357	0.91529	2.68
1627	*PRH2*	4.9843	2.5057	0.53795	-0.14019	2.68
1628	*NUP62CL*	3.4121	2.5797	2.0338	0.34077	2.68
1629	*TRIB1*	6.0406	3.2836	-1.2988	-3.7834	2.68
1630	*PHLDB3*	4.7924	3.5077	-0.27663	-2.2809	2.67
1631	*OR8K3*	4.9193	2.0665	1.0327	-0.67235	2.67
1632	*RIPK2*	3.643	3.4346	0.94035	-1.061	2.67
1633	*TRIM49*	3.6981	2.5922	1.7274	-2.1239	2.67
1634	*THAP4*	4.1282	2.9554	0.93222	0.57874	2.67
1635	*ZNF430*	3.7983	2.752	1.4627	-3.2089	2.67
1636	*SEC16B*	4.5208	3.1145	0.37723	-0.70187	2.67
1637	*MAS1*	4.9824	2.2457	0.78415	0.54456	2.67
1638	*LHX9*	3.2969	3.203	1.508	-0.6501	2.67
1639	*TSPAN6*	5.1156	4.3748	-1.4842	-2.2669	2.67
1640	*C1QTNF6*	4.5192	2.5793	0.9075	-1.5375	2.67
1641	*TERF2IP*	3.8445	2.9909	1.1701	-1.6347	2.67
1642	*AEBP1*	4.7014	2.5521	0.75163	-0.80248	2.67
1643	*CLEC2A*	2.9928	2.9647	2.0474	0.7832	2.67
1644	*ERI1*	4.1647	2.8185	1.0214	-3.4433	2.67
1645	*MAML2*	3.769	3.1421	1.0927	-0.24488	2.67
1646	*NME1*	3.9996	3.4122	0.58825	-0.8303	2.67
1647	*RUNX1T1*	4.101	4.0673	-0.1685	-1.5304	2.67
1648	*SEMA3B*	4.5208	4.2266	-0.74815	-1.35	2.67
1649	*TOR3A*	4.8939	3.8166	-0.71268	-0.76965	2.67

1650	*KCNJ16*	2.9869	2.7383	2.2718	0.86274	2.67
1651	*SPANXB1*	4.3997	3.1733	0.42339	-0.95068	2.67
1652	*TUBA8*	4.3505	3.2511	0.39392	0.29569	2.67
1653	*MKRN2OS*	4.0344	3.6364	0.3228	-2.5048	2.66
1654	*TMEM225*	3.9988	3.0529	0.94015	-1.6027	2.66
1655	*VASH2*	3.0373	2.7749	2.1771	0.65861	2.66
1656	*DDRGK1*	4.7775	4.3161	-1.1053	-1.2989	2.66
1657	*SIN3B*	4.1192	3.8951	-0.02602	-1.0175	2.66
1658	*TMPRSS11D*	3.8107	3.216	0.96023	-0.61839	2.66
1659	*LMO7*	4.6597	2.707	0.6201	-1.6907	2.66
1660	*GAK*	3.2293	3.2058	1.5511	-0.52825	2.66
1661	*RIBC1*	3.1952	2.6694	2.1205	0.59638	2.66
1662	*ANKFN1*	4.5797	2.0095	1.3956	0.42942	2.66
1663	*FBXO18*	4.0969	2.2804	1.6048	0.54427	2.66
1664	*OR1J1*	4.155	2.0908	1.735	-0.33524	2.66
1665	*PPP2R5C*	5.2502	1.7584	0.96984	0.5847	2.66
1666	*C4A*	4.4247	2.835	0.71813	-1.4685	2.66
1667	*HOXA6*	4.0024	2.7405	1.2346	-0.3306	2.66
1668	*TMED8*	4.3429	3.6063	0.02805	-0.00832	2.66
1669	*MTSS1L*	4.1858	2.8558	0.93345	-0.43974	2.66
1670	*RWDD1*	3.9609	3.078	0.93552	-0.68144	2.66
1671	*RBM12B*	3.8843	2.8992	1.1908	-1.2537	2.66
1672	*MAPK15*	3.5727	3.4714	0.9268	-2.3577	2.66
1673	*MYO1H*	4.9682	3.8145	-0.81193	-2.3602	2.66
1674	*ANKRD1*	4.8209	2.8666	0.28169	-1.0005	2.66
1675	*MAP7D1*	3.3976	2.7709	1.7991	1.3904	2.66
1676	*SMIM19*	3.5841	3.3383	1.045	-0.32779	2.66
1677	*DHRS4*	3.954	3.4574	0.55396	-1.0458	2.66
1678	*KCNMB3*	3.8579	2.0986	2.007	-0.27761	2.65
1679	*TSPAN4*	3.9529	2.308	1.7018	0.51807	2.65
1680	*HDAC4*	3.3499	2.6552	1.9553	-0.0398	2.65
1681	*FHDC1*	4.3348	2.6154	1.0095	0.73531	2.65
1682	*SYT9*	3.3168	2.3933	2.2493	-0.31411	2.65
1683	*GABRD*	3.784	2.629	1.5462	0.78273	2.65
1684	*BHMT2*	3.5341	3.1847	1.2391	-1.6076	2.65
1685	*SEMA3C*	5.4727	1.9377	0.54647	-1.1027	2.65
1686	*SCD5*	4.9258	3.0008	0.02904	-0.66635	2.65
1687	*TNRC6B*	4.3799	3.3461	0.22814	-0.02683	2.65
1688	*ZBTB25*	4.1303	3.1789	0.64197	-0.61963	2.65
1689	*MTCP1*	4.4978	4.0091	-0.5567	-1.1156	2.65
1690	*OR2F2*	2.6867	2.6601	2.6033	-1.2305	2.65
1691	*MTERF4*	3.7198	3.3823	0.8464	-1.5848	2.65
1692	*WISP2*	4.8921	2.21	0.84594	-0.65971	2.65
1693	*DCBLD2*	4.0081	2.2126	1.7271	-0.42573	2.65
1694	*SMIM10*	4.0028	2.6472	1.2977	-0.93967	2.65
1695	*TBC1D1*	5.6843	1.7094	0.55371	-1.8022	2.65

1696	*STPG1*	3.1955	2.6093	2.1391	-0.9382	2.65
1697	*SAA2-SAA4*	4.669	3.4803	-0.20721	-0.3433	2.65
1698	*EXPH5*	4.3615	2.5968	0.98351	0.34821	2.65
1699	*PYROXD1*	3.8075	3.034	1.098	-0.95701	2.65
1700	*HAS1*	4.4024	2.9489	0.58535	-1.5237	2.65
1701	*PRR29*	4.272	3.4769	0.18588	-0.27651	2.64
1702	*BCL9*	4.968	2.7429	0.22243	-1.0564	2.64
1703	*S1PR1*	4.2646	2.1768	1.4905	-1.4584	2.64
1704	*TMEM217*	4.2238	3.3874	0.31759	-0.95918	2.64
1705	*CBLN3*	3.9871	2.167	1.7738	-2.3452	2.64
1706	*ANKRD62*	3.1361	2.5959	2.1953	-0.41835	2.64
1707	*HMGXB4*	4.1857	3.8203	-0.07895	-1.1995	2.64
1708	*GDNF-AS1*	4.2777	2.9267	0.72251	-1.7766	2.64
1709	*CPXM1*	4.3461	2.8487	0.73137	-0.1136	2.64
1710	*G0S2*	4.6971	1.6889	1.5393	1.4797	2.64
1711	*HCAR3*	4.267	2.9982	0.65987	-0.19423	2.64
1712	*TMEM256*	4.4791	2.075	1.3692	-1.1201	2.64
1713	*TMEM183B*	4.2143	3.2844	0.42153	-0.76169	2.64
1714	*OR2A42*	3.1914	3.1512	1.5758	0.77768	2.64
1715	*SLC9C1*	4.1882	3.4664	0.2614	0.23329	2.64
1716	*C5orf52*	3.8356	3.1449	0.93523	-1.3176	2.64
1717	*SLC35G1*	2.993	2.605	2.3176	-0.34998	2.64
1718	*RDH14*	4.2936	2.7264	0.89402	0.80623	2.64
1719	*KLK15*	3.5436	2.7933	1.5771	-1.5663	2.64
1720	*C5orf30*	4.3914	3.1386	0.38066	-0.4102	2.64
1721	*PTCHD2*	4.8963	2.5491	0.46373	-0.78439	2.64
1722	*GBP4*	3.6761	3.2671	0.96447	-0.12601	2.64
1723	*GGTLC1*	4.4604	2.6037	0.84349	0.036324	2.64
1724	*DLGAP5*	4.261	2.8667	0.77594	-1.1027	2.63
1725	*GABRR1*	3.5613	3.1625	1.1795	-0.4158	2.63
1726	*SH3BP2*	4.3474	3.2772	0.27699	-2.3696	2.63
1727	*FHOD1*	4.3457	3.0595	0.4959	0.12927	2.63
1728	*GTF2A1L*	4.8502	3.4993	-0.4488	-1.5383	2.63
1729	*NINJ1*	3.8909	3.5481	0.46124	-1.7757	2.63
1730	*FMNL3*	3.9127	3.9017	0.08381	-0.59061	2.63
1731	*HTRA2*	4.9849	3.1897	-0.2765	-1.6417	2.63
1732	*ZNF716*	4.1027	2.5679	1.2274	0.97075	2.63
1733	*HES7*	3.7173	3.6432	0.5365	-0.89866	2.63
1734	*ATOH8*	3.9522	3.1866	0.75794	0.41567	2.63
1735	*GTDC1*	4.7159	2.9041	0.27454	0.098257	2.63
1736	*IL5RA*	4.2272	2.5294	1.1371	-0.52968	2.63
1737	*IGFBP1*	5.4159	3.0276	-0.55184	-1.8884	2.63
1738	*CATSPERG*	4.9844	1.824	1.0823	-0.54786	2.63
1739	*CRYBB2*	4.1271	3.7484	0.01415	-1.3287	2.63
1740	*PICK1*	4.5417	2.6268	0.71885	-1.5635	2.63
1741	*TUBGCP3*	5.1924	3.3469	-0.65198	-1.6877	2.63

1742	*ZNF705B*	3.5275	3.1235	1.236	-1.0599	2.63
1743	*PGLYRP2*	3.6503	3.3419	0.89416	-0.22378	2.63
1744	*ITGA2B*	4.2186	2.8224	0.84507	0.23912	2.63
1745	*FOXQ1*	5.0543	3.439	-0.60752	-1.3042	2.63
1746	*LRRC3B*	3.9608	3.7256	0.19646	-1.4192	2.63
1747	*IL1RAPL2*	3.6931	2.7228	1.4657	-1.0477	2.63
1748	*OR4K5*	3.3688	2.5943	1.9181	-0.89384	2.63
1749	*OR5C1*	4.2067	2.6827	0.99069	-1.0221	2.63
1750	*C15orf48*	5.7195	3.3734	-1.2152	-1.5059	2.63
1751	*CFAP69*	4.0608	2.5086	1.308	-0.71526	2.63
1752	*CRP*	3.5432	2.3377	1.9964	-0.71363	2.63
1753	*ZNF585B*	3.4493	3.1472	1.2774	0.25994	2.62
1754	*IGFBPL1*	4.4856	1.8195	1.5686	-1.1585	2.62
1755	*FAM72D*	4.3293	1.9205	1.6232	-1.7257	2.62
1756	*CEACAM21*	5.443	1.4698	0.9562	0.46987	2.62
1757	*PIP5K1A*	5.3874	2.1508	0.32923	-0.33638	2.62
1758	*OR52I2*	4.6763	3.2668	-0.07644	-0.95802	2.62
1759	*FAM131B*	4.2597	1.8218	1.784	0.4557	2.62
1760	*TRMT61A*	4.9555	4.0323	-1.1223	-1.3139	2.62
1761	*SLC30A10*	5.0545	1.9741	0.83488	-0.99175	2.62
1762	*SEC23A*	4.4645	3.2579	0.13956	-0.89294	2.62
1763	*EEF1E1*	4.0799	2.4685	1.3107	-0.34111	2.62
1764	*SLC17A7*	4.135	3.221	0.5013	-0.73979	2.62
1765	*TMEM102*	3.6347	2.9437	1.2787	-0.55975	2.62
1766	*VN1R5*	4.3285	1.9882	1.5384	-0.02451	2.62
1767	*ITGB8*	4.4635	3.4054	-0.01399	-0.14196	2.62
1768	*GABRA5*	3.9914	2.4297	1.4319	0.23824	2.62
1769	*CCL1*	4.8534	1.9002	1.0985	-1.8462	2.62
1770	*NCK1*	5.456	1.6145	0.781	-1.6597	2.62
1771	*ANO6*	5.3498	1.2887	1.2104	0.59175	2.62
1772	*MAPK6*	6.2181	2.4437	-0.81329	-1.0019	2.62
1773	*SLC23A2*	3.4779	2.6135	1.7571	-1.2241	2.62
1774	*SGCG*	3.5633	2.8971	1.3879	-0.17048	2.62
1775	*TGFB1I1*	5.0439	1.4604	1.3438	1.2807	2.62
1776	*CITED2*	2.8735	2.788	2.1848	1.0236	2.62
1777	*BTAF1*	3.6089	2.8233	1.4137	-0.47864	2.62
1778	*FKBP1B*	2.9673	2.7617	2.1114	1.2803	2.61
1779	*CHADL*	5.7516	1.9147	0.17372	-2.4172	2.61
1780	*OR10S1*	4.6259	3.6048	-0.39313	-0.62017	2.61
1781	*NEDD4*	5.217	2.5255	0.09472	-0.76366	2.61
1782	*INHBB*	3.9925	2.7987	1.045	-0.67691	2.61
1783	*TM4SF4*	4.3579	2.5115	0.96666	-1.1576	2.61
1784	*ST7*	3.1981	2.9024	1.7353	-0.35699	2.61
1785	*CASP10*	5.4336	2.0469	0.35343	-1.7994	2.61
1786	*C2orf91*	3.8893	2.4834	1.4607	-1.2169	2.61
1787	*TFF1*	3.2883	2.6976	1.8474	-0.27194	2.61

1788	*BMP7*	4.7022	2.3394	0.7902	-0.63242	2.61
1789	*OR6K2*	4.6845	2.219	0.92709	-1.3407	2.61
1790	*SLC28A1*	3.6067	3.5501	0.67222	-0.91644	2.61
1791	*CALHM1*	3.2122	2.3136	2.303	-0.28661	2.61
1792	*NABP1*	4.4656	2.332	1.0307	-1.3174	2.61
1793	*ZNF480*	4.2711	3.9677	-0.41123	-0.89218	2.61
1794	*KDM5A*	3.5027	3.149	1.1757	-1.047	2.61
1795	*F8*	3.841	3.2926	0.69327	0.16704	2.61
1796	*B3GNT8*	4.1337	2.9687	0.72251	-1.227	2.61
1797	*CELA1*	2.9116	2.5894	2.3219	-2.3904	2.61
1798	*HOXA11*	4.7559	3.2165	-0.14969	-0.17042	2.61
1799	*SCG2*	5.1785	1.4104	1.2325	-1.5763	2.61
1800	*LPXN*	3.1008	2.4545	2.2647	0.59448	2.61
1801	*PRKD2*	5.096	2.8255	-0.10176	-0.84229	2.61
1802	*C1orf64*	4.5261	3.5826	-0.28963	-0.47665	2.61
1803	*SCN2A*	4.1085	1.8815	1.8288	-0.99736	2.61
1804	*CDK14*	3.3357	2.7332	1.7496	-1.0436	2.61
1805	*PPIB*	4.1971	2.9426	0.67721	0.031969	2.61
1806	*ZDHHC19*	3.5285	3.5233	0.76041	-0.8929	2.60
1807	*MRI1*	4.8722	4.0551	-1.1189	-1.4258	2.60
1808	*CYP7B1*	4.1595	3.0533	0.59497	-1.5582	2.60
1809	*APOM*	4.3194	3.8081	-0.32061	-0.43505	2.60
1810	*PLA2G4D*	5.1957	2.7235	-0.11411	-1.95	2.60
1811	*GAPVD1*	3.8074	3.3653	0.63031	-1.2009	2.60
1812	*HMG20B*	3.5241	3.3617	0.91478	0.16024	2.60
1813	*PPARD*	3.1361	2.8832	1.7801	-0.97064	2.60
1814	*LRRC15*	4.661	3.2831	-0.1449	-1.4682	2.60
1815	*TMEM31*	4.384	2.6835	0.72983	-1.0654	2.60
1816	*MIP*	5.0714	3.251	-0.52741	-1.7589	2.60
1817	*SYT17*	4.2995	4.1237	-0.62875	-0.72674	2.60
1818	*SLC10A5*	4.8874	3.0877	-0.18146	-1.1223	2.60
1819	*MBD2*	3.8966	2.0117	1.8853	1.2066	2.60
1820	*DPM2*	4.6288	2.9057	0.25866	-1.6755	2.60
1821	*ST14*	3.5716	3.1945	1.0267	0.12137	2.60
1822	*SERTAD4*	5.2015	3.5348	-0.94583	-1.9678	2.60
1823	*KCNJ13*	3.6584	2.313	1.819	-1.3941	2.60
1824	*PDE5A*	4.0978	2.377	1.3147	0.72992	2.60
1825	*VEPH1*	4.179	3.6689	-0.06095	-1.7884	2.60
1826	*LOX*	3.7497	3.3495	0.6871	-0.88839	2.60
1827	*FAM219B*	5.1274	1.5848	1.0721	-1.3818	2.59
1828	*SH2D4B*	4.7057	2.7261	0.3521	-1.3481	2.59
1829	*ING2*	4.7701	3.0474	-0.0343	-0.12189	2.59
1830	*SLC26A9*	4.149	3.8538	-0.22131	-0.63196	2.59
1831	*ARHGEF3*	4.0608	2.8935	0.82678	-1.3371	2.59
1832	*GBX2*	3.5282	3.4063	0.8465	0.38164	2.59
1833	*JAK2*	3.6571	2.2608	1.8625	-1.7852	2.59

1834	*ZNF34*	4.1757	1.9293	1.6744	1.1214	2.59
1835	*TINAG*	4.1593	3.2373	0.38198	-0.30293	2.59
1836	*RPS6KA2*	3.6549	3.0018	1.1204	-0.60162	2.59
1837	*TMEM27*	2.8407	2.6821	2.254	1.0859	2.59
1838	*FAM43A*	4.1779	3.3179	0.27973	-0.85471	2.59
1839	*CES1*	4.1579	2.5533	1.0642	-1.2301	2.59
1840	*EDRF1*	4.6873	2.9101	0.17513	-0.61767	2.59
1841	*TIMM8B*	3.8445	2.8041	1.1222	0.020906	2.59
1842	*AHNAK2*	4.768	4.5903	-1.5894	-2.1709	2.59
1843	*CBX2*	5.2224	3.2554	-0.70976	-1.3472	2.59
1844	*ZNF578*	4.6926	3.7865	-0.71291	-1.439	2.59
1845	*GPN3*	4.7027	3.387	-0.32378	-0.44353	2.59
1846	*TSPO*	3.9515	2.8085	1.0055	0.78012	2.59
1847	*TDRD3*	5.5057	2.289	-0.02993	-1.882	2.59
1848	*ZNF621*	3.3535	2.4055	2.0052	-1.3978	2.59
1849	*TACO1*	3.7508	3.0538	0.95925	-0.74442	2.59
1850	*P2RY8*	4.3209	1.844	1.5988	0.56084	2.59
1851	*LYRM9*	4.0407	4.0108	-0.28823	-1.3184	2.59
1852	*FSTL3*	3.9687	2.5088	1.2852	-0.86694	2.59
1853	*CLMN*	4.722	2.4538	0.58443	0.49493	2.59
1854	*TSPEAR*	3.9947	2.5262	1.2379	-0.64164	2.59
1855	*NFE2*	3.3319	2.8639	1.562	-0.76074	2.59
1856	*FER*	4.4597	3.9499	-0.65389	-0.70927	2.59
1857	*MT3*	3.5559	2.4867	1.7118	-1.8975	2.58
1858	*RARRES2*	3.0903	2.4314	2.2326	1.0804	2.58
1859	*TOP2B*	4.2924	2.5446	0.91584	0.14574	2.58
1860	*ADGRD1*	4.9427	3.2515	-0.44353	-1.6525	2.58
1861	*PFN1*	3.5824	2.8903	1.2775	-0.52601	2.58
1862	*MND1*	4.1867	3.7854	-0.222	-1.2342	2.58
1863	*OR10A4*	3.9604	2.9224	0.86725	-2.1745	2.58
1864	*CTBP2*	4.8391	3.1671	-0.25827	-1.1621	2.58
1865	*MTNR1A*	5.6214	2.6353	-0.50978	-1.1605	2.58
1866	*KRTAP10-4*	5.0187	4.4516	-1.7247	-1.8714	2.58
1867	*LIPT1*	4.7148	2.1237	0.90532	0.10522	2.58
1868	*SYT10*	4.4514	2.431	0.85976	-0.77241	2.58
1869	*PGC*	3.9413	1.9778	1.8213	1.7266	2.58
1870	*CA9*	3.712	2.6392	1.3869	-0.95322	2.58
1871	*HS3ST6*	3.6363	3.4971	0.60406	-1.116	2.58
1872	*SIAH3*	4.1282	2.3204	1.2875	0.63161	2.58
1873	*ANKS1B*	2.7074	2.6755	2.3521	-0.60183	2.58
1874	*PARP11*	5.3886	2.0455	0.29711	-0.79711	2.58
1875	*MAP3K14*	3.3517	3.2927	1.0848	-0.22309	2.58
1876	*SMPDL3B*	4.9176	1.755	1.0555	-0.46265	2.58
1877	*ZNF112*	3.4584	3.2978	0.97132	-0.04361	2.58
1878	*TMEM202*	4.8881	3.3658	-0.52731	-1.9673	2.58
1879	*ZBTB49*	3.9341	3.6209	0.17112	-0.1134	2.58

1880	*SYCP1*	3.5241	3.501	0.70084	0.12597	2.58
1881	*TACC2*	3.2226	2.4158	2.0866	-1.7297	2.58
1882	*PDGFRB*	3.3231	3.287	1.1131	-1.7075	2.57
1883	*DENND5A*	5.3165	3.2793	-0.87482	-1.5978	2.57
1884	*SPO11*	4.3337	3.2347	0.15236	-0.65197	2.57
1885	*AGR3*	4.8076	3.7171	-0.80442	-1.2171	2.57
1886	*RALBP1*	3.4226	2.325	1.972	0.041724	2.57
1887	*FAM46D*	3.1701	2.9804	1.5685	-0.42999	2.57
1888	*ZNF530*	5.1767	2.3744	0.16742	-0.13028	2.57
1889	*SPTA1*	3.8708	3.467	0.37957	-0.45048	2.57
1890	*PPM1B*	4.3415	3.3077	0.0669	-0.63112	2.57
1891	*SLC25A34*	3.9763	2.2494	1.4886	-1.9668	2.57
1892	*TMEM233*	4.1825	2.331	1.2007	-0.75388	2.57
1893	*ZNF181*	3.9205	2.4259	1.3666	0.34162	2.57
1894	*TRIQK*	4.7955	2.1612	0.756	-2.6768	2.57
1895	*CD164L2*	2.7893	2.6488	2.2735	-2.0283	2.57
1896	*OSBPL6*	4.3644	2.6868	0.65699	0.030554	2.57
1897	*UNC79*	4.0667	2.2666	1.3744	-0.20942	2.57
1898	*NFATC3*	3.7104	2.5428	1.4544	-2.7379	2.57
1899	*SAMD4A*	3.6359	3.4798	0.59132	-1.917	2.57
1900	*DNASE2*	4.3944	3.3814	-0.07058	-1.399	2.57
1901	*CDH3*	3.9932	2.3748	1.337	-0.26161	2.57
1902	*ZNF233*	4.3186	3.3376	0.0473	-0.02523	2.57
1903	*PLA2G5*	5.2431	1.9463	0.51379	-2.3222	2.57
1904	*FAM47E-STBD1*	3.9185	2.562	1.2222	-0.7623	2.57
1905	*FHIT*	4.8593	1.4679	1.3753	-0.67461	2.57
1906	*ADCY1*	4.3225	3.9428	-0.5628	-1.7364	2.57
1907	*KRTAP5-3*	4.406	3.1577	0.13738	-1.0903	2.57
1908	*NIPAL2*	3.7036	2.3597	1.6376	-0.64458	2.57
1909	*CCDC50*	5.3058	1.5759	0.81481	-0.91061	2.57
1910	*CLDN7*	5.0392	2.3358	0.32102	-0.49941	2.57
1911	*ZNF254*	3.3928	2.3361	1.9651	-0.86691	2.56
1912	*LPAR6*	3.4728	2.5976	1.6233	-1.4151	2.56
1913	*BEX1*	4.3586	2.316	1.018	-1.7644	2.56
1914	*GRP*	4.099	3.1376	0.45591	-1.8038	2.56
1915	*OR2V1*	3.962	3.5718	0.15535	-0.43203	2.56
1916	*CDH15*	3.6423	2.3283	1.7185	0.69415	2.56
1917	*NAPB*	4.2006	3.869	-0.38384	-0.96719	2.56
1918	*CHM*	5.0362	1.8321	0.81592	-1.3296	2.56
1919	*C15orf40*	5.7123	3.2344	-1.2628	-1.7034	2.56
1920	*SLITRK2*	4.4612	2.8415	0.38094	-0.46728	2.56
1921	*ZMYND19*	3.5057	2.3754	1.8008	0.22057	2.56
1922	*DGUOK*	4.8047	1.771	1.1045	-3.2662	2.56
1923	*GPR142*	4.1496	3.7896	-0.26009	-1.1448	2.56
1924	*GCDH*	4.4241	2.2871	0.96721	0.38227	2.56
1925	*ERICH2*	3.516	2.5108	1.6486	-2.2381	2.56

1926	*GPR101*	4.8485	3.446	-0.61967	-1.6943	2.56
1927	*FAM109A*	4.8126	2.9538	-0.09663	-0.75889	2.56
1928	*ARHGAP25*	4.0223	3.6261	0.01846	-0.01399	2.56
1929	*DEFB4A*	3.5517	2.0813	2.0333	-0.44353	2.56
1930	*OR1E2*	3.5297	2.8373	1.2963	-2.7486	2.55
1931	*COQ10B*	4.9825	1.7633	0.91724	-1.5376	2.55
1932	*FAM213B*	4.5052	3.1123	0.04374	-0.60465	2.55
1933	*EPPIN*	3.3438	2.663	1.6518	-2.0622	2.55
1934	*RNASE11*	3.3287	2.7402	1.5893	-0.79108	2.55
1935	*DCUN1D5*	4.154	2.5054	0.99866	-1.7882	2.55
1936	*TMPRSS13*	3.4747	3.2924	0.88915	-0.02187	2.55
1937	*AFP*	3.9351	2.9311	0.78924	0.7006	2.55
1938	*TMEM229B*	4.3745	2.9138	0.36684	-0.94341	2.55
1939	*PDCD10*	3.143	2.8273	1.6839	-0.25971	2.55
1940	*RELB*	4.7333	1.5467	1.3742	-0.38248	2.55
1941	*PFDN4*	4.0319	2.4244	1.1946	0.27203	2.55
1942	*CNR2*	4.2222	3.9111	-0.48652	-2.1648	2.55
1943	*IL4I1*	4.1063	3.9751	-0.43561	-1.0178	2.55
1944	*HCLS1*	3.5644	2.4601	1.6211	0.11496	2.55
1945	*ZNF567*	3.7087	2.2297	1.7066	-1.2858	2.55
1946	*HMGB4*	5.3437	1.3256	0.97516	-3.3077	2.55
1947	*PACSIN2*	4.3814	3.6491	-0.38664	-0.84261	2.55
1948	*SLC10A6*	3.7065	2.9321	1.003	-1.6906	2.55
1949	*MTR*	3.699	2.0729	1.8684	-0.70697	2.55
1950	*SMIM15*	4.5909	1.8816	1.1669	0.51453	2.55
1951	*RNF25*	4.5336	3.138	-0.03297	-0.51785	2.55
1952	*TUBAL3*	6.2265	1.5375	-0.1284	-0.44353	2.55
1953	*AIM2*	4.8059	3.7277	-0.90001	-1.7434	2.54
1954	*HMP19*	3.5492	3.2066	0.87718	-0.86506	2.54
1955	*LCE3C*	4.9742	1.7802	0.87831	-1.1553	2.54
1956	*CCPG1*	4.3058	3.5864	-0.26018	-0.82897	2.54
1957	*PRDX4*	3.3829	2.8949	1.3526	0.75932	2.54
1958	*AKIP1*	4.5863	2.0104	1.0318	-0.52552	2.54
1959	*GNPDA2*	3.8902	3.8467	-0.10861	-0.13837	2.54
1960	*LCN8*	4.311	3.4021	-0.08496	-1.1253	2.54
1961	*OR5M1*	3.8752	3.2843	0.46706	-0.02804	2.54
1962	*PI4K2A*	4.0103	3.7052	-0.08937	-2.1817	2.54
1963	*TMEM130*	4.5762	4.4754	-1.4258	-2.6753	2.54
1964	*C6orf99*	4.7315	1.7859	1.1045	0.47011	2.54
1965	*ZNF83*	4.3959	3.2728	-0.04876	-2.101	2.54
1966	*CDK5RAP2*	4.0214	1.8656	1.7326	-0.43068	2.54
1967	*FBXO31*	5.2473	3.0582	-0.68869	-1.583	2.54
1968	*SIT1*	4.0309	2.7237	0.86091	-0.54972	2.54
1969	*SLC22A2*	4.5833	2.7528	0.27509	-1.4082	2.54
1970	*PACS2*	3.3448	3.3391	0.92699	0.78696	2.54
1971	*ARFGEF3*	3.9858	3.6996	-0.07656	-1.1885	2.54

1972	*MID1IP1*	3.1457	3.1058	1.355	1.1655	2.54
1973	*ABCA2*	4.5452	2.0337	1.0257	-1.6343	2.53
1974	*BSND*	5.2616	1.284	1.0578	-1.7567	2.53
1975	*CD1B*	4.4121	3.3788	-0.18847	-1.0903	2.53
1976	*HMGCLL1*	5.0613	2.9846	-0.44353	-1.8041	2.53
1977	*ZNF146*	4.3349	2.2334	1.032	0.40934	2.53
1978	*CACNA2D1*	4.822	1.5758	1.2023	-0.53086	2.53
1979	*ZNF346*	3.9914	3.4266	0.18078	-1.7646	2.53
1980	*LBX2*	4.8748	2.0536	0.66956	-1.5401	2.53
1981	*RUNX3*	3.3876	3.1409	1.0691	-0.33191	2.53
1982	*OPA3*	3.5237	2.1596	1.9115	-0.26978	2.53
1983	*CACNA1F*	3.618	3.4533	0.52193	-1.0089	2.53
1984	*GLMN*	3.9116	3.5269	0.15419	-1.9817	2.53
1985	*BAIAP2L2*	4.5879	2.6027	0.40178	-0.81714	2.53
1986	*NBPF7*	3.4253	3.2547	0.91197	-1.6447	2.53
1987	*SIGLEC5*	4.329	1.8761	1.3861	0.70578	2.53
1988	*CCDC167*	5.7045	1.7423	0.14422	-0.26093	2.53
1989	*COLGALT2*	2.9181	2.601	2.0719	1.4164	2.53
1990	*OR1S1*	3.0325	2.475	2.0827	0.26002	2.53
1991	*ZSCAN4*	4.4336	3.618	-0.46248	-1.2683	2.53
1992	*NABP2*	5.0652	2.6502	-0.12681	-1.5383	2.53
1993	*TGFA*	3.9289	2.5936	1.0644	0.19946	2.53
1994	*DPT*	5.2745	2.6444	-0.33437	-0.74551	2.53
1995	*SLC45A3*	3.9741	3.0614	0.54726	0.22102	2.53
1996	*GNB2*	3.4648	3.1271	0.98856	0.39882	2.53
1997	*AMN*	4.3049	3.2139	0.05795	-0.49704	2.53
1998	*RCAN1*	4.2242	3.003	0.34895	-0.65889	2.53
1999	*YIPF7*	4.5485	3.3903	-0.36357	-0.68109	2.53
2000	*KANK1*	4.1213	2.7828	0.66961	-2.0542	2.52
2001	*TOX3*	3.9605	3.3287	0.284	-1.6295	2.52
2002	*TTC26*	4.2405	3.3059	0.02667	-2.2403	2.52
2003	*ARSD*	3.7303	3.0297	0.81197	-0.78096	2.52
2004	*CETP*	3.6732	2.5529	1.3447	-1.8929	2.52
2005	*DUSP19*	4.6466	3.3643	-0.44353	-1.0386	2.52
2006	*ADGRB2*	3.9346	3.1613	0.46968	-1.4893	2.52
2007	*OR8B4*	3.4248	3.0891	1.0513	-1.5954	2.52
2008	*RUNDC3B*	3.8622	2.2153	1.4871	0.44356	2.52
2009	*ACOT4*	4.6558	3.5364	-0.6283	-1.7462	2.52
2010	*ZNF331*	3.8481	2.0331	1.6813	1.4686	2.52
2011	*LIX1L*	3.4868	2.972	1.1016	-1.0204	2.52
2012	*KIF9*	3.454	3.1629	0.94319	-0.72936	2.52
2013	*ZNF74*	3.7699	3.5663	0.22383	-0.87719	2.52
2014	*FRMD7*	4.6217	4.5549	-1.6174	-2.212	2.52
2015	*CXCL10*	3.7916	2.9824	0.78193	-0.89957	2.52
2016	*SHISA3*	3.9157	3.6063	0.03295	-1.3465	2.52
2017	*APOBEC3F*	4.7294	3.0481	-0.22301	-1.9987	2.52

2018	*ME2*	4.3065	3.265	-0.01788	-0.22133	2.52
2019	*C17orf70*	3.7346	3.4266	0.39025	-1.8649	2.52
2020	*CPD*	4.4524	2.5416	0.55692	-2.6062	2.52
2021	*AAAS*	3.6811	3.4919	0.3765	-2.6157	2.52
2022	*TRIM62*	4.1469	2.7992	0.60277	-0.66061	2.52
2023	*SNAPC2*	4.5456	3.6286	-0.62548	-1.8016	2.52
2024	*PTPRCAP*	4.8375	2.8685	-0.15733	-1.9622	2.52
2025	*FAM159A*	3.0797	2.7367	1.7312	0.022848	2.52
2026	*OR51D1*	3.22	2.9382	1.3893	-1.8227	2.52
2027	*ZNF790*	3.7348	3.442	0.36967	-1.0711	2.52
2028	*MBTPS1*	3.2315	2.9627	1.3514	0.79448	2.52
2029	*TNFRSF13C*	3.0082	2.4001	2.1371	-1.2252	2.52
2030	*OR2C1*	3.8947	3.1009	0.54961	-1.0726	2.52
2031	*C12orf74*	3.6885	2.121	1.7356	-0.35118	2.52
2032	*FLYWCH2*	4.0854	3.7728	-0.31487	-3.2312	2.51
2033	*S100A11*	2.9661	2.3886	2.1886	2.0281	2.51
2034	*XIAP*	4.296	2.4339	0.81301	-0.28712	2.51
2035	*HOXA1*	5.1256	1.8761	0.54063	-2.2313	2.51
2036	*ADORA3*	4.9348	1.8233	0.78382	-1.2605	2.51
2037	*RPRD1A*	4.3816	3.8522	-0.69287	-1.2936	2.51
2038	*SLTM*	3.9001	3.1657	0.47395	-3.4483	2.51
2039	*C1RL*	4.9343	1.5125	1.0928	0.33766	2.51
2040	*HACD1*	4.625	1.8748	1.0393	-1.3536	2.51
2041	*ASH2L*	3.2244	2.9241	1.3895	-0.49803	2.51
2042	*KRTAP21-2*	4.464	3.7355	-0.66169	-0.8004	2.51
2043	*SFXN5*	5.1542	2.5835	-0.20302	-0.76106	2.51
2044	*HTR1D*	3.9035	3.3521	0.27856	-1.666	2.51
2045	*MKL2*	4.226	2.9889	0.31824	-0.87644	2.51
2046	*BTRC*	3.7138	3.0711	0.74747	-1.2489	2.51
2047	*ADAM21*	3.3236	3.2668	0.94183	0.38075	2.51
2048	*PNPLA5*	4.3488	2.8581	0.32513	-1.7646	2.51
2049	*PDGFRL*	3.9218	3.2317	0.3773	0.37252	2.51
2050	*PIGN*	3.658	3.236	0.63535	-0.42361	2.51
2051	*ATP13A2*	4.4121	2.4875	0.62692	-0.88965	2.51
2052	*C2CD2L*	4.6096	2.9892	-0.07496	-2.0161	2.51
2053	*PPA1*	4.7599	1.961	0.80239	-0.0649	2.51
2054	*NAT14*	3.8472	3.4013	0.27373	-0.2765	2.51
2055	*KRTAP15-1*	3.4295	2.5495	1.5418	-0.14649	2.51
2056	*TMEM204*	4.8618	3.5961	-0.93727	-1.1267	2.51
2057	*TRIM2*	3.2861	2.9094	1.325	0.078477	2.51
2058	*OMG*	3.8689	3.4671	0.18355	-1.7043	2.51
2059	*FAM127B*	4.3409	2.055	1.1232	-0.65184	2.51
2060	*ZNF814*	3.2439	2.6042	1.6706	0.28213	2.51
2061	*ERC1*	3.343	2.7477	1.4276	-0.93489	2.51
2062	*BCL11A*	4.5446	2.7323	0.24096	-1.6891	2.51
2063	*XYLB*	3.6497	3.2498	0.61636	-0.44711	2.51

2064	*ACSM4*	3.7287	2.3249	1.4608	-1.5393	2.50
2065	*BASP1*	5.4449	1.056	1.0131	-0.22538	2.50
2066	*MRGPRX3*	5.5247	2.7032	-0.71443	-0.76015	2.50
2067	*ZNF761*	3.6883	3.3099	0.51517	-1.1209	2.50
2068	*TMEM14C*	4.3927	2.9673	0.15229	-1.1739	2.50
2069	*ZXDB*	3.1398	2.5388	1.8324	-0.09072	2.50
2070	*DACH2*	4.106	2.8493	0.55323	-0.30711	2.50
2071	*ZNF134*	4.2863	3.5323	-0.3105	-0.3922	2.50
2072	*RBAK-RBAKDN*	3.5406	2.0786	1.8889	-0.33131	2.50
2073	*NEK11*	4.0021	2.0482	1.4543	-0.24973	2.50
2074	*VSIG8*	4.5204	4.4704	-1.4864	-2.5226	2.50
2075	*C6orf62*	4.3832	4.2367	-1.1159	-1.4289	2.50
2076	*UQCRC1*	3.3126	2.3686	1.8187	-0.81178	2.50
2077	*KCNK3*	4.8459	1.9462	0.70671	-1.5684	2.50
2078	*KNG1*	4.4318	1.8527	1.2128	-2.8823	2.50
2079	*MDFIC*	3.7695	2.1504	1.573	1.3057	2.50
2080	*KRTDAP*	3.1595	2.6127	1.7199	-2.2381	2.50
2081	*MAD1L1*	4.9466	1.8257	0.71818	-2.075	2.50
2082	*C4orf17*	4.4278	2.491	0.57061	-1.5039	2.50
2083	*SPRYD4*	4.3221	3.0812	0.08403	-1.3675	2.50
2084	*RRP8*	4.707	2.372	0.4082	-0.98138	2.50
2085	*MRAP2*	4.8127	2.3941	0.27849	-0.81031	2.50
2086	*COA1*	3.6965	2.3102	1.4781	0.93632	2.49
2087	*SIVA1*	3.7084	2.2442	1.5321	-0.37692	2.49
2088	*SH3KBP1*	4.1292	3.0958	0.25951	-0.49028	2.49
2089	*ERGIC3*	3.9906	3.282	0.21175	-1.0036	2.49
2090	*ZNF683*	4.7699	3.105	-0.39079	-1.1218	2.49
2091	*SLC39A2*	3.764	3.5485	0.17128	-3.0713	2.49
2092	*PPIL1*	3.9064	3.7668	-0.18991	-1.2771	2.49
2093	*STYX*	5.1964	1.7881	0.49798	-1.0898	2.49
2094	*WDR91*	5.012	3.199	-0.72882	-1.1325	2.49
2095	*KRTAP13-2*	3.5336	2.0646	1.8833	0.70262	2.49
2096	*YTHDF3*	4.5933	3.1694	-0.28176	-1.0312	2.49
2097	*TREML4*	4.4503	3.9938	-0.96349	-1.0773	2.49
2098	*TLDC1*	4.0223	2.9014	0.55629	0.11567	2.49
2099	*BHLHE41*	5.3756	4.08	-1.9758	-2.3681	2.49
2100	*DCAF12L1*	3.6532	2.0215	1.8033	0.95048	2.49
2101	*RAD9B*	4.2688	2.9472	0.26036	-2.0838	2.49
2102	*NPPA*	3.3931	2.8201	1.2617	-0.27356	2.49
2103	*SPANXA1*	3.0054	2.8011	1.6674	0.098389	2.49
2104	*ATP6V1H*	4.2455	4.0189	-0.79083	-1.3988	2.49
2105	*TCEAL4*	3.8653	2.4295	1.1756	-1.2779	2.49
2106	*C9orf163*	4.1099	3.1205	0.23877	-1.2866	2.49
2107	*CAGE1*	3.2936	2.9868	1.1885	0.5805	2.49
2108	*FUT6*	4.4157	3.5526	-0.49953	-1.2619	2.49
2109	*IKZF5*	3.7598	3.7159	-0.00828	-0.91696	2.49

2110	*LMF2*	3.9639	2.4916	1.0119	0.061768	2.49
2111	*PTBP3*	3.5227	3.2955	0.64843	-1.3907	2.49
2112	*RND1*	5.2432	2.1134	0.10729	-1.4102	2.49
2113	*SERPINA3*	4.4862	1.6936	1.284	-0.54142	2.49
2114	*LCP2*	2.9477	2.2672	2.2479	0.1832	2.49
2115	*RETN*	3.7789	2.4454	1.2383	0.29117	2.49
2116	*MARVELD1*	3.6094	3.5618	0.29118	-1.9417	2.49
2117	*CFP*	5.5543	1.4941	0.41381	0.12053	2.49
2118	*ZNF285*	3.3699	3.162	0.93021	-0.19984	2.49
2119	*IL20*	3.1516	2.5428	1.7677	-0.44986	2.49
2120	*ADGRE3*	3.1226	2.6318	1.7065	0.46797	2.49
2121	*IGF1*	4.2768	4.262	-1.0793	-1.8695	2.49
2122	*KCNMA1*	3.6519	2.4467	1.3606	-1.6597	2.49
2123	*VN1R4*	4.0861	3.0451	0.32727	0.31672	2.49
2124	*R3HCC1L*	3.969	2.3695	1.1198	-1.5313	2.49
2125	*ASF1B*	5.0745	1.8477	0.53309	0.49883	2.49
2126	*PHF6*	3.552	2.5411	1.3621	0.40997	2.49
2127	*ITGA9*	3.9025	2.5166	1.0349	-1.1706	2.48
2128	*MPHOSPH9*	6.0937	2.2001	-0.84001	-0.95017	2.48
2129	*TEKT1*	3.9986	3.0748	0.37516	-1.7512	2.48
2130	*CST9*	4.0683	1.9193	1.4603	0.63445	2.48
2131	*LCMT2*	4.498	2.4578	0.49083	-0.08206	2.48
2132	*PLA2G12A*	3.9759	3.4019	0.06868	-1.3951	2.48
2133	*CCDC158*	3.1539	2.6197	1.6726	0.47319	2.48
2134	*TMEM184C*	4.2788	3.9779	-0.8118	-1.195	2.48
2135	*CCDC65*	3.5103	2.5739	1.3583	-1.9172	2.48
2136	*R3HDM1*	3.9105	2.8505	0.68026	0.35076	2.48
2137	*OR2T12*	3.5933	3.4851	0.36265	-1.9563	2.48
2138	*KLHL34*	4.4539	3.6755	-0.6886	-1.7285	2.48
2139	*GANAB*	4.4507	3.0951	-0.10525	-0.25741	2.48
2140	*B9D1*	3.3233	2.3259	1.7913	-1.1588	2.48
2141	*UEVLD*	3.3345	2.9816	1.1228	-1.1908	2.48
2142	*PKD1*	4.476	3.8731	-0.91192	-1.3542	2.48
2143	*GIMAP7*	3.9238	2.5308	0.98231	-1.9351	2.48
2144	*MID2*	3.1329	2.7704	1.5331	1.1841	2.48
2145	*CRBN*	3.3156	2.2911	1.8249	0.11201	2.48
2146	*PTGFRN*	4.5124	2.2699	0.64765	-0.01323	2.48
2147	*OR5L1*	3.673	3.6077	0.1486	-1.3769	2.48
2148	*CFAP45*	5.0472	1.816	0.56476	-0.06364	2.48
2149	*SVOPL*	4.476	2.1857	0.76425	0.00025	2.48
2150	*CARD18*	4.2806	2.135	1.009	-0.88804	2.47
2151	*NUDT5*	3.8717	2.0891	1.4638	1.3561	2.47
2152	*CAPN9*	4.7003	1.7111	1.0124	-2.0838	2.47
2153	*PPFIA4*	3.8964	3.3453	0.18039	-1.4611	2.47
2154	*KLF6*	5.0664	3.8048	-1.4493	-1.6975	2.47
2155	*IFI27*	4.7258	1.3888	1.3071	-0.29986	2.47

2156	*TTLL9*	4.16	2.0383	1.2228	-1.3787	2.47
2157	*GLO1*	3.8463	3.356	0.21634	-1.7507	2.47
2158	*SERPINF1*	3.6692	2.2444	1.5037	-0.2475	2.47
2159	*MIF*	5.1756	1.903	0.33664	-0.67981	2.47
2160	*MYC*	4.1961	1.9024	1.3144	-1.7107	2.47
2161	*ANKUB1*	4.1231	1.6789	1.6105	-1.7602	2.47
2162	*KLHL6*	4.3161	3.2383	-0.14198	-0.26653	2.47
2163	*SBK2*	3.9289	2.1882	1.2943	-1.0743	2.47
2164	*ING4*	4.6709	2.5905	0.14964	-1.7935	2.47
2165	*NLRP7*	4.0997	3.3167	-0.00555	-0.17446	2.47
2166	*ANKRD34A*	5.367	1.1966	0.84714	-1.3443	2.47
2167	*MICAL1*	4.7451	2.5876	0.07718	-1.3111	2.47
2168	*FLJ45513*	3.3781	3.0505	0.98008	-1.7079	2.47
2169	*STRIP1*	3.5578	2.4328	1.4177	-1.8598	2.47
2170	*SLC9C2*	3.8091	3.3477	0.24896	0.12995	2.47
2171	*OR6C4*	3.8833	1.8199	1.7008	-3.0028	2.47
2172	*OR5J2*	3.7069	2.0716	1.6252	-1.2357	2.47
2173	*CCKAR*	3.8384	1.9872	1.5781	-2.2078	2.47
2174	*CATSPER2*	3.9521	2.4413	1.0095	-1.1426	2.47
2175	*MAZ*	3.5707	2.7344	1.0961	-0.61655	2.47
2176	*PATE2*	3.8246	3.438	0.13738	0.054968	2.47
2177	*TIFAB*	3.3496	2.5804	1.4688	-0.7372	2.47
2178	*KPNA7*	4.4552	2.2075	0.73441	-2.3095	2.47
2179	*PORCN*	2.6392	2.4956	2.2621	-1.068	2.47
2180	*DPRX*	3.9899	3.9694	-0.56272	-1.149	2.47
2181	*ALDH1B1*	3.6371	2.7858	0.97266	-0.54028	2.47
2182	*DPY19L4*	4.3121	3.3511	-0.26832	-1.3128	2.46
2183	*GABPB2*	3.7603	2.1181	1.5151	1.2415	2.46
2184	*APPL1*	3.5823	3.1805	0.62804	0.068285	2.46
2185	*SAT1*	4.3217	1.9547	1.1138	-1.505	2.46
2186	*C17orf107*	4.0444	3.3643	-0.01938	-0.36571	2.46
2187	*RNF223*	3.6699	2.446	1.2726	0.53163	2.46
2188	*UGT1A3*	4.7065	3.5871	-0.90663	-1.4997	2.46
2189	*CLDN4*	4.1405	2.5761	0.66966	-0.4264	2.46
2190	*GSTA4*	3.5406	2.0346	1.8099	1.2057	2.46
2191	*NFATC4*	4.9242	3.0366	-0.57713	-0.84872	2.46
2192	*ZC3HC1*	3.9567	2.0256	1.3999	-0.24239	2.46
2193	*ORAI3*	4.5706	1.7638	1.045	-0.90149	2.46
2194	*RDX*	3.4172	2.392	1.5676	-1.6502	2.46
2195	*KLHL35*	3.6592	2.205	1.5123	-1.0852	2.46
2196	*AKAP10*	3.8365	1.7906	1.7493	-1.9915	2.46
2197	*EQTN*	3.6924	2.0843	1.5987	-3.3806	2.46
2198	*RTN2*	3.8372	3.618	-0.08064	-0.49286	2.46
2199	*ZNF518B*	3.769	3.2749	0.32816	-0.95224	2.46
2200	*GDNF*	2.7524	2.7467	1.8722	-1.4125	2.46
2201	*ARRDC1*	4.9551	4.2108	-1.7963	-1.9713	2.46

2202	*ENPP3*	3.8436	3.4133	0.11185	-0.58289	2.46
2203	*LAMA2*	4.8872	1.9622	0.51893	-0.44353	2.46
2204	*FOLR1*	4.7769	3.7423	-1.151	-2.1921	2.46
2205	*CAPN12*	4.5365	2.368	0.46328	-0.37417	2.46
2206	*DPP7*	5.5093	1.9087	-0.05066	-1.5848	2.46
2207	*PSD3*	4.0399	3.554	-0.22757	-0.39872	2.46
2208	*ENDOD1*	4.3932	2.4544	0.51575	-1.049	2.45
2209	*FAM102A*	3.5059	2.4666	1.3897	0.53106	2.45
2210	*ABRA*	3.3031	2.6931	1.3648	-0.04135	2.45
2211	*CCL11*	4.085	3.7881	-0.51221	-1.3835	2.45
2212	*C11orf96*	4.237	3.7445	-0.62065	-1.0861	2.45
2213	*B4GALT3*	5.0188	2.5131	-0.17629	-1.4325	2.45
2214	*NOS1AP*	3.5837	1.8925	1.8779	-1.4811	2.45
2215	*CYP3A43*	3.7314	3.5556	0.06617	-0.79825	2.45
2216	*C1orf21*	5.4341	2.4771	-0.55848	-0.79663	2.45
2217	*PNMA1*	3.0089	2.6949	1.6467	-1.5923	2.45
2218	*RS1*	4.2858	3.0416	0.02027	-0.33304	2.45
2219	*FCRLA*	4.8318	3.3494	-0.83459	-1.3033	2.45
2220	*OR6K6*	4.0505	3.2366	0.05775	-1.206	2.45
2221	*NFIX*	4.065	2.3578	0.9183	0.71966	2.45
2222	*SSX1*	4.0871	2.5707	0.68287	-0.58894	2.45
2223	*SLC25A52*	4.5701	1.7165	1.0538	-0.94472	2.45
2224	*ZNF253*	5.5399	1.5648	0.23528	-1.6315	2.45
2225	*CABYR*	4.2642	2.027	1.0468	-0.57078	2.45
2226	*P4HA2*	4.0313	3.8585	-0.55181	-0.9414	2.45
2227	*ATXN3*	4.4581	2.8375	0.04176	-0.03588	2.45
2228	*PHF11*	5.3657	1.5133	0.45807	-1.2679	2.45
2229	*OR1S2*	4.1374	2.8781	0.32041	-0.29414	2.45
2230	*TADA2B*	3.9694	3.0456	0.32043	-1.1925	2.45
2231	*RAB13*	4.9705	3.1372	-0.77284	-2.0113	2.44
2232	*TMEM183A*	3.5512	1.9848	1.7981	-0.1938	2.44
2233	*XG*	3.9407	3.4837	-0.09032	-1.2985	2.44
2234	*SPTY2D1*	3.8845	1.7403	1.7088	-0.54329	2.44
2235	*EGFL6*	4.568	2.56	0.20391	-1.8724	2.44
2236	*ATP1A4*	3.2455	2.5587	1.5276	1.4833	2.44
2237	*ASTE1*	5.3443	1.6071	0.38031	-1.479	2.44
2238	*PCDHA1*	4.1259	3.1529	0.05181	-2.6438	2.44
2239	*LRP12*	3.8324	2.8725	0.62444	0.12879	2.44
2240	*ZNF850*	3.9311	2.4438	0.95268	-1.5105	2.44
2241	*VAMP4*	5.9385	0.7189	0.66945	-1.3544	2.44
2242	*MARCH6*	5.1982	1.2352	0.89321	-1.8999	2.44
2243	*SLC2A5*	3.5961	2.0434	1.6854	1.4669	2.44
2244	*SMTNL2*	4.7915	1.6002	0.93207	-1.4526	2.44
2245	*MOXD1*	3.5402	3.3616	0.42022	-1.1287	2.44
2246	*NUAK2*	4.3428	3.4898	-0.51117	-1.3469	2.44
2247	*TRIM40*	3.5346	3.2522	0.53086	-0.41548	2.44

2248	*FAM184A*	3.7774	3.5464	-0.00662	-0.81546	2.44
2249	*COQ3*	5.1231	2.4966	-0.30528	-1.5304	2.44
2250	*EVC*	3.6502	2.9036	0.76022	-0.03924	2.44
2251	*SUN1*	4.0341	2.3937	0.88332	-0.59581	2.44
2252	*SPAG11B*	3.7756	2.362	1.1729	-1.2342	2.44
2253	*LOC729159*	3.106	2.9046	1.2987	-0.63502	2.44
2254	*TCP11L1*	5.8601	2.1928	-0.7443	-1.7235	2.44
2255	*ZNF610*	2.7122	2.6289	1.9665	-0.24142	2.44
2256	*ABHD14A*	3.5004	2.204	1.6021	-1.4545	2.44
2257	*DCAF5*	4.6611	4.4312	-1.7865	-2.4286	2.44
2258	*TIMM44*	5.5623	2.1867	-0.44353	-0.83277	2.44
2259	*ZNF41*	5.035	1.9912	0.27224	-1.5996	2.43
2260	*LRP2BP*	5.0261	4.2751	-2.0042	-2.6973	2.43
2261	*RNF26*	3.1246	2.1373	2.0336	-0.33234	2.43
2262	*WBSCR17*	3.8644	2.8694	0.56046	-0.37619	2.43
2263	*SMIM6*	5.186	1.505	0.60293	-1.1021	2.43
2264	*VAMP5*	4.1414	2.4143	0.73823	-1.8979	2.43
2265	*WWP2*	3.3133	3.1362	0.84357	0.013351	2.43
2266	*OR10K2*	4.3162	1.6199	1.356	0.096843	2.43
2267	*USP17L15*	4.0256	3.5495	-0.28317	-2.775	2.43
2268	*COMMD4*	4.0351	3.1295	0.12279	0.065382	2.43
2269	*MGAT4B*	5.7154	3.3185	-1.7466	-1.99	2.43
2270	*TMEM262*	4.7345	1.542	1.0068	-1.031	2.43
2271	*GRHL1*	4.064	2.0604	1.158	-0.24704	2.43
2272	*ST3GAL6*	3.8613	3.8573	-0.43634	-1.6891	2.43
2273	*L3MBTL1*	4.8117	2.1438	0.32624	-0.48085	2.43
2274	*OR9G1*	4.5208	3.3398	-0.57896	-0.81293	2.43
2275	*TSPAN19*	4.5113	3.1224	-0.35458	-0.40772	2.43
2276	*LUZP6*	3.2922	2.2986	1.6883	0.34091	2.43
2277	*NUMA1*	5.2108	1.7513	0.31611	-2.0208	2.43
2278	*EPHB1*	3.4122	2.3188	1.5471	0.15092	2.43
2279	*COMMD6*	3.7529	3.47	0.05454	-1.257	2.43
2280	*PTPN4*	4.0276	2.2169	1.0321	-0.55774	2.43
2281	*SREK1*	4.5143	2.0024	0.75943	-0.41687	2.43
2282	*PDIA4*	3.635	2.6316	1.0084	-1.5894	2.43
2283	*PJA1*	4.4755	3.0199	-0.22048	-1.0946	2.42
2284	*PHF21B*	4.3836	2.1633	0.72734	-1.7403	2.42
2285	*KLHL23*	4.4772	2.4814	0.31541	-1.4536	2.42
2286	*PRPSAP2*	4.1516	1.9556	1.1667	-1.0868	2.42
2287	*BPIFB1*	4.1327	3.5014	-0.36047	-0.54019	2.42
2288	*WDYHV1*	4.3079	3.8695	-0.90386	-1.1066	2.42
2289	*CCL15*	4.6857	2.2487	0.33799	-0.2372	2.42
2290	*TNFSF9*	5.8157	0.93409	0.52108	-1.0041	2.42
2291	*KIAA1524*	4.4076	1.9578	0.90406	-0.5018	2.42
2292	*FAM192A*	4.7555	2.862	-0.34906	-1.5722	2.42
2293	*KIAA0100*	3.8878	2.1167	1.2634	-2.0382	2.42

2294	*GCC2*	3.2061	3.2009	0.86053	0.61385	2.42
2295	*USH2A*	4.9153	2.1798	0.17147	-1.2867	2.42
2296	*LAYN*	2.7249	2.6258	1.9153	0.51421	2.42
2297	*ACAP3*	3.8079	3.1143	0.34328	-1.2519	2.42
2298	*CECR2*	2.8456	2.3273	2.0924	1.895	2.42
2299	*UBE3B*	4.6601	3.1042	-0.49941	-2.0116	2.42
2300	*KIF7*	4.3635	2.3144	0.58585	-0.34376	2.42
2301	*TMEM194A*	3.7524	3.5451	-0.03456	-2.1354	2.42
2302	*MAP3K2*	4.7726	3.0782	-0.58851	-0.63939	2.42
2303	*JPH2*	3.5846	2.9975	0.68011	-1.0186	2.42
2304	*THADA*	3.8694	3.7295	-0.33844	-0.41308	2.42
2305	*GLB1L3*	3.3861	2.7788	1.0947	-1.0978	2.42
2306	*STARD3*	4.7791	2.5002	-0.01973	-0.0777	2.42
2307	*FMO3*	3.8496	1.8337	1.5761	1.4245	2.42
2308	*CNBD2*	4.8488	4.025	-1.6146	-2.3005	2.42
2309	*HOXC6*	3.8265	2.8613	0.57124	-0.77997	2.42
2310	*PDP2*	3.9352	2.1141	1.2091	-1.25	2.42
2311	*SLPI*	4.0335	3.8494	-0.62476	-1.9292	2.42
2312	*EED*	4.9755	1.499	0.78274	-0.62747	2.42
2313	*PICALM*	4.8185	1.8296	0.6088	-1.1105	2.42
2314	*SCARB1*	4.6665	2.6874	-0.09709	-0.67141	2.42
2315	*THSD7A*	4.2029	3.5343	-0.48156	-1.631	2.42
2316	*FAM127A*	4.0272	2.6613	0.5659	-1.6597	2.42
2317	*IGDCC3*	3.2584	2.2096	1.7861	-0.76074	2.42
2318	*ZNF728*	4.3697	2.2131	0.66944	-0.44353	2.42
2319	*ABCA13*	4.9036	1.5257	0.821	0.081017	2.42
2320	*FAHD1*	5.0354	1.5927	0.62183	-1.4909	2.42
2321	*PPP2R3B*	4.212	3.1828	-0.1449	-1.0323	2.42
2322	*LPCAT4*	4.0472	2.6712	0.53149	0.38934	2.42
2323	*TRMT10B*	3.4219	2.9191	0.90845	-2.1523	2.42
2324	*ZNF461*	2.8142	2.6667	1.7679	-1.0384	2.42
2325	*FCRL5*	3.5876	1.9156	1.7441	-0.15226	2.42
2326	*TAS1R1*	3.9385	2.6545	0.65303	-2.2254	2.42
2327	*PROS1*	4.1069	2.4027	0.73603	-0.88278	2.42
2328	*NPY5R*	4.1541	2.3865	0.70487	0.17026	2.42
2329	*C2orf83*	3.4569	2.3422	1.4438	-1.8049	2.41
2330	*CTNNAL1*	5.196	1.3665	0.67885	-0.97982	2.41
2331	*KIAA1683*	4.9243	1.2823	1.0337	-0.13183	2.41
2332	*SLC44A5*	3.7258	3.0288	0.4856	-2.058	2.41
2333	*KIF4B*	3.8764	2.3018	1.0613	-0.36087	2.41
2334	*CXCL16*	4.081	2.8902	0.26709	-1.3419	2.41
2335	*CNNM2*	4.9237	3.2034	-0.88927	-1.0386	2.41
2336	*DSG1*	3.4442	2.9901	0.80248	-1.1958	2.41
2337	*LOC643355*	3.9664	3.1353	0.13453	-0.44744	2.41
2338	*TWF2*	3.3152	2.1061	1.8107	-1.0106	2.41
2339	*RCSD1*	3.564	3.5177	0.14885	-0.58806	2.41

2340	*SPINK6*	5.5043	1.7862	-0.06072	-0.08199	2.41
2341	*TUSC5*	4.1319	2.511	0.58678	-1.4474	2.41
2342	*NR2C1*	3.474	3.3001	0.45421	-0.9612	2.41
2343	*SPATA31C1*	3.9071	3.7646	-0.44353	-0.60727	2.41
2344	*GAREM*	3.0953	2.1471	1.9837	-0.24578	2.41
2345	*ARF5*	4.1032	2.848	0.27433	-2.5298	2.41
2346	*UPK3A*	5.2193	1.6429	0.36147	0.14277	2.41
2347	*NAF1*	4.138	3.8555	-0.77162	-1.6262	2.41
2348	*LDLRAP1*	3.9251	1.771	1.5243	-0.87195	2.41
2349	*VMO1*	3.719	2.9864	0.5094	-0.66215	2.40
2350	*MORN2*	4.3735	2.7615	0.07822	-0.64793	2.40
2351	*PABPC4L*	2.6353	2.4771	2.1004	-0.95682	2.40
2352	*FAM205A*	5.4646	1.9174	-0.17026	-2.0383	2.40
2353	*FABP2*	4.1513	2.6853	0.37511	-2.1984	2.40
2354	*G6PC3*	3.6297	2.7397	0.84205	-1.0172	2.40
2355	*POM121L12*	4.1298	2.7727	0.30799	-1.0696	2.40
2356	*HDAC5*	2.9761	2.46	1.7741	-1.2649	2.40
2357	*OR4C46*	4.6442	3.5625	-0.99699	-1.5619	2.40
2358	*PRB2*	5.0689	2.134	0.00631	-0.13293	2.40
2359	*HRH2*	2.9188	2.2369	2.0515	0.93074	2.40
2360	*PMAIP1*	5.2834	2.3233	-0.39971	-2.4168	2.40
2361	*RRBP1*	3.8786	2.4372	0.89116	-0.7361	2.40
2362	*NOTCH3*	4.1543	3.3924	-0.34014	-0.74141	2.40
2363	*MAGEB2*	4.0602	3.4347	-0.28926	-2.3287	2.40
2364	*ZCCHC6*	4.4831	2.5589	0.16218	-1.3055	2.40
2365	*RGPD3*	3.4644	2.0367	1.7027	0.77812	2.40
2366	*WNT9A*	4.4956	2.0146	0.69034	-0.7189	2.40
2367	*FAM19A3*	3.1477	2.8323	1.2204	0.66504	2.40
2368	*EVI2B*	4.8068	1.8858	0.50464	-0.47857	2.40
2369	*GCNT1*	2.8813	2.7532	1.5627	-0.02187	2.40
2370	*GPR157*	4.2789	2.7587	0.15915	-1.0284	2.40
2371	*FNBP4*	3.4305	2.0565	1.7084	-1.4624	2.40
2372	*PDE4B*	3.9444	1.9557	1.2941	1.1886	2.40
2373	*HRNR*	4.6734	1.8709	0.64725	0.30293	2.40
2374	*AAMDC*	3.3454	2.5911	1.2548	-1.9019	2.40
2375	*LOC79999*	4.1602	3.6998	-0.66992	-2.8628	2.40
2376	*KBTBD7*	3.7344	3.2441	0.21027	-1.8016	2.40
2377	*SPATA31A5*	4.2255	4.1528	-1.1898	-2.5392	2.40
2378	*CT47A5*	4.1543	3.5871	-0.55379	-1.2055	2.40
2379	*C8orf58*	4.5145	2.9762	-0.30336	-1.0291	2.40
2380	*TPM3*	3.1766	3.0978	0.91013	0.65059	2.39
2381	*CRMP1*	5.4676	1.1327	0.58355	0.53406	2.39
2382	*ATP6V0A4*	4.2991	3.9203	-1.0359	-1.3507	2.39
2383	*PHYKPL*	3.5745	2.8546	0.75321	-2.9054	2.39
2384	*PCDHA2*	4.4251	1.3843	1.3707	1.2214	2.39
2385	*SUSD6*	3.7516	3.0325	0.39467	-1.584	2.39

2386	*SLC10A7*	4.9665	1.4863	0.72543	0.70596	2.39
2387	*ANAPC4*	4.0014	3.9142	-0.7385	-1.6324	2.39
2388	*PTPN7*	4.4319	2.6657	0.07904	-1.1189	2.39
2389	*MAPK3*	4.3102	1.6857	1.1772	0.49084	2.39
2390	*MAN1C1*	3.6868	2.2646	1.2206	0.51273	2.39
2391	*PRB1*	3.3483	3.1092	0.71407	-0.78229	2.39
2392	*MRS2*	5.2572	2.5481	-0.63575	-1.3407	2.39
2393	*POU4F1*	4.4852	3.1147	-0.43101	-1.248	2.39
2394	*DGAT2*	3.3059	2.9942	0.86844	-1.2603	2.39
2395	*RNF44*	3.581	3.5283	0.05848	-1.1684	2.39
2396	*CUTC*	3.0461	2.4174	1.703	-0.98486	2.39
2397	*GPR171*	3.7267	3.1825	0.2573	-0.7064	2.39
2398	*SYNGAP1*	4.02	1.6936	1.4527	-1.2288	2.39
2399	*ITGAX*	4.6197	1.416	1.1302	-1.5441	2.39
2400	*SLC22A5*	3.8796	2.692	0.5941	0.072125	2.39
2401	*LOC286238*	3.7662	3.1652	0.2336	-2.2413	2.39
2402	*ZNF678*	2.6086	2.5459	2.009	1.1296	2.39
2403	*AVPR2*	3.5734	2.3727	1.2173	-1.5965	2.39
2404	*TRIM45*	3.9008	2.6249	0.6351	-0.0442	2.39
2405	*TMEM219*	3.2542	2.0408	1.8657	-0.03506	2.39
2406	*FAM104B*	3.5841	3.3359	0.23956	-0.98459	2.39
2407	*TMPRSS11A*	3.7898	3.3082	0.06002	-0.27016	2.39
2408	*CEP170B*	4.3117	2.9856	-0.13943	-0.90954	2.39
2409	*DGKD*	4.4362	4.3008	-1.5795	-2.08	2.39
2410	*OR2T10*	4.6791	2.7809	-0.30268	-1.4576	2.39
2411	*RGS7BP*	3.1028	2.5903	1.4634	-0.04135	2.39
2412	*MAST1*	4.2119	3.2776	-0.33312	-2.1848	2.39
2413	*TSPAN1*	3.7886	1.9179	1.4496	-0.42817	2.39
2414	*CCDC121*	4.2306	1.8335	1.0914	0.80953	2.39
2415	*RAB5C*	5.2372	2.8317	-0.91363	-2.0613	2.39
2416	*CPM*	3.7861	2.9089	0.45892	0.40342	2.38
2417	*GAS2L2*	3.7182	2.9411	0.49347	-1.6763	2.38
2418	*ABLIM3*	3.802	2.8191	0.53135	0.054336	2.38
2419	*CFL1*	3.0905	2.3667	1.6951	-1.5402	2.38
2420	*ABRACL*	3.6776	3.266	0.20851	-1.7577	2.38
2421	*NUDT11*	4.2517	3.4094	-0.50957	-2.6188	2.38
2422	*HIST1H2BO*	2.8152	2.4766	1.8596	0.069589	2.38
2423	*MED14OS*	4.5238	3.1701	-0.54604	-1.5125	2.38
2424	*TTC30B*	4.4257	1.5158	1.2063	0.47245	2.38
2425	*ARSE*	3.5217	2.4719	1.1535	-2.5795	2.38
2426	*PER2*	3.8627	3.3749	-0.09062	-0.17241	2.38
2427	*OR2T11*	2.736	2.4907	1.9197	1.1803	2.38
2428	*CPNE1*	4.7161	1.3823	1.0471	-1.1644	2.38
2429	*JUP*	4.8035	2.5085	-0.16814	-0.75548	2.38
2430	*NT5M*	3.2946	3.2043	0.64459	-0.5667	2.38
2431	*NME4*	3.6196	2.465	1.0584	0.89315	2.38

2432	*ARHGEF11*	4.5535	2.0046	0.58403	-0.57732	2.38
2433	*ANKRD61*	4.091	3.2228	-0.17243	-0.84001	2.38
2434	*GRIN2D*	6.092	0.73457	0.31401	-2.3062	2.38
2435	*PRRG4*	3.2047	2.6624	1.2728	0.58455	2.38
2436	*MKRN2*	2.8291	2.2561	2.0544	-2.7223	2.38
2437	*KRTAP12-1*	3.6159	2.0185	1.5047	0.9039	2.38
2438	*BRS3*	3.9977	2.6906	0.45067	-0.45119	2.38
2439	*KIF13B*	3.351	2.3404	1.4475	0.88805	2.38
2440	*C19orf38*	3.4705	1.9995	1.6675	0.003685	2.38
2441	*CBR1*	5.19	2.5052	-0.56036	-2.8398	2.38
2442	*MLLT10*	5.1279	2.8216	-0.81491	-0.81863	2.38
2443	*CPZ*	3.5524	1.8998	1.6814	-1.6586	2.38
2444	*ARL5A*	3.4892	2.6889	0.95539	0.284	2.38
2445	*THEM5*	3.5152	3.0195	0.59683	-0.03924	2.38
2446	*TBL1XR1*	4.0969	2.8034	0.23006	-2.2713	2.38
2447	*APBA1*	4.2718	3.1925	-0.33601	-0.79558	2.38
2448	*MYF6*	4.1776	3.0281	-0.07743	-1.2535	2.38
2449	*PTRHD1*	4.2573	2.762	0.10857	-0.19889	2.38
2450	*NASP*	4.0717	3.2598	-0.20419	-1.1891	2.38
2451	*ARL6IP4*	2.816	2.8015	1.5097	0.35851	2.38
2452	*HLA-A*	4.9368	2.2164	-0.02694	-0.60113	2.38
2453	*FSD2*	3.5636	1.8157	1.7468	-1.4764	2.38
2454	*TOMM40L*	4.7453	2.7184	-0.33821	-0.8462	2.38
2455	*AEBP2*	3.7859	2.8489	0.49055	-1.8253	2.38
2456	*ANKRD30B*	3.1278	2.9886	1.0082	-0.48991	2.37
2457	*CRNN*	4.1409	2.6846	0.2982	0.033383	2.37
2458	*LY75*	4.4254	2.2312	0.46631	0.27373	2.37
2459	*OR10A2*	3.7973	2.2245	1.0989	0.26509	2.37
2460	*C19orf47*	4.6626	1.8673	0.58868	-0.86923	2.37
2461	*CEACAM18*	3.8812	1.6955	1.5418	-1.0451	2.37
2462	*TMEM127*	3.442	3.0325	0.64351	-0.55516	2.37
2463	*AQP3*	3.5338	3.0565	0.52748	0.19028	2.37
2464	*PIK3CG*	4.6634	3.3893	-0.93555	-1.2935	2.37
2465	*MEX3B*	3.642	3.5473	-0.07219	-0.29292	2.37
2466	*AIM1*	4.03	2.3024	0.78393	-1.7649	2.37
2467	*EHD4*	5.4343	1.2398	0.44192	-1.0412	2.37
2468	*ADCY10*	4.2036	1.5076	1.4047	-1.6235	2.37
2469	*ASB1*	3.495	2.3629	1.2578	-1.7079	2.37
2470	*C4orf19*	4.6058	2.0539	0.45432	0.33187	2.37
2471	*FYCO1*	4.2232	1.5136	1.3763	0.24233	2.37
2472	*AIDA*	4.4736	1.3624	1.2727	-1.4174	2.37
2473	*SLFN14*	2.7691	2.2171	2.1223	1.1669	2.37
2474	*SREBF2*	5.5392	0.95143	0.61711	0.22088	2.37
2475	*PDE6A*	3.6954	1.7136	1.6986	0.22031	2.37
2476	*PTAFR*	3.5447	3.2884	0.27343	-1.8181	2.37
2477	*TUBB1*	4.2227	3.1238	-0.24037	-0.43313	2.37

2478	*SPSB3*	3.6195	2.386	1.1004	-0.05744	2.37
2479	*PLCB3*	3.4761	1.8466	1.7819	-2.0664	2.37
2480	*DUSP11*	5.1483	1.3405	0.61328	-5.7233	2.37
2481	*CFAP221*	4.4544	2.6522	-0.00551	-2.0586	2.37
2482	*MXRA8*	3.7377	2.4426	0.92019	0.54218	2.37
2483	*SERP2*	4.1232	3.1761	-0.19911	-1.6984	2.37
2484	*ASPA*	4.173	3.1661	-0.24126	-1.8435	2.37
2485	*C6orf229*	3.5508	2.5993	0.94747	0.53922	2.37
2486	*STAT3*	4.8299	1.5019	0.76425	-1.6676	2.37
2487	*ITGBL1*	4.0963	2.2861	0.71179	-0.73243	2.36
2488	*CXXC5*	4.2685	3.7461	-0.92186	-1.8941	2.36
2489	*OR4F15*	4.353	1.4248	1.3134	-1.8157	2.36
2490	*ARHGEF18*	3.5437	3.0673	0.4797	-2.4853	2.36
2491	*BMP8A*	3.6974	2.0741	1.319	-2.716	2.36
2492	*CIT*	3.0336	2.7771	1.2796	-1.8478	2.36
2493	*C11orf74*	3.6191	2.7483	0.7223	-2.1108	2.36
2494	*MYO7A*	5.0212	1.4416	0.62659	-0.3783	2.36
2495	*HEXDC*	4.1735	1.522	1.3933	0.17427	2.36
2496	*HNRNPA0*	4.546	4.1628	-1.6213	-1.63	2.36
2497	*CSF2*	3.2159	3.058	0.81265	-0.20663	2.36
2498	*AIP*	3.6033	3.072	0.40913	-0.85799	2.36
2499	*SYP*	3.5835	2.5891	0.91083	0.55001	2.36
2500	*SLC13A2*	2.7986	2.7696	1.5121	-0.68422	2.36
2501	*OR2T34*	3.7202	3.1987	0.16091	-0.44353	2.36
2502	*STAT5B*	2.4582	2.3944	2.2267	-0.85324	2.36
2503	*COL11A1*	3.8156	1.9708	1.2927	-1.1096	2.36
2504	*PGBD2*	4.2969	3.2919	-0.5113	-0.63826	2.36
2505	*CSGALNACT1*	3.8357	3.2941	-0.0525	-0.91701	2.36
2506	*ZNF222*	3.3969	2.3847	1.2934	-3.8595	2.36
2507	*RNF32*	3.5581	2.0484	1.4664	-0.76184	2.36
2508	*PCDHGC3*	3.4135	3.0653	0.59377	-1.7766	2.36
2509	*ZFP14*	4.8688	1.9148	0.28885	-0.83187	2.36
2510	*HTR3C*	5.3987	2.4169	-0.7435	-1.1836	2.36
2511	*ABCD1*	4.5089	2.6207	-0.05801	-0.54775	2.36
2512	*KCNH2*	2.9709	2.9621	1.1381	-0.51664	2.36
2513	*CD59*	4.2135	2.4387	0.41717	-0.14849	2.36
2514	*AKR1E2*	3.1361	2.5704	1.3627	-0.80436	2.36
2515	*LOC101928841*	4.1355	2.7598	0.17356	-0.52372	2.36
2516	*LIPA*	4.5301	1.4789	1.0595	-0.82833	2.36
2517	*GRB10*	4.8331	1.1721	1.0619	-0.40214	2.36
2518	*RPS21*	5.8381	2.1353	-0.90854	-1.0134	2.35
2519	*ZNF730*	3.6347	2.1542	1.2751	-0.38945	2.35
2520	*METTL21A*	4.6558	1.9634	0.44324	-1.632	2.35
2521	*ZNF214*	3.619	1.8272	1.6139	0.31183	2.35
2522	*ADAMTS10*	4.0141	2.4026	0.64207	-1.2884	2.35
2523	*ALDH1A1*	4.0494	2.5958	0.41346	-1.3495	2.35

2524	*NRD1*	4.1544	2.114	0.79022	-0.1611	2.35
2525	*NDST3*	3.2703	2.3419	1.4449	-0.49727	2.35
2526	*TSPAN13*	2.8631	2.1924	2.0011	-1.6776	2.35
2527	*MAFG*	3.9616	3.3646	-0.26968	-1.9935	2.35
2528	*MOB1B*	5.3287	2.0716	-0.34486	-1.6934	2.35
2529	*HTR3D*	4.1805	3.6922	-0.8175	-0.9562	2.35
2530	*TMEM168*	4.3665	1.8856	0.80062	-2.1058	2.35
2531	*PLXNA2*	2.874	2.5704	1.6066	-1.8538	2.35
2532	*SEZ6*	3.531	2.9727	0.54673	-1.2412	2.35
2533	*TBRG1*	5.0332	1.8243	0.19121	-0.84992	2.35
2534	*MSANTD1*	3.6784	1.8886	1.4814	-1.2692	2.35
2535	*CES3*	2.9633	2.8111	1.2736	-0.08283	2.35
2536	*PSMC6*	4.1918	3.2542	-0.39838	-1.7153	2.35
2537	*LMAN2L*	5.0462	1.1995	0.80184	-1.4684	2.35
2538	*ADAMTS19*	4.0137	3.6967	-0.66353	-0.78345	2.35
2539	*PCDH1*	3.5325	3.3053	0.20893	-0.62875	2.35
2540	*GBP5*	4.2998	2.9798	-0.23309	-0.52154	2.35
2541	*GJA3*	2.4993	2.4074	2.1382	-0.49001	2.35
2542	*IFNA6*	4.8633	1.6013	0.57427	-0.36705	2.35
2543	*C4BPA*	2.7482	2.1577	2.1323	-0.61429	2.35
2544	*RAB28*	3.9356	2.4685	0.63314	-0.27707	2.35
2545	*ZNF697*	2.738	2.4833	1.8154	-1.2118	2.35
2546	*FNDC4*	4.3827	3.7543	-1.1006	-1.8272	2.35
2547	*OTOP1*	4.1805	3.3497	-0.49389	-0.912	2.35
2548	*OR2J2*	3.0654	2.0585	1.9121	1.0927	2.35
2549	*MKLN1*	4.4243	1.814	0.79654	-1.7217	2.34
2550	*PABPC3*	2.973	2.6799	1.3789	-0.53856	2.34
2551	*GBP1*	4.1433	3.9482	-1.0613	-1.0739	2.34
2552	*CALB1*	3.8837	2.0977	1.0484	-0.11514	2.34
2553	*ZNF302*	2.5661	2.3435	2.1199	-1.2936	2.34
2554	*PKP2*	3.9064	3.2434	-0.12053	-0.36295	2.34
2555	*WWTR1*	3.5886	3.5068	-0.06657	-0.92868	2.34
2556	*OR10H2*	4.1562	2.773	0.09874	-1.3371	2.34
2557	*NXT2*	5.3852	0.90318	0.73816	-0.16749	2.34
2558	*EXOSC3*	4.7991	1.4668	0.7603	-0.07903	2.34
2559	*CCDC64B*	4.9432	1.2379	0.84157	-0.11902	2.34
2560	*SH3BP4*	4.1585	2.3859	0.47778	-0.50809	2.34
2561	*SMARCE1*	3.6983	2.4191	0.90347	0.6891	2.34
2562	*ZNF695*	4.7366	1.4812	0.79954	-0.10753	2.34
2563	*VTCN1*	3.3791	2.7107	0.92685	-1.7505	2.34
2564	*LRRC17*	3.3591	1.9709	1.6822	0.78117	2.34
2565	*FBXO27*	3.2252	2.9374	0.84897	0.30181	2.34
2566	*GFRA1*	4.6682	1.2788	1.0644	0.24059	2.34
2567	*SPANXN1*	3.7202	1.7023	1.5886	0.069884	2.34
2568	*OR14A16*	5.1847	0.95637	0.86844	0.60637	2.34
2569	*DSCR3*	2.9934	2.476	1.5401	0.48579	2.34

2570	*HIST2H2AC*	3.3736	3.2757	0.35928	-0.78098	2.34
2571	*CTHRC1*	2.6644	2.3368	2.0069	-1.295	2.34
2572	*RLN1*	3.7156	2.484	0.80838	0.49314	2.34
2573	*GNAQ*	4.9715	1.3944	0.64091	-0.67814	2.34
2574	*SERPINE2*	2.8711	2.3278	1.8079	-0.41754	2.34
2575	*CYP26B1*	4.372	2.1467	0.48753	0.06222	2.34
2576	*IL1RAP*	3.4634	2.0092	1.5329	1.3255	2.34
2577	*C8orf87*	4.103	2.7389	0.15971	-0.00014	2.33
2578	*MUC7*	3.0251	2.7092	1.2665	1.0576	2.33
2579	*FGB*	4.0113	3.2417	-0.25225	-1.4256	2.33
2580	*CCDC144NL*	5.0334	1.1931	0.7741	-1.5517	2.33
2581	*BACH2*	3.7188	3.481	-0.20256	-0.27771	2.33
2582	*SPRR4*	5.0586	1.5304	0.40794	-2.3145	2.33
2583	*ARV1*	4.3773	2.6279	-0.00828	-1.7297	2.33
2584	*GLIS2*	3.1426	2.7601	1.0938	-0.17081	2.33
2585	*PODNL1*	2.9653	2.4771	1.5535	-0.07496	2.33
2586	*CXCL6*	4.1077	1.4816	1.4056	1.0734	2.33
2587	*PTPN2*	4.6945	1.8082	0.49202	-1.3837	2.33
2588	*INPP5F*	3.9236	2.5646	0.50498	-1.4981	2.33
2589	*RNF4*	3.9458	2.5725	0.47313	-0.55509	2.33
2590	*KRTAP10-10*	3.5398	2.5749	0.8766	-1.4469	2.33
2591	*TCAIM*	4.8683	2.59	-0.4676	-0.75669	2.33
2592	*DNAJB11*	3.5538	2.5389	0.89701	-1.9582	2.33
2593	*APBA2*	5.2689	1.4973	0.22314	-0.82166	2.33
2594	*LOXL2*	3.6522	2.4546	0.88172	-1.2013	2.33
2595	*GJD3*	3.7828	3.2457	-0.04174	-0.34535	2.33
2596	*ACD*	2.6127	2.4614	1.912	-0.55418	2.33
2597	*VGLL3*	4.3751	2.1253	0.48567	-0.14568	2.33
2598	*AK3*	3.7661	1.8182	1.4015	-1.9728	2.33
2599	*GALK1*	5.0528	2.4733	-0.54155	-0.8604	2.33
2600	*DECR1*	3.8899	2.8998	0.19425	-0.10289	2.33
2601	*ZNF559*	3.6214	1.8312	1.5305	-0.43077	2.33
2602	*OR6Y1*	3.8637	3.616	-0.49746	-0.61334	2.33
2603	*TNFSF14*	3.0078	2.955	1.0177	-1.2086	2.33
2604	*ISM2*	4.5552	1.7852	0.63918	-1.2395	2.33
2605	*MTX2*	3.8415	2.4499	0.68801	-0.45663	2.33
2606	*CCDC168*	5.8236	2.4	-1.246	-2.1687	2.33
2607	*KRT13*	4.1852	1.5975	1.1934	-0.31934	2.33
2608	*PPP1R16A*	4.9564	2.0833	-0.06539	-1.7194	2.32
2609	*SLC34A3*	3.9264	3.4813	-0.43365	-1.0528	2.32
2610	*MRPL39*	4.071	2.7257	0.17695	-0.73944	2.32
2611	*CCDC169*	4.4484	2.558	-0.03289	-0.0801	2.32
2612	*STBD1*	3.7707	3.4014	-0.19943	-1.4736	2.32
2613	*SIRT7*	4.6483	1.6284	0.69513	-1.7001	2.32
2614	*LCE6A*	3.6915	2.7463	0.53265	-1.215	2.32
2615	*ZNF860*	4.0565	1.9722	0.94162	-1.6075	2.32

2616	*CPT2*	2.9603	2.9063	1.1036	-2.0171	2.32
2617	*TPCN2*	2.961	2.0534	1.9557	1.8451	2.32
2618	*RUSC1*	4.0261	2.9198	0.02335	-0.53031	2.32
2619	*PTPRT*	5.3814	2.2568	-0.66914	-1.2846	2.32
2620	*HTRA1*	3.553	3.3946	0.02124	-1.3371	2.32
2621	*DNAJC10*	2.6654	2.4656	1.8343	-1.0007	2.32
2622	*RDH11*	4.451	2.1833	0.3307	-1.4415	2.32
2623	*CHRD*	4.1733	1.65	1.1411	-1.9996	2.32
2624	*GNG11*	3.8142	3.36	-0.20983	-1.0099	2.32
2625	*FBXO40*	4.5537	2.5207	-0.11076	-0.80897	2.32
2626	*PPP6R1*	4.6496	1.4233	0.88999	-0.5605	2.32
2627	*KRTAP19-7*	3.3033	2.9324	0.72714	-0.95321	2.32
2628	*TFF2*	4.8823	2.0532	0.02701	-0.97611	2.32
2629	*ADAMTS15*	4.5057	2.8112	-0.3554	-0.41227	2.32
2630	*ZNF670*	4.2496	1.8628	0.84899	-0.78229	2.32
2631	*C1QTNF5*	2.9445	2.9171	1.0995	-1.1223	2.32
2632	*NCAN*	2.8659	2.2191	1.8752	0.18375	2.32
2633	*RFX2*	4.1982	2.2257	0.53539	-2.189	2.32
2634	*HOOK2*	3.6481	2.6223	0.68701	-0.07847	2.32
2635	*IL4R*	4.3094	2.1922	0.45378	-2.1873	2.32
2636	*HOXC11*	3.9097	3.2937	-0.24966	-1.3976	2.32
2637	*EPHX2*	4.3188	3.6143	-0.97952	-1.7078	2.32
2638	*PCDHA4*	4.0905	1.7611	1.1019	-1.7836	2.32
2639	*DHRS12*	3.6669	3.4429	-0.15787	-1.9008	2.32
2640	*C9*	3.6701	2.4661	0.81502	0.23092	2.32
2641	*SLC22A18*	4.3254	1.6903	0.93499	-0.01337	2.32
2642	*DEFB129*	3.9973	2.0661	0.88699	-1.4606	2.32
2643	*PPP1R3G*	3.9942	2.2822	0.67348	-0.33933	2.32
2644	*FFAR3*	5.0604	1.8687	0.02021	-0.15455	2.32
2645	*CCDC179*	3.9808	2.3822	0.58593	-0.95258	2.32
2646	*RBP5*	3.4633	2.0616	1.424	-2.3949	2.32
2647	*ELMO2*	4.0956	1.4659	1.3866	-0.86793	2.32
2648	*ZNF211*	4.2249	2.4855	0.2372	-1.3126	2.32
2649	*CECR1*	4.1376	3.7028	-0.89699	-1.8382	2.31
2650	*KRTAP19-8*	3.7758	2.9937	0.17389	-0.5287	2.31
2651	*CFAP97*	3.0821	2.7825	1.0787	-1.3554	2.31
2652	*SUSD5*	4.2947	2.9022	-0.25403	-1.1069	2.31
2653	*CYP2C9*	5.0588	2.5471	-0.66445	-1.0544	2.31
2654	*LRRC8A*	4.6143	2.2928	0.03422	-2.0436	2.31
2655	*RBM6*	3.3969	3.3639	0.17894	-0.20673	2.31
2656	*PPIG*	5.1261	1.5662	0.24682	-1.2845	2.31
2657	*TAT*	3.1844	2.6879	1.0663	-0.1059	2.31
2658	*NGF*	5.2549	1.1046	0.57803	-2.1983	2.31
2659	*TARS2*	4.9561	2.2627	-0.28159	-1.0792	2.31
2660	*AQP10*	2.7912	2.4039	1.7413	-2.8478	2.31
2661	*PLSCR5*	4.4743	2.4581	0.00309	-3.3806	2.31

2662	*ZBTB34*	3.0364	1.9873	1.9102	0.25651	2.31
2663	*ASPH*	3.9701	2.8387	0.12122	-1.3666	2.31
2664	*SLC1A7*	2.8879	2.6499	1.3918	-1.624	2.31
2665	*C17orf98*	4.3605	2.4361	0.13298	-0.12565	2.31
2666	*TCEAL7*	4.0029	3.5146	-0.58806	-1.1174	2.31
2667	*SIRPB1*	3.1309	2.6846	1.113	-1.3694	2.31
2668	*ZKSCAN7*	4.5625	3.971	-1.6058	-1.9073	2.31
2669	*KLC4*	3.5248	2.1136	1.2867	-0.55117	2.31
2670	*UNC45A*	4.7013	3.0495	-0.82582	-1.8909	2.31
2671	*OR5T3*	4.0256	2.8297	0.0695	-1.164	2.31
2672	*TRIP4*	3.9074	3.4603	-0.44374	-1.3755	2.31
2673	*URI1*	3.9269	3.5261	-0.52978	-1.2567	2.31
2674	*CLDN17*	3.4435	3.4299	0.04956	-0.735	2.31
2675	*LOC403312*	3.6677	2.2052	1.0498	-0.35379	2.31
2676	*PRL*	5.0734	2.7722	-0.92326	-1.0669	2.31
2677	*DBH*	5.8476	1.3634	-0.2889	-1.4168	2.31
2678	*LY96*	3.0511	2.6884	1.1823	-1.9567	2.31
2679	*C9orf117*	3.7505	2.8328	0.33744	-0.82631	2.31
2680	*SNX20*	4.422	1.623	0.87543	-0.73198	2.31
2681	*PKP3*	4.8694	1.3612	0.68934	-0.4778	2.31
2682	*RAET1G*	2.7965	2.0801	2.0424	-1.5884	2.31
2683	*EDA*	3.9191	1.7427	1.2564	0.91314	2.31
2684	*C19orf24*	4.3721	2.6998	-0.15499	-1.0221	2.31
2685	*CEP104*	4.838	1.1778	0.90056	0.50176	2.31
2686	*YDJC*	3.8134	1.7529	1.3494	-0.74604	2.31
2687	*TTC36*	5.0516	1.2608	0.60136	-2.2495	2.30
2688	*OR2B11*	3.2467	2.4897	1.1756	0.87825	2.30
2689	*SLC35A5*	4.3669	3.0023	-0.45893	-0.82573	2.30
2690	*ZHX1*	4.0746	2.0593	0.77557	0.066534	2.30
2691	*CTAGE6*	3.6786	2.5899	0.63921	-1.5848	2.30
2692	*ZNF395*	4.575	2.3818	-0.04968	-0.84523	2.30
2693	*ZNF600*	4.5392	3.7515	-1.3837	-1.4893	2.30
2694	*TRPC6*	3.9521	2.58	0.37386	-0.50048	2.30
2695	*MAP4K2*	4.1764	3.576	-0.84731	-0.86007	2.30
2696	*GKN1*	3.5223	2.2329	1.1482	-1.5737	2.30
2697	*GEM*	4.3658	1.4958	1.0401	-0.45069	2.30
2698	*HES5*	3.5605	2.478	0.86202	-1.729	2.30
2699	*NKX2-8*	3.6264	2.2714	1.0027	-0.68514	2.30
2700	*SLCO2A1*	4.6677	2.163	0.06892	-1.0458	2.30
2701	*ALG9*	3.8589	2.1525	0.88743	-1.2174	2.30
2702	*C12orf66*	4.7702	2.3288	-0.20194	-0.9728	2.30
2703	*NEK5*	3.0242	2.4373	1.4345	1.4129	2.30
2704	*HTATIP2*	4.0942	1.7197	1.0816	-1.7203	2.30
2705	*MBLAC2*	5.1127	2.2704	-0.48821	-0.78279	2.30
2706	*HESX1*	3.7125	2.3359	0.84631	-1.4856	2.30
2707	*GNAI1*	2.7407	2.2976	1.8541	-1.0626	2.30

2708	*BIRC6*	3.147	2.5662	1.1775	-3.1673	2.30
2709	*IL33*	4.4835	2.008	0.39643	-1.7675	2.30
2710	*LRRC71*	4.8108	2.0702	0.00665	-1.6453	2.30
2711	*RBM43*	4.3286	1.7574	0.8015	0.22658	2.30
2712	*FAM213A*	2.6145	2.484	1.7883	0.77673	2.30
2713	*MFAP3*	3.4545	2.4743	0.95785	-1.4789	2.30
2714	*CORO6*	3.882	2.5821	0.42162	-1.308	2.30
2715	*ATP11C*	3.6787	2.6805	0.526	-1.6684	2.30
2716	*PAPD7*	2.6379	2.5287	1.7184	-0.72635	2.30
2717	*RUNX1*	4.3414	2.2351	0.30692	-0.04053	2.29
2718	*CC2D2A*	3.6684	2.5447	0.66966	0.28842	2.29
2719	*BCL2L2*	3.794	3.1633	-0.07689	-0.79389	2.29
2720	*TRPM8*	4.3866	2.717	-0.22669	-0.23418	2.29
2721	*UBTD2*	3.7518	2.3514	0.77362	-2.0952	2.29
2722	*PSMB8*	4.3672	3.6134	-1.1053	-1.6664	2.29
2723	*TMEM11*	3.9764	3.1258	-0.22758	-0.57573	2.29
2724	*A2ML1*	5.1336	2.0429	-0.30218	-0.81034	2.29
2725	*EPHA6*	4.9522	1.2183	0.70302	0.047775	2.29
2726	*PPIL6*	3.6589	3.1974	0.01511	-1.5386	2.29
2727	*PRKAR1A*	3.3935	3.3442	0.13368	-2.4205	2.29
2728	*ASPN*	3.6154	2.4491	0.80583	-0.77184	2.29
2729	*TRAF4*	4.0579	1.7879	1.0245	-0.28164	2.29
2730	*ASCL4*	3.9733	1.5994	1.2972	0.43635	2.29
2731	*TMPRSS5*	4.2108	2.0899	0.56845	-0.80069	2.29
2732	*PET100*	5.0128	1.2438	0.61161	0.091077	2.29
2733	*KLRC4-KLRK1*	4.6691	2.0803	0.11542	-2.5442	2.29
2734	*TRIM36*	3.7027	3.6371	-0.47739	-0.95717	2.29
2735	*DHX36*	3.0282	2.6844	1.1491	0.098411	2.29
2736	*OR5P3*	3.2043	3.0662	0.59007	-0.0201	2.29
2737	*HAL*	3.4556	2.522	0.88288	-0.26518	2.29
2738	*CD276*	3.3214	2.1506	1.3869	-0.99175	2.29
2739	*CD163*	4.2243	1.8322	0.80154	-0.15394	2.29
2740	*PKD2L2*	3.595	2.2054	1.0465	0.82453	2.28
2741	*IQGAP1*	3.217	2.3999	1.2278	-0.55473	2.28
2742	*KLHL17*	4.3251	1.42	1.0995	0.52108	2.28
2743	*C6orf222*	4.2696	1.4438	1.1311	0.61322	2.28
2744	*ABCD3*	2.8557	2.4076	1.581	0.46801	2.28
2745	*GKN2*	3.7347	1.9652	1.144	-0.10484	2.28
2746	*PSTK*	2.5264	2.166	2.1509	-1.3888	2.28
2747	*SV2C*	3.2428	1.909	1.6906	0.4766	2.28
2748	*PCDHGB1*	5.2491	1.386	0.20588	-0.55441	2.28
2749	*FCRL4*	4.2751	3.0707	-0.50503	-0.74069	2.28
2750	*MAGEB1*	5.5725	1.449	-0.18124	-2.071	2.28
2751	*CHD1*	3.0461	3.0148	0.77802	-1.7278	2.28
2752	*IL36B*	3.6491	2.0503	1.1388	-1.1646	2.28
2753	*COLQ*	2.8726	2.694	1.2715	0.15898	2.28

2754	*RUSC2*	3.9276	3.8174	-0.90835	-1.3997	2.28
2755	*PFN3*	4.6666	1.7922	0.37684	-0.98031	2.28
2756	*GJD4*	4.6303	2.0139	0.19132	-0.44084	2.28
2757	*RARRES3*	3.887	2.972	-0.02371	-0.36315	2.28
2758	*CUL4B*	4.1932	2.0614	0.57945	-1.631	2.28
2759	*TEX19*	4.6417	1.2079	0.98421	-1.7845	2.28
2760	*EIF3K*	3.5946	2.301	0.93794	-0.578	2.28
2761	*C12orf49*	2.8014	2.1248	1.9062	-0.2531	2.28
2762	*SMIM13*	4.0111	2.4525	0.36853	-1.1043	2.28
2763	*PTPN14*	4.6005	2.5237	-0.29227	-1.4031	2.28
2764	*LONRF2*	3.055	2.4948	1.2793	-1.2896	2.28
2765	*ADRA1A*	4.91	1.9207	-0.0027	-1.2617	2.28
2766	*CHRNB4*	3.3993	2.5485	0.87878	-0.00823	2.28
2767	*MAP1LC3B*	3.8924	2.9253	0.00788	-1.0573	2.28
2768	*GATA4*	3.6447	2.1747	1.0059	0.019931	2.28
2769	*GPC6*	4.6872	2.7372	-0.60267	-0.9703	2.27
2770	*TMEM81*	2.7971	2.2735	1.7502	-0.58534	2.27
2771	*PNPLA4*	4.2475	2.7244	-0.15203	-1.5255	2.27
2772	*C5orf66*	4.4262	1.9278	0.46462	0.44056	2.27
2773	*STOML3*	3.473	2.7884	0.55662	-0.70014	2.27
2774	*IL26*	4.4176	3.3597	-0.9606	-2.5795	2.27
2775	*C16orf93*	4.2941	2.919	-0.39664	-2.5546	2.27
2776	*CXorf58*	3.3695	2.7237	0.72144	0.40396	2.27
2777	*TMEM265*	3.4494	2.3595	1.0026	-0.12428	2.27
2778	*SAAL1*	5.6644	2.4231	-1.2766	-1.4325	2.27
2779	*FBXL2*	4.0137	3.0934	-0.29632	-0.74833	2.27
2780	*LYZL4*	5.294	2.4982	-0.98159	-1.3504	2.27
2781	*APEX2*	3.5432	2.5178	0.74552	0.36493	2.27
2782	*SERPINE1*	5.4594	1.4088	-0.06199	-0.69245	2.27
2783	*DCAF4*	4.1312	3.0777	-0.40292	-1.9959	2.27
2784	*INIP*	4.3393	2.0955	0.37008	0.33331	2.27
2785	*LHCGR*	2.9006	2.4458	1.4569	-0.23375	2.27
2786	*MAGEA3*	3.8956	2.6635	0.24414	-1.8963	2.27
2787	*UTS2R*	3.7633	2.5055	0.53325	-1.468	2.27
2788	*TAS2R20*	3.8439	3.4367	-0.47884	-1.2118	2.27
2789	*DBX1*	3.6151	1.9768	1.2096	-0.49647	2.27
2790	*CAP2*	3.4496	3.22	0.13093	-0.42195	2.27
2791	*ITIH1*	3.8667	2.6371	0.29191	0.25364	2.27
2792	*COL7A1*	5.0016	2.8595	-1.0654	-2.3885	2.27
2793	*ADCY8*	3.6523	3.557	-0.41395	-1.6648	2.27
2794	*FOXR2*	4.9849	3.0085	-1.2015	-1.2572	2.26
2795	*SMAD3*	4.0106	3.415	-0.63415	-1.4311	2.26
2796	*MKRN1*	4.2439	1.3779	1.1689	0.9534	2.26
2797	*NOX5*	3.9087	3.521	-0.6399	-0.71669	2.26
2798	*SCGB2A2*	3.5012	2.7682	0.52022	-0.63709	2.26
2799	*PAGE3*	4.003	2.5852	0.20127	-1.2951	2.26

2800	*CDRT1*	4.3512	2.5235	-0.08601	-2.4661	2.26
2801	*CHMP2B*	4.5777	3.615	-1.4055	-1.4614	2.26
2802	*ZNF749*	4.2712	3.1752	-0.65946	-0.86787	2.26
2803	*TRABD2B*	2.5747	2.2783	1.9324	-0.91427	2.26
2804	*KIF23*	3.3515	3.1239	0.30673	-2.0614	2.26
2805	*SYDE1*	3.1831	1.8714	1.7273	1.1238	2.26
2806	*DYRK1A*	4.55	2.8036	-0.57308	-1.2277	2.26
2807	*SOX10*	3.3186	1.8114	1.6486	-0.12474	2.26
2808	*BTLA*	4.9159	3.5618	-1.6992	-2.9931	2.26
2809	*SEMA4C*	4.3053	1.5802	0.89273	-0.28258	2.26
2810	*BEND6*	3.346	2.7983	0.63356	-1.1766	2.26
2811	*GUCA1B*	4.6967	1.839	0.24169	-2.5593	2.26
2812	*PMP2*	2.4724	2.3185	1.9863	1.027	2.26
2813	*STX11*	3.313	2.816	0.64637	0.071769	2.26
2814	*FAM107A*	2.849	2.0882	1.8341	-5.3303	2.26
2815	*ENOSF1*	2.9748	1.9052	1.8905	-1.5091	2.26
2816	*CHMP1A*	3.0156	2.6063	1.1485	0.6339	2.26
2817	*ENDOV*	2.4417	2.2696	2.0591	-0.36281	2.26
2818	*FANCB*	4.2698	1.8769	0.62175	0.22862	2.26
2819	*LGALS9C*	3.3316	1.9134	1.5219	-1.4921	2.26
2820	*PAK1*	3.8373	3.819	-0.8897	-1.9023	2.26
2821	*CCDC117*	4.3937	2.4954	-0.12259	-0.40231	2.26
2822	*GPR135*	5.376	0.72478	0.66404	0.24793	2.25
2823	*BACE1*	2.7923	2.113	1.8595	-0.8338	2.25
2824	*GJB2*	3.3095	2.5607	0.89421	-0.25822	2.25
2825	*LYPD5*	3.5959	2.7422	0.42579	-1.0149	2.25
2826	*ARF6*	3.3497	2.9738	0.43727	-1.7119	2.25
2827	*PTX3*	4.3858	2.2198	0.15514	-1.236	2.25
2828	*LLGL1*	3.1852	2.0557	1.5196	-0.60032	2.25
2829	*ICOSLG*	4.8041	1.8623	0.09301	-0.69287	2.25
2830	*LTBP4*	2.6785	2.3355	1.745	-0.26747	2.25
2831	*FAM47C*	4.4245	3.5214	-1.1879	-1.5165	2.25
2832	*WI2-2373I1.2*	5.1626	1.6186	-0.0236	-1.3183	2.25
2833	*SPTBN2*	4.9517	1.7803	0.0251	-2.0059	2.25
2834	*PRR27*	2.9715	1.9385	1.8468	1.0352	2.25
2835	*UBC*	3.5385	1.6341	1.5837	0.78181	2.25
2836	*PANX1*	3.4365	2.5946	0.72441	-1.8297	2.25
2837	*ORM2*	3.5711	2.8121	0.37157	-1.6086	2.25
2838	*APH1A*	3.8187	2.7683	0.16742	0.083808	2.25
2839	*NFKBID*	4.6687	1.732	0.35364	0.099866	2.25
2840	*KIAA1522*	3.0109	2.6388	1.1042	-0.73155	2.25
2841	*PKP4*	4.2968	2.968	-0.51146	-1.5204	2.25
2842	*PFKFB3*	5.2481	2.9868	-1.4829	-1.6913	2.25
2843	*ANKRD18A*	3.6809	2.9364	0.13166	-0.84713	2.25
2844	*ASB2*	4.1045	2.1643	0.47991	-0.33524	2.25
2845	*ARSJ*	3.779	3.183	-0.21348	-1.8154	2.25

2846	*PLA2G2F*	3.8515	3.5486	-0.65169	-0.78229	2.25
2847	*S1PR2*	3.355	3.2945	0.09832	-0.55554	2.25
2848	*SLC8A2*	5.983	0.44198	0.32263	0.14975	2.25
2849	*SPRYD7*	4.4892	2.3001	-0.04343	-0.64467	2.25
2850	*CCNI*	3.3874	3.2315	0.1264	-0.38589	2.25
2851	*LINS*	5.6091	0.90635	0.22738	-0.20758	2.25
2852	*GPRC5D*	3.699	2.5986	0.44429	-2.0042	2.25
2853	*MAB21L1*	3.2961	2.0296	1.4161	-1.3296	2.25
2854	*IL17RD*	2.8317	2.5984	1.3095	-0.35325	2.25
2855	*SLC30A3*	5.2126	2.7084	-1.1815	-3.8687	2.25
2856	*PLD6*	5.588	1.2832	-0.13185	-1.0283	2.25
2857	*TMEM184A*	3.5182	3.2517	-0.03153	-0.97585	2.25
2858	*FAM155B*	3.757	2.4513	0.52983	-0.811	2.25
2859	*PARP14*	3.991	2.9222	-0.17566	-1.0797	2.25
2860	*LPAR5*	2.5367	2.364	1.8367	-0.74428	2.25
2861	*ETFDH*	3.4616	2.9077	0.3679	-2.1066	2.25
2862	*PLIN4*	3.6036	3.0056	0.12787	-0.42661	2.25
2863	*FREM2*	3.976	3.4452	-0.68466	-1.2291	2.25
2864	*CCSER1*	3.8937	3.5964	-0.75369	-1.0014	2.25
2865	*SLC25A44*	3.1285	2.1005	1.5073	1.4622	2.25
2866	*CYP2D6*	3.7051	2.9198	0.11095	-1.0704	2.25
2867	*SSX7*	3.7215	1.7296	1.2832	-2.401	2.24
2868	*C9orf153*	3.719	3.272	-0.25854	-0.75173	2.24
2869	*MAGEA5*	4.0851	2.5767	0.07047	-1.9624	2.24
2870	*OR1L3*	3.1822	2.8513	0.69842	-0.00317	2.24
2871	*FAM174A*	3.278	2.2209	1.2329	-1.0139	2.24
2872	*CGB7*	4.4641	1.4263	0.84021	-2.0384	2.24
2873	*SUPT20H*	3.5608	2.7452	0.42438	0.21115	2.24
2874	*FAM195A*	3.2586	3.1712	0.30034	-1.8851	2.24
2875	*GATA1*	4.9577	3.5864	-1.8154	-2.224	2.24
2876	*ECM2*	3.2868	1.9867	1.4547	0.33169	2.24
2877	*SPHAR*	5.1119	-0.62666			2.24
2878	*OR4D1*	2.6392	2.3775	1.7104	-0.97667	2.24
2879	*WDSUB1*	4.1818	2.334	0.2113	-1.9495	2.24
2880	*ADAM28*	3.9189	2.7311	0.07667	-0.66462	2.24
2881	*ACP2*	4.4057	4.2028	-1.882	-5.3303	2.24
2882	*CLMP*	3.4438	3.1143	0.16765	-1.237	2.24
2883	*LAX1*	4.3913	2.434	-0.09965	-1.0662	2.24
2884	*AMELX*	4.2872	1.3563	1.0819	0.24364	2.24
2885	*MOB4*	3.9278	2.7053	0.09207	-0.31145	2.24
2886	*PDK1*	4.185	1.5123	1.0277	-0.14425	2.24
2887	*EDN1*	5.3522	1.0295	0.34077	0.33438	2.24
2888	*VIPAS39*	3.5604	1.6357	1.5244	-0.71936	2.24
2889	*CT47B1*	3.1091	2.9189	0.69213	0.033607	2.24
2890	*TTC39A*	4.66	2.1407	-0.08232	-1.3916	2.24
2891	*TRPM2*	3.9497	2.9833	-0.21532	-1.0087	2.24

2892	*NLGN4X*	3.4407	2.3454	0.9314	0.83177	2.24
2893	*CDH6*	3.0721	2.8036	0.84066	-1.6559	2.24
2894	*TM2D2*	2.9034	2.5638	1.2484	-2.1523	2.24
2895	*SEC14L4*	5.1337	1.4784	0.10337	-0.27451	2.24
2896	*LRP5*	2.7033	2.5008	1.5109	0.39741	2.24
2897	*PLXNB3*	4.3104	3.295	-0.89194	-0.97227	2.24
2898	*ASPM*	5.1938	2.3585	-0.83912	-1.5031	2.24
2899	*RAET1L*	3.6549	2.4008	0.65618	-0.47186	2.24
2900	*DEFB107B*	2.2371				2.24
2901	*ZNF273*	3.7936	1.742	1.1733	-0.4013	2.24
2902	*ADGRL4*	2.6693	2.07	1.9687	-1.9624	2.24
2903	*ACSF3*	2.7238	2.4431	1.5405	-0.40785	2.24
2904	*CRLS1*	4.7157	3.0445	-1.053	-1.8575	2.24
2905	*TRMT1L*	3.7541	2.8201	0.13231	-1.4233	2.24
2906	*THEG5*	5.0315	1.0778	0.59644	-1.2284	2.24
2907	*MUM1L1*	3.2704	1.8538	1.5812	-1.2417	2.24
2908	*TYW5*	2.713	2.5111	1.4812	-0.77196	2.24
2909	*OR51I2*	3.4322	1.9168	1.3546	-0.07232	2.23
2910	*MAT2A*	3.6556	2.5094	0.53703	-0.95064	2.23
2911	*GALNT3*	4.6717	2.8532	-0.82368	-1.7512	2.23
2912	*AKR1C1*	5.8028	0.64061	0.25776	-2.5007	2.23
2913	*HMGB3*	5.6489	0.72379	0.32753	-1.1233	2.23
2914	*HIC1*	4.3879	3.1348	-0.82272	-1.2912	2.23
2915	*ETV3L*	3.597	3.4194	-0.31815	-2.0978	2.23
2916	*OXT*	3.3259	2.0758	1.2964	-1.1211	2.23
2917	*ACCS*	3.0802	2.8085	0.8092	-0.68565	2.23
2918	*RNF220*	3.2907	2.2256	1.1814	-0.38163	2.23
2919	*C20orf85*	2.4985	2.479	1.7199	-1.3593	2.23
2920	*RNF17*	4.9221	1.5917	0.18297	-1.0633	2.23
2921	*ERCC6L*	3.7929	2.5984	0.30419	-0.53437	2.23
2922	*HIGD1B*	3.975	1.5526	1.1674	-1.2456	2.23
2923	*LOC101928093*	4.1719	2.8625	-0.34093	-1.5739	2.23
2924	*PNPLA7*	5.3939	2.2353	-0.93789	-2.7214	2.23
2925	*UBE3C*	4.5007	2.8324	-0.64229	-1.2691	2.23
2926	*PELP1*	5.3609	1.7477	-0.41803	-0.68606	2.23
2927	*HRH3*	3.3198	2.0529	1.3164	-1.0189	2.23
2928	*XKRX*	5.2407	1.3529	0.09422	-0.87662	2.23
2929	*C16orf78*	3.204	2.487	0.99541	0.19092	2.23
2930	*FOXI1*	3.1128	2.6812	0.89213	0.63025	2.23
2931	*THNSL2*	3.4615	2.7888	0.43502	-0.38677	2.23
2932	*LOC100130451*	3.9784	2.2624	0.44385	0.42014	2.23
2933	*MICU1*	5.3741	0.71749	0.5922	-0.80379	2.23
2934	*SLC25A29*	4.053	2.7754	-0.1449	-1.8318	2.23
2935	*CNKSR1*	4.4594	2.4574	-0.23445	-0.47726	2.23
2936	*DNAJC5G*	4.3817	1.5814	0.71918	-0.53406	2.23
2937	*LAMTOR2*	2.9882	2.1877	1.5058	1.094	2.23

2938	*FNDC3B*	3.8915	2.8404	-0.05104	-0.53036	2.23
2939	*SCYL2*	2.8305	2.0834	1.7663	1.5354	2.23
2940	*C4orf29*	5.6751	0.65926	0.34578	-2.3517	2.23
2941	*CXorf66*	3.2785	2.5967	0.80183	-1.0948	2.23
2942	*CPPED1*	3.6745	1.6303	1.3709	-1.4745	2.23
2943	*SLC44A1*	4.5892	3.9237	-1.8376	-2.0062	2.23
2944	*FBXO46*	3.2942	1.8321	1.5477	-0.91134	2.22
2945	*FAM168A*	2.9744	2.3234	1.3754	-0.00823	2.22
2946	*LYSMD1*	5.279	1.7789	-0.38611	-0.97252	2.22
2947	*RBAK*	2.9097	2.8122	0.94988	0.4969	2.22
2948	*CCDC85A*	3.2856	2.6361	0.74769	0.68386	2.22
2949	*STMN1*	5.5804	0.60294	0.48494	-1.2301	2.22
2950	*SEC24C*	5.9597	0.64923	0.05766	-0.18568	2.22
2951	*ANKRD6*	3.7638	2.5288	0.37314	-1.4401	2.22
2952	*ARSK*	3.4223	3.3347	-0.09253	-0.5018	2.22
2953	*CRYZL1*	4.1079	2.1854	0.37085	-0.60082	2.22
2954	*SDCBP2*	4.456	2.6099	-0.40577	-0.65557	2.22
2955	*CYBA*	3.6515	3.0901	-0.08234	-2.058	2.22
2956	*DENND6B*	3.6318	3.081	-0.05603	-2.1418	2.22
2957	*FZD6*	3.4519	2.9047	0.29904	-1.7662	2.22
2958	*DIRAS1*	5.0422	0.88303	0.7291	0.43955	2.22
2959	*ZNF534*	5.0523	1.4637	0.13811	0.053745	2.22
2960	*OR7C2*	4.6561	1.4071	0.59087	0.39993	2.22
2961	*FUNDC1*	3.3212	2.7707	0.56196	-0.98717	2.22
2962	*EDF1*	3.6095	2.8992	0.14491	-3.2979	2.22
2963	*C6orf106*	3.8779	3.4435	-0.66787	-1.3148	2.22
2964	*ACKR3*	4.4758	1.7987	0.37887	0.099654	2.22
2965	*TLE1*	3.0589	1.9355	1.6588	-0.7829	2.22
2966	*CRIP2*	2.8989	2.0003	1.7523	-1.5744	2.22
2967	*CHURC1-FNTB*	3.823	3.2711	-0.44353	-2.1547	2.22
2968	*SERGEF*	3.0403	1.9516	1.6578	-0.89851	2.22
2969	*ZNF560*	3.1934	1.7729	1.6817	1.5276	2.22
2970	*ZSCAN30*	3.8543	3.6817	-0.89	-2.485	2.22
2971	*TMEM255B*	4.6106	2.7094	-0.67504	-0.77627	2.21
2972	*APIP*	5.5515	0.75238	0.34102	-1.0198	2.21
2973	*ZNF544*	5.0746	1.8552	-0.28761	-0.77934	2.21
2974	*APBB3*	4.5868	1.6806	0.37475	-1.4291	2.21
2975	*LVRN*	3.6579	2.6495	0.33377	-0.74505	2.21
2976	*ZNF714*	4.9497	0.9731	0.71807	-1.5876	2.21
2977	*EZH1*	3.9161	2.2846	0.43954	-0.19834	2.21
2978	*ZNF80*	3.9936	3.8464	-1.1998	-2.8553	2.21
2979	*TMEM189*	4.0294	3.2635	-0.65545	-3.0312	2.21
2980	*CA11*	4.5531	1.0975	0.98576	-0.67853	2.21
2981	*LGALS1*	4.1607	1.7749	0.70052	0.35737	2.21
2982	*ABCA8*	4.2631	3.4157	-1.0433	-1.714	2.21
2983	*FGF1*	4.0281	3.7002	-1.0951	-1.6939	2.21

2984	*SUDS3*	4.3316	3.337	-1.0359	-1.3565	2.21
2985	*KRR1*	3.9151	2.7789	-0.062	-1.7705	2.21
2986	*KRTAP20-3*	4.2729	3.4314	-1.0726	-1.4926	2.21
2987	*THYN1*	4.4267	2.4619	-0.25753	-0.27491	2.21
2988	*LYPD3*	2.7845	2.352	1.4924	-0.01111	2.21
2989	*GPR139*	3.3747	2.3837	0.86834	-1.1306	2.21
2990	*ZNF771*	4.4581	2.6119	-0.44353	-1.195	2.21
2991	*FLRT3*	5.863	1.5029	-0.73961	-1.95	2.21
2992	*ME1*	3.9143	1.5892	1.1224	-1.5791	2.21
2993	*HMGN3*	4.2834	2.809	-0.4677	-0.92623	2.21
2994	*OR2T33*	4.7379	1.2906	0.59588	0.17958	2.21
2995	*ACRBP*	3.7776	2.9091	-0.06337	-2.7245	2.21
2996	*COL22A1*	3.4267	1.8875	1.3083	-0.6726	2.21
2997	*LILRB3*	4.42	2.3077	-0.1059	-1.1021	2.21
2998	*LYPD4*	3.2074	1.942	1.4722	0.4371	2.21
2999	*MAGOHB*	4.593	1.3972	0.63011	0.57106	2.21
3000	*CYP4F22*	5.34	1.8649	-0.58495	-1.2572	2.21
3001	*AGER*	3.4532	2.043	1.1207	-1.2668	2.21
3002	*FCER1A*	3.1815	1.9301	1.503	1.29	2.20
3003	*CA8*	3.9644	1.5507	1.0991	-2.0851	2.20
3004	*CSRP2BP*	4.1539	1.5204	0.93858	0.49511	2.20
3005	*GAPDHS*	4.3547	3.0823	-0.8248	-1.0691	2.20
3006	*C1QC*	4.1894	1.7235	0.69816	-0.14957	2.20
3007	*AZIN2*	4.2286	2.1303	0.25152	-0.62153	2.20
3008	*ARHGAP35*	2.927	2.3492	1.3338	-1.6176	2.20
3009	*CALD1*	4.1148	3.1363	-0.6421	-1.0046	2.20
3010	*C12orf29*	3.7694	1.9915	0.84804	-2.5878	2.20
3011	*OLFML2B*	2.8516	2.4626	1.2929	1.1299	2.20
3012	*GINS1*	4.2654	3.3218	-0.98032	-1.377	2.20
3013	*OAT*	3.7451	3.4391	-0.57927	-2.1404	2.20
3014	*EZH2*	4.587	2.4271	-0.40976	-1.7591	2.20
3015	*HAGHL*	4.6692	2.0741	-0.13928	-1.1055	2.20
3016	*CAPN15*	4.3446	1.6959	0.56328	-0.30971	2.20
3017	*LOC100132146*	4.0602	3.4701	-0.92764	-1.0868	2.20
3018	*TSSC4*	5.0991	1.7714	-0.26832	-1.5848	2.20
3019	*LRPPRC*	3.7626	2.595	0.24438	-0.36536	2.20
3020	*SIRPD*	3.1687	2.9591	0.47336	-1.9622	2.20
3021	*SPRR2E*	3.7479	2.4305	0.4223	-2.6343	2.20
3022	*ERP29*	3.6863	3.0999	-0.18667	-1.8348	2.20
3023	*KCNIP4*	4.6374	2.3666	-0.40467	-1.2633	2.20
3024	*PTPRO*	2.9977	2.9415	0.65812	-0.04332	2.20
3025	*MCAM*	3.6799	3.3842	-0.46705	-0.60863	2.20
3026	*NYAP2*	5.3687	0.75048	0.47728	0.17607	2.20
3027	*ATRX*	4.5239	3.2183	-1.146	-1.8073	2.20
3028	*H2BFWT*	4.4824	2.1438	-0.03089	-0.11566	2.20
3029	*USP34*	3.4221	3.2198	-0.04736	-1.3574	2.20

3030	*CLEC2B*	5.0557	2.7959	-1.2574	-1.8649	2.20
3031	*DUSP28*	3.287	3.0034	0.30301	-0.02618	2.20
3032	*CACNA1H*	2.5908	2.5584	1.4436	-1.1575	2.20
3033	*SLC4A1AP*	5.2417	0.69496	0.65491	-0.69683	2.20
3034	*RPS3A*	4.5128	2.2397	-0.16128	-0.794	2.20
3035	*NETO1*	4.6972	1.9015	-0.00824	-0.85222	2.20
3036	*CYTH2*	3.66	2.3148	0.61561	-1.6413	2.20
3037	*NIN*	3.5086	1.8126	1.2691	-0.15079	2.20
3038	*MINPP1*	4.9864	1.3227	0.2809	-1.3197	2.20
3039	*GOLGA2*	3.8227	3.76	-0.99418	-2.2117	2.20
3040	*LOC730183*	5.2615	1.8899	-0.5639	-2.3623	2.20
3041	*LARGE*	4.6811	1.9079	-0.00197	-0.59838	2.20
3042	*UNC13B*	2.46	2.3391	1.7871	1.4941	2.20
3043	*ANO2*	3.4435	2.0112	1.1303	-1.2276	2.20
3044	*OPN1MW2*	2.5919	2.2205	1.7722	-1.584	2.19
3045	*S100B*	2.7231	1.9348	1.9256	0.033516	2.19
3046	*ZNF718*	4.1292	1.7488	0.70468	-0.54307	2.19
3047	*ARSG*	3.5492	3.4168	-0.38378	-1.6444	2.19
3048	*TUB*	3.2004	2.2744	1.1073	-1.2753	2.19
3049	*SLC31A2*	3.453	2.6378	0.48995	-0.36861	2.19
3050	*ZNF423*	3.4545	1.9065	1.2193	0.25863	2.19
3051	*SMARCA2*	3.0534	2.7182	0.80786	-2.1541	2.19
3052	*JMY*	2.8769	2.6477	1.0544	0.37483	2.19
3053	*TACC1*	4.9867	1.3594	0.23176	-0.05035	2.19
3054	*GLP2R*	4.3652	1.9028	0.30977	0.031433	2.19
3055	*RABL2B*	3.6784	2.4452	0.45396	-0.19566	2.19
3056	*ZAK*	3.8415	1.9937	0.74157	-1.2051	2.19
3057	*PHB2*	3.0367	2.817	0.72192	-2.2433	2.19
3058	*LARP1B*	3.5174	3.2189	-0.16195	-1.8219	2.19
3059	*DNAJB8*	3.4665	3.1378	-0.0304	-0.23196	2.19
3060	*MCM9*	3.6992	3.6313	-0.75762	-2.2948	2.19
3061	*PUS1*	3.5531	2.1334	0.8845	-0.50775	2.19
3062	*ZC3HAV1*	3.4928	1.7699	1.3068	0.44713	2.19
3063	*KRTAP19-6*	5.3939	1.2121	-0.03724	-1.5212	2.19
3064	*OR7D2*	4.8355	3.0229	-1.2903	-1.7804	2.19
3065	*MYOM2*	3.4633	3.4373	-0.33266	-1.9172	2.19
3066	*C18orf65*	3.6994	3.0145	-0.14627	-0.18896	2.19
3067	*LHX1*	3.4532	2.7736	0.33886	-0.44353	2.19
3068	*BRD1*	3.3901	3.0077	0.16772	-0.41502	2.19
3069	*IPPK*	3.7399	2.1824	0.64312	-0.41567	2.19
3070	*HELT*	3.9654	1.3946	1.2044	-0.61027	2.19
3071	*CD84*	3.9193	1.5496	1.0945	-0.20594	2.19
3072	*SYNPO*	2.8966	2.1051	1.561	0.60261	2.19
3073	*KIAA1407*	4.3981	1.3932	0.77129	-0.92025	2.19
3074	*CDKN1C*	3.3262	3.029	0.20665	-1.9166	2.19
3075	*TBC1D25*	3.1815	2.9642	0.41564	-1.6213	2.19

3076	*SLC39A12*	4.252	2.2938	0.01504	-1.4682	2.19
3077	*RAB1B*	3.3388	2.7554	0.46469	-1.0148	2.19
3078	*BMP5*	3.696	3.1646	-0.30258	-0.34227	2.19
3079	*DUS2*	3.9074	1.4764	1.1739	-1.3523	2.19
3080	*TMEM132E*	3.8945	2.1709	0.49221	-1.6346	2.19
3081	*C10orf62*	3.2522	2.6021	0.70287	-1.0544	2.19
3082	*ALDOA*	2.8432	2.2983	1.415	-0.64037	2.19
3083	*CCDC91*	3.1194	1.7716	1.6629	0.43843	2.18
3084	*VSIG4*	3.8252	1.5191	1.2083	-0.7853	2.18
3085	*CDC42BPG*	4.2559	1.7606	0.53427	-0.41703	2.18
3086	*TUBB2B*	3.1471	3.0327	0.36899	-1.6824	2.18
3087	*ZNF605*	5.3772	0.88613	0.28491	-0.42899	2.18
3088	*TPP1*	3.1314	2.5608	0.85534	0.26891	2.18
3089	*NUTF2*	4.8968	1.47	0.18033	-1.035	2.18
3090	*PDGFRA*	4.1805	2.6471	-0.28384	-2.7139	2.18
3091	*MEMO1*	4.7972	1.8928	-0.14649	-0.25056	2.18
3092	*KRIT1*	5.0059	1.7619	-0.22611	-1.2008	2.18
3093	*SIDT1*	5.3747	1.6037	-0.43674	-0.88584	2.18
3094	*ZFAND2A*	4.3508	2.3483	-0.15963	-1.6804	2.18
3095	*TCL1B*	2.807	2.481	1.2501	-0.45544	2.18
3096	*PPAP2C*	3.7719	3.5931	-0.83101	-1.416	2.18
3097	*KL*	4.5782	2.5582	-0.60267	-0.85002	2.18
3098	*LUM*	4.2915	2.1805	0.06165	-1.7466	2.18
3099	*RNF144A*	4.8473	0.8516	0.8346	0.62248	2.18
3100	*ARL5C*	3.3639	2.6745	0.4944	0.084092	2.18
3101	*PCDHGA11*	4.7123	1.8002	0.01988	-0.98194	2.18
3102	*UBR2*	3.2118	2.5574	0.76069	0.6088	2.18
3103	*TUBB3*	3.8079	1.8157	0.90472	-0.09645	2.18
3104	*PCDHGB3*	3.8793	2.0695	0.57936	0.07696	2.18
3105	*PRTFDC1*	2.5672	2.284	1.6768	0.71705	2.18
3106	*ODAM*	3.3303	2.1699	1.0277	-0.68388	2.18
3107	*LCA5*	4.9442	1.2066	0.37603	-0.53481	2.18
3108	*IRF4*	3.2574	3.2207	0.04858	-1.5304	2.18
3109	*GSTA2*	3.5992	3.1335	-0.20606	-1.2993	2.18
3110	*SMIM1*	4.5687	1.4614	0.49584	-0.34014	2.18
3111	*DOK6*	4.0833	1.3583	1.0841	-0.31633	2.18
3112	*CHCHD7*	4.6266	2.4778	-0.5794	-0.71561	2.18
3113	*C15orf26*	4.9638	0.86799	0.69116	-1.6776	2.17
3114	*POLR2J2*	4.3351	1.3736	0.81187	-1.8532	2.17
3115	*WT1*	3.1163	3.0487	0.35396	-0.96407	2.17
3116	*NKX2-3*	3.1491	2.9301	0.43916	-0.8828	2.17
3117	*TRIM9*	3.4132	3.0581	0.04597	-0.3064	2.17
3118	*KRTAP6-2*	3.1949	1.7207	1.6009	0.15863	2.17
3119	*MNX1*	3.9389	2.4082	0.16852	-1.0288	2.17
3120	*IFI27L2*	4.8358	1.1557	0.52293	-0.55117	2.17
3121	*TEX36*	3.6162	3.0753	-0.17772	-0.26798	2.17

3122	*FAM71F2*	3.4644	2.3263	0.72294	-0.39004	2.17
3123	*UNG*	3.8423	3.0847	-0.41738	-1.1845	2.17
3124	*ZNF789*	4.4057	1.6659	0.43769	-1.3617	2.17
3125	*BSCL2*	4.3096	3.7886	-1.589	-2.0613	2.17
3126	*UGT2B4*	2.5832	2.3542	1.5718	0.97673	2.17
3127	*CCR5*	4.7985	2.9453	-1.2371	-3.038	2.17
3128	*SPRR2F*	4.6285	0.94463	0.93279	-0.19661	2.17
3129	*RABEPK*	5.2937	2.3789	-1.1667	-1.2457	2.17
3130	*BCL2A1*	3.3999	2.0307	1.0744	0.98887	2.17
3131	*ATP6V0A2*	4.5457	2.6311	-0.6728	-1.28	2.17
3132	*FAM81B*	4.5036	1.8466	0.15352	-1.0596	2.17
3133	*PRPSAP1*	4.1203	3.0167	-0.63399	-2.0952	2.17
3134	*DAZAP2*	4.3551	2.4888	-0.34118	-0.4055	2.17
3135	*ZIC4*	5.3999	2.424	-1.3218	-1.8318	2.17
3136	*ZNF641*	4.4716	1.5101	0.51921	-0.81524	2.17
3137	*FKBP8*	3.953	1.6314	0.91428	-0.57797	2.17
3138	*LOC554223*	4.0919	1.3563	1.0503	-1.232	2.17
3139	*SLC16A6*	4.3887	2.8282	-0.71945	-0.87211	2.17
3140	*HDX*	5.0143	0.88265	0.5997	0.14429	2.17
3141	*OR5B3*	4.456	2.8151	-0.77562	-1.0435	2.17
3142	*TRABD2A*	4.8948	1.4015	0.19802	-0.14663	2.16
3143	*ZNF675*	3.4601	3.2048	-0.1715	-1.8076	2.16
3144	*GPR35*	3.2372	3.0601	0.19565	0.13811	2.16
3145	*UPRT*	6.1376	0.23591	0.11859	-0.86494	2.16
3146	*TIGAR*	5.2501	0.84514	0.39643	-0.78229	2.16
3147	*OR8S1*	4.6466	2.8367	-0.99175	-1.127	2.16
3148	*C15orf27*	4.6622	1.1004	0.72814	-0.64096	2.16
3149	*HSPB2*	4.914	1.913	-0.33821	-0.95321	2.16
3150	*CHD1L*	4.3223	1.1225	1.0436	-0.93592	2.16
3151	*PRDM5*	3.5033	2.7582	0.22661	-0.17526	2.16
3152	*RBMX2*	3.4731	1.5744	1.4406	-0.86482	2.16
3153	*ABCC6*	4.5836	0.98354	0.92007	-0.32806	2.16
3154	*PSPN*	4.1725	1.4138	0.90022	-0.3701	2.16
3155	*DPP3*	2.9704	2.706	0.80891	-0.81532	2.16
3156	*SPECC1L*	3.9732	1.6559	0.85583	-0.74014	2.16
3157	*CIRBP*	4.5626	1.2945	0.62766	-1.2993	2.16
3158	*JMJD6*	6.0008	2.3303	-1.8469	-3.9921	2.16
3159	*PIK3C2B*	4.1845	1.6097	0.68947	-0.48085	2.16
3160	*STYK1*	3.8446	2.0205	0.61791	-0.95708	2.16
3161	*ST8SIA2*	5.1146	1.629	-0.26314	-2.3557	2.16
3162	*CORO2B*	3.7373	2.9207	-0.17755	-0.64918	2.16
3163	*PRAMEF6*	4.5644	0.99389	0.92092	-0.99694	2.16
3164	*ANKRD37*	3.6166	3.5044	-0.64362	-1.0261	2.16
3165	*ABTB2*	2.689	2.3448	1.4429	0.016206	2.16
3166	*SPINT4*	3.9734	2.4005	0.10263	-1.4717	2.16
3167	*EGFL8*	4.7647	1.6412	0.06717	-0.00333	2.16

3168	*LIPJ*	2.9553	2.5397	0.97788	-1.3431	2.16
3169	*AKIRIN1*	5.5685	1.5414	-0.63746	-0.8531	2.16
3170	*TRMT112*	5.2463	2.1035	-0.87788	-1.5918	2.16
3171	*TAS1R2*	2.6968	2.5402	1.2343	-1.4683	2.16
3172	*SMPD4*	3.2679	3.0549	0.1483	-0.70544	2.16
3173	*ZCWPW2*	3.9442	3.7407	-1.2152	-1.3162	2.16
3174	*ZBTB6*	3.5915	1.6162	1.2616	-1.4284	2.16
3175	*C5orf46*	4.4872	1.0244	0.95752	0.66656	2.16
3176	*ZNF652*	3.9601	3.6932	-1.1843	-1.5633	2.16
3177	*OR5AK2*	3.6219	1.4236	1.423	-1.2505	2.16
3178	*BHLHB9*	3.6753	3.4457	-0.6548	-1.882	2.16
3179	*WDR27*	5.1127	0.89951	0.4533	-2.2297	2.16
3180	*NKX2-6*	4.5175	1.6707	0.27668	0.16167	2.15
3181	*USP35*	3.6217	2.5024	0.34067	-0.92764	2.15
3182	*PARP2*	4.2844	1.3328	0.84491	-0.54775	2.15
3183	*CUBN*	4.1546	3.1496	-0.84261	-1.529	2.15
3184	*CHRNA5*	4.2312	1.2343	0.99582	-1.2968	2.15
3185	*NT5DC2*	3.157	2.7169	0.58702	0.53982	2.15
3186	*ACPP*	4.4782	1.8055	0.17647	-1.0201	2.15
3187	*CPSF4*	2.9575	2.108	1.3934	-1.683	2.15
3188	*IFNAR2*	2.6473	2.444	1.3668	0.56522	2.15
3189	*CLASP1*	3.528	3.291	-0.36102	-0.45544	2.15
3190	*NAT1*	3.3659	2.8402	0.24958	-0.13837	2.15
3191	*EIF4A1*	4.7179	2.4233	-0.68606	-0.8792	2.15
3192	*KCNE1*	3.8307	1.4718	1.1521	0.81091	2.15
3193	*ANTXR2*	4.4768	2.009	-0.03316	-1.4504	2.15
3194	*CD74*	4.536	1.1218	0.79469	-0.62774	2.15
3195	*KIAA0753*	3.4778	2.5145	0.45699	-0.5287	2.15
3196	*SCMH1*	3.3593	2.1101	0.97985	-0.08097	2.15
3197	*YAP1*	3.1042	2.6755	0.66955	-0.43113	2.15
3198	*ADGRG2*	3.698	1.9818	0.76887	-1.1801	2.15
3199	*APOBEC4*	3.997	1.6355	0.81574	-0.16781	2.15
3200	*SCML4*	3.5576	1.9972	0.89291	0.49265	2.15
3201	*ITFG1*	4.0746	1.5941	0.77753	-0.91165	2.15
3202	*LDHC*	3.4474	2.0012	0.99575	-1.5965	2.15
3203	*OR6M1*	3.7632	1.6569	1.024	0.98239	2.15
3204	*PRICKLE2*	3.4501	2.6685	0.32481	-0.10374	2.15
3205	*AIMP1*	3.5879	2.6337	0.22171	-3.475	2.15
3206	*PRR15L*	3.9317	3.2249	-0.71443	-0.75537	2.15
3207	*UBR7*	3.3274	2.7233	0.39018	-0.55154	2.15
3208	*SOAT1*	3.7178	2.9956	-0.27263	-1.2754	2.15
3209	*OR6A2*	5.1456	1.3667	-0.07199	-0.73814	2.15
3210	*DIO1*	3.0733	2.1572	1.2098	-0.75099	2.15
3211	*BCAT2*	2.846	2.0102	1.5839	-0.18402	2.15
3212	*KRTAP11-1*	5.6701	0.81566	-0.04984	-1.4747	2.15
3213	*SHFM1*	3.8685	2.432	0.13535	-0.54093	2.15

3214	*LNP1*	3.8567	3.3864	-0.80774	-1.9619	2.15
3215	*SDC4*	3.3002	1.6432	1.4907	-3.3966	2.14
3216	*SPP1*	4.1177	2.4484	-0.13307	-0.97093	2.14
3217	*POLR2K*	3.3069	2.4996	0.62632	0.47537	2.14
3218	*OR2H2*	5.1961	0.69953	0.53636	0.34151	2.14
3219	*SEC31A*	3.5857	2.6648	0.17903	-0.20512	2.14
3220	*TMEM144*	3.6459	2.767	0.0154	-0.8828	2.14
3221	*SLC25A46*	3.0098	2.0054	1.412	-1.338	2.14
3222	*DRGX*	3.3921	2.7647	0.26896	-0.83434	2.14
3223	*SPATA31A6*	3.7708	2.8723	-0.21974	-0.49414	2.14
3224	*HSF1*	4.062	2.8219	-0.46076	-0.77141	2.14
3225	*TMEM161B*	3.246	2.6839	0.49311	-1.8921	2.14
3226	*TYRP1*	3.5021	1.5773	1.3417	-0.8756	2.14
3227	*TSHR*	3.1517	2.0962	1.1711	0.061648	2.14
3228	*LXN*	3.4736	3.165	-0.22006	-0.72882	2.14
3229	*PDLIM2*	3.021	2.0458	1.3512	0.97236	2.14
3230	*BIRC3*	3.873	3.1418	-0.59683	-1.3132	2.14
3231	*AMBRA1*	2.6308	2.6097	1.1758	1.1177	2.14
3232	*LRAT*	3.1483	2.686	0.58147	0.12633	2.14
3233	*SF3B6*	3.7636	2.1292	0.51986	-1.5894	2.14
3234	*OOSP2*	4.3543	2.2343	-0.17614	-1.3835	2.14
3235	*ANKRD12*	3.6014	1.4904	1.3199	-0.59223	2.14
3236	*MEST*	2.7585	2.562	1.0909	0.14017	2.14
3237	*SRFBP1*	3.3712	2.4115	0.62851	-0.68153	2.14
3238	*SIL1*	3.0361	3.0081	0.36594	-0.79663	2.14
3239	*RNF112*	4.5484	1.2076	0.65341	-0.29186	2.14
3240	*MFN1*	3.4057	2.763	0.23966	-0.08059	2.14
3241	*SLC9A3R2*	3.8363	1.4703	1.0997	-1.3675	2.14
3242	*MVD*	3.9995	1.5273	0.87907	0.62977	2.14
3243	*C1QTNF1*	4.6557	3.0226	-1.2728	-1.8557	2.14
3244	*PIAS2*	3.9028	2.281	0.22167	-1.5857	2.14
3245	*HFE*	3.5538	2.4042	0.44746	-0.00436	2.14
3246	*OR5AC2*	4.5313	2.5839	-0.70988	-1.368	2.14
3247	*ABCA3*	2.8977	2.5357	0.96946	-1.292	2.13
3248	*ZAN*	4.1038	2.1098	0.18841	-0.49857	2.13
3249	*C1QTNF8*	3.3483	1.7239	1.3294	-1.5235	2.13
3250	*PLEKHM3*	4.9192	0.89296	0.58859	0.013933	2.13
3251	*IVD*	3.5274	2.25	0.62293	-0.02963	2.13
3252	*KRBA1*	3.4994	2.5893	0.31146	0.086364	2.13
3253	*KLRC2*	3.7034	2.1622	0.53439	-0.2677	2.13
3254	*SLC4A11*	2.6231	2.3437	1.4319	0.87952	2.13
3255	*NDUFA6*	4.1191	2.3251	-0.04728	-1.1156	2.13
3256	*BORA*	4.5931	1.3468	0.45681	-1.4572	2.13
3257	*OAS2*	5.0237	1.9294	-0.55678	-0.87728	2.13
3258	*ALKBH8*	5.5152	0.52416	0.3563	-2.0778	2.13
3259	*ZNF692*	3.923	1.8036	0.66806	-1.0564	2.13

3260	*FBLN5*	3.0948	2.9963	0.3027	-0.49631	2.13
3261	*OR4F17*	3.3511	1.8359	1.2064	0.62	2.13
3262	*PLXNB1*	4.0514	1.5858	0.75517	-1.525	2.13
3263	*CYP4A11*	3.1823	1.7239	1.4852	0.82277	2.13
3264	*SLC25A23*	3.1178	1.6909	1.5825	-0.12852	2.13
3265	*COG1*	3.1943	1.8491	1.3477	-0.2032	2.13
3266	*MS4A8*	4.8716	1.2674	0.252	-2.4709	2.13
3267	*WNT1*	3.8535	2.5175	0.01976	-0.19897	2.13
3268	*FOXJ1*	4.1765	2.3473	-0.13314	-1.7745	2.13
3269	*ASB4*	4.0267	2.1185	0.24498	-0.53767	2.13
3270	*RHOXF1*	3.3298	3.0863	-0.02598	-1.1752	2.13
3271	*GNLY*	3.3718	2.2148	0.80277	-0.20375	2.13
3272	*ADGRF1*	3.3586	3.1639	-0.1358	-0.48801	2.13
3273	*MTUS2*	3.085	1.867	1.4331	0.015704	2.13
3274	*CD99L2*	3.4329	3.4291	-0.47704	-1.7538	2.13
3275	*DAPL1*	3.6759	2.2558	0.45325	0.017898	2.13
3276	*PIGL*	2.9541	2.4122	1.018	-2.2712	2.13
3277	*ADGRB3*	3.0081	2.4634	0.91238	-2.1616	2.13
3278	*PTPDC1*	4.7179	2.4383	-0.77308	-2.4018	2.13
3279	*SLC37A2*	4.903	2.0216	-0.54177	-2.374	2.13
3280	*BPHL*	4.6886	1.2315	0.46243	-0.63071	2.13
3281	*RIN1*	4.2787	2.3811	-0.27775	-1.5947	2.13
3282	*PTPN9*	3.2237	2.8341	0.32255	-1.6597	2.13
3283	*MERTK*	3.6313	2.596	0.15206	-1.1415	2.13
3284	*MAMLD1*	4.5103	1.635	0.23379	-0.93764	2.13
3285	*CEP126*	2.7827	2.6387	0.95685	-0.45663	2.13
3286	*SH3GLB1*	4.1544	2.2462	-0.02312	-0.48231	2.13
3287	*PEF1*	4.0558	1.2735	1.0477	-2.1472	2.13
3288	*OR52D1*	5.4475	0.69121	0.23778	-0.98504	2.13
3289	*ACSS3*	3.8123	1.6577	0.90583	-0.21998	2.13
3290	*TMEM19*	4.7015	2.3298	-0.65553	-1.4607	2.13
3291	*NDUFAF6*	3.3737	3.3246	-0.32344	-1.6523	2.12
3292	*PHC3*	4.3087	3.0127	-0.94747	-1.6306	2.12
3293	*PGA3*	3.6936	2.7544	-0.07422	-0.72349	2.12
3294	*TMEM201*	3.8615	3.4359	-0.92629	-1.551	2.12
3295	*TEX26*	3.946	2.1131	0.31179	0.21507	2.12
3296	*BTBD2*	3.7222	1.5294	1.1169	-1.5121	2.12
3297	*DIO3*	2.8114	2.0515	1.5055	-2.0346	2.12
3298	*EPM2AIP1*	5.0363	2.2413	-0.90922	-1.0884	2.12
3299	*MCCD1*	3.3759	3.2643	-0.27379	-1.4143	2.12
3300	*TMIGD3*	3.7742	3.0071	-0.41548	-2.1724	2.12
3301	*PBXIP1*	2.5637	1.9419	1.8601	1.5488	2.12
3302	*ZNF784*	3.0407	2.9612	0.36179	-0.58285	2.12
3303	*FCHSD2*	4.135	1.592	0.63628	-0.48232	2.12
3304	*FGF18*	3.8082	1.3199	1.2328	-1.03	2.12
3305	*KLK7*	4.2913	3.2441	-1.1756	-1.2249	2.12

3306	*RAI14*	2.523	2.0638	1.7724	-0.10176	2.12
3307	*ANGPTL5*	4.892	2.809	-1.3419	-1.7845	2.12
3308	*QRFP*	3.4681	2.6552	0.23533	-4.0089	2.12
3309	*RAI2*	3.2684	2.3349	0.75512	0.41415	2.12
3310	*KIAA0040*	3.8384	2.2831	0.23613	-0.50872	2.12
3311	*TMEM241*	5.3309	0.96727	0.05942	-0.72798	2.12
3312	*HOXB9*	4.0612	2.7856	-0.48982	-0.69678	2.12
3313	*SNURF*	4.4791	1.2456	0.63122	-0.36677	2.12
3314	*RMND5A*	3.8322	3.6883	-1.1676	-1.2277	2.12
3315	*KCNF1*	4.0873	2.9545	-0.68934	-1.3728	2.12
3316	*LPIN2*	4.1095	3.0197	-0.7771	-1.4572	2.12
3317	*HACL1*	2.8424	2.8146	0.69425	-0.1453	2.12
3318	*PMFBP1*	3.2844	1.9984	1.0665	0.51635	2.12
3319	*EMC10*	4.9802	1.0757	0.29321	-0.994	2.12
3320	*SERPINE3*	3.5234	2.1339	0.6915	-0.4873	2.12
3321	*MUC13*	4.3723	1.0242	0.94907	-0.90947	2.12
3322	*IFIT1*	3.4672	1.628	1.2503	-0.05937	2.12
3323	*CCDC171*	3.1832	1.6616	1.4982	-0.76728	2.11
3324	*ALAS2*	4.8681	1.1422	0.33252	0.25848	2.11
3325	*GALM*	3.8338	3.7013	-1.1927	-1.8102	2.11
3326	*PSTPIP2*	3.9571	1.7151	0.66965	-0.55699	2.11
3327	*MTHFD2*	3.1298	2.9257	0.28617	-1.2782	2.11
3328	*UBN2*	3.5326	3.3895	-0.58054	-1.2968	2.11
3329	*LPCAT1*	3.7407	1.5698	1.0299	-0.00825	2.11
3330	*NEO1*	2.943	2.9194	0.47738	-1.5225	2.11
3331	*RAG2*	3.7314	3.3819	-0.77368	-0.8198	2.11
3332	*HELQ*	4.0185	1.2002	1.1186	-1.2975	2.11
3333	*SS18*	3.945	3.2595	-0.86829	-1.3665	2.11
3334	*ANPEP*	4.8483	0.88802	0.59669	-1.3358	2.11
3335	*AMOT*	3.5702	3.288	-0.52574	-1.1585	2.11
3336	*CFHR3*	4.1616	1.9295	0.24009	-1.1712	2.11
3337	*USP30*	3.432	2.8208	0.07739	-0.0614	2.11
3338	*DNAJC1*	3.8284	1.6871	0.81405	-1.5039	2.11
3339	*RASAL1*	4.0332	1.8181	0.47818	0.006715	2.11
3340	*C15orf32*	3.4483	3.1822	-0.30166	-1.1546	2.11
3341	*TMEM210*	3.8944	2.3082	0.12545	-1.8107	2.11
3342	*APITD1*	4.7863	1.4966	0.04374	-0.12258	2.11
3343	*TAS2R19*	3.5117	1.7446	1.0688	-2.1956	2.11
3344	*CREB1*	3.8257	1.9123	0.58664	-0.05385	2.11
3345	*EPHX1*	3.9542	1.4296	0.94084	-3.051	2.11
3346	*ZNF611*	4.7852	0.9261	0.61325	-0.18187	2.11
3347	*WEE2*	4.5502	1.8035	-0.0292	-0.83133	2.11
3348	*CNST*	4.9801	1.2223	0.11808	-1.4081	2.11
3349	*IGFBP7*	3.7861	3.7069	-1.1729	-1.7599	2.11
3350	*DPYSL2*	3.2875	2.6307	0.39935	-0.86007	2.11
3351	*PDZD3*	3.4551	1.6264	1.2358	0.87907	2.11

3352	*NKX2-2*	3.3711	2.8357	0.1092	-2.0902	2.11
3353	*RAPGEF2*	4.0325	3.3156	-1.0333	-2.5865	2.10
3354	*KRTAP9-6*	4.2803	2.6677	-0.63405	-0.92484	2.10
3355	*MOSPD1*	3.3697	3.0589	-0.11567	-1.106	2.10
3356	*HSD3B2*	5.162	0.58355	0.56737	-1.761	2.10
3357	*MRGPRX4*	5.6206	0.92186	-0.2298	-0.27596	2.10
3358	*PLP2*	4.7471	1.5719	-0.00832	-0.26446	2.10
3359	*FRMD4A*	4.6708	1.2307	0.40897	-1.864	2.10
3360	*BRD2*	4.403	3.2966	-1.3892	-1.7247	2.10
3361	*SMR3A*	3.8709	2.2404	0.19908	-1.6805	2.10
3362	*NR4A2*	2.7188	2.3682	1.2224	0.27066	2.10
3363	*TMCC3*	4.9669	0.78415	0.5547	-1.3823	2.10
3364	*EPS8*	4.7341	1.2174	0.3523	-2.1496	2.10
3365	*IER2*	3.3383	2.317	0.64634	-0.28421	2.10
3366	*HIST1H2BN*	4.5911	2.1345	-0.42417	-1.3409	2.10
3367	*CHRNA10*	2.2457	2.2143	1.8414	-1.5956	2.10
3368	*MEX3A*	4.0114	1.4913	0.79764	-1.1283	2.10
3369	*AZU1*	4.0955	3.2139	-1.0095	-1.1297	2.10
3370	*ECEL1*	3.5922	3.379	-0.67153	-1.7407	2.10
3371	*PIK3C2G*	3.2249	2.0444	1.0298	0.38951	2.10
3372	*FAIM*	5.0117	0.77753	0.50438	-0.04968	2.10
3373	*GPR141*	3.9102	2.6318	-0.24856	-0.5597	2.10
3374	*NRAP*	4.5663	1.3611	0.3659	0.22103	2.10
3375	*BCL2L2-PABPN1*	4.1888	2.5038	-0.39955	-0.94777	2.10
3376	*ZSCAN16*	2.7255	2.3669	1.1984	-2.1444	2.10
3377	*LRRC7*	2.3347	2.2404	1.7131	-1.344	2.10
3378	*PDCD4*	3.5629	1.5932	1.1317	-1.688	2.10
3379	*HTR3A*	4.5652	1.6622	0.05958	-1.6664	2.10
3380	*USP9Y*	5.684	0.59098	0.00829	-0.95388	2.09
3381	*ERVV-1*	2.9547	2.7466	0.57972	-0.96083	2.09
3382	*CCDC154*	2.499	1.9566	1.8252	0.01825	2.09
3383	*ARMC5*	5.5451	1.2736	-0.53815	-2.0147	2.09
3384	*YIPF5*	2.9126	2.5125	0.85478	-1.1005	2.09
3385	*EIF2D*	3.8377	1.6881	0.75389	-1.1421	2.09
3386	*S100A14*	4.5783	1.3179	0.38184	-1.7935	2.09
3387	*TAS2R40*	4.3871	2.8315	-0.94113	-2.8823	2.09
3388	*OR8B12*	2.944	1.914	1.4186	0.70012	2.09
3389	*KCNU1*	4.1639	1.5061	0.60397	-0.22023	2.09
3390	*GSC2*	3.4318	3.1863	-0.34431	-2.3636	2.09
3391	*BST1*	4.0657	2.7434	-0.53584	-1.3697	2.09
3392	*INSC*	4.7023	1.0076	0.56191	-1.1472	2.09
3393	*SLC2A4*	4.064	3.6487	-1.4414	-2.2329	2.09
3394	*CRISPLD1*	2.763	2.0565	1.4512	-0.78026	2.09
3395	*DHRS4L1*	2.9968	1.6891	1.5842	0.075504	2.09
3396	*RYR2*	4.5953	1.2888	0.3849	-0.35111	2.09
3397	*KCNK1*	2.9947	2.5489	0.72355	0.43235	2.09

3398	*ASNA1*	4.4583	1.4848	0.32247	-1.0128	2.09
3399	*STRADA*	3.2591	3.0122	-0.00628	-0.10716	2.09
3400	*TLX3*	3.5704	2.1417	0.5526	-0.55274	2.09
3401	*SEMA4G*	5.0746	1.5284	-0.33881	-1.0977	2.09
3402	*MC1R*	4.125	2.0282	0.10939	-0.74744	2.09
3403	*ELF1*	3.9061	2.5581	-0.2038	-2.2877	2.09
3404	*PLAC1*	3.032	1.9038	1.3238	0.17688	2.09
3405	*KRTAP4-9*	5.1693	2.2714	-1.1815	-1.2985	2.09
3406	*USP21*	5.7636	1.6082	-1.1126	-1.8557	2.09
3407	*PRDM15*	3.5594	2.0302	0.66953	-2.3154	2.09
3408	*OSMR*	4.0406	1.1885	1.0299	-0.00196	2.09
3409	*CHML*	3.6548	1.7511	0.85308	-0.95964	2.09
3410	*TAS2R14*	4.4691	1.5276	0.26218	-1.0123	2.09
3411	*NXPE1*	3.0292	2.4202	0.80915	0.059745	2.09
3412	*GHR*	4.9747	0.89306	0.39076	-0.72348	2.09
3413	*ZFR2*	3.3322	2.5515	0.37426	-0.38163	2.09
3414	*DNAJC9*	4.3726	1.7694	0.11561	-1.011	2.09
3415	*DGKH*	4.0834	2.9577	-0.78444	-1.0197	2.09
3416	*C12orf71*	3.7079	1.6119	0.93573	-0.60976	2.09
3417	*CCDC62*	4.0848	2.3265	-0.15579	-0.84326	2.09
3418	*ZNF737*	2.6597	1.8815	1.713	-0.58289	2.08
3419	*FPR2*	2.911	2.6929	0.64923	-0.64982	2.08
3420	*NTRK3*	4.2697	2.0504	-0.06938	-1.6881	2.08
3421	*FGF13*	3.2726	1.9478	1.0301	0.29068	2.08
3422	*TTC29*	5.4204	1.118	-0.28819	-1.1303	2.08
3423	*LRRC48*	2.834	2.0703	1.3456	1.2321	2.08
3424	*GATA6*	2.7412	2.1232	1.3853	-0.36246	2.08
3425	*PLEK2*	4.0664	2.372	-0.19182	-0.49623	2.08
3426	*DPEP2*	2.5499	2.0112	1.6851	0.065751	2.08
3427	*INSL5*	3.7225	2.5313	-0.00829	-0.25893	2.08
3428	*TLR9*	3.6978	3.6758	-1.1282	-1.2092	2.08
3429	*EPB41L4B*	3.368	1.8895	0.98788	0.20921	2.08
3430	*OC400927-CSNK1*	4.1581	1.155	0.93183	-0.38901	2.08
3431	*PSMG1*	3.7273	2.4777	0.03984	-0.31584	2.08
3432	*MYO16*	3.3679	2.3788	0.49761	0.30616	2.08
3433	*GIGYF1*	4.1617	2.4075	-0.32586	-1.3078	2.08
3434	*FAM111B*	4.9965	1.3209	-0.07438	-1.7364	2.08
3435	*KLHL25*	3.7629	1.5439	0.93587	-1.1303	2.08
3436	*ALDH18A1*	4.4223	1.9872	-0.16693	-4.0255	2.08
3437	*MYB*	2.5794	2.1653	1.4967	1.1784	2.08
3438	*GJA8*	4.104	1.6023	0.53464	-1.2109	2.08
3439	*PCDHB9*	3.0421	2.4189	0.77921	-0.83684	2.08
3440	*CSNK2A2*	3.9319	2.2068	0.10146	-1.6128	2.08
3441	*LIMA1*	2.3616	2.1577	1.7208	-0.07158	2.08
3442	*TNFRSF1B*	3.0555	2.212	0.97198	-1.2863	2.08
3443	*IFIH1*	5.2288	0.90934	0.10106	-0.82307	2.08

3444	*C2orf42*	3.1323	2.6128	0.49398	-1.8251	2.08
3445	*NR1I2*	3.5043	2.0472	0.68698	-0.59793	2.08
3446	*HOXB3*	3.3112	2.248	0.67922	-0.5254	2.08
3447	*AES*	5.2377	3.0551	-2.0545	-2.1419	2.08
3448	*KBTBD3*	3.7116	3.0424	-0.51632	-1.2928	2.08
3449	*YES1*	3.2599	3.0364	-0.05867	-0.45847	2.08
3450	*ROBO2*	3.9244	1.2001	1.1131	-0.55134	2.08
3451	*PHKG1*	3.2216	1.9717	1.043	-0.24904	2.08
3452	*DEPDC7*	2.7051	2.5342	0.99642	-1.4518	2.08
3453	*C11orf71*	4.7862	2.1562	-0.70712	-1.5261	2.08
3454	*TRDMT1*	2.8621	1.8307	1.5409	0.21699	2.08
3455	*E2F8*	3.8388	1.4199	0.97471	-0.88094	2.08
3456	*PCDHA6*	3.8167	1.678	0.73869	-1.9794	2.08
3457	*ZNF649*	3.5514	3.4278	-0.74604	-1.1576	2.08
3458	*GIMAP1-GIMAP5*	3.8344	2.6655	-0.26713	-0.46372	2.08
3459	*KDF1*	4.0492	1.1676	1.0159	-1.7675	2.08
3460	*COLEC12*	3.5277	1.6943	1.01	-0.8606	2.08
3461	*RORB*	5.0305	1.6517	-0.45176	-0.59055	2.08
3462	*CYP21A2*	2.8748	1.8131	1.5424	0.055383	2.08
3463	*BRDT*	3.8961	2.3752	-0.04235	-1.2619	2.08
3464	*GPR3*	3.2708	1.6045	1.3522	-0.7067	2.08
3465	*GAL3ST2*	4.8797	2.4546	-1.1084	-1.4881	2.08
3466	*DNASE1L1*	3.0756	2.4358	0.71415	-2.7974	2.08
3467	*IQSEC3*	3.1579	2.3907	0.67647	-0.10851	2.08
3468	*SLC7A14*	4.8196	1.1506	0.25443	-0.52757	2.07
3469	*MS4A14*	2.9165	2.1672	1.1409	-0.21752	2.07
3470	*THAP10*	3.7063	1.414	1.1033	-1.8157	2.07
3471	*GML*	3.3098	2.8117	0.10195	-0.74011	2.07
3472	*ZNF570*	3.398	1.5332	1.292	-0.37942	2.07
3473	*CACNA2D2*	3.5175	2.769	-0.06539	-1.1492	2.07
3474	*MSI1*	3.7492	2.9688	-0.49803	-1.3023	2.07
3475	*CC2D2B*	3.7131	2.8641	-0.35735	-1.1241	2.07
3476	*DOCK4*	4.0365	3.0055	-0.82368	-1.4512	2.07
3477	*TATDN3*	3.8075	3.5475	-1.1382	-1.9041	2.07
3478	*NUDT8*	3.3448	2.2156	0.65491	-0.69886	2.07
3479	*CKAP2L*	5.0896	1.2304	-0.10483	-0.53815	2.07
3480	*TAL1*	3.5614	2.3968	0.25591	-0.66194	2.07
3481	*ITSN1*	3.9755	1.9877	0.25016	-0.44713	2.07
3482	*EPHA8*	4.1047	1.185	0.92356	0.005872	2.07
3483	*MYT1L*	3.3139	3.0634	-0.16631	-1.1821	2.07
3484	*TAF1L*	3.2944	3.1918	-0.27609	-0.55068	2.07
3485	*KIAA1324*	3.0551	2.0828	1.0722	-0.71371	2.07
3486	*CDH5*	3.7178	3.1291	-0.63757	-0.79821	2.07
3487	*PDCD5*	4.4763	2.0312	-0.29854	-0.43114	2.07
3488	*PPP1R12B*	3.4897	2.291	0.4282	-0.91269	2.07
3489	*E2F7*	3.1139	2.1858	0.90745	0.092283	2.07

3490	*TMEM132D*	5.1716	1.8559	-0.82044	-1.8341	2.07
3491	*DCAF6*	3.6252	1.887	0.6932	-1.5252	2.07
3492	*ACTR6*	5.3715	1.2922	-0.45867	-2.0778	2.07
3493	*OR2B3*	4.3568	2.9619	-1.1152	-1.1476	2.07
3494	*MSH2*	4.4756	1.4224	0.30523	-0.29054	2.07
3495	*LAT*	4.8984	1.2464	0.05782	-1.3382	2.07
3496	*ATP6AP1L*	3.2929	2.9558	-0.04793	-0.93683	2.07
3497	*CCL23*	3.2481	2.3528	0.59948	-1.9013	2.07
3498	*CYP1B1*	4.4939	1.1889	0.51575	-0.75711	2.07
3499	*GPR173*	4.76	2.805	-1.3665	-1.7065	2.07
3500	*TNP2*	4.6594	2.0945	-0.55586	-1.0742	2.07
3501	*MAGT1*	3.7355	2.8517	-0.39017	-1.9326	2.07
3502	*RPS4Y2*	3.7951	1.4488	0.95182	0.01812	2.07
3503	*BLK*	4.7362	1.1344	0.32485	-2.1187	2.07
3504	*PFKFB4*	4.3334	3.3307	-1.4688	-2.8922	2.07
3505	*CYB561*	5.201	0.53052	0.46329	-1.2167	2.06
3506	*FAM71D*	3.5926	2.5143	0.08783	-1.0359	2.06
3507	*CDCA4*	4.1044	2.396	-0.30667	-1.7577	2.06
3508	*CEBPB*	4.2985	1.6891	0.20469	-1.7188	2.06
3509	*CRTC2*	3.1291	2.8369	0.22608	-1.9284	2.06
3510	*PLN*	3.3363	1.9007	0.955	0.02195	2.06
3511	*LCN2*	3.1854	2.6082	0.39778	-0.93287	2.06
3512	*CMC2*	4.5735	1.385	0.23246	0.1072	2.06
3513	*MED22*	3.9318	3.5494	-1.2914	-2.3529	2.06
3514	*OR56A5*	4.3462	2.5467	-0.70463	-1.4299	2.06
3515	*PF4*	2.7326	2.1591	1.2962	0.32075	2.06
3516	*RAVER1*	3.9332	3.302	-1.0475	-1.2985	2.06
3517	*OBSL1*	4.7253	1.2977	0.16338	-0.32528	2.06
3518	*ESRP2*	5.6156	0.32699	0.24375	-1.7819	2.06
3519	*HS2ST1*	3.1291	2.1004	0.95659	-2.1572	2.06
3520	*ZNF300*	3.1872	1.5386	1.459	-0.68917	2.06
3521	*TMED4*	5.239	1.0565	-0.11263	-0.22044	2.06
3522	*TUBB4A*	5.5214	0.96637	-0.30608	-2.8917	2.06
3523	*CYTIP*	3.8912	2.6107	-0.32117	-1.344	2.06
3524	*WDR75*	3.9428	1.629	0.60858	-1.1852	2.06
3525	*N4BP3*	2.5486	2.5174	1.1143	0.50218	2.06
3526	*SEL1L*	3.6306	3.1252	-0.5765	-0.61694	2.06
3527	*DYNLRB2*	4.6581	0.85487	0.66502	-0.96962	2.06
3528	*FZR1*	3.4925	2.361	0.32441	-0.7912	2.06
3529	*ZNF781*	3.1751	2.4401	0.56187	-1.2893	2.06
3530	*AQP12A*	4.2445	3.4285	-1.4998	-2.581	2.06
3531	*C11orf65*	3.3169	2.3529	0.50326	0.18946	2.06
3532	*PIM1*	4.0391	1.3917	0.74161	-0.48816	2.06
3533	*IL11RA*	3.7458	1.764	0.66258	-0.7342	2.06
3534	*PTCD2*	3.6306	1.749	0.79262	0.2556	2.06
3535	*CYP4F12*	3.87	1.3293	0.97258	0.05938	2.06

3536	KRTAP9-9	3.8546	3.2468	-0.93011	-1.257	2.06
3537	MEGF9	4.0258	1.972	0.1734	-0.84158	2.06
3538	APOOL	2.658	2.089	1.424	1.1806	2.06
3539	PXDN	3.2348	1.6475	1.288	-0.75199	2.06
3540	TBPL2	4.6267	2.1153	-0.57224	-2.2385	2.06
3541	DMC1	4.4901	2.0251	-0.34597	-1.5352	2.06
3542	NMBR	5.5643	0.44994	0.15463	-0.60845	2.06
3543	IFNA10	4.7521	0.74577	0.66934	0.28367	2.06
3544	HIST1H2AB	2.2522	2.0741	1.8407	-1.2726	2.06
3545	GNG10	4.8094	2.6931	-1.3358	-1.3542	2.06
3546	SLFN5	3.9077	1.6543	0.60444	-0.95017	2.06
3547	PGRMC1	2.7739	2.2065	1.1859	-1.2676	2.06
3548	ITPKB	3.0566	2.6053	0.50271	0.38329	2.05
3549	PMS2	3.0283	1.9329	1.2026	-1.2995	2.05
3550	SLC44A4	3.7698	1.4171	0.97671	-0.32895	2.05
3551	PATE4	4.0342	1.297	0.82994	0.45306	2.05
3552	IRX6	3.816	2.5575	-0.21291	-2.914	2.05
3553	NTSR1	3.6926	2.4431	0.02489	-0.68761	2.05
3554	UBXN6	3.1548	2.4501	0.5517	-1.4036	2.05
3555	IFNL1	3.7424	1.8296	0.58187	0.042565	2.05
3556	UNC5A	3.3385	1.4832	1.3316	0.15802	2.05
3557	COL4A4	3.5381	1.9945	0.62056	-0.95105	2.05
3558	ATP13A4	3.9681	1.361	0.82336	-0.82524	2.05
3559	IRAK4	3.9515	1.5308	0.66955	-0.77667	2.05
3560	THNSL1	4.3633	1.3384	0.44981	-1.5212	2.05
3561	SLITRK6	2.8499	2.5361	0.76484	0.16938	2.05
3562	SCARA5	3.1517	2.3996	0.59946	-0.44705	2.05
3563	MFHAS1	3.7463	3.6191	-1.2159	-2.9834	2.05
3564	SCML2	3.9179	1.9952	0.23572	-0.78236	2.05
3565	SERPINA5	4.0968	2.8829	-0.832	-1.1945	2.05
3566	PGAM5	2.1939	2.0616	1.8921	-0.71363	2.05
3567	PDZD8	3.6035	1.8062	0.73745	0.32514	2.05
3568	CACHD1	4.035	3.1561	-1.046	-1.1815	2.05
3569	CHRNA1	3.0969	1.8979	1.1497	-1.4739	2.05
3570	NEK3	3.538	2.6544	-0.04817	-2.2264	2.05
3571	CNTN4	3.8976	2.957	-0.71093	-1.4415	2.05
3572	TET1	3.9058	1.6091	0.62764	-0.87934	2.05
3573	VWCE	3.0082	2.2988	0.83488	-0.15253	2.05
3574	SURF4	5.0767	0.62682	0.43717	-1.8698	2.05
3575	RAB5A	3.0103	1.9559	1.1744	0.26309	2.05
3576	H1F0	3.7019	1.7895	0.64843	-2.6072	2.05
3577	LOC100505841	2.8046	2.1124	1.222	-1.489	2.05
3578	MAGI3	3.2244	2.0009	0.91333	-0.87318	2.05
3579	MAGED2	3.2089	1.6102	1.3187	0.87741	2.05
3580	NAV3	3.4343	1.7179	0.98525	0.5639	2.05
3581	KHDRBS1	3.9344	3.2559	-1.0529	-1.5026	2.05

3582	*CIDEA*	3.2983	2.4413	0.39597	-0.57379	2.05
3583	*ASCL3*	4.5396	2.7242	-1.1316	-1.6288	2.04
3584	*ZNF804B*	3.2765	2.6779	0.17752	-1.5525	2.04
3585	*BMPR1B*	2.1937	2.056	1.8821	-1.696	2.04
3586	*FAM3D*	4.6055	2.1042	-0.57974	-1.0589	2.04
3587	*OR52B4*	3.4782	2.6201	0.03164	-1.0751	2.04
3588	*IL17RC*	3.1107	2.46	0.55896	-1.3279	2.04
3589	*GATM*	3.2163	1.9626	0.95025	0.72749	2.04
3590	*TMEM50B*	2.5803	2.4766	1.0706	-0.00112	2.04
3591	*LOC100287036*	3.3473	2.0895	0.68886	0.67225	2.04
3592	*PRSS21*	2.8985	1.9706	1.2563	-1.748	2.04
3593	*APOBR*	2.9733	2.8694	0.28258	-0.05413	2.04
3594	*TCP11X2*	3.0265	2.9004	0.19713	-1.3591	2.04
3595	*SLC4A3*	4.1445	2.3294	-0.35118	-0.89934	2.04
3596	*GCLM*	3.9873	1.6744	0.46095	0.066753	2.04
3597	*HNRNPAB*	5.005	0.61394	0.50358	-1.4749	2.04
3598	*TTF2*	4.3948	1.0994	0.62805	-1.5152	2.04
3599	*CYB561A3*	2.867	2.1033	1.151	0.17003	2.04
3600	*PENK*	3.0939	2.813	0.21168	-1.4524	2.04
3601	*OR56A1*	3.9651	3.1264	-0.97303	-1.5821	2.04
3602	*TIMM10B*	3.6671	2.7643	-0.31295	-0.93415	2.04
3603	*ZIC3*	2.0851	2.0511	1.9808	-1.9036	2.04
3604	*CTSO*	3.5327	1.5952	0.98905	-1.4511	2.04
3605	*MEF2D*	3.7726	2.5576	-0.2143	-0.89178	2.04
3606	*GPR1*	3.4019	2.1566	0.5573	-0.18502	2.04
3607	*PER3*	3.7571	1.4413	0.91721	-0.03251	2.04
3608	*MAX*	3.8623	1.9135	0.33948	-0.84542	2.04
3609	*EPHA5*	4.2759	1.2569	0.58191	0.45747	2.04
3610	*SLC25A20*	5.053	1.3991	-0.33758	-0.37185	2.04
3611	*KNDC1*	3.1742	1.9913	0.94896	-0.76106	2.04
3612	*RAB40C*	3.8805	2.7372	-0.50394	-0.54404	2.04
3613	*NFE2L1*	4.5794	0.882	0.65218	-0.00448	2.04
3614	*UCP1*	3.0472	2.4761	0.58901	-1.4323	2.04
3615	*SOX21*	2.7674	2.6077	0.73696	0.34848	2.04
3616	*TTC22*	4.263	1.5047	0.34426	0.30635	2.04
3617	*SLAMF7*	3.3368	3.2142	-0.43956	-0.65312	2.04
3618	*ACHE*	2.4641	2.3588	1.287	-2.5707	2.04
3619	*CISD2*	3.6906	1.6875	0.73029	0.69767	2.04
3620	*C17orf51*	3.4305	3.1563	-0.47843	-1.0708	2.04
3621	*MAPK8IP1*	5.08	0.88226	0.14567	0.03447	2.04
3622	*DENND1A*	4.7322	2.6213	-1.248	-1.5563	2.04
3623	*PITX3*	4.5425	2.1184	-0.55568	-1.3296	2.04
3624	*SLC9A1*	2.7205	2.1031	1.2812	-0.05504	2.03
3625	*ZBED4*	4.5715	1.2239	0.30896	-0.98129	2.03
3626	*CLCN7*	3.236	2.9559	-0.0877	-1.0134	2.03
3627	*CCDC96*	3.9173	2.2652	-0.07919	-1.7879	2.03

3628	*IDH2*	3.946	2.1726	-0.01696	-2.5206	2.03
3629	*RECQL5*	5.2554	1.494	-0.64918	-2.1351	2.03
3630	*SPTBN4*	4.5293	1.991	-0.42012	-0.71161	2.03
3631	*LMAN1L*	3.5522	2.6704	-0.12259	-1.9746	2.03
3632	*BZW2*	5.3509	0.5892	0.15909	-0.73462	2.03
3633	*IGSF10*	3.8633	1.6932	0.54196	-0.92629	2.03
3634	*ANKS4B*	2.2342	2.0273	1.8365	-1.0763	2.03
3635	*TAAR6*	4.1152	1.5092	0.47351	-0.07895	2.03
3636	*ANKRD23*	2.547	2.3708	1.1799	-0.55275	2.03
3637	*PNMAL1*	4.1403	1.7474	0.20971	-0.59647	2.03
3638	*MEF2A*	2.2811	1.9281	1.8882	0.34617	2.03
3639	*DCDC2B*	4.0309	3.5337	-1.4689	-2.0542	2.03
3640	*SPACA1*	5.1966	0.87217	0.02688	-0.64918	2.03
3641	*PPOX*	4.6478	0.76009	0.68763	-0.40534	2.03
3642	*PDGFA*	4.2461	1.4538	0.39528	-0.1958	2.03
3643	*CD300A*	3.7454	3.6929	-1.3439	-2.4244	2.03
3644	*FAM73B*	2.1699	2.0063	1.918	-1.4823	2.03
3645	*CRAMP1L*	3.4145	1.8459	0.83358	-0.38025	2.03
3646	*TBC1D17*	4.2818	1.6965	0.11544	-0.58743	2.03
3647	*PLEKHG7*	5.1098	0.94815	0.03503	-1.696	2.03
3648	*MZB1*	6.0936	1.5349	-1.536	-2.0242	2.03
3649	*LCN1*	4.2568	1.1246	0.71068	-0.28888	2.03
3650	*KCNK9*	3.0406	2.4372	0.61421	0.17727	2.03
3651	*GPR155*	3.0971	2.9449	0.04999	-1.0669	2.03
3652	*STAU2*	3.9987	3.3311	-1.2397	-1.9623	2.03
3653	*CCS*	4.1073	1.7911	0.19067	-0.08529	2.03
3654	*LRRC31*	3.6761	1.8751	0.53784	-1.1011	2.03
3655	*POMGNT1*	4.5211	0.92736	0.64045	-0.57042	2.03
3656	*GPM6A*	2.3127	2.2099	1.5661	-0.96161	2.03
3657	*XRCC2*	4.5491	0.95136	0.58812	-1.456	2.03
3658	*CLC*	3.578	2.7544	-0.24474	-0.26575	2.03
3659	*GRTP1*	2.963	2.1879	0.93608	-1.9028	2.03
3660	*SCN1B*	2.9558	2.8474	0.28345	-0.66914	2.03
3661	*CXXC1*	3.6987	2.7501	-0.36246	-0.55007	2.03
3662	*CNTFR*	3.3105	2.1426	0.63315	-0.37636	2.03
3663	*KIAA0226*	3.6354	1.5201	0.92988	0.14678	2.03
3664	*GAS2*	3.1206	2.8987	0.06583	-0.34915	2.03
3665	*SLC38A3*	3.5651	1.6757	0.84431	-0.01854	2.03
3666	*COL5A1*	3.0208	1.6145	1.4494	-0.39004	2.03
3667	*AVIL*	2.9585	2.9406	0.18552	-1.0921	2.03
3668	*GNAZ*	3.5769	2.7653	-0.25767	-0.44353	2.03
3669	*OXER1*	4.2472	1.7388	0.09689	-0.13743	2.03
3670	*HAVCR2*	3.5795	1.3546	1.1487	-1.4064	2.03
3671	*GIT1*	5.9519	0.35701	-0.22673	-0.53304	2.03
3672	*DCAF8L1*	4.3995	2.1047	-0.42378	-1.3851	2.03
3673	*DSP*	3.3232	1.6989	1.0581	0.46222	2.03

3674	*SEC11C*	3.9748	1.4375	0.66779	-1.5099	2.03
3675	*OSBPL2*	3.6219	1.7557	0.7014	-0.48415	2.03
3676	*DHX15*	3.3862	2.2457	0.44703	-2.3452	2.03
3677	*APOBEC3B*	4.4772	2.4558	-0.85686	-1.208	2.03
3678	*IL36G*	3.8481	3.0331	-0.80594	-1.7743	2.03
3679	*DIP2A*	3.2862	2.6059	0.18292	-2.2432	2.03
3680	*ETS2*	2.4762	2.0755	1.5209	-1.7158	2.02
3681	*UBL4A*	3.819	2.9061	-0.6536	-0.80144	2.02
3682	*KIAA2018*	2.5825	2.4081	1.0804	0.092069	2.02
3683	*ATP1B2*	4.0022	2.2204	-0.15313	-1.4709	2.02
3684	*MAPK8*	2.9296	2.2977	0.84165	-1.6014	2.02
3685	*EGLN3*	3.003	2.325	0.74036	-2.1217	2.02
3686	*EPHB4*	2.9017	2.2763	0.89035	-2.6289	2.02
3687	*MRAP*	5.1688	1.6354	-0.73708	-2.5795	2.02
3688	*PDX1*	4.3171	1.2364	0.51174	-0.61169	2.02
3689	*DSCAML1*	4.7778	1.6849	-0.39865	-0.62059	2.02
3690	*GOLGA6B*	4.5063	2.8438	-1.2864	-2.3222	2.02
3691	*AFF1*	4.4055	2.9246	-1.2676	-2.1674	2.02
3692	*TAF1C*	3.4932	3.0158	-0.44727	-0.83146	2.02
3693	*RANBP17*	4.8521	0.98029	0.2284	0.20413	2.02
3694	*EMILIN3*	3.3418	3.0836	-0.36528	-0.47473	2.02
3695	*MTFP1*	5.0152	1.9815	-0.93661	-1.1415	2.02
3696	*MGST3*	2.9277	2.6897	0.44245	-0.22854	2.02
3697	*C6orf1*	2.8986	2.7252	0.43571	-1.176	2.02
3698	*CROCC*	3.8728	1.8934	0.29307	-0.51002	2.02
3699	*FGF6*	4.1423	0.96932	0.94737	-1.9912	2.02
3700	*SPIN4*	3.4182	1.3489	1.2901	-1.2571	2.02
3701	*NATD1*	3.3894	1.7147	0.9487	-1.8484	2.02
3702	*SPANXN2*	2.9883	2.2402	0.82316	-0.26105	2.02
3703	*THRB*	2.8903	2.2522	0.90916	-1.1126	2.02
3704	*CISD1*	3.8209	2.7769	-0.5497	-2.2955	2.02
3705	*ZDHHC8*	2.7604	2.4789	0.80653	-0.35232	2.02
3706	*MCEE*	2.786	2.7055	0.55179	-0.2426	2.01
3707	*C10orf95*	4.8908	1.4404	-0.28877	-1.3205	2.01
3708	*SPPL2A*	2.3054	2.026	1.7107	-0.02943	2.01
3709	*C9orf72*	3.9069	1.7706	0.36397	-0.81152	2.01
3710	*LAMP5*	3.5916	1.7617	0.6875	-1.4414	2.01
3711	*CKMT1B*	2.8877	2.1615	0.98914	0.41321	2.01
3712	*GRIP2*	3.6394	1.8318	0.56702	-0.34006	2.01
3713	*S100P*	3.9478	1.411	0.67802	-2.5307	2.01
3714	*NUFIP1*	3.9481	1.7418	0.34398	0.093012	2.01
3715	*TEX30*	3.6382	1.5311	0.86403	-2.1873	2.01
3716	*NEFH*	4.1837	2.8404	-0.99217	-1.4402	2.01
3717	*AKAP13*	3.6036	1.3197	1.1081	-0.0083	2.01
3718	*MORN5*	3.9796	2.9714	-0.92078	-1.2884	2.01
3719	*THRAP3*	2.7761	2.3419	0.91158	-1.4317	2.01

3720	*TMEM68*	4.7251	1.704	-0.40121	-1.606	2.01
3721	*TMTC4*	6.0367	0.13513	-0.14427	-1.9833	2.01
3722	*CDCP1*	4.9587	1.0281	0.04071	-0.84291	2.01
3723	*JAKMIP2*	4.4585	1.4287	0.1385	-0.8737	2.01
3724	*CD63*	2.956	2.4083	0.66127	-1.4864	2.01
3725	*ADHFE1*	3.8673	1.8669	0.29119	-2.3251	2.01
3726	*BAIAP3*	4.0703	2.067	-0.11303	-0.44283	2.01
3727	*IFIT5*	4.65	1.5333	-0.15913	-1.962	2.01
3728	*GAB4*	2.5458	2.2738	1.2035	-0.67865	2.01
3729	*KCNMB4*	3.9074	1.2329	0.88146	0.26777	2.01
3730	*OR14I1*	3.416	2.7026	-0.09709	-0.52847	2.01
3731	*TLR8*	5.0406	1.3314	-0.35059	-0.79365	2.01
3732	*ANKLE1*	3.3752	1.9417	0.70399	-0.05	2.01
3733	*HSBP1L1*	5.0243	2.824	-1.8275	-1.9806	2.01
3734	*F11R*	3.7762	2.0081	0.23634	-0.34767	2.01
3735	*FOSL2*	5.2736	2.4646	-1.7179	-2.3017	2.01
3736	*PRR12*	3.904	1.3693	0.74587	-0.75388	2.01
3737	*ZFP69*	3.072	2.6409	0.30592	-0.08354	2.01
3738	*P2RY1*	3.1957	2.9798	-0.15766	-1.3141	2.01
3739	*FUT8*	4.4164	1.5488	0.05164	-2.0242	2.01
3740	*CTF1*	3.2391	2.7899	-0.01337	-1.0846	2.01
3741	*FBP2*	4.0648	1.901	0.04944	-0.18263	2.01
3742	*FAM178A*	4.2818	2.7553	-1.0221	-1.7065	2.01
3743	*GGN*	3.5549	3.3367	-0.87826	-0.90523	2.00
3744	*POU6F2*	4.1852	1.9562	-0.1283	-2.0851	2.00
3745	*SLC25A5*	4.5485	1.604	-0.14091	-0.96161	2.00
3746	*PTPN22*	3.7111	2.981	-0.68122	-1.1727	2.00
3747	*ITGA8*	3.1759	2.5369	0.29731	0.20167	2.00
3748	*ALKBH4*	3.4283	2.3252	0.25556	-0.20485	2.00
3749	*PPFIBP1*	3.5294	3.1577	-0.6799	-0.78229	2.00
3750	*FBLN7*	3.3241	1.3686	1.3144	-2.4735	2.00
3751	*IRGM*	5.0002	0.99449	0.01212	-0.96572	2.00
3752	*MRPS23*	3.339	2.6484	0.0192	-0.15216	2.00
3753	*OR51T1*	2.8497	2.5111	0.64558	-0.36876	2.00
3754	*ARMC9*	3.6681	2.6009	-0.26347	-0.82453	2.00
3755	*DNAJB13*	5.886	0.43602	-0.31674	-2.2951	2.00
3756	*SPRED1*	4.1199	1.3486	0.53613	0.29777	2.00
3757	*RSPH1*	2.8345	2.6074	0.56217	-1.7303	2.00
3758	*FHL1*	3.4486	1.731	0.82234	0.76074	2.00
3759	*C7orf43*	3.5433	2.7222	-0.26357	-0.30878	2.00
3760	*CBX7*	2.9083	2.365	0.72823	-0.4433	2.00
3761	*DRAXIN*	4.122	2.9006	-1.0211	-1.2163	2.00
3762	*SERPINA1*	4.4692	1.2586	0.27291	-0.73911	2.00
3763	*SLC18A1*	3.2547	1.3744	1.3715	-1.2195	2.00
3764	*RNF181*	3.742	2.5035	-0.24569	-1.5125	2.00
3765	*PPIF*	4.9954	0.70375	0.30021	-2.599	2.00

3766	*TNS4*	3.387	1.59	1.022	-0.69866	2.00
3767	*SLITRK1*	4.1716	1.5442	0.28261	0.071465	2.00
3768	*ZYG11B*	4.1726	1.013	0.81109	-1.3205	2.00
3769	*ABHD15*	3.4443	2.3908	0.15966	-1.3961	2.00
3770	*CSDE1*	4.1238	0.9548	0.9133	-1.2619	2.00
3771	*GIMAP4*	2.4763	1.8209	1.694	-0.41406	2.00
3772	*SOS2*	3.0734	2.2346	0.68273	-0.35676	2.00
3773	*HAT1*	3.0182	2.7271	0.24369	-1.1709	2.00
3774	*KRT39*	4.106	2.136	-0.25358	-2.425	2.00
3775	*KRTAP4-4*	3.9945	1.7395	0.25227	-2.4661	2.00
3776	*APP*	4.3335	0.96898	0.68365	-0.29906	2.00
3777	*EFCAB1*	2.9672	2.7552	0.26323	-0.44353	2.00
3778	*JAK1*	2.7509	2.5717	0.66275	-1.8816	2.00
3779	*BPIFC*	5.5848	0.91268	-0.5133	-1.2705	1.99
3780	*ZBED3*	3.8493	2.4391	-0.3052	-1.241	1.99
3781	*MTRNR2L9*	3.8563	2.7918	-0.66535	-2.2607	1.99
3782	*SLC22A25*	2.5532	2.4733	0.95617	-0.93474	1.99
3783	*PKN2*	3.7114	1.4256	0.84514	-1.6525	1.99
3784	*SPRR1A*	3.6624	2.1362	0.183	-1.0197	1.99
3785	*MED27*	4.7453	1.6355	-0.39967	-0.55409	1.99
3786	*OR2AK2*	5.4246	0.57698	-0.02101	-0.31411	1.99
3787	*RAB14*	3.9189	1.8608	0.20058	-0.63976	1.99
3788	*CLEC4E*	3.4066	1.5321	1.0412	-1.338	1.99
3789	*SGCD*	3.5929	1.827	0.5599	-2.7579	1.99
3790	*ALS2CR11*	4.0858	2.78	-0.88784	-1.3892	1.99
3791	*SAG*	3.3943	2.145	0.43614	0.020306	1.99
3792	*NPIPA7*	3.6496	3.0627	-0.73814	-1.1364	1.99
3793	*CST7*	3.5424	2.4338	-0.00464	-0.77748	1.99
3794	*PIP5K1C*	3.4609	1.3982	1.1119	-1.3226	1.99
3795	*SLC30A7*	2.741	2.2006	1.0289	-0.20877	1.99
3796	*TM9SF4*	3.6988	3.1149	-0.84337	-2.2473	1.99
3797	*ATF6B*	3.4875	2.555	-0.07238	-1.0821	1.99
3798	*ANKRD31*	4.7001	0.71584	0.55401	-0.03223	1.99
3799	*KIAA0586*	3.5724	1.3082	1.0877	-2.1947	1.99
3800	*C1orf234*	5.7698	0.87678	-0.67887	-1.258	1.99
3801	*BRICD5*	2.9489	2.2038	0.81428	-0.07796	1.99
3802	*AMH*	4.7666	0.8308	0.36935	0.035999	1.99
3803	*HNRNPCL2*	5.4062	0.30616	0.25411	-1.3637	1.99
3804	*FKTN*	3.2201	2.6946	0.0517	0.037191	1.99
3805	*VAV1*	3.4302	2.0351	0.50097	-1.146	1.99
3806	*TCEAL6*	3.0276	2.6411	0.29629	-1.3255	1.99
3807	*LTF*	2.2588	1.86	1.8458	-1.5593	1.99
3808	*APBB2*	5.3001	2.3004	-1.6362	-1.7585	1.99
3809	*C10orf105*	4.5997	1.1591	0.20528	-1.9766	1.99
3810	*BCL2L14*	4.6841	2.845	-1.5658	-1.7445	1.99
3811	*ZDHHC17*	4.7146	1.5492	-0.30086	-2.0218	1.99

3812	*SLC39A8*	4.1402	1.0577	0.76478	-0.74638	1.99
3813	*OTUD6A*	3.7474	1.1474	1.067	-1.0707	1.99
3814	*RPF1*	3.3175	2.2874	0.35584	-0.69542	1.99
3815	*ZNF620*	5.22	0.37868	0.36079	-1.5984	1.99
3816	*MICB*	3.755	3.224	-1.0223	-1.2676	1.99
3817	*CUZD1*	3.0879	2.5767	0.29117	-0.89866	1.99
3818	*GJB7*	5.4402	0.47464	0.04023	-0.64234	1.99
3819	*E4F1*	3.3633	2.6616	-0.06987	-1.0451	1.99
3820	*NUDT14*	4.1207	2.5977	-0.76361	-1.6799	1.98
3821	*CAMK2B*	3.0377	2.2758	0.64067	-0.44353	1.98
3822	*IL11*	5.1601	2.7993	-2.0059	-2.0337	1.98
3823	*UFSP1*	3.485	1.2649	1.2036	-1.6098	1.98
3824	*IFI44L*	4.1464	1.9516	-0.1449	-1.3291	1.98
3825	*MLC1*	4.9587	0.62931	0.36374	-0.36917	1.98
3826	*ATPAF1*	5.4424	2.1196	-1.6129	-2.1282	1.98
3827	*DHRS9*	3.3351	2.2836	0.32826	0.092952	1.98
3828	*PRR15*	3.0655	2.2419	0.63937	0.11317	1.98
3829	*CGB2*	3.6353	2.5238	-0.21242	-1.5752	1.98
3830	*KLF3*	3.0697	1.816	1.0609	-2.5057	1.98
3831	*LOC653602*	4.1617	2.5394	-0.75529	-1.6411	1.98
3832	*MSH3*	4.0367	1.4151	0.49307	-1.2845	1.98
3833	*RGL3*	3.1036	2.1173	0.72264	-0.67874	1.98
3834	*TACSTD2*	3.6933	1.6699	0.5799	-0.75711	1.98
3835	*TATDN1*	3.2772	2.6324	0.03342	-1.2577	1.98
3836	*APEX1*	4.0969	2.0576	-0.2122	-0.67352	1.98
3837	*SCLT1*	4.3612	0.80144	0.77904	0.18277	1.98
3838	*GALNT15*	3.965	1.5914	0.38494	-2.382	1.98
3839	*CDK5R2*	3.2071	2.2804	0.45215	-0.90772	1.98
3840	*ANAPC16*	3.297	1.7472	0.89532	-1.2728	1.98
3841	*TM4SF20*	5.1212	1.0095	-0.19422	-1.6218	1.98
3842	*DAOA*	2.5645	2.3452	1.0249	-1.0583	1.98
3843	*SLC22A3*	3.817	2.2072	-0.09036	-0.5853	1.98
3844	*SERINC5*	4.963	1.3492	-0.37888	-0.47472	1.98
3845	*SOX17*	5.2049	0.62795	0.09965	-0.90256	1.98
3846	*MAGEB18*	3.2934	2.0885	0.55029	-0.23353	1.98
3847	*BANK1*	2.665	1.9282	1.3388	-0.34001	1.98
3848	*BMX*	3.7181	1.2383	0.97528	0.46199	1.98
3849	*CHST1*	4.0659	1.2272	0.63732	-0.85928	1.98
3850	*PTGES2*	3.8257	1.9109	0.19246	-0.38471	1.98
3851	*USP36*	3.6812	1.8932	0.35351	-0.91944	1.98
3852	*PAK2*	2.3301	1.9729	1.6229	0.77174	1.98
3853	*B3GNT7*	3.1636	2.3855	0.37607	-0.14163	1.98
3854	*RFC3*	4.4362	0.91832	0.56958	-0.3866	1.97
3855	*KIFAP3*	4.7482	1.1249	0.0504	-0.01346	1.97
3856	*SLFN12*	2.9124	2.6004	0.40853	-1.2284	1.97
3857	*TRHDE*	2.7221	2.4865	0.71096	-0.154	1.97

3858	*NPR2*	3.8741	2.5697	-0.52426	-1.3767	1.97
3859	*SMOC2*	3.3253	3.1525	-0.55849	-0.71363	1.97
3860	*CDC26*	5.8599	0.07319	-0.01531	-1.4998	1.97
3861	*HMGN5*	5.8142	0.70752	-0.60619	-0.62213	1.97
3862	*FAM83B*	4.7272	0.85887	0.32887	-0.89258	1.97
3863	*GPX7*	3.6288	2.0472	0.23854	-0.47033	1.97
3864	*EDA2R*	4.6325	1.2052	0.07616	-0.12773	1.97
3865	*HAPLN3*	3.1149	2.9394	-0.14048	-0.86756	1.97
3866	*LMAN2*	5.1098	1.2438	-0.44134	-0.56195	1.97
3867	*CYSLTR2*	4.1788	1.2671	0.46539	0.36838	1.97
3868	*NPAS2*	5.189	0.75378	-0.03161	-2.2624	1.97
3869	*BOK*	3.8504	2.0364	0.02358	-1.5511	1.97
3870	*GSS*	4.8252	1.8201	-0.73511	-1.0529	1.97
3871	*TM6SF1*	2.9789	2.6715	0.25899	-2.0838	1.97
3872	*GJB4*	4.1126	3.4427	-1.6463	-2.3795	1.97
3873	*PITX2*	3.4752	1.5781	0.85497	-0.57261	1.97
3874	*MBD3L3*	3.5636	2.9159	-0.57134	-0.80607	1.97
3875	*SCAF4*	2.5868	1.7339	1.5871	0.17647	1.97
3876	*CEP164*	4.1532	1.3027	0.451	-1.0641	1.97
3877	*CDH17*	3.3959	2.1453	0.36466	-0.96751	1.97
3878	*KRTAP5-1*	4.4746	0.92033	0.51055	0.12402	1.97
3879	*ZBTB26*	2.7389	1.8619	1.3029	-0.10473	1.97
3880	*MGAT5B*	4.0628	1.2435	0.59723	-0.49481	1.97
3881	*CYLC1*	4.3807	2.6431	-1.1209	-1.6047	1.97
3882	*MAGEH1*	4.3688	1.8729	-0.34111	-1.0099	1.97
3883	*OR52E8*	3.3271	2.5122	0.06045	-1.7338	1.97
3884	*FPR3*	4.7015	2.7325	-1.535	-2.3488	1.97
3885	*C14orf79*	4.652	1.0107	0.23618	-1.2418	1.97
3886	*SAP30*	4.538	2.0504	-0.69146	-0.77141	1.97
3887	*ARHGEF6*	4.2992	2.1069	-0.51141	-1.0297	1.96
3888	*DCDC5*	3.9809	1.5561	0.35737	-0.62808	1.96
3889	*CNDP2*	4.1143	3.1146	-1.3361	-1.3808	1.96
3890	*SKAP1*	3.9618	1.5646	0.36569	0.20523	1.96
3891	*TMEM212*	4.3907	1.7392	-0.23883	-0.98208	1.96
3892	*ACOX2*	3.0712	2.685	0.13202	-1.2728	1.96
3893	*PLEKHA2*	4.8589	1.246	-0.21809	-1.4764	1.96
3894	*RHEBL1*	3.3756	1.9743	0.53686	-1.0712	1.96
3895	*CEP95*	3.0817	2.9617	-0.15681	-0.91757	1.96
3896	*CDKN2AIP*	4.6809	1.0052	0.2001	-0.7687	1.96
3897	*DNAJB4*	2.3633	2.0431	1.4791	1.1598	1.96
3898	*FIGLA*	3.7732	3.2477	-1.1364	-1.5101	1.96
3899	*OR8J1*	3.6978	1.9782	0.20845	-1.7547	1.96
3900	*HOXB8*	4.0782	1.6373	0.16888	-1.448	1.96
3901	*ARL9*	3.9542	2.4545	-0.52547	-1.1637	1.96
3902	*CARD11*	3.45	2.9608	-0.52858	-2.1599	1.96
3903	*MAPKAP1*	3.0834	1.8272	0.9713	-0.42356	1.96

3904	*KRT10*	3.4322	2.2489	0.19862	-1.4507	1.96
3905	*RAB1A*	2.8687	2.8226	0.18761	-0.64632	1.96
3906	*RNF170*	2.2053	2.1088	1.5646	1.4953	1.96
3907	*IFI6*	2.7979	1.9665	1.1142	1.0709	1.96
3908	*PPP1R13B*	4.2245	0.88539	0.76859	-1.4717	1.96
3909	*PAX6*	3.2253	1.9341	0.71905	-0.6602	1.96
3910	*TTLL2*	3.5606	2.8646	-0.54972	-1.1307	1.96
3911	*DEFB124*	2.8145	2.4395	0.62092	-0.89313	1.96
3912	*TEX40*	3.8926	2.5786	-0.59634	-1.5429	1.96
3913	*KIAA1551*	4.3722	1.4139	0.08858	-2.5153	1.96
3914	*RALYL*	4.9868	1.4641	-0.57704	-0.9673	1.96
3915	*TRMT10A*	3.017	1.9862	0.87059	-0.63009	1.96
3916	*APOA4*	4.0429	1.5211	0.30954	-1.6274	1.96
3917	*KAAG1*	2.6777	1.7452	1.45	-2.5519	1.96
3918	*ADPRM*	2.8313	2.2436	0.79706	-1.3053	1.96
3919	*NUDT3*	3.1307	2.1904	0.54834	-1.3705	1.96
3920	*FAM118A*	3.2054	2.346	0.31329	0.16099	1.95
3921	*NEDD4L*	2.7218	2.4876	0.65529	-1.8095	1.95
3922	*CAMK2A*	4.4131	1.2433	0.20767	0.10415	1.95
3923	*DEFA1B*	2.3613	2.3221	1.1795	-3.3058	1.95
3924	*DOPEY1*	2.8644	2.205	0.79305	-0.47423	1.95
3925	*STX2*	3.3595	3.1277	-0.62489	-1.5015	1.95
3926	*NAV2*	3.0984	2.7108	0.05194	-1.7619	1.95
3927	*TBC1D15*	3.8449	1.4462	0.56712	-0.25163	1.95
3928	*YTHDF1*	2.8576	1.8162	1.184	-1.5524	1.95
3929	*RNASE12*	4.6649	1.6949	-0.50227	-2.4491	1.95
3930	*DNAH8*	3.5779	2.482	-0.20242	-0.39817	1.95
3931	*REEP4*	4.5875	1.7059	-0.43694	-1.7188	1.95
3932	*ROPN1B*	2.916	2.7818	0.15863	-1.3701	1.95
3933	*GTF2IRD1*	3.4215	1.2951	1.1396	-0.64149	1.95
3934	*GIMAP1*	3.0508	2.7457	0.05866	-2.6322	1.95
3935	*TSPAN32*	3.8608	1.708	0.2859	-0.42588	1.95
3936	*NAP1L2*	3.1867	1.5967	1.0705	-0.00828	1.95
3937	*ABCC3*	4.4244	2.7174	-1.2884	-2.4015	1.95
3938	*PPT1*	3.1331	2.5802	0.1395	-1.2651	1.95
3939	*SDC1*	4.1375	0.91273	0.80203	0.31834	1.95
3940	*FREM1*	4.4628	0.75104	0.63841	-1.5467	1.95
3941	*WBSCR27*	3.6248	3.4342	-1.2075	-1.4373	1.95
3942	*RGS8*	4.3822	1.9109	-0.44236	-1.4819	1.95
3943	*PVRL4*	2.6124	1.7641	1.4738	-1.7184	1.95
3944	*NUCB2*	4.2954	0.88489	0.66961	-1.0695	1.95
3945	*CCDC39*	4.4506	1.8357	-0.43694	-1.2358	1.95
3946	*FOXD4L3*	4.2079	0.89321	0.74794	-2.5934	1.95
3947	*PIH1D3*	4.6622	0.78255	0.4024	-1.1016	1.95
3948	*KLC2*	3.435	2.2094	0.20213	-1.1823	1.95
3949	*DPEP3*	4.6758	1.1533	0.01701	-1.2486	1.95

3950	*EPHX4*	3.8663	2.7644	-0.78506	-1.0386	1.95
3951	*ZNF350*	3.5608	1.7106	0.57397	-2.0838	1.95
3952	*TEX33*	2.797	2.1324	0.91549	-0.44079	1.95
3953	*KRT84*	4.7565	0.80437	0.2836	-1.1964	1.95
3954	*TGIF2LY*	3.3676	2.0337	0.44307	-0.58131	1.95
3955	*C6orf48*	2.7669	2.4866	0.59052	-0.51689	1.95
3956	*SLC13A5*	2.7047	2.3301	0.80823	-0.16266	1.95
3957	*MECOM*	2.8331	2.0769	0.9328	-0.15226	1.95
3958	*GNAT2*	4.339	1.7645	-0.26125	-1.4509	1.95
3959	*ATAD1*	3.7726	2.8104	-0.74086	-1.683	1.95
3960	*RORA*	4.3735	1.185	0.28355	0.13558	1.95
3961	*PIGX*	4.186	1.9087	-0.25425	-1.1316	1.95
3962	*SOWAHA*	3.5738	1.3221	0.94376	0.28124	1.95
3963	*TMOD1*	4.8452	0.85727	0.1365	-1.707	1.95
3964	*ZNF491*	3.7138	1.8387	0.28566	-1.0922	1.95
3965	*ZNF264*	3.5821	1.3122	0.94338	0.38037	1.95
3966	*GSDMD*	4.3576	1.6099	-0.13025	-0.18636	1.95
3967	*KIAA0930*	3.5356	2.0304	0.26771	-1.2608	1.94
3968	*ZNF775*	4.938	1.143	-0.24794	-0.39078	1.94
3969	*ATP8B1*	2.5914	1.909	1.3314	0.76235	1.94
3970	*TP73*	2.9501	1.7081	1.1723	-1.6781	1.94
3971	*COL12A1*	3.468	2.921	-0.55929	-1.3583	1.94
3972	*NAPSA*	3.5053	1.8351	0.48897	-0.83753	1.94
3973	*DOK3*	2.1625	2.0659	1.6006	-1.0548	1.94
3974	*SLC41A2*	4.623	0.66956	0.53539	-0.29046	1.94
3975	*CCDC127*	4.5744	3.2564	-2.003	-2.4557	1.94
3976	*OSBPL10*	3.0452	1.9226	0.85974	0.44828	1.94
3977	*RBM5*	3.7836	2.3855	-0.34536	-0.3701	1.94
3978	*TLE4*	3.4339	2.5211	-0.1314	-0.64219	1.94
3979	*COL4A5*	3.9364	0.99705	0.88837	-1.2985	1.94
3980	*BTN1A1*	4.0929	2.2686	-0.53969	-0.97066	1.94
3981	*CRISP3*	4.4425	1.4633	-0.08437	-0.29929	1.94
3982	*TRIM74*	3.6862	1.3335	0.79888	0.32027	1.94
3983	*C1orf50*	3.5745	1.1841	1.0589	-1.9513	1.94
3984	*CTNNB1*	4.9038	1.3795	-0.46733	-1.8937	1.94
3985	*ADRBK2*	2.8826	2.5563	0.3765	-1.2328	1.94
3986	*LY86*	3.2984	2.1367	0.38004	-0.2613	1.94
3987	*LRFN3*	3.3458	1.7991	0.66953	-0.59008	1.94
3988	*CUEDC2*	4.2181	1.5409	0.05454	-1.1747	1.94
3989	*NPPB*	2.0268	1.982	1.8047	-1.7364	1.94
3990	*USP9X*	5.4345	0.70578	-0.32689	-0.60658	1.94
3991	*STRN4*	2.9458	1.4594	1.4075	-0.93726	1.94
3992	*KRTAP13-3*	3.332	1.5037	0.97699	0.1111	1.94
3993	*OPRK1*	3.5047	1.7833	0.52357	-0.0576	1.94
3994	*UGDH*	3.9282	0.97082	0.912	-0.00827	1.94
3995	*FBXO42*	4.2095	1.3661	0.23442	-0.87673	1.94

3996	*ID4*	3.427	2.7676	-0.38471	-2.3615	1.94
3997	*PDE6H*	4.3718	1.8409	-0.40348	-0.41643	1.94
3998	*SIAE*	5.5523	0.35443	-0.09922	-1.5608	1.94
3999	*B4GALT1*	2.8148	1.921	1.0709	-1.8348	1.94
4000	*TAS2R16*	3.9291	1.0899	0.7869	0.61626	1.94
4001	*DNASE2B*	2.7758	2.5818	0.44637	-0.31475	1.93
4002	*PRSS12*	3.705	1.1133	0.98511	-1.4089	1.93
4003	*HDAC1*	3.2924	1.7205	0.79047	0.7274	1.93
4004	*KIZ*	5.2272	0.52922	0.04637	-0.05538	1.93
4005	*TNFRSF11B*	4.5971	0.616	0.58934	-0.00831	1.93
4006	*SLC4A2*	3.5555	2.3324	-0.0862	-0.23857	1.93
4007	*TRAF6*	4.6589	1.3526	-0.20989	-1.748	1.93
4008	*PADI3*	2.7217	2.0895	0.98902	-2.716	1.93
4009	*FAM21C*	2.7456	1.7722	1.2819	0.11502	1.93
4010	*CCR8*	3.3042	1.8907	0.60448	-0.41064	1.93
4011	*TMBIM1*	4.2393	1.2102	0.34895	-0.99833	1.93
4012	*CT45A5*	3.5117	3.0901	-0.80394	-1.6799	1.93
4013	*OBP2B*	5.0286	1.0777	-0.30863	-1.951	1.93
4014	*DNAH11*	3.3408	2.6435	-0.18689	-1.8462	1.93
4015	*CTAG1A*	5.7469	0.1717	-0.12213	-0.88274	1.93
4016	*LCE3E*	2.36	2.2433	1.193	-1.4968	1.93
4017	*IVNS1ABP*	4.9633	0.61907	0.21366	-1.8311	1.93
4018	*SDC3*	2.6909	2.381	0.72413	-0.17305	1.93
4019	*HS1BP3*	2.8638	2.43	0.50214	-0.69564	1.93
4020	*S100A7L2*	3.6115	2.8842	-0.70106	-1.6663	1.93
4021	*PNOC*	4.8353	0.81815	0.13972	-0.95136	1.93
4022	*MSTN*	2.9891	2.4584	0.34489	-1.5959	1.93
4023	*ADAMTS18*	3.9114	1.137	0.74305	-1.0547	1.93
4024	*SGSH*	3.7976	2.1693	-0.1771	-0.9952	1.93
4025	*GRWD1*	5.6755	0.37511	-0.26125	-1.5082	1.93
4026	*SAC3D1*	4.1934	0.93868	0.65625	-0.45403	1.93
4027	*TCP1*	4.1917	1.578	0.01837	-0.18955	1.93
4028	*BSPRY*	3.3823	1.4435	0.96221	-0.70591	1.93
4029	*SERPINB7*	4.8065	0.78556	0.19546	-1.3686	1.93
4030	*METTL1*	3.4789	2.8925	-0.5842	-1.1821	1.93
4031	*MAOB*	2.802	2.0907	0.89401	-1.2331	1.93
4032	*PADI6*	4.0861	1.3147	0.38487	-1.5026	1.93
4033	*CSTB*	4.4756	0.68368	0.62632	-0.72479	1.93
4034	*SPX*	4.2347	2.9762	-1.4269	-1.9446	1.93
4035	*SLIT2*	3.314	1.5895	0.88024	-0.03957	1.93
4036	*FAM196B*	2.9987	2.0407	0.74419	-0.5911	1.93
4037	*CHAMP1*	2.4803	2.0204	1.2823	0.079048	1.93
4038	*SLC28A2*	3.7283	1.191	0.86333	0.32942	1.93
4039	*GLRX*	3.4158	1.9453	0.42124	-0.06464	1.93
4040	*TRPC4AP*	4.4629	1.6578	-0.33886	-1.9013	1.93
4041	*TANK*	4.0776	3.1638	-1.4596	-1.5672	1.93

4042	*RFK*	3.9863	2.8607	-1.0653	-2.0276	1.93
4043	*CRTAM*	4.3636	1.4209	-0.00369	-1.0809	1.93
4044	*PKD1L1*	5.0096	0.44563	0.32406	-0.07909	1.93
4045	*HERC1*	4.7911	2.2322	-1.2463	-1.5268	1.93
4046	*KLHDC9*	4.4292	0.68476	0.66132	-0.51304	1.93
4047	*SLC15A1*	4.1272	1.1308	0.51635	-2.5292	1.92
4048	*IGSF9*	2.7451	2.132	0.89644	0.080286	1.92
4049	*ZNF394*	4.0627	1.526	0.18069	0.1421	1.92
4050	*MALT1*	3.8778	1.1791	0.71191	-1.1747	1.92
4051	*MTMR8*	5.5039	0.16693	0.0973	-0.91317	1.92
4052	*TMEM214*	4.53	0.92279	0.31528	-1.28	1.92
4053	*SYNPO2L*	6.1852	0.79432	-1.212	-1.53	1.92
4054	*SLC28A3*	3.0598	2.1278	0.57918	-0.34241	1.92
4055	*CHODL*	3.2125	1.6482	0.90574	-1.2427	1.92
4056	*DCUN1D1*	4.6874	1.1022	-0.02341	-0.64083	1.92
4057	*TEX12*	5.4459	0.42422	-0.10528	-2.5198	1.92
4058	*WASF1*	3.3917	1.7637	0.60849	0.36853	1.92
4059	*CFAP70*	2.5327	1.8387	1.3923	-0.05504	1.92
4060	*MYBPH*	3.7317	1.1782	0.85163	-0.14151	1.92
4061	*STX19*	4.4392	2.2274	-0.90549	-1.6826	1.92
4062	*MDK*	3.7819	2.338	-0.35893	-1.0807	1.92
4063	*MTRNR2L8*	5.329	0.53752	-0.1057	-1.2186	1.92
4064	*DENND4C*	3.1076	2.399	0.25407	-1.3023	1.92
4065	*RNASE4*	5.6882	0.32159	-0.24942	-0.55904	1.92
4066	*OR7G2*	3.3165	1.4146	1.028	-0.65378	1.92
4067	*MAP4K4*	4.3466	1.1669	0.24437	-0.04287	1.92
4068	*DCTD*	4.5849	2.211	-1.0385	-1.6157	1.92
4069	*GPR89B*	3.1217	2.0125	0.62248	-0.94558	1.92
4070	*IL12A*	2.3287	1.7943	1.6331	-1.7466	1.92
4071	*ATF2*	5.1374	0.5432	0.07542	-1.2987	1.92
4072	*RAX*	2.93	1.6281	1.1965	0.50245	1.92
4073	*TCHH*	4.9738	1.0298	-0.25004	-0.31787	1.92
4074	*PLIN5*	3.7681	1.7211	0.26292	-1.3377	1.92
4075	*TMEM170B*	3.9231	1.9244	-0.09656	-0.38119	1.92
4076	*IP6K1*	3.4933	1.8725	0.38493	0.22057	1.92
4077	*FAM46B*	3.7175	1.439	0.59408	-1.3783	1.92
4078	*PNRC2*	4.6922	0.91493	0.14305	-0.96144	1.92
4079	*ELOVL6*	3.6065	1.2108	0.93279	0.43428	1.92
4080	*DDX43*	4.2805	1.0422	0.4261	-0.90161	1.92
4081	*FOXM1*	2.5227	1.7955	1.4302	-0.39722	1.92
4082	*MYL10*	2.8738	2.0444	0.82951	-0.69794	1.92
4083	*CAT*	3.5806	3.1888	-1.0224	-1.0639	1.92
4084	*TMEM33*	3.6758	2.3191	-0.24849	-0.8183	1.92
4085	*SETD5*	5.0573	0.37331	0.31479	-1.8842	1.92
4086	*ACOT7*	5.1593	0.67443	-0.08851	-1.0576	1.92
4087	*GBP3*	3.4338	2.129	0.18168	-0.46351	1.91

4088	*TRIM16L*	3.2101	2.0138	0.52046	-1.8314	1.91
4089	*ZNF707*	4.4108	1.125	0.20832	-1.2448	1.91
4090	*MGST2*	3.0594	2.6553	0.02784	-2.1984	1.91
4091	*CFAP52*	4.2626	2.5744	-1.0949	-1.3892	1.91
4092	*KLHL21*	2.5389	2.0435	1.1585	-1.9678	1.91
4093	*TAF4*	3.0029	2.3672	0.36965	0.078586	1.91
4094	*QSER1*	3.7738	2.6428	-0.67751	-1.4798	1.91
4095	*IL17RB*	2.7409	1.5615	1.4363	-1.8572	1.91
4096	*GIGYF2*	3.0548	1.4452	1.2383	-0.07172	1.91
4097	*THUMPD3*	2.5499	2.3669	0.82132	-1.9766	1.91
4098	*CCDC68*	3.4768	1.5826	0.67802	-1.5526	1.91
4099	*GPR137*	4.5285	0.66453	0.54271	0.24439	1.91
4100	*HLA-DOB*	2.9016	2.5767	0.25569	-1.9375	1.91
4101	*SLC47A1*	4.1575	2.7332	-1.1571	-1.8932	1.91
4102	*TSGA13*	4.7515	0.66965	0.31154	-1.7065	1.91
4103	*GOSR1*	5.242	1.6181	-1.1283	-1.8335	1.91
4104	*LAIR1*	3.2275	1.3341	1.1701	-1.7077	1.91
4105	*SUCO*	3.5913	3.4405	-1.3023	-1.6431	1.91
4106	*LOC149373*	2.3556	2.2168	1.1571	-0.81505	1.91
4107	*XPNPEP1*	5.319	0.4644	-0.0566	-0.77333	1.91
4108	*ADCK4*	2.6759	2.2441	0.80676	0.21455	1.91
4109	*SNX5*	3.2012	1.5384	0.98692	-1.6276	1.91
4110	*TM9SF2*	3.8841	2.8319	-0.99079	-2.4067	1.91
4111	*CD79A*	3.7621	1.2269	0.73578	0.47341	1.91
4112	*SH2B3*	5.2752	0.81574	-0.36654	-2.0283	1.91
4113	*RAPGEFL1*	3.4256	1.8541	0.44229	-1.4762	1.91
4114	*POSTN*	4.7899	0.70965	0.22191	-2.9354	1.91
4115	*ZNF829*	2.76	2.4456	0.51527	-0.44353	1.91
4116	*MS4A5*	3.7747	2.0018	-0.05637	-0.97595	1.91
4117	*CLINT1*	4.5197	0.60457	0.5935	-1.0091	1.91
4118	*SYT6*	5.0401	0.90557	-0.22803	-0.57018	1.91
4119	*HLA-C*	4.6061	1.1801	-0.06874	-0.87817	1.91
4120	*ANXA10*	3.6687	1.1091	0.93894	-0.97093	1.91
4121	*WDPCP*	3.7364	2.8877	-0.908	-1.1027	1.91
4122	*GPR148*	4.3779	1.5895	-0.25135	-1.6274	1.91
4123	*TCTEX1D1*	3.4506	1.9289	0.33545	0.016531	1.90
4124	*STKLD1*	3.7206	1.9481	0.045	-0.04792	1.90
4125	*ZNF439*	3.6867	2.9043	-0.87817	-1.773	1.90
4126	*ISCA1*	3.3808	1.9488	0.38204	-1.0391	1.90
4127	*FLVCR1*	3.0334	1.5389	1.1373	-2.0197	1.90
4128	*GGA2*	2.9235	2.8858	-0.09982	-1.3797	1.90
4129	*PADI4*	2.5366	2.0119	1.1605	-1.5736	1.90
4130	*DYNC1LI2*	3.9177	0.903	0.88827	0.28694	1.90
4131	*PFKFB1*	4.0519	1.105	0.55205	0.15749	1.90
4132	*ZDHHC13*	3.5187	2.8634	-0.67521	-0.84872	1.90
4133	*CSRNP1*	4.5637	1.2289	-0.08601	-0.51859	1.90

4134	*ZNF347*	3.575	3.1651	-1.0336	-1.0954	1.90
4135	*GIPC3*	4.1909	2.5778	-1.0623	-1.4682	1.90
4136	*CASZ1*	2.7214	2.3772	0.60749	-1.3498	1.90
4137	*CXorf67*	4.6512	0.79371	0.26076	-0.42658	1.90
4138	*NPNT*	4.6406	1.4536	-0.38937	-0.65924	1.90
4139	*PDPR*	2.3993	2.3629	0.94176	0.60858	1.90
4140	*SMIM5*	3.1993	2.7841	-0.27964	-0.76879	1.90
4141	*PLCE1*	4.1853	1.4078	0.10968	-0.29865	1.90
4142	*JOSD1*	5.8731	0.27314	-0.44575	-2.4018	1.90
4143	*C9orf84*	4.7781	0.46507	0.45695	-2.1496	1.90
4144	*AQP6*	3.2497	2.6127	-0.16295	-2.6456	1.90
4145	*CLN8*	2.6195	2.0063	1.0729	-1.1651	1.90
4146	*SLK*	2.1765	1.9434	1.5773	-0.79798	1.90
4147	*DUSP18*	4.2894	1.0439	0.36293	-0.43841	1.90
4148	*E2F2*	4.0753	3.0362	-1.4183	-2.7628	1.90
4149	*ALG12*	3.3515	1.6646	0.67707	-0.40125	1.90
4150	*VRTN*	4.2332	1.1382	0.32059	-1.7865	1.90
4151	*GNA12*	2.4621	1.9076	1.322	0.87408	1.90
4152	*OSTM1*	3.8327	2.1025	-0.24431	-1.0509	1.90
4153	*SLC26A7*	3.1123	2.1046	0.4739	-0.1601	1.90
4154	*ARHGAP36*	4.9904	0.99667	-0.29681	-1.1685	1.90
4155	*ATP4A*	2.9393	2.2029	0.54804	0.095053	1.90
4156	*MRFAP1L1*	4.2238	1.8963	-0.42989	-1.1477	1.90
4157	*INAFM2*	2.8386	2.1493	0.70225	-0.09	1.90
4158	*CDIP1*	2.2581	2.0161	1.4154	-0.31164	1.90
4159	*OR4A47*	5.0173	0.7708	-0.09892	-1.1795	1.90
4160	*HBG2*	4.5839	0.97762	0.1276	-1.3513	1.90
4161	*ZNF587*	3.5435	1.8457	0.29991	-1.6604	1.90
4162	*GRM3*	4.1778	1.8883	-0.37727	-0.38815	1.90
4163	*RCHY1*	4.5738	1.0416	0.07273	-0.45246	1.90
4164	*NDRG3*	4.3344	0.88385	0.46795	-0.11381	1.90
4165	*DENND2A*	3.5744	1.8898	0.22141	-0.97526	1.90
4166	*EMD*	3.6692	1.048	0.96819	-1.2907	1.90
4167	*TPPP2*	3.7739	2.4341	-0.52332	-1.1089	1.89
4168	*OR2M7*	4.1275	2.5209	-0.96407	-1.4881	1.89
4169	*TMED1*	4.1271	1.8469	-0.29194	-0.5898	1.89
4170	*FSCB*	5.0305	0.67393	-0.0227	-1.9573	1.89
4171	*WDR89*	4.0437	1.382	0.25504	-0.52679	1.89
4172	*HDAC10*	2.8729	2.7562	0.05148	-1.3296	1.89
4173	*RAD51AP2*	2.5335	2.3799	0.76684	-1.3074	1.89
4174	*SLC18A2*	4.0131	2.1045	-0.43776	-1.126	1.89
4175	*DAZ3*	2.7859	2.4832	0.41015	0.39006	1.89
4176	*LOC400863*	3.7634	1.0431	0.87266	-0.44134	1.89
4177	*PRY2*	4.1844	1.3132	0.18083	-1.725	1.89
4178	*RRNAD1*	3.2287	2.3451	0.10414	-1.3237	1.89
4179	*CDH20*	3.297	2.8926	-0.51278	-0.56737	1.89

4180	*MMEL1*	5.9459	0.74882	-1.0191	-2.0791	1.89
4181	*BAG5*	4.3961	1.5906	-0.31164	-2.2313	1.89
4182	*DEFB113*	4.795	0.61572	0.26415	-1.5415	1.89
4183	*SH2D3A*	3.4365	1.9341	0.30403	-1.0764	1.89
4184	*VAMP1*	5.1363	0.39188	0.14638	-1.0359	1.89
4185	*PAN3*	3.9242	3.277	-1.5268	-1.748	1.89
4186	*ZNF14*	5.0988	2.0477	-1.4722	-2.3885	1.89
4187	*DYNC1LI1*	3.5187	2.0436	0.11192	-0.43276	1.89
4188	*AKNAD1*	3.2139	1.5769	0.88332	-1.3668	1.89
4189	*GOLGA6A*	3.7834	3.0453	-1.1553	-1.1845	1.89
4190	*C6orf15*	3.9457	1.845	-0.11745	-0.59061	1.89
4191	*CALML4*	3.1345	1.8361	0.70264	-1.7988	1.89
4192	*ZFY*	5.2511	0.54868	-0.12681	-0.7757	1.89
4193	*TMEM70*	4.8986	0.65386	0.11986	-0.75762	1.89
4194	*DHDH*	3.0651	1.7718	0.83495	-0.42615	1.89
4195	*RBMY1E*	3.5632	1.8525	0.25544	-0.27421	1.89
4196	*STAP1*	3.3084	1.8686	0.49269	-0.01218	1.89
4197	*S100A6*	4.377	0.72847	0.56396	0.459	1.89
4198	*OR52M1*	3.1306	1.6946	0.84414	-0.49684	1.89
4199	*CRLF3*	3.1083	2.501	0.05963	-1.0185	1.89
4200	*CRYBB3*	3.7183	2.3812	-0.43057	-1.3255	1.89
4201	*MLPH*	3.7635	1.907	-0.00256	-0.52494	1.89
4202	*MAP2K7*	3.3421	2.5642	-0.23846	-0.93489	1.89
4203	*PLEK*	3.6496	1.8265	0.1915	-0.37282	1.89
4204	*PTPRN2*	4.0464	1.5081	0.11287	-1.1272	1.89
4205	*CDC27*	4.073	1.7618	-0.16749	-2.3552	1.89
4206	*TTPA*	4.2297	1.1964	0.24016	-0.9879	1.89
4207	*MYBPC1*	4.1132	2.2468	-0.69382	-1.7547	1.89
4208	*TPCN1*	4.5913	1.2178	-0.14312	-0.79962	1.89
4209	*CD34*	3.3106	2.2064	0.14819	-1.2195	1.89
4210	*FAM199X*	4.0785	1.321	0.26546	0.18914	1.89
4211	*LILRA5*	3.084	2.8614	-0.28169	-1.6089	1.89
4212	*PRR7*	5.0103	0.34578	0.30757	-0.04796	1.89
4213	*ATM*	4.1702	2.6029	-1.1117	-1.3568	1.89
4214	*ZNF592*	3.0198	1.7366	0.90495	-1.5502	1.89
4215	*ZNF689*	3.4615	1.4602	0.73945	-0.7329	1.89
4216	*PHF21A*	3.5742	1.995	0.09128	-0.72488	1.89
4217	*HSPA8*	3.2636	1.9625	0.43381	-1.0441	1.89
4218	*GSDMB*	2.6556	2.2214	0.78047	-0.54008	1.89
4219	*HBP1*	2.5046	1.9258	1.2265	-0.02105	1.89
4220	*SLC5A7*	2.3048	1.7848	1.5659	0.2664	1.89
4221	*KCNJ9*	2.7721	2.48	0.40277	0.30546	1.88
4222	*DBN1*	2.7155	2.4516	0.4876	-0.48905	1.88
4223	*C1QL2*	2.6316	2.3652	0.65788	-2.2599	1.88
4224	*RNF19B*	3.1238	1.4721	1.0578	-0.76541	1.88
4225	*SEC11A*	4.1473	2.9181	-1.4123	-1.7787	1.88

4226	*CAPZA2*	4.4102	1.153	0.0896	-0.78588	1.88
4227	*DOT1L*	4.4481	2.5383	-1.3338	-1.5817	1.88
4228	*OR4C11*	3.4905	2.1193	0.04269	-0.30559	1.88
4229	*GMIP*	4.5171	1.4374	-0.30279	-0.85829	1.88
4230	*VPS54*	3.2696	1.4788	0.90318	0.85108	1.88
4231	*GPR32*	3.3551	2.6289	-0.33246	-0.49867	1.88
4232	*TBC1D8B*	3.3979	3.2186	-0.96632	-1.3637	1.88
4233	*SCN4B*	4.2742	2.3666	-0.99088	-1.4407	1.88
4234	*IL10RA*	3.5709	2.1703	-0.09161	-0.50041	1.88
4235	*LRRC63*	3.1519	2.3396	0.15768	-0.38671	1.88
4236	*DNAJC30*	3.5619	1.2469	0.83845	-1.1641	1.88
4237	*NEU1*	4.9589	0.48719	0.20086	-0.91842	1.88
4238	*DDX26B*	3.961	0.90769	0.77753	-0.77038	1.88
4239	*ABHD3*	3.6776	1.9159	0.05267	-0.54579	1.88
4240	*COL6A6*	4.9756	0.66103	0.00926	-0.36192	1.88
4241	*ZMIZ2*	3.3861	2.8386	-0.57899	-2.4904	1.88
4242	*CPOX*	4.4489	1.6178	-0.42195	-2.1429	1.88
4243	*ICE2*	5.2528	0.57972	-0.18819	-0.95708	1.88
4244	*ANKS6*	2.9951	2.5596	0.08594	-2.0766	1.88
4245	*ZNF705G*	4.3157	1.7483	-0.4235	-0.71093	1.88
4246	*ZNF24*	2.7746	1.6385	1.2269	0.2536	1.88
4247	*TSPAN5*	4.3596	1.1685	0.11177	-1.7232	1.88
4248	*PBX4*	4.0496	1.7345	-0.1449	-0.25711	1.88
4249	*C15orf59*	2.4409	1.8838	1.3141	-1.2287	1.88
4250	*C2CD4B*	5.5582	0.096888	-0.01766	-1.136	1.88
4251	*LRRC23*	5.3654	1.3731	-1.1021	-2.2455	1.88
4252	*NACC1*	3.9439	1.017	0.67333	-1.2084	1.88
4253	*APOBEC1*	2.0372	1.8053	1.7899	-0.75512	1.88
4254	*LDOC1L*	2.5989	2.474	0.55945	-0.20687	1.88
4255	*GATB*	4.2666	2.7576	-1.392	-2.6491	1.88
4256	*DLAT*	3.5703	2.3631	-0.30135	-0.99876	1.88
4257	*MYO15A*	4.7224	0.74251	0.16601	-1.2071	1.88
4258	*TGIF1*	4.4723	0.71569	0.44282	-0.43875	1.88
4259	*DUSP16*	3.1825	2.268	0.178	-0.24356	1.88
4260	*CD58*	3.5775	2.2197	-0.16927	-1.0553	1.88
4261	*DUSP15*	4.4473	1.1121	0.06635	-1.1308	1.88
4262	*GUCA1A*	4.3745	0.62921	0.62181	-1.4633	1.88
4263	*NEB*	3.478	2.8579	-0.71051	-0.76371	1.88
4264	*KPTN*	3.9973	1.3638	0.26393	-2.4858	1.88
4265	*TGM4*	3.3856	1.3023	0.93707	0.9038	1.87
4266	*TMEM56-RWDD3*	3.3702	2.6519	-0.39838	-1.4557	1.87
4267	*TMEM62*	3.0738	2.8071	-0.25734	-1.6673	1.87
4268	*RAPGEF3*	2.8998	2.1702	0.55312	-0.03164	1.87
4269	*PRH1*	4.4356	0.82326	0.36378	-0.6799	1.87
4270	*FAF1*	3.6176	2.1398	-0.13488	-0.17712	1.87
4271	*METTL23*	4.3783	0.62309	0.62065	-0.6951	1.87

4272	*ZNF473*	3.2565	1.8656	0.4991	-0.9554	1.87
4273	*RASA4*	4.0626	2.7839	-1.2257	-1.5875	1.87
4274	*LRRN4CL*	4.8428	1.3914	-0.61374	-0.72776	1.87
4275	*GTSCR1*	4.2394	0.76817	0.6116	0.047153	1.87
4276	*DSG4*	2.508	1.7509	1.3597	-0.06641	1.87
4277	*KCNJ12*	3.3182	2.1713	0.12799	-0.11602	1.87
4278	*ERICH6*	3.4517	2.2992	-0.1339	-0.67846	1.87
4279	*ACBD3*	4.8646	0.70976	0.04192	-2.4825	1.87
4280	*TTC32*	4.3788	0.68035	0.55585	-0.2765	1.87
4281	*SLC12A4*	3.9863	2.4671	-0.83851	-1.8335	1.87
4282	*IFI30*	4.8709	0.95321	-0.20958	-1.8866	1.87
4283	*MMP17*	5.2001	1.0893	-0.67688	-1.917	1.87
4284	*CCNL1*	3.1361	1.7785	0.69763	0.057818	1.87
4285	*MORC1*	4.0307	2.3775	-0.79632	-1.1357	1.87
4286	*HN1L*	3.8257	2.2772	-0.49127	-1.7496	1.87
4287	*FUZ*	4.5166	1.5216	-0.42938	-0.63483	1.87
4288	*FAM49A*	4.1505	1.6799	-0.22191	-1.7789	1.87
4289	*MEX3D*	3.3028	3.1236	-0.81838	-0.88661	1.87
4290	*VCX*	4.3145	1.1257	0.1676	-0.09468	1.87
4291	*C6orf118*	4.4882	0.92815	0.19099	-2.1745	1.87
4292	*MT1G*	3.2849	1.5002	0.82109	-1.8081	1.87
4293	*MAP2K4*	5.0295	1.0752	-0.49902	-2.7277	1.87
4294	*PTGDR*	4.4163	1.5852	-0.39603	-1.4688	1.87
4295	*ST8SIA6*	3.7151	2.5287	-0.63912	-0.7819	1.87
4296	*OR1K1*	4.2665	1.6153	-0.27804	-1.8516	1.87
4297	*HSPBAP1*	4.0009	1.3557	0.24672	-0.74604	1.87
4298	*NCOA6*	4.6244	1.0984	-0.11955	-0.88279	1.87
4299	*SAMM50*	5.0567	0.6839	-0.13746	-1.0949	1.87
4300	*VPS37C*	3.4994	2.2024	-0.09906	-1.451	1.87
4301	*ZNF616*	2.7435	1.4413	1.4152	-1.0123	1.87
4302	*GNAI3*	3.7544	1.8661	-0.02056	-0.38011	1.87
4303	*SPAG4*	3.1206	1.3921	1.087	-1.5502	1.87
4304	*KCTD16*	3.1379	1.4652	0.99569	-0.88418	1.87
4305	*TJAP1*	4.9012	1.1125	-0.41568	-1.8049	1.87
4306	*GREM2*	3.0659	1.9024	0.62913	0.33767	1.87
4307	*S100A4*	3.8436	1.2152	0.5375	0.52635	1.87
4308	*CYP19A1*	3.4636	1.8577	0.27462	-0.05181	1.87
4309	*AFAP1*	3.9184	3.3608	-1.6847	-2.0351	1.86
4310	*NTNG1*	2.8206	1.9296	0.84357	0.1368	1.86
4311	*IP6K2*	3.1938	2.3611	0.03873	-0.70255	1.86
4312	*CCRN4L*	3.4865	2.8494	-0.74308	-1.296	1.86
4313	*LGALS7*	3.4357	3.1607	-1.0039	-2.6549	1.86
4314	*GAB3*	3.0808	1.9468	0.56456	-0.35894	1.86
4315	*ARHGAP29*	6.0891	0.15856	-0.65707	-2.0429	1.86
4316	*OR13C2*	2.8105	2.6551	0.12492	-0.74179	1.86
4317	*PROB1*	3.0344	2.1262	0.42954	0.10343	1.86

4318	*ANO9*	3.5898	2.0617	-0.062	-1.0216	1.86
4319	*C16orf70*	2.154	1.8965	1.5389	-0.61682	1.86
4320	*KDM4E*	4.335	0.96148	0.29117	-0.37213	1.86
4321	*VCAM1*	3.6946	2.6228	-0.73051	-2.2445	1.86
4322	*TBK1*	3.4584	1.9358	0.19215	-0.33709	1.86
4323	*CT45A6*	3.6814	1.2383	0.66434	-1.9251	1.86
4324	*PLA2G3*	5.304	0.25544	0.02442	-1.3505	1.86
4325	*TTC21A*	3.1746	2.9645	-0.55544	-0.73014	1.86
4326	*HAMP*	2.6786	2.2411	0.6633	-2.0423	1.86
4327	*SSX2IP*	3.3762	2.1836	0.0224	-0.66493	1.86
4328	*SWAP70*	4.0416	0.79797	0.74226	-1.2154	1.86
4329	*HOXA5*	2.9029	2.2047	0.47286	-2.1802	1.86
4330	*KLHL7*	4.478	1.3946	-0.29273	-2.1239	1.86
4331	*BSX*	3.3475	2.442	-0.20988	-0.94119	1.86
4332	*OR2A7*	3.7834	1.2588	0.53705	-0.39011	1.86
4333	*PDLIM1*	2.6438	2.1463	0.78909	-0.19618	1.86
4334	*GADL1*	3.2699	2.0721	0.23712	-1.5883	1.86
4335	*EDNRB*	2.6518	1.8854	1.0415	0.78462	1.86
4336	*EAF2*	3.1306	2.7342	-0.28632	-1.1898	1.86
4337	*IQCE*	3.5022	2.9177	-0.84151	-1.0933	1.86
4338	*HSPA13*	2.7602	2.0294	0.78805	-1.5352	1.86
4339	*CHEK2*	3.7614	1.1933	0.62265	0.031866	1.86
4340	*H6PD*	3.9589	2.4853	-0.86744	-1.3258	1.86
4341	*FZD4*	3.4271	1.3235	0.82607	0.30419	1.86
4342	*CPT1B*	3.3587	1.2574	0.96019	5.25E-05	1.86
4343	*SPECC1*	5.4397	0.13285	0.00293	-1.4859	1.86
4344	*OR8J3*	5.3879	0.9219	-0.73483	-1.4842	1.86
4345	*AS3MT*	2.3257	2.1778	1.0704	-2.7863	1.86
4346	*FAM53B*	3.5228	1.8486	0.20174	-1.2317	1.86
4347	*OR8I2*	3.7653	1.2498	0.55758	-1.4312	1.86
4348	*LRCH3*	3.37	2.8185	-0.61632	-1.7649	1.86
4349	*FOXB1*	4.5132	0.9031	0.15586	-0.31299	1.86
4350	*SIM2*	3.3703	2.3536	-0.15201	-2.4481	1.86
4351	*PF4V1*	4.253	0.97053	0.34829	-0.29953	1.86
4352	*TUBA1C*	4.6811	1.4376	-0.54826	-1.7931	1.86
4353	*PKNOX1*	3.1094	2.6397	-0.17952	-0.49867	1.86
4354	*FSHB*	2.6397	1.9186	1.0103	-0.6602	1.86
4355	*C10orf53*	3.4649	2.5223	-0.4188	-0.62585	1.86
4356	*NBPF8*	4.3409	1.1609	0.06603	-1.4048	1.86
4357	*HOOK3*	3.6806	2.0729	-0.18633	-0.531	1.86
4358	*TMEM134*	3.6892	1.5735	0.30413	-0.57567	1.86
4359	*MSL1*	3.7714	1.6391	0.15417	-1.5965	1.85
4360	*WDR45*	4.5004	1.2076	-0.14391	-0.66689	1.85
4361	*AP5S1*	2.509	1.7346	1.3181	0.14155	1.85
4362	*GPR119*	4.9855	1.9874	-1.4112	-2.7254	1.85
4363	*POU2F2*	3.5016	2.6998	-0.64278	-0.79392	1.85

4364	*IQCA1L*	4.4329	0.56529	0.56025	-0.48911	1.85
4365	*LUZP4*	5.7216	0.68717	-0.85059	-1.6368	1.85
4366	*TMSB15B*	3.7607	1.9701	-0.17331	-0.86358	1.85
4367	*ZNF317*	5.3138	0.33366	-0.09022	-0.35946	1.85
4368	*DPY19L2*	4.8737	0.34486	0.33826	-0.0171	1.85
4369	*TBC1D8*	3.4209	1.9101	0.22562	-0.42369	1.85
4370	*APBB1IP*	5.0784	0.61545	-0.13748	-0.97237	1.85
4371	*CORIN*	3.6999	2.6834	-0.82705	-1.2447	1.85
4372	*TIMD4*	3.7685	1.2917	0.49592	0.18216	1.85
4373	*C10orf35*	3.7863	1.6906	0.07914	-0.6622	1.85
4374	*B3GALTL*	4.6066	1.0113	-0.06271	-1.0416	1.85
4375	*SLC22A1*	4.2826	0.71509	0.55662	-0.22803	1.85
4376	*TMEM213*	3.0169	2.3172	0.21879	-1.2249	1.85
4377	*HAND1*	3.3235	2.2658	-0.03648	-0.33967	1.85
4378	*RBFOX1*	3.9153	1.0457	0.59119	-1.0344	1.85
4379	*LYRM1*	2.8386	2.3833	0.32941	0.067859	1.85
4380	*UBE2L3*	4.4613	0.72375	0.36615	-1.9358	1.85
4381	*OSBPL11*	3.502	1.771	0.27723	-1.183	1.85
4382	*B3GALT2*	4.4073	1.3073	-0.16468	-1.9099	1.85
4383	*TAP1*	4.6362	0.48648	0.4271	-1.3297	1.85
4384	*LGR5*	2.7071	2.5842	0.25825	-2.3678	1.85
4385	*ERCC6-PGBD3*	3.4253	1.1965	0.92716	-0.99137	1.85
4386	*PLS3*	3.6316	1.7694	0.14607	-0.73546	1.85
4387	*GINM1*	4.4	1.0784	0.06861	-0.28819	1.85
4388	*PON2*	3.9377	2.9188	-1.3114	-1.7911	1.85
4389	*PGM2*	5.4728	0.33464	-0.26237	-1.6826	1.85
4390	*RAB24*	3.1879	2.1521	0.2036	-0.32625	1.85
4391	*MBL2*	3.5542	2.9176	-0.92861	-1.761	1.85
4392	*FAM64A*	2.5832	2.5509	0.40862	-0.24887	1.85
4393	*UNC93B1*	3.5253	2.0484	-0.03102	-1.356	1.85
4394	*SOX8*	3.575	1.4246	0.54305	0.49463	1.85
4395	*LSR*	2.5452	2.1828	0.81297	-0.40482	1.85
4396	*AQP9*	2.8568	2.5729	0.1087	-1.9052	1.85
4397	*MPV17L2*	3.2499	2.4535	-0.16651	-0.4246	1.85
4398	*MIXL1*	4.042	2.4257	-0.93114	-1.5408	1.85
4399	*SCN1A*	3.2097	1.5709	0.75466	0.51723	1.85
4400	*UHRF1*	4.5062	0.68863	0.34032	-0.26357	1.85
4401	*TACR2*	2.6655	1.6409	1.2284	0.29118	1.84
4402	*IMPG2*	4.9837	0.50854	0.04244	-0.94747	1.84
4403	*TRPC1*	4.5766	0.48199	0.47594	-0.95964	1.84
4404	*DYM*	4.2438	0.85492	0.43515	-1.1761	1.84
4405	*MUT*	2.7304	1.5965	1.2051	1.1252	1.84
4406	*CEP152*	4.4055	0.72088	0.4055	0.17543	1.84
4407	*ZNF132*	4.0423	1.4611	0.02759	-1.8306	1.84
4408	*RAMP3*	3.1194	1.7718	0.63936	0.43058	1.84
4409	*WNT3A*	2.9588	1.386	1.1852	0.11018	1.84

4410	*COX6A2*	3.2949	2.2309	0.00408	-0.79573	1.84
4411	*ETV6*	3.8562	1.8746	-0.20105	-0.20626	1.84
4412	*LALBA*	4.4252	2.1743	-1.0706	-1.5512	1.84
4413	*FAM124A*	6.0557	0.31225	-0.84001	-3.1023	1.84
4414	*ACBD6*	3.3764	1.3339	0.81698	-0.54126	1.84
4415	*C22orf29*	4.023	2.6925	-1.1891	-1.4811	1.84
4416	*CDPF1*	3.3285	1.564	0.63342	-0.84841	1.84
4417	*MATK*	3.2672	2.3412	-0.08269	-0.42701	1.84
4418	*IGF1R*	3.2198	2.7397	-0.43398	-1.7072	1.84
4419	*GFI1B*	3.8094	2.0634	-0.34809	-0.40863	1.84
4420	*MAGEB10*	2.4618	1.8218	1.241	0.64075	1.84
4421	*NPW*	3.1439	2.0197	0.36064	-1.1108	1.84
4422	*RBM4B*	4.1066	1.9235	-0.50685	-1.3912	1.84
4423	*DHRS7B*	4.1625	0.78842	0.57188	-1.1715	1.84
4424	*GNMT*	2.6818	1.5467	1.2932	-1.7753	1.84
4425	*H2AFY*	5.3269	0.64059	-0.44661	-4.0089	1.84
4426	*ALLC*	2.5595	2.4621	0.49881	-1.8293	1.84
4427	*TRIP11*	2.9001	2.3521	0.26807	-0.04917	1.84
4428	*AADACL3*	5.0678	0.52531	-0.0729	-1.4104	1.84
4429	*IL1RL2*	3.993	2.0761	-0.54914	-2.012	1.84
4430	*ENPP7*	2.6019	1.892	1.0255	-1.1681	1.84
4431	*NDUFB1*	4.1812	0.93545	0.40171	-1.1144	1.84
4432	*ADH6*	3.6521	1.9891	-0.124	-0.77794	1.84
4433	*GOLGA6D*	3.0114	2.8211	-0.31624	-1.525	1.84
4434	*LAS1L*	3.6437	2.3951	-0.52269	-1.2643	1.84
4435	*ABCG1*	4.0963	1.4561	-0.03683	-1.5073	1.84
4436	*DSC3*	2.336	1.7517	1.4265	0.39603	1.84
4437	*RBM14-RBM4*	2.9325	2.5563	0.0251	-0.42877	1.84
4438	*MAP10*	2.8889	2.1453	0.47908	0.33208	1.84
4439	*REN*	3.2948	1.8138	0.40461	-0.67556	1.84
4440	*KRTAP3-1*	2.6498	1.9187	0.94468	-1.4043	1.84
4441	*PCDHGA8*	3.5692	1.8951	0.04877	-1.8503	1.84
4442	*ENO4*	3.9871	0.91266	0.61192	-0.36285	1.84
4443	*CALML3*	4.7304	1.8476	-1.0669	-1.526	1.84
4444	*CDKN2C*	3.9664	1.0952	0.4488	-1.1692	1.84
4445	*PQLC3*	4.4715	0.86991	0.16852	-1.1507	1.84
4446	*NUDT12*	3.248	1.5534	0.70812	-0.33788	1.84
4447	*TNFRSF12A*	4.8622	1.168	-0.52105	-2.2844	1.84
4448	*KCNQ4*	3.013	2.8205	-0.32443	-2.4338	1.84
4449	*TEX101*	3.5334	1.2337	0.74016	-1.1724	1.84
4450	*MIPOL1*	4.9079	0.4811	0.11761	-1.9654	1.84
4451	*TRAK2*	4.1829	1.83	-0.50637	-0.65895	1.84
4452	*RAB6A*	2.9037	1.4569	1.1456	0.7603	1.84
4453	*CALU*	4.0156	1.9848	-0.49425	-0.68627	1.84
4454	*NCR3*	3.2875	1.8748	0.34341	-1.0623	1.84
4455	*CLN6*	3.3591	3.0183	-0.87418	-2.2473	1.83

4456	*SNTG1*	3.9513	1.5968	-0.04542	-0.62307	1.83
4457	*FAM160A1*	4.1638	1.9992	-0.66164	-0.9481	1.83
4458	*TAF1D*	4.312	0.98486	0.20227	-0.56481	1.83
4459	*KCNQ3*	3.5551	2.4001	-0.45669	-1.3085	1.83
4460	*HTRA3*	2.5207	1.8477	1.1294	1.0782	1.83
4461	*MTHFR*	3.1408	2.1508	0.20432	-1.7027	1.83
4462	*RASA3*	2.3404	2.3086	0.8469	-1.626	1.83
4463	*ASIC4*	3.7631	1.9683	-0.23802	-1.8899	1.83
4464	*CLIP1*	3.9569	0.92224	0.61408	0.14404	1.83
4465	*LASP1*	4.1819	2.8086	-1.4976	-1.5401	1.83
4466	*MCUR1*	4.1825	0.79518	0.51497	-1.6288	1.83
4467	*ITPR1*	5.0103	1.0964	-0.6141	-1.7675	1.83
4468	*FAM9C*	2.7779	1.452	1.2598	-0.19851	1.83
4469	*RCCD1*	4.8367	0.40272	0.25015	-0.18718	1.83
4470	*CEACAM6*	2.65	1.8553	0.98304	-1.7109	1.83
4471	*C17orf80*	4.5766	0.46733	0.44389	-0.94777	1.83
4472	*FCGR2B*	5.488	0.33139	-0.33179	-1.4798	1.83
4473	*SASH3*	3.374	1.1919	0.92166	-1.6952	1.83
4474	*CCNL2*	4.0577	0.80993	0.61894	-1.5415	1.83
4475	*ACSL4*	3.132	1.499	0.85523	-1.25	1.83
4476	*SMIM9*	3.1771	1.2695	1.0394	0.41367	1.83
4477	*SEC22C*	2.3647	1.6087	1.5124	-0.50286	1.83
4478	*LRRC36*	3.3631	1.5467	0.5747	-0.12428	1.83
4479	*VILL*	3.2912	2.2825	-0.09082	-3.629	1.83
4480	*GPALPP1*	3.3411	1.1554	0.98629	-0.48959	1.83
4481	*POLM*	2.5896	1.7331	1.1581	0.43394	1.83
4482	*ZNF451*	4.1945	0.91095	0.37508	-3.8991	1.83
4483	*TMEM235*	2.9653	2.6917	-0.17663	-2.6476	1.83
4484	*GCHFR*	4.8341	0.81424	-0.16962	-1.8033	1.83
4485	*SPATA33*	4.6412	2.2848	-1.4473	-1.7945	1.83
4486	*PRR13*	4.5571	0.83087	0.08711	-0.76099	1.83
4487	*OR10AD1*	2.9847	2.5695	-0.08162	-0.34017	1.82
4488	*CLIP2*	3.2009	1.5295	0.74083	0.33579	1.82
4489	*KIF5C*	4.3916	0.91153	0.16792	-1.0955	1.82
4490	*ADGRF3*	4.3886	1.0676	0.01483	-1.4983	1.82
4491	*ZNF598*	4.1336	0.88229	0.45485	-0.17746	1.82
4492	*PLD1*	5.4087	0.38031	-0.31916	-1.9965	1.82
4493	*ASL*	4.805	1.7578	-1.094	-2.4642	1.82
4494	*NBEAL2*	2.9885	2.4062	0.07189	-1.52	1.82
4495	*CAMK1*	2.2193	2.0664	1.1795	-1.6089	1.82
4496	*CCL25*	3.229	1.1346	1.1005	-2.8019	1.82
4497	*TAPT1*	3.4235	1.0912	0.94909	-0.42938	1.82
4498	*TPRX1*	2.8909	1.8484	0.72332	0.61378	1.82
4499	*OSCAR*	3.3948	1.9333	0.13431	-4.247	1.82
4500	*ACADS*	3.3079	1.4573	0.69682	-0.9472	1.82
4501	*KIRREL*	3.5306	2.4735	-0.54229	-0.57342	1.82

4502	*CT45A2*	4.1199	-0.47913			1.82
4503	*SFTA2*	2.3271	2.014	1.12	-0.61281	1.82
4504	*ZNF497*	3.844	1.0197	0.59737	-1.3082	1.82
4505	*CEP128*	4.1173	2.4947	-1.1511	-2.8645	1.82
4506	*DHFRL1*	2.5111	2.2796	0.66964	-0.84337	1.82
4507	*GABRR3*	3.0776	2.0356	0.3463	-0.86261	1.82
4508	*PLVAP*	4.4033	1.3076	-0.25198	-2.2879	1.82
4509	*SEMA7A*	4.2026	2.0491	-0.79422	-2.7427	1.82
4510	*CAPN3*	4.0066	2.5452	-1.0949	-1.5912	1.82
4511	*GABARAPL1*	4.5431	1.1105	-0.1967	-0.99463	1.82
4512	*FAM131A*	2.9735	1.4686	1.0143	-1.6575	1.82
4513	*NUDT7*	4.1481	0.67592	0.63229	-1.3946	1.82
4514	*ACP1*	3.5573	1.6299	0.26835	-1.448	1.82
4515	*POTEB3*	4.6336	0.48128	0.34029	-1.9431	1.82
4516	*CCDC160*	4.4297	0.61036	0.41434	-2.0116	1.82
4517	*HIF3A*	3.3559	2.3268	-0.2283	-2.5452	1.82
4518	*PRR25*	4.0541	1.0984	0.3004	-1.8095	1.82
4519	*RSPH9*	3.8372	1.9046	-0.28952	-1.1075	1.82
4520	*ARAF*	3.6694	2.2832	-0.50221	-1.3184	1.82
4521	*BDKRB1*	3.0455	1.2686	1.1349	0.16498	1.82
4522	*CYP2A7*	3.8588	1.1817	0.40785	0.094924	1.82
4523	*KRT28*	5.251	0.17256	0.02418	-0.2273	1.82
4524	*MYCT1*	2.8064	2.1529	0.48477	-0.40223	1.81
4525	*ZNF100*	2.9114	1.3421	1.1896	-1.4852	1.81
4526	*SH2D2A*	3.2288	1.8415	0.37258	-0.34144	1.81
4527	*NEIL2*	2.9461	1.935	0.56161	-1.2301	1.81
4528	*SOCS2*	4.9258	0.33218	0.18366	0.074008	1.81
4529	*DRG2*	3.1105	1.5056	0.8254	0.49507	1.81
4530	*OR6B2*	3.1964	1.5595	0.68482	-0.06857	1.81
4531	*KCTD9*	4.3579	1.5062	-0.42411	-1.2758	1.81
4532	*FAM19A2*	2.9226	1.6751	0.84084	-1.5663	1.81
4533	*ECHDC2*	4.5399	0.72495	0.1733	-0.72468	1.81
4534	*DCT*	3.1433	1.3945	0.90025	-1.0304	1.81
4535	*KMT2B*	2.3152	1.7441	1.3785	-0.13224	1.81
4536	*RNF175*	1.9208	1.7889	1.7279	1.4211	1.81
4537	*CELF6*	5.0404	0.71818	-0.32121	-1.4736	1.81
4538	*GPR176*	3.2019	3.0518	-0.81746	-1.1465	1.81
4539	*EGLN1*	3.2476	1.9779	0.20644	-1.2277	1.81
4540	*NRROS*	3.2398	1.1702	1.0212	-1.57	1.81
4541	*OR2G6*	3.112	2.4778	-0.16091	-0.95474	1.81
4542	*TMPRSS11B*	2.5804	2.4461	0.40117	0.35867	1.81
4543	*FAM186B*	2.8959	1.3389	1.1903	-1.2411	1.81
4544	*GPR63*	4.0797	0.97793	0.36744	-1.2094	1.81
4545	*C8orf46*	4.4025	0.74095	0.28061	-1.3056	1.81
4546	*IL9R*	3.8706	1.5689	-0.01595	-1.4269	1.81
4547	*PAQR9*	3.7511	2.1572	-0.48973	-2.005	1.81

4548	*IRF3*	3.6116	2.1053	-0.29835	-1.1486	1.81
4549	*MAPK10*	3.3926	2.7366	-0.71119	-1.371	1.81
4550	*HLA-DRB5*	3.4596	1.5142	0.44404	0.052351	1.81
4551	*OR1F1*	2.3676	2.3253	0.72493	-1.0852	1.81
4552	*RGS19*	4.3546	2.5875	-1.5243	-4.0338	1.81
4553	*SRL*	5.0704	0.37537	-0.02943	-0.21797	1.81
4554	*ZDHHC16*	3.2024	2.3264	-0.1126	-0.98842	1.81
4555	*IGF2R*	4.9715	2.4129	-1.9686	-1.9938	1.81
4556	*FRG2*	4.5464	2.1423	-1.2736	-2.0249	1.81
4557	*TYMSOS*	4.4998	0.86038	0.05417	-1.7513	1.80
4558	*MYL9*	5.8886	0.013161	-0.48756	-1.5458	1.80
4559	*CARD10*	3.6578	2.7891	-1.0329	-1.2195	1.80
4560	*BTC*	3.9237	1.4258	0.06425	-1.4509	1.80
4561	*HACE1*	3.1337	2.5635	-0.28581	-0.39127	1.80
4562	*TEX15*	2.927	1.3493	1.135	-0.33444	1.80
4563	*SLC6A8*	5.2097	1.5211	-1.3209	-3.7932	1.80
4564	*POTEB2*	4.8714	1.7131	-1.1747	-1.5894	1.80
4565	*ZNF404*	3.117	2.9504	-0.65811	-0.85977	1.80
4566	*GIMD1*	4.1241	3.2055	-1.9213	-2.0542	1.80
4567	*RPL8*	4.3649	2.3004	-1.257	-2.4762	1.80
4568	*PRSS37*	2.7671	1.8835	0.75699	-0.92695	1.80
4569	*TMEM18*	4.0183	0.97691	0.41106	-1.6867	1.80
4570	*FUBP1*	3.3555	2.4852	-0.43467	-2.0102	1.80
4571	*NANS*	4.2434	1.4143	-0.25332	-1.0221	1.80
4572	*ASB16*	4.207	2.4085	-1.2114	-1.7789	1.80
4573	*CCL7*	4.2593	0.87196	0.27109	-0.53009	1.80
4574	*KCTD4*	4.4711	1.5566	-0.6254	-1.0884	1.80
4575	*SNED1*	3.3388	2.2088	-0.14556	-1.4541	1.80
4576	*PDF*	4.1244	2.0628	-0.78593	-1.7963	1.80
4577	*TBC1D32*	2.6749	1.7188	1.0068	-1.9523	1.80
4578	*CCDC129*	3.0841	1.8106	0.50457	0.48352	1.80
4579	*DNAH7*	3.4983	2.6835	-0.78329	-1.1764	1.80
4580	*BMP3*	3.8428	1.3075	0.24759	-0.20079	1.80
4581	*RGS14*	3.3772	1.4923	0.52744	-0.71723	1.80
4582	*COL9A2*	3.9082	0.8509	0.63773	-0.87725	1.80
4583	*LOC102724279*	5.4371	0.50701	-0.54743	-1.1062	1.80
4584	*CIZ1*	2.3569	2.219	0.81814	0.19038	1.80
4585	*BMP15*	4.4689	1.3696	-0.44528	-0.54786	1.80
4586	*GMFG*	3.0507	2.5399	-0.19984	-0.88684	1.80
4587	*NEUROD1*	3.16	2.1736	0.05705	-0.34352	1.80
4588	*EMILIN1*	3.7462	1.2928	0.35144	-1.5138	1.80
4589	*CNKSR3*	4.4695	0.66081	0.2592	-1.4209	1.80
4590	*LCE5A*	3.8432	1.9392	-0.39338	-0.50718	1.80
4591	*SULT2A1*	2.6325	2.607	0.14942	-0.63572	1.80
4592	*BCAN*	2.9173	2.428	0.0418	-1.5289	1.80
4593	*FBXW12*	3.7314	1.5436	0.11164	-1.0884	1.80

4594	*NOX3*	3.5909	1.6507	0.14491	-1.1491	1.80
4595	*TEP1*	3.3431	3.1629	-1.1214	-1.3007	1.79
4596	*OR10G9*	3.6087	1.0506	0.72504	-0.96407	1.79
4597	*SLC26A10*	2.6879	1.7922	0.90263	-2.9068	1.79
4598	*TRIM5*	3.3243	2.1227	-0.0658	-1.5911	1.79
4599	*DDO*	3.1056	2.9255	-0.65092	-1.3594	1.79
4600	*SAMD4B*	4.3902	2.1272	-1.1392	-1.478	1.79
4601	*USP12*	4.6299	1.4761	-0.72798	-1.4182	1.79
4602	*KIAA1429*	2.9535	2.1966	0.22426	-1.6049	1.79
4603	*GXYLT1*	4.4611	0.50981	0.40291	-2.4229	1.79
4604	*WDR45B*	3.3119	1.1613	0.89991	-0.7391	1.79
4605	*MTDH*	3.7495	1.8566	-0.23404	-2.3915	1.79
4606	*PSKH2*	3.7838	1.0686	0.51902	-1.9302	1.79
4607	*CDY1B*	3.6587	1.4325	0.27983	-0.31362	1.79
4608	*ATP6V1C2*	2.8892	2.1694	0.31225	-1.6105	1.79
4609	*FAM86C1*	3.6891	2.2095	-0.52839	-1.1637	1.79
4610	*MTCL1*	3.7366	0.95663	0.6763	-0.03342	1.79
4611	*FBXO22*	2.9716	2.1728	0.22456	-1.2027	1.79
4612	*NPC2*	3.1803	1.8816	0.30552	-0.54155	1.79
4613	*P2RY12*	4.5283	0.71573	0.12321	-0.98653	1.79
4614	*GSAP*	2.9269	1.8796	0.56041	-1.2732	1.79
4615	*SNAP91*	3.9105	1.2668	0.18868	-2.0953	1.79
4616	*S100A2*	4.2196	0.71638	0.42947	-2.0398	1.79
4617	*SELPLG*	3.2159	2.5344	-0.38655	-0.92028	1.79
4618	*GRID2*	4.8536	1.1567	-0.64986	-0.77127	1.79
4619	*AMY1B*	2.7254	2.277	0.35756	-0.5169	1.79
4620	*CERS4*	4.5049	1.2763	-0.42153	-3.8502	1.79
4621	*NIPSNAP3B*	3.9874	2.3255	-0.95388	-1.4509	1.79
4622	*ASXL3*	5.3132	1.1057	-1.0599	-2.095	1.79
4623	*KLHL22*	3.4971	1.5607	0.30105	-0.68911	1.79
4624	*TDP2*	4.0397	1.6454	-0.3271	-1.2305	1.79
4625	*MRM1*	3.1342	2.2871	-0.06383	-1.0021	1.79
4626	*STAC3*	3.6312	0.98335	0.7424	-1.545	1.79
4627	*PLD5*	1.9092	1.7263	1.7208	1.1035	1.79
4628	*SSPN*	3.5032	2.2966	-0.44353	-0.86123	1.79
4629	*FAM83D*	2.9803	2.6983	-0.32248	-1.1961	1.79
4630	*FOLR3*	3.4089	1.6604	0.2846	-1.2826	1.78
4631	*FSHR*	4.856	1.5232	-1.0258	-2.1802	1.78
4632	*DOCK2*	4.5625	2.4208	-1.6313	-2.28	1.78
4633	*ATG4B*	2.4771	2.457	0.4165	-0.38675	1.78
4634	*RBM39*	2.5183	2.3435	0.48703	-0.83584	1.78
4635	*C2*	2.728	2.3625	0.25821	-0.6118	1.78
4636	*GLRX3*	3.425	2.2175	-0.29402	-0.78229	1.78
4637	*SETD6*	3.5708	3.514	-1.7365	-1.8157	1.78
4638	*NOTCH1*	4.0386	0.81337	0.4961	-0.36246	1.78
4639	*OCA2*	3.6414	1.0213	0.6851	-0.06182	1.78

4640	*PSMD10*	4.2152	0.61617	0.51616	-1.1676	1.78
4641	*USP17L2*	3.0139	2.7078	-0.37417	-1.8822	1.78
4642	*KIT*	4.5948	1.1242	-0.37194	-1.2052	1.78
4643	*WDR63*	3.3723	1.5491	0.42458	0.20047	1.78
4644	*SULT1C4*	4.682	0.6335	0.02927	-0.17439	1.78
4645	*CD3G*	4.8061	0.89936	-0.36086	-0.43472	1.78
4646	*GZF1*	3.6224	1.3173	0.40417	-1.2827	1.78
4647	*CPNE2*	3.1505	2.1701	0.023	-0.35272	1.78
4648	*TOR4A*	2.9906	1.2282	1.1244	0.58508	1.78
4649	*DEFB104B*	3.0964	1.6217	0.62473	-1.0029	1.78
4650	*TFPI2*	4.7864	1.8498	-1.2935	-1.4498	1.78
4651	*L1TD1*	2.7583	1.912	0.67208	-0.08356	1.78
4652	*NDUFS1*	3.4638	1.9627	-0.08477	-0.65889	1.78
4653	*TCEB3B*	2.8491	2.2498	0.24221	-0.64164	1.78
4654	*SPEN*	2.5741	1.9402	0.82658	-0.4464	1.78
4655	*EXOC2*	4.1906	0.67748	0.4727	-1.1756	1.78
4656	*OXNAD1*	3.6545	1.0047	0.68058	-0.36855	1.78
4657	*PGK1*	2.616	1.6724	1.0505	-0.93024	1.78
4658	*DNTT*	4.61	0.45281	0.27574	-3.087	1.78
4659	*PGBD4*	3.4954	2.6674	-0.8253	-1.0486	1.78
4660	*SHMT1*	3.0849	2.696	-0.44353	-2.4193	1.78
4661	*DENND5B*	3.2516	1.1903	0.89509	-0.97509	1.78
4662	*CLTA*	3.2583	1.0428	1.0357	0.33194	1.78
4663	*SLC1A4*	4.1617	1.703	-0.52795	-1.8462	1.78
4664	*UBLCP1*	4.5649	1.3467	-0.57625	-0.91793	1.78
4665	*SEC61A2*	2.459	2.2953	0.58038	-2.1134	1.78
4666	*OR12D2*	3.8493	1.1399	0.34516	-1.12	1.78
4667	*ARHGAP33*	4.1817	0.70819	0.44389	-2.7384	1.78
4668	*RASSF9*	3.8081	1.9487	-0.42359	-1.0226	1.78
4669	*FAM107B*	4.9622	0.65359	-0.28301	-0.9382	1.78
4670	*KCNN3*	4.1919	2.2169	-1.0761	-1.6781	1.78
4671	*ZNF268*	3.3772	1.2828	0.67234	-0.36913	1.78
4672	*CPAMD8*	1.906	1.7857	1.6406	0.96239	1.78
4673	*TMEM108*	3.3284	1.4865	0.51694	-1.0343	1.78
4674	*GADD45A*	3.5984	2.0582	-0.32719	-1.1258	1.78
4675	*NR1D1*	4.8055	1.7972	-1.2735	-1.9748	1.78
4676	*HDDC3*	3.3958	1.3677	0.56529	-0.714	1.78
4677	*NOA1*	4.357	0.97102	0.00025	-1.8858	1.78
4678	*C17orf97*	5.3707	0.12602	-0.16868	-1.2367	1.78
4679	*SPERT*	4.1694	1.1016	0.05557	-2.672	1.78
4680	*CD164*	3.9352	2.119	-0.72772	-1.8836	1.78
4681	*B4GALNT1*	3.7139	2.0311	-0.41917	-2.0062	1.78
4682	*ZNF410*	2.3781	1.669	1.2778	1.0505	1.77
4683	*SPACA5B*	2.4474	2.4382	0.43862	-0.13635	1.77
4684	*OR51E1*	4.2023	1.5038	-0.38215	-1.0723	1.77
4685	*PDCD6*	4.2894	1.1182	-0.0869	-1.5243	1.77

4686	*ZC3H3*	4.3008	0.56359	0.45591	-0.41596	1.77
4687	*MATN3*	4.5289	0.82402	-0.03342	-1.2076	1.77
4688	*GPATCH3*	3.68	1.6866	-0.04862	-0.52786	1.77
4689	*OR4C45*	3.3911	2.1015	-0.17498	-0.3819	1.77
4690	*DGKA*	3.5789	3.4165	-1.6791	-1.8572	1.77
4691	*PLXNB2*	2.503	1.7172	1.0961	-0.47527	1.77
4692	*SNPH*	4.1885	2.2915	-1.1637	-2.0675	1.77
4693	*NCS1*	5.3472	0.12509	-0.15842	-0.8792	1.77
4694	*PHF19*	3.4076	1.7167	0.18857	-0.3589	1.77
4695	*OR10AG1*	4.5706	1.239	-0.49704	-1.7945	1.77
4696	*GAL3ST4*	3.2993	1.1491	0.86377	-0.18353	1.77
4697	*RAB3IP*	3.1183	2.9396	-0.74618	-3.1317	1.77
4698	*CCDC178*	4.9133	0.92474	-0.52643	-1.6186	1.77
4699	*NGLY1*	3.9479	0.87678	0.48647	-1.0212	1.77
4700	*PHLPP2*	2.8875	1.6807	0.74217	-1.6386	1.77
4701	*FOXD3*	4.6705	0.43125	0.20829	-2.4015	1.77
4702	*LDHB*	4.0129	2.2925	-0.99608	-1.0837	1.77
4703	*SIRPB2*	2.6044	2.4584	0.24553	-1.6766	1.77
4704	*CHAF1B*	5.2088	0.21956	-0.12054	-0.81496	1.77
4705	*PHYHIP*	3.0523	1.2322	1.023	-1.2975	1.77
4706	*MGP*	4.029	1.6647	-0.38675	-0.57067	1.77
4707	*EMP3*	3.6615	1.8534	-0.20849	-0.72654	1.77
4708	*OR6B3*	3.9152	0.98298	0.40814	-0.98238	1.77
4709	*N4BP2*	4.7197	1.8818	-1.2958	-1.522	1.77
4710	*PRSS8*	2.3528	2.2942	0.65808	-0.9519	1.77
4711	*OR5M11*	3.1115	1.4052	0.78827	-1.6411	1.77
4712	*COPZ1*	5.4908	0.40306	-0.58921	-1.1987	1.77
4713	*AWAT2*	2.6283	2.0896	0.58644	-1.6731	1.77
4714	*TRAFD1*	3.8792	0.86789	0.55585	-2.4735	1.77
4715	*NDUFB7*	2.2461	1.6641	1.3897	0.70422	1.77
4716	*HLA-B*	2.2418	1.6882	1.3692	-1.8591	1.77
4717	*BAK1*	3.199	1.9915	0.10865	0.076527	1.77
4718	*ZNF662*	4.9082	2.1995	-1.8087	-1.8906	1.77
4719	*SRXN1*	2.5599	1.378	1.3608	-0.47496	1.77
4720	*HEXB*	3.981	1.5653	-0.24946	-1.4741	1.77
4721	*DLK2*	5.1843	0.25894	-0.14649	-1.3458	1.77
4722	*TSTD3*	4.9275	0.74561	-0.37686	-0.60801	1.77
4723	*PDE6C*	3.6387	1.2825	0.37471	0.077067	1.77
4724	*C5orf22*	2.1897	1.8071	1.2991	-0.15263	1.77
4725	*GAGE2A*	4.6054	0.45567	0.23473	-0.4482	1.77
4726	*KIAA1841*	2.7691	1.8296	0.69676	-0.00832	1.77
4727	*MMP27*	3.2901	2.2073	-0.20239	-2.0837	1.77
4728	*SLFN11*	4.8674	0.94571	-0.51868	-1.2832	1.76
4729	*LRRC16A*	3.8262	1.3322	0.13578	-1.1886	1.76
4730	*SERPINB1*	5.1471	0.52306	-0.37619	-0.96144	1.76
4731	*UCHL1*	3.6231	1.9113	-0.24058	-0.60717	1.76

4732	*TPD52L3*	4.6672	1.1476	-0.52122	-1.3875	1.76
4733	*GUCA2B*	3.2922	2.5215	-0.52017	-1.9746	1.76
4734	*SRCIN1*	2.1866	1.7811	1.3258	-1.0898	1.76
4735	*OR52R1*	3.0336	1.7357	0.52383	-0.20952	1.76
4736	*NYAP1*	4.9572	0.84886	-0.51304	-2.4286	1.76
4737	*GPR158*	3.2054	1.8158	0.27146	-0.15394	1.76
4738	*ACOT2*	4.6245	0.74656	-0.07845	-1.2194	1.76
4739	*CNN3*	2.8212	2.23	0.24124	-0.37253	1.76
4740	*DCSTAMP*	3.2446	1.9753	0.07022	-1.4968	1.76
4741	*WDR59*	4.6996	0.60079	-0.01081	-0.74242	1.76
4742	*NEK8*	4.1151	2.2388	-1.0645	-1.4374	1.76
4743	*PIWIL3*	2.7189	1.7355	0.83445	-0.11953	1.76
4744	*SMAD9*	4.5083	1.6604	-0.88008	-1.5741	1.76
4745	*POR*	2.8943	2.0838	0.31033	-0.58295	1.76
4746	*SLC2A6*	3.4066	1.1242	0.75751	0.34657	1.76
4747	*LRRC14B*	4.3015	1.2268	-0.24035	-0.38796	1.76
4748	*BAG3*	3.5915	2.392	-0.69758	-2.0629	1.76
4749	*MBOAT7*	3.0042	1.7866	0.49511	0.048132	1.76
4750	*GLTSCR2*	3.7629	2.505	-0.98239	-2.329	1.76
4751	*ITGB3BP*	5.0738	0.30621	-0.0952	-0.35038	1.76
4752	*ERVV-2*	4.2321	1.3337	-0.28308	-0.35232	1.76
4753	*NPIPB9*	2.3887	1.7013	1.1917	-2.4709	1.76
4754	*ATP8A1*	4.3616	0.88577	0.03422	-1.2954	1.76
4755	*PRSS46*	3.0659	1.2284	0.98694	-0.2098	1.76
4756	*UTP14C*	4.6286	1.355	-0.70255	-1.0503	1.76
4757	*ABCF3*	4.0702	1.5472	-0.33645	-0.88418	1.76
4758	*GFY*	3.8109	1.8665	-0.39676	-1.776	1.76
4759	*LIPN*	3.9847	1.6117	-0.31641	-1.4561	1.76
4760	*TCTA*	4.5894	1.0632	-0.37323	-1.7647	1.76
4761	*HIAT1*	2.8825	1.2703	1.1265	1.0351	1.76
4762	*ZSCAN2*	4.0413	0.63118	0.60573	-1.5458	1.76
4763	*SCN4A*	2.8867	2.3169	0.07454	-0.2562	1.76
4764	*RAB3B*	3.0973	2.0168	0.16383	-0.89699	1.76
4765	*C3orf33*	4.957	2.013	-1.6926	-2.163	1.76
4766	*SETSIP*	5.5839	0.030302	-0.33741	-2.033	1.76
4767	*ZBED8*	3.0456	2.6135	-0.38231	-0.67243	1.76
4768	*TRIM61*	2.4249	2.3289	0.52247	-0.53689	1.76
4769	*IWS1*	3.6619	1.6226	-0.00829	-1.1287	1.76
4770	*LGR4*	3.9193	1.2217	0.13507	-0.24508	1.76
4771	*PPAN*	4.1652	0.64045	0.46987	-1.8371	1.76
4772	*FAM89A*	2.8245	2.1036	0.34664	-1.3727	1.76
4773	*WDR48*	3.5966	1.4721	0.20547	-1.0564	1.76
4774	*LYPLAL1*	2.9246	2.1361	0.21312	-0.63575	1.76
4775	*C6orf52*	3.7643	1.0862	0.4229	-1.2208	1.76
4776	*CCDC152*	2.7755	2.3877	0.10996	-0.77114	1.76
4777	*STK39*	4.0412	1.1434	0.08797	-0.22313	1.76

4778	*CCNB3*	3.5079	1.1289	0.63569	0.3281	1.76
4779	*OR13D1*	3.2955	1.401	0.57567	-0.22251	1.76
4780	*STIM2*	3.5178	1.0588	0.69473	-1.1534	1.76
4781	*LDB1*	5.9458	-0.21491	-0.45972	-2.1125	1.76
4782	*LCN6*	3.6752	0.80054	0.79535	-0.92284	1.76
4783	*RD3L*	4.814	0.29413	0.16285	-0.55586	1.76
4784	*AMY2B*	4.179	1.1842	-0.09268	-1.6524	1.76
4785	*IQGAP2*	4.4879	0.50788	0.27474	-0.00303	1.76
4786	*IP6K3*	3.7008	1.8067	-0.23758	-1.5667	1.76
4787	*NRP1*	2.1671	2.1016	0.99931	-0.60347	1.76
4788	*CASP3*	3.8618	0.72204	0.68303	-2.0146	1.76
4789	*ZBTB41*	4.0576	1.0265	0.1827	-0.58404	1.76
4790	*OR6F1*	2.6157	2.4631	0.18707	-0.30574	1.76
4791	*GLTPD2*	3.6805	2.4859	-0.90207	-1.2188	1.75
4792	*KCTD18*	2.6846	1.7562	0.82297	0.62749	1.75
4793	*ZFYVE19*	2.8452	2.498	-0.07952	-1.8453	1.75
4794	*HEY1*	2.7415	1.3376	1.1837	-1.0359	1.75
4795	*WARS2*	3.7919	1.3916	0.07906	-0.57967	1.75
4796	*ADAP1*	4.0635	2.6477	-1.4493	-1.4842	1.75
4797	*ANK1*	3.4987	2.8754	-1.1137	-1.359	1.75
4798	*CCDC69*	2.5173	1.6059	1.1367	-0.96647	1.75
4799	*C1orf123*	3.7305	1.4954	0.03357	-2.1802	1.75
4800	*MRPS15*	5.2488	0.13368	-0.12425	-1.3287	1.75
4801	*NINJ2*	2.8577	1.2976	1.1029	-0.86756	1.75
4802	*PTCHD3*	4.2426	2.045	-1.0303	-1.9935	1.75
4803	*WDR26*	3.8237	0.78888	0.64447	-1.2863	1.75
4804	*FAM71F1*	2.5816	2.2349	0.44052	-0.5271	1.75
4805	*REEP6*	2.844	2.24	0.17212	-1.5121	1.75
4806	*FAM24A*	3.0812	1.185	0.98936	-0.52154	1.75
4807	*PRRT2*	4.7738	0.97445	-0.49311	-1.6525	1.75
4808	*CEP41*	4.7796	1.334	-0.85871	-0.98842	1.75
4809	*TAAR9*	2.7077	2.5943	-0.04775	-1.1838	1.75
4810	*GUCA2A*	3.0579	1.8994	0.29636	-0.71063	1.75
4811	*SDHAF1*	3.1443	2.3828	-0.27359	-1.8179	1.75
4812	*CACNA1S*	5.4295	-0.04425	-0.1318	-0.45867	1.75
4813	*ATOH7*	3.7812	1.6744	-0.20246	-1.7024	1.75
4814	*STX7*	2.8616	2.2291	0.16232	0.15451	1.75
4815	*ZNF696*	3.1538	1.577	0.52198	-0.34266	1.75
4816	*TGIF2LX*	4.0672	1.1182	0.06701	-0.98907	1.75
4817	*GATSL2*	4.4018	1.6158	-0.76529	-1.4404	1.75
4818	*CNR1*	3.6052	1.9116	-0.26468	-0.58603	1.75
4819	*NES*	5.7279	0.04489	-0.52154	-0.97138	1.75
4820	*GRIK2*	3.3464	2.5725	-0.66767	-1.8253	1.75
4821	*OR52B2*	2.527	1.7249	0.99825	-0.43473	1.75
4822	*CCR10*	3.0011	1.3174	0.93087	-0.23297	1.75
4823	*MEA1*	5.2091	0.12358	-0.08446	-1.0301	1.75

4824	*NME2*	4.4033	2.445	-1.6011	-2.4595	1.75
4825	*CRYBG3*	2.9335	2.7671	-0.45403	-1.7967	1.75
4826	*PUS10*	3.7435	2.1266	-0.62423	-1.6181	1.75
4827	*SLC22A7*	3.4023	1.4629	0.38025	-0.07154	1.75
4828	*C14orf2*	3.2266	3.0356	-1.017	-1.6526	1.75
4829	*CHAC1*	4.3931	0.49834	0.35324	-1.6246	1.75
4830	*CBX8*	2.7742	2.5315	-0.06297	-0.29121	1.75
4831	*CLEC3A*	3.36	1.5342	0.34848	0.27664	1.75
4832	*TIFA*	3.5323	1.9803	-0.27044	-1.0743	1.75
4833	*BIK*	3.2908	3.0742	-1.1241	-1.7364	1.75
4834	*ARL14EPL*	4.5704	0.68273	-0.01337	-0.25227	1.75
4835	*SMAD7*	2.9299	2.7533	-0.44353	-1.8227	1.75
4836	*THRA*	2.8779	2.4224	-0.06103	-0.63912	1.75
4837	*INF2*	4.9462	1.2181	-0.92524	-1.3937	1.75
4838	*TMEM87B*	2.7755	2.7438	-0.28047	-0.81136	1.75
4839	*TNFRSF14*	3.7643	0.87333	0.59983	0.35462	1.75
4840	*SERPIND1*	3.089	2.0748	0.07319	-0.25803	1.75
4841	*MIDN*	4.1874	0.67533	0.37426	-1.0999	1.75
4842	*MTHFD1L*	4.087	2.8939	-1.7445	-2.4472	1.75
4843	*MRPS33*	3.8889	1.2333	0.11357	-0.01785	1.75
4844	*ACSL1*	5.1089	1.0691	-0.94312	-1.1805	1.74
4845	*ZBTB22*	2.9288	1.5808	0.72505	-1.1316	1.74
4846	*MAP3K11*	2.6453	2.0723	0.51667	0.43942	1.74
4847	*ABCG2*	4.2347	1.0594	-0.06182	-0.559	1.74
4848	*MYO1F*	5.2218	0.85335	-0.84337	-1.4287	1.74
4849	*CLCN2*	4.7026	1.3605	-0.83184	-2.2996	1.74
4850	*TGOLN2*	3.6173	1.7858	-0.17257	-0.8901	1.74
4851	*DRG1*	3.4987	1.5644	0.16693	-0.46056	1.74
4852	*PRMT9*	3.5642	1.122	0.54379	-0.43146	1.74
4853	*SOST*	2.6967	2.2934	0.23955	-0.62489	1.74
4854	*CDC42EP5*	3.0376	2.2057	-0.01399	-1.3769	1.74
4855	*JADE1*	2.8679	1.2778	1.0807	-1.0948	1.74
4856	*MS4A2*	4.043	0.76415	0.41894	0.071886	1.74
4857	*COX7A2*	3.3115	1.8396	0.07481	-0.52092	1.74
4858	*DNAJB7*	3.5714	1.7332	-0.08008	-1.8085	1.74
4859	*GTF2IRD2B*	2.5144	1.531	1.1788	-1.5538	1.74
4860	*PCDH20*	3.8228	0.96498	0.4358	-1.5765	1.74
4861	*LRRIQ3*	3.0639	2.6198	-0.46017	-2.4011	1.74
4862	*PPFIA3*	4.0714	0.68097	0.47084	-1.4273	1.74
4863	*ZNF30*	3.3267	1.2809	0.61525	-1.3523	1.74
4864	*MED29*	4.6019	1.8301	-1.2098	-1.5684	1.74
4865	*FAM122B*	3.606	2.0918	-0.4759	-0.50577	1.74
4866	*TRO*	3.07	1.2052	0.94444	-2.2064	1.74
4867	*NANP*	4.0934	1.5519	-0.42573	-1.1343	1.74
4868	*MKI67*	2.2543	1.8239	1.1407	-1.7215	1.74
4869	*ZNF48*	2.7027	1.7835	0.73238	-1.3948	1.74

4870	*BRAT1*	3.8551	2.8307	-1.4685	-1.4864	1.74
4871	*MMP12*	3.8365	1.6376	-0.25737	-0.30045	1.74
4872	*SPATA45*	3.8272	2.329	-0.9428	-2.1182	1.74
4873	*BLVRA*	4.2205	0.66027	0.33167	0.076188	1.74
4874	*C11orf30*	5.5017	0.17607	-0.46721	-0.8417	1.74
4875	*BNC1*	2.8332	1.8788	0.49816	-1.2065	1.74
4876	*ZNF398*	3.7666	1.6272	-0.18432	-0.42171	1.74
4877	*TCTE1*	3.5729	1.373	0.26246	-0.04618	1.74
4878	*IL3*	4.0321	0.631	0.54446	-0.37546	1.74
4879	*ZNF583*	2.7377	1.4344	1.0342	0.23549	1.74
4880	*UBE2O*	3.3307	1.516	0.35833	-0.98062	1.74
4881	*CIB2*	3.8058	2.3279	-0.93099	-2.2669	1.73
4882	*TBC1D9*	3.9834	1.5919	-0.37261	-0.95796	1.73
4883	*GRIA4*	4.7638	1.7075	-1.2694	-2.0536	1.73
4884	*ZC3H13*	3.7965	1.7757	-0.37052	-1.3247	1.73
4885	*CCNG1*	3.8845	0.7079	0.60902	-1.1866	1.73
4886	*GCM1*	3.5658	2.5248	-0.8897	-1.9513	1.73
4887	*RHOJ*	2.2013	2.0868	0.91252	-1.2852	1.73
4888	*COPS7B*	4.4129	0.48698	0.30057	-1.5368	1.73
4889	*CDKL5*	3.1576	1.853	0.18983	-0.28355	1.73
4890	*MMAA*	2.4973	1.6528	1.05	-0.32254	1.73
4891	*SEMA3G*	2.7984	2.5244	-0.12326	-0.47818	1.73
4892	*SLC30A5*	4.7201	1.0906	-0.61164	-1.1138	1.73
4893	*MPP3*	3.4365	1.3685	0.39364	-0.47083	1.73
4894	*LILRB2*	4.1145	2.4001	-1.316	-1.9227	1.73
4895	*MXRA7*	2.8021	1.8708	0.52528	-0.82628	1.73
4896	*PML*	2.3997	1.7608	1.0368	-0.36841	1.73
4897	*C11orf16*	4.3379	1.1288	-0.26984	-0.37787	1.73
4898	*MAP2K3*	4.2737	1.3671	-0.44511	-0.69865	1.73
4899	*FOXJ3*	3.0064	2.4633	-0.27443	-0.53843	1.73
4900	*CST3*	3.3733	1.2264	0.59549	-0.03316	1.73
4901	*LZTR1*	3.644	1.9578	-0.40671	-2.2794	1.73
4902	*IDE*	4.2255	0.57882	0.39043	-0.5293	1.73
4903	*NDC1*	2.9352	1.6468	0.61214	-1.5053	1.73
4904	*EVI5*	2.8496	2.2017	0.14196	-1.1008	1.73
4905	*GUCY1B3*	3.5176	1.7388	-0.06418	-1.6061	1.73
4906	*PPEF2*	5.5018	-0.1549	-0.15499	-0.95933	1.73
4907	*IER5L*	3.0544	2.018	0.11924	0.015043	1.73
4908	*RIMKLB*	2.6272	1.9865	0.57708	0.46392	1.73
4909	*CYP2W1*	2.4351	2.1003	0.65475	-1.853	1.73
4910	*POU3F4*	3.5072	0.84072	0.84049	-0.70438	1.73
4911	*GLI4*	3.1637	2.2346	-0.21049	-1.4504	1.73
4912	*GPR55*	3.3584	2.5344	-0.70513	-0.91685	1.73
4913	*KLHDC1*	4.0103	0.61689	0.5598	0.32083	1.73
4914	*GSDMC*	4.179	0.77651	0.23049	-0.51585	1.73
4915	*PLSCR4*	4.8281	0.80931	-0.45185	-1.4128	1.73

4916	*ZNF454*	2.9739	1.5553	0.65612	-0.04111	1.73
4917	*ZBTB24*	5.046	0.61874	-0.47964	-0.97164	1.73
4918	*UBALD2*	2.3624	2.2384	0.58319	0.3	1.73
4919	*PRR5*	3.5772	1.3897	0.21695	-1.0733	1.73
4920	*SKAP2*	3.3815	1.9794	-0.17712	-1.0443	1.73
4921	*KRT81*	5.0999	0.11164	-0.03036	-2.0706	1.73
4922	*PPP1R9A*	2.6128	2.2087	0.35931	-0.72529	1.73
4923	*CHST13*	2.5437	1.6864	0.9505	-0.68879	1.73
4924	*ANKDD1B*	2.8177	2.6011	-0.23916	-1.2719	1.73
4925	*CAMSAP3*	5.3852	0.98986	-1.197	-1.2932	1.73
4926	*KRT31*	3.9983	0.97324	0.20649	-1.9358	1.73
4927	*ELAVL2*	4.839	0.18778	0.15064	-1.049	1.73
4928	*ESRP1*	3.866	0.96444	0.34693	0.32682	1.73
4929	*AMIGO3*	3.9176	2.2121	-0.95322	-1.4491	1.73
4930	*FOXS1*	2.6513	2.2555	0.26956	-1.3959	1.73
4931	*KRTAP19-5*	3.847	0.72204	0.60721	-0.96946	1.73
4932	*OR6B1*	4.2375	0.67486	0.2638	-0.68303	1.73
4933	*PRKAG2*	2.5727	1.5542	1.049	-0.3282	1.73
4934	*CACFD1*	4.3269	1.001	-0.15212	-2.5302	1.73
4935	*FLNB*	4.1101	2.7549	-1.6893	-2.1282	1.73
4936	*HIP1R*	4.0042	1.5834	-0.4119	-0.67608	1.73
4937	*ATG101*	2.3839	1.7118	1.079	1.0335	1.72
4938	*ZNF607*	4.7273	0.23285	0.21428	-1.2211	1.72
4939	*PAMR1*	2.5601	1.5631	1.0502	0.52584	1.72
4940	*ATP1A3*	3.4185	0.98936	0.76532	-0.7329	1.72
4941	*GTF2A2*	4.303	1.0092	-0.13928	-1.3245	1.72
4942	*AGPAT4*	3.3079	1.6362	0.22797	-1.5204	1.72
4943	*C1QL3*	3.9727	0.64417	0.55491	0.21274	1.72
4944	*CLN5*	3.5588	1.2111	0.40041	0.088149	1.72
4945	*VSTM2L*	4.4272	1.1822	-0.44182	-1.4333	1.72
4946	*RMCX5-GPRASP2*	3.1143	2.1399	-0.08676	-0.41575	1.72
4947	*LINGO4*	3.3505	2.455	-0.63834	-1.3218	1.72
4948	*CABS1*	3.1539	1.134	0.87917	-1.153	1.72
4949	*NUDT16L1*	4.1359	1.7143	-0.68409	-1.8388	1.72
4950	*ATP5C1*	4.1703	1.1646	-0.17208	-1.4683	1.72
4951	*MRPL42*	3.624	2.6464	-1.1096	-2.4496	1.72
4952	*NEGR1*	3.3571	0.98637	0.8165	0.051389	1.72
4953	*USP17L21*	2.8752	1.9762	0.30826	-0.8604	1.72
4954	*ZNF283*	4.9073	0.15449	0.0973	-0.87198	1.72
4955	*CEACAM16*	2.8548	1.5082	0.79603	-1.8856	1.72
4956	*CTDSP1*	2.3121	1.6641	1.1825	-0.24817	1.72
4957	*AUTS2*	6.0977	1.2542	-2.1947	-2.3101	1.72
4958	*ZNF81*	3.0664	1.6546	0.43613	-0.05863	1.72
4959	*KRTAP1-4*	4.7906	0.21127	0.15516	-1.714	1.72
4960	*HIST1H2AK*	3.2776	1.5818	0.29707	-1.1228	1.72
4961	*PARD3B*	2.8389	1.8515	0.46366	-1.0005	1.72

4962	*RFXANK*	2.3495	1.6989	1.1048	-0.62307	1.72
4963	*OPCML*	4.8163	0.94144	-0.60465	-2.2947	1.72
4964	*OR1B1*	5.3281	-0.05911	-0.11621	-1.3613	1.72
4965	*SLAIN2*	4.2275	0.71562	0.20951	-1.2887	1.72
4966	*MCPH1*	1.9285	1.855	1.368	-0.25459	1.72
4967	*SHC4*	3.3809	0.93664	0.83301	-1.0288	1.72
4968	*HCRT*	4.2207	0.50033	0.42915	-0.48572	1.72
4969	*TXLNA*	5.2496	-0.04134	-0.05851	-0.35722	1.72
4970	*LTBP2*	3.901	0.6905	0.55745	-0.55002	1.72
4971	*TMCO5A*	2.5316	2.3065	0.3095	-0.06351	1.72
4972	*ADAMTS5*	3.732	0.75092	0.66416	-1.1943	1.72
4973	*SFT2D2*	3.0408	2.3073	-0.20166	-1.2842	1.72
4974	*ASCC1*	3.8952	1.7013	-0.4521	-0.66569	1.71
4975	*SRRM4*	4.2094	1.9464	-1.0125	-1.3627	1.71
4976	*IFNA21*	2.671	2.2747	0.19757	-0.68169	1.71
4977	*MAEA*	3.053	1.7298	0.3597	0.30022	1.71
4978	*VWA8*	4.5506	1.6516	-1.06	-1.7039	1.71
4979	*POT1*	4.1157	1.2822	-0.25708	-1.234	1.71
4980	*HNF4G*	4.3825	0.57604	0.18138	-1.4452	1.71
4981	*PRKCH*	2.6798	2.3153	0.14384	-0.18395	1.71
4982	*PRKCD*	3.613	0.93194	0.59379	-1.5436	1.71
4983	*GPR18*	4.9998	0.35756	-0.2192	-0.25971	1.71
4984	*ANP32C*	2.3943	1.6491	1.0945	0.82914	1.71
4985	*BMP8B*	3.371	1.7803	-0.01383	-0.94121	1.71
4986	*KCTD19*	3.9902	2.1637	-1.0172	-1.942	1.71
4987	*TP53INP1*	3.4712	1.2869	0.37811	-0.9279	1.71
4988	*ARHGAP30*	2.0673	1.665	1.4033	0.095265	1.71
4989	*C1orf189*	4.0504	1.1529	-0.0677	-1.5673	1.71
4990	*RSL24D1*	4.63	1.0827	-0.57713	-2.7406	1.71
4991	*BET1*	3.5198	2.8224	-1.2067	-2.1987	1.71
4992	*CLYBL*	3.9477	1.7716	-0.58451	-1.9036	1.71
4993	*DNAJB6*	2.7894	2.3983	-0.05341	-1.2098	1.71
4994	*NUDT22*	3.4945	1.4034	0.23609	-0.50961	1.71
4995	*VAMP7*	3.6327	1.4941	0.0069	-1.4998	1.71
4996	*UCMA*	2.4005	2.3605	0.37192	0.30283	1.71
4997	*DMGDH*	2.8109	2.5169	-0.19494	-1.3664	1.71
4998	*AP4E1*	4.1009	1.7042	-0.67342	-1.3533	1.71
4999	*ANXA4*	2.305	1.9998	0.82682	0.35788	1.71
5000	*ZNF614*	4.8436	0.28367	0.00396	-0.66997	1.71
5001	*TMEM244*	2.5012	1.9432	0.68667	-1.4185	1.71
5002	*TNFRSF11A*	2.6142	2.1593	0.35491	-2.1193	1.71
5003	*C7orf76*	2.6019	2.4997	0.02656	-1.9623	1.71
5004	*TBC1D26*	3.9029	0.72659	0.49861	0.2579	1.71
5005	*GPRC5B*	3.0065	2.0377	0.08211	-0.75767	1.71
5006	*CLIC2*	3.0221	1.826	0.2764	-0.87241	1.71
5007	*LSM4*	5.1501	0.18668	-0.21291	-2.0358	1.71

5008	*PPP2R2D*	2.4152	1.5087	1.1988	-0.69787	1.71
5009	*C19orf57*	5.0808	0.28489	-0.24376	-1.0278	1.71
5010	*TGIF2*	2.3068	1.8658	0.94904	-0.31645	1.71
5011	*GFPT2*	3.1665	1.555	0.39963	-0.6399	1.71
5012	*KIF5A*	5.2194	0.54867	-0.64748	-2.5081	1.71
5013	*UBXN2B*	2.7669	1.4713	0.88198	0.50669	1.71
5014	*AP5M1*	5.916	0.92767	-1.7235	-2.2165	1.71
5015	*OR2AG2*	3.5746	1.3054	0.24015	-1.0992	1.71
5016	*MYO3A*	4.9258	0.79371	-0.59961	-1.9287	1.71
5017	*RIMBP2*	2.9462	2.2045	-0.03161	-0.49716	1.71
5018	*RBMXL1*	3.3238	1.2748	0.51984	-0.53554	1.71
5019	*C2orf71*	2.6315	2.4505	0.03565	-2.3602	1.71
5020	*GSKIP*	1.9952	1.5905	1.5308	0.56247	1.71
5021	*PPP1R1B*	3.2609	1.8935	-0.03826	-0.59225	1.71
5022	*PRTG*	2.4079	1.8499	0.85789	0.84112	1.71
5023	*ZBTB7C*	3.8786	1.3313	-0.09431	-0.26429	1.71
5024	*PCDHGA9*	4.0705	0.79987	0.24514	-1.4701	1.71
5025	*CCZ1*	2.4619	1.4537	1.1995	-0.46064	1.71
5026	*ADAM12*	3.1527	1.9914	-0.02915	-1.5684	1.70
5027	*SPRY2*	4.0802	1.2691	-0.23481	-1.4852	1.70
5028	*OR1J4*	2.6288	1.6791	0.80532	-2.0465	1.70
5029	*EXOC5*	5.7029	0.025017	-0.61555	-2.7384	1.70
5030	*APOBEC2*	3.7228	2.6565	-1.2673	-1.4317	1.70
5031	*SUB1*	4.2387	0.80653	0.06592	-0.83243	1.70
5032	*RASL12*	2.434	2.3328	0.34431	-0.60465	1.70
5033	*EPN3*	5.0785	1.1417	-1.1098	-1.1793	1.70
5034	*KRT35*	3.3572	1.7662	-0.01327	-1.175	1.70
5035	*ZNF177*	4.6977	0.56594	-0.15366	-1.1713	1.70
5036	*MARC1*	3.9074	1.5853	-0.38319	-0.44353	1.70
5037	*TRERF1*	3.5526	1.4116	0.14451	-0.18353	1.70
5038	*OR9K2*	3.6261	1.7667	-0.28427	-0.44353	1.70
5039	*PIGS*	3.0103	1.2275	0.87036	-1.9623	1.70
5040	*SNX10*	3.995	0.63803	0.47491	-1.5651	1.70
5041	*CDCA7*	3.4038	3.2703	-1.5663	-1.6208	1.70
5042	*TRIM16*	2.4894	1.6013	1.0162	-0.74086	1.70
5043	*SLC4A4*	4.4587	0.60104	0.04714	-1.403	1.70
5044	*RAP1A*	2.9578	2.5939	-0.44499	-0.69319	1.70
5045	*ZNF576*	3.8097	2.1345	-0.83784	-2.1477	1.70
5046	*C11orf88*	2.1018	2.028	0.97636	-0.09802	1.70
5047	*RAET1E*	2.2456	1.4706	1.3884	-1.7655	1.70
5048	*TLN2*	4.1487	1.0795	-0.12425	-1.9358	1.70
5049	*TNFSF4*	2.2151	1.7877	1.1008	-1.9271	1.70
5050	*SERPINB9*	3.3799	1.411	0.31268	-0.78398	1.70
5051	*CLSPN*	2.3618	2.3202	0.42143	-0.52655	1.70
5052	*KBTBD6*	2.8753	2.5822	-0.35548	-0.69155	1.70
5053	*SCAF8*	2.7634	2.1415	0.19702	-2.0545	1.70

5054	*TMED9*	2.8903	1.2102	0.99834	-3.0575	1.70
5055	*INPP5B*	5.2341	0.88301	-1.0197	-1.8508	1.70
5056	*LOC101929372*	4.5271	1.0109	-0.44134	-1.3696	1.70
5057	*H1FX*	2.4439	1.5161	1.1365	0.33706	1.70
5058	*RB1*	2.0388	2.0099	1.0469	-0.92114	1.70
5059	*RNASE6*	2.8962	1.2001	0.99893	0.16118	1.70
5060	*IFNE*	2.5371	2.0736	0.484	-0.23053	1.70
5061	*ZNF69*	4.69	0.79841	-0.39416	-0.53931	1.70
5062	*TVP23C-CDRT4*	4.1571	0.90838	0.02859	-1.6885	1.70
5063	*SYCE1L*	3.4975	1.2591	0.33736	-0.6536	1.70
5064	*IZUMO4*	2.0452	1.5416	1.5066	-0.77114	1.70
5065	*CCL20*	4.5672	0.85457	-0.32906	-0.79035	1.70
5066	*A1CF*	2.9512	2.3032	-0.16207	-1.9163	1.70
5067	*DMWD*	4.0128	0.84586	0.23259	0.14819	1.70
5068	*YBX2*	4.1267	1.1521	-0.18822	-1.1977	1.70
5069	*COX14*	4.718	1.0828	-0.71041	-1.6765	1.70
5070	*CCL27*	3.272	2.6366	-0.81873	-3.4039	1.70
5071	*LMX1A*	3.9717	1.0413	0.07633	-1.165	1.70
5072	*MOAP1*	4.9964	0.73578	-0.64334	-2.3357	1.70
5073	*C1orf101*	3.5477	3.1471	-1.6072	-1.8269	1.70
5074	*SCGB3A1*	3.794	2.6228	-1.3292	-1.8582	1.70
5075	*PAGR1*	2.9158	2.1823	-0.01264	-0.96093	1.70
5076	*DEFB115*	3.305	1.4552	0.32509	-1.2411	1.70
5077	*GZMK*	3.5727	1.4661	0.04435	-0.44236	1.69
5078	*IFT172*	4.1214	0.79121	0.17014	-1.0485	1.69
5079	*CLASRP*	4.1158	1.223	-0.25618	-1.3892	1.69
5080	*MYH3*	3.1659	1.4715	0.44515	0.32083	1.69
5081	*NME8*	2.4718	1.3338	1.276	-1.384	1.69
5082	*CD180*	3.587	1.8213	-0.32699	-0.51799	1.69
5083	*C1QTNF9B*	2.5888	1.7175	0.77289	0.39232	1.69
5084	*TMEM74*	3.3264	1.999	-0.2469	-1.0798	1.69
5085	*HK2*	3.4621	0.91486	0.7013	-1.3057	1.69
5086	*TMEM129*	3.272	1.672	0.1342	-0.15422	1.69
5087	*SMPD1*	3.1662	1.7429	0.16888	-2.2483	1.69
5088	*JADE3*	3.9977	2.2676	-1.1897	-1.2739	1.69
5089	*RIPPLY3*	4.0304	0.80889	0.23624	0.13675	1.69
5090	*CCDC148*	3.8082	0.70236	0.5635	0.41384	1.69
5091	*CXCL13*	3.8378	1.9883	-0.75338	-1.7757	1.69
5092	*GSTT2B*	3.4302	1.4502	0.19155	-1.1923	1.69
5093	*MTG2*	3.6695	1.768	-0.36566	-0.98769	1.69
5094	*DMRT2*	4.9425	0.69628	-0.57042	-1.2699	1.69
5095	*ADPGK*	4.2151	0.65762	0.19522	-1.5588	1.69
5096	*DNAH2*	4.7075	0.19443	0.16545	-0.19735	1.69
5097	*FAM86B2*	3.5713	0.79316	0.7024	-2.2528	1.69
5098	*ZDHHC14*	3.5058	3.0255	-1.4652	-2.4784	1.69
5099	*USP1*	3.3158	1.0494	0.7007	-3.4683	1.69

5100	*PIWIL2*	3.4987	1.7776	-0.2106	-2.1926	1.69
5101	*NKX1-2*	4.3921	1.5404	-0.86725	-1.7865	1.69
5102	*HNRNPLL*	2.7215	1.6637	0.6799	-0.11784	1.69
5103	*API5*	3.5535	1.4015	0.10969	-0.88492	1.69
5104	*GOLGB1*	4.4959	0.31183	0.25687	-0.77778	1.69
5105	*RIPK1*	2.2154	1.5922	1.2568	0.91805	1.69
5106	*TRNAU1AP*	2.7899	1.8527	0.42156	-0.88898	1.69
5107	*DCAF7*	2.3355	2.0709	0.65691	0.038126	1.69
5108	*MAGEC3*	3.1525	2.2777	-0.36761	-0.60065	1.69
5109	*CHMP4C*	3.4685	1.6395	-0.04542	-1.3565	1.69
5110	*PFN4*	2.4943	1.8352	0.73229	-0.12228	1.69
5111	*IFT22*	3.6378	2.0218	-0.59816	-0.73708	1.69
5112	*TCF25*	3.9559	1.3533	-0.24856	-1.6417	1.69
5113	*STAT1*	3.3366	1.3772	0.34675	-1.855	1.69
5114	*TTL*	2.9501	2.5946	-0.4847	-1.8087	1.69
5115	*TLX2*	6.0123	-0.42708	-0.52552	-2.3615	1.69
5116	*USP24*	2.7655	2.7368	-0.44353	-0.58534	1.69
5117	*MEIOB*	3.4165	1.9216	-0.28052	-0.71753	1.69
5118	*MAN1A2*	2.8354	1.341	0.8804	-1.4529	1.69
5119	*EMC1*	4.1331	1.5729	-0.64943	-1.6791	1.69
5120	*CYB5B*	4.3089	1.553	-0.80545	-1.8136	1.69
5121	*FAM200B*	4.4017	1.6228	-0.96823	-2.1496	1.69
5122	*ADAMTS9*	3.2312	1.1205	0.70325	-0.43082	1.68
5123	*RNF207*	3.5404	1.1336	0.38026	-1.3525	1.68
5124	*MAGEB17*	4.2044	0.72679	0.12276	-0.46024	1.68
5125	*UQCC1*	4.0124	1.0299	0.01138	-0.9999	1.68
5126	*ARHGEF16*	4.5853	0.91135	-0.44353	-0.96788	1.68
5127	*LOC283710*	3.8351	1.3301	-0.11212	-2.4596	1.68
5128	*ZNF597*	3.4725	1.9481	-0.36761	-1.3759	1.68
5129	*CDC37L1*	3.1597	1.2844	0.60858	-0.82897	1.68
5130	*FUOM*	4.6996	1.4406	-1.0886	-1.695	1.68
5131	*TANC2*	3.8004	1.4723	-0.22131	-0.9619	1.68
5132	*XXYLT1*	3.9501	1.1378	-0.03675	-1.6174	1.68
5133	*GPR137C*	2.8651	2.0028	0.1832	-0.01401	1.68
5134	*CCZ1B*	3.4823	2.2552	-0.68693	-1.6182	1.68
5135	*GPD1*	4.3455	0.60948	0.09518	-2.1407	1.68
5136	*FAM78B*	4.2587	0.43887	0.35137	-0.87332	1.68
5137	*C10orf54*	4.7004	1.5382	-1.1898	-2.4966	1.68
5138	*GAGE10*	3.9399	3.2506	-2.1419	-2.7546	1.68
5139	*GRIK5*	2.5549	2.0581	0.43394	-0.67536	1.68
5140	*RASSF6*	3.4409	1.7703	-0.16455	-0.93287	1.68
5141	*STS*	2.811	2.6014	-0.36685	-0.44353	1.68
5142	*TNFRSF10B*	3.7649	0.64864	0.63157	0.004564	1.68
5143	*FZD1*	3.0073	1.7916	0.24574	-0.87062	1.68
5144	*UCK1*	4.0179	2.4508	-1.4249	-2.5707	1.68
5145	*MBTPS2*	3.3024	2.871	-1.1303	-2.3017	1.68

5146	*C14orf28*	3.8368	2.3631	-1.1573	-2.1108	1.68
5147	*TCEA2*	4.717	1.0353	-0.71016	-0.8462	1.68
5148	*TSKU*	3.921	1.4539	-0.33312	-2.1085	1.68
5149	*OR5K1*	4.7325	1.8218	-1.5128	-3.1665	1.68
5150	*DTL*	4.4519	1.047	-0.4578	-0.51799	1.68
5151	*HMX3*	3.6203	1.9616	-0.54226	-1.1221	1.68
5152	*FAM157A*	4.6072	2.057	-1.6246	-1.849	1.68
5153	*PEAK1*	4.0339	1.6699	-0.66468	-1.3478	1.68
5154	*OR51B2*	2.9981	1.8661	0.17482	-2.458	1.68
5155	*ATF7IP*	3.2274	1.182	0.62953	0.58609	1.68
5156	*CRK*	3.6053	1.8046	-0.37125	-1.7001	1.68
5157	*LSAMP*	3.6513	1.5307	-0.1438	-0.27669	1.68
5158	*LRRTM2*	2.1133	1.8428	1.0808	-0.86572	1.68
5159	*TRIM59*	2.6345	2.2659	0.13621	-0.67019	1.68
5160	*ZNF776*	2.8055	2.0484	0.1821	0.16107	1.68
5161	*ZSCAN1*	3.9978	1.446	-0.40785	-1.6176	1.68
5162	*ACAA1*	2.0114	1.7505	1.273	-0.74965	1.68
5163	*TEN1*	4.3188	0.52822	0.18787	-0.96529	1.68
5164	*SIMC1*	4.4569	0.93319	-0.35548	-1.5687	1.68
5165	*PCNT*	3.2302	1.7572	0.04687	-1.7299	1.68
5166	*LILRB4*	3.9318	1.1719	-0.07001	-0.77105	1.68
5167	*MELK*	3.5336	2.1321	-0.63214	-1.4089	1.68
5168	*LRRC59*	2.4039	1.9681	0.6608	-0.64556	1.68
5169	*GUCY2F*	4.9749	0.84716	-0.78956	-1.2241	1.68
5170	*COMMD1*	4.9113	1.0473	-0.9263	-1.257	1.68
5171	*VAC14*	4.9507	0.051371	0.02994	-1.2161	1.68
5172	*SYNPO2*	4.026	0.94125	0.06474	-2.1732	1.68
5173	*PTGIS*	3.489	1.2781	0.26487	-2.0187	1.68
5174	*GNPNAT1*	4.6884	0.35631	-0.01275	-0.44353	1.68
5175	*ADRBK1*	2.8207	1.8745	0.33664	-1.5383	1.68
5176	*STX10*	4.2687	1.5778	-0.81509	-1.2616	1.68
5177	*PTGES3L-AARSD1*	3.8953	1.5312	-0.39627	-0.62423	1.68
5178	*ZMYM2*	4.7386	1.6458	-1.3542	-1.6097	1.68
5179	*MICAL2*	4.0781	0.76859	0.18254	0.13956	1.68
5180	*SLC51A*	2.7172	1.496	0.81585	-0.36412	1.68
5181	*RNF150*	4.2178	0.63881	0.17242	-0.40291	1.68
5182	*AKAP9*	2.4357	2.1155	0.47667	-0.91134	1.68
5183	*SIGMAR1*	4.2907	0.49106	0.2448	-0.56423	1.68
5184	*EPN1*	4.3182	0.82008	-0.11216	-0.83856	1.68
5185	*TIMM17B*	3.1381	1.1792	0.70773	-1.1401	1.68
5186	*MATN1*	4.0934	1.2666	-0.33521	-0.40547	1.67
5187	*OSBPL1A*	3.7483	1.2621	0.01424	-1.1084	1.67
5188	*CDK20*	2.8858	2.3084	-0.16971	-2.1187	1.67
5189	*GATC*	4.8897	0.28826	-0.15424	-1.5401	1.67
5190	*STK31*	2.4821	1.387	1.1544	-0.7532	1.67
5191	*MAGEB16*	3.2839	1.1873	0.5522	0.005561	1.67

5192	*NAA16*	3.6569	0.95273	0.41353	-0.65512	1.67
5193	*PHOX2A*	4.0306	0.5347	0.45732	-0.45569	1.67
5194	*ACO2*	3.0794	2.0665	-0.12338	-0.44353	1.67
5195	*ADH7*	3.1933	3.0748	-1.2457	-1.4544	1.67
5196	*CD226*	5.2976	0.1673	-0.44353	-0.69377	1.67
5197	*KRT74*	3.9145	1.912	-0.80558	-1.9844	1.67
5198	*CELA3B*	4.5766	0.24308	0.20113	-1.1761	1.67
5199	*TTPAL*	2.6583	2.0493	0.31087	-1.8227	1.67
5200	*LRRC70*	3.5638	1.5819	-0.12876	-1.0905	1.67
5201	*SLC41A1*	3.1297	1.4378	0.44901	-1.7646	1.67
5202	*LMNTD2*	3.9912	1.2119	-0.18718	-2.2075	1.67
5203	*AKAP5*	3.7759	1.1489	0.09105	-2.0055	1.67
5204	*IQCA1*	2.6109	2.1112	0.29227	-1.2827	1.67
5205	*E2F6*	3.8363	1.6215	-0.44353	-1.4841	1.67
5206	*R3HCC1*	3.7149	0.88074	0.41609	-2.3517	1.67
5207	*ZNF133*	3.3404	1.3279	0.34162	-0.76728	1.67
5208	*NPY*	5.7331	0.39934	-1.1233	-2.2075	1.67
5209	*PIP4K2A*	5.524	0.44134	-0.95645	-1.095	1.67
5210	*OBSCN*	3.8392	0.87812	0.29114	-1.462	1.67
5211	*CMTM7*	3.6918	0.68002	0.63621	-0.91192	1.67
5212	*SCGB3A2*	3.8282	1.7878	-0.60806	-1.813	1.67
5213	*C1QTNF3*	4.7841	0.55579	-0.33198	-1.3385	1.67
5214	*C9orf24*	4.0142	1.7205	-0.72728	-1.9358	1.67
5215	*STON2*	2.9644	1.963	0.07988	-0.66841	1.67
5216	*FBF1*	3.029	2.603	-0.62508	-1.7372	1.67
5217	*MTHFD2L*	2.4202	1.4729	1.1136	-1.2917	1.67
5218	*HMOX1*	2.7619	2.4663	-0.22244	-1.0291	1.67
5219	*B3GALT1*	2.0592	1.9215	1.0246	-1.8348	1.67
5220	*TRIM15*	3.1609	1.7666	0.07695	-0.93116	1.67
5221	*ZNF831*	3.1725	1.1036	0.72814	-0.46153	1.67
5222	*PCLO*	4.1644	0.48305	0.35566	-0.36821	1.67
5223	*REV1*	4.9287	0.17122	-0.09723	-0.8756	1.67
5224	*PROZ*	4.1071	0.83961	0.05569	0.046166	1.67
5225	*COPS7A*	1.9855	1.6403	1.3746	0.85562	1.67
5226	*FCER2*	5.396	0.95208	-1.3492	-1.5524	1.67
5227	*OTOS*	3.4633	1.8101	-0.27501	-0.88278	1.67
5228	*ENSA*	4.6138	1.1484	-0.76389	-2.2879	1.67
5229	*TM4SF19*	2.8637	2.089	0.04515	-1.6748	1.67
5230	*CCDC40*	4.1297	1.1559	-0.28834	-0.68298	1.67
5231	*GUCY1A3*	4.7796	0.12878	0.08854	-0.90382	1.67
5232	*VCPIP1*	4.9958	0.63861	-0.63753	-1.9168	1.67
5233	*KIAA1468*	2.3339	2.2961	0.36684	-1.4681	1.67
5234	*UGT1A7*	3.9024	0.65784	0.43602	0.14156	1.67
5235	*ERMARD*	2.2264	2.0881	0.68176	0.45693	1.67
5236	*ATP13A3*	4.4491	0.34753	0.19879	-0.9472	1.67
5237	*PTTG2*	4.1238	0.51625	0.35534	-0.00827	1.67

5238	*FBXO33*	3.0005	2.3099	-0.31521	-0.98834	1.67
5239	*ITCH*	5.3942	0.25896	-0.6603	-2.0452	1.66
5240	*WDR44*	4.961	0.19103	-0.15992	-1.6836	1.66
5241	*ERCC8*	3.3632	0.92003	0.70849	-0.88775	1.66
5242	*IL17A*	2.7913	2.3056	-0.10524	-0.15965	1.66
5243	*KCNA10*	5.6171	-0.18246	-0.44353	-1.6746	1.66
5244	*NCAPH*	3.1212	2.3009	-0.43279	-1.1496	1.66
5245	*SCAMP1*	2.1751	1.6498	1.1617	-1.8102	1.66
5246	*SEMA3D*	4.4047	0.31635	0.26546	-2.5006	1.66
5247	*PPP1R14C*	3.8992	1.2001	-0.11454	-1.7606	1.66
5248	*C1orf137*	4.4323	0.39367	0.15744	-0.07692	1.66
5249	*C10orf126*	3.7726	1.4598	-0.24951	-1.191	1.66
5250	*NSFL1C*	4.5107	1.1005	-0.62852	-1.2301	1.66
5251	*ARF4*	4.4199	1.6091	-1.0482	-1.6007	1.66
5252	*DHX34*	4.4846	0.46648	0.02965	-0.30611	1.66
5253	*SMURF2*	2.9014	1.1594	0.91961	-1.1223	1.66
5254	*CFAP53*	2.9941	1.2979	0.68831	-0.30609	1.66
5255	*MPEG1*	2.5632	2.4409	-0.02429	-1.1964	1.66
5256	*ARAP2*	2.8057	2.7535	-0.58054	-1.6726	1.66
5257	*C8G*	3.1061	1.6905	0.18187	-2.0989	1.66
5258	*GSTO1*	3.4564	0.86686	0.65506	-1.0289	1.66
5259	*KCNA4*	2.9786	2.3709	-0.37121	-0.51447	1.66
5260	*ATXN7L3*	3.5119	1.8191	-0.35315	-2.9338	1.66
5261	*RNASEH1*	5.1645	0.63502	-0.82337	-3.9666	1.66
5262	*ETV1*	2.6222	1.2731	1.0805	-0.92916	1.66
5263	*CHRNE*	4.1093	1.8827	-1.0168	-2.1077	1.66
5264	*DDX25*	2.5459	1.3594	1.0698	-0.34148	1.66
5265	*SPARC*	3.5335	2.6091	-1.1676	-1.9269	1.66
5266	*GNB4*	2.5958	1.9039	0.47493	-0.45505	1.66
5267	*FOXD4L5*	3.812	1.7978	-0.63551	-1.5352	1.66
5268	*ELP2*	2.0082	1.6476	1.3172	1.2864	1.66
5269	*ZNF426*	4.2987	1.6583	-0.98421	-0.99079	1.66
5270	*VSTM4*	4.2666	0.85396	-0.14828	-0.36914	1.66
5271	*FGF20*	3.1089	2.7999	-0.93726	-1.1328	1.66
5272	*RSU1*	3.6832	1.9816	-0.69407	-1.2097	1.66
5273	*PRR23A*	2.7696	2.4085	-0.20742	-0.35716	1.66
5274	*NDUFB10*	3.9387	1.8035	-0.77155	-1.4959	1.66
5275	*SLC51B*	4.2768	0.79913	-0.10561	-1.761	1.66
5276	*C18orf21*	3.7872	0.74817	0.43386	-0.39186	1.66
5277	*NSUN2*	3.2592	0.98989	0.71905	-0.51219	1.66
5278	*RIBC2*	2.119	1.5876	1.2615	-2.1984	1.66
5279	*SLC45A2*	1.6835	1.6609	1.6229	1.0627	1.66
5280	*CCDC63*	3.2944	2.5437	-0.87128	-2.1859	1.66
5281	*PKIB*	5.8574	0.57373	-1.4644	-2.1177	1.66
5282	*ZNF546*	3.2097	1.1631	0.59297	-0.15649	1.66
5283	*RLN2*	3.2247	1.3698	0.37111	-1.297	1.66

5284	*SLC5A3*	2.9406	1.6664	0.35858	-2.1077	1.66
5285	*IRAK3*	3.2328	0.8924	0.83963	-0.56687	1.65
5286	*LRRC42*	3.7386	0.68772	0.53731	-0.10031	1.65
5287	*MAP3K15*	2.149	1.7853	1.0292	0.98233	1.65
5288	*PBLD*	3.0369	1.4486	0.47742	-0.21739	1.65
5289	*ENTPD3*	4.4432	0.44395	0.07554	-1.191	1.65
5290	*TMEM59L*	2.8488	1.5835	0.53031	-1.4442	1.65
5291	*ZSWIM3*	3.5936	2.5883	-1.2193	-1.7001	1.65
5292	*MIS12*	3.8841	0.76704	0.31087	-1.3253	1.65
5293	*FAM174B*	5.5466	0.92726	-1.5128	-2.7042	1.65
5294	*ITGB4*	2.4709	1.9734	0.51667	0.18682	1.65
5295	*SPRY4*	4.8184	0.11254	0.02998	-1.3416	1.65
5296	*ZCCHC10*	2.0302	1.937	0.99269	-0.31475	1.65
5297	*SORL1*	4.1116	1.1616	-0.31413	-0.56634	1.65
5298	*SOX5*	2.6973	2.6642	-0.40504	-2.508	1.65
5299	*TPO*	2.7133	2.1744	0.06875	-0.86412	1.65
5300	*FAM166B*	2.9454	1.7412	0.26972	-0.08851	1.65
5301	*VTA1*	4.2616	1.6445	-0.9512	-1.6913	1.65
5302	*DDIT4*	2.0863	1.5048	1.3632	1.0835	1.65
5303	*ZNF326*	2.0265	1.9914	0.93595	-0.42232	1.65
5304	*TRIB2*	4.6364	1.3947	-1.078	-1.6469	1.65
5305	*TMEM139*	3.7342	1.2038	0.01376	-0.93329	1.65
5306	*GPS2*	4.2441	1.8607	-1.154	-2.1893	1.65
5307	*GOT1L1*	4.2572	1.2196	-0.52601	-0.60659	1.65
5308	*CTTNBP2NL*	2.0747	1.955	0.91974	-0.36246	1.65
5309	*HAGH*	5.273	0.043791	-0.36761	-1.6456	1.65
5310	*TSPAN9*	3.4875	0.87762	0.58395	-0.98031	1.65
5311	*CDC14B*	2.9135	2.8912	-0.85739	-1.2052	1.65
5312	*PPP1R14B*	2.9978	2.0064	-0.05702	-0.86479	1.65
5313	*RAB43*	2.674	2.5549	-0.28254	-1.4116	1.65
5314	*SLC7A3*	4.478	0.67343	-0.20552	-0.94085	1.65
5315	*LARP6*	2.8233	2.0753	0.04637	-0.83566	1.65
5316	*SYT16*	3.241	2.4813	-0.77877	-0.80061	1.65
5317	*C7orf71*	3.6942	2.4463	-1.1973	-2.0452	1.65
5318	*TCOF1*	3.6968	1.9246	-0.67865	-0.91134	1.65
5319	*INO80C*	3.1721	1.667	0.10289	-0.81533	1.65
5320	*BLCAP*	4.4951	1.7033	-1.2573	-1.7441	1.65
5321	*LENEP*	5.1255	1.1508	-1.3358	-2.2955	1.65
5322	*MVB12A*	2.9686	1.6444	0.32647	-1.9372	1.65
5323	*KRTAP21-3*	1.7295	1.6688	1.5403	-0.18225	1.65
5324	*SLC39A7*	4.2262	1.46	-0.74887	-2.0827	1.65
5325	*SNX8*	4.4767	1.8441	-1.3835	-2.252	1.65
5326	*TFPT*	2.7553	2.3521	-0.1715	-1.0311	1.65
5327	*CSPG5*	3.9372	2.487	-1.4893	-2.1984	1.64
5328	*CFHR4*	3.8564	1.0393	0.03729	-0.40191	1.64
5329	*FAM26E*	3.8505	1.6921	-0.60961	-1.5719	1.64

5330	*C4orf26*	2.2771	1.4717	1.1825	-0.77155	1.64
5331	*TMEM259*	2.9394	2.3247	-0.3349	-0.58479	1.64
5332	*HMX1*	3.9067	1.196	-0.17389	-2.9338	1.64
5333	*TLDC2*	3.2337	1.6138	0.08083	-0.27857	1.64
5334	*DISP1*	3.1101	1.945	-0.12681	-2.7436	1.64
5335	*CCDC23*	4.1263	0.55475	0.24607	0.01812	1.64
5336	*FMO1*	5.0472	0.32726	-0.44742	-1.1566	1.64
5337	*C3orf52*	4.4082	1.0774	-0.55927	-1.0288	1.64
5338	*PRDM4*	3.2031	1.7378	-0.01518	-1.3507	1.64
5339	*ANO5*	2.312	1.3671	1.2453	0.35682	1.64
5340	*PDCD2L*	3.7101	2.0041	-0.79035	-1.27	1.64
5341	*HRAS*	3.3911	2.442	-0.91045	-1.2414	1.64
5342	*FBXO38*	4.0734	0.72361	0.1256	-0.2624	1.64
5343	*SLC5A10*	5.3579	0.076946	-0.51363	-0.94341	1.64
5344	*PLXND1*	3.5004	2.5158	-1.095	-1.8714	1.64
5345	*KLK2*	2.7557	1.228	0.93657	-1.317	1.64
5346	*IRGC*	3.8845	0.97587	0.05929	-0.30061	1.64
5347	*PCDHGA1*	2.8369	1.7162	0.36617	-1.6396	1.64
5348	*HSD3B7*	3.9765	0.98664	-0.04543	-2.1217	1.64
5349	*KRTAP1-1*	4.7279	0.61801	-0.42859	-2.5934	1.64
5350	*AGPAT9*	2.7444	2.0407	0.132	-0.48821	1.64
5351	*ITIH2*	5.2071	1.0551	-1.3457	-1.8157	1.64
5352	*PAPOLA*	4.1424	0.52157	0.25112	-0.28391	1.64
5353	*BCO1*	2.7025	1.2781	0.93423	-2.6705	1.64
5354	*PRRG3*	3.4081	1.0351	0.47083	0.41023	1.64
5355	*HOXB7*	2.4973	1.2478	1.1677	-1.4894	1.64
5356	*STARD5*	2.9609	1.0041	0.94643	-0.12469	1.64
5357	*AXIN2*	2.8493	2.5612	-0.49984	-1.2996	1.64
5358	*EFCAB5*	3.6821	0.75557	0.47254	-0.07935	1.64
5359	*SCN9A*	3.8394	0.91211	0.15829	-0.45786	1.64
5360	*KLRD1*	2.4307	1.8422	0.63621	0.10078	1.64
5361	*FAM204A*	4.0816	1.0148	-0.18762	-1.0919	1.64
5362	*CSPP1*	3.31	1.3493	0.24928	-1.1145	1.64
5363	*PANK1*	2.7577	1.62	0.5297	-1.8272	1.64
5364	*CNIH3*	4.1744	0.38561	0.34697	-0.2301	1.64
5365	*FAM120AOS*	4.2287	0.52879	0.14903	-0.45185	1.64
5366	*GOLGA8M*	4.6052	1.3526	-1.052	-1.8154	1.64
5367	*SPON1*	4.6116	0.45984	-0.16614	-1.8842	1.64
5368	*SHROOM3*	3.3223	1.9645	-0.3817	-1.3264	1.64
5369	*TACR3*	3.1676	1.7584	-0.02092	-0.61836	1.64
5370	*BSDC1*	4.7049	1.5189	-1.3189	-1.6343	1.63
5371	*HOMER3*	3.6391	1.234	0.03166	-1.4233	1.63
5372	*TXNDC9*	4.1306	1.193	-0.4191	-0.63584	1.63
5373	*STX8*	4.5884	0.35623	-0.04036	-1.4191	1.63
5374	*TMIGD1*	2.3058	2.0617	0.5365	-1.3836	1.63
5375	*SLC35B2*	3.6561	0.66185	0.58535	-1.4388	1.63

5376	*FAM63A*	4.8027	0.22302	-0.12278	-2.1312	1.63
5377	*LMO2*	3.4937	2.1888	-0.77963	-0.80876	1.63
5378	*C12orf4*	2.6474	1.2773	0.97774	-1.6335	1.63
5379	*RBBP7*	2.5208	1.6616	0.71939	-0.35441	1.63
5380	*MAGEA9B*	3.1857	1.2047	0.51117	-0.48884	1.63
5381	*SFR1*	2.6034	1.67	0.62805	-1.3507	1.63
5382	*GP1BB*	4.7946	0.46164	-0.35735	-2.8702	1.63
5383	*ELAVL3*	4.0147	1.0977	-0.21405	-0.3364	1.63
5384	*XKR7*	4.7488	0.41393	-0.26468	-0.50507	1.63
5385	*FAM180B*	3.7714	1.8262	-0.70137	-0.8504	1.63
5386	*EYA3*	3.8193	0.8403	0.23578	-0.83912	1.63
5387	*SLC34A1*	4.115	0.95197	-0.17199	-0.42573	1.63
5388	*ZNF727*	2.1102	1.9188	0.86571	0.17659	1.63
5389	*DZANK1*	5.0786	0.65341	-0.83739	-1.4823	1.63
5390	*IGFL1*	2.9163	1.1018	0.87648	-1.9398	1.63
5391	*KRTAP5-11*	3.8469	1.3604	-0.31309	-0.9644	1.63
5392	*OR5AS1*	3.7248	1.6365	-0.46751	-0.77038	1.63
5393	*C3orf22*	2.9528	1.7676	0.17324	-0.0541	1.63
5394	*CLDN23*	3.2126	2.15	-0.47001	-1.911	1.63
5395	*S100A3*	3.8705	2.1571	-1.1353	-1.6119	1.63
5396	*CDYL2*	4.3399	0.81599	-0.26384	-0.91236	1.63
5397	*MARCH5*	4.8085	0.28057	-0.19704	-0.22638	1.63
5398	*ODF3L2*	2.4294	2.1238	0.33869	-1.5588	1.63
5399	*HS3ST5*	3.2908	0.83939	0.76116	-1.0784	1.63
5400	*SOX7*	2.8233	1.1001	0.9677	0.2058	1.63
5401	*HSPB8*	3.3288	1.5518	0.00962	-0.2863	1.63
5402	*HUNK*	1.9706	1.5415	1.3779	1.3244	1.63
5403	*UBE3A*	4.4943	0.97511	-0.58028	-0.84419	1.63
5404	*HIST1H4D*	4.8741	0.67729	-0.66251	-2.3389	1.63
5405	*INSRR*	3.4576	0.8685	0.56252	-0.99832	1.63
5406	*ZNF205*	3.9815	1.3948	-0.48795	-0.72038	1.63
5407	*PRMT6*	2.6941	1.8981	0.29586	-0.16202	1.63
5408	*PYURF*	3.051	2.4518	-0.61494	-0.72553	1.63
5409	*RPA2*	3.8499	0.60364	0.43401	-1.0803	1.63
5410	*GALNT18*	3.9346	0.88982	0.06286	-0.58686	1.63
5411	*CD3E*	3.0883	2.2842	-0.48564	-2.3466	1.63
5412	*ULBP3*	3.6934	1.0828	0.11049	-0.78632	1.63
5413	*KRTAP10-7*	3.4734	2.2751	-0.86182	-1.2051	1.63
5414	*TARP*	2.7514	1.1164	1.0184	0.9593	1.63
5415	*STXBP5*	2.0338	1.5061	1.3457	0.66954	1.63
5416	*EPN2*	4.3049	1.2683	-0.68909	-2.071	1.63
5417	*RBPMS*	3.7072	1.004	0.1727	-2.5935	1.63
5418	*WDR24*	3.7706	1.1745	-0.06144	-0.48263	1.63
5419	*UBN1*	3.9752	1.7875	-0.88008	-2.0113	1.63
5420	*SNCAIP*	3.5933	0.65552	0.63208	0.54744	1.63
5421	*CYP11B2*	3.72	2.4895	-1.3287	-1.8714	1.63

5422	*HCAR1*	4.5122	0.36192	0.00651	-0.55122	1.63
5423	*LOC200726*	2.5619	1.7489	0.56949	-1.864	1.63
5424	*SCN10A*	3.3111	1.7668	-0.20039	-1.6243	1.63
5425	*SELK*	4.1105	0.61339	0.15312	-2.0363	1.63
5426	*KIAA0513*	4.1879	0.77703	-0.08832	-1.3203	1.63
5427	*FAM135A*	3.0674	1.326	0.48301	-0.17208	1.63
5428	*RFPL4A*	3.5581	2.7499	-1.4319	-1.5431	1.63
5429	*C10orf88*	2.2917	1.8658	0.71843	-0.56145	1.63
5430	*TRIM4*	3.8954	0.56999	0.41031	-0.8562	1.63
5431	*ZBTB12*	2.9238	2.8143	-0.86367	-1.4223	1.62
5432	*CRTAC1*	3.4325	2.696	-1.2541	-2.1202	1.62
5433	*SNX6*	4.1719	2.1177	-1.4154	-1.9375	1.62
5434	*TMEM123*	2.8332	2.1111	-0.07051	-0.3703	1.62
5435	*BTNL9*	2.3446	2.1729	0.35415	-0.27146	1.62
5436	*IL16*	3.5755	1.5243	-0.2283	-0.45569	1.62
5437	*PABPC1L2A*	3.0772	0.90799	0.88599	0.44793	1.62
5438	*KCNS3*	3.8438	0.52363	0.50316	-0.92642	1.62
5439	*MAP3K12*	3.9399	0.82297	0.10662	-0.44353	1.62
5440	*MSRB2*	2.6756	1.1881	1.0046	0.62611	1.62
5441	*CBLC*	2.7036	2.3367	-0.17202	-0.59264	1.62
5442	*USP25*	2.9483	1.7089	0.20954	-0.77097	1.62
5443	*RORC*	2.908	1.5904	0.36831	-1.1575	1.62
5444	*RAB4A*	2.9138	1.9337	0.01861	-0.63368	1.62
5445	*ADORA2B*	5.0062	0.27934	-0.42075	-0.44353	1.62
5446	*PLIN3*	3.1716	1.1484	0.54435	-1.7908	1.62
5447	*TMCO1*	4.3951	1.1841	-0.71522	-1.8087	1.62
5448	*IL15*	2.67	1.5619	0.62956	-0.08049	1.62
5449	*TOM1L1*	2.3217	1.3165	1.2227	0.49083	1.62
5450	*EIF1B*	2.5636	1.8361	0.46063	-1.7241	1.62
5451	*WDR78*	3.3493	0.78652	0.72425	-0.9879	1.62
5452	*KCTD1*	2.5743	2.519	-0.23372	-1.7585	1.62
5453	*SWT1*	2.2284	1.483	1.1481	-1.7378	1.62
5454	*CLEC4M*	5.4807	-0.10195	-0.5193	-0.65169	1.62
5455	*BDP1*	3.6981	1.4304	-0.26968	-1.8073	1.62
5456	*APELA*	3.0753	1.3332	0.4491	0.18702	1.62
5457	*TRIM64*	4.1288	0.65641	0.07112	-1.6801	1.62
5458	*GJA4*	3.0767	2.5193	-0.74011	-1.0663	1.62
5459	*ART4*	5.1801	0.70097	-1.0256	-1.3711	1.62
5460	*DCTN3*	4.9087	0.26746	-0.32075	-2.0697	1.62
5461	*SCLY*	3.4885	0.68479	0.68177	0.30034	1.62
5462	*PTK7*	3.719	1.0955	0.04039	-1.8121	1.62
5463	*ASXL1*	4.8617	1.3535	-1.3613	-2.2449	1.62
5464	*GPATCH2*	2.4647	2.4502	-0.06143	-1.111	1.62
5465	*H3F3C*	3.0094	1.1851	0.65884	0.15694	1.62
5466	*TIGD4*	4.8064	0.038871	0.00711	-0.9382	1.62
5467	*ZNF485*	5.0454	0.045942	-0.23922	-0.34906	1.62

5468	*SENP6*	3.1397	0.86236	0.84938	-3.4062	1.62
5469	*REEP1*	4.4312	0.40731	0.01243	-0.95057	1.62
5470	*SERPINB8*	3.9348	0.55526	0.36065	-0.56635	1.62
5471	*ELF4*	2.435	1.7351	0.6798	-1.9874	1.62
5472	*CTLA4*	4.5228	0.90782	-0.58096	-1.5061	1.62
5473	*TMEM56*	2.835	1.514	0.50055	-0.34987	1.62
5474	*LILRA6*	3.4758	1.0956	0.27812	-0.93188	1.62
5475	*SLC35F5*	4.2133	0.53688	0.09832	-1.5153	1.62
5476	*ASTN2*	4.1076	0.87731	-0.13669	-1.4197	1.62
5477	*ABCB5*	2.6766	1.5458	0.62537	-0.0062	1.62
5478	*FGD1*	3.3829	2.1497	-0.68495	-2.0433	1.62
5479	*CXCR3*	3.9729	0.9251	-0.05048	-0.10341	1.62
5480	*OR8G2*	5.3929	0.34909	-0.89474	-1.6526	1.62
5481	*PTH2R*	3.9283	1.6711	-0.75313	-1.6992	1.62
5482	*CXCL17*	2.5277	2.3922	-0.07372	-0.82875	1.62
5483	*ARPP19*	2.7838	2.7711	-0.70984	-1.4996	1.62
5484	*PRKAR1B*	3.5453	2.3562	-1.0567	-1.5302	1.61
5485	*CTXN2*	3.041	1.2373	0.56623	-0.01938	1.61
5486	*OR52J3*	2.4729	1.9915	0.37994	0.186	1.61
5487	*PALD1*	3.8418	1.8303	-0.82833	-0.83278	1.61
5488	*JMJD8*	4.1528	0.40753	0.28287	-1.1169	1.61
5489	*GSTA3*	2.7495	2.48	-0.38714	-0.51164	1.61
5490	*FBXL12*	4.3777	1.3735	-0.90947	-1.2396	1.61
5491	*PSMC1*	4.0265	1.7525	-0.93781	-1.2705	1.61
5492	*RHOU*	2.4143	2.0583	0.36788	-0.78911	1.61
5493	*L3MBTL2*	3.9495	0.63118	0.25913	-1.927	1.61
5494	*CHAC2*	3.3991	1.2977	0.14284	-2.2254	1.61
5495	*TSFM*	4.2314	0.75771	-0.14987	-0.8198	1.61
5496	*PABPN1L*	2.9636	1.0378	0.83702	-0.52896	1.61
5497	*ERBB3*	3.0551	1.1064	0.67638	-2.1648	1.61
5498	*HS6ST2*	4.5492	1.6164	-1.329	-1.9203	1.61
5499	*KRTAP8-1*	3.6903	0.73702	0.40904	-0.63687	1.61
5500	*OR2L8*	3.5093	1.0984	0.22748	-0.43488	1.61
5501	*ZNF92*	2.4866	1.8234	0.52418	0.38037	1.61
5502	*PIGO*	3.2159	1.9637	-0.34601	-0.69935	1.61
5503	*ITGA1*	2.4273	1.4641	0.94137	-2.3795	1.61
5504	*KRTAP19-1*	5.803	0.21321	-1.1845	-1.9431	1.61
5505	*PRPF3*	2.548	1.9703	0.3133	0.051282	1.61
5506	*CREB5*	3.7576	1.0861	-0.01275	-0.68992	1.61
5507	*SLC14A2*	3.8747	0.63198	0.32383	-0.79438	1.61
5508	*YTHDF2*	3.2443	1.214	0.37094	-1.5956	1.61
5509	*REV3L*	3.8382	3.1708	-2.1798	-3.0833	1.61
5510	*CYP24A1*	3.5946	0.64758	0.58689	0.46584	1.61
5511	*TMEM200B*	2.5039	2.2578	0.06698	-0.26949	1.61
5512	*ARHGAP17*	3.4457	1.74	-0.35735	-1.6626	1.61
5513	*POMGNT2*	4.0592	1.4107	-0.64219	-0.88876	1.61

5514	*DMXL1*	4.124	0.52075	0.18284	-1.1084	1.61
5515	*DNAAF1*	2.6756	1.4485	0.7034	-0.41505	1.61
5516	*FOXK1*	4.305	0.92244	-0.40118	-2.1926	1.61
5517	*RAB26*	3.1137	1.6922	0.01981	-0.77018	1.61
5518	*ADCYAP1R1*	4.5423	0.26234	0.02079	-1.2135	1.61
5519	*GPR20*	4.7557	0.19645	-0.12701	-0.4674	1.61
5520	*TRHR*	4.6142	0.16383	0.04651	-0.17629	1.61
5521	*TOMM70A*	4.6407	0.48014	-0.29652	-1.4913	1.61
5522	*SNX13*	4.2164	0.3066	0.3003	-0.85214	1.61
5523	*P4HB*	3.826	2.8311	-1.8341	-2.1956	1.61
5524	*NLRP12*	5.2141	0.1473	-0.53904	-2.0382	1.61
5525	*RBM25*	3.3385	2.0149	-0.53321	-0.84141	1.61
5526	*EXOC6B*	4.1619	0.87025	-0.21267	-1.342	1.61
5527	*SLC25A17*	3.3141	3.1579	-1.6528	-2.4405	1.61
5528	*NCOA3*	3.5869	1.2186	0.01352	-0.23533	1.61
5529	*SCFD1*	3.199	0.97612	0.64372	-1.0406	1.61
5530	*IDI2*	4.8791	0.88301	-0.94333	-2.5156	1.61
5531	*SFXN1*	3.5408	0.64629	0.63081	0.069986	1.61
5532	*CD52*	2.4765	2.1091	0.23173	0.067594	1.61
5533	*MTHFD1*	1.9155	1.6207	1.2811	-1.013	1.61
5534	*MAN1B1*	3.7963	1.036	-0.01599	-0.87396	1.61
5535	*SGCA*	2.8176	2.3486	-0.34996	-1.9878	1.61
5536	*CUL2*	4.0602	0.59341	0.16209	-0.91253	1.61
5537	*USP26*	1.8396	1.8098	1.1661	-0.91283	1.61
5538	*FAM45A*	2.983	1.6725	0.15898	-0.77404	1.60
5539	*PRDM7*	3.5725	2.3228	-1.082	-1.2903	1.60
5540	*TFIP11*	3.7195	1.6909	-0.59804	-0.90588	1.60
5541	*ZNF812*	5.5973	0.342	-1.1271	-1.2125	1.60
5542	*POMK*	4.3523	1.1162	-0.65667	-3.5581	1.60
5543	*DERL2*	4.8403	0.16107	-0.19042	-0.97986	1.60
5544	*SLCO5A1*	4.8281	1.1896	-1.2071	-1.666	1.60
5545	*CD86*	3.993	1.5564	-0.73911	-1.7034	1.60
5546	*PRAMEF2*	3.2315	2.0257	-0.44742	-0.5458	1.60
5547	*CCDC24*	3.1449	2.6605	-0.9968	-3.0219	1.60
5548	*KCNB2*	3.6212	0.67919	0.50728	0.34505	1.60
5549	*FDCSP*	2.6524	1.4998	0.65531	-0.35367	1.60
5550	*GPX5*	2.9253	2.6717	-0.79016	-1.6824	1.60
5551	*MAB21L3*	2.7836	2.2358	-0.21349	-1.0293	1.60
5552	*PARK7*	3.7384	1.2897	-0.22242	-1.7379	1.60
5553	*PIK3R6*	4.5133	0.61486	-0.32262	-0.9858	1.60
5554	*DNAJC27*	4.3276	1.1913	-0.71347	-0.8934	1.60
5555	*PGK2*	3.3415	1.0001	0.46373	-1.6756	1.60
5556	*PRKACB*	2.1187	1.5947	1.0914	0.32079	1.60
5557	*DAB1*	3.2462	1.6617	-0.1037	-0.48995	1.60
5558	*EVA1C*	3.3954	1.8927	-0.48419	-5.7716	1.60
5559	*DHRS7*	4.1734	1.6307	-1.0019	-1.7822	1.60

5560	*TEX9*	2.7018	2.0683	0.03176	-1.2056	1.60
5561	*FAM195B*	4.6085	0.27906	-0.08607	-0.80165	1.60
5562	*BEST1*	5.0306	-0.01496	-0.2146	-1.7364	1.60
5563	*MST1R*	4.0868	0.39834	0.31512	-2.0818	1.60
5564	*TMPRSS7*	3.0339	1.4423	0.32337	-0.63486	1.60
5565	*SLAMF9*	4.3313	0.6579	-0.19136	-0.78823	1.60
5566	*UBAC2*	4.7575	0.61885	-0.57899	-0.7068	1.60
5567	*HRSP12*	3.0664	1.0441	0.68684	-2.7566	1.60
5568	*TTC7A*	3.8738	1.3353	-0.41207	-0.85935	1.60
5569	*PCOLCE*	2.7768	2.3618	-0.34227	-2.0059	1.60
5570	*FNIP1*	3.2564	0.8204	0.71881	-0.19793	1.60
5571	*PDE10A*	3.9121	0.68105	0.20224	-0.37334	1.60
5572	*KCP*	4.5887	0.94611	-0.74086	-2.0952	1.60
5573	*IL18R1*	2.9008	1.7753	0.1177	-1.2845	1.60
5574	*CXADR*	2.5555	2.4634	-0.22673	-0.63753	1.60
5575	*ZNF682*	1.7756	1.5114	1.5049	-0.38937	1.60
5576	*LBHD1*	3.5145	0.97964	0.29658	0.010355	1.60
5577	*TMEM185A*	4.2965	1.0909	-0.59678	-1.237	1.60
5578	*EIF5A2*	3.4486	1.9461	-0.60468	-0.7614	1.60
5579	*ARMC3*	2.6822	1.2793	0.8281	-0.91913	1.60
5580	*IFITM3*	2.9821	2.0519	-0.24512	-0.4537	1.60
5581	*NT5C*	3.2701	1.9632	-0.44464	-1.5608	1.60
5582	*ARR3*	4.0011	1.223	-0.43683	-1.0451	1.60
5583	*LOC100652758*	3.7059	1.2985	-0.21806	-1.1463	1.60
5584	*KDELR2*	4.2961	1.1315	-0.64268	-1.1718	1.59
5585	*CREB3*	4.4271	0.73403	-0.37698	-1.307	1.59
5586	*COL4A6*	2.6278	2.3548	-0.19858	-3.0444	1.59
5587	*MOB2*	2.853	2.2554	-0.32455	-0.3576	1.59
5588	*FANCG*	4.1178	0.7007	-0.03472	-0.37215	1.59
5589	*CH25H*	2.6762	1.129	0.97836	-2.2385	1.59
5590	*WHAMM*	3.8438	0.98837	-0.04931	-0.38667	1.59
5591	*WDR35*	4.6344	0.40132	-0.25291	-0.37656	1.59
5592	*COL2A1*	4.3599	1.2181	-0.79663	-1.5467	1.59
5593	*CEP162*	4.191	0.38929	0.20105	-1.5908	1.59
5594	*PIK3IP1*	3.8358	1.9914	-1.0467	-1.5695	1.59
5595	*ZNRF4*	4.9522	1.4853	-1.6597	-1.8952	1.59
5596	*SNX14*	4.0069	0.42299	0.34629	-2.0548	1.59
5597	*CPT1C*	3.2888	0.99308	0.4923	-1.2985	1.59
5598	*SLC7A7*	4.2676	0.35108	0.1532	-0.90281	1.59
5599	*MRPS18C*	4.0323	0.92431	-0.1849	-1.3074	1.59
5600	*KCNE4*	2.927	1.1221	0.72224	-0.91379	1.59
5601	*B3GAT2*	3.5089	1.1885	0.07384	-1.5039	1.59
5602	*WBP1*	2.8386	1.7095	0.22299	-0.05054	1.59
5603	*RPRML*	4.0354	0.54222	0.19327	0.08196	1.59
5604	*GPRIN2*	3.1722	1.2549	0.34292	-0.0209	1.59
5605	*NKX6-1*	4.5215	0.24025	0.00711	-1.4435	1.59

5606	*TRIOBP*	3.5374	1.1496	0.08176	-1.5667	1.59
5607	*APOC2*	4.2841	0.42022	0.06348	-2.1321	1.59
5608	*TIAM1*	3.39	0.93837	0.43888	-0.18167	1.59
5609	*EHBP1*	3.8152	0.75886	0.19317	-0.78805	1.59
5610	*KIF15*	3.6169	2.5475	-1.3973	-2.8398	1.59
5611	*GOLPH3L*	2.66	1.1341	0.9708	0.8949	1.59
5612	*SIX3*	3.0505	2.491	-0.7769	-1.09	1.59
5613	*TFR2*	2.5503	1.2523	0.96077	-1.9874	1.59
5614	*USP28*	5.1611	-0.18108	-0.21692	-0.71753	1.59
5615	*C1orf146*	5.8607	-0.43276	-0.66583	-0.66948	1.59
5616	*THOC6*	2.7331	1.8187	0.21	-0.34566	1.59
5617	*CRY2*	4.2499	2.1742	-1.6636	-2.4405	1.59
5618	*DFFB*	3.3851	1.2035	0.17047	-1.6084	1.59
5619	*AKR1B15*	3.3244	0.88108	0.55316	-0.04588	1.59
5620	*C1orf204*	4.2716	1.1841	-0.69748	-0.83471	1.59
5621	*PRRX1*	2.9506	1.7632	0.04435	-0.55371	1.59
5622	*OR8A1*	2.3673	1.2227	1.1667	0.35826	1.59
5623	*SPIRE1*	2.2589	1.9581	0.53845	-1.6776	1.59
5624	*GALNT10*	3.0314	1.5637	0.16025	-0.44353	1.59
5625	*ALKBH3*	2.4403	1.5733	0.73928	-0.80743	1.58
5626	*ZNF148*	3.0898	2.2232	-0.56036	-1.5552	1.58
5627	*CYFIP1*	4.9728	-0.03695	-0.18328	-0.92721	1.58
5628	*PHLDA3*	2.9937	1.1829	0.57566	-2.1648	1.58
5629	*DNAJC16*	3.8468	1.409	-0.50357	-1.1479	1.58
5630	*RIPPLY2*	3.5542	0.67683	0.52067	-4.6085	1.58
5631	*AKAP2*	4.1484	0.72209	-0.12131	-0.74804	1.58
5632	*SLC20A2*	2.9973	2.0376	-0.28621	-0.38677	1.58
5633	*NRIP1*	1.8339	1.5479	1.3658	0.45962	1.58
5634	*ABCB4*	3.9191	1.0709	-0.24411	-1.5635	1.58
5635	*PAGE4*	3.4558	1.1775	0.11251	-2.0653	1.58
5636	*STMN2*	2.8389	1.4603	0.44653	-1.4221	1.58
5637	*DEPDC1*	4.0918	0.85849	-0.20669	-1.1321	1.58
5638	*TM4SF1*	5.2448	0.11205	-0.61363	-0.62875	1.58
5639	*ZNF777*	4.2545	0.3784	0.11011	-0.13144	1.58
5640	*ZNF704*	3.6416	1.599	-0.49902	-1.035	1.58
5641	*SNUPN*	3.7568	2.2564	-1.2724	-1.3994	1.58
5642	*OSBPL7*	3.4033	2.2737	-0.93626	-1.2952	1.58
5643	*DACT3*	4.7478	0.077652	-0.08582	-0.39235	1.58
5644	*GABRG1*	3.2554	0.90002	0.58385	-0.19577	1.58
5645	*PRODH2*	2.9326	2.2739	-0.46733	-1.5598	1.58
5646	*CDHR3*	3.011	2.1076	-0.37998	-2.5707	1.58
5647	*FAM124B*	5.3778	-0.2804	-0.36102	-1.6992	1.58
5648	*FRMD6*	3.4317	1.663	-0.3584	-0.68133	1.58
5649	*OTUD1*	4.9909	0.32397	-0.57857	-2.3265	1.58
5650	*YWHAZ*	3.9052	0.62064	0.20989	-0.44353	1.58
5651	*ITIH5*	3.7083	1.0022	0.02461	-1.912	1.58

5652	*C11orf87*	2.1523	2.1213	0.46149	-0.23999	1.58
5653	*GRB7*	3.8732	0.67452	0.18686	-0.23958	1.58
5654	*AKT3*	3.6752	0.54931	0.51	-0.10672	1.58
5655	*PRAMEF19*	2.9159	1.3255	0.49305	-0.36668	1.58
5656	*NFE4*	4.1473	0.39995	0.18664	-1.3946	1.58
5657	*ACSM3*	3.655	0.61017	0.4687	-2.8782	1.58
5658	*SGPP1*	5.1012	-0.02503	-0.34232	-1.9398	1.58
5659	*P2RY6*	3.2	1.5927	-0.0624	-0.92375	1.58
5660	*DISP2*	1.9172	1.5777	1.2346	-0.67842	1.58
5661	*ADCK5*	3.5787	0.88243	0.26813	-0.61989	1.58
5662	*FAM43B*	2.9254	0.99848	0.80493	-1.0737	1.58
5663	*IL2RA*	2.3183	2.1325	0.27751	-1.9175	1.58
5664	*TMEM231*	3.5432	0.74344	0.43942	-2.5065	1.58
5665	*FAM133A*	4.2316	0.92554	-0.43276	-0.49953	1.57
5666	*SMARCD3*	3.1974	0.88221	0.64429	-1.1825	1.57
5667	*IGFBP3*	2.9697	1.0724	0.68104	-0.94498	1.57
5668	*RNF222*	2.58	2.4771	-0.33428	-1.2995	1.57
5669	*SLC12A9*	3.0693	3.0505	-1.3978	-2.1185	1.57
5670	*TPH1*	3.8783	0.46918	0.37429	-0.97381	1.57
5671	*CNKSR2*	3.6608	0.56758	0.49275	-1.1135	1.57
5672	*CADM1*	3.245	1.5116	-0.03588	-0.86242	1.57
5673	*PCDHA3*	4.0288	0.37743	0.3141	-1.438	1.57
5674	*FABP5*	5.2714	0.53813	-1.0898	-1.3184	1.57
5675	*ZFAND3*	1.8356	1.4492	1.4337	-0.66873	1.57
5676	*NAA60*	2.3462	1.9177	0.45389	-1.4747	1.57
5677	*LOC339862*	3.3545	1.0341	0.32908	0.31279	1.57
5678	*BCKDK*	5.5419	-0.17554	-0.64918	-0.76606	1.57
5679	*ZFAND5*	5.17	0.18753	-0.6408	-2.0165	1.57
5680	*C19orf66*	2.9737	0.90081	0.84174	-0.96857	1.57
5681	*TUSC1*	4.1924	1.8576	-1.3338	-1.4767	1.57
5682	*ACTG2*	2.4312	1.4911	0.79352	-0.61595	1.57
5683	*CD69*	2.9909	2.2578	-0.53297	-0.90576	1.57
5684	*CSNK2A3*	4.7985	0.13984	-0.224	-0.8338	1.57
5685	*TFAP2D*	3.6645	1.8886	-0.83891	-1.8789	1.57
5686	*BAX*	2.4996	1.6024	0.61199	-1.1385	1.57
5687	*ENPP5*	4.6734	0.41806	-0.3787	-1.2655	1.57
5688	*OR4N2*	1.8254	1.7669	1.1203	-0.19839	1.57
5689	*GABRP*	2.0832	2.0188	0.61017	-0.066	1.57
5690	*RECQL4*	3.3787	0.94489	0.38708	0.35867	1.57
5691	*LACTB2*	3.9625	0.8258	-0.07799	-0.80498	1.57
5692	*SLC26A6*	2.714	1.5597	0.43635	-1.4469	1.57
5693	*CDH24*	3.5126	2.2573	-1.06	-2.6275	1.57
5694	*HOXD3*	4.5824	0.56115	-0.43473	-0.78951	1.57
5695	*RRAS*	4.5358	0.12602	0.04683	-1.2651	1.57
5696	*TNKS2*	3.325	0.70578	0.67665	-1.5304	1.57
5697	*CFHR1*	4.6473	0.90791	-0.84815	-1.7743	1.57

5698	*ALAS1*	3.9562	1.394	-0.64362	-1.3166	1.57
5699	*TCF20*	4.5312	0.30498	-0.12977	-2.1151	1.57
5700	*VASN*	2.8626	1.7558	0.08672	0.079497	1.57
5701	*PEX10*	4.3956	0.23325	0.07562	-0.82041	1.57
5702	*ZZEF1*	2.406	1.7162	0.58163	-1.0519	1.57
5703	*KIF2A*	3.2884	0.97931	0.43594	-0.64806	1.57
5704	*HPRT1*	4.4131	1.0775	-0.78775	-0.84504	1.57
5705	*OR5B21*	2.6235	1.1271	0.95207	-0.46711	1.57
5706	*MYPOP*	3.3338	1.9253	-0.55655	-1.9287	1.57
5707	*UGT1A5*	2.9974	2.6562	-0.95136	-1.3071	1.57
5708	*TRPA1*	3.405	2.2486	-0.95378	-1.7365	1.57
5709	*NAPEPLD*	3.4002	1.4361	-0.1371	-0.19183	1.57
5710	*GPR6*	3.7772	0.68351	0.23842	-1.7459	1.57
5711	*OGN*	4.4025	0.65697	-0.36045	-0.63687	1.57
5712	*POLDIP3*	4.8851	0.65278	-0.83912	-0.94296	1.57
5713	*CPLX3*	3.2026	1.5814	-0.08582	-0.95658	1.57
5714	*THEM4*	2.7114	2.3568	-0.3703	-0.99182	1.57
5715	*RPP25L*	2.9015	2.5844	-0.78866	-0.968	1.57
5716	*TYW1B*	5.9922	-0.13895	-1.1573	-1.7862	1.57
5717	*TRIM3*	4.6609	0.42052	-0.38553	-1.1487	1.57
5718	*SIGLEC11*	2.3773	2.0182	0.29909	-0.42859	1.56
5719	*ENOX2*	2.4283	2.1606	0.10565	-0.85236	1.56
5720	*IFNA16*	3.7557	1.0308	-0.09223	-1.7507	1.56
5721	*CLDN19*	4.225	0.35648	0.11177	0.049512	1.56
5722	*SLC25A6*	3.8719	0.57734	0.24264	-2.8019	1.56
5723	*MB*	4.9504	0.073858	-0.33241	-1.606	1.56
5724	*KLK5*	4.7044	0.69767	-0.71041	-1.7153	1.56
5725	*IQCD*	3.3624	3.0088	-1.6802	-2.7483	1.56
5726	*HIST2H2AB*	4.7725	0.93187	-1.0149	-1.584	1.56
5727	*DNAI2*	3.0665	1.4736	0.14903	-1.7994	1.56
5728	*FAM212B*	3.041	1.3891	0.25881	-0.22469	1.56
5729	*UBE2D1*	4.3566	0.29986	0.03187	-0.62307	1.56
5730	*LAP3*	2.8129	1.5677	0.30522	-1.1905	1.56
5731	*PITPNM1*	4.4645	1.1653	-0.94399	-1.7906	1.56
5732	*PPP2R2B*	3.5487	0.75424	0.38261	-0.29222	1.56
5733	*POTEI*	4.115	0.46994	0.0985	-1.6649	1.56
5734	*IL15RA*	2.737	1.6313	0.3145	-1.4916	1.56
5735	*ALKBH2*	4.3079	0.25115	0.1237	-0.21429	1.56
5736	*ZNRF3*	2.5546	2.0242	0.10131	-1.1833	1.56
5737	*C3orf67*	4.4927	0.26829	-0.08104	-1.6368	1.56
5738	*HMBS*	3.6448	1.0432	-0.00825	-1.4606	1.56
5739	*B3GALT6*	2.9457	1.3294	0.40444	-1.6263	1.56
5740	*CEMP1*	4.6641	0.34615	-0.33071	-1.4852	1.56
5741	*PRDX5*	3.9247	0.61957	0.13492	0.093787	1.56
5742	*FXYD6*	3.5965	1.159	-0.07689	-0.5047	1.56
5743	*CRIP1*	5.1826	-0.20038	-0.30376	-1.1209	1.56

5744	*TARM1*	3.484	0.68961	0.50476	-0.02553	1.56
5745	*PARD6B*	5.0139	0.048059	-0.38361	-1.6105	1.56
5746	*GPATCH8*	3.04	1.0113	0.62659	-1.1882	1.56
5747	*NBPF12*	3.539	0.76813	0.37051	-1.9573	1.56
5748	*KLHL12*	3.9886	0.56958	0.11931	-2.2745	1.56
5749	*ITGB1*	2.2905	1.635	0.75188	-1.5645	1.56
5750	*CAPN8*	3.7134	1.9345	-0.97066	-1.0945	1.56
5751	*TMEM106B*	3.8375	0.82849	0.01096	-1.5946	1.56
5752	*ARMT1*	3.3367	1.8461	-0.5059	-0.77018	1.56
5753	*REP15*	5.1606	-0.17566	-0.30804	-1.0359	1.56
5754	*OR6N1*	2.7517	1.1461	0.77801	0.47239	1.56
5755	*S100PBP*	3.5311	0.74126	0.40191	0.34241	1.56
5756	*FRMPD3*	3.4713	0.96682	0.23609	-1.2105	1.56
5757	*OR2AT4*	4.4003	0.2658	0.00756	-0.72201	1.56
5758	*DEFB130*	2.5489	2.0915	0.03282	-1.2915	1.56
5759	*CHST11*	3.2995	1.2335	0.13995	-0.3311	1.56
5760	*LRP3*	5.2759	0.35512	-0.95838	-1.6926	1.56
5761	*SLC35D3*	5.071	0.86953	-1.2683	-2.254	1.56
5762	*SLC10A1*	2.2251	1.7583	0.6884	-0.65145	1.56
5763	*BROX*	2.6415	2.4481	-0.41803	-1.095	1.56
5764	*GRM8*	2.7718	2.137	-0.23846	-0.49311	1.56
5765	*KDM4A*	3.0683	1.7353	-0.13328	-0.35744	1.56
5766	*MMGT1*	4.9248	1.7282	-1.985	-2.1058	1.56
5767	*ATF3*	4.0311	0.63571	-0.00066	-1.2657	1.56
5768	*WNT5B*	3.854	0.84309	-0.03116	-0.78941	1.56
5769	*AADAC*	2.7854	1.0559	0.8246	0.088509	1.56
5770	*GRID2IP*	3.0945	0.80412	0.76597	0.2955	1.55
5771	*UPB1*	3.6412	0.80025	0.22188	-0.39689	1.55
5772	*MAK16*	4.001	0.56611	0.09562	-1.5956	1.55
5773	*PIM2*	2.759	1.7913	0.11235	-0.4676	1.55
5774	*ADH5*	4.2177	0.23314	0.21148	-2.6683	1.55
5775	*METTL2A*	4.4535	0.11496	0.0936	-1.6271	1.55
5776	*KATNA1*	3.6915	2.1815	-1.2114	-1.4306	1.55
5777	*ASPG*	3.7347	2.942	-2.0161	-2.5419	1.55
5778	*ADIG*	5.0002	1.239	-1.5788	-2.0842	1.55
5779	*GLS2*	4.7308	0.099866	-0.17081	-2.1817	1.55
5780	*OR4C12*	2.2931	2.2873	0.07918	-1.1021	1.55
5781	*CSF1*	3.6208	0.77738	0.26076	0.1939	1.55
5782	*COX4I2*	2.9169	2.0041	-0.26245	-1.8038	1.55
5783	*TEX14*	2.6621	1.9913	0.00305	-0.3377	1.55
5784	*TTLL11*	5.4564	-0.38503	-0.41533	-1.3835	1.55
5785	*GOLGA8K*	4.3952	0.21295	0.04757	-0.99729	1.55
5786	*ZNF479*	2.7889	1.3075	0.55821	-0.17622	1.55
5787	*C19orf81*	2.2309	2.1613	0.26207	-2.5686	1.55
5788	*C17orf77*	2.6692	2.0087	-0.02387	-0.39012	1.55
5789	*SCHIP1*	4.3797	0.43954	-0.16569	-1.7263	1.55

5790	*MYO5B*	4.5304	0.48797	-0.36579	-1.0383	1.55
5791	*UPF1*	3.7447	0.47055	0.43717	-2.6024	1.55
5792	*STK33*	2.5674	2.1822	-0.09731	-2.0837	1.55
5793	*KHDRBS3*	3.3429	1.0577	0.25162	-0.76582	1.55
5794	*INHBA*	3.1754	1.3542	0.12258	-0.60072	1.55
5795	*OMA1*	3.5528	1.1051	-0.0083	-0.37213	1.55
5796	*THUMPD2*	2.6154	1.3485	0.68562	-1.8387	1.55
5797	*TAS2R39*	2.8021	1.066	0.78063	-0.14173	1.55
5798	*SAMD3*	2.8317	1.2959	0.52111	0.32176	1.55
5799	*WDR20*	2.9752	1.7479	-0.0746	-0.12945	1.55
5800	*RHOXF2B*	2.5622	1.513	0.57321	-1.3668	1.55
5801	*SMG8*	3.508	1.4165	-0.27612	-0.56492	1.55
5802	*HSBP1*	4.3431	0.52376	-0.22042	-0.61585	1.55
5803	*KRTAP10-5*	4.3174	0.30382	0.02521	-0.02965	1.55
5804	*KIAA2026*	3.3519	0.99875	0.29535	-1.5167	1.55
5805	*HSD17B14*	3.9173	2.0317	-1.3036	-1.5267	1.55
5806	*GOT1*	4.1722	0.47018	0.00283	-0.91981	1.55
5807	*PCMTD2*	4.1158	0.94146	-0.41237	-1.6044	1.55
5808	*SCN5A*	4.747	0.51984	-0.62395	-1.0359	1.55
5809	*ENPP4*	4.9015	0.077732	-0.33682	-1.5268	1.55
5810	*PDLIM5*	3.4033	0.92528	0.31371	-0.35449	1.55
5811	*REXO4*	3.6211	1.5286	-0.50748	-2.1984	1.55
5812	*TMEM55B*	3.2017	0.85669	0.58307	-0.07087	1.55
5813	*CLK2*	4.2692	0.74226	-0.3701	-0.72196	1.55
5814	*HIST1H1E*	3.073	2.7867	-1.219	-1.9533	1.55
5815	*REG3G*	3.9486	0.48577	0.2057	-1.9919	1.55
5816	*DACT2*	4.1789	0.25015	0.21099	-1.2693	1.55
5817	*PIGA*	2.7464	1.7034	0.19018	-1.6065	1.55
5818	*SYNE4*	3.4795	2.0049	-0.84452	-1.6182	1.55
5819	*ZNF320*	3.8484	1.5405	-0.74972	-1.0872	1.55
5820	*PPM1J*	3.0961	1.3549	0.18793	-0.26332	1.55
5821	*GRHL3*	3.4159	0.99591	0.22709	-0.48606	1.55
5822	*SHANK2*	3.4101	0.71987	0.50868	-1.5167	1.55
5823	*CCDC71L*	3.498	1.7515	-0.61092	-1.5544	1.55
5824	*DCTN2*	3.5954	1.3737	-0.33109	-0.47527	1.55
5825	*STK35*	4.2845	0.819	-0.46562	-1.9099	1.55
5826	*C2orf82*	4.0043	1.1681	-0.53487	-1.1399	1.55
5827	*ALX3*	2.8949	2.6305	-0.88839	-1.3118	1.55
5828	*PACRGL*	2.1479	1.6888	0.80023	-1.2568	1.55
5829	*ZSWIM2*	4.049	1.7504	-1.1631	-2.252	1.55
5830	*STAM2*	1.8897	1.6793	1.0664	0.8032	1.55
5831	*ELOVL5*	3.3703	0.66261	0.60198	-0.74913	1.54
5832	*OR9I1*	2.9823	2.5142	-0.86546	-1.4684	1.54
5833	*PLEKHF2*	3.4625	0.97837	0.19015	-0.78229	1.54
5834	*HRASLS5*	3.5868	1.2945	-0.251	-0.50133	1.54
5835	*SLC2A1*	2.3421	2.2401	0.04765	-1.2486	1.54

5836	*LCAT*	3.2138	0.98643	0.42918	-1.4946	1.54
5837	*GCKR*	5.059	0.013874	-0.44353	-0.93365	1.54
5838	*CCDC80*	3.7881	1.6181	-0.77733	-0.89957	1.54
5839	*GDPGP1*	4.2053	0.34192	0.08118	-0.43993	1.54
5840	*EIF5AL1*	3.6211	0.64483	0.36198	-1.4248	1.54
5841	*SPATS2L*	1.8668	1.8051	0.95576	0.23888	1.54
5842	*TRIM10*	3.4269	1.1176	0.08246	-0.4457	1.54
5843	*IPCEF1*	4.9465	0.71441	-1.0343	-1.122	1.54
5844	*PKDREJ*	4.72	0.088291	-0.18187	-1.6636	1.54
5845	*OR11H12*	3.3887	1.6617	-0.42401	-0.56773	1.54
5846	*ASCL2*	3.526	0.5631	0.53629	-2.0908	1.54
5847	*ARHGAP6*	2.2939	2.2112	0.11908	-0.16976	1.54
5848	*ZNF44*	2.6102	1.9211	0.09188	-0.43772	1.54
5849	*NOXA1*	2.6486	1.116	0.85815	-0.62629	1.54
5850	*PMM2*	3.812	0.5922	0.21848	-0.76355	1.54
5851	*ACTR3B*	3.6542	0.97636	-0.00822	-0.9169	1.54
5852	*EEF1D*	4.6284	0.17514	-0.18158	-1.3402	1.54
5853	*GAGE12H*	4.2959	0.53466	-0.20985	-0.32243	1.54
5854	*NKRF*	2.9307	1.3642	0.3254	-0.47577	1.54
5855	*CCDC61*	4.3883	0.33301	-0.1025	-1.6001	1.54
5856	*FAM135B*	1.9278	1.5983	1.0904	-0.79332	1.54
5857	*CCDC185*	2.5884	2.1869	-0.15884	-0.30145	1.54
5858	*SLC6A18*	4.4989	0.22384	-0.10704	-1.72	1.54
5859	*IL12RB2*	2.4797	1.6538	0.48175	-1.0089	1.54
5860	*MYO9A*	3.6245	0.6062	0.38442	-0.98238	1.54
5861	*MARK4*	4.1376	0.31778	0.15924	-1.0997	1.54
5862	*ZBTB7B*	2.5679	1.1423	0.90406	-1.6754	1.54
5863	*VPS37B*	3.0452	1.4874	0.08093	-1.2411	1.54
5864	*GDPD5*	3.7603	1.4163	-0.56329	-2.5264	1.54
5865	*PROSER1*	3.4541	0.8257	0.33331	-1.077	1.54
5866	*ENTPD7*	3.7824	1.2152	-0.38472	-0.94113	1.54
5867	*EXT2*	3.7938	0.68584	0.13256	-0.90256	1.54
5868	*CERCAM*	3.6898	1.6255	-0.70417	-1.1126	1.54
5869	*MILR1*	3.2357	0.79998	0.57509	-0.49704	1.54
5870	*C4orf46*	2.6859	1.2691	0.6556	-1.3837	1.54
5871	*VCY*	2.9361	2.1543	-0.47993	-2.2014	1.54
5872	*ACTBL2*	3.856	0.74161	0.01275	-0.94563	1.54
5873	*IL19*	3.7822	1.5932	-0.76671	-1.9219	1.54
5874	*LRRD1*	3.9281	1.148	-0.46743	-1.7683	1.54
5875	*SOD3*	2.5966	1.3932	0.61884	-1.9041	1.54
5876	*KIAA1328*	4.1038	0.35651	0.14773	-1.3542	1.54
5877	*DDHD1*	2.8401	1.4534	0.31365	-1.5052	1.54
5878	*MRPL32*	4.4906	0.95507	-0.83862	-1.2787	1.54
5879	*SPEG*	3.9117	1.0884	-0.39318	-1.3711	1.54
5880	*MKL1*	3.5252	2.5836	-1.502	-1.9814	1.54
5881	*CLCN1*	3.3447	2.0772	-0.81549	-1.34	1.54

5882	*BATF2*	3.8207	1.9017	-1.1169	-2.3563	1.54
5883	*NANOS1*	3.1332	1.2943	0.17765	-1.4434	1.54
5884	*TYROBP*	3.7918	0.57939	0.23385	-2.4394	1.54
5885	*RLF*	2.6494	1.5099	0.44527	-0.05733	1.53
5886	*SYDE2*	4.11	0.61764	-0.12329	-1.4067	1.53
5887	*FZD3*	3.1039	2.0844	-0.58404	-2.6675	1.53
5888	*UBAP1L*	3.2906	1.1866	0.12627	-0.85821	1.53
5889	*TDRD6*	2.529	1.4304	0.64397	-0.95321	1.53
5890	*CITED1*	4.4529	0.42261	-0.27291	-1.9622	1.53
5891	*C19orf12*	4.1202	1.0431	-0.56225	-1.5297	1.53
5892	*PAGE1*	2.9723	2.3039	-0.67536	-1.3254	1.53
5893	*CTNNA3*	3.7452	1.4723	-0.61675	-1.8157	1.53
5894	*CCDC153*	2.0276	1.8785	0.69402	0.22001	1.53
5895	*PPP3CC*	5.594	-0.39082	-0.60371	-0.68668	1.53
5896	*CXCR1*	3.9888	1.0612	-0.45103	-2.2664	1.53
5897	*ZNF471*	3.1364	1.0111	0.45096	-0.62017	1.53
5898	*FUT2*	3.9039	0.53655	0.15769	-0.90922	1.53
5899	*AGO2*	2.4425	1.1331	1.0212	0.063662	1.53
5900	*SLC16A8*	3.0295	1.5519	0.01539	-1.1122	1.53
5901	*OSBPL5*	5.2216	0.37684	-1.0019	-1.9624	1.53
5902	*LECT2*	3.1098	0.8546	0.63147	-2.3696	1.53
5903	*PSTPIP1*	3.7016	0.70846	0.18579	-0.84136	1.53
5904	*NEUROD4*	5.1602	-0.10716	-0.45754	-1.2837	1.53
5905	*APOA2*	3.6674	0.50231	0.42443	-1.4715	1.53
5906	*CHST6*	2.3783	1.5092	0.70661	-1.95	1.53
5907	*DAND5*	4.5555	0.074813	-0.03668	-0.59622	1.53
5908	*BMP4*	3.2461	2.7347	-1.3875	-1.9622	1.53
5909	*TXLNG*	4.8715	-0.06413	-0.2143	-0.62154	1.53
5910	*DCAF4L1*	4.5798	0.020906	-0.00832	-0.46747	1.53
5911	*ZNF197*	3.8308	0.39905	0.36174	-0.49034	1.53
5912	*CEACAM20*	2.4897	2.3577	-0.2562	-1.3282	1.53
5913	*LZIC*	2.9903	1.3983	0.20104	-1.2782	1.53
5914	*TAS2R1*	3.1445	1.9914	-0.54657	-1.8996	1.53
5915	*ARL13A*	3.8784	1.9173	-1.2079	-2.2021	1.53
5916	*PLAC8L1*	2.9797	1.818	-0.21148	-1.2442	1.53
5917	*PRTN3*	2.5049	2.1888	-0.10769	-1.1075	1.53
5918	*EPDR1*	4.1304	0.94623	-0.49071	-1.0153	1.53
5919	*MRVI1*	3.3643	0.72927	0.49156	-1.3416	1.53
5920	*HIST1H2BH*	1.6951	1.5326	1.3569	1.0688	1.53
5921	*PRM2*	3.5423	0.7007	0.34108	-1.2218	1.53
5922	*FRRS1L*	2.5485	1.5652	0.46992	-0.59259	1.53
5923	*TRIML1*	4.1432	0.38273	0.05725	-2.8521	1.53
5924	*N4BP2L2*	3.6338	1.3955	-0.44661	-1.6885	1.53
5925	*EBLN1*	2.0205	1.5253	1.0355	-1.1479	1.53
5926	*PAX2*	4.386	0.55094	-0.35604	-0.93011	1.53
5927	*OLFML2A*	3.2199	2.5117	-1.1508	-1.2277	1.53

5928	*PMCH*	2.6567	2.5935	-0.66948	-0.88213	1.53
5929	*ZNF550*	3.8053	1.4855	-0.71019	-1.0088	1.53
5930	*WDR41*	3.4923	0.7883	0.29984	-0.40822	1.53
5931	*ADAT3*	1.9487	1.424	1.2058	-0.12729	1.53
5932	*IMPA2*	4.1061	0.53073	-0.05841	-1.4684	1.53
5933	*CBFB*	5.0406	-0.06015	-0.4027	-1.5031	1.53
5934	*TXNDC11*	3.8667	0.64291	0.0674	-0.20165	1.53
5935	*CTAGE8*	2.6224	1.2524	0.70097	-1.661	1.53
5936	*PREPL*	3.0065	1.0717	0.49689	-0.00832	1.53
5937	*STX3*	2.935	2.2703	-0.63092	-1.9748	1.52
5938	*C2CD2*	3.2376	0.70899	0.62646	-1.3393	1.52
5939	*ATG9A*	3.4146	1.826	-0.6677	-0.96751	1.52
5940	*COL4A3*	2.0194	1.721	0.83229	0.2638	1.52
5941	*SHCBP1*	3.3574	1.2518	-0.03705	-0.20554	1.52
5942	*HOXB13*	2.8692	1.7362	-0.03462	-0.60371	1.52
5943	*TSNARE1*	3.1481	1.2292	0.19313	-1.3591	1.52
5944	*SMU1*	2.574	1.9207	0.07442	-1.165	1.52
5945	*HIVEP3*	5.2661	0.33847	-1.0364	-4.9314	1.52
5946	*TIMM9*	4.3832	0.33837	-0.15443	-0.93399	1.52
5947	*SIRT4*	3.6235	1.1532	-0.20958	-1.5598	1.52
5948	*ZNF415*	2.3149	2.0769	0.17276	-0.64793	1.52
5949	*PHYHD1*	3.8558	0.89213	-0.18348	-1.4185	1.52
5950	*MCEMP1*	2.8632	1.433	0.26822	-0.96583	1.52
5951	*OCEL1*	3.6048	0.62865	0.33086	-0.56634	1.52
5952	*DNAH1*	2.4965	1.9822	0.08527	-0.64857	1.52
5953	*SCRT1*	3.4547	1.5149	-0.4057	-1.0951	1.52
5954	*FAM65A*	2.7201	1.3358	0.50798	-1.106	1.52
5955	*ABCG4*	1.8494	1.658	1.0558	0.53797	1.52
5956	*BRWD1*	2.5579	1.2436	0.76087	0.53697	1.52
5957	*ORMDL2*	3.9371	2.2047	-1.5805	-1.6428	1.52
5958	*ERRFI1*	4.3803	0.15612	0.02471	-0.13006	1.52
5959	*PKN3*	2.218	1.4518	0.89072	-0.35472	1.52
5960	*CELA2A*	3.2579	0.69715	0.60532	-2.127	1.52
5961	*CTSZ*	3.4639	1.1286	-0.03492	-1	1.52
5962	*GUF1*	3.4191	0.84881	0.2887	-1.2746	1.52
5963	*C17orf78*	3.3695	2.3671	-1.1808	-1.2097	1.52
5964	*SP6*	3.9007	1.0977	-0.44353	-0.75351	1.52
5965	*BHLHE23*	3.7808	1.9684	-1.1944	-2.6805	1.52
5966	*MTL5*	4.7079	0.18841	-0.34158	-2.6549	1.52
5967	*MCIDAS*	4.9072	0.61108	-0.96367	-1.0582	1.52
5968	*ST6GALNAC4*	4.7679	0.053968	-0.26752	-1.2872	1.52
5969	*SFRP1*	4.0729	0.75336	-0.27244	-1.3728	1.52
5970	*SCARF1*	3.6188	0.67328	0.2606	-0.2279	1.52
5971	*FRMD4B*	4.6254	0.3074	-0.38059	-2.0586	1.52
5972	*PRKAB1*	2.6134	1.6356	0.30316	-0.51929	1.52
5973	*THOP1*	4.4139	1.0645	-0.92629	-2.4018	1.52

5974	*LYSMD2*	2.3279	2.2872	-0.06311	-0.13638	1.52
5975	*MTMR3*	1.7152	1.5909	1.2457	-0.36761	1.52
5976	*OR4K17*	2.4264	2.0995	0.0254	-0.81906	1.52
5977	*GNRHR*	4.1587	0.22274	0.16985	-1.7177	1.52
5978	*NAIP*	2.4662	1.146	0.93884	-0.79052	1.52
5979	*LARP7*	4.06	0.97477	-0.48538	-0.74192	1.52
5980	*ANKRD33*	3.0466	1.0973	0.40512	-0.75395	1.52
5981	*ATP11A*	2.8273	2.6453	-0.92375	-1.0153	1.52
5982	*KCTD3*	4.313	0.41346	-0.17766	-0.43231	1.52
5983	*RABGAP1*	4.328	0.42665	-0.20648	-1.2456	1.52
5984	*CLN3*	2.9133	2.4063	-0.77162	-0.78314	1.52
5985	*PPARGC1B*	3.423	0.60842	0.51629	-1.4797	1.52
5986	*LAPTM4B*	4.7814	0.78538	-1.0198	-2.2676	1.52
5987	*KRT7*	3.9132	0.70572	-0.07219	-0.78229	1.52
5988	*ZNF431*	4.6196	-0.00232	-0.07212	-0.55284	1.52
5989	*HOMER1*	4.1393	1.8396	-1.4338	-2.1369	1.52
5990	*FAM159B*	2.3968	2.0004	0.14783	-0.28778	1.52
5991	*ADAM11*	2.9599	2.4095	-0.82439	-1.407	1.52
5992	*ADIPOR1*	3.4525	1.3155	-0.22313	-0.92278	1.51
5993	*ZNF101*	3.6773	0.62206	0.24498	-3.1038	1.51
5994	*MRGPRE*	4.0011	1.2929	-0.74979	-0.8846	1.51
5995	*CCNK*	3.6349	1.0787	-0.16951	-0.42074	1.51
5996	*PRAMEF9*	4.9456	0.51906	-0.92086	-1.3955	1.51
5997	*ENC1*	3.5752	1.7465	-0.77828	-1.3039	1.51
5998	*KRT80*	3.9271	0.3755	0.2405	0.080834	1.51
5999	*SYNE3*	2.7393	1.147	0.65655	0.35036	1.51
6000	*PES1*	2.7594	2.2033	-0.42163	-1.5291	1.51
6001	*PRLHR*	2.6415	1.9487	-0.04919	-0.74141	1.51
6002	*RBFA*	2.5661	1.0827	0.89141	-1.3542	1.51
6003	*STOX1*	4.7426	0.52683	-0.72985	-0.83278	1.51
6004	*OR9A4*	2.5544	2.3891	-0.40581	-0.41317	1.51
6005	*NPHP4*	3.7646	0.59379	0.17837	-0.82326	1.51
6006	*ZNF708*	4.4993	0.71788	-0.68153	-1.5663	1.51
6007	*LRRC8D*	2.3883	1.3338	0.81294	0.57088	1.51
6008	*DECR2*	4.3226	0.33517	-0.12414	-1.5563	1.51
6009	*SPATS2*	3.4659	0.67506	0.39196	-0.38144	1.51
6010	*PRAMEF14*	4.8154	0.19463	-0.47739	-0.56922	1.51
6011	*GAS2L3*	3.2888	0.63413	0.60935	0.51748	1.51
6012	*XPO4*	3.9914	1.7358	-1.1959	-2.1213	1.51
6013	*MPZL3*	3.5849	0.73531	0.21027	-1.4025	1.51
6014	*OR5D13*	2.3371	1.4199	0.77348	-0.61114	1.51
6015	*RNF111*	3.9507	0.35451	0.22523	-0.94493	1.51
6016	*GRASP*	2.4023	1.9392	0.18854	-1.8354	1.51
6017	*MROH6*	3.3179	1.651	-0.43886	-2.2537	1.51
6018	*HOXD10*	2.7578	2.4709	-0.69913	-1.8154	1.51
6019	*FBXW10*	2.9465	1.5968	-0.0139	-0.95322	1.51

6020	*XDH*	2.9171	2.1298	-0.51785	-0.94472	1.51
6021	*LOC100133267*	2.3184	1.3359	0.87369	-0.78444	1.51
6022	*SUV39H1*	3.2026	0.69539	0.62925	-0.00805	1.51
6023	*ARID5B*	4.3595	0.17547	-0.00826	-2.3424	1.51
6024	*CADM3*	4.7863	0.5069	-0.76716	-1.3957	1.51
6025	*C14orf119*	4.3286	1.096	-0.90104	-1.1366	1.51
6026	*CKAP4*	4.5037	0.1016	-0.08183	-0.42958	1.51
6027	*PTMA*	3.6637	0.75852	0.10111	-1.5428	1.51
6028	*C16orf46*	4.2158	0.87192	-0.56492	-1.1357	1.51
6029	*PLEKHA7*	2.6399	1.6958	0.18653	-2.3287	1.51
6030	*SFTPB*	3.936	1.3886	-0.80315	-0.94466	1.51
6031	*ALPL*	1.9456	1.9449	0.63028	0.41919	1.51
6032	*GRAMD1C*	2.9342	0.94755	0.6389	-2.0429	1.51
6033	*GPBAR1*	2.3314	1.1663	1.0221	0.49035	1.51
6034	*ARHGEF28*	3.2531	2.5113	-1.2455	-1.8627	1.51
6035	*PRR23B*	2.2765	1.2286	1.0136	-1.0508	1.51
6036	*ZNF419*	3.9939	0.32154	0.20314	-0.51	1.51
6037	*GJC3*	3.826	0.94409	-0.25163	-2.1113	1.51
6038	*TSPYL6*	3.0224	1.373	0.1224	-0.3308	1.51
6039	*MUC21*	3.1416	2.5911	-1.2149	-1.4683	1.51
6040	*SPAM1*	3.0725	1.409	0.03609	-1.7307	1.51
6041	*CXorf65*	2.7934	1.1518	0.57176	-2.1055	1.51
6042	*DDX53*	3.7364	0.55612	0.22441	-1.63	1.51
6043	*LYPD6*	3.2738	1.6245	-0.38163	-0.42945	1.51
6044	*SERPINA7*	4.1688	1.1027	-0.75495	-0.87198	1.51
6045	*SLC30A1*	4.1504	1.6939	-1.3279	-1.479	1.51
6046	*PTGER1*	3.3381	1.3602	-0.18304	-0.5728	1.51
6047	*RXFP4*	2.9624	0.8259	0.72657	-0.8424	1.50
6048	*RPTOR*	4.3111	0.44004	-0.23628	-1.9693	1.50
6049	*SSH1*	3.084	0.94562	0.4833	-0.64186	1.50
6050	*SLC22A31*	3.0526	1.6177	-0.15831	-1.4104	1.50
6051	*ADSS*	4.0261	1.3752	-0.8903	-1.027	1.50
6052	*GRN*	4.1367	0.60721	-0.23497	-0.54246	1.50
6053	*IFFO2*	2.5388	1.0251	0.94391	-0.3486	1.50
6054	*CCL5*	2.9119	2.1257	-0.53045	-0.70406	1.50
6055	*KCNG1*	3.7007	1.4517	-0.64596	-1.9746	1.50
6056	*CCDC47*	3.6355	0.46797	0.40295	-0.84337	1.50
6057	*GPR161*	2.3735	1.3676	0.76501	-1.4527	1.50
6058	*MAGEA8*	4.5555	1.0346	-1.084	-1.8566	1.50
6059	*FAM126A*	2.2056	1.1674	1.1325	1.1166	1.50
6060	*STK25*	3.4226	1.2938	-0.21216	-2.012	1.50
6061	*DNAH17*	4.0278	1.1267	-0.65113	-1.4996	1.50
6062	*CMAS*	2.5616	2.048	-0.10664	-0.17658	1.50
6063	*KRTAP3-2*	3.5781	0.79423	0.12988	-0.9977	1.50
6064	*PIP4K2C*	5.1316	-0.27291	-0.35694	-2.3534	1.50
6065	*OR10X1*	3.6514	0.45979	0.39048	-0.29227	1.50

6066	*LRRIQ4*	1.9428	1.2992	1.2594	-0.03448	1.50
6067	*TARSL2*	4.313	1.9401	-1.7526	-1.7587	1.50
6068	*METTL15*	2.5931	2.5545	-0.64806	-1.9748	1.50
6069	*PIK3CA*	5.1511	0.91449	-1.5663	-2.7705	1.50
6070	*MNDA*	4.021	0.33537	0.14261	-0.68565	1.50
6071	*PFKL*	5.0933	0.18976	-0.7847	-2.0838	1.50
6072	*ALDH9A1*	3.0779	1.1166	0.30364	-1.2802	1.50
6073	*OTP*	3.0775	1.0379	0.38094	-0.20942	1.50
6074	*MMP23B*	2.7268	2.2093	-0.44094	-0.49563	1.50
6075	*PATL1*	2.7601	2.09	-0.35585	-1.3627	1.50
6076	*BMP6*	4.1649	0.22609	0.10206	-0.54847	1.50
6077	*SLC4A10*	5.2543	0.50009	-1.2623	-2.0553	1.50
6078	*PTPN5*	2.8205	1.2664	0.40464	-0.25609	1.50
6079	*DAZAP1*	2.1484	1.9032	0.43965	-1.8112	1.50
6080	*MPC1*	5.264	-0.37343	-0.39967	-0.79779	1.50
6081	*CNPY3*	4.7688	0.015765	-0.29442	-1.2272	1.50
6082	*SNRNP25*	3.7973	0.58593	0.10647	-1.0261	1.50
6083	*ARMS2*	3.1266	2.0917	-0.7288	-0.93696	1.50
6084	*ALOXE3*	2.4496	1.9466	0.08994	-0.47293	1.50
6085	*PTPN13*	3.7827	1.184	-0.48156	-1.0421	1.50
6086	*UGT2A2*	2.8559	1.3673	0.26163	-0.45012	1.49
6087	*SSH3*	4.1359	0.36987	-0.0211	-1.7882	1.49
6088	*TAP2*	2.6417	2.2591	-0.41621	-0.47948	1.49
6089	*VRK3*	3.2329	1.2206	0.03043	-0.28209	1.49
6090	*PCMTD1*	2.9014	2.7785	-1.1962	-1.5077	1.49
6091	*VWA2*	4.9662	0.42941	-0.91296	-1.3627	1.49
6092	*GPX8*	3.3379	0.62661	0.51793	-0.65839	1.49
6093	*OR4S1*	2.3399	1.411	0.73114	0.28875	1.49
6094	*FABP9*	5.1415	0.18203	-0.84158	-0.94954	1.49
6095	*RNF168*	2.7381	2.2969	-0.55311	-0.63312	1.49
6096	*FOXE3*	2.4348	2.2619	-0.21499	-0.92451	1.49
6097	*NUDT19*	4.2982	0.65015	-0.4674	-2.3381	1.49
6098	*CNTN5*	3.9499	0.7408	-0.2098	-1.8537	1.49
6099	*VDR*	3.1125	0.73902	0.62915	-1.536	1.49
6100	*COL1A1*	2.7914	1.3797	0.30903	0.057237	1.49
6101	*RIN3*	2.5329	1.8584	0.08825	-0.86123	1.49
6102	*OIT3*	4.7015	0.5272	-0.75023	-0.84248	1.49
6103	*TLR2*	3.4431	1.2468	-0.21148	-1.3751	1.49
6104	*SCGB1A1*	1.8439	1.5147	1.1197	-0.08362	1.49
6105	*SHISA9*	3.1246	0.91197	0.43954	0.36586	1.49
6106	*SYN3*	4.7186	-0.04085	-0.20202	-1.4688	1.49
6107	*SLC5A8*	3.4673	0.65594	0.35194	-0.56815	1.49
6108	*HIST1H2AA*	2.7916	1.6509	0.03259	-0.02811	1.49
6109	*GBE1*	2.8806	1.2086	0.38377	-0.67943	1.49
6110	*KLK1*	4.3843	0.14115	-0.05348	-0.94493	1.49
6111	*DDX24*	1.7368	1.673	1.0602	0.049989	1.49

6112	*MMP20*	3.6632	2.1098	-1.3031	-1.5886	1.49
6113	*SPRY3*	3.0299	1.607	-0.16774	-1.2015	1.49
6114	*TMF1*	4.0798	0.52357	-0.13436	-0.50419	1.49
6115	*DCHS1*	4.2969	0.78607	-0.6141	-2.4699	1.49
6116	*TSN*	4.1635	0.99573	-0.69112	-1.5821	1.49
6117	*EBP*	2.927	1.0233	0.51758	0.050728	1.49
6118	*ABHD11*	1.771	1.5819	1.1137	0.58526	1.49
6119	*TRAPPC12*	4.5346	0.83393	-0.9034	-1.3327	1.49
6120	*SPIN2A*	3.5462	0.70943	0.20802	0.029689	1.49
6121	*ZNF518A*	3.0322	1.4607	-0.0298	-0.61281	1.49
6122	*MAGEE1*	2.5162	1.2983	0.64807	-0.86772	1.49
6123	*PSAPL1*	2.9356	1.7807	-0.25444	-0.60554	1.49
6124	*RGS13*	3.1604	0.82505	0.47547	-2.223	1.49
6125	*ATP13A5*	2.2921	1.4682	0.70052	-0.35473	1.49
6126	*CCDC34*	5.6109	-0.49902	-0.65132	-1.1145	1.49
6127	*SLC5A4*	2.5195	2.2225	-0.28162	-0.64967	1.49
6128	*FGD6*	3.7007	0.65441	0.1049	-1.5568	1.49
6129	*ZNF551*	3.1558	1.2925	0.01116	-0.33081	1.49
6130	*OR4F21*	4.1003	0.80221	-0.44353	-1.7493	1.49
6131	*TXNDC2*	2.8287	1.2108	0.41933	-1.5715	1.49
6132	*CTAGE5*	3.575	1.6065	-0.72283	-0.84816	1.49
6133	*ATP7B*	3.9789	1.6178	-1.1383	-1.748	1.49
6134	*SLC23A3*	4.2258	0.26461	-0.03283	-1.2697	1.49
6135	*ZIC5*	2.9845	0.97705	0.4958	-0.30608	1.49
6136	*AHCTF1*	2.9298	1.1257	0.40166	-0.96857	1.49
6137	*RNF14*	2.8044	1.2124	0.43983	0.3014	1.49
6138	*TSPAN2*	5.4831	0.43639	-1.463	-2.8398	1.49
6139	*MLF2*	3.3245	0.68213	0.44898	-0.06794	1.49
6140	*UMODL1*	4.8466	0.18614	-0.57742	-0.67482	1.49
6141	*CCDC92*	4.7276	0.16555	-0.43886	-0.50087	1.48
6142	*CDK10*	4.5277	1.107	-1.181	-1.5707	1.48
6143	*FAM150B*	4.2722	0.14912	0.03238	-0.91127	1.48
6144	*GTF2A1*	3.2611	1.6641	-0.47192	-1.16	1.48
6145	*TMCC1*	4.2116	0.31701	-0.07545	-2.0436	1.48
6146	*GSTA5*	2.6793	1.4538	0.31936	-0.03366	1.48
6147	*SRSF10*	3.9363	0.40972	0.1062	-1.5663	1.48
6148	*CDCA8*	2.5173	1.6419	0.29244	0.17431	1.48
6149	*FAM81A*	3.632	0.68632	0.13245	-0.47227	1.48
6150	*GRM6*	4.5239	0.96553	-1.0402	-1.6158	1.48
6151	*ACTG1*	3.543	1.3579	-0.4521	-0.8934	1.48
6152	*FAM127C*	4.0678	0.25825	0.12253	-0.35735	1.48
6153	*CCL14*	3.0437	1.3183	0.08633	-1.135	1.48
6154	*CCDC28A*	3.4171	1.6207	-0.58949	-0.81548	1.48
6155	*APOBEC3H*	1.9886	1.8635	0.59553	-0.84872	1.48
6156	*ASTN1*	2.9474	0.80389	0.6956	-0.87504	1.48
6157	*FGFBP2*	4.0468	1.3472	-0.94739	-2.3496	1.48

6158	*RFWD3*	3.6466	0.82493	-0.02638	-2.5707	1.48
6159	*TMEM9*	2.9824	0.76114	0.7014	-2.01	1.48
6160	*LRRC6*	2.5138	1.2294	0.70073	-0.41995	1.48
6161	*AP1B1*	3.8933	0.29458	0.25604	-0.97477	1.48
6162	*ZDHHC20*	3.0736	0.80978	0.55854	-0.46415	1.48
6163	*FAN1*	1.6681	1.4734	1.3001	-1.1175	1.48
6164	*DHRS13*	5.556	-0.22235	-0.89219	-2.0952	1.48
6165	*CT62*	3.0891	1.8581	-0.50695	-3.4184	1.48
6166	*DLEU7*	4.5702	0.72019	-0.85059	-1.4312	1.48
6167	*MZT2A*	3.3021	0.8495	0.2868	-1.2937	1.48
6168	*TMEM35*	3.306	1.8619	-0.72973	-0.85467	1.48
6169	*HMX2*	3.3225	0.56133	0.55401	-1.2357	1.48
6170	*TBC1D20*	2.7968	0.91011	0.73079	-1.8645	1.48
6171	*OR2A1*	3.1432	0.74407	0.54869	-0.52495	1.48
6172	*SENP7*	3.6521	0.49803	0.28553	0.10263	1.48
6173	*RNASE8*	2.9434	2.0513	-0.55909	-0.75665	1.48
6174	*UBL7*	3.6276	0.48211	0.32561	-0.42137	1.48
6175	*MRPL2*	3.3424	0.60733	0.48467	-1.7164	1.48
6176	*OPN1SW*	2.5202	1.9901	-0.07636	-1.3047	1.48
6177	*POLI*	1.9885	1.5518	0.89214	-1.5171	1.48
6178	*IL23R*	4.9192	-0.10195	-0.38522	-2.3534	1.48
6179	*CTGF*	3.8952	0.42771	0.1083	-1.7782	1.48
6180	*ATOH1*	5.6004	0.25219	-1.4214	-1.7434	1.48
6181	*PCDHA13*	2.9092	1.1127	0.40872	-0.65417	1.48
6182	*PTPRU*	5.3662	0.25861	-1.1945	-1.8046	1.48
6183	*SMC1B*	4.5417	0.025264	-0.13683	-0.53058	1.48
6184	*WHSC1L1*	4.2659	1.0664	-0.90281	-1.135	1.48
6185	*ADGRL3*	3.1226	0.79887	0.50528	-0.19089	1.48
6186	*LEP*	1.6156	1.5447	1.2651	-2.1391	1.48
6187	*SPATA17*	4.0667	1.3675	-1.01	-1.0604	1.47
6188	*TESPA1*	3.9484	1.1794	-0.70361	-0.96072	1.47
6189	*IFNA17*	3.8343	0.58647	0.00329	-0.17991	1.47
6190	*ADAM22*	2.9902	1.6921	-0.25859	-1.3033	1.47
6191	*AVL9*	4.2644	0.60236	-0.44353	-0.82366	1.47
6192	*LRFN1*	2.5959	1.9514	-0.12456	-0.83305	1.47
6193	*RIMS1*	2.645	1.9146	-0.13688	-1.2633	1.47
6194	*COL9A3*	2.913	2.5135	-1.0066	-1.128	1.47
6195	*SLCO1B3*	3.183	1.2618	-0.02502	-2.3452	1.47
6196	*FAM131C*	3.0734	2.9311	-1.5848	-2.1357	1.47
6197	*HIGD1A*	1.9265	1.368	1.1246	-1.6665	1.47
6198	*ZNF571*	3.1776	0.7079	0.53314	-2.1796	1.47
6199	*EDN3*	2.2426	2.1601	0.01581	-0.74282	1.47
6200	*CCL16*	3.9235	0.81334	-0.31848	-2.9244	1.47
6201	*FAM110C*	4.6365	-0.08956	-0.12896	-1.498	1.47
6202	*SCRG1*	2.4073	1.9871	0.02267	-0.4221	1.47
6203	*PTK2*	3.5364	1.5806	-0.69995	-2.1576	1.47

6204	*ACTRT1*	2.4394	1.25	0.72742	-2.0116	1.47
6205	*C14orf105*	4.1527	0.93005	-0.66616	-0.90906	1.47
6206	*SIGLEC1*	4.8491	-0.20434	-0.2283	-0.76055	1.47
6207	*NSMCE1*	3.0433	0.97867	0.39359	-1.3402	1.47
6208	*FTHL17*	3.5064	0.55416	0.35463	-0.63673	1.47
6209	*HID1*	3.4408	2.1416	-1.1673	-1.7285	1.47
6210	*FCER1G*	2.2126	1.457	0.74434	-0.35694	1.47
6211	*GPR89A*	3.8211	1.0347	-0.44236	-1.9511	1.47
6212	*IFNA14*	2.3704	1.3138	0.72916	-2.5571	1.47
6213	*HIST1H2BM*	4.6961	0.45493	-0.73823	-1.7445	1.47
6214	*SLC6A1*	5.485	-0.3146	-0.758	-1.6494	1.47
6215	*INPP4A*	4.4979	0.70725	-0.79316	-1.6848	1.47
6216	*MAP4K1*	3.4368	2.4013	-1.4265	-1.5337	1.47
6217	*GAS2L1*	3.9047	0.69628	-0.18996	-1.3232	1.47
6218	*TMEM234*	3.7894	0.86308	-0.24157	-1.5502	1.47
6219	*PDZD9*	3.1194	1.7344	-0.44353	-0.64748	1.47
6220	*FHL5*	2.9149	1.0007	0.49436	-1.2935	1.47
6221	*PDIA2*	4.3719	0.4232	-0.38541	-0.75659	1.47
6222	*KIAA1191*	4.4552	0.17232	-0.21796	-0.33844	1.47
6223	*UGT2B7*	3.8132	1.2428	-0.64654	-1.2957	1.47
6224	*PLP1*	3.1629	1.206	0.04036	-1.3619	1.47
6225	*CCDC43*	3.9197	0.73245	-0.24327	-1.2192	1.47
6226	*CAMK2N1*	3.651	0.6471	0.11031	-0.33584	1.47
6227	*C11orf95*	3.7704	0.8689	-0.23109	-1.4356	1.47
6228	*MMP10*	2.0421	1.9844	0.38166	0.11185	1.47
6229	*ARHGEF5*	2.9164	1.089	0.40274	-0.35232	1.47
6230	*FAM170B*	3.4409	0.49118	0.47556	-1.8856	1.47
6231	*SLC25A19*	2.2808	1.2413	0.88399	-0.45336	1.47
6232	*ABHD16A*	3.0143	1.8704	-0.47965	-0.70448	1.47
6233	*CCL28*	4.1655	0.27961	-0.04015	-1.4333	1.47
6234	*NPHS2*	5.3704	0.39367	-1.3593	-2.4168	1.47
6235	*GRK7*	2.6877	2.0384	-0.32162	-1.5103	1.47
6236	*TRAPPC9*	4.5678	0.31026	-0.47412	-1.0552	1.47
6237	*DEGS2*	2.85	2.353	-0.79987	-1.8908	1.47
6238	*DCDC2*	3.6523	0.5676	0.18274	-0.41522	1.47
6239	*MCMDC2*	4.0908	0.25563	0.05615	-1.1912	1.47
6240	*HAX1*	2.8148	2.2365	-0.64902	-1.2697	1.47
6241	*CBWD6*	3.2626	1.4385	-0.29885	-1.1797	1.47
6242	*HN1*	5.2309	0.2261	-1.0549	-1.296	1.47
6243	*GALR1*	3.7027	0.37308	0.32631	-3.4188	1.47
6244	*RGMB*	2.5809	2.5118	-0.69102	-1.4685	1.47
6245	*KCNAB3*	3.2725	1.0666	0.06244	-0.60543	1.47
6246	*JAG2*	2.1933	1.369	0.83917	-0.50087	1.47
6247	*PRMT1*	2.5082	1.8413	0.05143	-0.58005	1.47
6248	*MIB2*	3.0987	0.71068	0.59146	-1.191	1.47
6249	*SHC1*	2.0428	1.6222	0.73576	0.42378	1.47

6250	*FRMD8*	4.7501	0.38184	-0.73155	-2.5414	1.47
6251	*C10orf91*	1.9743	1.2519	1.1737	-0.30939	1.47
6252	*PFKFB2*	2.1596	1.3582	0.88107	-1.3127	1.47
6253	*NKAIN1*	4.2496	0.33703	-0.18961	-0.34632	1.47
6254	*PCDHA8*	2.9446	1.1014	0.35085	-2.2136	1.47
6255	*TTC39B*	4.2125	1.5056	-1.3218	-2.1519	1.47
6256	*NEFL*	3.5248	0.80808	0.06312	-2.7627	1.47
6257	*TRIM44*	4.2421	1.1014	-0.94777	-3.0961	1.47
6258	*EPAS1*	2.92	0.86165	0.61334	-1.5635	1.46
6259	*MGAT5*	3.6265	0.86615	-0.0981	-2.669	1.46
6260	*WBSCR28*	2.8787	1.8635	-0.34777	-0.95423	1.46
6261	*NTPCR*	2.7174	1.895	-0.218	-1.4034	1.46
6262	*ITLN1*	2.3809	1.42	0.59348	-0.77346	1.46
6263	*ZNF705E*	4.8817	0.68351	-1.1713	-1.9748	1.46
6264	*KIAA0195*	4.5338	0.41626	-0.55741	-0.73834	1.46
6265	*OR10H3*	3.3495	1.0212	0.02183	-0.02738	1.46
6266	*KLF12*	3.1647	0.94627	0.2813	-1.522	1.46
6267	*WSCD1*	3.3965	0.63053	0.36374	-0.73708	1.46
6268	*ID3*	3.4146	0.56254	0.41311	0.047097	1.46
6269	*ZNF805*	2.7492	0.84092	0.80002	0.16779	1.46
6270	*TYW1*	3.9676	0.71917	-0.29699	-1.1791	1.46
6271	*TRIAP1*	5.6049	-0.1589	-1.0567	-1.1403	1.46
6272	*PQLC1*	4.3983	0.19404	-0.20434	-1.1322	1.46
6273	*DYDC1*	3.0189	1.5545	-0.18547	-0.48413	1.46
6274	*GMPPA*	5.2342	-0.37408	-0.47303	-2.4141	1.46
6275	*IQCF1*	1.9867	1.3632	1.0361	-0.16868	1.46
6276	*GABRG2*	4.7341	0.50722	-0.85542	-1.9408	1.46
6277	*CALML5*	3.5457	1.0371	-0.19706	-0.52949	1.46
6278	*SMDT1*	3.4941	1.3099	-0.41831	-2.9564	1.46
6279	*SF3B4*	3.8009	0.99599	-0.41231	-1.9075	1.46
6280	*LARP4*	3.6618	0.59723	0.12515	0.041717	1.46
6281	*IDO1*	2.2751	2.2624	-0.15437	-1.9287	1.46
6282	*C20orf202*	3.4063	0.77009	0.20613	-1.7691	1.46
6283	*CAMK2G*	4.3426	0.32946	-0.28961	-2.1018	1.46
6284	*TIMP1*	3.458	1.4318	-0.50754	-1.9213	1.46
6285	*C5orf15*	3.2181	0.6696	0.49392	0.29764	1.46
6286	*PRICKLE3*	3.607	0.55103	0.22289	0.20675	1.46
6287	*SNAP47*	4.2032	0.89502	-0.71819	-0.87395	1.46
6288	*STEAP1B*	2.2268	1.385	0.76817	-2.0632	1.46
6289	*CTSA*	2.9635	2.2358	-0.82081	-1.5703	1.46
6290	*NKX2-4*	4.1511	0.37448	-0.14736	-0.44353	1.46
6291	*BCAT1*	2.9683	1.5158	-0.1062	-1.3132	1.46
6292	*LIMS3*	3.7407	0.52529	0.11094	-0.46967	1.46
6293	*CST11*	2.9008	2.6703	-1.1945	-3.0779	1.46
6294	*MRFAP1*	4.5277	0.31569	-0.46716	-1.9727	1.46
6295	*ASMT*	3.6269	0.61161	0.13741	-0.0748	1.46

6296	*YY2*	4.224	0.32725	-0.17641	-1.1972	1.46
6297	*HSD17B1*	4.0342	0.67879	-0.33895	-1.0669	1.46
6298	*TEX13A*	3.311	0.89105	0.17146	-0.72515	1.46
6299	*PRR30*	3.9364	1.9579	-1.5212	-1.5255	1.46
6300	*HECW1*	3.0446	0.77323	0.55522	-1.5271	1.46
6301	*MAPRE2*	2.6235	2.0484	-0.29906	-1.659	1.46
6302	*GPR52*	4.5114	0.4092	-0.54786	-1.7044	1.46
6303	*AADACL2*	3.5087	0.53457	0.32938	-1.1836	1.46
6304	*ZBED2*	2.3147	1.638	0.41857	-0.89974	1.46
6305	*GJC1*	4.2514	0.446	-0.32689	-1.2075	1.46
6306	*SYNJ1*	5.2149	0.5653	-1.4104	-1.5781	1.46
6307	*FZD2*	2.6717	1.6078	0.0903	-1.632	1.46
6308	*ATPIF1*	2.4931	1.8217	0.05498	-1.5992	1.46
6309	*DLG3*	5.1789	0.67817	-1.4873	-2.7127	1.46
6310	*SIRT6*	3.0031	1.0431	0.32339	0.11495	1.46
6311	*AKR1D1*	3.4779	1.3365	-0.44506	-0.82326	1.46
6312	*ZNF524*	2.9252	1.5404	-0.09658	-0.3644	1.46
6313	*NSA2*	4.0303	2.3303	-1.9919	-2.4312	1.46
6314	*FAM175B*	3.2694	1.4547	-0.35641	-0.46767	1.46
6315	*TLL1*	2.9069	1.7544	-0.29571	-0.48701	1.46
6316	*ALOX12B*	3.9621	0.2261	0.1771	-0.88189	1.46
6317	*GPR87*	3.6607	0.49164	0.21281	-1.1907	1.46
6318	*ARMC4*	3.3791	2.2794	-1.2935	-1.747	1.46
6319	*NGFR*	2.7533	0.97486	0.63663	-1.3773	1.45
6320	*PGR*	3.7689	0.71281	-0.11939	-0.80713	1.45
6321	*OR52L1*	3.2205	2.187	-1.0452	-1.0614	1.45
6322	*MPP2*	5.0556	0.1106	-0.8046	-1.4878	1.45
6323	*BTN3A2*	4.2332	1.0184	-0.89059	-1.0681	1.45
6324	*SAMD5*	3.7821	1.0033	-0.42469	-1.9196	1.45
6325	*SLC25A47*	5.1325	-0.05534	-0.71717	-2.7546	1.45
6326	*SRSF4*	2.7527	1.2231	0.38398	-0.93707	1.45
6327	*ZNF688*	4.9996	-0.03366	-0.60641	-1.3016	1.45
6328	*CDC20*	3.7783	1.0426	-0.46228	-0.7647	1.45
6329	*OR13C8*	4.1173	0.51223	-0.27102	-0.61409	1.45
6330	*LAMC3*	2.2469	1.905	0.20556	-0.17982	1.45
6331	*ZC3H7B*	2.1895	1.567	0.60047	-0.13255	1.45
6332	*KLHL11*	3.2276	1.3741	-0.24474	-0.8597	1.45
6333	*WDR19*	2.9834	2.1682	-0.79525	-1.9758	1.45
6334	*ZMYM5*	3.0992	1.1009	0.15608	-0.35805	1.45
6335	*C5orf38*	3.5048	1.7874	-0.93621	-1.7459	1.45
6336	*PPM1M*	3.2275	0.76799	0.36049	-2.1947	1.45
6337	*INO80D*	4.4803	0.081586	-0.20681	-0.69124	1.45
6338	*ARFGEF1*	4.4196	0.81748	-0.88274	-1.773	1.45
6339	*NAP1L4*	3.4438	0.47547	0.43475	-1.1734	1.45
6340	*TCL1A*	3.5203	0.51211	0.32125	-0.70424	1.45
6341	*PSKH1*	3.0395	0.7993	0.51452	-0.01323	1.45

6342	*ADRA2A*	2.9697	1.0048	0.37868	0.29932	1.45
6343	*SPATA31D4*	3.7352	0.84707	-0.22963	-0.54811	1.45
6344	*NEK10*	3.5271	1.2851	-0.45989	-1.0777	1.45
6345	*GPHB5*	4.8361	-0.24019	-0.24401	-1.615	1.45
6346	*CTRB1*	3.154	2.5088	-1.3114	-1.7169	1.45
6347	*SIK3*	4.057	0.74438	-0.44998	-1.0645	1.45
6348	*FBXO5*	4.2891	0.26733	-0.20532	-1.8024	1.45
6349	*ERMP1*	2.3039	1.7345	0.31212	-0.04465	1.45
6350	*KIAA0319*	3.0113	0.73122	0.6071	-0.15855	1.45
6351	*NUTM1*	2.9706	1.1282	0.25012	-0.29396	1.45
6352	*SEPT11*	3.4946	1.5885	-0.73483	-1.257	1.45
6353	*SMARCD2*	3.1052	2.3698	-1.127	-1.8833	1.45
6354	*STX18*	4.1847	0.58179	-0.41857	-0.77624	1.45
6355	*C4orf32*	3.2238	1.391	-0.26747	-1.3125	1.45
6356	*IZUMO1R*	2.8259	2.1284	-0.60701	-1.1559	1.45
6357	*TMEM211*	2.7719	1.3823	0.19282	-1.3457	1.45
6358	*LCORL*	2.2086	1.0831	1.0549	-0.76728	1.45
6359	*TNFRSF6B*	2.8346	2.6257	-1.1138	-1.2975	1.45
6360	*GNAO1*	4.3104	0.51695	-0.48085	-0.83621	1.45
6361	*DESI1*	3.7324	0.68075	-0.06678	-0.3361	1.45
6362	*PRSS16*	4.0095	0.68971	-0.35445	-0.51301	1.45
6363	*CBLB*	4.0462	0.17117	0.12714	-2.1268	1.45
6364	*UBE2I*	4.1092	0.3755	-0.14048	-0.94252	1.45
6365	*PIRT*	3.8458	0.75122	-0.25314	-1.0838	1.45
6366	*MLH3*	2.6946	0.86859	0.78013	-0.64632	1.45
6367	*P2RY4*	2.2787	1.667	0.39718	-1.7364	1.45
6368	*COMP*	4.0849	1.1004	-0.84261	-1.4183	1.45
6369	*KIAA1045*	4.4397	1.0882	-1.186	-1.9302	1.45
6370	*SAA4*	2.1191	1.549	0.67352	-1.7113	1.45
6371	*PIBF1*	3.2788	0.61791	0.44395	-0.56564	1.45
6372	*MRPS22*	4.4107	0.34029	-0.41131	-0.7873	1.45
6373	*PKN1*	2.8422	1.5545	-0.05809	-0.25339	1.45
6374	*POLR3GL*	2.8669	0.76148	0.70943	-0.17393	1.45
6375	*DRD4*	2.3231	1.6024	0.41185	-0.70626	1.45
6376	*C17orf75*	3.5481	1.1122	-0.32471	-0.82174	1.45
6377	*IQCJ-SCHIP1*	3.8666	0.68028	-0.21138	-1.0016	1.45
6378	*ZKSCAN3*	3.7126	0.97904	-0.35641	-1.0323	1.45
6379	*USHBP1*	2.5458	1.0991	0.69008	-0.24548	1.44
6380	*CSNK2A1*	3.8868	1.1926	-0.74471	-0.87382	1.44
6381	*ATP2C2*	2.2265	2.0396	0.06853	-1.3847	1.44
6382	*JKAMP*	3.3179	1.069	-0.05241	-0.22114	1.44
6383	*ARMC1*	2.398	1.0134	0.92298	-1.3988	1.44
6384	*IER3*	3.0514	1.7127	-0.42994	-0.56961	1.44
6385	*SLC36A1*	2.7119	0.9875	0.63384	-1.3821	1.44
6386	*DDX50*	4.2096	0.31183	-0.18898	-2.1902	1.44
6387	*ANO3*	3.0218	1.3684	-0.05776	-0.95	1.44

6388	*INSR*	2.3975	1.9387	-0.0048	-1.5956	1.44
6389	*USP46*	4.2476	0.33116	-0.2481	-0.71493	1.44
6390	*CTNND1*	3.8178	0.54869	-0.03639	-2.2517	1.44
6391	*OR2B2*	2.8822	1.6876	-0.23983	-0.46371	1.44
6392	*FA2H*	4.296	0.33483	-0.30188	-1.2805	1.44
6393	*SBF1*	2.3646	1.074	0.88948	-3.3818	1.44
6394	*RASGRP2*	2.5153	1.7295	0.08307	-2.0028	1.44
6395	*TEX38*	2.6432	2.5728	-0.88877	-1.5863	1.44
6396	*GHITM*	5.2371	-0.01034	-0.90001	-1.1507	1.44
6397	*TSNAXIP1*	2.8647	1.6748	-0.21304	-1.2571	1.44
6398	*NKHD1-EIF4EBP*	3.5805	0.40753	0.3381	0.063283	1.44
6399	*ATG4C*	4.8072	0.040213	-0.5221	-1.6452	1.44
6400	*HDHD3*	4.5642	1.3724	-1.6115	-3.1273	1.44
6401	*ZNF212*	4.9952	0.40466	-1.0756	-1.5039	1.44
6402	*ZNF510*	2.9171	0.79678	0.60886	-1.7232	1.44
6403	*METTL25*	3.7212	1.9912	-1.3902	-2.1334	1.44
6404	*SLC22A16*	4.0671	0.27872	-0.02488	-0.58152	1.44
6405	*KCNK13*	2.4621	1.4484	0.41022	-0.5591	1.44
6406	*KLHL20*	3.9353	0.61455	-0.2293	-0.46755	1.44
6407	*ZFP28*	3.0887	1.2589	-0.0274	-2.1489	1.44
6408	*SPR*	3.9079	0.4561	-0.04469	-0.16974	1.44
6409	*TAS2R10*	2.5184	1.5566	0.24349	-1.5888	1.44
6410	*STARD6*	3.7096	0.86903	-0.26068	-2.5354	1.44
6411	*ACVRL1*	3.4748	1.0393	-0.19658	-1.5781	1.44
6412	*CTTN*	2.6692	2.2901	-0.64186	-1.3346	1.44
6413	*MYO1D*	3.7466	0.53623	0.03435	-0.10838	1.44
6414	*CLNS1A*	4.016	0.87013	-0.56924	-2.1044	1.44
6415	*ZSCAN20*	3.7744	0.63096	-0.08868	-2.1709	1.44
6416	*CTNNA1*	5.007	1.1996	-1.8906	-1.9622	1.44
6417	*FARSA*	3.1316	1.4419	-0.25953	-0.93329	1.44
6418	*FKBP6*	2.0323	1.7965	0.48483	-0.62596	1.44
6419	*RBFOX3*	2.4897	0.91386	0.90965	-1.9304	1.44
6420	*CD82*	3.3634	0.61278	0.337	-0.52458	1.44
6421	*OSER1*	5.5545	-0.39121	-0.85107	-1.8154	1.44
6422	*TRIM49B*	2.5723	1.547	0.19251	-1.0619	1.44
6423	*DUSP4*	5.1102	-0.26208	-0.53661	-1.0283	1.44
6424	*HDAC3*	3.5431	0.98637	-0.21797	-1.8575	1.44
6425	*C1orf198*	2.5107	1.7279	0.07189	-0.28742	1.44
6426	*IL17RA*	3.5824	1.2017	-0.47447	-1.3818	1.44
6427	*KRTAP19-3*	3.8705	0.22299	0.21603	0.18322	1.44
6428	*NIPSNAP3A*	2.4389	1.6008	0.26973	-0.24738	1.44
6429	*NELL1*	2.9549	1.6887	-0.33488	-1.63	1.44
6430	*C1QL4*	4.1542	0.15426	0.00025	-0.57147	1.44
6431	*CAMLG*	2.8384	0.76951	0.69978	-0.74572	1.44
6432	*RDH12*	1.8312	1.3037	1.1725	-2.1848	1.44
6433	*NCF4*	4.8094	0.88398	-1.3865	-1.8642	1.44

6434	*NDUFAF7*	2.3586	1.4492	0.49894	-1.3818	1.44
6435	*RGPD1*	3.044	1.246	0.01674	-0.43292	1.44
6436	*BDH1*	2.0299	1.7551	0.52168	-1.0528	1.44
6437	*HSPB3*	5.1254	0.34077	-1.1595	-3.2662	1.44
6438	*ESYT1*	4.263	0.17607	-0.1331	-1.8645	1.44
6439	*DLX5*	3.1397	0.95926	0.2061	-0.71684	1.44
6440	*ILK*	5.7	0.2124	-1.6085	-1.6112	1.43
6441	*ARMCX3*	2.3831	1.3488	0.57086	0.45952	1.43
6442	*CASP1*	3.7384	0.5829	-0.01883	-0.47557	1.43
6443	*GNPAT*	4.6787	0.99391	-1.371	-1.4429	1.43
6444	*SELL*	3.8172	1.2006	-0.71622	-2.0384	1.43
6445	*MACROD2*	3.6367	1.4473	-0.78325	-0.8191	1.43
6446	*SOX6*	2.4813	1.2254	0.59281	-0.07202	1.43
6447	*PDE9A*	3.8302	0.35573	0.1134	-0.67221	1.43
6448	*ZNF629*	2.2278	2.0923	-0.02105	-1.0003	1.43
6449	*XPOT*	3.3823	0.6751	0.24138	-0.31191	1.43
6450	*SOCS4*	2.7032	2.197	-0.60147	-1.478	1.43
6451	*NPC1L1*	3.5635	0.51516	0.21977	-3.1918	1.43
6452	*RC3H2*	3.3211	0.73927	0.23758	-0.60402	1.43
6453	*P2RY2*	4.7844	1.0934	-1.5806	-2.312	1.43
6454	*ARHGDIB*	2.7271	1.4338	0.13599	-0.5731	1.43
6455	*TAF9B*	1.9063	1.4368	0.95354	0.76184	1.43
6456	*CACNG8*	4.743	-0.00816	-0.43841	-2.0351	1.43
6457	*OSGIN2*	2.9311	1.3587	0.00628	-0.08851	1.43
6458	*CT47A9*	4.4617	0.066291	-0.23223	-0.89429	1.43
6459	*BCHE*	2.0659	1.623	0.60677	-0.91862	1.43
6460	*CSAG3*	4.3679	0.56073	-0.63328	-1.9891	1.43
6461	*ELF2*	2.9966	1.5007	-0.20249	-1.2577	1.43
6462	*CLU*	3.1645	0.951	0.17837	-1.5855	1.43
6463	*BHLHA9*	2.4865	1.4461	0.35921	0.258	1.43
6464	*BCOR*	4.3751	0.47286	-0.55741	-2.9354	1.43
6465	*SPOCK2*	3.0178	1.5141	-0.24164	-0.28073	1.43
6466	*ST18*	3.536	3.0486	-2.2951	-2.458	1.43
6467	*RASL11B*	3.3219	0.84494	0.12253	-1.1511	1.43
6468	*PSG6*	1.756	1.7431	0.78918	-0.47273	1.43
6469	*RAB8A*	2.9813	0.83525	0.4696	0.10049	1.43
6470	*HLCS*	5.3065	-0.33116	-0.68934	-2.255	1.43
6471	*CDX1*	3.1251	0.88257	0.27826	-1.6158	1.43
6472	*SLC26A1*	3.4066	1.0746	-0.19808	-1.6711	1.43
6473	*TRIM68*	1.9554	1.433	0.89321	-1.4747	1.43
6474	*MOGAT1*	3.3242	2.3313	-1.3743	-1.7945	1.43
6475	*CISD3*	5.5391	-0.34533	-0.91283	-3.887	1.43
6476	*HIF1A*	2.1333	1.9632	0.18357	-2.4227	1.43
6477	*TXLNB*	5.577	0.40726	-1.7044	-1.9168	1.43
6478	*GOLGA4*	3.084	2.0606	-0.86494	-0.95545	1.43
6479	*PROK2*	4.1176	1.289	-1.127	-1.3523	1.43

6480	*MON1B*	3.7903	0.36827	0.12068	-1.808	1.43
6481	*ARHGAP18*	2.5826	2.3504	-0.65378	-1.0087	1.43
6482	*ZNF227*	5.3718	-0.28704	-0.80594	-1.2797	1.43
6483	*DDX31*	3.6557	0.38148	0.2411	-0.44353	1.43
6484	*TTC31*	4.1422	0.62538	-0.48982	-1.7306	1.43
6485	*POMZP3*	3.3189	0.53423	0.42428	-1.9567	1.43
6486	*PUS3*	4.2089	0.18739	-0.11939	-1.4483	1.43
6487	*PYGM*	3.6437	0.50718	0.12579	-1.9945	1.43
6488	*NUDT9*	3.6987	0.9968	-0.41987	-5.3559	1.43
6489	*CDY2A*	3.8305	0.23536	0.20867	-0.11543	1.42
6490	*C3orf14*	3.8952	0.21667	0.16246	-0.67874	1.42
6491	*UGGT1*	2.6181	1.4502	0.20594	-0.73202	1.42
6492	*MBD3L1*	2.4086	1.7317	0.13367	-0.41307	1.42
6493	*SPATA31A1*	4.304	0.48005	-0.5101	-1.1043	1.42
6494	*CLCF1*	2.7969	1.1387	0.33821	-1.1872	1.42
6495	*ATG16L1*	2.833	2.2076	-0.76857	-1.1164	1.42
6496	*BRWD3*	3.5414	0.42209	0.30839	-1.748	1.42
6497	*SPATA31A3*	4.6933	0.10802	-0.53086	-0.76055	1.42
6498	*NADK2*	5.121	-0.30496	-0.54559	-2.1463	1.42
6499	*PLAG1*	3.3131	2.1847	-1.2277	-1.6876	1.42
6500	*MRPL40*	4.385	1.0108	-1.1263	-1.8778	1.42
6501	*HPSE2*	3.6837	0.51031	0.07429	-0.11897	1.42
6502	*IL13RA2*	3.4419	0.76799	0.05813	0.023338	1.42
6503	*AURKAIP1*	2.4882	0.95328	0.8264	-2.3013	1.42
6504	*MAGEA2B*	3.0958	0.73418	0.43717	-1.613	1.42
6505	*ATG14*	4.6431	0.4011	-0.77755	-0.98951	1.42
6506	*SATB1*	3.099	0.82348	0.34374	-0.58305	1.42
6507	*DENND4B*	2.0659	1.9129	0.28548	0.23049	1.42
6508	*L3MBTL3*	4.1878	0.16544	-0.08915	-0.98719	1.42
6509	*FBXL21*	3.737	0.89212	-0.36582	-1.7802	1.42
6510	*ZNF221*	2.328	1.5748	0.36026	0.28019	1.42
6511	*ANKRD60*	3.7649	1.0616	-0.56365	-1.205	1.42
6512	*LTB4R2*	3.6395	0.40797	0.21504	-0.63002	1.42
6513	*OR2W3*	3.2725	1.4206	-0.43077	-1.3085	1.42
6514	*ACBD4*	2.8526	1.8665	-0.45727	-0.99699	1.42
6515	*FIBCD1*	4.4043	-0.06913	-0.07367	-1.5194	1.42
6516	*FICD*	3.004	0.87216	0.3849	-1.9375	1.42
6517	*PROL1*	2.0218	1.885	0.35392	-1.2405	1.42
6518	*LHX3*	4.2807	0.73657	-0.75896	-0.83411	1.42
6519	*CTAGE1*	4.079	1.1775	-0.99958	-1.714	1.42
6520	*CACNG5*	3.319	0.98984	-0.0525	-0.96962	1.42
6521	*DIXDC1*	1.7727	1.296	1.1873	-0.47386	1.42
6522	*PVRIG*	2.7029	1.3918	0.16118	-1.1226	1.42
6523	*C19orf18*	3.3411	1.9197	-1.0051	-1.9474	1.42
6524	*NRIP3*	5.5302	-0.62306	-0.65191	-1.377	1.42
6525	*UBQLN4*	4.2545	0.019125	-0.01915	-1.5304	1.42

6526	*SPOCD1*	4.0573	0.67787	-0.48085	-1.2629	1.42
6527	*KLHL9*	2.3518	0.96068	0.94137	-0.49215	1.42
6528	*ZNF676*	3.3531	1.5137	-0.61579	-1.1689	1.42
6529	*PGBD3*	1.9967	1.4038	0.85027	0.43945	1.42
6530	*KIAA1614*	5.0434	0.37485	-1.1685	-1.1925	1.42
6531	*PIGT*	3.4444	0.66709	0.13813	-0.34516	1.42
6532	*CD93*	3.4587	0.98997	-0.19915	-1.4633	1.42
6533	*IMPA1*	2.5441	2.4377	-0.73244	-1.2728	1.42
6534	*WFS1*	2.5278	2.4373	-0.71626	-2.4172	1.42
6535	*GINS4*	3.851	0.79457	-0.39711	-0.53271	1.42
6536	*FTCD*	2.3837	1.1841	0.6799	-0.87898	1.42
6537	*CIART*	4.5341	-0.1097	-0.17679	-0.60083	1.42
6538	*DLL4*	3.3087	0.93597	0.00239	-1.2716	1.42
6539	*ZNF705D*	1.9408	1.8021	0.50377	0.26655	1.42
6540	*UBE2D4*	3.3114	0.77207	0.1628	0.068922	1.42
6541	*KRTAP29-1*	1.6302	1.6052	1.0106	-0.76088	1.42
6542	*CCNO*	2.6448	1.3984	0.20224	-1.6885	1.42
6543	*ZNF564*	4.0614	0.11498	0.06868	0.041724	1.42
6544	*KCTD21*	2.7307	2.4417	-0.92769	-1.4906	1.41
6545	*RSG1*	4.0801	1.7365	-1.5719	-1.9455	1.41
6546	*ZNF780A*	2.8808	1.4594	-0.09668	-1.9284	1.41
6547	*NACC2*	2.8233	1.2078	0.21184	-2.3154	1.41
6548	*GPR183*	2.3865	1.9757	-0.11939	-0.70137	1.41
6549	*FAM212A*	4.6681	0.026872	-0.4533	-2.8807	1.41
6550	*PDIA3*	3.2595	1.3077	-0.32773	-0.38767	1.41
6551	*GPX2*	4.8609	-0.30378	-0.31787	-0.72246	1.41
6552	*C9orf152*	4.3124	0.068008	-0.14163	-0.70506	1.41
6553	*MTRNR2L1*	3.0275	-0.20181			1.41
6554	*LACTBL1*	2.4874	1.2317	0.51899	0.083394	1.41
6555	*BFAR*	2.6168	2.0744	-0.4541	-2.4633	1.41
6556	*CD302*	3.0663	1.3494	-0.17979	-0.79449	1.41
6557	*COA5*	2.9742	0.98388	0.27774	-1.338	1.41
6558	*LRFN2*	2.7409	2.0388	-0.54417	-0.93112	1.41
6559	*TNK1*	3.737	0.31825	0.17974	-1.398	1.41
6560	*SOWAHD*	3.769	1.5784	-1.1125	-1.3033	1.41
6561	*TMEM150B*	4.3147	0.18955	-0.27034	-0.31494	1.41
6562	*FER1L6*	2.5308	0.85717	0.84552	-0.32728	1.41
6563	*PGA5*	3.0644	2.2639	-1.0949	-3.0564	1.41
6564	*PAX5*	3.7314	0.77385	-0.2723	-1.5294	1.41
6565	*CPB1*	3.8572	0.38765	-0.01245	-0.46228	1.41
6566	*FBXL16*	4.228	0.062296	-0.0579	-0.90991	1.41
6567	*DPYD*	3.7682	0.90769	-0.44353	-1.0153	1.41
6568	*ZCCHC13*	2.7739	0.76069	0.69741	-0.66767	1.41
6569	*CAPN11*	4.463	0.31952	-0.55134	-1.6289	1.41
6570	*SULT4A1*	2.0541	1.2815	0.89532	-0.39136	1.41
6571	*DCAF11*	2.5035	1.4798	0.24742	0.22935	1.41

6572	*SLC36A2*	3.8006	0.42348	0.00646	-0.98938	1.41
6573	*CLRN3*	3.3774	0.95947	-0.10633	-0.9272	1.41
6574	*DEFB127*	4.2256	0.34738	-0.34292	-0.86007	1.41
6575	*ZDHHC2*	3.9334	0.99324	-0.69683	-1.0393	1.41
6576	*LRP1B*	3.2726	0.60262	0.3544	-1.6131	1.41
6577	*GH2*	3.1706	1.1915	-0.13255	-0.54274	1.41
6578	*SULT6B1*	5.182	0.10417	-1.0571	-1.4485	1.41
6579	*HIST2H4B*	2.6521	1.2047	0.37149	0.18586	1.41
6580	*CLDN6*	3.679	0.49337	0.05557	-1.5369	1.41
6581	*B2M*	2.7146	0.87211	0.64115	-0.3159	1.41
6582	*USP15*	1.7071	1.2758	1.2447	0.11435	1.41
6583	*ZNF609*	4.7995	-0.13528	-0.43664	-3.182	1.41
6584	*FUT1*	1.8956	1.8521	0.47987	-0.00826	1.41
6585	*SLC22A20*	2.4221	1.6321	0.17237	-1.7018	1.41
6586	*SLC25A21*	4.1141	0.2034	-0.09112	-0.12613	1.41
6587	*MTF2*	2.8448	0.73877	0.64259	-0.53488	1.41
6588	*NXPH1*	2.3126	2.2111	-0.29801	-0.32699	1.41
6589	*ERCC3*	5.0242	0.073424	-0.87256	-1.202	1.41
6590	*LRRC28*	2.1494	1.2979	0.77774	-0.79558	1.41
6591	*TCEA3*	4.2196	0.27524	-0.26992	-0.53029	1.41
6592	*MAP2K1*	4.1261	1.3179	-1.2198	-1.6828	1.41
6593	*MAP7D3*	2.8304	0.87631	0.51694	-0.21491	1.41
6594	*SRD5A3*	4.3772	0.008092	-0.16176	-0.95802	1.41
6595	*CELF3*	3.5965	1.4578	-0.83095	-1.6617	1.41
6596	*EGR3*	3.0972	1.9731	-0.84748	-1.4538	1.41
6597	*KPNA2*	4.6021	0.14607	-0.52557	-1.9295	1.41
6598	*P2RY10*	3.6173	1.0718	-0.46698	-3.2255	1.41
6599	*DIO2*	2.3357	1.1549	0.7309	-0.20874	1.41
6600	*SHISA7*	2.9418	1.4075	-0.12908	-0.13539	1.41
6601	*TRIP10*	2.8578	1.4816	-0.11939	-1.4824	1.41
6602	*TMPRSS9*	2.0645	1.2786	0.87648	0.71892	1.41
6603	*FAT2*	2.9445	0.99307	0.28057	-0.01923	1.41
6604	*PTPRB*	4.7227	0.41904	-0.92375	-1.5213	1.41
6605	*CADPS*	2.17	1.7447	0.30309	-1.3978	1.41
6606	*KHDC3L*	4.6864	-0.06132	-0.4073	-1.082	1.41
6607	*AMPD2*	3.8348	0.62779	-0.24578	-1.478	1.41
6608	*ELK4*	3.2717	1.0856	-0.14323	-0.17488	1.40
6609	*OSGEP*	4.1231	0.43117	-0.34067	-0.42708	1.40
6610	*ADAM29*	3.4225	1.1957	-0.40465	-0.48156	1.40
6611	*FAM73A*	3.6677	0.59177	-0.04593	-1.5167	1.40
6612	*WDR53*	4.4125	0.61018	-0.80946	-1.3165	1.40
6613	*UGT2B11*	2.636	1.5469	0.03025	-0.95221	1.40
6614	*ZGRF1*	2.6116	0.83414	0.76682	-0.85612	1.40
6615	*UNC5C*	4.1165	0.083386	0.01252	-0.42859	1.40
6616	*OR10J1*	3.5685	1.7481	-1.1043	-2.5403	1.40
6617	*CYTH3*	2.3919	2.0138	-0.19357	-1.9089	1.40

6618	*ZCCHC2*	4.5652	0.60331	-0.95658	-1.6528	1.40
6619	*CPA6*	2.7294	2.287	-0.80517	-1.864	1.40
6620	*TXK*	5.0516	-0.31508	-0.52552	-1.088	1.40
6621	*AP3B2*	4.2809	0.077878	-0.14796	-1.3074	1.40
6622	*SUSD4*	3.4664	0.52804	0.2159	-1.57	1.40
6623	*SLC5A12*	3.2547	1.4578	-0.50265	-2.3065	1.40
6624	*GPR153*	3.4637	1.6038	-0.85771	-1.8896	1.40
6625	*PEG10*	3.4117	0.55896	0.23877	-2.217	1.40
6626	*PDZK1*	4.6483	0.83615	-1.2755	-1.8908	1.40
6627	*RASGRP3*	2.2335	1.0201	0.95355	-1.4501	1.40
6628	*ACOX1*	3.5736	2.1228	-1.4894	-1.8604	1.40
6629	*SNAI3*	3.2481	2.2123	-1.2543	-3.0045	1.40
6630	*TMEM88*	4.5121	0.85593	-1.162	-2.7568	1.40
6631	*REPIN1*	2.6885	1.1318	0.3848	-0.26018	1.40
6632	*UGT2A1*	2.3418	1.4898	0.37261	0.023843	1.40
6633	*IL2RG*	3.6888	2.0532	-1.5382	-1.6564	1.40
6634	*SLC47A2*	3.3086	0.7297	0.16515	-0.78313	1.40
6635	*SIGLEC7*	3.9626	0.82252	-0.58227	-0.89052	1.40
6636	*CT83*	4.6174	0.13442	-0.55002	-1.6921	1.40
6637	*SLC25A40*	3.6138	2.018	-1.4306	-1.6004	1.40
6638	*RALGAPA2*	2.8322	1.599	-0.23042	-1.6128	1.40
6639	*RPS4Y1*	3.3529	0.83413	0.01369	-0.83856	1.40
6640	*PTDSS1*	3.5973	1.1942	-0.59099	-1.4775	1.40
6641	*DPH7*	3.7368	0.61455	-0.15258	-2.9706	1.40
6642	*NWD1*	3.4337	0.89283	-0.12977	-1.0413	1.40
6643	*FAM209A*	2.7506	1.4954	-0.04931	-1.1989	1.40
6644	*ITIH3*	4.2542	0.16594	-0.22378	-0.85739	1.40
6645	*WASF3*	3.6472	0.3453	0.20381	-0.66253	1.40
6646	*TXNRD2*	2.9776	0.70533	0.51104	-4.3821	1.40
6647	*BTN2A2*	2.9323	1.3376	-0.07721	-0.88507	1.40
6648	*CST1*	3.2168	1.0314	-0.05706	-2.0885	1.40
6649	*OR5D18*	2.2872	1.5523	0.3515	-0.90084	1.40
6650	*MAGEA4*	3.7864	0.59057	-0.18682	-1.1196	1.40
6651	*LILRA2*	3.7587	0.39597	0.03546	-0.47964	1.40
6652	*GALP*	3.0917	1.5656	-0.46745	-2.0838	1.40
6653	*PPP4R1*	1.9066	1.1643	1.1182	-1.1388	1.40
6654	*DAW1*	4.4136	0.66956	-0.89424	-0.92553	1.40
6655	*STX5*	3.7728	0.79456	-0.37845	-1.5671	1.40
6656	*RPL36A*	1.4944	1.4887	1.2058	-2.017	1.40
6657	*KIAA1210*	3.8299	0.28562	0.073	-0.24946	1.40
6658	*PTGER2*	3.5009	0.54097	0.1466	-0.742	1.40
6659	*IDH3B*	2.9388	1.1471	0.10177	-1.4485	1.40
6660	*HIST1H3D*	4.321	0.12246	-0.25589	-1.5876	1.40
6661	*TEKT5*	4.6395	1.3199	-1.7743	-1.9125	1.40
6662	*ELANE*	3.2047	0.94323	0.03632	-0.11697	1.39
6663	*LCE1F*	3.4249	0.47297	0.28463	-1.0699	1.39

6664	*STARD3NL*	1.7301	1.2749	1.1771	-0.82656	1.39
6665	*TMEM246*	2.5553	1.0828	0.54388	-1.9558	1.39
6666	*BPI*	4.7843	0.10613	-0.70993	-1.2426	1.39
6667	*PPM1F*	3.1418	0.82682	0.21136	-0.9311	1.39
6668	*FDXR*	3.5495	0.77657	-0.14689	-1.7397	1.39
6669	*PRPS1L1*	2.9223	0.93595	0.32086	-1.3016	1.39
6670	*RGS17*	3.1844	0.50316	0.49141	-2.1089	1.39
6671	*DOLPP1*	3.7691	0.62632	-0.21666	-0.89401	1.39
6672	*FBXO36*	3.7241	1.206	-0.75275	-0.95577	1.39
6673	*SLC2A2*	2.8812	1.1879	0.1078	-1.6494	1.39
6674	*RHEB*	2.9906	2.1603	-0.97599	-2.0682	1.39
6675	*TFE3*	4.4357	0.82008	-1.0811	-2.095	1.39
6676	*XKR6*	3.4618	1.0236	-0.31166	-1.4174	1.39
6677	*PRKACG*	2.7902	1.9788	-0.59676	-0.95767	1.39
6678	*CCDC87*	3.1529	0.59135	0.42556	-0.64956	1.39
6679	*LRRC47*	5.1937	0.79593	-1.8202	-2.0337	1.39
6680	*SPATA12*	1.8494	1.3526	0.96644	-1.6524	1.39
6681	*FTSJ3*	3.7677	0.81861	-0.41812	-2.1332	1.39
6682	*HSD17B6*	3.8063	0.63825	-0.27669	-1.2262	1.39
6683	*ISOC2*	4.0223	1.1842	-1.0388	-1.2864	1.39
6684	*APOO*	3.1962	0.57788	0.39221	0.35272	1.39
6685	*TSHZ1*	2.6621	0.90894	0.59472	0.2083	1.39
6686	*RPS6KB1*	2.5112	0.95708	0.69731	-1.6173	1.39
6687	*ERLEC1*	3.3706	0.45364	0.34102	-0.45692	1.39
6688	*SLC27A5*	2.75	1.8325	-0.41758	-1.5956	1.39
6689	*GRAMD1A*	5.2663	-0.41285	-0.68896	-1.3169	1.39
6690	*DEF6*	4.1105	0.35673	-0.30375	-1.8007	1.39
6691	*KRTAP4-2*	3.9765	0.64511	-0.45886	-0.78048	1.39
6692	*SNX9*	3.6913	0.87559	-0.40515	-1.2567	1.39
6693	*GAGE12J*	3.0531	1.6812	-0.57301	-0.71009	1.39
6694	*FSCN3*	2.815	2.1577	-0.81256	-1.507	1.39
6695	*MR1*	4.6778	0.057706	-0.57579	-1.217	1.39
6696	*PAN2*	3.3363	0.49068	0.33245	-0.83773	1.39
6697	*CDK5R1*	3.5253	0.39971	0.23428	-1.691	1.39
6698	*MROH1*	2.9241	1.1364	0.09875	-0.04564	1.39
6699	*NEU2*	3.0322	0.60487	0.52138	-0.51689	1.39
6700	*SERP1*	1.9041	1.4992	0.75411	0.30481	1.39
6701	*SLCO4C1*	3.1088	0.77732	0.27048	-1.5715	1.39
6702	*USP17L28*	5.6162	-0.62833	-0.83138	-2.9308	1.39
6703	*PRSS57*	1.7256	1.606	0.82447	-0.89595	1.39
6704	*UTS2B*	2.2042	1.8991	0.05183	-1.2884	1.39
6705	*IFNG*	2.8408	2.7438	-1.4302	-2.3273	1.38
6706	*TMEM223*	4.2979	0.095183	-0.23941	-0.7423	1.38
6707	*LCP1*	3.5813	0.51261	0.05963	-0.57542	1.38
6708	*TPH2*	2.2094	1.5929	0.35115	-2.3602	1.38
6709	*USP45*	4.7447	1.0215	-1.6129	-2.5008	1.38

6710	*PANK3*	3.3561	0.69192	0.10415	0.01524	1.38
6711	*OR6C65*	3.9095	1.1588	-0.91647	-1.4702	1.38
6712	*MLF1*	5.4541	-0.3342	-0.96857	-2.1312	1.38
6713	*D2HGDH*	3.841	0.77621	-0.46589	-1.7149	1.38
6714	*PAK3*	4.2922	0.73444	-0.87536	-2.1848	1.38
6715	*CNGA2*	3.5061	1.7719	-1.127	-2.4208	1.38
6716	*KLRC3*	1.8217	1.4131	0.91566	-1.0142	1.38
6717	*DIRC1*	2.2148	1.9547	-0.02137	-0.88465	1.38
6718	*TAS2R42*	3.926	0.65032	-0.42892	-0.61908	1.38
6719	*HGSNAT*	4.1951	0.069412	-0.11762	-1.0014	1.38
6720	*PCBP2*	4.3426	0.88958	-1.0858	-1.6676	1.38
6721	*FHAD1*	4.4564	0.056806	-0.36689	-0.4978	1.38
6722	*DDX6*	3.3524	0.66954	0.12418	-0.76253	1.38
6723	*PQBP1*	3.4293	1.0607	-0.34416	-1.0386	1.38
6724	*ELP5*	3.8882	0.68323	-0.42585	-0.79443	1.38
6725	*HS3ST2*	4.0281	0.69218	-0.57479	-1.5552	1.38
6726	*ZMYND12*	2.8623	1.3678	-0.08472	-0.65806	1.38
6727	*FOXN2*	4.2073	0.38153	-0.44353	-1.6469	1.38
6728	*BABAM1*	3.6452	0.35364	0.14603	-2.3086	1.38
6729	*NDUFA7*	2.6636	1.2814	0.19908	-1.714	1.38
6730	*RPS6KA1*	4.5055	-0.16749	-0.19457	-1.246	1.38
6731	*FGD5*	3.7151	0.3616	0.06576	-0.44353	1.38
6732	*FAM57A*	3.9911	0.45577	-0.30454	-1.5401	1.38
6733	*RTKN*	4.2604	0.54286	-0.66194	-1.479	1.38
6734	*ASCL1*	3.8264	0.61569	-0.30078	-1.9622	1.38
6735	*RAMP2*	2.5007	1.222	0.41695	0.35667	1.38
6736	*HMBOX1*	4.2454	0.59077	-0.69655	-1.5014	1.38
6737	*LOC100506422*	2.4528	1.1088	0.57743	-2.808	1.38
6738	*RBMY1A1*	3.79	1.4197	-1.0707	-2.7863	1.38
6739	*KIAA1143*	2.8469	1.1776	0.11411	-1.3285	1.38
6740	*KLHL4*	2.6817	1.3418	0.11502	-1.7613	1.38
6741	*NGRN*	3.4176	1.1139	-0.39313	-1.2158	1.38
6742	*TMEM179*	4.8554	-0.25927	-0.45804	-2.4817	1.38
6743	*PDLIM7*	4.4124	0.89123	-1.1659	-1.3738	1.38
6744	*SMARCAD1*	2.4495	1.4245	0.26293	-0.81761	1.38
6745	*COL23A1*	2.4905	1.7183	-0.07224	-1.0901	1.38
6746	*SPANXA2*	3.2712	1.3712	-0.50589	-1.2568	1.38
6747	*CNOT4*	1.9725	1.8286	0.33194	-0.29752	1.38
6748	*TECR*	4.9309	0.42935	-1.2284	-1.4794	1.38
6749	*RGAG4*	3.2779	1.0364	-0.18246	-1.1153	1.38
6750	*MYH9*	2.1116	1.8469	0.17326	-0.94121	1.38
6751	*ZC4H2*	1.9357	1.4878	0.70814	-0.98366	1.38
6752	*PXMP4*	3.9829	0.5581	-0.40961	-0.86229	1.38
6753	*MNT*	2.3936	1.184	0.55356	-0.44353	1.38
6754	*CNGB3*	3.0684	1.0311	0.03091	-0.28943	1.38
6755	*SLC15A2*	3.5466	1.1969	-0.61356	-0.99585	1.38

6756	*LMBRD2*	3.247	0.58375	0.29853	-1.2272	1.38
6757	*ZMAT3*	2.566	1.3018	0.2606	-0.44687	1.38
6758	*LYPD8*	4.6538	0.55611	-1.0819	-1.161	1.38
6759	*LBR*	5.4077	-0.07056	-1.2097	-1.6383	1.38
6760	*C5*	5.3263	-0.17629	-1.0228	-1.5152	1.38
6761	*MEX3C*	2.7517	1.6429	-0.26743	-0.64399	1.38
6762	*ARL4C*	2.1875	1.677	0.26174	-2.1859	1.38
6763	*TSSK1B*	3.3228	0.75389	0.04924	-0.91913	1.38
6764	*MICU3*	4.7149	0.001963	-0.59119	-2.1706	1.38
6765	*KLK13*	3.3421	1.0481	-0.26468	-0.54179	1.38
6766	*ALMS1*	4.8774	0.28022	-1.0328	-1.8157	1.37
6767	*MEIS3*	4.2505	0.18624	-0.31248	-1.1028	1.37
6768	*SLC16A5*	3.1472	1.9584	-0.9815	-1.2684	1.37
6769	*PPP1R32*	4.7171	0.30757	-0.90073	-2.0609	1.37
6770	*EFR3A*	2.8431	0.70788	0.5727	0.46963	1.37
6771	*AMMECR1L*	3.6889	1.2065	-0.77236	-1.3132	1.37
6772	*RNF114*	4.4836	-0.11406	-0.24665	-2.1185	1.37
6773	*ADGRV1*	4.0587	0.25015	-0.18612	-2.2379	1.37
6774	*AIFM2*	4.3514	0.55834	-0.78718	-2.4268	1.37
6775	*CTAGE9*	5.0133	-0.39277	-0.49805	-2.4933	1.37
6776	*OR9A2*	2.6221	0.90028	0.59986	-0.29054	1.37
6777	*SLFN13*	5.269	-0.23796	-0.90922	-1.0151	1.37
6778	*TTYH3*	4.7768	0.10845	-0.76352	-1.3955	1.37
6779	*GPSM3*	2.4971	1.3876	0.23583	0.059093	1.37
6780	*ATP1B4*	2.116	1.4925	0.51024	-1.0942	1.37
6781	*LRRN4*	2.212	1.5949	0.3109	-0.45563	1.37
6782	*ZNF486*	4.013	0.58265	-0.47821	-2.8463	1.37
6783	*SH2D1A*	1.932	1.6426	0.54262	0.21358	1.37
6784	*CCNI2*	4.1384	0.65248	-0.67378	-0.77409	1.37
6785	*CCDC82*	2.669	1.0877	0.36026	-1.2246	1.37
6786	*SPEM1*	2.4703	2.0822	-0.43572	-0.45505	1.37
6787	*TMEM9B*	3.2873	1.2751	-0.44661	-0.77418	1.37
6788	*STXBP1*	2.9209	2.1025	-0.90797	-1.2393	1.37
6789	*PARD3*	2.5934	2.1056	-0.5836	-0.73659	1.37
6790	*OR2T6*	2.3489	1.9779	-0.21208	-1.047	1.37
6791	*ERCC6L2*	3.3527	0.86882	-0.10743	-0.71479	1.37
6792	*ENTHD2*	5.7288	-0.77284	-0.84337	-1.1758	1.37
6793	*PLCL1*	3.3904	0.49027	0.23168	-0.59662	1.37
6794	*NAALAD2*	4.1051	1.937	-1.9302	-2.3257	1.37
6795	*C1orf195*	4.5766	-0.1707	-0.29411	-1.3702	1.37
6796	*OTC*	3.4056	1.4073	-0.70147	-1.2437	1.37
6797	*ZNF286A*	3.9953	0.35705	-0.24148	-0.26052	1.37
6798	*RP2*	3.2285	0.73632	0.1454	-1.3249	1.37
6799	*FAM163B*	2.9617	2.3484	-1.2007	-1.7561	1.37
6800	*C22orf39*	4.0559	1.4597	-1.4064	-1.7139	1.37
6801	*SCAP*	4.0806	0.81451	-0.78591	-1.4972	1.37

6802	*RNF169*	1.9286	1.8958	0.2839	-1.8181	1.37
6803	*HIST2H3C*	2.4714	1.0934	0.54272	-1.3805	1.37
6804	*WFDC11*	3.661	1.1369	-0.69059	-1.0232	1.37
6805	*LPAR2*	3.5831	0.32932	0.19435	-0.92629	1.37
6806	*FAM3B*	3.62	1.6618	-1.1759	-1.3517	1.37
6807	*SLC22A6*	1.5758	1.536	0.99288	-0.96047	1.37
6808	*ZRANB2*	4.498	-0.19465	-0.19935	-1.1717	1.37
6809	*NLGN4Y*	3.834	1.8066	-1.5376	-2.7587	1.37
6810	*ENTPD5*	4.635	-0.25181	-0.28173	-0.6981	1.37
6811	*ASB15*	3.099	0.95209	0.04951	-1.0638	1.37
6812	*LOC100129083*	4.5282	0.0776	-0.50548	-0.61167	1.37
6813	*WWC1*	2.1072	1.447	0.54576	-0.47739	1.37
6814	*ANKH*	2.6715	1.6079	-0.18132	-0.98459	1.37
6815	*ALOX15*	3.6526	1.274	-0.82951	-0.87719	1.37
6816	*NDUFS8*	3.3251	1.022	-0.25018	-1.72	1.37
6817	*PSMA4*	4.1764	1.1231	-1.2027	-2.8111	1.37
6818	*TMEM39B*	2.3377	1.285	0.47357	-1.7328	1.37
6819	*PRDM9*	3.0288	2.404	-1.3366	-1.7219	1.37
6820	*SLC2A12*	4.6669	0.95925	-1.5309	-1.9055	1.37
6821	*LZTS2*	2.2586	1.6872	0.14798	-0.36232	1.36
6822	*ASTL*	3.7077	1.7269	-1.3412	-2.7127	1.36
6823	*PAEP*	3.6127	0.30283	0.17653	-0.18778	1.36
6824	*FKBP11*	2.0848	1.2136	0.79268	-1.3513	1.36
6825	*GRAMD3*	4.2112	-0.05026	-0.07219	-0.91913	1.36
6826	*LEPROT*	3.8843	0.19156	0.01243	-0.15684	1.36
6827	*CDH19*	3.6067	0.83508	-0.35478	-0.78229	1.36
6828	*PPAPDC3*	3.4941	0.37342	0.21945	-2.6806	1.36
6829	*ZNF521*	2.7231	2.5065	-1.1427	-1.3088	1.36
6830	*SGTB*	3.0716	1.0357	-0.02062	-0.83101	1.36
6831	*KRT222*	3.033	1.8263	-0.77284	-2.0423	1.36
6832	*SEP15*	3.9385	0.63648	-0.48897	-2.1496	1.36
6833	*TM7SF3*	2.9073	0.71886	0.45962	-0.44353	1.36
6834	*FGL2*	3.2573	2.5518	-1.7235	-2.3684	1.36
6835	*CHN1*	2.2429	1.5572	0.28495	-0.19268	1.36
6836	*MFI2*	2.2121	1.8308	0.04151	-1.3128	1.36
6837	*GAS7*	4.5785	-0.15891	-0.33536	-1.3896	1.36
6838	*ANAPC2*	4.8695	0.31494	-1.1006	-1.4061	1.36
6839	*TM4SF18*	3.2716	0.56133	0.25068	-1.1303	1.36
6840	*CD320*	2.4606	1.681	-0.05837	-0.86735	1.36
6841	*NFS1*	2.816	1.525	-0.25832	-0.80909	1.36
6842	*TBC1D10B*	2.6725	2.6271	-1.2185	-1.8909	1.36
6843	*CACNA1A*	4.7244	-0.20023	-0.44353	-1.6917	1.36
6844	*ZNF354B*	4.7169	0.68464	-1.3209	-1.9734	1.36
6845	*CENPU*	3.2722	0.58691	0.22126	-1.5375	1.36
6846	*ARL6IP6*	4.5851	1.5565	-2.0629	-2.0954	1.36
6847	*OST4*	2.7378	1.184	0.15653	-0.13461	1.36

6848	*COBL*	2.535	1.8504	-0.30726	-0.90003	1.36
6849	*OR10J3*	1.7947	1.2483	1.035	-0.03157	1.36
6850	*PXMP2*	4.0174	0.11858	-0.05851	-0.17298	1.36
6851	*TIGD6*	4.8745	-0.34816	-0.44888	-1.329	1.36
6852	*PEAR1*	3.5597	0.32001	0.19714	-1.0863	1.36
6853	*IKZF1*	3.3666	0.36163	0.34808	-3.6506	1.36
6854	*ARRDC4*	2.3484	1.0177	0.7095	0.63999	1.36
6855	*PRKG1*	4.7415	0.37956	-1.0467	-1.9678	1.36
6856	*CD14*	4.8107	0.21086	-0.94759	-1.6469	1.36
6857	*EXTL1*	2.3661	0.87842	0.82897	-1.8591	1.36
6858	*VNN2*	3.1593	0.81936	0.09429	-2.4832	1.36
6859	*AKAP14*	2.5653	1.6927	-0.186	-0.49925	1.36
6860	*CD1A*	2.6607	0.81522	0.596	-0.81509	1.36
6861	*APC*	3.4671	1.0497	-0.44499	-0.81793	1.36
6862	*TMEM229A*	4.6913	-0.18175	-0.43776	-1.6682	1.36
6863	*LOC388849*	4.1392	0.71476	-0.78229	-1.7378	1.36
6864	*HIST1H4I*	3.9222	0.50064	-0.35205	-1.2304	1.36
6865	*ANKRD42*	2.3111	1.2808	0.47809	-1.2867	1.36
6866	*KDM6B*	3.7509	0.28744	0.03084	-1.1212	1.36
6867	*FPGT-TNNI3K*	3.763	0.79011	-0.48419	-0.76934	1.36
6868	*ADAM10*	3.3096	0.6633	0.0951	-2.0022	1.36
6869	*FABP1*	2.5793	0.97892	0.5094	0.12387	1.36
6870	*FABP6*	4.0383	0.25313	-0.22451	-1.5155	1.36
6871	*STRIP2*	3.8489	0.11518	0.10265	-0.87195	1.36
6872	*CLDN34*	2.1949	1.3291	0.54269	-2.72	1.36
6873	*RXRA*	2.6509	1.6964	-0.28084	-1.1442	1.36
6874	*SLC26A3*	3.1501	0.59304	0.32211	-1.4951	1.36
6875	*SOS1*	2.8474	1.4212	-0.20601	-1.729	1.35
6876	*SYT15*	4.0229	0.39939	-0.36006	-1.8462	1.35
6877	*CLDN10*	1.8822	1.3231	0.85686	-0.54287	1.35
6878	*ICA1L*	3.3434	0.45747	0.26124	-1.8604	1.35
6879	*CXorf40B*	2.4275	1.6285	0.00488	-0.86925	1.35
6880	*TBCEL*	3.3763	0.86685	-0.18411	-0.97102	1.35
6881	*RHCE*	2.8261	1.0307	0.20167	-0.33603	1.35
6882	*WNT9B*	1.9185	1.8295	0.31041	-0.12128	1.35
6883	*CHMP4A*	3.2889	1.6194	-0.85027	-1.2935	1.35
6884	*NUDT17*	2.9366	0.87361	0.24775	-1.2497	1.35
6885	*NCAPG*	2.8436	1.2401	-0.02598	-0.20681	1.35
6886	*C16orf52*	2.1982	1.5227	0.33645	0.038212	1.35
6887	*TNFSF12*	2.9288	0.65812	0.47012	-0.17105	1.35
6888	*C11orf24*	2.4149	1.2329	0.40906	-0.41454	1.35
6889	*MAGEA1*	2.5081	1.4411	0.10729	-0.04693	1.35
6890	*MOB3B*	2.6449	0.97036	0.44018	-0.97623	1.35
6891	*NEU4*	2.8866	0.86502	0.3027	-0.22067	1.35
6892	*FAS*	2.1044	1.7887	0.16109	-1.611	1.35
6893	*STXBP5L*	2.4011	2.0534	-0.40174	-1.0711	1.35

6894	*ZSCAN5B*	3.5947	0.45427	0.00369	-0.58305	1.35
6895	*MIER1*	4.1867	0.95064	-1.0853	-1.7829	1.35
6896	*ROCK2*	3.6475	0.79669	-0.39244	-1.6065	1.35
6897	*CELF4*	4.7406	0.52243	-1.2114	-1.481	1.35
6898	*ZNF17*	3.9415	0.07586	0.03288	-1.2007	1.35
6899	*GLTSCR1*	2.7353	2.2392	-0.92427	-1.481	1.35
6900	*IFNA8*	2.9198	1.6174	-0.48744	-1.1121	1.35
6901	*KRTAP2-1*	1.9562	1.9557	0.13743	-1.0436	1.35
6902	*NR0B1*	3.9015	0.34011	-0.19331	-1.856	1.35
6903	*ZFPL1*	2.7584	2.3416	-1.0528	-1.3162	1.35
6904	*C16orf47*	1.9675	1.1744	0.90495	-0.91793	1.35
6905	*LMO1*	3.328	0.77097	-0.05219	-0.7463	1.35
6906	*KRTAP13-1*	3.8858	0.46702	-0.30606	-2.04	1.35
6907	*TDO2*	1.7694	1.408	0.86936	-0.59952	1.35
6908	*CKM*	4.4956	-0.18133	-0.26854	-0.98864	1.35
6909	*BTBD6*	2.4771	0.94807	0.61992	-1.8248	1.35
6910	*KIR3DL1*	4.9908	-0.01817	-0.92764	-1.6085	1.35
6911	*EIF1AX*	3.3572	0.40781	0.27912	-1.6656	1.35
6912	*DCLK1*	3.267	0.45371	0.3233	-1.9378	1.35
6913	*ATRNL1*	3.3772	0.72184	-0.05504	-0.77282	1.35
6914	*HAO2*	2.8211	1.185	0.0373	-0.9067	1.35
6915	*CAMKV*	3.8269	0.51973	-0.30376	-1.0481	1.35
6916	*PTCRA*	2.1025	1.0909	0.8494	-0.52726	1.35
6917	*EP300*	2.4169	0.84925	0.77587	-0.63127	1.35
6918	*ZNF841*	3.9569	0.73147	-0.64806	-0.77018	1.35
6919	*IFT52*	1.8902	1.8188	0.33126	-0.73695	1.35
6920	*SMYD3*	3.8921	0.11994	0.02759	-1.25	1.35
6921	*CTNNA2*	3.6259	0.41678	-0.00309	-0.67149	1.35
6922	*SESN3*	4.394	0.60871	-0.96407	-1.6072	1.35
6923	*AARD*	3.4306	0.37639	0.23163	-1.2573	1.35
6924	*ARID3C*	4.8669	0.12753	-0.95708	-2.2607	1.35
6925	*PAPPA2*	3.3689	0.66136	0.00702	-0.36654	1.35
6926	*DEPDC5*	4.3397	0.50617	-0.81003	-1.7488	1.35
6927	*PPP2R1B*	3.9426	0.40532	-0.31232	-2.2844	1.35
6928	*ATE1*	3.1183	0.78356	0.13367	-0.34819	1.35
6929	*CNNM1*	4.1469	0.12602	-0.23802	-1.0112	1.34
6930	*FFAR4*	3.9425	0.50438	-0.41207	-1.183	1.34
6931	*MYEOV*	4.151	0.54144	-0.65906	-1.8297	1.34
6932	*ZDHHC7*	3.5363	0.41203	0.0844	-1.6239	1.34
6933	*CTXN3*	3.3815	1.5399	-0.88876	-1.9686	1.34
6934	*FHL2*	2.6278	1.4527	-0.04847	-0.93562	1.34
6935	*MEF2C*	3.6228	0.60429	-0.19515	-1.3727	1.34
6936	*WNT6*	2.4032	2.3762	-0.748	-0.78445	1.34
6937	*RDH8*	3.7764	1.0697	-0.81528	-1.3226	1.34
6938	*B4GALNT4*	3.143	2.5186	-1.6316	-2.7705	1.34
6939	*TMSB15A*	4.3465	-0.1313	-0.18589	-1.3756	1.34

6940	*RRS1*	3.1529	1.5474	-0.67149	-2.2254	1.34
6941	*RASGRP1*	4.553	0.19242	-0.71669	-0.81448	1.34
6942	*IFRD1*	2.7718	0.74863	0.5083	0.25102	1.34
6943	*LDOC1*	2.7192	0.69807	0.61139	-0.44353	1.34
6944	*CIPC*	4.6642	1.0561	-1.6921	-2.5795	1.34
6945	*INVS*	3.169	2.003	-1.1444	-1.463	1.34
6946	*IDO2*	4.5802	0.14208	-0.6951	-0.88098	1.34
6947	*LRCH1*	2.7204	1.5935	-0.28752	-0.40577	1.34
6948	*CASR*	2.0977	1.49	0.4386	-1.2208	1.34
6949	*PKHD1*	2.0049	1.0765	0.94481	-1.1945	1.34
6950	*IL18*	3.5229	2.0618	-1.5588	-1.7316	1.34
6951	*PRKAR2B*	2.3981	2.0632	-0.43551	-1.5943	1.34
6952	*ZNF783*	2.401	1.4923	0.13052	-0.73552	1.34
6953	*PAPD5*	2.846	0.98721	0.19051	-1.0288	1.34
6954	*DSCR4*	1.6527	1.2751	1.0951	-1.2618	1.34
6955	*AMER1*	3.7376	2.4815	-2.197	-3.0974	1.34
6956	*WIPI2*	3.1498	1.405	-0.53346	-0.85512	1.34
6957	*C3orf30*	3.0621	0.63937	0.31943	-1.1039	1.34
6958	*RNF186*	2.8464	1.1781	-0.00464	-0.22601	1.34
6959	*NPVF*	2.7149	1.106	0.19776	-0.64956	1.34
6960	*MTMR10*	3.1627	1.0368	-0.18093	-0.62307	1.34
6961	*LCE1E*	1.4134	1.4001	1.2044	0.91707	1.34
6962	*ETV7*	2.4662	1.574	-0.0224	-1.6974	1.34
6963	*TSPAN10*	1.814	1.7935	0.40999	-0.94184	1.34
6964	*ANK3*	2.8418	2.3612	-1.1861	-1.3419	1.34
6965	*BTG1*	2.5809	1.4148	0.02091	-0.33119	1.34
6966	*AK1*	3.7836	0.85028	-0.61749	-1.2008	1.34
6967	*IFNW1*	2.8231	1.6781	-0.48492	-1.9168	1.34
6968	*IRF9*	3.2557	1.0209	-0.26055	-1.2684	1.34
6969	*CNTN2*	3.0449	2.2751	-1.3044	-1.4824	1.34
6970	*NCALD*	2.1652	1.8636	-0.01587	-0.16506	1.34
6971	*CNTD2*	3.0046	1.8181	-0.80978	-1.0212	1.34
6972	*HIST1H4F*	2.1079	0.99453	0.91012	-0.38491	1.34
6973	*BRCC3*	3.8004	0.44057	-0.22873	-1.0033	1.34
6974	*FURIN*	2.9692	2.2998	-1.257	-1.3353	1.34
6975	*PTGS2*	4.7756	0.21713	-0.98104	-1.4075	1.34
6976	*IDUA*	3.2146	0.95422	-0.15855	-1.1001	1.34
6977	*OR6V1*	1.586	1.2548	1.1692	0.22976	1.34
6978	*MTHFSD*	2.0211	1.4459	0.54299	0.081831	1.34
6979	*SLC17A4*	2.1777	1.2829	0.54921	0.094326	1.34
6980	*SNRPC*	2.7444	1.708	-0.44261	-1.0268	1.34
6981	*PPT2*	2.919	1.8867	-0.79641	-1.135	1.34
6982	*FAM83F*	2.2462	1.1964	0.56668	0.060946	1.34
6983	*MDP1*	1.8014	1.3144	0.89321	-2.0187	1.34
6984	*SLC35A3*	4.0353	0.82493	-0.85163	-1.1309	1.34
6985	*CCNY*	2.1875	1.5472	0.27373	-0.34653	1.34

6986	*ROBO4*	1.68	1.1706	1.1576	0.26156	1.34
6987	*SPINK7*	4.3529	0.098095	-0.44353	-0.51761	1.34
6988	*SEC14L5*	3.5696	1.1771	-0.73972	-0.91076	1.34
6989	*MOK*	4.3409	0.76489	-1.1005	-1.5312	1.34
6990	*GLRA4*	4.1076	0.80786	-0.9105	-1.2457	1.33
6991	*PCTP*	2.0268	1.7137	0.26438	-1.3622	1.33
6992	*HTR4*	2.5229	1.1505	0.33106	-1.205	1.33
6993	*ABL1*	3.116	0.5268	0.36116	-2.2104	1.33
6994	*ZNF645*	4.4757	0.28106	-0.75293	-1.9284	1.33
6995	*RIT1*	3.1283	1.0509	-0.17647	-0.58837	1.33
6996	*NDRG2*	2.8753	1.5107	-0.38376	-1.1958	1.33
6997	*CCDC124*	5.0444	-0.46218	-0.58088	-0.96012	1.33
6998	*RCOR3*	3.36	0.51318	0.12772	-0.4606	1.33
6999	*C11orf97*	2.3764	1.4189	0.2053	-0.89866	1.33
7000	*FGF7*	1.6961	1.6173	0.68701	0.46419	1.33
7001	*PRSS2*	3.6538	1.2767	-0.93011	-1.3285	1.33
7002	*NID1*	4.6639	0.034566	-0.69841	-0.88416	1.33
7003	*PCDHGA2*	4.9078	0.13729	-1.0453	-1.2935	1.33
7004	*SOX4*	2.5399	0.87689	0.58268	-2.7277	1.33
7005	*SNRPA1*	4.827	0.66293	-1.492	-2.5419	1.33
7006	*SLC25A48*	3.6649	0.58909	-0.25608	-1.7836	1.33
7007	*ACAT2*	1.5162	1.2603	1.2209	-2.3949	1.33
7008	*C6orf25*	3.4866	1.0055	-0.49481	-1.3961	1.33
7009	*TMPRSS3*	2.7549	0.95084	0.29117	0.19992	1.33
7010	*UBOX5*	4.1782	-0.06272	-0.11882	-0.75361	1.33
7011	*ASIC2*	3.0518	0.99326	-0.04876	-0.23045	1.33
7012	*PLCXD3*	4.6638	0.59244	-1.26	-1.2827	1.33
7013	*ZNF330*	1.8459	1.2814	0.86883	-0.23607	1.33
7014	*KANSL1*	4.164	0.92957	-1.0986	-1.7718	1.33
7015	*FKBP1A*	5.4207	-0.58603	-0.84001	-2.2142	1.33
7016	*CA13*	4.0901	0.077839	-0.17375	-0.93889	1.33
7017	*MS4A15*	2.6942	1.216	0.08386	-0.17622	1.33
7018	*TFAP2B*	5.0574	-0.46764	-0.5959	-1.8949	1.33
7019	*SOWAHB*	2.1287	1.0265	0.83804	-0.97944	1.33
7020	*LDLRAD1*	2.6942	2.5526	-1.2541	-1.416	1.33
7021	*REG4*	3.8412	0.21972	-0.06857	-0.12688	1.33
7022	*CFH*	2.2751	1.7633	-0.04669	-1.6759	1.33
7023	*TRIM22*	3.6448	0.79236	-0.44573	-0.68674	1.33
7024	*OR5AP2*	2.793	1.8462	-0.64793	-1.9036	1.33
7025	*SPACA5*	3.3156	0.96833	-0.29349	-0.65848	1.33
7026	*APOBEC3C*	2.5102	1.0725	0.40742	-0.47028	1.33
7027	*NEUROD2*	4.7611	-0.08348	-0.68825	-1.0088	1.33
7028	*C6orf132*	1.7089	1.1911	1.0892	-1.5866	1.33
7029	*RNASE10*	2.1546	1.4732	0.36133	-0.27359	1.33
7030	*ZNF469*	2.1472	1.7579	0.084	-1.5741	1.33
7031	*OR8H1*	2.5528	2.3268	-0.89269	-1.1325	1.33

7032	*ALOX15B*	2.6812	2.6286	-1.3237	-2.1706	1.33
7033	*MRPS7*	2.2127	1.0744	0.69893	-0.6114	1.33
7034	*OR52B6*	3.0978	1.6791	-0.79147	-1.3593	1.33
7035	*KIRREL3*	2.118	2.079	-0.21169	-1.4214	1.33
7036	*GPR156*	3.1695	0.98943	-0.1741	-2.4011	1.33
7037	*RTP3*	3.1694	1.3228	-0.50754	-1.1879	1.33
7038	*ZNF213*	3.2864	0.41473	0.28351	-0.66535	1.33
7039	*KIF2C*	3.985	0.69988	-0.70064	-1.9713	1.33
7040	*LCOR*	2.9226	1.1445	-0.0834	-0.14163	1.33
7041	*RBP1*	2.3253	2.1401	-0.4818	-0.98421	1.33
7042	*CPNE3*	4.621	0.13558	-0.77418	-1.536	1.33
7043	*JMJD1C*	3.0947	0.84507	0.0418	-0.54826	1.33
7044	*ATP6V0E2*	4.4035	-0.01791	-0.40501	-1.8087	1.33
7045	*LPL*	1.6219	1.2966	1.0619	-1.632	1.33
7046	*PDE8B*	2.4049	1.3683	0.20636	-1.7363	1.33
7047	*TMEM45A*	3.2194	3.0931	-2.3334	-4.1669	1.33
7048	*UBE4A*	3.991	0.315	-0.32717	-1.6989	1.33
7049	*HKR1*	3.9873	0.21869	-0.22726	-1.5194	1.33
7050	*CCDC71*	3.3385	0.85499	-0.21647	-1.4046	1.33
7051	*CAMKMT*	3.4825	0.91249	-0.4187	-1.1492	1.33
7052	*SIRT1*	4.4238	0.97399	-1.4233	-2.6889	1.32
7053	*PPP3CB*	3.1967	0.85124	-0.0741	-0.09188	1.32
7054	*CD4*	2.2534	1.0266	0.69327	0.12362	1.32
7055	*IL17REL*	1.5302	1.3194	1.123	-1.9041	1.32
7056	*C16orf54*	3.6639	0.36987	-0.0614	-0.33711	1.32
7057	*TTLL6*	4.9512	-0.14289	-0.83631	-1.8996	1.32
7058	*MINA*	5.5787	-0.15702	-1.4507	-2.4832	1.32
7059	*MXD1*	2.9848	0.8649	0.12112	-0.58676	1.32
7060	*ABHD14B*	2.7783	1.0734	0.11886	-1.0414	1.32
7061	*ZNF556*	3.1523	1.7112	-0.89401	-2.6805	1.32
7062	*RFX7*	3.2502	0.53274	0.18642	-2.0383	1.32
7063	*TMEM171*	2.1235	1.6448	0.19987	-1.3743	1.32
7064	*PUS7*	4.7295	0.51379	-1.2752	-1.4754	1.32
7065	*ZNF883*	3.7153	0.23344	0.01861	-0.51805	1.32
7066	*BLOC1S1*	3.7423	1.1265	-0.90154	-0.9653	1.32
7067	*C1orf194*	2.6022	1.3211	0.04392	-0.76355	1.32
7068	*AKAP8*	3.5756	0.41049	-0.01897	-0.64397	1.32
7069	*UTRN*	2.7774	0.77622	0.41252	-2.0629	1.32
7070	*PRDM6*	3.2703	0.53634	0.15921	-0.99079	1.32
7071	*KRTAP1-5*	3.0326	0.79772	0.13475	-0.62629	1.32
7072	*ACOXL*	1.8327	1.423	0.70864	-1.6891	1.32
7073	*GRM7*	3.4453	1.6026	-1.0841	-1.2278	1.32
7074	*HSFY1*	4.7755	-0.13288	-0.67934	-1.8187	1.32
7075	*PPRC1*	2.566	0.89321	0.50404	-0.37669	1.32
7076	*GKAP1*	4.9622	0.18317	-1.1826	-2.1342	1.32
7077	*KIR3DL3*	3.0451	0.53014	0.38747	-0.88277	1.32

7078	*CLIP3*	4.1445	0.69134	-0.87382	-1.7999	1.32
7079	*ESM1*	2.0391	1.8217	0.10069	-0.72406	1.32
7080	*NOC3L*	2.193	0.97778	0.79009	-1.1207	1.32
7081	*SLC27A2*	4.5003	0.53285	-1.0726	-1.62	1.32
7082	*MAG*	2.6198	2.4333	-1.093	-1.244	1.32
7083	*RSPH14*	4.0725	0.20802	-0.32087	-2.5571	1.32
7084	*OR5P2*	4.7875	-0.21291	-0.61528	-2.0629	1.32
7085	*DUSP8*	1.59	1.2572	1.1117	-1.2198	1.32
7086	*ABCG8*	2.4523	1.4936	0.01271	-1.078	1.32
7087	*C7orf50*	3.3902	2.1643	-1.5962	-5.7134	1.32
7088	*MRGPRX1*	3.9297	0.48812	-0.46017	-3.3281	1.32
7089	*RICTOR*	2.0778	0.98916	0.89019	-1.6597	1.32
7090	*WNT7A*	1.8497	1.1133	0.99414	-1.2935	1.32
7091	*INTS12*	2.4746	0.81241	0.66962	-1.5258	1.32
7092	*KLHDC7B*	3.9953	1.3206	-1.3593	-3.087	1.32
7093	*DPCR1*	1.7666	1.5589	0.63099	-2.0653	1.32
7094	*DPH5*	3.9618	0.7594	-0.76606	-1.3875	1.32
7095	*XPO7*	2.1269	1.7571	0.07113	-0.10524	1.32
7096	*NGFRAP1*	2.5009	1.9294	-0.47527	-1.5406	1.32
7097	*MAML3*	3.0305	0.66824	0.25595	-0.59007	1.32
7098	*TIMM21*	3.4952	0.71707	-0.25767	-1.9062	1.32
7099	*MN1*	3.9493	0.017858	-0.0131	-0.3193	1.32
7100	*BTN3A3*	3.523	0.55409	-0.124	-0.66945	1.32
7101	*LOC100289561*	2.3111	1.3422	0.29979	-2.6365	1.32
7102	*ERP27*	3.5899	0.19846	0.16442	-1.4597	1.32
7103	*RAB42*	1.8863	1.529	0.53697	-2.0559	1.32
7104	*L2HGDH*	3.8329	1.0564	-0.93837	-2.1987	1.32
7105	*ZNF584*	3.8888	0.50541	-0.44374	-1.1303	1.32
7106	*IKBKG*	5.1695	-0.33937	-0.88008	-3.2582	1.32
7107	*WFDC9*	2.3553	2.2813	-0.68729	-0.79745	1.32
7108	*LMF1*	2.2138	1.5151	0.22013	-1.2891	1.32
7109	*FLT4*	4.3119	0.24399	-0.60717	-1.742	1.32
7110	*GSK3B*	2.3368	1.9769	-0.36527	-0.64311	1.32
7111	*CEP55*	4.3669	0.38844	-0.80743	-1.6851	1.32
7112	*RBP4*	2.7229	1.2113	0.01338	-0.19136	1.32
7113	*THAP7*	2.0425	1.2337	0.66959	-1.5268	1.32
7114	*NRL*	2.3568	1.0544	0.53445	-0.19289	1.32
7115	*SPOPL*	2.6576	2.5561	-1.2705	-2.3204	1.31
7116	*GYG1*	3.1551	0.44405	0.34401	-1.5313	1.31
7117	*KRTAP7-1*	2.0191	0.97534	0.94778	-0.95	1.31
7118	*GPR75*	2.2885	0.9747	0.67879	-1.4102	1.31
7119	*GABRE*	3.9945	0.006056	-0.05926	-1.5125	1.31
7120	*CYP2U1*	4.9476	1.4194	-2.4268	-3.0179	1.31
7121	*DIP2B*	2.691	2.4978	-1.2493	-1.4873	1.31
7122	*HBA2*	3.2382	1.2427	-0.54188	-0.71467	1.31
7123	*NSRP1*	4.2611	-0.06433	-0.25794	-2.1239	1.31

7124	*KIF16B*	4.7977	0.67722	-1.5382	-1.6826	1.31
7125	*PRSS45*	2.8468	2.4142	-1.325	-1.9622	1.31
7126	*OR4D6*	4.8061	0.24672	-1.1169	-2.056	1.31
7127	*PYCR2*	3.5361	0.48977	-0.0907	-0.63309	1.31
7128	*MMD2*	1.9755	1.8166	0.14299	-0.84738	1.31
7129	*YY1*	2.7165	0.67857	0.53966	0.44402	1.31
7130	*OR13C3*	2.8451	0.96541	0.12367	-1.2997	1.31
7131	*MTMR11*	3.5541	1.3758	-0.99608	-2.7871	1.31
7132	*SIDT2*	3.0415	1.2428	-0.35064	-0.93057	1.31
7133	*ZDHHC22*	2.6533	0.90462	0.37546	-1.6477	1.31
7134	*CPVL*	4.1546	-0.02105	-0.20044	-0.58231	1.31
7135	*C2orf48*	2.3703	1.1355	0.42713	-0.5656	1.31
7136	*C9orf43*	3.7773	0.85526	-0.69985	-1.1095	1.31
7137	*KDM2B*	3.0334	1.7624	-0.86358	-1.4473	1.31
7138	*FOXO4*	2.222	1.5844	0.12471	-0.30496	1.31
7139	*KCNK10*	3.1411	0.8332	-0.04324	-1.2572	1.31
7140	*TCF19*	4.2639	0.090632	-0.42397	-0.44135	1.31
7141	*TBX3*	5.4205	0.21634	-1.7065	-1.7528	1.31
7142	*CERK*	4.3361	-0.0828	-0.32321	-0.39775	1.31
7143	*OR2K2*	4.8017	0.18292	-1.0553	-2.875	1.31
7144	*SSX3*	2.5744	1.3592	-0.00492	-2.0411	1.31
7145	*TMEM242*	2.1705	2.0964	-0.33826	-1.8095	1.31
7146	*CRABP2*	2.9061	0.57026	0.4521	-1.1344	1.31
7147	*ZNF653*	2.7195	0.70323	0.50565	-0.51722	1.31
7148	*PREX2*	2.631	1.177	0.11982	-1.0614	1.31
7149	*OR4M1*	4.7924	-0.35565	-0.50904	-1.1558	1.31
7150	*AGBL3*	1.8799	1.3411	0.70642	-0.47179	1.31
7151	*TMEM181*	3.4361	0.31906	0.1718	-1.3141	1.31
7152	*WDR70*	5.4763	-0.47183	-1.0779	-1.4657	1.31
7153	*LCE3D*	3.1191	0.58201	0.22523	-0.8792	1.31
7154	*ATG4A*	4.0726	0.24868	-0.39533	-1.2646	1.31
7155	*DPF1*	4.2404	0.049395	-0.36454	-0.86182	1.31
7156	*INHBE*	3.6003	1.4194	-1.0949	-1.4337	1.31
7157	*ALDH3B2*	4.1128	0.12694	-0.31504	-0.53458	1.31
7158	*FAM153A*	2.9512	1.3562	-0.38348	-0.8338	1.31
7159	*SPATA16*	2.5006	1.3018	0.12149	-0.28806	1.31
7160	*MTG1*	2.1895	1.6962	0.03704	-0.67203	1.31
7161	*CBLN2*	3.8238	0.3533	-0.25501	-1.1117	1.31
7162	*OTUD6B*	2.3485	1.5089	0.06442	-2.0392	1.31
7163	*CLIC4*	2.7692	1.3055	-0.15424	-0.58589	1.31
7164	*TAC4*	5.3247	-0.63214	-0.77241	-1.3627	1.31
7165	*ASXL2*	4.2971	0.32236	-0.69972	-1.0644	1.31
7166	*CCDC37*	2.9937	0.92051	0.00551	-1.7438	1.31
7167	*SLC6A3*	2.3772	1.2172	0.32504	-1.4234	1.31
7168	*FXR2*	3.4711	0.97836	-0.53058	-1.1729	1.31
7169	*LMLN*	2.4429	0.79471	0.68105	-1.5121	1.31

7170	*FAM207A*	3.2667	0.97589	-0.32397	-0.80909	1.31
7171	*C1orf68*	2.3352	1.6784	-0.09539	-1.2234	1.31
7172	*CSNK1A1L*	2.0626	1.7766	0.07871	-0.06657	1.31
7173	*OR8G5*	2.0391	0.95321	0.92534	-1.7874	1.31
7174	*MFSD10*	4.056	0.39486	-0.53361	-2.1419	1.31
7175	*SCX*	3.3862	1.0633	-0.53268	-0.58787	1.31
7176	*KIAA1211*	3.3307	1.2519	-0.66583	-1.3745	1.31
7177	*CEL*	2.5265	0.77752	0.61125	-0.03871	1.31
7178	*PPP1R27*	3.0273	0.5137	0.37426	-1.3294	1.31
7179	*OR52H1*	3.6427	0.784	-0.51152	-0.75974	1.31
7180	*PAX8*	4.0787	0.68352	-0.84806	-0.8504	1.30
7181	*SMURF1*	2.5977	0.65859	0.65733	-0.30606	1.30
7182	*TIMP3*	1.7794	1.1567	0.97736	-1.6225	1.30
7183	*SYNGR1*	2.9393	0.75055	0.22282	-1.48	1.30
7184	*SEPT3*	2.973	0.86459	0.07442	-1.4171	1.30
7185	*PAG1*	2.1723	1.2393	0.50023	-0.44353	1.30
7186	*KLF11*	4.7048	-0.28873	-0.50426	-2.5956	1.30
7187	*NPDC1*	1.8415	1.057	1.0132	-0.89592	1.30
7188	*RPUSD2*	4.2797	-0.05721	-0.31103	-0.9939	1.30
7189	*RASGRF1*	1.8714	1.3102	0.72978	-0.4854	1.30
7190	*MYBPC3*	3.0569	1.4909	-0.63643	-2.0758	1.30
7191	*VN1R1*	2.8319	2.069	-0.98966	-1.6176	1.30
7192	*KIAA0141*	4.0373	0.088541	-0.21468	-1.0326	1.30
7193	*COX8A*	4.7297	0.23807	-1.0567	-1.4213	1.30
7194	*S100A16*	2.9353	0.67615	0.29865	-0.52397	1.30
7195	*GPC2*	2.2034	0.9061	0.79872	-1.4692	1.30
7196	*LCLAT1*	4.5576	0.85005	-1.4997	-1.5243	1.30
7197	*OR51M1*	3.8078	0.76316	-0.66301	-2.1759	1.30
7198	*OR4X2*	3.2538	0.87821	-0.22422	-0.3146	1.30
7199	*MURC*	2.9255	2.1189	-1.137	-1.2825	1.30
7200	*DEFA3*	4.625	0.18105	-0.89947	-1.2108	1.30
7201	*TMEM52B*	4.079	0.42931	-0.602	-1.3929	1.30
7202	*GRK4*	2.1719	1.1445	0.58984	-0.72334	1.30
7203	*HIST1H4L*	2.1864	1.9615	-0.24262	-0.31848	1.30
7204	*GATAD1*	2.6957	1.9364	-0.72684	-1.2895	1.30
7205	*TRPM5*	2.7355	0.62805	0.53898	-1.271	1.30
7206	*SDSL*	2.159	1.4115	0.33085	-2.4405	1.30
7207	*HEXIM2*	3.608	1.6615	-1.3683	-1.8251	1.30
7208	*CSRNP3*	4.7686	-0.22313	-0.64467	-1.2356	1.30
7209	*AMER3*	2.6671	1.9177	-0.68565	-1.1226	1.30
7210	*SYT7*	3.0581	1.3403	-0.49926	-1.246	1.30
7211	*AIFM1*	3.2547	0.9159	-0.2715	-0.55002	1.30
7212	*PIR*	2.0995	1.886	-0.08748	-0.20739	1.30
7213	*LOC645382*	4.6219	-0.31092	-0.41307	-2.0267	1.30
7214	*PLEKHG4B*	4.9919	0.046873	-1.141	-1.291	1.30
7215	*PTH2*	4.8089	-0.15953	-0.75169	-1.237	1.30

7216	*C5orf24*	3.5332	0.9019	-0.53775	-1.3218	1.30
7217	*GULP1*	1.8217	1.1911	0.88385	-2.7379	1.30
7218	*RCN3*	2.3009	1.0569	0.53845	-1.6128	1.30
7219	*EFCAB9*	2.8489	0.90742	0.13911	-1.5125	1.30
7220	*PIF1*	3.5906	0.74765	-0.44353	-1.6626	1.30
7221	*DPYSL5*	2.6095	1.049	0.23504	-0.70231	1.30
7222	*RBBP9*	3.4914	1.6973	-1.2954	-2.5119	1.30
7223	*MRC2*	4.1735	0.058484	-0.33933	-0.34308	1.30
7224	*ZNF121*	1.6588	1.6054	0.62741	-1.6587	1.30
7225	*RALGAPA1*	2.002	1.6464	0.24309	-0.16438	1.30
7226	*GLYATL3*	4.0865	-0.06374	-0.13182	-0.62596	1.30
7227	*SCAND1*	3.4044	0.59341	-0.10716	-0.35367	1.30
7228	*OR2C3*	1.9997	1.8217	0.06813	-0.06319	1.30
7229	*LOC389895*	4.2916	0.15417	-0.55675	-0.85345	1.30
7230	*TBPL1*	2.6262	0.91268	0.3501	-1.0039	1.30
7231	*GGH*	3.1589	0.89943	-0.16946	-1.988	1.30
7232	*RNASEH2B*	5.6948	-0.2715	-1.5346	-3.0054	1.30
7233	*TRAM1*	2.3853	1.4271	0.07586	-1.1004	1.30
7234	*CPNE4*	4.323	0.031912	-0.46764	-0.68781	1.30
7235	*OR51Q1*	3.7742	0.95686	-0.84419	-0.87318	1.30
7236	*GNPTAB*	3.7536	0.43378	-0.30056	-0.67298	1.30
7237	*OR51A7*	3.1185	1.5112	-0.7436	-0.7757	1.30
7238	*SPATA31E1*	3.1493	0.77005	-0.03348	-0.59143	1.30
7239	*GEMIN2*	4.6848	0.26156	-1.0614	-2.1321	1.29
7240	*REG3A*	2.9876	0.72377	0.17348	-1.1512	1.29
7241	*XKRY*	2.8395	1.4887	-0.44353	-0.55163	1.29
7242	*SPRR3*	2.4768	0.82135	0.58647	-2.2075	1.29
7243	*PLET1*	4.8901	-0.08846	-0.91721	-1.4122	1.29
7244	*DFNA5*	3.1008	1.1496	-0.36668	-0.90462	1.29
7245	*STOM*	4.7291	-0.23831	-0.60717	-1.9065	1.29
7246	*ADAM19*	4.7643	-0.39856	-0.48231	-0.89974	1.29
7247	*NEBL*	3.8583	0.17873	-0.15439	-1.287	1.29
7248	*ADAM8*	5.1858	0.002076	-1.3055	-1.8157	1.29
7249	*SMARCC2*	3.7468	0.3881	-0.25264	-1.0796	1.29
7250	*KCNK17*	4.4524	0.51759	-1.0878	-1.2353	1.29
7251	*UGT8*	3.7198	0.13368	0.02789	-1.7018	1.29
7252	*KCNA5*	2.4416	1.3565	0.08309	-0.75448	1.29
7253	*IDH3G*	3.6141	0.50127	-0.23444	-1.7065	1.29
7254	*ADAM32*	2.5947	1.2782	0.008	-0.21201	1.29
7255	*PARP4*	4.1306	0.038291	-0.28896	-1.6597	1.29
7256	*TSC22D4*	1.6001	1.3007	0.97805	0.7565	1.29
7257	*HCRTR1*	1.9177	1.3807	0.58021	0.40441	1.29
7258	*C14orf93*	4.4148	0.55654	-1.093	-1.4061	1.29
7259	*DCAF12L2*	3.0528	1.0421	-0.21666	-0.90542	1.29
7260	*HAUS1*	3.1542	2.2204	-1.4969	-1.6262	1.29
7261	*DTNB*	3.5177	0.7429	-0.38361	-0.62306	1.29

7262	*UBTD1*	2.2221	1.2246	0.43028	-0.2146	1.29
7263	*MYOC*	4.7644	0.24209	-1.13	-2.2352	1.29
7264	*HDDC2*	2.7241	0.90791	0.24325	-0.7602	1.29
7265	*EPS8L1*	3.6517	1.3372	-1.1138	-2.2473	1.29
7266	*SLC25A32*	2.3517	1.3362	0.18614	0.12559	1.29
7267	*NEU3*	3.0599	0.58197	0.23139	-1.1108	1.29
7268	*KCNA2*	2.4866	1.4731	-0.08646	-0.14649	1.29
7269	*ERVW-1*	4.5291	0.3464	-1.0024	-1.4541	1.29
7270	*HENMT1*	2.7957	1.0817	-0.005	-1.3139	1.29
7271	*PPP1R17*	3.7007	1.8652	-1.6939	-1.9431	1.29
7272	*OR11A1*	4.6541	0.64084	-1.4233	-1.6339	1.29
7273	*ANXA13*	2.1933	1.3103	0.36776	-0.57084	1.29
7274	*RGS3*	2.9156	0.92011	0.03509	-0.18775	1.29
7275	*SLC25A24*	2.4701	0.84333	0.55656	-0.35259	1.29
7276	*PCDH19*	1.8617	1.3492	0.65871	-1.0644	1.29
7277	*CAPN2*	3.4293	0.26122	0.17905	-1.4467	1.29
7278	*CD27*	4.6354	1.0162	-1.7822	-2.1412	1.29
7279	*DCLRE1B*	2.4462	0.87831	0.54424	-2.9338	1.29
7280	*MANSC1*	4.9355	-0.44353	-0.62362	-1.3235	1.29
7281	*TMEM125*	5.0272	0.72256	-1.8816	-2.7384	1.29
7282	*AP5Z1*	4.021	0.78129	-0.93517	-1.7338	1.29
7283	*SSR2*	5.8916	-0.71939	-1.3055	-1.9722	1.29
7284	*CYP39A1*	2.5469	0.904	0.41542	-0.80828	1.29
7285	*PSMD13*	3.5844	0.54691	-0.26518	-0.82044	1.29
7286	*FGF17*	4.6452	1.3252	-2.1055	-2.2311	1.29
7287	*FAM229B*	3.0028	0.48398	0.37747	0.23574	1.29
7288	*ALX4*	4.6201	-0.08194	-0.67482	-2.3689	1.29
7289	*NETO2*	4.0204	0.66344	-0.82208	-2.2599	1.29
7290	*FGFR2*	3.2723	0.67415	-0.08549	-1.0579	1.29
7291	*CAP1*	5.3058	-0.11854	-1.3271	-1.3464	1.29
7292	*FRG2C*	2.8482	0.69513	0.31649	-0.45015	1.29
7293	*ABCB7*	4.678	0.34899	-1.1673	-2.0593	1.29
7294	*FAM120A*	2.3723	0.92356	0.56241	-2.3378	1.29
7295	*AGMAT*	3.8084	0.56162	-0.51398	-1.9558	1.29
7296	*PCDH12*	3.9092	0.33434	-0.38862	-1.3728	1.28
7297	*IGSF5*	2.4301	2.3703	-0.94563	-1.6339	1.28
7298	*F11*	3.6649	1.7015	-1.5121	-1.7462	1.28
7299	*DEFB108B*	4.2026	0.31468	-0.66358	-0.91215	1.28
7300	*LRRC46*	1.9448	1.0363	0.87239	-1.2963	1.28
7301	*C19orf71*	2.903	1.3588	-0.40863	-1.119	1.28
7302	*OR52E4*	3.8908	0.63968	-0.67801	-1.3287	1.28
7303	*GIF*	5.3094	-0.66948	-0.78784	-0.84141	1.28
7304	*CALN1*	3.4614	0.54241	-0.15385	-1.57	1.28
7305	*TAS2R3*	2.1445	0.88379	0.82106	-0.18009	1.28
7306	*CHPF*	3.75	0.16085	-0.0629	-0.31504	1.28
7307	*OR52I1*	3.4751	0.5531	-0.18133	-1.7585	1.28

7308	*OR11H6*	4.3176	-0.09312	-0.37797	-1.626	1.28
7309	*ZBTB47*	3.4923	0.37835	-0.02428	-1.6152	1.28
7310	*DRP2*	3.5466	0.30505	-0.00613	-0.46153	1.28
7311	*CFAP43*	3.4616	0.98984	-0.60604	-1.4606	1.28
7312	*SHH*	4.0234	0.80877	-0.98709	-1.1043	1.28
7313	*RBM44*	3.475	0.81091	-0.44185	-1.5631	1.28
7314	*IL10*	4.4052	0.24958	-0.811	-1.371	1.28
7315	*SH3BP1*	4.6749	0.18818	-1.0197	-2.5349	1.28
7316	*HIP1*	4.1992	0.35716	-0.7131	-0.79813	1.28
7317	*C3AR1*	2.4472	1.0512	0.34426	-0.21809	1.28
7318	*VWDE*	3.4396	0.34603	0.0552	-0.00504	1.28
7319	*AEN*	3.6013	1.4496	-1.2104	-1.5121	1.28
7320	*MIOX*	3.1999	0.60219	0.03815	-1.4151	1.28
7321	*TOMM40*	3.41	0.33072	0.09878	-1.6374	1.28
7322	*TRIM32*	3.6924	0.57397	-0.42743	-0.47358	1.28
7323	*CYP2C19*	4.2287	0.42547	-0.81535	-1.36	1.28
7324	*COQ9*	1.8848	1.8362	0.11748	-3.6696	1.28
7325	*BIRC7*	2.6827	0.76864	0.38682	-1.8604	1.28
7326	*OR10Q1*	3.3924	1.171	-0.72635	-1.088	1.28
7327	*FBXO17*	3.3516	1.1966	-0.71161	-1.2404	1.28
7328	*GATS*	2.9259	1.1296	-0.21897	-1.8224	1.28
7329	*MIR1-1HG*	2.784	1.5922	-0.54089	-1.162	1.28
7330	*NTN5*	3.222	0.69164	-0.07903	-2.2951	1.28
7331	*FAM76B*	2.2638	1.3582	0.21148	-0.76344	1.28
7332	*CD300LD*	3.1184	2.4349	-1.72	-2.1965	1.28
7333	*COMMD3-BMI1*	2.4367	1.9696	-0.57359	-1.9431	1.28
7334	*DMRT3*	3.8487	0.66955	-0.68598	-2.3904	1.28
7335	*BGLAP*	2.8566	1.1018	-0.12651	-0.16579	1.28
7336	*EIF4B*	3.8434	0.19975	-0.21126	-1.5031	1.28
7337	*IL32*	3.4993	0.82828	-0.4958	-0.66788	1.28
7338	*REM1*	2.4931	1.8689	-0.5305	-1.9417	1.28
7339	*MMP7*	3.1422	0.80664	-0.11781	-0.2122	1.28
7340	*C11orf94*	3.8525	0.12724	-0.14995	-0.2492	1.28
7341	*TRPM6*	2.6304	0.63006	0.56844	-1.7399	1.28
7342	*PPM1N*	3.288	0.85474	-0.31434	-0.7819	1.28
7343	*HTR2B*	2.3363	2.1203	-0.62833	-1.6526	1.28
7344	*PRKCZ*	2.7798	0.80835	0.23983	-1.2145	1.28
7345	*SAMD15*	5.3648	-0.43113	-1.1067	-1.3023	1.28
7346	*THAP9*	3.9943	0.20894	-0.37686	-1.987	1.28
7347	*OPN4*	4.295	0.46124	-0.93011	-1.0102	1.28
7348	*KLLN*	4.0314	1.6389	-1.8443	-1.8642	1.28
7349	*ETHE1*	2.9182	0.53553	0.37201	-0.53481	1.28
7350	*RBP2*	3.7934	0.45284	-0.42062	-1.077	1.28
7351	*CAST*	2.2702	1.5206	0.03442	-0.65754	1.28
7352	*EEF2K*	3.8109	0.18332	-0.16908	-1.0684	1.28
7353	*KCTD17*	2.5167	1.1462	0.16091	0.12463	1.27

7354	*USP27X*	3.3499	0.81171	-0.33826	-0.64327	1.27
7355	*PHF7*	2.7336	0.60473	0.48364	-1.0187	1.27
7356	*BARX1*	3.4674	0.37307	-0.02108	-0.0279	1.27
7357	*ZNF816*	3.7493	0.43589	-0.36624	-0.72789	1.27
7358	*MLXIPL*	3.3784	0.72221	-0.28198	-1.8792	1.27
7359	*ZBTB2*	3.1493	0.37192	0.29708	-1.6664	1.27
7360	*PRCP*	3.0273	1.0328	-0.24196	-1.2951	1.27
7361	*CYB5R2*	1.5482	1.3267	0.94203	-0.54875	1.27
7362	*CCDC15*	3.0311	1.7456	-0.96	-1.4757	1.27
7363	*NBPF11*	3.7448	1.7004	-1.6288	-1.9305	1.27
7364	*CDK3*	2.4333	0.9539	0.42909	-2.0126	1.27
7365	*CADPS2*	4.0813	0.3841	-0.64943	-1.6419	1.27
7366	*OR10A6*	3.1119	0.49966	0.20388	-0.23883	1.27
7367	*TNFSF10*	4.5647	0.32564	-1.0761	-1.9984	1.27
7368	*WASF2*	5.5835	-0.66718	-1.1021	-1.1598	1.27
7369	*EEA1*	3.9197	0.87193	-0.97776	-1.2474	1.27
7370	*TMEM86B*	1.7583	1.6308	0.42399	-1.151	1.27
7371	*CPA4*	2.1052	1.2525	0.45515	-0.21486	1.27
7372	*INPP4B*	2.3287	0.78498	0.69873	-2.0205	1.27
7373	*TEX28*	1.9586	1.4508	0.40221	-1.6443	1.27
7374	*ACAN*	3.6241	0.37835	-0.19089	-0.6908	1.27
7375	*FABP3*	3.4995	1.4121	-1.1005	-1.5243	1.27
7376	*MYCN*	2.6619	1.1856	-0.03643	-1.4195	1.27
7377	*HEPN1*	4.2855	0.34932	-0.82402	-1.0952	1.27
7378	*HSD17B11*	2.9203	1.5786	-0.68813	-0.91253	1.27
7379	*TIGD1*	4.7804	0.89452	-1.8642	-3.0112	1.27
7380	*TNNC2*	3.6783	0.6253	-0.49326	-1.175	1.27
7381	*MARCH4*	4.117	0.53571	-0.8424	-1.1571	1.27
7382	*PURB*	1.6894	1.5827	0.53818	-1.5901	1.27
7383	*TET3*	5.2761	0.61232	-2.0791	-2.5754	1.27
7384	*C9orf47*	2.4637	1.6181	-0.2732	-1.4454	1.27
7385	*GBA2*	4.1265	0.82809	-1.1461	-1.8836	1.27
7386	*NUAK1*	1.7712	1.4397	0.59722	-0.58662	1.27
7387	*LNPEP*	2.5259	1.1759	0.106	-1.5305	1.27
7388	*CTSL*	3.9735	-0.00014	-0.16607	-1.0506	1.27
7389	*SERTAD3*	3.5193	1.0142	-0.72725	-1.6033	1.27
7390	*SPATA4*	3.0221	0.96975	-0.18588	-1.067	1.27
7391	*ICT1*	2.5136	0.78731	0.50439	-2.2825	1.27
7392	*NDP*	1.6002	1.1997	1.0041	-1.0924	1.27
7393	*AMBN*	1.9337	1.5244	0.34501	-0.09546	1.27
7394	*IMPACT*	1.9654	1.6006	0.23698	0.18324	1.27
7395	*IL18BP*	2.5686	2.1647	-0.93114	-1.251	1.27
7396	*NUP93*	3.5835	1.1864	-0.96776	-1.3946	1.27
7397	*PLEKHH3*	2.9026	0.86412	0.03392	-0.04156	1.27
7398	*REG1A*	4.8279	-0.31688	-0.71077	-2.3487	1.27
7399	*SLC15A3*	3.5758	0.15401	0.06988	-1.6112	1.27

7400	*ZNF429*	2.7101	1.6659	-0.5765	-1.2481	1.27
7401	*LTN1*	3.5872	0.87634	-0.66462	-1.8646	1.27
7402	*DRICH1*	1.7906	1.2067	0.80044	0.068299	1.27
7403	*DEFA6*	2.8426	1.4752	-0.5203	-1.025	1.27
7404	*WDR18*	4.1254	0.16776	-0.49897	-0.86066	1.26
7405	*NDUFS4*	2.3552	1.3266	0.1119	-1.6701	1.26
7406	*PCDHA11*	3.1636	0.97929	-0.34959	-0.83145	1.26
7407	*COPZ2*	1.9887	0.99686	0.80755	-0.93325	1.26
7408	*LYST*	3.5968	0.54365	-0.34886	-0.91981	1.26
7409	*TNFSF11*	3.9835	1.5433	-1.7364	-2.4386	1.26
7410	*DCC*	2.1994	1.4427	0.14574	-2.2044	1.26
7411	*ESYT2*	3.2331	0.35833	0.19604	-1.1932	1.26
7412	*ASB5*	3.3699	0.24401	0.17348	-0.22952	1.26
7413	*CTAG1B*	4.7478	0.8258	-1.7865	-2.3517	1.26
7414	*AKAP6*	2.5696	1.7034	-0.48623	-0.49984	1.26
7415	*KIF25*	2.566	1.2403	-0.0205	-3.9666	1.26
7416	*ZCWPW1*	2.17	0.94524	0.66962	-0.41669	1.26
7417	*C17orf89*	4.7358	-0.38912	-0.56205	-1.4365	1.26
7418	*DNER*	4.0871	0.9147	-1.2174	-1.3004	1.26
7419	*CFI*	2.0293	1.7785	-0.02387	-0.80654	1.26
7420	*LATS1*	5.0234	0.24777	-1.4873	-1.8297	1.26
7421	*ARHGEF25*	2.281	1.0478	0.45426	-1.4189	1.26
7422	*PNMA2*	2.891	1.0227	-0.13144	-0.64177	1.26
7423	*TMEM95*	2.2372	1.0171	0.52665	-1.7935	1.26
7424	*PI4K2B*	2.9717	0.69654	0.11229	-1.0884	1.26
7425	*FDFT1*	3.0014	0.85237	-0.07334	-0.65444	1.26
7426	*TEKT4*	3.2736	0.79994	-0.29314	-3.887	1.26
7427	*LYG2*	4.098	0.15963	-0.47757	-0.48808	1.26
7428	*OSTN*	2.7748	0.71822	0.28645	-1.2568	1.26
7429	*ZBTB44*	2.539	1.0229	0.21736	-2.4862	1.26
7430	*N4BP2L1*	4.7873	0.15282	-1.162	-1.2772	1.26
7431	*NDUFV3*	3.0943	0.56322	0.12053	-0.48131	1.26
7432	*MAS1L*	2.1671	1.4249	0.186	-1.3141	1.26
7433	*NSMF*	4.7535	0.020642	-0.99644	-1.9984	1.26
7434	*SRRM5*	3.0553	0.3799	0.34244	-0.53931	1.26
7435	*ARNTL2*	4.6861	0.70719	-1.6158	-1.6867	1.26
7436	*RPA1*	3.4039	1.4928	-1.1206	-1.2466	1.26
7437	*TRIM6-TRIM34*	3.3736	1.1492	-0.74671	-1.1271	1.26
7438	*TMEM180*	3.1813	1.8678	-1.2736	-2.213	1.26
7439	*ZNF182*	3.3824	0.55728	-0.16483	-0.72625	1.26
7440	*SIX1*	2.4606	1.7652	-0.45106	-0.73844	1.26
7441	*ABCB1*	3.4826	0.74915	-0.45705	-1.1316	1.26
7442	*LRRC58*	2.836	1.1714	-0.23523	-1.1815	1.26
7443	*ODF1*	3.6692	0.53391	-0.43101	-1.9867	1.26
7444	*CSRP3*	3.4898	0.14582	0.13631	0.024092	1.26
7445	*APAF1*	2.9643	0.74443	0.06152	-0.68729	1.26

7446	*MXI1*	3.888	0.59146	-0.70952	-1.0005	1.26
7447	*HIPK4*	2.8679	1.0903	-0.18962	-2.8684	1.26
7448	*GHRHR*	3.0952	1.5069	-0.83436	-1.027	1.26
7449	*SH2D4A*	3.6303	0.074813	0.06259	-2.4955	1.26
7450	*NLGN3*	4.217	0.38255	-0.83222	-1.5096	1.26
7451	*CNEP1R1*	4.3634	0.73803	-1.3343	-1.5658	1.26
7452	*PIAS4*	2.7763	2.0204	-1.0298	-1.9661	1.26
7453	*DSG2*	3.9832	0.097096	-0.31475	-0.33232	1.26
7454	*CPS1*	2.8934	1.1887	-0.31698	-2.0593	1.26
7455	*HEG1*	2.6052	0.59473	0.56468	0.12836	1.25
7456	*PLA2R1*	3.679	0.27668	-0.19107	-0.49657	1.25
7457	*YJEFN3*	2.606	0.96659	0.19033	-0.83497	1.25
7458	*P4HA3*	1.5896	1.332	0.84117	-0.93273	1.25
7459	*ZNF490*	5.0194	-0.11759	-1.1391	-2.3389	1.25
7460	*MTRNR2L2*	4.9107	-0.12446	-1.0236	-1.433	1.25
7461	*UCHL5*	2.1662	1.102	0.49431	-1.7829	1.25
7462	*CARD6*	2.8008	1.2974	-0.33659	-1.3053	1.25
7463	*CREB3L2*	3.9675	0.42895	-0.63486	-1.1679	1.25
7464	*AIFM3*	2.1451	1.6968	-0.08064	-1.1135	1.25
7465	*MZT2B*	4.904	0.80776	-1.9513	-2.5865	1.25
7466	*FSCN1*	3.9889	0.41801	-0.64671	-1.3218	1.25
7467	*RSRC1*	3.4765	0.1642	0.11931	-0.85392	1.25
7468	*FBXL20*	2.5393	0.68934	0.53093	-0.09021	1.25
7469	*LCE2A*	4.0752	0.71306	-1.0293	-1.1395	1.25
7470	*ADM*	3.602	0.30735	-0.15058	-1.3983	1.25
7471	*CEND1*	2.3513	1.6176	-0.21126	-1.9295	1.25
7472	*TP53AIP1*	3.058	1.3264	-0.62684	-1.4841	1.25
7473	*FSTL1*	1.5699	1.1381	1.0487	-2.2065	1.25
7474	*DDX28*	3.7847	0.006683	-0.03623	-0.51023	1.25
7475	*MPST*	3.4612	0.80292	-0.51023	-1.098	1.25
7476	*SLC33A1*	2.6001	2.2148	-1.0611	-1.6622	1.25
7477	*CLDN20*	2.0501	1.6151	0.08818	-1.0576	1.25
7478	*CD244*	3.3221	0.26954	0.16167	-1.2531	1.25
7479	*HILPDA*	3.1641	0.94374	-0.35499	-0.68811	1.25
7480	*ELMSAN1*	2.3851	1.5862	-0.21868	-1.6726	1.25
7481	*ATP1A1*	3.0466	0.35864	0.34715	-0.68259	1.25
7482	*WTAP*	2.3733	1.0972	0.28182	-1.4717	1.25
7483	*RAB3D*	3.4305	1.8553	-1.5382	-1.8589	1.25
7484	*RNF185*	5.3076	-0.20784	-1.3523	-1.4997	1.25
7485	*HNRNPH3*	1.8727	1.832	0.04272	0.028035	1.25
7486	*IL34*	3.9402	0.70459	-0.89752	-1.3246	1.25
7487	*ACTL6A*	3.3999	0.71489	-0.36971	-0.56818	1.25
7488	*CLDN18*	5.1547	-0.67149	-0.73814	-1.9534	1.25
7489	*ZBTB21*	4.1903	-0.15399	-0.29139	-0.31916	1.25
7490	*PFDN1*	3.8079	0.058704	-0.12212	-2.0066	1.25
7491	*PM20D2*	2.2537	1.0798	0.40995	0.075935	1.25

7492	*PARM1*	4.0101	0.52402	-0.79149	-1.7526	1.25
7493	*FOXD4L4*	3.9113	0.13896	-0.3077	-1.6014	1.25
7494	*HMGCL*	2.2539	1.0669	0.42137	-0.93415	1.25
7495	*ARFIP2*	2.2898	1.7296	-0.27771	-1.5436	1.25
7496	*ESRRB*	2.5347	0.95373	0.25246	-0.7443	1.25
7497	*GBGT1*	2.7761	1.0264	-0.062	-0.08586	1.25
7498	*GHSR*	3.8415	0.0528	-0.15545	-0.43919	1.25
7499	*FAM184B*	4.7544	0.037251	-1.0529	-1.1344	1.25
7500	*NR3C2*	4.0475	0.77323	-1.0824	-1.1025	1.25
7501	*TAS2R8*	3.0925	0.64417	0.00067	-1.2141	1.25
7502	*PRAMEF20*	2.6339	0.77941	0.32373	-2.4244	1.25
7503	*STXBP2*	2.509	1.2977	-0.07205	-0.18488	1.24
7504	*DBX2*	2.9045	0.6742	0.1557	-1.9794	1.24
7505	*MRPS5*	3.0344	0.75616	-0.05693	-0.95677	1.24
7506	*OLIG3*	1.9833	1.0072	0.742	-1.6109	1.24
7507	*TBCB*	4.635	0.06955	-0.9728	-1.5865	1.24
7508	*P2RX7*	2.1787	1.1636	0.38938	-1.1222	1.24
7509	*AMTN*	3.5216	0.74907	-0.54271	-1.0459	1.24
7510	*RDH10*	2.6959	0.90329	0.12843	-0.65489	1.24
7511	*H2BFM*	2.3492	1.7099	-0.33251	-0.44088	1.24
7512	*MBNL1*	4.25	0.55825	-1.0824	-1.6401	1.24
7513	*PARP16*	3.953	0.58649	-0.81381	-1.908	1.24
7514	*C20orf24*	4.9653	-0.36705	-0.87318	-1.8076	1.24
7515	*CYP4X1*	3.3465	1.9291	-1.5508	-2.252	1.24
7516	*PPFIA2*	3.5016	1.5631	-1.341	-1.9099	1.24
7517	*RABEP2*	3.4536	1.0813	-0.81147	-1.0062	1.24
7518	*BEX4*	1.8778	1.8291	0.01647	-0.07612	1.24
7519	*RANBP10*	2.7933	0.74308	0.18668	-0.02674	1.24
7520	*EREG*	3.0098	0.64728	0.06592	-1.2347	1.24
7521	*PDHA1*	4.6651	-0.29292	-0.64932	-0.83059	1.24
7522	*OR13C4*	2.4838	0.6614	0.57711	-2.5004	1.24
7523	*SPANXN3*	3.2452	1.6189	-1.1421	-1.4856	1.24
7524	*HOXA3*	2.4074	1.0854	0.2289	-2.1616	1.24
7525	*TET2*	3.8937	0.43341	-0.60547	-0.89392	1.24
7526	*OCSTAMP*	2.7318	1.1676	-0.17783	-1.4448	1.24
7527	*PDXK*	1.9806	1.0588	0.68194	0.018783	1.24
7528	*PHF13*	4.2037	0.46168	-0.9442	-1.9023	1.24
7529	*SAMD7*	3.9873	0.17513	-0.44236	-1.987	1.24
7530	*NLRC5*	2.9814	0.39691	0.34146	-0.3221	1.24
7531	*CACUL1*	4.1269	-0.20194	-0.20571	-0.36531	1.24
7532	*C11orf44*	2.9554	0.5809	0.18195	-0.30192	1.24
7533	*SLC35G6*	3.9057	0.27966	-0.46731	-1.6014	1.24
7534	*LDAH*	1.4862	1.3324	0.89945	0.34054	1.24
7535	*ADORA1*	1.7826	1.7589	0.17647	-0.55585	1.24
7536	*C8orf74*	5.2678	0.48487	-2.0363	-2.3424	1.24
7537	*ZFX*	2.551	0.91279	0.252	-0.87208	1.24

7538	*MSX1*	1.832	1.1229	0.76079	0.66962	1.24
7539	*POTEG*	4.0362	0.5484	-0.86925	-1.4747	1.24
7540	*C12orf40*	3.1739	1.9659	-1.4252	-1.7364	1.24
7541	*PYGB*	2.2592	1.6714	-0.21666	-0.44353	1.24
7542	*CCL4*	2.7556	0.51145	0.44547	-0.39081	1.24
7543	*SPESP1*	2.9145	1.3258	-0.52853	-0.8604	1.24
7544	*OR12D3*	3.9559	0.69701	-0.94119	-1.1825	1.24
7545	*TMEM254*	3.9084	0.34706	-0.54402	-2.095	1.24
7546	*FANCE*	4.4939	-0.0426	-0.74015	-0.75351	1.24
7547	*AREG*	2.1569	1.7741	-0.222	-1.6128	1.24
7548	*SQLE*	3.3342	0.30309	0.0695	-0.03675	1.24
7549	*RBL2*	3.9329	0.78604	-1.0134	-1.4023	1.24
7550	*DEFA4*	1.8247	1.5369	0.34331	-0.01323	1.23
7551	*SERPINA4*	2.0336	1.6278	0.04297	-0.92312	1.23
7552	*CXCL5*	3.3152	0.42215	-0.03304	-0.50429	1.23
7553	*C1orf94*	2.4451	1.2567	0.00192	-0.59804	1.23
7554	*CDH2*	2.8924	2.3304	-1.5194	-1.8636	1.23
7555	*APOC1*	1.6597	1.345	0.69837	-0.81163	1.23
7556	*VBP1*	3.0113	1.5256	-0.83435	-1.9541	1.23
7557	*RPS3*	4.234	0.36888	-0.9007	-1.1865	1.23
7558	*MRPS6*	4.1727	-0.21998	-0.25124	-1.1958	1.23
7559	*DCUN1D4*	3.6564	1.505	-1.4614	-2.095	1.23
7560	*PNMT*	4.1345	0.078186	-0.51302	-2.5004	1.23
7561	*PCDHAC2*	2.5956	1.038	0.06587	-2.1687	1.23
7562	*RNPEP*	3.9361	-0.09946	-0.13753	-0.80234	1.23
7563	*MESDC1*	3.3935	0.3665	-0.06165	-0.11145	1.23
7564	*FAM189B*	3.5654	0.29836	-0.16574	-0.61483	1.23
7565	*CCIN*	4.2266	-0.03857	-0.49071	-2.1906	1.23
7566	*KRT6B*	2.7322	0.72574	0.23822	-0.02576	1.23
7567	*CENPM*	3.8629	0.16852	-0.33538	-1.7424	1.23
7568	*SLC44A3*	3.2916	0.97008	-0.56564	-1.9844	1.23
7569	*COL5A2*	4.8456	-0.08609	-1.0635	-2.3915	1.23
7570	*PEX26*	1.6232	1.1041	0.96862	0.19922	1.23
7571	*DGAT2L6*	3.8173	0.52584	-0.64748	-1.2135	1.23
7572	*TNFRSF10A*	2.4264	1.4704	-0.20127	-1.5395	1.23
7573	*ZNF658*	2.9437	1.1508	-0.39907	-1.5663	1.23
7574	*NUP54*	4.0097	0.069497	-0.38428	-1.9023	1.23
7575	*PSMA8*	3.9542	0.54044	-0.80144	-1.9567	1.23
7576	*CALM1*	1.5327	1.3695	0.7883	-0.21814	1.23
7577	*SP3*	2.8959	1.7157	-0.92131	-1.1145	1.23
7578	*C9orf135*	3.6172	1.6316	-1.5587	-1.6776	1.23
7579	*CCNE1*	1.6988	1.3042	0.6871	0.13805	1.23
7580	*ALOX5AP*	1.9167	1.5155	0.25747	-0.09554	1.23
7581	*CCDC74B*	2.2626	2.0812	-0.6548	-1.1222	1.23
7582	*TEX2*	2.9742	0.78382	-0.07026	-1.0799	1.23
7583	*TMEM126A*	2.4418	0.71297	0.53261	-0.14606	1.23

7584	*GBP7*	3.35	0.30932	0.02789	-1.7805	1.23
7585	*HNRNPUL2*	2.8776	2.5877	-1.7783	-2.4829	1.23
7586	*MYOZ2*	2.8397	0.44248	0.40476	-0.15226	1.23
7587	*SSR4*	2.3944	2.1694	-0.87706	-1.6696	1.23
7588	*C21orf91*	2.2786	1.6277	-0.21993	-1.329	1.23
7589	*PIEZO2*	3.4273	1.2679	-1.0089	-1.1848	1.23
7590	*SYN2*	1.8587	1.5494	0.27752	-1.2421	1.23
7591	*CT47A8*	2.4949	1.9063	-0.71588	-1.7591	1.23
7592	*RNF34*	4.5254	0.38113	-1.222	-3.301	1.23
7593	*C6orf163*	1.5792	1.1047	1.0002	0.7564	1.23
7594	*CDKN3*	2.8091	1.5606	-0.68606	-2.2413	1.23
7595	*KCNA6*	2.7747	1.2149	-0.30609	-3.4888	1.23
7596	*GLTP*	2.3304	0.99354	0.35935	-1.7676	1.23
7597	*FGF22*	2.9007	0.68001	0.10175	-0.45544	1.23
7598	*AMY1A*	3.5669	0.48657	-0.37108	-0.47557	1.23
7599	*MB21D1*	1.5224	1.2902	0.86898	-0.77184	1.23
7600	*SIX5*	3.9615	0.014205	-0.29442	-1.4507	1.23
7601	*C1QTNF4*	1.3946	1.2828	1.0037	0.050642	1.23
7602	*KBTBD11*	4.4156	0.13052	-0.86698	-1.0846	1.23
7603	*HOXC5*	2.6564	1.6272	-0.60563	-1.4154	1.23
7604	*TMEFF1*	2.4269	0.81814	0.43282	0.36064	1.23
7605	*OR8G1*	2.8937	1.1571	-0.37334	-0.81947	1.23
7606	*THSD1*	1.6556	1.4915	0.53014	0.40872	1.23
7607	*SZT2*	2.4534	1.0128	0.20959	-1.0995	1.23
7608	*MAPK4*	1.9744	1.2586	0.44215	-1.067	1.23
7609	*FBRS*	3.9921	0.88291	-1.2001	-1.3479	1.22
7610	*LHFPL5*	3.8969	0.35544	-0.57822	-0.63399	1.22
7611	*NAV1*	4.7387	0.2261	-1.2912	-1.9623	1.22
7612	*NIT2*	2.4717	1.741	-0.53931	-1.9979	1.22
7613	*PALM*	3.7225	0.14909	-0.19842	-0.27924	1.22
7614	*PPP2R1A*	1.5062	1.3704	0.79651	-2.0069	1.22
7615	*PAFAH1B1*	2.7597	0.50952	0.40308	-1.772	1.22
7616	*LIN37*	4.1139	-0.00702	-0.43466	-0.78203	1.22
7617	*ADPRH*	3.7356	0.058977	-0.1227	-2.3488	1.22
7618	*SOCS7*	4.1499	0.74421	-1.2234	-1.609	1.22
7619	*GFOD2*	3.8748	0.12479	-0.32892	-0.9858	1.22
7620	*C1orf226*	3.1537	0.66958	-0.15385	-0.22068	1.22
7621	*MARK1*	2.9547	0.93209	-0.21803	-0.64574	1.22
7622	*ABCA9*	4.1836	1.0157	-1.5309	-5.3042	1.22
7623	*ADRA2C*	4.2347	-0.06231	-0.50473	-2.2844	1.22
7624	*FRMD1*	3.4263	0.95819	-0.71717	-1.5082	1.22
7625	*SMARCA4*	3.1024	2.0771	-1.5138	-1.7342	1.22
7626	*OR52N5*	3.9267	-0.05599	-0.20537	-0.79726	1.22
7627	*PID1*	2.8047	1.1948	-0.3342	-1.2084	1.22
7628	*ZNF502*	1.3263	1.1168			1.22
7629	*RAB19*	1.9062	1.0011	0.75727	-1.0811	1.22

7630	*CNIH1*	4.5338	-0.03058	-0.83938	-2.3424	1.22
7631	*MAP3K7*	1.7654	1.7615	0.13677	0.021444	1.22
7632	*KRT20*	2.4976	1.6933	-0.52739	-0.61779	1.22
7633	*ROS1*	4.3711	0.64807	-1.3574	-2.1749	1.22
7634	*FBXW9*	2.1593	1.9401	-0.43875	-0.70466	1.22
7635	*TNFRSF21*	3.5704	0.048185	0.0405	-0.75778	1.22
7636	*JOSD2*	3.7761	0.12559	-0.24368	-0.65339	1.22
7637	*MECR*	2.285	1.5238	-0.15139	-0.56076	1.22
7638	*PRICKLE1*	3.9997	0.96682	-1.3092	-2.3857	1.22
7639	*CCP110*	2.8679	2.2831	-1.4944	-2.0535	1.22
7640	*SLC8A1*	3.4783	0.90579	-0.72764	-1.9713	1.22
7641	*HDGF*	3.6655	0.43401	-0.44353	-0.97786	1.22
7642	*RASAL2*	3.7054	0.2372	-0.28715	-0.82402	1.22
7643	*TTI1*	4.4497	-0.17402	-0.62145	-1.1709	1.22
7644	*ECHDC1*	2.1447	0.76987	0.73907	-1.8094	1.22
7645	*BANF2*	3.7534	0.28631	-0.3863	-3.1439	1.22
7646	*LY6G6F*	3.0467	0.38259	0.22286	-1.3205	1.22
7647	*HDAC8*	1.9404	1.6784	0.03318	-0.06973	1.22
7648	*ZNF260*	4.5284	-0.43407	-0.44353	-1.5722	1.22
7649	*TSPAN31*	2.4207	1.4757	-0.24569	-1.8095	1.22
7650	*SGMS2*	4.8302	-0.29739	-0.88267	-2.0629	1.22
7651	*PRG4*	4.1289	-0.03528	-0.44353	-1.2171	1.22
7652	*ABCA6*	4.5922	0.1106	-1.0528	-1.3585	1.22
7653	*HGF*	4.9994	-0.52601	-0.82368	-1.2004	1.22
7654	*C7orf66*	3.7537	0.70583	-0.81001	-2.1315	1.22
7655	*CHCHD6*	2.846	0.85708	-0.05396	-0.13322	1.22
7656	*OCIAD1*	4.9851	-0.44261	-0.8934	-1.3053	1.22
7657	*OR9Q2*	3.838	0.22342	-0.41239	-0.83337	1.22
7658	*PTAR1*	5.3454	-0.78959	-0.90713	-1.5095	1.22
7659	*RUFY2*	2.8545	1.1651	-0.37108	-1.8779	1.22
7660	*GK5*	3.3209	1.4758	-1.1484	-1.9693	1.22
7661	*IQSEC2*	1.7552	1.3233	0.56967	-0.95184	1.22
7662	*EIF1AY*	3.8213	0.14253	-0.31596	-1.7567	1.22
7663	*ST20*	3.5606	0.26445	-0.17734	-0.23315	1.22
7664	*ABHD17C*	1.9374	1.1403	0.56986	-0.22324	1.22
7665	*CEP57*	4.3564	0.097322	-0.80636	-0.85943	1.22
7666	*TXNRD3NB*	3.3921	0.74036	-0.48606	-1.4619	1.22
7667	*MTCH1*	2.6069	0.91221	0.12611	-2.9211	1.22
7668	*XAF1*	3.56	0.28611	-0.20142	-1.3729	1.21
7669	*MMRN1*	3.7171	1.2204	-1.2935	-1.3346	1.21
7670	*PDE1B*	2.8388	1.1617	-0.35665	-2.3244	1.21
7671	*ZNF266*	3.582	1.5081	-1.4474	-1.6092	1.21
7672	*PIK3CB*	2.8509	1.9011	-1.1094	-1.9094	1.21
7673	*SNX30*	2.3018	1.7341	-0.39342	-1.213	1.21
7674	*INTS4*	2.9298	0.66252	0.05	-1.316	1.21
7675	*RIMKLA*	2.3333	0.80747	0.50015	-1.2163	1.21

7676	*ABO*	2.5311	0.56905	0.54066	-1.3279	1.21
7677	*WNT8A*	3.0294	0.5128	0.09836	-2.229	1.21
7678	*TMEM41A*	3.533	0.90858	-0.80129	-1.3864	1.21
7679	*DOK5*	3.0494	1.0344	-0.44353	-1.855	1.21
7680	*GAS6*	3.1314	0.43955	0.06875	-0.43966	1.21
7681	*MAPK9*	4.1381	-0.06539	-0.43335	-0.43587	1.21
7682	*NUDT16*	3.1328	0.38449	0.12206	-0.74875	1.21
7683	*FAM47B*	2.8953	2.2742	-1.5304	-1.7165	1.21
7684	*ZFHX4*	2.7949	0.68476	0.15937	-1.4717	1.21
7685	*C21orf33*	3.2171	0.89646	-0.47577	-0.92718	1.21
7686	*CLEC2L*	3.9214	-0.12896	-0.15499	-0.7771	1.21
7687	*AP4M1*	4.5009	0.91475	-1.7802	-2.0218	1.21
7688	*DPH3P1*	3.518	1.3297	-1.2132	-1.3132	1.21
7689	*HYI*	3.8621	0.45661	-0.68659	-1.7782	1.21
7690	*CLIC1*	2.5553	1.4051	-0.32863	-1.707	1.21
7691	*NAALADL1*	2.8618	0.62168	0.14824	-2.3251	1.21
7692	*OR6C68*	2.038	1.4719	0.12154	-1.6525	1.21
7693	*SHROOM4*	2.4852	1.4789	-0.33266	-1.1451	1.21
7694	*KIDINS220*	2.9525	0.55812	0.11975	-0.49438	1.21
7695	*CALR*	3.6254	0.7937	-0.78899	-1.8567	1.21
7696	*LCE3B*	2.6006	0.74132	0.28806	-1.7049	1.21
7697	*BLM*	3.2571	0.41919	-0.04635	-1.9554	1.21
7698	*RTEL1*	5.9886	-1.1067	-1.2525	-1.2964	1.21
7699	*FAM155A*	3.4128	0.29118	-0.07613	-0.45644	1.21
7700	*GPCPD1*	4.0571	-0.00833	-0.42127	-0.70752	1.21
7701	*HDAC9*	2.0212	0.92582	0.68011	-0.92975	1.21
7702	*RAB11FIP4*	5.1448	0.00711	-1.525	-1.8085	1.21
7703	*PROC*	3.019	0.41861	0.18898	-2.6717	1.21
7704	*RIOK3*	4.8841	-0.42195	-0.83627	-1.2192	1.21
7705	*POLR1A*	3.9454	1.1218	-1.4416	-1.6078	1.21
7706	*SLC16A2*	3.5535	0.98197	-0.91045	-2.0343	1.21
7707	*FAM129A*	2.0291	0.93044	0.66404	-1.7947	1.21
7708	*KIAA1161*	3.0525	0.89569	-0.32515	-2.1103	1.21
7709	*LCK*	3.5734	1.2133	-1.1637	-1.6025	1.21
7710	*ZNF582*	2.972	0.86377	-0.21291	-0.25444	1.21
7711	*C10orf99*	4.8862	-0.01286	-1.2505	-2.4633	1.21
7712	*AP3M2*	2.0813	1.2916	0.24988	-1.3542	1.21
7713	*ATP8A2*	2.3439	1.7327	-0.45397	-1.4689	1.21
7714	*THEM6*	3.2286	0.34157	0.05235	-0.08768	1.21
7715	*PCBD1*	1.8841	0.92052	0.81777	-0.68535	1.21
7716	*PLEKHA5*	2.2283	0.73304	0.6604	-0.48247	1.21
7717	*FAT4*	2.9011	1.3902	-0.67019	-0.78229	1.21
7718	*TFPI*	1.726	1.0102	0.8844	-0.68869	1.21
7719	*OR5L2*	3.8543	0.091295	-0.32524	-1.2111	1.21
7720	*MUC15*	4.0048	0.50678	-0.89155	-1.1468	1.21
7721	*PTPRS*	2.3482	1.0339	0.23731	-1.7547	1.21

7722	*ART5*	2.0334	1.1781	0.40742	-1.8382	1.21
7723	*AP1M1*	2.2705	0.73002	0.61789	-0.30737	1.21
7724	*LRRC30*	2.6656	0.82742	0.12492	-1.8349	1.21
7725	*HRCT1*	3.2832	2.087	-1.7526	-1.7782	1.21
7726	*STK4*	3.2624	1.9262	-1.5729	-1.9105	1.21
7727	*BRK1*	4.8795	-0.17509	-1.0904	-1.3371	1.20
7728	*STXBP6*	3.9697	0.19797	-0.55441	-0.60701	1.20
7729	*KLRF2*	2.702	0.97109	-0.06021	-1.343	1.20
7730	*PLEKHM1*	2.5008	1.173	-0.06253	-0.6878	1.20
7731	*TMEM51*	1.7991	1.393	0.41913	-1.2014	1.20
7732	*ZBTB33*	2.9012	0.56486	0.14465	-0.95896	1.20
7733	*BLOC1S5*	4.5332	-0.33456	-0.58797	-1.224	1.20
7734	*CUL1*	3.2402	0.33218	0.03797	-2.1599	1.20
7735	*GOPC*	3.903	0.46788	-0.76083	-1.2234	1.20
7736	*LTA*	4.5875	-0.4735	-0.50405	-1.9623	1.20
7737	*ACPT*	2.4732	0.83429	0.30228	-2.2125	1.20
7738	*SNRPB*	4.1518	-0.15232	-0.3908	-1.4596	1.20
7739	*CASS4*	3.4066	0.14037	0.06168	-0.69286	1.20
7740	*POGK*	4.1071	0.50129	-1.0003	-1.8478	1.20
7741	*IFNAR1*	1.6438	1.3949	0.56882	-2.6072	1.20
7742	*ACVR2A*	3.2443	0.50239	-0.13938	-0.81247	1.20
7743	*ZFHX2*	4.0595	0.80351	-1.2572	-1.7645	1.20
7744	*PERM1*	4.8335	-0.10979	-1.1189	-1.2694	1.20
7745	*ZNF681*	4.0979	0.095292	-0.58894	-0.68303	1.20
7746	*SLC4A1*	1.7242	1.6845	0.19472	-2.0895	1.20
7747	*ALKBH6*	2.6325	2.532	-1.5611	-1.725	1.20
7748	*OR5K2*	2.8738	1.2477	-0.51845	-1.0955	1.20
7749	*METRNL*	1.5856	1.4735	0.54394	-1.1034	1.20
7750	*COMTD1*	2.2192	1.593	-0.20946	-1.4407	1.20
7751	*CTTNBP2*	2.3106	1.2797	0.01243	-0.72279	1.20
7752	*SUPT20HL1*	4.2274	0.000118	-0.62527	-0.81863	1.20
7753	*GIPC1*	5.4437	-0.08164	-1.7602	-1.8157	1.20
7754	*CDY2B*	3.3877	0.42034	-0.20663	-0.24491	1.20
7755	*ZNF511*	3.6329	0.64987	-0.68192	-1.4185	1.20
7756	*C1orf127*	3.7862	0.17208	-0.35757	-0.52494	1.20
7757	*OLFM1*	3.0764	0.96863	-0.44459	-0.74059	1.20
7758	*TMED3*	4.3305	0.058664	-0.78959	-1.337	1.20
7759	*ZNF528*	2.9908	0.53903	0.06919	-0.05737	1.20
7760	*MMP16*	3.147	0.5381	-0.08622	-0.93002	1.20
7761	*KLK8*	4.1207	-0.09698	-0.42503	-0.8458	1.20
7762	*OR3A3*	3.068	0.96971	-0.43928	-0.92742	1.20
7763	*MUC3A*	4.3856	-0.10619	-0.68136	-2.3487	1.20
7764	*SEPHS2*	2.058	2.0458	-0.50601	-1.3742	1.20
7765	*PDZD2*	1.542	1.3183	0.73687	0.38767	1.20
7766	*PGPEP1L*	3.344	0.79798	-0.54484	-1.1305	1.20
7767	*GORASP1*	3.0699	0.79324	-0.26625	-1.8908	1.20

7768	*SIGLEC10*	2.408	1.2453	-0.0566	-2.2276	1.20
7769	*IGFL3*	3.6303	0.22193	-0.25622	-0.88053	1.20
7770	*KRT15*	4.1728	-0.28047	-0.29652	-1.525	1.20
7771	*DLG4*	3.7208	-0.04218	-0.08352	-0.53239	1.20
7772	*FGF3*	2.7368	0.88904	-0.03148	-0.99638	1.20
7773	*PTPRZ1*	4.3738	0.48467	-1.2646	-1.3966	1.20
7774	*RPS15*	4.6371	0.056101	-1.0999	-1.1925	1.20
7775	*KRT23*	3.4335	0.1389	0.02064	-0.59743	1.20
7776	*ERBB4*	2.2745	1.1471	0.17035	-0.84158	1.20
7777	*GTF2B*	4.3221	-0.03304	-0.69735	-1.7029	1.20
7778	*SYTL5*	1.863	0.99426	0.73388	-0.93696	1.20
7779	*PNPLA6*	4.4164	-0.04968	-0.7757	-2.5153	1.20
7780	*NT5C1B*	2.3811	1.6049	-0.3952	-1.1008	1.20
7781	*HBM*	1.8746	1.2645	0.45091	-0.90014	1.20
7782	*MARVELD2*	2.7847	1.1162	-0.31244	-1.6707	1.20
7783	*CCNF*	2.5574	2.5261	-1.4954	-1.6718	1.20
7784	*AKR1C2*	3.6513	0.51054	-0.57408	-1.666	1.20
7785	*DUSP1*	1.975	0.88343	0.72823	-1.0863	1.20
7786	*GUCY2C*	2.2808	2.1203	-0.81524	-2.9931	1.20
7787	*MYO6*	2.4758	0.99078	0.11922	-0.96433	1.20
7788	*SUPT3H*	2.594	1.4708	-0.47929	-1.072	1.20
7789	*SFTPD*	2.7094	0.61412	0.26182	-1.5134	1.20
7790	*C10orf142*	3.1871	1.0122	-0.6141	-1.0954	1.20
7791	*C5orf49*	3.9599	0.32436	-0.69972	-1.3053	1.19
7792	*CHRNA6*	2.3552	1.439	-0.20969	-0.30843	1.19
7793	*IRF5*	4.1916	-0.21059	-0.39673	-1.6093	1.19
7794	*ZNF12*	2.9298	0.64084	0.01338	-0.95224	1.19
7795	*SPATA3*	1.8031	1.1521	0.62871	-1.5269	1.19
7796	*MCAT*	3.7567	1.154	-1.327	-1.4804	1.19
7797	*NSMCE2*	3.5467	0.31424	-0.27841	-0.69006	1.19
7798	*PINX1*	2.9273	0.85515	-0.19995	-1.2027	1.19
7799	*LIPE*	2.7558	1.9438	-1.1173	-1.7117	1.19
7800	*TIMP2*	3.4606	0.52501	-0.4046	-1.8297	1.19
7801	*FAM136A*	3.8997	0.52416	-0.84436	-1.4156	1.19
7802	*MRGPRD*	1.9441	0.83454	0.80057	-0.86494	1.19
7803	*SSTR3*	4.3585	0.97965	-1.7591	-2.2888	1.19
7804	*ATP5G3*	3.9127	0.50345	-0.83713	-1.448	1.19
7805	*SUPT16H*	4.905	-0.54155	-0.78482	-2.1073	1.19
7806	*DTWD1*	2.7817	0.80823	-0.01218	-0.63512	1.19
7807	*SHE*	2.7783	0.64171	0.15724	-0.44691	1.19
7808	*RAB39A*	1.8788	1.1576	0.53875	0.000993	1.19
7809	*CNPPD1*	4.2563	-0.09866	-0.58315	-0.70463	1.19
7810	*GOLGA7*	4.2551	-0.15961	-0.52182	-1.5243	1.19
7811	*ST6GALNAC3*	5.3648	-0.70442	-1.0868	-1.6356	1.19
7812	*C6orf89*	2.4026	0.69851	0.47241	-1.4039	1.19
7813	*UQCC3*	2.7458	1.6926	-0.86494	-1.3517	1.19

7814	*CKS2*	3.6217	1.6821	-1.7322	-2.0682	1.19
7815	*CYP4F2*	4.5461	-0.2347	-0.74003	-1.9623	1.19
7816	*WDR34*	3.388	1.458	-1.2755	-1.4168	1.19
7817	*SLC37A4*	3.3314	0.79492	-0.55641	-1.8627	1.19
7818	*KCNQ5*	2.8983	1.1048	-0.43473	-1.2448	1.19
7819	*GAB1*	3.086	0.96745	-0.48669	-1.3007	1.19
7820	*SLC26A8*	3.851	0.85891	-1.1432	-1.316	1.19
7821	*ADGRG7*	2.4111	1.1494	0.00598	-0.27333	1.19
7822	*ZNF668*	5.17	-0.26844	-1.3353	-1.3373	1.19
7823	*ICMT*	2.769	1.1804	-0.38349	-1.4048	1.19
7824	*CRYL1*	3.5968	1.05	-1.0811	-1.3643	1.19
7825	*CD83*	3.8169	0.52736	-0.77889	-0.90387	1.19
7826	*PIEZO1*	3.9061	-0.08544	-0.25611	-1.4707	1.19
7827	*KCNA7*	2.8489	1.4099	-0.69463	-1.4206	1.19
7828	*RSRP1*	2.879	0.67899	0.00504	-1.3645	1.19
7829	*PNPLA1*	1.9151	1.1325	0.51482	-0.86884	1.19
7830	*B4GAT1*	1.683	1.6501	0.22919	-1.8157	1.19
7831	*CAPZA1*	2.0375	0.95152	0.57307	-0.39998	1.19
7832	*TMEM175*	4.5119	0.4967	-1.4473	-2.1685	1.19
7833	*TRPV3*	4.7069	-0.25225	-0.8934	-1.4636	1.19
7834	*ZNF568*	2.5445	1.52	-0.5038	-1.2755	1.19
7835	*DVL2*	3.7444	0.57317	-0.75721	-2.7427	1.19
7836	*FOXO1*	2.5371	0.72283	0.30033	-0.09792	1.19
7837	*CNPY1*	1.9983	1.7146	-0.1534	-1.3694	1.19
7838	*ACSL6*	4.2005	-0.01037	-0.63112	-1.4014	1.19
7839	*MYOM3*	3.805	0.62155	-0.86775	-1.4173	1.19
7840	*SLC35E2B*	2.7305	1.6926	-0.86479	-1.5382	1.19
7841	*CLPX*	2.4842	0.8415	0.23161	-1.1848	1.19
7842	*CLK3*	4.6162	-0.5196	-0.53963	-0.88458	1.19
7843	*SEL1L2*	4.417	1.1015	-1.962	-2.3086	1.19
7844	*HECTD3*	3.5749	0.40139	-0.42014	-0.54345	1.19
7845	*PINLYP*	5.6103	-0.36751	-1.687	-1.8631	1.19
7846	*CTNND2*	5.7283	-0.99768	-1.175	-1.7061	1.19
7847	*INPP5J*	2.8375	1.1074	-0.38933	-0.53947	1.19
7848	*RPAIN*	4.8796	-0.64164	-0.68303	-2.1794	1.18
7849	*ZFP62*	3.3605	1.8761	-1.6824	-1.9023	1.18
7850	*KIF18B*	4.5331	0.19497	-1.1739	-1.7072	1.18
7851	*DAPK3*	3.0869	0.75028	-0.28371	-0.72725	1.18
7852	*SPTSSA*	3.2027	0.95268	-0.60223	-2.2381	1.18
7853	*B4GALT4*	4.8653	-0.54329	-0.76934	-2.6964	1.18
7854	*FAM98C*	2.3687	1.097	0.08672	-1.57	1.18
7855	*BNIPL*	2.6946	1.9988	-1.141	-1.1935	1.18
7856	*SNRPN*	3.9515	-0.12926	-0.26999	-0.7015	1.18
7857	*BBS5*	3.0727	0.91787	-0.43841	-1.057	1.18
7858	*OR1A1*	2.1699	1.6729	-0.29089	-1.2426	1.18
7859	*NCF2*	3.1004	0.30209	0.14941	-0.06021	1.18

7860	*PLCG1*	3.4909	0.31685	-0.2562	-1.2364	1.18
7861	*IL18RAP*	2.5625	1.6373	-0.64851	-1.1665	1.18
7862	*YBX1*	2.5944	2.2089	-1.2525	-1.6542	1.18
7863	*C16orf97*	2.0421	1.1401	0.36806	-1.6712	1.18
7864	*NME7*	1.8375	1.3748	0.33789	-0.01628	1.18
7865	*PEX5L*	3.2328	0.25015	0.06683	0.045836	1.18
7866	*STEAP3*	3.8053	0.022271	-0.27844	-0.72142	1.18
7867	*MGC57346-CRHR1*	4.5951	-0.44661	-0.60003	-2.3552	1.18
7868	*ZNF572*	4.2756	0.15744	-0.88465	-1.448	1.18
7869	*PDK3*	3.4386	0.37011	-0.26093	-1.5954	1.18
7870	*URGCP*	3.9964	0.05116	-0.50067	-1.4509	1.18
7871	*MED10*	3.3362	0.57164	-0.36165	-0.70315	1.18
7872	*CORO7*	3.0049	1.458	-0.91676	-1.0935	1.18
7873	*DUPD1*	3.6617	0.28741	-0.40305	-0.97791	1.18
7874	*ZNF99*	2.414	0.90689	0.22402	-1.3554	1.18
7875	*TAF1B*	3.0567	0.74596	-0.25802	-0.52105	1.18
7876	*KHDC1L*	4.4575	-0.18092	-0.73202	-1.5082	1.18
7877	*KCNG2*	3.0095	0.73559	-0.20105	-1.9107	1.18
7878	*BCO2*	3.1467	1.1578	-0.76055	-1.5022	1.18
7879	*CHST4*	1.6541	1.2003	0.68939	-0.15375	1.18
7880	*TBKBP1*	2.9558	0.33012	0.25756	0.20167	1.18
7881	*MTSS1*	2.662	1.1939	-0.31487	-1.3183	1.18
7882	*BEST3*	3.4017	0.43825	-0.30003	-0.96173	1.18
7883	*HBZ*	2.885	0.75115	-0.09681	-0.54121	1.18
7884	*LMO4*	1.4053	1.1664	0.96756	0.55616	1.18
7885	*SLC39A5*	2.8813	0.85912	-0.20181	-2.6921	1.18
7886	*MS4A6E*	3.0334	1.4498	-0.94546	-1.1598	1.18
7887	*CYB5RL*	2.4309	1.2483	-0.14166	-0.78229	1.18
7888	*ANKRD7*	2.9901	1.7682	-1.2211	-2.7629	1.18
7889	*NAA11*	3.3344	1.1134	-0.91096	-1.3294	1.18
7890	*ABHD17B*	3.9662	0.092673	-0.52255	-1.3207	1.18
7891	*BEND2*	2.1465	1.0699	0.31982	-0.66505	1.18
7892	*CERKL*	2.0343	1.3282	0.17236	-0.20958	1.18
7893	*FAM222B*	4.898	-0.25295	-1.1105	-2.7374	1.18
7894	*ZNF432*	2.6355	0.49518	0.40372	-1.7802	1.18
7895	*POFUT2*	2.3816	1.1659	-0.01388	-0.9363	1.18
7896	*PSG7*	2.9815	1.1732	-0.62154	-1.2071	1.18
7897	*LZTFL1*	2.8355	0.64793	0.04902	-2.4435	1.18
7898	*FAM200A*	2.5896	1.4412	-0.49902	-1.135	1.18
7899	*HLA-E*	2.6848	1.0852	-0.23916	-0.94576	1.18
7900	*CD44*	2.2946	0.6802	0.55551	0.22548	1.18
7901	*MASP1*	4.3936	0.047789	-0.91215	-1.1642	1.18
7902	*PRB4*	3.2906	1.4652	-1.2267	-1.7134	1.18
7903	*RPS6KA4*	1.6585	1.2771	0.59348	-1.6597	1.18
7904	*ASB3*	3.0242	0.34095	0.16383	-0.29114	1.18
7905	*AP5B1*	5.3973	-0.48654	-1.3818	-1.4607	1.18

7906	*IMMP1L*	3.1841	0.37761	-0.03288	-0.95224	1.18
7907	*P2RY13*	2.4911	2.262	-1.2244	-1.2958	1.18
7908	*UGCG*	4.1891	0.012544	-0.67313	-1.1853	1.18
7909	*GPATCH11*	4.6211	0.15112	-1.2442	-1.8157	1.18
7910	*EPX*	3.7398	0.66159	-0.87366	-1.258	1.18
7911	*APOB*	2.4046	0.57679	0.54514	-1.3481	1.18
7912	*MEGF8*	2.1314	1.4935	-0.09869	-1.2775	1.18
7913	*TMEM54*	2.456	2.0735	-1.0036	-4.2747	1.18
7914	*UNC5CL*	3.8173	0.29852	-0.59128	-2.5056	1.17
7915	*EFCAB7*	1.6475	1.1684	0.70854	-0.73443	1.17
7916	*GNB3*	2.228	1.1924	0.10343	-1.1519	1.17
7917	*CLDND1*	4.0786	0.093218	-0.64808	-1.9397	1.17
7918	*KCNK7*	3.7892	0.14449	-0.41079	-1.5696	1.17
7919	*EYA1*	3.6903	1.0558	-1.2242	-1.3255	1.17
7920	*SLC22A24*	2.7919	0.76967	-0.04015	-1.1319	1.17
7921	*EXT1*	3.9866	0.10415	-0.56961	-0.92718	1.17
7922	*HBD*	4.2548	0.040234	-0.77398	-1.4536	1.17
7923	*HIST1H2BB*	3.9282	0.17732	-0.58495	-0.8897	1.17
7924	*KIR2DL1*	2.7663	0.60896	0.14487	-2.3244	1.17
7925	*TEX29*	2.5088	0.60803	0.40329	-0.68133	1.17
7926	*MRPL30*	3.6515	0.27162	-0.40339	-1.232	1.17
7927	*MRPL51*	4.9331	0.048185	-1.463	-1.6826	1.17
7928	*OR4M2*	3.2959	0.27944	-0.05715	-2.223	1.17
7929	*DCPS*	2.7232	1.0098	-0.21487	-1.7268	1.17
7930	*SKIV2L2*	4.0613	1.4077	-1.9511	-2.6635	1.17
7931	*APOL5*	3.183	1.9362	-1.6023	-1.9522	1.17
7932	*ASAH1*	2.4268	1.0614	0.02733	-0.31126	1.17
7933	*UBE2U*	3.2339	0.30505	-0.0235	-0.86725	1.17
7934	*ZNF770*	3.232	0.1435	0.1395	-1.9559	1.17
7935	*COX18*	3.657	0.30138	-0.44353	-1.5337	1.17
7936	*MPC2*	4.6808	0.8179	-1.984	-2.0007	1.17
7937	*RAB11FIP1*	2.2376	1.4019	-0.12485	-1.4415	1.17
7938	*KRT85*	4.3876	0.071812	-0.94511	-2.0277	1.17
7939	*UBL4B*	3.7388	0.35683	-0.58136	-2.1108	1.17
7940	*FBXO7*	3.112	0.35566	0.04626	-1.7512	1.17
7941	*FLJ44635*	3.5546	0.81227	-0.85296	-2.3292	1.17
7942	*COL3A1*	2.5852	1.2116	-0.28294	-1.3573	1.17
7943	*REXO1*	2.1356	1.3166	0.06093	-0.88272	1.17
7944	*CACNB2*	3.7682	0.43155	-0.68706	-0.75665	1.17
7945	*MROH5*	1.9667	1.2091	0.33549	-2.1816	1.17
7946	*MAN2A1*	2.5439	0.95306	0.01427	-0.65368	1.17
7947	*TMUB1*	3.8827	0.002584	-0.37429	-1.2024	1.17
7948	*PHF1*	4.4652	0.36105	-1.3162	-1.5225	1.17
7949	*RASGEF1C*	4.4783	-0.15339	-0.81542	-2.3577	1.17
7950	*CHRNB3*	2.8722	1.5431	-0.90606	-1.3402	1.17
7951	*ZPBP2*	2.5085	2.2887	-1.2884	-1.6105	1.17

7952	*ARNT2*	2.7145	0.78312	0.01027	-1.6935	1.17
7953	*ANXA8L1*	3.8224	0.51973	-0.83436	-2.5125	1.17
7954	*NPM3*	2.3679	1.2864	-0.14868	-1.6663	1.17
7955	*RFTN1*	4.2976	-0.13757	-0.65453	-2.9283	1.17
7956	*LACRT*	3.1284	0.48073	-0.10397	-1.2067	1.17
7957	*XIRP2*	2.6175	1.1079	-0.22082	-1.8869	1.17
7958	*GPC1*	4.6104	-0.29271	-0.81334	-1.433	1.17
7959	*ACVR1B*	4.0561	-0.09356	-0.45867	-1.6664	1.17
7960	*LRIT2*	2.4824	1.9457	-0.92485	-1.2226	1.17
7961	*AGAP2*	4.0333	0.50033	-1.0315	-2.0016	1.17
7962	*ANKRD27*	3.2761	0.36462	-0.13947	-0.29579	1.17
7963	*CDCA5*	1.3496	1.2433	0.90816	-1.3749	1.17
7964	*KRT37*	1.6201	1.1862	0.69416	0.2834	1.17
7965	*CHMP3*	3.1337	0.18941	0.17725	-0.99182	1.17
7966	*C20orf144*	1.638	1.0649	0.79737	-0.27548	1.17
7967	*EXTL2*	3.565	0.44829	-0.51468	-1.6688	1.17
7968	*TEAD3*	2.7577	0.76313	-0.0224	-0.73342	1.17
7969	*PNMA6A*	3.8416	0.16445	-0.50828	-1.4151	1.17
7970	*SIAH1*	1.6937	0.92894	0.87499	-1.5364	1.17
7971	*NTM*	3.2406	0.66003	-0.40374	-2.0682	1.17
7972	*PKIG*	3.0787	0.58533	-0.16814	-1.468	1.17
7973	*ALDH5A1*	3.5437	0.045209	-0.09334	-0.28742	1.17
7974	*SMAP2*	2.3269	1.0403	0.12773	-1.2226	1.16
7975	*CNNM4*	1.9831	1.9158	-0.40406	-0.7331	1.16
7976	*RAB18*	3.5091	1.4975	-1.5121	-2.0878	1.16
7977	*PCYT1B*	4.0377	0.24637	-0.78963	-1.3198	1.16
7978	*ZNF540*	2.3578	1.9111	-0.77475	-1.0158	1.16
7979	*CCDC136*	2.0129	0.91268	0.56846	-2.283	1.16
7980	*NXF1*	1.7399	1.4057	0.34841	-1.3742	1.16
7981	*FRS3*	3.2736	1.543	-1.3226	-1.8157	1.16
7982	*BRF1*	1.9682	0.82051	0.70495	-1.5568	1.16
7983	*FGFR4*	2.7076	1.7785	-0.99277	-1.9608	1.16
7984	*CDHR1*	2.8985	1.0383	-0.44353	-0.75953	1.16
7985	*PLSCR1*	3.3646	0.11948	0.00876	-2.4018	1.16
7986	*ZG16B*	1.2671	1.2623	0.96313	-2.4815	1.16
7987	*NDUFB5*	3.4631	0.82472	-0.79548	-0.79745	1.16
7988	*ASS1*	2.4029	0.61574	0.47368	-1.1447	1.16
7989	*HIPK2*	2.0253	1.6855	-0.21852	-1.0359	1.16
7990	*TSHZ2*	4.9746	-0.19793	-1.2846	-1.8799	1.16
7991	*C8orf34*	2.5675	0.59519	0.32887	-1.893	1.16
7992	*PHTF2*	4.3133	-0.38495	-0.43694	-1.5598	1.16
7993	*OR9Q1*	5.1959	-0.47818	-1.2264	-1.7845	1.16
7994	*ADCK1*	4.0334	0.30562	-0.8478	-1.274	1.16
7995	*OR52K1*	3.1251	0.7875	-0.42195	-0.73911	1.16
7996	*FUNDC2*	3.1242	1.3248	-0.95893	-1.9734	1.16
7997	*MAP3K3*	3.9514	0.82601	-1.2884	-1.6711	1.16

7998	*HIST1H2BK*	2.5769	1.9847	-1.0733	-1.6419	1.16
7999	*OLFM3*	4.2513	0.41759	-1.1807	-1.7512	1.16
8000	*GNPTG*	2.472	0.64864	0.36689	-1.0033	1.16
8001	*VSIG10L*	3.7018	0.53353	-0.74819	-1.3465	1.16
8002	*PXDNL*	3.4351	0.68534	-0.63357	-0.72095	1.16
8003	*PBX1*	2.7387	1.5264	-0.77853	-1.5426	1.16
8004	*RRP1B*	2.365	1.0572	0.06333	-1.0795	1.16
8005	*TRPS1*	4.5395	0.42831	-1.4824	-1.7364	1.16
8006	*PARP6*	4.209	0.44498	-1.1696	-1.4524	1.16
8007	*PDZD4*	3.2005	0.71604	-0.4323	-0.72493	1.16
8008	*PRIMPOL*	3.7527	1.2836	-1.5522	-1.8724	1.16
8009	*LOC388780*	3.1978	0.62119	-0.33503	-0.73896	1.16
8010	*KCTD8*	2.434	0.91407	0.1358	-1.5418	1.16
8011	*KBTBD12*	3.8396	0.30136	-0.65768	-0.7463	1.16
8012	*UBAC1*	4.3325	-0.21563	-0.63405	-1.6456	1.16
8013	*FAM227B*	4.0901	0.031661	-0.63903	-0.74568	1.16
8014	*ZNF280B*	4.2934	-0.0509	-0.7602	-1.0932	1.16
8015	*ZZZ3*	3.5835	0.48995	-0.59217	-2.0851	1.16
8016	*SMYD4*	2.6351	1.6453	-0.79987	-2.2329	1.16
8017	*ZNF558*	3.6574	0.1136	-0.29082	-1.8405	1.16
8018	*C1orf54*	1.7066	0.9847	0.78807	-1.3978	1.16
8019	*CLIC6*	4.1708	0.038212	-0.73155	-0.99049	1.16
8020	*LY6G6C*	1.8183	1.47	0.18841	-0.87312	1.16
8021	*KLHL3*	2.6271	1.9963	-1.1468	-1.9431	1.16
8022	*SYNDIG1*	3.512	0.51334	-0.54887	-1.5647	1.16
8023	*OR6C1*	3.4163	0.03422	0.02538	-1.3728	1.16
8024	*TMEM38A*	2.6231	1.1169	-0.26446	-2.2578	1.16
8025	*KLF13*	3.9723	-0.12866	-0.36913	-0.40962	1.16
8026	*RGPD5*	3.7319	0.4195	-0.67707	-0.78766	1.16
8027	*KRT5*	3.4063	0.51143	-0.44353	-0.60541	1.16
8028	*TNNI1*	5.0177	0.008772	-1.5524	-2.425	1.16
8029	*LHX8*	3.26	0.47249	-0.26018	-0.62489	1.16
8030	*ARL8B*	3.3793	0.18234	-0.08993	-1.8583	1.16
8031	*C12orf43*	1.4123	1.2007	0.85843	-2.3552	1.16
8032	*MEI4*	3.2197	0.32373	-0.07232	-1.1739	1.16
8033	*MYO19*	2.8555	0.52622	0.08877	-1.1354	1.16
8034	*LPPR3*	4.6794	-0.18461	-1.0256	-1.4682	1.16
8035	*ATP6V0D2*	4.8586	0.14783	-1.5375	-1.5598	1.16
8036	*EXTL3*	2.8407	0.56773	0.06023	-0.0708	1.16
8037	*C4orf3*	3.7833	0.29438	-0.6093	-1.0889	1.16
8038	*POU4F3*	2.2368	1.2575	-0.02594	-1.0217	1.16
8039	*C9orf89*	3.0536	1.1324	-0.71785	-1.6213	1.16
8040	*SKOR2*	2.3554	1.4071	-0.29457	-0.42279	1.16
8041	*DCBLD1*	1.4591	1.2843	0.72432	-1.2008	1.16
8042	*SCGB1D1*	2.7196	0.98327	-0.23523	-0.78725	1.16
8043	*IMPAD1*	3.968	-0.04845	-0.45222	-0.63187	1.16

8044	*H2AFZ*	2.1443	0.73717	0.5851	-0.01839	1.16
8045	*HSPB1*	3.44	0.73702	-0.71051	-1.5235	1.16
8046	*PPP1R16B*	3.0853	0.89908	-0.51796	-2.033	1.16
8047	*AHSP*	2.4583	0.61099	0.39693	-1.9844	1.16
8048	*AGT*	4.8052	-0.0378	-1.3026	-1.4783	1.15
8049	*CACNA1G*	4.2035	1.0767	-1.8157	-2.278	1.15
8050	*ARHGAP8*	1.9531	0.98367	0.52723	-0.90834	1.15
8051	*ORAI1*	2.7074	0.69369	0.06152	-2.1523	1.15
8052	*LYRM2*	1.4583	1.1543	0.84982	0.66539	1.15
8053	*MAGEB4*	3.2428	0.22738	-0.00822	-0.59136	1.15
8054	*ZNF740*	4.0111	-0.21666	-0.33268	-1.0777	1.15
8055	*MLST8*	4.082	-0.07341	-0.54734	-2.4858	1.15
8056	*NRSN1*	2.8971	1.2512	-0.68811	-1.3345	1.15
8057	*ONECUT2*	4.8326	-0.48968	-0.88503	-2.2888	1.15
8058	*KRT78*	2.9034	1.1689	-0.61483	-2.1143	1.15
8059	*SSX4*	3.0154	0.81559	-0.3744	-1.1126	1.15
8060	*CBR4*	2.8019	2.1476	-1.4932	-3.3669	1.15
8061	*OTUD7B*	3.8568	0.352	-0.7532	-1.8282	1.15
8062	*ZNF660*	3.0009	0.66964	-0.21539	-0.7873	1.15
8063	*DNM1*	2.9349	0.46691	0.05282	-0.10031	1.15
8064	*CWH43*	4.7527	-0.08123	-1.217	-1.6524	1.15
8065	*TENM2*	3.5975	0.4902	-0.63348	-2.1478	1.15
8066	*LOC100129697*	2.4002	1.8754	-0.82255	-0.90922	1.15
8067	*C9orf41*	3.0027	0.6318	-0.18229	-0.20425	1.15
8068	*NANOS2*	4.4009	0.20541	-1.1543	-1.855	1.15
8069	*SEC61A1*	3.599	0.43086	-0.57787	-0.7492	1.15
8070	*TUBA4A*	3.1836	1.597	-1.329	-1.3865	1.15
8071	*FBXL7*	4.252	0.11283	-0.91343	-1.0726	1.15
8072	*PP2D1*	2.7785	0.98977	-0.31688	-2.0476	1.15
8073	*CAV3*	2.7209	0.80615	-0.07588	-0.18786	1.15
8074	*SLA*	4.3005	-0.22469	-0.62514	-0.72819	1.15
8075	*MYD88*	4.3273	0.29629	-1.1733	-1.4985	1.15
8076	*PGGT1B*	3.3922	1.4769	-1.4192	-1.7802	1.15
8077	*PPP1CA*	1.3753	1.0449	1.0295	-0.51886	1.15
8078	*ADAMTS17*	4.7623	-0.19404	-1.1199	-1.5526	1.15
8079	*IRAK2*	3.2243	0.23089	-0.00686	-0.14513	1.15
8080	*OLIG2*	3.4725	0.10278	-0.12795	-1.7865	1.15
8081	*PXT1*	2.9144	0.49825	0.03406	-0.89068	1.15
8082	*GIPC2*	2.823	1.9433	-1.3199	-1.3957	1.15
8083	*CFAP36*	4.6832	0.046655	-1.2839	-1.8751	1.15
8084	*LETM2*	3.1507	0.28236	0.01276	-0.5584	1.15
8085	*EVI5L*	1.8164	1.178	0.451	-2.8019	1.15
8086	*C6*	2.66	0.71412	0.07057	-1.6597	1.15
8087	*PIGB*	2.4216	2.0386	-1.0158	-2.069	1.15
8088	*PRAC1*	4.2548	-0.0391	-0.77289	-1.9511	1.15
8089	*CYTL1*	2.3983	1.7751	-0.73199	-0.9858	1.15

8090	*KLHL8*	2.2014	0.9997	0.24016	-1.5632	1.15
8091	*ALPK1*	2.0876	1.0011	0.35245	-1.3996	1.15
8092	*CGA*	4.5869	-0.12108	-1.0248	-1.3808	1.15
8093	*HELZ*	3.6876	0.21634	-0.46333	-1.3585	1.15
8094	*PISD*	4.1891	0.80248	-1.5524	-1.9891	1.15
8095	*SOX18*	2.7848	0.80445	-0.15141	-2.9338	1.15
8096	*WNT7B*	3.389	0.13556	-0.08748	-0.2595	1.15
8097	*TRIM17*	3.9696	-0.19524	-0.33806	-0.83169	1.15
8098	*ZDHHC5*	2.8061	1.507	-0.87725	-2.746	1.15
8099	*MTFR1*	3.1425	0.1477	0.14546	-1.3496	1.15
8100	*PAM16*	5.2666	-0.82757	-1.0041	-4.1213	1.14
8101	*SAXO2*	3.4349	0.59593	-0.59622	-1.5568	1.14
8102	*BTBD19*	2.2559	1.0507	0.12772	-0.64707	1.14
8103	*IL2RB*	2.7006	0.88367	-0.15085	-2.2319	1.14
8104	*SLC25A53*	3.0913	0.47851	-0.13656	-1.2019	1.14
8105	*KRTAP4-8*	2.3405	1.364	-0.27144	-1.5315	1.14
8106	*DTWD2*	2.7829	0.57913	0.0708	0.052769	1.14
8107	*ZNF354A*	2.8512	0.38252	0.19869	-1.4206	1.14
8108	*ZNF470*	1.6105	1.2362	0.58504	-1.808	1.14
8109	*BOLA1*	2.9303	1.2555	-0.75504	-2.6543	1.14
8110	*PAPL*	1.7505	1.2463	0.43325	-0.53961	1.14
8111	*ALOX5*	2.2334	0.96268	0.2336	-2.2078	1.14
8112	*C9orf116*	4.1878	-0.04919	-0.71009	-1.9619	1.14
8113	*CCDC85B*	4.6756	-0.24946	-0.99768	-2.4101	1.14
8114	*CLRN2*	4.3759	-0.22977	-0.71768	-1.2426	1.14
8115	*KIF14*	2.6527	2.2014	-1.4258	-2.1155	1.14
8116	*ZPR1*	2.743	0.42629	0.25886	-0.30608	1.14
8117	*PCDHB8*	2.6207	0.57608	0.23058	-0.68896	1.14
8118	*OVOL3*	2.7189	1.1646	-0.4571	-1.9116	1.14
8119	*NAA35*	2.9074	1.5014	-0.98241	-1.4789	1.14
8120	*ANP32B*	3.9235	0.2606	-0.75778	-1.3353	1.14
8121	*MLXIP*	4.2353	0.019024	-0.82833	-1.1743	1.14
8122	*RAX2*	3.6599	0.11094	-0.34536	-0.83527	1.14
8123	*AKR1B10*	2.2128	1.3457	-0.13369	-1.0153	1.14
8124	*MAP4K3*	3.1743	0.66025	-0.41031	-1.2438	1.14
8125	*FBXO44*	2.7045	1.006	-0.2863	-0.70618	1.14
8126	*FLG2*	3.6416	1.468	-1.6855	-2.3235	1.14
8127	*MRPL11*	3.9111	0.90907	-1.3973	-2.1261	1.14
8128	*BUD13*	4.9948	0.1718	-1.7438	-2.8085	1.14
8129	*ZNF131*	1.6208	1.3404	0.46135	-1.5795	1.14
8130	*ZNF839*	3.5332	1.0354	-1.1474	-1.2855	1.14
8131	*CSAG1*	1.7738	1.5012	0.14601	-0.01918	1.14
8132	*IRX3*	3.198	0.32903	-0.10815	-2.4012	1.14
8133	*SHISA8*	2.2197	1.2403	-0.04154	-0.5893	1.14
8134	*ACMSD*	3.2632	0.72312	-0.56963	-1.4999	1.14
8135	*USP17L7*	2.1391	1.5739	-0.2969	-0.35892	1.14

8136	*NOTUM*	3.1726	0.26202	-0.01938	-2.2766	1.14
8137	*APOA1BP*	3.0399	0.32908	0.04479	-1.4082	1.14
8138	*ZNF526*	2.9196	0.62365	-0.12998	-1.3007	1.14
8139	*PGAP1*	2.461	2.3217	-1.3702	-2.1461	1.14
8140	*BRE*	3.8963	0.70199	-1.1872	-1.7658	1.14
8141	*CHP1*	3.8538	0.021013	-0.46382	-1.5	1.14
8142	*C15orf41*	2.9883	1.1149	-0.69262	-1.6386	1.14
8143	*HOXD8*	1.8203	0.93503	0.65508	-1.0343	1.14
8144	*C4orf48*	4.646	-0.52343	-0.71238	-1.1417	1.14
8145	*ADPRHL2*	1.8943	1.3751	0.14058	-0.99137	1.14
8146	*WFIKKN1*	1.6455	1.0043	0.75948	0.17981	1.14
8147	*TMEM248*	1.7881	1.0263	0.59329	-0.8397	1.14
8148	*CELF1*	1.9632	1.6195	-0.17509	-0.66973	1.14
8149	*TNS3*	2.7903	2.0731	-1.4563	-1.5696	1.14
8150	*SRRD*	1.8108	1.1106	0.48551	-1.6284	1.14
8151	*DSTYK*	3.795	0.021801	-0.41012	-0.62684	1.14
8152	*KLF15*	1.3481	1.1016	0.95668	-0.01366	1.14
8153	*MTRNR2L3*	3.5298	0.40422	-0.52764	-1.7251	1.14
8154	*PLGRKT*	2.1966	0.88742	0.32159	-1.017	1.14
8155	*C1orf110*	1.8528	1.7029	-0.15062	-0.58231	1.14
8156	*NOTO*	5.2857	-0.49724	-1.3837	-1.7802	1.13
8157	*NBEA*	2.7625	0.63882	0.00149	-0.1394	1.13
8158	*FERMT1*	2.7251	1.0196	-0.34227	-1.0494	1.13
8159	*HAS3*	3.0566	0.26862	0.07621	-1.6007	1.13
8160	*HIST1H4A*	3.0266	0.87268	-0.49812	-1.222	1.13
8161	*ZSCAN31*	2.0508	0.98974	0.36048	-0.71032	1.13
8162	*KRTAP5-7*	2.6924	0.38885	0.31941	-0.20946	1.13
8163	*ADGRL1*	2.9625	0.51107	-0.07312	-1.9589	1.13
8164	*SDCCAG8*	2.9947	0.32118	0.08359	-0.1875	1.13
8165	*BUB1*	3.8747	1.1091	-1.5849	-2.5103	1.13
8166	*OR6C75*	4.5992	-0.08253	-1.1189	-1.4064	1.13
8167	*SPACA7*	1.5496	1.0378	0.80961	-1.122	1.13
8168	*ARHGAP20*	2.5109	0.74	0.14551	-1.2815	1.13
8169	*IL6*	2.0217	0.90388	0.47012	-1.9846	1.13
8170	*NSUN6*	4.2248	0.1475	-0.97667	-1.3153	1.13
8171	*NXPE2*	2.3158	0.56262	0.51709	-0.62862	1.13
8172	*INAFM1*	4.204	0.42497	-1.2338	-1.9338	1.13
8173	*CHRNB2*	4.2387	-0.08672	-0.75717	-1.3078	1.13
8174	*JPH4*	1.7516	1.4127	0.22939	-0.16753	1.13
8175	*RGSL1*	2.4804	0.46861	0.44453	0.15487	1.13
8176	*COX7A1*	3.3722	0.41085	-0.38975	-1.0224	1.13
8177	*FPGT*	3.09	0.8936	-0.59061	-1.0364	1.13
8178	*MMP28*	2.59	0.89123	-0.08956	-1.0315	1.13
8179	*TCHHL1*	2.6461	0.6642	0.08106	-1.5679	1.13
8180	*RASGEF1B*	3.8172	0.90586	-1.3322	-1.505	1.13
8181	*HOXB2*	1.3846	1.2797	0.72654	-1.6731	1.13

8182	*THAP3*	2.4217	1.135	-0.16614	-1.0186	1.13
8183	*ADD3*	3.6932	0.49515	-0.79821	-3.1519	1.13
8184	*PATE3*	4.8264	-0.59013	-0.84687	-1.5312	1.13
8185	*CISH*	3.6429	0.31268	-0.56659	-0.82663	1.13
8186	*AKAP7*	4.3182	-0.13757	-0.79282	-1.6527	1.13
8187	*ANKRD13D*	4.9371	-0.256	-1.2936	-1.6119	1.13
8188	*ERLIN1*	3.8034	-0.00829	-0.40805	-1.5364	1.13
8189	*SYNGR4*	2.5642	0.58139	0.24131	0.077067	1.13
8190	*DCLK2*	3.3473	0.028984	0.01047	-0.31035	1.13
8191	*S1PR5*	2.5726	0.84658	-0.03246	-1.3554	1.13
8192	*PLOD2*	4.4203	-0.50048	-0.53406	-0.81505	1.13
8193	*CNTF*	4.9109	-0.05759	-1.4685	-1.7407	1.13
8194	*OR4C16*	3.4612	0.71758	-0.79663	-1.0211	1.13
8195	*CDH8*	1.5823	1.0849	0.71474	-1.6597	1.13
8196	*CDA*	3.223	1.293	-1.135	-1.2837	1.13
8197	*TMEM109*	4.7253	0.13174	-1.4773	-1.696	1.13
8198	*CRX*	2.2558	1.3924	-0.26949	-1.2412	1.13
8199	*NICN1*	3.7807	0.065124	-0.46716	-0.75619	1.13
8200	*GPR37L1*	3.4605	0.36129	-0.44353	-1.7908	1.13
8201	*CFAP44*	2.324	0.56974	0.4845	-1.0296	1.13
8202	*BCL3*	2.0859	0.74962	0.54217	-1.942	1.13
8203	*SPA17*	2.6136	0.85067	-0.08719	-0.18393	1.13
8204	*PELI1*	4.2848	0.17781	-1.0858	-1.6065	1.13
8205	*LSM12*	4.0669	-0.18185	-0.50837	-0.78594	1.13
8206	*CFAP58*	3.9894	0.90538	-1.5182	-2.2352	1.13
8207	*FKRP*	4.5391	-0.54851	-0.61437	-1.1945	1.13
8208	*MPO*	1.9747	1.8574	-0.45628	-1.8212	1.13
8209	*TRMT6*	4.4962	-0.06464	-1.0561	-1.1096	1.13
8210	*ANGPTL3*	2.4396	1.1459	-0.21205	-0.5655	1.12
8211	*A1BG*	3.4324	0.19996	-0.25912	-0.44353	1.12
8212	*SLC9A3*	4.4497	0.077878	-1.1547	-1.5956	1.12
8213	*CDC23*	4.3662	0.016066	-1.0094	-1.9442	1.12
8214	*PTPN1*	3.4289	1.6428	-1.6989	-1.7908	1.12
8215	*METTL7A*	4.6248	-0.57506	-0.67764	-0.78482	1.12
8216	*VPS4A*	3.1368	0.1466	0.08735	-1.7639	1.12
8217	*CALCRL*	3.5083	0.99725	-1.1354	-2.8849	1.12
8218	*SH3RF2*	3.4379	0.309	-0.37702	-1.7646	1.12
8219	*PKD2*	1.4277	1.1897	0.75228	0.34745	1.12
8220	*NRDE2*	4.8744	0.13368	-1.6386	-2.0791	1.12
8221	*MCF2*	2.9929	0.62505	-0.2486	-0.61767	1.12
8222	*ABHD6*	1.5816	1.0477	0.73963	-0.0159	1.12
8223	*SLC25A27*	1.8972	1.6453	-0.17383	-0.57701	1.12
8224	*RNASE13*	3.4294	0.44696	-0.50855	-1.3292	1.12
8225	*RGR*	2.1933	1.2359	-0.06182	-1.9249	1.12
8226	*DLG2*	1.6253	1.1807	0.56093	-0.7351	1.12
8227	*PXK*	3.1026	1.7204	-1.4561	-1.6913	1.12

8228	*ARL14EP*	1.6741	1.6675	0.02366	-0.95708	1.12
8229	*RPS2*	3.3803	-0.00827	-0.01068	-2.1347	1.12
8230	*PTGES3L*	3.0384	0.76604	-0.44353	-0.7329	1.12
8231	*FAM104A*	3.6122	0.67648	-0.92781	-2.3036	1.12
8232	*GAL3ST1*	2.3903	0.72074	0.24975	-0.02864	1.12
8233	*STX1A*	3.7678	-0.01264	-0.39468	-1.0126	1.12
8234	*RNLS*	3.6332	-0.0996	-0.17345	-0.43674	1.12
8235	*RPS12*	2.4757	1.8423	-0.95838	-1.4809	1.12
8236	*ADCYAP1*	2.6516	1.4291	-0.72201	-2.5878	1.12
8237	*LAPTM5*	1.8652	1.1042	0.38873	-1.9091	1.12
8238	*GRIFIN*	2.7626	0.83474	-0.23931	-0.32004	1.12
8239	*CCDC126*	2.8289	1.4298	-0.9007	-1.8219	1.12
8240	*ENTHD1*	3.3437	0.3533	-0.34014	-1.7495	1.12
8241	*RAD54B*	3.3073	1.9234	-1.8744	-2.8647	1.12
8242	*NAA50*	4.3762	-0.06876	-0.9512	-3.2856	1.12
8243	*GRAPL*	2.5253	0.72043	0.10907	-0.25484	1.12
8244	*UTF1*	5.0169	-0.74965	-0.91269	-4.7884	1.12
8245	*PI15*	2.2055	1.5761	-0.42818	-1.4486	1.12
8246	*HTN3*	3.5838	0.076911	-0.3077	-2.5645	1.12
8247	*SIRPA*	4.01	-0.27376	-0.38402	-1.4273	1.12
8248	*TSPAN33*	2.4297	0.77392	0.14852	-0.227	1.12
8249	*C7orf25*	2.6481	0.82438	-0.12153	-1.3445	1.12
8250	*ATG10*	2.0992	0.66966	0.58006	0.27802	1.12
8251	*OPTC*	3.217	0.70344	-0.57172	-0.99574	1.12
8252	*KRTAP26-1*	3.2931	0.89643	-0.84158	-1.2195	1.12
8253	*FRA10AC1*	2.335	0.67948	0.3327	-0.726	1.12
8254	*TAF12*	4.0067	0.28371	-0.94333	-2.0277	1.12
8255	*RECK*	3.1154	0.68874	-0.45727	-1.8909	1.12
8256	*XCL1*	4.9431	0.14337	-1.7397	-2.5419	1.12
8257	*KLC1*	2.6623	0.78772	-0.10329	-0.53999	1.12
8258	*MARCH7*	2.4556	1.528	-0.63691	-2.588	1.12
8259	*NCAPH2*	2.3607	0.85271	0.13273	-0.44135	1.12
8260	*USP17L12*	2.7914	1.1299	-0.57579	-0.87208	1.12
8261	*IQCJ*	4.8221	-0.2317	-1.2451	-2.4709	1.12
8262	*PRR11*	3.6195	0.52921	-0.80395	-1.2306	1.11
8263	*NAB2*	1.6813	1.0497	0.61354	0.49645	1.11
8264	*RIMBP3C*	3.4032	0.67328	-0.73219	-1.0012	1.11
8265	*GYPC*	1.6346	0.87981	0.82888	-0.61353	1.11
8266	*CHD3*	5.0368	-0.66362	-1.03	-1.3473	1.11
8267	*DAB2*	3.6681	0.31609	-0.64117	-2.4151	1.11
8268	*DPP9-AS1*	2.1917	1.9148	-0.76366	-1.4682	1.11
8269	*AJAP1*	3.0906	0.50002	-0.24797	-1.1156	1.11
8270	*KRTAP12-3*	2.5106	0.7968	0.03497	-0.62372	1.11
8271	*RAB31*	2.9204	0.85297	-0.43107	-1.106	1.11
8272	*SIGLEC8*	2.0986	1.4923	-0.24926	-0.73826	1.11
8273	*CORO2A*	2.7446	1.2848	-0.68889	-1.2475	1.11

8274	*EPB41*	3.4503	0.57298	-0.68303	-1.8869	1.11
8275	*PIP*	2.535	1.6504	-0.84558	-0.90772	1.11
8276	*CERS5*	3.666	0.41322	-0.74086	-1.8631	1.11
8277	*TCEB3*	2.1416	0.78541	0.41125	-1.615	1.11
8278	*EFHD1*	3.5083	0.16218	-0.33231	-1.7547	1.11
8279	*SPATA31C2*	1.6932	1.3427	0.30154	-0.70988	1.11
8280	*SIX2*	3.5304	1.7306	-1.9237	-2.5057	1.11
8281	*GLUD2*	2.6745	1.14	-0.47743	-1.0267	1.11
8282	*CCDC93*	2.3806	0.57773	0.37868	-1.6984	1.11
8283	*FIGNL2*	2.6	0.77087	-0.03476	-0.91842	1.11
8284	*FGF4*	2.0786	0.69682	0.56035	-1.2541	1.11
8285	*CIRH1A*	5.1245	-0.85606	-0.93383	-1.545	1.11
8286	*HTR1F*	3.5514	-0.06269	-0.15416	-1.0564	1.11
8287	*TRMU*	3.7969	0.67421	-1.1369	-1.2511	1.11
8288	*ALB*	2.9172	1.8698	-1.4538	-2.3684	1.11
8289	*TEDDM1*	2.6583	0.63668	0.0369	-0.90028	1.11
8290	*GTF2H2C*	1.9587	1.8516	-0.47864	-2.6463	1.11
8291	*IPO7*	2.7233	0.44434	0.16383	-1.0956	1.11
8292	*EFNB1*	4.2082	0.023261	-0.90028	-2.1179	1.11
8293	*OLFM4*	3.8505	0.38541	-0.90492	-1.0277	1.11
8294	*AMMECR1*	3.3646	0.63264	-0.66652	-2.2108	1.11
8295	*PLEKHG3*	2.8808	0.57025	-0.12054	-1.1282	1.11
8296	*LPP*	1.5787	1.2907	0.46058	-0.03146	1.11
8297	*CNRIP1*	3.3743	0.003701	-0.04817	-0.84844	1.11
8298	*USP17L22*	2.3194	0.77323	0.23632	-0.07031	1.11
8299	*RABL6*	3.1822	0.75372	-0.60701	-2.4197	1.11
8300	*TNIK*	3.9146	0.60934	-1.1953	-1.7855	1.11
8301	*FFAR2*	4.2896	-0.00355	-0.95794	-1.8392	1.11
8302	*DYSF*	1.4337	1.2124	0.6804	-0.88039	1.11
8303	*OR2AP1*	4.0225	-0.28173	-0.41561	-1.2137	1.11
8304	*CYP4F8*	2.3637	1.331	-0.36957	-1.0623	1.11
8305	*MICALL2*	3.81	0.75411	-1.2393	-1.2955	1.11
8306	*ZNF488*	4.779	0.20146	-1.6559	-1.8256	1.11
8307	*HHAT*	4.1571	0.23996	-1.0727	-1.6934	1.11
8308	*HTRA4*	2.2167	1.8567	-0.75059	-1.0769	1.11
8309	*GSTK1*	2.0787	1.2128	0.0303	-1.5063	1.11
8310	*TOM1*	1.7547	1.5397	0.02597	-0.2372	1.11
8311	*RPS5*	2.9426	0.32677	0.05003	-1.1965	1.11
8312	*DHH*	3.4075	0.003685	-0.09208	-1.1977	1.11
8313	*GLIPR1L1*	1.5954	1.2111	0.51192	-2.7813	1.11
8314	*BAGE2*	2.6795	1.5038	-0.86506	-1.5805	1.11
8315	*VIMP*	2.9652	1.0838	-0.73172	-1.8253	1.11
8316	*MAN2A2*	1.8199	0.84625	0.65096	-1.175	1.11
8317	*ZSWIM5*	2.356	1.6588	-0.69787	-1.0215	1.11
8318	*AZIN1*	3.7466	0.70864	-1.1387	-3.031	1.11
8319	*SYNM*	4.0633	0.3674	-1.1144	-1.3287	1.11

8320	*AFG3L2*	3.2364	0.25096	-0.17208	-1.7467	1.11
8321	*HIST3H2A*	4.0697	1.3872	-2.1421	-2.5453	1.10
8322	*FRK*	2.8238	0.65221	-0.16176	-0.73348	1.10
8323	*HOXC8*	2.7179	0.36628	0.2299	-0.56938	1.10
8324	*SLC6A17*	2.4425	1.5389	-0.66773	-1.0507	1.10
8325	*KCNMB2*	3.6917	0.032031	-0.41025	-0.8903	1.10
8326	*CACNG1*	2.5303	0.80808	-0.02502	-0.02697	1.10
8327	*IHH*	4.3186	0.061681	-1.0671	-1.3264	1.10
8328	*PDZK1IP1*	2.2469	1.2955	-0.22972	-0.41476	1.10
8329	*DNAJC6*	4.6637	-0.198	-1.154	-1.6251	1.10
8330	*WIPF2*	3.0434	0.50218	-0.23418	-0.87395	1.10
8331	*AP3S2*	3.1248	0.14975	0.03663	-2.0116	1.10
8332	*ERMAP*	2.7365	1.018	-0.44353	-1.5128	1.10
8333	*IQCF3*	1.392	1.1055	0.81272	-0.26161	1.10
8334	*TRIM60*	1.6613	1.1425	0.50615	-0.7623	1.10
8335	*CCDC112*	5.4669	-0.94052	-1.2165	-1.6992	1.10
8336	*RRH*	2.4862	1.1587	-0.3351	-2.1151	1.10
8337	*PSMB9*	1.8833	0.85162	0.57427	-2.9538	1.10
8338	*SYCE2*	1.8857	1.8218	-0.39872	-0.53971	1.10
8339	*FGG*	2.771	0.69165	-0.15424	-1.882	1.10
8340	*PADI1*	1.5377	1.2489	0.5212	-1.0168	1.10
8341	*PEX11A*	2.9531	0.65132	-0.29729	-1.515	1.10
8342	*LILRB5*	4.1281	0.1267	-0.94859	-1.358	1.10
8343	*NOMO3*	3.5564	0.37721	-0.62747	-1.5232	1.10
8344	*GTSE1*	3.7277	0.60138	-1.0231	-1.2393	1.10
8345	*SEPT1*	4.7208	-0.64888	-0.76635	-2.3251	1.10
8346	*OR7A5*	3.8877	0.32919	-0.91165	-2.2385	1.10
8347	*CCDC70*	2.3749	0.79265	0.13672	-0.90468	1.10
8348	*LYN*	3.0482	0.39853	-0.14252	-1.5255	1.10
8349	*SFXN2*	2.519	1.228	-0.44353	-1.2655	1.10
8350	*SLC12A5*	1.7968	1.1444	0.36195	0.16792	1.10
8351	*KIAA1755*	2.5295	0.65313	0.12019	-2.9564	1.10
8352	*CSF3*	2.6016	0.51931	0.18166	-0.25839	1.10
8353	*ZFP36L2*	3.8147	0.15971	-0.67199	-1.3988	1.10
8354	*FAM173A*	3.1852	0.076377	0.04083	-0.53785	1.10
8355	*NKX6-3*	3.1102	0.26472	-0.07341	-1.3912	1.10
8356	*LYAR*	2.1324	1.0646	0.10419	-1.5672	1.10
8357	*CACNG6*	1.5166	1.3145	0.46987	-1.0337	1.10
8358	*CFAP47*	3.5544	1.0226	-1.2763	-1.8112	1.10
8359	*HIGD1C*	2.5308	1.365	-0.59511	-1.2324	1.10
8360	*PKM*	3.5985	0.86236	-1.1605	-1.716	1.10
8361	*LRRC75B*	4.1308	-0.41207	-0.4187	-0.93011	1.10
8362	*GPX1*	2.9941	0.60057	-0.29546	-1.1666	1.10
8363	*MTERF3*	3.1113	0.74287	-0.55528	-1.2703	1.10
8364	*PALM2-AKAP2*	3.4033	0.44829	-0.55288	-1.6891	1.10
8365	*RAB30*	3.7592	1.719	-2.1798	-2.201	1.10

8366	*C9orf66*	3.3672	1.4751	-1.5447	-1.545	1.10
8367	*HMGB2*	1.7276	1.4971	0.07273	-0.24137	1.10
8368	*TIMM23B*	3.3857	0.10638	-0.19494	-0.9879	1.10
8369	*DNAH3*	5.379	-0.70074	-1.3814	-2.0062	1.10
8370	*MRPL23*	3.1417	1.1899	-1.0351	-1.1135	1.10
8371	*MGST1*	2.3394	1.1475	-0.19054	-0.73781	1.10
8372	*SAPCD1*	1.5938	1.074	0.62787	-0.97237	1.10
8373	*ODF3*	4.6633	-0.07557	-1.2927	-1.9625	1.10
8374	*CD300LF*	2.3739	0.54622	0.37489	-0.4674	1.10
8375	*PLBD1*	3.7357	-0.20695	-0.23481	-0.4674	1.10
8376	*SYNC*	4.2791	-0.20361	-0.78221	-2.5806	1.10
8377	*PMM1*	3.7551	0.61838	-1.0814	-2.4642	1.10
8378	*PCDH8*	1.3778	1.3669	0.54736	-2.2993	1.10
8379	*LSS*	4.5599	-0.40353	-0.86435	-1.6243	1.10
8380	*SLC2A7*	1.3559	1.0074	0.92779	0.049777	1.10
8381	*ANGPT2*	1.8272	1.3953	0.06847	-0.94841	1.10
8382	*KIF19*	1.7327	1.153	0.40496	-0.34251	1.10
8383	*LIFR*	2.8935	0.59474	-0.198	-0.51302	1.10
8384	*LAMA3*	2.8537	0.54764	-0.1112	-0.83623	1.10
8385	*MX1*	3.542	1.3541	-1.606	-2.1936	1.10
8386	*LPPR1*	2.0624	0.74524	0.48176	-2.6289	1.10
8387	*GYPB*	2.1683	0.58529	0.53505	-2.0895	1.10
8388	*ARHGDIG*	2.2692	0.64483	0.37448	-1.9622	1.10
8389	*ZNF691*	2.3008	0.81226	0.17505	-0.32569	1.10
8390	*AACS*	1.7943	0.99063	0.50286	0.3576	1.10
8391	*SNAPC5*	3.4318	0.40936	-0.55388	-0.99608	1.10
8392	*ZNF865*	3.2778	0.044461	-0.03528	-0.17663	1.10
8393	*ACSL3*	2.7356	2.3703	-1.8191	-1.9673	1.10
8394	*ATG2B*	4.4666	1.1942	-2.374	-2.6275	1.10
8395	*UGT2B10*	3.5209	1.3507	-1.5848	-1.8503	1.10
8396	*BNC2*	3.6084	-0.09312	-0.2287	-1.1421	1.10
8397	*URGCP-MRPS24*	4.0681	-0.36649	-0.4155	-1.3211	1.10
8398	*DNAH9*	2.5677	1.9002	-1.1822	-1.5373	1.10
8399	*EPB41L4A*	3.6059	0.28812	-0.60845	-2.0437	1.10
8400	*MTMR4*	3.1975	0.090585	-0.00311	-1.7591	1.09
8401	*FGF11*	3.6011	0.18574	-0.50208	-0.80144	1.09
8402	*DHX30*	3.489	0.054696	-0.25904	-1.7874	1.09
8403	*GZMB*	2.6471	0.88031	-0.24337	-1.4764	1.09
8404	*LRRTM3*	4.3643	-0.45923	-0.62154	-1.9758	1.09
8405	*SRPK1*	3.4014	1.6978	-1.8157	-1.9055	1.09
8406	*RNF139*	2.4042	0.86236	0.01636	-0.88634	1.09
8407	*OR2M4*	3.2055	1.0354	-0.95816	-1.6802	1.09
8408	*LRRC32*	3.6642	0.033637	-0.41538	-0.67138	1.09
8409	*LHB*	4.4208	-0.46747	-0.6714	-1.2215	1.09
8410	*BTBD8*	4.7803	-0.31688	-1.1825	-1.2867	1.09
8411	*NCAPG2*	3.9756	0.66956	-1.3644	-2.1217	1.09

8412	*NPFFR2*	2.694	0.95737	-0.3713	-0.92879	1.09
8413	*RFPL2*	2.3007	0.66577	0.31308	-1.5267	1.09
8414	*PLCB2*	2.6388	1.1411	-0.50165	-1.5128	1.09
8415	*UBE2Q1*	3.6686	-0.13545	-0.25527	-1.7107	1.09
8416	*MARCKSL1*	4.521	-0.29114	-0.95321	-1.1605	1.09
8417	*SH2D5*	3.2501	0.12282	-0.09677	-0.81541	1.09
8418	*CA4*	2.3874	0.79764	0.09091	-1.445	1.09
8419	*ALAD*	5.3253	-0.97549	-1.0741	-1.6855	1.09
8420	*HINT3*	1.8647	1.7307	-0.32009	-1.2438	1.09
8421	*TAS1R3*	3.9004	0.71022	-1.3356	-1.5304	1.09
8422	*CDX4*	2.4931	0.80519	-0.02354	-0.9142	1.09
8423	*CSTL1*	2.0711	0.91461	0.28831	-1.177	1.09
8424	*NFKB2*	4.0923	0.48317	-1.3016	-1.4913	1.09
8425	*OTUB2*	4.2367	0.36231	-1.3255	-1.3292	1.09
8426	*TMEM182*	4.5511	-0.49564	-0.78229	-1.1034	1.09
8427	*TGFBRAP1*	3.8841	0.15223	-0.7635	-3.3669	1.09
8428	*MAPRE3*	2.3793	1.0336	-0.14009	-2.0383	1.09
8429	*SUSD1*	3.8763	0.30635	-0.9105	-1.124	1.09
8430	*BTBD1*	2.7528	1.6903	-1.1713	-1.5654	1.09
8431	*PFKP*	3.7162	-0.15293	-0.29241	-0.82172	1.09
8432	*LOC100996693*	3.7053	0.40039	-0.83542	-1.3148	1.09
8433	*CCT8L2*	2.9601	0.36641	-0.05663	-2.6313	1.09
8434	*AFM*	3.0674	0.38542	-0.18358	-1.2705	1.09
8435	*CHCHD3*	2.6967	0.35881	0.21342	-1.0095	1.09
8436	*NLRX1*	3.5929	-0.02502	-0.29995	-3.914	1.09
8437	*SEC16A*	4.6206	1.1774	-2.5302	-2.5386	1.09
8438	*CMTR1*	5.5319	-1.082	-1.1825	-1.9748	1.09
8439	*GABPA*	2.4799	1.1184	-0.3316	-1.0813	1.09
8440	*HSPA14*	2.5847	0.93943	-0.25748	-1.1743	1.09
8441	*LANCL1*	1.7789	1.7694	-0.2827	-1.8193	1.09
8442	*PIN4*	2.7067	0.70292	-0.14406	-0.36513	1.09
8443	*OPTN*	2.1864	1.1152	-0.03607	-1.6649	1.09
8444	*ERI3*	2.0971	0.84672	0.32159	-1.2744	1.09
8445	*ARHGAP4*	5.3019	-0.73219	-1.3043	-1.7561	1.09
8446	*AURKB*	3.4793	0.36922	-0.5834	-1.1846	1.09
8447	*MUC5B*	1.6911	1.1009	0.47288	-1.917	1.09
8448	*CLPSL1*	2.7713	1.7383	-1.2448	-1.3453	1.09
8449	*LRRC25*	4.0213	-0.11781	-0.63872	-1.4682	1.09
8450	*VAPA*	1.9004	1.4356	-0.07151	-1.2304	1.09
8451	*CSHL1*	3.1116	1.0759	-0.92339	-1.0328	1.09
8452	*C11orf85*	2.9778	0.1507	0.13561	-1.3343	1.09
8453	*AK5*	2.5991	0.35272	0.31206	-0.98361	1.09
8454	*FOLH1*	3.6581	0.85874	-1.2534	-3.0644	1.09
8455	*APOE*	4.0372	0.13598	-0.9101	-1.3937	1.09
8456	*TTC39C*	3.7856	1.1477	-1.6703	-2.1202	1.09
8457	*NAT8L*	3.2101	0.89551	-0.8428	-1.0031	1.09

8458	*WDR62*	2.8988	0.39617	-0.03222	-0.35836	1.09
8459	*COMMD5*	4.6063	-0.55291	-0.79083	-1.2684	1.09
8460	*FUBP3*	3.5687	0.70185	-1.0091	-2.1282	1.09
8461	*ADRB2*	2.0237	1.3848	-0.14749	-1.9912	1.09
8462	*CREBL2*	2.0882	0.88078	0.29118	-0.02978	1.09
8463	*CIC*	4.6436	-0.27145	-1.1122	-2.4084	1.09
8464	*ARHGAP12*	3.5396	-0.09878	-0.18133	-0.77284	1.09
8465	*DHX58*	3.7859	1.4353	-1.9622	-2.3466	1.09
8466	*C11orf63*	2.8335	0.56823	-0.14309	-0.59981	1.09
8467	*TNFRSF10C*	1.8882	0.87658	0.49376	-0.95469	1.09
8468	*ZNF596*	1.9873	0.65056	0.62056	-1.8484	1.09
8469	*RGN*	1.2559	1.1805	0.82133	0.083113	1.09
8470	*IGFBP6*	4.0746	1.0057	-1.8227	-1.9175	1.09
8471	*CSH1*	1.6572	1.2945	0.30552	-1.6434	1.09
8472	*TRIM58*	1.7912	1.197	0.26863	-1.4754	1.09
8473	*PTGR1*	3.5967	0.23339	-0.57379	-0.80771	1.09
8474	*ABCC1*	3.4252	-0.03882	-0.13022	-2.0953	1.09
8475	*PLD4*	2.6638	0.85239	-0.26009	-0.96962	1.09
8476	*SLC6A7*	3.6224	1.8723	-2.2388	-2.2947	1.09
8477	*ZNF706*	4.5172	-0.47107	-0.79079	-1.203	1.09
8478	*UBE2QL1*	2.8677	0.56312	-0.17591	-0.31116	1.08
8479	*DOCK6*	2.1448	0.99439	0.11544	-0.14132	1.08
8480	*KCMF1*	3.3568	0.23696	-0.33933	-2.1612	1.08
8481	*TRAF3IP2*	2.7539	1.674	-1.1742	-2.1541	1.08
8482	*ZBED5*	1.8108	1.1439	0.29893	-0.07475	1.08
8483	*TUBG2*	2.7433	2.022	-1.5118	-1.9559	1.08
8484	*OR4D2*	2.1108	0.9731	0.16938	-0.65924	1.08
8485	*C16orf86*	3.5253	0.44472	-0.71743	-1.5104	1.08
8486	*PRRC2A*	2.8046	1.2861	-0.83869	-1.0584	1.08
8487	*POU2F3*	1.1703	1.1657	0.91466	-1.0766	1.08
8488	*IFITM2*	1.7592	1.6501	-0.1591	-1.4902	1.08
8489	*DKKL1*	2.5207	1.036	-0.3068	-1.0576	1.08
8490	*GOLGA6L4*	3.3463	0.7069	-0.80351	-0.83753	1.08
8491	*RGS10*	3.7964	0.4308	-0.97768	-1.1148	1.08
8492	*MYL1*	2.3252	1.3159	-0.39171	-0.87846	1.08
8493	*KIF1A*	3.7576	-0.20239	-0.30609	-0.34036	1.08
8494	*TMEM263*	2.7964	0.48193	-0.02974	-2.4873	1.08
8495	*TFAP4*	3.1495	0.18027	-0.08293	-1.4061	1.08
8496	*TRIM52*	2.4485	0.55552	0.24278	-0.30208	1.08
8497	*RNF217*	2.3643	0.69777	0.18343	-0.30609	1.08
8498	*RP1L1*	2.5769	0.56242	0.10613	-2.1197	1.08
8499	*LCE1D*	1.5016	1.0236	0.71966	-0.79485	1.08
8500	*BICC1*	2.8176	0.50751	-0.08039	-0.10575	1.08
8501	*CYBB*	2.5542	1.0365	-0.34635	-0.44353	1.08
8502	*GPR37*	4.5043	-0.00828	-1.2517	-1.9558	1.08
8503	*EVL*	1.5501	1.468	0.22609	5.25E-05	1.08

8504	*ADIPOR2*	2.294	2.1555	-1.2056	-1.6998	1.08
8505	*S100A7A*	3.7907	-0.18381	-0.36311	-2.3876	1.08
8506	*DEFB106B*	2.7492	-0.58705			1.08
8507	*LRRC29*	2.2042	0.67494	0.36389	-0.57207	1.08
8508	*VHL*	5.1905	-0.64769	-1.3004	-2.2343	1.08
8509	*KLRG1*	2.9953	1.0231	-0.77602	-2.7566	1.08
8510	*SDHD*	4.2611	-0.32009	-0.69866	-1.2666	1.08
8511	*IFITM10*	2.8539	0.43059	-0.04339	-0.91192	1.08
8512	*FOXN1*	3.7385	-0.07348	-0.4247	-0.49968	1.08
8513	*KIF21A*	1.7241	1.6993	-0.18327	-0.64767	1.08
8514	*UHRF1BP1*	3.4727	-0.06034	-0.17238	-1.3994	1.08
8515	*OR1L8*	2.515	0.7349	-0.01	-1.3973	1.08
8516	*KIAA1462*	2.693	0.77875	-0.2325	-2.1541	1.08
8517	*QKI*	3.6399	0.091453	-0.49216	-0.9472	1.08
8518	*OR4F16*	3.3439	0.30728	-0.41239	-1.3694	1.08
8519	*MIA2*	3.0898	0.75887	-0.61017	-1.3409	1.08
8520	*SIK2*	3.2964	0.39038	-0.44832	-1.0923	1.08
8521	*C20orf173*	1.7069	1.442	0.08929	-1.124	1.08
8522	*MOGAT3*	3.829	-0.25941	-0.33152	-0.96661	1.08
8523	*EHF*	4.5766	-0.22726	-1.1114	-1.9673	1.08
8524	*AQP8*	2.342	0.82905	0.06658	-0.50731	1.08
8525	*OR13A1*	2.9438	1.0617	-0.76857	-1.5073	1.08
8526	*C19orf45*	2.1694	0.99891	0.06774	0.05242	1.08
8527	*RINT1*	3.5922	0.36232	-0.71905	-2.3389	1.08
8528	*TSPAN18*	3.8211	0.62581	-1.2125	-1.4685	1.08
8529	*NTN4*	4.1574	0.21938	-1.1426	-1.6525	1.08
8530	*C9orf171*	4.1326	-0.42378	-0.47485	-1.615	1.08
8531	*SPAG7*	2.0171	1.8722	-0.65553	-1.3769	1.08
8532	*TMEM17*	1.4447	1.2343	0.55401	-0.51931	1.08
8533	*TYMS*	2.3813	0.56035	0.29118	-2.0256	1.08
8534	*EIF2S1*	3.5989	1.3938	-1.7614	-2.0452	1.08
8535	*FILIP1*	4.5215	-0.45505	-0.83527	-0.9857	1.08
8536	*RPUSD4*	2.6709	0.38514	0.17508	-0.69006	1.08
8537	*IPO8*	2.7193	0.47026	0.04149	-1.2324	1.08
8538	*METTL14*	3.1621	0.72355	-0.65553	-1.6672	1.08
8539	*KIAA0922*	3.0575	0.29641	-0.12408	-0.8083	1.08
8540	*KRTAP20-1*	3.06	2.2243	-2.0545	-2.0834	1.08
8541	*TFCP2*	2.1001	1.9748	-0.84529	-1.3465	1.08
8542	*ESRRA*	3.8302	0.47086	-1.0716	-1.6286	1.08
8543	*SMAP1*	2.5478	0.57218	0.10941	-1.8692	1.08
8544	*ZSWIM8*	4.1707	0.11251	-1.054	-1.626	1.08
8545	*DDN*	4.4411	0.048425	-1.2608	-1.3098	1.08
8546	*OR2J3*	2.3266	1.2261	-0.3247	-0.3658	1.08
8547	*STK24*	3.5753	0.39078	-0.73853	-1.6289	1.08
8548	*B3GAT1*	2.8552	0.77193	-0.39998	-0.85467	1.08
8549	*SPHKAP*	4.575	-0.65035	-0.69815	-1.1031	1.08

8550	*SETDB1*	3.3853	-0.04486	-0.11452	-1.3842	1.08
8551	*APLP2*	4.2916	0.05102	-1.1169	-2.3602	1.08
8552	*CTBS*	1.6141	1.0557	0.55553	-2.2432	1.08
8553	*PSG2*	2.4485	0.68003	0.09665	-0.91236	1.08
8554	*FAM181A*	1.2611	1.1849	0.77887	-0.42064	1.07
8555	*PGLYRP4*	1.5606	1.3536	0.31054	-1.6368	1.07
8556	*TNNT2*	2.8666	0.80045	-0.44353	-0.71077	1.07
8557	*ZIK1*	4.8723	-0.61338	-1.0355	-1.2172	1.07
8558	*VIPR2*	3.6118	-0.04513	-0.34327	-0.87109	1.07
8559	*GDA*	2.8969	0.58537	-0.2594	-1.1919	1.07
8560	*CBR3*	2.7194	0.65655	-0.15337	-1.8366	1.07
8561	*LY6H*	2.7864	0.93864	-0.50265	-2.5707	1.07
8562	*FANCA*	2.5217	1.2197	-0.51912	-1.0032	1.07
8563	*RNF215*	2.8483	0.65567	-0.28176	-0.30739	1.07
8564	*HCFC1*	4.0138	0.44884	-1.2411	-2.1239	1.07
8565	*TAS2R30*	2.4913	0.80445	-0.07427	-0.4221	1.07
8566	*SMG1*	5.2505	-0.91779	-1.1114	-1.9984	1.07
8567	*KCNIP1*	4.2138	-0.44353	-0.55158	-0.69754	1.07
8568	*PTPN11*	4.1984	-0.13539	-0.84523	-2.9646	1.07
8569	*PANK4*	4.3019	0.47862	-1.5635	-2.6289	1.07
8570	*RBM10*	2.6428	0.80141	-0.2283	-1.2574	1.07
8571	*TCERG1L*	3.7968	-0.13429	-0.44661	-1.0576	1.07
8572	*TFF3*	2.4234	0.42358	0.36833	-1.3117	1.07
8573	*TAF15*	2.782	0.61845	-0.18662	-1.3711	1.07
8574	*COCH*	2.1425	0.72059	0.3488	-0.3351	1.07
8575	*COL24A1*	4.5225	-0.0304	-1.2815	-1.3583	1.07
8576	*CCT3*	2.8823	0.26025	0.06783	-2.8051	1.07
8577	*MYLIP*	1.5725	1.3056	0.33119	-2.0107	1.07
8578	*FLVCR2*	2.0363	0.97658	0.19565	-1.7886	1.07
8579	*BDKRB2*	2.3071	0.87275	0.02849	-1.1094	1.07
8580	*EVA1A*	2.5198	0.91317	-0.22469	-1.348	1.07
8581	*NCKAP1L*	3.6328	0.12276	-0.54818	-0.59823	1.07
8582	*ZNF496*	3.6612	0.16511	-0.61946	-0.92629	1.07
8583	*PGM2L1*	2.6643	0.58168	-0.03924	-1.2024	1.07
8584	*USP13*	2.5264	0.42153	0.25877	-1.1331	1.07
8585	*FGFBP1*	2.0747	1.0155	0.11535	0.059357	1.07
8586	*SMCP*	3.2888	0.93563	-1.0191	-2.0304	1.07
8587	*KIF22*	2.7623	0.75738	-0.31475	-2.0301	1.07
8588	*ITGB1BP1*	3.7988	-0.2873	-0.30677	-0.81217	1.07
8589	*SLAMF1*	4.2201	-0.45928	-0.55655	-0.94777	1.07
8590	*DTX3*	2.5911	1.1776	-0.56586	-2.6376	1.07
8591	*ITGB1BP2*	1.8341	1.6752	-0.30723	-0.59716	1.07
8592	*BCL6B*	2.2903	2.0172	-1.1063	-2.2154	1.07
8593	*OR4F4*	2.5013	1.1856	-0.48575	-1.4415	1.07
8594	*ECE1*	1.2599	1.2455	0.6956	-0.17111	1.07
8595	*XRCC3*	5.7698	-1.2027	-1.3662	-1.6007	1.07

8596	*GLT8D1*	4.0124	-0.34411	-0.46748	-1.4206	1.07
8597	*SLC12A3*	3.7463	0.22275	-0.76882	-1.743	1.07
8598	*GRAMD1B*	3.6061	0.3645	-0.77042	-1.5884	1.07
8599	*RHBDD2*	3.3444	-0.00492	-0.13962	-0.77848	1.07
8600	*EIF4A2*	1.3049	1.0362	0.85862	0.30154	1.07
8601	*MGLL*	2.5471	0.64819	0.00396	-0.5183	1.07
8602	*LFNG*	2.0993	2.0167	-0.91701	-1.5608	1.07
8603	*C9orf57*	3.3284	0.9896	-1.1193	-2.3496	1.07
8604	*MBD6*	3.9239	0.078829	-0.80442	-1.1212	1.07
8605	*IFIT2*	3.0542	0.25827	-0.11468	-0.82826	1.07
8606	*PIH1D2*	1.4906	1.1841	0.52293	-0.70976	1.07
8607	*ANKRD18B*	2.0043	0.71805	0.47389	-0.73143	1.07
8608	*ZNF311*	2.8116	1.6121	-1.2277	-1.371	1.07
8609	*LAMA4*	3.4608	0.56332	-0.82833	-1.6218	1.07
8610	*HVCN1*	3.4203	0.77017	-0.99474	-1.2915	1.07
8611	*TRIM33*	2.2064	0.88068	0.10822	-1.9008	1.07
8612	*BEND5*	2.9842	0.5894	-0.38028	-2.9834	1.06
8613	*GABRA4*	1.5655	0.88248	0.74438	-0.53661	1.06
8614	*TCAP*	1.9671	1.7344	-0.50935	-1.5149	1.06
8615	*DACH1*	2.2152	0.50053	0.47612	-0.42226	1.06
8616	*TSC22D2*	1.8617	0.82981	0.50025	-0.73787	1.06
8617	*MYBPC2*	2.6062	1.5082	-0.92384	-2.3577	1.06
8618	*SYT4*	5.3551	-0.55815	-1.6065	-1.6208	1.06
8619	*LRIG2*	3.2571	-0.02341	-0.04332	-0.33752	1.06
8620	*C1GALT1*	1.6215	1.0507	0.51733	-0.67702	1.06
8621	*BNIP2*	1.8878	1.6447	-0.34315	-0.61017	1.06
8622	*RDH5*	2.2649	1.0575	-0.13381	-0.13622	1.06
8623	*ZNF595*	5.1107	-0.72038	-1.202	-1.5082	1.06
8624	*FZD5*	4.4162	-0.15551	-1.0726	-1.1053	1.06
8625	*CDK16*	2.8619	0.65057	-0.32567	-1.5246	1.06
8626	*HLA-DQB1*	3.357	0.24134	-0.41207	-1.1461	1.06
8627	*CT47A3*	2.2023	0.9802	0.00363	-0.04968	1.06
8628	*EXOSC7*	2.3206	0.62291	0.24242	-1.6469	1.06
8629	*RSC1A1*	2.0603	0.76148	0.36413	-0.6951	1.06
8630	*GZMM*	2.3464	1.6831	-0.84375	-1.8711	1.06
8631	*PNCK*	2.1835	0.6876	0.31419	-0.63976	1.06
8632	*BBS7*	2.5556	1.6221	-0.9925	-1.0752	1.06
8633	*TMEM110*	4.622	-0.47696	-0.9599	-1.4325	1.06
8634	*CYP3A7-CYP3A51P*	2.0912	0.60261	0.49122	-1.8741	1.06
8635	*ACOT13*	2.6962	0.68499	-0.19626	-2.2866	1.06
8636	*AIM1L*	3.7438	-0.16884	-0.39081	-0.68889	1.06
8637	*NAP1L5*	2.0351	1.6712	-0.52255	-1.5952	1.06
8638	*SLC22A23*	3.2374	0.12678	-0.1808	-2.2432	1.06
8639	*IL12B*	3.8952	-0.13563	-0.5763	-0.66034	1.06
8640	*JSRP1*	3.1471	0.55041	-0.51464	-1.5744	1.06
8641	*SPATA6L*	1.537	1.1245	0.52057	-0.30091	1.06

8642	*CRTAP*	3.1867	1.028	-1.0347	-1.1053	1.06
8643	*MGAM2*	4.8801	-0.45972	-1.2411	-1.3591	1.06
8644	*LCE1A*	2.125	0.81979	0.23375	-0.25241	1.06
8645	*AP1AR*	2.6837	0.7659	-0.27146	-1.4684	1.06
8646	*FAM160A2*	4.4538	0.088577	-1.3643	-1.4306	1.06
8647	*TPRG1L*	3.729	-0.14256	-0.40933	-2.0791	1.06
8648	*MAD2L2*	2.7523	1.6317	-1.2071	-1.7766	1.06
8649	*TOPORS*	2.3819	0.79669	-0.00314	-1.3194	1.06
8650	*UROD*	3.1996	0.36129	-0.38553	-0.95842	1.06
8651	*TMEM132C*	2.9059	0.13862	0.13058	-0.13916	1.06
8652	*PRKDC*	3.1158	1.3178	-1.2586	-1.4272	1.06
8653	*RGS21*	3.9945	-0.14795	-0.67235	-1.4204	1.06
8654	*ZNF232*	2.8618	0.75131	-0.43946	-3.8314	1.06
8655	*TMEM69*	1.6311	1.3467	0.1957	-2.9818	1.06
8656	*SPPL2C*	3.6399	0.50741	-0.97433	-1.3577	1.06
8657	*LRRC55*	2.3598	0.75405	0.05894	-0.27755	1.06
8658	*CRLF1*	2.5485	0.41343	0.21086	-2.1143	1.06
8659	*DDIT4L*	1.8655	1.2163	0.09098	-1.124	1.06
8660	*C8A*	2.0817	0.75214	0.33838	-0.36391	1.06
8661	*TPBGL*	1.7907	1.0612	0.31678	-1.7807	1.06
8662	*FOXP4*	1.983	0.76584	0.41968	-1.0888	1.06
8663	*CRYBA1*	3.1456	0.21358	-0.19096	-0.4735	1.06
8664	*C11orf45*	2.0417	0.80629	0.31999	-0.38559	1.06
8665	*HIST1H4C*	3.9345	0.58153	-1.3481	-2.7991	1.06
8666	*IL25*	3.6579	0.40039	-0.89129	-2.4328	1.06
8667	*DHRS1*	2.6399	0.26577	0.2606	-1.9326	1.06
8668	*RMND1*	2.0123	1.2717	-0.11773	-1.7317	1.06
8669	*FCRL1*	2.1643	0.73466	0.2672	-0.98292	1.06
8670	*KCNH8*	2.4032	1.5206	-0.75796	-1.3668	1.06
8671	*POU1F1*	3.6929	-0.10911	-0.4187	-1.0092	1.06
8672	*DGKG*	2.4108	0.52995	0.22384	-3.1836	1.05
8673	*FGF23*	3.6964	-0.0886	-0.44353	-1.7888	1.05
8674	*PRSS48*	4.87	-0.14289	-1.5635	-2.1342	1.05
8675	*ISLR*	2.4248	1.6983	-0.95964	-1.0802	1.05
8676	*COL25A1*	3.2168	0.31543	-0.36917	-1.3127	1.05
8677	*TPP2*	4.6058	-0.22943	-1.2141	-1.2277	1.05
8678	*MYBL1*	4.2591	0.14094	-1.2383	-2.0337	1.05
8679	*AICDA*	4.4909	-0.56598	-0.76495	-1.2867	1.05
8680	*MAF1*	4.079	0.51745	-1.4373	-1.4996	1.05
8681	*NFKBIA*	2.3463	1.7399	-0.92823	-1.6649	1.05
8682	*CD37*	2.8448	1.257	-0.94399	-1.8478	1.05
8683	*PARP15*	4.6417	-0.1549	-1.329	-1.7466	1.05
8684	*NKAIN2*	2.1127	1.2731	-0.22803	-0.31244	1.05
8685	*FLNA*	3.5534	0.30172	-0.69831	-1.2783	1.05
8686	*TMEM232*	1.292	1.0049	0.85962	-1.2815	1.05
8687	*RNF212B*	3.9876	0.3541	-1.1858	-2.0351	1.05

8688	*DES*	3.2008	0.22548	-0.2715	-1.094	1.05
8689	*RPLP1*	5.39	-0.40121	-1.8341	-2.2771	1.05
8690	*ALYREF*	2.611	0.53812	0.00556	-0.71123	1.05
8691	*P2RX1*	3.5285	-0.01633	-0.35899	-2.3534	1.05
8692	*ATP1B3*	3.0477	1.1877	-1.0824	-1.872	1.05
8693	*ENHO*	2.4853	0.93921	-0.27198	-1.1547	1.05
8694	*KLHL32*	2.8707	1.2006	-0.91901	-1.4051	1.05
8695	*KMT2E*	3.5755	0.025902	-0.44951	-1.1957	1.05
8696	*HERC3*	2.0936	1.3714	-0.31342	-1.7695	1.05
8697	*LCTL*	3.1054	0.50263	-0.45663	-0.67253	1.05
8698	*HES6*	3.1967	0.67976	-0.72585	-1.7323	1.05
8699	*PAGE5*	3.5647	-0.03832	-0.37612	-2.7442	1.05
8700	*LEFTY1*	4.4138	-0.49423	-0.76966	-1.6801	1.05
8701	*CENPN*	2.8895	1.2143	-0.95423	-1.5022	1.05
8702	*PARD6G*	3.61	0.13046	-0.59168	-2.0483	1.05
8703	*DENND2C*	2.7668	1.5577	-1.1759	-1.8248	1.05
8704	*CGREF1*	2.7834	0.42847	-0.06333	-1.9237	1.05
8705	*LDHAL6B*	3.0311	0.36086	-0.24387	-0.41139	1.05
8706	*PPP1R21*	4.2507	-0.35718	-0.74566	-1.9435	1.05
8707	*SRPK2*	2.5989	0.6408	-0.0923	-1.3184	1.05
8708	*HSPB11*	1.9607	1.1581	0.02802	-1.6999	1.05
8709	*OR11H2*	3.3269	0.2962	-0.47636	-1.0904	1.05
8710	*FBXO4*	4.5074	0.29113	-1.653	-2.4022	1.05
8711	*SCAPER*	1.6281	1.2432	0.27398	-2.0181	1.05
8712	*DLX3*	2.164	0.98874	-0.0083	-1.2301	1.05
8713	*ACVR2B*	1.6074	1.5151	0.02187	-0.56681	1.05
8714	*FLII*	3.6638	-0.2283	-0.29114	-0.96196	1.05
8715	*ETFA*	1.5309	1.332	0.28141	-0.04288	1.05
8716	*CD8A*	2.0656	2.0511	-0.97357	-1.7316	1.05
8717	*CPLX4*	3.4112	0.50376	-0.77255	-1.6527	1.05
8718	*RHBG*	2.2283	0.64162	0.27221	-1.0386	1.05
8719	*ZMYND15*	2.3134	1.5568	-0.72825	-1.4536	1.05
8720	*MAP7D2*	2.4689	1.287	-0.61436	-2.9538	1.05
8721	*SCGB1D4*	4.8442	-0.4125	-1.2902	-1.3556	1.05
8722	*LDLRAD2*	3.2397	0.23598	-0.33426	-2.3036	1.05
8723	*AHSG*	3.7585	1.1776	-1.7947	-2.537	1.05
8724	*ZFP37*	4.238	0.15417	-1.251	-2.2811	1.05
8725	*MOCS3*	2.5197	1.4166	-0.79558	-2.7689	1.05
8726	*PCDH11Y*	1.5185	1.2408	0.38129	-0.77095	1.05
8727	*PRAMEF1*	2.0881	1.2339	-0.18195	-0.46586	1.05
8728	*CFTR*	2.4426	0.83229	-0.13487	-1.1385	1.05
8729	*LEMD3*	2.4204	0.54994	0.16959	-0.0014	1.05
8730	*COLEC10*	2.3121	1.1219	-0.29418	-1.5182	1.05
8731	*SYNCRIP*	3.2817	0.94319	-1.0863	-2.4221	1.05
8732	*TPX2*	2.9224	1.189	-0.97381	-1.7782	1.05
8733	*CLDN1*	2.1067	0.73695	0.29391	-0.31505	1.05

8734	*GK2*	1.7536	0.95032	0.43341	-1.4154	1.05
8735	*UPP2*	2.0413	0.84243	0.25253	-0.05667	1.05
8736	*CWC25*	5.1005	-0.7409	-1.2234	-2.1938	1.05
8737	*SLC9A2*	3.6564	-0.1386	-0.38361	-2.0537	1.04
8738	*XKR9*	2.8198	0.87394	-0.5603	-1.0529	1.04
8739	*LRMP*	2.582	0.49105	0.06014	-0.44353	1.04
8740	*CHKB*	3.2547	0.59723	-0.71893	-2.7849	1.04
8741	*RHPN1*	1.7063	1.2334	0.19304	-0.87335	1.04
8742	*NUPL2*	3.1274	0.45962	-0.45531	-1.5393	1.04
8743	*LRRN2*	3.0409	0.12142	-0.03097	-1.9673	1.04
8744	*DIS3L*	3.7631	-0.1865	-0.44774	-1.94	1.04
8745	*SRD5A1*	2.7562	0.57985	-0.20743	-0.94881	1.04
8746	*MDM4*	3.0483	0.62398	-0.54373	-2.1342	1.04
8747	*TMEM198*	1.4055	0.96502	0.75622	-2.3235	1.04
8748	*WDR13*	1.4323	0.95746	0.73692	-1.7646	1.04
8749	*EIF3H*	2.202	1.2851	-0.36106	-1.1322	1.04
8750	*PRUNE*	4.0432	0.35324	-1.2705	-1.2837	1.04
8751	*PIPOX*	2.0195	0.74601	0.3593	-0.5183	1.04
8752	*FLYWCH1*	2.395	0.69891	0.03066	-1.2174	1.04
8753	*SCP2D1*	2.1131	1.9447	-0.93329	-1.5125	1.04
8754	*FES*	1.0551	1.054	1.0152	-0.03588	1.04
8755	*SPINK9*	4.6993	-0.17772	-1.3982	-1.4143	1.04
8756	*NAA38*	3.5771	0.37382	-0.82796	-1.1726	1.04
8757	*TAF3*	1.3517	1.1841	0.58686	-1.697	1.04
8758	*ABHD13*	3.9202	-0.01403	-0.78364	-2.3248	1.04
8759	*NTRK1*	2.7724	0.20593	0.14415	-0.07288	1.04
8760	*OR8B3*	3.2511	0.027145	-0.15596	-0.75786	1.04
8761	*SHISA4*	3.1747	0.2658	-0.31838	-1.4373	1.04
8762	*GZMA*	2.1887	1.4523	-0.51929	-1.8227	1.04
8763	*ZNF630*	2.196	1.4711	-0.54544	-1.0355	1.04
8764	*PLA1A*	3.446	0.022499	-0.34751	-0.46757	1.04
8765	*PRRC2B*	1.4581	1.2847	0.37817	-0.35462	1.04
8766	*NIPA2*	3.1933	1.1014	-1.1739	-1.505	1.04
8767	*SCNN1B*	4.1925	-0.47206	-0.60003	-0.93865	1.04
8768	*CCNA1*	3.2372	0.71119	-0.82805	-0.95629	1.04
8769	*GORAB*	2.2595	1.6211	-0.76033	-1.4684	1.04
8770	*FNDC3A*	1.8392	1.7221	-0.44134	-1.0918	1.04
8771	*SNRNP70*	3.4318	0.060946	-0.37317	-0.83145	1.04
8772	*CXCL14*	2.6834	0.50977	-0.07541	-0.30078	1.04
8773	*ACOT11*	3.0281	0.46482	-0.37592	-1.5631	1.04
8774	*TNNT1*	1.7759	1.6155	-0.275	-1.359	1.04
8775	*CCDC42*	2.0238	0.8693	0.22311	-0.03866	1.04
8776	*MSANTD4*	2.8492	0.20352	0.06291	-0.25317	1.04
8777	*F10*	4.7354	-0.41681	-1.2032	-2.4394	1.04
8778	*CARNS1*	2.7162	0.9243	-0.52521	-0.73938	1.04
8779	*LOC100130357*	3.9351	-0.03767	-0.78229	-0.85389	1.04

8780	*STMN4*	1.3491	1.0022	0.76255	-0.1307	1.04
8781	*SLC39A14*	1.1151	1.0863	0.912	0.46625	1.04
8782	*FABP4*	2.1463	1.8159	-0.85027	-2.203	1.04
8783	*ZNF256*	2.9758	0.57062	-0.43507	-0.81509	1.04
8784	*FAM118B*	3.9077	-0.23523	-0.56145	-1.3762	1.04
8785	*SLC25A42*	2.2312	1.1566	-0.27844	-1.7232	1.04
8786	*FOXF2*	2.2831	2.1284	-1.3031	-1.5658	1.04
8787	*STK19*	2.6487	0.36866	0.08979	-0.89741	1.04
8788	*MGAM*	4.3202	-0.33104	-0.88213	-1.0385	1.04
8789	*MTHFS*	1.7933	0.99901	0.31424	-2.2844	1.04
8790	*WDR92*	2.1184	0.71004	0.27765	-1.0444	1.04
8791	*CDC42BPA*	4.012	1.0695	-1.9758	-2.0258	1.04
8792	*TTC8*	3.1253	1.3738	-1.3935	-1.4405	1.04
8793	*TMEM209*	3.7855	-0.30094	-0.37913	-1.707	1.04
8794	*C19orf26*	4.1717	-0.30777	-0.75876	-0.84858	1.04
8795	*C9orf91*	2.6478	0.96477	-0.50793	-1.5171	1.03
8796	*KLK4*	3.3485	0.48942	-0.73333	-1.8366	1.03
8797	*VSX2*	2.6519	2.268	-1.8157	-2.5198	1.03
8798	*RADIL*	3.829	-0.33788	-0.38754	-1.7505	1.03
8799	*NLRP1*	3.7585	-0.01099	-0.64496	-2.4331	1.03
8800	*HDHD2*	3.0912	0.21428	-0.20338	-1.1685	1.03
8801	*SAMD11*	2.3689	0.51562	0.21727	-1.2936	1.03
8802	*RXRB*	3.7913	0.82957	-1.5194	-1.8929	1.03
8803	*PTPRR*	3.5207	0.58984	-1.0092	-1.3416	1.03
8804	*NQO1*	3.1386	0.10416	-0.14252	-0.52034	1.03
8805	*WBP1L*	4.2137	-0.28391	-0.82989	-1.696	1.03
8806	*ABI1*	2.6616	0.52823	-0.09238	-1.1444	1.03
8807	*TMEM190*	2.31	1.817	-1.0297	-1.146	1.03
8808	*HSF5*	2.69	0.82961	-0.42248	-0.63826	1.03
8809	*TUBD1*	3.0061	1.0594	-0.96938	-1.1742	1.03
8810	*C10orf131*	3.0051	0.10931	-0.01845	-2.3154	1.03
8811	*MINK1*	3.5458	1.2422	-1.6926	-2.6973	1.03
8812	*DGCR6*	2.1939	0.63881	0.26193	-0.60095	1.03
8813	*HPCAL1*	2.171	1.1589	-0.23607	-0.58929	1.03
8814	*ASIC5*	2.2407	1.0165	-0.16343	-2.0895	1.03
8815	*SNRPB2*	2.0766	1.0578	-0.04085	-0.91307	1.03
8816	*LRRK1*	2.4223	0.85084	-0.17986	-0.30525	1.03
8817	*FBXO8*	4.4808	-0.61358	-0.77462	-0.81531	1.03
8818	*CACNA1E*	1.8765	0.98304	0.23292	-0.72142	1.03
8819	*RPE65*	4.2183	-0.17129	-0.95461	-1.7591	1.03
8820	*RCAN2*	2.1611	0.82695	0.10416	0.10106	1.03
8821	*PPAP2B*	1.1869	1.1027	0.8022	-1.5997	1.03
8822	*CSMD3*	3.2009	0.26809	-0.378	-0.402	1.03
8823	*WDHD1*	5.17	-0.63348	-1.4467	-2.2311	1.03
8824	*C1GALT1C1*	2.3758	0.44439	0.26891	-1.9936	1.03
8825	*POP1*	2.8908	0.53063	-0.33357	-1.2104	1.03

8826	*BOLL*	1.6322	1.1975	0.2579	-0.55387	1.03
8827	*ITPK1*	2.2417	1.32	-0.47438	-1.6003	1.03
8828	*INSM1*	2.362	1.329	-0.60439	-2.4858	1.03
8829	*TMEM44*	2.6809	0.29032	0.11441	-0.27925	1.03
8830	*CYP1A1*	4.7297	-0.41447	-1.2301	-1.3686	1.03
8831	*DTHD1*	2.6999	0.9226	-0.5377	-1.0212	1.03
8832	*UVRAG*	3.4536	0.2124	-0.58173	-0.65924	1.03
8833	*CCDC173*	2.4036	1.358	-0.67764	-2.6485	1.03
8834	*OR52E6*	3.0881	0.48987	-0.49483	-0.80442	1.03
8835	*IPP*	3.9672	-0.44133	-0.44353	-1.2273	1.03
8836	*ENPP1*	3.4065	0.06324	-0.38767	-1.9988	1.03
8837	*RPTN*	2.8728	0.14678	0.06247	-0.39944	1.03
8838	*TMEM177*	1.5782	1.3279	0.17503	-0.34964	1.03
8839	*OR10G8*	4.1191	-0.11939	-0.91886	-2.9198	1.03
8840	*PSMD4*	2.398	0.3618	0.32041	-2.1068	1.03
8841	*CHST5*	1.6245	1.2076	0.24723	-0.2765	1.03
8842	*HINT1*	1.4037	1.1895	0.48548	0.15563	1.03
8843	*SMN1*	3.3708	-0.07288	-0.22006	-1.8049	1.03
8844	*PPP6R3*	3.2713	1.5751	-1.7688	-1.9341	1.03
8845	*SPTB*	4.9707	-0.43077	-1.4625	-2.2844	1.03
8846	*SSMEM1*	3.5251	0.019024	-0.4674	-1.474	1.03
8847	*CCL2*	2.6802	0.5365	-0.14068	-0.91357	1.03
8848	*FAM156A*	4.0595	-0.25875	-0.72612	-0.86267	1.02
8849	*PAPOLB*	1.8835	0.74429	0.44594	-1.2219	1.02
8850	*EGLN2*	4.1999	-0.44572	-0.68122	-1.4469	1.02
8851	*CASP5*	2.03	1.0387	0.00408	-0.48021	1.02
8852	*FAT1*	2.8801	0.41169	-0.22081	-0.77217	1.02
8853	*FXYD3*	3.7391	-0.26798	-0.40118	-1.9326	1.02
8854	*SGCE*	1.5635	1.5385	-0.03257	-0.84542	1.02
8855	*CRELD1*	1.75	0.80329	0.51607	-1.3599	1.02
8856	*TBC1D31*	3.5936	-0.04968	-0.47499	-0.60054	1.02
8857	*AGBL4*	2.4898	0.53176	0.04723	-1.8707	1.02
8858	*NPL*	3.5289	-0.16814	-0.29292	-1.0529	1.02
8859	*SLC22A14*	3.1235	0.93401	-0.99037	-1.2906	1.02
8860	*IFNL2*	3.3238	1.204	-1.4607	-2.1018	1.02
8861	*ADGRA3*	3.9895	0.08603	-1.0091	-1.6559	1.02
8862	*C19orf80*	3.4111	0.78316	-1.1282	-1.8076	1.02
8863	*TMEM243*	2.4403	0.49111	0.13447	-2.3804	1.02
8864	*PSG5*	4.4827	-0.51845	-0.89866	-2.0267	1.02
8865	*PHYH*	2.31	0.69252	0.06301	-0.78229	1.02
8866	*ANKRD20A4*	1.599	1.092	0.37379	-0.37704	1.02
8867	*MMADHC*	3.5018	0.61867	-1.0561	-2.6973	1.02
8868	*PARL*	3.4043	0.50725	-0.8475	-1.6907	1.02
8869	*RND3*	2.8164	0.38155	-0.13436	-0.69128	1.02
8870	*FZD10*	3.6345	0.21342	-0.78444	-0.87673	1.02
8871	*SPOCK1*	3.0177	0.50672	-0.46155	-0.95933	1.02

8872	*ANGPTL7*	3.7351	0.18707	-0.85943	-1.4567	1.02
8873	*NRM*	3.3255	1.0421	-1.305	-1.4299	1.02
8874	*AFTPH*	3.4413	0.81109	-1.1898	-1.536	1.02
8875	*GDAP1L1*	2.8394	0.67439	-0.45191	-1.0265	1.02
8876	*BCAR3*	2.3891	0.39999	0.27273	-0.24269	1.02
8877	*XAGE3*	3.934	0.66498	-1.5375	-1.6586	1.02
8878	*UNC119B*	2.7795	0.35184	-0.0702	-0.27672	1.02
8879	*CLDN16*	3.9877	-0.14198	-0.78472	-1.6524	1.02
8880	*BOC*	2.1746	0.52169	0.36413	-0.76361	1.02
8881	*ZBED9*	3.4089	0.53353	-0.88203	-1.8642	1.02
8882	*AIRE*	4.4743	-0.3576	-1.0564	-1.4151	1.02
8883	*GLIPR1*	3.8823	-0.06824	-0.75408	-1.1617	1.02
8884	*SNCG*	5.0882	-0.06618	-1.9622	-2.1947	1.02
8885	*RAB17*	3.0599	0.19327	-0.19354	-0.84426	1.02
8886	*AQP7*	2.7136	0.50617	-0.16038	-0.95527	1.02
8887	*ACTN2*	3.0796	0.53281	-0.55311	-1.6847	1.02
8888	*CHUK*	3.0686	0.016271	-0.02658	-0.40738	1.02
8889	*STAMBP*	3.1971	0.7294	-0.86821	-2.4472	1.02
8890	*OR4Q3*	3.3657	0.71832	-1.0267	-1.8077	1.02
8891	*FAM173B*	5.9432	-0.92427	-1.962	-2.0851	1.02
8892	*GUCA1C*	4.1886	0.28749	-1.4197	-1.5781	1.02
8893	*MED31*	2.315	0.64847	0.09277	-2.1287	1.02
8894	*SLURP1*	2.1736	1.4296	-0.54708	-1.0481	1.02
8895	*ZNF235*	4.1989	0.3057	-1.4485	-1.8725	1.02
8896	*TMOD3*	1.9611	1.0137	0.07872	-2.6635	1.02
8897	*SLC40A1*	3.7128	0.88608	-1.5454	-2.2276	1.02
8898	*PPBP*	2.5422	1.1447	-0.63357	-1.4465	1.02
8899	*IGFLR1*	4.6865	-0.70391	-0.92956	-1.344	1.02
8900	*OR2A12*	3.4939	0.57693	-1.0178	-1.4935	1.02
8901	*LENG1*	3.6812	0.11798	-0.74815	-1.035	1.02
8902	*DBT*	2.1953	1.4108	-0.55531	-1.1706	1.02
8903	*XKR3*	4.1088	-0.34014	-0.71819	-1.9619	1.02
8904	*C8orf48*	3.461	0.46602	-0.87694	-1.0104	1.02
8905	*KCTD2*	2.3475	0.3669	0.33545	-1.4487	1.02
8906	*INHA*	2.5353	0.72132	-0.20681	-1.8909	1.02
8907	*NMU*	4.4242	-0.01876	-1.3559	-1.9168	1.02
8908	*NLRP4*	3.1711	0.282	-0.40374	-1.5462	1.02
8909	*HSD17B12*	3.5036	0.13485	-0.59008	-1.9705	1.02
8910	*IL17F*	2.0317	1.1631	-0.14649	-1.4359	1.02
8911	*GPRC6A*	1.064	0.99401	0.98991	0.60444	1.02
8912	*MIA*	3.6188	0.2269	-0.79798	-1.4364	1.02
8913	*ADRA2B*	3.9441	-0.07649	-0.82	-1.7553	1.02
8914	*MRPS27*	4.9951	-0.74815	-1.1998	-1.4299	1.02
8915	*CLPS*	4.0913	-0.02692	-1.0175	-1.479	1.02
8916	*MTRNR2L5*	1.9642	0.74969	0.33271	-1.4364	1.02
8917	*CYTH4*	1.693	0.83303	0.51984	-1.3783	1.02

8918	*DTD1*	1.3342	1.2033	0.50771	-1.2867	1.02
8919	*SYCP2*	2.1411	0.80418	0.09972	-0.60841	1.02
8920	*ACTN1*	3.6628	1.1692	-1.7874	-2.2277	1.01
8921	*IL5*	3.7613	0.1111	-0.82811	-1.505	1.01
8922	*MICALL1*	2.1181	0.48969	0.43614	-0.77185	1.01
8923	*KHSRP*	6.0156	-1.0718	-1.8999	-2.0837	1.01
8924	*USP17L30*	2.22	1.3373	-0.51363	-0.55492	1.01
8925	*AATK*	3.3181	0.53611	-0.81072	-2.1111	1.01
8926	*DCAF12*	2.8984	0.54977	-0.40642	-1.0884	1.01
8927	*OR2A5*	2.5323	0.94018	-0.43077	-1.8478	1.01
8928	*COL1A2*	3.0392	0.17599	-0.17403	-0.30038	1.01
8929	*RASSF8*	3.1683	0.89685	-1.0243	-1.6463	1.01
8930	*B3GALT5*	2.9836	0.12081	-0.06364	-2.5386	1.01
8931	*NARR*	4.0094	-0.00439	-0.96471	-1.3209	1.01
8932	*CYP2C8*	4.8738	-0.39389	-1.4402	-2.0303	1.01
8933	*CRHR1*	4.3539	-0.63826	-0.6765	-0.72326	1.01
8934	*CRB2*	2.9179	1.0612	-0.94027	-1.3266	1.01
8935	*NXPH4*	4.4128	-0.10845	-1.2665	-2.2692	1.01
8936	*NDUFS6*	2.3808	1.5575	-0.90154	-1.5984	1.01
8937	*REM2*	4.0353	-0.13096	-0.86793	-2.4784	1.01
8938	*FAM186A*	2.4863	0.81532	-0.26617	-1.1289	1.01
8939	*PPIP5K1*	1.975	0.75422	0.30583	-0.61852	1.01
8940	*FABP12*	2.4867	0.33905	0.20899	-2.5334	1.01
8941	*RPGR*	3.5203	0.44492	-0.93112	-1.2086	1.01
8942	*CT47A11*	3.1799	0.78283	-0.92911	-1.9446	1.01
8943	*AP2A1*	1.5899	1.0944	0.34843	0.12788	1.01
8944	*VPS51*	3.5352	0.63571	-1.1385	-1.407	1.01
8945	*BIN2*	3.5072	0.72975	-1.2051	-1.4141	1.01
8946	*MAMSTR*	4.2981	0.008409	-1.2753	-1.9513	1.01
8947	*ZPLD1*	2.1163	1.0715	-0.15678	-1.6755	1.01
8948	*APOLD1*	2.8419	0.9954	-0.80641	-2.0198	1.01
8949	*HLA-G*	3.0675	1.7164	-1.7541	-2.5477	1.01
8950	*MED26*	2.5496	1.7192	-1.2391	-1.5644	1.01
8951	*PIGQ*	2.4363	0.52691	0.06617	-0.23536	1.01
8952	*DIRAS3*	1.2571	1.1341	0.63785	0.39417	1.01
8953	*ARHGAP21*	3.0388	0.68386	-0.69497	-1.9935	1.01
8954	*CCL22*	3.2805	0.26885	-0.52167	-0.79934	1.01
8955	*C2orf70*	4.4012	-0.21964	-1.1545	-1.4325	1.01
8956	*KRCC1*	3.4469	0.58818	-1.0095	-1.577	1.01
8957	*SLC6A2*	3.5719	-0.15153	-0.39494	-0.96173	1.01
8958	*PRDM16*	4.6127	-0.61515	-0.97252	-2.0488	1.01
8959	*PDE4C*	2.1391	1.6496	-0.76379	-0.93525	1.01
8960	*CMKLR1*	3.4725	0.11075	-0.56003	-0.97549	1.01
8961	*LOC730159*	2.3141	0.72758	-0.01854	-0.41642	1.01
8962	*METTL11B*	3.6448	-0.14906	-0.4744	-2.9632	1.01
8963	*MED13L*	2.6925	0.47245	-0.14563	-0.17323	1.01

8964	*SRY*	4.6778	-0.37125	-1.2876	-2.1696	1.01
8965	*S100A8*	3.9913	-0.00829	-0.96497	-2.4112	1.01
8966	*KLHL41*	3.3922	0.06609	-0.44136	-1.0212	1.01
8967	*PLA2G15*	2.7634	0.64093	-0.38754	-0.84395	1.01
8968	*TMEM260*	2.8475	0.34671	-0.17752	-0.53029	1.01
8969	*LSP1*	1.6511	0.90252	0.46288	-1.1713	1.01
8970	*MMP25*	4.0456	0.059245	-1.0884	-1.8341	1.01
8971	*TENM4*	4.0392	0.44389	-1.468	-2.6939	1.01
8972	*PRAC2*	3.4218	0.64939	-1.0564	-1.6763	1.00
8973	*FARP1*	3.7033	-0.22943	-0.46017	-2.0908	1.00
8974	*CDC73*	2.9981	0.26556	-0.25022	-2.2644	1.00
8975	*SESN1*	4.0759	-0.11897	-0.94536	-0.96395	1.00
8976	*DUSP5*	2.87	1.0578	-0.91701	-2.0675	1.00
8977	*SERTM1*	3.9686	0.38081	-1.3387	-1.7407	1.00
8978	*ARHGEF9*	3.1592	1.0898	-1.2386	-1.8293	1.00
8979	*ZNF436*	2.2793	1.2514	-0.5206	-0.73823	1.00
8980	*HUS1B*	1.8952	1.2392	-0.12433	-1.9338	1.00
8981	*TSC22D1*	1.2851	1.1512	0.57229	-0.71317	1.00
8982	*PROM2*	2.5624	1.5177	-1.072	-1.5671	1.00
8983	*KRTAP9-2*	2.2981	0.55603	0.15285	-1.6143	1.00
8984	*WFDC13*	2.5899	0.5712	-0.15567	-1.268	1.00
8985	*FXYD4*	3.4824	-0.02342	-0.45443	-0.99418	1.00
8986	*GNG3*	4.1056	-0.24591	-0.85552	-1.1756	1.00
8987	*ZNF333*	1.3421	0.89593	0.76588	-0.73728	1.00
8988	*C10orf25*	3.2349	0.074008	-0.30607	-1.2884	1.00
8989	*MOBP*	2.3206	0.80359	-0.12213	-0.53361	1.00
8990	*FIZ1*	2.3968	0.60687	-0.00164	-0.48101	1.00
8991	*CD1D*	3.5188	0.54668	-1.0635	-1.257	1.00
8992	*PTPRN*	2.7879	1.0757	-0.86182	-2.3145	1.00
8993	*ANKRD13B*	1.3331	0.93177	0.73674	-1.4234	1.00
8994	*FNDC8*	3.0985	0.30051	-0.39779	-0.67731	1.00
8995	*KRT82*	1.7948	0.66293	0.54271	-1.9659	1.00
8996	*IFI16*	3.1216	0.15514	-0.27676	-0.81509	1.00
8997	*MANSC4*	3.6962	-0.01874	-0.67801	-1.6356	1.00
8998	*DCLRE1A*	3.4129	0.13535	-0.54889	-1.3419	1.00
8999	*LRP11*	2.7187	0.16358	0.11566	-0.14427	1.00
9000	*AK9*	1.4294	1.0145	0.55379	0.19481	1.00
9001	*ADAMTSL4*	3.2182	-0.06587	-0.15464	-0.58005	1.00
9002	*CLEC3B*	2.6881	0.60138	-0.29186	-1.2474	1.00
9003	*ZNF35*	3.4853	-0.0265	-0.46124	-1.4062	1.00
9004	*MYL3*	2.5154	1.0341	-0.55207	-1.4414	1.00
9005	*GBA*	3.6178	0.30722	-0.9279	-1.0948	1.00
9006	*TEC*	2.8036	0.17295	0.02003	-2.0593	1.00
9007	*MMD*	2.479	0.50113	0.01511	-0.20532	1.00
9008	*INPP1*	2.2553	0.56457	0.17526	-0.51182	1.00
9009	*PABPC5*	2.225	0.7435	0.02648	-4.1136	1.00

9010	*ANKRD33B*	4.3933	0.3701	-1.7688	-2.1357	1.00
9011	*TRIM28*	2.8247	0.2669	-0.09731	-0.41642	1.00
9012	*ALS2*	4.3147	-0.03091	-1.2896	-2.298	1.00
9013	*CCDC67*	4.3459	-0.30877	-1.043	-2.7221	1.00
9014	*MTURN*	2.3177	0.78549	-0.10911	-2.2075	1.00
9015	*ASIP*	2.4221	1.3946	-0.82345	-2.3101	1.00
9016	*LMO3*	2.2632	1.2839	-0.55424	-1.5544	1.00
9017	*SPACA3*	3.7671	0.67638	-1.4507	-1.5912	1.00
9018	*SHANK3*	3.0007	0.48128	-0.49016	-1.1685	1.00
9019	*RMI2*	2.6024	0.66261	-0.27548	-1.6323	1.00
9020	*HIST1H3E*	5.1982	-0.15148	-2.0574	-2.278	1.00
9021	*CEP76*	1.8612	1.7298	-0.60262	-1.1199	1.00
9022	*ATG2A*	1.6646	0.85125	0.47215	0.11075	1.00
9023	*FBXL19*	3.7955	-0.38428	-0.42356	-1.578	1.00
9024	*VDAC2*	2.7654	0.21699	0.00499	-1.4615	1.00
9025	*C6orf47*	3.9191	-0.31508	-0.61829	-1.4932	1.00
9026	*SMCO3*	1.2847	0.89979	0.80093	-0.66488	1.00
9027	*IPO13*	3.2947	0.57564	-0.88507	-1.7807	1.00
9028	*NLRP14*	5.5107	-0.71009	-1.8157	-2.3032	0.99
9029	*CTSE*	2.6504	0.49173	-0.15746	-1.3184	0.99
9030	*LGR6*	3.1539	0.090093	-0.25941	-0.44866	0.99
9031	*LHFPL3*	1.9365	0.66302	0.3849	-2.5934	0.99
9032	*CHRNA9*	3.6242	-0.28994	-0.35044	-1.2464	0.99
9033	*BAZ2B*	3.6769	-0.02287	-0.67138	-2.3096	0.99
9034	*SIGLECL1*	2.8831	1.219	-1.12	-1.4994	0.99
9035	*RSPO3*	4.007	0.74959	-1.7745	-3.5428	0.99
9036	*KRT12*	1.5256	1.0352	0.42116	-1.5962	0.99
9037	*ATP9B*	3.0833	1.4025	-1.5039	-1.6636	0.99
9038	*CWC22*	3.8166	0.65738	-1.4924	-1.6243	0.99
9039	*LGALS9*	3.392	0.50675	-0.91874	-1.0688	0.99
9040	*OR2AG1*	1.8051	1.4952	-0.3205	-0.37417	0.99
9041	*GAGE12I*	2.169	2.0929	-1.2827	-1.8102	0.99
9042	*RNF2*	3.0988	0.054625	-0.17432	-1.278	0.99
9043	*ANKRD55*	2.7454	0.59967	-0.36689	-1.6085	0.99
9044	*ZRSR2*	3.6445	0.19726	-0.86358	-1.3557	0.99
9045	*IRF1*	4.8308	-0.52426	-1.3285	-1.6597	0.99
9046	*CMTM8*	3.485	0.5883	-1.0953	-1.161	0.99
9047	*ZNF180*	2.165	1.0832	-0.2705	-0.45152	0.99
9048	*SEMA6B*	3.6883	-0.17054	-0.54008	-1.2792	0.99
9049	*FARS2*	3.0505	0.26985	-0.34275	-0.80061	0.99
9050	*KIAA1024*	5.0758	-0.25597	-1.8427	-2.8628	0.99
9051	*PRAMEF15*	2.0277	1.7712	-0.82208	-1.6764	0.99
9052	*LPCAT3*	1.35	1.0769	0.54939	-0.18229	0.99
9053	*LSM11*	3.324	0.54891	-0.89667	-1.803	0.99
9054	*HFM1*	2.2777	1.125	-0.42658	-1.8598	0.99
9055	*FAM50A*	3.7177	0.6833	-1.4256	-2.0553	0.99

9056	*ESPN*	5.0614	-0.47327	-1.6129	-1.7743	0.99
9057	*FN3KRP*	4.5944	-0.35652	-1.2628	-1.3574	0.99
9058	*ERICH1*	1.4648	1.1674	0.34276	-0.78229	0.99
9059	*PECAM1*	3.3543	-0.17238	-0.2072	-0.51469	0.99
9060	*CHRFAM7A*	1.4106	0.84854	0.71536	0.062078	0.99
9061	*MUM1*	4.0893	-0.2505	-0.86479	-2.555	0.99
9062	*ALG10B*	2.9177	0.16038	-0.1049	-2.1576	0.99
9063	*NEUROD6*	3.234	0.82344	-1.0843	-1.3016	0.99
9064	*SNX32*	3.3575	0.48331	-0.86861	-2.2171	0.99
9065	*ARIH2*	3.5509	-0.23428	-0.34535	-0.72038	0.99
9066	*RELA*	3.3522	-0.1666	-0.21563	-2.224	0.99
9067	*EPS8L2*	3.7651	-0.26098	-0.53437	-1.8591	0.99
9068	*ING1*	3.27	0.004671	-0.30535	-2.7736	0.99
9069	*ANP32D*	2.8828	0.20422	-0.11788	-0.41359	0.99
9070	*ATP1A2*	1.4164	0.81004	0.74243	0.44887	0.99
9071	*CNOT1*	2.5459	0.32329	0.09956	-1.8223	0.99
9072	*ZBED6CL*	3.5713	-0.06413	-0.53845	-0.92214	0.99
9073	*MARC2*	3.5304	1.0298	-1.5916	-1.9404	0.99
9074	*PAICS*	4.2383	-0.60655	-0.66362	-1.0576	0.99
9075	*RAB40B*	2.196	1.1243	-0.35447	-1.7538	0.99
9076	*MYH10*	1.6035	0.76737	0.59474	-2.0214	0.99
9077	*AGPS*	3.024	0.095844	-0.15499	-0.39017	0.99
9078	*CRISP2*	3.2864	0.002484	-0.32495	-2.7221	0.99
9079	*ARHGAP31*	3.6555	0.006708	-0.70051	-0.7757	0.99
9080	*WNK2*	2.6418	0.45325	-0.13353	-0.80249	0.99
9081	*DDX54*	2.1083	0.89187	-0.03962	-0.57078	0.99
9082	*RABIF*	3.4464	-0.03492	-0.45254	-1.6495	0.99
9083	*CCDC116*	3.1361	0.94342	-1.1207	-1.9047	0.99
9084	*EXOC3L4*	2.7964	0.67915	-0.51722	-1.3954	0.99
9085	*CSNK1G3*	1.4257	1.0702	0.46191	-1.4622	0.99
9086	*TEX264*	2.7436	1.6993	-1.4852	-1.7709	0.99
9087	*RNF224*	3.0568	0.24928	-0.34927	-1.448	0.99
9088	*NRCAM*	2.8461	0.24289	-0.13244	-0.51302	0.99
9089	*LRP6*	3.5073	0.30398	-0.855	-2.179	0.99
9090	*ZNF329*	4.2139	0.32898	-1.5866	-1.7153	0.99
9091	*RAB3IL1*	3.7902	-0.18039	-0.6536	-1.6251	0.99
9092	*HEPACAM*	3.0032	0.36038	-0.40761	-1.0826	0.99
9093	*TPSB2*	2.5098	0.23771	0.20794	-0.42074	0.99
9094	*TTC14*	3.2298	0.071102	-0.34558	-2.7008	0.99
9095	*H1FNT*	2.9934	1.3432	-1.3813	-2.1143	0.99
9096	*ZFP30*	1.1865	1.0724	0.69576	-1.1995	0.98
9097	*CALR3*	2.8546	0.71901	-0.61958	-1.9794	0.98
9098	*NGB*	3.7967	-0.18717	-0.65581	-1.0908	0.98
9099	*PBX2*	2.9458	0.11388	-0.10615	-1.3827	0.98
9100	*ATP5H*	1.2805	1.0105	0.66253	-1.4282	0.98
9101	*RAB32*	2.1478	1.4529	-0.64748	-1.25	0.98

9102	*SCGB1C1*	2.3894	0.94458	-0.38112	-1.6654	0.98
9103	*NOC2L*	4.181	0.33766	-1.5658	-1.8405	0.98
9104	*ABHD12*	2.6646	0.16344	0.12402	-1.4997	0.98
9105	*EDAR*	1.9337	1.7062	-0.68818	-1.3298	0.98
9106	*ADAMTS16*	4.05	0.2003	-1.2987	-3.1335	0.98
9107	*KIAA1644*	1.9024	0.84645	0.20268	-2.312	0.98
9108	*TBATA*	1.42	1.0564	0.47468	-1.4754	0.98
9109	*SEMA4F*	2.9763	0.17739	-0.20284	-0.20338	0.98
9110	*NAGS*	1.8634	1.6843	-0.59696	-1.0342	0.98
9111	*CCDC12*	2.2898	0.4413	0.21879	-0.69615	0.98
9112	*PRSS55*	3.3475	0.045365	-0.44299	-1.2656	0.98
9113	*CFAP46*	2.6354	1.0513	-0.73708	-0.84659	0.98
9114	*C15orf56*	2.3026	0.82598	-0.17928	-0.27631	0.98
9115	*BAHCC1*	4.9089	-0.41738	-1.544	-2.1412	0.98
9116	*ACSM2A*	5.0652	-0.8606	-1.2574	-1.4467	0.98
9117	*LRRIQ1*	1.8591	1.276	-0.18822	-1.0271	0.98
9118	*SNRPE*	1.7132	1.0795	0.15417	-1.5658	0.98
9119	*C22orf15*	2.5758	0.39754	-0.02667	-0.13281	0.98
9120	*SORD*	1.5983	1.2992	0.04902	-1.1564	0.98
9121	*C5orf47*	3.304	0.055982	-0.41383	-1.6695	0.98
9122	*CMTM6*	3.4386	0.47673	-0.96962	-1.049	0.98
9123	*EPC1*	4.3165	-0.05477	-1.3165	-2.2955	0.98
9124	*NAT8B*	3.4469	0.17441	-0.67625	-0.827	0.98
9125	*PTS*	4.9358	-0.35735	-1.6339	-1.9237	0.98
9126	*ELMOD2*	3.1789	0.35655	-0.59215	-0.72053	0.98
9127	*PRNP*	3.399	0.32829	-0.78444	-1.9891	0.98
9128	*ANHX*	3.0159	0.96387	-1.0372	-1.7266	0.98
9129	*MLNR*	5.2551	-0.61119	-1.7018	-2.4112	0.98
9130	*ZNF75D*	3.0332	0.44884	-0.54101	-2.1599	0.98
9131	*NR2F2*	2.4042	0.58598	-0.04919	-0.14793	0.98
9132	*DEPTOR*	3.311	0.18336	-0.55359	-1.4783	0.98
9133	*PRAMEF8*	2.9873	0.052475	-0.09982	-0.14858	0.98
9134	*SLC43A3*	4.0196	-0.48486	-0.59521	-0.68222	0.98
9135	*AKT1*	2.8587	0.30689	-0.22617	-0.5204	0.98
9136	*LRBA*	4.1914	-0.44198	-0.81034	-0.86698	0.98
9137	*IFNGR1*	4.497	-0.74011	-0.81798	-1.2955	0.98
9138	*TP53BP2*	1.4071	1.1234	0.40836	-0.10044	0.98
9139	*ANKRD20A2*	2.3161	1.8075	-1.1848	-1.6636	0.98
9140	*DDT*	3.0399	0.30927	-0.41087	-2.6485	0.98
9141	*DEFB116*	2.2408	0.49507	0.20228	-0.94848	0.98
9142	*CYP2A13*	2.107	0.86544	-0.03586	-0.27903	0.98
9143	*ZNF154*	1.2419	0.95004	0.74421	-0.75192	0.98
9144	*MTPN*	3.771	0.31864	-1.1537	-2.056	0.98
9145	*SP9*	1.3408	0.84242	0.75259	0.49538	0.98
9146	*TBCE*	2.7864	1.3877	-1.2386	-1.2935	0.98
9147	*HRH1*	2.0518	0.66151	0.22209	-0.15385	0.98

9148	*ECH1*	1.7263	1.6641	-0.45553	-2.0347	0.98
9149	*METTL8*	3.7406	0.15092	-0.95682	-1.5458	0.98
9150	*MRPL37*	4.3924	-0.1342	-1.3237	-1.7493	0.98
9151	*PTPN18*	4.388	-0.31869	-1.1351	-1.5396	0.98
9152	*UVSSA*	2.346	0.97686	-0.38901	-1.0224	0.98
9153	*FAM105A*	3.9472	0.71324	-1.7266	-3.064	0.98
9154	*BTNL2*	3.3373	-0.14247	-0.26159	-0.80581	0.98
9155	*PNPO*	2.0429	1.5579	-0.66897	-2.3719	0.98
9156	*KCTD7*	2.1651	0.46439	0.30209	-1.5115	0.98
9157	*ZC2HC1A*	2.0922	0.76694	0.07197	-1.5544	0.98
9158	*RAB11A*	3.8976	-0.44353	-0.52392	-1.5722	0.98
9159	*HIST1H2BG*	1.6003	1.4728	-0.14304	-0.73668	0.98
9160	*IBTK*	1.5841	0.87244	0.47122	-1.2647	0.98
9161	*FGL1*	2.9792	0.29492	-0.34675	-1.1709	0.98
9162	*GPX6*	2.1492	0.46212	0.31588	0.14394	0.98
9163	*TCP10L2*	2.9045	0.10118	-0.07893	-0.15499	0.98
9164	*EML3*	2.0357	0.55567	0.33523	0.038507	0.98
9165	*STK32B*	2.9118	1.2013	-1.1866	-2.3795	0.98
9166	*FMNL2*	4.2655	-0.52447	-0.81505	-1.2261	0.98
9167	*LOC100134391*	2.2261	0.38204	0.31742	-0.30822	0.98
9168	*PCSK5*	1.3828	0.97	0.57247	-0.49481	0.98
9169	*NDUFA2*	2.7021	0.94515	-0.72294	-0.93525	0.97
9170	*VASP*	4.5156	-0.19249	-1.3988	-2.8823	0.97
9171	*USP19*	3.2289	0.84912	-1.154	-1.802	0.97
9172	*KRTAP3-3*	2.6784	1.8435	-1.5984	-2.0791	0.97
9173	*C9orf40*	2.1054	0.51917	0.29864	-0.11527	0.97
9174	*NAP1L1*	3.272	0.83922	-1.1881	-2.6716	0.97
9175	*DNAI1*	3.193	0.58934	-0.85931	-2.1502	0.97
9176	*OR5D16*	2.6007	0.58937	-0.26712	-0.70532	0.97
9177	*PRR5L*	3.5071	0.041147	-0.62649	-1.9172	0.97
9178	*CKMT2*	3.8165	-0.34199	-0.55302	-1.2426	0.97
9179	*PACSIN1*	1.5272	0.75428	0.63967	-1.0949	0.97
9180	*PLCD4*	3.4011	0.13771	-0.61779	-2.29	0.97
9181	*ADIRF*	2.9607	0.96998	-1.0097	-1.6731	0.97
9182	*POF1B*	2.0921	0.70247	0.12494	-2.0433	0.97
9183	*BMP10*	2.2945	0.53438	0.09061	-1.3694	0.97
9184	*PRR20E*	2.7877	0.71607	-0.5845	-1.6755	0.97
9185	*ZNF827*	1.1772	0.93576	0.80518	-1.1875	0.97
9186	*RPRM*	4.2649	0.39699	-1.7438	-2.3174	0.97
9187	*SNAP29*	3.336	0.42879	-0.84674	-1.8157	0.97
9188	*PRKAG3*	2.4377	0.99692	-0.51745	-0.65145	0.97
9189	*TAC3*	2.2436	0.61237	0.06116	-0.24191	0.97
9190	*PCDHB2*	4.1791	-0.41383	-0.84816	-3.1921	0.97
9191	*OR5AR1*	1.3882	1.3491	0.1795	-1.4354	0.97
9192	*OR7G1*	3.8427	0.16896	-1.0953	-1.8995	0.97
9193	*MT1E*	3.4695	0.021388	-0.57503	-1.268	0.97

9194	*SLC26A11*	2.7646	0.53121	-0.38044	-0.88143	0.97
9195	*VPS11*	3.6193	0.12446	-0.8285	-1.2472	0.97
9196	*ZC3H6*	1.7367	0.62912	0.54939	-0.91028	0.97
9197	*KRTAP5-8*	1.3424	0.80548	0.76656	0.18083	0.97
9198	*OR2M2*	1.4823	0.9173	0.51379	-1.94	0.97
9199	*SLC27A4*	2.2914	0.47748	0.14437	-1.5448	0.97
9200	*OTX1*	3.6906	-0.27333	-0.50436	-1.9943	0.97
9201	*NCEH1*	3.243	0.30923	-0.63935	-0.65393	0.97
9202	*PAQR7*	3.81	-0.23297	-0.66419	-1.3266	0.97
9203	*TMEM14A*	3.2098	0.002272	-0.29982	-0.33191	0.97
9204	*ADD2*	2.6955	0.495	-0.2783	-0.37686	0.97
9205	*GLYATL2*	4.451	-0.14828	-1.3907	-1.6276	0.97
9206	*SOX11*	2.6892	0.71252	-0.48982	-1.0323	0.97
9207	*AMPD3*	3.615	0.006369	-0.70957	-0.99448	0.97
9208	*AP3D1*	3.2042	0.24472	-0.53766	-0.54744	0.97
9209	*GTF3C2*	2.4831	1.6372	-1.2094	-1.3457	0.97
9210	*CCL17*	2.3897	1.0766	-0.55601	-0.86123	0.97
9211	*TWIST1*	3.4734	0.24772	-0.81136	-1.5687	0.97
9212	*MYH4*	3.1562	0.045209	-0.29305	-0.99526	0.97
9213	*ATG13*	3.5981	-0.27707	-0.41308	-0.50474	0.97
9214	*MIB1*	4.4873	-0.15943	-1.4204	-3.2419	0.97
9215	*BPIFA3*	3.7406	0.021312	-0.85456	-2.0277	0.97
9216	*OARD1*	4.0893	-0.50419	-0.67888	-2.0927	0.97
9217	*FPGS*	4.0759	0.49846	-1.6682	-3.1229	0.97
9218	*ADGRG6*	1.7931	0.92282	0.19018	-1.8046	0.97
9219	*FAM160B2*	1.5882	0.71542	0.60228	-0.32466	0.97
9220	*NSDHL*	2.2471	0.4371	0.22117	-0.43859	0.97
9221	*SLC24A5*	3.5726	-0.05505	-0.61329	-0.66577	0.97
9222	*TDRD9*	4.1374	-0.07224	-1.1619	-1.2574	0.97
9223	*FBXO28*	2.4771	1.0303	-0.60628	-1.0442	0.97
9224	*SFTPA2*	1.3733	0.78594	0.74098	-0.95258	0.97
9225	*GLT6D1*	2.371	0.7515	-0.22313	-2.3629	0.97
9226	*RAPGEF6*	1.3316	0.7876	0.77938	-2.3949	0.97
9227	*OPRM1*	1.909	0.58665	0.40281	0.16544	0.97
9228	*RSAD2*	4.5035	-0.40427	-1.2008	-1.2097	0.97
9229	*NUP88*	1.5552	1.4705	-0.12896	-1.8995	0.97
9230	*MBP*	2.2984	1.9791	-1.3808	-1.6601	0.97
9231	*RGS1*	4.4869	-0.64851	-0.94171	-1.8714	0.97
9232	*NR4A1*	1.5219	1.0386	0.33583	-0.14762	0.97
9233	*SLC34A2*	1.7156	0.742	0.43844	-1.4747	0.97
9234	*ERCC6*	4.3141	0.17607	-1.5949	-2.1108	0.97
9235	*DMBX1*	3.1248	0.82516	-1.0564	-2.0992	0.96
9236	*CD163L1*	2.4991	0.39392	0.00046	-0.96878	0.96
9237	*SSUH2*	3.2241	-0.05841	-0.27244	-1.1199	0.96
9238	*STMND1*	1.8417	0.8072	0.24339	-1.5373	0.96
9239	*HEPHL1*	2.5618	0.71832	-0.38815	-1.8157	0.96

9240	*EXOSC5*	2.9287	0.12559	-0.16234	-2.3452	0.96
9241	*PLAUR*	3.0465	-0.07748	-0.07903	-1.1904	0.96
9242	*LGALS7B*	3.8126	-0.11865	-0.80675	-2.8553	0.96
9243	*ORMDL3*	2.5128	0.22286	0.15053	-0.77038	0.96
9244	*RNF157*	2.2569	0.4966	0.13265	-1.9367	0.96
9245	*MFSD11*	3.2345	-0.1496	-0.19915	-1.01	0.96
9246	*KBTBD4*	1.3941	0.93201	0.55956	-1.765	0.96
9247	*KDM1B*	3.127	0.26849	-0.50989	-1.4019	0.96
9248	*IL7R*	1.9017	1.7869	-0.80341	-2.0134	0.96
9249	*SULF1*	3.8542	-0.19043	-0.7789	-1.1529	0.96
9250	*B3GNTL1*	2.3889	0.5969	-0.10116	-0.35328	0.96
9251	*ERGIC1*	1.6161	1.2633	0.00452	-1.3769	0.96
9252	*DLX4*	2.6855	1.2253	-1.0273	-1.6246	0.96
9253	*INTS8*	3.4365	0.29458	-0.8478	-2.5561	0.96
9254	*ATP5L*	3.3143	0.39047	-0.82166	-1.8572	0.96
9255	*HMCN1*	4.7491	-0.25597	-1.6105	-2.381	0.96
9256	*TXNDC16*	3.6241	-0.35899	-0.38248	-0.57513	0.96
9257	*GTPBP2*	2.8305	0.68209	-0.63014	-1.3542	0.96
9258	*SLC16A3*	3.1874	0.53862	-0.84457	-1.1338	0.96
9259	*C17orf104*	2.4015	0.4429	0.03663	-1.5901	0.96
9260	*CDY1*	2.0628	0.97988	-0.16266	-1.5544	0.96
9261	*TRMT2A*	2.241	1.4002	-0.76124	-1.0501	0.96
9262	*BTD*	2.7196	0.42427	-0.26466	-1.1904	0.96
9263	*KIAA0101*	2.8833	0.68986	-0.69411	-0.70463	0.96
9264	*PRDM12*	4.0072	0.16975	-1.2985	-1.6033	0.96
9265	*NOS3*	3.9994	0.26037	-1.3814	-1.4636	0.96
9266	*ATP10B*	1.6737	1.2651	-0.06044	-1.7571	0.96
9267	*ZNF639*	2.0545	0.49193	0.33119	-1.151	0.96
9268	*CUX2*	2.0644	0.69779	0.1148	-1.6339	0.96
9269	*TM2D3*	2.84	1.0813	-1.0446	-1.9179	0.96
9270	*WDFY2*	3.0273	-0.018	-0.13273	-1.4856	0.96
9271	*CPSF6*	3.0341	-0.01264	-0.14517	-1.1453	0.96
9272	*DNAAF2*	3.6141	-0.21938	-0.51929	-0.97776	0.96
9273	*VPREB1*	1.7377	1.1056	0.03207	-0.04323	0.96
9274	*CHIT1*	1.3122	1.092	0.47102	-0.39433	0.96
9275	*IL27RA*	1.1476	0.96542	0.76167	-1.1676	0.96
9276	*CBFA2T3*	2.3895	0.47483	0.01	-1.7495	0.96
9277	*LAMB3*	2.5815	0.45714	-0.16543	-0.24551	0.96
9278	*SLC22A18AS*	3.6098	-0.34364	-0.3931	-1.0621	0.96
9279	*PDDC1*	1.7874	0.84999	0.2352	-0.03676	0.96
9280	*C18orf32*	2.3719	0.36198	0.13733	-0.20663	0.96
9281	*CSDC2*	2.0309	1.0682	-0.22813	-0.82398	0.96
9282	*CRB3*	3.7945	0.53127	-1.4555	-1.5952	0.96
9283	*ENPP6*	3.6968	-0.25206	-0.57484	-1.2935	0.96
9284	*CEACAM1*	2.4943	0.21771	0.1572	-1.0948	0.96
9285	*TRADD*	3.6599	0.94688	-1.7392	-1.8184	0.96

9286	*SGK223*	5.6991	-0.88272	-1.9502	-2.8849	0.96
9287	*KDM7A*	3.0787	-0.06913	-0.14391	-0.92046	0.96
9288	*DMRTA1*	2.9139	0.27812	-0.32728	-2.1321	0.95
9289	*LRRC4C*	3.5319	-0.3128	-0.35441	-0.49953	0.95
9290	*MRGPRF*	3.0866	0.88791	-1.1105	-1.2935	0.95
9291	*PIK3C2A*	2.655	0.62398	-0.41502	-2.1419	0.95
9292	*DLGAP3*	1.8324	1.5233	-0.49322	-1.9622	0.95
9293	*EFHC2*	1.5408	0.67206	0.64951	-0.19342	0.95
9294	*CHGB*	4.2167	-0.51642	-0.83808	-2.8628	0.95
9295	*CLDN3*	3.5378	0.81333	-1.4894	-1.6281	0.95
9296	*RNF128*	2.8861	0.13021	-0.15509	-1.0458	0.95
9297	*PDGFC*	3.1856	-0.11363	-0.21267	-0.29089	0.95
9298	*BAHD1*	1.8661	1.1693	-0.17647	-0.85236	0.95
9299	*CCL26*	2.4241	0.79277	-0.35816	-1.0315	0.95
9300	*KEL*	4.5804	-0.59174	-1.1305	-2.8327	0.95
9301	*HSPB9*	4.4311	0.19946	-1.7726	-2.1572	0.95
9302	*FGF14*	4.3021	-0.23957	-1.2055	-1.8554	0.95
9303	*ZBTB8A*	3.1418	1.6772	-1.9621	-2.5707	0.95
9304	*RASD2*	2.91	0.41436	-0.46757	-2.1108	0.95
9305	*LRIG1*	4.1883	-0.51666	-0.81517	-0.84523	0.95
9306	*SP2*	4.6075	-0.80256	-0.94965	-2.3751	0.95
9307	*PTK6*	1.3074	1.1777	0.36943	-2.1669	0.95
9308	*SP4*	3.0703	0.99568	-1.2118	-2.0697	0.95
9309	*EPHA4*	2.7049	0.72608	-0.57742	-2.2528	0.95
9310	*CNBD1*	3.04	1.3207	-1.5073	-2.2951	0.95
9311	*HIST2H2AA4*	4.4314	-0.5231	-1.0564	-1.8382	0.95
9312	*HIST1H4G*	3.3448	0.52919	-1.0236	-1.6313	0.95
9313	*DYNLT3*	3.2556	-0.10364	-0.30215	-0.4492	0.95
9314	*CMBL*	2.0414	0.99411	-0.18589	-0.98026	0.95
9315	*C21orf2*	2.4683	0.21218	0.16903	-0.25605	0.95
9316	*C14orf178*	4.8173	0.26323	-2.2311	-3.1921	0.95
9317	*IGSF22*	1.8366	0.94913	0.06301	-1.6575	0.95
9318	*SRPX*	2.0686	0.53345	0.24615	-0.40104	0.95
9319	*ZSCAN5A*	4.9026	-0.64769	-1.4076	-2.0782	0.95
9320	*ZNF57*	2.3731	0.40051	0.07356	-1.3113	0.95
9321	*SOX1*	2.297	0.47342	0.07632	-1.4893	0.95
9322	*PROSC*	2.1766	0.63011	0.03998	0.011761	0.95
9323	*SENP3*	4.5589	-0.50133	-1.2109	-2.4595	0.95
9324	*C12orf10*	1.6188	0.9699	0.25747	-1.359	0.95
9325	*SLC9A4*	2.5869	1.0304	-0.77114	-1.6488	0.95
9326	*PLD3*	3.0505	0.7254	-0.92987	-0.9327	0.95
9327	*BRINP1*	4.4413	-0.2715	-1.3247	-1.4998	0.95
9328	*CD1E*	5.054	-1.0283	-1.1812	-1.3023	0.95
9329	*CMTR2*	1.9029	0.57888	0.36202	-1.2834	0.95
9330	*GLMP*	2.2692	0.44355	0.13096	-1.478	0.95
9331	*TBC1D30*	2.2726	0.55491	0.0157	-0.72157	0.95

9332	*CEACAM3*	1.9184	0.56676	0.35793	-0.74604	0.95
9333	*CCDC177*	2.3437	1.084	-0.58534	-1.7782	0.95
9334	*ULBP1*	2.4394	0.82505	-0.4221	-1.4182	0.95
9335	*CDKN2A*	1.9514	0.67735	0.21332	-0.78096	0.95
9336	*DDX3Y*	3.4573	0.039168	-0.6548	-2.3623	0.95
9337	*C22orf24*	3.3539	-0.15561	-0.35674	-1.6125	0.95
9338	*RASA2*	1.9364	0.83652	0.06861	-0.43964	0.95
9339	*IL17B*	1.4896	1.2595	0.09207	-0.74576	0.95
9340	*ZNF16*	3.101	-0.07692	-0.18381	-1.2049	0.95
9341	*C10orf90*	2.6174	0.92252	-0.70132	-0.84278	0.95
9342	*MIEN1*	3.0649	0.39779	-0.62489	-1.9308	0.95
9343	*CEBPA*	3.0397	0.26983	-0.47206	-1.4033	0.95
9344	*SLC44A2*	4.7917	-0.38059	-1.5744	-1.7577	0.95
9345	*ACTRT3*	2.1076	0.48816	0.24082	-0.68813	0.95
9346	*IFT122*	3.4517	0.73607	-1.3523	-2.2169	0.95
9347	*HIST1H3H*	2.9244	0.16197	-0.25095	-0.8397	0.95
9348	*VWA1*	4.2712	0.15938	-1.5962	-2.1605	0.94
9349	*SLC41A3*	2.4008	1.622	-1.1888	-1.3664	0.94
9350	*TBX4*	2.5692	0.55068	-0.28588	-1.4914	0.94
9351	*EID2*	3.9228	0.39016	-1.479	-2.1412	0.94
9352	*SLC25A12*	4.383	-0.59183	-0.95753	-1.6419	0.94
9353	*KLB*	2.3737	0.39468	0.06461	-1.2324	0.94
9354	*MUTYH*	3.9397	-0.36786	-0.73911	-0.97369	0.94
9355	*CDK11A*	2.7265	0.15254	-0.04703	-0.48416	0.94
9356	*RAB41*	1.4741	1.1154	0.24234	-0.55675	0.94
9357	*ARID4B*	4.3895	-0.26423	-1.2936	-1.5724	0.94
9358	*DBNL*	3.7336	0.47677	-1.379	-1.8102	0.94
9359	*HBA1*	3.1367	0.46651	-0.77196	-1.0181	0.94
9360	*COMMD8*	3.1786	0.038005	-0.3859	-0.9109	0.94
9361	*TMEM143*	2.0862	1.1336	-0.38998	-0.62047	0.94
9362	*SEPT7*	1.7953	0.52306	0.51143	-0.60441	0.94
9363	*GALNTL5*	5.6911	-1.2623	-1.5992	-2.0343	0.94
9364	*R3HDM4*	1.9226	0.49003	0.4166	-0.72095	0.94
9365	*KRTAP23-1*	3.4964	-0.05428	-0.61297	-1.5131	0.94
9366	*NHLH1*	2.0974	0.53893	0.19272	-0.24741	0.94
9367	*OR2L5*	3.4078	0.13081	-0.70998	-2.7221	0.94
9368	*TBC1D2*	3.3866	0.76489	-1.3237	-1.5052	0.94
9369	*DGKQ*	3.8656	0.1573	-1.1958	-1.6836	0.94
9370	*MRPL55*	3.1901	-0.03784	-0.32546	-2.1368	0.94
9371	*KMO*	1.5552	0.67432	0.59648	-0.95802	0.94
9372	*MORC4*	1.5739	0.83166	0.41996	-0.80248	0.94
9373	*GRHL2*	2.6201	0.95101	-0.7458	-1.095	0.94
9374	*PUSL1*	3.3628	0.15963	-0.69712	-1.3627	0.94
9375	*C5orf34*	2.621	1.146	-0.94171	-1.691	0.94
9376	*HYPK*	2.5631	0.25289	0.00884	-2.6217	0.94
9377	*NXF2*	1.4083	0.84496	0.57155	-0.85059	0.94

9378	*TRAPPC10*	2.3892	0.59437	-0.15953	-0.18876	0.94
9379	*USO1*	2.0404	0.76144	0.0217	-1.8341	0.94
9380	*FAM209B*	5.2574	-1.0005	-1.4337	-1.8856	0.94
9381	*RPS16*	2.2126	0.89422	-0.28374	-0.94956	0.94
9382	*OR13H1*	3.2809	0.24924	-0.70708	-0.73708	0.94
9383	*TAS2R38*	2.01	0.87194	-0.0597	-0.15447	0.94
9384	*CCL13*	1.7462	1.585	-0.50953	-0.77159	0.94
9385	*LLPH*	3.5694	0.83015	-1.578	-1.6796	0.94
9386	*CPED1*	3.2958	-0.07644	-0.39817	-1.3039	0.94
9387	*NCOA7*	3.6548	0.046816	-0.88062	-1.5458	0.94
9388	*TBC1D23*	4.0113	0.34839	-1.539	-1.83	0.94
9389	*PIK3R1*	2.8474	0.83693	-0.86399	-1.0453	0.94
9390	*SDCBP*	2.8911	0.44675	-0.51799	-1.6759	0.94
9391	*KCNMB1*	1.9318	0.79459	0.09276	-0.44843	0.94
9392	*HBG1*	1.7886	0.5743	0.45582	-0.54252	0.94
9393	*NFYC*	1.8745	1.5695	-0.62644	-1.9404	0.94
9394	*CAMK2D*	2.9476	1.0379	-1.1685	-1.3542	0.94
9395	*EBLN2*	2.438	0.81815	-0.43966	-1.4302	0.94
9396	*RSPH3*	2.3738	0.65244	-0.20988	-2.4633	0.94
9397	*PYCR1*	2.9595	0.20565	-0.34887	-0.56098	0.94
9398	*SYT2*	2.3481	0.94455	-0.47642	-0.83278	0.94
9399	*C12orf54*	4.0805	-0.22469	-1.0399	-1.2167	0.94
9400	*VPS18*	2.5281	1.0166	-0.72881	-1.5787	0.94
9401	*MIIP*	2.1451	0.72634	-0.05599	-1.1592	0.94
9402	*HAP1*	2.2415	0.36658	0.20721	0.011291	0.94
9403	*KIAA1804*	2.6638	0.63413	-0.48272	-3.4957	0.94
9404	*SMPD3*	3.8821	0.41678	-1.4841	-2.0147	0.94
9405	*ARHGDIA*	3.1577	0.39153	-0.73465	-2.8398	0.94
9406	*FCGR3A*	2.8159	0.27065	-0.2735	-1.6802	0.94
9407	*SPCS2*	3.386	0.14048	-0.71363	-1.0093	0.94
9408	*PLSCR2*	3.3652	1.1184	-1.6709	-1.8751	0.94
9409	*TMEM52*	1.2379	0.83583	0.73815	0.5381	0.94
9410	*PARK2*	3.4318	-0.13189	-0.48829	-1.3818	0.94
9411	*ZNF557*	5.0886	-0.91269	-1.3643	-1.6065	0.94
9412	*EMP1*	2.9648	0.58987	-0.74389	-2.1187	0.94
9413	*PLAC9*	1.9743	0.92295	-0.08675	-1.2719	0.94
9414	*PCDHA10*	2.5088	0.84114	-0.54219	-1.4364	0.94
9415	*COX6B2*	3.424	-0.01745	-0.59981	-1.6567	0.94
9416	*NEK1*	2.946	0.000336	-0.13961	-1.2603	0.94
9417	*TAS2R31*	2.1367	1.1372	-0.46724	-0.82172	0.94
9418	*CIB3*	2.9718	0.7152	-0.88062	-1.3279	0.94
9419	*OR6P1*	4.3523	0.10613	-1.6524	-1.9727	0.94
9420	*ZNF384*	1.0873	1.0385	0.68013	-1.5183	0.94
9421	*SALL2*	3.6538	-0.4046	-0.4433	-1.1096	0.94
9422	*KRTAP10-6*	1.4912	0.96328	0.3501	-1.3633	0.93
9423	*BLZF1*	3.0731	0.64348	-0.91234	-1.4168	0.93

9424	*NRBP2*	3.459	-0.02912	-0.62672	-0.82921	0.93
9425	*MS4A13*	3.6239	0.25077	-1.0723	-1.2651	0.93
9426	*PRDM8*	1.5954	1.4433	-0.23638	-1.1317	0.93
9427	*TSPYL2*	2.3061	0.67237	-0.17629	-0.9612	0.93
9428	*GIP*	2.5726	0.44348	-0.2146	-0.85931	0.93
9429	*C4orf47*	1.4317	0.81801	0.55092	0.12097	0.93
9430	*MAVS*	1.8747	1.5241	-0.5986	-1.438	0.93
9431	*ZNF474*	3.0447	0.50855	-0.75354	-3.3966	0.93
9432	*CHMP1B*	3.2781	0.56038	-1.0388	-2.5743	0.93
9433	*OVCH2*	4.0698	-0.40961	-0.86089	-1.802	0.93
9434	*EVPLL*	1.1244	0.85331	0.82137	0.09768	0.93
9435	*FOSL1*	2.6438	0.12044	0.03435	-1.7134	0.93
9436	*LARS2*	4.5952	-0.02993	-1.767	-1.9819	0.93
9437	*SNTG2*	3.5292	0.54452	-1.2755	-1.3358	0.93
9438	*L3MBTL4*	3.4007	-0.01938	-0.58353	-0.68133	0.93
9439	*SLC19A1*	2.5411	0.19604	0.0606	-0.05573	0.93
9440	*ATG16L2*	3.1649	0.62059	-0.98803	-1.6085	0.93
9441	*FOXP3*	3.0095	0.37301	-0.58554	-0.61169	0.93
9442	*HCFC2*	4.0533	0.32994	-1.5865	-1.7068	0.93
9443	*SOBP*	1.9913	1.4652	-0.66008	-1.666	0.93
9444	*SMPX*	3.0412	0.6846	-0.92948	-1.5167	0.93
9445	*SIX6*	2.5174	0.65788	-0.37913	-0.61483	0.93
9446	*TNFRSF4*	3.7578	0.1059	-1.0691	-1.3479	0.93
9447	*TCF3*	2.1748	0.66948	-0.05	-1.536	0.93
9448	*TRIML2*	1.8651	0.56815	0.36017	0.28429	0.93
9449	*CPSF4L*	2.0108	0.44573	0.3359	-2.0545	0.93
9450	*ST3GAL4*	1.3246	1.0754	0.39221	-0.80675	0.93
9451	*NCAM1*	3.4627	0.19462	-0.86556	-1.683	0.93
9452	*SLC43A2*	2.8326	0.22545	-0.26652	-1.203	0.93
9453	*OR51F2*	2.2412	0.27944	0.27053	-1.3886	0.93
9454	*TRIM6*	3.6092	0.1385	-0.95749	-1.4994	0.93
9455	*SLC7A13*	3.1237	0.39289	-0.72656	-0.74943	0.93
9456	*HARS2*	2.3626	1.0547	-0.62762	-1.1034	0.93
9457	*RBM26*	2.821	0.55802	-0.59045	-0.72654	0.93
9458	*CKB*	4.1498	-0.42038	-0.9414	-2.2856	0.93
9459	*DEFB135*	4.1227	0.46402	-1.7991	-5.5895	0.93
9460	*ZNF304*	1.3858	0.92518	0.47636	-3.2419	0.93
9461	*SULT1A3*	2.2727	0.7429	-0.22915	-0.8756	0.93
9462	*AMDHD2*	3.4699	0.25706	-0.94152	-1.4452	0.93
9463	*CDHR2*	3.566	0.00147	-0.78229	-0.83081	0.93
9464	*ZNF541*	3.9516	-0.57921	-0.58771	-1.879	0.93
9465	*SARDH*	3.9953	0.61452	-1.8253	-2.565	0.93
9466	*POLR3G*	4.7638	-0.68606	-1.2934	-1.988	0.93
9467	*NCR2*	2.0102	1.0184	-0.24578	-2.8849	0.93
9468	*SPG21*	3.1702	0.46938	-0.85697	-2.278	0.93
9469	*DDX19A*	2.4808	1.0335	-0.73185	-1.1986	0.93

9470	*PPAPDC1A*	2.2368	0.35673	0.18761	-0.27811	0.93
9471	*FNDC9*	1.6845	0.97526	0.12081	-1.304	0.93
9472	*EML5*	2.6725	0.35935	-0.25163	-1.5672	0.93
9473	*COL15A1*	2.6288	0.40183	-0.2505	-0.54142	0.93
9474	*VWA5B1*	4.2138	-0.48659	-0.94759	-2.7384	0.93
9475	*SLC35A2*	4.4	-0.13539	-1.4856	-2.2154	0.93
9476	*SHROOM1*	1.4619	0.73309	0.58354	-1.7312	0.93
9477	*ARG2*	2.7667	0.23572	-0.224	-0.40319	0.93
9478	*ECI1*	2.8841	0.62385	-0.72969	-1.4584	0.93
9479	*LACC1*	2.3557	0.75415	-0.33253	-0.93892	0.93
9480	*CCDC58*	2.4194	2.4124	-2.0545	-2.2154	0.93
9481	*LOC113230*	2.4322	1.2853	-0.94119	-1.8975	0.93
9482	*VWC2L*	2.9426	0.34843	-0.51585	-1.6934	0.93
9483	*BARHL2*	2.6242	0.26086	-0.11023	-2.3811	0.92
9484	*CYB5R1*	3.6774	-0.2293	-0.67342	-1.5099	0.92
9485	*ADM2*	2.4988	0.23694	0.03866	-0.4311	0.92
9486	*TMEM151A*	3.9742	-0.10473	-1.0951	-2.6889	0.92
9487	*MPP6*	3.265	-0.18269	-0.30801	-1.1674	0.92
9488	*PCGF5*	1.9054	0.81614	0.05273	-0.30883	0.92
9489	*MC5R*	2.6315	0.10737	0.03508	-1.0799	0.92
9490	*BPIFB2*	1.7472	0.99021	0.03515	-1.2421	0.92
9491	*HEBP2*	2.6945	1.1896	-1.1138	-3.0595	0.92
9492	*WDR43*	4.4837	-0.59507	-1.1184	-1.2295	0.92
9493	*DOC2B*	4.1417	-0.4449	-0.92828	-1.9802	0.92
9494	*BCAP31*	4.3016	-0.04002	-1.4936	-1.5979	0.92
9495	*BLOC1S3*	4.2718	-0.12489	-1.379	-2.9234	0.92
9496	*KCNH5*	1.6393	0.8389	0.28866	0.037225	0.92
9497	*TMEM179B*	2.4139	0.87217	-0.51928	-1.3019	0.92
9498	*C3orf20*	2.1129	1.2035	-0.55011	-1.9763	0.92
9499	*PROKR1*	1.8085	0.64943	0.30814	0.18128	0.92
9500	*MT1HL1*	3.1737	0.63025	-1.0385	-1.9885	0.92
9501	*HTR3B*	1.4489	0.73632	0.57896	-1.9372	0.92
9502	*FAIM2*	2.0716	0.6696	0.02237	-1.4028	0.92
9503	*GATA3*	2.8832	-0.02494	-0.09541	-0.49999	0.92
9504	*SLC5A6*	4.4423	-0.60138	-1.0785	-1.2628	0.92
9505	*LITAF*	2.8154	0.12969	-0.18282	-1.5312	0.92
9506	*TOB1*	1.8	1.7174	-0.75603	-1.3162	0.92
9507	*OSTF1*	1.0972	1.0013	0.6624	-0.42777	0.92
9508	*H3F3B*	2.3259	1.5568	-1.122	-2.6593	0.92
9509	*TMEM57*	1.4994	1.1142	0.14641	-1.8157	0.92
9510	*ZFP91*	1.3777	0.89978	0.48216	-1.4639	0.92
9511	*CSF1R*	2.3794	1.7006	-1.3205	-1.5671	0.92
9512	*CAPS2*	3.1442	-0.06466	-0.32015	-0.95044	0.92
9513	*BZW1*	2.7867	0.090471	-0.11811	-1.8435	0.92
9514	*ZC2HC1C*	2.7501	0.32836	-0.31968	-0.9745	0.92
9515	*RHOV*	2.6635	1.0716	-0.97648	-1.2884	0.92

9516	*COL28A1*	2.8282	0.68455	-0.75414	-0.81496	0.92
9517	*ABCC12*	1.9301	1.3644	-0.53613	-0.56145	0.92
9518	*ARHGAP28*	3.0147	0.34019	-0.59847	-0.78221	0.92
9519	*CXXC4*	1.6417	0.80145	0.313	-1.6243	0.92
9520	*SH3D21*	3.3083	0.54097	-1.0942	-2.278	0.92
9521	*TIAM2*	4.133	-0.13094	-1.2471	-2.1896	0.92
9522	*ESCO2*	1.9398	0.55092	0.26289	-0.12795	0.92
9523	*SLC12A8*	3.7222	-0.31414	-0.6548	-1.658	0.92
9524	*TSSK3*	2.2837	0.31015	0.15931	-0.0211	0.92
9525	*ABLIM2*	1.101	0.91654	0.73478	-1.5073	0.92
9526	*GALNT6*	2.8636	0.14215	-0.25415	-0.51363	0.92
9527	*HYKK*	1.3067	1.2013	0.24312	-0.30933	0.92
9528	*CDKL4*	2.177	0.38209	0.19045	-1.5039	0.92
9529	*HEMK1*	2.1984	0.61278	-0.06264	-0.49138	0.92
9530	*TRIM41*	2.3773	0.27695	0.09356	-0.52255	0.92
9531	*SEC61B*	2.5975	1.2007	-1.0506	-1.3689	0.92
9532	*M1AP*	3.8062	0.46677	-1.5271	-1.7766	0.92
9533	*CHST3*	2.5891	0.46379	-0.30715	-1.0704	0.92
9534	*NHLRC1*	2.926	0.8055	-0.9858	-1.6371	0.92
9535	*SLC22A10*	1.1387	0.87806	0.72892	-1.0863	0.92
9536	*C15orf61*	2.3055	1.2767	-0.83676	-1.26	0.92
9537	*ZBTB37*	2.738	1.3069	-1.3	-1.5153	0.91
9538	*E2F3*	3.5402	-0.1095	-0.68727	-1.578	0.91
9539	*PDK4*	1.3388	0.96396	0.44041	-1.1414	0.91
9540	*KRTAP20-2*	4.0101	0.088405	-1.3556	-1.6731	0.91
9541	*ALPK3*	1.6861	1.3632	-0.30653	-0.36236	0.91
9542	*TFB2M*	2.3353	0.92907	-0.52197	-1.6571	0.91
9543	*LDLRAD4*	1.9211	0.51292	0.30732	-0.69754	0.91
9544	*EPHX3*	2.8746	0.20971	-0.34304	-0.67458	0.91
9545	*SNAP25*	1.457	0.84267	0.44136	-0.15323	0.91
9546	*ANO4*	2.0063	0.68585	0.04818	-0.38428	0.91
9547	*KAZN*	3.47	0.033716	-0.76389	-1.5373	0.91
9548	*PNRC1*	2.6212	0.18656	-0.06824	-2.3449	0.91
9549	*GABRR2*	3.9966	0.39547	-1.6526	-2.0301	0.91
9550	*GLDC*	2.5725	0.24598	-0.07903	-0.16277	0.91
9551	*SP110*	5.1005	-0.94219	-1.4197	-1.8775	0.91
9552	*DNAL1*	3.4663	-0.06938	-0.65839	-1.4557	0.91
9553	*HTATSF1*	4.1741	-0.1386	-1.2975	-1.2983	0.91
9554	*TOMM20*	3.1527	-0.03366	-0.38195	-1.2027	0.91
9555	*COG7*	4.2669	-0.5038	-1.0261	-1.6157	0.91
9556	*KIAA1147*	3.249	1.2905	-1.8038	-1.9075	0.91
9557	*KLK12*	3.5859	0.025101	-0.8756	-2.3338	0.91
9558	*SAP30L*	3.7334	-0.28508	-0.71305	-0.76866	0.91
9559	*ERG*	1.8317	0.62448	0.27907	-0.54354	0.91
9560	*UBR3*	2.3346	0.87263	-0.47246	-1.061	0.91
9561	*EIF3B*	3.3727	0.23298	-0.87129	-1.1267	0.91

9562	*TNFSF15*	1.6353	1.4303	-0.33191	-0.62306	0.91
9563	*SLMAP*	3.1225	0.89541	-1.2845	-1.5817	0.91
9564	*UTY*	2.1672	0.47158	0.09435	-0.14878	0.91
9565	*POU6F1*	4.1259	0.33313	-1.7266	-2.5005	0.91
9566	*MC2R*	1.7503	0.84114	0.14058	-1.7496	0.91
9567	*LOC100129924*	3.3086	0.49297	-1.0705	-2.2117	0.91
9568	*PLA2G6*	2.7606	0.96402	-0.99448	-1.2064	0.91
9569	*C5orf28*	3.5551	0.13475	-0.96031	-3.8624	0.91
9570	*NADSYN1*	4.5737	-0.17566	-1.6689	-1.8098	0.91
9571	*ADRA1B*	2.5884	0.94	-0.79932	-1.4683	0.91
9572	*GCA*	3.0505	0.8264	-1.1487	-2.428	0.91
9573	*CLPSL2*	1.6121	0.79624	0.31975	-0.42859	0.91
9574	*PRIMA1*	2.2834	0.76588	-0.32123	-0.48119	0.91
9575	*DLC1*	1.1956	1.1645	0.36744	-0.74618	0.91
9576	*TBX15*	2.0155	0.43185	0.28018	-1.7602	0.91
9577	*YKT6*	2.4374	0.83128	-0.54126	-1.2806	0.91
9578	*ELAC2*	2.9405	0.84196	-1.0551	-1.3532	0.91
9579	*IGFBP2*	3.5383	-0.08187	-0.72907	-1.3942	0.91
9580	*NHLRC3*	1.821	0.67689	0.22922	-2.4015	0.91
9581	*FOLR2*	2.2408	0.74076	-0.25493	-1.2973	0.91
9582	*RFNG*	2.8939	0.18854	-0.35606	-1.5068	0.91
9583	*IL20RB*	3.5747	-0.34738	-0.50138	-1.1524	0.91
9584	*FCN2*	3.2784	0.30856	-0.86147	-1.0104	0.91
9585	*SLC23A1*	3.3884	-0.23777	-0.42524	-1.2163	0.91
9586	*NUP155*	3.0717	0.19791	-0.54422	-1.1634	0.91
9587	*OR4C3*	2.0548	1.4528	-0.78229	-1.602	0.91
9588	*ACVR1C*	2.9642	0.30269	-0.54219	-1.877	0.91
9589	*TSEN2*	3.4481	-0.20557	-0.51817	-1.2253	0.91
9590	*RPS10-NUDT3*	3.5517	0.81691	-1.6447	-2.4557	0.91
9591	*FANCI*	1.9946	1.3275	-0.59823	-0.71834	0.91
9592	*ACAD11*	2.9239	1.2526	-1.4527	-1.5956	0.91
9593	*SLC22A15*	1.5079	1.3315	-0.11567	-1.4534	0.91
9594	*ZCCHC14*	3.1232	0.45733	-0.85697	-1.0254	0.91
9595	*NSUN3*	2.3656	0.47116	-0.1138	-0.7391	0.91
9596	*CGRRF1*	2.5767	0.48864	-0.34255	-2.254	0.91
9597	*ALDH4A1*	1.7867	0.47172	0.46356	-0.59762	0.91
9598	*UNKL*	2.3262	0.87414	-0.47911	-2.005	0.91
9599	*JAKMIP1*	3.6855	-0.15504	-0.81007	-0.84542	0.91
9600	*RAB3A*	2.4736	0.15938	0.08699	-2.2381	0.91
9601	*ACKR2*	1.1601	0.79894	0.76079	-0.52896	0.91
9602	*SLC39A6*	3.0702	0.93679	-1.2872	-1.8591	0.91
9603	*PWWP2B*	2.1063	0.36161	0.25156	-1.1555	0.91
9604	*AVP*	4.2803	-0.19525	-1.3666	-1.9251	0.91
9605	*RAD54L*	5.1771	-1.1385	-1.3209	-1.8219	0.91
9606	*MRPS24*	2.2514	0.90315	-0.43808	-1.7783	0.91
9607	*BEGAIN*	1.9764	1.8975	-1.1583	-1.4545	0.91

9608	*CCNB2*	1.6471	1.4649	-0.39804	-2.0277	0.90
9609	*KIAA0895L*	3.9357	0.13885	-1.3619	-2.3204	0.90
9610	*ADAM18*	4.782	-0.95154	-1.1202	-1.6216	0.90
9611	*FBXL4*	4.0098	-0.36987	-0.92975	-1.2411	0.90
9612	*DOCK7*	2.9729	-0.08163	-0.18116	-1.714	0.90
9613	*CCDC105*	3.3513	0.36392	-1.0055	-1.2647	0.90
9614	*SUCLG1*	1.9473	0.72975	0.03151	-1.2666	0.90
9615	*STAG2*	2.7656	1.3416	-1.3987	-1.8822	0.90
9616	*VWA7*	3.3248	-0.10704	-0.51002	-1.4168	0.90
9617	*VIT*	1.9805	0.59361	0.13267	-1.8575	0.90
9618	*NR1I3*	2.6513	0.67399	-0.61892	-1.5959	0.90
9619	*IL3RA*	1.367	1.3455	-0.0062	-0.28677	0.90
9620	*TTC4*	3.1644	0.5799	-1.0388	-1.5448	0.90
9621	*CCDC113*	1.4594	1.3327	-0.08675	-0.84457	0.90
9622	*DEFB123*	1.8754	0.51648	0.31323	-0.30045	0.90
9623	*REG1B*	2.1666	1.5901	-1.0516	-1.078	0.90
9624	*CCER2*	2.6548	0.55698	-0.50689	-0.70442	0.90
9625	*LYPD1*	1.3697	0.80123	0.53367	-0.40742	0.90
9626	*HOXC10*	3.1253	0.25015	-0.6714	-0.78229	0.90
9627	*RSPH10B2*	1.9241	0.65427	0.12494	-1.0528	0.90
9628	*ARHGEF26*	1.5436	0.78732	0.37229	-0.42708	0.90
9629	*MAEL*	1.3683	0.73915	0.59519	-1.339	0.90
9630	*BIN3*	3.3333	-0.0248	-0.60604	-1.5268	0.90
9631	*PSMG4*	4.0744	-0.5967	-0.77562	-1.7577	0.90
9632	*BAALC*	3.1463	-0.17129	-0.27423	-1.3364	0.90
9633	*ZNF593*	1.3233	0.7057	0.67131	-0.1288	0.90
9634	*TM9SF1*	2.4594	1.2633	-1.0231	-2.382	0.90
9635	*EFNA1*	2.16	0.27747	0.26202	0.043375	0.90
9636	*FMNL1*	4.1688	-0.21944	-1.25	-1.4214	0.90
9637	*CPEB3*	2.9103	0.3323	-0.5442	-1.4924	0.90
9638	*SPATA22*	1.8732	1.0774	-0.25225	-2.6072	0.90
9639	*OR4E2*	2.3769	0.19975	0.12166	-1.1964	0.90
9640	*MSH4*	1.9458	0.38375	0.36873	-0.75889	0.90
9641	*CHRM3*	1.2977	0.76001	0.64033	-0.12598	0.90
9642	*KRTAP24-1*	1.4741	1.2485	-0.02475	-0.42354	0.90
9643	*NPR3*	3.41	-0.30232	-0.4102	-0.7532	0.90
9644	*ASUN*	1.9264	0.70807	0.06281	-0.43628	0.90
9645	*HECTD2*	3.1029	0.21507	-0.62099	-1.1754	0.90
9646	*RARRES1*	2.002	0.78671	-0.0918	-0.87019	0.90
9647	*KLF2*	2.483	0.50376	-0.28994	-1.7177	0.90
9648	*LSM10*	5.0573	-0.84291	-1.5176	-2.907	0.90
9649	*ARGLU1*	1.7518	0.56676	0.37761	-1.4597	0.90
9650	*GYLTL1B*	3.2046	0.14183	-0.65109	-0.79225	0.90
9651	*OR2G2*	2.7622	0.085857	-0.15357	-0.15385	0.90
9652	*C15orf53*	2.5865	0.40216	-0.29484	-2.641	0.90
9653	*SCAF1*	3.5455	0.83686	-1.6891	-2.5016	0.90

9654	*TIMM17A*	2.0461	0.46952	0.17644	0.09986	0.90
9655	*EMCN*	2.2353	0.9419	-0.48524	-1.589	0.90
9656	*ARHGAP24*	2.2194	0.6295	-0.15699	-1.0426	0.90
9657	*PRDM2*	3.8366	-0.34927	-0.79573	-1.5165	0.90
9658	*RIC3*	1.3613	0.96187	0.36623	-0.19136	0.90
9659	*H2AFJ*	3.985	-0.12176	-1.1754	-1.8646	0.90
9660	*MIR205HG*	3.3323	-0.20105	-0.44353	-0.77159	0.90
9661	*APOBEC3D*	1.9983	0.36925	0.31999	-1.2756	0.90
9662	*ACVR1*	2.0941	0.39435	0.19892	-0.28712	0.90
9663	*ZNF446*	2.6474	0.068356	-0.02864	-0.74774	0.90
9664	*KDM5C*	2.9345	1.0342	-1.2816	-2.4815	0.90
9665	*KIAA1919*	2.3799	0.21235	0.09433	-0.00092	0.90
9666	*PRMT8*	3.2325	0.61905	-1.1651	-1.3785	0.90
9667	*RPL37A*	3.0525	0.060454	-0.42658	-1.5195	0.90
9668	*HGH1*	3.8216	0.027969	-1.1637	-1.4204	0.90
9669	*ZMYND8*	4.4284	-0.05504	-1.6885	-1.7766	0.89
9670	*CXCL1*	4.0697	0.05242	-1.4375	-2.3154	0.89
9671	*TBX20*	2.3788	0.48588	-0.18006	-1.9168	0.89
9672	*ARHGEF39*	4.7226	0.61036	-2.6495	-3.2745	0.89
9673	*SPANXN5*	1.9071	1.2073	-0.43114	-1.554	0.89
9674	*TSC22D3*	1.5916	0.8577	0.23385	-2.0383	0.89
9675	*USP4*	1.7128	0.5995	0.37073	-0.11745	0.89
9676	*SPATA19*	1.8514	0.68622	0.14537	-1.277	0.89
9677	*SLC6A15*	1.7421	1.0848	-0.14427	-1.2628	0.89
9678	*TRMT44*	2.7297	0.57812	-0.62531	-0.81448	0.89
9679	*ANKRD13C*	3.3854	-0.13849	-0.56507	-1.4757	0.89
9680	*FAM216A*	5.097	-0.33156	-2.0837	-2.3876	0.89
9681	*LYZ*	3.0614	0.4506	-0.83027	-1.3851	0.89
9682	*ACACB*	3.1576	0.52808	-1.0041	-2.101	0.89
9683	*AAMP*	2.0526	0.73484	-0.10647	-1.0769	0.89
9684	*GABPB1*	3.8563	-0.18934	-0.98634	-1.9866	0.89
9685	*SEMA3F*	4.807	-0.51363	-1.6137	-2.0466	0.89
9686	*ZNF414*	2.5777	0.50912	-0.40765	-0.91632	0.89
9687	*RBM42*	4.6004	-0.14513	-1.7766	-1.984	0.89
9688	*ITPR3*	1.6531	0.99657	0.02895	-0.50828	0.89
9689	*IFRD2*	1.1841	0.91331	0.58115	-0.97784	0.89
9690	*TADA2A*	1.3559	0.92367	0.39727	-1.9175	0.89
9691	*SVEP1*	2.8294	0.013685	-0.16644	-0.50207	0.89
9692	*SART1*	3.2886	-0.15787	-0.45454	-0.78177	0.89
9693	*STARD9*	4.0167	-0.28822	-1.0529	-1.259	0.89
9694	*FOXR1*	2.3661	0.45259	-0.14362	-0.85564	0.89
9695	*TNKS1BP1*	4.6696	-0.74392	-1.2517	-5.8457	0.89
9696	*PIM3*	2.912	0.29119	-0.52944	-2.092	0.89
9697	*HIST1H2AD*	4.2802	0.050441	-1.6587	-2.1347	0.89
9698	*STAP2*	2.571	0.84298	-0.74308	-1.8272	0.89
9699	*SDC2*	2.6917	0.45188	-0.47304	-1.6525	0.89

9700	*COMMD10*	3.6069	0.39778	-1.3358	-2.5004	0.89
9701	*CPXM2*	2.6442	0.030467	-0.00586	-1.6827	0.89
9702	*DDX60*	1.0506	1.0069	0.61041	-1.5494	0.89
9703	*SMCR8*	3.7247	-0.3548	-0.70202	-1.7517	0.89
9704	*CD99*	2.0377	0.8262	-0.19658	-0.76337	0.89
9705	*PDS5A*	3.0893	0.62099	-1.043	-2.8928	0.89
9706	*CNTRL*	3.6166	-0.34327	-0.60692	-1.5913	0.89
9707	*ZNF25*	4.0658	-0.60845	-0.79147	-1.9065	0.89
9708	*FAM170A*	2.1972	0.32443	0.14408	-1.2111	0.89
9709	*RASSF2*	2.5126	2.0209	-1.8679	-2.6003	0.89
9710	*ZNF385A*	1.9682	0.56191	0.13547	-0.15718	0.89
9711	*WISP3*	1.3031	1.0361	0.3263	-0.12728	0.89
9712	*PRKRIP1*	2.136	0.42233	0.10696	-1.466	0.89
9713	*ABCA12*	3.1933	0.19686	-0.72488	-2.2766	0.89
9714	*DOLK*	3.3967	-0.21348	-0.51805	-1.257	0.89
9715	*STARD10*	4.4022	-0.16927	-1.5684	-1.677	0.89
9716	*SNTN*	3.5899	0.68912	-1.6146	-2.012	0.89
9717	*ZASP*	1.4659	1.0104	0.18807	-0.63866	0.89
9718	*SORBS3*	2.4081	0.7712	-0.51496	-0.89387	0.89
9719	*VEZF1*	1.9293	1.4314	-0.6968	-1.7567	0.89
9720	*PLAC4*	1.9248	0.55632	0.18216	-0.2302	0.89
9721	*CCDC89*	2.3525	0.22074	0.08999	-1.5095	0.89
9722	*GFAP*	2.6825	0.71641	-0.73604	-3.9446	0.89
9723	*FAXDC2*	4.557	-0.44353	-1.4507	-2.8402	0.89
9724	*RACGAP1*	2.055	1.0201	-0.41239	-1.1532	0.89
9725	*ZNF215*	3.0894	0.53844	-0.96632	-1.0528	0.89
9726	*INS*	1.5551	0.99547	0.11075	-0.66604	0.89
9727	*MYO18A*	2.1165	1.1798	-0.63554	-2.0538	0.89
9728	*PAFAH2*	2.1796	1.7229	-1.2427	-1.8817	0.89
9729	*PLA2G7*	1.7246	0.60331	0.33119	-2.5153	0.89
9730	*OR2S2*	1.4951	0.78316	0.38056	-0.59263	0.89
9731	*NSMAF*	3.6145	-0.21666	-0.73911	-1.1193	0.89
9732	*PRKG2*	1.7003	1.2223	-0.26399	-1.8631	0.89
9733	*PHLPP1*	2.1009	0.51115	0.04617	-0.66587	0.89
9734	*HSD17B4*	3.0977	0.72497	-1.165	-1.9624	0.89
9735	*FAM69C*	3.0014	-0.04759	-0.29647	-0.60321	0.89
9736	*AHDC1*	1.3257	0.69063	0.64095	-1.4684	0.89
9737	*KRBOX1*	4.543	0.076234	-1.9622	-2.1334	0.89
9738	*DLG5*	2.4685	1.601	-1.4132	-2.2255	0.89
9739	*EIF4G3*	1.8164	0.74847	0.09132	-0.19311	0.89
9740	*NHSL2*	3.7278	-0.22492	-0.84718	-1.5383	0.89
9741	*RPS10*	2.2249	0.81638	-0.3866	-1.2371	0.88
9742	*C9orf129*	3.5135	-0.25493	-0.60423	-1.1503	0.88
9743	*PLA2G4B*	2.5342	0.80718	-0.68706	-1.4335	0.88
9744	*KISS1R*	3.9989	-0.55833	-0.78651	-1.1553	0.88
9745	*MLIP*	3.0122	1.0961	-1.4553	-1.5212	0.88

9746	*CTAGE15*	1.6051	1.4904	-0.44353	-1.1002	0.88
9747	*GSTM5*	3.2255	-0.17285	-0.40109	-0.86744	0.88
9748	*OXLD1*	4.9087	-0.78959	-1.4683	-4.5653	0.88
9749	*CSF2RA*	2.7532	0.97997	-1.0824	-1.3198	0.88
9750	*GRK5*	2.5244	0.097241	0.02503	-1.2085	0.88
9751	*CHRM4*	2.8355	-0.03116	-0.15831	-0.27982	0.88
9752	*HMHA1*	4.7282	-0.40692	-1.6753	-1.7322	0.88
9753	*TBC1D3B*	2.6881	0.47762	-0.51994	-1.6976	0.88
9754	*SNIP1*	2.7642	0.04324	-0.16242	-0.333	0.88
9755	*OR10C1*	3.0758	-0.12659	-0.3048	-1.197	0.88
9756	*GNG5*	3.9235	-0.27016	-1.0092	-1.7507	0.88
9757	*GABRA3*	4.1405	-0.02008	-1.4767	-2.8316	0.88
9758	*IQCG*	1.4386	1.1146	0.09045	-0.64278	0.88
9759	*CDH13*	3.5217	0.65513	-1.5354	-1.8219	0.88
9760	*ACTL7A*	2.2859	0.39559	-0.04064	-0.5138	0.88
9761	*RTN4RL2*	1.8826	0.84679	-0.09043	-1.043	0.88
9762	*SLC10A4*	3.6772	-0.30237	-0.73591	-1.8804	0.88
9763	*EVX2*	2.5685	0.27344	-0.20338	-1.927	0.88
9764	*MOB3C*	2.4202	0.74607	-0.52795	-0.54155	0.88
9765	*MAPKBP1*	1.686	0.54868	0.40342	-1.2349	0.88
9766	*SSR3*	2.7724	0.28482	-0.41917	-0.47304	0.88
9767	*EIF4E2*	1.9248	0.77026	-0.05738	-5.0141	0.88
9768	*BOD1*	1.3239	0.95622	0.35683	-0.06044	0.88
9769	*SH2D7*	4.1111	-0.68386	-0.79065	-1.5956	0.88
9770	*ARL8A*	4.5501	-0.36792	-1.5458	-2.1404	0.88
9771	*ATP5J2-PTCD1*	2.7837	0.72665	-0.87418	-1.9423	0.88
9772	*TMEM207*	3.164	0.29116	-0.81932	-1.1357	0.88
9773	*PAF1*	4.1906	-0.13627	-1.4189	-1.6597	0.88
9774	*CMTM5*	3.5066	0.063013	-0.93453	-1.3851	0.88
9775	*PAIP2B*	1.8258	0.68725	0.12097	-0.41073	0.88
9776	*RAD21L1*	0.93194	0.85789	0.84366	-0.34187	0.88
9777	*TGFBR3L*	2.2351	0.31766	0.08015	-0.83784	0.88
9778	*OR10G4*	2.7185	0.50465	-0.59028	-0.94733	0.88
9779	*HIST1H2AG*	2.713	0.071886	-0.15203	-0.60368	0.88
9780	*DNASE1L2*	4.191	-0.68193	-0.87768	-0.96379	0.88
9781	*CT47A10*	3.0878	0.035844	-0.49322	-1.4299	0.88
9782	*RNF31*	3.6379	-0.04858	-0.95933	-1.1858	0.88
9783	*FBXO34*	1.7701	0.86996	-0.01116	-0.16415	0.88
9784	*FAM129C*	1.4509	1.2188	-0.04189	-0.39698	0.88
9785	*TESK1*	3.4275	-0.17663	-0.62307	-0.69961	0.88
9786	*ZNF507*	2.8953	0.00844	-0.27596	-2.0602	0.88
9787	*GORASP2*	2.6035	0.47351	-0.44927	-0.55678	0.88
9788	*TMEM249*	4.2199	-0.28605	-1.307	-2.5754	0.88
9789	*C2orf76*	1.4428	1.3878	-0.20378	-1.1263	0.88
9790	*C17orf99*	2.5425	0.52708	-0.44353	-0.45847	0.88
9791	*INSIG1*	5.2149	-0.27719	-2.3118	-3.9349	0.88

9792	*SPATA21*	3.6234	0.20209	-1.2001	-1.8341	0.88
9793	*LYRM7*	3.6227	0.46266	-1.4601	-1.4878	0.88
9794	*ATP2C1*	2.2182	0.77718	-0.37139	-1.5155	0.87
9795	*BCR*	5.3145	0.66019	-3.3519	-3.729	0.87
9796	*HSD11B2*	3.2931	-0.29942	-0.37103	-0.63572	0.87
9797	*FAM175A*	3.0641	0.17706	-0.61899	-1.3663	0.87
9798	*GPLD1*	1.1548	0.91929	0.54787	0.47483	0.87
9799	*NOL12*	3.2849	-0.2804	-0.38349	-1.5022	0.87
9800	*NARF*	1.3501	1.0537	0.21702	-1.9722	0.87
9801	*ENTPD1*	1.5899	0.6071	0.42375	-1.2437	0.87
9802	*DOCK8*	4.0073	0.010651	-1.3976	-2.0895	0.87
9803	*CXorf21*	2.3134	0.27655	0.02949	-0.52712	0.87
9804	*CXorf36*	1.9014	0.54576	0.1719	0.006927	0.87
9805	*ZNF225*	2.4824	0.6576	-0.52149	-0.80946	0.87
9806	*HIST1H2BE*	2.0286	1.4331	-0.84365	-1.6158	0.87
9807	*FOPNL*	2.4363	0.4001	-0.21964	-1.1703	0.87
9808	*NEXN*	2.6489	0.033178	-0.0658	-0.26592	0.87
9809	*GOSR2*	1.2914	1.0718	0.25236	-0.61356	0.87
9810	*SDK1*	1.7173	0.58659	0.31145	0.032177	0.87
9811	*FN1*	2.7382	0.16693	-0.28986	-1.1968	0.87
9812	*TNFRSF8*	1.6849	0.84771	0.08154	-0.27609	0.87
9813	*RPS6KL1*	2.7247	0.052148	-0.16282	-1.2008	0.87
9814	*MAFB*	2.8824	0.32298	-0.59244	-0.62174	0.87
9815	*RPL21*	3.5281	0.15861	-1.0741	-1.6213	0.87
9816	*ILVBL*	2.4234	0.19737	-0.00825	-2.6675	0.87
9817	*ITIH6*	2.3695	0.84709	-0.60445	-1.6383	0.87
9818	*FAM166A*	1.3499	1.1074	0.1539	-0.70558	0.87
9819	*ART3*	3.8323	-0.02511	-1.1964	-1.2936	0.87
9820	*ZNF76*	4.9502	-1.083	-1.2571	-1.5956	0.87
9821	*CENPP*	2.1388	0.91449	-0.44353	-2.2432	0.87
9822	*HEATR5A*	2.5031	0.42059	-0.3146	-1.6803	0.87
9823	*MEI1*	3.9288	-0.40584	-0.91396	-1.1886	0.87
9824	*PLEKHO1*	3.8677	-0.44445	-0.81433	-1.3479	0.87
9825	*DPM1*	3.2133	-0.21484	-0.39043	-1.2388	0.87
9826	*CDH1*	2.5815	0.25825	-0.23215	-1.0841	0.87
9827	*MKKS*	2.2386	0.25294	0.1148	-1.2616	0.87
9828	*LRRN3*	3.3167	-0.01068	-0.70175	-3.6928	0.87
9829	*CYCS*	3.2375	0.74883	-1.3829	-1.5346	0.87
9830	*HARBI1*	1.2631	1.0805	0.25931	0.15924	0.87
9831	*OCM*	3.9387	-0.0426	-1.2935	-2.2165	0.87
9832	*NCMAP*	2.284	0.18114	0.13714	0.098257	0.87
9833	*PRKAA2*	2.1685	1.2567	-0.82326	-1.5073	0.87
9834	*GPR83*	2.3667	0.44838	-0.21317	-0.5183	0.87
9835	*PRODH*	2.3573	0.66202	-0.41835	-1.5837	0.87
9836	*SERPINA10*	3.6313	-0.37106	-0.65949	-0.84743	0.87
9837	*SCNN1G*	4.6133	-0.96222	-1.0508	-1.2365	0.87

9838	*SLC38A5*	1.3729	0.70642	0.52025	-0.16695	0.87
9839	*TMEM173*	1.3663	1.1361	0.09713	-0.043	0.87
9840	*MFSD2A*	2.8331	0.86742	-1.1012	-2.1628	0.87
9841	*TMEM218*	2.6483	1.3428	-1.3918	-1.7074	0.87
9842	*CDC42SE1*	3.7799	-0.36035	-0.82044	-0.93885	0.87
9843	*LRRC38*	1.7635	0.64291	0.19223	-1.4252	0.87
9844	*RGS4*	3.9737	-0.3783	-0.99768	-1.3478	0.87
9845	*KBTBD13*	4.5697	-0.79149	-1.1807	-2.5839	0.87
9846	*SH2D1B*	2.1423	0.71068	-0.25619	-0.98047	0.87
9847	*SYT3*	2.8831	0.29117	-0.57824	-4.3949	0.87
9848	*PLLP*	3.493	-0.20434	-0.69333	-1.348	0.87
9849	*CNIH4*	2.7053	0.81803	-0.92872	-1.0765	0.86
9850	*SMIM2*	3.6726	-0.03406	-1.0443	-1.1797	0.86
9851	*KCNIP3*	2.578	0.3267	-0.31051	-1.4764	0.86
9852	*MSGN1*	3.2892	0.095647	-0.79108	-0.92078	0.86
9853	*ATXN7L1*	2.9706	0.30598	-0.68388	-0.69604	0.86
9854	*GSTP1*	3.6354	-0.30497	-0.73835	-1.4015	0.86
9855	*TFEB*	2.7062	0.42375	-0.53856	-1.6288	0.86
9856	*DUOX1*	2.5654	0.401	-0.37592	-1.5883	0.86
9857	*FAM84B*	3.7305	-0.50221	-0.63826	-1.6208	0.86
9858	*ZBTB11*	3.2475	0.02563	-0.68335	-0.79202	0.86
9859	*NOSTRIN*	3.285	-0.21979	-0.47567	-0.57045	0.86
9860	*PAOX*	2.8796	0.59868	-0.8895	-2.1566	0.86
9861	*TAS2R4*	1.9359	0.3515	0.30081	-0.14778	0.86
9862	*ABCA10*	3.3605	1.0803	-1.8529	-2.1947	0.86
9863	*CDNF*	2.534	0.49741	-0.44353	-0.46763	0.86
9864	*MYL6B*	1.3709	0.67274	0.5439	-0.69891	0.86
9865	*SMARCA5*	2.379	0.95694	-0.74913	-1.5767	0.86
9866	*PODXL2*	3.6666	-0.47696	-0.60307	-1.1095	0.86
9867	*CCR6*	1.9755	0.37588	0.23508	-1.12	0.86
9868	*EPS8L3*	2.2006	1.2407	-0.85564	-2.0383	0.86
9869	*CTPS2*	1.6998	0.57698	0.30863	0.008554	0.86
9870	*SMC6*	1.2851	0.75598	0.54429	-1.175	0.86
9871	*BLOC1S4*	2.2466	0.24738	0.091	-0.0466	0.86
9872	*KIF6*	1.0183	0.96903	0.59762	-0.20621	0.86
9873	*RBM46*	2.2795	0.742	-0.43702	-1.4682	0.86
9874	*ZNF687*	2.9521	0.81498	-1.1826	-1.2797	0.86
9875	*SLCO1C1*	3.2497	1.4558	-2.1217	-3.1051	0.86
9876	*HHLA1*	4.4461	-0.76569	-1.0975	-1.7266	0.86
9877	*BCL2L15*	3.3169	-0.17556	-0.55849	-1.6116	0.86
9878	*MRPS30*	2.5366	0.26145	-0.21532	-1.2146	0.86
9879	*PPM1L*	4.0334	-0.54786	-0.90303	-1.0367	0.86
9880	*LIMS2*	2.761	0.2274	-0.40605	-5.6424	0.86
9881	*TMEM206*	3.1295	0.47755	-1.0248	-1.204	0.86
9882	*ZNF318*	3.1201	0.76713	-1.305	-2.3065	0.86
9883	*TRIO*	2.4125	0.73762	-0.56841	-1.0925	0.86

9884	*MXRA5*	3.2858	-0.23176	-0.47244	-1.5552	0.86
9885	*NXF3*	1.9677	0.81679	-0.20324	-0.27892	0.86
9886	*CEACAM7*	4.6922	-0.79319	-1.3178	-1.3465	0.86
9887	*LTB*	2.9157	1.3866	-1.7215	-2.7042	0.86
9888	*EVA1B*	1.1674	1.0209	0.39248	0.15417	0.86
9889	*AURKC*	2.2398	0.21026	0.13043	-1.7378	0.86
9890	*PLEKHG5*	4.0072	-0.67461	-0.75213	-1.1989	0.86
9891	*USP40*	1.7433	0.49398	0.34277	-1.2411	0.86
9892	*HNRNPCL1*	4.0154	0.063498	-1.4996	-1.7782	0.86
9893	*ADAM23*	2.9232	0.25253	-0.59662	-1.3287	0.86
9894	*LRRC8B*	2.9005	0.10349	-0.42524	-1.7616	0.86
9895	*PRSS54*	1.4071	1.1621	0.00943	-1.6112	0.86
9896	*SLC31A1*	3.8025	-0.48833	-0.73585	-1.7507	0.86
9897	*PIGP*	2.2891	0.31701	-0.02943	-0.70443	0.86
9898	*VPS13B*	2.485	0.55162	-0.46064	-1.7935	0.86
9899	*OR6C70*	2.8888	-0.04416	-0.26898	-0.28308	0.86
9900	*KRT16*	2.7744	0.042576	-0.24142	-0.64458	0.86
9901	*LHFP*	1.4147	1.283	-0.1224	-1.3694	0.86
9902	*ZMAT1*	1.4909	0.64084	0.44331	0.31041	0.86
9903	*REPS2*	1.1829	1.1175	0.27293	-2.3145	0.86
9904	*NDEL1*	4.6205	-0.42593	-1.6213	-1.9734	0.86
9905	*STK38L*	2.6739	0.15359	-0.25452	-1.4946	0.86
9906	*ZNF385B*	1.6999	0.55449	0.31824	-0.30045	0.86
9907	*HERC5*	1.2969	1.1068	0.16852	-0.37417	0.86
9908	*TMSB4X*	3.6734	0.4482	-1.5499	-1.8761	0.86
9909	*KIAA1467*	2.4629	1.1032	-0.99508	-1.9227	0.86
9910	*PAWR*	1.5895	0.54154	0.43912	0.088767	0.86
9911	*ARHGAP10*	3.2248	0.17199	-0.82833	-1.7911	0.86
9912	*MAFK*	3.5131	-0.07094	-0.87395	-2.2676	0.86
9913	*DQX1*	2.1166	0.76393	-0.31244	-0.37791	0.86
9914	*APEH*	1.8701	0.50306	0.19474	-0.60258	0.86
9915	*SPNS1*	2.0238	0.8659	-0.32193	-1.5556	0.86
9916	*CDRT4*	1.2563	0.66736	0.64372	0.148	0.86
9917	*MSRB1*	2.4654	0.71461	-0.61264	-0.97252	0.86
9918	*ERCC4*	3.5164	-0.15394	-0.79535	-1.2399	0.86
9919	*FAM26F*	4.5457	-0.80581	-1.173	-1.1964	0.86
9920	*BACE2*	1.3339	0.79061	0.4418	-2.0752	0.86
9921	*ORAI2*	1.6237	0.83755	0.10504	0.021712	0.86
9922	*PARP12*	4.4271	-0.45615	-1.4055	-1.6447	0.86
9923	*GRIA2*	1.9187	1.5648	-0.91842	-1.5297	0.86
9924	*SEPT8*	2.0823	1.0832	-0.60174	-0.70732	0.85
9925	*ESD*	2.2809	0.60652	-0.32428	-0.85799	0.85
9926	*FAM161A*	1.894	0.89296	-0.22455	-1.3644	0.85
9927	*ATP2B4*	3.3216	0.4726	-1.2329	-1.3496	0.85
9928	*SLC6A16*	2.324	1.3809	-1.1442	-1.7247	0.85
9929	*MSANTD2*	1.702	0.93187	-0.07341	-1.7068	0.85

9930	*HBS1L*	3.1096	0.088179	-0.63753	-0.77616	0.85
9931	*ATP6V1E2*	2.5748	0.13558	-0.15145	-1.4123	0.85
9932	*AGAP6*	1.7609	1.6976	-0.89988	-2.9678	0.85
9933	*FITM1*	3.2916	-0.15596	-0.57704	-1.2867	0.85
9934	*CREBRF*	3.5455	0.41089	-1.3978	-1.9709	0.85
9935	*OR1Q1*	2.4986	0.30139	-0.24142	-0.98486	0.85
9936	*ASAH2*	2.9102	0.031866	-0.38472	-1.0259	0.85
9937	*PRR23C*	1.0161	0.98392	0.55641	-1.0475	0.85
9938	*ATP6V1E1*	2.6758	0.84514	-0.96492	-1.9065	0.85
9939	*TMSB10*	2.4503	1.3902	-1.2852	-1.9727	0.85
9940	*NRIP2*	1.372	0.61455	0.56796	0.25771	0.85
9941	*ZNF547*	2.3073	0.67866	-0.43202	-1.5611	0.85
9942	*TRMT10C*	3.5001	0.35283	-1.2995	-2.7191	0.85
9943	*C16orf89*	1.8282	0.42359	0.30019	-1.8528	0.85
9944	*NMRK2*	1.405	1.2706	-0.12397	-0.78345	0.85
9945	*SCRN1*	3.4312	0.17562	-1.0553	-1.3857	0.85
9946	*MYH1*	4.308	0.25771	-2.0146	-2.2712	0.85
9947	*EPHB2*	1.9247	0.93092	-0.30487	-1.3495	0.85
9948	*CXCL3*	4.1159	-0.52458	-1.0413	-1.1991	0.85
9949	*COQ2*	2.5174	0.9242	-0.89167	-1.0231	0.85
9950	*CACNB4*	4.0801	0.047718	-1.578	-2.6376	0.85
9951	*GARNL3*	2.4939	0.092509	-0.0378	-0.84158	0.85
9952	*LARP4B*	2.8387	0.052562	-0.34356	-0.42609	0.85
9953	*C3orf35*	1.7584	1.4637	-0.67472	-1.1507	0.85
9954	*ABCD4*	3.7877	0.44439	-1.6848	-1.7364	0.85
9955	*HELB*	4.3402	-0.47353	-1.3198	-1.8002	0.85
9956	*GLI1*	2.287	0.17614	0.08342	-0.0907	0.85
9957	*EFNA5*	4.1573	0.043375	-1.6561	-2.3046	0.85
9958	*EML4*	3.9111	0.039836	-1.4064	-1.5651	0.85
9959	*MPPED1*	2.3015	0.50578	-0.26429	-0.59455	0.85
9960	*NECAP2*	4.1926	-0.57448	-1.0775	-1.8909	0.85
9961	*CLTCL1*	3.7542	-0.12995	-1.084	-2.7941	0.85
9962	*HOXD12*	3.0285	-0.01188	-0.47665	-1.0613	0.85
9963	*ZBTB3*	2.8375	0.81004	-1.1085	-2.058	0.85
9964	*PTK2B*	2.0127	0.61518	-0.08915	-1.0551	0.85
9965	*NPR1*	2.0479	0.71182	-0.22106	-0.7331	0.85
9966	*DEPDC1B*	2.436	0.17071	-0.06816	-1.584	0.85
9967	*UBL5*	2.5948	0.59529	-0.65184	-2.2613	0.85
9968	*ITPRIP*	3.4011	0.068986	-0.93294	-1.4878	0.85
9969	*ZNF280C*	3.5179	-0.25608	-0.72473	-1.7073	0.85
9970	*NHS*	1.5801	0.62998	0.32673	-2.8398	0.85
9971	*RAPGEF5*	2.3335	0.11143	0.09132	-1.1387	0.85
9972	*ZNF440*	1.7945	1.0942	-0.35292	-0.40749	0.85
9973	*PRKD1*	3.1886	0.070081	-0.72324	-1.1768	0.85
9974	*MCTP2*	2.938	0.5355	-0.94052	-1.3729	0.84
9975	*SIGIRR*	2.432	0.40537	-0.30564	-0.57001	0.84

9976	*NOTCH2*	3.0952	-0.07692	-0.48707	-1.6049	0.84
9977	*SERPINB12*	1.9066	0.49406	0.1304	-1.2951	0.84
9978	*BMP2K*	1.2865	0.7839	0.46044	-0.34451	0.84
9979	*BBIP1*	3.8575	-0.53069	-0.79641	-1.0951	0.84
9980	*TFEC*	2.133	0.3849	0.01243	-0.2704	0.84
9981	*SLC9B1*	2.649	0.56059	-0.67934	-1.3526	0.84
9982	*TULP3*	2.7701	1.6686	-1.9086	-2.4481	0.84
9983	*ZNF562*	1.7724	0.39643	0.36048	-0.34886	0.84
9984	*GATA2*	3.1228	0.106	-0.69955	-2.0949	0.84
9985	*DLG1*	2.1036	0.55641	-0.1313	-0.95933	0.84
9986	*MRPL10*	2.801	0.14671	-0.41967	-2.0548	0.84
9987	*PLTP*	3.7775	-0.12428	-1.1263	-1.7979	0.84
9988	*PTPRG*	4.0858	-0.37888	-1.181	-1.2666	0.84
9989	*C8B*	2.6195	0.12739	-0.22181	-0.68095	0.84
9990	*SLC38A11*	2.5987	0.36093	-0.43488	-0.83459	0.84
9991	*GABRA1*	1.9245	0.64799	-0.04796	-1.3078	0.84
9992	*ZNF619*	2.4797	0.62365	-0.5795	-0.90757	0.84
9993	*HSPH1*	3.8595	0.19374	-1.5297	-2.1445	0.84
9994	*SEPT10*	3.8595	-0.29445	-1.043	-1.3353	0.84
9995	*HLA-DPA1*	3.4066	0.32753	-1.2125	-1.2876	0.84
9996	*B4GALT7*	2.0334	0.71104	-0.22302	-1.7577	0.84
9997	*HAVCR1*	2.28	0.3605	-0.1198	-0.21407	0.84
9998	*ZNF503*	3.7196	-0.0164	-1.1834	-1.8382	0.84
9999	*OR2T3*	2.7909	0.12142	-0.3931	-0.69961	0.84
10000	*CAMP*	2.9027	-0.0083	-0.37612	-0.45185	0.84
10001	*GPAA1*	3.0957	-0.15139	-0.42652	-1.1017	0.84
10002	*GOLT1A*	3.1278	0.44901	-1.0598	-2.0095	0.84
10003	*RBPMS2*	3.3547	-0.39433	-0.44353	-0.49824	0.84
10004	*GYG2*	4.696	-0.95321	-1.2261	-2.1004	0.84
10005	*UBE2Q2*	2.0449	1.1893	-0.71753	-1.1726	0.84
10006	*ST8SIA3*	3.2727	0.025891	-0.78295	-2.5127	0.84
10007	*ANKRD26*	3.1008	0.33778	-0.92326	-1.7618	0.84
10008	*RRP7A*	3.5835	-0.35891	-0.71009	-2.3023	0.84
10009	*OR5M3*	4.4317	0.13096	-2.0483	-3.4283	0.84
10010	*BARD1*	1.0483	0.96611	0.4979	0.22092	0.84
10011	*RXRG*	3.1663	0.84519	-1.4997	-1.8323	0.84
10012	*PAPD4*	2.5791	-0.00357	-0.06407	-0.66107	0.84
10013	*SMARCB1*	3.3989	-0.10473	-0.78279	-2.3235	0.84
10014	*CYP2F1*	3.2029	-0.32666	-0.36583	-1.4856	0.84
10015	*DGCR14*	3.6348	0.26867	-1.3945	-1.8291	0.84
10016	*C5AR2*	1.6331	1.1589	-0.28335	-0.55284	0.84
10017	*SYTL4*	4.2594	-0.45529	-1.296	-2.1648	0.84
10018	*MFSD12*	4.011	-0.69915	-0.8038	-1.4998	0.84
10019	*ZNF772*	1.2278	0.91479	0.36479	-0.41359	0.84
10020	*UBE2L6*	1.7726	1.0961	-0.36266	-0.96962	0.84
10021	*TAS2R46*	3.9737	-0.17663	-1.2912	-1.4969	0.84

10022	*YIPF6*	3.2023	0.32065	-1.0178	-1.6251	0.84
10023	*IQCH*	1.2658	0.82411	0.41519	-0.45707	0.84
10024	*NOD1*	3.3645	0.88961	-1.7493	-1.8046	0.83
10025	*HSPA9*	3.3649	0.089583	-0.94989	-1.6789	0.83
10026	*PIGK*	4.157	-0.03224	-1.6208	-2.0351	0.83
10027	*CACNA1C*	2.4287	0.59865	-0.52359	-1.7438	0.83
10028	*SCAMP4*	1.5741	1.1166	-0.18707	-2.4405	0.83
10029	*ADAM9*	1.6095	1.2579	-0.36401	-1.9284	0.83
10030	*CD207*	1.8627	0.57942	0.05962	-1.3371	0.83
10031	*RARS*	2.7876	0.53256	-0.81932	-1.1685	0.83
10032	*CABP2*	2.1037	1.4207	-1.0248	-1.8636	0.83
10033	*SIPA1L3*	4.9852	-1.0054	-1.4809	-1.5965	0.83
10034	*OR8D4*	3.0847	0.092413	-0.67868	-1.9621	0.83
10035	*TDG*	3.892	0.064489	-1.4584	-2.7784	0.83
10036	*C8orf76*	2.0447	0.31401	0.13862	0.12728	0.83
10037	*DPY19L1*	1.5727	0.90263	0.0205	-2.1956	0.83
10038	*RHBDD1*	3.2352	-0.08265	-0.65758	-1.2618	0.83
10039	*VGLL2*	2.6159	0.76772	-0.8897	-1.6469	0.83
10040	*ADAMTS7*	1.3991	0.80653	0.28742	-0.3271	0.83
10041	*TNFRSF19*	1.492	0.76676	0.23339	-1.1511	0.83
10042	*MARCH11*	0.96981	0.77718	0.74484	0.54726	0.83
10043	*FXYD1*	1.2101	0.85322	0.42813	-0.44353	0.83
10044	*FAM13B*	2.5105	0.46439	-0.48368	-2.2866	0.83
10045	*GALNT16*	3.5257	0.0077	-1.0426	-1.507	0.83
10046	*GDF10*	1.7193	1.5835	-0.81264	-1.4852	0.83
10047	*RABGAP1L*	2.6345	0.15379	-0.29862	-1.3426	0.83
10048	*OR11H4*	3.1758	0.17593	-0.86229	-1.371	0.83
10049	*DUSP23*	1.9567	0.39128	0.14083	-0.14716	0.83
10050	*DENND3*	2.3873	1.157	-1.0563	-1.5962	0.83
10051	*ISOC1*	2.6303	0.13279	-0.27522	-1.5273	0.83
10052	*LACE1*	1.9758	0.30138	0.21066	-0.85617	0.83
10053	*STON1*	2.4011	0.082463	0.00424	-0.31299	0.83
10054	*CD46*	1.3128	1.139	0.036	-1.12	0.83
10055	*ZNF8*	1.7849	0.49953	0.20314	0.006651	0.83
10056	*ITLN2*	3.8768	-0.2456	-1.1437	-2.555	0.83
10057	*SESN2*	2.054	1.1527	-0.71935	-0.96791	0.83
10058	*SCN2B*	2.6625	0.77231	-0.94747	-4.9483	0.83
10059	*ZNF226*	2.101	0.7244	-0.33844	-3.3058	0.83
10060	*OR2T4*	2.7921	0.80432	-1.1098	-2.3461	0.83
10061	*NEDD9*	3.4283	-0.42671	-0.51549	-2.0629	0.83
10062	*SCP2*	1.8141	0.71125	-0.03944	-0.56507	0.83
10063	*CAMSAP2*	3.9425	-0.54427	-0.91234	-1.8443	0.83
10064	*TMC8*	4.3549	-0.80823	-1.0613	-1.8751	0.83
10065	*ANGEL2*	1.5773	0.54151	0.36641	-1.4824	0.83
10066	*TCTN1*	5.0108	-1.208	-1.3183	-2.5125	0.83
10067	*OPRD1*	3.7579	-0.25794	-1.016	-1.94	0.83

10068	*ADAT1*	0.87679	0.82264	0.78393	-1.7061	0.83
10069	*RNF10*	4.3298	-0.44353	-1.403	-2.26	0.83
10070	*SMN2*	3.7169	-0.30753	-0.92781	-1.2743	0.83
10071	*MRPL14*	4.7705	-0.92486	-1.3643	-2.012	0.83
10072	*KCNS1*	3.4914	0.31994	-1.33	-2.5754	0.83
10073	*MYL5*	2.8572	0.12226	-0.49818	-0.70057	0.83
10074	*COL6A5*	2.9161	-0.15439	-0.28058	-0.48118	0.83
10075	*OGFOD3*	1.3533	1.1495	-0.02187	-0.08446	0.83
10076	*UBFD1*	4.5715	-0.46205	-1.6288	-2.2343	0.83
10077	*ACADM*	1.6998	0.6201	0.16025	-1.765	0.83
10078	*IRF2BPL*	2.3154	0.097924	0.06487	-0.07436	0.83
10079	*CNTD1*	3.771	-0.53009	-0.76371	-1.2908	0.83
10080	*FGFR3*	1.5139	1.2629	-0.30038	-0.59643	0.83
10081	*UBAP2*	1.3709	0.58003	0.52482	-1.3407	0.83
10082	*SEMA6A*	2.114	0.241	0.12019	-0.2765	0.83
10083	*SLC29A4*	1.6982	0.45311	0.32349	0.10037	0.82
10084	*PLA2G2E*	1.9677	1.9302	-1.4234	-2.586	0.82
10085	*DUOXA2*	4.3754	-0.88873	-1.0129	-1.0796	0.82
10086	*TTLL10*	1.1534	1.0462	0.27327	-0.33704	0.82
10087	*C1QTNF2*	3.1353	-0.19532	-0.46731	-1.9442	0.82
10088	*C1orf61*	1.1894	1.1018	0.18144	-0.37557	0.82
10089	*C7orf62*	2.4753	0.21608	-0.21887	-0.40714	0.82
10090	*IMPG1*	3.0397	0.015765	-0.58334	-1.2198	0.82
10091	*ZFPM2*	2.3111	0.13801	0.02287	-1.7665	0.82
10092	*ISL2*	2.4901	0.42035	-0.43946	-0.5718	0.82
10093	*TLR6*	2.7556	0.93531	-1.2201	-2.6567	0.82
10094	*ZC3H12B*	1.4572	0.76694	0.24637	-2.1219	0.82
10095	*BRIX1*	3.6212	-0.03006	-1.1207	-2.2167	0.82
10096	*ZNF791*	1.373	1.0167	0.08041	-1.2926	0.82
10097	*CLECL1*	3.1408	-0.28559	-0.38529	-1.8816	0.82
10098	*KHDRBS2*	1.8803	0.40192	0.18761	-1.7911	0.82
10099	*DAZ2*	2.3901	0.87663	-0.79798	-1.7061	0.82
10100	*SLC35E3*	2.625	1.1303	-1.2867	-1.6618	0.82
10101	*OR8D1*	1.1824	0.69377	0.59214	-0.0937	0.82
10102	*ROR1*	3.0823	0.7361	-1.3501	-1.9341	0.82
10103	*DOCK10*	4.6049	-0.78229	-1.3567	-1.8246	0.82
10104	*OR5B12*	3.5923	-0.22653	-0.90003	-1.0425	0.82
10105	*CSNK1D*	3.6193	-0.26714	-0.88661	-1.2517	0.82
10106	*GPR50*	2.7459	0.64483	-0.92613	-1.0273	0.82
10107	*NXT1*	4.0477	-0.63406	-0.94912	-1.4255	0.82
10108	*TTLL4*	3.3122	-0.03081	-0.817	-1.4566	0.82
10109	*WRN*	3.4161	0.014271	-0.96632	-1.7757	0.82
10110	*ZNF343*	2.6403	0.26595	-0.44353	-0.63452	0.82
10111	*ZNF674*	1.6604	0.7476	0.05256	-0.06618	0.82
10112	*RPL35*	1.9076	0.40349	0.1486	-1.384	0.82
10113	*ZNF768*	3.6385	-0.41139	-0.76755	-1.4811	0.82

10114	*BMPER*	2.3231	1.1987	-1.0623	-1.6648	0.82
10115	*MAP3K13*	3.0764	0.16733	-0.78439	-0.93107	0.82
10116	*EHD1*	4.7912	-0.93592	-1.3961	-1.8645	0.82
10117	*PLGLB2*	1.1261	0.71129	0.62128	-1.72	0.82
10118	*PCBP1*	2.6168	0.2171	-0.37602	-1.1587	0.82
10119	*SYAP1*	2.1559	1.1167	-0.81541	-1.1812	0.82
10120	*SPATC1*	2.6064	0.46514	-0.61467	-0.67846	0.82
10121	*KATNAL1*	2.2077	0.64807	-0.39997	-0.40398	0.82
10122	*MC4R*	3.548	-0.43674	-0.65553	-2.5103	0.82
10123	*FAM149B1*	4.9997	-1.0372	-1.5077	-1.8966	0.82
10124	*RABEP1*	2.9816	0.52282	-1.0497	-1.455	0.82
10125	*C3orf70*	4.7884	-0.93287	-1.4031	-2.4955	0.82
10126	*PAQR5*	4.0369	0.098257	-1.6836	-2.6463	0.82
10127	*DDIT3*	1.6964	0.97771	-0.2227	-0.69815	0.82
10128	*MCCC1*	1.3955	0.99784	0.05634	-0.43478	0.82
10129	*SH3PXD2A*	2.704	0.78829	-1.0435	-1.4402	0.82
10130	*MXD4*	2.7435	0.45984	-0.75472	-1.6913	0.82
10131	*CRYGS*	2.9503	0.72805	-1.2302	-2.0818	0.82
10132	*ASPDH*	1.7897	0.75012	-0.0925	-0.5898	0.82
10133	*FSBP*	1.7212	0.43453	0.29115	0.076188	0.82
10134	*GABRG3*	4.8881	-1.1974	-1.244	-1.6597	0.82
10135	*KPRP*	1.5488	0.58812	0.30864	-0.53911	0.82
10136	*RRAD*	1.99	1.0721	-0.61737	-1.5634	0.81
10137	*DERL3*	1.748	1.3235	-0.62814	-0.81101	0.81
10138	*GPATCH2L*	2.4009	0.63732	-0.5961	-1.1729	0.81
10139	*PPP1R42*	2.1358	0.35781	-0.05252	-2.3857	0.81
10140	*TCERG1*	2.4719	0.78652	-0.81757	-2.0126	0.81
10141	*GLB1*	1.6773	0.69338	0.0693	-0.17549	0.81
10142	*SEL1L3*	2.6309	0.49202	-0.68303	-1.9827	0.81
10143	*SLCO1B1*	3.8939	-0.5656	-0.88843	-1.0724	0.81
10144	*STX1B*	2.8858	0.46392	-0.90991	-1.6817	0.81
10145	*GRXCR1*	1.9572	0.36875	0.1134	-1.677	0.81
10146	*DHRS2*	4.4118	0.19804	-2.1714	-2.3461	0.81
10147	*ADGRF4*	1.3873	0.62252	0.42783	-0.48085	0.81
10148	*PTX4*	2.1314	1.227	-0.92097	-1.213	0.81
10149	*ATP5J*	5.5467	-1.0145	-2.0953	-2.1217	0.81
10150	*MAPK11*	3.9487	0.33438	-1.8462	-2.1151	0.81
10151	*C11orf52*	1.4165	1.3186	-0.29861	-0.83749	0.81
10152	*DYX1C1*	3.0314	0.04945	-0.64441	-0.67629	0.81
10153	*SGK494*	4.5489	-0.87673	-1.236	-1.2729	0.81
10154	*TBC1D7*	2.3752	0.88995	-0.82921	-2.2537	0.81
10155	*KTI12*	1.6313	1.1136	-0.30975	-1.5722	0.81
10156	*IGFALS*	2.4121	0.37386	-0.35098	-1.3542	0.81
10157	*LCN9*	3.2796	0.69659	-1.5425	-1.9112	0.81
10158	*CYB5R4*	3.1944	-0.27076	-0.49095	-2.5878	0.81
10159	*EDNRA*	1.2108	1.0111	0.20954	-0.96572	0.81

10160	*TCF7L2*	2.9889	-0.1376	-0.41994	-0.70705	0.81
10161	*LMCD1*	3.3554	-0.41643	-0.50804	-2.005	0.81
10162	*PTPRA*	1.2039	0.80114	0.42522	-1.5911	0.81
10163	*ZNF236*	3.5884	-0.31164	-0.84652	-2.2508	0.81
10164	*DPYSL3*	1.0441	0.90552	0.47937	-1.5212	0.81
10165	*WDR54*	2.0011	0.55139	-0.12414	-0.14312	0.81
10166	*C1orf233*	2.2331	1.0457	-0.85194	-1.117	0.81
10167	*SSTR4*	1.0047	0.79948	0.6225	-1.2934	0.81
10168	*ACTR1B*	2.3007	1.988	-1.8623	-2.0181	0.81
10169	*FOSB*	3.8846	0.063662	-1.5225	-1.8908	0.81
10170	*XRCC6BP1*	2.6581	-0.04064	-0.19179	-1.3579	0.81
10171	*FZD7*	1.7185	0.69837	0.00877	-1.7068	0.81
10172	*ROPN1*	3.9473	0.63392	-2.1566	-2.7063	0.81
10173	*TDRP*	3.2093	0.32948	-1.1145	-1.3111	0.81
10174	*KAZALD1*	1.8671	0.77261	-0.21587	-1.7044	0.81
10175	*ARHGEF37*	4.6497	-0.46721	-1.7591	-2.2214	0.81
10176	*SERINC2*	2.7872	-0.03697	-0.3271	-2.4785	0.81
10177	*NDUFAF2*	2.3166	0.59545	-0.48916	-0.91343	0.81
10178	*MYL2*	2.2955	0.44138	-0.31411	-0.35896	0.81
10179	*DSE*	4.43	-0.79711	-1.2109	-1.7956	0.81
10180	*PARVG*	2.6276	0.41381	-0.61967	-1.6547	0.81
10181	*FILIP1L*	2.764	-0.09774	-0.24493	-0.93696	0.81
10182	*SNX3*	3.0324	-0.27219	-0.33922	-0.45223	0.81
10183	*UROC1*	1.9702	0.28838	0.16177	-1.7512	0.81
10184	*RPAP3*	4.9736	-1.1558	-1.3985	-1.6891	0.81
10185	*GALNT12*	2.3242	0.24021	-0.14558	-0.7819	0.81
10186	*C12orf80*	3.5988	0.090452	-1.2705	-1.6072	0.81
10187	*MTRF1L*	3.1189	-0.00826	-0.69199	-1.5956	0.81
10188	*CACNG4*	1.8754	1.0862	-0.54329	-1.5959	0.81
10189	*CCDC84*	3.1957	0.34155	-1.1196	-1.3686	0.81
10190	*AKAP1*	4.0021	0.23099	-1.8157	-1.8872	0.81
10191	*SMOC1*	2.1973	0.2159	0.00418	-0.11018	0.81
10192	*FAR1*	2.9742	0.78246	-1.3403	-1.3542	0.81
10193	*HIPK3*	4.4867	-0.96878	-1.1016	-1.8938	0.81
10194	*CELF5*	2.4087	0.40424	-0.39731	-1.095	0.81
10195	*DGCR2*	1.7535	1.5061	-0.84411	-1.5724	0.81
10196	*OR10G7*	3.4273	-0.04795	-0.9644	-1.3836	0.80
10197	*PARP9*	1.7971	0.90587	-0.28915	-2.0952	0.80
10198	*MCM10*	1.6991	0.4543	0.26031	-2.3243	0.80
10199	*PRSS58*	1.8904	0.954	-0.43077	-2.312	0.80
10200	*PROM1*	3.938	-0.15715	-1.3686	-1.8462	0.80
10201	*IL36A*	2.7109	0.88918	-1.1879	-2.072	0.80
10202	*EXOC3L2*	2.7345	0.26387	-0.58691	-0.77848	0.80
10203	*ITGA7*	2.1859	0.50484	-0.27941	-1.8859	0.80
10204	*PNP*	2.4407	0.36257	-0.39214	-1.0914	0.80
10205	*PLA2G1B*	2.1633	1.0756	-0.82921	-1.7076	0.80

10206	*SERTAD2*	0.92033	0.79291	0.69449	-1.2985	0.80
10207	*TRIM49C*	1.6213	0.63529	0.15108	-0.79057	0.80
10208	*DMKN*	3.0016	-0.24442	-0.35015	-1.1836	0.80
10209	*TRA2A*	3.8633	-0.67458	-0.78229	-1.0564	0.80
10210	*ECD*	3.052	-0.07232	-0.57397	-1.3903	0.80
10211	*BCL2L10*	3.3547	-0.1309	-0.81943	-1.122	0.80
10212	*C2orf78*	3.1157	-0.33821	-0.37335	-0.49056	0.80
10213	*NWD2*	3.0626	-0.20771	-0.45122	-2.4901	0.80
10214	*UCKL1*	0.98949	0.89401	0.51997	-0.26267	0.80
10215	*NNMT*	3.9111	-0.70002	-0.80847	-1.7279	0.80
10216	*PXDC1*	3.457	0.11845	-1.173	-1.2703	0.80
10217	*RNF180*	2.3375	0.50115	-0.43706	-1.6684	0.80
10218	*HIVEP2*	4.2542	-0.20023	-1.6525	-2.1357	0.80
10219	*DPP9*	3.7134	0.24439	-1.5568	-1.5724	0.80
10220	*CDKN2AIPNL*	2.7322	0.26787	-0.59987	-0.80599	0.80
10221	*ZCCHC4*	2.4241	0.040889	-0.0648	-1.0059	0.80
10222	*YTHDC1*	1.8067	0.5365	0.0566	-1.3593	0.80
10223	*SGSM2*	2.5716	0.15656	-0.32842	-1.3572	0.80
10224	*HEYL*	1.1293	1.0951	0.17443	0.11456	0.80
10225	*GPR27*	2.9498	0.45695	-1.011	-1.1043	0.80
10226	*JAM2*	2.6734	0.1087	-0.3866	-0.44313	0.80
10227	*FADS1*	1.764	0.37182	0.25944	-1.9259	0.80
10228	*FAIM3*	3.4429	-0.1931	-0.85508	-1.7911	0.80
10229	*HSD3B1*	3.591	-0.18235	-1.0142	-2.7171	0.80
10230	*KLHDC3*	3.3483	0.10131	-1.0553	-1.9567	0.80
10231	*OGFOD2*	2.9211	0.42976	-0.95749	-1.1305	0.80
10232	*MYO5C*	1.4875	0.55717	0.34868	-1.0528	0.80
10233	*C4orf27*	1.2598	0.59737	0.53514	-0.59711	0.80
10234	*SETX*	1.3645	1.3359	-0.30811	-2.4338	0.80
10235	*CAPN1*	2.4362	0.82342	-0.86756	-2.1921	0.80
10236	*FBXO15*	2.0406	0.64946	-0.2982	-1.0765	0.80
10237	*SOX30*	2.5254	0.33086	-0.46441	-1.7718	0.80
10238	*TMEM170A*	3.4879	-0.18864	-0.90791	-1.7112	0.80
10239	*LDB2*	1.9085	0.53479	-0.0534	-1.0558	0.80
10240	*TOM1L2*	2.498	0.072685	-0.1815	-1.5862	0.80
10241	*FOXJ2*	2.8448	-0.12456	-0.33191	-1.2867	0.80
10242	*F7*	2.8334	-0.02163	-0.42369	-0.87128	0.80
10243	*C14orf142*	2.4676	0.9346	-1.0158	-1.5568	0.80
10244	*SH3RF1*	3.4191	-0.30692	-0.72666	-1.4299	0.80
10245	*UBE2A*	1.4854	1.248	-0.34814	-0.44353	0.80
10246	*SCUBE1*	3.8755	-0.70945	-0.78236	-1.7585	0.79
10247	*RTTN*	2.9798	0.07636	-0.67292	-0.86412	0.79
10248	*AIF1*	2.6678	0.4863	-0.77144	-1.1805	0.79
10249	*CTDSPL*	4.6434	-0.34882	-1.9123	-2.0196	0.79
10250	*OR51B6*	3.0722	-0.20283	-0.48725	-0.54741	0.79
10251	*RIMS2*	5.6746	-0.93107	-2.3615	-2.5006	0.79

10252	*HRC*	2.3755	0.37028	-0.36451	-1.2662	0.79
10253	*BPTF*	1.7327	0.95951	-0.31109	-0.68416	0.79
10254	*DCP1B*	2.4019	0.88385	-0.90606	-2.1151	0.79
10255	*RASIP1*	2.1713	0.16197	0.04624	-2.0326	0.79
10256	*USP38*	3.0617	-0.31535	-0.36724	-0.44353	0.79
10257	*CD40LG*	4.1433	-0.47929	-1.2852	-3.4111	0.79
10258	*DHX32*	2.7103	0.57743	-0.90933	-1.8352	0.79
10259	*MCRS1*	1.2928	0.95876	0.12623	-0.65489	0.79
10260	*PGM5*	3.5085	0.014683	-1.1456	-1.8238	0.79
10261	*SLC37A1*	0.94095	0.87752	0.55809	-0.28633	0.79
10262	*CBWD5*	2.233	1.1794	-1.0359	-1.1479	0.79
10263	*ANKRD36*	3.0343	-0.3129	-0.34496	-0.50283	0.79
10264	*PLAC8*	1.3329	0.74716	0.29622	-0.7167	0.79
10265	*LHFPL1*	3.7187	0.30874	-1.6524	-2.5754	0.79
10266	*PROP1*	1.4879	0.9652	-0.07947	-0.15667	0.79
10267	*SLC29A3*	2.3327	0.58116	-0.54402	-0.82472	0.79
10268	*TSPAN12*	3.0537	-0.3077	-0.37641	-0.92427	0.79
10269	*HSDL1*	3.2658	-0.00278	-0.89392	-1.0589	0.79
10270	*PUS7L*	2.219	0.35402	-0.20552	-1.9054	0.79
10271	*ANKRD22*	1.6598	0.85048	-0.14309	-1.7526	0.79
10272	*NFU1*	2.6176	0.21699	-0.46759	-1.1401	0.79
10273	*TOMM34*	1.2201	0.79518	0.35163	-1.9099	0.79
10274	*FCGR1A*	3.0456	0.23395	-0.91283	-2.1947	0.79
10275	*OR7C1*	1.2928	1.2494	-0.17577	-0.54274	0.79
10276	*ZKSCAN8*	1.6238	1.394	-0.65178	-1.3382	0.79
10277	*CS*	3.9581	-0.76973	-0.82311	-1.1205	0.79
10278	*RAP1B*	4.989	-1.1856	-1.4388	-2.3294	0.79
10279	*PDE7B*	1.8563	0.91726	-0.40938	-0.58894	0.79
10280	*SLC35G5*	3.4101	-0.25892	-0.78812	-1.785	0.79
10281	*KRT3*	2.1603	0.28586	-0.08354	-0.25981	0.79
10282	*WISP1*	3.9115	-0.21146	-1.3379	-1.6325	0.79
10283	*OR6S1*	3.0853	0.42056	-1.1438	-2.0099	0.79
10284	*COX5B*	2.6825	0.25176	-0.57241	-1.1487	0.79
10285	*STK16*	2.0771	0.49312	-0.20848	-1.8538	0.79
10286	*SLC50A1*	3.3863	-0.4636	-0.56098	-1.2662	0.79
10287	*VWC2*	1.3414	1.2012	-0.18093	-0.50246	0.79
10288	*DTYMK*	1.5363	1.5131	-0.68804	-0.69008	0.79
10289	*RPGRIP1L*	1.5775	0.53279	0.24924	-0.14685	0.79
10290	*TAS2R7*	1.1553	0.89213	0.31154	-1.6635	0.79
10291	*KRTAP10-11*	2.3198	0.59321	-0.55437	-0.97658	0.79
10292	*MBTD1*	2.3235	0.14713	-0.11306	-2.1318	0.79
10293	*METTL18*	1.7283	1.4075	-0.77908	-0.82514	0.79
10294	*LYL1*	2.4638	0.5491	-0.65714	-2.003	0.79
10295	*LRRC57*	2.1731	0.8289	-0.64683	-0.88277	0.79
10296	*MPV17*	2.1416	0.74753	-0.53437	-0.58485	0.78
10297	*TCEB3CL2*	1.8116	1.3373	-0.79453	-1.0111	0.78

10298	*SGK2*	3.2979	0.4078	-1.3517	-1.6612	0.78
10299	*MANEAL*	2.8958	1.3201	-1.862	-2.312	0.78
10300	*TMX1*	2.5516	0.91611	-1.1145	-1.5671	0.78
10301	*ASIC1*	1.5611	0.42459	0.36727	-0.97794	0.78
10302	*ZBTB40*	2.3038	0.75877	-0.70998	-1.677	0.78
10303	*SIGLEC15*	3.6398	0.34346	-1.631	-1.6663	0.78
10304	*BARHL1*	3.5575	-0.11162	-1.0942	-1.8095	0.78
10305	*NFIA*	4.4105	-0.44445	-1.6146	-1.8792	0.78
10306	*C2orf81*	2.2473	0.58543	-0.48156	-3.1229	0.78
10307	*HHATL*	2.6713	-0.05456	-0.26575	-1.8869	0.78
10308	*RAG1*	3.9321	-0.6176	-0.96422	-1.0231	0.78
10309	*CDK17*	2.453	0.74731	-0.85194	-2.3573	0.78
10310	*KRT77*	1.5119	0.74882	0.08715	-1.0753	0.78
10311	*C16orf62*	4.4253	-0.98653	-1.0912	-1.172	0.78
10312	*UGT3A2*	2.9383	0.016992	-0.60837	-1.7954	0.78
10313	*SCGB1D2*	1.7666	0.43928	0.14042	-1.1805	0.78
10314	*BOD1L1*	2.6078	0.19521	-0.45672	-1.1317	0.78
10315	*FAM71B*	1.4012	1.259	-0.31396	-1.4247	0.78
10316	*PIKFYVE*	3.0749	1.5886	-2.3174	-2.3885	0.78
10317	*ATL1*	3.4562	-0.46123	-0.64888	-0.99368	0.78
10318	*DACT1*	3.6879	-0.1037	-1.2395	-1.5603	0.78
10319	*NFATC2*	2.5501	0.52293	-0.72916	-1.3574	0.78
10320	*ZFYVE16*	1.7454	0.54063	0.05771	-1.3496	0.78
10321	*GPR88*	3.4982	-0.34767	-0.8071	-1.2954	0.78
10322	*SUZ12*	2.8185	-0.07138	-0.40374	-2.207	0.78
10323	*ONECUT3*	1.4024	0.834	0.10662	-1.301	0.78
10324	*HBB*	2.2357	1.0171	-0.9101	-4.5174	0.78
10325	*PIK3R5*	3.0976	-0.19793	-0.55741	-1.1337	0.78
10326	*GPR149*	3.3876	-0.11049	-0.93525	-1.3982	0.78
10327	*PLK5*	2.9704	-0.14637	-0.48231	-1.3356	0.78
10328	*PNMAL2*	2.3689	0.11868	-0.14622	-0.54973	0.78
10329	*RAB36*	1.5351	0.97281	-0.16687	-1.4773	0.78
10330	*HTR5A*	2.3905	0.41593	-0.46647	-1.4584	0.78
10331	*TPD52L1*	3.9585	-0.09964	-1.5193	-1.6274	0.78
10332	*MTRF1*	1.3708	0.78469	0.18352	-0.67253	0.78
10333	*MBIP*	1.4205	0.78556	0.1311	-1.3946	0.78
10334	*OR6T1*	2.2119	0.21342	-0.0887	-1.695	0.78
10335	*RBP3*	1.8972	1.1339	-0.6951	-2.0505	0.78
10336	*ZFHX3*	3.6934	-0.17054	-1.1879	-1.9013	0.78
10337	*GARS*	1.6059	0.70459	0.02439	-1.2169	0.78
10338	*CD300E*	3.2432	0.31562	-1.2246	-2.3517	0.78
10339	*PLCXD2*	1.9618	0.50452	-0.13218	-1.7338	0.78
10340	*IL1F10*	1.848	1.1674	-0.68222	-1.8577	0.78
10341	*C17orf64*	1.9436	0.33355	0.05556	-1.1177	0.78
10342	*PRKCSH*	2.3979	0.1829	-0.24887	-1.388	0.78
10343	*FAM210B*	2.706	0.25769	-0.63207	-1.6194	0.78

10344	*TNNI3*	3.3665	-0.39944	-0.63554	-0.85931	0.78
10345	*OTOG*	2.6155	0.51683	-0.8012	-1.8205	0.78
10346	*PPEF1*	1.0245	1.0122	0.29337	-0.66353	0.78
10347	*CD9*	1.6089	0.50517	0.21583	-1.4185	0.78
10348	*TPSAB1*	1.7904	0.39161	0.1469	-0.95461	0.78
10349	*ALDH1L1*	3.2387	-0.41748	-0.49241	-2.2385	0.78
10350	*ZDHHC23*	3.2859	-0.18547	-0.77185	-1.0564	0.78
10351	*BPIFA1*	3.1265	0.35466	-1.1529	-1.3336	0.78
10352	*C17orf74*	3.5832	0.056101	-1.3114	-2.5764	0.78
10353	*ISG15*	1.5932	1.3654	-0.63125	-1.8076	0.78
10354	*CACTIN*	4.2852	-0.92339	-1.0354	-2.0134	0.78
10355	*TSPAN3*	2.5941	0.27663	-0.54465	-1.5382	0.78
10356	*CENPW*	4.7051	-0.64944	-1.7299	-2.112	0.78
10357	*PNMA5*	1.4454	0.60772	0.27244	-0.52917	0.78
10358	*GPR146*	3.6666	-0.26093	-1.0803	-2.7416	0.78
10359	*NUDT2*	1.7789	0.41266	0.13367	-1.7373	0.78
10360	*ELK3*	1.9592	0.28077	0.08526	-0.49347	0.78
10361	*RABAC1*	1.9404	0.51563	-0.13218	-2.1134	0.77
10362	*ATP2A1*	2.9209	0.45067	-1.0485	-1.368	0.77
10363	*IPO11*	5.4226	-1.4112	-1.6885	-2.4456	0.77
10364	*CTNNBIP1*	3.4427	0.008243	-1.1282	-1.4541	0.77
10365	*ANKRD54*	0.94214	0.85405	0.5251	-1.658	0.77
10366	*PRKRA*	1.234	0.75099	0.3359	0.079335	0.77
10367	*CUL7*	3.2959	-0.16245	-0.81272	-1.0581	0.77
10368	*ZSCAN25*	2.2769	0.32666	-0.28348	-0.31252	0.77
10369	*SYNE2*	0.87217	0.82828	0.61886	-0.4573	0.77
10370	*CASP7*	3.9051	0.15433	-1.7407	-1.9495	0.77
10371	*TTC17*	1.7117	0.49843	0.10845	-0.44353	0.77
10372	*SETD7*	2.8948	0.089763	-0.66696	-1.4388	0.77
10373	*MIF4GD*	1.0762	1.0349	0.20614	-1.1113	0.77
10374	*CCDC51*	3.1432	-0.39872	-0.4273	-2.7509	0.77
10375	*MASTL*	3.9421	-0.47672	-1.1492	-1.4185	0.77
10376	*ECE2*	2.4829	0.021801	-0.18978	-0.99699	0.77
10377	*ST8SIA4*	3.242	0.61104	-1.5383	-1.6158	0.77
10378	*ACY3*	4.8338	-1.2008	-1.3184	-1.5493	0.77
10379	*ZNF483*	2.8293	0.6123	-1.1275	-2.3121	0.77
10380	*DDI2*	2.6504	-0.05445	-0.28335	-1.1488	0.77
10381	*FAF2*	3.8488	-0.6202	-0.91807	-1.4681	0.77
10382	*TRIM14*	2.5264	0.46572	-0.68192	-1.9321	0.77
10383	*COTL1*	3.3776	0.34142	-1.4089	-2.0645	0.77
10384	*CKAP2*	2.6439	0.29378	-0.62792	-1.0567	0.77
10385	*KLK10*	1.3456	1.0208	-0.05715	-1.4016	0.77
10386	*MDFI*	1.9393	0.32059	0.04779	-1.3203	0.77
10387	*RIOK2*	3.1839	0.22609	-1.1034	-1.6306	0.77
10388	*GOLGA6C*	3.8896	-0.77396	-0.8098	-1.4584	0.77
10389	*LPO*	1.8337	0.23758	0.23385	-3.2856	0.77

10390	*INCA1*	1.3268	1.0566	-0.07831	-1.6071	0.77
10391	*STRBP*	2.2306	0.30193	-0.22784	-1.3034	0.77
10392	*LAMB1*	2.7461	0.44134	-0.88278	-0.97549	0.77
10393	*USP29*	2.6844	0.045563	-0.42545	-0.7513	0.77
10394	*FOXC1*	1.4373	1.1206	-0.25387	-0.51851	0.77
10395	*STOML1*	4.6545	-0.90296	-1.448	-2.1342	0.77
10396	*SMIM4*	3.3709	0.14654	-1.2141	-2.0895	0.77
10397	*POFUT1*	2.7014	0.28917	-0.6878	-1.3931	0.77
10398	*KRT33A*	1.7872	1.2934	-0.77853	-1.7014	0.77
10399	*GAPT*	2.0807	0.6854	-0.46404	-1.8031	0.77
10400	*SMIM22*	2.0969	0.2171	-0.01202	-1.7125	0.77
10401	*DGCR8*	3.8845	-0.51454	-1.0682	-2.508	0.77
10402	*LRRC49*	3.0962	1.1802	-1.9748	-2.3204	0.77
10403	*MTFR2*	2.0871	0.13367	0.08063	-0.94912	0.77
10404	*DUOX2*	3.0691	0.55428	-1.3221	-2.1574	0.77
10405	*ZNF654*	4.3949	-0.01938	-2.0752	-4.0172	0.77
10406	*QDPR*	3.1555	-0.38393	-0.47147	-2.9638	0.77
10407	*NKX2-5*	2.2128	1.9665	-1.8792	-2.382	0.77
10408	*PCDHA7*	4.9612	-1.2444	-1.4168	-2.3689	0.77
10409	*GLYATL1*	1.124	0.71441	0.46116	-0.78592	0.77
10410	*TOR1AIP1*	4.7279	-0.60083	-1.8282	-1.8759	0.77
10411	*WWP1*	3.5019	-0.59763	-0.60543	-1.2465	0.77
10412	*NDUFS7*	3.227	-0.13962	-0.78963	-1.4123	0.77
10413	*MFSD6L*	1.5384	0.7873	-0.02799	-0.5775	0.77
10414	*C10orf120*	3.1737	0.1136	-0.98976	-1.1826	0.77
10415	*REC8*	3.0241	0.53052	-1.2573	-2.3936	0.77
10416	*TASP1*	4.6	-0.93489	-1.3686	-1.4234	0.77
10417	*ARPC1A*	3.9292	-0.41967	-1.2141	-1.3199	0.77
10418	*RNGTT*	2.1735	0.48118	-0.36023	-1.2193	0.76
10419	*GABRA2*	3.7967	-0.09975	-1.4034	-1.7945	0.76
10420	*PHLDB2*	4.5413	-1.0039	-1.2448	-1.4919	0.76
10421	*MAGEA9*	2.9546	0.5894	-1.2515	-2.0952	0.76
10422	*EPHA2*	3.1766	0.092716	-0.97716	-1.197	0.76
10423	*HLA-DOA*	3.3724	-0.22166	-0.85931	-1.0852	0.76
10424	*RNF216*	2.5263	0.23188	-0.46748	-0.7763	0.76
10425	*MAPRE1*	2.9515	-0.18996	-0.47116	-1.8248	0.76
10426	*THTPA*	2.1898	1.184	-1.0849	-2.3452	0.76
10427	*RPL17-C18orf32*	3.8077	-0.09142	-1.4277	-1.8109	0.76
10428	*MAGEL2*	2.5008	-0.01099	-0.20264	-0.36394	0.76
10429	*OGDHL*	4.4222	-0.60425	-1.5308	-1.671	0.76
10430	*ZNF98*	0.9713	0.86127	0.45457	-1.1014	0.76
10431	*KCNJ4*	3.6205	-0.49212	-0.84195	-1.2846	0.76
10432	*PRR23D1*	2.1571	1.4095	-1.2802	-2.2311	0.76
10433	*IFI27L1*	1.1925	1.1617	-0.06838	-0.94653	0.76
10434	*ANKRD65*	3.0723	0.16666	-0.9532	-1.7322	0.76
10435	*USP54*	3.9011	-0.6122	-1.0033	-1.2792	0.76

10436	*ZNF709*	2.5472	-0.00422	-0.25762	-2.3877	0.76
10437	*CLCA2*	2.3211	0.77231	-0.80812	-1.1028	0.76
10438	*CTSH*	2.7602	-0.06576	-0.40976	-2.2694	0.76
10439	*GRIP1*	4.2359	-0.55773	-1.3935	-1.5235	0.76
10440	*ST8SIA5*	3.3157	0.56201	-1.5941	-1.7503	0.76
10441	*LOC100505478*	1.2296	0.7448	0.30903	-0.00542	0.76
10442	*CYP27C1*	3.1058	-0.18908	-0.63355	-0.78199	0.76
10443	*LSM7*	4.5996	-0.60976	-1.7074	-2.2721	0.76
10444	*DNAJC19*	3.0347	0.61924	-1.372	-2.1187	0.76
10445	*MARCO*	2.0644	0.97641	-0.75944	-2.3557	0.76
10446	*TBXA2R*	2.3577	0.017123	-0.09431	-0.2424	0.76
10447	*MACROD1*	1.3549	0.97787	-0.05226	-0.81793	0.76
10448	*PPARGC1A*	3.5336	0.57784	-1.8318	-2.1541	0.76
10449	*EXOC7*	1.2693	1.0277	-0.01774	-1.8157	0.76
10450	*TPRKB*	2.9052	-0.15922	-0.46741	-1.4616	0.76
10451	*RTCA*	2.6384	0.33037	-0.69045	-1.1251	0.76
10452	*SERPINB11*	1.2158	0.58065	0.48128	-0.26779	0.76
10453	*ACRC*	2.6919	0.52555	-0.93999	-0.95184	0.76
10454	*HSPB6*	3.9257	-0.5911	-1.0576	-1.4299	0.76
10455	*HSDL2*	4.2658	0.12307	-2.112	-2.5844	0.76
10456	*FBXL8*	3.5556	-0.53114	-0.74875	-1.5548	0.76
10457	*PCDHB7*	2.8431	1.01	-1.5789	-1.6352	0.76
10458	*ZNF189*	1.6338	0.51107	0.12927	-1.4423	0.76
10459	*FAM71C*	2.6682	0.33523	-0.72936	-1.0753	0.76
10460	*SLC16A1*	2.3653	0.042209	-0.13357	-1.6564	0.76
10461	*SUSD2*	2.3985	1.077	-1.202	-1.2302	0.76
10462	*PRR36*	1.6408	0.80931	-0.17663	-0.33133	0.76
10463	*RASA4B*	1.1529	1.0642	0.05627	-1.1395	0.76
10464	*KRTAP5-9*	2.0273	1.2836	-1.0376	-4.134	0.76
10465	*AIG1*	3.8335	-0.72725	-0.83327	-1.8631	0.76
10466	*ZGPAT*	1.4667	1.0639	-0.25773	-1.6455	0.76
10467	*EMX1*	2.5562	-0.06903	-0.21452	-1.9133	0.76
10468	*SEPT9*	3.9003	-0.58499	-1.0433	-2.2947	0.76
10469	*CR1L*	1.6363	0.47547	0.15986	-0.06441	0.76
10470	*VAMP2*	1.4738	0.46987	0.32795	-0.07273	0.76
10471	*CBLN1*	3.2949	0.15245	-1.1759	-1.9624	0.76
10472	*ATP11B*	2.2638	0.12189	-0.11452	-1.8615	0.76
10473	*OR10V1*	1.7072	1.0064	-0.44353	-2.0629	0.76
10474	*KRTAP10-9*	3.2704	-0.25286	-0.74755	-1.0492	0.76
10475	*CAPSL*	3.521	-0.25821	-0.9929	-3.3367	0.76
10476	*PAQR4*	1.3935	0.56342	0.31269	0.044791	0.76
10477	*NVL*	3.0473	0.14191	-0.91968	-1.2264	0.76
10478	*SYT14*	2.6364	0.30572	-0.67265	-1.0212	0.76
10479	*BEAN1*	2.3348	0.49557	-0.56218	-1.1256	0.76
10480	*IL17RE*	4.6003	-0.8966	-1.4356	-2.1282	0.76
10481	*PCDHGB6*	2.9764	0.004801	-0.71382	-1.0677	0.76

10482	*ZCCHC8*	2.0206	0.20314	0.04359	-1.207	0.76
10483	*ENKUR*	2.741	-0.14289	-0.3328	-1.9242	0.76
10484	*HOXC12*	2.4482	0.10825	-0.29194	-2.0102	0.75
10485	*OPLAH*	3.0453	0.14068	-0.9215	-1.205	0.75
10486	*KRTAP12-2*	2.7058	0.094786	-0.53704	-1.4823	0.75
10487	*C11orf84*	1.4269	0.96977	-0.13392	-1.257	0.75
10488	*GTF3C3*	3.3242	0.29391	-1.3556	-1.4736	0.75
10489	*XRN1*	3.5321	-0.11437	-1.1569	-1.5337	0.75
10490	*ABHD16B*	2.9215	0.1106	-0.77241	-1.4299	0.75
10491	*UPF2*	2.439	0.019779	-0.19969	-0.56681	0.75
10492	*PXN*	3.7188	-0.58305	-0.87681	-1.1017	0.75
10493	*TMEM14E*	1.4726	1.3784	-0.59217	-0.6683	0.75
10494	*ZFAND1*	2.3348	0.99791	-1.0739	-1.403	0.75
10495	*MOS*	4.6142	-1.073	-1.2827	-2.5753	0.75
10496	*LRRC10*	3.0993	0.45939	-1.3007	-1.3604	0.75
10497	*SERPINI2*	1.2443	0.92761	0.0859	-0.23531	0.75
10498	*CHSY1*	2.0827	1.1608	-0.98572	-2.4333	0.75
10499	*DNAJC5*	5.5677	-1.3836	-1.9269	-5.6631	0.75
10500	*GABRB3*	1.599	0.68647	-0.02943	-1.7263	0.75
10501	*SLC6A19*	2.3458	0.26904	-0.3589	-1.5314	0.75
10502	*SAMD12*	3.427	-0.57564	-0.59581	-1.962	0.75
10503	*COA4*	1.9336	0.57958	-0.25767	-1.3823	0.75
10504	*OCIAD2*	1.2361	0.81182	0.20724	-0.27315	0.75
10505	*RRM1*	2.6745	0.058631	-0.47862	-0.63584	0.75
10506	*TAF1A*	1.0734	1.0671	0.11383	-1.5138	0.75
10507	*SERPINF2*	1.9636	1.0611	-0.77117	-1.1075	0.75
10508	*CUL9*	4.1801	-0.4628	-1.4639	-1.9308	0.75
10509	*MGRN1*	2.98	-0.2562	-0.47125	-1.5611	0.75
10510	*ABCC5*	1.4967	1.2286	-0.47304	-1.6836	0.75
10511	*PLEKHN1*	4.9611	-1.1665	-1.5429	-2.5655	0.75
10512	*CHCHD2*	2.7683	0.32334	-0.84015	-1.7122	0.75
10513	*YPEL2*	2.7027	1.067	-1.5186	-1.7954	0.75
10514	*PEBP4*	2.2536	1.6609	-1.6635	-1.8107	0.75
10515	*HNRNPCL3*	1.7744	0.37511	0.10111	-0.53661	0.75
10516	*USP53*	2.849	0.004468	-0.60289	-1.25	0.75
10517	*TAF5L*	1.6873	0.35719	0.2048	-0.49598	0.75
10518	*CATSPER3*	2.0556	0.15571	0.03686	-0.31976	0.75
10519	*C16orf90*	1.3453	0.56824	0.33441	-2.2075	0.75
10520	*DEFB132*	2.3372	0.58173	-0.67149	-0.69748	0.75
10521	*PHGDH*	1.8263	1.4315	-1.0104	-1.5913	0.75
10522	*TRPC3*	0.94689	0.90031	0.39967	-1.6542	0.75
10523	*SHB*	2.8776	0.39634	-1.0272	-1.3162	0.75
10524	*ITGAV*	3.2762	0.18588	-1.2159	-1.9234	0.75
10525	*GTPBP3*	1.1573	0.56379	0.52455	0.047591	0.75
10526	*NFASC*	3.4622	0.060357	-1.2774	-1.6956	0.75
10527	*IDS*	0.83163	0.81238	0.60109	-0.58554	0.75

10528	*LBX1*	2.3495	0.26856	-0.37343	-0.54515	0.75
10529	*KCNV2*	1.4998	0.66554	0.07918	0.018683	0.75
10530	*IKZF4*	2.9339	-0.20849	-0.48169	-1.0733	0.75
10531	*LAMTOR4*	3.3623	0.8165	-1.9351	-2.0661	0.75
10532	*LIG4*	3.7754	0.046811	-1.5788	-1.6617	0.75
10533	*PRR19*	4.1453	-0.00831	-1.8938	-2.0062	0.75
10534	*S100A10*	3.5434	0.32485	-1.626	-2.1648	0.75
10535	*SDHAF3*	1.9468	0.3523	-0.05738	-0.67231	0.75
10536	*GGT5*	4.6395	-0.44353	-1.9548	-2.2743	0.75
10537	*POU2F1*	2.5406	0.76167	-1.0629	-1.0989	0.75
10538	*ZEB1*	2.188	0.095742	-0.04457	-2.0725	0.75
10539	*FLT3*	2.0567	0.91797	-0.73673	-1.3089	0.75
10540	*LRRC61*	1.3456	1.1752	-0.28384	-1.5568	0.75
10541	*ADCY4*	2.7217	-0.23929	-0.24596	-1.2135	0.75
10542	*NIFK*	2.328	0.23041	-0.32237	-1.8127	0.75
10543	*HDGFRP3*	2.3809	1.8729	-2.0181	-2.2044	0.75
10544	*MSI2*	1.7822	0.45832	-0.00503	-0.43756	0.75
10545	*PAPSS1*	1.4646	1.214	-0.44353	-1.0275	0.75
10546	*ZRANB1*	0.9894	0.81238	0.43322	-0.49127	0.75
10547	*FUT11*	1.7917	0.7941	-0.35134	-2.8922	0.74
10548	*NDST1*	1.5351	1.1424	-0.44353	-0.61783	0.74
10549	*CDCA3*	3.2398	0.94595	-1.9523	-2.4215	0.74
10550	*MSMB*	1.2395	0.85245	0.14129	-1.8714	0.74
10551	*UAP1L1*	3.9731	-0.44498	-1.2952	-1.9417	0.74
10552	*HOXB5*	1.4487	1.4433	-0.65919	-2.1798	0.74
10553	*OR2L13*	4.1826	-0.59045	-1.3604	-1.5153	0.74
10554	*RPS6KB2*	1.6854	0.3323	0.21388	-0.06364	0.74
10555	*MYCBP*	3.3566	0.040889	-1.1665	-2.8007	0.74
10556	*YPEL4*	3.6255	-0.30965	-1.0858	-2.0138	0.74
10557	*FAM86B1*	4.558	-1.0467	-1.2816	-1.3399	0.74
10558	*RPL22*	1.2125	0.72339	0.29361	-1.1814	0.74
10559	*PSIP1*	1.904	0.95444	-0.62947	-1.6796	0.74
10560	*WNT11*	1.1254	0.55491	0.5484	0.15515	0.74
10561	*TPM4*	3.0065	0.24242	-1.0204	-2.4312	0.74
10562	*TIMM50*	3.1032	0.70647	-1.5817	-3.1149	0.74
10563	*PPP3R2*	1.9976	0.12243	0.10724	-0.58602	0.74
10564	*XPNPEP2*	1.5796	0.84092	-0.1934	-1.3451	0.74
10565	*GAGE2D*	3.4323	-0.15039	-1.0551	-1.3457	0.74
10566	*APOC3*	2.8716	0.059357	-0.70503	-1.0936	0.74
10567	*CAMSAP1*	1.5442	0.40718	0.27427	-0.10341	0.74
10568	*C19orf67*	2.3847	-0.03335	-0.1258	-0.69961	0.74
10569	*SMYD5*	1.8978	1.5711	-1.2448	-2.3244	0.74
10570	*TXNDC17*	1.1318	0.91214	0.18015	0.03574	0.74
10571	*PPP3CA*	3.7019	-0.69463	-0.78345	-1.5302	0.74
10572	*PI4KB*	2.0869	0.19793	-0.0616	-1.3437	0.74
10573	*SFI1*	2.13	0.61705	-0.52447	-1.711	0.74

10574	*PKP1*	3.1271	-0.20857	-0.696	-0.90387	0.74
10575	*DCTPP1*	3.0675	0.15514	-1.0016	-1.142	0.74
10576	*CXorf51B*	0.95551	0.70299	0.56243	-0.73073	0.74
10577	*CHN2*	3.876	-0.64398	-1.0111	-1.1076	0.74
10578	*TRAPPC6B*	2.7485	0.58786	-1.1155	-1.1912	0.74
10579	*SMG6*	2.097	0.19646	-0.07297	-1.8283	0.74
10580	*PSMG3*	3.4453	-0.40785	-0.8202	-1.6993	0.74
10581	*LOXHD1*	1.8786	0.68258	-0.34402	-2.4333	0.74
10582	*CYP2J2*	3.5391	-0.31807	-1.0041	-1.4043	0.74
10583	*TTC5*	1.3708	1.0236	-0.17749	-1.1944	0.74
10584	*LILRA4*	1.0324	0.71163	0.47269	-2.1791	0.74
10585	*SDF2*	2.7299	0.078277	-0.5915	-1.9362	0.74
10586	*SLC2A8*	3.0398	0.73702	-1.5604	-1.7306	0.74
10587	*CDK2AP2*	3.0782	-0.35746	-0.50463	-0.59791	0.74
10588	*AK2*	2.2904	1.7971	-1.872	-2.3488	0.74
10589	*LOC101929983*	3.0205	-0.20874	-0.59716	-1.2234	0.74
10590	*OPHN1*	1.3451	0.66293	0.20649	-0.09423	0.74
10591	*TIMM8A*	1.9066	0.27266	0.03497	-0.91842	0.74
10592	*FANCM*	1.6392	0.57241	0.00226	-1.3701	0.74
10593	*CLP1*	1.8326	0.86339	-0.48231	-1.3804	0.74
10594	*ST8SIA1*	2.6154	1.3089	-1.7107	-2.7849	0.74
10595	*NACA2*	3.2674	-0.18229	-0.87257	-1.6571	0.74
10596	*PKLR*	1.5706	1.1761	-0.53554	-1.5719	0.74
10597	*CRYAA*	2.1228	0.73736	-0.64982	-1.3903	0.74
10598	*WHSC1*	3.2188	0.13705	-1.146	-2.5103	0.74
10599	*WDR73*	1.0584	0.90972	0.24167	-1.8813	0.74
10600	*TRIM77*	3.6279	-0.27421	-1.1442	-1.8267	0.74
10601	*NKD2*	2.9405	-0.27631	-0.45475	-0.46752	0.74
10602	*WNT8B*	2.498	0.060229	-0.34926	-2.0351	0.74
10603	*TMEM221*	1.318	0.56804	0.32277	-1.358	0.74
10604	*ATP9A*	3.1997	-0.19915	-0.79319	-1.5633	0.74
10605	*CDC42BPB*	5.1189	-1.0737	-1.8382	-2.0368	0.74
10606	*APLP1*	1.3795	0.61645	0.21028	-0.49016	0.74
10607	*DDX23*	1.0246	0.91369	0.2678	-0.61818	0.74
10608	*RRP12*	1.1602	1.046	-0.00081	-0.88561	0.74
10609	*GLRA2*	1.8761	1.1164	-0.78728	-1.5195	0.74
10610	*ENOX1*	3.4726	-0.55676	-0.71077	-1.0796	0.74
10611	*PPP2R5B*	1.8479	1.093	-0.73599	-1.8908	0.73
10612	*UBAP2L*	2.5273	0.36907	-0.69215	-1.3517	0.73
10613	*ELAC1*	2.4024	-0.05841	-0.14048	-0.77404	0.73
10614	*CARD14*	3.7104	-0.48179	-1.0258	-1.95	0.73
10615	*FGR*	2.8115	-0.03371	-0.575	-1.6599	0.73
10616	*FBXO16*	1.5232	1.1982	-0.51886	-1.4164	0.73
10617	*DNAH6*	2.0996	1.3594	-1.2566	-1.6524	0.73
10618	*EHD3*	3.413	-0.30863	-0.9027	-1.2815	0.73
10619	*EFHD2*	2.0644	0.92295	-0.78591	-0.79392	0.73

10620	*ZNF324*	2.0581	0.086332	0.05664	-0.56598	0.73
10621	*DMRTC1*	1.3	0.57159	0.32946	-1.5155	0.73
10622	*ALPP*	1.4167	0.96716	-0.18283	-1.3549	0.73
10623	*C10orf32*	1.9352	0.48891	-0.22327	-1.3231	0.73
10624	*LOC158434*	1.8914	1.4647	-1.1553	-2.9028	0.73
10625	*POLB*	3.7864	-0.45012	-1.1367	-1.3875	0.73
10626	*GUCY1A2*	1.6915	0.8482	-0.34037	-0.36731	0.73
10627	*NPHS1*	3.9841	-0.78591	-0.99906	-2.0538	0.73
10628	*DRD3*	1.7342	0.75036	-0.28742	-1.0889	0.73
10629	*RBM18*	3.2318	0.15901	-1.1944	-3.1836	0.73
10630	*ZMYM3*	2.3014	0.83796	-0.94341	-1.4448	0.73
10631	*VAV2*	1.3477	0.75766	0.09044	-0.49095	0.73
10632	*TCP11L2*	2.4487	0.097322	-0.35023	-0.37629	0.73
10633	*SLC52A3*	2.2088	0.56958	-0.58495	-1.4841	0.73
10634	*HOXA2*	3.0306	0.47742	-1.3148	-1.5663	0.73
10635	*ZNF764*	5.2834	-0.89186	-2.1984	-2.2879	0.73
10636	*NCL*	2.134	0.56844	-0.50961	-0.781	0.73
10637	*GOLGA8J*	1.7815	0.83556	-0.42545	-0.80594	0.73
10638	*GDPD3*	2.5946	1.0199	-1.4234	-1.8348	0.73
10639	*SLC25A18*	3.6215	-0.6408	-0.78966	-1.739	0.73
10640	*SLC16A12*	2.0456	0.53271	-0.38744	-1.7595	0.73
10641	*GGT6*	2	0.75545	-0.56461	-0.87748	0.73
10642	*ZFP69B*	2.9932	0.51453	-1.3199	-1.3966	0.73
10643	*C11orf73*	2.8032	1.0756	-1.6917	-1.8792	0.73
10644	*PRRT4*	1.1402	0.74863	0.2981	-1.7364	0.73
10645	*FAM72A*	2.1011	0.60353	-0.5183	-1.0954	0.73
10646	*SPAG8*	2.1774	0.075908	-0.06698	-0.78965	0.73
10647	*BMI1*	4.4632	-0.99175	-1.2852	-1.6288	0.73
10648	*PRPF6*	1.688	0.89755	-0.39932	-1.0948	0.73
10649	*TGFBR2*	3.4332	-0.07137	-1.1758	-1.6164	0.73
10650	*WSCD2*	2.4353	-0.05219	-0.19778	-2.0488	0.73
10651	*SMS*	3.1745	-0.3433	-0.64639	-2.1328	0.73
10652	*WDR83OS*	1.2223	0.57596	0.3861	-1.3676	0.73
10653	*KCNV1*	1.8131	0.35814	0.01234	-0.88279	0.73
10654	*OR10R2*	1.1235	1.0338	0.02626	-0.70391	0.73
10655	*KISS1*	3.938	-0.30339	-1.4527	-3.0804	0.73
10656	*UNC13D*	2.8828	-0.23196	-0.46973	-2.0858	0.73
10657	*HIST1H3C*	2.7657	0.51931	-1.1043	-1.3721	0.73
10658	*TTC24*	4.9931	-0.47211	-2.3405	-2.4961	0.73
10659	*NUBPL*	2.314	0.32643	-0.46017	-1.7407	0.73
10660	*SLC38A2*	2.6484	1.2379	-1.7061	-1.9075	0.73
10661	*OCLN*	2.2509	-0.00437	-0.0665	-1.3666	0.73
10662	*SPATA13*	2.3113	0.34215	-0.47381	-2.9772	0.73
10663	*DLGAP2*	2.8046	-0.00826	-0.61682	-1.1169	0.73
10664	*PA2G4*	1.2197	0.616	0.34374	-1.7513	0.73
10665	*CST6*	2.3056	0.13558	-0.26177	-1.4829	0.73

10666	*ALPPL2*	2.0211	0.23258	-0.07439	-0.56922	0.73
10667	*NRGN*	2.303	0.14733	-0.27244	-1.4276	0.73
10668	*RFFL*	1.6287	0.66682	-0.11771	-0.54058	0.73
10669	*ATF4*	2.377	0.24339	-0.44277	-0.44353	0.73
10670	*EDN2*	2.9472	0.23006	-1.0005	-1.8702	0.73
10671	*CSAD*	1.4586	0.46327	0.25333	-0.21499	0.73
10672	*OR6K3*	2.5893	0.44838	-0.86298	-1.8227	0.72
10673	*STK40*	1.9869	0.43201	-0.24519	-0.70517	0.72
10674	*COL6A2*	1.8005	0.39495	-0.02184	-1.5467	0.72
10675	*PTMS*	1.36	0.84302	-0.02943	-0.1449	0.72
10676	*ADAMTSL1*	1.3052	0.87644	-0.00826	-0.31976	0.72
10677	*EPT1*	1.2849	0.62916	0.25904	-0.91396	0.72
10678	*ERAS*	1.1894	0.58811	0.3946	-0.26083	0.72
10679	*GPR182*	2.1368	0.026786	0.0073	-1.1747	0.72
10680	*SLC35F1*	1.574	1.4306	-0.83459	-1.0475	0.72
10681	*HCN3*	1.9657	1.7647	-1.5604	-2.0952	0.72
10682	*ANXA3*	1.2378	0.63583	0.29569	-2.2167	0.72
10683	*ANK2*	1.399	0.88455	-0.11452	-1.8068	0.72
10684	*FEM1C*	3.9448	-0.82439	-0.95184	-1.049	0.72
10685	*FGFRL1*	1.8587	0.36774	-0.05814	-2.7245	0.72
10686	*SEPN1*	3.1024	-0.35892	-0.57564	-2.1145	0.72
10687	*RAB2A*	2.8452	0.27879	-0.95629	-1.1325	0.72
10688	*MED24*	1.9693	0.28022	-0.08248	-0.80069	0.72
10689	*NELL2*	3.089	0.5897	-1.5137	-2.0146	0.72
10690	*DEC1*	1.6604	0.3623	0.14219	-1.0323	0.72
10691	*SMCHD1*	1.2997	0.61432	0.2485	-1.7044	0.72
10692	*RBM48*	2.4038	0.005991	-0.24741	-1.8507	0.72
10693	*TOX4*	3.1759	-0.33404	-0.68027	-1.2061	0.72
10694	*CTSS*	1.8928	0.26025	0.00845	-0.78588	0.72
10695	*CDC34*	2.0993	0.9191	-0.85712	-1.5775	0.72
10696	*MGAT4D*	3.1025	-0.28431	-0.65695	-1.8038	0.72
10697	*NHSL1*	1.8381	1.2205	-0.89741	-1.677	0.72
10698	*FASTKD5*	2.8034	0.10266	-0.745	-1.5883	0.72
10699	*SLC30A4*	3.0494	-0.01623	-0.87328	-1.3814	0.72
10700	*ADARB2*	1.0231	0.93431	0.20188	-0.1207	0.72
10701	*USP33*	2.5795	0.50701	-0.92905	-1.5696	0.72
10702	*PIGG*	1.3661	0.95719	-0.16624	-2.4815	0.72
10703	*TMEM67*	2.6095	0.36252	-0.8152	-2.5571	0.72
10704	*PHF14*	3.5516	-0.2671	-1.1277	-1.2195	0.72
10705	*CANT1*	3.1815	0.30181	-1.3268	-2.2593	0.72
10706	*CRYGN*	3.4959	0.049791	-1.3903	-1.8996	0.72
10707	*SAP130*	1.9436	1.244	-1.0342	-2.4576	0.72
10708	*RARB*	5.0722	-0.91317	-2.0062	-2.0871	0.72
10709	*ABT1*	1.1969	1.1314	-0.17597	-1.4636	0.72
10710	*EIF2B3*	0.73531	0.73146	0.68528	-1.2371	0.72
10711	*DNAJC21*	4.2711	-0.78313	-1.3364	-1.9622	0.72

10712	*ZDHHC18*	3.2022	0.15856	-1.2094	-2.1298	0.72
10713	*OR8H2*	1.4171	0.52142	0.21281	-0.27159	0.72
10714	*EIF3E*	1.3986	0.69035	0.06199	-0.2313	0.72
10715	*PRPH*	2.3608	0.78265	-0.9929	-1.0436	0.72
10716	*RARA*	1.6387	0.83056	-0.31878	-1.6095	0.72
10717	*NOSIP*	1.9326	0.30864	-0.09119	-1.3288	0.72
10718	*TNFRSF18*	1.1746	0.82307	0.1517	-1.0013	0.72
10719	*RHOT1*	2.4573	0.20894	-0.51764	-3.1751	0.72
10720	*ASB18*	1.374	0.91767	-0.1435	-2.6313	0.72
10721	*OR10G2*	4.6463	-0.78579	-1.7129	-2.462	0.72
10722	*CILP2*	3.2712	0.25132	-1.3753	-1.5176	0.72
10723	*TCEANC2*	2.6296	0.6556	-1.1383	-1.9622	0.72
10724	*MAT2B*	2.4373	0.48151	-0.77241	-1.9372	0.72
10725	*COBLL1*	3.5375	-0.52469	-0.86756	-1.2141	0.72
10726	*SMCO1*	1.4666	0.51053	0.16756	-1.3551	0.71
10727	*C16orf95*	2.1558	0.63221	-0.6445	-1.8581	0.71
10728	*VWA3A*	2.8419	0.26262	-0.96206	-2.1859	0.71
10729	*STPG2*	1.9492	0.32601	-0.13354	-0.52649	0.71
10730	*UQCC2*	2.036	0.39273	-0.28734	-0.75287	0.71
10731	*TTC34*	3.4692	-0.15153	-1.1765	-1.2867	0.71
10732	*FAM53A*	2.8677	-0.24896	-0.4783	-0.87681	0.71
10733	*OR52E2*	1.5657	0.67663	-0.10288	-2.8636	0.71
10734	*ZCCHC18*	1.429	0.53063	0.17824	-0.87332	0.71
10735	*SPG11*	2.4136	0.4304	-0.70685	-0.77399	0.71
10736	*F2RL1*	3.2627	-0.51363	-0.61264	-0.96492	0.71
10737	*MAGEA11*	3.1644	0.034674	-1.063	-1.5598	0.71
10738	*NOP9*	2.9647	0.05102	-0.88008	-2.4141	0.71
10739	*KIFC1*	2.1759	0.5224	-0.56324	-2.485	0.71
10740	*GJB6*	4.7014	-0.92781	-1.6386	-1.7928	0.71
10741	*LRRC4B*	2.877	-0.29546	-0.44675	-0.91335	0.71
10742	*ILDR1*	0.96695	0.66549	0.50216	0.46644	0.71
10743	*CCND1*	1.709	0.56162	-0.13651	-2.2167	0.71
10744	*LYPD6B*	3.3653	-0.59028	-0.64164	-1.2864	0.71
10745	*FAM98B*	2.6602	0.86884	-1.3961	-2.2809	0.71
10746	*BCL7B*	1.0595	0.5928	0.48014	-1.5809	0.71
10747	*DCDC2C*	2.4799	0.42265	-0.77019	-1.6979	0.71
10748	*SURF6*	3.3483	-0.47476	-0.74184	-1.6796	0.71
10749	*HKDC1*	1.1253	0.58403	0.42221	-0.51467	0.71
10750	*CHST2*	4.415	-0.89941	-1.3842	-2.0452	0.71
10751	*ROM1*	3.3104	-0.43292	-0.74611	-0.90149	0.71
10752	*NTHL1*	2.2265	0.031866	-0.12725	-0.55815	0.71
10753	*LEMD2*	3.0054	-0.14406	-0.73077	-1.0585	0.71
10754	*BIVM-ERCC5*	1.5848	0.40132	0.14433	0.011164	0.71
10755	*SPSB4*	2.2114	0.10585	-0.18699	-0.51931	0.71
10756	*FGF5*	2.4902	-0.0175	-0.34308	-0.98194	0.71
10757	*KRTAP16-1*	2.0071	0.69902	-0.57681	-0.71896	0.71

10758	*SCNN1D*	3.6731	-0.73517	-0.80907	-0.91982	0.71
10759	*NKAIN4*	1.1966	0.66293	0.26888	-0.69325	0.71
10760	*ERN1*	1.9628	0.49689	-0.33148	-0.44353	0.71
10761	*FEZ1*	2.7238	0.30989	-0.90652	-1.2667	0.71
10762	*GGT7*	2.9339	-0.40312	-0.40398	-2.2456	0.71
10763	*FAM71A*	1.3187	0.7847	0.02334	-0.04564	0.71
10764	*ABCC9*	1.4717	0.57601	0.07828	-0.23796	0.71
10765	*TNPO1*	3.9758	-0.34227	-1.5099	-1.8952	0.71
10766	*CLDN11*	2.6681	-0.271	-0.2735	-1.4584	0.71
10767	*LRRC37A2*	0.91814	0.77708	0.42745	-0.19975	0.71
10768	*PTGER3*	1.6557	0.5484	-0.08217	-1.9397	0.71
10769	*OXSM*	2.7274	0.30691	-0.91429	-1.8338	0.71
10770	*SCUBE2*	2.3678	0.30747	-0.55541	-1.1637	0.71
10771	*TROVE2*	2.402	-0.10835	-0.17383	-0.78965	0.71
10772	*TIMM22*	4.6784	-1.2079	-1.3507	-2.3204	0.71
10773	*CXorf51A*	2.3881	0.18947	-0.45847	-0.82974	0.71
10774	*RNF40*	3.1367	0.056625	-1.0757	-2.8019	0.71
10775	*LENG9*	1.6846	0.42831	0.00368	-0.74331	0.71
10776	*RFXAP*	2.2698	0.30207	-0.45544	-1.2411	0.71
10777	*OR13J1*	3.4841	-0.44353	-0.92415	-2.3707	0.71
10778	*BIRC8*	1.2643	0.75092	0.10118	-0.33985	0.71
10779	*FRMPD4*	2.5368	-0.06121	-0.35946	-0.80594	0.71
10780	*SULT1C2*	2.7831	0.35036	-1.018	-1.7591	0.71
10781	*SGSM3*	1.9665	1.0225	-0.87429	-2.5901	0.70
10782	*SELENBP1*	2.866	0.093836	-0.84595	-1.0884	0.70
10783	*DUS1L*	4.0338	-0.44099	-1.4803	-1.8441	0.70
10784	*TMEM100*	4.3308	-0.51995	-1.6984	-2.3557	0.70
10785	*CYSLTR1*	1.5529	0.48201	0.0774	-0.61682	0.70
10786	*TMED2*	2.4347	0.7361	-1.0586	-1.134	0.70
10787	*UGT1A8*	2.2118	0.009703	-0.11119	-1.1977	0.70
10788	*SLC2A14*	1.5007	0.83896	-0.23034	-0.99871	0.70
10789	*IFI44*	1.9312	0.25525	-0.07822	-1.2684	0.70
10790	*EPB41L3*	1.0123	0.82489	0.27022	-0.35718	0.70
10791	*IL20RA*	3.3019	-0.04172	-1.1529	-1.333	0.70
10792	*BEST4*	1.8928	0.36404	-0.14994	-0.87128	0.70
10793	*LMTK2*	1.9691	0.22358	-0.08618	-1.2852	0.70
10794	*TYRO3*	2.3478	0.44266	-0.68453	-1.9099	0.70
10795	*RPL26*	3.0495	-0.36877	-0.57546	-2.1117	0.70
10796	*ZNF467*	0.81473	0.66112	0.62883	-0.19183	0.70
10797	*GRPEL2*	3.8919	-0.79108	-0.99694	-1.0654	0.70
10798	*PPP1R1C*	2.7596	0.53774	-1.1944	-1.9068	0.70
10799	*GET4*	2.8258	-0.08975	-0.63348	-2.7016	0.70
10800	*PHF3*	3.4143	0.050668	-1.3627	-1.8033	0.70
10801	*ERGIC2*	2.1204	0.42502	-0.44353	-0.81329	0.70
10802	*RAB22A*	1.5667	0.29221	0.24249	-0.29798	0.70
10803	*DEFB131*	2.3459	0.18009	-0.42545	-1.1081	0.70

10804	*AZGP1*	3.4506	0.047153	-1.3973	-1.5831	0.70
10805	*TIAF1*	1.5675	0.41298	0.11874	-0.41642	0.70
10806	*FCGR2A*	1.4002	0.71902	-0.02022	-0.97123	0.70
10807	*PRX*	2.8935	-0.2408	-0.55371	-1.1316	0.70
10808	*SPINT3*	4.1234	-0.59716	-1.4273	-1.5672	0.70
10809	*MPP1*	2.2779	0.84201	-1.021	-1.4994	0.70
10810	*HTR3E*	1.367	0.89372	-0.16249	-0.29713	0.70
10811	*C17orf67*	3.621	-0.58451	-0.93837	-0.93878	0.70
10812	*PTP4A2*	2.093	0.47131	-0.46623	-0.55927	0.70
10813	*HOMEZ*	0.8032	0.73902	0.55522	-1.4688	0.70
10814	*MCFD2*	1.2626	0.97328	-0.13903	-0.29232	0.70
10815	*CPEB1*	3.2785	0.41241	-1.5949	-2.0995	0.70
10816	*FMO4*	1.5934	0.37149	0.13016	-0.24829	0.70
10817	*NBPF3*	2.9762	-0.11759	-0.76389	-1.4789	0.70
10818	*YWHAB*	3.4907	-0.66731	-0.72877	-1.7702	0.70
10819	*MKS1*	2.2153	0.93545	-1.0564	-1.5201	0.70
10820	*TMEM150C*	2.3551	-0.09142	-0.16944	-1.4415	0.70
10821	*PCP2*	0.89844	0.66958	0.52466	0.21805	0.70
10822	*MYOZ1*	1.6782	0.48958	-0.07561	-0.84337	0.70
10823	*CT47A4*	2.8987	0.12297	-0.92956	-1.8297	0.70
10824	*FAM151A*	3.3447	-0.4046	-0.84841	-1.8856	0.70
10825	*DHDDS*	1.4748	1.195	-0.5788	-0.88272	0.70
10826	*R3HDML*	4.5451	-0.95321	-1.5022	-1.7322	0.70
10827	*ADCY5*	0.93097	0.75357	0.40349	-0.63399	0.70
10828	*ARHGAP44*	1.8907	0.78149	-0.58611	-1.456	0.70
10829	*GIN1*	1.0749	0.84029	0.169	-0.01111	0.69
10830	*ZNF710*	1.4751	0.78283	-0.17383	-0.49635	0.69
10831	*ZNF493*	0.73626	0.73253	0.61455	-1.0134	0.69
10832	*POLD4*	2.8192	-0.17635	-0.55958	-1.4725	0.69
10833	*PEX11G*	5.3862	-1.4234	-1.8804	-2.3063	0.69
10834	*KATNAL2*	2.2656	0.73478	-0.91913	-1.0097	0.69
10835	*MMP8*	4.3948	-0.54523	-1.7709	-2.2046	0.69
10836	*WDR81*	1.3187	0.39822	0.36147	-1.4284	0.69
10837	*ACO1*	1.9945	0.14345	-0.05985	-2.0697	0.69
10838	*FAM220A*	3.3409	0.8408	-2.1036	-2.1347	0.69
10839	*ADGRA1*	2.9072	-0.20318	-0.62596	-2.1648	0.69
10840	*SAP25*	3.0232	-0.07202	-0.87318	-1.6525	0.69
10841	*SHKBP1*	2.6126	-0.0749	-0.46213	-0.55015	0.69
10842	*ADPRHL1*	2.8187	0.2138	-0.95708	-0.95775	0.69
10843	*MS4A7*	2.3252	0.5013	-0.75169	-1.3955	0.69
10844	*PPP1R15B*	3.3141	-0.60083	-0.63939	-0.95105	0.69
10845	*RPRD2*	2.7643	-0.32677	-0.36443	-1.8656	0.69
10846	*MED7*	2.791	0.88044	-1.5984	-1.855	0.69
10847	*ECI2*	1.6426	0.41919	0.01102	-0.02486	0.69
10848	*AMT*	3.3401	0.34728	-1.615	-1.9107	0.69
10849	*ZNF862*	1.7694	0.35153	-0.04898	-0.52106	0.69

10850	*UBE2D2*	2.9323	0.13442	-0.99508	-1.251	0.69
10851	*MMP3*	3.7466	-0.77018	-0.90501	-2.0277	0.69
10852	*HLX*	0.76394	0.69539	0.61199	-2.1669	0.69
10853	*NOBOX*	0.78024	0.70154	0.58939	-1.2018	0.69
10854	*FOXD4L6*	3.1668	0.68881	-1.7845	-2.5981	0.69
10855	*KDM5D*	1.1682	0.89872	0.00408	-0.01352	0.69
10856	*MCTS1*	4.1778	-1.0514	-1.0561	-1.2547	0.69
10857	*TRMT1*	1.9385	1.792	-1.6604	-2.2794	0.69
10858	*MZF1*	1.3913	0.54343	0.13481	0.028062	0.69
10859	*CELA3A*	2.3392	0.58419	-0.85427	-1.7577	0.69
10860	*USP5*	2.3304	-0.04198	-0.22069	-1.0528	0.69
10861	*LRP1*	3.976	-0.43998	-1.4684	-1.9567	0.69
10862	*GPI*	3.4563	-0.12158	-1.2694	-1.6213	0.69
10863	*UBALD1*	1.8413	0.24605	-0.02223	-1.6907	0.69
10864	*DLGAP4*	1.6774	1.1515	-0.76505	-0.77284	0.69
10865	*TNNC1*	0.87135	0.65572	0.53623	-0.47527	0.69
10866	*DDHD2*	1.8264	1.1126	-0.8759	-1.0491	0.69
10867	*FCRLB*	4.0831	0.001156	-2.0218	-2.2809	0.69
10868	*STRA13*	1.4676	1.1152	-0.52055	-0.90268	0.69
10869	*RFX1*	1.8364	0.48166	-0.2561	-1.794	0.69
10870	*RGPD8*	2.261	0.24325	-0.44444	-2.2948	0.69
10871	*VGF*	1.2105	1.1254	-0.27755	-0.27933	0.69
10872	*GAGE1*	1.5209	0.71498	-0.17764	-0.53036	0.69
10873	*TSPAN17*	3.0709	0.31054	-1.3232	-1.5212	0.69
10874	*C1orf167*	3.3471	-0.16631	-1.1226	-1.4402	0.69
10875	*NAGK*	3.0571	-0.42399	-0.57513	-1.8714	0.69
10876	*TMSB4Y*	2.2184	0.37005	-0.53069	-1.803	0.69
10877	*FSIP1*	3.2828	0.083089	-1.3085	-2.1072	0.69
10878	*MORF4L2*	3.8671	-0.27453	-1.5356	-1.8751	0.69
10879	*RANBP6*	1.4486	1.1144	-0.50668	-2.0953	0.69
10880	*NUP50*	1.5952	1.0336	-0.57296	-0.75169	0.69
10881	*PKHD1L1*	1.2833	0.52691	0.2448	-2.217	0.69
10882	*BMS1*	4.9779	-0.93878	-1.9846	-2.1947	0.68
10883	*SMOX*	4.725	-1.3111	-1.3602	-1.7662	0.68
10884	*SCOC*	1.7704	1.1617	-0.8789	-2.5655	0.68
10885	*CHST15*	2.8622	0.98804	-1.7971	-1.9479	0.68
10886	*LYRM5*	1.6596	0.87135	-0.47911	-0.87898	0.68
10887	*GGCT*	4.6474	-1.2264	-1.3694	-2.1802	0.68
10888	*EMX2*	1.1465	1.04	-0.13748	-0.50978	0.68
10889	*ROR2*	3.4538	-0.23223	-1.1727	-1.9919	0.68
10890	*NEUROG1*	1.6305	0.87812	-0.46017	-3.4924	0.68
10891	*TSR1*	2.3906	0.88146	-1.2241	-1.4221	0.68
10892	*IQGAP3*	1.0588	0.93435	0.05456	-0.99888	0.68
10893	*SNX11*	1.3991	1.0071	-0.35889	-0.40467	0.68
10894	*NUGGC*	4.6713	-1.2145	-1.4102	-2.1187	0.68
10895	*HERC6*	2.422	0.26252	-0.63956	-1.2051	0.68

10896	*RNF126*	3.4066	-0.4433	-0.91862	-1.1989	0.68
10897	*OMD*	4.894	-1.3582	-1.492	-1.7029	0.68
10898	*HHIPL2*	1.0559	0.53068	0.45695	0.38259	0.68
10899	*GLUL*	1.6165	0.27912	0.14652	-0.43543	0.68
10900	*YBEY*	1.6013	0.31697	0.12189	-0.17554	0.68
10901	*PSMC3IP*	1.4999	0.83258	-0.29327	-0.64665	0.68
10902	*PLOD1*	3.1882	-0.20166	-0.94739	-1.329	0.68
10903	*GDF11*	2.3851	-0.16966	-0.17643	-1.4622	0.68
10904	*SENP8*	3.3178	-0.62307	-0.65695	-0.92308	0.68
10905	*BTBD10*	2.6278	-0.12995	-0.46011	-1.3617	0.68
10906	*MCF2L2*	0.97445	0.57144	0.49105	-1.1977	0.68
10907	*ABHD1*	2.5883	0.35573	-0.90713	-1.1747	0.68
10908	*KY*	3.2097	0.44703	-1.62	-2.1796	0.68
10909	*AMICA1*	3.1958	-0.24304	-0.91612	-0.95414	0.68
10910	*C1orf111*	2.0466	1.0358	-1.0466	-2.1224	0.68
10911	*DNAJC8*	3.9959	-0.54094	-1.4192	-2.2088	0.68
10912	*AHCYL1*	3.8334	-0.67137	-1.1263	-2.0629	0.68
10913	*PIGR*	2.1788	1.6705	-1.8157	-1.8645	0.68
10914	*PDE4D*	3.3015	-0.43841	-0.83005	-1.987	0.68
10915	*ADGRL2*	0.85373	0.84271	0.33605	-1.5865	0.68
10916	*SALL3*	2.6068	0.89315	-1.4683	-1.9727	0.68
10917	*LCE1C*	2.8802	0.054208	-0.90518	-1.4375	0.68
10918	*MSX2*	2.3226	-0.12189	-0.17172	-1.9953	0.68
10919	*ITGB5*	1.5346	0.67147	-0.17781	-1.6565	0.68
10920	*NFE2L3*	1.6416	0.96132	-0.57573	-2.1445	0.68
10921	*PPP1R1A*	1.5071	0.69628	-0.17622	-1.0344	0.68
10922	*CCNB1*	5.2913	-1.2845	-1.9802	-2.0687	0.68
10923	*IFNGR2*	1.9223	0.098389	0.00589	-1.3198	0.68
10924	*VKORC1*	1.2329	0.72973	0.06374	-1.6525	0.68
10925	*CES4A*	2.6632	-0.04621	-0.59168	-1.0151	0.68
10926	*PRPH2*	1.4271	1.2777	-0.68115	-1.2109	0.67
10927	*ADAM15*	1.3325	0.86771	-0.17721	-0.81883	0.67
10928	*PPP5C*	4.1671	-0.73483	-1.4094	-2.2948	0.67
10929	*TTLL12*	3.1261	-0.0484	-1.0557	-1.8157	0.67
10930	*SFT2D1*	2.0908	0.50298	-0.5718	-1.2386	0.67
10931	*JUND*	1.8488	0.85619	-0.68387	-0.9722	0.67
10932	*MPLKIP*	1.0537	0.84427	0.12307	-0.0236	0.67
10933	*C8orf89*	1.0091	0.71452	0.29731	0.24772	0.67
10934	*NEUROG2*	2.4266	0.59007	-0.99574	-1.3864	0.67
10935	*LOC400736*	1.7634	0.25978	-0.00264	-0.54121	0.67
10936	*RNF122*	2.9854	0.40145	-1.3666	-2.0146	0.67
10937	*DISC1*	2.1493	0.30113	-0.43022	-1.1531	0.67
10938	*DCXR*	1.3543	0.63608	0.02895	-0.53775	0.67
10939	*MTCH2*	1.1094	0.74739	0.16192	-0.46298	0.67
10940	*LIG1*	3.7741	-0.48659	-1.2697	-1.5167	0.67
10941	*MYCBPAP*	4.8641	-0.99146	-1.8557	-2.5292	0.67

10942	*CDS1*	1.5188	0.48991	0.00711	-1.7882	0.67
10943	*ALDH16A1*	3.2961	-0.17601	-1.1043	-1.1951	0.67
10944	*MTMR7*	1.5051	0.5186	-0.00832	-2.5461	0.67
10945	*PSORS1C1*	1.4856	0.86236	-0.33321	-0.50441	0.67
10946	*TSSK2*	2.6277	0.092625	-0.70558	-0.86267	0.67
10947	*LMBRD1*	1.2852	0.63036	0.09894	-0.92086	0.67
10948	*CFAP57*	1.6153	0.79326	-0.39481	-1.1805	0.67
10949	*ZNF286B*	2.203	0.35709	-0.54665	-0.90279	0.67
10950	*MAPKAPK2*	2.405	0.11036	-0.50246	-0.51679	0.67
10951	*LGI3*	1.3368	0.92762	-0.25227	-2.3629	0.67
10952	*GAD2*	3.6253	-0.57042	-1.0444	-1.4331	0.67
10953	*SLC17A2*	1.6179	0.2397	0.15281	-1.4303	0.67
10954	*RLIM*	2.514	0.98748	-1.4913	-1.5754	0.67
10955	*FAM117A*	1.2228	0.44215	0.34522	-1.2318	0.67
10956	*MCF2L*	1.1816	0.47688	0.35163	-1.3633	0.67
10957	*ADGRG5*	1.139	0.7937	0.07702	-2.4015	0.67
10958	*DPYS*	1.2718	0.72139	0.01577	-2.1888	0.67
10959	*TNFRSF1A*	2.9001	-0.26314	-0.62807	-1.2915	0.67
10960	*PRSS35*	4.6194	-1.0796	-1.5312	-3.1458	0.67
10961	*KHK*	3.6231	0.67406	-2.2888	-4.3274	0.67
10962	*FAM20A*	0.97824	0.92294	0.10682	-1.12	0.67
10963	*NCDN*	2.8459	-0.17525	-0.66274	-1.7652	0.67
10964	*ARL14*	1.6521	0.97726	-0.62154	-2.1478	0.67
10965	*PDZD7*	1.8899	0.62398	-0.50623	-0.78439	0.67
10966	*CEBPD*	1.1911	0.52712	0.28939	-1.1704	0.67
10967	*PRSS41*	1.3563	1.2836	-0.63248	-0.95445	0.67
10968	*SLC1A3*	1.0137	0.66215	0.33123	-1.6932	0.67
10969	*LOC102723859*	2.7311	0.003835	-0.72803	-1.7528	0.67
10970	*SKA3*	1.5874	0.25924	0.15946	-0.14145	0.67
10971	*PCYT2*	1.5275	0.69249	-0.21392	-0.73455	0.67
10972	*DSTN*	1.4704	0.32956	0.2058	-0.78444	0.67
10973	*PLEKHA6*	1.8802	0.83564	-0.71077	-1.0614	0.67
10974	*CINP*	1.3901	0.82759	-0.21291	-1.3627	0.67
10975	*GLS*	3.6119	-0.2227	-1.3851	-1.8816	0.67
10976	*PTPN6*	1.8217	0.82297	-0.64096	-2.9931	0.67
10977	*TRPT1*	2.2299	-0.08813	-0.13963	-0.72941	0.67
10978	*NOX4*	1.0497	0.85887	0.09355	-1.8568	0.67
10979	*ANGPT1*	2.6558	0.48551	-1.1402	-1.4127	0.67
10980	*ITGB6*	3.3416	-0.64654	-0.69399	-1.28	0.67
10981	*CDHR4*	1.5618	0.54202	-0.10315	-0.27407	0.67
10982	*CD160*	1.9662	0.05644	-0.02351	-0.6408	0.67
10983	*SOCS6*	1.172	0.76704	0.05866	-1.5393	0.67
10984	*KCNK2*	2.0848	1.0526	-1.1402	-1.3976	0.67
10985	*RSBN1*	2.4098	0.38242	-0.79596	-1.5635	0.67
10986	*KRTAP4-11*	1.0273	0.62858	0.34019	-1.0091	0.67
10987	*MAGEA10*	0.96453	0.68993	0.34021	-0.0881	0.66

10988	*LCMT1*	0.7618	0.75688	0.47594	-1.3689	0.66
10989	*DOHH*	1.8396	1.0106	-0.85586	-1.2833	0.66
10990	*PRKAB2*	2.8024	0.44187	-1.251	-1.2572	0.66
10991	*HSD17B10*	2.4118	0.20193	-0.62047	-2.5057	0.66
10992	*C5orf64*	2.8843	-0.20512	-0.68684	-1.2352	0.66
10993	*EMG1*	3.3194	0.21608	-1.5441	-2.0882	0.66
10994	*ESR2*	2.6255	0.16047	-0.79488	-0.99249	0.66
10995	*SPDYE5*	3.0109	-0.17719	-0.84326	-2.0414	0.66
10996	*C9orf170*	3.1502	-0.38473	-0.77597	-1.8305	0.66
10997	*RDH13*	3.9384	-0.90553	-1.0442	-1.5265	0.66
10998	*CDC123*	2.9984	0.18128	-1.1912	-1.5276	0.66
10999	*OR10A5*	2.7438	-0.08107	-0.67441	-2.1357	0.66
11000	*NOL3*	0.95207	0.85505	0.18069	-1.0318	0.66
11001	*BRD7*	0.79384	0.66953	0.52359	-0.14722	0.66
11002	*ZSCAN29*	1.1161	0.73926	0.13144	-1.3294	0.66
11003	*SGIP1*	1.8297	1.0774	-0.92087	-1.6105	0.66
11004	*PALM3*	1.8396	0.36895	-0.22235	-0.78229	0.66
11005	*KRBA2*	3.061	0.64372	-1.7188	-2.8174	0.66
11006	*PLXNA3*	1.7969	1.4897	-1.3007	-1.589	0.66
11007	*HHIPL1*	1.2004	0.93248	-0.14701	-0.55002	0.66
11008	*FOXE1*	4.0081	-0.55492	-1.4684	-1.9938	0.66
11009	*SLC6A12*	2.5975	0.023861	-0.6373	-2.9068	0.66
11010	*SFTPC*	3.8616	-0.8462	-1.0328	-1.3165	0.66
11011	*ARPP21*	2.4483	-0.14396	-0.32231	-0.37494	0.66
11012	*SMIM20*	1.4344	1.219	-0.67137	-1.2027	0.66
11013	*EIF2B4*	3.6122	-0.52061	-1.1103	-1.2312	0.66
11014	*KRTAP10-2*	1.4655	0.83745	-0.32191	-1.6564	0.66
11015	*FAM206A*	2.9682	0.49083	-1.478	-5.3559	0.66
11016	*TELO2*	3.2882	-0.59772	-0.7095	-1.4624	0.66
11017	*STAT4*	4.0024	-0.10524	-1.9179	-2.0904	0.66
11018	*LYZL2*	2.4993	-0.02722	-0.49564	-2.0863	0.66
11019	*LCN10*	2.0178	0.38763	-0.42923	-0.85389	0.66
11020	*SALL4*	2.7624	-0.20302	-0.58349	-0.72157	0.66
11021	*EML1*	1.4665	0.94074	-0.4317	-2.1834	0.66
11022	*HSP90B1*	2.6724	-0.29255	-0.40539	-1.6597	0.66
11023	*MSLN*	2.4391	-0.21258	-0.25225	-0.58336	0.66
11024	*ZNF513*	2.2057	1.1225	-1.3547	-2.0447	0.66
11025	*S1PR3*	3.6088	-0.51893	-1.1169	-1.473	0.66
11026	*PCNXL2*	3.9201	-0.79548	-1.1521	-1.4116	0.66
11027	*CANX*	1.3376	0.64786	-0.01323	-2.0653	0.66
11028	*NANOS3*	1.3665	0.34273	0.26279	-1.7078	0.66
11029	*INS-IGF2*	3.5268	-0.48911	-1.0669	-2.9354	0.66
11030	*PMEPA1*	1.8761	0.061507	0.03203	-1.9935	0.66
11031	*FOXN3*	2.0952	0.59853	-0.7241	-1.3259	0.66
11032	*CRELD2*	2.4441	0.17873	-0.65324	-2.069	0.66
11033	*C1QTNF9B-AS1*	1.6659	0.4209	-0.1178	-1.5282	0.66

11034	*CAMKK1*	1.2166	0.71224	0.04013	-0.98983	0.66
11035	*LRRC27*	0.80421	0.59287	0.57091	-0.61254	0.66
11036	*NPHP1*	2.8616	-0.023	-0.87096	-3.4957	0.66
11037	*MSANTD3-TMEFF1*	0.91989	0.734	0.31235	-0.49505	0.66
11038	*OR8B8*	1.3291	0.62527	0.01175	-0.60388	0.66
11039	*LOC101928436*	1.1484	0.96942	-0.15226	-0.72059	0.66
11040	*YOD1*	2.9427	0.064978	-1.0422	-1.0424	0.66
11041	*ELMO1*	1.1107	0.60513	0.24963	-0.64671	0.66
11042	*IBSP*	0.84181	0.60152	0.52169	-1.18	0.66
11043	*FLAD1*	1.214	0.48689	0.264	-1.7566	0.65
11044	*CD247*	2.8801	0.3663	-1.2823	-1.4926	0.65
11045	*APRT*	1.9362	0.23415	-0.20626	-0.86493	0.65
11046	*PRR20A*	1.0341	0.56483	0.36502	-1.5663	0.65
11047	*GPKOW*	4.6663	-1.0498	-1.6528	-2.1532	0.65
11048	*DCAKD*	2.8217	0.51644	-1.3762	-1.5826	0.65
11049	*ZNF143*	0.85686	0.73966	0.36468	-1.9367	0.65
11050	*SP8*	1.7247	0.38315	-0.14672	-1.0271	0.65
11051	*TMEM163*	1.0963	0.54299	0.3214	-0.30715	0.65
11052	*KRT27*	2.3902	-0.15596	-0.27379	-1.0033	0.65
11053	*HDAC6*	1.5067	0.70146	-0.24801	-1.5687	0.65
11054	*ANKRD2*	1.8578	0.4005	-0.29905	-1.3751	0.65
11055	*C2orf49*	3.2961	-0.24966	-1.0872	-1.3766	0.65
11056	*NIPAL4*	1.4963	0.58191	-0.11939	-2.103	0.65
11057	*SERPINA12*	3.5548	-0.45016	-1.1463	-1.2444	0.65
11058	*BLOC1S6*	1.3988	0.32954	0.22978	-0.80958	0.65
11059	*CCER1*	2.8738	0.18854	-1.1043	-1.3226	0.65
11060	*NUP107*	2.481	0.031866	-0.55492	-0.87328	0.65
11061	*ADCY6*	3.1408	0.087344	-1.2705	-1.4619	0.65
11062	*PCDHB10*	1.1023	0.80071	0.05429	-1.1977	0.65
11063	*NKIRAS1*	2.4507	-0.02041	-0.4735	-1.8066	0.65
11064	*CLK4*	1.7287	1.0991	-0.87128	-0.994	0.65
11065	*IL1RL1*	1.2622	1.1899	-0.49563	-0.54027	0.65
11066	*DNAJC5B*	3.3617	-0.62881	-0.77667	-3.3058	0.65
11067	*MTA2*	1.371	0.38425	0.20086	-1.8157	0.65
11068	*MT2A*	2.236	1.1931	-1.4732	-1.6243	0.65
11069	*MAFF*	3.6688	-0.44353	-1.2699	-2.8111	0.65
11070	*HLA-DQA1*	1.6894	0.52817	-0.26252	-0.3589	0.65
11071	*BRCA1*	1.5189	0.96531	-0.52944	-1.2797	0.65
11072	*PLA2G2D*	1.8043	0.38309	-0.23289	-0.66434	0.65
11073	*TLK2*	2.5209	0.49769	-1.0641	-1.6664	0.65
11074	*MESP2*	1.3413	0.86176	-0.24874	-0.97167	0.65
11075	*OR51B4*	3.1051	-0.14239	-1.0092	-2.0989	0.65
11076	*C6orf7*	2.6397	0.48764	-1.1743	-1.8642	0.65
11077	*HSD17B3*	3.0313	-0.1262	-0.95321	-2.5153	0.65
11078	*TPM1*	3.1584	0.18055	-1.3878	-1.9801	0.65
11079	*PRKCE*	2.468	0.42253	-0.93991	-1.0502	0.65

11080	*USP32*	3.2544	-0.23015	-1.0737	-1.2568	0.65
11081	*UQCRB*	3.4362	0.069884	-1.5558	-2.5103	0.65
11082	*KRTAP9-1*	2.8626	0.34469	-1.2572	-1.4136	0.65
11083	*SULT1C3*	1.0073	0.96104	-0.0185	-0.19675	0.65
11084	*APCDD1*	1.291	0.3539	0.30457	0.25927	0.65
11085	*CEP170*	2.7232	0.29504	-1.0688	-1.2246	0.65
11086	*IFNA13*	3.4714	-0.74015	-0.78314	-1.5264	0.65
11087	*RBM17*	4.1221	-0.9105	-1.2635	-1.5404	0.65
11088	*CDH18*	1.8569	0.94904	-0.858	-1.0584	0.65
11089	*TAB3*	1.6342	0.19879	0.11423	-0.52255	0.65
11090	*CPA3*	3.8913	-0.69325	-1.2509	-1.7793	0.65
11091	*LRTM1*	2.5121	0.66217	-1.2277	-1.9179	0.65
11092	*USE1*	2.3563	-0.01683	-0.39331	-1.2908	0.65
11093	*SNX18*	1.8654	0.042086	0.03839	-0.28228	0.65
11094	*TTC13*	5.3573	-1.2021	-2.2094	-2.9931	0.65
11095	*ZNF322*	2.0154	0.12367	-0.19364	-0.55764	0.65
11096	*TRIM8*	4.6276	-1.04	-1.6435	-1.7658	0.65
11097	*ZNF699*	1.5342	0.45269	-0.04308	-1.2895	0.65
11098	*GPER1*	2.5906	-0.27877	-0.36821	-1.3101	0.65
11099	*OS9*	2.5728	1.1637	-1.7935	-1.8364	0.65
11100	*SNRNP48*	2.8491	0.32631	-1.2329	-1.5795	0.65
11101	*GRXCR2*	4.0516	-0.54501	-1.5644	-1.6368	0.65
11102	*STT3B*	3.8596	-0.81515	-1.1027	-1.9321	0.65
11103	*ASB9*	3.2589	-0.06657	-1.2507	-1.6731	0.65
11104	*COL8A1*	3.2163	-0.57429	-0.70244	-1.8306	0.65
11105	*CASC10*	3.3999	-0.09423	-1.367	-1.4289	0.65
11106	*CD248*	1.4535	0.27393	0.2099	-0.50752	0.65
11107	*RRAS2*	3.5743	-0.77534	-0.86181	-2.01	0.65
11108	*MAGED1*	1.0885	0.45861	0.38996	-0.24055	0.65
11109	*PLXNA1*	2.0633	0.3822	-0.50924	-1.7845	0.65
11110	*GLCE*	2.6609	0.10103	-0.82582	-1.7466	0.65
11111	*KIF21B*	1.278	0.63205	0.02589	-1.1907	0.65
11112	*MISP*	1.8791	0.7166	-0.66008	-0.71093	0.65
11113	*CNTN1*	2.4931	0.11577	-0.67378	-1.1974	0.65
11114	*LLGL2*	5.0216	-0.74141	-2.3452	-2.6254	0.64
11115	*DBNDD2*	1.0044	0.53966	0.39053	-1.1463	0.64
11116	*ETAA1*	1.1657	0.39713	0.37148	-0.80771	0.64
11117	*ZNF484*	1.0967	0.60551	0.23201	-1.6844	0.64
11118	*SLC25A33*	1.3533	0.55443	0.02541	-2.5934	0.64
11119	*SLC22A17*	1.3658	1.1744	-0.60959	-2.0955	0.64
11120	*PAXIP1*	2.9542	-0.43702	-0.58822	-0.97565	0.64
11121	*UQCRH*	2.0902	0.75889	-0.92174	-1.0908	0.64
11122	*ARMCX6*	2.025	0.2658	-0.36412	-1.06	0.64
11123	*EFEMP2*	3.4079	-0.47282	-1.0086	-1.0191	0.64
11124	*TP53BP1*	1.7049	0.1233	0.09805	-0.17597	0.64
11125	*CT55*	1.3969	0.95444	-0.42524	-0.52643	0.64

11126	*TANGO2*	1.737	0.87673	-0.68766	-0.74768	0.64
11127	*EFCAB3*	3.3168	0.81982	-2.2108	-2.2456	0.64
11128	*ARPC2*	1.1498	0.46726	0.30819	-0.77997	0.64
11129	*ERP44*	1.9726	1.0437	-1.0912	-1.3573	0.64
11130	*TRAF3*	2.5213	0.42168	-1.0187	-1.1084	0.64
11131	*GOLGA8O*	2.2015	0.30668	-0.58403	-1.9919	0.64
11132	*RALA*	2.575	-0.00268	-0.64851	-1.6208	0.64
11133	*ZSWIM6*	1.1399	0.60184	0.18203	-3.7736	0.64
11134	*OXCT1*	1.383	0.43228	0.10748	-0.45572	0.64
11135	*LRIT1*	1.0849	0.5982	0.23887	0.10206	0.64
11136	*C6orf203*	2.2672	0.49542	-0.84069	-1.6435	0.64
11137	*CD2*	1.1739	0.88085	-0.13339	-1.8321	0.64
11138	*PWP1*	1.485	0.75512	-0.31884	-1.4385	0.64
11139	*DCAF16*	1.3021	0.32886	0.28896	-1.0054	0.64
11140	*MYSM1*	2.3371	0.16551	-0.58305	-3.2419	0.64
11141	*ARTN*	3.6806	-0.79788	-0.96572	-2.4112	0.64
11142	*ZNF91*	1.7282	1.0742	-0.88547	-1.42	0.64
11143	*RGPD4*	1.369	0.49817	0.04928	-0.17013	0.64
11144	*ATL3*	3.4915	-0.31808	-1.2573	-1.6769	0.64
11145	*ATG4D*	3.9089	-0.17714	-1.8157	-2.072	0.64
11146	*TSPY10*	3.0744	-0.39423	-0.76457	-1.7566	0.64
11147	*FBXW5*	2.3337	0.28617	-0.70618	-2.1523	0.64
11148	*KAT6B*	3.7577	-0.37544	-1.4686	-1.8885	0.64
11149	*PEG3*	2.0321	0.99015	-1.1096	-1.6001	0.64
11150	*MAGEB5*	2.5052	0.53673	-1.1298	-1.522	0.64
11151	*FABP7*	2.4793	0.4888	-1.0564	-1.8033	0.64
11152	*LY6E*	1.2195	0.42698	0.26472	-1.0986	0.64
11153	*ATF7*	3.0096	-0.53947	-0.55993	-0.81943	0.64
11154	*MAGOH*	1.7762	1.4073	-1.2737	-1.4474	0.64
11155	*MYADM*	4.5038	-0.76049	-1.8341	-2.0574	0.64
11156	*HPS3*	1.9287	0.21027	-0.22977	-0.94583	0.64
11157	*OR1N1*	1.2348	0.85468	-0.18043	-1.6143	0.64
11158	*NCR1*	3.6737	-0.85296	-0.91221	-1.3542	0.64
11159	*F2RL3*	2.6218	1.0925	-1.806	-1.8999	0.64
11160	*IFNA5*	2.9411	-0.16888	-0.86403	-1.8792	0.64
11161	*CRYZ*	1.7463	0.27022	-0.10861	-2.0476	0.64
11162	*TK2*	2.9962	-0.1372	-0.95119	-1.9125	0.64
11163	*PCDHGA10*	1.2094	0.40298	0.29519	-1.8523	0.64
11164	*RBBP6*	2.9965	0.16057	-1.25	-2.1068	0.64
11165	*NUP210L*	1.9951	0.94688	-1.0358	-1.4329	0.64
11166	*C3orf38*	2.3391	0.49378	-0.92823	-1.9953	0.63
11167	*XIRP1*	1.5726	0.32967	0.00158	-1.4455	0.63
11168	*OAS1*	4.4055	-1.2226	-1.2792	-1.4081	0.63
11169	*KANK4*	1.4199	0.69407	-0.21079	-0.81034	0.63
11170	*A4GALT*	3.6443	-0.82897	-0.91236	-4.2477	0.63
11171	*WDR55*	0.99996	0.49335	0.4092	-0.95842	0.63

11172	*TTC9*	1.3308	1.2816	-0.71009	-0.83497	0.63
11173	*FTMT*	1.0703	0.79764	0.03435	-0.03422	0.63
11174	*PLAGL1*	3.4298	-0.5639	-0.96463	-1.0933	0.63
11175	*RGS16*	4.1995	-0.70942	-1.5901	-1.9326	0.63
11176	*YBX3*	2.1931	1.2476	-1.5408	-1.7158	0.63
11177	*TMC2*	1.4328	1.0457	-0.57899	-1.5568	0.63
11178	*CDH12*	1.267	0.99902	-0.36705	-1.451	0.63
11179	*CXCR4*	1.3913	0.52992	-0.02242	-1.8877	0.63
11180	*ZNF735*	3.6384	-0.53589	-1.2051	-1.6474	0.63
11181	*C12orf45*	4.4433	-0.99604	-1.55	-1.8324	0.63
11182	*ATP8B2*	0.92266	0.72637	0.24793	-0.94777	0.63
11183	*MKX*	2.8841	0.098319	-1.0863	-1.5073	0.63
11184	*FAM19A5*	4.2843	-0.64632	-1.742	-2.3795	0.63
11185	*OR56A4*	1.4017	0.85667	-0.36467	-2.3178	0.63
11186	*CAND2*	2.4534	0.44249	-1.0028	-1.337	0.63
11187	*PABPC1L2B*	2.4747	0.48959	-1.0718	-1.2139	0.63
11188	*C6orf10*	0.95467	0.68352	0.25425	-0.04036	0.63
11189	*TMEM203*	3.6932	-0.60183	-1.1995	-1.2635	0.63
11190	*AMER2*	1.9725	0.39392	-0.47485	-1.072	0.63
11191	*KIF24*	3.4734	-0.44353	-1.1391	-1.9844	0.63
11192	*NECAB2*	1.8029	0.06743	0.02021	-0.10273	0.63
11193	*INTS6*	2.0989	0.35226	-0.56079	-1.5712	0.63
11194	*GPR4*	2.905	0.23041	-1.2457	-1.4181	0.63
11195	*CYP51A1*	2.9056	-0.33657	-0.68021	-1.3096	0.63
11196	*ZNF804A*	4.1854	-0.59662	-1.7001	-2.0791	0.63
11197	*PDPK1*	4.3047	-1.1968	-1.2195	-1.7316	0.63
11198	*C11orf58*	3.3287	-0.53254	-0.90824	-1.2159	0.63
11199	*TFAP2E*	0.9693	0.47553	0.44254	-0.67546	0.63
11200	*CMC4*	3.861	-0.58349	-1.3905	-2.0197	0.63
11201	*TMEM5*	1.9715	0.3605	-0.44547	-1.0068	0.63
11202	*DDX59*	1.1667	1.1422	-0.42248	-2.3435	0.63
11203	*SLC7A11*	1.7486	0.19445	-0.05663	-0.3744	0.63
11204	*PCSK9*	1.3598	0.62749	-0.10176	-0.1339	0.63
11205	*SNX33*	2.5597	-0.02542	-0.64888	-1.6587	0.63
11206	*NMI*	2.0459	0.25529	-0.41596	-0.42369	0.63
11207	*ITGB2*	3.94	-0.6764	-1.3787	-1.4206	0.63
11208	*TMEM253*	2.5835	0.2881	-0.98749	-1.0633	0.63
11209	*FBXO24*	3.2028	-0.55278	-0.76606	-2.103	0.63
11210	*ATRIP*	1.5857	1.0157	-0.71746	-1.4501	0.63
11211	*RABGGTB*	2.7312	-0.09828	-0.75049	-1.989	0.63
11212	*PDLIM3*	1.0691	0.87312	-0.06044	-1.1631	0.63
11213	*PCDHB1*	1.9243	0.55379	-0.59703	-1.1071	0.63
11214	*LAMP3*	1.4039	0.71221	-0.23523	-3.2089	0.63
11215	*ZNF7*	1.7542	0.25601	-0.12957	-0.46614	0.63
11216	*CD36*	2.0102	0.32484	-0.45449	-1.4385	0.63
11217	*PARVB*	2.6815	0.29391	-1.0951	-1.1114	0.63

11218	*ARHGEF17*	2.8126	-0.15013	-0.78229	-1.7585	0.63
11219	*TAS2R43*	2.613	0.15359	-0.88702	-1.2169	0.63
11220	*MYPN*	1.5658	0.96819	-0.65503	-1.2743	0.63
11221	*TRIM43*	1.6247	0.21814	0.03541	-0.15476	0.63
11222	*XK*	1.3776	0.70759	-0.2073	-2.221	0.63
11223	*ITGAM*	2.2041	0.18203	-0.50911	-1.0947	0.63
11224	*UQCRHL*	2.7463	-0.42564	-0.44417	-1.3366	0.63
11225	*NUDT15*	1.5169	0.74008	-0.38161	-1.4951	0.63
11226	*CARS*	1.7802	0.72606	-0.63157	-1.1075	0.62
11227	*C11orf68*	1.1929	1.0541	-0.37275	-1.0728	0.62
11228	*PALMD*	2.1438	0.82008	-1.0898	-1.1126	0.62
11229	*FOXB2*	1.0487	0.66663	0.15852	-0.17745	0.62
11230	*MAP2K5*	2.3079	-0.0706	-0.36427	-0.9879	0.62
11231	*TMEM101*	0.76637	0.57476	0.5309	-0.96788	0.62
11232	*POLA1*	4.0439	0.01398	-2.1859	-2.6635	0.62
11233	*TMPRSS11E*	1.8712	0.3341	-0.33336	-0.43005	0.62
11234	*GPATCH1*	2.5413	-0.24665	-0.42373	-2.8019	0.62
11235	*C9orf85*	1.1614	0.70917	-4.4E-05	-0.10184	0.62
11236	*SPEF2*	1.7297	0.082695	0.05813	-0.33397	0.62
11237	*URAD*	2.5281	0.18586	-0.84348	-0.95658	0.62
11238	*PDE12*	1.3783	0.34219	0.14984	-0.5687	0.62
11239	*MPHOSPH8*	2.3584	-0.02915	-0.45906	-0.88274	0.62
11240	*GREB1*	1.998	0.40461	-0.53254	-1.2362	0.62
11241	*MAGEB6*	3.9122	-0.2367	-1.8057	-2.2004	0.62
11242	*PRSS23*	2.4817	0.19661	-0.80909	-1.6403	0.62
11243	*C19orf70*	1.0136	0.86372	-0.00826	-1.5167	0.62
11244	*ZGLP1*	2.6967	0.2105	-1.0393	-1.0399	0.62
11245	*MT4*	2.5834	-0.13072	-0.58485	-1.6289	0.62
11246	*LRRC39*	2.1375	0.61068	-0.88098	-2.1984	0.62
11247	*FASLG*	3.198	-0.63753	-0.69365	-1.1536	0.62
11248	*GOLIM4*	1.6668	0.10682	0.09241	-1.6763	0.62
11249	*C1S*	1.0246	0.45188	0.38954	-0.49322	0.62
11250	*TMEM135*	1.5809	0.56676	-0.28197	-0.55492	0.62
11251	*SSBP2*	1.7844	0.91836	-0.83718	-2.4527	0.62
11252	*SBK1*	3.3208	0.13715	-1.5949	-2.7838	0.62
11253	*ARL17B*	3.9853	-0.93474	-1.1887	-3.2616	0.62
11254	*TM9SF3*	1.173	0.70104	-0.01292	-0.59739	0.62
11255	*FHL3*	1.7782	0.079734	0.00312	-0.31052	0.62
11256	*SMNDC1*	2.0842	0.91674	-1.1407	-2.1811	0.62
11257	*CSN2*	4.0184	-1.0434	-1.1152	-1.5458	0.62
11258	*VSIG1*	1.9272	0.75503	-0.82447	-1.6641	0.62
11259	*ARHGEF10*	1.7925	0.73512	-0.67029	-1.1449	0.62
11260	*NKG7*	1.3614	0.35399	0.14194	-0.40605	0.62
11261	*NFX1*	2.0501	0.25796	-0.45119	-1.6867	0.62
11262	*HIST1H2BJ*	4.1269	-0.79611	-1.4747	-1.5054	0.62
11263	*THEMIS2*	1.6593	0.14294	0.05361	-0.04952	0.62

11264	*MRPS31*	3.088	-0.53987	-0.69287	-0.78443	0.62
11265	*CSTF2*	1.2619	0.4209	0.17242	-1.57	0.62
11266	*PLCXD1*	2.9019	0.62388	-1.6711	-2.672	0.62
11267	*TMEM80*	0.89643	0.6201	0.33774	-0.84897	0.62
11268	*MYL6*	1.5776	0.16383	0.11254	-1.2476	0.62
11269	*RMDN3*	3.5222	-0.40398	-1.2646	-1.5671	0.62
11270	*CTU1*	2.7606	0.30735	-1.2145	-1.606	0.62
11271	*PRSS1*	2.7407	-0.22589	-0.66194	-1.8443	0.62
11272	*ELK1*	3.6535	-0.75974	-1.0424	-1.2936	0.62
11273	*ANAPC11*	3.2404	-0.64961	-0.73984	-1.7228	0.62
11274	*VPS26B*	1.1764	0.56009	0.11435	-1.6975	0.62
11275	*SLC22A13*	2.8792	-0.24036	-0.78823	-2.0483	0.62
11276	*PPM1D*	1.9913	0.29421	-0.435	-1.1049	0.62
11277	*PON1*	1.1879	1.1061	-0.44353	-1.5138	0.62
11278	*SFMBT1*	2.23	-0.17311	-0.20771	-1.2867	0.62
11279	*ZKSCAN2*	2.0265	-0.07997	-0.09757	-1.77	0.62
11280	*LRRFIP1*	3.019	0.21771	-1.3887	-1.6982	0.62
11281	*OR4K1*	1.497	0.29421	0.0566	-0.87318	0.62
11282	*CLDN25*	0.9357	0.93071	-0.01938	-2.075	0.62
11283	*ZFP2*	1.5732	1.1359	-0.86223	-1.2277	0.62
11284	*RBM41*	0.64116	0.62108	0.58461	-1.1448	0.62
11285	*STEAP4*	0.90512	0.58434	0.3573	-1.1962	0.62
11286	*SLC27A3*	2.112	0.37416	-0.64033	-1.1046	0.62
11287	*GPR65*	1.7252	0.36032	-0.24036	-1.2719	0.62
11288	*RTKN2*	1.9774	-0.01352	-0.11897	-0.91913	0.61
11289	*PARP8*	1.4079	0.22239	0.21452	-1.0985	0.61
11290	*SCN3B*	2.0778	0.21027	-0.44353	-1.0669	0.61
11291	*NLRP2*	3.3636	-0.52811	-0.99137	-1.3125	0.61
11292	*TTC23L*	3.2732	0.15831	-1.5884	-2.8402	0.61
11293	*FDX1*	3.5636	-0.2868	-1.4337	-1.479	0.61
11294	*C16orf82*	1.2292	1.0569	-0.44353	-1.6526	0.61
11295	*TAS2R60*	1.9048	1.0134	-1.078	-1.8963	0.61
11296	*PSMA7*	2.499	-0.18074	-0.47862	-1.5171	0.61
11297	*TMEM169*	1.9219	0.62281	-0.7061	-1.7247	0.61
11298	*FAM120B*	1.0441	0.66344	0.13093	-0.14548	0.61
11299	*FAM24B*	1.4105	0.86615	-0.43845	-0.62306	0.61
11300	*DNALI1*	2.2585	0.098877	-0.5193	-0.56403	0.61
11301	*METTL20*	2.1259	0.001801	-0.28972	-0.44198	0.61
11302	*EWSR1*	3.716	-0.88905	-0.98915	-1.6524	0.61
11303	*TNFAIP8L3*	3.1096	0.50816	-1.7813	-1.916	0.61
11304	*SERPING1*	2.5592	-0.10044	-0.62306	-0.94422	0.61
11305	*KNCN*	2.5057	-0.27843	-0.3921	-0.60845	0.61
11306	*P2RY11*	1.323	0.54063	-0.02974	-1.144	0.61
11307	*CABP5*	1.7122	0.33657	-0.21526	-1.1641	0.61
11308	*ZMYM6*	0.87899	0.65764	0.29629	-1.729	0.61
11309	*PTGDR2*	2.1938	0.48872	-0.8504	-1.0945	0.61

11310	*DAO*	1.9338	0.4358	-0.53775	-1.3173	0.61
11311	*TNP1*	3.4568	-0.78956	-0.83627	-1.8698	0.61
11312	*DNAJC22*	2.7662	-0.08018	-0.85531	-1.6799	0.61
11313	*MARVELD3*	3.3909	-0.301	-1.2595	-1.8398	0.61
11314	*GPAT2*	3.3098	-0.16885	-1.3112	-1.3218	0.61
11315	*PATZ1*	1.1451	1.0922	-0.4082	-1.6626	0.61
11316	*PRR14*	2.3577	0.46888	-0.99783	-1.2076	0.61
11317	*OR4D5*	1.3485	0.2735	0.20651	-0.31052	0.61
11318	*UBXN8*	3.1715	-0.44353	-0.89957	-1.7395	0.61
11319	*UCN3*	1.1258	0.45741	0.24469	-0.18432	0.61
11320	*PGAP3*	1.8759	0.12888	-0.17746	-0.96632	0.61
11321	*PCDHB5*	1.9869	0.20227	-0.36295	-0.67482	0.61
11322	*TMPRSS4*	2.0637	0.80675	-1.0443	-1.3377	0.61
11323	*P4HTM*	3.1883	-0.10479	-1.2576	-2.5198	0.61
11324	*AADAT*	2.5362	0.086847	-0.79875	-1.0529	0.61
11325	*MTFMT*	1.1645	0.43614	0.22329	-2.1921	0.61
11326	*ZC3H18*	1.7893	0.78918	-0.75491	-1.4563	0.61
11327	*KRT86*	1.8642	0.76409	-0.80498	-1.856	0.61
11328	*BMPR2*	2.1461	0.25432	-0.57763	-0.58054	0.61
11329	*MRPL9*	1.9183	-0.03405	-0.06163	-0.08357	0.61
11330	*NUDT10*	1.4194	0.54633	-0.14312	-1.0373	0.61
11331	*AGRP*	1.7676	0.037191	0.01713	-0.23678	0.61
11332	*TNFAIP8L2-SCNM1*	0.89994	0.62785	0.29405	-0.11049	0.61
11333	*ARHGAP23*	1.7132	1.6375	-1.5294	-2.5806	0.61
11334	*COQ10A*	2.9236	-0.44353	-0.65906	-2.4141	0.61
11335	*USP17L3*	3.6002	-0.57558	-1.2037	-1.2352	0.61
11336	*METTL22*	1.9718	0.19156	-0.34259	-0.61941	0.61
11337	*CNIH2*	2.7359	0.11643	-1.0331	-1.1504	0.61
11338	*FASTK*	1.613	0.27818	-0.07199	-0.31885	0.61
11339	*PDE3A*	1.7964	1.5896	-1.5671	-3.3136	0.61
11340	*UFSP2*	4.3284	-1.2356	-1.2745	-1.8646	0.61
11341	*C5orf63*	2.5046	0.52671	-1.2142	-2.6072	0.61
11342	*ZBTB9*	2.2552	-0.21111	-0.22803	-2.4221	0.61
11343	*ZNF28*	2.5652	-0.19851	-0.55068	-0.74572	0.61
11344	*CHST12*	2.1382	0.69694	-1.0197	-1.7474	0.61
11345	*PCSK1N*	1.5205	0.77104	-0.47692	-2.1324	0.60
11346	*IKZF2*	3.8879	-0.68565	-1.3892	-1.5375	0.60
11347	*LEKR1*	2.1353	0.21571	-0.53815	-1.2801	0.60
11348	*TTLL1*	4.9397	-1.2756	-1.8527	-2.3357	0.60
11349	*SGTA*	3.3991	-0.44353	-1.1442	-1.7322	0.60
11350	*HS6ST1*	2.1613	0.95397	-1.3044	-1.7435	0.60
11351	*GRIN3A*	0.71031	0.56364	0.53647	-1.808	0.60
11352	*TMPRSS12*	3.0641	-0.40564	-0.85002	-1.3961	0.60
11353	*KCNG4*	2.8048	0.20881	-1.2058	-1.2127	0.60
11354	*SPAG1*	1.5	1.219	-0.91134	-0.9599	0.60
11355	*ZNF765*	2.6222	-0.34443	-0.47018	-1.5116	0.60

11356	*MGAT4A*	4.5395	-1.3554	-1.3767	-1.5086	0.60
11357	*CLEC4C*	2.0862	-0.13743	-0.14198	-1.2226	0.60
11358	*ACOT6*	3.7662	-0.59239	-1.3674	-2.2508	0.60
11359	*ZSCAN9*	4.0179	-0.22769	-1.984	-2.0458	0.60
11360	*DCTN1*	3.7324	-0.78221	-1.144	-2.5255	0.60
11361	*DDX19B*	2.5708	0.59245	-1.3585	-1.3963	0.60
11362	*CARD16*	2.5857	0.27104	-1.0528	-2.0407	0.60
11363	*CDH23*	4.196	-1.0824	-1.3098	-1.7443	0.60
11364	*PIGZ*	0.97252	0.93061	-0.09956	-0.47685	0.60
11365	*IRS2*	1.1312	0.74124	-0.06902	-1.6368	0.60
11366	*ENGASE*	2.197	-0.03288	-0.36086	-0.98158	0.60
11367	*GPATCH4*	2.3432	0.12678	-0.66989	-1.9022	0.60
11368	*OBFC1*	1.4554	0.36854	-0.02406	-0.06876	0.60
11369	*ISCU*	1.1418	0.81513	-0.15771	-0.77667	0.60
11370	*FAM72B*	5.4301	-1.0654	-2.5655	-2.8928	0.60
11371	*MAGEB3*	1.3562	0.35324	0.08942	-2.9538	0.60
11372	*AQP2*	2.9082	0.18797	-1.2975	-2.0838	0.60
11373	*OR1J2*	3.3964	-0.44287	-1.155	-1.632	0.60
11374	*RAD51B*	3.0213	-0.22492	-0.99888	-1.6494	0.60
11375	*S100A12*	0.9712	0.48113	0.34516	-0.00564	0.60
11376	*RNASE2*	1.438	0.3037	0.05569	-0.29673	0.60
11377	*LYVE1*	3.3176	-0.33958	-1.1807	-3.1397	0.60
11378	*SLC13A4*	1.4574	0.65496	-0.31544	-0.97356	0.60
11379	*GOLGA6L9*	2.8082	0.86983	-1.882	-2.3557	0.60
11380	*LRRC72*	2.55	0.29818	-1.0528	-1.2643	0.60
11381	*ZNF816-ZNF321P*	3.5525	-0.37045	-1.3878	-3.0961	0.60
11382	*RBMS2*	1.7261	0.45012	-0.38219	-0.83808	0.60
11383	*FBXO10*	2.6889	-0.16485	-0.73061	-1.6691	0.60
11384	*ZNF608*	4.0523	-0.69678	-1.5634	-1.9397	0.60
11385	*PEX12*	3.8993	0.28628	-2.3936	-4.8617	0.60
11386	*TMEM215*	3.3631	-0.2192	-1.3523	-1.5898	0.60
11387	*LOC728485*	1.1222	0.76	-0.09104	-0.79225	0.60
11388	*LEAP2*	1.7569	0.36498	-0.33205	-3.5362	0.60
11389	*CCSER2*	2.1583	0.25237	-0.62125	-3.1806	0.60
11390	*NR5A1*	3.1113	-0.56773	-0.75433	-1.8575	0.60
11391	*MRPS14*	2.1769	-0.12108	-0.26893	-2.1791	0.60
11392	*PAAF1*	0.83715	0.8086	0.14066	-0.7602	0.60
11393	*UBE2M*	3.3411	-0.77241	-0.78229	-2.2712	0.60
11394	*SMARCC1*	1.8051	0.098994	-0.11826	-2.4889	0.60
11395	*TAAR2*	4.4817	0.066478	-2.7626	-5.632	0.60
11396	*CDC5L*	2.1828	0.53982	-0.93898	-1.8692	0.59
11397	*SFTPA1*	1.3912	0.35112	0.04102	-1.0102	0.59
11398	*CCDC180*	1.8942	0.17683	-0.28825	-1.3377	0.59
11399	*RPP25*	2.4409	-0.19176	-0.46721	-1.1752	0.59
11400	*RBM47*	2.328	0.14603	-0.69223	-0.7443	0.59
11401	*NDST4*	1.7952	0.099701	-0.11448	-1.7766	0.59

11402	*NDUFB2*	4.1012	-0.2159	-2.1055	-3.4314	0.59
11403	*SNX31*	2.2124	-0.06253	-0.3703	-0.75511	0.59
11404	*ENAM*	1.275	0.28082	0.22369	-1.1512	0.59
11405	*FAM179B*	1.2908	0.47234	0.0155	-1.2673	0.59
11406	*EFCAB11*	3.5638	-0.40427	-1.3814	-2.0055	0.59
11407	*SOHLH1*	3.5424	-0.08417	-1.6802	-3.0179	0.59
11408	*GPHA2*	2.4456	-0.00464	-0.66309	-1.2293	0.59
11409	*TTC1*	1.6207	0.23635	-0.08183	-1.1326	0.59
11410	*PRKACA*	4.0714	-0.8892	-1.4076	-1.7595	0.59
11411	*PCDHB13*	2.9067	0.25958	-1.3932	-1.5348	0.59
11412	*OCM2*	1.2068	0.35535	0.21077	-1.4442	0.59
11413	*RPL29*	2.5557	-0.07036	-0.71312	-1.9758	0.59
11414	*ALS2CL*	3.9312	-0.41373	-1.7457	-1.9559	0.59
11415	*ECT2*	2.0385	0.58978	-0.85871	-2.397	0.59
11416	*PRF1*	4.1276	-1.065	-1.2935	-2.1798	0.59
11417	*AATF*	3.4872	-0.38348	-1.3349	-1.4433	0.59
11418	*DEFB125*	1.3964	1.26	-0.88784	-1.5901	0.59
11419	*CCDC13*	1.2893	0.55041	-0.07257	-0.78337	0.59
11420	*DDR1*	1.7814	0.087849	-0.10247	-1.1552	0.59
11421	*ZNF628*	2.1957	0.021939	-0.45122	-1.2903	0.59
11422	*DENND1B*	2.8727	0.31877	-1.4256	-2.0706	0.59
11423	*CASC3*	2.8008	0.11584	-1.151	-1.2784	0.59
11424	*RAF1*	1.985	0.84128	-1.0611	-1.6208	0.59
11425	*SLC37A3*	2.7316	-0.47383	-0.49266	-0.70952	0.59
11426	*ADCK2*	1.4572	0.20314	0.10413	-1.2321	0.59
11427	*C10orf82*	1.7241	0.27119	-0.23297	-0.3794	0.59
11428	*BCLAF1*	3.6451	-0.46745	-1.4154	-2.6778	0.59
11429	*NALCN*	2.2345	-0.02895	-0.44353	-0.78842	0.59
11430	*AQP12B*	1.3852	0.35071	0.02416	0.015107	0.59
11431	*ASB13*	2.813	-0.11049	-0.94255	-1.3801	0.59
11432	*CSNK2B*	3.2166	-0.7057	-0.75128	-1.1108	0.59
11433	*ERCC2*	3.8633	-0.60003	-1.5038	-1.9125	0.59
11434	*UCN2*	2.9486	-0.43385	-0.75529	-0.84151	0.59
11435	*SLC20A1*	1.071	0.39803	0.28926	-0.41266	0.59
11436	*CLTC*	1.1451	0.51436	0.09832	-0.49941	0.59
11437	*ELOVL3*	1.118	0.33851	0.29991	0.065984	0.59
11438	*FAM208A*	2.2759	-0.03135	-0.48862	-0.70327	0.59
11439	*PHTF1*	3.2855	0.037238	-1.5671	-1.8822	0.59
11440	*ABI3BP*	3.9434	-1.0315	-1.1566	-2.5008	0.59
11441	*KRTAP9-7*	1.7323	0.71166	-0.68869	-1.6413	0.59
11442	*FAM3A*	2.5059	-0.22444	-0.52637	-0.8071	0.59
11443	*LAT2*	1.944	0.41368	-0.60315	-2.033	0.58
11444	*PRR9*	1.9239	1.1669	-1.3382	-1.615	0.58
11445	*FRMD5*	0.6911	0.56421	0.49693	0.061693	0.58
11446	*TMEM126B*	1.7679	0.22609	-0.2429	-0.72666	0.58
11447	*SCPEP1*	2.889	-0.03675	-1.1012	-1.2411	0.58

11448	*FCGRT*	4.1822	-0.60845	-1.8227	-2.101	0.58
11449	*WDR90*	2.3469	-0.0351	-0.56132	-1.4954	0.58
11450	*PCDHGC5*	3.2782	-0.38077	-1.147	-2.684	0.58
11451	*LGALS8*	1.1346	0.52061	0.09518	-0.29314	0.58
11452	*CLEC1A*	0.70679	0.62558	0.41801	-1.6221	0.58
11453	*ACSS1*	1.2654	0.28864	0.19604	-0.25941	0.58
11454	*CCDC17*	0.76144	0.5341	0.45442	-0.52343	0.58
11455	*CD1C*	1.3721	0.67787	-0.30029	-0.48916	0.58
11456	*OXGR1*	1.1011	0.57714	0.07062	-6.2991	0.58
11457	*CDX2*	1.5502	0.13826	0.0603	-1.061	0.58
11458	*TAL2*	0.6052	0.60333	0.53974	-0.49564	0.58
11459	*LAIR2*	2.725	0.75545	-1.7322	-2.0351	0.58
11460	*PRKX*	1.7134	0.50036	-0.46749	-0.92486	0.58
11461	*LOC389602*	4.3648	-1.2324	-1.3865	-1.4294	0.58
11462	*WDFY1*	3.231	-0.36549	-1.1207	-2.7705	0.58
11463	*ENPP2*	0.86459	0.49513	0.38487	-0.30457	0.58
11464	*BANP*	3.3334	-0.36047	-1.2284	-1.5305	0.58
11465	*AKR1C4*	2.4331	0.10535	-0.79541	-1.226	0.58
11466	*ANGEL1*	1.6879	1.0031	-0.94859	-1.7247	0.58
11467	*CCDC88B*	1.9559	0.004523	-0.21821	-0.87768	0.58
11468	*ARHGAP32*	3.461	-0.43841	-1.2815	-1.9727	0.58
11469	*WFIKKN2*	1.0008	0.92231	-0.18214	-1.2379	0.58
11470	*SLC52A2*	1.3212	0.86765	-0.44814	-1.4061	0.58
11471	*KRTAP5-10*	2.0155	0.048185	-0.32355	-0.87109	0.58
11472	*LGALS3*	1.5695	0.39642	-0.22593	-0.59557	0.58
11473	*OR4F5*	1.6987	0.81253	-0.77196	-2.2996	0.58
11474	*TRMT12*	3.6209	-0.31976	-1.5631	-1.8849	0.58
11475	*ZFAND6*	3.8379	-0.66215	-1.4392	-3.1939	0.58
11476	*ABCC4*	3.3009	-0.66761	-0.89699	-1.5415	0.58
11477	*SPATA31D3*	1.0905	0.72896	-0.08529	-1.072	0.58
11478	*ALG1*	3.8166	-0.70394	-1.3785	-1.6206	0.58
11479	*PGAM4*	4.2053	-1.1866	-1.285	-1.6921	0.58
11480	*SLC39A3*	1.0121	0.66519	0.05607	-0.52426	0.58
11481	*SLC1A6*	2.6735	0.18287	-1.1241	-1.6526	0.58
11482	*KANK2*	1.1487	1.0925	-0.5101	-1.7512	0.58
11483	*DIS3L2*	0.74645	0.62542	0.35805	0.08651	0.58
11484	*POMT1*	1.4469	1.107	-0.82415	-1.2532	0.58
11485	*FSCN2*	1.2011	0.76053	-0.23203	-2.8995	0.58
11486	*PEX14*	1.4816	0.70038	-0.45296	-0.79169	0.58
11487	*ARSF*	2.3821	-0.10831	-0.5451	-1.0632	0.58
11488	*KMT2A*	1.7004	1.1449	-1.1169	-1.4428	0.58
11489	*ZNF581*	2.2989	0.21297	-0.78362	-1.3837	0.58
11490	*LHPP*	2.1587	0.78047	-1.211	-1.5901	0.58
11491	*CD40*	3.4662	-0.31469	-1.4234	-1.5824	0.58
11492	*S100A9*	1.6095	0.32681	-0.20892	-0.94536	0.58
11493	*MYBL2*	2.4546	-0.28819	-0.43931	-2.2732	0.58

11494	*SLC2A10*	2.5838	0.38754	-1.2444	-1.4713	0.58
11495	*HNRNPF*	3.2058	-0.11232	-1.3666	-2.7056	0.58
11496	*OPA1*	1.2783	0.98148	-0.53304	-2.9494	0.58
11497	*BEND4*	1.4767	0.31276	-0.06276	-0.09139	0.58
11498	*MFSD2B*	3.4005	-0.3098	-1.3644	-1.7806	0.58
11499	*POMT2*	0.86661	0.6755	0.1825	-1.7577	0.57
11500	*TRIM64C*	1.7336	0.70297	-0.7122	-0.90296	0.57
11501	*MINOS1*	3.453	-0.80213	-0.92664	-1.2422	0.57
11502	*STARD8*	1.8326	0.051282	-0.1604	-1.8066	0.57
11503	*LYZL1*	2.6708	-0.12998	-0.81765	-1.197	0.57
11504	*TNXB*	3.4487	-0.50961	-1.2165	-1.5965	0.57
11505	*IQSEC1*	1.8264	0.17543	-0.27961	-1.658	0.57
11506	*AASDHPPT*	3.308	-0.41319	-1.1726	-1.3627	0.57
11507	*KRT33B*	0.96075	0.63684	0.12445	-0.87898	0.57
11508	*BRI3*	2.0783	0.36404	-0.72128	-1.4572	0.57
11509	*RCN1*	2.7454	-0.11033	-0.91429	-2.4564	0.57
11510	*SSTR2*	1.0384	0.54045	0.14176	-0.94085	0.57
11511	*MYH14*	2.3741	-0.13429	-0.51929	-1.9727	0.57
11512	*ZUFSP*	1.1409	0.29605	0.28305	-2.5754	0.57
11513	*TBC1D3K*	3.392	-0.76355	-0.90854	-1.6876	0.57
11514	*ZNF263*	1.3498	0.42059	-0.05081	-2.1766	0.57
11515	*CHRNA2*	3.733	-0.45069	-1.5633	-2.3065	0.57
11516	*ATXN1L*	2.8426	-0.30056	-0.82307	-0.90303	0.57
11517	*TES*	1.7067	0.71653	-0.70647	-0.9285	0.57
11518	*SLC35E1*	4.1556	-0.726	-1.714	-2.163	0.57
11519	*UTP20*	1.9367	0.12616	-0.34846	-1.0553	0.57
11520	*CNDP1*	2.4155	0.22886	-0.93057	-1.3561	0.57
11521	*RCAN3*	0.94071	0.77423	-0.00153	-1.2474	0.57
11522	*BMP2*	2.9994	0.26209	-1.5491	-1.9442	0.57
11523	*IFT81*	1.7957	0.6425	-0.72825	-0.74586	0.57
11524	*SCGB2A1*	1.3514	0.32745	0.03061	-0.1624	0.57
11525	*CCDC54*	3.3334	-0.80879	-0.81542	-2.42	0.57
11526	*EFS*	2.0954	-0.10888	-0.27744	-1.2064	0.57
11527	*UPK2*	2.7052	0.13547	-1.1332	-1.5996	0.57
11528	*SBK3*	0.60799	0.60149	0.49793	-1.0997	0.57
11529	*DAPK1*	1.4841	1.1289	-0.90588	-1.3496	0.57
11530	*TREML1*	1.7783	0.32653	-0.39791	-0.60109	0.57
11531	*ZNF135*	1.1557	0.65662	-0.10547	-1.0232	0.57
11532	*ADH4*	1.1391	0.82808	-0.26125	-1.0949	0.57
11533	*ATIC*	3.1901	-0.00827	-1.4764	-2.537	0.57
11534	*PPP1R35*	1.7124	1.5305	-1.5376	-1.7072	0.57
11535	*TPRN*	2.4938	0.27856	-1.0697	-1.8038	0.57
11536	*PATE1*	1.1972	0.58517	-0.07997	-0.86861	0.57
11537	*NRTN*	2.9033	0.085787	-1.2867	-1.6157	0.57
11538	*PNN*	1.4528	0.56585	-0.31644	-0.41955	0.57
11539	*DHX57*	0.91411	0.42876	0.3593	0.039168	0.57

11540	*PDE6D*	1.0849	0.51428	0.10177	-2.0831	0.57
11541	*SULT2B1*	3.104	-0.19835	-1.2049	-1.7714	0.57
11542	*RIMBP3*	2.081	0.86464	-1.245	-2.1829	0.57
11543	*LPCAT2*	2.194	0.54825	-1.0421	-1.2127	0.57
11544	*C1GALT1C1L*	2.8567	-0.31916	-0.8397	-1.8272	0.57
11545	*MAGEC2*	1.4761	0.35188	-0.13072	-1.2534	0.57
11546	*RGS22*	1.5574	0.36493	-0.22611	-0.54402	0.57
11547	*SRP19*	3.8298	-1.058	-1.0756	-1.3194	0.57
11548	*UMPS*	3.9992	-1.0585	-1.2448	-1.3246	0.57
11549	*CPN2*	2.1139	0.19616	-0.61515	-1.4261	0.56
11550	*KIAA0907*	1.4161	0.2426	0.03565	-0.20142	0.56
11551	*MED17*	2.825	0.2536	-1.3851	-2.6675	0.56
11552	*SLC12A1*	3.1826	-0.46181	-1.0273	-1.6066	0.56
11553	*ZNF367*	4.5065	-1.3038	-1.5094	-2.0165	0.56
11554	*TMPRSS2*	2.6963	0.19908	-1.2027	-2.6062	0.56
11555	*TUBB6*	3.5236	-0.75053	-1.083	-1.4864	0.56
11556	*NRP2*	1.0355	0.35068	0.30301	-2.2599	0.56
11557	*AIMP2*	1.2719	0.77039	-0.35315	-1.3718	0.56
11558	*CLDN14*	1.3311	0.28408	0.07384	-0.96718	0.56
11559	*SRGAP2C*	3.0951	-0.53025	-0.87594	-2.5276	0.56
11560	*CENPF*	2.5584	0.54439	-1.4143	-1.8849	0.56
11561	*SLC19A2*	0.70889	0.52529	0.45306	-0.52786	0.56
11562	*NDUFC2-KCTD14*	3.8311	-0.95321	-1.1912	-1.9734	0.56
11563	*RNPEPL1*	1.9949	0.1473	-0.45604	-1.1169	0.56
11564	*PTP4A1*	3.8156	-0.84816	-1.2829	-1.545	0.56
11565	*FAM150A*	2.3144	-0.3051	-0.32581	-0.97329	0.56
11566	*SAMD10*	2.078	0.054707	-0.4497	-0.62174	0.56
11567	*ATP6V0E1*	2.9688	0.38711	-1.6734	-1.7507	0.56
11568	*XPNPEP3*	4.0741	-1.0838	-1.309	-1.3873	0.56
11569	*NAMPT*	0.88791	0.84999	-0.05914	-1.7397	0.56
11570	*TRAF3IP3*	2.205	0.60213	-1.1287	-1.4683	0.56
11571	*ZBED1*	1.2758	0.35893	0.04338	-0.20808	0.56
11572	*BCL7C*	1.4674	0.14438	0.06595	-0.69059	0.56
11573	*FAM122A*	1.3698	0.36987	-0.06224	-1.048	0.56
11574	*IGFBP4*	2.0445	0.18891	-0.55601	-1.027	0.56
11575	*IMPDH1*	3.0407	-0.44353	-0.91981	-1.0041	0.56
11576	*TBXAS1*	1.4444	0.7921	-0.55992	-1.0313	0.56
11577	*TNN*	1.08	0.80389	-0.20805	-0.30326	0.56
11578	*ZNF354C*	3.2088	0.27747	-1.8107	-2.2413	0.56
11579	*OR6N2*	2.3135	-0.29697	-0.34122	-0.75603	0.56
11580	*SH3BGRL3*	1.4014	0.24174	0.03203	-1.6339	0.56
11581	*ALG14*	2.1033	0.51745	-0.94576	-1.1653	0.56
11582	*AGBL1*	0.66959	0.56655	0.43882	-1.1144	0.56
11583	*HIST2H2BF*	3.0898	-0.56926	-0.8462	-2.2495	0.56
11584	*RFESD*	1.4816	0.37385	-0.18158	-0.20681	0.56
11585	*DTNBP1*	2.8361	-0.34434	-0.82	-1.1743	0.56

11586	*OR2W5*	0.85388	0.48225	0.33495	-1.9326	0.56
11587	*RCC2*	1.9136	0.19441	-0.43704	-1.5304	0.56
11588	*PLA2G10*	2.9742	-0.56241	-0.74086	-1.1479	0.56
11589	*SLC7A8*	2.8419	-0.42177	-0.74932	-1.1263	0.56
11590	*C20orf197*	2.2032	0.57987	-1.1138	-1.2736	0.56
11591	*FUT7*	2.5276	-0.16903	-0.68977	-1.8076	0.56
11592	*RNASEH2A*	3.4913	0.23881	-2.0622	-2.201	0.56
11593	*LYG1*	1.6162	0.0628	-0.0112	-1.1464	0.56
11594	*PLEKHB1*	1.9655	0.53727	-0.83527	-2.1605	0.56
11595	*TLR4*	2.386	0.080148	-0.79904	-3.0408	0.56
11596	*BPGM*	2.8021	0.020275	-1.1553	-2.1496	0.56
11597	*ZFYVE28*	2.5245	-0.10628	-0.75161	-1.392	0.56
11598	*MOB1A*	0.91327	0.60879	0.14319	-0.34927	0.56
11599	*GATAD2A*	1.7382	0.3254	-0.39963	-2.5043	0.55
11600	*THOC5*	4.518	-1.3056	-1.5493	-1.6141	0.55
11601	*CD48*	2.0974	0.16132	-0.59653	-2.0923	0.55
11602	*COX17*	1.8137	0.49425	-0.64687	-1.5508	0.55
11603	*CDON*	2.0675	0.44707	-0.85388	-1.7865	0.55
11604	*OR51A2*	2.295	0.23339	-0.86951	-1.4055	0.55
11605	*JAK3*	3.5605	-0.70558	-1.1977	-1.5171	0.55
11606	*KLF17*	1.4735	0.58315	-0.39959	-0.95405	0.55
11607	*NMB*	1.4015	0.17427	0.08072	-0.01323	0.55
11608	*RABGGTA*	1.6807	0.86392	-0.88839	-1.4994	0.55
11609	*KLHL2*	1.612	0.028786	0.01531	-0.95136	0.55
11610	*SC5D*	1.3848	0.57318	-0.30192	-0.47304	0.55
11611	*SKP2*	2.9037	-0.33583	-0.91317	-2.0181	0.55
11612	*CHRND*	0.81498	0.78631	0.05282	-0.69815	0.55
11613	*LRRC24*	0.80955	0.64874	0.19573	-1.7377	0.55
11614	*KLHL24*	1.8194	0.34848	-0.51405	-1.227	0.55
11615	*COL4A2*	2.0248	-0.06779	-0.30358	-1.6526	0.55
11616	*CA14*	1.5653	0.33739	-0.24951	-2.5007	0.55
11617	*IRS1*	3.2395	-0.66434	-0.92255	-1.6469	0.55
11618	*STK17A*	1.9486	0.5355	-0.83276	-1.6527	0.55
11619	*PDHX*	1.491	0.27023	-0.11003	-0.64538	0.55
11620	*SLC35B4*	2.9104	0.14254	-1.4018	-1.8269	0.55
11621	*KNTC1*	2.6501	0.041736	-1.041	-1.3292	0.55
11622	*KREMEN1*	2.5802	0.20466	-1.1343	-1.202	0.55
11623	*ALCAM*	0.83658	0.45012	0.36321	-0.17016	0.55
11624	*MAP3K8*	1.7485	0.098319	-0.19715	-1.1432	0.55
11625	*SLC5A5*	1.6589	0.66019	-0.66973	-2.0304	0.55
11626	*IGSF9B*	1.7952	0.42378	-0.57018	-2.0704	0.55
11627	*LOC100144595*	3.9554	-1.0791	-1.2277	-1.6306	0.55
11628	*EDDM3B*	1.1359	0.59052	-0.07787	-1.0476	0.55
11629	*OR51F1*	1.5292	0.51554	-0.39719	-2.0116	0.55
11630	*SPATA31A7*	1.6075	0.12602	-0.08604	-1.7658	0.55
11631	*VSTM2A*	4.6632	-1.0695	-1.9474	-3.4111	0.55

11632	*APOL1*	1.3359	0.87933	-0.56929	-0.84204	0.55
11633	*LGALS4*	3.2345	-0.63826	-0.95221	-1.5337	0.55
11634	*OR2A4*	0.97611	0.82709	-0.16	-0.80675	0.55
11635	*IRX1*	1.743	0.016379	-0.117	-1.9357	0.55
11636	*NOD2*	1.1922	0.25432	0.19503	-0.19166	0.55
11637	*STON1-GTF2A1L*	0.60905	0.59683	0.43304	-1.2421	0.55
11638	*DNAJB5*	3.2398	-0.42506	-1.1761	-1.2267	0.55
11639	*GSG1*	3.2992	-0.40961	-1.251	-1.6731	0.55
11640	*LOR*	0.97216	0.41913	0.24723	-2.0995	0.55
11641	*UQCR10*	1.8263	0.00068	-0.18847	-1.247	0.55
11642	*PDIK1L*	2.6029	-0.0886	-0.8759	-1.1308	0.55
11643	*NUDT6*	2.2277	0.29438	-0.88438	-1.5634	0.55
11644	*CRHR2*	2.5254	-0.0298	-0.85943	-1.2048	0.55
11645	*ANGPT4*	1.3321	0.57385	-0.26989	-0.83059	0.55
11646	*KRTAP1-3*	0.87086	0.56217	0.20255	-4.6085	0.55
11647	*SOHLH2*	1.3385	0.32067	-0.02394	-0.18143	0.55
11648	*ZADH2*	1.591	1.2433	-1.1991	-1.8275	0.55
11649	*GIMAP2*	0.98709	0.86957	-0.22197	-1.5271	0.54
11650	*ZFPM1*	2.5345	-0.29633	-0.60362	-2.1238	0.54
11651	*SPANXN4*	1.2713	0.24806	0.11518	-1.0557	0.54
11652	*RBBP5*	2.8102	-0.03456	-1.1417	-2.4018	0.54
11653	*NUSAP1*	1.7543	-0.0175	-0.10381	-1.507	0.54
11654	*ALOX12*	2.9772	-0.32644	-1.0184	-1.5001	0.54
11655	*GRPR*	4.0043	-0.55192	-1.8202	-1.8596	0.54
11656	*KIF2B*	2.3058	-0.25181	-0.4221	-2.0613	0.54
11657	*KLHL10*	1.6278	0.10111	-0.0975	-0.6344	0.54
11658	*CST8*	1.5037	0.95705	-0.83005	-1.0476	0.54
11659	*GPR25*	1.77	0.031869	-0.17129	-0.461	0.54
11660	*FAM90A1*	2.4226	-0.36451	-0.42771	-0.8897	0.54
11661	*MARCH9*	3.1476	-0.09208	-1.4265	-1.591	0.54
11662	*SCAF11*	1.0324	0.39738	0.19837	-0.37417	0.54
11663	*FAM193A*	0.77457	0.68367	0.16914	-0.85617	0.54
11664	*SERPINB4*	1.5052	0.28617	-0.16518	-0.2347	0.54
11665	*SELM*	2.0321	0.5982	-1.0051	-2.4784	0.54
11666	*HLA-DPB1*	3.3356	-0.55196	-1.1585	-1.468	0.54
11667	*SLC6A4*	2.8119	-0.36641	-0.82081	-1.6051	0.54
11668	*PPP1R10*	2.586	-0.41786	-0.54374	-1.5525	0.54
11669	*ZDHHC6*	2.3148	0.049196	-0.7412	-1.4603	0.54
11670	*LRRC3*	2.2429	0.59641	-1.2167	-3.087	0.54
11671	*DNLZ*	3.8528	-0.90606	-1.3249	-1.356	0.54
11672	*C7orf34*	3.9225	-1.0614	-1.2393	-1.6796	0.54
11673	*CNTNAP3B*	3.9911	-1.1762	-1.1932	-3.3213	0.54
11674	*PJA2*	2.28	-0.30777	-0.35193	-0.79483	0.54
11675	*PHOX2B*	1.1984	0.61905	-0.19718	-1.0663	0.54
11676	*SV2B*	3.9885	-0.41476	-1.9559	-2.3557	0.54
11677	*COL4A3BP*	1.7032	0.65903	-0.74489	-0.9191	0.54

11678	*TRPV1*	2.1076	0.88398	-1.3743	-1.987	0.54
11679	*KLHDC10*	4.0356	-1.1282	-1.2915	-1.4683	0.54
11680	*LZTS3*	3.5361	-0.56047	-1.3606	-2.2167	0.54
11681	*DNA2*	2.5636	0.095684	-1.0455	-1.481	0.54
11682	*TMEM174*	1.0232	0.59813	-0.00833	-1.5235	0.54
11683	*MLYCD*	2.1201	0.76623	-1.274	-1.8364	0.54
11684	*SSNA1*	2.0061	0.33119	-0.726	-1.7073	0.54
11685	*ARX*	1.4363	0.2439	-0.06926	-1.6448	0.54
11686	*CSTF2T*	1.1267	0.39424	0.08868	-1.3615	0.54
11687	*SUPT20HL2*	1.5223	1.2322	-1.146	-2.4454	0.54
11688	*YIPF3*	1.1084	0.51575	-0.01568	-1.6921	0.54
11689	*OR5AU1*	3.665	-0.17931	-1.8777	-1.8976	0.54
11690	*HABP4*	1.3405	0.25344	0.01369	-2.4647	0.54
11691	*ACBD5*	1.0826	0.53127	-0.00832	-0.52021	0.54
11692	*OR2F1*	1.9887	0.047726	-0.4312	-0.86889	0.54
11693	*NEK4*	1.397	0.40747	-0.19977	-1.2763	0.53
11694	*DTX3L*	2.154	0.20381	-0.75351	-1.3007	0.53
11695	*LATS2*	1.4855	1.4121	-1.2935	-2.1574	0.53
11696	*L1CAM*	4.9072	-1.632	-1.6724	-1.8636	0.53
11697	*NDUFA8*	2.2242	0.60177	-1.2234	-1.3542	0.53
11698	*HOXC9*	2.597	0.56009	-1.5552	-2.2485	0.53
11699	*EZR*	1.7094	0.53875	-0.64632	-1.794	0.53
11700	*ADAMTS6*	1.1588	0.42899	0.0136	-1.4143	0.53
11701	*BAIAP2*	1.1549	0.51621	-0.07005	-2.0036	0.53
11702	*PRRG2*	0.91932	0.39355	0.28812	-1.7235	0.53
11703	*PROSER3*	1.2522	0.20243	0.14582	-1.4683	0.53
11704	*NF2*	0.84302	0.80598	-0.04919	-0.71553	0.53
11705	*PPY*	2.5507	-0.10934	-0.84238	-1.605	0.53
11706	*TMEM165*	1.5541	0.097548	-0.05357	-0.65606	0.53
11707	*ZNF444*	1.0425	0.41717	0.13807	-0.6251	0.53
11708	*SMG5*	1.8411	1.193	-1.4364	-2.0593	0.53
11709	*PODXL*	1.3722	0.51748	-0.29232	-1.8283	0.53
11710	*TRIM46*	0.84518	0.64155	0.11033	-0.51549	0.53
11711	*ZNF280A*	0.74242	0.46706	0.38728	-0.67688	0.53
11712	*TLK1*	2.1302	0.82334	-1.3572	-1.6523	0.53
11713	*TST*	3.4196	-0.85829	-0.96529	-1.7611	0.53
11714	*MT1B*	1.8093	0.12865	-0.34252	-1.5806	0.53
11715	*PRPS1*	1.863	0.027839	-0.29652	-1.2646	0.53
11716	*LRRN1*	0.6143	0.5758	0.40336	-0.80978	0.53
11717	*BBS10*	3.1659	-0.76237	-0.81031	-1.2776	0.53
11718	*PHGR1*	1.0874	0.44439	0.05866	-2.3449	0.53
11719	*SEPT6*	3.0625	0.83963	-2.3121	-2.3189	0.53
11720	*ZIM2*	0.92313	0.53037	0.13459	-2.2139	0.53
11721	*METTL10*	3.1551	-0.31035	-1.2571	-2.5571	0.53
11722	*GALNT7*	0.90034	0.6528	0.03435	-0.65035	0.53
11723	*NUDCD2*	2.9975	-0.61958	-0.79083	-2.3265	0.53

11724	*C7orf49*	3.4003	-0.48659	-1.3268	-1.7994	0.53
11725	*MSRA*	1.1699	0.59211	-0.17683	-1.5739	0.53
11726	*STUB1*	1.8494	-0.0082	-0.25606	-1.4935	0.53
11727	*CLRN1*	2.0972	0.64116	-1.1543	-1.3353	0.53
11728	*UBE2E3*	2.0447	0.071115	-0.53321	-0.73944	0.53
11729	*PITPNB*	1.5337	0.94819	-0.89974	-1.0564	0.53
11730	*HIST1H4B*	2.2024	-0.16041	-0.46092	-1.4754	0.53
11731	*C17orf47*	3.2547	-0.28303	-1.3912	-1.7572	0.53
11732	*GNA11*	0.9309	0.35764	0.29118	-1.6893	0.53
11733	*ACTR5*	1.5292	0.27195	-0.22166	-1.5954	0.53
11734	*LAMB2*	2.08	-0.19357	-0.30743	-0.32858	0.53
11735	*BEND3*	1.4579	0.64417	-0.52349	-0.81496	0.53
11736	*TLR3*	1.5478	0.63025	-0.59959	-1.9417	0.53
11737	*ANLN*	3.1073	-0.63971	-0.8897	-1.4365	0.53
11738	*ATP1B1*	2.5484	-0.11664	-0.855	-1.2947	0.53
11739	*KRTAP22-1*	4.6247	-1.4597	-1.5884	-1.7266	0.53
11740	*LRSAM1*	1.2488	0.17905	0.14867	-0.45449	0.53
11741	*PPCS*	2.5698	0.081535	-1.0751	-1.1921	0.53
11742	*GPT*	0.87458	0.79521	-0.09362	-0.53666	0.53
11743	*RILP*	1.2106	0.1979	0.16758	-1.8714	0.53
11744	*RELL1*	0.75233	0.41263	0.41083	-1.7078	0.53
11745	*SDCCAG3*	0.98237	0.54647	0.04556	-1.6339	0.52
11746	*HIST1H3J*	2.0458	0.83804	-1.311	-2.3557	0.52
11747	*SLC35B1*	3.4536	-0.6951	-1.186	-1.6709	0.52
11748	*ZC3H12A*	1.9355	0.60262	-0.96572	-1.9727	0.52
11749	*GDPD4*	1.4128	0.42143	-0.26207	-0.51398	0.52
11750	*DPAGT1*	3.4182	-0.77924	-1.067	-1.8352	0.52
11751	*TMEM104*	1.8262	0.86048	-1.1148	-2.305	0.52
11752	*NHLRC4*	1.2872	0.55945	-0.27486	-0.96671	0.52
11753	*BTF3L4*	0.67451	0.62835	0.26852	0.267	0.52
11754	*TLE3*	1.0863	0.53073	-0.04606	-2.0949	0.52
11755	*HSF4*	4.4644	-0.67141	-2.2223	-5.8184	0.52
11756	*PSMB5*	3.6299	-0.55196	-1.5073	-1.7822	0.52
11757	*TMEM63B*	3.4416	-0.50877	-1.3625	-1.7758	0.52
11758	*OCLM*	1.0178	0.48465	0.06767	-1.2572	0.52
11759	*GSN*	2.5538	-0.31411	-0.6706	-1.7129	0.52
11760	*ZNF622*	1.8651	0.36012	-0.65679	-0.74249	0.52
11761	*DOC2A*	2.5829	-0.26584	-0.74864	-2.672	0.52
11762	*ERV3-1*	2.2561	-0.19627	-0.49216	-0.93999	0.52
11763	*STK10*	3.3591	-0.75053	-1.0415	-2.0064	0.52
11764	*KLKB1*	0.7579	0.68028	0.12606	-1.6176	0.52
11765	*TRIM65*	1.8671	0.30414	-0.60744	-1.5604	0.52
11766	*ZFP3*	1.1831	0.78605	-0.40556	-0.70244	0.52
11767	*ORC4*	1.4847	1.4812	-1.4028	-1.6527	0.52
11768	*EIF4EBP2*	2.6481	0.076792	-1.1619	-1.6124	0.52
11769	*BCAS3*	0.75464	0.64586	0.16232	-0.95753	0.52

11770	*ARIH1*	3.1055	0.71021	-2.2533	-2.2593	0.52
11771	*AP1M2*	0.95967	0.37249	0.23008	-0.74069	0.52
11772	*C18orf63*	1.1004	0.50722	-0.04564	-0.07138	0.52
11773	*PSG4*	1.1394	0.23161	0.19074	-1.6525	0.52
11774	*OR5D14*	5.5666	-1.5235	-2.4815	-5.3303	0.52
11775	*ITPR2*	1.1599	0.44961	-0.04799	-0.70202	0.52
11776	*DNAAF3*	2.6912	-0.18791	-0.94255	-1.1974	0.52
11777	*TMED7*	1.9492	0.054869	-0.44353	-0.67691	0.52
11778	*ZMYM1*	0.83524	0.72607	-0.00081	-0.17138	0.52
11779	*VRK1*	1.0894	0.24511	0.2256	-0.20023	0.52
11780	*PGAP2*	1.8275	-0.12457	-0.14293	-0.81001	0.52
11781	*OR2D2*	1.6097	0.011164	-0.06142	-0.63542	0.52
11782	*DEFB136*	2.9924	-0.62782	-0.80577	-1.0999	0.52
11783	*TCEANC*	1.3049	0.59553	-0.34275	-0.5231	0.52
11784	*RND2*	1.6556	0.030874	-0.12891	-0.31698	0.52
11785	*FGF8*	1.2621	0.64541	-0.35023	-1.0626	0.52
11786	*KCTD12*	1.1563	0.67888	-0.27844	-0.75173	0.52
11787	*NDUFC1*	4.0294	-1.1804	-1.2936	-1.631	0.52
11788	*SLC17A9*	2.4315	0.88177	-1.7585	-2.1796	0.52
11789	*RENBP*	3.2176	-0.70132	-0.96161	-1.5569	0.52
11790	*NDUFA10*	2.939	0.19377	-1.5781	-2.1176	0.52
11791	*C16orf96*	1.4276	0.53936	-0.41237	-2.3292	0.52
11792	*ZFP42*	1.8442	0.18879	-0.47843	-0.7423	0.52
11793	*OR2A25*	1.8512	-0.09709	-0.19995	-1.6164	0.52
11794	*NKAPL*	2.0035	0.37687	-0.827	-1.5724	0.52
11795	*NEURL4*	1.1292	0.94222	-0.51811	-1.4234	0.52
11796	*ACOT9*	1.4649	0.86992	-0.78183	-1.0712	0.52
11797	*HYLS1*	2.111	-0.12642	-0.43167	-0.49138	0.52
11798	*YLPM1*	1.0106	0.9931	-0.45119	-1.9591	0.52
11799	*ANKRD35*	0.8554	0.67347	0.02334	-1.6696	0.52
11800	*DUSP14*	2.4784	-0.18633	-0.7412	-2.1429	0.52
11801	*TOP3A*	2.0559	0.90323	-1.4097	-1.4787	0.52
11802	*NPS*	1.4127	0.21632	-0.08118	-1.9623	0.52
11803	*MYH15*	2.9509	-0.09652	-1.3068	-2.4946	0.52
11804	*IER5*	3.4375	0.44681	-2.337	-2.3426	0.52
11805	*HR*	1.6891	0.22358	-0.36569	-1.8908	0.52
11806	*ZDHHC3*	3.4715	-0.6761	-1.2485	-1.6186	0.52
11807	*ABCC10*	3.9908	-1.1756	-1.2684	-1.9624	0.52
11808	*LMOD1*	1.0094	0.87883	-0.34247	-0.54744	0.52
11809	*DNAJA2*	2.1354	0.31287	-0.90281	-1.5635	0.52
11810	*PTCH2*	1.0379	0.55577	-0.04927	-0.71347	0.51
11811	*AMZ1*	1.458	0.082627	0.00367	-2.5935	0.51
11812	*DPPA3*	2.8302	0.12149	-1.4076	-1.526	0.51
11813	*KIAA1211L*	0.98126	0.46635	0.09472	-0.38248	0.51
11814	*SHISA6*	3.4122	-0.77534	-1.0948	-1.7407	0.51
11815	*RBM34*	3.5029	-0.73552	-1.2258	-1.2306	0.51

11816	*DNAJB2*	1.3122	0.32059	-0.0925	-0.57827	0.51
11817	*LCE4A*	1.7204	1.1937	-1.3741	-2.1036	0.51
11818	*FAM228B*	2.3505	-0.16574	-0.64496	-1.7024	0.51
11819	*HPS5*	1.2437	0.30432	-0.00823	-0.10101	0.51
11820	*SLC35E2*	0.80647	0.58201	0.15123	0.019201	0.51
11821	*GTF3C4*	3.2604	-0.60852	-1.1126	-2.1364	0.51
11822	*MEIG1*	2.5393	0.57941	-1.5801	-1.7655	0.51
11823	*PPP2R5E*	0.71478	0.61639	0.20724	-0.66652	0.51
11824	*KRTAP2-4*	2.1809	0.72921	-1.3721	-1.6989	0.51
11825	*ANKRD66*	3.2424	-0.74638	-0.95835	-1.1791	0.51
11826	*LRP2*	1.8309	0.25143	-0.54467	-0.9728	0.51
11827	*SH3PXD2B*	1.3806	0.67459	-0.51826	-1.3617	0.51
11828	*PTGIR*	1.2555	0.15336	0.12753	-0.91701	0.51
11829	*LAMB4*	2.5839	0.17894	-1.227	-1.8946	0.51
11830	*PDE1C*	3.3516	-0.46763	-1.3486	-2.0016	0.51
11831	*HYAL2*	4.5299	-0.76371	-2.2313	-3.4957	0.51
11832	*UBR5*	0.74519	0.42316	0.36628	-1.154	0.51
11833	*CUTA*	3.5869	-0.67313	-1.3795	-1.9302	0.51
11834	*NR1H4*	0.85111	0.84967	-0.1672	-2.0466	0.51
11835	*ILDR2*	1.2811	0.65365	-0.40171	-1.0177	0.51
11836	*KIF1C*	0.80124	0.41519	0.31649	0.012112	0.51
11837	*APOPT1*	0.57432	0.54151	0.41696	-1.8946	0.51
11838	*DNAJA1*	2.0317	-0.05801	-0.44134	-1.0335	0.51
11839	*EIF3L*	2.9267	-0.19995	-1.1945	-2.1214	0.51
11840	*CX3CR1*	0.75683	0.60832	0.167	-1.5649	0.51
11841	*BID*	2.0964	-0.01656	-0.54786	-2.6212	0.51
11842	*UBASH3A*	2.1651	-0.2804	-0.35306	-1.0706	0.51
11843	*DHCR24*	1.5901	0.23258	-0.29123	-0.74291	0.51
11844	*ALG10*	1.2806	1.2301	-0.97956	-1.2488	0.51
11845	*DPY19L3*	1.9993	0.94668	-1.4149	-1.547	0.51
11846	*LRRC16B*	3.8518	-0.9728	-1.3481	-2.0466	0.51
11847	*ZNF679*	2.289	-0.16581	-0.59231	-0.94422	0.51
11848	*RPL35A*	2.4919	-0.3576	-0.60343	-2.3828	0.51
11849	*TBC1D4*	3.5539	-0.56405	-1.4596	-1.8689	0.51
11850	*ANKRD34B*	4.0852	-0.96997	-1.5852	-2.0687	0.51
11851	*KRTAP2-2*	3.0932	-0.38044	-1.183	-1.4064	0.51
11852	*AP3S1*	1.4934	0.033126	0.00292	-2.4094	0.51
11853	*PRSS22*	0.89724	0.80758	-0.17554	-0.18955	0.51
11854	*LCN15*	2.7024	-0.30375	-0.86996	-1.9302	0.51
11855	*HTR7*	2.0144	0.28128	-0.76725	-1.4368	0.51
11856	*SNTB2*	2.7758	-0.17952	-1.068	-1.2426	0.51
11857	*TCEB3C*	0.68036	0.48287	0.36397	0.16344	0.51
11858	*POLE2*	1.8978	-0.10074	-0.26992	-1.7561	0.51
11859	*WFDC1*	1.0236	0.59627	-0.09312	-0.19169	0.51
11860	*NFKBIZ*	1.3603	0.45447	-0.28836	-1.1035	0.51
11861	*RABL2A*	3.1202	-0.77475	-0.81932	-1.1974	0.51

11862	*TSGA10*	1.6734	0.45712	-0.60604	-1.25	0.51
11863	*MGAT3*	3.0852	0.57416	-2.1351	-2.796	0.51
11864	*BRINP3*	4.1754	-0.94777	-1.7034	-2.4197	0.51
11865	*SAMD14*	1.9601	-0.00164	-0.43472	-1.3784	0.51
11866	*MORC3*	1.3788	0.63157	-0.48669	-0.77733	0.51
11867	*LMBR1*	0.70898	0.58147	0.23189	-0.82921	0.51
11868	*ARHGEF2*	1.3101	0.45486	-0.24327	-2.2277	0.51
11869	*C3*	2.7479	0.15456	-1.3818	-4.4937	0.51
11870	*HLA-DRB1*	1.0365	0.72734	-0.24578	-2.0178	0.51
11871	*CORT*	1.2577	0.27415	-0.0146	-1.6281	0.51
11872	*NCBP2*	1.0419	0.29943	0.17578	-1.0399	0.51
11873	*AP1S3*	0.69404	0.50311	0.31908	-0.62185	0.51
11874	*MCM2*	2.3113	-0.30585	-0.48956	-1.821	0.51
11875	*KRTAP4-7*	1.0697	0.50065	-0.05456	-0.81798	0.51
11876	*NMS*	2.069	-0.18019	-0.37466	-1.4147	0.50
11877	*STRN3*	1.9574	0.85552	-1.2988	-2.1873	0.50
11878	*LAMC2*	1.1811	0.89846	-0.56545	-0.76821	0.50
11879	*KLF8*	1.6718	0.31802	-0.47577	-1.367	0.50
11880	*ATP5G2*	2.0977	-0.05066	-0.53346	-0.73673	0.50
11881	*BLID*	1.3605	0.28161	-0.12866	-0.30697	0.50
11882	*FAR2*	2.0695	0.33669	-0.8929	-1.8033	0.50
11883	*PRG2*	0.8577	0.35803	0.29693	0.26121	0.50
11884	*GJA9*	2.9101	-0.66107	-0.73686	-5.7811	0.50
11885	*HSPA1L*	1.8362	1.0581	-1.3823	-1.6691	0.50
11886	*POTEM*	2.5249	-0.28994	-0.72304	-2.9578	0.50
11887	*TUBA3D*	1.6197	0.092873	-0.20112	-1.7188	0.50
11888	*SEC14L3*	1.2876	0.69094	-0.4675	-0.7484	0.50
11889	*SCIN*	1.6115	0.61323	-0.71403	-0.79453	0.50
11890	*NEIL3*	1.7434	0.70459	-0.93764	-0.96846	0.50
11891	*SLC17A8*	1.0945	1.0741	-0.65906	-0.78784	0.50
11892	*PRRT1*	0.60155	0.52501	0.38297	0.047056	0.50
11893	*HADHB*	1.9215	0.61495	-1.027	-1.0576	0.50
11894	*SEC23IP*	2.4565	-0.14309	-0.80594	-2.8405	0.50
11895	*ESRRG*	2.1929	-0.00303	-0.68252	-1.6365	0.50
11896	*TRMT2B*	1.8866	0.32631	-0.7057	-1.8869	0.50
11897	*NDUFA11*	2.7852	-0.61796	-0.66008	-0.96878	0.50
11898	*ANKRD52*	1.3013	0.58021	-0.37461	-0.65971	0.50
11899	*KCND2*	2.8039	-0.58822	-0.71032	-0.75128	0.50
11900	*CSRP1*	1.8487	0.45573	-0.79909	-1.6691	0.50
11901	*BPY2B*	1.4581	0.055343	-0.00832	-1.2239	0.50
11902	*C19orf73*	1.6377	0.31206	-0.44575	-2.0242	0.50
11903	*MIER2*	2.6225	-0.50324	-0.61549	-1.7113	0.50
11904	*COL26A1*	1.1665	0.72762	-0.39048	-1.6636	0.50
11905	*ACCSL*	0.95681	0.33323	0.21343	-0.57573	0.50
11906	*C3orf18*	1.8529	0.38805	-0.73812	-1.6306	0.50
11907	*NECAP1*	3.8524	-1.0224	-1.3275	-1.85	0.50

11908	*STAT2*	2.1016	-0.14506	-0.45515	-2.0368	0.50
11909	*ACBD7*	0.98421	0.62097	-0.10405	-0.80475	0.50
11910	*CTH*	1.1904	1.1002	-0.78956	-1.4197	0.50
11911	*GNB1*	2.7828	-0.60424	-0.67801	-1.8842	0.50
11912	*KIAA0825*	1.7504	0.080802	-0.33085	-2.004	0.50
11913	*RGS9*	1.1709	0.23456	0.09354	0.082543	0.50
11914	*TMEM191C*	3.4825	-0.64496	-1.3393	-2.0478	0.50
11915	*OR4D9*	2.7288	-0.10661	-1.1244	-1.9534	0.50
11916	*SCRN3*	2.2746	-0.3185	-0.45867	-1.0942	0.50
11917	*PCDHA5*	2.3813	0.69864	-1.583	-2.3602	0.50
11918	*DYNLL2*	2.6267	0.19074	-1.3218	-2.0483	0.50
11919	*NFRKB*	2.357	-0.41786	-0.44353	-1.1409	0.50
11920	*ZNF853*	1.9531	0.24191	-0.69958	-1.714	0.50
11921	*TMEM99*	0.79809	0.50078	0.19645	-1.8085	0.50
11922	*CCDC102B*	1.8798	-0.05408	-0.33139	-0.57397	0.50
11923	*PARS2*	2.5687	0.20565	-1.2802	-1.4218	0.50
11924	*MAGEA2*	1.8431	-0.1637	-0.18612	-1.0557	0.50
11925	*RANBP9*	2.3114	-0.26895	-0.5497	-2.4078	0.50
11926	*HSF2*	3.6565	-0.70945	-1.4544	-2.4496	0.50
11927	*NQO2*	3.1239	-0.77404	-0.85794	-2.4853	0.50
11928	*ROPN1L*	1.1298	0.31394	0.04796	-0.41178	0.50
11929	*DIRAS2*	3.8378	-1.0889	-1.2577	-1.4919	0.50
11930	*ACE*	2.0973	-0.16122	-0.4449	-0.89392	0.50
11931	*TMEM120B*	2.6739	-0.34173	-0.84147	-1.4883	0.50
11932	*PVALB*	2.3931	-0.05905	-0.84345	-1.3648	0.50
11933	*MAGEE2*	1.4298	1.1347	-1.0739	-1.4853	0.50
11934	*ANOS1*	1.4962	0.002242	-0.00823	-1.2399	0.50
11935	*NPIPA8*	1.2935	0.84041	-0.64616	-1.2616	0.50
11936	*CGGBP1*	2.4956	-0.28076	-0.72725	-0.93099	0.50
11937	*SYCN*	2.9306	-0.54469	-0.89832	-5.4539	0.50
11938	*MRPL52*	3.0775	-0.0975	-1.4926	-3.3502	0.50
11939	*NOXRED1*	0.99101	0.252	0.24358	-0.32866	0.50
11940	*TMEM257*	0.81921	0.44223	0.22431	-1.4284	0.50
11941	*GAREML*	2.1303	0.55963	-1.2052	-2.6463	0.49
11942	*C1orf168*	0.70424	0.41605	0.36403	-1.1407	0.49
11943	*LANCL3*	5.2444	-1.2782	-2.4825	-2.8823	0.49
11944	*URM1*	1.0713	0.85562	-0.44353	-1.5552	0.49
11945	*DCK*	0.68374	0.49003	0.30954	-0.67382	0.49
11946	*TPD52L2*	3.257	-0.68169	-1.0932	-3.0757	0.49
11947	*HEBP1*	2.8001	-0.24569	-1.0728	-1.1576	0.49
11948	*ZNF155*	3.1078	-0.1375	-1.4894	-1.7422	0.49
11949	*PSME2*	2.0711	0.32892	-0.92065	-1.5814	0.49
11950	*PAM*	0.78676	0.73157	-0.03951	-1.8102	0.49
11951	*G2E3*	0.61696	0.56728	0.29457	0.011164	0.49
11952	*OR6C76*	4.3673	-1.0944	-1.7945	-1.9251	0.49
11953	*LAG3*	1.445	0.36422	-0.33109	-0.66475	0.49

11954	*CTSF*	1.6645	0.004855	-0.19144	-1.1286	0.49
11955	*KCNH7*	1.9837	0.10168	-0.60869	-1.0168	0.49
11956	*OR4K2*	2.5611	0.85182	-1.9366	-2.0002	0.49
11957	*OR1M1*	1.0928	0.34843	0.03435	-1.0359	0.49
11958	*KRTAP9-4*	1.4493	0.11434	-0.09006	-0.90588	0.49
11959	*SGK3*	2.4835	-0.1856	-0.82547	-0.91292	0.49
11960	*ANKMY1*	2.4147	0.25651	-1.1989	-1.2592	0.49
11961	*GUCD1*	0.77129	0.57061	0.12949	-0.35343	0.49
11962	*SEPP1*	2.9731	-0.54302	-0.95895	-1.8102	0.49
11963	*GAR1*	1.8938	-0.01417	-0.4093	-0.58864	0.49
11964	*LIN28B*	1.4639	0.29777	-0.29199	-0.63791	0.49
11965	*NUTM2F*	1.6966	0.32175	-0.54875	-1.067	0.49
11966	*ABI3*	1.6078	0.7295	-0.86841	-1.4352	0.49
11967	*TBC1D12*	2.7684	0.25614	-1.5558	-1.6618	0.49
11968	*CRAT*	2.6709	-0.5223	-0.68236	-1.4684	0.49
11969	*TNFRSF13B*	1.7264	0.71252	-0.9733	-1.2615	0.49
11970	*RTP1*	1.5899	0.70589	-0.83022	-1.6796	0.49
11971	*DNAJC13*	2.5312	-0.33152	-0.73443	-0.93465	0.49
11972	*BNIP3L*	1.201	0.61617	-0.35205	-1.5502	0.49
11973	*C6orf226*	1.205	0.68556	-0.42634	-1.6567	0.49
11974	*CSPG4*	2.7453	0.67464	-1.9559	-2.4172	0.49
11975	*TAF8*	2.3383	-0.03686	-0.83749	-0.85712	0.49
11976	*C2orf88*	1.0257	0.23725	0.20075	0.19377	0.49
11977	*NT5DC3*	0.82089	0.74017	-0.09789	-0.24348	0.49
11978	*RXFP2*	2.248	-0.37461	-0.41025	-1.2034	0.49
11979	*RBCK1*	2.1339	1.2014	-1.8724	-2.796	0.49
11980	*KLHL15*	2.0294	1.5741	-2.1419	-2.508	0.49
11981	*CPQ*	3.6707	-1.0723	-1.137	-1.8016	0.49
11982	*ADAD2*	1.0871	0.26195	0.11164	-1.9727	0.49
11983	*SLC3A2*	1.5314	0.39341	-0.46424	-1.1605	0.49
11984	*ICAM3*	2.55	0.52271	-1.6128	-1.7428	0.49
11985	*MYOD1*	2.9463	-0.34048	-1.1461	-1.9172	0.49
11986	*ZNF846*	1.0688	0.77299	-0.38248	-1.5762	0.49
11987	*TCEAL8*	1.5062	0.80745	-0.85471	-3.1921	0.49
11988	*DUT*	2.3629	-0.42697	-0.4771	-0.93838	0.49
11989	*CNOT6L*	1.2554	0.64076	-0.43782	-2.0771	0.49
11990	*C3orf79*	1.0049	0.62059	-0.16749	-0.25757	0.49
11991	*LSM14B*	2.4329	0.37263	-1.3476	-1.6037	0.49
11992	*NMNAT2*	2.0012	0.023346	-0.56737	-1.5355	0.49
11993	*ACADL*	2.6974	-0.56586	-0.67441	-1.0942	0.49
11994	*WNT2*	2.4031	-0.1097	-0.83631	-1.365	0.49
11995	*SLC7A2*	1.1609	1.04	-0.74392	-2.0871	0.49
11996	*TPI1*	1.9303	-0.22521	-0.24829	-0.93726	0.49
11997	*JAM3*	0.75524	0.38592	0.31368	-0.3513	0.48
11998	*SLITRK4*	0.91467	0.42618	0.11365	-0.11965	0.48
11999	*ACE2*	1.7345	-0.09482	-0.18547	-1.1356	0.48

12000	*NTN1*	1.9171	0.2522	-0.71553	-1.4951	0.48
12001	*TYMP*	2.6563	-0.44353	-0.75913	-1.6848	0.48
12002	*PHRF1*	1.304	0.46614	-0.31724	-1.4931	0.48
12003	*SNX12*	3.8111	-0.76036	-1.5988	-2.6652	0.48
12004	*TRMT13*	2.5096	0.24772	-1.3057	-3.1086	0.48
12005	*L3HYPDH*	1.3933	0.22925	-0.17105	-0.28457	0.48
12006	*UBE2N*	4.9861	-1.1817	-2.3534	-2.9772	0.48
12007	*BRCA2*	2.5997	-0.28058	-0.86853	-1.1125	0.48
12008	*HP*	2.0987	-0.12005	-0.52826	-1.6759	0.48
12009	*PDCD6IP*	1.2244	0.49944	-0.27379	-0.82846	0.48
12010	*OR1L1*	1.3476	0.22988	-0.12795	-0.37537	0.48
12011	*CCDC90B*	1.1528	0.83903	-0.54264	-1.3599	0.48
12012	*MUC6*	0.94066	0.83381	-0.32545	-2.4067	0.48
12013	*SATL1*	2.643	-0.45669	-0.7376	-0.79573	0.48
12014	*TMEM199*	2.5477	-0.33231	-0.76716	-1.8253	0.48
12015	*UBXN1*	2.6416	0.033945	-1.2276	-1.6789	0.48
12016	*TRIP13*	1.3146	0.33245	-0.19924	-0.28484	0.48
12017	*LRCOL1*	1.3594	0.61421	-0.52585	-0.97346	0.48
12018	*POU4F2*	0.89385	0.63328	-0.08023	-0.6794	0.48
12019	*TCP10L*	0.54526	0.48466	0.41678	-0.21079	0.48
12020	*TMEM63A*	2.9562	-0.67695	-0.83278	-1.18	0.48
12021	*FIG4*	2.4861	-0.27911	-0.76108	-1.8611	0.48
12022	*EFNB3*	3.4828	-0.8383	-1.1989	-1.8487	0.48
12023	*LYPLA1*	0.68756	0.49511	0.26203	-1.8398	0.48
12024	*TDRKH*	0.69509	0.54897	0.20038	-0.40359	0.48
12025	*TMEM176A*	2.7171	-0.40646	-0.86698	-1.5425	0.48
12026	*SUV420H2*	1.0724	0.82342	-0.45219	-2.3629	0.48
12027	*STC2*	1.0594	0.23583	0.14783	-1.2815	0.48
12028	*GPR12*	1.0333	0.21956	0.19019	-1.8376	0.48
12029	*RGS7*	2.6096	0.52793	-1.6951	-1.8341	0.48
12030	*CARD8*	1.1804	0.91919	-0.65724	-1.4765	0.48
12031	*MUC20*	1.6336	0.070305	-0.26159	-0.44417	0.48
12032	*PRRG1*	1.6194	1.0805	-1.2576	-1.7428	0.48
12033	*ZNF296*	1.0647	0.32273	0.05463	-0.44043	0.48
12034	*PROKR2*	3.0876	-0.58028	-1.067	-1.3416	0.48
12035	*MEAF6*	1.3334	0.16938	-0.06308	-0.62444	0.48
12036	*ABCA1*	1.9912	0.6504	-1.2021	-2.9638	0.48
12037	*PLSCR3*	1.517	0.39788	-0.47577	-1.3299	0.48
12038	*HCN2*	0.71037	0.50701	0.22171	-1.7507	0.48
12039	*LUZP2*	1.5454	0.36606	-0.47263	-1.7845	0.48
12040	*RGL4*	3.834	-0.97381	-1.4214	-1.4893	0.48
12041	*NDUFS3*	1.6114	0.60061	-0.77346	-1.2111	0.48
12042	*UGT2B15*	1.4536	0.17905	-0.19422	-0.33142	0.48
12043	*ATP5G1*	1.2184	0.90241	-0.68303	-2.217	0.48
12044	*TMEM158*	1.6538	0.5756	-0.79167	-1.4342	0.48
12045	*PCP4L1*	1.0994	0.63592	-0.29759	-1.3353	0.48

12046	*TAGAP*	4.7119	-1.285	-1.9894	-2.1947	0.48
12047	*VCX2*	1.5576	1.1187	-1.2393	-3.114	0.48
12048	*RHOC*	1.2213	0.82239	-0.60687	-1.2705	0.48
12049	*ACOT8*	1.0871	0.55181	-0.20227	-0.90296	0.48
12050	*PIP4K2B*	0.65469	0.43125	0.35049	0.28913	0.48
12051	*FFAR1*	1.5926	0.76464	-0.92129	-2.255	0.48
12052	*IL27*	2.2687	-0.37266	-0.46032	-0.62947	0.48
12053	*SLC2A4RG*	0.56834	0.5573	0.30962	-1.9295	0.48
12054	*TNRC6C*	1.0708	0.39376	-0.03039	-0.06364	0.48
12055	*DOCK3*	1.0046	0.80511	-0.37602	-1.4364	0.48
12056	*HARS*	3.4824	-0.117	-1.9326	-1.9342	0.48
12057	*MLLT1*	4.0914	-0.86445	-1.7945	-2.0407	0.48
12058	*COASY*	2.5863	-0.44354	-0.71196	-1.268	0.48
12059	*SGCZ*	1.7462	0.49143	-0.80853	-1.0254	0.48
12060	*MTRNR2L10*	1.1073	0.18309	0.13831	-1.1718	0.48
12061	*MAL2*	3.4772	-0.12915	-1.9195	-2.0384	0.48
12062	*LRRC41*	3.0934	-0.31126	-1.3544	-1.3759	0.48
12063	*CDV3*	0.83748	0.43539	0.15398	-1.6951	0.48
12064	*C1orf141*	0.84303	0.67326	-0.08948	-0.27841	0.48
12065	*HIST1H2BA*	3.0202	-0.53058	-1.0635	-1.398	0.48
12066	*TCN1*	2.9096	-0.25048	-1.2331	-1.507	0.48
12067	*MDN1*	3.4545	-0.38219	-1.6463	-2.0682	0.48
12068	*TBC1D14*	1.3163	0.41716	-0.3077	-0.45279	0.48
12069	*ELAVL1*	2.1798	0.23694	-0.99137	-1.2303	0.48
12070	*UTP18*	3.2162	-0.80129	-0.98955	-2.2743	0.48
12071	*HNF1A*	2.563	-0.52655	-0.61136	-1.6285	0.48
12072	*AP3M1*	2.7931	0.28077	-1.649	-1.7989	0.47
12073	*KRT17*	3.2045	-0.51868	-1.2619	-1.2915	0.47
12074	*CTDNEP1*	2.2589	-0.35272	-0.4829	-1.1823	0.47
12075	*MS4A10*	1.2674	0.68961	-0.53437	-1.1278	0.47
12076	*MRPL45*	2.7371	-0.02083	-1.2937	-1.4544	0.47
12077	*FCN3*	1.5929	0.35194	-0.52426	-1.4683	0.47
12078	*VSX1*	0.79008	0.4525	0.17756	-0.18229	0.47
12079	*GCGR*	1.8582	0.78817	-1.227	-1.8157	0.47
12080	*HLA-DQA2*	3.0648	-0.71772	-0.93115	-1.5149	0.47
12081	*C11orf98*	1.448	1.0632	-1.0953	-2.2282	0.47
12082	*PRB3*	2.8249	-0.25832	-1.151	-2.3804	0.47
12083	*LETM1*	2.4366	-0.21664	-0.80641	-1.8366	0.47
12084	*IRGQ*	1.0313	0.70405	-0.32219	-1.1576	0.47
12085	*LMNB1*	2.1954	1.0209	-1.8033	-1.9559	0.47
12086	*ZNF433*	1.3815	0.22203	-0.19136	-2.08	0.47
12087	*ZSCAN23*	1.0776	0.17043	0.16383	-0.95893	0.47
12088	*RPL31*	2.9126	-0.31727	-1.1836	-1.4572	0.47
12089	*TM4SF5*	2.2819	-0.35172	-0.51912	-1.548	0.47
12090	*CECR5*	2.8059	-0.13105	-1.2647	-1.6066	0.47
12091	*FRRS1*	1.5959	0.08651	-0.2723	-1.1718	0.47

12092	*MSS51*	2.7206	-0.47607	-0.83545	-1.5815	0.47
12093	*METTL12*	0.83337	0.38206	0.19351	-0.43694	0.47
12094	*YY1AP1*	1.3806	0.077177	-0.04986	-2.8051	0.47
12095	*KRTAP10-3*	2.53	-0.46923	-0.65291	-1.0921	0.47
12096	*RBP7*	0.70137	0.37869	0.32713	-1.0178	0.47
12097	*LIMS3L*	0.7766	0.44248	0.18809	-0.42359	0.47
12098	*SFXN4*	0.88034	0.58987	-0.0633	-1.6058	0.47
12099	*CCNJ*	2.1956	-0.15919	-0.63009	-1.882	0.47
12100	*NTSR2*	3.2184	-0.41323	-1.3991	-1.5424	0.47
12101	*KIF17*	3.4979	-0.56251	-1.5294	-2.217	0.47
12102	*INTU*	1.0948	0.26835	0.04252	-0.05408	0.47
12103	*MPP7*	2.3129	-0.10307	-0.80436	-1.3294	0.47
12104	*C9orf16*	2.4771	-0.49564	-0.57637	-1.3319	0.47
12105	*SWI5*	1.9744	-0.26055	-0.30936	-0.76606	0.47
12106	*MAK*	1.3541	0.51053	-0.46268	-1.6129	0.47
12107	*MRPL27*	1.3756	0.78334	-0.758	-2.2254	0.47
12108	*SPIDR*	0.92815	0.49737	-0.02484	-1.4894	0.47
12109	*TNIP2*	0.71918	0.61857	0.06291	-1.344	0.47
12110	*CHRDL2*	0.72666	0.37293	0.30093	-0.66513	0.47
12111	*KIAA0368*	1.0529	0.40862	-0.06143	-1.2692	0.47
12112	*METAP1D*	1.0045	0.39219	0.00337	-0.69245	0.47
12113	*CBX6*	3.0439	-0.16574	-1.479	-2.3491	0.47
12114	*FRG1*	2.9838	0.40731	-1.9922	-3.9836	0.47
12115	*ANKRD63*	0.54523	0.45584	0.39779	-0.26206	0.47
12116	*TCAF1*	1.3986	0.016531	-0.01665	-2.8216	0.47
12117	*KMT2D*	1.0858	0.37868	-0.066	-1.5871	0.47
12118	*ADGRE1*	3.0091	-0.34496	-1.2666	-1.2864	0.47
12119	*IDNK*	4.1665	-0.59008	-2.1798	-2.9438	0.47
12120	*RDM1*	1.2362	1.0199	-0.86047	-1.9623	0.47
12121	*MPPE1*	1.48	0.73494	-0.81932	-0.99526	0.47
12122	*ATP2B2*	3.1215	0.24143	-1.9711	-3.0779	0.46
12123	*TFDP2*	1.8391	-0.15649	-0.29186	-0.66146	0.46
12124	*TP63*	3.1439	-0.59503	-1.159	-1.9797	0.46
12125	*IGSF3*	1.9956	1.0334	-1.6394	-2.3689	0.46
12126	*RPL38*	0.75087	0.33153	0.30635	-1.8477	0.46
12127	*CD68*	2.9548	-0.23832	-1.3282	-1.7407	0.46
12128	*CD5*	1.8319	0.017998	-0.46314	-1.7501	0.46
12129	*KRTAP4-12*	3.7156	-0.96583	-1.3637	-1.8366	0.46
12130	*OR51A4*	0.68351	0.45434	0.2481	-1.7575	0.46
12131	*KHDC1*	0.91478	0.79312	-0.32254	-1.4841	0.46
12132	*ARPIN*	0.75407	0.5505	0.08041	0.015519	0.46
12133	*IL1RN*	3.2349	-0.79711	-1.0529	-1.9558	0.46
12134	*GPBP1*	1.1088	1.027	-0.75099	-1.7049	0.46
12135	*TBCCD1*	2.6297	-0.00394	-1.2411	-2.2886	0.46
12136	*NLRP9*	3.9882	-1.1918	-1.4123	-2.0837	0.46
12137	*PILRA*	0.92056	0.51997	-0.05701	-0.36476	0.46

12138	*SLC25A38*	3.6694	-0.74013	-1.547	-2.4858	0.46
12139	*COQ6*	1.3548	0.67522	-0.64801	-0.67722	0.46
12140	*OSR1*	3.7155	-1.0313	-1.3023	-2.1984	0.46
12141	*KCNJ15*	2.0228	-0.06833	-0.57301	-2.0488	0.46
12142	*ANKMY2*	1.1351	0.32753	-0.08177	-0.82833	0.46
12143	*GTSF1L*	1.3545	0.7844	-0.75824	-1.316	0.46
12144	*CENPT*	4.0169	-1.2635	-1.3729	-1.8441	0.46
12145	*RHOA*	1.4695	1.1341	-1.2234	-1.8908	0.46
12146	*PATL2*	3.3106	-0.83184	-1.0993	-1.3966	0.46
12147	*ZBTB7A*	3.3124	-0.10427	-1.829	-2.112	0.46
12148	*DEFB105B*	2.4695	-0.16038	-0.93005	-0.97681	0.46
12149	*NINL*	1.469	0.012762	-0.10392	-2.674	0.46
12150	*PCDHGB7*	1.8541	1.4692	-1.9455	-2.1161	0.46
12151	*ZNF223*	1.1639	0.93159	-0.71772	-1.3393	0.46
12152	*THEMIS*	1.5845	0.70479	-0.91192	-1.0297	0.46
12153	*NPC1*	1.6701	-0.03046	-0.26252	-1.2135	0.46
12154	*DUSP22*	0.62919	0.42022	0.32673	-0.17521	0.46
12155	*MRPL50*	1.1406	1.0095	-0.77409	-1.5201	0.46
12156	*HOXB1*	1.2249	0.19804	-0.04713	-0.94636	0.46
12157	*PPIA*	0.73591	0.6378	0.00068	-0.84786	0.46
12158	*ADAMTS12*	0.97281	0.64991	-0.24887	-2.0008	0.46
12159	*PPM1G*	0.95586	0.63872	-0.22097	-1.5167	0.46
12160	*KCNH4*	1.7943	-0.08724	-0.33346	-1.1135	0.46
12161	*WDR36*	1.1959	0.96881	-0.79138	-2.4363	0.46
12162	*HINT2*	4.3183	-1.4098	-1.5375	-1.7647	0.46
12163	*MRAS*	1.0299	0.9556	-0.61483	-2.7254	0.46
12164	*TWISTNB*	3.8717	-1.1244	-1.377	-1.8879	0.46
12165	*TXNDC12*	1.8871	0.58923	-1.1061	-2.2397	0.46
12166	*RAP1GAP2*	0.89686	0.34933	0.12305	-0.28806	0.46
12167	*TTC7B*	2.3504	-0.03316	-0.94827	-1.7377	0.46
12168	*RAB11FIP2*	0.91607	0.725	-0.27291	-1.448	0.46
12169	*PRR34*	3.1433	-0.64078	-1.1349	-1.28	0.46
12170	*SNX21*	1.929	0.34832	-0.91138	-0.94027	0.46
12171	*GJB3*	2.4282	0.35573	-1.4182	-3.4131	0.46
12172	*ING3*	0.90144	0.89213	-0.42817	-0.44353	0.46
12173	*ARHGEF19*	0.94574	0.85733	-0.43776	-0.84001	0.46
12174	*MAP1LC3A*	1.4865	0.85764	-0.98026	-1.8961	0.45
12175	*CCDC150*	2.5235	-0.41239	-0.74743	-1.8087	0.45
12176	*CAV2*	0.54334	0.41716	0.40281	-1.8154	0.45
12177	*TMEM261*	0.71037	0.53898	0.11383	-2.2392	0.45
12178	*CCDC140*	2.652	0.090452	-1.3795	-2.4094	0.45
12179	*FLRT1*	3.5105	-0.73577	-1.4122	-2.3452	0.45
12180	*CORO7-PAM16*	0.60784	0.45211	0.30223	-2.4741	0.45
12181	*POLR2J3*	2.9007	-0.12191	-1.4168	-1.549	0.45
12182	*LRRC74A*	0.948	0.30826	0.1054	-1.2505	0.45
12183	*CELA2B*	0.85669	0.67291	-0.16836	-0.20848	0.45

12184	*PYGO1*	2.0049	-0.26409	-0.38059	-1.2302	0.45
12185	*STAT6*	0.81944	0.36726	0.17331	0.13894	0.45
12186	*ACAT1*	2.7667	-0.49789	-0.9092	-1.5448	0.45
12187	*SYVN1*	3.7634	-0.69155	-1.7125	-1.9802	0.45
12188	*DOK2*	1.9382	0.68459	-1.2646	-1.2917	0.45
12189	*CD79B*	1.6278	-0.00829	-0.26161	-0.63332	0.45
12190	*TEX22*	0.80612	0.33652	0.21478	-1.5011	0.45
12191	*CYP4F3*	0.60282	0.56529	0.18914	-0.54097	0.45
12192	*ADAMTSL5*	3.4576	-0.90606	-1.195	-1.4073	0.45
12193	*WDR82*	1.9977	-0.26892	-0.3723	-1.4852	0.45
12194	*PCGF6*	3.9888	-1.2132	-1.4197	-1.9334	0.45
12195	*RAMP1*	0.60604	0.44659	0.30301	-2.2998	0.45
12196	*MNAT1*	0.66539	0.53287	0.15715	-1.5383	0.45
12197	*EXOC8*	2.5563	-0.33723	-0.86479	-2.0613	0.45
12198	*VMAC*	4.0273	-1.0942	-1.5795	-2.1429	0.45
12199	*LACTB*	3.3598	-0.78229	-1.2259	-1.2485	0.45
12200	*CAAP1*	1.4284	0.761	-0.83784	-2.2866	0.45
12201	*C12orf76*	2.6836	-0.55956	-0.77333	-0.92868	0.45
12202	*CHID1*	3.5099	-0.3866	-1.7735	-2.7191	0.45
12203	*XPO6*	3.005	-0.05908	-1.5962	-2.3349	0.45
12204	*CYP20A1*	1.68	0.38492	-0.71522	-0.91913	0.45
12205	*TFDP1*	4.3355	-0.92144	-2.0653	-2.716	0.45
12206	*SFTA3*	2.235	-0.12259	-0.76366	-1.1704	0.45
12207	*TNNT3*	1.6402	-0.11497	-0.17679	-0.48572	0.45
12208	*ZNF835*	0.83528	0.37727	0.13558	-2.1239	0.45
12209	*CSNK1E*	2.3996	-0.50246	-0.54934	-1.7307	0.45
12210	*NOP2*	2.6667	-0.5866	-0.73362	-1.2019	0.45
12211	*RRAGA*	0.8286	0.82664	-0.30911	-1.5992	0.45
12212	*NTF4*	1.2736	0.46396	-0.39149	-0.61496	0.45
12213	*FAM193B*	2.4773	0.15516	-1.2876	-2.1319	0.45
12214	*CDKL1*	1.2129	0.77163	-0.64011	-0.77891	0.45
12215	*PTPRE*	2.1295	-0.24516	-0.54207	-1.8318	0.45
12216	*DCN*	1.9399	-0.22235	-0.37656	-2.0223	0.45
12217	*PCDHGA7*	1.0599	0.40826	-0.12803	-1.3331	0.45
12218	*ARHGAP9*	3.2767	-0.25225	-1.6847	-2.6635	0.45
12219	*SMC3*	4.2361	-1.3071	-1.5894	-2.0613	0.45
12220	*CARKD*	0.71911	0.39352	0.22608	-0.36847	0.45
12221	*SLC24A1*	2.9622	-0.4978	-1.1258	-2.5865	0.45
12222	*VTI1A*	0.97533	0.47624	-0.11355	-1.0502	0.45
12223	*SCO2*	1.3536	0.042209	-0.05841	-0.74594	0.45
12224	*FBXW4*	3.4724	-0.8929	-1.2427	-1.2457	0.45
12225	*ADRB1*	3.7835	-1.0741	-1.3727	-2.3602	0.45
12226	*MVB12B*	2.3544	-0.09789	-0.91981	-1.3334	0.45
12227	*NDUFAF4*	1.6324	0.34957	-0.64632	-2.2951	0.45
12228	*POLR3E*	3.3852	-0.99298	-1.0573	-1.186	0.44
12229	*CCL3*	1.594	1.3201	-1.5806	-2.614	0.44

12230	*PCBP4*	4.0792	-0.56251	-2.1834	-2.329	0.44
12231	*TMEM186*	4.102	-1.18	-1.589	-2.0466	0.44
12232	*MAGI2*	0.70898	0.33655	0.28742	-0.87036	0.44
12233	*ARL4D*	1.8507	-0.10146	-0.41678	-0.46155	0.44
12234	*VPS33A*	2.2285	-0.12788	-0.76965	-2.2207	0.44
12235	*WDR93*	0.86552	0.54218	-0.077	-2.5725	0.44
12236	*NFXL1*	2.1551	0.26994	-1.0946	-1.9919	0.44
12237	*ATP6V1A*	0.82103	0.32228	0.18664	0.17094	0.44
12238	*TAF10*	0.7399	0.49478	0.09453	-1.9727	0.44
12239	*CDK2AP1*	3.2659	-0.10861	-1.8291	-2.6416	0.44
12240	*TMEM194B*	1.2412	0.11742	-0.03046	-1.4433	0.44
12241	*C2orf57*	3.5946	-1.0252	-1.2417	-2.3244	0.44
12242	*UBXN10*	1.7681	-0.18053	-0.25999	-0.44353	0.44
12243	*C2orf73*	1.3302	0.45389	-0.45663	-1.583	0.44
12244	*CEP131*	2.2629	-0.19609	-0.73938	-1.0148	0.44
12245	*MMP19*	1.0599	0.93868	-0.67144	-2.5795	0.44
12246	*NLRP11*	0.91376	0.2124	0.20008	-1.6891	0.44
12247	*UBD*	1.0437	0.81522	-0.5328	-1.4544	0.44
12248	*KERA*	0.80449	0.71578	-0.19422	-0.98653	0.44
12249	*MANEA*	1.695	-0.02131	-0.34767	-1.9811	0.44
12250	*SPRR2A*	1.114	0.67755	-0.46565	-1.2929	0.44
12251	*MS4A1*	1.2934	0.37684	-0.34496	-1.2666	0.44
12252	*SSPO*	2.3082	-0.3586	-0.62456	-1.6476	0.44
12253	*FBXO30*	1.5884	0.47311	-0.73708	-2.9032	0.44
12254	*SI*	0.82519	0.47351	0.0251	-1.8523	0.44
12255	*INSL4*	0.56519	0.44924	0.30727	-0.3931	0.44
12256	*ENAH*	0.86519	0.275	0.18074	-0.38618	0.44
12257	*GSC*	0.90619	0.29909	0.11502	-2.6416	0.44
12258	*C10orf71*	2.2278	-0.33565	-0.57241	-0.63035	0.44
12259	*LGSN*	1.4375	0.258	-0.37797	-0.46052	0.44
12260	*ZBED6*	3.5621	-1.0369	-1.209	-1.5592	0.44
12261	*MRPL35*	3.3942	-0.78956	-1.2908	-1.984	0.44
12262	*MEP1B*	2.9532	0.083863	-1.7235	-1.9559	0.44
12263	*PAX3*	3.8667	-0.92916	-1.6246	-1.7149	0.44
12264	*KCNH3*	1.6898	0.31558	-0.69362	-1.0232	0.44
12265	*AVEN*	1.3749	0.82811	-0.89155	-1.1025	0.44
12266	*NCLN*	3.0871	-0.66363	-1.1122	-2.1197	0.44
12267	*DDX52*	3.6708	-0.77908	-1.5805	-1.6192	0.44
12268	*KLHL36*	0.98771	0.26017	0.06281	-1.9302	0.44
12269	*CBY1*	2.496	0.042086	-1.2276	-1.6826	0.44
12270	*TBCC*	1.4519	0.34019	-0.482	-2.213	0.44
12271	*CXCL8*	3.1787	-0.81496	-1.0542	-2.5006	0.44
12272	*SERPINB10*	1.9259	0.4676	-1.084	-1.0955	0.44
12273	*SND1*	3.1901	-0.73398	-1.1468	-1.3651	0.44
12274	*ETF1*	1.2853	0.55925	-0.53619	-2.5153	0.44
12275	*MARS2*	2.917	-0.53766	-1.0711	-1.4125	0.44

12276	*CAND1*	1.6326	1.1943	-1.5189	-1.686	0.44
12277	*TMOD2*	1.2891	0.016479	0.00226	-0.5795	0.44
12278	*LTB4R*	1.5375	-0.05094	-0.18068	-1.4156	0.44
12279	*LMAN1*	0.58548	0.53298	0.18677	-1.9591	0.44
12280	*WSB2*	3.0778	-0.80641	-0.96647	-1.0639	0.43
12281	*SLC36A4*	2.3216	0.013259	-1.0302	-2.5007	0.43
12282	*TRIM49D1*	0.98916	0.55696	-0.24157	-1.1287	0.43
12283	*ANXA7*	3.8285	-1.1692	-1.3549	-1.6559	0.43
12284	*TRAPPC3L*	2.1574	-0.23777	-0.61528	-1.7585	0.43
12285	*GABARAPL2*	2.2982	-0.33705	-0.65733	-2.2653	0.43
12286	*COL10A1*	1.3781	0.22737	-0.30181	-1.2224	0.43
12287	*GRAP2*	1.0117	0.51997	-0.22852	-0.9968	0.43
12288	*KIAA1107*	1.5078	0.77485	-0.97982	-1.2679	0.43
12289	*MTRNR2L4*	2.3813	0.12888	-1.2075	-1.4361	0.43
12290	*CTR9*	1.9989	0.041704	-0.7385	-1.6835	0.43
12291	*GNE*	2.0708	0.23504	-1.0041	-1.0741	0.43
12292	*SHISA2*	3.463	-0.43471	-1.7266	-1.7577	0.43
12293	*SIRT5*	3.1108	-0.44353	-1.3668	-1.4864	0.43
12294	*ZNF141*	3.2152	0.051769	-1.9673	-2.5934	0.43
12295	*TREML2*	3.5391	-0.86298	-1.377	-1.864	0.43
12296	*KIFC2*	1.3236	0.19646	-0.22133	-2.0383	0.43
12297	*UBQLN1*	2.5295	0.13942	-1.3711	-2.1648	0.43
12298	*ZCCHC7*	2.1331	-0.28807	-0.54791	-0.58892	0.43
12299	*PM20D1*	2.2163	-0.36401	-0.55554	-1.6756	0.43
12300	*ZCRB1*	2.7744	-0.29414	-1.1848	-2.3222	0.43
12301	*SEMA3E*	2.6069	-0.51467	-0.79711	-1.6682	0.43
12302	*GOLT1B*	0.69366	0.36994	0.23091	-1.3959	0.43
12303	*SLMO1*	0.56109	0.48778	0.24494	-0.14701	0.43
12304	*PPARA*	2.1475	-0.38667	-0.46761	-0.55844	0.43
12305	*CCNC*	0.74926	0.42975	0.11383	-0.54373	0.43
12306	*PLA2G4E*	1.0717	0.41875	-0.19869	-0.19996	0.43
12307	*KRTAP17-1*	4.2222	-1.1898	-1.7407	-2.1354	0.43
12308	*ENTPD6*	1.6019	-0.06967	-0.24066	-0.42884	0.43
12309	*CSNK1G1*	1.2605	1.21	-1.179	-1.7037	0.43
12310	*KLF16*	3.4714	-0.96661	-1.2135	-2.9298	0.43
12311	*POLR2M*	1.7236	0.05102	-0.48336	-1.8961	0.43
12312	*PYCARD*	3.0603	-0.11824	-1.6525	-1.7936	0.43
12313	*POGLUT1*	3.7317	-1.03	-1.4132	-1.4864	0.43
12314	*ACER1*	4.1174	-0.79243	-2.0387	-2.1732	0.43
12315	*HRASLS*	0.58555	0.39867	0.30155	-0.58888	0.43
12316	*MYLK3*	3.3958	-0.88507	-1.2251	-1.2318	0.43
12317	*PCNXL3*	1.5524	-0.04556	-0.22209	-1.3948	0.43
12318	*ZNF517*	2.1909	0.42299	-1.3292	-1.6741	0.43
12319	*LOC650293*	4.1391	-0.96375	-1.8909	-2.069	0.43
12320	*ZNF358*	2.3645	-0.16681	-0.91456	-1.4108	0.43
12321	*GNG13*	1.8285	-0.1421	-0.40338	-0.61112	0.43

12322	*C1D*	1.1117	0.85238	-0.68122	-1.5167	0.43
12323	*USP17L17*	3.713	-0.24946	-2.1807	-2.829	0.43
12324	*PRRC2C*	0.58665	0.40273	0.29267	0.068606	0.43
12325	*PBX3*	0.77641	0.4888	0.01653	0.000252	0.43
12326	*FAM92B*	2.0952	-0.16294	-0.65092	-1.6904	0.43
12327	*HPS1*	1.1576	0.42876	-0.30609	-0.49266	0.43
12328	*CNBP*	1.3146	0.20971	-0.24428	-1.6322	0.43
12329	*ATP12A*	1.3455	0.70081	-0.76702	-1.2034	0.43
12330	*GJC2*	4.2696	-1.4388	-1.5522	-1.8462	0.43
12331	*PDZD11*	2.718	-0.09164	-1.3478	-1.9404	0.43
12332	*CYP4V2*	0.6088	0.39713	0.26891	-0.46024	0.42
12333	*EVPL*	3.5183	-0.87694	-1.3666	-1.6193	0.42
12334	*DNAJA4*	3.0408	-0.41087	-1.3554	-2.0953	0.42
12335	*CD200R1*	1.4517	-0.0813	-0.09703	-0.82452	0.42
12336	*HELLS*	1.8296	-0.0236	-0.53285	-0.79798	0.42
12337	*LRP10*	1.5809	0.88098	-1.1897	-1.4758	0.42
12338	*CBX1*	2.7317	-0.66174	-0.79983	-0.86772	0.42
12339	*PDK2*	2.3356	0.50465	-1.5707	-1.635	0.42
12340	*FAM169B*	1.1482	0.45281	-0.33152	-0.98574	0.42
12341	*RFC4*	0.84092	0.67435	-0.24715	-1.7065	0.42
12342	*ODF2L*	1.3725	1.3575	-1.4625	-1.5848	0.42
12343	*APOA1*	0.62089	0.61252	0.034	-0.28228	0.42
12344	*HIST1H1A*	3.2505	0.038212	-2.0226	-2.4094	0.42
12345	*GCK*	0.92534	0.45421	-0.11492	-0.35352	0.42
12346	*SCFD2*	2.6985	-0.62792	-0.80613	-1.1547	0.42
12347	*STARD13*	1.386	0.54697	-0.66992	-0.72825	0.42
12348	*TMC1*	3.7512	-0.73247	-1.7567	-2.0197	0.42
12349	*NOVA2*	1.2202	0.40338	-0.3617	-1.8371	0.42
12350	*ANKRD44*	0.50283	0.42627	0.33245	-2.6354	0.42
12351	*AHCYL2*	1.6628	0.28061	-0.682	-1.3089	0.42
12352	*RBSN*	1.1566	0.9493	-0.84457	-1.4469	0.42
12353	*FBXL17*	2.3585	0.048765	-1.146	-1.8207	0.42
12354	*PRSS38*	1.9621	0.1469	-0.8478	-2.3517	0.42
12355	*KRT1*	1.3332	0.30066	-0.37282	-1.1344	0.42
12356	*SYNPR*	1.2537	0.14818	-0.14104	-1.5524	0.42
12357	*ZNF124*	1.1996	0.15759	-0.09647	-0.12613	0.42
12358	*ASH1L*	2.5985	-0.52329	-0.81536	-0.94171	0.42
12359	*XAB2*	1.8609	0.10845	-0.71009	-1.1242	0.42
12360	*PGA4*	1.2977	0.37869	-0.41716	-1.5431	0.42
12361	*PHEX*	2.7428	-0.43376	-1.0503	-1.3132	0.42
12362	*CHGA*	1.6102	0.61348	-0.966	-2.0946	0.42
12363	*SKOR1*	1.4199	1.2327	-1.3951	-1.6895	0.42
12364	*ADAP2*	2.5678	-0.15626	-1.1543	-1.4248	0.42
12365	*RHBDF1*	2.9861	-0.26018	-1.4688	-2.0267	0.42
12366	*ZC2HC1B*	0.48494	0.42625	0.34559	-0.19704	0.42
12367	*C14orf166*	1.3827	0.41626	-0.54274	-0.57379	0.42

12368	*CAPRIN2*	2.9711	-0.17566	-1.5395	-2.1463	0.42
12369	*MED16*	2.1349	-0.2607	-0.61827	-1.4031	0.42
12370	*BCCIP*	0.97532	0.26121	0.0193	-3.6612	0.42
12371	*COPE*	1.6135	0.31895	-0.67702	-0.90151	0.42
12372	*SEMA6C*	2.4037	-0.01458	-1.1344	-1.6041	0.42
12373	*CEP295NL*	0.99061	0.22855	0.03549	-1.2074	0.42
12374	*NUDT1*	1.2595	0.13369	-0.13871	-0.49724	0.42
12375	*REST*	1.4296	0.50751	-0.683	-0.97776	0.42
12376	*AKTIP*	2.3749	-0.43219	-0.68869	-1.6974	0.42
12377	*TRPC5OS*	1.1176	0.54526	-0.40938	-0.46724	0.42
12378	*FOXO6*	4.1202	-0.78503	-2.0817	-2.3811	0.42
12379	*CEBPG*	3.4739	-0.67702	-1.5436	-2.458	0.42
12380	*HSPA2*	1.4993	-0.07816	-0.16862	-1.5738	0.42
12381	*MOGAT2*	1.8438	0.86502	-1.4566	-1.7874	0.42
12382	*CCDC137*	2.3439	-0.1834	-0.90872	-1.1955	0.42
12383	*MED15*	0.97387	0.88499	-0.60719	-1.5424	0.42
12384	*C1QL1*	2.7299	-0.17525	-1.3033	-2.0629	0.42
12385	*IKBKE*	2.6466	-0.37982	-1.0156	-1.4087	0.42
12386	*RPP40*	4.216	-1.2075	-1.7578	-2.1484	0.42
12387	*RGS11*	0.58978	0.4775	0.18281	-0.67791	0.42
12388	*RSPH10B*	1.5786	-0.10495	-0.22525	-1.3574	0.42
12389	*CCDC79*	0.97316	0.41564	-0.14151	-1.138	0.42
12390	*GPR150*	1.1524	0.89613	-0.80146	-0.92389	0.42
12391	*KCTD10*	0.81921	0.72204	-0.29427	-0.627	0.42
12392	*PRR20D*	0.92408	0.73219	-0.41178	-0.93621	0.41
12393	*SH3D19*	1.9194	-0.2492	-0.42653	-1.4576	0.41
12394	*TNKS*	2.0574	0.036582	-0.8504	-2.397	0.41
12395	*ZNF443*	0.56964	0.45188	0.22171	-1.3795	0.41
12396	*TSPAN16*	2.2847	0.63242	-1.6739	-2.2248	0.41
12397	*ARMCX1*	2.5255	0.004082	-1.2867	-1.8392	0.41
12398	*DEFA5*	1.2966	0.015107	-0.06937	-1.6382	0.41
12399	*RXFP3*	1.6509	-0.08868	-0.32001	-0.6908	0.41
12400	*PCDH18*	2.1615	-0.29647	-0.62307	-0.86261	0.41
12401	*FAM162A*	0.86161	0.85134	-0.47107	-1.4801	0.41
12402	*P3H3*	3.1448	-0.78951	-1.1135	-1.1357	0.41
12403	*PLXNC1*	2.8136	-0.17712	-1.3949	-1.4644	0.41
12404	*PRMT3*	1.0593	0.54744	-0.36518	-1.1584	0.41
12405	*BAMBI*	0.98874	0.82446	-0.57224	-1.4368	0.41
12406	*CACNA2D3*	3.1434	-0.37367	-1.5294	-1.8463	0.41
12407	*MALRD1*	3.4233	-0.89794	-1.2852	-1.3296	0.41
12408	*RSL1D1*	1.955	0.3437	-1.0586	-1.2109	0.41
12409	*BPIFA2*	3.3447	-0.89204	-1.213	-1.594	0.41
12410	*PLGLB1*	2.846	-0.48968	-1.1169	-2.6485	0.41
12411	*JCHAIN*	0.94889	0.18972	0.10061	-0.19494	0.41
12412	*KRT83*	0.8907	0.79361	-0.44533	-1.6289	0.41
12413	*TMX3*	1.7643	0.094216	-0.62114	-1.5632	0.41

12414	*MCL1*	3.2326	-0.78221	-1.2132	-1.3884	0.41
12415	*RAD21*	0.48216	0.47261	0.28124	-0.15139	0.41
12416	*CHRNG*	1.3421	0.45273	-0.55958	-0.78229	0.41
12417	*AVPR1A*	1.5693	0.19561	-0.53025	-1.267	0.41
12418	*CSN3*	2.8255	-0.57531	-1.0164	-1.6866	0.41
12419	*UHRF1BP1L*	0.79112	0.26574	0.17659	-1.72	0.41
12420	*ZNF445*	3.3905	-0.75944	-1.3978	-1.4926	0.41
12421	*LRR1*	3.9188	-1.3399	-1.3457	-2.1947	0.41
12422	*IFI35*	1.3216	0.13633	-0.22544	-0.93112	0.41
12423	*ZNF22*	3.2268	-0.58985	-1.4048	-1.7804	0.41
12424	*ACADVL*	1.921	0.76167	-1.4507	-1.9338	0.41
12425	*NR1H2*	2.6785	-0.50595	-0.94113	-1.6524	0.41
12426	*SLN*	3.1098	-0.74972	-1.1287	-1.3689	0.41
12427	*ZFAND4*	2.6523	-0.69333	-0.72881	-0.78672	0.41
12428	*WDR33*	1.2901	-0.01774	-0.04233	-0.36579	0.41
12429	*INPP5D*	1.647	1.2989	-1.7177	-1.9242	0.41
12430	*CCL21*	0.90155	0.25382	0.07237	0.061247	0.41
12431	*PLA2G4C*	3.6745	-0.49941	-1.9479	-1.995	0.41
12432	*ZCCHC16*	1.704	-0.23796	-0.23892	-1.4574	0.41
12433	*SLC43A1*	0.79649	0.3146	0.11572	-1.8102	0.41
12434	*B4GALNT2*	1.4314	0.49801	-0.70309	-1.0696	0.41
12435	*TMC6*	2.7436	-0.24442	-1.2733	-2.2543	0.41
12436	*OR2AE1*	1.0279	0.15053	0.04735	-0.14944	0.41
12437	*CXorf56*	1.7045	0.56217	-1.0423	-1.5801	0.41
12438	*MRLN*	2.0426	0.49731	-1.3165	-1.4946	0.41
12439	*MYH6*	2.3757	0.28648	-1.4392	-2.3065	0.41
12440	*RAB6C*	1.128	0.5599	-0.46583	-1.077	0.41
12441	*ZMAT4*	3.5507	-1.067	-1.2619	-1.3637	0.41
12442	*EIF5B*	2.0513	-0.15561	-0.67435	-1.2447	0.41
12443	*FBXO2*	1.3358	0.70635	-0.8213	-1.5485	0.41
12444	*CALHM2*	2.7499	-0.37592	-1.1533	-1.4076	0.41
12445	*B4GALNT3*	1.8387	0.22286	-0.84151	-2.3689	0.41
12446	*PDC*	0.57427	0.34469	0.30098	-0.72157	0.41
12447	*SERPINI1*	1.7397	-0.1305	-0.38937	-3.3744	0.41
12448	*AMIGO2*	3.1173	-0.5141	-1.3835	-1.5482	0.41
12449	*COL6A1*	0.98217	0.41953	-0.182	-2.1018	0.41
12450	*C3orf17*	0.99951	0.39299	-0.17534	-0.54244	0.41
12451	*MRGPRX2*	0.50483	0.47914	0.23281	0.055477	0.41
12452	*RNF125*	1.6787	-0.16531	-0.2969	-0.47528	0.41
12453	*ATXN2*	2.1535	-0.17241	-0.76606	-2.005	0.41
12454	*AKAP11*	2.7151	-0.29739	-1.2027	-1.545	0.41
12455	*AGRN*	0.74396	0.63535	-0.16549	-0.77847	0.40
12456	*SH2B1*	1.6701	-0.21833	-0.23931	-1.348	0.40
12457	*C1orf177*	1.1593	0.77524	-0.72294	-1.7414	0.40
12458	*CDCP2*	1.0023	0.74269	-0.53362	-1.7397	0.40
12459	*FXR1*	1.0399	0.98093	-0.80954	-1.364	0.40

12460	*XBP1*	3.1054	-0.83557	-1.0589	-2.9525	0.40
12461	*CELSR2*	0.87656	0.50541	-0.17188	-0.30355	0.40
12462	*OPN1LW*	0.93712	0.57856	-0.30692	-0.57241	0.40
12463	*THAP6*	1.8768	-0.0359	-0.63405	-1.4316	0.40
12464	*DEPDC4*	1.4676	0.046064	-0.30706	-0.99037	0.40
12465	*AGTRAP*	2.3553	-0.05456	-1.095	-1.4919	0.40
12466	*SLC35E4*	2.9184	-0.78579	-0.92861	-1.4597	0.40
12467	*RNF135*	2.0479	-0.12884	-0.7167	-1.5115	0.40
12468	*ZNF648*	0.70117	0.66077	-0.1604	-1.8503	0.40
12469	*MYF5*	1.8224	0.44676	-1.0679	-5.3431	0.40
12470	*MED9*	1.1523	0.10954	-0.06126	-0.36196	0.40
12471	*PQLC2*	3.9858	-1.0589	-1.7268	-1.8348	0.40
12472	*ZFP92*	2.5611	-0.13185	-1.2292	-1.7386	0.40
12473	*RBMY1F*	0.55325	0.49275	0.15316	0.044397	0.40
12474	*CASKIN1*	0.68774	0.39742	0.11388	-0.24079	0.40
12475	*NRXN3*	1.4293	-0.07204	-0.15842	-0.52607	0.40
12476	*WFDC6*	2.3351	-0.31342	-0.82311	-0.9512	0.40
12477	*SH3GLB2*	0.64697	0.43672	0.11411	-2.6101	0.40
12478	*BRSK1*	1.9993	-0.35805	-0.44353	-1.0922	0.40
12479	*ANXA9*	1.6507	1.3205	-1.7745	-2.2955	0.40
12480	*GLI3*	3.0726	-0.8147	-1.0614	-2.4328	0.40
12481	*SLC22A12*	4.0513	-1.2208	-1.6343	-2.1407	0.40
12482	*SCEL*	2.2425	-0.44353	-0.60323	-1.0146	0.40
12483	*ZHX1-C8orf76*	2.139	-0.31772	-0.62556	-1.6682	0.40
12484	*C1orf210*	1.8282	-0.13178	-0.50075	-2.2732	0.40
12485	*ENO1*	0.67486	0.66085	-0.14009	-1.7443	0.40
12486	*WDR5*	1.3544	0.37368	-0.53304	-0.95527	0.40
12487	*STK3*	0.68041	0.34241	0.17215	-1.5467	0.40
12488	*SLC25A13*	1.3603	1.1396	-1.305	-1.9284	0.40
12489	*C20orf78*	3.351	-0.94882	-1.2079	-1.4686	0.40
12490	*IFNB1*	0.52402	0.49202	0.17824	-0.3579	0.40
12491	*CRYBA2*	2.1909	-0.28169	-0.71723	-1.4234	0.40
12492	*MEN1*	3.1672	-0.38103	-1.5959	-2.0409	0.40
12493	*TSEN54*	0.94568	0.53106	-0.28668	-0.66326	0.40
12494	*CNTLN*	1.2598	0.1167	-0.18718	-1.7908	0.40
12495	*EXD2*	2.1889	0.52568	-1.5253	-1.8885	0.40
12496	*NPTXR*	1.0194	0.67288	-0.5059	-2.3937	0.40
12497	*MYO7B*	2.5093	-0.15513	-1.1698	-1.3987	0.39
12498	*C7orf60*	2.9673	-0.70973	-1.0739	-1.3496	0.39
12499	*GMEB2*	2.2103	-0.05784	-0.96962	-1.1184	0.39
12500	*HSFY2*	0.66019	0.57321	-0.0509	-2.0818	0.39
12501	*MRO*	1.1617	0.39939	-0.3787	-1.6885	0.39
12502	*LOC100129940*	1.1918	0.71993	-0.72969	-2.0807	0.39
12503	*CCL24*	0.61272	0.42443	0.14451	-1.4511	0.39
12504	*C12orf60*	3.4148	-0.89009	-1.3433	-2.0197	0.39
12505	*OR2M5*	4.4155	-0.92255	-2.312	-3.2688	0.39

12506	*DGKK*	2.7556	-0.69287	-0.88181	-0.99608	0.39
12507	*PRPF38A*	3.5543	-1.0304	-1.344	-2.7705	0.39
12508	*NSUN5*	0.70606	0.49925	-0.02563	-0.31508	0.39
12509	*HGFAC*	1.3746	0.61493	-0.81068	-1.697	0.39
12510	*TSKS*	0.78385	0.75185	-0.35685	-1.0441	0.39
12511	*RBM15B*	1.5555	-0.11349	-0.26369	-0.5489	0.39
12512	*MLLT6*	3.9119	-1.3597	-1.3743	-1.4507	0.39
12513	*NLK*	0.89921	0.18918	0.08947	-0.31359	0.39
12514	*FRMPD2*	1.516	-0.05369	-0.28631	-2.1984	0.39
12515	*MAP3K10*	2.2665	-0.47781	-0.61271	-0.94172	0.39
12516	*HPX*	2.293	-0.46405	-0.65402	-1.2393	0.39
12517	*ELFN1*	2.1914	0.31212	-1.329	-1.62	0.39
12518	*SLC29A1*	2.8275	-0.2069	-1.4467	-2.1046	0.39
12519	*ARCN1*	1.843	-0.30007	-0.36913	-1.1491	0.39
12520	*RNF113B*	1.8934	0.96509	-1.6866	-1.7235	0.39
12521	*ERICH5*	1.0502	0.073137	0.04779	-1.8002	0.39
12522	*SH3GL3*	0.90307	0.21188	0.05538	-1.6866	0.39
12523	*ADNP*	2.0869	0.3488	-1.2656	-1.3607	0.39
12524	*KLK11*	0.69597	0.4323	0.04174	-2.2117	0.39
12525	*TMEM120A*	2.1326	0.083746	-1.0466	-1.5663	0.39
12526	*HCST*	2.4	0.071123	-1.3023	-1.707	0.39
12527	*TP53TG3B*	1.0101	0.37603	-0.21797	-1.05	0.39
12528	*MON2*	2.0013	0.52005	-1.3542	-1.6843	0.39
12529	*PARVA*	3.6073	-0.46749	-1.9727	-2.184	0.39
12530	*GOLGA8N*	3.676	-1.0229	-1.4864	-2.0476	0.39
12531	*STX16*	0.68144	0.34241	0.14234	-0.09344	0.39
12532	*TWF1*	3.1933	0.51644	-2.5442	-2.9987	0.39
12533	*OLR1*	0.83335	0.52902	-0.19704	-1.3523	0.39
12534	*POLR3K*	1.297	0.21436	-0.34796	-0.40605	0.39
12535	*OR9G4*	0.62834	0.46664	0.06821	-0.91343	0.39
12536	*PCDH7*	3.3359	-0.3512	-1.8219	-1.9295	0.39
12537	*GMFB*	0.62056	0.29607	0.24612	-1.3635	0.39
12538	*WDR7*	1.8496	-0.11126	-0.57586	-0.89494	0.39
12539	*SUV420H1*	0.93804	0.38688	-0.16263	-0.46056	0.39
12540	*MRPL1*	0.82379	0.35012	-0.01168	-1.4567	0.39
12541	*UBA6*	1.1496	0.9114	-0.89892	-2.0105	0.39
12542	*C1QB*	3.2156	-0.994	-1.0596	-1.9833	0.39
12543	*LMNTD1*	2.1087	0.99561	-1.9431	-1.9484	0.39
12544	*SMC1A*	1.5674	0.028439	-0.43468	-0.81761	0.39
12545	*VPS29*	3.0671	-0.82833	-1.078	-1.4859	0.39
12546	*DTD2*	1.75	0.14463	-0.73389	-1.4564	0.39
12547	*NSF*	1.5296	0.57405	-0.94345	-1.4894	0.39
12548	*TPD52*	1.1386	0.078531	-0.0576	-1.204	0.39
12549	*TMEM53*	2.1636	-0.17323	-0.83101	-1.4819	0.39
12550	*DAAM2*	1.0148	0.12189	0.02109	-0.48204	0.39
12551	*PPARG*	1.7111	0.19492	-0.74868	-1.4154	0.39

12552	*AXIN1*	0.91802	0.60926	-0.37001	-0.47447	0.39
12553	*SOX3*	2.622	0.10226	-1.5671	-1.8248	0.39
12554	*CCDC57*	0.5598	0.49216	0.1049	-3.0219	0.39
12555	*RAP1GAP*	1.3651	0.653	-0.86157	-1.2493	0.39
12556	*WDR17*	1.0924	0.042547	0.02099	-1.088	0.39
12557	*IRX2*	1.167	0.48815	-0.49977	-1.1533	0.39
12558	*CCDC18*	3.312	-0.80301	-1.3544	-2.1902	0.38
12559	*ZNF561*	1.7273	0.42709	-1.0005	-2.1648	0.38
12560	*CD3D*	2.9424	-0.6728	-1.1169	-2.1919	0.38
12561	*GLA*	1.3152	0.65146	-0.81505	-1.7428	0.38
12562	*TESK2*	3.5263	-1.0204	-1.3543	-1.9748	0.38
12563	*SLC30A6*	1.5994	0.25501	-0.70379	-1.208	0.38
12564	*DRD5*	2.0082	-0.38472	-0.47395	-1.5073	0.38
12565	*SORBS1*	1.7904	0.84999	-1.4913	-2.0539	0.38
12566	*SERPINB5*	1.9949	0.16475	-1.0113	-2.3452	0.38
12567	*CADM4*	1.2301	0.55468	-0.63659	-2.6289	0.38
12568	*F2*	1.6776	-0.198	-0.33205	-1.1897	0.38
12569	*TP53TG3C*	2.8374	-0.08047	-1.6105	-2.4615	0.38
12570	*SPICE1*	2.3623	-0.08915	-1.1271	-2.5198	0.38
12571	*EIF3M*	4.1389	-1.1739	-1.8191	-2.581	0.38
12572	*DDX39B*	0.50009	0.4261	0.21956	-1.9341	0.38
12573	*SPATA9*	0.9219	0.80358	-0.57974	-1.2214	0.38
12574	*SPATC1L*	1.5994	0.39005	-0.84542	-1.0324	0.38
12575	*GHRH*	1.4558	-0.10126	-0.21054	-1.7301	0.38
12576	*FOXO3*	1.1796	0.2641	-0.30018	-0.96946	0.38
12577	*PCM1*	3.2692	-0.49961	-1.6263	-1.9251	0.38
12578	*SYT11*	1.495	0.60069	-0.95244	-1.3407	0.38
12579	*ANKRD49*	2.0232	0.22286	-1.1035	-1.6464	0.38
12580	*CGB8*	0.48296	0.37081	0.28866	-1.3159	0.38
12581	*PYCRL*	2.3279	-0.03667	-1.1494	-1.39	0.38
12582	*INHBC*	2.3811	-0.45777	-0.78229	-1.0421	0.38
12583	*EPS15*	1.8058	0.62639	-1.2912	-1.301	0.38
12584	*ANKRD53*	4.3206	-1.4024	-1.7797	-2.5663	0.38
12585	*SIPA1L1*	1.4119	-0.13518	-0.13885	-0.66358	0.38
12586	*FAP*	2.3288	-0.04968	-1.1415	-1.7338	0.38
12587	*BAP1*	1.9133	-0.24569	-0.53009	-1.4835	0.38
12588	*OR2G3*	0.98954	0.5661	-0.4187	-2.0953	0.38
12589	*KIR2DL4*	1.0557	0.52472	-0.44353	-0.48535	0.38
12590	*BBS9*	1.3297	0.15055	-0.34431	-1.6842	0.38
12591	*SNX15*	1.4758	0.021592	-0.36189	-1.0576	0.38
12592	*PLA2G16*	1.7991	0.6944	-1.358	-2.7442	0.38
12593	*COG6*	0.52748	0.49817	0.1098	-0.42403	0.38
12594	*TMEM37*	0.84595	0.1561	0.13308	-0.88418	0.38
12595	*SCG5*	1.9338	0.06603	-0.86479	-2.7223	0.38
12596	*AMOTL1*	2.9443	-0.23447	-1.5752	-2.0404	0.38
12597	*NFKBIE*	1.5783	-0.12074	-0.32355	-0.80897	0.38

12598	*PCDHB15*	1.2521	0.010651	-0.12891	-2.0423	0.38
12599	*BTG4*	2.6761	0.13956	-1.6819	-2.1709	0.38
12600	*MYZAP*	2.3724	-0.45449	-0.78444	-1.0824	0.38
12601	*B3GNT4*	1.0237	0.59615	-0.48648	-0.84345	0.38
12602	*NUDT18*	1.7341	-0.11133	-0.48968	-0.50355	0.38
12603	*ZNF778*	1.5988	0.21523	-0.681	-1.6098	0.38
12604	*OR2L3*	1.638	0.15436	-0.65971	-0.85329	0.38
12605	*ESYT3*	1.4442	0.267	-0.57974	-2.4084	0.38
12606	*FKBP10*	1.9618	0.36505	-1.1955	-1.4913	0.38
12607	*EFCAB14*	1.0103	0.15515	-0.03422	-0.40772	0.38
12608	*RPL7L1*	0.90753	0.16852	0.0547	-1.209	0.38
12609	*PAFAH1B2*	1.6945	-0.12039	-0.44353	-0.84419	0.38
12610	*EBF4*	0.82853	0.31938	-0.01805	-1.3852	0.38
12611	*CUL3*	2.6626	-0.00698	-1.5274	-1.5673	0.38
12612	*RMDN1*	3.2094	-1.0388	-1.043	-2.1334	0.38
12613	*UBE2K*	3.8734	-1.1125	-1.6339	-2.116	0.38
12614	*KRTAP22-2*	2.5537	-0.36233	-1.0653	-1.067	0.38
12615	*MARS*	1.1121	0.6425	-0.62881	-2.0337	0.38
12616	*PZP*	3.3298	-0.90554	-1.2985	-2.0164	0.38
12617	*HK3*	2.2358	-0.50569	-0.60582	-0.89866	0.37
12618	*GPR39*	1.2035	0.38825	-0.46749	-1.4017	0.37
12619	*POPDC2*	2.2026	-0.12852	-0.95224	-2.1849	0.37
12620	*GRIN2A*	1.9915	0.37011	-1.2437	-1.9705	0.37
12621	*CCDC120*	1.0853	0.12666	-0.09418	-0.71768	0.37
12622	*SLC4A9*	3.0899	-0.80004	-1.1723	-1.7061	0.37
12623	*C11orf42*	0.98977	0.9548	-0.82789	-1.3282	0.37
12624	*OSBPL3*	0.89475	0.80088	-0.57899	-0.71381	0.37
12625	*PMVK*	2.4701	-0.38472	-0.96878	-1.257	0.37
12626	*KDSR*	3.5736	-0.83631	-1.6213	-1.7466	0.37
12627	*SLC1A5*	0.71094	0.27348	0.13096	-1.2543	0.37
12628	*NPAS1*	1.9184	-0.0014	-0.80168	-1.6676	0.37
12629	*KLF9*	0.92427	0.67866	-0.48801	-0.64514	0.37
12630	*SLC24A4*	0.53037	0.53031	0.05419	-0.71381	0.37
12631	*GSG2*	2.7351	-0.34036	-1.2815	-1.8938	0.37
12632	*JMJD7-PLA2G4B*	2.2336	-0.09964	-1.0208	-1.2444	0.37
12633	*CCDC7*	3.1345	-0.87601	-1.1456	-1.1739	0.37
12634	*AARS*	2.3376	-0.28336	-0.94184	-1.0884	0.37
12635	*HTT*	2.137	-0.39731	-0.62747	-1.2281	0.37
12636	*TAPBPL*	0.67758	0.57053	-0.13746	-1.1637	0.37
12637	*PANX3*	0.61736	0.35453	0.1385	-1.677	0.37
12638	*TCN2*	1.2702	0.63945	-0.79932	-1.0933	0.37
12639	*PHF2*	1.8875	-0.12971	-0.64793	-2.0017	0.37
12640	*OR51E2*	0.67857	0.46392	-0.03356	-1.653	0.37
12641	*KIAA2022*	1.8687	0.86367	-1.6237	-1.9541	0.37
12642	*CROT*	2.4427	-0.15766	-1.1764	-2.1419	0.37
12643	*RPS13*	3.4723	-0.51779	-1.8461	-2.2248	0.37

12644	*AHRR*	1.3743	0.10874	-0.37522	-1.2815	0.37
12645	*PTGFR*	1.4609	0.07789	-0.43101	-2.4409	0.37
12646	*PTPRK*	1.3203	0.054536	-0.26712	-0.48659	0.37
12647	*DCAF8*	1.3658	0.55536	-0.81348	-2.2866	0.37
12648	*PRMT7*	0.99465	0.20646	-0.09459	-1.0611	0.37
12649	*ADGRE2*	1.6722	-0.16614	-0.39971	-0.51679	0.37
12650	*ACTL10*	1.1003	0.61328	-0.60744	-2.0326	0.37
12651	*GPD1L*	1.2055	0.26936	-0.36896	-1.8227	0.37
12652	*BCL7A*	0.76171	0.25687	0.08711	-1.6785	0.37
12653	*CNOT11*	3.1838	-1.0346	-1.0454	-1.5219	0.37
12654	*FAM114A2*	3.7196	-1.143	-1.4732	-1.7956	0.37
12655	*PLEKHG4*	1.0034	0.28222	-0.18301	-1.13	0.37
12656	*NFIB*	2.2605	-0.27861	-0.8803	-1.9493	0.37
12657	*APOC4*	1.3564	0.053608	-0.30863	-0.63511	0.37
12658	*PLB1*	1.8987	-0.06618	-0.73143	-2.0287	0.37
12659	*TEAD4*	3.3841	-0.65714	-1.626	-2.3573	0.37
12660	*CER1*	0.93165	0.28701	-0.11781	-0.72728	0.37
12661	*FGD2*	1.6213	0.36498	-0.88566	-1.035	0.37
12662	*ELL3*	2.3582	0.21879	-1.4764	-1.908	0.37
12663	*MEOX1*	2.9793	-0.92781	-0.95105	-1.9997	0.37
12664	*ZHX2*	1.6583	0.76302	-1.3209	-1.3218	0.37
12665	*OR4K14*	2.2171	-0.45486	-0.66194	-2.098	0.37
12666	*HOXC4*	1.1088	0.15662	-0.16642	-0.46089	0.37
12667	*SLC35C1*	1.6101	0.61766	-1.1288	-2.1282	0.37
12668	*CD274*	0.49817	0.46215	0.13862	-0.72612	0.37
12669	*GNB1L*	1.55	0.89957	-1.3517	-1.7753	0.37
12670	*MAP3K19*	2.633	-0.26949	-1.2662	-1.739	0.37
12671	*PFDN5*	1.2237	0.080097	-0.20663	-2.5844	0.37
12672	*CLEC12B*	0.87871	0.20328	0.01474	-1.9751	0.37
12673	*SYPL1*	1.0952	0.73364	-0.73247	-0.8988	0.37
12674	*HSD17B8*	1.4777	0.1144	-0.49601	-1.1169	0.37
12675	*EYS*	1.3594	0.62816	-0.89155	-1.1021	0.37
12676	*SCT*	4.4839	-1.631	-1.7585	-2.5943	0.36
12677	*ZDHHC11*	2.7244	-0.52075	-1.1095	-2.4652	0.36
12678	*LIN52*	3.12	-0.69754	-1.3285	-1.7577	0.36
12679	*OR4P4*	1.709	-0.02738	-0.58822	-1.4767	0.36
12680	*PRPF40B*	0.94025	0.76885	-0.61591	-1.2393	0.36
12681	*GALNT5*	1.4317	0.28284	-0.62202	-1.124	0.36
12682	*SAXO1*	1.7074	-0.04763	-0.56753	-2.2021	0.36
12683	*CPB2*	1.413	0.10413	-0.42536	-1.4154	0.36
12684	*PREP*	0.83862	0.20845	0.04356	-1.452	0.36
12685	*KCNE2*	2.0604	-0.32419	-0.6469	-1.2867	0.36
12686	*ARHGAP27*	0.84876	0.62094	-0.38044	-2.1616	0.36
12687	*AAED1*	4.2956	-1.4467	-1.7602	-2.892	0.36
12688	*POTEA*	1.6453	0.056297	-0.61436	-1.8057	0.36
12689	*CCL3L3*	0.81654	0.49695	-0.22645	-0.66654	0.36

12690	*PITRM1*	1.6565	0.61894	-1.1886	-1.6497	0.36
12691	*CCDC149*	2.071	-0.31981	-0.66445	-0.73707	0.36
12692	*H2AFB1*	2.8446	0.12335	-1.8816	-2.7042	0.36
12693	*MPDU1*	1.5424	-0.19357	-0.26252	-1.8463	0.36
12694	*MCHR1*	1.9123	-0.08716	-0.73896	-1.9054	0.36
12695	*C11orf49*	1.1314	0.64303	-0.68889	-1.3718	0.36
12696	*STK11*	0.75145	0.62682	-0.29292	-0.76955	0.36
12697	*PHF5A*	1.1476	0.1018	-0.16549	-1.4223	0.36
12698	*ENTPD8*	1.5496	0.56252	-1.0283	-1.6636	0.36
12699	*HPGD*	1.3354	0.78302	-1.0347	-1.3932	0.36
12700	*APLF*	0.98283	0.96132	-0.86047	-1.041	0.36
12701	*KLF7*	2.7725	-0.4119	-1.277	-1.3607	0.36
12702	*PLXNA4*	0.46167	0.43311	0.18868	-1.2864	0.36
12703	*B3GALNT1*	1.2618	0.83314	-1.0129	-1.6176	0.36
12704	*RBM33*	1.2327	0.60136	-0.75205	-1.95	0.36
12705	*NT5E*	1.24	0.73555	-0.89382	-1.2666	0.36
12706	*NOL4L*	3.0235	-0.71987	-1.222	-1.5336	0.36
12707	*SSR1*	3.1489	-0.67889	-1.3884	-1.4557	0.36
12708	*KIAA1715*	0.91503	0.64788	-0.48131	-1.8689	0.36
12709	*SELV*	2.6106	-0.31109	-1.2187	-1.3389	0.36
12710	*DNAJB12*	1.1925	0.40361	-0.51539	-1.084	0.36
12711	*MYO18B*	3.4634	-0.89649	-1.4864	-2.075	0.36
12712	*OSBP2*	2.1242	-0.38157	-0.66232	-0.75639	0.36
12713	*RAB4B*	0.68447	0.25125	0.14449	-1.0796	0.36
12714	*TMEM154*	2.5931	-0.70212	-0.8108	-2.0572	0.36
12715	*SLC25A3*	2.6961	-0.05653	-1.5603	-1.7213	0.36
12716	*GDF15*	1.7388	-0.03348	-0.6262	-1.5302	0.36
12717	*CPA1*	1.1516	0.63805	-0.71063	-1.9495	0.36
12718	*CCDC125*	1.1277	0.4332	-0.48232	-1.5467	0.36
12719	*CLDN12*	0.75516	0.38432	-0.06111	-0.48893	0.36
12720	*LRCH4*	2.205	-0.56036	-0.56634	-1.6663	0.36
12721	*F8A2*	3.6594	-0.49423	-2.0871	-2.2473	0.36
12722	*PRY*	1.0289	0.49258	-0.44353	-0.7431	0.36
12723	*ENO2*	3.6901	-0.89756	-1.7149	-2.4381	0.36
12724	*LRRC19*	2.0431	0.75207	-1.7179	-2.2485	0.36
12725	*C1orf105*	1.2145	1.0868	-1.2241	-2.0773	0.36
12726	*PTPN12*	0.53526	0.37901	0.16249	-0.38553	0.36
12727	*RPL28*	3.6355	-1.1316	-1.4273	-2.0146	0.36
12728	*TIGD2*	1.0013	0.7069	-0.63336	-0.94583	0.36
12729	*OOEP*	1.2678	0.47724	-0.67064	-1.3058	0.36
12730	*SUMO2*	0.5757	0.40177	0.09659	-2.0283	0.36
12731	*NAT8*	3.4586	-0.26713	-2.1179	-2.252	0.36
12732	*CST5*	2.7874	-0.39035	-1.3235	-1.7667	0.36
12733	*NNAT*	3.6634	-1.0239	-1.5671	-2.4858	0.36
12734	*RNF166*	1.7542	0.85457	-1.5366	-1.9938	0.36
12735	*MATN4*	3.8604	-0.97346	-1.8157	-2.0113	0.36

12736	*BRPF3*	1.2321	-0.07537	-0.08624	-0.90576	0.36
12737	*C1orf162*	1.4046	0.35446	-0.68944	-1.9785	0.36
12738	*TGM5*	3.8588	-1.3209	-1.4688	-1.6193	0.36
12739	*RAB40A*	0.52217	0.41603	0.13046	-1.3593	0.36
12740	*TKFC*	1.4595	0.24364	-0.63554	-0.82921	0.36
12741	*HNRNPA1L2*	0.6695	0.39105	0.00518	-0.50283	0.36
12742	*UBTF*	1.4784	0.01731	-0.42999	-1.9269	0.36
12743	*ADM5*	1.1349	0.27932	-0.3495	-0.44353	0.35
12744	*PRAP1*	0.93502	0.70866	-0.57967	-1.2628	0.35
12745	*NAT6*	0.76482	0.23542	0.06366	-0.35059	0.35
12746	*SERF2*	3.5671	-0.13486	-2.3696	-2.4784	0.35
12747	*SPTAN1*	1.5532	0.26655	-0.75778	-1.9455	0.35
12748	*BLMH*	2.5896	-0.53487	-0.99277	-1.3235	0.35
12749	*AMIGO1*	0.7398	0.20384	0.11746	-1.6893	0.35
12750	*TTF1*	2.5042	-0.34292	-1.1002	-2.1438	0.35
12751	*CPN1*	0.7806	0.65953	-0.37942	-1.8557	0.35
12752	*CCDC186*	1.668	0.34895	-0.95677	-1.1289	0.35
12753	*KLF4*	1.292	0.091413	-0.32332	-1.4076	0.35
12754	*VASH1*	1.2198	-0.02339	-0.13843	-0.60343	0.35
12755	*PKNOX2*	2.4154	-0.03972	-1.3184	-1.7782	0.35
12756	*SPATA2L*	2.9895	-0.87955	-1.0529	-2.4853	0.35
12757	*SOGA1*	1.7806	-0.16018	-0.56337	-1.0564	0.35
12758	*ALKBH5*	0.99059	0.58984	-0.52343	-1.6759	0.35
12759	*PSG11*	2.0862	0.009609	-1.039	-1.9484	0.35
12760	*ABCF2*	2.5549	-0.31727	-1.181	-1.6116	0.35
12761	*THBS2*	0.69815	0.63278	-0.27443	-0.53285	0.35
12762	*NENF*	1.697	0.65971	-1.3019	-2.462	0.35
12763	*KATNBL1*	0.81875	0.42847	-0.19332	-1.5865	0.35
12764	*EEF1A2*	0.70302	0.37588	-0.02662	-1.0092	0.35
12765	*DDX20*	0.56821	0.36513	0.11841	-0.52372	0.35
12766	*GDI1*	1.4276	-0.11212	-0.2638	-0.59028	0.35
12767	*PPP1R3E*	1.1519	0.72222	-0.82255	-1.5682	0.35
12768	*CEPT1*	0.52289	0.30868	0.21995	-0.95895	0.35
12769	*RDH16*	1.0448	0.14247	-0.13758	-0.33091	0.35
12770	*ZNF780B*	1.2667	0.38033	-0.59743	-1.2967	0.35
12771	*PAK4*	1.1812	0.40829	-0.5404	-0.90588	0.35
12772	*LHFPL4*	4.6945	-1.3338	-2.312	-2.7465	0.35
12773	*ZBTB38*	2.6089	-0.37495	-1.1856	-1.877	0.35
12774	*GTF2H1*	2.7433	-0.74367	-0.95184	-1.4914	0.35
12775	*TMBIM4*	0.94123	0.29744	-0.1911	-1.5225	0.35
12776	*LOC100130880*	0.81884	0.21512	0.01282	-0.5313	0.35
12777	*CCDC33*	1.8612	-0.3196	-0.49565	-1.2393	0.35
12778	*HNMT*	0.94269	0.43923	-0.33689	-1.1521	0.35
12779	*SPIB*	1.3489	0.49055	-0.79453	-2.2712	0.35
12780	*ADH1C*	0.74964	0.24134	0.05263	-1.392	0.35
12781	*EPO*	1.2066	0.37252	-0.5357	-1.5906	0.35

12782	*LGALS16*	3.4113	-0.92975	-1.4385	-2.1342	0.35
12783	*PPP1R18*	3.9849	-0.69865	-2.2432	-2.7728	0.35
12784	*RIF1*	1.4206	0.063606	-0.44134	-1.7306	0.35
12785	*ATOX1*	1.5499	-0.06364	-0.44353	-0.53069	0.35
12786	*SLCO1B7*	0.47277	0.38592	0.18263	0.13578	0.35
12787	*RAB11B*	3.4807	-0.78229	-1.6571	-2.1809	0.35
12788	*KLK3*	0.75845	0.33595	-0.05495	-0.44353	0.35
12789	*COPS4*	2.4659	-0.55929	-0.86744	-1.5577	0.35
12790	*ANXA5*	3.3983	-0.99275	-1.367	-1.5703	0.35
12791	*POU5F1*	1.6977	0.000252	-0.65949	-1.7595	0.35
12792	*TAAR5*	0.47011	0.46058	0.10737	-1.3294	0.35
12793	*NEDD8-MDP1*	1.9249	0.35566	-1.2438	-1.7915	0.35
12794	*CABP7*	2.5286	-0.0409	-1.4523	-1.8816	0.35
12795	*PPIH*	0.97026	0.11609	-0.0515	-1.4597	0.34
12796	*CALCA*	2.2054	0.011667	-1.1825	-1.6847	0.34
12797	*BTBD3*	1.4964	0.6285	-1.091	-1.8761	0.34
12798	*LRRC37A*	1.9782	-0.17026	-0.77409	-1.2055	0.34
12799	*JARID2*	0.93333	0.13547	-0.0354	-1.492	0.34
12800	*ABCG5*	1.5898	-0.15509	-0.40292	-1.3794	0.34
12801	*DNAJC3*	2.3586	0.14048	-1.4683	-2.1046	0.34
12802	*LY9*	1.5719	0.65411	-1.1953	-1.4864	0.34
12803	*CBX3*	1.2969	0.53779	-0.8046	-1.6276	0.34
12804	*SLC25A2*	1.3256	0.87789	-1.1739	-1.5525	0.34
12805	*PCK1*	1.109	0.41823	-0.49778	-0.93579	0.34
12806	*DEK*	0.80898	0.12633	0.09402	-0.73502	0.34
12807	*RAB2B*	0.90012	0.18267	-0.05348	-0.52329	0.34
12808	*DENND6A*	3.2874	-0.82366	-1.4359	-1.8297	0.34
12809	*GOLGA8B*	3.1996	-0.93011	-1.2417	-2.1496	0.34
12810	*UNC5D*	1.1826	0.17101	-0.32828	-1.0206	0.34
12811	*CAMK4*	0.99363	0.30735	-0.2765	-0.72666	0.34
12812	*PPP3R1*	1.3114	0.10209	-0.3891	-1.5992	0.34
12813	*TEK*	0.8867	0.15992	-0.0235	-0.98754	0.34
12814	*NRN1*	1.4293	0.1293	-0.53578	-0.57285	0.34
12815	*RBMY1D*	0.47677	0.39778	0.148	-1.3165	0.34
12816	*ZNF408*	0.83556	0.57453	-0.38767	-1.6525	0.34
12817	*TMEM251*	1.1578	0.32387	-0.46032	-0.53041	0.34
12818	*KIAA2013*	2.7109	0.056044	-1.7458	-2.1626	0.34
12819	*MEGF11*	0.8311	0.11435	0.0752	-1.9967	0.34
12820	*ZNF90*	1.6769	0.50206	-1.1587	-1.8646	0.34
12821	*C9orf139*	0.64529	0.21262	0.16214	-0.53911	0.34
12822	*SERF1A*	1.5122	-0.20806	-0.28472	-1.7781	0.34
12823	*BCAS1*	0.44955	0.39529	0.17417	-1.1807	0.34
12824	*SDK2*	1.5789	0.097822	-0.65811	-0.75491	0.34
12825	*TENM1*	0.87423	0.099278	0.04456	0.009954	0.34
12826	*KCNE5*	0.76777	0.68772	-0.43793	-0.86718	0.34
12827	*FAM20C*	1.5061	0.45617	-0.94517	-1.6553	0.34

12828	*ATP5J2*	0.97501	0.062587	-0.02228	-1.0626	0.34
12829	*DENR*	2.5681	-0.71856	-0.83436	-1.8157	0.34
12830	*CETN3*	0.93447	0.52567	-0.44575	-2.2825	0.34
12831	*CAPG*	1.1342	0.71227	-0.83276	-1.7802	0.34
12832	*ASB12*	0.47554	0.28798	0.24891	-0.53529	0.34
12833	*NUPR1L*	3.2638	-0.75304	-1.4994	-1.8478	0.34
12834	*ACTR1A*	0.6088	0.48551	-0.08298	-0.21262	0.34
12835	*CSK*	0.83475	0.41241	-0.23607	-2.7016	0.34
12836	*SLC25A25*	1.719	-0.19422	-0.51373	-1.5393	0.34
12837	*CCDC3*	1.2282	0.95063	-1.1685	-2.1732	0.34
12838	*LHX4*	3.8991	-0.4073	-2.4815	-3.2901	0.34
12839	*PPME1*	1.2824	0.033922	-0.30611	-0.34533	0.34
12840	*UBE2Q2L*	1.2694	0.32682	-0.58603	-0.94341	0.34
12841	*ERCC1*	1.638	0.28933	-0.91767	-2.1669	0.34
12842	*TGFBR1*	0.76478	0.4002	-0.1564	-2.4518	0.34
12843	*CD53*	3.0869	-0.79225	-1.2864	-1.4819	0.34
12844	*FAM71E2*	1.0366	0.116	-0.1449	-1.6213	0.34
12845	*ERN2*	1.2424	0.25837	-0.49396	-0.62307	0.34
12846	*RRAGD*	2.3334	-0.0429	-1.2837	-3.0124	0.34
12847	*CCDC73*	0.57736	0.3849	0.04383	-1.7918	0.34
12848	*CIB1*	1.7136	0.3073	-1.0153	-1.7129	0.34
12849	*ABL2*	1.0093	0.035623	-0.04002	-0.23777	0.33
12850	*SLC7A4*	1.1794	0.73711	-0.91253	-0.94019	0.33
12851	*CYP4F11*	3.4966	-1.0904	-1.403	-1.4894	0.33
12852	*SEPT2*	1.2831	0.20048	-0.48085	-1.3991	0.33
12853	*PCDHGA12*	1.0896	0.84592	-0.93287	-2.0452	0.33
12854	*MB21D2*	0.90285	0.69078	-0.59119	-0.97891	0.33
12855	*EIF4E3*	1.7355	0.57904	-1.3127	-1.6951	0.33
12856	*CCDC42B*	2.6125	-0.4939	-1.1169	-1.6494	0.33
12857	*DMTF1*	2.1402	-0.49295	-0.64599	-2.4633	0.33
12858	*SSU72*	0.91161	0.74669	-0.65758	-1.6635	0.33
12859	*PROSER2*	0.94374	0.078277	-0.02138	-1.4325	0.33
12860	*CDRT15*	0.69871	0.33634	-0.03456	-0.41703	0.33
12861	*VAT1*	1.73	0.93717	-1.6672	-2.3537	0.33
12862	*CIR1*	1.4525	0.49511	-0.94777	-1.9833	0.33
12863	*KAT7*	1.5061	0.19482	-0.70136	-2.565	0.33
12864	*SAMHD1*	2.5497	-0.4705	-1.0797	-1.6216	0.33
12865	*LDHA*	2.6726	-0.62886	-1.0444	-1.2189	0.33
12866	*HIST1H2BD*	0.62614	0.37073	0.00087	-1.2525	0.33
12867	*LCE2D*	0.85771	0.29629	-0.15766	-1.2097	0.33
12868	*GOLGA6L10*	2.564	-0.29513	-1.2728	-2.5361	0.33
12869	*NUB1*	1.1261	0.36293	-0.49311	-1.1387	0.33
12870	*CUL5*	1.4478	0.31165	-0.76371	-1.2736	0.33
12871	*TMEM88B*	2.2254	-0.13461	-1.0953	-2.2167	0.33
12872	*LINGO1*	2.76	-0.03338	-1.7313	-2.0548	0.33
12873	*MEF2B*	1.9684	-0.47033	-0.50339	-1.0987	0.33

12874	*ZNF345*	0.99926	0.20031	-0.20512	-0.94472	0.33
12875	*RGL2*	1.2872	0.74883	-1.0416	-1.4234	0.33
12876	*HNRNPH1*	1.6498	-0.17606	-0.47952	-0.5328	0.33
12877	*CDKAL1*	1.8011	0.18336	-0.99043	-1.7495	0.33
12878	*CA3*	0.49798	0.33954	0.15608	0.13042	0.33
12879	*ZSCAN10*	1.3855	-0.05429	-0.33799	-1.0863	0.33
12880	*ZNF385C*	1.9626	-0.38198	-0.58743	-2.308	0.33
12881	*COLGALT1*	2.0975	0.22464	-1.3292	-1.9166	0.33
12882	*TAC1*	0.64941	0.32777	0.01511	-1.0315	0.33
12883	*MYDGF*	0.76694	0.45479	-0.23166	-2.6445	0.33
12884	*EDIL3*	4.4831	-1.2766	-2.217	-3.8314	0.33
12885	*MAP4K5*	0.99409	0.01805	-0.02345	-1.2118	0.33
12886	*SYNDIG1L*	1.7561	0.69943	-1.4683	-1.5579	0.33
12887	*MCM3*	2.8436	-0.4933	-1.3633	-2.1105	0.33
12888	*GPR34*	1.1183	0.14582	-0.27742	-0.5328	0.33
12889	*DPH6*	3.6251	-0.9027	-1.7364	-2.2292	0.33
12890	*ABR*	1.1225	0.025575	-0.16242	-1.1666	0.33
12891	*DPY30*	1.2807	0.26985	-0.56518	-2.2485	0.33
12892	*C15orf38-AP3S2*	2.8093	-0.69146	-1.1333	-1.219	0.33
12893	*SEZ6L2*	3.2547	-1.0999	-1.1706	-2.7374	0.33
12894	*LMO7DN*	0.81768	0.19631	-0.03006	-0.95753	0.33
12895	*TOB2*	1.0159	0.70864	-0.74086	-1.7134	0.33
12896	*FMO5*	1.9313	-0.02606	-0.92157	-1.7846	0.33
12897	*CGB1*	0.49383	0.26853	0.22055	-1.1617	0.33
12898	*HCN4*	1.7489	0.56594	-1.3322	-2.1829	0.33
12899	*TMEM133*	0.93403	0.48157	-0.43305	-0.44353	0.33
12900	*MAP2*	1.8656	0.25192	-1.1357	-2.2218	0.33
12901	*TAOK3*	0.69837	0.36116	-0.07891	-0.61807	0.33
12902	*FUCA1*	1.5604	0.24743	-0.82729	-1.4207	0.33
12903	*ZNF239*	0.67516	0.25848	0.04685	-1.6422	0.33
12904	*GOLM1*	1.1608	0.12623	-0.30743	-0.35279	0.33
12905	*NRG3*	2.3178	-0.53506	-0.80341	-1.3531	0.33
12906	*LNX1*	1.202	0.086766	-0.3099	-0.37196	0.33
12907	*IPO9*	2.5467	0.17147	-1.7412	-1.743	0.33
12908	*ADCY9*	0.63178	0.58744	-0.24287	-1.1317	0.33
12909	*MPDZ*	1.3681	-0.15181	-0.24003	-0.8108	0.33
12910	*PTTG1IP*	0.85179	0.45202	-0.32773	-0.80548	0.33
12911	*HSPA1A*	0.75885	0.56457	-0.34814	-1.6984	0.33
12912	*ALDH7A1*	1.0849	0.10414	-0.21405	-1.4361	0.32
12913	*C6orf120*	1.1301	-0.05863	-0.09671	-1.4538	0.32
12914	*ZNF420*	1.7312	-0.32162	-0.43488	-0.69157	0.32
12915	*AKNA*	1.6721	0.40382	-1.1021	-1.2975	0.32
12916	*DUXA*	2.3561	-0.36971	-1.0126	-2.7317	0.32
12917	*PDHB*	3.332	-1.1595	-1.1988	-1.2441	0.32
12918	*PCDH17*	1.4266	-0.15177	-0.30209	-0.94364	0.32
12919	*GLT1D1*	2.1387	0.63518	-1.8013	-2.0446	0.32

12920	*RFTN2*	2.2194	0.045762	-1.2935	-1.7364	0.32
12921	*ZMYM4*	2.7648	-0.25912	-1.535	-2.5725	0.32
12922	*AUNIP*	3.1188	-1.0318	-1.1169	-2.6672	0.32
12923	*PMF1*	1.3071	0.10618	-0.44353	-2.0791	0.32
12924	*CCDC176*	1.0001	0.75181	-0.78229	-3.0045	0.32
12925	*CD5L*	1.0183	0.17256	-0.22287	-1.0682	0.32
12926	*AGGF1*	1.288	0.8237	-1.1439	-1.9833	0.32
12927	*ZNF736*	0.82263	0.69988	-0.55531	-1.1642	0.32
12928	*TBC1D22B*	0.61063	0.45104	-0.09459	-1.9318	0.32
12929	*PLEKHG6*	1.1295	0.41902	-0.58221	-1.6528	0.32
12930	*SLC25A39*	0.51455	0.38421	0.06361	0.014048	0.32
12931	*OR5M8*	1.022	0.36609	-0.42708	-1.9086	0.32
12932	*CPEB2*	0.83829	0.76876	-0.64742	-1.0857	0.32
12933	*KCTD20*	0.67581	0.32273	-0.04099	-1.089	0.32
12934	*STX4*	3.2295	-0.68167	-1.5913	-2.0116	0.32
12935	*TBC1D10A*	1.0861	1.0455	-1.1759	-1.9623	0.32
12936	*HIST1H2BC*	1.5255	-0.24569	-0.32466	-0.73911	0.32
12937	*NOV*	0.78974	0.15819	0.00684	-1.2225	0.32
12938	*TTC23*	0.46167	0.34102	0.15122	-1.186	0.32
12939	*S100Z*	2.5203	-0.61746	-0.94897	-1.6952	0.32
12940	*GNGT2*	1.7149	0.69919	-1.4607	-1.7846	0.32
12941	*ANKDD1A*	0.90769	0.37779	-0.33315	-0.89316	0.32
12942	*TIMMDC1*	0.66623	0.5016	-0.2157	-1.7577	0.32
12943	*FDX1L*	3.3224	-0.8071	-1.5632	-1.5757	0.32
12944	*PLEKHA3*	1.182	0.17427	-0.4046	-1.3165	0.32
12945	*PURA*	2.8444	-0.90281	-0.99049	-1.5406	0.32
12946	*XRCC6*	3.8832	-1.0671	-1.8651	-2.0789	0.32
12947	*PSME4*	1.413	0.080058	-0.54302	-0.78775	0.32
12948	*RNASET2*	3.9031	-1.3878	-1.5658	-1.6401	0.32
12949	*LUC7L*	2.4864	-0.54054	-0.99676	-1.4607	0.32
12950	*LAMP2*	0.87891	0.87638	-0.80633	-2.39	0.32
12951	*SECTM1*	0.72393	0.46466	-0.24016	-1.4498	0.32
12952	*MSC*	3.3033	-1.1739	-1.183	-1.6659	0.32
12953	*FAM188A*	1.0655	0.33295	-0.45246	-0.96031	0.32
12954	*OR5H1*	0.68288	0.46721	-0.20445	-1.6555	0.32
12955	*SEZ6L*	1.7322	-0.17647	-0.61017	-2.1648	0.32
12956	*UBE2E1*	2.2535	-0.3555	-0.95322	-1.4511	0.31
12957	*APH1B*	1.5549	-0.18118	-0.42934	-1.2937	0.31
12958	*KDELC2*	1.5905	0.17568	-0.82208	-1.1912	0.31
12959	*TNFAIP8L1*	1.6303	0.72478	-1.4116	-2.7379	0.31
12960	*C7orf77*	0.80918	0.75046	-0.61641	-1.0313	0.31
12961	*DGKE*	1.3914	-0.07282	-0.37563	-0.46268	0.31
12962	*PLAGL2*	2.5981	-0.81522	-0.84001	-1.9278	0.31
12963	*TMEM141*	1.9419	-0.49095	-0.50815	-2.1404	0.31
12964	*FASTKD3*	1.4284	-0.14207	-0.34363	-1.631	0.31
12965	*DNAJC15*	1.1809	0.56796	-0.80675	-1.338	0.31

12966	*RUVBL1*	1.3404	0.45096	-0.8504	-1.0824	0.31
12967	*CCDC25*	1.1372	0.27529	-0.47183	-0.94897	0.31
12968	*AGAP1*	1.0901	0.12588	-0.27541	-1.7338	0.31
12969	*RNF20*	2.3173	0.056768	-1.4335	-2.7623	0.31
12970	*VEGFC*	0.55033	0.2175	0.17217	-0.58691	0.31
12971	*CHAD*	3.6695	-0.99699	-1.733	-1.7995	0.31
12972	*TBR1*	0.76489	0.5237	-0.34927	-2.1251	0.31
12973	*ERICH3*	1.1417	0.92367	-1.1263	-1.6836	0.31
12974	*SNCB*	2.1298	-0.25295	-0.93868	-1.8376	0.31
12975	*MTIF3*	2.0881	-0.00995	-1.141	-1.6105	0.31
12976	*TFCP2L1*	0.84537	0.23246	-0.14117	-1.8435	0.31
12977	*EXO5*	2.9241	-0.99137	-0.99608	-1.9459	0.31
12978	*ACTL6B*	2.4473	0.020029	-1.5311	-2.4244	0.31
12979	*MIOS*	2.1233	-0.29121	-0.89593	-0.98574	0.31
12980	*FLOT1*	0.95434	0.44297	-0.46155	-1.2346	0.31
12981	*GBP2*	2.2504	0.30909	-1.6243	-1.7911	0.31
12982	*PLEKHA1*	1.6583	0.23544	-0.95868	-1.3694	0.31
12983	*TMEM178B*	1.3228	0.43735	-0.82696	-1.1852	0.31
12984	*ASB10*	1.3815	0.10365	-0.55275	-1.658	0.31
12985	*HIATL1*	1.153	0.10354	-0.32466	-1.4055	0.31
12986	*EGFL7*	2.2986	-0.01883	-1.3481	-1.772	0.31
12987	*RAB6B*	1.2885	0.56755	-0.92444	-1.94	0.31
12988	*MGEA5*	1.5611	0.71885	-1.3496	-1.9997	0.31
12989	*PSMC5*	1.9142	0.44422	-1.4299	-1.6885	0.31
12990	*MOCS2*	0.91321	0.29904	-0.28391	-2.2528	0.31
12991	*DHX40*	3.608	-1.0212	-1.6587	-2.2075	0.31
12992	*KIF11*	3.168	-0.79165	-1.4485	-1.7065	0.31
12993	*ARRDC2*	2.4268	-0.4155	-1.0846	-1.1053	0.31
12994	*RTF1*	2.5054	-0.17705	-1.4022	-1.6949	0.31
12995	*VKORC1L1*	2.1033	-0.20423	-0.97333	-1.8443	0.31
12996	*FRY*	0.6994	0.21745	0.00806	-0.44198	0.31
12997	*DAAM1*	0.8516	0.050931	0.02185	-0.33741	0.31
12998	*PRKCG*	0.49899	0.48679	-0.06177	-1.7591	0.31
12999	*MAL*	1.0967	0.15078	-0.32677	-1.7009	0.31
13000	*HSD11B1L*	4.6868	-1.8366	-1.9295	-2.3537	0.31
13001	*ERO1LB*	3.7701	-0.99226	-1.8572	-2.3525	0.31
13002	*KCNRG*	2.5431	-0.50472	-1.1189	-1.1856	0.31
13003	*IRX5*	2.4285	-0.59921	-0.91045	-1.1357	0.31
13004	*CENPH*	1.1514	0.33222	-0.56492	-1.3591	0.31
13005	*ULBP2*	0.99062	0.53719	-0.60953	-0.95504	0.31
13006	*CLCN4*	0.77215	0.43717	-0.29114	-1.2977	0.31
13007	*RERG*	2.4874	-0.35899	-1.2125	-1.4019	0.31
13008	*CCDC30*	0.57799	0.28275	0.05503	-1.317	0.31
13009	*CHST9*	1.7734	0.72823	-1.587	-1.6648	0.30
13010	*SGSM1*	2.578	-0.5946	-1.0688	-1.4498	0.30
13011	*GTPBP6*	0.83111	0.38135	-0.29792	-2.1947	0.30

13012	*OR10H5*	1.9062	0.16733	-1.1595	-1.7533	0.30
13013	*ASPSCR1*	0.85138	0.17884	-0.11729	-0.49684	0.30
13014	*ANTXR1*	0.43191	0.34151	0.1395	-0.42046	0.30
13015	*TGS1*	0.50604	0.24958	0.1573	-1.8604	0.30
13016	*CAMK2N2*	2.6701	-0.22742	-1.5312	-2.0218	0.30
13017	*OR56B1*	1.0411	0.26286	-0.39248	-0.82768	0.30
13018	*CDK11B*	0.60384	0.42522	-0.11781	-1.5684	0.30
13019	*USP17L8*	3.2819	-0.6568	-1.714	-2.0664	0.30
13020	*KLHL29*	1.8526	0.20767	-1.1492	-1.8112	0.30
13021	*SPANXC*	0.62844	0.19503	0.08753	-0.81155	0.30
13022	*RSF1*	2.3497	0.53344	-1.9727	-2.5007	0.30
13023	*OR4A15*	3.3344	-1.1529	-1.2719	-2.6275	0.30
13024	*KCNS2*	0.62915	0.53561	-0.25597	-0.3866	0.30
13025	*FOXK2*	1.8886	0.29982	-1.2803	-3.0219	0.30
13026	*ZIM3*	0.55312	0.23974	0.11525	-0.1435	0.30
13027	*TMEM92*	0.79659	0.53569	-0.4247	-2.22	0.30
13028	*IL13*	1.3078	0.9004	-1.3007	-1.6508	0.30
13029	*TARBP1*	0.93664	0.2954	-0.32665	-1.027	0.30
13030	*SLC25A45*	1.2424	-0.05096	-0.28609	-0.90192	0.30
13031	*YIPF1*	0.64766	0.32361	-0.06675	-1.3718	0.30
13032	*PEMT*	0.54202	0.39341	-0.03164	-2.672	0.30
13033	*MYO5A*	0.75598	0.5179	-0.3703	-1.1492	0.30
13034	*NARS*	0.95433	0.046036	-0.09738	-1.4541	0.30
13035	*SSFA2*	3.2399	-1.1322	-1.205	-1.7675	0.30
13036	*NFYA*	1.4406	0.17112	-0.71078	-1.2867	0.30
13037	*COMMD3*	1.3577	-0.19331	-0.26347	-0.73202	0.30
13038	*MRPS17*	3.1085	-0.60221	-1.6069	-1.6139	0.30
13039	*TMEM25*	1.5396	-0.06631	-0.57429	-1.8714	0.30
13040	*SPINK5*	0.92109	0.30279	-0.32495	-0.9968	0.30
13041	*NT5DC1*	1.2873	0.20251	-0.5911	-1.6469	0.30
13042	*FRG2B*	1.7986	-0.44887	-0.45124	-0.94493	0.30
13043	*STATH*	2.1865	0.36457	-1.6526	-1.9054	0.30
13044	*VPS53*	1.9015	0.32523	-1.3294	-2.2763	0.30
13045	*ATXN7L3B*	2.9559	-0.45373	-1.6049	-2.1732	0.30
13046	*FLI1*	0.54479	0.4616	-0.11145	-0.34111	0.30
13047	*MORN4*	2.4335	-0.14795	-1.3911	-1.6691	0.30
13048	*CASD1*	1.0623	0.66948	-0.83739	-1.3665	0.30
13049	*LDHAL6A*	0.87112	0.24958	-0.22673	-0.78229	0.30
13050	*RTN1*	0.9072	0.5381	-0.55134	-2.0384	0.30
13051	*OR5T1*	1.7742	0.42647	-1.3068	-2.6456	0.30
13052	*OGFOD1*	0.68585	0.30586	-0.09789	-1.2099	0.30
13053	*EIF3J*	2.7211	-0.35891	-1.4689	-1.5524	0.30
13054	*CENPJ*	1.4245	-0.12333	-0.40793	-0.75817	0.30
13055	*NCOA5*	1.2528	0.8717	-1.232	-1.6162	0.30
13056	*ALDH2*	1.2301	-0.04536	-0.29267	-1.3617	0.30
13057	*PEX1*	2.6256	-0.66914	-1.0644	-1.8792	0.30

13058	*KANK3*	1.2802	-0.14701	-0.24137	-0.6251	0.30
13059	*ANKRD28*	1.3502	0.11542	-0.57429	-1.4597	0.30
13060	*BPIFB3*	0.35456	0.27765	0.25899	-0.61555	0.30
13061	*ARNTL*	1.5436	0.56698	-1.2202	-1.8682	0.30
13062	*ZNF569*	1.002	0.59361	-0.70558	-0.93162	0.30
13063	*SMUG1*	2.9908	-0.80727	-1.2936	-1.7364	0.30
13064	*TRIM71*	0.83762	0.088247	-0.03623	-0.59647	0.30
13065	*OR5A2*	2.3062	0.52879	-1.9455	-2.6072	0.30
13066	*AGFG2*	1.0058	0.36145	-0.4783	-2.011	0.30
13067	*COPA*	2.3106	0.079723	-1.5016	-2.016	0.30
13068	*IGSF6*	2.966	0.45892	-2.537	-3.2595	0.30
13069	*SLC10A2*	1.9184	0.70584	-1.7364	-2.0946	0.30
13070	*TKTL1*	0.66358	0.21311	0.00998	-0.5203	0.30
13071	*IRF6*	2.3461	-0.27911	-1.1815	-2.4172	0.30
13072	*HNRNPD*	1.278	-0.08654	-0.30609	-0.91283	0.30
13073	*TAS2R5*	0.50885	0.26163	0.11435	-1.0751	0.29
13074	*KTN1*	2.051	-0.07227	-1.0942	-1.2246	0.29
13075	*LARP1*	1.9145	-0.14211	-0.88804	-0.94312	0.29
13076	*CPNE7*	2.9284	-0.62928	-1.4151	-1.5611	0.29
13077	*MFSD1*	0.73954	0.58119	-0.43694	-1.0095	0.29
13078	*TGFBI*	2.494	-0.45572	-1.1545	-2.5346	0.29
13079	*LRGUK*	0.56598	0.31369	0.00277	-0.30326	0.29
13080	*PLRG1*	2.7792	-0.8462	-1.0509	-1.7649	0.29
13081	*KIAA0020*	1.4202	-0.02854	-0.50961	-1.2995	0.29
13082	*CAPZA3*	1.2312	0.44554	-0.79474	-1.6524	0.29
13083	*PGLYRP3*	0.7006	0.45098	-0.26996	-1.0283	0.29
13084	*TRPV5*	1.3328	0.33838	-0.78959	-1.1429	0.29
13085	*NBR1*	3.9315	-1.0761	-1.9748	-2.29	0.29
13086	*OTOP2*	0.62822	0.15387	0.09832	-2.9772	0.29
13087	*EFCAB2*	0.76572	0.42745	-0.31294	-1.9172	0.29
13088	*LOC100506388*	1.5721	-0.10216	-0.58985	-1.5039	0.29
13089	*ISYNA1*	1.0127	0.19141	-0.32466	-1.5225	0.29
13090	*HAO1*	1.3415	-0.02092	-0.44135	-1.2576	0.29
13091	*FANCD2OS*	1.938	-0.20822	-0.85199	-1.3132	0.29
13092	*DIEXF*	0.74021	0.43498	-0.29752	-0.97393	0.29
13093	*RARS2*	3.4214	-0.66731	-1.877	-1.8996	0.29
13094	*GUCY2D*	0.65784	0.64897	-0.43043	-2.907	0.29
13095	*ZNF366*	1.1717	-0.06833	-0.22763	-0.83277	0.29
13096	*TMEM43*	0.9887	0.02792	-0.14117	-1.0907	0.29
13097	*OR4C13*	2.2529	0.034936	-1.4128	-1.8404	0.29
13098	*C2CD4C*	1.5024	0.15485	-0.78229	-1.4776	0.29
13099	*USP17L11*	1.1597	0.26433	-0.54921	-1.3216	0.29
13100	*SLC36A3*	0.99785	0.010955	-0.13487	-0.36821	0.29
13101	*ZNF721*	1.0198	0.16511	-0.31132	-0.95044	0.29
13102	*DESI2*	2.6248	-0.38025	-1.3718	-2.0541	0.29
13103	*OR51V1*	1.7734	-0.44353	-0.45786	-1.1395	0.29

13104	*QRICH2*	2.9654	-0.9619	-1.1316	-1.1651	0.29
13105	*FAU*	1.6361	0.14492	-0.91013	-1.6012	0.29
13106	*SCGB1C2*	0.70671	0.49407	-0.33089	-1.9255	0.29
13107	*EIF2AK2*	1.7968	-0.45604	-0.47136	-1.6116	0.29
13108	*FZD8*	1.6382	-0.18859	-0.58049	-1.3208	0.29
13109	*PPP2R2C*	0.88608	0.72504	-0.74219	-2.1502	0.29
13110	*POLE*	3.0951	-0.6386	-1.5884	-2.224	0.29
13111	*FAM163A*	1.9951	-0.00456	-1.124	-1.2566	0.29
13112	*TTC19*	1.3434	0.59503	-1.0726	-1.172	0.29
13113	*ACSL5*	2.2811	-0.31885	-1.098	-1.6525	0.29
13114	*PPIL4*	1.2188	0.98977	-1.3451	-1.5194	0.29
13115	*DHRS4L2*	2.7064	0.060969	-1.9047	-2.0466	0.29
13116	*ZNF717*	0.77656	0.59128	-0.50589	-0.94827	0.29
13117	*CLNK*	2.2781	-0.19053	-1.2261	-1.3346	0.29
13118	*CDK15*	1.3864	0.78479	-1.3104	-2.0999	0.29
13119	*PLEKHG2*	2.1257	0.54076	-1.806	-2.223	0.29
13120	*SNTB1*	0.96949	-0.00833	-0.10088	-1.1553	0.29
13121	*LIN7B*	1.5701	-0.02072	-0.68911	-1.7649	0.29
13122	*ZMAT2*	0.77419	0.37868	-0.2928	-0.78754	0.29
13123	*ARHGAP22*	0.96347	0.4644	-0.56812	-1.748	0.29
13124	*STK32A*	0.99547	0.56981	-0.70581	-1.3085	0.29
13125	*RNF212*	2.3765	-0.4082	-1.1096	-4.7884	0.29
13126	*INSL3*	0.56732	0.24322	0.04806	-0.17509	0.29
13127	*SMIM17*	1.7097	-0.11793	-0.73419	-1.6158	0.29
13128	*RBM19*	2.436	-0.58633	-0.99217	-1.3298	0.29
13129	*MED8*	0.54972	0.16453	0.14261	-1.5348	0.29
13130	*USP42*	0.56568	0.18851	0.10242	-1.0564	0.29
13131	*ACYP2*	2.6787	-0.89258	-0.9304	-1.8293	0.29
13132	*CCL18*	2.7361	-0.66164	-1.2192	-1.2485	0.29
13133	*LGI1*	1.1162	-0.06413	-0.19735	-1.0178	0.28
13134	*CREBZF*	0.52019	0.31774	0.01649	-1.8611	0.28
13135	*UGGT2*	1.7107	-0.39664	-0.46017	-1.0003	0.28
13136	*RRP36*	1.4767	0.32911	-0.95315	-1.5707	0.28
13137	*SYTL2*	2.3223	-0.66731	-0.80234	-1.1676	0.28
13138	*TGM7*	0.55195	0.2836	0.01621	-1.1466	0.28
13139	*BAAT*	0.68631	0.53898	-0.37358	-2.4328	0.28
13140	*ZNF701*	2.0619	0.18341	-1.3941	-1.5906	0.28
13141	*BZRAP1*	2.439	-0.55029	-1.0385	-1.6885	0.28
13142	*OR1E1*	1.9571	-0.168	-0.93895	-1.1102	0.28
13143	*OR2B6*	1.0245	0.91864	-1.0947	-1.5104	0.28
13144	*HIST1H2AI*	1.0082	0.25931	-0.42004	-1.8851	0.28
13145	*U2AF1L4*	2.7584	-0.1747	-1.7365	-2.4094	0.28
13146	*OR5M9*	1.917	0.58233	-1.6524	-1.773	0.28
13147	*CYP3A5*	1.0565	0.17295	-0.38428	-1.5801	0.28
13148	*RBBP8NL*	1.2735	-0.05456	-0.37417	-1.631	0.28
13149	*TECRL*	0.98259	0.43947	-0.57825	-0.80146	0.28

13150	*ANXA6*	1.0343	0.012414	-0.20292	-2.0059	0.28
13151	*PRKCA*	1.6395	0.44773	-1.2437	-1.9302	0.28
13152	*TYW3*	1.3076	0.099372	-0.56444	-1.843	0.28
13153	*COL19A1*	2.7664	-0.77789	-1.1461	-1.1758	0.28
13154	*DENND4A*	1.1735	-0.02163	-0.30965	-1.8038	0.28
13155	*MAP6D1*	1.2292	0.068993	-0.45604	-1.8838	0.28
13156	*ANKRD20A3*	1.4515	-0.25762	-0.35211	-1.5331	0.28
13157	*TBL1X*	1.7042	-0.17298	-0.68999	-1.6306	0.28
13158	*TMEM26*	1.5352	-0.31414	-0.37995	-1.0691	0.28
13159	*RBMX*	1.8744	-0.00833	-1.025	-1.3632	0.28
13160	*COPS6*	0.51639	0.45083	-0.12725	-0.70976	0.28
13161	*SEC23B*	1.5089	0.18147	-0.8504	-0.95527	0.28
13162	*IL1R2*	3.6038	-1.2705	-1.4935	-2.2094	0.28
13163	*PIP5KL1*	3.4378	-1.1944	-1.4036	-1.748	0.28
13164	*ACYP1*	0.44793	0.34532	0.04584	-0.12189	0.28
13165	*CD7*	3.8417	-1.1287	-1.8742	-2.9143	0.28
13166	*C1orf229*	0.62565	0.11317	0.09965	-1.9965	0.28
13167	*CRISPLD2*	0.4834	0.45449	-0.09984	-2.1066	0.28
13168	*NDUFA4*	1.1524	0.29744	-0.61436	-1.3694	0.28
13169	*SLC7A5*	0.72742	0.24671	-0.14122	-1.3849	0.28
13170	*CHD6*	1.0251	0.10965	-0.30279	-0.46665	0.28
13171	*ASB8*	1.2781	0.083593	-0.53009	-1.8996	0.28
13172	*ESCO1*	0.74858	0.52571	-0.44353	-0.56489	0.28
13173	*CD2AP*	0.58729	0.18898	0.05386	-0.94563	0.28
13174	*TMED7-TICAM2*	1.2694	0.31229	-0.75205	-1.8609	0.28
13175	*IGF2BP1*	1.5293	-0.15679	-0.54345	-0.88279	0.28
13176	*FADS2*	0.91459	0.60736	-0.69312	-0.7229	0.28
13177	*FAM178B*	0.90985	0.039496	-0.12096	-0.69073	0.28
13178	*ANKRD39*	1.4883	0.45681	-1.1169	-1.6836	0.28
13179	*RPL10L*	0.8797	-0.01508	-0.03675	-0.20848	0.28
13180	*RNASEK*	0.61627	0.50175	-0.29027	-0.63147	0.28
13181	*USMG5*	1.981	-0.53969	-0.61394	-0.88465	0.28
13182	*ZNF157*	0.59461	0.23846	-0.0062	-0.84134	0.28
13183	*EDARADD*	2.4591	0.10421	-1.7365	-2.0682	0.28
13184	*SLC22A11*	1.5832	-0.31842	-0.43845	-0.67395	0.28
13185	*RIMBP3B*	2.2822	-0.21821	-1.238	-1.3293	0.28
13186	*APOBEC3A*	2.8935	0.16266	-2.2313	-3.5756	0.27
13187	*TSTA3*	0.63433	0.21886	-0.02908	-1.7855	0.27
13188	*SLC46A2*	0.66345	0.61421	-0.45355	-0.71063	0.27
13189	*DDX60L*	1.3641	-0.02658	-0.51396	-0.55816	0.27
13190	*TSPYL5*	1.6153	0.36143	-1.1532	-2.1364	0.27
13191	*SCN11A*	1.9841	-0.48767	-0.67298	-0.79195	0.27
13192	*VPREB3*	0.75846	0.50819	-0.44353	-1.3729	0.27
13193	*N6AMT2*	1.0187	-0.07031	-0.12552	-1.4287	0.27
13194	*CLCN3*	0.72975	0.58279	-0.49107	-0.76582	0.27
13195	*LRRC26*	1.3025	0.087154	-0.56963	-1.0824	0.27

13196	*MADD*	0.5014	0.1857	0.13228	-1.5653	0.27
13197	*ZNF552*	2.1543	-0.66146	-0.67395	-1.9041	0.27
13198	*CCNT1*	0.75322	0.61081	-0.5458	-0.58553	0.27
13199	*RTL1*	0.80282	0.18187	-0.16651	-1.1021	0.27
13200	*NRSN2*	3.2998	-0.32466	-2.1574	-2.5647	0.27
13201	*PRDX2*	1.5021	0.29711	-0.98314	-1.7364	0.27
13202	*IFNK*	2.2345	-0.7061	-0.71268	-1.613	0.27
13203	*MYL12B*	1.0779	0.8964	-1.1598	-1.6246	0.27
13204	*NOP16*	2.5428	-0.50588	-1.2229	-1.2704	0.27
13205	*MNS1*	1.6375	-0.01861	-0.80607	-1.1598	0.27
13206	*PSEN1*	0.80687	0.33157	-0.32589	-0.68303	0.27
13207	*OGT*	1.6244	0.40853	-1.2208	-1.6555	0.27
13208	*ARFGAP3*	2.2607	-0.65068	-0.79813	-1.6266	0.27
13209	*NOXO1*	3.5892	-1.202	-1.577	-2.6939	0.27
13210	*ST7L*	2.7256	-0.95423	-0.96206	-2.3509	0.27
13211	*TRPV4*	2.2867	0.17475	-1.6524	-2.1956	0.27
13212	*LAMC1*	0.49605	0.29665	0.01621	-0.89269	0.27
13213	*SFXN3*	1.6173	0.8979	-1.7068	-2.5008	0.27
13214	*DAP3*	1.2193	-0.16485	-0.2465	-1.3433	0.27
13215	*PITPNC1*	1.179	0.21219	-0.58349	-2.1477	0.27
13216	*ATF5*	1.8888	-0.12456	-0.95682	-1.4325	0.27
13217	*CACNA1B*	0.55932	0.15515	0.09251	-0.78651	0.27
13218	*GCFC2*	1.0967	0.34471	-0.63511	-0.65924	0.27
13219	*IGLON5*	0.98197	-0.03697	-0.13928	-0.74013	0.27
13220	*MAT1A*	0.92643	0.38415	-0.50555	-1.3517	0.27
13221	*USP39*	0.77008	0.052299	-0.0175	-1.0716	0.27
13222	*PPP1R3B*	0.62844	0.30144	-0.12642	-0.80686	0.27
13223	*RBM27*	1.4726	-0.29042	-0.37913	-0.72825	0.27
13224	*DYTN*	1.164	0.1824	-0.54373	-1.4474	0.27
13225	*STX12*	1.0582	0.21171	-0.46747	-1.616	0.27
13226	*GYPE*	1.5377	-0.36747	-0.36913	-0.83469	0.27
13227	*RNFT2*	1.1825	0.25883	-0.64166	-0.76982	0.27
13228	*IL31*	1.8805	-0.31145	-0.76955	-1.9912	0.27
13229	*CLCC1*	1.6782	0.23807	-1.1169	-1.4591	0.27
13230	*ARHGEF33*	1.8217	0.27676	-1.3002	-1.3148	0.27
13231	*VTN*	2.7855	-0.45705	-1.5306	-2.5584	0.27
13232	*NIPAL3*	0.82378	0.27244	-0.29905	-1.4684	0.27
13233	*SLC38A8*	3.0906	-0.89155	-1.4027	-1.8212	0.27
13234	*SLCO6A1*	0.90604	0.47089	-0.58156	-1.9019	0.27
13235	*GRIN2C*	1.845	-0.48021	-0.56947	-1.7365	0.27
13236	*VSTM5*	0.53081	0.34989	-0.08572	-2.4933	0.26
13237	*ODF4*	2.6496	0.60333	-2.458	-2.825	0.26
13238	*DGAT1*	1.3274	1.0525	-1.5855	-2.2461	0.26
13239	*DSEL*	1.1552	0.17236	-0.53361	-1.3505	0.26
13240	*FGFR1*	1.9974	0.11075	-1.3151	-1.9559	0.26
13241	*LPPR4*	1.1912	0.77628	-1.175	-2.2948	0.26

13242	*LTA4H*	1.3797	0.21587	-0.80349	-1.6676	0.26
13243	*PEX6*	0.81109	0.78918	-0.8083	-1.333	0.26
13244	*P2RX5*	3.246	-0.38655	-2.0682	-2.8316	0.26
13245	*GJB5*	0.55758	0.4252	-0.19169	-0.30349	0.26
13246	*LRRC52*	0.99501	0.068606	-0.27291	-2.2485	0.26
13247	*C2orf68*	3.0519	-0.73071	-1.5309	-1.7068	0.26
13248	*MTM1*	0.73858	0.16089	-0.1095	-1.2383	0.26
13249	*SHPRH*	2.2139	-0.51703	-0.90743	-1.397	0.26
13250	*ALK*	1.3523	0.11832	-0.68133	-1.1888	0.26
13251	*MT1X*	1.9937	-0.40467	-0.8003	-1.6047	0.26
13252	*GGTLC2*	0.61199	0.33869	-0.16205	-0.67321	0.26
13253	*CD33*	1.2558	-0.05698	-0.41025	-2.0177	0.26
13254	*PLXDC2*	2.8204	-0.29305	-1.739	-2.7317	0.26
13255	*KLHDC8B*	0.81699	0.65417	-0.68303	-0.83527	0.26
13256	*OSTC*	0.51793	0.25552	0.01326	-0.89059	0.26
13257	*SEMA4A*	2.3097	-0.10243	-1.4206	-2.3287	0.26
13258	*GP5*	2.5144	-0.35805	-1.3701	-1.8999	0.26
13259	*UPF3A*	2.6152	-0.03587	-1.7935	-3.2023	0.26
13260	*INPP5E*	0.57318	0.39716	-0.1851	-0.2694	0.26
13261	*KRT71*	0.53795	0.23846	0.00882	-0.41703	0.26
13262	*HBEGF*	0.86634	0.067594	-0.15041	-0.93696	0.26
13263	*GLUD1*	3.4735	-0.69961	-1.9912	-2.4642	0.26
13264	*APOL4*	1.8217	-0.31309	-0.72635	-0.98983	0.26
13265	*UPK1A*	1.6714	-0.30419	-0.58589	-0.8597	0.26
13266	*KRT24*	0.699	0.48025	-0.39897	-2.0363	0.26
13267	*SOX9*	0.75031	0.73596	-0.70659	-3.9921	0.26
13268	*ATP10A*	2.9983	-0.18858	-2.0301	-2.2108	0.26
13269	*PWWP2A*	0.86909	0.67348	-0.76352	-0.89204	0.26
13270	*CTNS*	3.0369	-1.0528	-1.2052	-1.3821	0.26
13271	*TSEN15*	2.6108	-0.19042	-1.6417	-1.7887	0.26
13272	*SKIL*	1.3252	0.37477	-0.92131	-1.9622	0.26
13273	*CACNG3*	2.6576	-0.52473	-1.3543	-2.5728	0.26
13274	*UBQLN3*	2.7407	0.01805	-1.9811	-2.6257	0.26
13275	*POLR1E*	0.60959	0.41125	-0.24327	-0.2868	0.26
13276	*C17orf105*	0.87211	0.4154	-0.51002	-1.5132	0.26
13277	*CCR3*	3.7494	-1.3199	-1.6525	-2.6652	0.26
13278	*METTL9*	0.66946	0.55033	-0.44374	-0.48538	0.26
13279	*GPR108*	0.97037	0.52156	-0.7167	-1.4909	0.26
13280	*PTTG1*	3.0774	-0.36582	-1.9368	-2.2473	0.26
13281	*ATG3*	0.71308	0.3868	-0.32516	-0.76942	0.26
13282	*POLR2E*	0.96431	0.37008	-0.56027	-1.4281	0.26
13283	*TTN*	2.1231	-0.26055	-1.0889	-1.3773	0.26
13284	*RPL18A*	2.5782	-0.74141	-1.0633	-1.4258	0.26
13285	*C15orf62*	2.3849	-0.53615	-1.0756	-1.305	0.26
13286	*ZFP1*	0.74748	0.29981	-0.27498	-2.105	0.26
13287	*SSX2*	2.2631	-0.15276	-1.3382	-1.4856	0.26

13288	*OIP5*	1.3179	0.17128	-0.71717	-0.73202	0.26
13289	*TAS2R50*	2.1173	0.10131	-1.4473	-1.7247	0.26
13290	*FADS6*	0.80248	0.53269	-0.56444	-0.77521	0.26
13291	*ECM1*	1.1933	-0.14306	-0.28003	-0.95105	0.26
13292	*AOC3*	0.81508	0.16736	-0.21291	-1.0095	0.26
13293	*HNRNPL*	1.4919	0.25617	-0.97865	-2.3154	0.26
13294	*MMP2*	1.2626	0.54342	-1.0367	-2.0301	0.26
13295	*SMAD5*	1.2468	-0.01705	-0.46056	-1.2934	0.26
13296	*ACADSB*	2.9006	-0.46738	-1.6644	-2.674	0.26
13297	*FAH*	1.2823	0.3781	-0.89186	-1.9722	0.26
13298	*PIK3AP1*	0.88845	0.32362	-0.44353	-1.1727	0.26
13299	*PIH1D1*	1.5496	-0.22708	-0.55464	-1.3478	0.26
13300	*NIT1*	1.3511	-0.09762	-0.48561	-0.85095	0.26
13301	*MTX1*	3.1284	-0.86941	-1.4916	-1.5544	0.26
13302	*KAT5*	1.3251	0.99971	-1.5588	-1.7022	0.26
13303	*ZDHHC12*	1.278	0.51028	-1.0224	-1.485	0.26
13304	*TNFSF12-TNFSF1*[illegible]	0.73844	0.55385	-0.52711	-2.1926	0.26
13305	*RAB21*	3.1968	-0.9968	-1.4359	-1.4747	0.25
13306	*ACTR10*	2.9173	-0.50781	-1.6463	-2.4371	0.25
13307	*ITPRIPL2*	0.73659	0.064489	-0.03836	-0.402	0.25
13308	*ZFP57*	0.44684	0.25904	0.05677	-1.5978	0.25
13309	*ANKRD11*	0.87347	0.096106	-0.2073	-1.8678	0.25
13310	*PIGC*	0.75354	0.40756	-0.39924	-0.95495	0.25
13311	*ERC2*	1.2182	0.71334	-1.1698	-2.1921	0.25
13312	*ADGB*	1.3894	1.1155	-1.7438	-1.8212	0.25
13313	*TGFB1*	0.64948	0.15662	-0.04502	-1.3153	0.25
13314	*RNF133*	1.4413	-0.17744	-0.503	-1.4137	0.25
13315	*TLE6*	1.9383	-0.2251	-0.95321	-1.0528	0.25
13316	*COL17A1*	1.9646	0.66956	-1.8751	-2.3033	0.25
13317	*ASAH2B*	0.6879	0.093005	-0.02187	-0.80869	0.25
13318	*LCN12*	3.9412	-1.5874	-1.5959	-1.773	0.25
13319	*PPP2R5D*	0.30678	0.29956	0.15092	-0.79319	0.25
13320	*ZNF385D*	0.52027	0.20646	0.03043	-0.11314	0.25
13321	*LRRC34*	0.71993	0.66286	-0.62596	-1.1872	0.25
13322	*SLC12A2*	2.5968	-0.67141	-1.1698	-2.4853	0.25
13323	*PPP5D1*	1.5449	0.021236	-0.81058	-2.2599	0.25
13324	*SPINK8*	1.9554	-0.59959	-0.60058	-0.64678	0.25
13325	*TICAM1*	1.1025	-0.10946	-0.23825	-1.0014	0.25
13326	*DR1*	1.1969	-0.16069	-0.28164	-1.3476	0.25
13327	*ZNF665*	0.74341	0.21148	-0.20287	-1.0088	0.25
13328	*NUTM2A*	3.2332	-0.03356	-2.4481	-2.5764	0.25
13329	*CA6*	3.2923	-1.0901	-1.4509	-2.0476	0.25
13330	*FAM189A1*	1.9656	0.45421	-1.6689	-2.011	0.25
13331	*MOSPD2*	2.1486	-0.32748	-1.071	-3.1297	0.25
13332	*KRTAP4-6*	3.9246	-1.2352	-1.9397	-2.3251	0.25
13333	*NPBWR1*	1.8463	-0.03909	-1.0589	-1.8646	0.25

13334	*UQCRQ*	1.4283	0.86236	-1.5426	-1.8095	0.25
13335	*RXFP1*	0.73987	0.15419	-0.14639	-0.3828	0.25
13336	*ZNHIT3*	2.3491	-0.76165	-0.84069	-2.0989	0.25
13337	*TMED6*	2.1887	-0.5986	-0.84337	-2.2449	0.25
13338	*CHFR*	1.4599	0.36473	-1.0782	-1.9472	0.25
13339	*SPATA25*	1.0483	0.53134	-0.83411	-0.8528	0.25
13340	*SNRPD1*	0.31317	0.21588	0.2158	-1.6218	0.25
13341	*PTCD3*	3.1373	-1.031	-1.3619	-2.444	0.25
13342	*ZNF468*	1.8722	-0.29076	-0.83739	-1.2097	0.25
13343	*PDE4A*	2.22	-0.33174	-1.1451	-1.9455	0.25
13344	*RABGEF1*	2.387	-0.58495	-1.0589	-1.4234	0.25
13345	*RAB12*	1.3985	0.25633	-0.91296	-1.1421	0.25
13346	*RBM15*	1.0652	0.31825	-0.64164	-2.4151	0.25
13347	*OVOL2*	0.69701	0.32713	-0.28242	-1.2098	0.25
13348	*TRIM49D2*	0.96786	0.057442	-0.28393	-1.6788	0.25
13349	*STARD7*	2.8125	-0.74184	-1.3294	-2.5189	0.25
13350	*ABCB11*	1.4178	0.21618	-0.8936	-1.8679	0.25
13351	*CNTNAP4*	0.77909	0.15516	-0.19422	-0.82833	0.25
13352	*LMBR1L*	1.4782	0.21632	-0.95545	-2.2473	0.25
13353	*UTS2*	2.7028	-0.00819	-1.9558	-2.4785	0.25
13354	*TRIM69*	0.42524	0.3822	-0.06892	-0.61436	0.25
13355	*TIE1*	0.65117	0.4185	-0.33191	-1.0118	0.25
13356	*ZCCHC24*	0.84203	0.014586	-0.1201	-1.992	0.25
13357	*TFAM*	1.1842	0.46277	-0.91192	-1.5526	0.25
13358	*NFE2L2*	1.9881	-0.37417	-0.8789	-1.0321	0.25
13359	*UCN*	1.0749	0.46124	-0.80191	-2.0363	0.24
13360	*PITPNA*	1.2088	0.20909	-0.68383	-2.2461	0.24
13361	*LTV1*	2.5357	0.20215	-2.0042	-2.5354	0.24
13362	*RPS19BP1*	0.41025	0.34397	-0.02105	-2.1224	0.24
13363	*ZNF208*	1.5239	-0.37642	-0.41476	-1.1034	0.24
13364	*MOV10L1*	0.41247	0.19462	0.12522	-1.6316	0.24
13365	*FAM160B1*	1.6579	0.42215	-1.3478	-1.4519	0.24
13366	*C19orf35*	0.61036	0.58268	-0.46092	-2.0242	0.24
13367	*GPR62*	2.2375	-0.41127	-1.0942	-1.7683	0.24
13368	*CNTNAP5*	2.664	-0.50394	-1.4287	-2.2203	0.24
13369	*OR10H4*	1.1694	0.1957	-0.63436	-0.68259	0.24
13370	*ZNF549*	2.4161	0.16183	-1.8478	-2.1036	0.24
13371	*CR2*	1.0656	0.2336	-0.56947	-1.2766	0.24
13372	*AK8*	1.0265	-0.02226	-0.27453	-1.5212	0.24
13373	*LKAAEAR1*	3.4079	-1.0584	-1.6199	-2.3063	0.24
13374	*SETD1B*	1.5048	0.49602	-1.2715	-1.7192	0.24
13375	*C9orf69*	2.2823	-0.43478	-1.1184	-2.1723	0.24
13376	*ARL6IP5*	2.482	-0.73029	-1.023	-1.6278	0.24
13377	*IFNL3*	0.98458	0.72684	-0.98292	-1.4258	0.24
13378	*TTC25*	3.201	-0.58479	-1.8879	-1.9404	0.24
13379	*TMEM222*	3.5197	-1.3633	-1.4282	-1.5393	0.24

13380	*NUDT13*	1.9105	0.92337	-2.1068	-2.2879	0.24
13381	*C5orf42*	0.6021	0.33338	-0.20849	-0.97103	0.24
13382	*CYSRT1*	1.9376	-0.32611	-0.88495	-1.8112	0.24
13383	*SPATA24*	1.114	-0.02658	-0.36242	-2.2533	0.24
13384	*SHC2*	0.67368	0.085982	-0.0354	-0.74875	0.24
13385	*TPST2*	1.5913	-0.1875	-0.6799	-1.1388	0.24
13386	*ZDHHC9*	1.816	0.3001	-1.3946	-1.6939	0.24
13387	*CLLU1*	0.65403	0.19443	-0.12709	-0.16242	0.24
13388	*CD55*	1.5364	0.38114	-1.1962	-2.3826	0.24
13389	*CRISP1*	2.4311	0.027886	-1.739	-3.4957	0.24
13390	*GRHPR*	0.96157	0.47648	-0.71905	-1.9213	0.24
13391	*UCP2*	1.1802	0.026897	-0.48925	-0.90001	0.24
13392	*OTX2*	2.7243	-0.18167	-1.8253	-2.262	0.24
13393	*THEGL*	3.389	-1.146	-1.526	-1.5415	0.24
13394	*AZI2*	0.69364	0.20424	-0.18175	-0.2408	0.24
13395	*MYLK*	0.75321	0.23932	-0.27672	-1.589	0.24
13396	*SLC3A1*	2.7277	-0.82226	-1.1897	-2.1219	0.24
13397	*OR7G3*	0.75178	0.37143	-0.40824	-2.1014	0.24
13398	*PBK*	0.3326	0.26202	0.11968	-0.71896	0.24
13399	*CSN1S1*	1.1185	0.28685	-0.69155	-2.5198	0.24
13400	*WTH3DI*	3.8492	-1.2717	-1.8642	-2.0267	0.24
13401	*TCIRG1*	1.8657	-0.48086	-0.6716	-1.5898	0.24
13402	*ADGRG3*	1.0105	0.38053	-0.67812	-2.2748	0.24
13403	*C2orf74*	3.7128	-1.1383	-1.8636	-1.8938	0.24
13404	*POLR3F*	1.7991	-0.30192	-0.78639	-1.3814	0.24
13405	*FANCL*	1.6115	0.59114	-1.4919	-2.9308	0.24
13406	*ANKRD17*	1.4996	-0.30241	-0.48669	-1.7959	0.24
13407	*IQCB1*	0.56929	0.55315	-0.41207	-0.74875	0.24
13408	*FRS2*	0.74631	0.4075	-0.44353	-1.7034	0.24
13409	*FIGN*	1.0721	0.60896	-0.97149	-1.6456	0.24
13410	*STAC2*	0.33158	0.25162	0.12509	-0.03046	0.24
13411	*ZBTB4*	2.3523	-0.24856	-1.397	-1.4087	0.24
13412	*MAMDC2*	0.50708	0.40046	-0.20107	-1.1912	0.24
13413	*DNAJC28*	1.3496	-0.06253	-0.58143	-0.60113	0.24
13414	*BCL2L11*	2.9451	-0.78951	-1.4501	-1.9474	0.24
13415	*UPP1*	1.1851	-0.11968	-0.36066	-0.98239	0.23
13416	*SNW1*	2.5408	-0.87078	-0.96529	-1.1768	0.23
13417	*PRLR*	3.0378	-0.86506	-1.4682	-1.7383	0.23
13418	*OAZ3*	2.3221	0.53001	-2.1478	-2.5934	0.23
13419	*C11orf86*	1.5238	0.049686	-0.86923	-1.0178	0.23
13420	*SEC31B*	2.4049	-0.5409	-1.1605	-1.7072	0.23
13421	*EML6*	2.9174	-0.00317	-2.2108	-3.0959	0.23
13422	*TRUB1*	0.60091	0.14451	-0.04218	-0.65503	0.23
13423	*TBX2*	2.0545	0.44943	-1.8007	-3.0378	0.23
13424	*GLOD5*	0.70504	0.4209	-0.42352	-0.57054	0.23
13425	*TTLL5*	1.1759	0.10748	-0.58143	-1.2431	0.23

13426	*SKI*	1.0392	0.48824	-0.82573	-1.4509	0.23
13427	*SGOL2*	2.5866	-0.82789	-1.058	-1.8335	0.23
13428	*SGK1*	0.31766	0.21312	0.16903	-1.1738	0.23
13429	*MYO1E*	0.59299	0.34589	-0.2391	-0.97381	0.23
13430	*CDHR5*	0.75731	0.19282	-0.25047	-2.0146	0.23
13431	*ABHD4*	1.272	0.23519	-0.80847	-1.5552	0.23
13432	*WDR60*	2.8659	0.057397	-2.2254	-2.4174	0.23
13433	*AP2A2*	0.86619	0.003671	-0.17261	-1.2284	0.23
13434	*DCP2*	1.6363	-0.19587	-0.74459	-2.6805	0.23
13435	*INTS9*	0.72339	0.5894	-0.61698	-1.0944	0.23
13436	*IGF2BP2*	0.85062	0.64689	-0.80315	-1.0528	0.23
13437	*NDN*	0.47149	0.40964	-0.18718	-0.69345	0.23
13438	*STARD4*	3.09	-1.1733	-1.2235	-2.1517	0.23
13439	*SLC26A2*	0.63127	0.54286	-0.48104	-1.6832	0.23
13440	*POLG*	0.97812	0.12402	-0.41025	-0.58485	0.23
13441	*RAD52*	0.81849	0.053516	-0.18026	-2.4797	0.23
13442	*ADGRG1*	1.7875	0.99921	-2.095	-2.7931	0.23
13443	*EEF1B2*	1.2001	0.77063	-1.2792	-1.8202	0.23
13444	*ERO1L*	1.009	0.39196	-0.70973	-2.0384	0.23
13445	*RFT1*	1.8017	-0.37949	-0.73155	-1.9541	0.23
13446	*PCDHAC1*	1.1732	0.5811	-1.0645	-1.1872	0.23
13447	*ATP8B3*	3.7047	-0.60604	-2.4094	-2.7705	0.23
13448	*CASC1*	1.6426	0.30293	-1.2569	-1.7366	0.23
13449	*SRP54*	0.79458	0.29117	-0.39722	-1.3298	0.23
13450	*ZNF512*	0.84938	0.23402	-0.39636	-0.66364	0.23
13451	*GJA5*	1.5005	0.5661	-1.3818	-1.6699	0.23
13452	*TNFSF13B*	1.0209	0.081493	-0.41791	-0.83801	0.23
13453	*C8orf86*	0.45389	0.15078	0.07973	-0.31309	0.23
13454	*COL21A1*	1.1778	0.34238	-0.83627	-1.5302	0.23
13455	*FMO2*	0.43851	0.15379	0.09154	-0.06809	0.23
13456	*HOXD1*	0.39018	0.15845	0.13483	0.068131	0.23
13457	*TTC16*	1.2676	0.030628	-0.61491	-0.68389	0.23
13458	*PPP6R2*	0.70446	0.40077	-0.42358	-1.3409	0.23
13459	*ZNF527*	0.70317	0.64679	-0.67007	-2.8327	0.23
13460	*PLCD1*	1.3418	0.068529	-0.73066	-1.6128	0.23
13461	*MET*	1.3415	0.2179	-0.88055	-0.90281	0.23
13462	*LOXL3*	0.62779	0.52933	-0.47862	-1.8311	0.23
13463	*CRABP1*	2.3734	0.18155	-1.879	-2.0323	0.23
13464	*DFNB59*	1.4269	0.45179	-1.2032	-2.0999	0.23
13465	*ACSBG2*	0.38496	0.35826	-0.06787	-0.69345	0.23
13466	*WDR5B*	1.3123	0.33	-0.96703	-1.073	0.23
13467	*TOMM6*	1.9337	0.038871	-1.298	-1.6199	0.22
13468	*F8A1*	1.7083	0.14548	-1.1793	-1.3993	0.22
13469	*RNF103-CHMP3*	0.99463	0.81034	-1.1316	-2.2631	0.22
13470	*ANKRD50*	1.4545	-0.05706	-0.7241	-1.7004	0.22
13471	*CYYR1*	3.1232	-1.0529	-1.3973	-1.8792	0.22

13472	*GP9*	2.976	-0.63691	-1.6664	-1.8941	0.22
13473	*OR6C74*	1.0008	0.82277	-1.151	-1.3336	0.22
13474	*HECW2*	1.0594	0.49134	-0.87833	-0.97499	0.22
13475	*BDH2*	0.85877	0.057862	-0.24512	-1.4326	0.22
13476	*ZNF852*	1.0086	0.95631	-1.2935	-1.6469	0.22
13477	*OR7A17*	1.6249	-0.45803	-0.49565	-0.51525	0.22
13478	*DZIP1L*	0.59503	0.084189	-0.00816	-2.0016	0.22
13479	*MAD2L1*	0.94392	0.32443	-0.59743	-1.2151	0.22
13480	*CRYBA4*	1.9944	-0.61338	-0.71051	-1.5817	0.22
13481	*ATP5D*	1.9731	-0.44517	-0.85779	-1.6632	0.22
13482	*SFT2D3*	0.54569	0.35573	-0.23158	-2.1947	0.22
13483	*ABHD5*	0.66949	0.16939	-0.16962	-0.63511	0.22
13484	*TMEM151B*	1.5161	0.08533	-0.93307	-1.8562	0.22
13485	*CBX4*	3.8612	-1.1934	-1.9996	-2.9804	0.22
13486	*LRP5L*	1.2144	0.078277	-0.62476	-0.97952	0.22
13487	*CDC14A*	1.3016	0.27856	-0.9124	-3.6612	0.22
13488	*GRIN3B*	1.5634	-0.37358	-0.52269	-1.5707	0.22
13489	*SLITRK5*	0.74341	0.18925	-0.26653	-1.691	0.22
13490	*FKBP9*	2.4251	-0.47447	-1.2846	-2.1873	0.22
13491	*CC2D1B*	0.86364	0.71572	-0.9135	-1.9023	0.22
13492	*ADGRF2*	1.1249	0.056342	-0.51571	-1.9112	0.22
13493	*FNDC7*	0.93074	-0.05133	-0.21407	-1.8157	0.22
13494	*MAPK12*	2.3363	-0.05575	-1.6176	-1.6934	0.22
13495	*MAP2K2*	1.7695	0.40857	-1.5165	-1.6098	0.22
13496	*MAST4*	1.1737	-0.15049	-0.36195	-1.9934	0.22
13497	*MBD3L5*	1.3596	0.6852	-1.3852	-1.4442	0.22
13498	*PHYHIPL*	0.77685	0.39872	-0.51599	-0.67137	0.22
13499	*NR4A3*	0.87087	0.30181	-0.51321	-0.88666	0.22
13500	*KIN*	1.357	0.67524	-1.3729	-1.9758	0.22
13501	*OR6C2*	3.0566	-0.62017	-1.7782	-2.4015	0.22
13502	*LSM14A*	0.37751	0.23983	0.04075	-0.26537	0.22
13503	*C15orf65*	0.79818	0.30088	-0.443	-1.7585	0.22
13504	*AP2B1*	1.401	0.062155	-0.80713	-0.92086	0.22
13505	*STXBP4*	1.0903	0.79324	-1.2279	-1.2711	0.22
13506	*PEPD*	0.6366	0.39867	-0.37976	-0.90813	0.22
13507	*WDR83*	2.3484	-0.72095	-0.97237	-1.363	0.22
13508	*TMEM185B*	3.3601	-1.2736	-1.4316	-2.3811	0.22
13509	*MFNG*	3.2808	-0.93448	-1.6917	-1.772	0.22
13510	*PSG3*	1.2989	-0.02908	-0.61535	-1.3564	0.22
13511	*CLASP2*	0.79312	0.46439	-0.60315	-1.0564	0.22
13512	*MRPS18B*	1.3574	0.1059	-0.80909	-1.7221	0.22
13513	*FUT4*	2.1871	-0.2083	-1.3253	-2.1544	0.22
13514	*TM2D1*	0.87942	-0.10466	-0.12213	-1.4076	0.22
13515	*OR4D11*	0.83612	0.064371	-0.24794	-0.43906	0.22
13516	*PDLIM4*	4.3509	-1.7364	-1.962	-2.1444	0.22
13517	*DOK7*	0.59974	0.16736	-0.11527	-0.84042	0.22

13518	*FAM83A*	1.4926	0.80025	-1.6411	-2.2895	0.22
13519	*HPS4*	0.82454	0.58476	-0.75778	-1.696	0.22
13520	*CALM3*	1.2434	-0.0566	-0.53619	-1.0019	0.22
13521	*CRACR2A*	3.2131	-0.76405	-1.7994	-2.4885	0.22
13522	*ECSCR*	1.5214	-0.29647	-0.57616	-1.3605	0.22
13523	*NDUFB11*	3.859	-1.4811	-1.7292	-2.4405	0.22
13524	*CWC15*	0.42385	0.39086	-0.16624	-0.43628	0.22
13525	*CAMK1G*	2.9499	-0.76015	-1.5418	-2.4961	0.22
13526	*WNK3*	0.68969	0.48761	-0.53012	-1.2014	0.22
13527	*KDM4C*	1.0383	0.22723	-0.61836	-0.75369	0.22
13528	*ACSF2*	1.2142	-0.0083	-0.55909	-1.803	0.22
13529	*PIGW*	1.6938	-0.42373	-0.62423	-1.2353	0.22
13530	*MYOT*	1.1968	0.52179	-1.0733	-1.2339	0.22
13531	*THOC1*	0.68939	0.48856	-0.53312	-1.6885	0.21
13532	*MRRF*	0.79592	0.55436	-0.70558	-1.7322	0.21
13533	*ZNF646*	0.89379	0.59291	-0.84261	-0.87418	0.21
13534	*SNAPIN*	0.91708	0.083194	-0.35638	-1.0356	0.21
13535	*NCSTN*	1.0196	0.43794	-0.8146	-2.5414	0.21
13536	*ZNF644*	2.2859	-0.46228	-1.1815	-1.4932	0.21
13537	*NKX1-1*	1.3273	0.36346	-1.0493	-1.3612	0.21
13538	*ACSS2*	1.3275	-0.26639	-0.42005	-0.6937	0.21
13539	*DDA1*	1.7905	-0.54219	-0.60754	-1.1401	0.21
13540	*USH1C*	0.56882	0.17386	-0.10196	-1.1607	0.21
13541	*FGF21*	1.3035	0.28281	-0.94597	-1.4841	0.21
13542	*TRNP1*	1.4392	-0.01883	-0.78096	-1.0433	0.21
13543	*ZNF142*	0.90402	0.56025	-0.82488	-3.2116	0.21
13544	*FXN*	0.90917	-0.01505	-0.25612	-0.46733	0.21
13545	*MIEF1*	3.3512	-1.3098	-1.4039	-2.1334	0.21
13546	*NBN*	2.3998	-0.47882	-1.2837	-1.3903	0.21
13547	*ADORA2A*	0.89512	0.012516	-0.2704	-0.97237	0.21
13548	*C17orf53*	2.3638	0.015704	-1.7438	-2.6589	0.21
13549	*PAK7*	0.99928	-0.0918	-0.27178	-0.5171	0.21
13550	*DNMBP*	2.5786	-0.4187	-1.5243	-1.8227	0.21
13551	*SERTAD1*	3.4636	-0.44146	-2.3871	-4.0736	0.21
13552	*TTC30A*	1.1428	-0.01665	-0.49177	-0.59183	0.21
13553	*STMN3*	3.131	-1.1508	-1.346	-1.7947	0.21
13554	*POLR2G*	1.7485	-0.33114	-0.7835	-1.1247	0.21
13555	*FNBP1*	1.1508	-0.02433	-0.49275	-1.0863	0.21
13556	*OR8K5*	1.7575	-0.04249	-1.0823	-1.6841	0.21
13557	*FTL*	0.49008	0.41457	-0.27226	-0.64497	0.21
13558	*EIF4E1B*	1.9999	-0.43422	-0.93343	-1.3173	0.21
13559	*CADM2*	0.66185	0.27562	-0.30611	-0.99079	0.21
13560	*FAM161B*	1.3098	-0.05851	-0.62017	-0.84801	0.21
13561	*CBWD2*	2.1736	0.20646	-1.7495	-1.9938	0.21
13562	*BBS4*	0.95253	0.62527	-0.94777	-1.8572	0.21
13563	*C9orf3*	1.2081	0.13115	-0.70954	-1.1422	0.21

13564	*MDC1*	1.0339	0.40146	-0.80577	-0.91165	0.21
13565	*ELN*	3.5805	-1.3878	-1.5635	-1.8212	0.21
13566	*C18orf25*	0.92554	0.019479	-0.31596	-1.2335	0.21
13567	*C2CD4A*	0.9328	0.3488	-0.65345	-1.2803	0.21
13568	*LINC01272*	1.4104	0.50023	-1.2837	-3.1211	0.21
13569	*OR4D10*	0.50722	0.37229	-0.25317	-1.0109	0.21
13570	*EBI3*	2.5283	0.026387	-1.9284	-2.1125	0.21
13571	*CWC27*	3.8524	-1.4597	-1.7675	-1.7874	0.21
13572	*AGPAT6*	0.7106	0.10415	-0.19004	-1.4913	0.21
13573	*USP17L10*	2.0848	-0.40377	-1.0564	-2.6491	0.21
13574	*DEFB4B*	0.88746	0.2041	-0.46698	-0.68153	0.21
13575	*CDC6*	1.1494	0.44659	-0.97161	-1.6724	0.21
13576	*NDUFC2*	3.7097	0.11284	-3.1998	-3.3093	0.21
13577	*GLT8D2*	0.74036	0.45712	-0.5765	-1.4684	0.21
13578	*ANKZF1*	0.98159	0.017548	-0.37856	-0.51204	0.21
13579	*MUC16*	2.8403	-1.0175	-1.2027	-1.3926	0.21
13580	*C10orf113*	0.52765	0.25314	-0.16097	-0.35571	0.21
13581	*CXCR5*	0.52217	0.39939	-0.30208	-1.1487	0.21
13582	*HRK*	0.61186	0.4485	-0.44173	-1.2113	0.21
13583	*MUSTN1*	0.64585	0.4169	-0.4457	-1.9968	0.21
13584	*MYH7B*	1.2407	-0.24142	-0.38385	-1.9953	0.21
13585	*RASSF5*	2.3366	-0.27055	-1.4509	-1.5149	0.21
13586	*SKA2*	1.9127	-0.48688	-0.81111	-1.1686	0.20
13587	*NET1*	1.6112	0.30668	-1.3032	-1.3092	0.20
13588	*KRT2*	2.2837	-0.43068	-1.2393	-2.312	0.20
13589	*SBSN*	0.48577	0.14654	-0.01899	-0.89474	0.20
13590	*CHD8*	2.3559	-0.26717	-1.4762	-3.1008	0.20
13591	*MZT1*	2.4533	-0.79663	-1.045	-2.7736	0.20
13592	*CA10*	0.88913	0.10642	-0.38503	-1.6989	0.20
13593	*ICAM1*	1.093	0.086989	-0.56972	-0.98709	0.20
13594	*SUCLA2*	2.5014	0.11095	-2.003	-2.0146	0.20
13595	*SLC8B1*	0.45098	0.28923	-0.13111	-0.88877	0.20
13596	*EXO1*	1.4014	0.13139	-0.92375	-1.8908	0.20
13597	*HIST3H3*	4.1398	-1.7164	-1.8157	-2.781	0.20
13598	*AMACR*	1.2081	1.0064	-1.6069	-1.822	0.20
13599	*ULK2*	1.133	-0.00831	-0.51752	-0.62514	0.20
13600	*KRT36*	1.8874	0.16062	-1.4409	-1.7533	0.20
13601	*KCNQ2*	1.7203	0.024455	-1.1385	-1.5263	0.20
13602	*FMR1*	3.6373	-0.81522	-2.2165	-2.6895	0.20
13603	*CDK4*	1.8849	-0.18547	-1.0947	-1.4135	0.20
13604	*ATL2*	0.74318	-0.04339	-0.09515	-1.4747	0.20
13605	*SELE*	1.1395	-0.10051	-0.43475	-1.082	0.20
13606	*KNSTRN*	1.0233	0.40928	-0.82921	-2.1893	0.20
13607	*GABRA6*	1.3095	-0.33548	-0.37073	-1.5781	0.20
13608	*NRXN1*	2.8611	-0.58633	-1.6731	-2.3857	0.20
13609	*TBC1D2B*	0.46086	0.30631	-0.16574	-0.60655	0.20

13610	*CD72*	2.3076	-0.54039	-1.1659	-1.2071	0.20
13611	*MARCH2*	2.4854	0.06609	-1.9511	-2.6343	0.20
13612	*SMAD1*	1.9849	-0.40245	-0.98241	-1.882	0.20
13613	*TRAIP*	3.2773	-1.06	-1.6174	-1.7219	0.20
13614	*CT45A3*	1.8572	0.018995	-1.277	-2.2456	0.20
13615	*STAR*	1.6609	0.31027	-1.3728	-1.8107	0.20
13616	*RAC2*	0.8996	-0.06246	-0.23977	-1.0359	0.20
13617	*OR52A5*	0.42802	0.32263	-0.15339	-0.60113	0.20
13618	*CCDC175*	1.9444	-0.48317	-0.86403	-0.98842	0.20
13619	*GSTCD*	2.8497	-1.0293	-1.2234	-1.4557	0.20
13620	*ZNF638*	1.3327	0.53082	-1.2666	-1.5212	0.20
13621	*MAP3K5*	1.5888	0.00294	-0.99563	-2.6805	0.20
13622	*GH1*	2.6191	-0.97054	-1.0565	-1.5797	0.20
13623	*CLEC14A*	0.30938	0.281	0.00168	-1.316	0.20
13624	*ATP5E*	0.62521	0.1564	-0.19007	-1.7777	0.20
13625	*NR3C1*	0.91268	0.24704	-0.56825	-0.76389	0.20
13626	*CCDC85C*	1.7307	0.14882	-1.2884	-1.7493	0.20
13627	*SIPA1L2*	0.82008	0.421	-0.65053	-1.3363	0.20
13628	*BCL6*	1.1424	-0.26857	-0.28348	-0.91813	0.20
13629	*HAUS2*	2.753	0.62365	-2.7863	-3.582	0.20
13630	*VSTM1*	0.77824	0.59784	-0.78672	-1.7507	0.20
13631	*PAH*	3.3595	-1.2934	-1.479	-1.9446	0.20
13632	*BRF2*	1.1069	-0.23368	-0.28742	-2.3857	0.20
13633	*C1orf87*	1.4489	0.31774	-1.1817	-2.1155	0.19
13634	*NDUFAF3*	1.3651	-0.37125	-0.40933	-2.641	0.19
13635	*RAB11FIP3*	1.8972	-0.01574	-1.2975	-1.5741	0.19
13636	*PDE8A*	0.85257	0.50506	-0.77404	-1.1016	0.19
13637	*KRTAP27-1*	0.89867	-0.08887	-0.2263	-1.3148	0.19
13638	*EIF6*	1.288	-0.14836	-0.55976	-0.66598	0.19
13639	*FAM25G*	1.1017	0.31766	-0.84069	-2.0171	0.19
13640	*F12*	2.1678	-0.67837	-0.91148	-1.0643	0.19
13641	*RNMTL1*	0.4629	0.26733	-0.15339	-1.154	0.19
13642	*EARS2*	0.66068	0.48144	-0.56803	-1.1834	0.19
13643	*HEATR6*	1.821	-0.59572	-0.65169	-1.7658	0.19
13644	*HSPE1-MOB4*	0.5253	0.046507	0.00169	-1.2185	0.19
13645	*OR7A10*	0.32228	0.23479	0.01638	-0.02922	0.19
13646	*FMR1NB*	1.2483	-0.11118	-0.56405	-1.5954	0.19
13647	*VAX2*	1.0777	0.60979	-1.1152	-1.2529	0.19
13648	*KDR*	2.2438	-0.14536	-1.5264	-2.2078	0.19
13649	*UBXN7*	2.541	-0.98015	-0.98882	-1.4681	0.19
13650	*C9orf142*	3.1382	-1.0945	-1.4717	-2.7277	0.19
13651	*ZNF808*	2.299	-0.38361	-1.344	-1.7072	0.19
13652	*ARL16*	1.3531	0.57419	-1.3561	-1.8207	0.19
13653	*SPAG17*	1.6244	-0.06764	-0.98569	-1.2036	0.19
13654	*HSD17B13*	1.9952	-0.56586	-0.85983	-1.162	0.19
13655	*CCDC182*	1.9518	-0.52739	-0.85848	-1.2304	0.19

13656	*C1orf159*	2.7623	-0.88666	-1.3098	-1.9075	0.19
13657	*ASNS*	1.6276	-0.30993	-0.75304	-1.9511	0.19
13658	*STK32C*	0.7425	-0.0717	-0.10619	-1.4572	0.19
13659	*HMGCR*	2.1881	-0.01451	-1.61	-2.042	0.19
13660	*CEACAM19*	1.576	-0.30927	-0.70361	-0.93555	0.19
13661	*VAMP3*	3.5867	-1.4028	-1.6208	-1.7188	0.19
13662	*GLRB*	0.68717	0.018621	-0.14403	-0.26206	0.19
13663	*SCML1*	0.65853	0.25544	-0.35232	-0.49247	0.19
13664	*ZNF362*	0.2833	0.21501	0.06326	-1.0317	0.19
13665	*PNLIPRP2*	1.0985	0.33158	-0.86889	-0.86903	0.19
13666	*MUCL1*	0.49303	0.19242	-0.12495	-1.9434	0.19
13667	*LIPI*	2.3786	-0.58289	-1.2357	-1.4852	0.19
13668	*HNRNPR*	2.1443	0.50344	-2.088	-2.2528	0.19
13669	*NAE1*	2.4969	-0.51377	-1.4234	-1.8478	0.19
13670	*TGFBR3*	0.76271	0.20829	-0.41266	-1.6318	0.19
13671	*PPFIA1*	0.72624	0.45591	-0.62423	-2.6201	0.19
13672	*ST6GALNAC5*	2.8836	-1.0335	-1.2927	-1.8645	0.19
13673	*JTB*	1.9931	-0.62514	-0.81058	-2.0032	0.19
13674	*FAM111A*	0.58992	0.21702	-0.25291	-1.6913	0.18
13675	*FRYL*	1.1603	0.53997	-1.1466	-1.8651	0.18
13676	*CASP14*	1.0399	-0.08187	-0.40519	-0.61421	0.18
13677	*ADAMTS13*	0.71578	0.007553	-0.17073	-0.95233	0.18
13678	*SPHK2*	0.6246	0.21893	-0.29171	-0.90412	0.18
13679	*HIRA*	0.66269	0.47207	-0.5832	-1.6179	0.18
13680	*SFRP5*	1.5277	-0.28384	-0.69237	-0.90028	0.18
13681	*IL12RB1*	2.13	-0.46738	-1.1131	-1.8574	0.18
13682	*PRPF40A*	1.949	-0.56681	-0.83277	-1.3148	0.18
13683	*RGS2*	1.2515	0.19827	-0.90104	-1.5115	0.18
13684	*PKD1L3*	0.79154	-0.06121	-0.18229	-1.8106	0.18
13685	*INADL*	0.904	0.1206	-0.47685	-0.98728	0.18
13686	*LOC100130705*	0.39417	0.28993	-0.13707	-2.7127	0.18
13687	*NHP2*	3.0909	-0.62093	-1.923	-2.0535	0.18
13688	*CEP250*	0.74157	0.25842	-0.45373	-0.87688	0.18
13689	*TRIM21*	1.9286	-0.6785	-0.70391	-1.7345	0.18
13690	*TNNI2*	1.5176	0.11609	-1.0878	-2.1105	0.18
13691	*CYP1A2*	0.26487	0.18081	0.10017	0.066827	0.18
13692	*SRMS*	0.43414	0.17032	-0.06045	-0.58752	0.18
13693	*UNK*	1.3908	0.56905	-1.4172	-2.8071	0.18
13694	*TAF13*	0.46736	0.16363	-0.08887	-0.23814	0.18
13695	*YTHDC2*	0.43844	0.2928	-0.18916	-1.2198	0.18
13696	*SNAI1*	1.4818	-0.07367	-0.86744	-1.3711	0.18
13697	*FBRSL1*	2.1351	-0.58661	-1.009	-1.1553	0.18
13698	*SEBOX*	0.75952	0.65856	-0.87881	-1.6395	0.18
13699	*POC1B*	0.72652	0.25626	-0.44353	-2.2692	0.18
13700	*TYSND1*	0.53535	0.16279	-0.15961	-0.37519	0.18
13701	*FCGR3B*	2.8071	-0.76529	-1.5043	-2.2945	0.18

13702	*ZNF32*	1.0575	0.054536	-0.57477	-1.5884	0.18
13703	*KIAA1598*	0.30878	0.29117	-0.06364	-0.17588	0.18
13704	*POC5*	1.5872	0.27765	-1.3294	-1.6796	0.18
13705	*DOCK11*	0.5967	0.27836	-0.33984	-1.3517	0.18
13706	*FXYD5*	0.60194	0.57147	-0.63826	-1.6306	0.18
13707	*TRIM55*	1.214	0.49883	-1.1803	-1.3437	0.18
13708	*RPSA*	1.4198	0.40844	-1.2958	-1.392	0.18
13709	*GMNC*	0.80424	0.15744	-0.43005	-1.7493	0.18
13710	*SLX4*	1.2549	0.078951	-0.8038	-1.463	0.18
13711	*NUBP2*	0.64894	-0.03634	-0.08307	-1.3232	0.18
13712	*OR52N1*	2.2623	-0.68003	-1.0533	-1.0693	0.18
13713	*CYP4B1*	1.1448	0.4513	-1.0672	-1.4507	0.18
13714	*ZBTB45*	3.285	-0.42841	-2.329	-2.4094	0.18
13715	*PPA2*	1.6179	-0.35546	-0.73577	-2.0452	0.18
13716	*CLDN24*	0.83318	0.077055	-0.3836	-0.42756	0.18
13717	*WBSCR22*	1.2985	-0.05308	-0.71936	-1.2608	0.18
13718	*JAG1*	1.9214	-0.03091	-1.3647	-1.5253	0.18
13719	*CCDC78*	2.8147	-0.58575	-1.7034	-3.2632	0.18
13720	*LGALSL*	1.0776	0.29569	-0.84841	-0.98568	0.17
13721	*DCAF4L2*	0.65435	0.0961	-0.22589	-0.68466	0.17
13722	*RAB27A*	2.0522	-0.18328	-1.345	-2.0466	0.17
13723	*PRR14L*	0.40747	0.17555	-0.05918	-1.1742	0.17
13724	*RBM14*	1.4914	0.5188	-1.4864	-1.8157	0.17
13725	*C20orf194*	1.7194	0.56966	-1.7655	-2.0197	0.17
13726	*PYGO2*	0.70188	0.03708	-0.21563	-1.0372	0.17
13727	*ANKRD30A*	2.5346	-0.52122	-1.4911	-2.3918	0.17
13728	*TSPY1*	0.81109	-0.12213	-0.168	-1.8275	0.17
13729	*NELFA*	0.63586	0.060232	-0.17517	-1.2249	0.17
13730	*SRGAP1*	0.75055	0.42694	-0.65752	-0.78097	0.17
13731	*HSPA12B*	1.8826	-0.32122	-1.042	-1.1108	0.17
13732	*CCR2*	2.0908	-0.13756	-1.4341	-2.5189	0.17
13733	*PACS1*	1.4181	-0.00824	-0.89167	-1.1991	0.17
13734	*HECA*	2.7146	-0.53058	-1.6663	-1.7766	0.17
13735	*TOR1A*	0.76142	0.47992	-0.72479	-1.2004	0.17
13736	*E2F5*	0.40145	0.08403	0.0303	-0.16685	0.17
13737	*PABPC1*	3.163	-1.1251	-1.5227	-1.668	0.17
13738	*TCTE3*	0.77639	0.63802	-0.89977	-1.7999	0.17
13739	*MCU*	1.1612	-0.02072	-0.62629	-1.7434	0.17
13740	*C4B*	0.58412	0.057252	-0.12795	-1.8707	0.17
13741	*PPID*	0.89512	0.33765	-0.72222	-0.92907	0.17
13742	*NRXN2*	0.8464	-0.15394	-0.18195	-0.90501	0.17
13743	*SVIL*	1.1927	0.7076	-1.3899	-2.033	0.17
13744	*HSFX1*	0.64437	0.12975	-0.2641	-1.0057	0.17
13745	*CEP68*	0.66324	0.004409	-0.15853	-0.21283	0.17
13746	*OR52W1*	1.2467	-0.2957	-0.44267	-0.53747	0.17
13747	*GALNTL6*	0.66502	0.073132	-0.22988	-0.50104	0.17

13748	*VANGL2*	2.5132	-0.83143	-1.1735	-1.219	0.17
13749	*PHF10*	0.47043	0.17495	-0.13754	-0.59521	0.17
13750	*LCA5L*	0.71435	0.36734	-0.57408	-1.7303	0.17
13751	*PTBP1*	1.2183	0.042235	-0.75293	-1.5598	0.17
13752	*PFDN6*	0.6799	0.53302	-0.70532	-1.6343	0.17
13753	*APTX*	0.8626	0.42556	-0.78255	-1.8578	0.17
13754	*CCDC166*	0.96676	-0.03798	-0.42369	-0.53799	0.17
13755	*DHX16*	1.6654	-0.19706	-0.96349	-2.0791	0.17
13756	*DGKI*	0.95311	0.30333	-0.75213	-1.1929	0.17
13757	*KRT38*	1.4172	-0.1693	-0.74459	-0.8147	0.17
13758	*HOXA10*	2.8928	-1.084	-1.3055	-2.2507	0.17
13759	*SLC13A3*	0.35016	0.10642	0.04624	-1.1734	0.17
13760	*HP1BP3*	0.62997	0.008845	-0.13622	-1.3098	0.17
13761	*ZNF174*	1.9841	-0.37837	-1.1039	-2.3949	0.17
13762	*GSTM2*	2.182	-0.54366	-1.1367	-1.53	0.17
13763	*RP9*	1.1009	0.15099	-0.75068	-0.77521	0.17
13764	*FAM47E*	2.2433	-0.76106	-0.98239	-0.99368	0.17
13765	*TBL1Y*	1.6021	0.041514	-1.1442	-1.5569	0.17
13766	*COPB1*	1.867	-0.5701	-0.79813	-1.9863	0.17
13767	*ERAL1*	2.8685	-0.83459	-1.5352	-1.5824	0.17
13768	*SASH1*	1.18	0.52356	-1.2059	-1.7065	0.17
13769	*CYP2B6*	0.85199	0.27104	-0.62613	-1.7474	0.17
13770	*PTPRD*	2.5806	-0.99508	-1.0889	-1.3287	0.17
13771	*SUN5*	0.65897	0.25204	-0.41486	-1.4703	0.17
13772	*MOCOS*	0.40421	0.075758	0.01511	-0.36575	0.17
13773	*RNASE7*	2.9097	-1.0863	-1.3288	-1.5375	0.16
13774	*ALDH8A1*	0.26558	0.12223	0.10565	-0.34077	0.16
13775	*C10orf12*	0.65175	0.50479	-0.66363	-1.4597	0.16
13776	*NR1D2*	1.1109	0.26696	-0.88503	-2.7336	0.16
13777	*ACAP2*	0.37802	0.066303	0.04816	-2.0074	0.16
13778	*SLCO4A1*	0.50712	0.42651	-0.44177	-1.0838	0.16
13779	*KATNB1*	1.0597	-0.1865	-0.38219	-1.6098	0.16
13780	*SYN1*	0.8539	-0.05048	-0.3129	-1.5425	0.16
13781	*GNPDA1*	1.3454	0.11456	-0.9703	-1.175	0.16
13782	*HIST1H3A*	1.0763	0.033945	-0.62065	-1.1287	0.16
13783	*PLEKHA8*	2.5296	-0.59045	-1.4498	-4.0338	0.16
13784	*IL37*	0.97498	-0.08474	-0.40136	-0.65368	0.16
13785	*GALR2*	1.027	-0.08922	-0.45012	-1.2004	0.16
13786	*BCS1L*	0.361	0.23712	-0.11083	-1.7658	0.16
13787	*LEPR*	1.2927	-0.34816	-0.45803	-1.0094	0.16
13788	*MGA*	3.6625	-1.3246	-1.8523	-2.1709	0.16
13789	*RGS20*	0.57604	0.1221	-0.21258	-1.5121	0.16
13790	*TUBB8*	3.3842	-0.02864	-2.8702	-3.114	0.16
13791	*SLC25A26*	0.96387	-0.07528	-0.40338	-0.59959	0.16
13792	*PEX3*	2.8318	-0.99079	-1.3565	-4.0005	0.16
13793	*EMB*	2.139	-0.29427	-1.3607	-1.4682	0.16

13794	*SMARCA1*	1.0552	0.59085	-1.1626	-1.4576	0.16
13795	*PHACTR2*	1.63	0.78082	-1.928	-2.1354	0.16
13796	*STX6*	0.75462	0.027653	-0.30003	-1.5297	0.16
13797	*ZNF843*	0.83233	-0.16607	-0.18502	-1.7797	0.16
13798	*DUSP27*	1.0165	0.28353	-0.81883	-0.9272	0.16
13799	*IKBIP*	0.28028	0.13507	0.06584	-0.66082	0.16
13800	*PPM1A*	2.5481	-0.52034	-1.547	-1.9269	0.16
13801	*TRANK1*	0.52555	0.1916	-0.23642	-1.5052	0.16
13802	*CDK18*	0.94744	0.93574	-1.4036	-1.7945	0.16
13803	*SAP18*	2.6261	-0.93407	-1.2135	-2.6188	0.16
13804	*EEF2*	1.1276	-0.15953	-0.49073	-1.3513	0.16
13805	*GPR152*	0.90196	-0.17588	-0.24877	-1.3295	0.16
13806	*GSG1L*	0.89923	0.42891	-0.85084	-1.5754	0.16
13807	*SLC16A7*	0.49564	0.23974	-0.25936	-1.3057	0.16
13808	*CPSF2*	2.1228	-0.80548	-0.84136	-2.1264	0.16
13809	*SMIM14*	1.0935	-0.09378	-0.52397	-1.8398	0.16
13810	*NR1H3*	0.88319	0.0898	-0.49727	-1.0993	0.16
13811	*GSDMA*	1.2163	-0.29305	-0.44787	-0.77963	0.16
13812	*ANAPC10*	1.7462	-0.61889	-0.65199	-0.77289	0.16
13813	*OR5W2*	0.51467	0.4365	-0.47587	-0.56274	0.16
13814	*FOCAD*	1.097	-0.19562	-0.42631	-0.70944	0.16
13815	*GALT*	0.76877	-0.0645	-0.22921	-0.62476	0.16
13816	*GATSL3*	1.7436	1.2554	-2.5243	-2.716	0.16
13817	*AIPL1*	0.4161	0.38146	-0.32296	-0.59001	0.16
13818	*SELP*	1.7146	-0.59738	-0.64313	-2.1205	0.16
13819	*PCGF2*	1.0605	0.30878	-0.89595	-1.4625	0.16
13820	*LNX2*	1.9705	-0.15394	-1.344	-1.4162	0.16
13821	*RPL39*	0.5536	0.040832	-0.12247	-3.1578	0.16
13822	*KRTCAP2*	1.6602	-0.46589	-0.7229	-2.0698	0.16
13823	*ABCB8*	3.1954	-1.219	-1.5052	-1.856	0.16
13824	*KDM4B*	2.8658	-0.3205	-2.075	-2.1187	0.16
13825	*GPR15*	2.768	-0.97792	-1.3198	-1.6266	0.16
13826	*TBX21*	1.0564	0.12345	-0.70976	-1.473	0.16
13827	*TMEM105*	1.4008	0.53727	-1.4681	-1.8471	0.16
13828	*MC3R*	0.56252	0.20143	-0.29422	-1.1696	0.16
13829	*VPS52*	0.48693	0.48324	-0.50053	-2.1466	0.16
13830	*SLC39A1*	2.0128	-0.25291	-1.2903	-1.525	0.16
13831	*CCM2*	0.821	0.58811	-0.94052	-1.5061	0.16
13832	*EDEM1*	1.5701	0.26685	-1.3694	-2.3036	0.16
13833	*TUBB4B*	1.3107	0.46124	-1.3053	-2.6289	0.16
13834	*SHCBP1L*	1.6737	0.049791	-1.2574	-1.6934	0.16
13835	*LOC100131303*	1.0134	0.9513	-1.4994	-2.6683	0.16
13836	*CSMD1*	0.65341	0.005565	-0.19404	-1.6125	0.15
13837	*IFT27*	1.701	-0.46979	-0.76635	-1.1065	0.15
13838	*OBP2A*	0.58143	0.54991	-0.66752	-1.4087	0.15
13839	*DYRK1B*	3.1024	-1.3183	-1.3203	-2.2108	0.15

13840	*CHCHD5*	0.75892	0.27246	-0.56769	-0.78725	0.15
13841	*SLC15A5*	0.60924	0.56505	-0.71093	-0.98239	0.15
13842	*LBH*	3.5545	-0.97648	-2.1151	-2.2232	0.15
13843	*SIKE1*	0.56182	0.24092	-0.33985	-0.63939	0.15
13844	*CDIPT*	1.4664	0.95155	-1.9554	-2.6593	0.15
13845	*IFT43*	0.48201	0.18951	-0.20906	-2.5707	0.15
13846	*ARPC4*	0.44646	0.19552	-0.17979	-1.1986	0.15
13847	*SLC35G2*	0.93085	-0.03695	-0.43201	-0.46738	0.15
13848	*SLC9A6*	0.58664	-0.05066	-0.07435	-2.3615	0.15
13849	*CEP83*	1.1883	0.04123	-0.7679	-1.4061	0.15
13850	*FBXW11*	0.62181	0.11287	-0.27453	-1.4234	0.15
13851	*CCDC102A*	1.5636	0.78697	-1.8906	-2.2021	0.15
13852	*ACOT1*	1.4921	-0.46761	-0.56501	-0.89887	0.15
13853	*TRIM67*	2.2325	-0.21974	-1.5538	-2.1919	0.15
13854	*AFF4*	1.569	-0.40692	-0.70327	-1.4141	0.15
13855	*METTL13*	1.9775	-0.67684	-0.84248	-0.94576	0.15
13856	*ZNF93*	0.53823	0.22862	-0.30878	-1.3878	0.15
13857	*APITD1-CORT*	0.93984	-0.20512	-0.27675	-1.1511	0.15
13858	*CLEC11A*	1.0042	-0.07909	-0.46733	-2.3178	0.15
13859	*SPEF1*	1.6975	-0.31062	-0.92911	-1.4258	0.15
13860	*ARL17A*	1.6599	-0.30208	-0.90084	-1.8264	0.15
13861	*FCAR*	0.67742	0.079385	-0.30038	-1.1687	0.15
13862	*DEXI*	1.2889	-0.24005	-0.5925	-0.74912	0.15
13863	*HOMER2*	1.5905	-0.01358	-1.1212	-1.1533	0.15
13864	*MTFR1L*	0.51892	0.27719	-0.34111	-0.42062	0.15
13865	*TMEM87A*	1.0207	0.025575	-0.59163	-1.1587	0.15
13866	*CXorf40A*	1.1417	-0.1851	-0.50283	-0.63125	0.15
13867	*OR2T35*	0.63315	0.15623	-0.33568	-2.0804	0.15
13868	*NNT*	3.2727	-0.95645	-1.8636	-1.9953	0.15
13869	*MGAT1*	0.38916	0.17891	-0.11543	-1.9282	0.15
13870	*CD151*	1.3249	-0.39971	-0.47376	-2.3349	0.15
13871	*DEFB119*	2.5572	-1.0283	-1.0775	-1.566	0.15
13872	*TMEM200C*	1.2835	0.34151	-1.1737	-1.9686	0.15
13873	*COX6B1*	1.7527	0.17015	-1.4717	-2.1984	0.15
13874	*CORO1C*	1.3871	-0.45339	-0.48336	-0.77227	0.15
13875	*UTP23*	0.32177	0.29429	-0.16624	-3.2662	0.15
13876	*COX5A*	0.59682	0.050482	-0.19778	-1.5243	0.15
13877	*LIPF*	1.129	-0.20506	-0.47496	-1.7364	0.15
13878	*EEF2KMT*	0.56528	0.2374	-0.35441	-0.66274	0.15
13879	*CENPB*	2.3828	-0.44353	-1.4913	-1.72	0.15
13880	*BPNT1*	2.1477	-0.35128	-1.3488	-1.7221	0.15
13881	*CCNDBP1*	0.97221	0.16922	-0.69418	-2.3936	0.15
13882	*ZC3H12C*	1.2068	-0.30878	-0.45119	-1.4034	0.15
13883	*SMAD6*	0.55507	0.44466	-0.55296	-2.2044	0.15
13884	*IGSF8*	1.3451	0.080851	-0.9797	-1.6428	0.15
13885	*TRIM54*	1.3377	0.33888	-1.2306	-1.5424	0.15

13886	*BET1L*	0.7044	0.54846	-0.80743	-1.5099	0.15
13887	*ATXN2L*	1.8804	-0.42936	-1.0058	-1.5073	0.15
13888	*FBXL13*	0.70352	0.006056	-0.26575	-1.3694	0.15
13889	*HNRNPC*	0.44554	0.27562	-0.27814	-1.2071	0.15
13890	*OR4L1*	0.20228	0.14084	0.09972	-0.13361	0.15
13891	*SLITRK3*	3.0361	-0.86556	-1.728	-2.3293	0.15
13892	*HORMAD2*	0.28841	0.16681	-0.01298	-1.5282	0.15
13893	*CASP2*	1.0713	-0.15051	-0.47857	-2.0407	0.15
13894	*RBX1*	1.4857	-0.3205	-0.72348	-1.2572	0.15
13895	*DEFB110*	0.61104	0.30864	-0.47854	-1.8212	0.15
13896	*SUFU*	1.2461	-0.02092	-0.78588	-1.3078	0.15
13897	*ZNF813*	1.6635	-0.0083	-1.2159	-1.683	0.15
13898	*SP100*	0.74969	0.28567	-0.59763	-2.1713	0.15
13899	*DIAPH1*	0.5	0.12678	-0.18937	-0.79202	0.15
13900	*DDX3X*	0.77227	0.026519	-0.36177	-0.44353	0.15
13901	*SLC11A2*	1.571	-0.45531	-0.6794	-2.4067	0.15
13902	*TAS2R13*	2.3604	-0.25984	-1.6648	-1.8038	0.15
13903	*NBPF10*	1.3119	0.34565	-1.2234	-1.4955	0.14
13904	*FLG*	0.28793	0.10767	0.03809	-1.0304	0.14
13905	*TMIE*	2.0611	-0.72896	-0.90014	-0.93837	0.14
13906	*TRIM50*	1.2122	-0.36582	-0.41454	-0.91427	0.14
13907	*POLR2B*	2.252	-0.57408	-1.2464	-1.6469	0.14
13908	*MAGEC1*	1.2846	-0.33893	-0.51494	-1.6026	0.14
13909	*PRDM1*	1.1426	-0.25915	-0.45302	-1.63	0.14
13910	*EPHA1*	1.0053	-0.19925	-0.37641	-0.77697	0.14
13911	*CLSTN3*	2.9498	-1.0024	-1.5182	-2.017	0.14
13912	*ZNF580*	0.79143	0.64117	-1.0044	-1.19	0.14
13913	*BVES*	0.74392	0.21633	-0.53264	-0.9341	0.14
13914	*USP6*	1.4409	-0.30312	-0.71032	-1.6597	0.14
13915	*SGPL1*	0.55629	0.31054	-0.43998	-1.6709	0.14
13916	*MCM5*	0.3392	0.31701	-0.22972	-0.62223	0.14
13917	*SYF2*	0.88227	0.38268	-0.83883	-1.9558	0.14
13918	*FCHO1*	1.1713	0.56444	-1.3104	-2.0629	0.14
13919	*C20orf141*	0.36369	0.17121	-0.10981	-0.40863	0.14
13920	*MKRN3*	0.766	0.059575	-0.40104	-2.2631	0.14
13921	*TUBGCP6*	1.2539	-0.04886	-0.78229	-1.039	0.14
13922	*DSN1*	3.5811	-1.2743	-1.8851	-2.3871	0.14
13923	*MFSD5*	1.9131	0.089165	-1.5809	-1.9237	0.14
13924	*SHOX*	0.78036	-0.02394	-0.33524	-1.2896	0.14
13925	*ASB11*	1.6128	0.011742	-1.2036	-1.8238	0.14
13926	*RCE1*	1.7378	-0.54264	-0.77428	-1.6575	0.14
13927	*MROH2A*	0.78918	-0.05413	-0.31535	-1.8707	0.14
13928	*MRPL57*	4.1723	-1.244	-2.5103	-2.9525	0.14
13929	*MTMR9*	2.5543	-0.51664	-1.6199	-1.7697	0.14
13930	*HSD11B1*	1.6042	0.17307	-1.3599	-2.2621	0.14
13931	*TPM2*	0.74854	-0.13276	-0.19915	-1.5436	0.14

13932	*PSPC1*	0.41169	0.25176	-0.24697	-0.64851	0.14
13933	*UQCRC2*	2.5295	-0.78718	-1.3273	-1.4917	0.14
13934	*AQP5*	0.93573	0.40848	-0.92926	-2.8229	0.14
13935	*TRAF7*	1.0304	0.57356	-1.1891	-2.071	0.14
13936	*SYT13*	1.2711	0.94044	-1.7971	-2.7728	0.14
13937	*MS4A4A*	1.5743	-0.47211	-0.6878	-1.8751	0.14
13938	*LPIN3*	1.8631	-0.16995	-1.2788	-1.4572	0.14
13939	*CASP6*	3.5202	-1.4545	-1.6524	-1.9199	0.14
13940	*SETD4*	0.66956	0.074609	-0.3316	-1.2797	0.14
13941	*APBB1*	1.9227	-0.31587	-1.1943	-1.4448	0.14
13942	*ZSCAN26*	2.1769	0.11889	-1.8834	-2.262	0.14
13943	*SPATA18*	1.0238	-0.13185	-0.47964	-1.0164	0.14
13944	*NPHP3*	3.6465	-1.5568	-1.6776	-2.0613	0.14
13945	*CACNB3*	0.96224	0.67671	-1.227	-1.2485	0.14
13946	*TAAR8*	0.7183	0.2892	-0.596	-1.6964	0.14
13947	*LRRC2*	0.7106	0.10206	-0.40118	-1.7974	0.14
13948	*CCL8*	0.42665	0.12445	-0.13962	-2.2046	0.14
13949	*AMD1*	1.2114	-0.09709	-0.70289	-1.9772	0.14
13950	*VPS41*	1.8053	-0.68934	-0.70532	-1.1912	0.14
13951	*MSMO1*	0.98504	0.24839	-0.82311	-2.3776	0.14
13952	*FAAH*	0.60626	0.071134	-0.26717	-0.44353	0.14
13953	*COA6*	0.54925	0.25407	-0.39318	-0.96673	0.14
13954	*TMEM40*	1.6796	-0.36086	-0.90922	-1.5722	0.14
13955	*PCDHGA6*	0.79847	-0.03097	-0.35896	-0.47632	0.14
13956	*BTK*	1.1966	-0.33584	-0.45236	-1.8046	0.14
13957	*SH3GL1*	1.1082	-0.15226	-0.54797	-0.62005	0.14
13958	*CCDC97*	2.4796	-0.98158	-1.0904	-1.7001	0.14
13959	*DYNC1H1*	1.2678	0.22314	-1.0838	-1.9725	0.14
13960	*GPBP1L1*	1.314	0.054282	-0.96161	-2.6837	0.14
13961	*LARS*	0.30293	0.058283	0.04435	-0.29801	0.14
13962	*TP53RK*	0.84201	0.40361	-0.84026	-2.2948	0.14
13963	*PTP4A3*	0.95206	0.24853	-0.79548	-1.6848	0.14
13964	*SNAPC4*	2.3007	-0.38355	-1.5132	-2.2653	0.13
13965	*WEE1*	2.5819	-0.09527	-2.0837	-2.7068	0.13
13966	*RANBP2*	1.5781	0.27752	-1.4527	-1.7247	0.13
13967	*MPZL1*	1.4742	0.022664	-1.0951	-1.534	0.13
13968	*LUZP1*	0.38181	0.19646	-0.17658	-0.8345	0.13
13969	*PPIE*	0.5313	0.20182	-0.33213	-0.84075	0.13
13970	*GTF2F2*	1.4908	-0.44353	-0.64683	-1.3926	0.13
13971	*PHOSPHO1*	1.2741	0.44327	-1.3173	-1.6935	0.13
13972	*CLCN6*	0.67455	0.043375	-0.31818	-0.62065	0.13
13973	*USPL1*	0.49422	-0.04717	-0.04799	-1.9622	0.13
13974	*RNASE3*	1.2404	-0.14639	-0.69502	-1.1629	0.13
13975	*RAB3GAP1*	1.7477	-0.63152	-0.71772	-1.4696	0.13
13976	*DOPEY2*	0.66957	0.20677	-0.47818	-1.3245	0.13
13977	*EXOC4*	0.85095	0.19463	-0.64851	-2.312	0.13

13978	*SLC18A3*	0.37811	0.22792	-0.20933	-1.7822	0.13
13979	*RQCD1*	2.5986	-0.88584	-1.3162	-2.4197	0.13
13980	*TXN*	0.76604	0.29116	-0.66164	-1.1479	0.13
13981	*SLC39A13*	0.53689	-0.05346	-0.0881	-2.1282	0.13
13982	*GCG*	0.91432	0.34848	-0.86793	-0.9653	0.13
13983	*PERP*	1.0046	0.53955	-1.1511	-1.7177	0.13
13984	*IL22RA2*	1.8036	-0.18461	-1.227	-2.4386	0.13
13985	*SPON2*	0.95169	0.35042	-0.91045	-0.97103	0.13
13986	*UBE2J1*	0.451	0.098411	-0.15783	-1.5744	0.13
13987	*TTC9B*	1.2333	0.32175	-1.1637	-1.9054	0.13
13988	*ARSA*	0.46998	0.27047	-0.34987	-1.1858	0.13
13989	*HLA-F*	1.0288	0.1059	-0.7443	-1.3053	0.13
13990	*KLF14*	0.47249	0.39358	-0.47671	-0.84836	0.13
13991	*RPH3A*	0.6696	0.44688	-0.72764	-1.4533	0.13
13992	*EXOG*	2.1275	-0.84446	-0.89424	-1.2639	0.13
13993	*SEMA4D*	1.7722	-0.0172	-1.3666	-1.398	0.13
13994	*SCRIB*	0.90342	0.5013	-1.0165	-2.0305	0.13
13995	*CALHM3*	0.64447	0.21027	-0.4674	-2.1616	0.13
13996	*DIAPH2*	3.2587	-1.1229	-1.7493	-3.1211	0.13
13997	*ZNF664*	0.98891	0.24364	-0.84624	-0.95224	0.13
13998	*CPNE9*	2.2377	-0.8897	-0.96196	-1.5876	0.13
13999	*APMAP*	1.3594	0.21413	-1.1897	-1.796	0.13
14000	*SNX24*	2.4112	-0.52902	-1.4996	-2.3755	0.13
14001	*CAMKK2*	1.1578	0.55802	-1.3336	-1.5485	0.13
14002	*LIN9*	1.8098	-0.60659	-0.82128	-2.6003	0.13
14003	*TSR3*	3.0275	-0.85236	-1.7945	-2.412	0.13
14004	*FGF12*	1.2302	0.48465	-1.3347	-1.4527	0.13
14005	*ZNF615*	1.0539	0.38184	-1.0564	-1.6447	0.13
14006	*OR5R1*	0.2658	0.080936	0.0321	-0.89957	0.13
14007	*DEDD*	0.94458	-0.27313	-0.29291	-1.1135	0.13
14008	*CXCL2*	0.66764	0.48128	-0.77056	-1.0333	0.13
14009	*CCDC108*	1.9712	-0.56581	-1.0271	-1.3066	0.13
14010	*TCF23*	0.52459	0.20031	-0.34688	-0.45361	0.13
14011	*TOMM7*	0.93971	0.25991	-0.82172	-1.0837	0.13
14012	*CDH16*	0.8257	-0.0061	-0.44353	-1.3325	0.13
14013	*GCNT3*	0.828	0.80452	-1.2568	-1.9013	0.13
14014	*CGB*	0.68689	0.028777	-0.34077	-1.1795	0.12
14015	*ARID5A*	0.56782	0.31649	-0.51017	-1.63	0.12
14016	*BAG2*	2.0178	0.23469	-1.879	-2.3615	0.12
14017	*JAGN1*	1.793	0.32243	-1.7434	-1.8345	0.12
14018	*SCNM1*	0.59621	0.31004	-0.53455	-0.87706	0.12
14019	*CCDC81*	4.1652	-0.70442	-3.0911	-3.5736	0.12
14020	*ABCC2*	1.1143	0.52174	-1.2676	-2.1264	0.12
14021	*RLTPR*	3.4004	-1.1132	-1.9191	-2.7568	0.12
14022	*TRPM1*	1.0767	-0.29146	-0.41748	-2.1888	0.12
14023	*UGT1A6*	0.34533	0.32554	-0.3032	-0.73069	0.12

14024	*MRPL47*	1.0084	0.36247	-1.0057	-1.334	0.12
14025	*ZNF425*	0.41263	0.20594	-0.25375	-0.94252	0.12
14026	*RHOBTB1*	0.42234	0.21512	-0.27303	-1.032	0.12
14027	*PPHLN1*	1.2703	-0.06844	-0.83791	-0.93726	0.12
14028	*FGF16*	1.5623	0.012433	-1.2109	-3.1991	0.12
14029	*PARN*	0.24435	0.2126	-0.09476	-0.51514	0.12
14030	*CCBE1*	0.72697	0.078477	-0.44374	-1.2265	0.12
14031	*SS18L1*	0.83693	0.052712	-0.52916	-0.85536	0.12
14032	*PAX9*	0.33072	0.13367	-0.10392	-1.9885	0.12
14033	*TIGIT*	1.9873	-0.78651	-0.84042	-0.8756	0.12
14034	*ST5*	1.7296	-0.36917	-1.0013	-3.3677	0.12
14035	*TOPAZ1*	1.2194	0.97055	-1.831	-2.1046	0.12
14036	*TRIM25*	1.4636	0.65341	-1.7591	-1.7908	0.12
14037	*KRAS*	1.2671	-0.13638	-0.77397	-0.89238	0.12
14038	*ARFGAP1*	1.3434	-0.17459	-0.81306	-1.1972	0.12
14039	*EXOSC8*	2.5973	-0.84693	-1.3951	-1.4168	0.12
14040	*TPR*	0.49772	0.28998	-0.43292	-0.69397	0.12
14041	*CNTN6*	2.2397	-0.75593	-1.1303	-1.6988	0.12
14042	*XKR4*	2.3855	-0.99217	-1.0406	-1.5765	0.12
14043	*FBXL15*	0.73723	0.09021	-0.47485	-0.97784	0.12
14044	*HSPA12A*	1.3313	-0.15679	-0.82281	-1.8995	0.12
14045	*SETD3*	2.5441	-0.54028	-1.6525	-1.8751	0.12
14046	*AK6*	0.71104	0.30181	-0.66164	-0.71041	0.12
14047	*CCDC159*	0.66961	-0.15194	-0.1672	-0.39851	0.12
14048	*C8orf37*	0.8136	-0.00831	-0.45563	-1.0293	0.12
14049	*C15orf43*	1.898	-0.38676	-1.1619	-1.9305	0.12
14050	*SLC7A10*	2.9897	-1.0926	-1.548	-1.6449	0.12
14051	*FOXI2*	0.53045	0.38056	-0.56376	-1.5502	0.12
14052	*PRR22*	1.39	-0.30268	-0.7404	-0.78923	0.12
14053	*TRAF3IP1*	0.86784	-0.07743	-0.44353	-0.6602	0.12
14054	*HIST1H3I*	0.91198	0.33366	-0.89892	-3.3546	0.12
14055	*CSNK1A1*	4.3726	-1.6707	-2.3557	-2.712	0.12
14056	*HSCB*	0.99807	-0.22569	-0.42697	-0.94341	0.12
14057	*OR10Z1*	0.8457	0.03717	-0.53799	-0.78363	0.11
14058	*AMY1C*	2.6048	-0.53619	-1.7241	-2.2277	0.11
14059	*HEATR1*	0.97384	-0.14419	-0.48708	-0.72666	0.11
14060	*XCR1*	1.9484	-0.57042	-1.0355	-1.57	0.11
14061	*ZFYVE21*	0.46796	0.19132	-0.31694	-0.70546	0.11
14062	*PRSS53*	1.7251	-0.48669	-0.89724	-1.1387	0.11
14063	*ST6GALNAC6*	0.3606	0.14118	-0.16069	-1.2739	0.11
14064	*DEFB112*	0.45661	0.42744	-0.54331	-1.2618	0.11
14065	*PSMB2*	1.7828	-0.15649	-1.2864	-1.5956	0.11
14066	*POLR3A*	1.9005	-0.66358	-0.89741	-1.9397	0.11
14067	*C19orf68*	0.21212	0.18894	-0.06373	-0.15822	0.11
14068	*SCRT2*	0.65565	0.52616	-0.84471	-1.4788	0.11
14069	*MKNK1*	0.8692	0.20034	-0.73259	-1.1383	0.11

14070	*GNG8*	0.34469	0.13635	-0.14449	-1.1897	0.11
14071	*P2RX3*	0.71341	-0.09448	-0.28254	-0.76033	0.11
14072	*NDNL2*	2.5708	-1.0697	-1.1653	-2.1814	0.11
14073	*ANXA8*	1.1954	-0.15041	-0.71063	-1.6487	0.11
14074	*OR2T1*	0.88185	0.62831	-1.1759	-2.0791	0.11
14075	*SARM1*	1.4561	0.3616	-1.4836	-1.8039	0.11
14076	*TULP2*	0.66957	0.10729	-0.44353	-2.3811	0.11
14077	*PCDH11X*	0.468	0.027154	-0.16249	-1.5194	0.11
14078	*RPS27A*	1.5283	-0.33179	-0.86403	-1.3542	0.11
14079	*RTN4RL1*	1.5505	-0.38103	-0.83713	-1.1135	0.11
14080	*ZNF160*	2.8032	-0.81248	-1.6587	-1.7591	0.11
14081	*EIF4G2*	0.28798	0.21077	-0.16836	-1.8523	0.11
14082	*MT1F*	0.47575	0.31176	-0.45786	-0.77105	0.11
14083	*ZPBP*	2.3502	-0.33799	-1.6826	-1.9844	0.11
14084	*NEK9*	1.2345	0.26472	-1.1705	-1.5461	0.11
14085	*CECR6*	1.6037	-0.40359	-0.8716	-2.3552	0.11
14086	*WNT10B*	1.1959	-0.00827	-0.85943	-0.87748	0.11
14087	*ZNF417*	0.37237	0.33953	-0.38597	-1.5289	0.11
14088	*PGD*	1.1744	-0.32785	-0.5223	-3.1656	0.11
14089	*SECISBP2*	2.9986	-0.50978	-2.1648	-2.3389	0.11
14090	*TSSK4*	1.9245	-0.48002	-1.1212	-1.1385	0.11
14091	*ZNF501*	1.7146	-0.65352	-0.73823	-1.0528	0.11
14092	*PCDHGC4*	2.8999	-0.6603	-1.9172	-2.3204	0.11
14093	*OTUB1*	2.1864	-0.64793	-1.2167	-1.6424	0.11
14094	*TBCA*	1.6518	0.30293	-1.6339	-2.5935	0.11
14095	*SRRM2*	2.4822	-0.89235	-1.2697	-2.095	0.11
14096	*KLHL1*	1.1669	-0.34158	-0.50535	-1.8157	0.11
14097	*TDRD10*	1.1165	-0.0227	-0.77397	-2.0022	0.11
14098	*OLFML3*	0.25827	0.056342	0.00408	-0.75832	0.11
14099	*C8orf44-SGK3*	1.3572	-0.16435	-0.87536	-0.88503	0.11
14100	*PUM2*	0.40424	0.16515	-0.25225	-0.96583	0.11
14101	*MTF1*	1.5403	0.19477	-1.4181	-1.4741	0.11
14102	*NUP153*	3.2396	-0.9481	-1.9746	-5.6002	0.11
14103	*BRD8*	1.4428	0.21771	-1.3437	-2.3235	0.11
14104	*SV2A*	0.81089	0.44296	-0.93707	-0.94583	0.11
14105	*KCTD11*	0.25601	0.08307	-0.02242	-2.2117	0.11
14106	*C16orf45*	0.87071	0.50708	-1.0614	-1.2716	0.11
14107	*NTNG2*	0.44101	0.055402	-0.18082	-1.7766	0.11
14108	*C19orf60*	1.5678	0.20713	-1.4597	-1.7936	0.11
14109	*AMN1*	0.32445	0.15185	-0.16107	-0.64002	0.11
14110	*ACSM2B*	0.36325	0.27104	-0.31947	-2.0851	0.10
14111	*MOV10*	0.7288	0.025762	-0.44043	-1.666	0.10
14112	*DYNC2LI1*	0.88189	0.34555	-0.91399	-0.96	0.10
14113	*HAPLN2*	1.3778	-0.19778	-0.86829	-2.625	0.10
14114	*FRAT1*	1.4269	0.3523	-1.4683	-1.6158	0.10
14115	*RHBDL3*	2.3724	-0.30811	-1.7547	-3.3367	0.10

14116	*ARHGAP26*	1.5333	0.059027	-1.2829	-2.9841	0.10
14117	*KDM8*	0.6854	0.0951	-0.47136	-0.64866	0.10
14118	*ACSM5*	0.6696	0.47755	-0.83808	-1.8952	0.10
14119	*ANKRD45*	0.2753	0.066884	-0.03356	-0.30898	0.10
14120	*TAF11*	0.76866	-0.09397	-0.36649	-2.0436	0.10
14121	*NBPF4*	1.4454	0.61576	-1.7533	-2.1367	0.10
14122	*PGBD5*	0.50836	0.46918	-0.67044	-1.4339	0.10
14123	*CATSPERB*	0.70494	0.12773	-0.52587	-1.3437	0.10
14124	*CTDP1*	2.7264	-1.191	-1.2288	-1.7906	0.10
14125	*ACAA2*	1.5138	-0.39248	-0.81501	-1.6339	0.10
14126	*NUP188*	1.3825	-0.35617	-0.72021	-1.6131	0.10
14127	*MAGEA6*	2.4419	-0.97768	-1.1587	-1.7686	0.10
14128	*GMCL1*	0.68177	0.5333	-0.912	-1.3393	0.10
14129	*MLH1*	0.30932	0.19879	-0.20512	-0.86298	0.10
14130	*ANKRD10*	1.6234	-0.40142	-0.91913	-1.7822	0.10
14131	*PEA15*	1.2138	0.69775	-1.6089	-3.9492	0.10
14132	*CCDC38*	1.3265	-0.48449	-0.53938	-0.56972	0.10
14133	*LRFN4*	0.24294	0.10691	-0.04792	-1.279	0.10
14134	*TRPV6*	0.66949	0.38094	-0.74875	-2.3534	0.10
14135	*PILRB*	0.51644	-0.10176	-0.11327	-1.2277	0.10
14136	*EP400*	1.1133	-0.301	-0.51122	-0.54197	0.10
14137	*SPAG6*	0.21342	0.1106	-0.02314	-1.4754	0.10
14138	*PPP2R5A*	1.0043	0.095684	-0.80154	-1.3982	0.10
14139	*RAPH1*	0.37109	0.18627	-0.25893	-2.201	0.10
14140	*CES2*	0.56104	0.13154	-0.39494	-0.71317	0.10
14141	*MSN*	1.0441	0.32513	-1.072	-1.6086	0.10
14142	*PLEKHD1*	2.1129	-0.25564	-1.5604	-1.9326	0.10
14143	*EFCAB12*	0.82474	0.049283	-0.57742	-1.222	0.10
14144	*ADA*	1.1993	-0.1123	-0.79083	-2.4784	0.10
14145	*BSG*	2.2564	-0.75373	-1.2071	-1.3756	0.10
14146	*RGS6*	0.5736	0.20038	-0.47929	-0.95423	0.10
14147	*CYLC2*	0.41761	0.36664	-0.48962	-0.86861	0.10
14148	*USP2*	0.49468	0.24525	-0.44541	-0.83972	0.10
14149	*SRP14*	0.32696	0.18841	-0.222	-1.1733	0.10
14150	*TAF7L*	0.77927	-0.06794	-0.4191	-0.98842	0.10
14151	*MOG*	1.0988	-0.05504	-0.75205	-1.0096	0.10
14152	*OTOP3*	0.97235	-0.2372	-0.44353	-1.6926	0.10
14153	*NLRP13*	0.53998	0.32373	-0.57212	-1.5894	0.10
14154	*HYOU1*	2.5148	-1.0948	-1.1288	-2.0634	0.10
14155	*RPL36AL*	1.4586	0.17321	-1.3407	-1.4902	0.10
14156	*RAB40AL*	0.53078	0.027032	-0.26677	-0.73826	0.10
14157	*FAM122C*	0.66963	0.4315	-0.8108	-1.0046	0.10
14158	*SSSCA1*	0.91	-0.28928	-0.33116	-1.7052	0.10
14159	*VANGL1*	0.15449	0.14314	-0.00829	-0.78229	0.10
14160	*SBF2*	0.47399	0.1179	-0.30326	-1.6648	0.10
14161	*KRI1*	2.7278	-0.96555	-1.4741	-2.1732	0.10

14162	*FSTL4*	0.65202	0.41325	-0.77733	-2.1519	0.10
14163	*ZNF85*	1.2115	-0.14918	-0.77667	-1.6739	0.10
14164	*HCFC1R1*	2.5106	-0.43332	-1.7918	-2.0553	0.10
14165	*SH3BGRL*	2.9733	-1.0567	-1.632	-2.3776	0.09
14166	*ADK*	0.45793	0.25552	-0.4295	-1.115	0.09
14167	*PSAT1*	1.7455	-0.59055	-0.87109	-1.1258	0.09
14168	*TXNL4B*	0.60448	-0.04002	-0.28062	-1.4527	0.09
14169	*C7orf33*	0.35355	0.12633	-0.19755	-0.85199	0.09
14170	*NHEJ1*	1.9558	-0.60869	-1.066	-1.6039	0.09
14171	*BRD9*	0.59299	0.32361	-0.63575	-2.8849	0.09
14172	*IFIT3*	2.9207	-1.2135	-1.4265	-2.1938	0.09
14173	*KIAA0232*	1.964	-0.38347	-1.3	-1.7153	0.09
14174	*PRPF39*	0.34161	0.28881	-0.35009	-0.54714	0.09
14175	*TTC38*	0.39053	-0.00278	-0.1082	-0.68021	0.09
14176	*SPACA4*	0.64808	-0.15143	-0.2179	-1.8598	0.09
14177	*MATR3*	2.0554	-0.72391	-1.0528	-1.1126	0.09
14178	*E2F1*	0.40063	0.33888	-0.46107	-1.8557	0.09
14179	*PLEKHO2*	1.2176	0.54731	-1.4867	-1.6653	0.09
14180	*BMP1*	1.9558	-0.78229	-0.89752	-1.1844	0.09
14181	*TSPAN15*	0.78114	-0.15702	-0.34816	-1.377	0.09
14182	*TAX1BP1*	2.289	-0.16634	-1.8478	-2.0301	0.09
14183	*KCNA3*	0.96511	-0.14449	-0.54657	-0.64002	0.09
14184	*ASPHD1*	0.47215	0.038046	-0.23688	-1.8849	0.09
14185	*CCNB1IP1*	1.1068	-0.40666	-0.42775	-0.89895	0.09
14186	*USP14*	0.85753	-0.251	-0.33613	-0.75548	0.09
14187	*DHRS11*	0.66286	-0.15247	-0.24061	-1.7394	0.09
14188	*PLAU*	1.1278	1.1164	-1.9749	-2.186	0.09
14189	*DFNB31*	0.29662	0.045563	-0.07299	-1.6597	0.09
14190	*MYNN*	0.14671	0.071115	0.05073	-0.80861	0.09
14191	*CCAR2*	1.8336	-0.11078	-1.4544	-1.5308	0.09
14192	*MBNL2*	0.78696	-0.07541	-0.44353	-1.6921	0.09
14193	*RUFY4*	2.2907	-0.82	-1.2031	-1.8031	0.09
14194	*FSD1*	1.1709	-0.41281	-0.49071	-2.1264	0.09
14195	*POLR2C*	1.3403	0.40969	-1.483	-1.8402	0.09
14196	*TMEM237*	0.9807	0.62646	-1.3409	-1.3729	0.09
14197	*JAZF1*	1.4802	-0.14244	-1.072	-1.4732	0.09
14198	*FOXRED1*	1.1557	0.15938	-1.0496	-1.7505	0.09
14199	*UQCR11*	1.1833	-0.32704	-0.59083	-1.2884	0.09
14200	*FRAS1*	1.7624	0.46526	-1.9623	-2.9772	0.09
14201	*SMTN*	2.9081	-1.2481	-1.3955	-1.8157	0.09
14202	*OR4K13*	1.0362	0.32334	-1.0951	-1.2864	0.09
14203	*SLC27A6*	0.66317	0.4856	-0.88435	-2.5208	0.09
14204	*ZER1*	1.309	-0.29953	-0.74572	-0.82439	0.09
14205	*NUP43*	1.0898	-0.23796	-0.58822	-1.1363	0.09
14206	*CLUH*	0.7821	-0.00339	-0.51547	-1.1962	0.09
14207	*RELT*	0.29117	0.28154	-0.31035	-0.49443	0.09

14208	*OR2D3*	1.073	-0.33456	-0.47632	-1.1241	0.09
14209	*SHPK*	0.48979	0.47345	-0.7015	-2.2381	0.09
14210	*C2orf66*	0.60444	-0.1497	-0.19422	-1.5165	0.09
14211	*VPS45*	1.118	0.89884	-1.7566	-1.9802	0.09
14212	*GEMIN8*	1.0471	-0.23292	-0.55505	-1.5984	0.09
14213	*SLC4A5*	0.31193	0.097436	-0.15098	-0.82307	0.09
14214	*C7orf57*	0.8337	0.82035	-1.3961	-1.8724	0.09
14215	*SHC3*	0.26355	0.032616	-0.04046	-0.94927	0.09
14216	*SPINK1*	0.41698	0.073084	-0.23476	-0.91296	0.09
14217	*WAC*	0.48671	0.12704	-0.35896	-2.101	0.08
14218	*TMEM161A*	2.7047	-1.1074	-1.3427	-1.3599	0.08
14219	*NCAM2*	1.1135	-0.00182	-0.85712	-1.0765	0.08
14220	*MVK*	0.32659	0.17555	-0.24797	-0.358	0.08
14221	*WBP2NL*	1.1144	0.071506	-0.93217	-1.2619	0.08
14222	*CDO1*	2.1614	-0.7734	-1.1344	-2.374	0.08
14223	*CBX5*	1.7238	-0.42276	-1.0483	-1.4234	0.08
14224	*POLR1B*	1.1674	-0.44353	-0.47199	-1.6956	0.08
14225	*UBTFL1*	1.5634	0.057397	-1.3701	-1.6129	0.08
14226	*MDH1*	1.0681	0.66648	-1.4841	-2.2879	0.08
14227	*GSX2*	0.56146	-0.12667	-0.18504	-0.22043	0.08
14228	*COL11A2*	0.99442	-0.14925	-0.59572	-1.1815	0.08
14229	*MYRF*	2.6666	-1.0564	-1.3613	-1.3769	0.08
14230	*A2M*	0.41131	0.002435	-0.16485	-1.9116	0.08
14231	*CEP72*	2.824	-0.57308	-2.0028	-2.1519	0.08
14232	*METTL7B*	0.61031	0.04489	-0.4072	-2.4517	0.08
14233	*PRR3*	0.70504	0.69371	-1.1508	-1.5894	0.08
14234	*TCHP*	0.62164	0.12149	-0.49652	-0.93465	0.08
14235	*RASL10B*	0.22314	0.02563	-0.00309	-0.85768	0.08
14236	*EEF1G*	2.1544	-0.57408	-1.335	-1.6461	0.08
14237	*C1orf228*	1.1099	0.089784	-0.95461	-1.4087	0.08
14238	*KIRREL2*	0.67198	0.13947	-0.56687	-1.1422	0.08
14239	*C2orf54*	1.2484	-0.10146	-0.90281	-1.059	0.08
14240	*C7orf69*	1.4761	-0.13737	-1.095	-2.2809	0.08
14241	*KRTAP9-3*	1.0504	-0.09082	-0.71626	-1.3409	0.08
14242	*SPRR2B*	2.4241	-0.46741	-1.7134	-2.1151	0.08
14243	*HUS1*	3.1851	-0.875	-2.0669	-2.239	0.08
14244	*ZMPSTE24*	0.63277	-0.18353	-0.20663	-1.0158	0.08
14245	*PRR20B*	1.3986	-0.26575	-0.8903	-0.91823	0.08
14246	*NLRC3*	2.2642	-0.61798	-1.4039	-1.5817	0.08
14247	*EXOSC6*	1.3665	-0.3555	-0.76909	-1.6245	0.08
14248	*KCNH1*	0.596	0.18761	-0.54178	-1.5082	0.08
14249	*RNF11*	1.1733	-0.34113	-0.59128	-0.67004	0.08
14250	*CSRP2*	0.67698	-0.15394	-0.28357	-1.2505	0.08
14251	*PIAS3*	0.70898	-0.20242	-0.26714	-1.9157	0.08
14252	*HIST1H4E*	0.37869	0.19012	-0.33205	-2.2962	0.08
14253	*LOC401052*	0.67399	0.15724	-0.59472	-0.79035	0.08

14254	*ARMC6*	2.0338	-0.59128	-1.2065	-2.3357	0.08
14255	*GAGE12C*	2.0997	-0.6424	-1.222	-2.1219	0.08
14256	*SPRYD3*	0.98173	-0.28598	-0.46153	-2.0902	0.08
14257	*RRN3*	3.1321	-1.1472	-1.7512	-2.2237	0.08
14258	*ZNF10*	0.67902	0.025582	-0.47158	-1.4036	0.08
14259	*DCAF10*	0.83089	0.80603	-1.4043	-3.1578	0.08
14260	*SELO*	2.1182	-0.7635	-1.1226	-2.6201	0.08
14261	*BCL2*	0.83459	0.62919	-1.2317	-1.716	0.08
14262	*SLC46A1*	0.50751	0.46122	-0.73695	-1.6001	0.08
14263	*SPDYE3*	1.9391	-0.62028	-1.0875	-1.8267	0.08
14264	*RASEF*	0.43778	0.18091	-0.38779	-0.50104	0.08
14265	*PROCR*	1.2302	-0.42386	-0.57551	-1.8775	0.08
14266	*AMY2A*	0.749	0.25411	-0.77241	-2.6003	0.08
14267	*DHRSX*	1.3226	-0.34653	-0.74586	-0.84801	0.08
14268	*BTG2*	1.1811	-0.15543	-0.79584	-1.1846	0.08
14269	*VIPR1*	1.416	0.13369	-1.3199	-1.8398	0.08
14270	*CCDC88C*	1.5287	-0.57825	-0.72095	-2.278	0.08
14271	*HLTF*	0.64346	0.25593	-0.66992	-0.70325	0.08
14272	*RBM4*	0.58931	0.17276	-0.53321	-2.1334	0.08
14273	*RAB3GAP2*	2.8898	-0.9636	-1.6981	-2.3594	0.08
14274	*HEPACAM2*	0.85724	-0.29292	-0.33638	-1.3393	0.08
14275	*ZNF169*	2.1397	-0.87739	-1.0344	-1.8636	0.08
14276	*DDX51*	0.80457	-0.20869	-0.36821	-1.01	0.08
14277	*TMCO2*	0.79104	0.36049	-0.92486	-4.3295	0.08
14278	*OR51G1*	1.3071	0.061507	-1.1439	-1.6044	0.07
14279	*PITPNM2*	0.51723	0.019879	-0.31294	-2.2879	0.07
14280	*FAM151B*	3.0831	-1.1287	-1.7303	-2.3517	0.07
14281	*TANGO6*	1.6094	-0.10425	-1.2815	-2.201	0.07
14282	*CTSB*	0.91288	0.17924	-0.86884	-2.0548	0.07
14283	*ACTRT2*	0.54593	0.056547	-0.38112	-0.81217	0.07
14284	*CNOT10*	1.4649	-0.56444	-0.6794	-0.82789	0.07
14285	*RBM38*	0.36848	0.051389	-0.20107	-1.5219	0.07
14286	*SDHAF2*	1.5849	-0.54283	-0.82337	-1.4538	0.07
14287	*C7orf55-LUC7L2*	0.97338	-0.00268	-0.75414	-1.8702	0.07
14288	*HLA-DQB2*	1.8556	-0.45443	-1.1848	-1.6199	0.07
14289	*CACNG2*	0.34685	-0.00487	-0.12595	-0.88391	0.07
14290	*TREH*	1.4341	-0.57899	-0.64002	-2.7678	0.07
14291	*RNH1*	2.1891	-0.9512	-1.0243	-1.1303	0.07
14292	*OR7E24*	0.89213	0.6976	-1.377	-2.3065	0.07
14293	*INO80B*	0.35671	0.16473	-0.30899	-1.386	0.07
14294	*HOXD11*	0.66962	0.29586	-0.75388	-1.4031	0.07
14295	*ZSWIM1*	0.80313	0.46135	-1.0529	-1.7493	0.07
14296	*EFTUD1*	1.4424	-0.21491	-1.0164	-3.2873	0.07
14297	*TMEM167A*	0.15229	0.11639	-0.05784	-0.80341	0.07
14298	*SUN2*	0.43657	-0.03356	-0.1926	-1.1287	0.07
14299	*SUMO1*	1.7979	0.035843	-1.6237	-1.7577	0.07

14300	*PRAME*	0.89844	-0.34232	-0.34632	-0.77514	0.07
14301	*MRPS2*	0.54692	0.19351	-0.53078	-0.94052	0.07
14302	*GSTM3*	0.76226	0.066172	-0.61889	-1.2973	0.07
14303	*APOF*	2.7889	-0.77733	-1.8023	-2.2338	0.07
14304	*CHMP5*	0.89076	0.49401	-1.1761	-1.9367	0.07
14305	*PAIP2*	0.31176	0.023908	-0.12784	-2.8478	0.07
14306	*NME3*	0.40836	-0.08446	-0.11621	-1.0391	0.07
14307	*WDR72*	1.7385	-0.21349	-1.3184	-1.4685	0.07
14308	*SYT12*	0.39423	-0.08164	-0.10687	-0.67801	0.07
14309	*LMNB2*	1.2046	0.081171	-1.0811	-1.7683	0.07
14310	*CREM*	1.071	0.53673	-1.4034	-2.6889	0.07
14311	*MAOA*	2.092	-0.14091	-1.7495	-2.1984	0.07
14312	*SAMD9L*	0.70647	0.26607	-0.77105	-1.6731	0.07
14313	*SHISA5*	0.50027	-0.0836	-0.21526	-1.4717	0.07
14314	*BRI3BP*	0.66954	-0.01739	-0.45185	-1.2633	0.07
14315	*SPTBN1*	1.649	-0.06523	-1.3837	-2.4718	0.07
14316	*TCFL5*	0.64732	-0.20554	-0.24238	-2.0952	0.07
14317	*IFT140*	0.25665	0.000534	-0.06021	-1.0656	0.07
14318	*TOR1AIP2*	2.5797	-1.1585	-1.2251	-1.4954	0.07
14319	*RPP38*	2.7879	-0.36734	-2.2254	-2.4328	0.07
14320	*INPP5K*	1.5154	-0.20843	-1.1121	-1.4858	0.06
14321	*REEP3*	0.55037	0.26122	-0.61675	-1.8804	0.06
14322	*ISX*	0.46892	-0.04015	-0.23404	-1.7947	0.06
14323	*SEMG2*	1.4648	-0.17676	-1.095	-2.1182	0.06
14324	*CAV1*	0.56046	0.054397	-0.4221	-0.52552	0.06
14325	*FSD1L*	0.92294	-0.16263	-0.56764	-1.2226	0.06
14326	*CWF19L1*	0.89887	0.33339	-1.0407	-1.1807	0.06
14327	*TPTE*	3.1965	-1.1105	-1.8946	-1.9578	0.06
14328	*TRAPPC2L*	3.4479	-1.4141	-1.843	-2.29	0.06
14329	*NCF1*	0.77365	-0.13963	-0.44353	-2.8229	0.06
14330	*ZNF441*	1.0488	-0.06585	-0.79253	-1.1757	0.06
14331	*MROH2B*	0.38839	0.18932	-0.38843	-0.45695	0.06
14332	*LIX1*	0.90273	-0.26575	-0.4497	-1.4683	0.06
14333	*CT45A7*	0.72248	-0.13638	-0.39932	-1.5579	0.06
14334	*COPRS*	1.0101	-0.21794	-0.6057	-1.578	0.06
14335	*COQ5*	2.3205	-0.36127	-1.773	-2.4328	0.06
14336	*FAM72C*	0.53464	-0.16263	-0.18589	-1.4997	0.06
14337	*AFAP1L1*	0.70561	-0.03196	-0.48756	-2.2433	0.06
14338	*MPI*	1.437	0.24633	-1.4976	-1.6469	0.06
14339	*CCNJL*	0.49655	-0.1126	-0.19869	-1.701	0.06
14340	*LHX2*	1.1366	-0.39527	-0.55641	-1.1395	0.06
14341	*CASC5*	0.80081	0.21184	-0.82844	-1.8651	0.06
14342	*BCAS2*	0.5394	0.21274	-0.56812	-2.0134	0.06
14343	*KBTBD2*	2.0949	-0.40281	-1.5082	-1.9494	0.06
14344	*ORAOV1*	0.74443	-0.15783	-0.40353	-2.3164	0.06
14345	*FOXH1*	2.483	-0.77241	-1.5282	-1.9367	0.06

14346	*NUDT4*	1.5482	-0.54813	-0.8181	-1.2485	0.06
14347	*IFNA2*	0.455	0.037733	-0.31157	-1.8031	0.06
14348	*CENPV*	0.92051	-0.3635	-0.37591	-2.1444	0.06
14349	*TTC28*	3.1711	-1.4269	-1.5632	-3.1195	0.06
14350	*RBM22*	1.4806	-0.38351	-0.91696	-1.4204	0.06
14351	*CRNKL1*	1.1162	0.058283	-0.99463	-1.2839	0.06
14352	*TMA7*	3.0113	-0.65199	-2.1798	-2.6445	0.06
14353	*KRT79*	0.565	-0.04127	-0.34443	-1.3275	0.06
14354	*FITM2*	1.5399	-0.6558	-0.70501	-1.8946	0.06
14355	*NUP35*	1.3706	-0.00331	-1.1888	-1.6004	0.06
14356	*CCDC103*	2.2041	-0.90693	-1.12	-1.577	0.06
14357	*CD38*	0.30854	0.16852	-0.30007	-2.0181	0.06
14358	*IAPP*	0.77982	-0.27055	-0.33253	-0.63357	0.06
14359	*PAQR3*	2.5777	-0.61779	-1.7845	-2.2343	0.06
14360	*ZNF713*	0.38681	0.21463	-0.42609	-0.78229	0.06
14361	*CCBL1*	2.3063	-0.38173	-1.7493	-2.3557	0.06
14362	*NARS2*	0.84228	0.33109	-0.99834	-1.9291	0.06
14363	*TALDO1*	1.2581	-0.10178	-0.98159	-2.1947	0.06
14364	*SYS1*	2.1916	-0.6568	-1.3604	-1.8376	0.06
14365	*GGT1*	0.41647	0.048132	-0.29069	-0.78439	0.06
14366	*IL1B*	0.69055	0.23385	-0.7512	-1.6525	0.06
14367	*ACR*	2.2765	-0.44353	-1.6599	-1.9166	0.06
14368	*MYH11*	0.49992	0.11577	-0.44353	-3.0687	0.06
14369	*SDPR*	1.8043	-0.46112	-1.1718	-1.4568	0.06
14370	*MTMR12*	0.94047	-0.32621	-0.44353	-1.1929	0.06
14371	*FAM47A*	0.67885	0.05795	-0.56634	-1.2647	0.06
14372	*ELSPBP1*	1.0379	-0.06513	-0.80248	-1.1921	0.06
14373	*PARP1*	1.3183	-0.54797	-0.60095	-1.6125	0.06
14374	*GANC*	1.3195	0.34243	-1.4926	-2.1023	0.06
14375	*MXD3*	1.1677	-0.24415	-0.75437	-1.222	0.06
14376	*IRG1*	0.9734	-0.30359	-0.50075	-1.105	0.06
14377	*DDX4*	0.48856	0.041348	-0.36086	-1.236	0.06
14378	*SAGE1*	0.8868	-0.09924	-0.61873	-2.6705	0.06
14379	*NKX3-2*	0.72429	-0.2372	-0.31865	-0.99277	0.06
14380	*KLRB1*	1.0299	-0.41207	-0.4494	-0.89235	0.06
14381	*ST3GAL3*	1.7484	-0.64011	-0.94019	-1.1193	0.06
14382	*TRAPPC1*	0.5188	-0.06364	-0.28778	-0.64571	0.06
14383	*STRN*	2.4856	-0.78097	-1.5375	-2.1187	0.06
14384	*PDSS1*	0.45405	0.22464	-0.5116	-1.9133	0.06
14385	*EIF3F*	2.3049	-0.52968	-1.6086	-1.8684	0.06
14386	*ABCB9*	0.66949	0.10642	-0.61029	-0.91192	0.06
14387	*UBE3D*	1.1709	0.033415	-1.041	-1.5782	0.05
14388	*CPSF3L*	1.6125	0.086199	-1.5357	-1.554	0.05
14389	*PSMD9*	0.39979	0.39826	-0.63513	-1.9383	0.05
14390	*UTP15*	0.5006	-0.13753	-0.2005	-1.197	0.05
14391	*AFF2*	0.6082	-0.20642	-0.23931	-0.71987	0.05

14392	*RNF121*	1.328	-0.23446	-0.93112	-1.4448	0.05
14393	*H2AFY2*	0.6802	0.3593	-0.87772	-2.4491	0.05
14394	*SUMF2*	0.40857	0.072713	-0.31951	-1.7462	0.05
14395	*RPL13A*	3.6635	-1.7466	-1.7554	-1.9567	0.05
14396	*SPNS3*	0.64312	0.51131	-0.99368	-1.6867	0.05
14397	*TRIM27*	0.64739	0.1718	-0.6602	-1.3613	0.05
14398	*EPC2*	0.84592	0.59007	-1.278	-1.9047	0.05
14399	*SMG9*	1.6616	0.11559	-1.6199	-5.9418	0.05
14400	*FTO*	0.97648	0.088149	-0.90757	-1.7483	0.05
14401	*CEP290*	3.1682	-0.95802	-2.0536	-2.1255	0.05
14402	*KDM3B*	0.83213	-0.27491	-0.40095	-0.66708	0.05
14403	*HAPLN1*	1.4439	0.024941	-1.3127	-2.412	0.05
14404	*FRZB*	0.72619	-0.26737	-0.30335	-2.672	0.05
14405	*ING5*	0.47829	0.47464	-0.79798	-2.5125	0.05
14406	*C17orf62*	1.6007	-0.47818	-0.9686	-1.3343	0.05
14407	*RASL11A*	2.1287	-0.25403	-1.7209	-1.9622	0.05
14408	*FN3K*	1.6179	0.28138	-1.7457	-1.8604	0.05
14409	*CHI3L2*	1.4635	0.062832	-1.3728	-1.452	0.05
14410	*SGPP2*	1.2888	0.96933	-2.1046	-2.2264	0.05
14411	*RAVER2*	2.7479	-1.1508	-1.4442	-1.9068	0.05
14412	*SKP1*	3.2621	-1.2505	-1.8591	-3.1939	0.05
14413	*CLDN2*	1.261	-0.44742	-0.66119	-0.98585	0.05
14414	*GRIK3*	0.61339	-0.16435	-0.2969	-2.2094	0.05
14415	*GMPR2*	2.0684	-0.50127	-1.4151	-1.9086	0.05
14416	*DCAF13*	0.61672	0.22036	-0.68565	-1.9213	0.05
14417	*WASH1*	0.9177	-0.02368	-0.7436	-1.4841	0.05
14418	*PGAM2*	0.79175	0.29113	-0.93392	-3.2912	0.05
14419	*MRPS12*	1.0835	-0.45339	-0.48119	-1.6601	0.05
14420	*GNAT1*	3.4559	-1.6049	-1.7024	-2.9054	0.05
14421	*TMEM208*	0.86356	0.2126	-0.9285	-1.1761	0.05
14422	*ST20-MTHFS*	1.9448	-0.27811	-1.5213	-1.8653	0.05
14423	*PLCH2*	0.88809	-0.10484	-0.63826	-1.0386	0.05
14424	*UNC119*	1.0522	0.0973	-1.0048	-1.1444	0.05
14425	*IER3IP1*	0.29953	-0.02098	-0.13389	-0.14506	0.05
14426	*PAIP1*	3.0782	-1.448	-1.4873	-2.9897	0.05
14427	*ZBTB20*	0.31366	0.16806	-0.33933	-2.0626	0.05
14428	*GNG12*	0.97316	-0.01606	-0.81496	-1.3132	0.05
14429	*EFHC1*	2.0395	-0.32699	-1.571	-1.7177	0.05
14430	*TMEM150A*	0.98137	-0.05801	-0.78229	-1.849	0.05
14431	*PRKAR2A*	1.9161	-0.34998	-1.4252	-2.7158	0.05
14432	*CNPY4*	1.135	-0.20976	-0.7853	-1.5965	0.05
14433	*UFD1L*	1.7886	-0.56825	-1.0809	-1.4829	0.05
14434	*RGCC*	0.4766	0.078218	-0.41596	-0.51221	0.05
14435	*SH2D6*	1.8187	-0.28197	-1.3987	-2.2352	0.05
14436	*ELF5*	1.6149	0.005207	-1.4826	-2.6593	0.05
14437	*POP7*	1.83	-0.16634	-1.5268	-2.0055	0.05

14438	*H2AFX*	1.2058	-0.14906	-0.91996	-1.9938	0.05
14439	*COLCA1*	1.3881	-0.21666	-1.035	-3.0444	0.05
14440	*XPA*	1.1968	-0.36861	-0.69287	-1.1659	0.05
14441	*DLX2*	1.4015	-0.62792	-0.63826	-1.6456	0.05
14442	*RWDD2A*	1.2658	0.26025	-1.391	-1.4964	0.05
14443	*XPO1*	0.88615	-0.30777	-0.44353	-1.9538	0.04
14444	*OR11G2*	1.2414	-0.42841	-0.67889	-1.8698	0.04
14445	*RNF141*	0.35855	-0.09907	-0.12563	-0.97876	0.04
14446	*UBE2Z*	1.1419	0.61775	-1.626	-2.8181	0.04
14447	*YAF2*	0.70768	0.36965	-0.94406	-0.97303	0.04
14448	*NPFF*	0.53719	0.17837	-0.58289	-1.0782	0.04
14449	*CXCR2*	0.47831	-0.1194	-0.2293	-0.70475	0.04
14450	*GTF3C5*	1.052	0.34656	-1.2691	-1.4488	0.04
14451	*POM121*	0.61765	-0.01725	-0.47244	-1.0268	0.04
14452	*MRTO4*	0.55465	-0.03288	-0.39494	-0.67007	0.04
14453	*NGDN*	0.87226	0.13956	-0.88503	-1.545	0.04
14454	*MFRP*	3.0671	-1.2884	-1.6525	-2.337	0.04
14455	*PLG*	1.2503	-0.33112	-0.79319	-1.0167	0.04
14456	*TOMM20L*	0.90094	-0.03784	-0.73739	-0.79932	0.04
14457	*PPM1K*	1.1393	0.058918	-1.0727	-2.1507	0.04
14458	*SENP2*	1.5634	0.15743	-1.5979	-1.808	0.04
14459	*CREB3L1*	2.5593	-0.86459	-1.5722	-1.7179	0.04
14460	*LGALS14*	0.27963	0.097436	-0.25478	-1.9365	0.04
14461	*AUH*	0.44002	-0.15085	-0.1677	-0.76955	0.04
14462	*ASF1A*	1.4851	-0.43906	-0.92553	-1.0344	0.04
14463	*TRIM39-RPP21*	0.55152	0.15938	-0.59045	-1.4841	0.04
14464	*RPL10A*	3.3876	-1.4683	-1.7994	-2.2955	0.04
14465	*DCAF15*	0.66487	-0.12758	-0.41748	-1.2235	0.04
14466	*ZMIZ1*	0.53532	-0.05505	-0.36112	-1.3771	0.04
14467	*TCEB1*	0.98208	0.55442	-1.4182	-2.9068	0.04
14468	*LTBR*	0.152	0.016357	-0.05165	-0.13189	0.04
14469	*ZNF836*	1.2303	0.25142	-1.3665	-1.8334	0.04
14470	*CARTPT*	3.1556	-1.1953	-1.8456	-2.1596	0.04
14471	*CHD2*	0.85843	0.69182	-1.4368	-1.6268	0.04
14472	*CYB561D1*	0.45121	0.14609	-0.48419	-0.63187	0.04
14473	*PHF20*	0.68584	0.14108	-0.71465	-1.5748	0.04
14474	*ARID1A*	0.37059	0.31999	-0.57899	-1.8406	0.04
14475	*BACH1*	0.40749	0.35512	-0.65201	-2.9068	0.04
14476	*CACNA2D4*	0.53876	-0.1589	-0.26949	-2.4174	0.04
14477	*RNF138*	0.84448	0.28658	-1.0212	-1.7462	0.04
14478	*DNAAF5*	0.47055	-0.09671	-0.26409	-1.4537	0.04
14479	*USP6NL*	0.28831	-0.0429	-0.13576	-1.6437	0.04
14480	*IFNA1*	1.4921	-0.5101	-0.87256	-1.5634	0.04
14481	*SIRT3*	0.53797	0.42852	-0.85739	-1.1932	0.04
14482	*RAB37*	0.29212	0.25944	-0.44353	-1.8689	0.04
14483	*PNKD*	0.68707	0.020456	-0.60085	-2.1342	0.04

14484	*METTL21C*	1.4993	-0.38351	-1.0095	-2.0778	0.04
14485	*MEP1A*	0.44364	-0.0067	-0.33107	-0.50201	0.04
14486	*SLIT3*	0.35557	0.24544	-0.49525	-1.1907	0.04
14487	*GPR31*	1.5017	-0.12048	-1.2755	-2.3601	0.04
14488	*XPR1*	0.5365	-0.00112	-0.43027	-1.1665	0.04
14489	*NOP58*	0.792	0.056445	-0.74392	-0.82311	0.03
14490	*NDUFS2*	1.1627	-0.49926	-0.55898	-0.63368	0.03
14491	*NPAS4*	1.2656	0.13896	-1.3007	-1.6597	0.03
14492	*HHLA2*	0.85028	0.32173	-1.0691	-3.7236	0.03
14493	*HIST1H2AH*	2.0925	-0.80936	-1.1812	-1.5164	0.03
14494	*TEAD1*	0.51092	-0.05186	-0.35733	-0.57579	0.03
14495	*MYOG*	0.36761	0.19477	-0.46153	-0.73896	0.03
14496	*CCDC181*	1.3489	0.16383	-1.4123	-1.7591	0.03
14497	*PDCD2*	3.1536	-1.0838	-1.9695	-2.1956	0.03
14498	*KIF26B*	2.8595	-1.082	-1.6776	-1.8537	0.03
14499	*SPAG9*	0.1466	0.034812	-0.08163	-1.2135	0.03
14500	*COX7A2L*	2.2335	-0.70188	-1.4319	-1.5204	0.03
14501	*PNLIPRP1*	0.51887	0.11411	-0.53332	-0.98719	0.03
14502	*SARS*	0.6627	0.20292	-0.76748	-2.7427	0.03
14503	*LIM2*	1.3737	-0.09654	-1.1792	-1.3582	0.03
14504	*DDB2*	0.23703	0.20268	-0.34228	-0.79149	0.03
14505	*TMEM240*	1.1805	0.30663	-1.3899	-1.6957	0.03
14506	*MTRNR2L7*	0.098148	0.073963	-0.07512	-1.8095	0.03
14507	*CACNB1*	0.67388	-0.12795	-0.4497	-0.76433	0.03
14508	*NOS2*	0.66961	0.54502	-1.1184	-2.2434	0.03
14509	*EXD1*	0.73245	0.57498	-1.2118	-1.3389	0.03
14510	*CCDC107*	1.1165	0.092774	-1.1138	-1.8443	0.03
14511	*FRMD3*	2.2005	0.009147	-2.1151	-2.8181	0.03
14512	*MFAP5*	0.38147	-0.04543	-0.24157	-0.38219	0.03
14513	*PDE4DIP*	0.97566	0.35805	-1.2399	-2.7566	0.03
14514	*CNTN3*	0.76104	-0.03127	-0.63614	-1.4154	0.03
14515	*PHKA1*	0.21734	0.12515	-0.24904	-0.94609	0.03
14516	*UGT1A9*	0.95587	-0.37215	-0.49045	-0.62372	0.03
14517	*FBXL3*	1.3084	-0.22008	-0.99508	-1.0005	0.03
14518	*CLVS1*	0.71737	-0.22324	-0.40115	-2.1055	0.03
14519	*PPP1R36*	0.16074	0.001415	-0.06966	-0.44353	0.03
14520	*LRP8*	0.57023	-0.00023	-0.47781	-1.4568	0.03
14521	*ATF7IP2*	1.7826	-0.00268	-1.6881	-1.9515	0.03
14522	*SURF1*	2.5243	-1.1754	-1.2572	-1.4299	0.03
14523	*MBOAT2*	3.0748	-1.2371	-1.7466	-2.3689	0.03
14524	*MALSU1*	2.3135	0.18932	-2.412	-3.2582	0.03
14525	*SAR1A*	0.50043	0.007935	-0.4181	-1.9186	0.03
14526	*EIF3I*	0.65863	-0.01558	-0.55335	-1.2715	0.03
14527	*TIRAP*	1.305	-0.48708	-0.72839	-1.0764	0.03
14528	*EGR1*	0.42468	-0.11106	-0.22445	-0.24864	0.03
14529	*CMYA5*	1.213	-0.54887	-0.57513	-1.5904	0.03

14530	*TOP1MT*	0.58055	0.50314	-0.99474	-1.0134	0.03
14531	*NT5C1B-RDH14*	1.2088	-0.34903	-0.77114	-1.4544	0.03
14532	*CHKA*	0.4504	-0.09295	-0.27016	-1.6759	0.03
14533	*RASSF10*	1.1531	-0.36258	-0.70394	-2.6753	0.03
14534	*CHPT1*	1.2523	-0.461	-0.70601	-1.5482	0.03
14535	*ACTL9*	1.9356	-0.78503	-1.0667	-1.9459	0.03
14536	*RASGRP4*	2.2435	-0.79981	-1.3599	-1.6838	0.03
14537	*CWF19L2*	2.4707	-0.9728	-1.4142	-1.714	0.03
14538	*LOC100996634*	1.0848	-0.27961	-0.72157	-1.2154	0.03
14539	*PRAMEF11*	0.52757	-0.2168	-0.22783	-1.0131	0.03
14540	*C10orf107*	0.31702	0.16813	-0.4027	-0.52269	0.03
14541	*SPTSSB*	0.36325	-0.1284	-0.15303	-1.6023	0.03
14542	*CLUAP1*	0.5519	-0.09723	-0.37424	-2.1983	0.03
14543	*ZNF680*	0.97366	-0.16623	-0.7288	-3.4924	0.03
14544	*IGF2BP3*	1.0347	0.11462	-1.071	-1.6251	0.03
14545	*NME9*	0.71578	-0.14183	-0.49636	-0.83095	0.03
14546	*LRIG3*	0.5977	-0.17418	-0.34597	-0.74743	0.03
14547	*MRPS25*	1.495	-0.65537	-0.76212	-1.9055	0.03
14548	*TRPC4*	3.5606	-1.522	-1.9622	-2.0242	0.03
14549	*DDX42*	0.70907	-0.04689	-0.58661	-2.103	0.03
14550	*NPM1*	2.5527	-1.2297	-1.2485	-1.9794	0.02
14551	*TIMM23*	1.6635	-0.68193	-0.90713	-2.1873	0.02
14552	*POPDC3*	1.0325	0.21634	-1.1747	-2.5442	0.02
14553	*NEURL1B*	0.24919	0.11223	-0.28796	-2.2108	0.02
14554	*LOC81691*	0.23111	0.22142	-0.37982	-1.1071	0.02
14555	*PEX11B*	0.60965	-0.09403	-0.44353	-0.73922	0.02
14556	*METTL2B*	1.1027	-0.13483	-0.89592	-1.8087	0.02
14557	*ST3GAL2*	0.74731	0.52934	-1.205	-1.448	0.02
14558	*GABBR1*	0.60031	0.54	-1.0691	-2.8928	0.02
14559	*HPN*	0.29893	0.027839	-0.25583	-0.32414	0.02
14560	*TRAT1*	1.4198	-0.45119	-0.89784	-1.6802	0.02
14561	*RAB34*	1.3569	-0.35196	-0.93465	-1.5726	0.02
14562	*ARID2*	0.48253	0.004561	-0.41716	-0.94171	0.02
14563	*ASCC3*	0.19992	-0.03623	-0.09523	-1.4276	0.02
14564	*CYP2C18*	2.1402	-0.65368	-1.4195	-1.6525	0.02
14565	*EMC6*	0.31244	0.19871	-0.44433	-1.3017	0.02
14566	*NRBF2*	0.51261	0.25105	-0.69727	-1.5263	0.02
14567	*SPRR1B*	1.2584	0.43722	-1.63	-2.2434	0.02
14568	*MTIF2*	1.6795	-0.57301	-1.0413	-1.7428	0.02
14569	*CCDC110*	0.97486	-0.22131	-0.6886	-1.3134	0.02
14570	*LYRM4*	2.1946	-1.0564	-1.0737	-1.1281	0.02
14571	*ACTN3*	0.92645	-0.0729	-0.78951	-3.164	0.02
14572	*TSEN34*	1.5091	-0.45663	-0.98925	-1.338	0.02
14573	*OR2T27*	0.3454	0.26472	-0.54724	-0.78398	0.02
14574	*NXNL2*	0.87719	0.5456	-1.3602	-1.701	0.02
14575	*TBC1D21*	1.8576	-0.56687	-1.2284	-1.7735	0.02

14576	*CXorf57*	0.41697	-0.04277	-0.31191	-1.0918	0.02
14577	*FBXO39*	0.45363	0.43735	-0.82879	-1.2534	0.02
14578	*FNDC1*	1.0167	-0.10258	-0.85194	-1.8975	0.02
14579	*WDR86*	1.8144	-0.44353	-1.3098	-1.4141	0.02
14580	*RASL10A*	0.83566	0.068993	-0.84375	-0.88008	0.02
14581	*C1QTNF7*	1.4673	-0.22235	-1.1845	-1.7647	0.02
14582	*LRRTM4*	0.39759	-0.15276	-0.18445	-0.26231	0.02
14583	*C1orf106*	1.7563	-0.80847	-0.88797	-1.6255	0.02
14584	*SEPW1*	0.52604	0.16057	-0.62762	-0.90161	0.02
14585	*TLCD2*	0.19205	0.054921	-0.18808	-0.99694	0.02
14586	*FECH*	1.929	-0.58509	-1.2852	-1.7496	0.02
14587	*SLC16A4*	1.1634	-0.15855	-0.94625	-1.399	0.02
14588	*PANK2*	0.99303	-0.21743	-0.71717	-1.195	0.02
14589	*NPEPL1*	0.62681	0.37869	-0.94739	-1.6691	0.02
14590	*PALM2*	0.93999	-0.26252	-0.62114	-1.6208	0.02
14591	*TTR*	2.1921	-0.84457	-1.2912	-1.5874	0.02
14592	*CST2*	2.6424	-1.183	-1.4034	-3.1229	0.02
14593	*SLC17A1*	0.78904	0.19924	-0.93307	-1.632	0.02
14594	*DNTTIP1*	1.055	-0.35211	-0.64788	-1.7049	0.02
14595	*PPAPDC2*	0.11005	-0.02615	-0.02908	-0.78337	0.02
14596	*CASKIN2*	0.45582	0.33443	-0.73599	-1.0384	0.02
14597	*LAMTOR1*	2.8229	-1.2898	-1.4789	-1.5182	0.02
14598	*GOT2*	1.6683	-0.59212	-1.0221	-1.4962	0.02
14599	*NKTR*	0.29836	0.018783	-0.26369	-0.28907	0.02
14600	*MCHR2*	1.2044	-0.38361	-0.76755	-1.3218	0.02
14601	*VSNL1*	1.2057	-0.31624	-0.83631	-1.3353	0.02
14602	*GLB1L*	0.8351	0.17417	-0.95717	-1.2396	0.02
14603	*SIGLEC14*	1.8187	-0.59195	-1.1747	-1.5744	0.02
14604	*UBE4B*	0.7252	0.3877	-1.0614	-2.0363	0.02
14605	*GBX1*	1.9746	-0.55371	-1.3694	-1.6676	0.02
14606	*EXOC6*	2.5698	-0.87768	-1.6411	-2.0337	0.02
14607	*SPANXD*	0.67353	0.46804	-1.0906	-2.2607	0.02
14608	*SMIM11*	0.35927	0.088577	-0.39856	-1.0564	0.02
14609	*GTF2H5*	0.96644	0.30746	-1.2252	-2.7777	0.02
14610	*NTAN1*	0.44466	0.027947	-0.42397	-2.5442	0.02
14611	*KCNB1*	0.47204	-0.20318	-0.22133	-1.0399	0.02
14612	*CLDN15*	3.3585	-1.4597	-1.8523	-3.164	0.02
14613	*TMEM236*	2.9312	-1.2572	-1.6276	-2.1143	0.02
14614	*BCL10*	0.88577	-0.31547	-0.52426	-1.4234	0.02
14615	*ZNF532*	1.0701	-0.42779	-0.59643	-1.0516	0.02
14616	*RIN2*	1.5481	-0.30065	-1.2023	-1.4919	0.02
14617	*BAG4*	0.56844	0.44441	-0.96857	-3.4314	0.01
14618	*C17orf49*	0.79382	0.037116	-0.78705	-1.7493	0.01
14619	*HOGA1*	0.13794	0.03977	-0.1339	-0.13627	0.01
14620	*NOL4*	0.59573	0.13771	-0.68977	-1.3835	0.01
14621	*PHACTR1*	1.2382	-0.43944	-0.75511	-1.5684	0.01

14622	*RUNDC1*	0.68062	0.33652	-0.97509	-1.09	0.01
14623	*C17orf85*	0.24729	0.10075	-0.30608	-1.6437	0.01
14624	*KRT4*	0.38981	0.095647	-0.44353	-1.8033	0.01
14625	*NEMF*	0.22155	0.033945	-0.21385	-0.77748	0.01
14626	*NAT10*	1.4872	-0.54972	-0.89591	-2.0831	0.01
14627	*BRINP2*	0.49944	0.33766	-0.79697	-1.5635	0.01
14628	*DHRS3*	0.96837	0.22275	-1.151	-1.6796	0.01
14629	*HYDIN*	0.40498	0.10266	-0.46762	-1.8654	0.01
14630	*UBE2J2*	1.8962	-0.68405	-1.1726	-1.6707	0.01
14631	*TPSD1*	0.1293	0.044352	-0.13446	-0.65811	0.01
14632	*PRPF19*	0.42931	0.050589	-0.44073	-1.4504	0.01
14633	*C7orf26*	1.6157	-0.16119	-1.4154	-1.6759	0.01
14634	*ALDH1A3*	0.51125	0.46635	-0.93892	-1.175	0.01
14635	*HIST1H1B*	1.255	0.43487	-1.6524	-2.2712	0.01
14636	*OR1I1*	1.2671	-0.08417	-1.1459	-2.1398	0.01
14637	*CMPK2*	0.8875	0.22021	-1.0707	-2.0407	0.01
14638	*USP17L20*	1.2043	-0.48961	-0.67809	-1.8453	0.01
14639	*RNF214*	0.43709	-0.08324	-0.31737	-1.754	0.01
14640	*DEFB107A*	1.2167	0.43888	-1.6206	-3.6516	0.01
14641	*SST*	0.33366	0.2405	-0.53931	-0.85389	0.01
14642	*ZNF563*	0.24777	0.090715	-0.304	-2.0304	0.01
14643	*WBP2*	1.4689	-0.29341	-1.1418	-1.6463	0.01
14644	*ROBO3*	0.30139	-0.0186	-0.24942	-1.534	0.01
14645	*CLEC6A*	0.78288	0.035409	-0.78579	-1.1958	0.01
14646	*ST13*	1.0654	0.079385	-1.1125	-2.0337	0.01
14647	*ZNF219*	1.5319	-0.37116	-1.1287	-2.5007	0.01
14648	*KRT6A*	1.5559	-0.72927	-0.79483	-1.021	0.01
14649	*ARRB2*	1.6876	0.11589	-1.7718	-3.158	0.01
14650	*DNAJB14*	2.1787	-0.30065	-1.8478	-2.2694	0.01
14651	*PACRG*	1.1293	-0.02583	-1.0737	-2.2945	0.01
14652	*MSL2*	1.1344	-0.44374	-0.66107	-1.1194	0.01
14653	*EHHADH*	0.69538	-0.00498	-0.66098	-1.2027	0.01
14654	*CNFN*	0.32496	0.002405	-0.2994	-0.79781	0.01
14655	*TTI2*	0.90948	0.065918	-0.94759	-1.8879	0.01
14656	*CTU2*	2.5528	-1.186	-1.3399	-1.8238	0.01
14657	*DZIP3*	0.62797	0.42261	-1.0248	-1.6613	0.01
14658	*CCDC151*	1.3851	-0.53639	-0.82337	-1.1943	0.01
14659	*ZNF140*	1.6475	0.51174	-2.1342	-2.3525	0.01
14660	*PCDHB11*	3.7826	-1.2399	-2.5178	-2.8594	0.01
14661	*CHAT*	2.5023	-1.0528	-1.4252	-1.9054	0.01
14662	*KIF1B*	1.4624	-0.1507	-1.2876	-2.0277	0.01
14663	*HFE2*	0.26461	-0.00178	-0.23977	-1.2705	0.01
14664	*ZNF766*	1.7084	0.23725	-1.9227	-3.6718	0.01
14665	*ARGFX*	1.4182	-0.11101	-1.2846	-5.7998	0.01
14666	*RBPJL*	1.4916	-0.57301	-0.89699	-2.6438	0.01
14667	*ULK3*	0.47997	0.43264	-0.89219	-1.4066	0.01

14668	*MAP3K4*	0.96682	-0.19128	-0.75523	-1.043	0.01
14669	*C12orf56*	0.12019	0.025299	-0.12565	-0.89699	0.01
14670	*LIG3*	1.0651	-0.05504	-0.9904	-1.9474	0.01
14671	*MFF*	1.6603	-0.01604	-1.6251	-1.7493	0.01
14672	*GPRC5C*	0.71177	-0.27096	-0.42254	-0.78097	0.01
14673	*ZNF438*	0.659	-0.29167	-0.3495	-0.60604	0.01
14674	*RBMXL2*	0.59473	0.2124	-0.78956	-1.2277	0.01
14675	*FMOD*	0.70073	-0.09323	-0.59088	-1.1043	0.01
14676	*CIAO1*	0.67755	0.047789	-0.70961	-1.4108	0.01
14677	*PMPCB*	0.94917	0.16431	-1.098	-1.1018	0.01
14678	*CLK1*	0.46534	-0.11886	-0.33121	-1.768	0.01
14679	*IDH3A*	0.6313	-0.26086	-0.35531	-1.3128	0.01
14680	*CCDC141*	0.73314	0.14487	-0.86403	-1.2135	0.00
14681	*ABAT*	1.1583	-0.14925	-0.99508	-1.3721	0.00
14682	*CKLF-CMTM1*	2.5441	-1.2574	-1.2733	-2.012	0.00
14683	*STAM*	0.66128	-0.09182	-0.55676	-2.5791	0.00
14684	*FRMPD1*	1.0429	-0.21182	-0.81846	-0.96407	0.00
14685	*MAPK7*	1.1163	-0.1931	-0.91096	-1.4401	0.00
14686	*CHD4*	0.73596	-0.08582	-0.638	-1.3253	0.00
14687	*CDC42EP3*	0.39497	-0.14987	-0.23335	-0.42451	0.00
14688	*TBRG4*	1.5101	-0.66577	-0.83278	-1.0087	0.00
14689	*RASD1*	1.4437	-0.15699	-1.2756	-1.5406	0.00
14690	*PTCH1*	0.38252	0.18719	-0.55941	-0.82903	0.00
14691	*THG1L*	0.71281	-0.19272	-0.51038	-3.1719	0.00
14692	*PRR32*	0.5128	0.032842	-0.53666	-0.61779	0.00
14693	*DCST1*	0.083155	0.023698	-0.0981	-2.6122	0.00
14694	*S100A13*	0.43594	0.24242	-0.66973	-1.8724	0.00
14695	*RAPGEF4*	0.59967	-0.13528	-0.45669	-1.2277	0.00
14696	*UBASH3B*	1.154	-0.13746	-1.0092	-1.2879	0.00
14697	*PCSK7*	3.5757	-1.0528	-2.5156	-3.0911	0.00
14698	*F9*	0.97281	0.16308	-1.1298	-2.2589	0.00
14699	*MTTP*	0.63928	-0.08118	-0.55291	-1.6105	0.00
14700	*CDC42*	0.51333	0.048199	-0.55677	-1.676	0.00
14701	*TKTL2*	1.2018	-0.0062	-1.1911	-1.3151	0.00
14702	*SREK1IP1*	2.6018	-0.29454	-2.3036	-3.3146	0.00
14703	*ARHGAP11B*	0.26994	0.094294	-0.36127	-0.76176	0.00
14704	*CLPTM1L*	0.32171	0.26236	-0.58183	-2.3204	0.00
14705	*FAT3*	2.7127	-1.2426	-1.4682	-1.9203	0.00
14706	*FAM13C*	0.99061	0.44717	-1.4368	-1.6525	0.00
14707	*CDK1*	0.78343	0.060939	-0.84348	-1.2267	0.00
14708	*LRIF1*	1.1229	-0.54559	-0.57761	-1.5099	0.00
14709	*CCDC77*	0.67363	0.62691	-1.3016	-1.6855	0.00
14710	*EIF2B1*	1.0563	-0.46752	-0.58994	-1.0452	0.00
14711	*APOBEC3G*	1.0328	-0.26575	-0.76893	-2.2809	0.00
14712	*IGFL4*	0.34227	0.078186	-0.42279	-1.4156	0.00
14713	*LONP2*	1.4416	-0.52017	-0.92427	-1.2692	0.00

14714	*SLC9B2*	0.148	0.068212	-0.21944	-1.3475	0.00
14715	*NAGA*	0.072219	0.010651	-0.08675	-1.3772	0.00
14716	*AGXT*	0.94679	-0.42359	-0.52712	-1.7065	0.00
14717	*DDAH2*	0.79018	0.058283	-0.8525	-1.2608	0.00
14718	*TUT1*	0.60632	-0.18691	-0.42353	-2.043	0.00
14719	*POLR1D*	0.67748	0.66252	-1.3444	-2.1873	0.00
14720	*HIVEP1*	1.1507	0.14587	-1.3016	-1.3457	0.00
14721	*ZNF625*	0.99378	0.34486	-1.3443	-1.7595	0.00
14722	*WNT4*	0.5526	0.35566	-0.91427	-1.2517	0.00
14723	*GOLPH3*	2.0286	-0.92025	-1.1144	-3.2735	0.00
14724	*MLX*	1.2368	0.16326	-1.4062	-1.455	0.00
14725	*GLE1*	0.6979	-0.25181	-0.45305	-2.462	0.00
14726	*SLC30A2*	0.45525	0.29982	-0.76217	-1.2935	0.00
14727	*IGFBP5*	0.74965	-0.22585	-0.53125	-0.72585	0.00
14728	*STC1*	2.0783	-0.81529	-1.2705	-1.7462	0.00
14729	*OR5K3*	0.42128	0.042086	-0.47126	-0.71988	0.00
14730	*RAB10*	0.65119	0.52504	-1.1844	-1.2284	0.00
14731	*LHX5*	2.366	-1.1545	-1.2198	-2.012	0.00
14732	*SULF2*	0.99747	0.4234	-1.4294	-1.8123	0.00
14733	*RIIAD1*	0.5099	-0.19577	-0.32429	-0.96776	0.00
14734	*TRIM26*	1.2501	-0.46303	-0.79871	-1.1619	0.00
14735	*ATP2A2*	1.3233	-0.21646	-1.12	-1.2008	0.00
14736	*WLS*	0.6799	0.15374	-0.8471	-1.3739	0.00
14737	*ISG20*	1.3834	-0.35232	-1.0451	-1.5959	0.00
14738	*RNF41*	0.31222	-0.00082	-0.32546	-0.7757	0.00
14739	*GRB14*	0.93429	0.086818	-1.0355	-1.9533	0.00
14740	*DSG3*	1.0898	0.45486	-1.5597	-1.9997	-0.01
14741	*FAM65B*	0.99081	0.45639	-1.4625	-1.962	-0.01
14742	*SRBD1*	1.1596	0.29867	-1.4745	-1.7106	-0.01
14743	*NREP*	0.65314	0.027969	-0.69866	-1.5306	-0.01
14744	*FAM177B*	0.45381	0.11774	-0.58929	-1.4269	-0.01
14745	*FAM149A*	0.72553	-0.04769	-0.69631	-0.73301	-0.01
14746	*HES2*	0.78428	-0.38041	-0.42291	-3.6398	-0.01
14747	*CRIPAK*	1.0913	-0.04621	-1.0651	-1.4405	-0.01
14748	*SNAPC1*	0.37234	0.20442	-0.59683	-1.165	-0.01
14749	*ATP10D*	0.57587	0.1072	-0.70325	-1.1212	-0.01
14750	*C2orf15*	0.65987	0.63624	-1.3165	-2.4815	-0.01
14751	*FAM32A*	1.7159	-0.36751	-1.3689	-2.9844	-0.01
14752	*RPL9*	1.0105	-0.17037	-0.86157	-1.6176	-0.01
14753	*TFG*	1.198	-0.43376	-0.78591	-1.2171	-0.01
14754	*ZNF396*	1.7537	-0.64712	-1.1297	-2.8647	-0.01
14755	*GDF6*	0.92	0.3642	-1.3085	-1.4116	-0.01
14756	*LCE3A*	0.78876	-0.20552	-0.60754	-1.5393	-0.01
14757	*LGALS3BP*	1.9009	-0.77462	-1.1511	-1.7782	-0.01
14758	*GRAMD4*	0.67882	0.22058	-0.92435	-3.2735	-0.01
14759	*ACSBG1*	0.77909	0.1452	-0.95136	-1.7065	-0.01

14760	*TAF5*	1.8424	-0.72201	-1.1479	-1.2837	-0.01
14761	*ENY2*	0.49122	0.27132	-0.79043	-1.7646	-0.01
14762	*APOL2*	1.0371	-0.09807	-0.96718	-1.7804	-0.01
14763	*C17orf82*	0.68847	-0.17425	-0.54259	-0.9653	-0.01
14764	*TIAL1*	0.028715	-0.02128	-0.03686	-0.36749	-0.01
14765	*ACTL7B*	1.8741	0.35451	-2.2586	-2.5934	-0.01
14766	*NPBWR2*	0.42752	-0.22094	-0.23698	-0.32486	-0.01
14767	*GPR179*	1.235	0.091878	-1.3573	-1.5049	-0.01
14768	*C22orf23*	2.2684	-0.94364	-1.3556	-1.5238	-0.01
14769	*ITPKA*	1.0131	-0.49846	-0.54555	-2.2413	-0.01
14770	*REXO2*	0.7376	0.051512	-0.8203	-2.0495	-0.01
14771	*LTK*	0.43421	-0.02298	-0.44299	-2.1572	-0.01
14772	*SLC39A9*	2.5321	-1.1631	-1.4018	-1.4168	-0.01
14773	*SERHL2*	1.2509	-0.08975	-1.1956	-1.2867	-0.01
14774	*CT47A7*	1.094	-0.0975	-1.0318	-1.0995	-0.01
14775	*ZNF282*	0.28522	0.12282	-0.44353	-0.74011	-0.01
14776	*SPAG5*	1.5418	-0.75529	-0.82211	-1.4359	-0.01
14777	*ALG1L2*	0.46625	0.2635	-0.7655	-0.91981	-0.01
14778	*EIF2S2*	0.43228	-0.02447	-0.44575	-1.8014	-0.01
14779	*FUS*	0.44132	0.005506	-0.4854	-1.984	-0.01
14780	*VGLL1*	0.12471	-0.05585	-0.10828	-1.1056	-0.01
14781	*BIN1*	1.3259	-0.21438	-1.1511	-1.1817	-0.01
14782	*CXorf49B*	1.7635	-0.38011	-1.4233	-1.7874	-0.01
14783	*ZNF274*	1.7172	-0.80853	-0.94912	-1.8698	-0.01
14784	*ZBTB17*	0.51199	0.2015	-0.75448	-1.1703	-0.01
14785	*DUSP2*	2.5732	-1.3057	-1.3089	-2.0778	-0.01
14786	*PLK2*	0.32713	0.049777	-0.4187	-1.4824	-0.01
14787	*SNRNP27*	1.4316	-0.52943	-0.94441	-1.9867	-0.01
14788	*CENPA*	0.93194	0.25563	-1.2302	-1.8275	-0.01
14789	*DRAM2*	0.71415	-0.31808	-0.43886	-1.2719	-0.01
14790	*LILRB1*	0.34228	0.18739	-0.57397	-1.2863	-0.01
14791	*ARL6IP1*	2.5601	-0.8988	-1.7056	-2.508	-0.01
14792	*HTR6*	1.2206	-0.00111	-1.2655	-2.2888	-0.02
14793	*RUNDC3A*	1.3806	-0.62548	-0.80146	-1.1169	-0.02
14794	*SCYL3*	1.24	-0.54847	-0.73787	-1.6759	-0.02
14795	*NUP133*	1.2206	-0.22301	-1.0441	-1.0947	-0.02
14796	*VRK2*	0.76478	-0.2765	-0.53584	-2.2727	-0.02
14797	*LRRTM1*	1.3602	-0.47338	-0.93533	-1.9504	-0.02
14798	*LPGAT1*	1.0285	-0.06364	-1.0149	-2.4655	-0.02
14799	*OR14J1*	0.47742	-0.01371	-0.51494	-1.951	-0.02
14800	*CLCNKB*	1.2851	-0.24797	-1.0884	-1.2952	-0.02
14801	*DMRTC1B*	0.69499	0.50317	-1.25	-3.034	-0.02
14802	*PCMT1*	0.65144	-0.0391	-0.66543	-1.7879	-0.02
14803	*LIN54*	0.13779	0.019567	-0.21094	-0.56281	-0.02
14804	*CCKBR*	0.44805	0.38467	-0.8865	-2.6438	-0.02
14805	*PNLIPRP3*	0.32118	0.097548	-0.47353	-1.5375	-0.02

14806	*TUBB*	1.8727	-0.40534	-1.5225	-1.7438	-0.02
14807	*SATB2*	2.1932	-0.80405	-1.4454	-1.7513	-0.02
14808	*CAPNS1*	1.2993	-0.35111	-1.0047	-1.5911	-0.02
14809	*MMP14*	2.4502	-1.209	-1.2981	-2.674	-0.02
14810	*MRPL22*	0.60896	-0.09025	-0.57564	-1.3808	-0.02
14811	*NIPA1*	0.32813	0.13256	-0.51768	-0.91256	-0.02
14812	*FIGF*	0.73147	0.72314	-1.5125	-4.1058	-0.02
14813	*SYNJ2*	1.0963	-0.25792	-0.89641	-1.6437	-0.02
14814	*PC*	0.34077	0.28036	-0.6794	-1.6796	-0.02
14815	*BAG1*	0.94387	0.21191	-1.2141	-1.5971	-0.02
14816	*PVRL2*	0.31943	-0.17936	-0.19851	-1.3607	-0.02
14817	*EGF*	0.78469	0.21634	-1.0606	-1.1564	-0.02
14818	*WDR11*	0.34909	-0.117	-0.29209	-0.87673	-0.02
14819	*ATP8B4*	0.89321	0.45901	-1.4129	-2.2014	-0.02
14820	*EDDM3A*	0.27161	0.033893	-0.36679	-2.3405	-0.02
14821	*VAT1L*	2.1442	-1.0841	-1.122	-1.621	-0.02
14822	*OR6C6*	1.5552	0.75789	-2.3751	-2.6275	-0.02
14823	*PPP1R13L*	2.1076	-0.75542	-1.4142	-2.1662	-0.02
14824	*ZNF655*	1.4863	-0.47642	-1.0726	-1.1144	-0.02
14825	*OR2V2*	1.828	-0.59762	-1.2936	-1.794	-0.02
14826	*MED21*	1.0724	0.15924	-1.296	-1.3941	-0.02
14827	*TNFAIP8*	0.83068	0.78944	-1.6847	-2.0042	-0.02
14828	*SDF4*	2.1613	-0.88784	-1.3382	-1.9251	-0.02
14829	*POTEC*	0.28017	0.20131	-0.54651	-1.0196	-0.02
14830	*MED4*	0.5892	0.067247	-0.72204	-1.4829	-0.02
14831	*OLAH*	0.38955	0.29533	-0.7513	-1.8478	-0.02
14832	*CEP295*	0.60384	0.049015	-0.72085	-2.3452	-0.02
14833	*CYB5A*	0.46502	-0.22921	-0.30444	-1.4962	-0.02
14834	*NBEAL1*	2.1538	-1.0191	-1.2036	-1.7745	-0.02
14835	*QPCT*	0.99477	-0.1227	-0.94341	-2.1921	-0.02
14836	*PCNX*	0.19463	0.14163	-0.40823	-0.45193	-0.02
14837	*IL2*	1.415	-0.02583	-1.4614	-1.8016	-0.02
14838	*FAM102B*	1.2419	-0.1672	-1.1472	-2.3357	-0.02
14839	*WIPI1*	3.0215	-1.5434	-1.5508	-1.6575	-0.02
14840	*PRAMEF12*	0.56421	0.50072	-1.1381	-1.9162	-0.02
14841	*CAMTA2*	1.2244	0.72209	-2.0197	-3.1317	-0.02
14842	*SLC52A1*	0.68744	0.63689	-1.3978	-1.4364	-0.02
14843	*CCDC9*	2.9212	-1.3492	-1.6456	-2.2533	-0.02
14844	*TMEM47*	1.5302	-0.4521	-1.1526	-1.3479	-0.02
14845	*FCHO2*	0.58849	0.46699	-1.1309	-1.3668	-0.03
14846	*CCR9*	1.4003	-0.31475	-1.161	-1.3835	-0.03
14847	*RBKS*	1.1724	-0.3464	-0.90281	-1.8219	-0.03
14848	*DUSP7*	0.49996	-0.09268	-0.48416	-2.6289	-0.03
14849	*PIGM*	1.0291	-0.15649	-0.94954	-2.0885	-0.03
14850	*GGNBP2*	1.168	-0.59921	-0.64725	-1.1133	-0.03
14851	*NDUFS5*	1.1356	0.37555	-1.5898	-1.7328	-0.03

14852	*LEF1*	1.0764	-0.11897	-1.0364	-1.1207	-0.03
14853	*ATP5S*	1.0545	-0.2345	-0.89931	-0.97751	-0.03
14854	*TUBGCP2*	1.3732	-0.51182	-0.94255	-1.6098	-0.03
14855	*NUDCD1*	0.66316	0.091533	-0.83627	-1.2708	-0.03
14856	*NPIPB6*	1.7562	-0.04486	-1.7935	-1.8941	-0.03
14857	*SLC25A51*	0.761	0.42143	-1.2646	-2.5092	-0.03
14858	*GTF2H2*	1.3786	-0.19804	-1.2633	-1.8508	-0.03
14859	*PFKM*	1.048	0.39428	-1.5271	-1.7493	-0.03
14860	*FAM9B*	0.46477	0.27747	-0.82757	-2.0851	-0.03
14861	*SLC2A13*	0.79121	0.68177	-1.5588	-1.9041	-0.03
14862	*AVPR1B*	0.54927	-0.15424	-0.48144	-0.9925	-0.03
14863	*EFNA2*	0.72307	0.001035	-0.81087	-0.90492	-0.03
14864	*DOCK1*	0.99511	-0.50067	-0.58167	-2.6574	-0.03
14865	*TMED10*	2.5182	-0.56411	-2.0414	-2.1404	-0.03
14866	*TSPAN7*	0.52471	0.19941	-0.81152	-0.9497	-0.03
14867	*USH1G*	0.19327	-0.13962	-0.14151	-1.1685	-0.03
14868	*KIAA1456*	0.86582	0.4223	-1.3766	-1.6208	-0.03
14869	*CLEC4G*	0.56668	-0.19804	-0.46107	-0.78229	-0.03
14870	*MRPL54*	0.91172	0.50373	-1.5079	-1.5441	-0.03
14871	*EFNA3*	1.6122	-0.52372	-1.1817	-1.3153	-0.03
14872	*NAAA*	0.096469	-0.02812	-0.16267	-1.2755	-0.03
14873	*B3GAT3*	0.63535	0.24537	-0.97549	-1.8392	-0.03
14874	*OR5B2*	-0.01948	-0.02845	-0.04799	-0.8202	-0.03
14875	*SEC14L6*	0.55574	0.04489	-0.69665	-0.9416	-0.03
14876	*GPR78*	0.55507	0.55011	-1.2052	-1.8478	-0.03
14877	*SLCO1A2*	1.4515	-0.03643	-1.5153	-1.5959	-0.03
14878	*PSMC2*	1.3028	-0.09354	-1.3101	-1.595	-0.03
14879	*RAB33B*	1.0007	0.28	-1.386	-1.5144	-0.04
14880	*ADAT2*	0.35613	0.13367	-0.59516	-1.0273	-0.04
14881	*ASB14*	0.39528	-0.15876	-0.34227	-1.162	-0.04
14882	*GFM2*	1.0049	-0.05804	-1.0529	-1.4306	-0.04
14883	*P2RX2*	0.76989	-0.43264	-0.44417	-0.46728	-0.04
14884	*NFKBIB*	1.9694	-0.76389	-1.3141	-1.8879	-0.04
14885	*OR51I1*	1.0666	0.33336	-1.5099	-1.6199	-0.04
14886	*MTA3*	0.5998	0.43552	-1.146	-2.4221	-0.04
14887	*FOLH1B*	0.4558	-0.22675	-0.34266	-2.1616	-0.04
14888	*RPL7*	1.3935	-0.09179	-1.416	-2.4761	-0.04
14889	*HNRNPCL4*	3.3203	-1.4878	-1.9474	-2.0538	-0.04
14890	*TMEM63C*	0.83881	-0.18135	-0.77317	-1.0036	-0.04
14891	*FOXD4*	1.392	0.36759	-1.8759	-2.088	-0.04
14892	*MMP24*	0.74195	-0.22886	-0.62952	-1.5018	-0.04
14893	*MAP7*	0.013119	-0.02646	-0.10392	-0.89052	-0.04
14894	*THSD4*	0.43376	0.14764	-0.69904	-2.6072	-0.04
14895	*EBF2*	0.19645	0.051282	-0.36549	-0.84858	-0.04
14896	*IL6ST*	0.62542	0.12351	-0.86802	-1.2987	-0.04
14897	*WDTC1*	3.4776	-1.63	-1.9678	-2.184	-0.04

14898	*ICAM4*	0.90187	-0.32409	-0.69826	-1.4762	-0.04
14899	*SERPINH1*	1.9196	-0.87792	-1.1632	-5.6834	-0.04
14900	*UBR4*	1.9274	-0.50282	-1.5461	-1.7643	-0.04
14901	*BCL9L*	0.63121	0.30293	-1.0557	-1.8443	-0.04
14902	*FAM177A1*	0.55698	0.25098	-0.92975	-1.677	-0.04
14903	*PRLH*	0.24928	0.016392	-0.38786	-2.3222	-0.04
14904	*N4BP1*	1.0263	-0.06763	-1.0809	-1.9341	-0.04
14905	*SEMA5A*	0.52683	-0.23158	-0.41754	-1.191	-0.04
14906	*SLC35G4*	0.30472	-0.20338	-0.22422	-2.2165	-0.04
14907	*GRIK4*	0.94035	0.14156	-1.2051	-1.8311	-0.04
14908	*PIK3R3*	0.94956	-0.50978	-0.56453	-0.71443	-0.04
14909	*RUSC1-AS1*	0.43282	0.16218	-0.71986	-1.2981	-0.04
14910	*NME1-NME2*	1.3114	-0.56947	-0.86698	-1.805	-0.04
14911	*DDX27*	0.75279	-0.00014	-0.87772	-1.041	-0.04
14912	*PIFO*	1.4121	-0.568	-0.96962	-1.3478	-0.04
14913	*TBX18*	0.81325	-0.15845	-0.78229	-1.3118	-0.04
14914	*SUN3*	1.2353	-0.56501	-0.79798	-1.6208	-0.04
14915	*CCL19*	2.4574	-1.1538	-1.4317	-1.4684	-0.04
14916	*FAM71E1*	2.3135	-1.1358	-1.3058	-1.8589	-0.04
14917	*NDUFB3*	0.39772	-0.25839	-0.2681	-1.8408	-0.04
14918	*FAM198A*	1.0962	-0.08842	-1.1367	-1.151	-0.04
14919	*UNC13A*	1.3712	-0.2168	-1.2845	-2.0267	-0.04
14920	*MBNL3*	0.12285	-0.07106	-0.18283	-2.3287	-0.04
14921	*UGT2B17*	0.58992	0.04816	-0.76966	-2.0452	-0.04
14922	*ELL2*	0.81997	0.11634	-1.0684	-1.6583	-0.04
14923	*BMPR1A*	0.62055	-0.04678	-0.70591	-2.1498	-0.04
14924	*CFD*	0.87281	0.55577	-1.5608	-1.8157	-0.04
14925	*SDHB*	1.0265	0.40799	-1.5671	-2.3033	-0.04
14926	*FKBP7*	1.0043	-0.3706	-0.76671	-1.9107	-0.04
14927	*ODF2*	0.55442	-0.25608	-0.43251	-0.86123	-0.04
14928	*INA*	2.8623	-1.236	-1.761	-2.1351	-0.04
14929	*NSG1*	-0.02773	-0.04439	-0.06468	-1.6368	-0.05
14930	*CLIC5*	3.142	-1.5608	-1.7188	-1.8816	-0.05
14931	*PCK2*	0.44551	-0.01337	-0.56982	-1.7536	-0.05
14932	*GGCX*	1.7281	-0.2263	-1.6395	-1.6447	-0.05
14933	*FOXF1*	1.857	-0.78718	-1.2079	-2.0303	-0.05
14934	*SLC25A10*	0.62692	0.063324	-0.82844	-1.1156	-0.05
14935	*NPIPA2*	2.2716	-0.88666	-1.5232	-1.6887	-0.05
14936	*GALNT13*	0.29415	0.079836	-0.51266	-2.5081	-0.05
14937	*PADI2*	2.0414	-0.73861	-1.4416	-1.783	-0.05
14938	*BRMS1*	0.3521	-0.04751	-0.44353	-0.82628	-0.05
14939	*MPHOSPH10*	0.46987	0.44142	-1.0503	-1.9065	-0.05
14940	*ITPKC*	2.4244	-0.98104	-1.5824	-2.5477	-0.05
14941	*LOXL4*	0.44105	-0.00058	-0.58011	-0.87169	-0.05
14942	*BRAF*	0.62527	-0.00178	-0.76358	-1.3604	-0.05
14943	*C17orf100*	1.1123	0.60243	-1.855	-1.9052	-0.05

14944	*MYLK4*	1.0269	-0.57546	-0.59183	-0.9295	-0.05
14945	*ZAP70*	0.088704	0.07582	-0.30559	-0.58553	-0.05
14946	*MSH5*	0.94448	-0.23146	-0.85409	-1.4567	-0.05
14947	*MST1*	0.9593	0.080148	-1.181	-1.2903	-0.05
14948	*MITD1*	0.55639	-0.27486	-0.42439	-2.051	-0.05
14949	*TRPV2*	1.062	-0.22082	-0.98459	-1.5214	-0.05
14950	*CHSY3*	0.51174	0.046264	-0.70171	-0.85093	-0.05
14951	*NDUFV2*	1.9204	-0.50535	-1.559	-2.7068	-0.05
14952	*FAM134B*	0.36473	0.3053	-0.81524	-2.2599	-0.05
14953	*LDLRAD3*	2.428	-1.2193	-1.3542	-1.6726	-0.05
14954	*BCL2L12*	1.4618	-0.12866	-1.4789	-2.0068	-0.05
14955	*EFEMP1*	0.62329	-0.13183	-0.63746	-1.7407	-0.05
14956	*BRMS1L*	1.479	-0.73739	-0.88775	-1.2426	-0.05
14957	*AAGAB*	1.5222	-0.44136	-1.227	-1.4683	-0.05
14958	*GPR160*	2.2605	-1.0175	-1.3892	-1.9446	-0.05
14959	*TBCK*	1.622	-0.65178	-1.1169	-2.4322	-0.05
14960	*ADGRF5*	0.70886	-0.10116	-0.75487	-1.5914	-0.05
14961	*SPG20*	0.79589	0.75574	-1.6989	-2.6267	-0.05
14962	*MGAT2*	0.40783	-0.1741	-0.38161	-0.9733	-0.05
14963	*TSPY4*	0.29118	-0.12786	-0.31121	-0.9285	-0.05
14964	*RETNLB*	0.95717	-0.42671	-0.67853	-1.083	-0.05
14965	*ATP5I*	1.0066	-0.43176	-0.72334	-1.0773	-0.05
14966	*TOE1*	0.42787	-0.02705	-0.55002	-1.0019	-0.05
14967	*HPCAL4*	1.8636	-0.87647	-1.1365	-1.3784	-0.05
14968	*BEND7*	0.19351	-0.00823	-0.33468	-0.87208	-0.05
14969	*NDRG1*	1.7573	-0.62531	-1.2815	-1.5738	-0.05
14970	*ANAPC5*	3.005	-1.2568	-1.8982	-2.3145	-0.05
14971	*TMEM110-MUSTN1*	0.56292	-0.23957	-0.47447	-3.1051	-0.05
14972	*SMO*	1.3287	-0.30715	-1.1727	-1.4087	-0.05
14973	*DPEP1*	0.41563	0.044921	-0.61302	-1.1075	-0.05
14974	*SHARPIN*	0.90913	-0.43488	-0.62679	-1.3411	-0.05
14975	*NFKBIL1*	1.6469	-0.55281	-1.2473	-1.5996	-0.05
14976	*PCF11*	0.10688	0.042515	-0.30371	-1.7646	-0.05
14977	*ELF3*	1.1061	-0.37976	-0.88278	-1.8033	-0.05
14978	*ATG5*	1.6043	-0.47964	-1.2815	-1.8376	-0.05
14979	*ANKRD40*	0.055485	-0.09689	-0.11607	-1.3116	-0.05
14980	*COX10*	0.33406	-0.2441	-0.24812	-1.337	-0.05
14981	*ZNF195*	1.002	-0.21576	-0.94466	-1.5244	-0.05
14982	*LOC441155*	0.80359	-0.00392	-0.95893	-1.7991	-0.05
14983	*GDF1*	1.7453	-0.81119	-1.0935	-1.6527	-0.05
14984	*CMTM1*	1.0588	-0.52187	-0.6972	-1.1316	-0.05
14985	*WDR64*	0.88035	-0.19054	-0.85027	-1.7793	-0.05
14986	*NLRP8*	0.54	-0.22803	-0.47412	-2.2319	-0.05
14987	*RFX4*	0.60831	0.18363	-0.95461	-2.1354	-0.05
14988	*SMIM23*	0.33545	0.18216	-0.68109	-1.9038	-0.05
14989	*DAG1*	0.046128	0.006186	-0.21632	-0.5442	-0.05

14990	*QRICH1*	1.682	0.19205	-2.0384	-2.4014	-0.05
14991	*PIWIL4*	0.50535	-0.16295	-0.50685	-1.2234	-0.05
14992	*CDKN2D*	0.67487	0.040706	-0.88098	-1.0088	-0.06
14993	*DFFA*	0.50243	0.1989	-0.86744	-1.0496	-0.06
14994	*ICE1*	0.87021	0.4185	-1.4553	-1.5558	-0.06
14995	*MORC2*	0.080715	0.049826	-0.29794	-0.80146	-0.06
14996	*TMEM119*	3.0987	-1.5415	-1.7247	-2.2059	-0.06
14997	*C14orf1*	1.3406	0.66952	-2.1792	-2.8125	-0.06
14998	*SNAPC3*	1.001	0.026876	-1.197	-1.4811	-0.06
14999	*ADD1*	0.62071	-0.21398	-0.57732	-0.65951	-0.06
15000	*DIRC2*	0.65962	-0.25794	-0.57307	-1.0413	-0.06
15001	*BOP1*	0.027623	-0.06199	-0.13737	-2.8647	-0.06
15002	*RSAD1*	0.86236	-0.48808	-0.54639	-2.2074	-0.06
15003	*UBE2G2*	1.1169	0.44095	-1.7307	-1.8954	-0.06
15004	*GPRIN3*	1.2466	-0.23878	-1.181	-1.677	-0.06
15005	*RBL1*	1.0747	0.078551	-1.3268	-1.481	-0.06
15006	*CHERP*	0.84974	0.031869	-1.0557	-1.2774	-0.06
15007	*ATCAY*	0.74321	-0.12418	-0.79319	-1.5235	-0.06
15008	*ZNF786*	0.64233	0.18334	-0.9999	-1.9107	-0.06
15009	*SLC29A2*	0.47467	-0.01574	-0.63336	-1.2837	-0.06
15010	*SBNO2*	1.2562	-0.69495	-0.73814	-1.7606	-0.06
15011	*GMNN*	0.19259	0.008446	-0.37913	-0.95505	-0.06
15012	*ORC1*	0.68288	0.16141	-1.0224	-1.365	-0.06
15013	*ADARB1*	0.76977	-0.15882	-0.78916	-1.2639	-0.06
15014	*GOLGA8A*	2.1325	-1.1321	-1.1796	-1.9772	-0.06
15015	*SSX4B*	0.79248	0.5137	-1.4856	-1.9413	-0.06
15016	*AGTPBP1*	0.54804	0.13633	-0.86384	-0.89052	-0.06
15017	*SETD2*	0.6959	0.041702	-0.91721	-1.3542	-0.06
15018	*ERMN*	0.85911	-0.14132	-0.89866	-1.1585	-0.06
15019	*C12orf65*	0.41594	0.068299	-0.66535	-1.2159	-0.06
15020	*TEF*	0.37182	-0.22131	-0.33191	-2.4328	-0.06
15021	*ATP5F1*	0.44354	0.34162	-0.96718	-1.4633	-0.06
15022	*SMC5*	0.91486	0.40783	-1.5054	-1.9423	-0.06
15023	*SLC25A16*	0.51375	-0.30086	-0.39565	-1.0952	-0.06
15024	*PLCL2*	1.3087	-0.04729	-1.445	-1.7209	-0.06
15025	*IL6R*	0.53319	0.004026	-0.72128	-1.0863	-0.06
15026	*CNOT7*	1.1055	-0.30481	-0.98574	-2.1105	-0.06
15027	*FTSJ2*	0.5938	-0.33681	-0.44353	-2.1264	-0.06
15028	*BPY2*	0.11287	-0.01371	-0.28664	-0.43226	-0.06
15029	*HMGCS2*	1.252	0.18147	-1.6213	-1.9965	-0.06
15030	*CTDSPL2*	0.55445	0.088102	-0.83138	-2.9144	-0.06
15031	*CHD7*	2.1798	-0.98314	-1.386	-1.433	-0.06
15032	*RPP14*	0.08223	0.005713	-0.27741	-1.3136	-0.06
15033	*ENPEP*	0.33952	0.041238	-0.57134	-0.94019	-0.06
15034	*COX6C*	1.1086	0.10557	-1.4048	-1.9319	-0.06
15035	*CCL4L2*	0.71476	0.42398	-1.3294	-1.7567	-0.06

15036	*HMSD*	0.87394	-0.34751	-0.71717	-1.9686	-0.06
15037	*SH3GL2*	0.86616	-0.16781	-0.8903	-2.056	-0.06
15038	*RNF5*	1.092	-0.37282	-0.91165	-1.0231	-0.06
15039	*PRRT3*	1.5666	-0.25527	-1.5045	-1.6744	-0.06
15040	*ZNRF2*	1.2796	-0.62476	-0.84816	-1.0413	-0.06
15041	*PRR35*	1.7432	-0.60873	-1.3282	-4.0419	-0.06
15042	*MOGS*	0.2824	0.067594	-0.54373	-1.6799	-0.06
15043	*OR13F1*	0.59297	0.38037	-1.1673	-1.2573	-0.06
15044	*ANKIB1*	1.5516	-0.7329	-1.0131	-1.2075	-0.06
15045	*ABCC11*	0.4315	-0.06413	-0.56185	-1.0868	-0.06
15046	*GAS1*	0.48879	0.22714	-0.9135	-2.4405	-0.07
15047	*PLEKHS1*	0.7492	0.18854	-1.1357	-1.3517	-0.07
15048	*PRR21*	0.71677	-0.2694	-0.64616	-1.4162	-0.07
15049	*CTDSP2*	0.31201	-0.10258	-0.40824	-1.4557	-0.07
15050	*ZW10*	1.9286	-0.69377	-1.434	-3.2632	-0.07
15051	*SNCA*	3.5226	-1.4842	-2.2392	-2.6998	-0.07
15052	*RANBP1*	0.91767	-0.30377	-0.81539	-1.0838	-0.07
15053	*CX3CL1*	0.38185	-0.26851	-0.3153	-1.6597	-0.07
15054	*MRPS10*	0.28412	0.22321	-0.70976	-0.86435	-0.07
15055	*C5orf51*	0.34126	0.1049	-0.64918	-1.1043	-0.07
15056	*GRIN1*	0.80251	0.26279	-1.2694	-1.6086	-0.07
15057	*PEX13*	0.59762	0.29709	-1.0992	-1.6193	-0.07
15058	*MAP6*	0.47543	0.18317	-0.86358	-1.5165	-0.07
15059	*CDC42EP4*	0.89127	-0.34481	-0.75148	-1.3961	-0.07
15060	*NUMBL*	0.78709	-0.39466	-0.5975	-2.262	-0.07
15061	*SNX17*	0.26759	0.076527	-0.54921	-2.1868	-0.07
15062	*FAM171B*	0.82613	-0.32766	-0.70361	-0.87768	-0.07
15063	*CGNL1*	0.31246	0.11308	-0.63099	-1.1811	-0.07
15064	*BLVRB*	1.8761	-0.6886	-1.3935	-1.4852	-0.07
15065	*UGT1A10*	0.080441	0.053631	-0.34036	-0.60223	-0.07
15066	*FAM65C*	1.5207	-0.80613	-0.92108	-1.4711	-0.07
15067	*DMXL2*	0.71448	-0.24491	-0.6761	-1.8583	-0.07
15068	*GPR85*	0.72458	0.5681	-1.4997	-2.0134	-0.07
15069	*GS1-259H13.2*	2.7899	-1.3445	-1.6525	-3.2901	-0.07
15070	*KIR2DL3*	0.71252	-0.44353	-0.47676	-1.0243	-0.07
15071	*ZNF664-FAM101A*	1.1095	-0.58732	-0.73061	-1.2291	-0.07
15072	*LOC388813*	0.26768	-0.07545	-0.40136	-3.1458	-0.07
15073	*ISLR2*	0.43022	0.1422	-0.78236	-2.6376	-0.07
15074	*SLC22A4*	0.58319	-0.17641	-0.61829	-1.5121	-0.07
15075	*ACTR3C*	0.5122	-0.30811	-0.41596	-1.7829	-0.07
15076	*TMEM89*	2.7382	-1.4364	-1.5138	-1.6731	-0.07
15077	*LHFPL2*	0.63165	-0.34844	-0.49541	-1.4781	-0.07
15078	*TBC1D22A*	0.1448	0.048765	-0.40605	-1.6976	-0.07
15079	*GOLGA5*	0.43491	-0.20988	-0.43812	-0.48862	-0.07
15080	*ZNF618*	1.0547	-0.0875	-1.1812	-1.9874	-0.07
15081	*NF1*	1.0717	0.4782	-1.765	-1.8851	-0.07

15082	*APLN*	0.42648	0.38127	-1.0231	-3.0974	-0.07
15083	*PASD1*	0.57355	-0.35436	-0.43481	-1.0041	-0.07
15084	*LONP1*	1.0512	0.32015	-1.587	-1.9751	-0.07
15085	*OR13G1*	1.588	-0.07719	-1.7268	-1.9623	-0.07
15086	*CTPS1*	0.81708	-0.11965	-0.9135	-2.1868	-0.07
15087	*ZNF234*	0.1834	0.12753	-0.52712	-1.5261	-0.07
15088	*SLC39A10*	0.43235	-0.14762	-0.50151	-1.7599	-0.07
15089	*ACLY*	2.6327	-1.1566	-1.6934	-2.4268	-0.07
15090	*HS3ST3A1*	0.57921	0.52817	-1.325	-2.4718	-0.07
15091	*NOG*	0.21925	-0.07913	-0.35847	-1.3052	-0.07
15092	*CARHSP1*	1.2168	-0.12145	-1.3139	-1.6617	-0.07
15093	*VTI1B*	1.1106	-0.52086	-0.80847	-1.1773	-0.07
15094	*OPN3*	0.79239	-0.47197	-0.54048	-1.8087	-0.07
15095	*CYP4Z1*	2.3655	-1.1825	-1.4036	-2.6539	-0.07
15096	*INTS2*	0.68273	0.078551	-0.98241	-1.6836	-0.07
15097	*DCUN1D3*	1.9489	-0.84134	-1.3288	-1.4747	-0.07
15098	*SYTL3*	0.99435	-0.23596	-0.97982	-1.2628	-0.07
15099	*FCGR1B*	0.35584	0.043375	-0.62114	-0.98026	-0.07
15100	*C14orf169*	0.48777	0.001501	-0.71134	-2.0871	-0.07
15101	*ZNF599*	0.56052	0.42287	-1.2055	-1.7103	-0.07
15102	*PLCG2*	0.82075	-0.23446	-0.80863	-1.879	-0.07
15103	*ZC3H7A*	1.5201	-0.69146	-1.0528	-1.6604	-0.07
15104	*UGP2*	0.17582	-0.19925	-0.20112	-1.0158	-0.07
15105	*C19orf48*	1.5504	-0.77241	-1.0029	-3.0112	-0.07
15106	*VPS37A*	0.73177	0.58103	-1.5379	-1.7161	-0.08
15107	*TOPBP1*	0.33184	-0.01181	-0.54515	-1.4893	-0.08
15108	*APOL6*	1.9126	-0.4015	-1.7364	-2.1282	-0.08
15109	*ALG3*	0.31889	0.052562	-0.59886	-0.9225	-0.08
15110	*DHPS*	0.51482	0.084092	-0.82656	-1.7313	-0.08
15111	*ZBTB48*	0.43842	-0.04502	-0.6218	-1.1978	-0.08
15112	*GPR19*	0.17052	0.13779	-0.53924	-0.75625	-0.08
15113	*TGIF2-C20orf24*	0.41442	0.35438	-0.9999	-1.205	-0.08
15114	*GFRA4*	2.4052	-1.1866	-1.4498	-2.8941	-0.08
15115	*TSPYL4*	0.76544	0.04383	-1.0416	-1.4428	-0.08
15116	*ZNF514*	0.89005	-0.16737	-0.95614	-1.1834	-0.08
15117	*OLFM2*	0.60849	-0.10743	-0.73457	-1.806	-0.08
15118	*RBM8A*	0.62197	-0.27961	-0.57683	-1.3507	-0.08
15119	*ADAM17*	0.24949	0.003285	-0.48862	-1.6403	-0.08
15120	*GLRA3*	0.29943	-0.15141	-0.38428	-1.843	-0.08
15121	*RAD23A*	0.065047	-0.07885	-0.22258	-0.41395	-0.08
15122	*ATXN1*	2.5729	-1.0153	-1.7948	-1.8487	-0.08
15123	*ABCA5*	1.826	-0.9703	-1.0933	-1.4732	-0.08
15124	*SOWAHC*	0.53039	-0.07903	-0.68934	-2.5386	-0.08
15125	*F13B*	0.79289	-0.05643	-0.97463	-1.3559	-0.08
15126	*C21orf140*	0.7952	-0.50467	-0.52949	-1.0741	-0.08
15127	*BTBD17*	0.49337	0.019393	-0.75182	-0.87482	-0.08

15128	*GRM1*	0.76313	-0.4818	-0.5204	-0.70993	-0.08
15129	*EMC9*	0.64312	0.43983	-1.3232	-2.3689	-0.08
15130	*GPR174*	0.31574	0.12351	-0.67962	-1.2284	-0.08
15131	*BAIAP2L1*	0.49197	0.3488	-1.082	-2.8723	-0.08
15132	*PRKCDBP*	1.0174	-0.34148	-0.91757	-2.2804	-0.08
15133	*HLA-DMB*	0.93166	-0.02022	-1.1533	-1.716	-0.08
15134	*RAB25*	0.3295	-0.10392	-0.46765	-0.6386	-0.08
15135	*ZFP36L1*	0.69631	0.09276	-1.0312	-1.6047	-0.08
15136	*KIF1BP*	1.9814	-0.91307	-1.3114	-2.7042	-0.08
15137	*SNF8*	0.66487	-0.20592	-0.70202	-1.9318	-0.08
15138	*CDK13*	0.61343	0.11542	-0.97252	-2.8111	-0.08
15139	*HEMGN*	1.184	-0.27555	-1.1533	-1.2684	-0.08
15140	*MAPK8IP3*	0.74223	0.03422	-1.0218	-1.4885	-0.08
15141	*YIF1B*	0.62371	0.071151	-0.94121	-1.224	-0.08
15142	*KLHL42*	1.2645	-0.68999	-0.82098	-1.8799	-0.08
15143	*FAM227A*	0.91321	-0.26409	-0.89595	-1.0984	-0.08
15144	*KIAA1958*	0.1459	-0.04444	-0.35029	-1.4043	-0.08
15145	*CFAP74*	0.33377	0.23104	-0.81496	-2.2094	-0.08
15146	*IMMP2L*	0.24438	-0.19858	-0.29727	-2.0629	-0.08
15147	*TADA3*	1.5118	-0.19618	-1.568	-2.1848	-0.08
15148	*HOXC13*	1.453	-0.00817	-1.6975	-2.1802	-0.08
15149	*PRADC1*	0.51587	-0.28907	-0.47987	-1.6949	-0.08
15150	*RNF149*	0.13046	-0.08868	-0.29494	-0.68911	-0.08
15151	*CCDC142*	1.0532	0.37076	-1.6776	-2.6999	-0.08
15152	*QTRTD1*	0.68649	-0.21484	-0.72612	-3.0687	-0.08
15153	*RAB3C*	1.0283	-0.62747	-0.65654	-1.7437	-0.09
15154	*CDH10*	0.31864	0.29741	-0.87208	-1.1698	-0.09
15155	*PDXP*	2.1212	-0.73424	-1.6431	-1.8318	-0.09
15156	*CST9L*	0.49579	-0.06413	-0.68781	-1.7119	-0.09
15157	*HSP90AB1*	0.3755	0.19223	-0.82472	-1.0323	-0.09
15158	*GNB5*	0.95205	-0.27453	-0.93465	-1.6836	-0.09
15159	*HIPK1*	0.10767	-0.01905	-0.3464	-0.95708	-0.09
15160	*DUOXA1*	1.6469	0.22526	-2.1305	-2.28	-0.09
15161	*CAPZB*	0.43295	0.40361	-1.0952	-1.6239	-0.09
15162	*SH3YL1*	2.0036	-1.0707	-1.192	-1.5601	-0.09
15163	*SYK*	0.39081	-0.04935	-0.60065	-2.0147	-0.09
15164	*CAPN7*	0.55825	0.41247	-1.2306	-1.5881	-0.09
15165	*HNRNPK*	1.0089	-0.26469	-1.0049	-1.2797	-0.09
15166	*NRN1L*	0.41008	-0.07036	-0.60083	-0.66628	-0.09
15167	*OSBPL9*	0.63969	-0.44353	-0.45742	-2.3389	-0.09
15168	*TCEA1*	0.56657	-0.08868	-0.73961	-0.89752	-0.09
15169	*GREM1*	1.596	-0.59045	-1.2681	-1.9794	-0.09
15170	*PAFAH1B3*	0.95208	-0.36086	-0.85389	-1.0211	-0.09
15171	*ZBBX*	2.4287	-0.89052	-1.8016	-3.1751	-0.09
15172	*AOX1*	0.078829	0.050668	-0.39318	-1.7073	-0.09
15173	*C2orf27A*	1.423	-0.59791	-1.0889	-1.6268	-0.09

15174	*NAT16*	1.0215	-0.46348	-0.82226	-0.85285	-0.09
15175	*OR5V1*	0.58843	-0.33169	-0.52154	-1.5616	-0.09
15176	*EYA2*	0.11836	-0.15876	-0.22674	-0.98158	-0.09
15177	*SMCO4*	1.3832	-0.76076	-0.8897	-1.6368	-0.09
15178	*RNPC3*	1.8236	-0.75835	-1.3326	-2.1111	-0.09
15179	*PPIC*	0.15122	0.093005	-0.51182	-2.4422	-0.09
15180	*RUVBL2*	1.9634	-1.0241	-1.2079	-1.8013	-0.09
15181	*CYP3A4*	0.70993	0.1049	-1.0838	-1.72	-0.09
15182	*POTED*	3.1004	-1.0743	-2.2951	-2.5804	-0.09
15183	*COL14A1*	1.3806	-0.73708	-0.91253	-2.5535	-0.09
15184	*HMGN2*	0.54846	-0.00331	-0.81522	-1.9678	-0.09
15185	*AGA*	1.8259	-0.75724	-1.3389	-1.3966	-0.09
15186	*CTSK*	2.5472	-1.309	-1.5085	-1.7979	-0.09
15187	*UBR1*	0.21727	-0.02156	-0.46636	-1.2736	-0.09
15188	*DTNA*	0.96714	0.27156	-1.5095	-1.5343	-0.09
15189	*CCRL2*	0.062952	-0.1515	-0.18246	-0.44353	-0.09
15190	*SMIM24*	0.40372	-0.13984	-0.53526	-1.3346	-0.09
15191	*PTGDS*	1.0812	-0.51204	-0.84063	-2.9068	-0.09
15192	*RPL27A*	0.94952	0.54334	-1.7647	-2.0398	-0.09
15193	*MRPL36*	0.90007	0.59146	-1.7642	-1.8853	-0.09
15194	*RNF6*	0.70819	0.30691	-1.2879	-2.2452	-0.09
15195	*LCE2B*	0.10006	0.007602	-0.38096	-1.6343	-0.09
15196	*TRPC5*	2.0769	-0.95775	-1.3926	-2.1085	-0.09
15197	*HGS*	0.69084	0.2288	-1.1932	-1.7364	-0.09
15198	*FAM210A*	0.46356	-0.2748	-0.46298	-1.3478	-0.09
15199	*ZBTB8OS*	1.7483	-0.17081	-1.8537	-2.1354	-0.09
15200	*KLHL18*	0.39732	-0.0665	-0.60717	-1.2896	-0.09
15201	*NEK7*	0.34535	-0.17921	-0.44353	-1.0564	-0.09
15202	*ACAD9*	0.53423	0.10217	-0.91403	-1.743	-0.09
15203	*CFAP126*	0.54097	-0.24973	-0.56938	-1.1759	-0.09
15204	*TBC1D16*	0.71042	-0.42808	-0.56076	-0.93802	-0.09
15205	*ZNF462*	1.9314	-0.96463	-1.2457	-2.8574	-0.09
15206	*EXOSC1*	0.3573	0.25826	-0.89518	-0.92823	-0.09
15207	*NBPF14*	0.19726	0.052791	-0.53271	-1.0435	-0.09
15208	*NFAT5*	0.90405	0.25061	-1.4375	-1.9995	-0.09
15209	*ZCCHC9*	3.4654	-1.5797	-2.1695	-2.2543	-0.09
15210	*DDX55*	0.18364	0.15787	-0.62585	-0.7342	-0.09
15211	*SNX19*	1.3484	-0.72488	-0.90791	-1.4998	-0.09
15212	*MUSK*	0.70963	-0.48659	-0.50748	-1.2017	-0.09
15213	*RAD23B*	0.74049	-0.43472	-0.59099	-1.9455	-0.10
15214	*PINK1*	0.024417	-0.02502	-0.28497	-1.8994	-0.10
15215	*ADGRE5*	1.1734	0.002265	-1.4614	-2.0817	-0.10
15216	*RHOF*	1.6136	-0.02958	-1.8698	-1.9623	-0.10
15217	*GC*	0.23307	0.15805	-0.67731	-0.72798	-0.10
15218	*FKBP14*	1.0233	-0.00446	-1.3056	-1.7241	-0.10
15219	*C3orf80*	0.2249	0.16561	-0.67791	-1.4823	-0.10

15220	*ZC3H4*	0.91458	-0.09982	-1.1027	-1.4715	-0.10
15221	*NPAP1*	0.33526	-0.27315	-0.35052	-0.91757	-0.10
15222	*CREG2*	2.6911	-1.2474	-1.7322	-1.7466	-0.10
15223	*SPDYE6*	0.41993	0.13235	-0.84177	-0.92326	-0.10
15224	*ANKS3*	2.5618	-1.2169	-1.6356	-2.0337	-0.10
15225	*NRF1*	2.7461	-1.4568	-1.5815	-2.5008	-0.10
15226	*WDR37*	1.6636	-0.49166	-1.4644	-2.914	-0.10
15227	*AMELY*	0.40077	0.011133	-0.70546	-0.82696	-0.10
15228	*TNFRSF17*	0.085374	0.017406	-0.39664	-0.62803	-0.10
15229	*LANCL2*	1.2626	-0.53406	-1.0231	-1.613	-0.10
15230	*STRA6*	0.45954	0.20713	-0.96144	-1.3475	-0.10
15231	*NAGLU*	0.20905	0.16894	-0.67313	-1.4401	-0.10
15232	*ANKK1*	0.72077	0.20086	-1.2171	-2.0121	-0.10
15233	*C6orf201*	0.7127	-0.29114	-0.71717	-0.84151	-0.10
15234	*NUP62*	0.69539	-0.01706	-0.97433	-1.5294	-0.10
15235	*NSMCE4A*	0.65056	0.52738	-1.4741	-1.7364	-0.10
15236	*TOX*	0.89211	-0.45847	-0.73023	-0.78793	-0.10
15237	*CXorf49*	0.45861	0.017346	-0.77255	-0.84542	-0.10
15238	*AFMID*	1.1816	0.48372	-1.9623	-3.0312	-0.10
15239	*SP140*	0.96268	0.50897	-1.7709	-2.224	-0.10
15240	*HACD2*	0.41241	0.093599	-0.80594	-1.6759	-0.10
15241	*CACNA1D*	0.62376	0.49941	-1.4233	-1.4864	-0.10
15242	*DSC2*	2.6618	-1.2775	-1.6847	-2.0126	-0.10
15243	*MEPCE*	2.2164	-0.93878	-1.5781	-2.5007	-0.10
15244	*SIM1*	0.075215	-0.06007	-0.31641	-1.9305	-0.10
15245	*ANKRD36B*	0.49189	-0.03221	-0.76106	-0.91415	-0.10
15246	*NKX2-1*	0.52028	-0.29614	-0.52587	-1.5971	-0.10
15247	*PDE1A*	0.66958	0.1827	-1.1545	-1.94	-0.10
15248	*MTMR1*	1.1412	0.17324	-1.6176	-2.5298	-0.10
15249	*LRRC14*	0.61166	0.27571	-1.191	-1.4364	-0.10
15250	*MBD3L4*	1.107	0.083386	-1.4944	-2.095	-0.10
15251	*RAB11FIP5*	1.579	0.048175	-1.9319	-1.9579	-0.10
15252	*SLC35C2*	0.061411	-0.16752	-0.19947	-1.0909	-0.10
15253	*TMEM147*	1.9599	-0.45979	-1.8057	-1.9474	-0.10
15254	*RPAP1*	0.60884	0.59437	-1.5095	-2.3958	-0.10
15255	*VGLL4*	0.22608	-0.21327	-0.31981	-0.73362	-0.10
15256	*RAPSN*	0.71302	-0.11567	-0.9059	-2.3449	-0.10
15257	*SPATA8*	0.45695	0.31468	-1.0802	-2.1709	-0.10
15258	*SSC5D*	2.2413	-0.74705	-1.8033	-2.5442	-0.10
15259	*ZNF324B*	0.18753	0.075215	-0.57273	-1.5294	-0.10
15260	*POU5F2*	0.30878	-0.2604	-0.35892	-0.37858	-0.10
15261	*OAF*	0.19547	-0.1829	-0.32328	-1.7149	-0.10
15262	*ASPHD2*	0.89785	0.18099	-1.3903	-1.6401	-0.10
15263	*MAN2C1*	0.11922	-0.05125	-0.3808	-0.95753	-0.10
15264	*TUSC2*	0.55916	-0.4057	-0.46689	-2.5513	-0.10
15265	*SRRT*	2.9904	-1.4154	-1.8885	-1.9623	-0.10

15266	*CREB3L4*	0.10724	-0.04753	-0.37384	-1.1888	-0.10
15267	*KRTAP21-1*	0.2761	0.14487	-0.73577	-0.78963	-0.10
15268	*STK36*	0.78393	-0.40098	-0.69867	-1.3162	-0.11
15269	*NUFIP2*	0.74835	0.2288	-1.2935	-1.3416	-0.11
15270	*ACY1*	1.6081	-0.95154	-0.9733	-1.1807	-0.11
15271	*ZNF334*	1.0702	-0.30899	-1.078	-1.144	-0.11
15272	*LIPG*	0.30791	0.052475	-0.67801	-1.9833	-0.11
15273	*DNPH1*	0.56123	0.020985	-0.90014	-0.98574	-0.11
15274	*RIPK3*	0.70641	-0.12189	-0.9027	-1.3057	-0.11
15275	*PSMF1*	0.29114	0.22896	-0.83851	-1.9186	-0.11
15276	*ZNF319*	0.39098	-0.14449	-0.56564	-1.8157	-0.11
15277	*SIRT2*	0.36116	-0.19889	-0.48156	-2.6046	-0.11
15278	*FLCN*	0.51897	0.36776	-1.2071	-1.8816	-0.11
15279	*PPAN-P2RY11*	0.40355	-0.06308	-0.66146	-2.1068	-0.11
15280	*DMBT1*	0.24352	0.018487	-0.5834	-0.8397	-0.11
15281	*LPIN1*	0.061345	-0.01742	-0.36583	-1.1379	-0.11
15282	*PNPLA8*	0.24289	-0.168	-0.39731	-0.43305	-0.11
15283	*DNAH12*	0.44033	-0.15145	-0.61138	-2.6911	-0.11
15284	*NPEPPS*	0.86484	-0.33126	-0.8562	-1.3924	-0.11
15285	*LRRC8E*	0.69114	0.51028	-1.5246	-1.7576	-0.11
15286	*C14orf39*	0.3417	-0.22645	-0.43886	-1.0099	-0.11
15287	*FAM8A1*	2.1471	-1.1689	-1.3023	-2.0465	-0.11
15288	*KRT18*	2.339	-1.078	-1.5855	-1.9792	-0.11
15289	*PPP1R8*	1.4575	-0.88055	-0.90233	-1.3145	-0.11
15290	*CNGA1*	0.92282	0.26871	-1.5171	-2.5434	-0.11
15291	*XRCC1*	0.19182	0.051278	-0.56873	-0.65434	-0.11
15292	*GREB1L*	0.76492	0.059436	-1.1511	-1.6552	-0.11
15293	*PTPRJ*	0.40848	-0.18568	-0.5497	-2.5617	-0.11
15294	*OR5B17*	2.1145	-0.46303	-1.9789	-2.0818	-0.11
15295	*USP17L4*	0.42304	-0.33619	-0.41469	-1.9123	-0.11
15296	*CDK9*	0.78179	-0.44353	-0.66652	-2.2343	-0.11
15297	*PLEKHB2*	0.79193	-0.06028	-1.0606	-1.297	-0.11
15298	*KRTAP19-2*	0.2274	-0.04238	-0.51435	-0.78842	-0.11
15299	*TBC1D29*	0.16366	-0.05094	-0.44353	-0.76942	-0.11
15300	*PSAP*	0.55047	0.20718	-1.0889	-2.017	-0.11
15301	*RAD17*	2.094	-0.79043	-1.6356	-1.6906	-0.11
15302	*PTRF*	0.29928	-0.2842	-0.34964	-0.86986	-0.11
15303	*EFNB2*	1.3568	0.006098	-1.6975	-2.7568	-0.11
15304	*ZNF747*	0.50614	-0.3565	-0.48524	-0.68889	-0.11
15305	*ERI2*	0.31304	0.034352	-0.68586	-1.2955	-0.11
15306	*DNAJC14*	0.085857	-0.17781	-0.24723	-0.76505	-0.11
15307	*MAPK8IP2*	1.1841	-0.59903	-0.92427	-1.0248	-0.11
15308	*ERH*	0.71036	-0.02563	-1.0243	-1.589	-0.11
15309	*TMEM50A*	0.36129	-0.15141	-0.55002	-1.1082	-0.11
15310	*HES4*	0.99523	-0.27166	-1.0667	-1.9375	-0.11
15311	*ARHGAP42*	3.1423	-1.5243	-1.9623	-2.1477	-0.11

15312	*PIANP*	0.94457	-0.5788	-0.71032	-1.3268	-0.11
15313	*SREBF1*	0.86148	-0.30843	-0.89892	-1.5307	-0.12
15314	*TLN1*	0.47735	-0.18382	-0.6399	-1.7512	-0.12
15315	*OR10A3*	0.97181	-0.00344	-1.3148	-1.5082	-0.12
15316	*KLK6*	0.24303	-0.19753	-0.39209	-0.96161	-0.12
15317	*TRAF1*	0.7847	-0.15394	-0.97776	-1.1802	-0.12
15318	*TMEM30B*	0.50115	-0.3589	-0.48973	-1.4248	-0.12
15319	*CYP27A1*	0.19864	0.15359	-0.70106	-1.6208	-0.12
15320	*POTEJ*	0.98549	0.1182	-1.4527	-2.1364	-0.12
15321	*MMP1*	0.083498	0.014096	-0.44766	-1.4224	-0.12
15322	*DEAF1*	0.56256	0.40217	-1.3148	-2.0613	-0.12
15323	*PEX2*	0.8723	-0.59544	-0.62708	-1.067	-0.12
15324	*CHCHD10*	1.019	0.003476	-1.3729	-1.6302	-0.12
15325	*AOC1*	1.6627	-0.65163	-1.3617	-1.7235	-0.12
15326	*BCL2L1*	0.99875	0.41475	-1.7642	-1.8698	-0.12
15327	*USP18*	0.4357	-0.15313	-0.63341	-1.111	-0.12
15328	*SCGB2B2*	2.373	-1.031	-1.6935	-1.8157	-0.12
15329	*CHRNA3*	0.54063	0.27355	-1.1659	-1.433	-0.12
15330	*CCDC170*	1.7113	-0.52757	-1.5369	-1.6193	-0.12
15331	*KCNK12*	0.50562	0.47672	-1.3358	-2.1198	-0.12
15332	*SSB*	2.4179	-1.3146	-1.4568	-1.6044	-0.12
15333	*TMEM230*	2.4837	-0.94759	-1.8909	-2.1477	-0.12
15334	*NANOGNB*	2.6648	-1.3047	-1.7163	-1.8203	-0.12
15335	*TTC21B*	1.1616	-0.66107	-0.85739	-1.639	-0.12
15336	*LRTM2*	1.4188	-0.61199	-1.1637	-1.2284	-0.12
15337	*FBXO47*	0.7843	0.24723	-1.3892	-2.5571	-0.12
15338	*ILF3*	0.5102	0.016015	-0.88391	-1.0266	-0.12
15339	*KRTAP6-3*	0.46439	0.3065	-1.1287	-1.8742	-0.12
15340	*KRT73*	1.4889	-0.80311	-1.0443	-1.5343	-0.12
15341	*AGL*	0.37448	-0.27911	-0.45399	-1.0643	-0.12
15342	*FADD*	0.27688	-0.00431	-0.63198	-0.70944	-0.12
15343	*ZNF23*	0.32946	-0.30832	-0.3828	-2.0995	-0.12
15344	*AJUBA*	0.43755	-0.20104	-0.59842	-1.4981	-0.12
15345	*CKMT1A*	0.74116	-0.25723	-0.84626	-1.0564	-0.12
15346	*RAD51C*	0.54615	-0.34793	-0.56218	-0.95682	-0.12
15347	*C4orf22*	1.9465	-0.36835	-1.9436	-2.3405	-0.12
15348	*FAM46C*	0.28622	0.14946	-0.80168	-1.4841	-0.12
15349	*CYP17A1*	0.4126	-0.37417	-0.40453	-1.1585	-0.12
15350	*TEX43*	0.77063	-0.29713	-0.84001	-2.0553	-0.12
15351	*SPTLC2*	0.51516	-0.43841	-0.44353	-1.0769	-0.12
15352	*RTFDC1*	0.61924	0.48251	-1.4689	-1.536	-0.12
15353	*MT1H*	0.3755	-0.0649	-0.67776	-1.6334	-0.12
15354	*BRD3*	1.1314	0.30802	-1.8066	-2.3689	-0.12
15355	*SLC5A2*	0.81856	-0.13757	-1.0482	-1.3591	-0.12
15356	*RC3H1*	0.32919	-0.33121	-0.36536	-2.0553	-0.12
15357	*MITF*	-0.02395	-0.09635	-0.24715	-2.2691	-0.12

15358	*CD70*	0.65417	-0.27088	-0.75094	-0.98102	-0.12
15359	*LCE1B*	0.14463	0.10141	-0.614	-1.0158	-0.12
15360	*MMP11*	0.26699	-0.26099	-0.37429	-0.89218	-0.12
15361	*SRPRB*	0.24205	0.044461	-0.65503	-1.3965	-0.12
15362	*ZNF165*	1.2101	-0.75876	-0.8213	-1.8623	-0.12
15363	*IREB2*	0.28096	0.004997	-0.65606	-1.7365	-0.12
15364	*TSHB*	1.0595	-0.65553	-0.7757	-2.2172	-0.12
15365	*SMIM7*	-0.04795	-0.10133	-0.22361	-1.175	-0.12
15366	*COL27A1*	0.41286	-0.34665	-0.43951	-1.6288	-0.12
15367	*RPL23*	1.3095	-0.44353	-1.2393	-1.3664	-0.12
15368	*AMPD1*	2.8987	-1.4566	-1.8157	-3.6506	-0.12
15369	*ZNF407*	1.8173	-0.75625	-1.4361	-1.7422	-0.13
15370	*YEATS2*	0.29432	0.15264	-0.82208	-1.5707	-0.13
15371	*WFDC10B*	0.85564	-0.43544	-0.79535	-1.6389	-0.13
15372	*UBQLN2*	1.1306	-0.62306	-0.88272	-1.773	-0.13
15373	*BANF1*	0.98249	-0.50093	-0.85698	-1.0426	-0.13
15374	*POU3F1*	0.086266	-0.10142	-0.36086	-0.45185	-0.13
15375	*DVL3*	0.77002	0.57736	-1.7235	-1.8073	-0.13
15376	*CEBPE*	0.56164	0.18233	-1.1209	-1.5121	-0.13
15377	*SERAC1*	1.6432	-0.7679	-1.2525	-2.3491	-0.13
15378	*FOXP2*	0.48391	-0.02883	-0.83276	-2.5535	-0.13
15379	*KIAA1024L*	1.1429	-0.38222	-1.1384	-1.5491	-0.13
15380	*MRPS34*	0.72204	0.081909	-1.1817	-2.3405	-0.13
15381	*GRPEL1*	0.013619	-0.0795	-0.31199	-0.64313	-0.13
15382	*ADIPOQ*	0.55507	0.25861	-1.1921	-1.6893	-0.13
15383	*MMS19*	0.45173	-0.17588	-0.65476	-2.2712	-0.13
15384	*CT47A2*	-0.00823	-0.11251	-0.25832	-5.6109	-0.13
15385	*RFPL3*	0.71115	0.12163	-1.2119	-2.1859	-0.13
15386	*FUT10*	0.31071	0.22274	-0.91269	-1.8253	-0.13
15387	*CIAPIN1*	0.76	-0.49778	-0.6424	-1.8909	-0.13
15388	*HTR1B*	0.77873	0.33951	-1.4994	-2.2044	-0.13
15389	*ALDH1L2*	2.1367	-1.0733	-1.4448	-2.6498	-0.13
15390	*RBMS1*	0.34893	0.032955	-0.76366	-1.7316	-0.13
15391	*ALDOC*	0.19795	0.090843	-0.67071	-1.5263	-0.13
15392	*LIMK2*	0.72974	-0.18347	-0.92823	-1.124	-0.13
15393	*LGALS2*	1.0713	-0.33638	-1.1169	-1.6457	-0.13
15394	*RCBTB2*	0.59709	0.19829	-1.1774	-2.0652	-0.13
15395	*CRCP*	0.33789	0.079397	-0.80047	-1.3899	-0.13
15396	*CHTOP*	0.53741	0.26496	-1.1856	-1.3722	-0.13
15397	*ZNF229*	0.82921	-0.33824	-0.875	-1.4523	-0.13
15398	*FETUB*	2.9215	-1.0699	-2.2357	-3.6181	-0.13
15399	*CLDN8*	0.86122	-0.19671	-1.049	-1.5544	-0.13
15400	*SHOX2*	1.6664	-0.56444	-1.4878	-2.4699	-0.13
15401	*LRRC1*	0.34051	-0.16518	-0.56251	-1.3892	-0.13
15402	*CTSD*	0.17335	-0.16781	-0.39457	-0.43329	-0.13
15403	*DNAJB3*	0.061507	-0.14097	-0.30975	-0.66616	-0.13

15404	*HNRNPA3*	0.52403	-0.44444	-0.46918	-1.2975	-0.13
15405	*REL*	0.41716	0.40295	-1.2098	-1.4452	-0.13
15406	*UFL1*	2.308	-1.3364	-1.3627	-1.5077	-0.13
15407	*ICK*	2.7754	-1.1021	-2.0645	-3.2454	-0.13
15408	*PCNP*	1.0788	-0.71553	-0.75448	-1.4584	-0.13
15409	*POLG2*	0.3946	-0.1549	-0.63152	-1.5658	-0.13
15410	*DNHD1*	0.48692	0.050441	-0.93099	-1.729	-0.13
15411	*FBXO11*	0.13158	-0.14117	-0.38473	-1.9292	-0.13
15412	*SLC12A6*	0.23148	-0.1386	-0.48725	-1.1243	-0.13
15413	*RSPO2*	-0.02176	-0.05784	-0.31487	-1.455	-0.13
15414	*ZNF207*	0.097241	-0.00527	-0.48659	-0.6122	-0.13
15415	*SAYSD1*	0.094966	-0.22533	-0.26508	-2.0197	-0.13
15416	*ENDOU*	0.093921	-0.13436	-0.35589	-2.2732	-0.13
15417	*FAM171A2*	0.2372	0.21972	-0.85387	-1.7282	-0.13
15418	*FLT1*	0.94736	0.86236	-2.2078	-2.2223	-0.13
15419	*HAUS5*	0.30573	-0.23766	-0.46631	-1.3802	-0.13
15420	*CHCHD1*	-0.00833	-0.13677	-0.2562	-0.48617	-0.13
15421	*RASGEF1A*	0.68371	-0.50332	-0.58183	-1.0315	-0.13
15422	*ACAP1*	1.4447	-0.28335	-1.5634	-1.9624	-0.13
15423	*KRTAP2-3*	0.10994	-0.19948	-0.31492	-0.72227	-0.13
15424	*SEPT5*	0.70479	0.11742	-1.227	-1.8238	-0.13
15425	*TCF7*	0.90314	-0.45563	-0.85236	-0.97138	-0.13
15426	*MRPS28*	0.3011	-0.20454	-0.50201	-1.4801	-0.14
15427	*PLBD2*	0.43087	0.20038	-1.0369	-1.3593	-0.14
15428	*C3orf84*	0.16225	-0.15992	-0.40816	-2.599	-0.14
15429	*FERMT3*	0.47913	0.16922	-1.0564	-1.2352	-0.14
15430	*NT5C3B*	0.063795	-0.23528	-0.2367	-1.3363	-0.14
15431	*EN1*	0.94502	0.16131	-1.5149	-1.9404	-0.14
15432	*RNF145*	1.9163	-0.79987	-1.525	-1.9534	-0.14
15433	*PPP2R3A*	0.60079	0.081831	-1.0912	-2.3154	-0.14
15434	*CLEC7A*	0.92018	-0.45544	-0.87418	-1.2165	-0.14
15435	*MTERF1*	0.42151	-0.35735	-0.47381	-2.5513	-0.14
15436	*PRSS56*	0.35665	0.10557	-0.87198	-1.0809	-0.14
15437	*RAB44*	2.3989	-1.3982	-1.4108	-1.4442	-0.14
15438	*TVP23A*	1.0873	0.22121	-1.7188	-1.9175	-0.14
15439	*WIBG*	0.20224	0.021388	-0.63436	-1.9659	-0.14
15440	*SRP72*	0.73703	0.14429	-1.2922	-1.5352	-0.14
15441	*CRACR2B*	0.25604	0.070178	-0.73853	-1.191	-0.14
15442	*NAA10*	0.27132	-0.13922	-0.54555	-1.9552	-0.14
15443	*CTCFL*	0.32563	-0.03585	-0.70369	-0.95064	-0.14
15444	*RFX8*	0.8072	-0.55437	-0.66684	-1.6779	-0.14
15445	*DNAJC17*	1.471	-0.51562	-1.3699	-1.775	-0.14
15446	*NKPD1*	1.5054	-0.80727	-1.1138	-1.307	-0.14
15447	*ZNF337*	0.27564	0.21413	-0.90606	-1.088	-0.14
15448	*HPD*	0.26341	-0.03058	-0.64932	-0.91343	-0.14
15449	*UBXN2A*	1.6359	-0.96675	-1.0871	-1.8443	-0.14

15450	*TMEM98*	0.44457	-0.02451	-0.83912	-1.0871	-0.14
15451	*NAB1*	0.44278	0.32102	-1.183	-1.3666	-0.14
15452	*CILP*	0.28767	0.16498	-0.87198	-1.2912	-0.14
15453	*PAPLN*	0.65684	-0.14663	-0.93024	-1.6648	-0.14
15454	*SNRK*	1.4079	-0.76355	-1.0651	-1.5458	-0.14
15455	*SSBP1*	0.60053	-0.44353	-0.57899	-0.73565	-0.14
15456	*STIM1*	0.70504	-0.08424	-1.0433	-1.388	-0.14
15457	*TRIM29*	0.88514	-0.33588	-0.9728	-1.6401	-0.14
15458	*LPA*	0.94839	0.51992	-1.892	-1.9112	-0.14
15459	*SRPX2*	2.2597	-0.61249	-2.071	-2.5934	-0.14
15460	*FAHD2A*	0.73277	-0.39885	-0.75778	-1.3132	-0.14
15461	*ITIH4*	0.31367	-0.15504	-0.58267	-0.95242	-0.14
15462	*SOCS5*	0.772	-0.51931	-0.67764	-0.82368	-0.14
15463	*SARNP*	0.53831	-0.45563	-0.50775	-0.94042	-0.14
15464	*LOC100507462*	0.56846	0.25454	-1.2496	-2.4205	-0.14
15465	*IGSF23*	0.004997	-0.0679	-0.36412	-2.4052	-0.14
15466	*OPN5*	1.0881	-0.74207	-0.77439	-0.78672	-0.14
15467	*BAD*	2.9448	-1.6086	-1.765	-1.94	-0.14
15468	*PRPF4*	0.81223	-0.50324	-0.73814	-1.9619	-0.14
15469	*RET*	1.4872	0.057026	-1.9734	-2.0852	-0.14
15470	*TMEM86A*	1.3244	-0.62947	-1.1244	-1.2632	-0.14
15471	*SLC38A4*	0.61937	0.23127	-1.2817	-1.7577	-0.14
15472	*RPN1*	2.0828	-1.2007	-1.3132	-1.7437	-0.14
15473	*SPATA5*	2.0248	-1.0288	-1.4271	-2.9544	-0.14
15474	*TMEM30A*	0.17147	-0.11621	-0.48652	-0.72798	-0.14
15475	*MFSD6*	0.69766	-0.55409	-0.57506	-1.3773	-0.14
15476	*PTPRQ*	0.31119	0.12964	-0.87332	-1.6023	-0.14
15477	*GLCCI1*	0.38728	-0.35604	-0.46399	-0.48659	-0.14
15478	*ICAM5*	0.94799	-0.43005	-0.9521	-2.3118	-0.14
15479	*GHRL*	0.41043	0.10658	-0.9519	-1.9404	-0.14
15480	*NAP1L3*	0.9578	-0.60701	-0.78651	-1.4998	-0.15
15481	*MFSD3*	0.75829	-0.0205	-1.1739	-1.3851	-0.15
15482	*XAGE2*	1.7771	-0.70442	-1.5099	-2.0197	-0.15
15483	*TM7SF2*	2.618	-1.3966	-1.6604	-1.912	-0.15
15484	*TAGLN3*	2.1068	-1.0293	-1.5167	-1.7323	-0.15
15485	*MAML1*	0.91939	-0.58985	-0.76985	-1.7829	-0.15
15486	*RNPS1*	1.5077	-0.78194	-1.1671	-1.8585	-0.15
15487	*NELFB*	0.28792	0.21028	-0.93969	-1.4076	-0.15
15488	*KDM6A*	0.88215	-0.05139	-1.2736	-2.0952	-0.15
15489	*SHF*	0.68754	-0.02974	-1.1008	-1.2127	-0.15
15490	*PLK3*	1.0205	-0.30618	-1.1576	-1.4633	-0.15
15491	*DAP*	1.0792	0.13006	-1.653	-2.829	-0.15
15492	*DMRTB1*	2.2814	-0.73511	-1.9912	-3.1229	-0.15
15493	*FCRL6*	0.72543	0.59519	-1.7658	-1.9713	-0.15
15494	*KCNK4*	0.46876	-0.02044	-0.89351	-2.3014	-0.15
15495	*BPIFB4*	0.24633	-0.18528	-0.50731	-1.4946	-0.15

15496	*DNM2*	1.5761	-0.79596	-1.2276	-2.5361	-0.15
15497	*OR10A7*	0.88398	0.85388	-2.1859	-2.6543	-0.15
15498	*KIF18A*	1.2654	0.28076	-1.9945	-2.2059	-0.15
15499	*JUN*	0.36957	-0.20207	-0.61599	-0.90663	-0.15
15500	*SPATA2*	0.5094	-0.34535	-0.61261	-0.64397	-0.15
15501	*ZNF555*	0.89861	-0.01439	-1.3328	-1.6306	-0.15
15502	*NFYB*	0.67638	-0.09539	-1.0323	-1.1808	-0.15
15503	*PPM1H*	0.28141	0.087154	-0.81999	-0.95842	-0.15
15504	*CYP3A7*	0.6504	-0.12015	-0.98238	-1.9708	-0.15
15505	*KLHDC2*	0.11527	-0.17746	-0.39081	-1.0063	-0.15
15506	*TTC33*	2.0832	-0.78705	-1.7493	-2.1155	-0.15
15507	*LST1*	0.93628	-0.58403	-0.80594	-1.9295	-0.15
15508	*PCDHA9*	1.4335	-0.80014	-1.0871	-2.0629	-0.15
15509	*OR10T2*	0.64211	-0.45727	-0.63903	-1.0323	-0.15
15510	*TPT1*	1.0756	-0.74219	-0.78784	-2.3977	-0.15
15511	*FKBP2*	0.50127	0.004855	-0.96201	-1.0221	-0.15
15512	*COL18A1*	0.084291	-0.13042	-0.40998	-1.4042	-0.15
15513	*COL5A3*	0.83149	-0.62041	-0.66731	-0.96808	-0.15
15514	*EIF4E*	0.59905	-0.37424	-0.68115	-1.0562	-0.15
15515	*STIP1*	-0.06416	-0.0665	-0.32605	-1.803	-0.15
15516	*DDR2*	0.85727	-0.04877	-1.2657	-1.8596	-0.15
15517	*PPIAL4E*	0.5661	0.39078	-1.4141	-1.8591	-0.15
15518	*AKAP17A*	0.87943	-0.24201	-1.0954	-1.2972	-0.15
15519	*ZNF217*	0.43282	0.3065	-1.1974	-1.2142	-0.15
15520	*SRSF1*	1.022	-0.08832	-1.3924	-2.1132	-0.15
15521	*OMP*	2.4779	-1.2716	-1.6653	-1.7718	-0.15
15522	*RAN*	0.96341	-0.08944	-1.3339	-1.5569	-0.15
15523	*FBXW7*	0.50286	0.18187	-1.1448	-1.3107	-0.15
15524	*DBF4B*	0.018611	-0.23814	-0.24072	-1.6597	-0.15
15525	*ZNF891*	0.2551	-0.10427	-0.61114	-1.3285	-0.15
15526	*GNAT3*	1.0037	0.11767	-1.583	-1.7757	-0.15
15527	*MEDAG*	0.45862	-0.43176	-0.48888	-1.7906	-0.15
15528	*DENND2D*	0.047897	-0.24117	-0.26892	-0.6532	-0.15
15529	*ACKR1*	2.8029	-1.4258	-1.8402	-2.1798	-0.15
15530	*IZUMO1*	1.0491	-0.71443	-0.79821	-1.4775	-0.15
15531	*C1orf131*	0.15169	0.061507	-0.67702	-1.6146	-0.15
15532	*AKT2*	0.72433	-0.40751	-0.78229	-1.1218	-0.16
15533	*MAP1A*	-0.07087	-0.07884	-0.3159	-0.89147	-0.16
15534	*SCAMP5*	1.0231	0.38102	-1.8698	-2.1802	-0.16
15535	*FAM117B*	0.38996	0.38986	-1.2457	-1.5132	-0.16
15536	*NDOR1*	0.33485	-0.26779	-0.53437	-1.6663	-0.16
15537	*EI24*	0.94162	-0.35339	-1.0561	-1.4469	-0.16
15538	*ARPC4-TTLL3*	0.020267	-0.0757	-0.41338	-2.2254	-0.16
15539	*CERS2*	1.0956	-0.20112	-1.3633	-1.7647	-0.16
15540	*MICAL3*	0.047356	-0.15568	-0.36084	-1.4234	-0.16
15541	*FOXC2*	1.5593	-0.2715	-1.7577	-3.2901	-0.16

15542	*DNAJC25*	0.9582	0.25176	-1.6799	-3.8755	-0.16
15543	*P3H2*	1.1793	-0.40092	-1.2485	-1.4048	-0.16
15544	*KSR1*	0.33636	-0.03429	-0.77227	-1.7945	-0.16
15545	*HOXA9*	0.73453	-0.04927	-1.1556	-1.9302	-0.16
15546	*TCEB2*	0.50851	0.017417	-0.99694	-1.0878	-0.16
15547	*POLD3*	0.87109	-0.6551	-0.68741	-1.5782	-0.16
15548	*PGS1*	3.2456	-1.4463	-2.2712	-2.796	-0.16
15549	*PQLC2L*	0.31181	0.25872	-1.043	-1.31	-0.16
15550	*NLN*	0.4785	-0.40532	-0.54579	-0.76516	-0.16
15551	*OR5AN1*	3.2083	-1.6587	-2.0223	-2.2352	-0.16
15552	*UBXN11*	1.7491	-0.32737	-1.8946	-2.2771	-0.16
15553	*RYK*	0.4423	-0.07106	-0.84411	-0.87594	-0.16
15554	*TTC3*	0.2482	-0.33231	-0.38901	-0.75974	-0.16
15555	*YARS*	0.40836	0.17792	-1.0599	-1.3947	-0.16
15556	*ANXA2R*	0.51604	-0.44353	-0.54651	-2.4405	-0.16
15557	*GDF9*	0.24322	0.050441	-0.7679	-1.0273	-0.16
15558	*C2CD3*	0.79289	0.1866	-1.4544	-2.2866	-0.16
15559	*KCTD5*	0.96281	-0.70947	-0.72881	-1.8157	-0.16
15560	*HMGA2*	1.828	-0.85107	-1.4544	-2.2809	-0.16
15561	*THSD7B*	0.63179	-0.1749	-0.93489	-2.9087	-0.16
15562	*LGALS13*	-0.05982	-0.11145	-0.30876	-1.9827	-0.16
15563	*C9orf78*	0.99291	-0.69958	-0.77409	-1.0442	-0.16
15564	*EIF2B2*	0.20086	-0.30966	-0.37199	-1.7215	-0.16
15565	*CYTH1*	0.36722	-0.11637	-0.73211	-0.88767	-0.16
15566	*DEFB121*	0.078312	-0.18858	-0.37125	-1.7335	-0.16
15567	*GNA14*	0.084209	-0.06364	-0.50339	-1.9302	-0.16
15568	*FBXO41*	0.062155	-0.1647	-0.38077	-1.2684	-0.16
15569	*LSM1*	1.1423	-0.44353	-1.1825	-1.5894	-0.16
15570	*ZWILCH*	1.2999	-0.44572	-1.3387	-2.2528	-0.16
15571	*SLC4A7*	2.3956	-1.3427	-1.5375	-2.9354	-0.16
15572	*GLIS3*	0.53014	-0.40411	-0.61114	-1.7845	-0.16
15573	*IGSF1*	1.5512	-1.0024	-1.0343	-2.2311	-0.16
15574	*GPR21*	0.84361	-0.4221	-0.90733	-0.92217	-0.16
15575	*B4GALT5*	0.4282	-0.04207	-0.87208	-1.8478	-0.16
15576	*OSGEPL1*	0.40069	0.33361	-1.2208	-1.864	-0.16
15577	*LMNA*	0.82379	-0.52914	-0.78229	-0.81478	-0.16
15578	*PSMA1*	2.3187	-1.3903	-1.4172	-1.6477	-0.16
15579	*T*	0.25826	-0.13255	-0.61497	-1.2098	-0.16
15580	*TMEM8C*	0.34244	0.066398	-0.8984	-1.9353	-0.16
15581	*INSL6*	2.0925	-1.2208	-1.3619	-1.5243	-0.16
15582	*KEAP1*	0.72239	-0.55832	-0.65489	-0.89809	-0.16
15583	*SDHC*	1.3221	-0.58654	-1.2267	-2.5934	-0.16
15584	*DKK1*	0.71104	0.083498	-1.2867	-1.6887	-0.16
15585	*MPL*	0.98257	-0.17721	-1.2988	-1.857	-0.16
15586	*IQCC*	0.62912	-0.25821	-0.86459	-1.5752	-0.16
15587	*CHD5*	0.12226	-0.20338	-0.41359	-1.584	-0.16

15588	*KIF3C*	-0.04449	-0.218	-0.23252	-0.83972	-0.17
15589	*PANX2*	0.36676	-0.17345	-0.6886	-2.3762	-0.17
15590	*TMIGD2*	0.62998	-0.46032	-0.66535	-1.4234	-0.17
15591	*SCNN1A*	0.66316	-0.4778	-0.68192	-1.5225	-0.17
15592	*FBXO6*	1.5548	-0.7436	-1.3098	-2.0055	-0.17
15593	*KLHL14*	0.30839	-0.1283	-0.67887	-1.7125	-0.17
15594	*SHROOM2*	0.89485	0.81067	-2.2044	-3.4062	-0.17
15595	*SLC35G3*	0.4082	0.13685	-1.0453	-1.1117	-0.17
15596	*P4HA1*	-0.05967	-0.154	-0.2868	-1.9567	-0.17
15597	*CPT1A*	2.6418	-1.4442	-1.6992	-1.7332	-0.17
15598	*GNL2*	0.54746	-0.43077	-0.61892	-1.9168	-0.17
15599	*FAM196A*	1.7222	-0.43469	-1.7906	-3.324	-0.17
15600	*SGCB*	0.75246	-0.07472	-1.181	-1.2829	-0.17
15601	*LURAP1*	0.41193	-0.16459	-0.75128	-1.5082	-0.17
15602	*NCAPD3*	0.18963	-0.0648	-0.62963	-1.0446	-0.17
15603	*KHNYN*	0.976	-0.51862	-0.96326	-1.9678	-0.17
15604	*KRTAP4-3*	1.5633	-0.9744	-1.0956	-1.4234	-0.17
15605	*COPS2*	0.79291	-0.32216	-0.97768	-2.8702	-0.17
15606	*SCARA3*	0.04123	-0.15692	-0.39124	-0.57489	-0.17
15607	*SMYD1*	0.034566	-0.22327	-0.31835	-1.2946	-0.17
15608	*ZNF280D*	1.7707	-1.0385	-1.2411	-2.1947	-0.17
15609	*SPTLC1*	0.66666	-0.05357	-1.122	-2.2381	-0.17
15610	*CD3EAP*	2.4625	-0.97982	-1.9919	-2.0748	-0.17
15611	*PRC1*	0.97072	0.12199	-1.6033	-2.4719	-0.17
15612	*CAMK1D*	1.2163	0.22085	-1.9479	-2.425	-0.17
15613	*C19orf43*	1.4023	-0.88447	-1.0289	-1.151	-0.17
15614	*KDM4D*	0.75545	-0.0412	-1.226	-3.7932	-0.17
15615	*SEC24A*	1.3848	-0.82989	-1.0669	-1.3443	-0.17
15616	*LRRC56*	0.77017	-0.55563	-0.72722	-1.3643	-0.17
15617	*JPH3*	0.35535	-0.28982	-0.57825	-1.7874	-0.17
15618	*NPTN*	0.21989	-0.33136	-0.40142	-2.1143	-0.17
15619	*MCTP1*	0.9884	-0.72819	-0.77444	-1.1055	-0.17
15620	*ETNPPL*	0.13942	0.12463	-0.77895	-1.4336	-0.17
15621	*GALNT9*	0.61705	0.29116	-1.4234	-1.5848	-0.17
15622	*PGAM1*	0.35018	-0.31206	-0.55359	-1.3962	-0.17
15623	*ARHGEF4*	0.29711	-0.33253	-0.48021	-1.3453	-0.17
15624	*KCTD13*	1.0022	-0.25126	-1.2666	-1.8341	-0.17
15625	*LAMA5*	2.2167	-1.3183	-1.4141	-1.4524	-0.17
15626	*MRPL44*	0.8152	-0.09984	-1.2318	-1.671	-0.17
15627	*HTR2C*	0.16852	-0.20023	-0.48486	-1.2259	-0.17
15628	*NACAD*	2.6472	-1.1344	-2.0303	-2.3174	-0.17
15629	*PRKD3*	0.58556	-0.27702	-0.82805	-1.1066	-0.17
15630	*ANKRD13A*	1.0457	-0.4796	-1.0863	-2.071	-0.17
15631	*LIN7C*	1.1714	-0.77863	-0.9135	-1.8107	-0.17
15632	*SLC7A6*	1.1694	-0.18143	-1.5121	-2.1798	-0.17
15633	*RNF167*	0.42261	0.15359	-1.1005	-2.4829	-0.17

15634	*SPATA20*	0.095844	0.083593	-0.70391	-3.7438	-0.17
15635	*LYPLA2*	0.81854	-0.36571	-0.97786	-2.1089	-0.18
15636	*CDC16*	1.8956	-0.54027	-1.8804	-1.9526	-0.18
15637	*APPBP2*	1.2428	-0.57787	-1.1905	-1.5426	-0.18
15638	*PCDH10*	0.25238	-0.32567	-0.45296	-1.1354	-0.18
15639	*ETFB*	1.1564	-0.69565	-0.98747	-1.4507	-0.18
15640	*IDI1*	1.2108	0.10845	-1.8462	-2.4912	-0.18
15641	*POTEH*	0.1119	-0.28819	-0.35111	-0.46441	-0.18
15642	*ANG*	1.4488	-0.97583	-1.0011	-1.5458	-0.18
15643	*FAM26D*	0.11893	-0.27843	-0.36963	-1.53	-0.18
15644	*ECSIT*	0.29555	-0.07322	-0.75182	-1.5031	-0.18
15645	*MCMBP*	0.57921	0.078477	-1.1881	-1.7081	-0.18
15646	*SYT1*	0.13621	0.065894	-0.73348	-1.2036	-0.18
15647	*AMHR2*	0.73634	-0.42936	-0.83856	-0.86533	-0.18
15648	*HHEX*	-0.15548	-0.18	-0.19704	-0.33484	-0.18
15649	*TNFAIP8L2*	0.10337	-0.19457	-0.44136	-1.7056	-0.18
15650	*TP53TG3*	0.22969	-0.27631	-0.48599	-1.4103	-0.18
15651	*C16orf71*	0.75131	-0.46206	-0.82256	-1.0162	-0.18
15652	*MAN2B2*	0.018313	-0.14966	-0.40231	-0.58787	-0.18
15653	*RPLP0*	0.3334	-0.40774	-0.4595	-0.49001	-0.18
15654	*TSC1*	0.45764	0.042799	-1.0344	-2.3357	-0.18
15655	*EVI2A*	0.50074	-0.2412	-0.79367	-1.0435	-0.18
15656	*ZBTB39*	0.90819	-0.2481	-1.1943	-1.4182	-0.18
15657	*MED11*	1.0708	-0.57579	-1.03	-1.0773	-0.18
15658	*C11orf21*	0.66702	-0.55354	-0.64896	-1.1327	-0.18
15659	*SYT5*	0.5995	-0.5597	-0.57646	-1.5587	-0.18
15660	*DEFB103A*	1.2495	-0.15141	-1.6349	-1.7219	-0.18
15661	*IL1A*	0.40077	-0.17116	-0.76856	-1.7996	-0.18
15662	*COX16*	0.5041	-0.11263	-0.93107	-2.7068	-0.18
15663	*PHOSPHO2*	0.18229	-0.30878	-0.41359	-1.2855	-0.18
15664	*TMEM159*	1.1805	-0.39791	-1.3232	-2.3235	-0.18
15665	*VPRBP*	0.96966	-0.20668	-1.3036	-2.5546	-0.18
15666	*CPA2*	4.1613	-2.2135	-2.4885	-3.4314	-0.18
15667	*POU5F1B*	0.68308	-0.14132	-1.084	-1.5031	-0.18
15668	*SIAH2*	0.32525	0.16382	-1.0318	-1.5588	-0.18
15669	*NHLRC2*	0.91376	-0.20489	-1.252	-1.9686	-0.18
15670	*ZNF837*	0.8159	-0.34402	-1.0153	-1.6158	-0.18
15671	*TMEM187*	0.054282	-0.25344	-0.34535	-2.5361	-0.18
15672	*HRASLS2*	0.88308	-0.63976	-0.78784	-1.5436	-0.18
15673	*BFSP2*	0.11973	-0.00828	-0.65606	-1.9455	-0.18
15674	*DLX6*	0.47452	0.45571	-1.4753	-2.6201	-0.18
15675	*CSTF1*	0.035088	-0.09468	-0.48572	-0.62928	-0.18
15676	*NPIPB5*	0.19572	-0.18427	-0.55773	-2.4885	-0.18
15677	*DBP*	2.5207	-1.1866	-1.8816	-2.0164	-0.18
15678	*PCP4*	1.3445	-0.84225	-1.0507	-1.1676	-0.18
15679	*NDUFB6*	1.0568	-0.29599	-1.3104	-1.8461	-0.18

15680	*FLOT2*	0.24356	0.002893	-0.79833	-1.9624	-0.18
15681	*TMEM131*	0.56404	-0.394	-0.72227	-1.8775	-0.18
15682	*TUBA3C*	0.46868	-0.14164	-0.88159	-1.7313	-0.18
15683	*KRT25*	2.0884	-1.1676	-1.4758	-1.8881	-0.19
15684	*GUK1*	0.34029	0.14819	-1.0443	-1.4852	-0.19
15685	*NUTM2B*	0.13885	-0.168	-0.52833	-0.9512	-0.19
15686	*CBL*	2.3606	-1.2411	-1.6791	-2.8478	-0.19
15687	*SMAGP*	0.38929	-0.09892	-0.8504	-2.1592	-0.19
15688	*SRGN*	-0.0643	-0.23977	-0.256	-2.1072	-0.19
15689	*XPC*	0.13215	-0.279	-0.41486	-0.67546	-0.19
15690	*FEN1*	0.89713	-0.44353	-1.0158	-2.1342	-0.19
15691	*FOXA2*	0.38578	0.15784	-1.1069	-1.2113	-0.19
15692	*OR51S1*	1.0393	-0.69939	-0.90478	-2.1182	-0.19
15693	*FAM134A*	0.96739	-0.18494	-1.348	-2.6267	-0.19
15694	*PPP1CB*	1.9748	-0.18214	-2.3601	-2.892	-0.19
15695	*ATG9B*	-0.02164	-0.13816	-0.40765	-0.58851	-0.19
15696	*HOXD4*	0.91178	0.12873	-1.6086	-2.0602	-0.19
15697	*GRM2*	0.3438	-0.271	-0.64194	-1.1461	-0.19
15698	*PTHLH*	0.15579	-0.02187	-0.70391	-2.9134	-0.19
15699	*SLC16A14*	0.088875	0.025715	-0.68466	-1.0878	-0.19
15700	*FAM3C*	0.79601	-0.08059	-1.2864	-1.917	-0.19
15701	*UBE2F*	0.98359	-0.15277	-1.4023	-1.9844	-0.19
15702	*ITGB3*	-0.02883	-0.21175	-0.33131	-1.6693	-0.19
15703	*OVOL1*	0.1827	-0.20268	-0.55282	-1.1448	-0.19
15704	*HPDL*	0.54746	0.50661	-1.6274	-2.1522	-0.19
15705	*OGDH*	1.7484	-0.59959	-1.7223	-2.3601	-0.19
15706	*SH3BP5L*	0.31011	-0.2863	-0.59738	-1.1804	-0.19
15707	*CD2BP2*	-0.01001	-0.24942	-0.31414	-1.5789	-0.19
15708	*TPPP*	1.3884	-0.39722	-1.5651	-1.9715	-0.19
15709	*THAP1*	1.6617	-0.46319	-1.7745	-2.017	-0.19
15710	*C8orf33*	0.67866	-0.41844	-0.83638	-1.1617	-0.19
15711	*MYEF2*	0.21415	-0.33169	-0.45972	-0.75053	-0.19
15712	*TGM1*	2.7402	-1.5274	-1.7903	-1.8324	-0.19
15713	*BECN1*	0.47448	-0.14132	-0.91176	-2.2218	-0.19
15714	*ERBB2*	1.9872	-0.97588	-1.5904	-2.1023	-0.19
15715	*TMEM245*	0.83773	0.073517	-1.4929	-1.8387	-0.19
15716	*TCF7L1*	0.66619	0.45802	-1.7073	-2.6431	-0.19
15717	*MYO9B*	0.42673	-0.28233	-0.7275	-1.7102	-0.19
15718	*POLR2H*	0.96148	-0.36914	-1.1758	-2.1072	-0.19
15719	*FATE1*	0.045836	-0.07219	-0.55844	-1.8157	-0.19
15720	*CEP44*	0.27752	-0.28176	-0.58143	-0.70546	-0.20
15721	*SLC2A3*	0.43491	0.12772	-1.1491	-1.1747	-0.20
15722	*OR1D2*	0.37835	-0.08409	-0.88098	-0.94194	-0.20
15723	*AFF3*	0.093569	-0.02138	-0.65906	-1.6926	-0.20
15724	*TMEM258*	0.57397	-0.50961	-0.65155	-1.8402	-0.20
15725	*SLC1A2*	0.89475	-0.07785	-1.4047	-1.8789	-0.20

15726	*DEFB105A*	1.664	-0.98577	-1.2666	-1.879	-0.20
15727	*RHBDL1*	-0.11975	-0.23289	-0.2379	-2.1187	-0.20
15728	*ZBTB18*	0.66061	0.50612	-1.7581	-2.0411	-0.20
15729	*GTF2H4*	0.39283	-0.25238	-0.73202	-0.81994	-0.20
15730	*HIST1H4J*	0.66253	-0.59763	-0.65682	-1.6722	-0.20
15731	*FASN*	0.71749	0.43208	-1.742	-1.7745	-0.20
15732	*ABCB6*	1.0648	-0.43746	-1.2198	-1.2464	-0.20
15733	*SEC13*	0.67533	-0.32637	-0.9425	-1.4809	-0.20
15734	*DAGLB*	0.47807	-0.11771	-0.95402	-1.2765	-0.20
15735	*CALCB*	0.46377	-0.45292	-0.6047	-1.9105	-0.20
15736	*PRDX6*	1.1336	-0.45185	-1.2756	-1.3554	-0.20
15737	*CEP85*	0.5665	-0.3644	-0.79663	-1.5512	-0.20
15738	*HEXIM1*	0.65278	0.03708	-1.2848	-3.5043	-0.20
15739	*GBP6*	1.2474	-0.87673	-0.96583	-2.4718	-0.20
15740	*PVR*	0.33109	0.00553	-0.93214	-1.1821	-0.20
15741	*OR5A1*	2.8979	-1.4841	-2.011	-2.1103	-0.20
15742	*GIMAP6*	0.63803	-0.30312	-0.93368	-1.3101	-0.20
15743	*UBA2*	0.34835	-0.21526	-0.73301	-1.3296	-0.20
15744	*RPL32*	0.46034	-0.39149	-0.66897	-0.83231	-0.20
15745	*HSF2BP*	0.33635	-0.0576	-0.87993	-1.855	-0.20
15746	*PPP1R15A*	0.23996	-0.33253	-0.509	-5.1074	-0.20
15747	*GTF3C6*	0.60413	-0.42808	-0.77777	-1.9168	-0.20
15748	*C2orf80*	1.4972	-1.0256	-1.0739	-1.3455	-0.20
15749	*MED19*	0.61954	-0.24296	-0.9793	-2.1696	-0.20
15750	*AREL1*	0.38336	-0.1241	-0.86229	-1.3593	-0.20
15751	*ROMO1*	0.3645	-0.41205	-0.55586	-2.103	-0.20
15752	*PRUNE2*	0.30373	-0.25295	-0.65512	-1.3604	-0.20
15753	*POLR1C*	0.78824	-0.22256	-1.1706	-1.9473	-0.20
15754	*PFN2*	0.53529	-0.21946	-0.92129	-1.6085	-0.20
15755	*POLE4*	0.28193	0.084469	-0.97255	-3.0124	-0.20
15756	*USP17L24*	2.3459	-1.3635	-1.5888	-1.9651	-0.20
15757	*JAKMIP3*	0.45029	0.35108	-1.4087	-2.3904	-0.20
15758	*HLA-DMA*	-0.0925	-0.2372	-0.27811	-0.96201	-0.20
15759	*HIRIP3*	0.51973	-0.32779	-0.80014	-1.2867	-0.20
15760	*KCNJ1*	2.0458	-1.0252	-1.6288	-1.6885	-0.20
15761	*NT5C2*	0.75465	0.67742	-2.0407	-3.4888	-0.20
15762	*RPL37*	1.2386	-0.46733	-1.3808	-1.6526	-0.20
15763	*FAM83G*	0.23542	0.18732	-1.0324	-1.3511	-0.20
15764	*LYSMD3*	0.8936	-0.42349	-1.0803	-1.4128	-0.20
15765	*TMEM145*	0.39232	0.31279	-1.3162	-1.5212	-0.20
15766	*ACAD8*	0.31078	-0.37676	-0.54621	-0.84336	-0.20
15767	*ZNF341*	0.59348	-0.51585	-0.68992	-2.1187	-0.20
15768	*MGARP*	0.11552	-0.33266	-0.3952	-0.69841	-0.20
15769	*WDR74*	0.85981	0.040077	-1.5125	-1.5491	-0.20
15770	*FH*	0.46545	0.28749	-1.3666	-2.3857	-0.20
15771	*TRAPPC6A*	0.87856	0.13794	-1.6315	-1.9768	-0.21

15772	*PLEKHH1*	0.15043	-0.03313	-0.73259	-0.88736	-0.21
15773	*TERF2*	0.095567	-0.07297	-0.63791	-1.5213	-0.21
15774	*GPR33*	1.6979	-0.56664	-1.747	-2.2945	-0.21
15775	*SNRPA*	1.0287	0.27221	-1.9175	-2.3017	-0.21
15776	*AAR2*	1.8541	-1.0907	-1.38	-1.8055	-0.21
15777	*CCDC64*	0.71555	0.37368	-1.7061	-2.0028	-0.21
15778	*SAV1*	0.57985	-0.44353	-0.75388	-1.879	-0.21
15779	*MCM8*	0.38623	-0.09323	-0.91061	-0.9554	-0.21
15780	*PPP2R4*	0.69258	-0.11619	-1.1943	-1.3677	-0.21
15781	*COPG2*	0.54936	-0.43634	-0.73202	-0.95629	-0.21
15782	*ZNF684*	0.77368	-0.57542	-0.81757	-1.7194	-0.21
15783	*KCTD14*	1.3526	-0.53541	-1.4368	-2.1151	-0.21
15784	*CPSF7*	0.49337	-0.42378	-0.69003	-2.4784	-0.21
15785	*RHOQ*	1.0522	-0.64467	-1.0293	-1.4064	-0.21
15786	*NAIF1*	0.33767	-0.39885	-0.5613	-3.3337	-0.21
15787	*PRM1*	0.80342	-0.69132	-0.73529	-2.1018	-0.21
15788	*RPS6KA3*	0.012638	-0.1778	-0.4583	-0.62527	-0.21
15789	*EDC3*	2.0483	-0.88208	-1.7901	-2.0055	-0.21
15790	*WFDC2*	-0.09789	-0.22045	-0.30608	-1.2339	-0.21
15791	*PPP4C*	0.57599	-0.19213	-1.0093	-1.496	-0.21
15792	*ZNF75A*	2.6028	-1.4233	-1.806	-2.0553	-0.21
15793	*KCNIP2*	1.5085	-0.97781	-1.1585	-2.6805	-0.21
15794	*VIM*	1.8948	-1.1263	-1.3966	-2.1404	-0.21
15795	*PSMB6*	0.49944	0.14324	-1.2708	-1.4498	-0.21
15796	*LY75-CD302*	0.59574	-0.28406	-0.94255	-2.6803	-0.21
15797	*STOX2*	0.64372	-0.56485	-0.70998	-1.1067	-0.21
15798	*CRYGC*	0.74302	-0.49778	-0.87662	-0.95759	-0.21
15799	*SLC25A22*	0.4344	0.095588	-1.1617	-1.589	-0.21
15800	*TAF9*	0.82054	-0.71016	-0.7425	-1.6105	-0.21
15801	*RNF123*	-0.04553	-0.29263	-0.29414	-1.2215	-0.21
15802	*CBFA2T2*	0.74318	-0.65901	-0.71669	-1.0813	-0.21
15803	*RPS9*	2.5348	-1.3683	-1.7991	-2.796	-0.21
15804	*ATP5O*	0.95133	-0.53025	-1.0545	-2.0214	-0.21
15805	*SPPL3*	0.26758	-0.25353	-0.64806	-1.4047	-0.21
15806	*SLC35F4*	1.5783	0.38033	-2.5934	-2.6805	-0.21
15807	*GJA1*	1.6194	-0.87598	-1.379	-1.5293	-0.21
15808	*KRT75*	-0.0275	-0.15657	-0.45185	-0.97477	-0.21
15809	*AIF1L*	0.21539	-0.2052	-0.64671	-1.8737	-0.21
15810	*SLC16A9*	0.52332	0.040531	-1.2014	-1.716	-0.21
15811	*AKT1S1*	1.3188	-0.38472	-1.5722	-1.9023	-0.21
15812	*IRF2*	0.37441	-0.26895	-0.74365	-1.951	-0.21
15813	*EIF4EBP3*	0.4084	0.31108	-1.358	-3.1568	-0.21
15814	*ZNF45*	0.34851	0.2961	-1.2832	-1.6597	-0.21
15815	*UHRF2*	0.25848	-0.0351	-0.86358	-2.6964	-0.21
15816	*C21orf62*	0.29586	-0.30371	-0.63341	-1.7652	-0.21
15817	*CSMD2*	0.5366	-0.58851	-0.58949	-2.3204	-0.21

15818	*BBS2*	0.97032	-0.78099	-0.83231	-0.87817	-0.21
15819	*MMP13*	0.43472	-0.00472	-1.073	-1.748	-0.21
15820	*BTBD7*	0.61741	-0.33266	-0.92781	-1.2383	-0.21
15821	*IMPDH2*	0.69471	-0.13543	-1.2036	-2.0008	-0.21
15822	*VWA9*	0.91948	-0.62073	-0.94399	-1.7268	-0.22
15823	*MESP1*	0.93499	-0.29879	-1.2815	-2.2167	-0.22
15824	*CHIC1*	0.46422	0.23955	-1.3492	-1.3591	-0.22
15825	*ARMCX5*	0.52927	-0.43906	-0.73668	-0.98585	-0.22
15826	*CEP135*	0.2732	-0.4317	-0.48829	-0.96857	-0.22
15827	*ZFP41*	0.38454	0.065282	-1.0973	-2.1108	-0.22
15828	*SFRP2*	-0.08064	-0.23875	-0.32861	-0.34181	-0.22
15829	*SERPINA11*	1.1914	-0.50524	-1.3343	-1.6246	-0.22
15830	*SART3*	1.9354	-0.95645	-1.6281	-1.7142	-0.22
15831	*RUFY1*	0.26863	-0.36233	-0.55586	-0.72825	-0.22
15832	*C15orf57*	2.3666	-0.93107	-2.0851	-2.5361	-0.22
15833	*TMPRSS11F*	0.33676	-0.43634	-0.55002	-3.3367	-0.22
15834	*ACTR8*	0.44638	-0.25205	-0.84457	-1.9602	-0.22
15835	*RCVRN*	1.0166	-0.4499	-1.2172	-1.3729	-0.22
15836	*MED23*	1.2792	-0.71953	-1.2109	-1.8582	-0.22
15837	*OR52N2*	1.4202	-0.97982	-1.0918	-2.1351	-0.22
15838	*INMT*	0.34115	0.19975	-1.1925	-1.42	-0.22
15839	*RTP5*	1.3649	-0.64219	-1.3751	-2.9494	-0.22
15840	*LOC102724957*	0.56579	-0.51859	-0.70074	-1.7266	-0.22
15841	*DBR1*	2.364	-0.76606	-2.252	-2.4331	-0.22
15842	*TARS*	0.73122	-0.39308	-0.99241	-2.1873	-0.22
15843	*LAMTOR5*	0.43087	-0.2704	-0.81509	-3.3367	-0.22
15844	*MTMR6*	0.60661	0.043739	-1.3067	-1.4034	-0.22
15845	*WDR87*	1.0589	-0.82897	-0.88784	-2.5442	-0.22
15846	*MAPK13*	2.163	-0.56171	-2.2593	-2.6498	-0.22
15847	*NCKIPSD*	-0.03491	-0.15626	-0.46705	-1.5306	-0.22
15848	*ECHDC3*	0.72272	-0.44774	-0.93474	-1.6682	-0.22
15849	*IZUMO2*	0.75823	-0.27541	-1.1426	-1.1964	-0.22
15850	*NOC4L*	1.2871	-0.60543	-1.3419	-2.0113	-0.22
15851	*DAXX*	0.56073	-0.43845	-0.78314	-1.0107	-0.22
15852	*MYO10*	0.34835	-0.43884	-0.57142	-0.79711	-0.22
15853	*ADAMTS2*	-0.06145	-0.11219	-0.48854	-1.2162	-0.22
15854	*ERLIN2*	0.52749	0.47987	-1.6696	-2.3876	-0.22
15855	*ANKRD20A1*	1.1396	-0.01531	-1.7866	-1.9271	-0.22
15856	*SF1*	0.56915	-0.53987	-0.69186	-2.4038	-0.22
15857	*TRAK1*	0.37779	-0.51221	-0.52853	-0.56376	-0.22
15858	*RNF187*	0.91433	0.083863	-1.6617	-1.917	-0.22
15859	*ARG1*	0.48128	0.14807	-1.2935	-1.3296	-0.22
15860	*TOP2A*	1.039	0.046235	-1.7495	-2.0601	-0.22
15861	*GPR22*	0.91449	-0.5193	-1.0596	-1.7874	-0.22
15862	*ARMCX4*	0.29782	-0.37578	-0.58654	-1.4048	-0.22
15863	*EFCAB6*	0.90876	-0.54307	-1.0303	-1.864	-0.22

15864	*RRM2B*	2.6274	-1.5341	-1.7591	-1.9028	-0.22
15865	*CLIC3*	0.50365	-0.36557	-0.80548	-2.5298	-0.22
15866	*OR8U1*	1.1907	-0.42177	-1.4375	-2.8863	-0.22
15867	*KLHL28*	0.82626	-0.5183	-0.97784	-1.1487	-0.22
15868	*ST6GALNAC2*	0.25152	0.20572	-1.1278	-1.1607	-0.22
15869	*REPS1*	0.014271	-0.10628	-0.57857	-0.8613	-0.22
15870	*IGHMBP2*	0.43719	-0.1227	-0.98585	-1.0485	-0.22
15871	*NOTCH4*	0.8204	0.062587	-1.5544	-1.6356	-0.22
15872	*NOP14*	0.10289	-0.32143	-0.4537	-1.5984	-0.22
15873	*CIITA*	0.48201	-0.06214	-1.0925	-1.309	-0.22
15874	*MRPL48*	0.96442	-0.78229	-0.85478	-2.3486	-0.22
15875	*SPDYE1*	1.0095	-0.79316	-0.88981	-0.89854	-0.22
15876	*INTS1*	0.43235	-0.38059	-0.72635	-1.0301	-0.22
15877	*ATP4B*	0.28226	-0.46754	-0.49007	-3.4182	-0.23
15878	*LIPH*	1.7369	-1.0856	-1.329	-1.6594	-0.23
15879	*DRD2*	0.42958	-0.46767	-0.63971	-0.97046	-0.23
15880	*RNASEL*	0.41177	-0.36562	-0.7241	-2.6889	-0.23
15881	*NFAM1*	0.48445	-0.47769	-0.68586	-1.4601	-0.23
15882	*MED25*	1.2001	-0.71077	-1.1685	-1.4754	-0.23
15883	*SNRNP40*	1.5884	-1.0489	-1.2187	-2.5852	-0.23
15884	*DLX1*	1.9314	-1.2736	-1.3371	-1.5552	-0.23
15885	*AGPAT2*	0.60513	-0.54887	-0.73565	-2.4815	-0.23
15886	*EAPP*	2.9047	-1.6116	-1.9727	-2.6376	-0.23
15887	*C9orf62*	0.51927	-0.51494	-0.6858	-1.0088	-0.23
15888	*ZNF594*	0.11365	-0.35161	-0.44353	-1.6842	-0.23
15889	*RCOR2*	1.4261	-0.80349	-1.3042	-1.4361	-0.23
15890	*PEX16*	1.2977	-0.54409	-1.4375	-2.1893	-0.23
15891	*SLC25A37*	0.50884	-0.30029	-0.89261	-1.499	-0.23
15892	*SLC9A3R1*	-0.03229	-0.23719	-0.41533	-1.349	-0.23
15893	*PDCD1LG2*	0.61378	-0.59871	-0.70074	-0.81918	-0.23
15894	*TMEM220*	1.8207	-1.1508	-1.3565	-1.5171	-0.23
15895	*MGME1*	1.0066	-0.30611	-1.3878	-3.5278	-0.23
15896	*ANKLE2*	0.15113	0.10415	-0.94341	-1.748	-0.23
15897	*SNX22*	0.12366	-0.13984	-0.67395	-2.8807	-0.23
15898	*KRBOX4*	-0.07191	-0.21964	-0.39885	-0.61264	-0.23
15899	*SRA1*	1.3626	-0.53069	-1.5247	-2.0553	-0.23
15900	*PSMD12*	0.51986	-0.01545	-1.1974	-2.3471	-0.23
15901	*DNAJA3*	2.0583	-0.81505	-1.9367	-2.5603	-0.23
15902	*TMEM140*	1.3397	-0.43634	-1.5971	-2.0176	-0.23
15903	*MPC1L*	0.23768	-0.39457	-0.5377	-1.0871	-0.23
15904	*MYCL*	1.2705	-0.92288	-1.043	-2.9283	-0.23
15905	*PIK3C3*	0.32416	-0.03784	-0.98211	-1.0295	-0.23
15906	*CHMP7*	1.0214	-0.35063	-1.367	-1.5658	-0.23
15907	*UXS1*	0.34636	-0.05715	-0.98572	-1.7018	-0.23
15908	*HIST1H4H*	0.3721	0.054707	-1.1242	-2.0411	-0.23
15909	*NOL7*	1.3001	-0.92553	-1.0726	-1.7963	-0.23

15910	*DLL1*	0.1979	-0.34077	-0.55741	-2.4268	-0.23
15911	*RFPL1*	0.3198	-0.34691	-0.67321	-2.9236	-0.23
15912	*OR4N4*	0.82244	0.30863	-1.8318	-1.8462	-0.23
15913	*ARFRP1*	1.143	-0.3271	-1.5167	-2.3424	-0.23
15914	*CCDC115*	0.20086	0.18746	-1.0894	-1.2846	-0.23
15915	*UBAP1*	0.10782	-0.2193	-0.58994	-0.73443	-0.23
15916	*SMPD2*	1.9216	-0.89474	-1.729	-2.3372	-0.23
15917	*YEATS4*	0.44933	0.10802	-1.2595	-1.3218	-0.23
15918	*GCC1*	1.3041	-0.09807	-1.9089	-3.9751	-0.23
15919	*OTUD3*	0.6799	-0.68896	-0.69395	-1.4491	-0.23
15920	*EFR3B*	0.35299	-0.41406	-0.6421	-0.73604	-0.23
15921	*CFHR2*	2.7399	-1.4684	-1.9758	-2.2456	-0.23
15922	*OASL*	1.0837	-0.79902	-0.98925	-1.1216	-0.23
15923	*HSH2D*	0.76028	-0.34906	-1.1169	-1.4312	-0.24
15924	*GSX1*	1.2967	-0.08601	-1.9166	-2.1893	-0.24
15925	*MED14*	1.9837	-1.1472	-1.5425	-2.8478	-0.24
15926	*C10orf76*	0.67278	-0.59157	-0.78959	-1.3292	-0.24
15927	*C2CD5*	1.1583	-0.62114	-1.2465	-3.1086	-0.24
15928	*IL17C*	0.35364	-0.44134	-0.62213	-1.0407	-0.24
15929	*SLC5A11*	0.71478	-0.62479	-0.8003	-1.5031	-0.24
15930	*FAM129B*	1.6289	-0.32494	-2.0164	-3.5736	-0.24
15931	*DMRT1*	1.5329	-1.018	-1.2288	-1.7945	-0.24
15932	*PTPN23*	1.9158	-1.2405	-1.3905	-3.6757	-0.24
15933	*COG3*	-0.07673	-0.13009	-0.50869	-0.95722	-0.24
15934	*CNOT8*	0.47126	0.41068	-1.5978	-2.4094	-0.24
15935	*TP53TG5*	0.25012	-0.12418	-0.84291	-1.6604	-0.24
15936	*AGFG1*	1.6917	-0.83676	-1.5724	-1.9089	-0.24
15937	*ITPRIPL1*	0.79192	-0.00828	-1.5016	-1.5568	-0.24
15938	*CCDC83*	0.7058	-0.2223	-1.2016	-1.4055	-0.24
15939	*C14orf177*	0.48497	-0.0908	-1.1124	-1.1496	-0.24
15940	*CCDC109B*	0.014931	-0.31295	-0.42046	-1.2145	-0.24
15941	*GPA33*	-0.11354	-0.12556	-0.48022	-2.3036	-0.24
15942	*RFX5*	0.67805	0.00784	-1.4064	-1.9474	-0.24
15943	*FANCF*	0.71104	-0.35905	-1.0726	-1.36	-0.24
15944	*TMCO4*	0.57494	-0.17016	-1.1258	-1.208	-0.24
15945	*ALDOB*	0.91239	-0.48973	-1.1439	-2.0165	-0.24
15946	*ULK1*	0.55504	-0.24401	-1.0328	-1.8707	-0.24
15947	*TMTC3*	0.10931	0.079762	-0.91134	-1.3899	-0.24
15948	*RAB29*	0.48714	-0.02232	-1.1872	-2.0791	-0.24
15949	*C19orf33*	0.045563	-0.36705	-0.40109	-1.4597	-0.24
15950	*RNASEH2C*	1.0777	-0.41973	-1.3808	-2.3644	-0.24
15951	*ELP6*	0.37459	-0.45748	-0.64088	-2.0863	-0.24
15952	*NPLOC4*	0.58268	-0.33253	-0.97393	-1.9622	-0.24
15953	*SLC16A11*	0.275	0.20215	-1.2015	-2.1108	-0.24
15954	*TRIM7*	0.23698	-0.38574	-0.5765	-0.67801	-0.24
15955	*SLC26A4*	1.6562	-1.1508	-1.2312	-1.4841	-0.24

15956	*SPPL2B*	2.2258	-1.2259	-1.7266	-2.3389	-0.24
15957	*DDX17*	1.7016	-0.7097	-1.7188	-1.7538	-0.24
15958	*OR5F1*	0.84965	-0.16277	-1.4142	-1.4284	-0.24
15959	*THRSP*	0.91211	-0.80455	-0.83511	-0.9442	-0.24
15960	*ZNF763*	0.75628	0.41923	-1.9038	-3.0974	-0.24
15961	*ETV2*	1.1191	-0.91685	-0.93057	-2.3261	-0.24
15962	*TMEM191B*	0.6433	-0.17052	-1.2014	-1.3316	-0.24
15963	*RANGRF*	0.43211	0.24349	-1.4043	-2.2586	-0.24
15964	*FREM3*	0.53862	-0.11057	-1.1575	-2.1085	-0.24
15965	*LONRF3*	2.3489	-1.2571	-1.8227	-1.9446	-0.24
15966	*FBXO21*	0.21315	-0.12876	-0.81543	-2.43	-0.24
15967	*C20orf203*	0.41263	-0.36311	-0.78098	-1.7149	-0.24
15968	*AGAP11*	-0.08064	-0.21833	-0.43251	-1.5026	-0.24
15969	*OR1D5*	0.14137	-0.33812	-0.53481	-0.68522	-0.24
15970	*RPS6KA6*	0.50712	-0.27961	-0.96031	-1.8611	-0.24
15971	*GSTM4*	0.31744	-0.10928	-0.94119	-2.3033	-0.24
15972	*ARRDC3*	-0.07982	-0.27855	-0.37602	-0.58703	-0.24
15973	*TNC*	0.58558	-0.53661	-0.78363	-1.0049	-0.24
15974	*PXYLP1*	0.48465	0.14018	-1.3597	-1.5307	-0.24
15975	*C7*	0.47342	0.284	-1.4932	-1.6836	-0.25
15976	*ARID1B*	0.76684	0.014205	-1.5171	-2.5008	-0.25
15977	*XPO5*	0.53244	-0.5903	-0.6785	-1.2063	-0.25
15978	*BHLHE22*	-0.05129	-0.32121	-0.36404	-0.97865	-0.25
15979	*BNIP3*	0.32504	-0.2192	-0.84291	-0.89529	-0.25
15980	*KRT6C*	0.88577	-0.35675	-1.2661	-1.8575	-0.25
15981	*SMTNL1*	0.7407	-0.61779	-0.86047	-2.4784	-0.25
15982	*ZP2*	0.059245	-0.31815	-0.47929	-1.0919	-0.25
15983	*NBPF20*	0.13087	-0.20994	-0.65946	-1.8872	-0.25
15984	*RBM20*	0.80365	0.12548	-1.6682	-2.7191	-0.25
15985	*SLC6A14*	0.61334	-0.47486	-0.87768	-1.6791	-0.25
15986	*FEZF2*	1.8934	-0.34476	-2.2879	-2.3235	-0.25
15987	*SUGP2*	0.39896	-0.51549	-0.62306	-0.97952	-0.25
15988	*GFPT1*	1.1839	-0.89593	-1.0286	-2.1496	-0.25
15989	*ATF6*	-0.1611	-0.25996	-0.31984	-1.8157	-0.25
15990	*CYP26C1*	0.48677	-0.44353	-0.78439	-1.7655	-0.25
15991	*PURG*	1.0294	-0.49073	-1.28	-1.5494	-0.25
15992	*CKS1B*	0.68203	-0.01811	-1.4057	-1.8227	-0.25
15993	*ADRA1D*	0.46505	-0.04542	-1.1619	-1.7073	-0.25
15994	*ZC3H15*	0.22085	-0.25261	-0.71063	-1.1387	-0.25
15995	*USP11*	0.51575	-0.57448	-0.68376	-1.2027	-0.25
15996	*SSH2*	0.2826	-0.0261	-0.99917	-1.5788	-0.25
15997	*THBS3*	0.4303	0.38377	-1.5568	-1.7364	-0.25
15998	*FBN3*	0.66338	-0.31126	-1.0952	-2.2599	-0.25
15999	*DDX10*	1.7609	-1.1384	-1.3663	-2.4456	-0.25
16000	*CTNNBL1*	0.13093	0.086138	-0.96144	-1.7215	-0.25
16001	*AR*	0.28345	-0.24501	-0.78325	-1.4076	-0.25

16002	*RSPO1*	1.1671	-0.4055	-1.5073	-1.9329	-0.25
16003	*ARL13B*	0.86108	-0.78484	-0.82289	-1.5645	-0.25
16004	*DERA*	0.40814	-0.54734	-0.60845	-1.7963	-0.25
16005	*CH17-360D5.1*	0.36972	0.00068	-1.1185	-1.6626	-0.25
16006	*POLR2J*	0.73274	0.32263	-1.8038	-3.8779	-0.25
16007	*SUPT7L*	0.59849	-0.00058	-1.347	-1.448	-0.25
16008	*DEFB133*	0.4488	-0.14151	-1.0564	-1.1113	-0.25
16009	*DDX18*	0.5805	-0.47412	-0.85594	-1.0898	-0.25
16010	*DTX4*	0.2261	-0.45607	-0.52008	-0.64574	-0.25
16011	*MRPS35*	0.096888	-0.36991	-0.47707	-1.2431	-0.25
16012	*GPHN*	2.1603	-1.3226	-1.5884	-1.6836	-0.25
16013	*PRAMEF5*	0.42877	0.21595	-1.3956	-1.4614	-0.25
16014	*CALCOCO1*	0.55344	-0.25875	-1.0456	-1.1577	-0.25
16015	*FBXL18*	1.5311	-0.64192	-1.6401	-1.9899	-0.25
16016	*PNPLA3*	1.3282	0.089257	-2.1685	-2.6222	-0.25
16017	*OR1L4*	0.50321	0.38156	-1.6368	-1.8838	-0.25
16018	*C2orf72*	0.95045	-0.40749	-1.2968	-1.9175	-0.25
16019	*ZNF117*	-0.02598	-0.36371	-0.36451	-0.75192	-0.25
16020	*PKD1L2*	0.62338	-0.67573	-0.70188	-1.0948	-0.25
16021	*SP5*	0.26315	-0.13183	-0.88561	-1.5876	-0.25
16022	*AAK1*	0.73215	-0.63152	-0.85536	-1.5598	-0.25
16023	*C1orf185*	0.25636	-0.30763	-0.70442	-1.1698	-0.25
16024	*C12orf73*	0.97085	-0.23418	-1.4951	-2.1798	-0.25
16025	*NDRG4*	-0.12786	-0.26314	-0.36774	-0.80595	-0.25
16026	*FARSB*	0.45221	-0.35272	-0.86047	-1.106	-0.25
16027	*ITM2C*	0.43259	0.27334	-1.4682	-1.8191	-0.25
16028	*ADCY3*	-0.20512	-0.24234	-0.31671	-0.44353	-0.25
16029	*MRPS18A*	0.15937	0.024107	-0.94841	-1.8587	-0.25
16030	*SCCPDH*	0.89539	-0.44353	-1.2171	-2.071	-0.26
16031	*MRPL21*	0.54988	0.024885	-1.341	-1.5759	-0.26
16032	*LINGO3*	3.054	-1.4852	-2.3357	-2.7056	-0.26
16033	*COPS3*	1.3404	-0.54784	-1.5598	-2.2631	-0.26
16034	*SLC25A41*	0.39528	0.12206	-1.2852	-1.89	-0.26
16035	*HIC2*	1.2967	-0.94393	-1.1207	-1.9023	-0.26
16036	*CDH9*	0.75145	-0.55365	-0.96622	-1.8562	-0.26
16037	*PPCDC*	1.3109	-0.17105	-1.9099	-2.1319	-0.26
16038	*TRIM13*	0.20047	-0.39313	-0.5775	-2.1616	-0.26
16039	*MCOLN2*	1.0339	-0.80897	-0.9952	-1.1108	-0.26
16040	*DCANP1*	0.36231	0.2474	-1.3802	-1.3814	-0.26
16041	*ANKFY1*	0.36882	-0.40118	-0.73853	-0.91842	-0.26
16042	*SLBP*	0.12307	-0.27453	-0.61946	-1.1095	-0.26
16043	*KRT14*	1.1971	-0.83074	-1.141	-1.478	-0.26
16044	*LOC101927322*	0.53005	-0.58289	-0.72304	-2.462	-0.26
16045	*C17orf59*	-0.06896	-0.24555	-0.46205	-0.71077	-0.26
16046	*SLC22A9*	0.55552	0.34367	-1.6759	-1.9326	-0.26
16047	*FAM120C*	0.21479	-0.43572	-0.55586	-0.9225	-0.26

16048	*PDE3B*	1.2827	-0.44711	-1.6129	-2.8288	-0.26
16049	*RBM12*	0.29479	0.010038	-1.0824	-1.5711	-0.26
16050	*OR2A2*	0.43459	-0.5584	-0.65402	-0.75192	-0.26
16051	*PTGS1*	0.35451	0.06506	-1.1977	-1.8537	-0.26
16052	*KANSL3*	-0.19422	-0.2748	-0.31004	-0.92868	-0.26
16053	*SF3B5*	0.26579	-0.12908	-0.91617	-2.1158	-0.26
16054	*MADCAM1*	0.30681	-0.04162	-1.045	-1.1704	-0.26
16055	*IAH1*	2.1088	-1.3218	-1.5671	-1.589	-0.26
16056	*TTBK1*	0.04816	-0.39872	-0.43016	-1.5901	-0.26
16057	*ZNF746*	0.9791	-0.46733	-1.2936	-1.8844	-0.26
16058	*PRSS50*	0.62321	-0.53815	-0.86861	-1.7829	-0.26
16059	*MYL4*	0.6885	-0.5639	-0.90865	-2.3729	-0.26
16060	*ZYG11A*	1.2016	-0.72214	-1.2647	-1.515	-0.26
16061	*SLC38A6*	0.53063	0.32916	-1.6456	-2.0908	-0.26
16062	*ATP13A1*	0.017417	-0.10071	-0.70418	-0.99137	-0.26
16063	*NRG2*	1.1285	-0.6057	-1.3107	-2.0267	-0.26
16064	*MRPL18*	1.798	-0.81217	-1.7766	-1.872	-0.26
16065	*MALL*	2.4948	-1.129	-2.1566	-3.3367	-0.26
16066	*WBSCR16*	0.50129	0.17112	-1.4633	-1.8157	-0.26
16067	*GNAL*	0.62146	0.028715	-1.4429	-1.7077	-0.26
16068	*EYA4*	0.33676	-0.46766	-0.66215	-1.526	-0.26
16069	*FOS*	1.184	-0.51653	-1.4607	-1.5212	-0.26
16070	*FOXD2*	0.71474	-0.58136	-0.92711	-1.2647	-0.26
16071	*ZC3H12D*	0.44778	-0.49564	-0.74594	-0.91944	-0.26
16072	*VPS35*	0.66502	0.00962	-1.4686	-2.1125	-0.26
16073	*ZMYND11*	0.91832	-0.22763	-1.4852	-1.5866	-0.26
16074	*FGA*	0.39573	-0.59183	-0.59871	-1.1193	-0.26
16075	*MPV17L*	0.57613	-0.19146	-1.1796	-1.8884	-0.26
16076	*TSC2*	0.88521	-0.46743	-1.2133	-2.1239	-0.27
16077	*CHCHD4*	0.16266	0.096388	-1.0557	-1.1101	-0.27
16078	*OR10J5*	0.72192	-0.53078	-0.9879	-1.03	-0.27
16079	*BGN*	0.39098	-0.49045	-0.69759	-0.91253	-0.27
16080	*MTNR1B*	-0.17696	-0.30424	-0.31641	-1.2694	-0.27
16081	*UMOD*	0.49885	-0.35135	-0.94546	-1.1918	-0.27
16082	*COG4*	0.15458	-0.23252	-0.72085	-2.1342	-0.27
16083	*EHMT2*	2.0137	-1.2244	-1.5883	-2.2059	-0.27
16084	*ABHD12B*	1.0757	0.012183	-1.8885	-2.1696	-0.27
16085	*EML2*	0.79519	-0.33321	-1.2632	-1.7783	-0.27
16086	*NPIPA3*	0.61063	-0.62306	-0.78959	-1.5415	-0.27
16087	*ADH1A*	-0.09079	-0.09716	-0.61421	-1.0273	-0.27
16088	*RAD51*	0.43339	0.36658	-1.6023	-2.7504	-0.27
16089	*DDX58*	-0.13997	-0.21899	-0.44353	-1.1226	-0.27
16090	*QPRT*	0.58777	-0.62306	-0.76755	-1.1417	-0.27
16091	*TMEM79*	0.2464	-0.38901	-0.6608	-2.2507	-0.27
16092	*SKIV2L*	1.6077	-0.79378	-1.6176	-1.8238	-0.27
16093	*CLCA1*	0.67583	-0.3098	-1.1704	-2.0116	-0.27

16094	*C22orf46*	0.60353	-0.6176	-0.79035	-1.0248	-0.27
16095	*C19orf44*	1.4922	-1.0803	-1.2169	-2.2866	-0.27
16096	*BOLA2B*	0.27164	-0.50961	-0.568	-1.0743	-0.27
16097	*RPL39L*	1.7014	-1.2411	-1.2663	-1.3068	-0.27
16098	*CHRNB1*	0.60357	0.016186	-1.4269	-1.5671	-0.27
16099	*PRIM2*	0.63873	-0.60717	-0.84042	-1.3542	-0.27
16100	*TPST1*	0.43282	-0.06809	-1.1743	-2.2704	-0.27
16101	*CPSF3*	1.3932	-0.17653	-2.0277	-2.3795	-0.27
16102	*UCHL3*	2.0351	-1.0685	-1.7782	-1.8996	-0.27
16103	*HMGCS1*	0.022717	-0.40693	-0.42743	-1.6278	-0.27
16104	*ZSCAN12*	0.42414	-0.31916	-0.9168	-1.8707	-0.27
16105	*SLC35D2*	1.1909	-0.84419	-1.1591	-1.7266	-0.27
16106	*MCOLN3*	0.93056	-0.3744	-1.3694	-1.8435	-0.27
16107	*CCDC94*	1.3059	-0.65873	-1.4613	-1.9655	-0.27
16108	*COL13A1*	0.10453	-0.06896	-0.85006	-1.2699	-0.27
16109	*TAOK1*	0.90484	-0.81778	-0.90411	-1.1914	-0.27
16110	*CLPP*	1.2712	-1.0425	-1.0467	-1.8856	-0.27
16111	*CGN*	0.69256	-0.20778	-1.3058	-1.3199	-0.27
16112	*TG*	0.4261	0.31889	-1.5671	-1.6796	-0.27
16113	*CA7*	0.26451	-0.38425	-0.70391	-1.0091	-0.27
16114	*C10orf55*	0.094966	-0.44133	-0.47739	-2.1648	-0.27
16115	*DDX46*	0.5061	-0.32637	-1.0044	-1.5101	-0.27
16116	*MS4A6A*	1.2187	-0.78229	-1.2616	-1.8975	-0.28
16117	*SPZ1*	0.38954	-0.0227	-1.1945	-2.1438	-0.28
16118	*CFAP20*	1.004	-0.91388	-0.91807	-1.8538	-0.28
16119	*MT1A*	0.25848	-0.04149	-1.0452	-2.0323	-0.28
16120	*PI3*	0.066909	-0.2735	-0.62213	-2.3366	-0.28
16121	*CAPN14*	0.19282	0.047417	-1.0691	-1.7134	-0.28
16122	*VPS9D1*	0.80717	-0.12421	-1.5121	-3.0804	-0.28
16123	*UBA3*	1.234	-0.75934	-1.3042	-2.1113	-0.28
16124	*PCNA*	0.39081	-0.53619	-0.68439	-1.0252	-0.28
16125	*UBIAD1*	0.89745	-0.58229	-1.1453	-1.6216	-0.28
16126	*RNF7*	0.39738	-0.0464	-1.1812	-2.5655	-0.28
16127	*TUBA3E*	0.75365	-0.73853	-0.8458	-2.3601	-0.28
16128	*EPB42*	0.43061	-0.15339	-1.1084	-1.7486	-0.28
16129	*UFM1*	0.62144	-0.5635	-0.8892	-2.2485	-0.28
16130	*C22orf34*	1.11	-0.28464	-1.659	-1.7766	-0.28
16131	*SIRPG*	-0.16518	-0.20106	-0.46755	-1.8782	-0.28
16132	*GLYCTK*	-0.12922	-0.3485	-0.36106	-0.46769	-0.28
16133	*KALRN*	0.23309	0.17311	-1.245	-1.4998	-0.28
16134	*ST6GAL2*	0.023344	-0.24441	-0.61779	-1.2705	-0.28
16135	*TMEM132B*	0.39912	0.19494	-1.4333	-2.2021	-0.28
16136	*FIP1L1*	0.1939	-0.02913	-1.0046	-1.8683	-0.28
16137	*TMEM41B*	0.18536	-0.10664	-0.91921	-1.6678	-0.28
16138	*DKK4*	1.1594	-0.95802	-1.0421	-1.1027	-0.28
16139	*TROAP*	0.65949	-0.50555	-0.99474	-2.7852	-0.28

16140	*SEPT4*	0.79577	-0.49777	-1.1393	-1.7959	-0.28
16141	*ASB6*	1.3646	-0.92375	-1.2822	-4.0662	-0.28
16142	*FBN2*	-0.11327	-0.28588	-0.44353	-1.773	-0.28
16143	*SLX1A*	0.63031	0.1959	-1.6693	-2.012	-0.28
16144	*IL9*	0.3946	-0.22131	-1.0164	-1.3743	-0.28
16145	*WARS*	0.427	-0.44353	-0.82733	-2.0952	-0.28
16146	*CD101*	0.089763	-0.39313	-0.54177	-1.0016	-0.28
16147	*UBA7*	-0.14513	-0.24738	-0.45296	-1.0782	-0.28
16148	*TRAPPC8*	1.0382	-0.33139	-1.5524	-1.6105	-0.28
16149	*NUP98*	0.069589	-0.37658	-0.53957	-1.1349	-0.28
16150	*CSF3R*	0.25439	-0.27892	-0.82208	-0.97053	-0.28
16151	*CARD9*	0.72936	0.54151	-2.1179	-2.4322	-0.28
16152	*TSPAN14*	0.70459	-0.63953	-0.91335	-1.3574	-0.28
16153	*AKAP8L*	1.1002	-0.67146	-1.2786	-1.448	-0.28
16154	*AGAP4*	0.80466	0.027886	-1.683	-2.4174	-0.28
16155	*WASL*	-0.01504	-0.20103	-0.63572	-1.584	-0.28
16156	*C10orf111*	0.42428	0.23329	-1.5099	-1.5485	-0.28
16157	*RBM28*	0.2824	-0.3316	-0.80322	-1.0695	-0.28
16158	*FOXN4*	0.68979	-0.12054	-1.4218	-1.445	-0.28
16159	*MMP21*	2.1152	-1.2297	-1.739	-1.908	-0.28
16160	*MME*	0.16268	-0.23484	-0.78229	-2.1505	-0.28
16161	*LOH12CR1*	1.1221	-0.56053	-1.4174	-1.8768	-0.29
16162	*SF3A3*	0.76029	-0.32815	-1.2884	-1.5663	-0.29
16163	*TEX10*	0.80788	-0.32087	-1.3433	-1.6469	-0.29
16164	*PRND*	0.29464	-0.12446	-1.0273	-1.7306	-0.29
16165	*C2orf61*	0.75467	0.32461	-1.9367	-2.5852	-0.29
16166	*FBXO9*	0.54251	-0.66616	-0.73389	-0.91944	-0.29
16167	*DEFB118*	0.38683	-0.42004	-0.82435	-1.3976	-0.29
16168	*VOPP1*	1.2857	-0.42671	-1.7179	-2.7465	-0.29
16169	*GPC4*	0.27286	-0.5596	-0.57235	-1.0486	-0.29
16170	*CIDEB*	0.89964	-0.84659	-0.91392	-1.9065	-0.29
16171	*USP37*	0.56773	-0.54872	-0.88008	-0.98468	-0.29
16172	*CCK*	0.47661	-0.4849	-0.85558	-1.7911	-0.29
16173	*PLEKHJ1*	0.29955	-0.57899	-0.58542	-0.63255	-0.29
16174	*SPIC*	0.92462	-0.77418	-1.0156	-1.1797	-0.29
16175	*SFSWAP*	1.258	-0.9374	-1.187	-1.9075	-0.29
16176	*PAXBP1*	0.37296	-0.32059	-0.91886	-1.1756	-0.29
16177	*NPIPA1*	0.51436	0.31379	-1.6952	-1.7332	-0.29
16178	*C1orf53*	1.7798	-0.91921	-1.729	-1.9404	-0.29
16179	*SNAP23*	1.4044	-0.46757	-1.8057	-2.3118	-0.29
16180	*TAMM41*	1.184	-0.97454	-1.0793	-1.5415	-0.29
16181	*MSTO1*	0.23329	-0.37282	-0.73202	-1.9326	-0.29
16182	*LOC100129216*	-0.01438	-0.33511	-0.52269	-1.7552	-0.29
16183	*IRF7*	0.37558	-0.12408	-1.1241	-1.4299	-0.29
16184	*CRIP3*	1.924	-1.3288	-1.4688	-2.9977	-0.29
16185	*MAP1B*	0.18932	-0.14536	-0.91779	-1.8487	-0.29

16186	*SLC46A3*	0.41231	-0.32193	-0.96422	-2.907	-0.29
16187	*HSD17B7*	-0.01125	-0.41987	-0.44283	-1.4702	-0.29
16188	*COLEC11*	1.2991	-0.88898	-1.285	-1.676	-0.29
16189	*ZHX3*	0.57416	-0.60083	-0.85002	-1.2792	-0.29
16190	*LRIT3*	1.0701	-0.78651	-1.1608	-2.224	-0.29
16191	*PARG*	0.39868	-0.44855	-0.82757	-2.5725	-0.29
16192	*FYTTD1*	0.028892	-0.01088	-0.8956	-0.9797	-0.29
16193	*MEOX2*	-0.09032	-0.21974	-0.56753	-0.61999	-0.29
16194	*AQP11*	2.4178	-1.3955	-1.8999	-2.1294	-0.29
16195	*KCNAB1*	0.46044	0.08661	-1.426	-1.6984	-0.29
16196	*OTOL1*	0.032952	-0.12664	-0.78812	-1.0088	-0.29
16197	*CDK19*	0.62164	-0.7162	-0.78754	-1.1183	-0.29
16198	*GEMIN6*	0.23955	-0.05944	-1.0626	-3.1317	-0.29
16199	*FAM110D*	0.81061	-0.78705	-0.90635	-2.1816	-0.29
16200	*TMC3*	1.0165	-0.93712	-0.96225	-1.15	-0.29
16201	*FAM222A*	0.95631	-0.89167	-0.94797	-1.9175	-0.29
16202	*BCKDHB*	0.68522	-0.24488	-1.3237	-2.0872	-0.29
16203	*C2CD4D*	0.79175	-0.82663	-0.84903	-1.5637	-0.29
16204	*KIF26A*	0.36658	-0.27566	-0.97488	-2.1398	-0.29
16205	*DBI*	0.33951	-0.00827	-1.2159	-1.6913	-0.29
16206	*ENTPD4*	0.36048	-0.16202	-1.0838	-1.1002	-0.30
16207	*VSTM2B*	1.7298	-0.93005	-1.6855	-3.2873	-0.30
16208	*CENPBD1*	0.44354	-0.50419	-0.82547	-2.0541	-0.30
16209	*UBE2B*	0.12823	-0.43385	-0.58143	-0.77597	-0.30
16210	*TMEM55A*	2.8225	-1.4767	-2.2329	-2.2385	-0.30
16211	*BOLA3*	0.48714	-0.44353	-0.93107	-2.6208	-0.30
16212	*BTNL10*	0.84346	-0.52017	-1.2114	-1.6089	-0.30
16213	*NPM2*	0.19529	-0.52552	-0.5584	-1.3785	-0.30
16214	*TOLLIP*	0.012276	-0.11781	-0.78439	-1.244	-0.30
16215	*CLSTN1*	1.1087	-0.78229	-1.2171	-2.0534	-0.30
16216	*NOP10*	0.18216	-0.28158	-0.79409	-2.0305	-0.30
16217	*NDUFB8*	0.45939	-0.25181	-1.1012	-1.5824	-0.30
16218	*CLGN*	1.4088	-0.72553	-1.578	-1.6635	-0.30
16219	*PROX2*	1.6064	-1.0094	-1.4919	-2.4703	-0.30
16220	*IL21R*	1.348	-1.0801	-1.163	-1.6007	-0.30
16221	*KIAA2012*	0.83416	-0.84446	-0.88702	-1.3677	-0.30
16222	*EIF3G*	0.99046	-0.53868	-1.35	-1.5962	-0.30
16223	*LRRK2*	0.044791	-0.35379	-0.58994	-1.6943	-0.30
16224	*RNF13*	0.29824	-0.43077	-0.76671	-0.80641	-0.30
16225	*ARHGAP40*	0.61785	-0.56251	-0.95469	-2.101	-0.30
16226	*SLC39A11*	0.22467	-0.44185	-0.68228	-1.0761	-0.30
16227	*SNRNP35*	1.539	-0.57515	-1.8636	-2.8687	-0.30
16228	*FAM76A*	0.28149	0.21653	-1.3978	-1.7129	-0.30
16229	*OR52N4*	0.7011	-0.20338	-1.398	-2.8521	-0.30
16230	*SFN*	0.80855	0.67632	-2.3858	-3.3598	-0.30
16231	*ZNF71*	0.85867	-0.28384	-1.4773	-1.5901	-0.30

16232	*SWSAP1*	1.1552	-0.32286	-1.7352	-2.8702	-0.30
16233	*ZNF565*	0.11164	-0.36295	-0.65163	-1.5073	-0.30
16234	*DYNLL1*	0.15852	-0.38796	-0.67458	-1.3183	-0.30
16235	*SACM1L*	0.3445	0.023658	-1.2728	-1.8587	-0.30
16236	*CPXCR1*	0.14757	-0.45455	-0.59829	-2.1363	-0.30
16237	*KIR2DS4*	-0.19996	-0.21868	-0.48673	-2.0698	-0.30
16238	*AGAP5*	0.092716	0.063089	-1.0614	-1.2288	-0.30
16239	*JDP2*	0.1468	0.090093	-1.1442	-2.0303	-0.30
16240	*GFI1*	0.15006	-0.26008	-0.79744	-1.0385	-0.30
16241	*LYPD2*	0.82269	-0.28524	-1.4452	-1.5667	-0.30
16242	*STAB2*	0.53673	-0.20621	-1.2383	-2.4642	-0.30
16243	*POLR3C*	0.77012	-0.44353	-1.2352	-1.4567	-0.30
16244	*PRRX2*	0.50404	0.018621	-1.4319	-1.7829	-0.30
16245	*HIBCH*	1.1873	-0.95423	-1.1426	-1.4509	-0.30
16246	*DUSP3*	0.34343	-0.4599	-0.79357	-0.96997	-0.30
16247	*DPPA4*	1.2368	-0.43694	-1.7113	-1.7595	-0.30
16248	*PSD4*	0.11596	-0.44935	-0.57825	-3.596	-0.30
16249	*SRGAP2*	1.358	-1.0452	-1.2246	-2.5513	-0.30
16250	*POTEB*	1.0218	-0.93878	-0.99508	-1.0739	-0.30
16251	*HIGD2A*	-0.01823	-0.28628	-0.60781	-1.2806	-0.30
16252	*PDSS2*	-0.0813	-0.19706	-0.63542	-1.7493	-0.30
16253	*C7orf72*	0.098451	-0.08213	-0.93011	-2.0247	-0.30
16254	*CCNH*	0.3998	0.21744	-1.5312	-2.0301	-0.30
16255	*PRR20C*	2.2323	-1.3973	-1.7493	-1.8363	-0.30
16256	*MICALCL*	0.38855	0.00962	-1.3134	-3.0112	-0.31
16257	*EMC8*	0.38037	-0.46757	-0.82928	-1.5737	-0.31
16258	*SAA1*	0.86319	-0.40749	-1.3727	-1.4299	-0.31
16259	*ISG20L2*	0.062111	0.008065	-0.98719	-1.1848	-0.31
16260	*HM13*	2.5037	-1.6477	-1.7734	-1.7994	-0.31
16261	*WWOX*	0.62769	-0.45403	-1.0912	-1.7438	-0.31
16262	*GDF2*	0.62919	-0.15145	-1.3955	-1.8154	-0.31
16263	*ACTN4*	0.53258	-0.14193	-1.309	-1.5633	-0.31
16264	*CXCL12*	0.074609	-0.16382	-0.82959	-1.3836	-0.31
16265	*SRSF3*	0.4521	-0.26518	-1.1061	-3.266	-0.31
16266	*SFPQ*	0.20665	-0.48744	-0.64149	-0.93399	-0.31
16267	*RTP2*	0.24514	-0.56701	-0.60147	-2.9736	-0.31
16268	*OR4K15*	1.8524	-1.0474	-1.7297	-2.5535	-0.31
16269	*ZNF185*	0.088767	0.087566	-1.1016	-1.8572	-0.31
16270	*ANXA11*	0.54013	-0.63172	-0.83402	-1.8478	-0.31
16271	*C11orf57*	0.73895	-0.49266	-1.1727	-1.8095	-0.31
16272	*TMEM106C*	0.75771	-0.42766	-1.2568	-1.3008	-0.31
16273	*COMT*	-0.04927	-0.08284	-0.79467	-2.4328	-0.31
16274	*DET1*	0.94612	-0.81555	-1.0576	-1.4951	-0.31
16275	*RING1*	0.50349	0.33404	-1.7653	-1.7865	-0.31
16276	*WRNIP1*	1.1212	-0.55992	-1.4893	-2.528	-0.31
16277	*SIGLEC6*	2.2677	-1.3892	-1.8076	-2.2125	-0.31

16278	*EXOC3*	0.12995	-0.51152	-0.54829	-1.2418	-0.31
16279	*EIF2AK3*	0.47721	-0.29304	-1.1145	-1.9494	-0.31
16280	*HOXB4*	0.043861	0.016744	-0.9925	-1.7215	-0.31
16281	*TAS2R9*	0.55792	-0.49926	-0.99175	-1.0952	-0.31
16282	*RPL3*	0.05102	-0.16927	-0.81501	-1.3593	-0.31
16283	*SLC35D1*	0.53795	-0.72095	-0.7512	-2.1732	-0.31
16284	*SPDEF*	0.2878	-0.52914	-0.69287	-2.1151	-0.31
16285	*DCTN5*	0.056779	-0.41922	-0.5718	-1.4642	-0.31
16286	*ADH1B*	0.6399	-0.78444	-0.79065	-1.7649	-0.31
16287	*SPRED2*	0.58614	-0.27088	-1.251	-2.2879	-0.31
16288	*TMEM252*	0.63528	0.37603	-1.9484	-2.6113	-0.31
16289	*DHODH*	1.0293	-0.86941	-1.0989	-1.1496	-0.31
16290	*RPS29*	1.4184	-0.76955	-1.5886	-1.9334	-0.31
16291	*FANCD2*	-0.1851	-0.37185	-0.38296	-0.57564	-0.31
16292	*ITGA3*	-0.02404	-0.32638	-0.58971	-2.0667	-0.31
16293	*RFX6*	1.8289	-1.3008	-1.4686	-1.9624	-0.31
16294	*SAMD13*	0.54936	0.16073	-1.6523	-1.9495	-0.31
16295	*USP17L5*	-0.1096	-0.38996	-0.44277	-1.2684	-0.31
16296	*SH3BP5*	0.089278	0.031743	-1.0682	-1.1592	-0.32
16297	*PSD2*	0.78013	-0.50251	-1.2249	-1.5608	-0.32
16298	*ASCC2*	0.45883	-0.21182	-1.1943	-1.2432	-0.32
16299	*DXO*	0.18719	-0.56769	-0.56841	-2.5292	-0.32
16300	*MEGF10*	2.3141	-1.5227	-1.7404	-1.9891	-0.32
16301	*ERF*	0.4139	-0.43473	-0.92828	-1.3074	-0.32
16302	*SPRY1*	-0.16294	-0.34327	-0.44353	-0.87858	-0.32
16303	*PRPF31*	-0.12067	-0.16438	-0.66569	-1.0594	-0.32
16304	*IK*	-0.12659	-0.36371	-0.46107	-1.4792	-0.32
16305	*COLCA2*	0.90934	-0.50067	-1.3604	-1.6754	-0.32
16306	*TNFRSF25*	-0.00621	-0.056	-0.88987	-0.94897	-0.32
16307	*GLTSCR1L*	-0.01742	-0.19943	-0.73961	-2.5795	-0.32
16308	*NR0B2*	0.069884	-0.1468	-0.87997	-1.2317	-0.32
16309	*C9orf92*	0.446	-0.29076	-1.1132	-1.8508	-0.32
16310	*ZCCHC12*	1.5675	-1.232	-1.2935	-1.9673	-0.32
16311	*RHOB*	0.54804	-0.55586	-0.95045	-1.5996	-0.32
16312	*SERINC3*	0.58504	-0.57899	-0.96492	-2.4405	-0.32
16313	*ITPA*	1.2984	-0.50588	-1.7517	-1.7663	-0.32
16314	*DEFB114*	1.6823	-0.84713	-1.7945	-1.9686	-0.32
16315	*STAU1*	-0.11035	-0.39579	-0.45323	-1.8534	-0.32
16316	*TMEM65*	-0.11164	-0.2875	-0.5605	-1.8275	-0.32
16317	*PTGES3*	-0.16321	-0.33444	-0.46236	-0.47645	-0.32
16318	*BICD2*	1.2281	-1.0596	-1.1309	-1.4498	-0.32
16319	*NAPRT*	0.46439	-0.60604	-0.82098	-1.8297	-0.32
16320	*MLLT3*	0.42665	-0.45403	-0.93555	-1.1886	-0.32
16321	*GPD2*	0.62605	-0.64268	-0.94723	-1.5059	-0.32
16322	*ITGA4*	0.71846	-0.74774	-0.93652	-0.99529	-0.32
16323	*CCDC155*	0.47086	0.15303	-1.5898	-2.0218	-0.32

16324	*CT47A6*	0.1695	-0.21031	-0.92553	-1.7076	-0.32
16325	*CHURC1*	-0.11527	-0.36451	-0.48821	-2.3096	-0.32
16326	*TMC7*	0.25617	-0.394	-0.8303	-1.7068	-0.32
16327	*EDEM2*	0.52335	-0.01629	-1.4752	-1.7743	-0.32
16328	*EFNA4*	0.080715	-0.01439	-1.035	-1.4841	-0.32
16329	*RFC2*	0.10367	-0.4216	-0.65075	-1.0089	-0.32
16330	*ACACA*	-0.06884	-0.20886	-0.69286	-1.3299	-0.32
16331	*CD200R1L*	1.0493	-0.36035	-1.6604	-1.9681	-0.32
16332	*GOLGA6L1*	0.56217	-0.62493	-0.9101	-1.6525	-0.32
16333	*HPCA*	1.2028	-0.72566	-1.4509	-1.7753	-0.32
16334	*HIST3H2BB*	0.8907	-0.37376	-1.4909	-1.8804	-0.32
16335	*GLI2*	0.82348	-0.11406	-1.6836	-2.1419	-0.32
16336	*VPS37D*	0.76556	-0.39214	-1.3478	-1.8724	-0.32
16337	*ZKSCAN4*	1.8963	-1.2264	-1.6443	-2.1502	-0.32
16338	*HYPM*	0.69988	-0.73111	-0.94345	-1.539	-0.32
16339	*C21orf59*	2.3762	-1.3235	-2.0277	-2.2766	-0.33
16340	*TAX1BP3*	0.28617	-0.54219	-0.71939	-1.8087	-0.33
16341	*UNC50*	0.43325	-0.39883	-1.0099	-1.5125	-0.33
16342	*SLAMF8*	3.551	-2.1984	-2.3296	-2.4022	-0.33
16343	*CASP9*	0.15435	-0.33709	-0.79444	-1.5181	-0.33
16344	*CHST7*	1.5139	-1.1439	-1.3492	-1.9794	-0.33
16345	*TERF1*	0.55396	0.055556	-1.589	-2.6289	-0.33
16346	*CCAR1*	1.1428	-0.72582	-1.3971	-1.5676	-0.33
16347	*NUP160*	0.008382	-0.40897	-0.58131	-1.925	-0.33
16348	*KREMEN2*	0.23097	-0.4055	-0.80823	-1.5235	-0.33
16349	*KRT26*	0.079734	-0.32308	-0.73944	-0.92427	-0.33
16350	*ITSN2*	0.20545	-0.37888	-0.80946	-1.0773	-0.33
16351	*TRPM7*	0.68587	-0.06007	-1.6089	-2.4328	-0.33
16352	*MRPS11*	0.79156	-0.86324	-0.91215	-1.6586	-0.33
16353	*CCR4*	0.56934	-0.14697	-1.4087	-1.696	-0.33
16354	*TADA1*	0.087154	-0.38265	-0.69102	-1.2443	-0.33
16355	*ZFYVE26*	0.18281	-0.38655	-0.78313	-1.7718	-0.33
16356	*CHTF8*	0.73049	-0.8424	-0.8759	-1.0501	-0.33
16357	*KRTAP5-5*	-0.09512	-0.43526	-0.45854	-1.2867	-0.33
16358	*OVGP1*	0.008624	-0.3521	-0.64546	-1.3176	-0.33
16359	*NR2C2*	-0.06694	-0.26246	-0.6603	-1.9653	-0.33
16360	*ATP5A1*	-0.20499	-0.33404	-0.45069	-0.57273	-0.33
16361	*CMTM2*	2.2596	-1.6034	-1.6463	-1.9041	-0.33
16362	*TIGD5*	0.47045	0.21577	-1.677	-1.7688	-0.33
16363	*SET*	0.42171	-0.42808	-0.9858	-2.024	-0.33
16364	*NEDD8*	-0.10255	-0.36824	-0.52238	-1.5632	-0.33
16365	*PCED1B*	0.25096	-0.58771	-0.65668	-0.88695	-0.33
16366	*PHF23*	0.83593	-0.52587	-1.305	-1.5635	-0.33
16367	*KAT8*	1.0472	-0.86725	-1.1754	-2.2044	-0.33
16368	*CHRAC1*	1.0312	-0.43113	-1.5971	-2.9538	-0.33
16369	*COIL*	0.23395	-0.39606	-0.83505	-1.9245	-0.33

16370	*NTN3*	0.9446	-0.71772	-1.2244	-1.6712	-0.33
16371	*SEC24D*	0.44099	-0.26186	-1.1771	-1.8453	-0.33
16372	*GTF2E2*	0.4496	-0.08887	-1.3591	-1.624	-0.33
16373	*FANCC*	0.14948	-0.0304	-1.1184	-1.3098	-0.33
16374	*SLFNL1*	-0.09229	-0.11023	-0.79744	-2.264	-0.33
16375	*RBMY1J*	0.5728	0.23438	-1.8087	-2.0593	-0.33
16376	*ZEB2*	1.4096	-0.06005	-2.3517	-3.1229	-0.33
16377	*ZP3*	0.26956	-0.6093	-0.66242	-1.6246	-0.33
16378	*RIMS4*	0.18994	-0.01705	-1.1758	-1.251	-0.33
16379	*DNAJC11*	0.91916	-0.71819	-1.204	-2.425	-0.33
16380	*OR6C3*	0.84519	-0.82978	-1.0197	-1.5118	-0.33
16381	*SYNJ2BP*	-0.31531	-0.33136	-0.35891	-0.77678	-0.34
16382	*CLEC18C*	2.4771	-1.6885	-1.7945	-2.3876	-0.34
16383	*SERPINC1*	-0.09459	-0.32087	-0.59061	-1.205	-0.34
16384	*TTYH2*	0.38532	-0.35537	-1.0361	-1.463	-0.34
16385	*C10orf128*	0.69415	-0.13984	-1.5608	-1.5651	-0.34
16386	*KPNA4*	0.90732	-0.53767	-1.3766	-1.9811	-0.34
16387	*CYB5D2*	0.31268	-0.57691	-0.74308	-1.8402	-0.34
16388	*TMEM164*	0.5662	-0.22577	-1.3481	-1.8996	-0.34
16389	*TEX261*	1.7441	-1.3078	-1.4442	-1.6065	-0.34
16390	*TLR5*	0.44955	-0.63643	-0.82368	-1.2049	-0.34
16391	*GIMAP5*	0.78922	-0.57513	-1.2261	-1.3296	-0.34
16392	*ZNF250*	0.16588	-0.27152	-0.90663	-1.3097	-0.34
16393	*DDI1*	0.43912	-0.55844	-0.89351	-2.5146	-0.34
16394	*HOXA4*	0.68151	-0.37913	-1.3153	-1.626	-0.34
16395	*EOMES*	0.34044	-0.22588	-1.1283	-1.5394	-0.34
16396	*AP2M1*	2.0497	-1.485	-1.5787	-1.6443	-0.34
16397	*SUPT5H*	0.15694	-0.39272	-0.77828	-1.468	-0.34
16398	*CYP8B1*	1.0372	-0.29759	-1.7538	-2.4013	-0.34
16399	*MAP1LC3C*	0.84074	-0.72488	-1.1316	-1.5817	-0.34
16400	*RNF144B*	0.30391	-0.48564	-0.83471	-1.3032	-0.34
16401	*VCAN*	0.25826	-0.61138	-0.66353	-0.93489	-0.34
16402	*FYN*	1.8472	-0.72348	-2.1412	-2.5725	-0.34
16403	*USP51*	0.27655	-0.56769	-0.7264	-2.033	-0.34
16404	*DYRK3*	0.23111	0.073963	-1.3232	-2.0629	-0.34
16405	*GTF2I*	0.84841	-0.81448	-1.0529	-1.4741	-0.34
16406	*NKAP*	0.49801	-0.066	-1.451	-1.7718	-0.34
16407	*IFNLR1*	0.86516	-0.8606	-1.0243	-2.4885	-0.34
16408	*GNGT1*	0.13498	0.048059	-1.204	-1.5524	-0.34
16409	*UBE2T*	-0.21502	-0.33961	-0.46768	-0.70335	-0.34
16410	*MASP2*	0.62656	-0.2732	-1.3758	-1.5884	-0.34
16411	*BYSL*	0.51887	-0.19658	-1.3453	-5.4774	-0.34
16412	*ATP6V1B1*	0.95235	-0.76762	-1.2079	-1.4428	-0.34
16413	*PSMA6*	0.56585	-0.66865	-0.92078	-3.0638	-0.34
16414	*LRRFIP2*	0.22714	-0.56154	-0.69006	-0.74743	-0.34
16415	*ILF2*	0.71371	-0.75586	-0.98359	-1.6251	-0.34

16416	*RHBDL2*	0.57229	-0.726	-0.87318	-1.2352	-0.34
16417	*FNTA*	0.2663	0.048673	-1.3427	-2.1577	-0.34
16418	*UACA*	0.28485	-0.55909	-0.75511	-1.5952	-0.34
16419	*GJD2*	0.14992	-0.30427	-0.87536	-1.9461	-0.34
16420	*ALKBH1*	0.22992	-0.61092	-0.64888	-1.9166	-0.34
16421	*ELL*	0.065253	-0.25295	-0.84291	-2.0423	-0.34
16422	*AHI1*	-0.10031	-0.43906	-0.49314	-1.3978	-0.34
16423	*PITX1*	0.69518	-0.01401	-1.714	-2.0949	-0.34
16424	*MAN1A1*	-0.07219	-0.25056	-0.71063	-3.7736	-0.34
16425	*KIAA0895*	1.3427	-1.1851	-1.1914	-1.2867	-0.34
16426	*MS4A12*	0.66479	-0.60717	-1.0918	-1.806	-0.34
16427	*UNC93A*	0.79216	-0.64016	-1.1867	-1.3361	-0.34
16428	*DCST2*	0.070673	-0.20987	-0.8959	-0.99059	-0.35
16429	*DNAL4*	0.50078	0.35177	-1.8885	-2.1523	-0.35
16430	*OR2M3*	0.27103	-0.06606	-1.2411	-2.404	-0.35
16431	*NUP210*	0.093548	-0.18996	-0.94113	-1.5894	-0.35
16432	*TPK1*	0.3057	-0.25261	-1.0907	-1.7646	-0.35
16433	*MARCH10*	0.27469	-0.26036	-1.0528	-1.3178	-0.35
16434	*ESR1*	0.19262	-0.38898	-0.84563	-1.4689	-0.35
16435	*UBA5*	0.63153	-0.29713	-1.377	-1.6262	-0.35
16436	*SLC30A8*	0.83543	-0.45214	-1.4261	-1.8702	-0.35
16437	*DND1*	0.55843	0.42767	-2.0303	-2.2044	-0.35
16438	*F5*	-0.00834	-0.16195	-0.87418	-1.072	-0.35
16439	*C14orf159*	0.36517	-0.67702	-0.73288	-1.3766	-0.35
16440	*PHACTR3*	0.31269	-0.53345	-0.82573	-1.5688	-0.35
16441	*HMOX2*	2.2878	-1.635	-1.701	-2.2007	-0.35
16442	*ANKEF1*	0.27293	-0.14918	-1.1743	-1.4734	-0.35
16443	*TRMT11*	-0.22097	-0.33754	-0.49212	-1.0863	-0.35
16444	*SLC22A8*	0.31543	-0.00331	-1.364	-3.0246	-0.35
16445	*FGF9*	0.086929	-0.09384	-1.0467	-2.9031	-0.35
16446	*ZNF585A*	-0.23796	-0.26237	-0.55387	-1.1383	-0.35
16447	*COA7*	-0.07018	-0.11162	-0.87318	-2.1108	-0.35
16448	*POP4*	0.38783	0.2086	-1.6524	-1.9099	-0.35
16449	*CYFIP2*	0.49205	-0.49953	-1.0489	-1.1207	-0.35
16450	*PHF12*	0.19752	-0.07341	-1.181	-1.7507	-0.35
16451	*COX7B*	-0.01088	-0.03572	-1.0107	-1.5454	-0.35
16452	*HADH*	1.005	-0.88159	-1.1821	-1.5524	-0.35
16453	*NDNF*	0.56345	0.19927	-1.8219	-2.3509	-0.35
16454	*GNG2*	0.48883	-0.62523	-0.92415	-0.99834	-0.35
16455	*CCDC114*	0.43264	0.18259	-1.6759	-1.6921	-0.35
16456	*PLA2G4F*	0.116	-0.08253	-1.0944	-2.1085	-0.35
16457	*COQ7*	0.1582	0.11813	-1.3378	-1.658	-0.35
16458	*TEFM*	0.44887	-0.66604	-0.84627	-1.0645	-0.35
16459	*FOXG1*	0.73058	-0.58752	-1.2071	-1.5679	-0.35
16460	*MRPL34*	0.11219	-0.42634	-0.75092	-0.99313	-0.36
16461	*VPS26A*	0.035695	-0.29549	-0.80542	-1.5073	-0.36

16462	*RNF213*	0.12019	-0.26273	-0.92288	-1.6371	-0.36
16463	*MUS81*	0.55956	-0.78963	-0.83692	-0.97053	-0.36
16464	*C5orf45*	0.71045	-0.45569	-1.3218	-1.626	-0.36
16465	*SUGT1*	0.44675	-0.49408	-1.0198	-1.716	-0.36
16466	*AGMO*	0.17128	-0.38296	-0.85594	-1.3292	-0.36
16467	*CERS1*	1.3549	-1.2015	-1.2214	-1.6804	-0.36
16468	*BTBD11*	0.21441	-0.0536	-1.229	-1.6283	-0.36
16469	*CEBPZ*	0.30216	0.28096	-1.6525	-1.7513	-0.36
16470	*TUFM*	0.69659	-0.59157	-1.175	-2.0553	-0.36
16471	*GOLGA3*	0.45442	-0.36914	-1.1553	-1.2145	-0.36
16472	*ZACN*	0.95277	-0.47338	-1.5507	-2.1532	-0.36
16473	*RIC8A*	0.813	-0.79453	-1.0898	-1.584	-0.36
16474	*FAM221A*	-0.05201	-0.27903	-0.74141	-1.5383	-0.36
16475	*TNFRSF10D*	0.5805	-0.21796	-1.4361	-1.8714	-0.36
16476	*C19orf25*	0.57154	-0.60235	-1.043	-3.4256	-0.36
16477	*MTUS1*	-0.04425	-0.17517	-0.85513	-0.9904	-0.36
16478	*TUBGCP4*	-0.15978	-0.18791	-0.72723	-1.1687	-0.36
16479	*DLK1*	0.33602	-0.17606	-1.2349	-1.8636	-0.36
16480	*TSPY2*	0.99812	-0.65012	-1.4233	-1.7188	-0.36
16481	*PCDHB4*	-0.16202	-0.36047	-0.55302	-1.1472	-0.36
16482	*SNX7*	0.35388	-0.27423	-1.1553	-1.4775	-0.36
16483	*SCAMP2*	0.30373	-0.13571	-1.2439	-2.3915	-0.36
16484	*ID1*	0.41353	0.06977	-1.5598	-1.9326	-0.36
16485	*ATAD3B*	0.029089	-0.43854	-0.66731	-1.2301	-0.36
16486	*TEAD2*	-0.11216	-0.27672	-0.68813	-2.6208	-0.36
16487	*ATAT1*	0.26733	-0.53712	-0.80771	-1.3148	-0.36
16488	*SHOC2*	0.71674	-0.83784	-0.95708	-1.4448	-0.36
16489	*ZC3H10*	0.22414	-0.56079	-0.74192	-0.98642	-0.36
16490	*LINGO2*	0.8205	-0.15499	-1.7445	-1.9041	-0.36
16491	*GPR132*	0.19852	-0.14348	-1.1343	-3.3935	-0.36
16492	*BAG6*	1.0085	-0.98452	-1.1034	-2.1952	-0.36
16493	*PTGR2*	0.36257	-0.07235	-1.3701	-1.9107	-0.36
16494	*RBMS3*	0.35979	-0.57787	-0.86398	-1.7079	-0.36
16495	*RYR1*	1.5512	-1.0819	-1.5524	-2.6376	-0.36
16496	*ACSM6*	1.3901	-1.1169	-1.3565	-1.7061	-0.36
16497	*ABCF1*	-0.10772	-0.1258	-0.84992	-2.1577	-0.36
16498	*CLOCK*	0.70356	-0.69898	-1.0886	-1.7517	-0.36
16499	*STAG1*	0.037844	0.019274	-1.1412	-1.8004	-0.36
16500	*ANO10*	0.039166	-0.09689	-1.0273	-1.6422	-0.36
16501	*SLC25A43*	1.7958	-1.3127	-1.5684	-1.9757	-0.36
16502	*SCUBE3*	0.15149	-0.51405	-0.72304	-0.8728	-0.36
16503	*DHX38*	0.30207	-0.19268	-1.1964	-1.4019	-0.36
16504	*PELI2*	1.2094	-1.0948	-1.2027	-1.8453	-0.36
16505	*S100A5*	0.32406	-0.68706	-0.726	-1.2935	-0.36
16506	*FTSJ1*	0.85651	-0.93217	-1.0142	-3.6472	-0.36
16507	*PPM1E*	0.016186	-0.46745	-0.63939	-3.5043	-0.36

16508	*RPS28*	0.18173	-0.55999	-0.71626	-1.1484	-0.36
16509	*ZNF70*	0.71892	-0.90542	-0.90824	-0.92689	-0.36
16510	*HADHA*	0.94774	-0.55387	-1.4894	-1.8822	-0.37
16511	*PVRL3*	0.61584	0.16661	-1.8789	-2.0748	-0.37
16512	*LOC391003*	0.40829	-0.70546	-0.79987	-1.0669	-0.37
16513	*TCF12*	-0.11965	-0.34144	-0.6362	-1.2644	-0.37
16514	*FAM110B*	0.56135	-0.68042	-0.97966	-1.9623	-0.37
16515	*CASQ1*	1.127	-0.8903	-1.3367	-1.9727	-0.37
16516	*DMPK*	0.48662	-0.79149	-0.79558	-1.3694	-0.37
16517	*NYNRIN*	0.33047	-0.29979	-1.1312	-1.3253	-0.37
16518	*PDCL3*	1.015	-0.93199	-1.1836	-1.2299	-0.37
16519	*C11orf40*	1.7282	-1.2924	-1.5373	-1.9689	-0.37
16520	*FBXO48*	3.349	-1.7462	-2.7063	-2.7546	-0.37
16521	*KRT9*	0.43544	-0.6828	-0.85622	-2.6028	-0.37
16522	*DCX*	0.33864	-0.48833	-0.95495	-1.0102	-0.37
16523	*ARHGAP5*	0.18649	-0.45119	-0.84001	-2.0871	-0.37
16524	*CHMP2A*	0.13558	-0.44353	-0.79983	-1.2367	-0.37
16525	*LIMK1*	0.1583	-0.26466	-1.0024	-1.4284	-0.37
16526	*CRKL*	0.21678	-0.0188	-1.3068	-1.85	-0.37
16527	*EN2*	-0.04526	-0.26717	-0.79711	-1.9358	-0.37
16528	*NFKB1*	-0.03262	-0.23306	-0.84563	-1.2407	-0.37
16529	*ACTA1*	0.59964	0.044746	-1.7567	-1.9125	-0.37
16530	*ZG16*	0.15852	-0.51713	-0.75665	-0.79632	-0.37
16531	*RGP1*	-0.19089	-0.30457	-0.62047	-1.3916	-0.37
16532	*ZNF579*	0.12481	0.000172	-1.241	-1.4994	-0.37
16533	*PLXDC1*	-0.09	-0.15953	-0.86821	-1.3892	-0.37
16534	*TSLP*	0.25678	-0.66215	-0.71257	-1.8238	-0.37
16535	*PHLDA2*	1.1919	-0.64725	-1.6653	-2.8181	-0.37
16536	*FAM110A*	0.044855	-0.23113	-0.93592	-2.1956	-0.37
16537	*MBD3*	1.2145	-0.67812	-1.6587	-2.4491	-0.37
16538	*ABI2*	0.48253	-0.39171	-1.2141	-1.3937	-0.37
16539	*PDS5B*	0.58937	-0.54097	-1.1717	-2.224	-0.37
16540	*FOXL1*	0.04883	-0.05031	-1.1223	-4.9979	-0.37
16541	*ARPC5L*	0.30141	-0.48042	-0.94493	-1.5744	-0.37
16542	*CCDC53*	0.07586	-0.44353	-0.75641	-1.7585	-0.37
16543	*SLA2*	0.80493	-0.85149	-1.079	-1.2356	-0.38
16544	*DZIP1*	0.59007	0.35402	-2.0697	-2.2456	-0.38
16545	*RCC1*	0.074528	-0.31976	-0.88062	-1.4883	-0.38
16546	*SLC17A3*	1.6209	-1.0005	-1.7483	-2.2021	-0.38
16547	*PHC1*	0.17695	0.11164	-1.4168	-1.8157	-0.38
16548	*ACTC1*	0.32666	-0.67889	-0.7771	-1.3292	-0.38
16549	*PSG1*	0.20832	-0.08719	-1.2514	-2.5865	-0.38
16550	*NSD1*	1.7076	-1.2936	-1.545	-1.6281	-0.38
16551	*RAB35*	0.59959	-0.85163	-0.87898	-1.0923	-0.38
16552	*FAM63B*	-0.00492	-0.17744	-0.95045	-1.635	-0.38
16553	*CSRNP2*	-0.16698	-0.44353	-0.52238	-1.0567	-0.38

16554	*DGCR6L*	0.2083	0.043977	-1.3864	-2.775	-0.38
16555	*ZDHHC4*	-0.25762	-0.41336	-0.46404	-1.5877	-0.38
16556	*EMILIN2*	0.075627	-0.3014	-0.9105	-1.7056	-0.38
16557	*IVL*	0.50612	-0.75408	-0.88876	-1.8856	-0.38
16558	*POLR2F*	1.1802	-0.48731	-1.8297	-1.9446	-0.38
16559	*HYAL4*	-0.02232	-0.55387	-0.56076	-1.3916	-0.38
16560	*CDKL2*	-0.20562	-0.43473	-0.49961	-0.60845	-0.38
16561	*DHTKD1*	0.48741	-0.71077	-0.91886	-1.078	-0.38
16562	*ZNF335*	0.4804	-0.46248	-1.1607	-1.9748	-0.38
16563	*SLC45A4*	-0.18019	-0.34751	-0.61544	-0.68813	-0.38
16564	*MYL12A*	0.5653	-0.21964	-1.4894	-2.0542	-0.38
16565	*TTC27*	0.53363	-0.82098	-0.85799	-1.062	-0.38
16566	*OR4S2*	0.35036	-0.7068	-0.79149	-1.9172	-0.38
16567	*ST6GAL1*	-0.2417	-0.37856	-0.52853	-4.1669	-0.38
16568	*USP47*	0.26788	-0.10743	-1.3101	-2.4703	-0.38
16569	*RHPN2*	0.048909	-0.55464	-0.64552	-1.8616	-0.38
16570	*CHRNA4*	0.39383	-0.04542	-1.4998	-3.5394	-0.38
16571	*SCRN2*	0.90583	-1.0141	-1.0436	-1.3686	-0.38
16572	*ITK*	0.56058	-0.55554	-1.1585	-1.4688	-0.38
16573	*NELFCD*	0.29314	-0.17208	-1.278	-2.2059	-0.39
16574	*DNPEP*	0.93649	-0.88199	-1.2124	-2.1085	-0.39
16575	*MACF1*	0.66286	-0.20621	-1.615	-2.1419	-0.39
16576	*USP7*	0.001688	-0.40714	-0.75328	-1.4859	-0.39
16577	*TICAM2*	-0.26537	-0.43398	-0.46032	-0.69841	-0.39
16578	*WDR25*	0.089784	-0.23977	-1.01	-1.1575	-0.39
16579	*TYK2*	0.19908	-0.33731	-1.0218	-1.6112	-0.39
16580	*DCP1A*	-0.10006	-0.5038	-0.55851	-2.4764	-0.39
16581	*TRAM2*	-0.01788	-0.23404	-0.91103	-0.98421	-0.39
16582	*RHOG*	0.87168	0.37459	-2.4094	-2.599	-0.39
16583	*EID2B*	0.31895	-0.4594	-1.0232	-2.2732	-0.39
16584	*CETN1*	0.87504	-0.70546	-1.3343	-1.4561	-0.39
16585	*WNT2B*	0.64372	-0.43587	-1.3738	-3.2172	-0.39
16586	*CCDC157*	0.081831	0.000461	-1.2486	-1.8963	-0.39
16587	*ZBTB5*	0.40405	-0.08446	-1.4864	-1.5212	-0.39
16588	*PSMA3*	0.37252	-0.08887	-1.4509	-1.6216	-0.39
16589	*FAM96B*	0.43982	-0.30496	-1.3033	-1.6463	-0.39
16590	*SLC19A3*	0.58119	-0.39817	-1.3523	-1.3878	-0.39
16591	*FEZ2*	-0.00301	-0.36761	-0.80048	-2.1198	-0.39
16592	*DDTL*	0.21931	-0.49056	-0.90001	-1.6492	-0.39
16593	*NPTX1*	0.93283	-0.24715	-1.8572	-2.4595	-0.39
16594	*CHORDC1*	-0.12421	-0.29292	-0.75472	-1.3808	-0.39
16595	*XCL2*	0.8875	0.36064	-2.4205	-2.4933	-0.39
16596	*FUT5*	0.27717	-0.69377	-0.75625	-1.584	-0.39
16597	*SLC24A3*	0.46554	-0.13563	-1.5031	-2.1794	-0.39
16598	*TLR7*	0.78114	-0.87673	-1.0782	-1.7507	-0.39
16599	*FAM21A*	1.9712	-1.3023	-1.8435	-2.4205	-0.39

16600	*CTC1*	1.1339	-0.46756	-1.842	-2.1295	-0.39
16601	*CDR2*	0.61677	-0.19234	-1.6004	-1.6246	-0.39
16602	*GPRIN1*	-0.06185	-0.15313	-0.9612	-2.0817	-0.39
16603	*HERPUD2*	0.6823	-0.76882	-1.09	-2.4917	-0.39
16604	*FDPS*	0.87211	-0.1427	-1.9065	-4.2477	-0.39
16605	*EGR2*	0.46227	-0.29953	-1.3409	-1.4234	-0.39
16606	*FUCA2*	0.78899	-0.81965	-1.1484	-1.9219	-0.39
16607	*NCOA1*	-0.16039	-0.30811	-0.71063	-2.1092	-0.39
16608	*MRPL13*	1.1241	-0.34148	-1.9622	-2.2127	-0.39
16609	*GPSM2*	0.47413	-0.33937	-1.3148	-1.4031	-0.39
16610	*KIAA1109*	0.30301	-0.26348	-1.22	-2.7627	-0.39
16611	*PPFIBP2*	-0.16485	-0.47338	-0.54402	-1.6951	-0.39
16612	*MAP3K7CL*	0.26123	0.028777	-1.4736	-2.1126	-0.39
16613	*HSPE1*	0.82872	-1.0033	-1.0095	-1.0884	-0.39
16614	*SNX25*	0.041176	-0.14244	-1.0838	-2.1945	-0.40
16615	*PRELID1*	-0.26625	-0.40469	-0.5142	-1.1338	-0.40
16616	*C16orf91*	0.52737	-0.70442	-1.0091	-1.6105	-0.40
16617	*TMEM200A*	0.22609	-0.17635	-1.236	-1.3008	-0.40
16618	*KRTAP13-4*	0.54891	-0.7342	-1.0013	-1.0901	-0.40
16619	*NARFL*	0.22275	-0.23931	-1.1703	-1.4944	-0.40
16620	*RIPPLY1*	0.31465	-0.02484	-1.4768	-1.7363	-0.40
16621	*RGAG1*	0.086717	-0.33517	-0.93889	-2.4422	-0.40
16622	*DPF2*	0.52353	-0.47473	-1.2363	-2.223	-0.40
16623	*ARMC7*	0.059003	-0.52552	-0.72101	-0.9703	-0.40
16624	*TBC1D3F*	0.94304	-0.64575	-1.4856	-1.739	-0.40
16625	*UBA1*	-0.20057	-0.37106	-0.61779	-1.9622	-0.40
16626	*KCNJ3*	-0.03528	-0.53815	-0.61663	-1.5264	-0.40
16627	*DNAJC2*	0.67965	-0.82524	-1.0452	-1.1283	-0.40
16628	*PPAP2A*	0.06609	-0.46384	-0.79319	-1.2438	-0.40
16629	*ZNF18*	-0.13085	-0.22886	-0.83143	-0.99838	-0.40
16630	*PPP1R11*	1.0572	-0.8471	-1.4024	-1.9696	-0.40
16631	*RHAG*	-0.15143	-0.28838	-0.75408	-1.4338	-0.40
16632	*CRB1*	1.1533	-0.74013	-1.6072	-2.0138	-0.40
16633	*COL6A3*	0.66381	-0.85594	-1.0019	-1.2193	-0.40
16634	*TNFAIP2*	0.81713	-0.90346	-1.1081	-1.4258	-0.40
16635	*METTL16*	-0.06892	-0.35617	-0.77018	-2.3391	-0.40
16636	*SSC4D*	0.15744	-0.53045	-0.82255	-2.7509	-0.40
16637	*RPP30*	1.142	-0.62378	-1.7149	-2.0237	-0.40
16638	*RNF38*	0.13442	0.13427	-1.4681	-1.8813	-0.40
16639	*ZNF43*	0.14319	-0.62179	-0.72172	-1.1936	-0.40
16640	*CHI3L1*	0.0951	-0.52238	-0.77308	-0.99448	-0.40
16641	*ZYX*	-0.03506	-0.52711	-0.63966	-1.3007	-0.40
16642	*PDE2A*	0.032469	-0.23625	-0.99838	-1.049	-0.40
16643	*SORCS2*	0.040477	-0.16651	-1.077	-1.3961	-0.40
16644	*ZNF3*	-0.03668	-0.07193	-1.0952	-1.2694	-0.40
16645	*ZWINT*	-0.15039	-0.16207	-0.89186	-1.252	-0.40

16646	*KIF3A*	0.66949	-0.63198	-1.2421	-1.7306	-0.40
16647	*RNMT*	1.7876	-1.285	-1.7075	-2.0871	-0.40
16648	*CCT2*	-0.28901	-0.40136	-0.51467	-2.4703	-0.40
16649	*CDH26*	0.15264	-0.29227	-1.067	-2.1004	-0.40
16650	*PRSS3*	2.1415	-1.3865	-1.9622	-3.213	-0.40
16651	*COX20*	0.18725	-0.38527	-1.0092	-1.7001	-0.40
16652	*PPP1R12A*	-0.06132	-0.51468	-0.63152	-1.9473	-0.40
16653	*VMA21*	0.061507	-0.24431	-1.0256	-1.3736	-0.40
16654	*ZNF281*	0.34273	-0.10838	-1.4428	-2.0384	-0.40
16655	*LSMEM1*	0.1337	-0.10716	-1.237	-1.4553	-0.40
16656	*SYNJ2BP-COX16*	0.097417	-0.14208	-1.1671	-2.016	-0.40
16657	*FBXO45*	0.3421	-0.51825	-1.0359	-1.1601	-0.40
16658	*RHO*	-0.16122	-0.49867	-0.55284	-0.62881	-0.40
16659	*PTCHD1*	2.1576	-1.55	-1.8207	-3.8595	-0.40
16660	*CCDC36*	0.54829	-0.74572	-1.0158	-1.4527	-0.40
16661	*ADGRB1*	-0.09317	-0.13055	-0.9904	-1.1716	-0.40
16662	*MRE11A*	0.43632	-0.56815	-1.0825	-1.8909	-0.40
16663	*MRGBP*	-0.14632	-0.34533	-0.72348	-1.547	-0.41
16664	*PDIA6*	0.92608	-0.98769	-1.154	-1.6086	-0.41
16665	*NUPL1*	-0.0261	-0.34718	-0.84261	-1.4392	-0.41
16666	*KXD1*	0.70797	-1.51913			-0.41
16667	*IQCF5*	1.1851	-0.58965	-1.8129	-2.5827	-0.41
16668	*RRP15*	0.23246	-0.08162	-1.3689	-2.381	-0.41
16669	*SERPINB6*	0.30631	-0.56241	-0.96215	-1.0359	-0.41
16670	*ST6GALNAC1*	0.42253	-0.23989	-1.4036	-1.8996	-0.41
16671	*TF*	-0.19128	-0.37213	-0.65781	-2.0337	-0.41
16672	*LIN28A*	0.75838	-0.70601	-1.2736	-1.7266	-0.41
16673	*KIF20A*	1.6278	-1.3189	-1.5302	-1.6964	-0.41
16674	*TEX35*	-0.30613	-0.35986	-0.55563	-1.2514	-0.41
16675	*LRRC4*	0.41666	-0.11759	-1.5212	-1.5462	-0.41
16676	*RB1CC1*	0.21963	-0.04896	-1.3932	-2.4077	-0.41
16677	*FAM157B*	1.5649	-0.85236	-1.9353	-2.4381	-0.41
16678	*WFDC12*	0.99686	-0.95206	-1.2677	-2.1577	-0.41
16679	*CAPN5*	0.004697	-0.46758	-0.7602	-2.0382	-0.41
16680	*GGA3*	2.1224	-1.6395	-1.7065	-2.8849	-0.41
16681	*CYP11A1*	0.39397	0.20224	-1.8202	-2.4322	-0.41
16682	*MECP2*	-0.3908	-0.39689	-0.43634	-0.88839	-0.41
16683	*FKBP4*	0.30432	-0.0205	-1.5091	-1.5099	-0.41
16684	*ELMOD1*	0.87673	-0.95893	-1.1432	-1.4125	-0.41
16685	*POLN*	0.83536	-0.88438	-1.1765	-1.9075	-0.41
16686	*SOX13*	-0.08719	-0.34077	-0.79821	-1.7056	-0.41
16687	*PCDHB6*	1.7615	-1.189	-1.7991	-1.8202	-0.41
16688	*CT47A1*	0.50465	-0.256	-1.4754	-3.5623	-0.41
16689	*USP48*	0.40503	-0.65169	-0.98026	-2.4172	-0.41
16690	*ABCA7*	-0.124	-0.51221	-0.59088	-1.0351	-0.41
16691	*SF3B1*	0.29413	-0.63187	-0.88963	-1.5943	-0.41

16692	*C2orf27B*	-0.11314	-0.46761	-0.64689	-1.7161	-0.41
16693	*HGC6.3*	0.097436	-0.44353	-0.88199	-1.2867	-0.41
16694	*GLOD4*	0.31749	-0.64164	-0.90508	-1.1676	-0.41
16695	*TAF6L*	0.19463	-0.45505	-0.96938	-1.4455	-0.41
16696	*PKD2L1*	1.3893	-0.92781	-1.6917	-2.1151	-0.41
16697	*TUSC3*	-0.20104	-0.25999	-0.77097	-2.0113	-0.41
16698	*ISPD*	0.33066	-0.27961	-1.2832	-1.8219	-0.41
16699	*CTRC*	0.79112	-0.74649	-1.2772	-1.3198	-0.41
16700	*KIAA0319L*	0.52388	-0.13843	-1.62	-1.8926	-0.41
16701	*SLC38A1*	0.5536	-0.08851	-1.7001	-1.8946	-0.41
16702	*SORCS3*	0.42709	-0.4433	-1.219	-2.397	-0.41
16703	*ELOVL2*	-0.00825	-0.29879	-0.92948	-2.7509	-0.41
16704	*PNKP*	0.49925	-0.60012	-1.1357	-1.6297	-0.41
16705	*DDX41*	0.16689	-0.51998	-0.88372	-1.4418	-0.41
16706	*MYH7*	0.72713	0.64275	-2.6075	-2.9632	-0.41
16707	*OTUD7A*	0.85789	-0.44353	-1.6527	-3.1317	-0.41
16708	*ZIC2*	0.22212	-0.588	-0.87536	-1.1025	-0.41
16709	*PGBD1*	0.093906	-0.5067	-0.83027	-0.98719	-0.41
16710	*NMT2*	0.59214	-0.596	-1.2393	-2.1412	-0.41
16711	*EIF1*	0.33597	-0.47314	-1.106	-1.7307	-0.41
16712	*DRAM1*	0.14084	-0.59678	-0.78793	-1.3114	-0.41
16713	*NUDCD3*	1.1141	-0.94015	-1.4182	-1.9894	-0.41
16714	*PARP10*	0.3832	-0.60794	-1.0231	-1.1888	-0.42
16715	*CD96*	0.14884	0.087344	-1.4841	-2.2888	-0.42
16716	*FAM188B*	-0.03414	-0.19753	-1.0164	-3.0209	-0.42
16717	*GRIK1*	0.42316	-0.05201	-1.62	-1.8065	-0.42
16718	*C1QA*	1.2789	-1.2119	-1.3179	-1.3298	-0.42
16719	*TMEM91*	0.45249	-0.72994	-0.97499	-1.9052	-0.42
16720	*MPZL2*	0.023338	-0.32677	-0.94912	-2.1463	-0.42
16721	*FOXD4L1*	1.5296	-1.1987	-1.5847	-1.8223	-0.42
16722	*VPS25*	0.010555	-0.09054	-1.1756	-2.0069	-0.42
16723	*TMEM216*	-0.00823	-0.4571	-0.79243	-1.0248	-0.42
16724	*SPINT2*	0.77909	-0.85768	-1.181	-1.2623	-0.42
16725	*SLC30A9*	-0.02484	-0.14048	-1.0948	-1.6047	-0.42
16726	*ATP6V1D*	0.42961	-0.06117	-1.6289	-1.7366	-0.42
16727	*WDR66*	0.71094	-0.35733	-1.6143	-1.6428	-0.42
16728	*MID1*	-0.1082	-0.32087	-0.83277	-1.7285	-0.42
16729	*ARSI*	0.33086	0.16491	-1.7585	-2.7546	-0.42
16730	*NMT1*	0.40937	-0.60016	-1.0726	-1.2684	-0.42
16731	*NT5C1A*	0.60858	-0.89186	-0.98026	-2.5056	-0.42
16732	*PAK6*	0.10498	-0.47183	-0.89741	-1.4258	-0.42
16733	*GPR82*	2.1509	-1.6429	-1.7726	-1.8751	-0.42
16734	*CCDC184*	-0.05524	-0.07692	-1.1325	-2.3707	-0.42
16735	*ZNF19*	0.069497	-0.44353	-0.89258	-1.5401	-0.42
16736	*PHF20L1*	0.25931	-0.28173	-1.2443	-1.5613	-0.42
16737	*IFITM5*	0.4629	0.12787	-1.8583	-2.0351	-0.42

16738	*MVP*	1.9956	-1.0945	-2.1687	-2.3293	-0.42
16739	*ABHD2*	-0.04678	-0.50718	-0.71421	-1.1595	-0.42
16740	*RAD51D*	0.89321	-0.83913	-1.3226	-1.8816	-0.42
16741	*MYH8*	0.55379	-0.78588	-1.0367	-1.1214	-0.42
16742	*DTX2*	0.060104	-0.0299	-1.2995	-1.7746	-0.42
16743	*C19orf53*	0.42233	-0.36557	-1.3282	-1.4312	-0.42
16744	*USP22*	0.27373	-0.15835	-1.3875	-3.0097	-0.42
16745	*TRAM1L1*	0.41353	-0.81433	-0.87198	-1.3623	-0.42
16746	*HMGN4*	-0.23523	-0.31694	-0.72095	-1.1084	-0.42
16747	*B4GALT2*	0.028002	-0.03222	-1.2705	-1.3162	-0.42
16748	*HDAC11*	0.33577	-0.198	-1.4132	-1.42	-0.43
16749	*SKIDA1*	-0.00823	-0.41716	-0.85018	-1.6525	-0.43
16750	*EXOC3L1*	0.66957	-0.1496	-1.7956	-1.8561	-0.43
16751	*FAM92A1*	1.1611	-1.1283	-1.3085	-1.4725	-0.43
16752	*OXSR1*	0.68447	-0.67007	-1.2903	-1.3198	-0.43
16753	*KIAA0556*	-0.0953	-0.55649	-0.62739	-0.97425	-0.43
16754	*FTH1*	1.1697	-0.77333	-1.6759	-2.224	-0.43
16755	*DEF8*	0.15306	-0.55147	-0.88164	-1.1822	-0.43
16756	*COMMD2*	0.58207	-0.52949	-1.3327	-1.6527	-0.43
16757	*TCF4*	0.44453	-0.44353	-1.2816	-1.6528	-0.43
16758	*DDX39A*	0.38094	-0.76358	-0.8988	-1.7577	-0.43
16759	*RAB15*	0.33184	-0.79904	-0.81509	-1.2684	-0.43
16760	*ZFP64*	-0.09638	-0.29596	-0.89009	-1.1441	-0.43
16761	*FAM126B*	-0.07889	-0.17249	-1.0315	-1.4316	-0.43
16762	*AP4S1*	0.81248	-0.8071	-1.2895	-2.462	-0.43
16763	*C1orf116*	0.015765	-0.16483	-1.1354	-1.2837	-0.43
16764	*ARMC2*	-0.04965	-0.54621	-0.69003	-0.98572	-0.43
16765	*YME1L1*	1.0655	-0.54604	-1.8056	-1.8846	-0.43
16766	*C1orf43*	0.27172	-0.54934	-1.009	-1.535	-0.43
16767	*HIST1H2BI*	1.0628	-1.1384	-1.2113	-2.2318	-0.43
16768	*IFT74*	1.1959	-0.57586	-1.9086	-2.8928	-0.43
16769	*CYB561D2*	0.18732	-0.10119	-1.3759	-1.4361	-0.43
16770	*TMEM239*	0.85612	-1.0067	-1.1415	-1.7602	-0.43
16771	*OR7D4*	0.89819	-0.22131	-1.9695	-2.7331	-0.43
16772	*ZNF750*	0.37523	-0.80368	-0.86517	-1.5948	-0.43
16773	*C11orf54*	0.082077	-0.64571	-0.73051	-0.98853	-0.43
16774	*DHRS7C*	0.75712	-0.38522	-1.6663	-2.3292	-0.43
16775	*FAM25C*	1.5838	-1.1384	-1.7407	-1.808	-0.43
16776	*FAM83H*	0.29438	-0.44353	-1.1472	-2.5844	-0.43
16777	*APOL3*	-0.10178	-0.40849	-0.78839	-0.93114	-0.43
16778	*FSTL5*	0.77831	-0.19107	-1.8869	-2.101	-0.43
16779	*C21orf58*	0.032576	-0.59716	-0.73511	-2.3287	-0.43
16780	*GPC3*	0.12545	-0.14477	-1.2815	-2.252	-0.43
16781	*MROH9*	0.93654	-0.8756	-1.3619	-1.4452	-0.43
16782	*TP53*	-0.10753	-0.36427	-0.83005	-0.91981	-0.43
16783	*SOGA3*	-0.04919	-0.3205	-0.93287	-1.7024	-0.43

16784	*TJP3*	0.92433	-0.93592	-1.2912	-1.6636	-0.43
16785	*PER1*	0.41974	-0.15385	-1.5687	-1.8046	-0.43
16786	*NDUFA5*	0.1836	-0.06124	-1.4252	-1.4649	-0.43
16787	*CDC37*	1.2549	-1.1193	-1.4392	-5.0141	-0.43
16788	*MAGI1*	0.38482	-0.70078	-0.98798	-1.0838	-0.43
16789	*UBE2H*	0.076188	-0.62833	-0.75205	-1.0711	-0.43
16790	*ZNF669*	0.13063	-0.69915	-0.73577	-1.0168	-0.43
16791	*CRYGA*	-0.0248	-0.33984	-0.94255	-1.3033	-0.44
16792	*CASC4*	0.85768	-0.57547	-1.5895	-1.9772	-0.44
16793	*DNAJC18*	-0.2304	-0.36996	-0.70756	-1.5118	-0.44
16794	*EIF5*	-0.35784	-0.38862	-0.56225	-1.4258	-0.44
16795	*TXNDC8*	0.88276	-0.67599	-1.5171	-1.677	-0.44
16796	*NXF2B*	0.17274	-0.54679	-0.93707	-1.8441	-0.44
16797	*SSBP4*	0.46245	-0.74011	-1.0344	-1.4448	-0.44
16798	*TSPY3*	0.52567	-0.40633	-1.4317	-1.8921	-0.44
16799	*CD81*	0.3919	0.086138	-1.7906	-2.5707	-0.44
16800	*SOX15*	0.61696	-0.78229	-1.1479	-2.1572	-0.44
16801	*XAGE1E*	0.19908	-0.55846	-0.95388	-1.2048	-0.44
16802	*EXOSC10*	-0.03791	-0.33297	-0.9442	-1.3766	-0.44
16803	*SORBS2*	0.33331	-0.2539	-1.3945	-1.6268	-0.44
16804	*ZNF106*	-0.27612	-0.46559	-0.57546	-1.248	-0.44
16805	*FAM19A4*	0.36026	-0.68598	-0.99357	-1.3664	-0.44
16806	*CT47A12*	1.067	-0.66647	-1.7203	-2.2691	-0.44
16807	*ACIN1*	0.75545	-0.29681	-1.7804	-1.8014	-0.44
16808	*DVL1*	0.47391	-0.42859	-1.3676	-2.2403	-0.44
16809	*ADTRP*	1.3258	-0.62862	-2.0196	-2.458	-0.44
16810	*GALNT2*	0.42438	-0.64571	-1.1021	-2.7325	-0.44
16811	*PSMC3*	0.12979	-0.41239	-1.0413	-1.1357	-0.44
16812	*GP1BA*	0.13779	-0.4055	-1.0564	-1.0576	-0.44
16813	*CDKN1B*	1.6431	-1.3023	-1.6676	-1.828	-0.44
16814	*GALNT14*	0.21664	-0.02974	-1.5144	-1.8462	-0.44
16815	*CYP2E1*	-0.24501	-0.37274	-0.70988	-1.0293	-0.44
16816	*HMGA1*	0.83604	-0.855	-1.3088	-2.1561	-0.44
16817	*NEIL1*	0.50969	-0.6689	-1.1693	-1.9953	-0.44
16818	*EBAG9*	0.36538	-0.09924	-1.5962	-5.1808	-0.44
16819	*GRSF1*	1.2977	-1.1388	-1.4893	-2.2117	-0.44
16820	*HABP2*	-0.16374	-0.40693	-0.7602	-1.4824	-0.44
16821	*KCNJ14*	-0.39352	-0.44572	-0.49177	-0.83627	-0.44
16822	*ZBTB1*	-0.07367	-0.14994	-1.1089	-1.8642	-0.44
16823	*IST1*	2.2095	-1.7056	-1.8366	-2.3755	-0.44
16824	*KLHL26*	1.0576	-0.53078	-1.8611	-2.0407	-0.44
16825	*MDM2*	1.1425	-0.90028	-1.5782	-1.8577	-0.45
16826	*KRTAP4-5*	0.20138	-0.2748	-1.2633	-2.3689	-0.45
16827	*SLC14A1*	0.34183	-0.12958	-1.5494	-2.3585	-0.45
16828	*KAT2B*	0.77868	-0.80498	-1.311	-2.6999	-0.45
16829	*FHOD3*	0.13369	-0.62073	-0.85027	-1.2766	-0.45

16830	*NCOA4*	0.4616	-0.7532	-1.0461	-1.0602	-0.45
16831	*CNNM3*	0.18739	-0.63152	-0.89401	-1.4998	-0.45
16832	*SUCLG2*	0.1473	-0.67141	-0.81433	-1.2691	-0.45
16833	*THPO*	0.48535	-0.34886	-1.4754	-2.2172	-0.45
16834	*KCNJ10*	-0.21587	-0.23276	-0.8934	-1.0571	-0.45
16835	*SNRPD2*	0.29824	-0.16986	-1.4705	-1.5906	-0.45
16836	*XAGE5*	0.044275	-0.34886	-1.0385	-1.1943	-0.45
16837	*ATP6V1C1*	0.9606	-0.87837	-1.4258	-2.2276	-0.45
16838	*MMS22L*	0.59723	-0.79474	-1.1463	-2.0492	-0.45
16839	*ETV5*	-0.19911	-0.44975	-0.69629	-1.0664	-0.45
16840	*CHST14*	1.0423	-1.072	-1.3162	-2.9578	-0.45
16841	*VPS13A*	0.51796	-0.88702	-0.97702	-1.6072	-0.45
16842	*AHSA2*	1.0543	-0.92956	-1.4725	-2.9283	-0.45
16843	*MDH2*	0.096631	-0.65113	-0.79423	-1.0697	-0.45
16844	*EPRS*	0.39464	-0.71009	-1.0336	-2.0304	-0.45
16845	*TTBK2*	0.38316	-0.65968	-1.0726	-1.3818	-0.45
16846	*ITGA6*	0.51223	-0.87817	-0.9858	-1.8297	-0.45
16847	*RMND5B*	0.40253	-0.37104	-1.3836	-1.682	-0.45
16848	*PBOV1*	-0.14868	-0.30065	-0.90281	-1.537	-0.45
16849	*SNX4*	-0.22963	-0.44088	-0.68229	-1.2602	-0.45
16850	*MAP3K6*	1.2223	-1.0613	-1.5138	-2.428	-0.45
16851	*EIF1AD*	0.70035	-0.85389	-1.1995	-2.0895	-0.45
16852	*ARRB1*	1.0809	-1.0819	-1.3526	-2.1605	-0.45
16853	*NUP205*	0.7313	-0.91767	-1.1676	-1.2884	-0.45
16854	*DKK3*	0.28522	-0.18101	-1.4584	-2.072	-0.45
16855	*TACR1*	0.90394	-1.012	-1.2464	-1.3348	-0.45
16856	*NUP85*	0.41703	-0.80558	-0.96632	-1.8364	-0.45
16857	*DLST*	0.57693	-0.58231	-1.3498	-1.8885	-0.45
16858	*TRAPPC2*	1.9161	-1.2525	-2.0196	-2.1213	-0.45
16859	*TMED5*	0.75389	-0.90947	-1.2008	-1.9984	-0.45
16860	*DHFR*	0.2527	-0.64206	-0.96946	-1.0424	-0.45
16861	*KLC3*	0.32443	-0.67441	-1.0115	-3.1939	-0.45
16862	*FAM169A*	0.082752	-0.44745	-0.99746	-1.1385	-0.45
16863	*C8orf82*	-0.28581	-0.38103	-0.69622	-1.4841	-0.45
16864	*NKAIN3*	0.57482	-0.50046	-1.4376	-2.1019	-0.45
16865	*ZNF624*	0.60661	-0.57967	-1.3905	-2.2879	-0.45
16866	*PTEN*	-0.29563	-0.40428	-0.66432	-0.98266	-0.45
16867	*RMI1*	-0.34118	-0.43841	-0.58509	-1.7716	-0.45
16868	*HELZ2*	0.24059	-0.74471	-0.86096	-2.6177	-0.46
16869	*C7orf31*	0.077878	-0.0576	-1.3864	-1.5204	-0.46
16870	*UNC5B*	0.20681	-0.6945	-0.87898	-2.4013	-0.46
16871	*ODF3B*	1.3947	-1.0212	-1.742	-2.4718	-0.46
16872	*KCNT1*	-0.12866	-0.39451	-0.84539	-1.3755	-0.46
16873	*CCDC86*	0.40836	-0.50057	-1.2772	-1.5962	-0.46
16874	*GCNT2*	0.66152	-0.93661	-1.0944	-1.0992	-0.46
16875	*HNRNPA2B1*	-0.02808	-0.3016	-1.0402	-1.3078	-0.46

16876	*DNASE1*	0.6271	-0.7008	-1.2963	-1.9695	-0.46
16877	*NXPE4*	0.53569	-0.80442	-1.1016	-1.1388	-0.46
16878	*PCDHGA4*	-0.27159	-0.48235	-0.61643	-1.8611	-0.46
16879	*ARL2BP*	0.6696	-0.36467	-1.6759	-2.0116	-0.46
16880	*PYY*	0.36562	-0.45699	-1.2827	-3.2454	-0.46
16881	*KIAA1549L*	0.53271	-0.76083	-1.1461	-1.6007	-0.46
16882	*ORC6*	-0.11998	-0.59178	-0.66251	-1.7679	-0.46
16883	*CFHR5*	0.45842	-0.3159	-1.5173	-3.0157	-0.46
16884	*C15orf39*	0.40757	-0.41323	-1.37	-1.7571	-0.46
16885	*RAD50*	0.4089	-0.09344	-1.6913	-1.9567	-0.46
16886	*NUMB*	0.15465	-0.76358	-0.76716	-1.2259	-0.46
16887	*OFD1*	0.54035	-0.50048	-1.416	-2.3885	-0.46
16888	*RHOT2*	0.45584	-0.28825	-1.5467	-2.1264	-0.46
16889	*PSORS1C2*	0.87325	0.5873	-2.8398	-3.4804	-0.46
16890	*FAM134C*	0.31649	-0.23638	-1.4596	-1.927	-0.46
16891	*CCDC146*	-0.14306	-0.2157	-1.0211	-1.5558	-0.46
16892	*C10orf67*	0.77507	-0.52105	-1.6343	-2.6113	-0.46
16893	*ATAD2*	0.49719	-0.41469	-1.4633	-2.3357	-0.46
16894	*PARP3*	0.80454	-0.53304	-1.6525	-2.8702	-0.46
16895	*EDC4*	0.21497	-0.48875	-1.1073	-1.2483	-0.46
16896	*QSOX2*	0.53716	-0.66191	-1.2565	-1.2823	-0.46
16897	*HEATR9*	0.86989	-1.0093	-1.2426	-1.2666	-0.46
16898	*SH3TC2*	0.3946	-0.64888	-1.1289	-1.1968	-0.46
16899	*CXCL11*	0.080928	0.037279	-1.5018	-2.5156	-0.46
16900	*GDPD1*	0.032511	-0.64278	-0.7747	-0.85059	-0.46
16901	*ENKD1*	0.10504	-0.32219	-1.1681	-1.682	-0.46
16902	*PLK4*	-0.05613	-0.54972	-0.77946	-2.3101	-0.46
16903	*DERL1*	0.13368	-0.56714	-0.95321	-1.1354	-0.46
16904	*MCM7*	0.93277	-0.99346	-1.3267	-2.6416	-0.46
16905	*TRIM43B*	0.85937	-1.0142	-1.2343	-1.3162	-0.46
16906	*METTL3*	-0.25305	-0.43626	-0.70074	-1.2845	-0.46
16907	*NXNL1*	0.35324	-0.61665	-1.127	-4.0338	-0.46
16908	*FAM132B*	-0.19512	-0.35669	-0.83862	-1.6852	-0.46
16909	*BRD4*	0.21781	-0.44574	-1.1631	-1.9686	-0.46
16910	*ZNF442*	0.18078	-0.49311	-1.0793	-1.9811	-0.46
16911	*CIDEC*	-0.16962	-0.39689	-0.82547	-2.6169	-0.46
16912	*NPY1R*	1.0835	-1.141	-1.3349	-1.6251	-0.46
16913	*SH2B2*	0.064869	-0.2005	-1.257	-1.8219	-0.46
16914	*ANKRA2*	0.34565	-0.37544	-1.3633	-1.3892	-0.46
16915	*DRD1*	0.25102	-0.32237	-1.3218	-1.5212	-0.46
16916	*MFSD8*	-0.02747	-0.46124	-0.90462	-1.3291	-0.46
16917	*LRRC3C*	0.32643	-0.7967	-0.92366	-1.1156	-0.46
16918	*POLR2L*	0.26231	-0.64675	-1.0095	-1.348	-0.46
16919	*ZNF589*	1.1646	-0.83883	-1.72	-2.9772	-0.46
16920	*GOLGA8H*	0.40438	-0.45669	-1.3425	-1.8154	-0.46
16921	*THOC7*	0.71186	-0.7679	-1.3399	-2.0407	-0.47

16922	*C15orf54*	0.82828	-0.90616	-1.3183	-1.7232	-0.47
16923	*OR2T29*	0.92036	-0.58084	-1.7364	-3.8028	-0.47
16924	*GEN1*	0.27479	-0.44353	-1.2284	-1.8112	-0.47
16925	*FAM219A*	0.83015	-0.73853	-1.4894	-1.615	-0.47
16926	*MOCS1*	0.80962	-0.78172	-1.4258	-1.9054	-0.47
16927	*DSC1*	-0.16559	-0.56411	-0.66912	-2.0679	-0.47
16928	*CYP26A1*	0.85099	-0.83862	-1.4112	-2.1936	-0.47
16929	*LEFTY2*	0.062474	-0.32293	-1.1391	-1.2817	-0.47
16930	*EPHA10*	0.59415	-0.81787	-1.1768	-1.5256	-0.47
16931	*EPB41L1*	-0.31688	-0.47889	-0.6057	-2.412	-0.47
16932	*ARL4A*	0.23768	-0.50075	-1.1391	-2.4642	-0.47
16933	*TNFRSF9*	0.77843	-0.13473	-2.0465	-2.2746	-0.47
16934	*UTP3*	1.1885	-1.2167	-1.3753	-2.7384	-0.47
16935	*GNRH1*	1.8259	-1.5424	-1.6891	-2.1282	-0.47
16936	*C6orf165*	0.62329	-0.30878	-1.7203	-3.2089	-0.47
16937	*IQUB*	0.01805	-0.09539	-1.3285	-1.7526	-0.47
16938	*USP17L13*	-0.19885	-0.28032	-0.9285	-0.93465	-0.47
16939	*CSTA*	0.26443	-0.42369	-1.2485	-2.2254	-0.47
16940	*LGMN*	1.9955	-1.5815	-1.8219	-2.5806	-0.47
16941	*INTS3*	0.72957	-0.207	-1.9305	-1.9357	-0.47
16942	*TRIM34*	0.48846	-0.91944	-0.97952	-1.9165	-0.47
16943	*DLEC1*	0.93606	-1.0212	-1.3267	-1.6707	-0.47
16944	*RNF146*	0.32681	-0.7456	-0.99446	-1.6016	-0.47
16945	*RPP21*	0.81532	-0.78044	-1.4482	-1.6824	-0.47
16946	*SMR3B*	0.67996	-0.98597	-1.1091	-1.1968	-0.47
16947	*CNGA4*	0.17463	-0.50793	-1.083	-2.5707	-0.47
16948	*SRC*	1.0414	0.1507	-2.6101	-2.6376	-0.47
16949	*ELMO3*	0.44547	-0.62306	-1.241	-1.9619	-0.47
16950	*FAM83E*	0.083155	-0.52035	-0.98214	-1.2016	-0.47
16951	*LSMEM2*	0.35889	-0.5707	-1.2084	-1.3965	-0.47
16952	*HAS2*	-0.16888	-0.37998	-0.8715	-0.93789	-0.47
16953	*CDAN1*	0.17097	-0.45759	-1.1344	-1.2987	-0.47
16954	*RWDD4*	1.384	-1.2571	-1.5491	-2.8398	-0.47
16955	*APOD*	-0.1401	-0.60604	-0.6761	-1.9794	-0.47
16956	*SAMD8*	1.5293	-0.96463	-1.9874	-2.3904	-0.47
16957	*ISCA2*	0.36609	-0.43005	-1.3606	-1.392	-0.47
16958	*KIAA0391*	-0.32397	-0.43737	-0.66787	-1.7865	-0.48
16959	*SYMPK*	0.5886	-0.01346	-2.005	-2.3496	-0.48
16960	*ZNHIT2*	1.0595	-0.85093	-1.6386	-1.6626	-0.48
16961	*UBE2S*	0.31895	-0.16971	-1.5803	-3.051	-0.48
16962	*DYNAP*	0.98642	-1.0202	-1.398	-1.4794	-0.48
16963	*STRC*	0.63915	-0.98421	-1.0871	-1.4962	-0.48
16964	*EMC4*	0.46629	-0.81509	-1.0838	-1.6607	-0.48
16965	*C16orf92*	1.2503	-1.1901	-1.4932	-1.7016	-0.48
16966	*GFRA2*	0.54719	-0.60794	-1.3723	-1.5971	-0.48
16967	*PSMA5*	-0.00222	-0.56919	-0.86245	-1.8568	-0.48

16968	*SLC9A8*	0.1479	-0.49177	-1.0903	-1.129	-0.48
16969	*CALCOCO2*	0.48225	-0.88503	-1.0315	-1.5765	-0.48
16970	*EFCAB13*	0.72377	-0.44353	-1.7153	-1.8219	-0.48
16971	*CBLL1*	-0.37907	-0.41155	-0.64725	-0.8128	-0.48
16972	*ZNF365*	0.14103	-0.60841	-0.97053	-1.3047	-0.48
16973	*WBP11*	0.48494	-0.44933	-1.4736	-1.6469	-0.48
16974	*SAPCD2*	0.046831	-0.12184	-1.3637	-2.2866	-0.48
16975	*MYBBP1A*	-0.29509	-0.44299	-0.70101	-1.2825	-0.48
16976	*PRAMEF7*	-0.11385	-0.15777	-1.1676	-1.7591	-0.48
16977	*MAP9*	-0.14252	-0.16343	-1.1341	-2.0351	-0.48
16978	*ETNK1*	0.20971	-0.54274	-1.1071	-1.2845	-0.48
16979	*DHX35*	1.3769	-1.236	-1.5814	-1.5847	-0.48
16980	*IGIP*	-0.3787	-0.49635	-0.56929	-1.0283	-0.48
16981	*HIST2H3A*	0.18054	-0.71077	-0.9142	-2.0553	-0.48
16982	*RTCB*	0.31852	-0.73143	-1.0317	-1.7749	-0.48
16983	*CMTM3*	-0.03826	-0.7004	-0.70601	-1.1384	-0.48
16984	*ADO*	0.087344	-0.54847	-0.98421	-1.7577	-0.48
16985	*GDI2*	0.46439	-0.7132	-1.1967	-1.9219	-0.48
16986	*FIBIN*	0.73478	-0.19107	-1.9894	-2.4322	-0.48
16987	*GMEB1*	0.21099	-0.74014	-0.91708	-1.7667	-0.48
16988	*AWAT1*	0.25085	-0.71009	-0.98769	-1.5137	-0.48
16989	*CAB39*	0.31734	-0.726	-1.0385	-1.3409	-0.48
16990	*MYBPHL*	-0.06987	-0.54177	-0.83627	-1.7056	-0.48
16991	*ADAL*	0.28778	-0.64313	-1.0928	-1.5762	-0.48
16992	*METTL4*	0.066705	0.040796	-1.5558	-1.7956	-0.48
16993	*C11orf80*	0.25796	-0.57479	-1.1325	-2.1648	-0.48
16994	*AGO4*	0.11117	-0.53877	-1.0221	-1.1808	-0.48
16995	*HGD*	-0.18153	-0.50992	-0.75944	-1.1316	-0.48
16996	*PCBP3*	-0.0044	-0.44353	-1.0049	-1.3851	-0.48
16997	*TBC1D10C*	0.19114	-0.18388	-1.4614	-1.4713	-0.48
16998	*ELFN2*	0.37772	-0.44541	-1.3872	-2.1207	-0.48
16999	*HECTD4*	-0.30975	-0.4376	-0.70947	-1.6062	-0.49
17000	*SDS*	-0.10195	-0.54112	-0.81478	-1.6636	-0.49
17001	*C17orf50*	1.2373	-1.0944	-1.6023	-2.312	-0.49
17002	*SLC5A1*	0.45988	-0.25832	-1.6636	-3.2454	-0.49
17003	*WDR77*	0.010272	-0.52122	-0.95162	-2.2771	-0.49
17004	*JMJD7*	1.4255	-0.79821	-2.0902	-3.6729	-0.49
17005	*CSTF3*	-0.08523	-0.36194	-1.0165	-1.3515	-0.49
17006	*PSMD5*	0.44138	-0.20338	-1.7018	-2.4873	-0.49
17007	*GOLGA8R*	0.73068	-1.0243	-1.1706	-2.0851	-0.49
17008	*RFWD2*	0.46795	-0.73069	-1.2027	-1.6934	-0.49
17009	*INO80E*	-0.30811	-0.57379	-0.58403	-1.8484	-0.49
17010	*KLHL5*	0.64585	-0.90281	-1.2106	-2.8628	-0.49
17011	*SUOX*	0.22548	-0.5871	-1.106	-1.1637	-0.49
17012	*SNTA1*	0.38367	-0.81237	-1.0391	-1.2355	-0.49
17013	*RFPL4B*	0.12307	-0.04635	-1.545	-1.6158	-0.49

17014	*TSNAX*	0.48157	-0.63936	-1.3111	-2.1068	-0.49
17015	*CDK6*	-0.06844	-0.10911	-1.2917	-2.7931	-0.49
17016	*OR13C5*	0.16164	-0.75148	-0.88094	-2.0398	-0.49
17017	*EOGT*	0.97673	-1.1076	-1.3407	-3.0314	-0.49
17018	*CMIP*	0.64718	-0.64075	-1.479	-1.6371	-0.49
17019	*ERAP2*	0.36116	-0.77733	-1.0576	-1.7018	-0.49
17020	*IBA57*	0.46245	-0.00112	-1.9357	-3.6076	-0.49
17021	*B4GALT6*	0.78265	-1.0987	-1.1591	-1.3743	-0.49
17022	*PSMA2*	0.5153	-0.84624	-1.1448	-1.8804	-0.49
17023	*ZNF575*	-0.01545	-0.58349	-0.87694	-1.7203	-0.49
17024	*PAQR6*	0.42579	-0.84337	-1.0606	-1.3478	-0.49
17025	*GK*	0.45578	-0.51585	-1.4203	-1.5165	-0.49
17026	*PPP4R2*	-0.00127	-0.50953	-0.9703	-3.2348	-0.49
17027	*GGPS1*	0.021592	-0.05612	-1.4473	-2.1182	-0.49
17028	*DNAH5*	-0.00153	-0.61675	-0.86367	-1.4788	-0.49
17029	*ORC5*	1.8792	-1.5849	-1.7766	-2.1125	-0.49
17030	*ANGPTL4*	0.56229	-1.0187	-1.027	-1.7667	-0.49
17031	*PTER*	-0.39136	-0.4594	-0.63332	-1.1133	-0.49
17032	*ORC3*	1.2293	-0.91236	-1.8022	-2.5066	-0.50
17033	*SHANK1*	0.47781	-0.75192	-1.2125	-1.7882	-0.50
17034	*KIF12*	0.48618	-0.67441	-1.2985	-1.5719	-0.50
17035	*SIX4*	0.81435	-0.27303	-2.0301	-3.2089	-0.50
17036	*RPS17*	0.95397	-0.93789	-1.5049	-2.7728	-0.50
17037	*PSMG2*	0.31569	-0.49953	-1.3055	-2.6208	-0.50
17038	*PHLDB1*	-0.35675	-0.52004	-0.61281	-2.9473	-0.50
17039	*PLA2G2C*	0.28896	-0.29194	-1.4869	-2.0277	-0.50
17040	*PRDX1*	-0.07432	-0.35894	-1.058	-1.2411	-0.50
17041	*RPS20*	-0.14244	-0.51406	-0.83676	-1.5434	-0.50
17042	*PNISR*	0.60934	-0.65875	-1.445	-2.6275	-0.50
17043	*GRINA*	-0.08702	-0.47227	-0.93525	-1.7306	-0.50
17044	*PDAP1*	2.0084	-1.3371	-2.1685	-4.0172	-0.50
17045	*TRDN*	0.48255	-0.89059	-1.0893	-1.8538	-0.50
17046	*MYO1B*	0.049555	-0.62065	-0.92629	-1.925	-0.50
17047	*FAM35A*	0.4737	-0.9471	-1.0247	-1.1831	-0.50
17048	*MAP1LC3B2*	-0.06833	-0.6835	-0.74672	-0.94252	-0.50
17049	*ACP5*	-0.17755	-0.60223	-0.71893	-1.0905	-0.50
17050	*VEGFA*	0.21219	-0.81701	-0.89401	-1.7709	-0.50
17051	*DEFB134*	-0.1433	-0.18068	-1.1752	-3.0483	-0.50
17052	*LZTS1*	-0.30379	-0.31582	-0.88008	-2.1085	-0.50
17053	*RPS6*	0.25446	-0.48659	-1.268	-2.3871	-0.50
17054	*TK1*	0.19547	-0.81535	-0.88062	-1.2763	-0.50
17055	*TRPM3*	-0.01103	-0.46167	-1.0279	-1.6456	-0.50
17056	*DDX5*	-0.2372	-0.59647	-0.66865	-1.9673	-0.50
17057	*TCF15*	0.21667	-0.68934	-1.0297	-3.0314	-0.50
17058	*ELP3*	1.2313	-1.3478	-1.3864	-2.7546	-0.50
17059	*RAB38*	0.1661	0.049677	-1.7209	-1.855	-0.50

17060	*POLR2D*	0.37192	-0.54373	-1.334	-1.6524	-0.50
17061	*SYCP2L*	0.15064	0.14306	-1.8007	-1.8813	-0.50
17062	*IKZF3*	-0.25603	-0.30371	-0.94739	-3.1458	-0.50
17063	*CLCNKA*	-0.46286	-0.50046	-0.54391	-2.1264	-0.50
17064	*TP53I13*	-0.42497	-0.43244	-0.65023	-1.7497	-0.50
17065	*EIF2S3*	0.98173	-0.35571	-2.1347	-2.1794	-0.50
17066	*SYPL2*	-0.01264	-0.43481	-1.0613	-2.1496	-0.50
17067	*SMEK2*	0.5355	-0.07895	-1.9656	-2.4946	-0.50
17068	*SUPT6H*	0.96942	-0.8198	-1.6614	-2.5292	-0.50
17069	*ZFYVE1*	-0.22496	-0.26125	-1.0267	-1.6597	-0.50
17070	*PSMD8*	-0.04644	-0.56972	-0.89794	-1.03	-0.50
17071	*AURKA*	-0.10247	-0.31965	-1.0927	-1.1343	-0.50
17072	*VDAC1*	-0.22153	-0.28173	-1.0136	-2.8702	-0.51
17073	*DNMT1*	0.03708	-0.52329	-1.0313	-2.3676	-0.51
17074	*ALDH3A2*	0.090843	-0.44353	-1.1656	-1.6759	-0.51
17075	*PPP1R37*	-0.21393	-0.61073	-0.69463	-1.9696	-0.51
17076	*ARNT*	0.82325	-1.1605	-1.1825	-1.8842	-0.51
17077	*VNN3*	0.69777	-0.85002	-1.368	-1.6356	-0.51
17078	*CFL2*	-0.17731	-0.50087	-0.84426	-1.4233	-0.51
17079	*BMF*	0.067212	-0.46762	-1.1223	-1.3008	-0.51
17080	*SARS2*	0.22517	-0.8108	-0.93865	-1.1137	-0.51
17081	*CNOT6*	0.66404	-0.78672	-1.4024	-3.2901	-0.51
17082	*BUB1B*	-0.25545	-0.61167	-0.6593	-1.5971	-0.51
17083	*RAB7B*	-0.10911	-0.25515	-1.163	-1.5289	-0.51
17084	*RAE1*	0.58442	-0.44353	-1.6684	-2.1089	-0.51
17085	*COX7C*	0.89619	-0.44456	-1.9792	-2.3086	-0.51
17086	*KLHL40*	2.1693	-1.4996	-2.1984	-2.5478	-0.51
17087	*PRDM13*	-0.08039	-0.66107	-0.78807	-0.8597	-0.51
17088	*C17orf58*	-0.30975	-0.40465	-0.81539	-1.4545	-0.51
17089	*ZCCHC3*	1.4409	-1.4507	-1.5225	-1.7134	-0.51
17090	*SPIRE2*	0.16177	-0.7757	-0.91944	-1.6847	-0.51
17091	*R3HDM2*	0.3084	-0.78102	-1.0614	-1.4507	-0.51
17092	*STK26*	0.36192	-0.6182	-1.278	-1.7077	-0.51
17093	*PRDM10*	-0.00546	-0.68825	-0.84091	-2.8051	-0.51
17094	*FAM114A1*	-0.02854	-0.53328	-0.97303	-1.6689	-0.51
17095	*GATA5*	0.25931	-0.2636	-1.531	-1.8066	-0.51
17096	*SEC22A*	-0.1635	-0.38815	-0.98421	-1.6049	-0.51
17097	*ABHD17A*	-0.0378	-0.44955	-1.0494	-1.9172	-0.51
17098	*CLEC1B*	0.53888	-0.63172	-1.445	-2.5392	-0.51
17099	*HIST1H2AE*	0.23442	-0.70957	-1.0635	-1.7935	-0.51
17100	*ADAMTS1*	-0.14026	-0.42884	-0.97252	-1.4265	-0.51
17101	*C10orf2*	0.057793	-0.65169	-0.94816	-1.5335	-0.51
17102	*TMEM39A*	1.9267	-1.5382	-1.9308	-2.1404	-0.51
17103	*CLSTN2*	0.44955	-0.82996	-1.1631	-2.3244	-0.51
17104	*MARCH8*	1.0697	-1.1459	-1.4681	-1.8157	-0.51
17105	*C2orf69*	1.1479	-0.79365	-1.9013	-3.9399	-0.52

17106	*C2orf50*	0.17672	-0.62881	-1.0954	-1.7989	-0.52
17107	*RNF208*	-0.04678	-0.54884	-0.95321	-1.0312	-0.52
17108	*RIT2*	0.65454	-0.76725	-1.4368	-1.5684	-0.52
17109	*SLC12A7*	0.006708	-0.25981	-1.2968	-1.4625	-0.52
17110	*DYNC1I1*	-0.04898	-0.43608	-1.0656	-1.3085	-0.52
17111	*GNA15*	0.27992	-0.34353	-1.4871	-1.7145	-0.52
17112	*GADD45GIP1*	0.66953	-0.95045	-1.2705	-1.9912	-0.52
17113	*GRAP*	0.15694	-0.74875	-0.96144	-2.201	-0.52
17114	*VPS13C*	-0.00822	-0.30424	-1.2411	-1.631	-0.52
17115	*INO80*	0.031661	-0.23658	-1.3504	-2.5008	-0.52
17116	*TEPP*	0.44957	-0.74774	-1.2572	-1.6072	-0.52
17117	*NR2E1*	0.56529	-0.97944	-1.1415	-1.4258	-0.52
17118	*LRRC66*	-0.07406	-0.62889	-0.85389	-2.4829	-0.52
17119	*KDM1A*	-0.3417	-0.5329	-0.6828	-1.241	-0.52
17120	*CDH22*	-0.36728	-0.49694	-0.69319	-1.6343	-0.52
17121	*NGEF*	-0.05429	-0.71908	-0.78545	-1.2171	-0.52
17122	*NAA25*	1.3134	-1.3118	-1.5633	-1.6484	-0.52
17123	*INCENP*	0.020499	-0.35193	-1.2304	-1.4507	-0.52
17124	*SHQ1*	0.065948	-0.53346	-1.095	-1.8724	-0.52
17125	*ZNF138*	1.1135	-1.1388	-1.5375	-1.7078	-0.52
17126	*UBE2V1*	1.0072	-0.5409	-2.0303	-2.1198	-0.52
17127	*TRA2B*	-0.18283	-0.27462	-1.1089	-1.4607	-0.52
17128	*ITFG2*	0.25715	-0.2263	-1.5976	-2.1361	-0.52
17129	*LGI2*	2.2085	-1.4741	-2.3036	-2.6343	-0.52
17130	*TRIM31*	0.6523	-0.62476	-1.5978	-1.6597	-0.52
17131	*ARHGEF35*	0.11062	-0.28928	-1.392	-1.9296	-0.52
17132	*ARHGAP11A*	0.38653	-0.79365	-1.1644	-1.1904	-0.52
17133	*PNPT1*	0.009675	-0.0175	-1.5649	-2.1755	-0.52
17134	*SLC25A30*	-0.24501	-0.31052	-1.0172	-2.0613	-0.52
17135	*UGT2B28*	0.71957	-0.77805	-1.5153	-1.55	-0.52
17136	*ATRN*	0.19524	-0.83773	-0.93217	-1.329	-0.52
17137	*RPS19*	1.4112	-1.4684	-1.5182	-1.6604	-0.53
17138	*PTGES*	0.67358	-0.86494	-1.3851	-1.7235	-0.53
17139	*BARX2*	0.2313	-0.89494	-0.91283	-1.258	-0.53
17140	*FAM96A*	0.23847	-0.32558	-1.4894	-1.5756	-0.53
17141	*BFSP1*	-0.21557	-0.44353	-0.91793	-1.714	-0.53
17142	*NCOR2*	-0.09468	-0.43488	-1.0475	-1.4754	-0.53
17143	*SPCS3*	0.33517	-0.85163	-1.0621	-1.1384	-0.53
17144	*GTPBP10*	1.4618	-1.1765	-1.864	-2.9931	-0.53
17145	*SULT1A4*	0.24869	-0.69502	-1.1343	-1.6526	-0.53
17146	*CSH2*	-0.01493	-0.68032	-0.88634	-1.7963	-0.53
17147	*COX6A1*	0.11649	-0.39248	-1.3058	-1.4034	-0.53
17148	*OTOF*	-0.1715	-0.25606	-1.1545	-2.0042	-0.53
17149	*RPS18*	0.42874	-0.91642	-1.0953	-1.6434	-0.53
17150	*SSTR1*	0.49299	-0.72095	-1.3565	-1.4185	-0.53
17151	*SLC35F3*	0.30105	-0.6066	-1.2815	-1.7496	-0.53

17152	*ZNF257*	-0.05909	-0.45491	-1.0734	-1.4715	-0.53
17153	*PPDPF*	-0.24773	-0.57546	-0.76437	-0.86158	-0.53
17154	*POLD1*	0.64251	-0.29054	-1.94	-3.4611	-0.53
17155	*WDR61*	0.82532	-1.1011	-1.3125	-1.6383	-0.53
17156	*GATAD2B*	-0.09182	-0.21955	-1.2786	-2.5414	-0.53
17157	*SYCP3*	-0.36868	-0.44043	-0.78096	-1.5781	-0.53
17158	*MLANA*	0.65646	-0.61264	-1.6339	-2.0697	-0.53
17159	*DENND1C*	-0.14859	-0.61092	-0.83278	-1.1685	-0.53
17160	*TMPRSS6*	-0.08207	-0.08601	-1.4246	-1.9789	-0.53
17161	*MRPL12*	0.31394	-0.49941	-1.4103	-1.4252	-0.53
17162	*ISY1-RAB43*	-0.02583	-0.08265	-1.4873	-1.7874	-0.53
17163	*RPRD1B*	-0.11437	-0.30834	-1.1734	-1.9768	-0.53
17164	*COA3*	-0.17281	-0.44353	-0.98103	-1.3346	-0.53
17165	*ARID3A*	1.055	-1.1461	-1.5068	-1.8849	-0.53
17166	*OR1L6*	0.66958	-1.1224	-1.1463	-1.3837	-0.53
17167	*RPL41*	0.6239	-0.81152	-1.4156	-1.8041	-0.53
17168	*PIGH*	-0.46583	-0.47472	-0.66675	-0.98902	-0.54
17169	*HUWE1*	0.52236	-0.89518	-1.2356	-1.5052	-0.54
17170	*SETDB2*	2.067	-1.5125	-2.163	-2.224	-0.54
17171	*SEC61G*	0.48253	-0.39603	-1.695	-1.773	-0.54
17172	*OR4F29*	-0.44353	-0.45663	-0.70993	-2.0062	-0.54
17173	*RASSF3*	-0.45656	-0.55891	-0.59462	-1.6626	-0.54
17174	*PDE6G*	1.1539	-0.76366	-2.0016	-3.4888	-0.54
17175	*TREM2*	-0.28717	-0.65417	-0.67137	-2.1477	-0.54
17176	*TMEM8B*	1.0895	-1.244	-1.4597	-2.4078	-0.54
17177	*OR5I1*	0.57298	-1.0344	-1.1533	-1.433	-0.54
17178	*FAM25A*	0.11779	-0.781	-0.95224	-1.8129	-0.54
17179	*GPRASP1*	0.75553	-0.50872	-1.8623	-1.9163	-0.54
17180	*WDR47*	0.17526	-0.01493	-1.7766	-1.7874	-0.54
17181	*NPAS3*	0.34144	-0.77697	-1.181	-1.6071	-0.54
17182	*WSB1*	0.58786	-0.95933	-1.2474	-1.6435	-0.54
17183	*HEXA*	-0.08395	-0.44353	-1.0917	-1.4998	-0.54
17184	*HDGFRP2*	1.7733	-1.4027	-1.99	-2.7277	-0.54
17185	*SUGCT*	-0.301	-0.58561	-0.73612	-0.81044	-0.54
17186	*BAZ1B*	-0.23339	-0.58058	-0.80936	-1.2576	-0.54
17187	*NDST2*	-0.24262	-0.6573	-0.72341	-1.622	-0.54
17188	*MT1M*	0.39528	-0.86047	-1.1585	-1.3976	-0.54
17189	*MBOAT4*	-0.16205	-0.18125	-1.2815	-2.2064	-0.54
17190	*EIF3C*	0.22156	-0.85739	-0.99059	-2.1143	-0.54
17191	*SYBU*	0.19377	-0.79225	-1.0283	-2.8174	-0.54
17192	*PRKCB*	2.2641	-1.4644	-2.4268	-3.2057	-0.54
17193	*CGB5*	0.59007	-1.0726	-1.1446	-1.8557	-0.54
17194	*KIAA0355*	-0.3552	-0.47485	-0.79711	-1.8157	-0.54
17195	*P3H4*	0.61834	0.091413	-2.337	-2.4481	-0.54
17196	*ZNHIT1*	-0.09459	-0.33315	-1.2004	-1.5943	-0.54
17197	*NDUFV1*	0.19772	-0.36885	-1.4584	-1.4841	-0.54

17198	*ZNF543*	0.28432	-0.86506	-1.049	-2.1523	-0.54
17199	*ZNF251*	0.75465	-1.1421	-1.2438	-1.4893	-0.54
17200	*MTA1*	0.083912	-0.17554	-1.5408	-1.571	-0.54
17201	*RIC8B*	0.7514	-0.76854	-1.6158	-2.0208	-0.54
17202	*FDXACB1*	0.54445	-0.26714	-1.9107	-5.1074	-0.54
17203	*PTCD1*	0.54005	-0.24569	-1.9305	-2.7509	-0.55
17204	*GEMIN7*	-0.03303	-0.40731	-1.1959	-1.4423	-0.55
17205	*GPANK1*	0.44576	-0.26177	-1.8212	-2.4394	-0.55
17206	*SRSF6*	1.1921	-1.3465	-1.4829	-2.0363	-0.55
17207	*XKR5*	0.76884	-0.97786	-1.4287	-2.7941	-0.55
17208	*DIMT1*	0.043666	-0.83874	-0.84411	-2.565	-0.55
17209	*PHB*	-0.39997	-0.49987	-0.74013	-1.6785	-0.55
17210	*FBP1*	0.29731	-0.39932	-1.539	-1.9494	-0.55
17211	*BRSK2*	-0.32637	-0.61779	-0.69915	-1.1807	-0.55
17212	*TXNRD1*	-0.38461	-0.44353	-0.81545	-2.4047	-0.55
17213	*RPL13*	0.3407	-0.347	-1.6373	-1.7963	-0.55
17214	*DNAJC25-GNG10*	0.4896	-0.64826	-1.4852	-1.8348	-0.55
17215	*TMEM138*	-0.28335	-0.67321	-0.68813	-1.1845	-0.55
17216	*NHP2L1*	0.19141	-0.6951	-1.141	-1.9865	-0.55
17217	*SOX12*	0.48795	-0.2875	-1.8454	-3.0687	-0.55
17218	*ARMC12*	-0.26891	-0.60048	-0.77559	-1.2302	-0.55
17219	*HEY2*	0.65856	-0.85739	-1.4485	-2.5308	-0.55
17220	*RPLP2*	1.1085	-1.2034	-1.5525	-1.6047	-0.55
17221	*TCP10*	0.58763	-0.46757	-1.7702	-3.3455	-0.55
17222	*TTYH1*	0.19908	-0.69286	-1.1566	-1.2936	-0.55
17223	*AARS2*	0.98058	-1.1676	-1.4636	-2.1187	-0.55
17224	*ADAMTSL3*	0.90732	-0.63971	-1.9195	-2.4015	-0.55
17225	*SARAF*	-0.16546	-0.51133	-0.97588	-1.8858	-0.55
17226	*HPGDS*	-0.19614	-0.24148	-1.2171	-3.0154	-0.55
17227	*TRIP6*	0.74282	-0.63405	-1.7652	-1.9534	-0.55
17228	*ADCK3*	0.6609	-0.79788	-1.5195	-1.8275	-0.55
17229	*NAT2*	-0.08556	-0.76033	-0.8108	-2.3265	-0.55
17230	*EIF2B5*	0.64019	-1.1263	-1.1713	-1.2692	-0.55
17231	*ATXN10*	-0.0083	-0.75099	-0.89832	-1.8629	-0.55
17232	*PAPOLG*	-0.34356	-0.35665	-0.95803	-1.8112	-0.55
17233	*ST3GAL5*	1.0411	-1.3453	-1.3542	-1.6218	-0.55
17234	*CACNG7*	0.047153	-0.68706	-1.0187	-1.2995	-0.55
17235	*DUSP12*	-0.25794	-0.31884	-1.0819	-2.0423	-0.55
17236	*CCNA2*	-0.37678	-0.55815	-0.72488	-0.98434	-0.55
17237	*CASK*	-0.14207	-0.16377	-1.3542	-1.7109	-0.55
17238	*XRCC4*	0.71631	-0.84523	-1.5348	-3.5736	-0.55
17239	*WIZ*	0.3454	-0.36877	-1.6413	-1.7971	-0.55
17240	*SLC38A10*	0.58487	-0.44246	-1.8076	-2.4607	-0.56
17241	*DDB1*	0.2083	-0.86494	-1.0093	-1.7745	-0.56
17242	*VEZT*	0.28506	-0.59791	-1.3542	-1.6663	-0.56
17243	*HMGXB3*	0.48317	-0.57404	-1.5762	-1.8606	-0.56

17244	*LIMCH1*	0.72405	-0.76655	-1.6251	-3.301	-0.56
17245	*HERC2*	-0.0757	-0.54739	-1.0448	-1.1048	-0.56
17246	*SEH1L*	0.66963	-1.151	-1.1881	-1.2867	-0.56
17247	*SERPINB3*	0.2066	-0.69561	-1.1817	-1.6867	-0.56
17248	*SPC25*	-0.11594	-0.50453	-1.0528	-1.8208	-0.56
17249	*CCM2L*	0.23953	-0.26055	-1.6526	-2.0421	-0.56
17250	*TUBB2A*	-0.37929	-0.60781	-0.68684	-0.79043	-0.56
17251	*GCSH*	0.074813	-0.22048	-1.5289	-1.5959	-0.56
17252	*MMRN2*	0.34354	-0.42415	-1.5954	-2.614	-0.56
17253	*VAV3*	0.3629	-0.90549	-1.1337	-1.1825	-0.56
17254	*PRAM1*	-0.44951	-0.59521	-0.63172	-1.2572	-0.56
17255	*ACP6*	0.16799	-0.60494	-1.2399	-1.2837	-0.56
17256	*DMP1*	0.049395	0.013351	-1.7397	-1.7935	-0.56
17257	*C16orf13*	-0.28012	-0.6093	-0.78863	-2.1576	-0.56
17258	*TBC1D3*	-0.49539	-0.5837	-0.59937	-2.0642	-0.56
17259	*ANKRD16*	-0.01885	-0.81087	-0.8489	-1.7855	-0.56
17260	*HYAL1*	-0.08207	-0.74572	-0.85163	-1.3593	-0.56
17261	*PLD2*	0.67212	-0.93878	-1.4128	-1.6027	-0.56
17262	*EIF3A*	-0.3703	-0.43251	-0.87737	-1.5544	-0.56
17263	*LIAS*	1.1993	-1.3948	-1.4852	-1.8456	-0.56
17264	*NECAB1*	0.40902	-0.93314	-1.1566	-2.0384	-0.56
17265	*NCKAP1*	-0.03704	-0.50828	-1.1354	-1.677	-0.56
17266	*AP3B1*	0.098257	-0.29186	-1.4873	-1.9686	-0.56
17267	*LSM6*	-0.50676	-0.54537	-0.62881	-1.3264	-0.56
17268	*MAP1S*	0.16173	-0.15085	-1.6934	-2.3534	-0.56
17269	*NUS1*	0.08783	-0.86123	-0.91013	-1.165	-0.56
17270	*WDR88*	0.81063	-1.0481	-1.4473	-2.1187	-0.56
17271	*SLC13A1*	2.1575	-1.8642	-1.9789	-2.9471	-0.56
17272	*SOAT2*	0.50568	-1.0019	-1.19	-1.4049	-0.56
17273	*TRAPPC4*	0.47342	-0.74013	-1.4197	-2.6024	-0.56
17274	*ANO8*	0.062547	-0.83143	-0.91823	-1.1587	-0.56
17275	*NUPR1*	0.79418	-1.2163	-1.2655	-2.4718	-0.56
17276	*SNX16*	-0.15209	-0.75944	-0.77661	-1.0261	-0.56
17277	*SNX29*	0.93709	-1.0947	-1.5306	-1.6255	-0.56
17278	*CNP*	-0.10661	-0.22292	-1.3611	-2.1354	-0.56
17279	*MAU2*	0.061984	-0.81313	-0.93967	-1.0748	-0.56
17280	*HYAL3*	-0.05926	-0.37045	-1.2616	-2.071	-0.56
17281	*NRARP*	0.13043	-0.78823	-1.0338	-1.2429	-0.56
17282	*AKR7A2*	0.6852	-1.1067	-1.2717	-1.5722	-0.56
17283	*XKR8*	-0.48254	-0.5581	-0.65291	-1.4942	-0.56
17284	*TOP1*	0.51396	-0.75076	-1.4579	-2.3118	-0.56
17285	*ASGR2*	-0.3337	-0.60147	-0.76055	-1.6525	-0.57
17286	*BTF3*	-0.25265	-0.38349	-1.06	-1.36	-0.57
17287	*UBXN4*	0.18898	-0.86047	-1.0255	-1.1866	-0.57
17288	*SRRM3*	0.3314	-0.43264	-1.5959	-2.2449	-0.57
17289	*AKAP12*	0.8377	-0.60658	-1.9284	-3.1665	-0.57

17290	*HEATR4*	0.2896	-0.25844	-1.7296	-2.3573	-0.57
17291	*UPK1B*	0.76505	-1.2261	-1.2386	-1.5103	-0.57
17292	*DGKB*	0.70768	-0.39775	-2.0113	-2.7254	-0.57
17293	*DDX56*	-0.32505	-0.62514	-0.75227	-0.77401	-0.57
17294	*INTS5*	0.22032	-0.82848	-1.0954	-1.9065	-0.57
17295	*CYP2R1*	0.72838	-0.8514	-1.5814	-1.7914	-0.57
17296	*PLCB4*	-0.21893	-0.58771	-0.89866	-1.3242	-0.57
17297	*IARS2*	0.62503	-1.0741	-1.2566	-1.7956	-0.57
17298	*CCDC130*	-0.41803	-0.48021	-0.80909	-2.0126	-0.57
17299	*PSMB4*	0.33302	-0.80567	-1.2365	-1.3003	-0.57
17300	*DDX11*	-0.39759	-0.44353	-0.86903	-0.87673	-0.57
17301	*MEPE*	0.52306	-0.73071	-1.5039	-1.507	-0.57
17302	*PIGY*	0.31212	0.31074	-2.3357	-2.4912	-0.57
17303	*HIST1H4K*	-0.24196	-0.68416	-0.78676	-1.1284	-0.57
17304	*NAA30*	1.035	-1.3183	-1.4312	-2.0542	-0.57
17305	*CLDN22*	-0.36035	-0.596	-0.75876	-1.8154	-0.57
17306	*NCCRP1*	-0.03976	-0.76966	-0.90768	-0.95545	-0.57
17307	*ZBTB10*	-0.07743	-0.23638	-1.4046	-2.0384	-0.57
17308	*NPRL3*	1.3795	-1.2167	-1.882	-2.9338	-0.57
17309	*MRPL3*	0.29893	-0.83631	-1.1872	-2.8928	-0.57
17310	*SLC25A15*	-0.18414	-0.38361	-1.1575	-1.5305	-0.58
17311	*FGF2*	-0.44353	-0.50793	-0.77418	-2.1648	-0.58
17312	*SPG7*	0.60515	-0.94052	-1.3903	-1.5837	-0.58
17313	*LSG1*	-0.00975	-0.67764	-1.0395	-1.388	-0.58
17314	*ICA1*	0.35232	-1.0231	-1.0564	-1.6561	-0.58
17315	*NEURL3*	-0.28571	-0.4187	-1.0228	-3.1568	-0.58
17316	*RSPH6A*	0.31179	-0.37358	-1.6663	-2.2473	-0.58
17317	*GTF3C1*	0.17359	-0.70193	-1.2006	-2.381	-0.58
17318	*MINOS1-NBL1*	1.6754	-1.5354	-1.8698	-2.1066	-0.58
17319	*TNMD*	0.49695	-0.56069	-1.6663	-1.9678	-0.58
17320	*NLRP3*	0.25068	-0.48131	-1.4996	-1.8157	-0.58
17321	*FAM69B*	-0.47944	-0.48646	-0.7647	-0.9562	-0.58
17322	*HMMR*	0.59762	-0.7503	-1.5781	-1.9513	-0.58
17323	*GTSF1*	0.019815	-0.00564	-1.7459	-2.3222	-0.58
17324	*RHBDF2*	1.4195	-1.1169	-2.0351	-6.0397	-0.58
17325	*PSMD14*	0.12199	-0.46951	-1.385	-1.5138	-0.58
17326	*C15orf52*	0.33119	0.021696	-2.0871	-2.1687	-0.58
17327	*MPND*	0.27706	-0.91374	-1.0989	-1.9811	-0.58
17328	*PVRL1*	-0.24019	-0.62307	-0.87318	-2.3795	-0.58
17329	*TREX1*	0.27744	-0.32279	-1.6921	-2.1648	-0.58
17330	*C11orf31*	0.29798	-0.7512	-1.2867	-1.5965	-0.58
17331	*C20orf27*	0.33101	-0.63405	-1.438	-1.6001	-0.58
17332	*USP17L19*	0.12853	-0.27421	-1.5957	-2.1132	-0.58
17333	*NDUFB4*	0.1696	-0.4433	-1.4685	-1.8906	-0.58
17334	*SRGAP2B*	0.3929	-0.46767	-1.6676	-1.8066	-0.58
17335	*TP53I3*	0.11456	-0.8478	-1.01	-1.1921	-0.58

17336	*IKBKAP*	-0.3129	-0.71381	-0.71839	-0.75128	-0.58
17337	*SDF2L1*	1.2521	-1.1429	-1.8557	-1.8636	-0.58
17338	*GBAS*	-0.29865	-0.34443	-1.1035	-1.1804	-0.58
17339	*SEMA6D*	0.45531	-0.18353	-2.0197	-4.1058	-0.58
17340	*EHBP1L1*	0.34151	-0.21666	-1.8735	-1.9789	-0.58
17341	*CSE1L*	1.0214	-1.3068	-1.4638	-1.6707	-0.58
17342	*SLC27A1*	-0.29953	-0.71093	-0.74011	-2.7689	-0.58
17343	*LRRC37B*	1.0789	-0.85392	-1.9757	-2.5452	-0.58
17344	*DHX9*	0.40797	-0.71717	-1.4416	-1.9736	-0.58
17345	*RYR3*	0.26123	-0.98314	-1.0297	-1.2071	-0.58
17346	*C4orf36*	1.3952	-1.5054	-1.6419	-1.8557	-0.58
17347	*GABARAP*	0.97532	-1.13	-1.5992	-2.2075	-0.58
17348	*PMF1-BGLAP*	-0.29124	-0.41575	-1.0469	-1.8872	-0.58
17349	*RPL14*	-0.40893	-0.44353	-0.90256	-1.8759	-0.59
17350	*DPH2*	-0.00546	-0.76366	-0.98653	-1.7766	-0.59
17351	*ZNF586*	-0.13231	-0.56427	-1.0596	-1.5306	-0.59
17352	*STAMBPL1*	0.29943	-0.77418	-1.2816	-2.2385	-0.59
17353	*YPEL5*	-0.37194	-0.42701	-0.95858	-1.5877	-0.59
17354	*RPS24*	0.22514	-0.27155	-1.7119	-2.6101	-0.59
17355	*TMEM136*	-0.15514	-0.53684	-1.0669	-1.5671	-0.59
17356	*CPTP*	0.27172	-0.65764	-1.3736	-1.7855	-0.59
17357	*TRIM63*	-0.23805	-0.47263	-1.0493	-1.4234	-0.59
17358	*VPS36*	-0.48492	-0.59662	-0.67887	-2.221	-0.59
17359	*MUL1*	-0.24904	-0.25583	-1.2568	-2.0752	-0.59
17360	*RAD1*	0.028732	-0.8934	-0.89802	-1.8355	-0.59
17361	*ENO3*	0.8205	-1.209	-1.3743	-1.6726	-0.59
17362	*FOXD1*	0.11948	-0.92711	-0.95545	-2.6805	-0.59
17363	*DHX33*	-0.39224	-0.57513	-0.79697	-0.85612	-0.59
17364	*AKR1A1*	0.07886	-0.83756	-1.0057	-1.4837	-0.59
17365	*SLC25A4*	0.47629	-0.73546	-1.5073	-2.5226	-0.59
17366	*HSPB7*	-0.32153	-0.38098	-1.0644	-2.2108	-0.59
17367	*N6AMT1*	-0.19704	-0.76671	-0.80332	-1.7072	-0.59
17368	*FAM189A2*	0.71357	-1.0328	-1.448	-1.917	-0.59
17369	*GRIPAP1*	-0.39494	-0.52343	-0.85027	-0.91283	-0.59
17370	*DCHS2*	-0.07094	-0.46476	-1.2338	-1.7807	-0.59
17371	*B3GNT6*	-0.20663	-0.68869	-0.87418	-1.0092	-0.59
17372	*KRT34*	-0.08474	-0.78784	-0.89741	-1.356	-0.59
17373	*ZDHHC21*	1.2305	-1.0131	-1.9899	-2.3949	-0.59
17374	*CTAG2*	0.52515	-0.36084	-1.9372	-1.9493	-0.59
17375	*FBXL14*	1.3733	-0.93011	-2.2169	-2.4633	-0.59
17376	*LTC4S*	0.63328	-1.0987	-1.3085	-1.8154	-0.59
17377	*MSANTD3*	-0.26564	-0.65053	-0.8604	-1.2684	-0.59
17378	*GART*	-0.09644	-0.71849	-0.96183	-1.0564	-0.59
17379	*GNL1*	0.045319	-0.24273	-1.5795	-2.0021	-0.59
17380	*OR13C9*	0.08921	-0.83696	-1.0297	-1.2198	-0.59
17381	*PLCZ1*	0.37148	-0.59678	-1.5524	-1.6908	-0.59

17382	*WRAP73*	0.11212	-0.92811	-0.9619	-1.7526	-0.59
17383	*ATP7A*	0.91454	-1.1612	-1.5314	-1.7253	-0.59
17384	*MED18*	-0.01973	-0.84086	-0.91766	-1.29	-0.59
17385	*LRP4*	0.44865	-0.36336	-1.864	-2.5865	-0.59
17386	*ZNFX1*	-0.15481	-0.50718	-1.118	-1.5244	-0.59
17387	*TCAF2*	-0.03757	-0.49908	-1.244	-2.2413	-0.59
17388	*IARS*	0.32523	-0.31091	-1.7956	-2.0818	-0.59
17389	*TPGS1*	0.054102	-0.69333	-1.1426	-1.279	-0.59
17390	*FAM60A*	0.68421	-0.97499	-1.4929	-2.4331	-0.59
17391	*POLA2*	0.093724	-0.49007	-1.3875	-1.8115	-0.59
17392	*C11orf91*	0.48187	-1.1011	-1.165	-1.9945	-0.59
17393	*KCND3*	1.2042	-1.3128	-1.6759	-2.0407	-0.59
17394	*GPX4*	0.50949	-1.107	-1.1872	-1.5375	-0.59
17395	*FKBPL*	-0.07288	-0.44056	-1.2716	-1.6941	-0.60
17396	*GPR68*	0.17412	-0.65877	-1.3009	-2.0283	-0.60
17397	*ZFAND2B*	1.8564	-1.7268	-1.9175	-2.2344	-0.60
17398	*CLEC4A*	0.25407	-0.53361	-1.5095	-2.6289	-0.60
17399	*ZBTB32*	0.64194	-0.48659	-1.9446	-2.1412	-0.60
17400	*AGO1*	0.18967	-0.44374	-1.5375	-1.6893	-0.60
17401	*KCNJ2*	0.00553	-0.47254	-1.3255	-1.4981	-0.60
17402	*WDR12*	0.43389	-0.65512	-1.5719	-2.5442	-0.60
17403	*RCOR1*	0.66495	-1.219	-1.2404	-1.7386	-0.60
17404	*AKAP4*	0.035088	-0.87318	-0.95708	-2.2593	-0.60
17405	*C16orf58*	0.74244	-1.2386	-1.3009	-2.1342	-0.60
17406	*GBF1*	-0.44056	-0.54714	-0.81101	-1.3379	-0.60
17407	*BAZ1A*	-0.47099	-0.62307	-0.70546	-1.0685	-0.60
17408	*PYDC2*	0.4092	-0.74086	-1.4686	-2.1662	-0.60
17409	*PTGER4*	0.52775	-0.62307	-1.7056	-2.0548	-0.60
17410	*ADAM30*	0.035844	-0.19053	-1.6463	-2.1687	-0.60
17411	*LRG1*	-0.26093	-0.7443	-0.79641	-1.3604	-0.60
17412	*BTG3*	0.14824	-0.91433	-1.0364	-2.3222	-0.60
17413	*BCAP29*	0.87867	-1.2339	-1.4473	-1.4683	-0.60
17414	*CYP2S1*	1.071	-1.2772	-1.5965	-2.3689	-0.60
17415	*PARD6A*	-0.08812	-0.83497	-0.88159	-1.7696	-0.60
17416	*LUC7L3*	0.9719	-0.94368	-1.8333	-1.8825	-0.60
17417	*KIAA0754*	0.31254	-0.64338	-1.4745	-2.2606	-0.60
17418	*GXYLT2*	0.24831	-0.34819	-1.7073	-2.0062	-0.60
17419	*DPP4*	0.097548	-0.74638	-1.1587	-2.7465	-0.60
17420	*AKIRIN2*	1.1481	-1.0737	-1.882	-2.6201	-0.60
17421	*C10orf10*	-0.25056	-0.45194	-1.1055	-1.5663	-0.60
17422	*HIST2H2BE*	0.45699	-0.90606	-1.359	-1.4994	-0.60
17423	*AADACL4*	-0.35769	-0.62588	-0.82628	-1.3209	-0.60
17424	*FZD9*	-0.00527	-0.443	-1.364	-1.9326	-0.60
17425	*SNN*	-0.48085	-0.49499	-0.83773	-1.7512	-0.60
17426	*FAM217B*	0.14522	-0.59662	-1.3633	-2.3615	-0.60
17427	*CENPI*	0.43331	-1.009	-1.2393	-1.5282	-0.60

17428	*PAPPA*	0.55941	-1.122	-1.2525	-2.283	-0.61
17429	*CENPL*	0.23212	-0.38131	-1.6663	-2.1612	-0.61
17430	*FCHSD1*	1.7484	-1.6952	-1.8689	-2.2064	-0.61
17431	*RPEL1*	0.50263	-1.1282	-1.1912	-1.2291	-0.61
17432	*VWA3B*	0.5181	-0.96846	-1.3666	-1.4243	-0.61
17433	*ACTR2*	0.28933	-0.70708	-1.3995	-1.9605	-0.61
17434	*HOOK1*	0.69476	-1.067	-1.4469	-2.7427	-0.61
17435	*GYS1*	0.88158	-1.3427	-1.3597	-1.754	-0.61
17436	*SIGLEC9*	0.14773	-0.53584	-1.433	-1.8121	-0.61
17437	*PANO1*	0.22012	-1.0087	-1.0335	-1.4963	-0.61
17438	*SUSD3*	-0.06687	-0.26443	-1.491	-2.8928	-0.61
17439	*ABCC8*	1.4805	-1.5965	-1.7068	-1.8885	-0.61
17440	*ZNF703*	0.4727	-0.61114	-1.6866	-1.8879	-0.61
17441	*POLH*	0.14975	-0.73728	-1.2399	-2.2292	-0.61
17442	*CPEB4*	0.67749	-0.99508	-1.5099	-1.5949	-0.61
17443	*ADAMTS3*	-0.13276	-0.31126	-1.3835	-1.6604	-0.61
17444	*MROH7*	0.038212	-0.81007	-1.0564	-1.1117	-0.61
17445	*DCTN6*	0.29113	-0.3328	-1.7879	-2.075	-0.61
17446	*CEACAM5*	0.59967	-1.0934	-1.3363	-2.3949	-0.61
17447	*LOC101060389*	0.019987	-0.28457	-1.567	-1.7754	-0.61
17448	*GRIN2B*	-0.18133	-0.7299	-0.92078	-1.7667	-0.61
17449	*RTBDN*	0.79892	-0.81536	-1.8157	-2.0171	-0.61
17450	*CCDC174*	-0.00831	-0.71421	-1.1097	-3.2595	-0.61
17451	*NUCB1*	0.22181	-0.88142	-1.1731	-1.2079	-0.61
17452	*KRTAP10-12*	0.29928	-0.93621	-1.1974	-1.6218	-0.61
17453	*RGPD6*	0.43414	-0.77	-1.4999	-1.6952	-0.61
17454	*C5orf67*	-0.38796	-0.43746	-1.011	-1.4883	-0.61
17455	*USP17L29*	0.70433	-1.2261	-1.3162	-1.5904	-0.61
17456	*DAPK2*	-0.03184	-0.74638	-1.0602	-2.3452	-0.61
17457	*U2SURP*	0.085671	-0.29442	-1.6302	-1.6848	-0.61
17458	*NBPF6*	0.4079	-0.92828	-1.3203	-1.6523	-0.61
17459	*PTPRH*	0.28293	-0.78229	-1.3427	-1.635	-0.61
17460	*DUX4*	0.57518	-1.1189	-1.2985	-1.3393	-0.61
17461	*ZFYVE27*	0.54494	-1.0645	-1.3237	-1.6682	-0.61
17462	*ARHGAP15*	0.10613	-0.77323	-1.177	-1.8435	-0.61
17463	*TMA16*	-0.04439	-0.48862	-1.3127	-1.5522	-0.62
17464	*AP1G2*	-0.53574	-0.58404	-0.7264	-1.9746	-0.62
17465	*TSSK6*	0.93459	-0.98099	-1.8002	-1.8207	-0.62
17466	*ZMAT5*	0.15724	-0.57865	-1.4277	-1.5343	-0.62
17467	*TMOD4*	0.00962	-0.36957	-1.4893	-1.8382	-0.62
17468	*RHD*	0.67615	-1.049	-1.4772	-1.7149	-0.62
17469	*SLC6A6*	-0.06641	-0.42373	-1.3604	-2.0538	-0.62
17470	*MFAP4*	0.39585	-0.60183	-1.6447	-2.1198	-0.62
17471	*MORN3*	0.36371	-1.0576	-1.1571	-2.0242	-0.62
17472	*TNFSF13*	0.50078	-0.5528	-1.7991	-3.1998	-0.62
17473	*GPSM1*	-0.24764	-0.37824	-1.2258	-1.3172	-0.62

17474	*DEFA1*	-0.12603	-0.84996	-0.87639	-1.8044	-0.62
17475	*PMP22*	-0.26638	-0.44353	-1.1429	-1.8995	-0.62
17476	*VARS2*	0.14287	-0.82069	-1.175	-1.3216	-0.62
17477	*BIRC2*	-0.37692	-0.56485	-0.91127	-1.0172	-0.62
17478	*PSMD7*	-0.12416	-0.71939	-1.0095	-1.4081	-0.62
17479	*PLEKHF1*	-0.49953	-0.59903	-0.75529	-2.3811	-0.62
17480	*EMC2*	0.75467	-0.75287	-1.8557	-2.6431	-0.62
17481	*PKDCC*	0.31744	-0.60845	-1.5635	-3.2582	-0.62
17482	*HS6ST3*	0.53339	-0.99033	-1.3977	-1.8975	-0.62
17483	*SEPT12*	0.58309	-0.9481	-1.4905	-2.3017	-0.62
17484	*FAM153B*	-0.22572	-0.41917	-1.2114	-1.8869	-0.62
17485	*FST*	0.25115	-1.027	-1.0809	-1.6268	-0.62
17486	*CRYAB*	-0.28188	-0.60113	-0.97477	-1.2935	-0.62
17487	*ZNF506*	-0.48086	-0.50227	-0.87466	-2.6024	-0.62
17488	*TTLL7*	0.6183	-1.0878	-1.3892	-1.7726	-0.62
17489	*FCF1*	0.11223	-0.96173	-1.0095	-1.2471	-0.62
17490	*CIB4*	-0.04665	-0.50924	-1.3033	-2.3235	-0.62
17491	*NPY2R*	-0.02799	-0.78229	-1.049	-2.0548	-0.62
17492	*SLC35B3*	0.47395	-1.0297	-1.3039	-1.8157	-0.62
17493	*KRT8*	0.62261	-0.5204	-1.962	-2.0181	-0.62
17494	*RTN3*	-0.31524	-0.64851	-0.89699	-1.7718	-0.62
17495	*BIVM*	0.17047	-0.15375	-1.8775	-2.0423	-0.62
17496	*C20orf195*	0.41231	-1.1337	-1.1395	-1.7554	-0.62
17497	*RAC3*	0.14867	-1.001	-1.0091	-1.8686	-0.62
17498	*WNK4*	-0.14649	-0.73546	-0.97982	-2.6762	-0.62
17499	*KLHDC7A*	-0.32121	-0.50992	-1.0315	-1.7994	-0.62
17500	*FBN1*	1.7454	-1.6424	-1.9668	-2.7863	-0.62
17501	*CEP350*	0.61051	-1.1571	-1.3189	-2.0483	-0.62
17502	*NTF3*	2.0855	-1.8568	-2.095	-2.6201	-0.62
17503	*EFTUD2*	-0.15375	-0.26892	-1.4448	-1.9997	-0.62
17504	*FAM109B*	-0.44353	-0.60214	-0.82256	-1.1148	-0.62
17505	*CREG1*	0.29824	-0.71363	-1.4533	-1.499	-0.62
17506	*PCDHB14*	1.1595	-1.1133	-1.9165	-2.4454	-0.62
17507	*MGAT4C*	0.093139	-0.38574	-1.578	-1.6746	-0.62
17508	*UPF3B*	-0.41456	-0.61972	-0.83909	-1.5947	-0.62
17509	*MFAP3L*	1.0793	-1.4248	-1.5289	-1.6143	-0.62
17510	*HNF1B*	-0.18871	-0.64851	-1.0383	-3.4957	-0.63
17511	*PDIA5*	2.0603	-1.828	-2.1085	-2.4141	-0.63
17512	*AP1G1*	0.2464	-1.0019	-1.1209	-1.5482	-0.63
17513	*PTPN3*	0.64633	-1.0231	-1.4997	-1.8282	-0.63
17514	*POLR3B*	0.35548	-0.7458	-1.4864	-1.8523	-0.63
17515	*PAX1*	0.2081	-0.77853	-1.3085	-1.3704	-0.63
17516	*NBAS*	-0.40961	-0.44353	-1.0259	-1.8724	-0.63
17517	*AMBP*	1.419	-1.4557	-1.8443	-1.9473	-0.63
17518	*NAPA*	-0.35757	-0.75774	-0.76743	-2.6201	-0.63
17519	*ZNF773*	0.25444	-0.51805	-1.62	-2.4456	-0.63

17520	*TDGF1*	-0.1024	-0.62307	-1.1595	-1.4132	-0.63
17521	*TOMM5*	-0.03675	-0.77963	-1.0707	-1.1244	-0.63
17522	*GPR137B*	0.51181	-0.61169	-1.7874	-2.796	-0.63
17523	*NMD3*	0.91245	-1.2258	-1.5741	-2.0454	-0.63
17524	*FAM187B*	0.43825	-1.1144	-1.2114	-1.8908	-0.63
17525	*ISM1*	-0.34376	-0.65293	-0.89178	-1.2332	-0.63
17526	*VARS*	-0.38971	-0.42015	-1.0799	-1.3128	-0.63
17527	*CENPC*	0.53546	-0.73865	-1.6867	-1.864	-0.63
17528	*MRPL41*	0.37471	-1.0797	-1.1868	-2.1424	-0.63
17529	*YWHAH*	-0.57899	-0.62306	-0.6901	-1.6597	-0.63
17530	*DCAF17*	0.018313	-0.84147	-1.071	-1.3358	-0.63
17531	*ICOS*	0.52794	-0.9442	-1.4789	-1.6186	-0.63
17532	*ATF1*	0.89324	-1.2135	-1.577	-1.6339	-0.63
17533	*RBPJ*	1.2675	-1.463	-1.7018	-2.0146	-0.63
17534	*VWA5A*	0.56381	-1.1566	-1.3055	-2.5008	-0.63
17535	*BTBD18*	-0.05066	-0.44198	-1.4057	-1.6403	-0.63
17536	*RHNO1*	0.79291	-1.2342	-1.4574	-2.1612	-0.63
17537	*ANKRD24*	-0.27857	-0.62581	-0.99489	-1.5752	-0.63
17538	*TRIM48*	0.45813	-0.5613	-1.7963	-1.9997	-0.63
17539	*TCF21*	-0.509	-0.6683	-0.72237	-1.8503	-0.63
17540	*ALX1*	-0.32699	-0.35038	-1.2234	-1.2339	-0.63
17541	*TKT*	-0.11099	-0.16055	-1.6296	-1.643	-0.63
17542	*RTP4*	-0.03565	-0.60028	-1.2655	-1.4028	-0.63
17543	*ASNSD1*	-0.2613	-0.66321	-0.97792	-1.5204	-0.63
17544	*RETSAT*	0.48331	-1.1354	-1.251	-1.8076	-0.63
17545	*PSMD3*	-0.06143	-0.47158	-1.3701	-1.5981	-0.63
17546	*SLCO2B1*	0.63483	-1.0291	-1.5099	-2.3424	-0.63
17547	*ECT2L*	-0.08586	-0.30677	-1.5125	-1.635	-0.64
17548	*EVX1*	-0.07612	-0.72825	-1.1008	-3.1492	-0.64
17549	*SLC16A13*	-0.18241	-0.53845	-1.1851	-1.2276	-0.64
17550	*TPMT*	-0.02943	-0.12908	-1.748	-2.7171	-0.64
17551	*SP1*	-0.02342	-0.05759	-1.8256	-2.2895	-0.64
17552	*SAE1*	1.6604	-1.7591	-1.8081	-1.9695	-0.64
17553	*TMX2*	0.39507	-0.79305	-1.5113	-1.5195	-0.64
17554	*NIPSNAP1*	-0.01323	-0.14649	-1.7507	-4.2184	-0.64
17555	*GRAMD2*	-0.12784	-0.86403	-0.91913	-1.5988	-0.64
17556	*HSPA4L*	-0.47964	-0.49525	-0.93661	-1.6963	-0.64
17557	*SUMF1*	0.92778	-0.49926	-2.3405	-2.3618	-0.64
17558	*CDS2*	0.54846	-0.71987	-1.7407	-2.0553	-0.64
17559	*PPP1R7*	0.4035	-0.88702	-1.4287	-2.6376	-0.64
17560	*ZFYVE9*	1.0588	-0.8284	-2.1429	-2.8174	-0.64
17561	*RRAGC*	-0.42254	-0.50524	-0.98572	-2.2599	-0.64
17562	*RBFOX2*	0.17653	-0.9612	-1.13	-1.1354	-0.64
17563	*BNIP1*	0.2836	-1.0871	-1.1126	-2.4058	-0.64
17564	*ETNK2*	-0.42373	-0.68192	-0.8108	-2.0453	-0.64
17565	*ZNF416*	-0.21692	-0.79573	-0.90382	-1.0506	-0.64

17566	*TEKT2*	0.038256	-0.33139	-1.624	-3.454	-0.64
17567	*TNRC6A*	0.81111	-1.0704	-1.6602	-2.3768	-0.64
17568	*CHAF1A*	0.47506	-1.035	-1.3606	-1.707	-0.64
17569	*TNNI3K*	-0.05665	-0.82256	-1.0415	-1.438	-0.64
17570	*MAPKAPK5*	0.27065	-1.0318	-1.1613	-1.2474	-0.64
17571	*PRPF38B*	0.45421	-1.1711	-1.2058	-1.8076	-0.64
17572	*PTPN20*	-0.59981	-0.61164	-0.71134	-1.7316	-0.64
17573	*SMAD4*	0.38881	-0.91103	-1.4017	-1.7845	-0.64
17574	*HDAC7*	0.11561	-0.48352	-1.5561	-1.7766	-0.64
17575	*NOL9*	-0.09774	-0.4102	-1.4185	-2.0071	-0.64
17576	*ZNF284*	-0.56947	-0.66865	-0.69146	-1.8371	-0.64
17577	*EIF4EBP1*	1.2385	-1.4252	-1.7438	-3.0595	-0.64
17578	*CA12*	0.61693	-1.1382	-1.4098	-1.8508	-0.64
17579	*DYNLRB1*	-0.32433	-0.52329	-1.0863	-1.6802	-0.64
17580	*TMEM45B*	0.97477	-1.1925	-1.7185	-2.6343	-0.65
17581	*AGXT2*	0.20005	-1.0614	-1.0753	-1.5568	-0.65
17582	*SETBP1*	0.17909	-0.75437	-1.3637	-1.7364	-0.65
17583	*SRRM1*	0.61176	-1.028	-1.5235	-1.5941	-0.65
17584	*CKAP5*	-0.29925	-0.38527	-1.2569	-1.9073	-0.65
17585	*UXT*	-0.4433	-0.4796	-1.0204	-1.8591	-0.65
17586	*DPH1*	0.16922	-0.69866	-1.4141	-2.9031	-0.65
17587	*SPAST*	-0.2122	-0.43385	-1.2985	-1.6486	-0.65
17588	*KANSL2*	-0.22569	-0.71561	-1.0036	-1.3433	-0.65
17589	*MED13*	-0.46752	-0.47528	-1.0041	-1.9912	-0.65
17590	*POTEE*	-0.17606	-0.72666	-1.0467	-1.683	-0.65
17591	*OR11H1*	0.13776	-1.0359	-1.0529	-2.4858	-0.65
17592	*CHRDL1*	-0.07432	-0.8458	-1.0315	-1.1739	-0.65
17593	*TMEM116*	1.102	-1.4473	-1.6075	-1.677	-0.65
17594	*PRCD*	0.40904	-0.33179	-2.0301	-2.3915	-0.65
17595	*ARFGEF2*	0.3113	-0.84786	-1.418	-1.6597	-0.65
17596	*PIGU*	0.18009	-1.0413	-1.0953	-1.1692	-0.65
17597	*CABIN1*	0.16326	-0.18722	-1.9326	-2.9736	-0.65
17598	*STAT5A*	-0.42204	-0.64268	-0.89186	-1.0951	-0.65
17599	*HIST2H2AA3*	0.21343	-0.99123	-1.1793	-2.5226	-0.65
17600	*DPPA2*	0.57469	-0.95708	-1.5752	-1.9168	-0.65
17601	*EXOSC2*	0.031293	-0.36242	-1.6268	-1.9954	-0.65
17602	*CYP27B1*	-0.17058	-0.7819	-1.0055	-1.4832	-0.65
17603	*SPCS1*	0.46022	-0.81058	-1.6086	-3.0769	-0.65
17604	*WDR46*	0.62769	-1.2932	-1.2935	-1.5579	-0.65
17605	*TNFAIP1*	0.13115	-0.57824	-1.5132	-1.9367	-0.65
17606	*ZNF519*	-0.23722	-0.63643	-1.0871	-2.3496	-0.65
17607	*SNRPF*	0.21449	-0.98211	-1.1932	-1.392	-0.65
17608	*ZNF566*	0.07554	-1.0178	-1.0187	-1.9757	-0.65
17609	*OR4B1*	-0.04449	-0.90484	-1.012	-1.3957	-0.65
17610	*RPL23A*	0.11134	-0.91409	-1.1591	-2.1177	-0.65
17611	*TMEM178A*	1.0275	-1.1189	-1.8707	-3.1273	-0.65

17612	*NCKAP5*	-0.54851	-0.56485	-0.8504	-1.3226	-0.65
17613	*WBP4*	-0.0164	-0.7443	-1.205	-1.3078	-0.66
17614	*USP17L26*	0.62542	-1.0229	-1.57	-1.8016	-0.66
17615	*SLCO3A1*	-0.17264	-0.82524	-0.96962	-2.3154	-0.66
17616	*MRPL53*	-0.39532	-0.5701	-1.0025	-1.316	-0.66
17617	*TSPAN11*	0.28527	-0.78951	-1.4639	-3.8314	-0.66
17618	*AGBL5*	-0.25915	-0.56225	-1.1472	-2.4557	-0.66
17619	*TNK2*	0.62609	-0.90772	-1.6877	-2.3517	-0.66
17620	*SPDYE2B*	0.98428	-0.97477	-1.9794	-1.9811	-0.66
17621	*MKNK2*	-0.32262	-0.53661	-1.1124	-1.8219	-0.66
17622	*ADGRG4*	0.12433	-0.20202	-1.8946	-2.7191	-0.66
17623	*FAHD2B*	-0.09468	-0.77969	-1.0995	-1.5514	-0.66
17624	*MYRIP*	-0.31035	-0.50979	-1.1538	-1.4407	-0.66
17625	*SOD2*	1.739	-1.7783	-1.9351	-2.0218	-0.66
17626	*DCTN4*	0.060407	-0.91968	-1.1199	-1.723	-0.66
17627	*ADAR*	0.33513	-1.0302	-1.2845	-1.5448	-0.66
17628	*TLE2*	-0.46749	-0.51467	-0.99892	-1.2829	-0.66
17629	*THUMPD1*	-0.15953	-0.75512	-1.067	-1.252	-0.66
17630	*RCL1*	-0.10527	-0.75491	-1.122	-1.6116	-0.66
17631	*WNT10A*	0.60803	-1.2817	-1.3092	-1.7963	-0.66
17632	*CT45A10*	-0.61521	-1.9624	0.59412	-0.07993	-0.66
17633	*SRSF12*	0.27752	-0.91061	-1.3505	-1.7608	-0.66
17634	*HAUS7*	-0.5271	-0.67417	-0.7827	-1.0229	-0.66
17635	*ETV3*	-0.13461	-0.89699	-0.95321	-1.0884	-0.66
17636	*FGFBP3*	-0.21157	-0.81501	-0.95877	-1.2208	-0.66
17637	*FGF10*	0.51887	-0.85236	-1.6525	-1.7829	-0.66
17638	*ZNF175*	-0.10032	-0.80636	-1.0801	-2.1347	-0.66
17639	*ACTR3*	-0.38198	-0.62969	-0.97565	-1.6208	-0.66
17640	*STXBP3*	0.45719	-1.0187	-1.426	-2.4557	-0.66
17641	*SCARF2*	-0.01352	-0.23368	-1.7407	-2.0614	-0.66
17642	*GPR26*	0.59211	-0.9918	-1.5883	-2.2161	-0.66
17643	*GPR162*	0.5443	-1.0661	-1.4688	-1.6524	-0.66
17644	*VPS28*	0.052674	-0.75626	-1.2884	-3.2887	-0.66
17645	*RTN4*	-0.273	-0.37282	-1.3479	-1.3604	-0.66
17646	*ZCCHC11*	0.49507	-1.1709	-1.3198	-1.7745	-0.67
17647	*HOPX*	-0.57484	-0.59738	-0.82524	-0.93885	-0.67
17648	*FEV*	-0.0606	-0.41502	-1.5227	-2.4633	-0.67
17649	*YWHAE*	0.007297	-0.53012	-1.4775	-1.8462	-0.67
17650	*GCAT*	-0.45821	-0.50809	-1.0351	-1.2284	-0.67
17651	*ARHGEF7*	0.66952	-1.1191	-1.5524	-1.5738	-0.67
17652	*PREB*	0.51657	-1.1713	-1.3473	-2.239	-0.67
17653	*DDIAS*	-0.46765	-0.67629	-0.86027	-1.3727	-0.67
17654	*GIPR*	-0.46756	-0.76055	-0.77895	-1.3478	-0.67
17655	*CYB5R3*	0.69306	-0.76206	-1.9395	-2.2357	-0.67
17656	*PPP1R26*	0.043556	-0.08575	-1.9664	-2.3602	-0.67
17657	*IMP4*	-0.11619	-0.50752	-1.3852	-1.4085	-0.67

17658	*PCDHB3*	0.87394	-1.1158	-1.7675	-2.3696	-0.67
17659	*CHST10*	0.56404	-1.0158	-1.5582	-1.9833	-0.67
17660	*PSENEN*	0.088102	-0.5866	-1.5116	-2.3636	-0.67
17661	*ALKBH7*	0.84949	-1.2159	-1.6447	-1.6974	-0.67
17662	*CAPN10*	0.1357	-0.68917	-1.459	-1.5712	-0.67
17663	*BCAS4*	1.065	-1.525	-1.5525	-1.7219	-0.67
17664	*SRSF11*	-0.50878	-0.58315	-0.9216	-1.5212	-0.67
17665	*MAGEF1*	1.2884	-1.4788	-1.8253	-2.278	-0.67
17666	*SLC7A6OS*	0.055414	-0.61429	-1.4577	-2.0623	-0.67
17667	*PCDHGB5*	-0.09789	-0.83101	-1.0878	-1.3617	-0.67
17668	*OR52K2*	-0.01351	-0.68422	-1.3191	-1.7743	-0.67
17669	*POU2AF1*	0.083863	-0.36549	-1.7364	-2.1334	-0.67
17670	*SLC10A3*	0.044461	-0.73389	-1.3293	-1.9321	-0.67
17671	*SAFB2*	-0.27933	-0.44687	-1.2936	-1.8212	-0.67
17672	*KLHDC8A*	-0.41079	-0.54544	-1.0644	-1.2324	-0.67
17673	*JRKL*	-0.02205	-0.85018	-1.1487	-2.2607	-0.67
17674	*LONRF1*	0.80812	-1.0648	-1.7652	-2.0851	-0.67
17675	*MRPL43*	0.28332	-0.76379	-1.5415	-2.2804	-0.67
17676	*PI16*	0.32753	-0.85194	-1.4998	-2.0277	-0.67
17677	*TDP1*	0.99469	-1.3665	-1.6524	-2.3036	-0.67
17678	*PASK*	-0.28248	-0.68386	-1.0589	-1.0954	-0.68
17679	*PRKCI*	0.35076	-0.78097	-1.5954	-1.6453	-0.68
17680	*IL36RN*	-0.23565	-0.74365	-1.047	-1.0511	-0.68
17681	*DAGLA*	1.04	-1.1309	-1.9362	-2.0337	-0.68
17682	*TINF2*	0.54633	-1.0283	-1.5458	-2.0153	-0.68
17683	*PAK1IP1*	0.55829	-1.1043	-1.4824	-1.7413	-0.68
17684	*MEIS2*	0.005184	-0.97595	-1.0582	-1.438	-0.68
17685	*MOSPD3*	-0.38826	-0.64149	-0.9999	-1.0344	-0.68
17686	*CATSPER1*	-0.53856	-0.68825	-0.80295	-1.1585	-0.68
17687	*PSMD2*	0.68144	-1.309	-1.4024	-1.5375	-0.68
17688	*IRF2BP2*	-0.42609	-0.48982	-1.1149	-2.0134	-0.68
17689	*ADSL*	-0.16263	-0.74192	-1.1263	-1.5898	-0.68
17690	*TFAP2C*	-0.38319	-0.67842	-0.96993	-1.754	-0.68
17691	*NDUFA1*	-0.30153	-0.81017	-0.92131	-1.438	-0.68
17692	*RGL1*	0.64372	-1.3231	-1.3542	-1.9997	-0.68
17693	*OSGIN1*	-0.27599	-0.65145	-1.1075	-1.3728	-0.68
17694	*SPN*	1.1706	-1.4789	-1.7268	-1.8523	-0.68
17695	*TBCD*	0.33017	-1.154	-1.2135	-1.4299	-0.68
17696	*SSRP1*	-0.41794	-0.57261	-1.0483	-1.7496	-0.68
17697	*EID3*	-0.51467	-0.61796	-0.90993	-2.1018	-0.68
17698	*MBD5*	0.20565	-0.60289	-1.6463	-1.72	-0.68
17699	*GPN1*	-0.13025	-0.45552	-1.4588	-1.9837	-0.68
17700	*NONO*	-0.22082	-0.74743	-1.078	-1.7266	-0.68
17701	*PDCD1*	1.3111	-1.0559	-2.3017	-3.6868	-0.68
17702	*DSCC1*	-0.32677	-0.78503	-0.93489	-1.9821	-0.68
17703	*AARSD1*	0.35094	-1.0741	-1.3237	-2.4518	-0.68

17704	*LOC388282*	-0.00139	-0.36297	-1.6847	-1.7049	-0.68
17705	*PCID2*	-0.01323	-0.29546	-1.7412	-2.9031	-0.68
17706	*ABHD10*	-0.20774	-0.83961	-1.0049	-1.8779	-0.68
17707	*GLDN*	0.63841	-1.3165	-1.3756	-1.7874	-0.68
17708	*BUD31*	0.45103	-1.043	-1.462	-1.8629	-0.68
17709	*RD3*	0.05378	-0.83402	-1.274	-1.3478	-0.68
17710	*DARS2*	0.00553	-0.9346	-1.1303	-1.5364	-0.69
17711	*NME6*	0.099654	-0.94597	-1.2135	-1.2411	-0.69
17712	*ZNF672*	-0.34751	-0.66164	-1.0517	-2.8863	-0.69
17713	*RRAGB*	0.22609	-0.74013	-1.5485	-1.9375	-0.69
17714	*KAT2A*	0.5884	-1.1745	-1.4773	-1.6617	-0.69
17715	*CPNE5*	0.57489	-1.1463	-1.4929	-1.8963	-0.69
17716	*VPS39*	-0.09334	-0.87997	-1.091	-1.2719	-0.69
17717	*CLDN5*	-0.43276	-0.6981	-0.93407	-1.0707	-0.69
17718	*SPOP*	0.012881	-0.82208	-1.2571	-1.6934	-0.69
17719	*TRMT61B*	1.3287	-1.5806	-1.8157	-2.7509	-0.69
17720	*SPNS2*	0.78934	-1.0838	-1.7745	-1.9523	-0.69
17721	*NPAT*	-0.61358	-0.65714	-0.79832	-2.6201	-0.69
17722	*PFAS*	-0.02738	-0.88008	-1.162	-1.3209	-0.69
17723	*LPAR1*	-0.33198	-0.79641	-0.94184	-1.2032	-0.69
17724	*IGLL1*	0.42349	-1.238	-1.2576	-1.5897	-0.69
17725	*YPEL1*	0.76534	-1.3948	-1.4428	-1.9065	-0.69
17726	*C3orf62*	-0.44353	-0.60604	-1.0239	-1.5598	-0.69
17727	*ZNF823*	0.40742	-1.0185	-1.4625	-1.4873	-0.69
17728	*ZC3H11A*	-0.13063	-0.76965	-1.1752	-1.3199	-0.69
17729	*PRPF4B*	-0.20663	-0.43634	-1.433	-1.5502	-0.69
17730	*SYT8*	0.27528	-0.90663	-1.4452	-1.4852	-0.69
17731	*PTPN21*	0.88094	-1.1825	-1.7766	-3.8028	-0.69
17732	*IL1R1*	0.32657	-0.71464	-1.6907	-2.1983	-0.69
17733	*TMEM156*	-0.1097	-0.67809	-1.2915	-1.6469	-0.69
17734	*LYZL6*	-0.01401	-0.80176	-1.2641	-1.3616	-0.69
17735	*ALG2*	-0.35716	-0.44353	-1.2792	-1.3818	-0.69
17736	*H2AFB3*	0.14964	-0.9512	-1.2792	-1.4692	-0.69
17737	*KARS*	-0.43313	-0.71353	-0.93901	-1.2455	-0.70
17738	*OR2A14*	0.03977	-0.97784	-1.1479	-2.1577	-0.70
17739	*INSM2*	0.17576	-1.1212	-1.1421	-1.2792	-0.70
17740	*TRAPPC11*	0.51874	-1.2659	-1.3406	-2.4917	-0.70
17741	*GTF2IRD2*	-0.48233	-0.66655	-0.93889	-1.8961	-0.70
17742	*CCDC138*	1.119	-1.3978	-1.8095	-2.4873	-0.70
17743	*NBPF15*	-0.17483	-0.89204	-1.0221	-2.6803	-0.70
17744	*MTOR*	0.77965	-1.3279	-1.5408	-2.0704	-0.70
17745	*SLC25A14*	1.3127	-1.5401	-1.8636	-1.9748	-0.70
17746	*ZNF806*	-0.05126	-0.94052	-1.1027	-1.1357	-0.70
17747	*PNPLA2*	0.76538	-1.4141	-1.4469	-1.9946	-0.70
17748	*RRM2*	0.46648	-1.2169	-1.3453	-2.7566	-0.70
17749	*C9orf50*	0.82918	-1.3805	-1.5448	-3.6181	-0.70

17750	*SAMD9*	0.27509	-0.63325	-1.738	-2.4815	-0.70
17751	*RPS4X*	-0.16209	-0.8656	-1.0695	-1.2186	-0.70
17752	*S100A7*	-0.42075	-0.76606	-0.91296	-1.8157	-0.70
17753	*APC2*	0.1689	-0.28012	-1.9894	-2.8718	-0.70
17754	*UBL3*	0.068008	-0.87719	-1.2915	-2.105	-0.70
17755	*AQP4*	-0.11845	-0.66136	-1.3226	-1.3492	-0.70
17756	*HCN1*	0.004551	-0.22378	-1.8836	-2.7042	-0.70
17757	*TVP23C*	0.8875	-1.3864	-1.6047	-2.0384	-0.70
17758	*MAP2K6*	-0.07138	-0.94441	-1.0884	-1.5576	-0.70
17759	*TLCD1*	0.43648	-0.91343	-1.6288	-1.6488	-0.70
17760	*HLF*	0.005894	-0.5605	-1.5512	-1.8941	-0.70
17761	*CATSPERD*	-0.02018	-0.12433	-1.962	-2.9323	-0.70
17762	*RAB9A*	0.36422	-1.0842	-1.3878	-4.7747	-0.70
17763	*FERMT2*	0.25142	-0.47367	-1.8871	-2.5495	-0.70
17764	*GAD1*	0.48074	-1.2249	-1.3663	-2.1965	-0.70
17765	*PIK3CD*	0.39116	-1.2132	-1.2895	-1.3627	-0.70
17766	*SRCAP*	0.27291	-0.43226	-1.9548	-2.9068	-0.70
17767	*MPP5*	-0.16337	-0.58289	-1.3701	-2.2533	-0.71
17768	*AKAP3*	-0.10103	-0.23166	-1.7845	-1.9622	-0.71
17769	*ELOF1*	-0.13429	-0.60353	-1.3808	-2.4901	-0.71
17770	*C7orf73*	0.002715	-0.8147	-1.3071	-1.6525	-0.71
17771	*KLHL33*	0.61874	-0.99958	-1.7386	-1.7804	-0.71
17772	*HTR2A*	-0.29327	-0.43043	-1.397	-1.7688	-0.71
17773	*RILPL2*	-0.01366	-0.256	-1.8516	-1.9089	-0.71
17774	*CHEK1*	0.1688	-0.39556	-1.8946	-2.0277	-0.71
17775	*SMC4*	0.30645	-0.78651	-1.6457	-1.9269	-0.71
17776	*ATP6V0C*	0.044225	-0.70463	-1.4655	-1.5263	-0.71
17777	*PPWD1*	-0.04057	-0.53971	-1.548	-2.7689	-0.71
17778	*PTCHD4*	0.43931	-0.79035	-1.7782	-2.9706	-0.71
17779	*NEDD1*	-0.43077	-0.5613	-1.1384	-2.0541	-0.71
17780	*POLDIP2*	0.7052	-1.278	-1.5579	-2.0226	-0.71
17781	*FAM172A*	0.29569	-1.1214	-1.3057	-2.1477	-0.71
17782	*CREBBP*	-0.51722	-0.68612	-0.92948	-2.0407	-0.71
17783	*PDPN*	-0.19735	-0.88272	-1.0528	-3.1751	-0.71
17784	*CDK12*	-0.50251	-0.69407	-0.93725	-2.2856	-0.71
17785	*BBS1*	1.624	-1.7935	-1.9673	-2.7191	-0.71
17786	*SCD*	0.3214	-1.0315	-1.4269	-1.9678	-0.71
17787	*OR8B2*	0.34105	-0.99838	-1.4798	-1.5577	-0.71
17788	*WWC3*	-0.3205	-0.49449	-1.3246	-3.087	-0.71
17789	*OVCA2*	-0.13111	-0.80978	-1.1991	-1.6386	-0.71
17790	*TBL3*	0.23609	-1.0425	-1.3346	-1.4683	-0.71
17791	*UBE2D3*	0.46439	-0.69407	-1.9116	-1.9967	-0.71
17792	*NOL10*	1.7071	-1.1833	-2.6675	-3.0769	-0.71
17793	*CDK2*	0.7963	-1.1888	-1.7526	-1.9975	-0.72
17794	*METAP1*	-0.15919	-0.81517	-1.1727	-2.033	-0.72
17795	*VENTX*	1.5262	-1.2151	-2.4595	-2.8316	-0.72

17796	*NIPBL*	-0.40534	-0.64943	-1.0945	-1.4206	-0.72
17797	*DARS*	-0.16607	-0.35548	-1.6324	-2.217	-0.72
17798	*PELI3*	-0.21809	-0.84603	-1.0914	-1.6442	-0.72
17799	*TUBA1A*	0.41564	-1.0048	-1.5672	-2.2955	-0.72
17800	*KCNQ1*	1.1159	-1.4625	-1.8101	-2.7149	-0.72
17801	*SLIT1*	-0.20227	-0.70463	-1.25	-1.367	-0.72
17802	*NCK2*	0.010955	-1.0033	-1.165	-2.5535	-0.72
17803	*GAGE2B*	0.1181	-0.9857	-1.2912	-1.5797	-0.72
17804	*SERPINA6*	-0.47835	-0.66604	-1.0149	-1.1759	-0.72
17805	*GTF3A*	-0.31294	-0.87067	-0.97595	-2.2403	-0.72
17806	*LRRC43*	0.49269	-0.85943	-1.7935	-1.8714	-0.72
17807	*RPN2*	-0.23368	-0.83272	-1.0942	-1.7338	-0.72
17808	*FAM181B*	-0.43471	-0.77284	-0.95321	-1.1016	-0.72
17809	*ATP6V0A1*	0.67727	-1.3542	-1.4842	-1.7215	-0.72
17810	*C14orf37*	1.1403	-1.3823	-1.9196	-2.9909	-0.72
17811	*ACAD10*	-0.14244	-0.44137	-1.5781	-1.9772	-0.72
17812	*ERVFRD-1*	-0.36728	-0.46741	-1.3288	-2.9931	-0.72
17813	*PRR23D2*	0.67588	-0.29209	-2.5477	-3.064	-0.72
17814	*AXDND1*	-0.17238	-0.47782	-1.5145	-1.5707	-0.72
17815	*CNOT3*	-0.3328	-0.82757	-1.0048	-1.4125	-0.72
17816	*CENPO*	-0.01255	-0.62453	-1.5284	-1.6027	-0.72
17817	*HIST1H2AJ*	0.6168	-1.1126	-1.6707	-1.9623	-0.72
17818	*RPS15A*	0.010089	-1.0718	-1.1048	-2.1419	-0.72
17819	*RPIA*	0.06743	-0.66462	-1.57	-1.7068	-0.72
17820	*SPP2*	-0.7004	-0.71032	-0.75778	-2.1407	-0.72
17821	*SP140L*	-0.20302	-0.61675	-1.3506	-1.3542	-0.72
17822	*ATP2B1*	0.14844	-0.88124	-1.439	-1.5018	-0.72
17823	*CT45A9*	-0.41486	-0.49727	-1.26	-1.6268	-0.72
17824	*HRH4*	-0.53304	-0.81485	-0.82514	-2.1496	-0.72
17825	*CA5A*	0.65432	-1.3403	-1.4886	-1.8355	-0.72
17826	*SCAI*	0.10845	-0.71988	-1.5635	-1.6893	-0.72
17827	*OR5H14*	0.38683	-0.89957	-1.6626	-5.1372	-0.73
17828	*PRAMEF10*	-0.52649	-0.72372	-0.9285	-1.509	-0.73
17829	*RGPD2*	0.59223	-1.0068	-1.7662	-1.9244	-0.73
17830	*NOB1*	-0.46024	-0.53133	-1.1893	-2.0066	-0.73
17831	*TEKT3*	-0.46741	-0.56634	-1.1472	-1.5182	-0.73
17832	*NISCH*	0.3523	-0.72085	-1.8126	-2.5878	-0.73
17833	*GMDS*	-0.59061	-0.75487	-0.83623	-1.9774	-0.73
17834	*JRK*	0.041958	-0.97552	-1.2498	-1.531	-0.73
17835	*KDELR3*	0.73014	-1.388	-1.526	-1.6787	-0.73
17836	*TP53TG3D*	-0.05492	-0.35972	-1.7702	-1.9984	-0.73
17837	*TCTEX1D2*	-0.26714	-0.95149	-0.96718	-1.7018	-0.73
17838	*LY6D*	-0.00832	-0.9311	-1.2474	-2.6582	-0.73
17839	*VWA5B2*	-0.46205	-0.72237	-1.0044	-1.4368	-0.73
17840	*DRC1*	-0.37287	-0.78362	-1.0328	-1.5663	-0.73
17841	*POLRMT*	-0.37925	-0.44353	-1.3679	-2.0071	-0.73

17842	*RTN4IP1*	0.37578	-0.79453	-1.7753	-3.096	-0.73
17843	*KCTD15*	-0.23196	-0.46953	-1.4941	-2.2165	-0.73
17844	*ELOVL7*	0.15149	-1.1214	-1.226	-3.2168	-0.73
17845	*PPP2CB*	0.50292	-1.2242	-1.4791	-1.5922	-0.73
17846	*PCGF1*	0.14884	-1.0168	-1.3331	-1.8528	-0.73
17847	*IMMT*	-0.00239	-1.0717	-1.1279	-1.9054	-0.73
17848	*MAFA*	-0.57001	-0.69972	-0.93368	-0.99694	-0.73
17849	*PRSS42*	0.16906	-0.79726	-1.5773	-1.8702	-0.74
17850	*VPS13D*	-0.45649	-0.81152	-0.93895	-2.0323	-0.74
17851	*ARMC10*	0.52646	-1.1564	-1.5773	-2.7931	-0.74
17852	*EPPK1*	0.2539	-0.87898	-1.583	-2.1685	-0.74
17853	*LOC101927572*	0.047789	-0.90532	-1.3526	-1.4415	-0.74
17854	*C20orf96*	0.006278	-0.92375	-1.2935	-1.2975	-0.74
17855	*LETMD1*	-0.52495	-0.75511	-0.93107	-1.9622	-0.74
17856	*THBS4*	-0.35899	-0.35986	-1.4951	-2.2473	-0.74
17857	*DTX1*	-0.33758	-0.91236	-0.96422	-1.2211	-0.74
17858	*POMP*	-0.20916	-0.85747	-1.1487	-1.575	-0.74
17859	*ITGA2*	0.29991	-0.98572	-1.5302	-2.0629	-0.74
17860	*ATPAF2*	-0.20771	-0.83163	-1.1772	-1.5583	-0.74
17861	*CCT7*	-0.1283	-1.0189	-1.0696	-1.2585	-0.74
17862	*RPL34*	0.186	-1.0467	-1.3565	-2.796	-0.74
17863	*OR5K4*	0.50691	-1.3288	-1.3961	-2.1155	-0.74
17864	*EGFLAM*	-0.3817	-0.73364	-1.1027	-1.4132	-0.74
17865	*CPLX1*	-0.30576	-0.81846	-1.0945	-1.9965	-0.74
17866	*UNC13C*	0.728	-0.90993	-2.0368	-2.1926	-0.74
17867	*AGK*	0.56295	-1.1108	-1.6709	-1.7911	-0.74
17868	*PCDHGA3*	-0.23447	-0.85199	-1.1332	-3.0502	-0.74
17869	*OXCT2*	-0.30863	-0.60262	-1.309	-1.6251	-0.74
17870	*HOXA13*	-0.26711	-0.62469	-1.3291	-1.5633	-0.74
17871	*CDH4*	-0.09723	-0.82757	-1.2995	-1.5082	-0.74
17872	*C6orf58*	-0.05684	-0.91096	-1.257	-2.9354	-0.74
17873	*ZNF275*	-0.4072	-0.69712	-1.1214	-1.6993	-0.74
17874	*SIK1*	0.3463	-1.1169	-1.4566	-1.9794	-0.74
17875	*KCNG3*	0.085425	-0.84523	-1.4683	-1.831	-0.74
17876	*PCYT1A*	-0.26055	-0.80226	-1.1656	-1.613	-0.74
17877	*MTMR2*	-0.24269	-0.73301	-1.2531	-2.0181	-0.74
17878	*MON1A*	0.98984	-1.3023	-1.9168	-2.7931	-0.74
17879	*CLEC4D*	-0.13341	-0.71063	-1.386	-1.8446	-0.74
17880	*MEFV*	-0.02502	-0.80146	-1.4043	-1.8238	-0.74
17881	*CCNYL1*	-0.26267	-0.76635	-1.2024	-1.3593	-0.74
17882	*BLNK*	-0.04729	-0.75452	-1.4306	-1.5026	-0.74
17883	*LGALS9B*	0.010873	-0.34553	-1.8979	-2.1648	-0.74
17884	*RNASE9*	-0.15715	-0.83961	-1.2358	-1.9558	-0.74
17885	*AMOTL2*	-0.21857	-0.22422	-1.7906	-2.2809	-0.74
17886	*CCT8*	-0.08664	-0.93114	-1.2158	-2.0904	-0.74
17887	*SF3B3*	0.20985	-0.59622	-1.8473	-2.1445	-0.74

17888	*RRP9*	-0.26161	-0.61892	-1.3542	-1.6184	-0.74
17889	*SDE2*	-0.12659	-0.44884	-1.6594	-2.262	-0.74
17890	*CBWD3*	-0.44353	-0.52495	-1.2677	-1.3617	-0.75
17891	*RITA1*	-0.10536	-0.67555	-1.4554	-3.0378	-0.75
17892	*NOMO2*	-0.7052	-0.75437	-0.77791	-0.78566	-0.75
17893	*ATAD5*	-0.03697	-0.92926	-1.2719	-1.5073	-0.75
17894	*C6orf141*	-0.37054	-0.81501	-1.0529	-1.2052	-0.75
17895	*TIPRL*	-0.20242	-0.99059	-1.046	-1.677	-0.75
17896	*FAM20B*	0.70861	-1.1989	-1.7488	-2.8388	-0.75
17897	*GPS1*	0.74794	-1.151	-1.8366	-2.7863	-0.75
17898	*MYT1*	1.7433	-1.9397	-2.0436	-2.5571	-0.75
17899	*NELFE*	-0.2347	-0.44574	-1.5598	-1.8869	-0.75
17900	*ELP4*	0.40947	-1.2357	-1.4142	-1.4375	-0.75
17901	*RAB7A*	1.0441	-1.5722	-1.7134	-1.7266	-0.75
17902	*CYHR1*	0.14445	-0.86384	-1.5227	-1.5471	-0.75
17903	*RBMXL3*	0.22516	-1.1796	-1.2901	-1.8305	-0.75
17904	*C4BPB*	0.023338	-0.83708	-1.4311	-2.2589	-0.75
17905	*SPIN3*	-0.27453	-0.98239	-0.98882	-1.9794	-0.75
17906	*ACOX3*	-0.00066	-0.50394	-1.7413	-2.3164	-0.75
17907	*PELO*	-0.21385	-0.93725	-1.0954	-1.8453	-0.75
17908	*THAP8*	0.17631	-0.84308	-1.583	-2.0697	-0.75
17909	*ZNF720*	-0.13871	-0.83739	-1.274	-2.8388	-0.75
17910	*ARL15*	-0.26125	-0.57573	-1.4132	-1.5795	-0.75
17911	*OAZ1*	0.47914	-1.2163	-1.5138	-1.6599	-0.75
17912	*POU3F2*	0.14471	-1.1763	-1.2195	-1.4741	-0.75
17913	*GEMIN4*	-0.16774	-0.63472	-1.4487	-1.7363	-0.75
17914	*KIF3B*	0.050178	-0.49846	-1.8031	-2.0116	-0.75
17915	*ZNF512B*	-0.14097	-0.84419	-1.2666	-1.5354	-0.75
17916	*BEST2*	-0.13963	-0.73673	-1.3769	-1.5552	-0.75
17917	*RAD9A*	-0.37417	-0.79481	-1.0852	-1.7285	-0.75
17918	*APLNR*	0.34839	-0.77159	-1.831	-2.9977	-0.75
17919	*KIF27*	-0.32824	-0.79641	-1.1333	-1.6435	-0.75
17920	*CABP4*	0.14337	-0.76379	-1.6386	-2.1921	-0.75
17921	*TNS1*	0.22021	-0.69748	-1.7822	-2.1648	-0.75
17922	*GPR17*	-0.53114	-0.7602	-0.96857	-1.0191	-0.75
17923	*SYNGR2*	0.068993	-0.1194	-2.2097	-2.4328	-0.75
17924	*VNN1*	0.18765	-0.78579	-1.6636	-3.3666	-0.75
17925	*ADAMTSL2*	-0.20227	-0.7443	-1.3173	-2.1723	-0.75
17926	*NUP214*	0.066172	-0.71161	-1.6186	-1.7413	-0.75
17927	*FOXP1*	0.19539	-1.1659	-1.2963	-1.5524	-0.76
17928	*GIT2*	0.19052	-0.6706	-1.7879	-1.8598	-0.76
17929	*MSR1*	0.26121	-0.74069	-1.7911	-1.9255	-0.76
17930	*SLC25A28*	0.36853	-1.1202	-1.5194	-3.6071	-0.76
17931	*OR3A2*	-0.53093	-0.75162	-0.98925	-2.0055	-0.76
17932	*LIMD1*	0.020714	-0.66073	-1.6335	-2.8174	-0.76
17933	*NOX1*	0.19462	-1.2118	-1.2566	-2.0384	-0.76

17934	*NDUFA12*	0.40229	-0.43674	-2.2397	-2.6561	-0.76
17935	*WNK1*	-0.30232	-0.92025	-1.0529	-2.7016	-0.76
17936	*RNF225*	-0.14289	-0.75099	-1.3818	-2.0016	-0.76
17937	*MIS18A*	-0.3643	-0.67441	-1.237	-1.4709	-0.76
17938	*LAD1*	-0.02618	-1.0528	-1.1975	-1.4986	-0.76
17939	*PAGE2B*	-0.06562	-0.76154	-1.4495	-2.0752	-0.76
17940	*MYO1C*	-0.36749	-0.79365	-1.1169	-1.4809	-0.76
17941	*ARID3B*	-0.0291	-1.0499	-1.2026	-1.5016	-0.76
17942	*PSME3*	0.16473	-0.89941	-1.547	-1.8631	-0.76
17943	*LIPK*	-0.60852	-0.80256	-0.87482	-2.071	-0.76
17944	*CCDC183*	-0.19943	-0.94777	-1.1391	-3.0179	-0.76
17945	*CCDC27*	0.16285	-0.9612	-1.4893	-2.1921	-0.76
17946	*MED6*	-0.21635	-0.99943	-1.073	-1.1328	-0.76
17947	*ACTB*	-0.0511	-0.91874	-1.3199	-2.0734	-0.76
17948	*PSMB3*	0.36646	-1.2677	-1.3892	-1.6949	-0.76
17949	*BATF3*	0.53295	-1.1067	-1.72	-2.4595	-0.76
17950	*KIAA0430*	0.5809	-1.238	-1.6368	-1.8609	-0.76
17951	*AGPAT3*	-0.50726	-0.75603	-1.0328	-2.2907	-0.77
17952	*MCM6*	1.1592	-1.5379	-1.918	-2.6851	-0.77
17953	*HPR*	0.004325	-1.0737	-1.2284	-1.9919	-0.77
17954	*LEO1*	-0.2539	-0.57546	-1.4689	-3.051	-0.77
17955	*CYGB*	0.040946	-0.23796	-2.103	-2.9834	-0.77
17956	*PCDH15*	0.504	-0.83808	-1.9664	-3.0408	-0.77
17957	*GNB2L1*	0.13599	-1.1384	-1.2989	-2.2485	-0.77
17958	*BTBD9*	0.10845	-1.1021	-1.3078	-2.4172	-0.77
17959	*PSME1*	-0.19806	-0.86884	-1.238	-3.2901	-0.77
17960	*SCN7A*	-0.43726	-0.9089	-0.95933	-1.3782	-0.77
17961	*LIMD2*	0.18898	-1.0528	-1.4423	-1.5971	-0.77
17962	*YRDC*	0.14087	-1.0948	-1.3542	-1.9099	-0.77
17963	*RASSF1*	-0.26128	-0.86725	-1.1807	-1.8929	-0.77
17964	*CLEC18A*	0.40022	-1.3416	-1.3711	-1.4789	-0.77
17965	*RPL6*	-0.43008	-0.56377	-1.3197	-1.7386	-0.77
17966	*UBE2G1*	0.12548	-1.1763	-1.2628	-2.0251	-0.77
17967	*WIPF3*	0.32903	-1.2169	-1.4258	-1.5375	-0.77
17968	*SPIN1*	-0.44134	-0.8828	-0.99298	-1.4998	-0.77
17969	*PGLYRP1*	-0.40191	-0.68992	-1.2261	-1.2782	-0.77
17970	*METAP2*	-0.53557	-0.75749	-1.0267	-1.7675	-0.77
17971	*SMC2*	0.30098	-0.48669	-2.1347	-2.4415	-0.77
17972	*POLR2I*	0.1739	-1.2109	-1.2845	-2.4328	-0.77
17973	*IRF2BP1*	-0.5335	-0.84674	-0.94171	-1.3593	-0.77
17974	*LRRC74B*	-0.59206	-0.76882	-0.96144	-1.3751	-0.77
17975	*IKBKB*	0.50414	-0.83624	-1.9906	-2.1984	-0.77
17976	*CDK7*	-0.4013	-0.8756	-1.0458	-2.3849	-0.77
17977	*WDR76*	0.005379	-0.86772	-1.461	-1.5212	-0.77
17978	*MTBP*	-0.44353	-0.86298	-1.017	-2.4825	-0.77
17979	*RPGRIP1*	-0.0359	-0.93107	-1.3567	-1.5883	-0.77

17980	*CRYM*	0.22609	-0.68727	-1.8631	-1.9495	-0.77
17981	*SLC35A4*	-0.0409	-0.69847	-1.5866	-1.6653	-0.78
17982	*NCOA2*	0.23385	-1.1015	-1.4597	-2.1089	-0.78
17983	*HSPG2*	0.17593	-1.1662	-1.3371	-1.6989	-0.78
17984	*PRDX3*	0.75804	-0.80897	-2.278	-2.2844	-0.78
17985	*SLC38A7*	-0.04874	-0.97303	-1.3074	-1.5393	-0.78
17986	*QARS*	0.18055	-1.2247	-1.2852	-1.5408	-0.78
17987	*ADRM1*	0.49005	-1.3457	-1.474	-1.486	-0.78
17988	*RPL36*	0.46067	-0.89424	-1.8963	-2.1319	-0.78
17989	*IL17D*	0.56384	-0.54097	-2.3537	-2.6041	-0.78
17990	*PPP1CC*	-0.05026	-0.62833	-1.6524	-1.8387	-0.78
17991	*KCNT2*	-0.25339	-1.03	-1.0482	-1.6193	-0.78
17992	*RUNX2*	-0.17994	-0.25425	-1.8975	-2.3195	-0.78
17993	*SPAG16*	-0.16415	-0.19552	-1.9734	-1.988	-0.78
17994	*GAB2*	0.23912	-1.1698	-1.4034	-1.8975	-0.78
17995	*VPS72*	-0.20848	-1.0482	-1.0782	-1.8646	-0.78
17996	*RRP1*	0.22922	-1.1727	-1.3918	-3.9492	-0.78
17997	*SS18L2*	-0.14701	-0.40498	-1.7836	-1.7905	-0.78
17998	*C6orf223*	-0.00126	-0.55441	-1.7817	-2.2165	-0.78
17999	*DUSP21*	-0.63643	-0.85027	-0.85296	-1.3573	-0.78
18000	*EIF3CL*	-0.30209	-0.63483	-1.4031	-1.53	-0.78
18001	*KRTAP6-1*	-0.04538	-0.57429	-1.7213	-2.3474	-0.78
18002	*HAPLN4*	-0.30756	-0.88199	-1.1545	-1.309	-0.78
18003	*RGS18*	0.79993	-1.1921	-1.9534	-2.3776	-0.78
18004	*EPM2A*	0.13796	-0.90154	-1.584	-2.1873	-0.78
18005	*C17orf102*	-0.34349	-0.77196	-1.2343	-1.8692	-0.78
18006	*DDOST*	-0.35015	-0.97303	-1.0268	-1.0602	-0.78
18007	*MFN2*	0.68743	-1.3646	-1.6734	-2.5489	-0.78
18008	*GLIPR2*	-0.13752	-0.41298	-1.8033	-2.6935	-0.78
18009	*RPL19*	-0.67716	-0.82293	-0.85623	-1.1136	-0.79
18010	*TBC1D3H*	-0.37417	-0.83138	-1.1524	-1.8073	-0.79
18011	*TMEM160*	1.1873	-1.1012	-2.4456	-5.7525	-0.79
18012	*G6PD*	-0.52477	-0.8239	-1.0111	-2.2075	-0.79
18013	*POM121L2*	-0.65455	-0.75472	-0.95068	-2.0126	-0.79
18014	*SMARCD1*	-0.54959	-0.8681	-0.94296	-2.1533	-0.79
18015	*ABCE1*	-0.39565	-0.43633	-1.5289	-1.7675	-0.79
18016	*RGMA*	-0.14646	-1.0564	-1.1585	-1.5026	-0.79
18017	*SEMA5B*	-0.16832	-0.71093	-1.4824	-1.525	-0.79
18018	*MAPK1*	0.20136	-1.1169	-1.4473	-2.1648	-0.79
18019	*FAM9A*	-0.33856	-1.0092	-1.0156	-1.237	-0.79
18020	*KLK14*	0.12094	-1.2215	-1.2633	-1.4811	-0.79
18021	*DEDD2*	0.12312	-0.96718	-1.5206	-1.6221	-0.79
18022	*IQCF6*	0.26885	-0.90161	-1.7323	-2.0249	-0.79
18023	*POM121C*	-0.06057	-0.87046	-1.4365	-1.5064	-0.79
18024	*NDC80*	-0.319	-0.62479	-1.4269	-3.7031	-0.79
18025	*FNTB*	-0.35308	-0.6602	-1.358	-1.3675	-0.79

18026	*LY6G6D*	-0.35694	-0.45748	-1.5569	-5.9834	-0.79
18027	*MUC1*	-0.37872	-0.7602	-1.2326	-1.6139	-0.79
18028	*C20orf196*	0.11191	-1.227	-1.2569	-1.5077	-0.79
18029	*IL31RA*	-0.00414	-1.1317	-1.2364	-1.4187	-0.79
18030	*RMDN2*	-0.34229	-0.781	-1.2505	-1.6594	-0.79
18031	*ARL2*	-0.07154	-0.68999	-1.6176	-2.0351	-0.79
18032	*LDB3*	-0.30237	-0.95321	-1.1247	-1.4588	-0.79
18033	*GTPBP4*	-0.55175	-0.90386	-0.92486	-1.3864	-0.79
18034	*CRADD*	0.75207	-0.78651	-2.3461	-2.7376	-0.79
18035	*ITGAE*	-0.13895	-1.0644	-1.1773	-2.5865	-0.79
18036	*HSPA5*	-0.36377	-0.64577	-1.3723	-1.5335	-0.79
18037	*KLK9*	-0.5731	-0.86123	-0.94815	-1.3835	-0.79
18038	*TMPPE*	-0.5635	-0.82375	-0.99619	-1.8443	-0.79
18039	*ITGA11*	-0.05322	-0.92553	-1.4048	-1.6934	-0.79
18040	*BATF*	0.45156	-0.82311	-2.012	-2.6096	-0.79
18041	*PAX4*	0.82699	-1.0951	-2.1158	-2.1407	-0.79
18042	*QPCTL*	-0.0702	-0.93725	-1.3766	-2.2046	-0.79
18043	*ANO1*	-0.28901	-0.73424	-1.3617	-2.0602	-0.79
18044	*VAPB*	0.15515	-0.11251	-2.428	-2.5725	-0.80
18045	*TUBG1*	0.25584	-0.88171	-1.7599	-2.5386	-0.80
18046	*PPIL3*	0.052522	-0.56518	-1.8737	-2.3615	-0.80
18047	*FBXW2*	-0.46747	-0.93188	-0.9879	-2.0593	-0.80
18048	*PRKCQ*	0.40269	-1.1836	-1.6069	-3.0608	-0.80
18049	*SQSTM1*	-0.31227	-0.86459	-1.2113	-1.7018	-0.80
18050	*RPS6KA5*	0.22057	-1.1713	-1.4401	-1.6276	-0.80
18051	*THEG*	-0.28336	-1.054	-1.0553	-1.0855	-0.80
18052	*GPAM*	0.40041	-1.3851	-1.4085	-1.714	-0.80
18053	*CD177*	-0.64751	-0.65692	-1.0907	-1.3097	-0.80
18054	*EIF5A*	-0.55898	-0.78004	-1.0564	-1.8154	-0.80
18055	*MAP4*	-0.35735	-1.0092	-1.0293	-1.3684	-0.80
18056	*MRPL38*	-0.11621	-1.042	-1.2393	-1.358	-0.80
18057	*GTF2H3*	-0.43473	-0.86027	-1.103	-1.9802	-0.80
18058	*POGZ*	-0.40112	-0.9935	-1.0048	-1.5781	-0.80
18059	*CETN2*	-0.36861	-0.79641	-1.2352	-1.6844	-0.80
18060	*USP10*	0.20962	-1.0905	-1.5206	-1.7169	-0.80
18061	*KCNH6*	0.52394	-1.3154	-1.6104	-1.9342	-0.80
18062	*GON4L*	-0.52496	-0.69815	-1.1808	-3.6071	-0.80
18063	*ZNHIT6*	-0.43385	-0.79596	-1.1743	-2.337	-0.80
18064	*TMX4*	-0.02488	-0.16207	-2.2172	-2.3036	-0.80
18065	*HNRNPM*	-0.00252	-0.60987	-1.7918	-1.8066	-0.80
18066	*CDT1*	-0.76513	-0.81368	-0.8259	-1.8771	-0.80
18067	*KPNA6*	0.21297	-0.96632	-1.6575	-1.8157	-0.80
18068	*SDR39U1*	0.37688	-0.98688	-1.802	-3.6459	-0.80
18069	*ACTA2*	-0.33821	-0.92375	-1.151	-2.0059	-0.80
18070	*SSTR5*	0.008446	-0.8462	-1.5765	-1.9442	-0.80
18071	*PIGV*	-0.12552	-0.90991	-1.3797	-1.3821	-0.81

18072	*NCBP1*	-0.19885	-0.73834	-1.4788	-1.6397	-0.81
18073	*LRFN5*	0.11514	-0.89794	-1.6356	-1.658	-0.81
18074	*ITGA10*	0.12694	-1.2436	-1.3025	-1.4015	-0.81
18075	*SIN3A*	-0.31654	-0.5775	-1.526	-1.6443	-0.81
18076	*MAGED4*	0.44581	-1.2297	-1.6368	-2.4784	-0.81
18077	*CCSAP*	-0.72466	-0.7272	-0.97029	-1.5735	-0.81
18078	*AGTR1*	-0.51304	-0.72553	-1.1836	-1.5212	-0.81
18079	*TCF24*	0.46058	-1.4416	-1.4421	-3.1229	-0.81
18080	*EMC3*	-0.34148	-0.61033	-1.4722	-1.742	-0.81
18081	*ATP2A3*	-0.39885	-0.81999	-1.2055	-2.641	-0.81
18082	*ANO7*	-0.40281	-0.95182	-1.0707	-1.8076	-0.81
18083	*SLC17A5*	0.02465	-0.56411	-1.8872	-2.264	-0.81
18084	*SLC8A3*	0.11412	-1.2342	-1.3071	-1.8115	-0.81
18085	*GPR107*	0.43085	-0.89794	-1.962	-2.236	-0.81
18086	*MICU2*	0.22852	-1.3226	-1.3354	-1.5861	-0.81
18087	*KBTBD8*	-0.27453	-0.74618	-1.4093	-1.9284	-0.81
18088	*USP31*	-0.11697	-0.43481	-1.8816	-2.4703	-0.81
18089	*CTBP1*	-0.01175	-1.1872	-1.2362	-1.6653	-0.81
18090	*SPINK4*	0.16764	-1.1351	-1.4684	-1.5752	-0.81
18091	*BPIFB6*	-0.32183	-0.95105	-1.1659	-1.9757	-0.81
18092	*IPO4*	-0.34327	-1.0231	-1.0741	-1.2343	-0.81
18093	*PPIAL4F*	0.52665	-1.2021	-1.7652	-2.2292	-0.81
18094	*USB1*	-0.38673	-0.87898	-1.1763	-1.2903	-0.81
18095	*MFSD9*	-0.30782	-0.37912	-1.7551	-1.838	-0.81
18096	*TARDBP*	0.20227	-0.74155	-1.9047	-2.0016	-0.81
18097	*TOR1B*	-0.01344	-0.88465	-1.5465	-1.624	-0.81
18098	*DEFB128*	0.21353	-1.2816	-1.3766	-2.0629	-0.81
18099	*MAPK1IP1L*	-0.04896	-1.1384	-1.2576	-1.4607	-0.81
18100	*KCNC2*	-0.5716	-0.89525	-0.97918	-1.3019	-0.82
18101	*CRY1*	0.26829	-1.0005	-1.7149	-2.5478	-0.82
18102	*CFLAR*	-0.08091	-1.0782	-1.2884	-1.7129	-0.82
18103	*MRPS21*	-0.57732	-0.61494	-1.2572	-1.5408	-0.82
18104	*ATP5L2*	0.25904	-1.0567	-1.6528	-2.0791	-0.82
18105	*GLG1*	0.68472	-1.525	-1.6105	-2.4642	-0.82
18106	*CDC25B*	-0.68565	-0.86287	-0.90687	-1.0424	-0.82
18107	*ARL3*	0.61327	-1.4919	-1.5782	-2.7379	-0.82
18108	*FAM183A*	0.21133	-1.2987	-1.3707	-1.9998	-0.82
18109	*OR8H3*	-0.21563	-0.86756	-1.3751	-1.9163	-0.82
18110	*CDK5RAP1*	0.29779	-1.3148	-1.4423	-2.071	-0.82
18111	*TACC3*	-0.52847	-0.95717	-0.97572	-1.2915	-0.82
18112	*TWSG1*	-0.28825	-0.89186	-1.2816	-2.2343	-0.82
18113	*TSPY8*	-0.66708	-0.71988	-1.078	-1.0898	-0.82
18114	*ALG1L*	-0.27801	-0.86047	-1.3267	-1.4926	-0.82
18115	*VCPKMT*	0.1973	-0.99608	-1.6682	-1.8775	-0.82
18116	*CEP19*	0.27723	-1.356	-1.3903	-1.6989	-0.82
18117	*UTP6*	0.23864	-1.3218	-1.386	-1.9367	-0.82

18118	*ATP2B3*	0.24021	-0.78956	-1.9219	-2.0384	-0.82
18119	*HOXD13*	-0.19997	-0.75824	-1.5138	-2.264	-0.82
18120	*NFATC1*	-0.06844	-1.0344	-1.3721	-3.2348	-0.82
18121	*CAD*	-0.35308	-0.92157	-1.202	-1.9107	-0.83
18122	*ATP5B*	-0.09922	-1.1856	-1.192	-1.3191	-0.83
18123	*C12orf42*	0.44675	-0.99871	-1.9269	-2.1873	-0.83
18124	*RHOBTB2*	-0.51592	-0.83276	-1.1327	-1.6316	-0.83
18125	*VIP*	-0.30454	-1.0258	-1.1511	-1.4607	-0.83
18126	*SIGLEC12*	0.18646	-1.1216	-1.5467	-1.6047	-0.83
18127	*PCNXL4*	-0.06343	-0.44353	-1.9758	-2.071	-0.83
18128	*WDFY4*	-0.08018	-1.1325	-1.2733	-1.72	-0.83
18129	*KRT32*	-0.47001	-0.93652	-1.0796	-2.2094	-0.83
18130	*POLR3D*	0.76582	-1.5918	-1.6604	-2.0953	-0.83
18131	*FAM229A*	0.073809	-0.88584	-1.6748	-2.4018	-0.83
18132	*ANKRD32*	-0.42177	-0.66251	-1.4039	-1.9622	-0.83
18133	*GLYR1*	-0.19364	-0.67146	-1.6246	-2.5934	-0.83
18134	*KRTCAP3*	0.67594	-0.8504	-2.3154	-3.6181	-0.83
18135	*ATP6V0B*	-0.15181	-1.0945	-1.2464	-1.3792	-0.83
18136	*EPG5*	0.098319	-1.1792	-1.4135	-2.1089	-0.83
18137	*U2AF2*	0.47249	-0.20512	-2.7623	-3.5278	-0.83
18138	*VN1R2*	-0.21508	-0.72877	-1.5524	-2.2694	-0.83
18139	*PRG3*	-0.43726	-0.87382	-1.1888	-1.4994	-0.83
18140	*CHIA*	-0.13688	-0.79485	-1.5687	-1.9865	-0.83
18141	*LCT*	-0.3337	-1.0335	-1.1337	-3.7932	-0.83
18142	*SMIM12*	-0.54197	-0.89501	-1.0641	-1.7068	-0.83
18143	*FBLN2*	-0.09493	-1.065	-1.3416	-1.3501	-0.83
18144	*HIST1H2AM*	-0.47857	-0.61663	-1.4087	-2.3063	-0.83
18145	*FAM185A*	0.033893	-0.50053	-2.0392	-2.1177	-0.84
18146	*GDAP2*	0.86091	-1.183	-2.1848	-2.4718	-0.84
18147	*MRPS26*	-0.77144	-0.81798	-0.91779	-2.2223	-0.84
18148	*SERPINB13*	0.8794	-1.5722	-1.8157	-2.4933	-0.84
18149	*C22orf42*	-0.0232	-1.1892	-1.297	-1.5434	-0.84
18150	*DBNDD1*	0.11133	-1.2098	-1.411	-1.5243	-0.84
18151	*CD22*	-0.47806	-0.76361	-1.268	-1.9168	-0.84
18152	*ABTB1*	-0.54884	-0.90206	-1.0589	-1.5604	-0.84
18153	*C8orf22*	0.003285	-1.1676	-1.348	-3.8124	-0.84
18154	*EID1*	0.79832	-1.3773	-1.9347	-2.4015	-0.84
18155	*ZNF2*	-0.77462	-0.8504	-0.89009	-1.3426	-0.84
18156	*ACRV1*	0.33948	-0.28352	-2.5725	-2.8776	-0.84
18157	*TEX13B*	-0.10704	-0.79149	-1.6218	-2.1217	-0.84
18158	*TRAPPC3*	-0.58289	-0.65189	-1.2867	-1.8759	-0.84
18159	*C18orf8*	-0.63575	-0.92157	-0.96572	-1.2639	-0.84
18160	*KLHL30*	0.13705	-0.52587	-2.1351	-2.1792	-0.84
18161	*MRPL33*	-0.61643	-0.85392	-1.0557	-1.5884	-0.84
18162	*TBC1D3C*	-0.56605	-0.97252	-0.98749	-1.9954	-0.84
18163	*ANTXRL*	-0.3464	-0.8934	-1.2864	-2.781	-0.84

18164	*CEP120*	-0.17298	-1.1522	-1.2027	-1.6557	-0.84
18165	*TRIM35*	-0.61344	-0.832	-1.0829	-1.1566	-0.84
18166	*CCHCR1*	0.20958	-0.85427	-1.8869	-2.0682	-0.84
18167	*HHIP*	0.37811	-0.95759	-1.9523	-1.962	-0.84
18168	*PRDM14*	-0.07472	-0.93465	-1.5225	-1.7287	-0.84
18169	*FIBP*	0.31949	-1.085	-1.7667	-2.4328	-0.84
18170	*RBM7*	-0.10243	-0.87257	-1.5604	-2.8288	-0.85
18171	*HSP90AA1*	0.15481	-1.2867	-1.4039	-2.412	-0.85
18172	*SH3TC1*	0.44466	-1.4614	-1.5212	-3.0961	-0.85
18173	*OTOGL*	0.28766	-0.98951	-1.8364	-2.0304	-0.85
18174	*CPSF1*	-0.72529	-0.78878	-1.0243	-1.6526	-0.85
18175	*MED12*	0.87228	-1.6861	-1.7261	-2.4112	-0.85
18176	*NPIPA5*	-0.45005	-0.81178	-1.2786	-1.4234	-0.85
18177	*CDC40*	-0.66731	-0.8518	-1.0224	-1.7911	-0.85
18178	*F3*	-0.57513	-0.87257	-1.0949	-2.0351	-0.85
18179	*CLDND2*	0.14973	-1.2935	-1.3993	-1.8966	-0.85
18180	*RPL10*	0.096242	-0.88584	-1.7538	-1.8695	-0.85
18181	*LSM5*	-0.45345	-0.50251	-1.5904	-2.01	-0.85
18182	*NEK6*	-0.66353	-0.72038	-1.1629	-1.3479	-0.85
18183	*KCNK18*	-0.15866	-0.8108	-1.5781	-1.658	-0.85
18184	*CBWD1*	-0.44353	-0.80234	-1.3017	-2.1709	-0.85
18185	*LEMD1*	-0.0146	-1.1898	-1.3434	-3.2405	-0.85
18186	*ZKSCAN5*	-0.07193	-0.67556	-1.8007	-2.0653	-0.85
18187	*GPR180*	0.037116	-1.2792	-1.3074	-1.4584	-0.85
18188	*TBX6*	-0.34962	-0.89219	-1.3085	-1.4773	-0.85
18189	*SPDL1*	-0.04612	-0.98842	-1.5167	-2.5302	-0.85
18190	*DALRD3*	-0.33787	-0.93789	-1.2756	-2.3154	-0.85
18191	*TNFAIP3*	-0.05175	-0.94393	-1.5568	-2.8797	-0.85
18192	*CFAP61*	-0.21821	-1.1075	-1.2273	-1.2577	-0.85
18193	*RPL11*	0.071812	-0.87955	-1.7459	-2.3949	-0.85
18194	*PAX7*	0.28506	-0.9512	-1.8885	-2.0028	-0.85
18195	*LRRC10B*	0.13231	-0.86526	-1.8233	-1.8435	-0.85
18196	*TTC37*	-0.04085	-1.0576	-1.4584	-2.6072	-0.85
18197	*B3GALNT2*	-0.55791	-0.56157	-1.4392	-3.1051	-0.85
18198	*GLIPR1L2*	-0.44498	-0.94747	-1.1665	-1.831	-0.85
18199	*SLC6A13*	-0.63214	-0.87444	-1.0528	-1.7158	-0.85
18200	*COPS8*	-0.38198	-0.86556	-1.3141	-1.6213	-0.85
18201	*EBPL*	-0.33848	-0.92077	-1.3036	-2.1566	-0.85
18202	*SLC7A9*	-0.44353	-0.51653	-1.6047	-2.3014	-0.85
18203	*ALG5*	-0.75161	-0.85386	-0.96012	-1.8363	-0.86
18204	*C2orf44*	-0.04457	-1.2014	-1.3203	-1.7967	-0.86
18205	*DNAJC7*	0.37062	-1.1439	-1.7963	-2.4456	-0.86
18206	*ZNF224*	0.18719	-0.21452	-2.5452	-2.8206	-0.86
18207	*DNAJC12*	-0.67599	-0.81068	-1.0889	-2.7626	-0.86
18208	*CYLD*	-0.27434	-0.89136	-1.4141	-1.6722	-0.86
18209	*NEURL2*	-0.30608	-0.68813	-1.5874	-1.9622	-0.86

18210	*C1QBP*	0.16793	-1.1138	-1.6365	-2.5677	-0.86
18211	*KIFC3*	-0.74392	-0.77583	-1.0633	-1.3633	-0.86
18212	*WIF1*	-0.2985	-0.9105	-1.3741	-1.7117	-0.86
18213	*SELT*	-0.33895	-0.95893	-1.2864	-2.4784	-0.86
18214	*C1orf27*	1.1403	-1.0385	-2.6889	-3.1761	-0.86
18215	*CFC1*	0.17256	-1.3364	-1.4233	-1.5125	-0.86
18216	*SLC25A1*	0.39669	-1.268	-1.716	-1.7606	-0.86
18217	*IMP3*	-0.14403	-1.1214	-1.322	-1.9162	-0.86
18218	*MEIKIN*	-0.62407	-0.7476	-1.2165	-3.3669	-0.86
18219	*ETS1*	-0.69287	-0.85296	-1.043	-1.244	-0.86
18220	*NR6A1*	0.60604	-1.2852	-1.9116	-2.9736	-0.86
18221	*BBX*	-0.15385	-1.0231	-1.4141	-2.0885	-0.86
18222	*PRICKLE4*	-0.02232	-0.89593	-1.6731	-1.9446	-0.86
18223	*COX4I1*	0.17372	-1.0871	-1.6787	-1.9326	-0.86
18224	*BTN2A1*	-0.36311	-0.98925	-1.2412	-1.4142	-0.86
18225	*CMTM4*	-0.0862	-0.49778	-2.0107	-2.1626	-0.86
18226	*C12orf79*	-0.01195	-1.2646	-1.3184	-2.0351	-0.86
18227	*MRPL17*	-0.51845	-0.6258	-1.451	-1.6306	-0.87
18228	*QRSL1*	-0.58058	-0.71363	-1.3017	-2.1921	-0.87
18229	*CITED4*	0.95013	-1.6731	-1.8742	-2.0483	-0.87
18230	*TMC4*	0.048059	-1.1613	-1.4842	-2.3885	-0.87
18231	*ZNF626*	-0.33601	-0.70581	-1.5568	-1.8737	-0.87
18232	*CNGA3*	0.36192	-1.2884	-1.6741	-2.8406	-0.87
18233	*EIF4H*	0.39018	-1.4467	-1.545	-1.7879	-0.87
18234	*RAB9B*	0.66434	-0.31333	-2.9538	-3.7266	-0.87
18235	*CRYBB1*	-0.23846	-0.95987	-1.4055	-1.7911	-0.87
18236	*ADGRA2*	0.29943	-1.1429	-1.7608	-2.0452	-0.87
18237	*DBF4*	-0.16325	-0.79821	-1.6447	-2.1347	-0.87
18238	*U2AF1*	0.12804	-1.1732	-1.5611	-1.6281	-0.87
18239	*ATMIN*	-0.03016	-1.2295	-1.348	-1.658	-0.87
18240	*PIK3R2*	0.069078	-0.87773	-1.8016	-2.1321	-0.87
18241	*ORC2*	-0.36086	-1.0035	-1.2466	-1.6476	-0.87
18242	*SERBP1*	-0.30286	-0.76355	-1.545	-2.4946	-0.87
18243	*STOML2*	-0.81031	-0.81282	-0.98867	-0.99463	-0.87
18244	*CDC42SE2*	-0.4589	-1.0291	-1.1263	-1.3457	-0.87
18245	*POC1A*	-0.03196	-0.93865	-1.6437	-1.8775	-0.87
18246	*TXN2*	-0.11653	-1.1303	-1.3675	-3.9779	-0.87
18247	*HMCES*	-0.53668	-0.9765	-1.1016	-2.7736	-0.87
18248	*JUNB*	-0.54479	-0.97113	-1.0997	-1.8537	-0.87
18249	*NTRK2*	1.2246	-1.8908	-1.9495	-2.4741	-0.87
18250	*LSM8*	0.17305	-1.2174	-1.5744	-1.739	-0.87
18251	*NDUFA4L2*	-0.26369	-0.70289	-1.6525	-2.8367	-0.87
18252	*SRPR*	0.39446	-1.4183	-1.5956	-1.7652	-0.87
18253	*PPAT*	-0.22414	-1.0119	-1.3842	-1.5988	-0.87
18254	*C9orf131*	-0.01678	-1.1802	-1.4234	-1.802	-0.87
18255	*CAMTA1*	0.026876	-1.2867	-1.3637	-1.72	-0.87

18256	*STX17*	-0.11695	-0.93489	-1.5727	-1.8157	-0.87
18257	*GNL3*	-0.43295	-0.88702	-1.3078	-1.7459	-0.88
18258	*BAZ2A*	0.15856	-0.47862	-2.3096	-2.382	-0.88
18259	*POP5*	-0.5193	-0.89501	-1.2167	-1.6128	-0.88
18260	*P2RX4*	0.26893	-1.2709	-1.632	-1.7978	-0.88
18261	*USP17L27*	-0.26592	-1.1479	-1.2202	-1.316	-0.88
18262	*BUB3*	-0.34506	-0.81152	-1.4777	-2.1212	-0.88
18263	*URB1*	-0.662	-0.84386	-1.13	-2.2385	-0.88
18264	*GTPBP8*	-0.7241	-0.93474	-0.97914	-1.9695	-0.88
18265	*WNT5A*	0.27588	-0.73517	-2.1796	-3.6038	-0.88
18266	*MSL3*	0.17562	-1.2246	-1.5915	-1.9295	-0.88
18267	*TERT*	-0.48021	-0.83276	-1.3279	-1.5832	-0.88
18268	*NOL11*	0.10312	-0.94536	-1.7991	-1.8714	-0.88
18269	*CRHBP*	-0.04717	-1.1974	-1.397	-2.262	-0.88
18270	*ISY1*	-0.67432	-0.76821	-1.1992	-2.0429	-0.88
18271	*IGFN1*	-0.07337	-1.2208	-1.3478	-1.7538	-0.88
18272	*MGC50722*	-0.44634	-0.97393	-1.2217	-2.5414	-0.88
18273	*BCORL1*	-0.2873	-0.93368	-1.4221	-1.5806	-0.88
18274	*THBD*	0.24025	-1.3903	-1.4944	-1.7649	-0.88
18275	*ESF1*	-0.62099	-0.93237	-1.0912	-2.1125	-0.88
18276	*CHMP4B*	0.054282	-1.0666	-1.6336	-1.8441	-0.88
18277	*TFB1M*	-0.46228	-0.75625	-1.4276	-1.9172	-0.88
18278	*TULP1*	0.24612	-1.4325	-1.462	-2.6003	-0.88
18279	*ARFIP1*	0.42399	-1.156	-1.9166	-2.0483	-0.88
18280	*CFB*	-0.3943	-0.83739	-1.4171	-1.6663	-0.88
18281	*KDM2A*	-0.49241	-0.91396	-1.2457	-2.005	-0.88
18282	*ZNF879*	-0.17622	-1.0384	-1.4392	-1.7149	-0.88
18283	*FBXW8*	-0.49598	-0.72515	-1.4333	-1.5901	-0.88
18284	*IL23A*	0.49003	-1.3246	-1.8212	-2.9818	-0.89
18285	*EME1*	0.32411	-1.1558	-1.8297	-2.0016	-0.89
18286	*PALB2*	0.06576	-1.3211	-1.4064	-1.42	-0.89
18287	*C19orf84*	-0.65714	-0.88267	-1.122	-1.1343	-0.89
18288	*SMIM18*	0.20116	-1.3926	-1.4704	-2.8463	-0.89
18289	*CD8B*	0.16025	-1.3288	-1.496	-2.0548	-0.89
18290	*CHRM5*	-0.7001	-0.85799	-1.1075	-1.1263	-0.89
18291	*SUPV3L1*	-0.36821	-0.78362	-1.5139	-1.6422	-0.89
18292	*FOXI3*	-0.38406	-0.93865	-1.3457	-2.0116	-0.89
18293	*HECTD1*	-0.41139	-0.81496	-1.445	-1.6241	-0.89
18294	*SERINC4*	-0.74308	-0.8737	-1.0559	-1.418	-0.89
18295	*ZNF287*	-0.07439	-1.2461	-1.3525	-1.743	-0.89
18296	*TMEM8A*	0.38037	-1.4584	-1.5959	-1.8402	-0.89
18297	*RALGPS2*	-0.35111	-0.76582	-1.5579	-1.9398	-0.89
18298	*DNTTIP2*	-0.55002	-0.69397	-1.4341	-1.8316	-0.89
18299	*IL21*	-0.36761	-0.97595	-1.3356	-3.596	-0.89
18300	*TRUB2*	-0.20512	-0.68727	-1.7884	-5.6002	-0.89
18301	*CNTNAP3*	-0.08477	-1.2676	-1.334	-1.4996	-0.90

18302	*CALB2*	0.69516	-1.6456	-1.7364	-1.984	-0.90
18303	*SNRPG*	-0.19995	-0.83669	-1.6526	-1.8577	-0.90
18304	*RNFT1*	-0.04009	-0.83145	-1.8193	-2.2323	-0.90
18305	*STRAP*	-0.15439	-0.80341	-1.7364	-2.4268	-0.90
18306	*GOLGA6L6*	0.16906	-1.3721	-1.4926	-1.6711	-0.90
18307	*RPL17*	-0.2098	-1.2033	-1.2829	-1.9326	-0.90
18308	*PTPRF*	-0.29549	-0.92464	-1.4761	-1.7735	-0.90
18309	*LRRC20*	-0.76606	-0.91701	-1.0188	-1.2302	-0.90
18310	*RPS23*	-0.28009	-0.88143	-1.5408	-1.8755	-0.90
18311	*WDR4*	-0.1138	-0.77394	-1.8154	-2.4221	-0.90
18312	*POLR3H*	-0.11044	-1.2729	-1.3198	-1.5592	-0.90
18313	*TPGS2*	-0.25181	-0.72553	-1.7263	-2.8928	-0.90
18314	*RBM23*	0.33274	-1.2876	-1.7493	-2.8553	-0.90
18315	*RSPH4A*	0.53276	-0.81034	-2.428	-2.5865	-0.90
18316	*LIPT2*	0.30909	-1.1943	-1.8219	-1.9727	-0.90
18317	*TGM2*	-0.08601	-1.0828	-1.5405	-1.6644	-0.90
18318	*RNF151*	-0.04432	-0.73172	-1.9358	-1.9965	-0.90
18319	*EIF4G1*	-0.21905	-0.93727	-1.5558	-2.1478	-0.90
18320	*VPS8*	-0.50676	-0.68133	-1.5243	-1.5401	-0.90
18321	*BRAP*	0.49383	-1.0102	-2.1984	-2.2586	-0.90
18322	*MED28*	0.5736	-1.5824	-1.7065	-3.1119	-0.91
18323	*C16orf59*	-0.12189	-0.7328	-1.8629	-2.5764	-0.91
18324	*PRMT2*	1.2299	-1.6891	-2.2594	-2.7813	-0.91
18325	*COPG1*	-0.46628	-0.81904	-1.4353	-1.5064	-0.91
18326	*ARHGEF38*	0.965	-1.843	-1.8435	-1.9131	-0.91
18327	*SLC6A20*	-0.56938	-0.77255	-1.3808	-1.4376	-0.91
18328	*UNC45B*	0.3523	-1.3617	-1.714	-3.2519	-0.91
18329	*TNPO3*	-0.63946	-0.97338	-1.1108	-4.2256	-0.91
18330	*DAB2IP*	-0.86494	-0.89438	-0.96572	-1.8046	-0.91
18331	*MDGA2*	-0.00178	-1.2694	-1.4544	-1.6649	-0.91
18332	*ATP5SL*	-0.46153	-0.88465	-1.3851	-1.7462	-0.91
18333	*LYSMD4*	-0.48756	-0.8897	-1.3552	-1.8157	-0.91
18334	*PROX1*	-0.56564	-0.75073	-1.4162	-1.9186	-0.91
18335	*OR10H1*	-0.6636	-0.97381	-1.0952	-2.011	-0.91
18336	*GPN2*	-0.55047	-0.86358	-1.3198	-2.5513	-0.91
18337	*MRPL49*	-0.61249	-0.76344	-1.358	-1.7743	-0.91
18338	*ARF1*	0.13438	-1.2884	-1.5801	-1.7578	-0.91
18339	*SLC2A9*	-0.45867	-0.49403	-1.7822	-1.8707	-0.91
18340	*ZBTB14*	-0.38296	-1.1223	-1.2302	-1.9175	-0.91
18341	*ACER3*	-0.5183	-0.95105	-1.2666	-2.2732	-0.91
18342	*FIGNL1*	-0.33136	-1.0297	-1.3771	-1.8435	-0.91
18343	*NBL1*	-0.56442	-0.84042	-1.3393	-1.7209	-0.91
18344	*RILPL1*	-0.51713	-0.64496	-1.5824	-2.2363	-0.91
18345	*MLN*	-0.08719	-0.97333	-1.6934	-2.0134	-0.92
18346	*MAPKAPK3*	0.096888	-1.2935	-1.5598	-2.0545	-0.92
18347	*DNAJB1*	-0.06253	-1.2202	-1.4741	-1.6128	-0.92

18348	*IYD*	-0.17606	-1.1925	-1.3912	-1.9172	-0.92
18349	*TIMM13*	-0.43626	-0.78313	-1.5408	-1.6608	-0.92
18350	*ARHGAP1*	-0.55625	-0.57669	-1.6281	-2.7303	-0.92
18351	*EXOSC4*	-0.61057	-0.74715	-1.4053	-3.8502	-0.92
18352	*EIF2A*	0.66522	-1.1016	-2.3282	-2.6208	-0.92
18353	*ZNF20*	0.53073	-1.278	-2.0181	-2.7678	-0.92
18354	*EXOC1*	-0.30438	-0.9225	-1.539	-1.7882	-0.92
18355	*HSD17B2*	-0.29291	-1.1566	-1.3183	-1.6164	-0.92
18356	*CA1*	0.1059	-0.98366	-1.8963	-1.9811	-0.92
18357	*CSGALNACT2*	-0.77018	-0.85739	-1.1487	-1.9338	-0.93
18358	*NDUFAF5*	-0.79813	-0.80405	-1.1742	-1.2114	-0.93
18359	*GGACT*	0.3775	-1.2567	-1.8975	-1.9107	-0.93
18360	*WDR49*	-0.5911	-0.60508	-1.5806	-2.1574	-0.93
18361	*GPRC5A*	-0.51549	-0.66623	-1.5959	-1.6216	-0.93
18362	*OR1A2*	-0.1059	-0.84345	-1.8321	-2.217	-0.93
18363	*RNF115*	-0.13576	-1.2864	-1.3643	-1.438	-0.93
18364	*SDAD1*	-0.5206	-0.54008	-1.7263	-1.9965	-0.93
18365	*FAM84A*	0.60761	-1.6885	-1.707	-2.2064	-0.93
18366	*ATP6V1G1*	0.057611	-0.4847	-2.3623	-3.4804	-0.93
18367	*RALGDS*	-0.04218	-1.2985	-1.4493	-1.9936	-0.93
18368	*DLL3*	0.37254	-1.4287	-1.7364	-2.2432	-0.93
18369	*HINFP*	-0.06631	-1.2418	-1.4878	-1.9965	-0.93
18370	*ADAMDEC1*	0.22798	-1.2884	-1.7364	-2.3197	-0.93
18371	*TBP*	-0.22428	-1.223	-1.3496	-2.3389	-0.93
18372	*C8orf88*	0.53251	-1.386	-1.9435	-3.1836	-0.93
18373	*MYO1A*	0.52375	-1.5817	-1.7397	-1.8557	-0.93
18374	*ZNF830*	-0.13185	-1.305	-1.3627	-1.4686	-0.93
18375	*LAGE3*	0.11473	-1.4325	-1.4824	-1.7538	-0.93
18376	*HMGB1*	0.12155	-1.4568	-1.468	-1.6313	-0.93
18377	*PDXDC1*	-0.52574	-0.88971	-1.3912	-1.5436	-0.94
18378	*RARG*	-0.32355	-1.154	-1.3296	-1.7726	-0.94
18379	*RUFY3*	-0.49565	-0.87931	-1.4325	-1.8272	-0.94
18380	*VDAC3*	0.43872	-1.2687	-1.9789	-3.4532	-0.94
18381	*CD300C*	-0.33705	-1.0918	-1.3818	-1.7322	-0.94
18382	*SNX27*	-0.16892	-1.1002	-1.5425	-1.6014	-0.94
18383	*GAL*	0.27373	-1.3701	-1.716	-2.0953	-0.94
18384	*HLA-DRA*	-0.71016	-0.75437	-1.3506	-1.7745	-0.94
18385	*RGS9BP*	-0.49045	-1.1461	-1.1793	-2.4647	-0.94
18386	*CDADC1*	-0.59119	-0.59856	-1.6276	-2.0548	-0.94
18387	*DUSP6*	0.33475	-1.3465	-1.8066	-2.5725	-0.94
18388	*KCNC4*	-0.38053	-1.1856	-1.2546	-1.4684	-0.94
18389	*SEPSECS*	-0.17058	-0.23846	-2.412	-2.4933	-0.94
18390	*ELOVL1*	0.92769	-1.7554	-1.9936	-2.0949	-0.94
18391	*SPSB1*	-0.02722	-0.99768	-1.7974	-1.8462	-0.94
18392	*MUC5AC*	-0.29905	-1.0443	-1.4805	-1.9196	-0.94
18393	*YAE1D1*	-0.3316	-0.75038	-1.742	-2.1612	-0.94

18394	*CLVS2*	-0.73565	-1.0386	-1.0529	-2.092	-0.94
18395	*GCN1L1*	-0.17285	-1.0467	-1.6092	-2.8963	-0.94
18396	*C7orf61*	-0.76516	-0.98907	-1.0797	-2.2285	-0.94
18397	*PLEKHM2*	-0.78098	-0.82375	-1.2297	-1.4034	-0.94
18398	*GLB1L2*	-0.43818	-1.0564	-1.3399	-1.8076	-0.94
18399	*HOXA7*	-0.69184	-0.84542	-1.3007	-2.555	-0.95
18400	*ATP6V0D1*	-0.44353	-0.80969	-1.5852	-1.8604	-0.95
18401	*EPHB3*	-0.38901	-0.57824	-1.8714	-2.4078	-0.95
18402	*TMEM97*	0.33639	-1.4234	-1.7526	-2.1794	-0.95
18403	*FER1L5*	0.034036	-1.2676	-1.6065	-2.1426	-0.95
18404	*PLS1*	0.004468	-0.90206	-1.9474	-2.0223	-0.95
18405	*DAD1*	-0.48085	-0.76882	-1.5962	-2.1239	-0.95
18406	*COX15*	-0.72814	-1.0564	-1.0623	-1.4142	-0.95
18407	*NUF2*	-0.39636	-1.1212	-1.3295	-1.5082	-0.95
18408	*RPS6KC1*	-0.63357	-0.68303	-1.5306	-2.0256	-0.95
18409	*NUBP1*	0.30938	-1.5408	-1.6164	-2.3846	-0.95
18410	*SRGAP3*	-0.18614	-1.2198	-1.4423	-1.7577	-0.95
18411	*TECTB*	-0.79319	-0.88503	-1.1752	-1.3078	-0.95
18412	*ARIH2OS*	0.14155	-0.33705	-2.6609	-3.2005	-0.95
18413	*GMPS*	0.068131	-1.4467	-1.4789	-2.1046	-0.95
18414	*KLHL38*	0.258	-1.4536	-1.6635	-2.5571	-0.95
18415	*PRDM11*	0.16442	-1.0248	-2.0012	-2.4642	-0.95
18416	*AQP1*	-0.21701	-1.2855	-1.3591	-2.1444	-0.95
18417	*EIF3D*	-0.19422	-1.3332	-1.3349	-1.5307	-0.95
18418	*PSMD6*	0.41894	-1.2214	-2.0601	-2.2139	-0.95
18419	*EIF2AK4*	-0.75489	-0.98468	-1.1272	-1.3823	-0.96
18420	*AP2S1*	-0.79367	-0.87989	-1.1973	-1.5589	-0.96
18421	*C9orf156*	-0.32871	-1.1524	-1.3923	-1.4782	-0.96
18422	*NUDC*	0.82411	-1.3416	-2.3577	-5.7035	-0.96
18423	*SYNGR3*	-0.39017	-1.175	-1.3104	-2.412	-0.96
18424	*TIGD7*	-0.29327	-0.99834	-1.5861	-1.9302	-0.96
18425	*TRIM47*	-0.00379	-1.3915	-1.4859	-2.268	-0.96
18426	*DSPP*	-0.46089	-0.79187	-1.6288	-2.0059	-0.96
18427	*HSPA6*	-0.22131	-1.2936	-1.3668	-1.8816	-0.96
18428	*EME2*	-0.48659	-1.1845	-1.2114	-3.7236	-0.96
18429	*MYLK2*	-0.86223	-0.9857	-1.0354	-1.047	-0.96
18430	*ITGAL*	0.10699	-1.011	-1.9794	-3.3443	-0.96
18431	*RALY*	-0.15766	-1.2876	-1.4388	-3.596	-0.96
18432	*KCNC1*	0.22531	-1.53	-1.5795	-1.8219	-0.96
18433	*AKR1B1*	0.2678	-1.3743	-1.7806	-2.4244	-0.96
18434	*CTAGE4*	-0.76771	-0.76832	-1.3513	-1.6365	-0.96
18435	*TMEM184B*	0.31536	-1.1218	-2.0818	-2.312	-0.96
18436	*RSRC2*	-0.1496	-1.3132	-1.4287	-1.5289	-0.96
18437	*MRPS16*	-0.41965	-0.90003	-1.5737	-1.5792	-0.96
18438	*DMAP1*	-0.0394	-1.2481	-1.6066	-1.9768	-0.96
18439	*VAMP8*	-0.39386	-1.0589	-1.4415	-2.0717	-0.96

18440	*FSIP2*	-0.59025	-1.0232	-1.2837	-1.6586	-0.97
18441	*SERINC1*	-0.41087	-0.72227	-1.7652	-1.7662	-0.97
18442	*EIF4A3*	0.52363	-1.451	-1.9734	-2.4873	-0.97
18443	*HPSE*	-0.74011	-1.0097	-1.151	-1.3132	-0.97
18444	*UBE2C*	-0.50748	-1.1448	-1.25	-2.0267	-0.97
18445	*TAF4B*	-0.43572	-1.0796	-1.3878	-1.7179	-0.97
18446	*RLN3*	-0.18185	-0.92235	-1.7999	-2.1239	-0.97
18447	*SMEK1*	-0.02584	-1.3496	-1.5304	-1.5949	-0.97
18448	*AQR*	-0.19225	-0.98842	-1.7268	-2.3509	-0.97
18449	*LTBP1*	-0.00827	-1.3668	-1.5357	-1.6587	-0.97
18450	*FRAT2*	-0.33363	-1.0585	-1.5195	-1.545	-0.97
18451	*CCDC28B*	-0.44986	-1.2293	-1.2347	-1.7718	-0.97
18452	*PMEL*	-0.59225	-0.91823	-1.4036	-2.1205	-0.97
18453	*TRIM64B*	-0.63328	-1.0641	-1.2169	-1.7679	-0.97
18454	*MARK3*	-0.46716	-1.116	-1.3316	-2.2856	-0.97
18455	*ASPRV1*	-0.51161	-0.8191	-1.5854	-1.6826	-0.97
18456	*INTS10*	-0.23203	-0.68727	-2.0016	-2.508	-0.97
18457	*SSX5*	0.6844	-1.151	-2.4595	-2.8463	-0.98
18458	*PROK1*	-0.25407	-1.2489	-1.4234	-2.0817	-0.98
18459	*TSR2*	0.45815	-1.1676	-2.2171	-2.4562	-0.98
18460	*NOLC1*	0.11095	-1.338	-1.701	-2.3573	-0.98
18461	*CRH*	-0.59008	-0.99644	-1.3429	-1.7753	-0.98
18462	*ZNF267*	0.52847	-1.6524	-1.8076	-1.8508	-0.98
18463	*SETD1A*	-0.52876	-1.1357	-1.2694	-2.4422	-0.98
18464	*NACA*	-0.27263	-1.0889	-1.5727	-2.2732	-0.98
18465	*EIF4ENIF1*	-0.03537	-1.392	-1.5101	-1.6368	-0.98
18466	*CMPK1*	-0.25941	-0.8638	-1.8157	-1.9686	-0.98
18467	*SLC6A11*	-0.16249	-0.46731	-2.3145	-2.6024	-0.98
18468	*TJP2*	-0.2507	-0.78313	-1.9107	-2.1984	-0.98
18469	*CEP89*	-0.24598	-1.3053	-1.3946	-1.6356	-0.98
18470	*C2orf16*	-0.23528	-0.80946	-1.9068	-2.672	-0.98
18471	*USP20*	-0.73061	-0.97149	-1.2497	-1.7804	-0.98
18472	*PNO1*	-0.09774	-1.2761	-1.5782	-1.6867	-0.98
18473	*LOC388436*	-0.28357	-1.1754	-1.4935	-1.8725	-0.98
18474	*ORM1*	-0.05212	-1.3542	-1.5467	-2.7502	-0.98
18475	*GADD45G*	-0.3644	-1.2127	-1.3771	-1.7235	-0.98
18476	*COX11*	-0.35805	-0.93806	-1.6594	-1.9556	-0.99
18477	*GPR45*	-0.55477	-0.98129	-1.4206	-1.6984	-0.99
18478	*KIR3DL2*	0.056445	-1.2867	-1.7266	-1.962	-0.99
18479	*ZNF574*	-0.72798	-1.0162	-1.2135	-2.1919	-0.99
18480	*CRLF2*	-0.71669	-1.1095	-1.1415	-1.3711	-0.99
18481	*CBLN4*	-0.33705	-1.2646	-1.3675	-2.2456	-0.99
18482	*NT5C3A*	-0.21148	-1.2195	-1.5383	-1.8952	-0.99
18483	*KLHDC4*	-0.32719	-0.93112	-1.7115	-2.4741	-0.99
18484	*INSIG2*	-0.48842	-0.98668	-1.4962	-2.1038	-0.99
18485	*NAPG*	1.0414	-1.988	-2.0249	-2.2669	-0.99

18486	*SPATA5L1*	-0.83912	-1.0131	-1.1212	-2.101	-0.99
18487	*FAM180A*	0.34989	-1.6047	-1.7247	-2.0042	-0.99
18488	*CDH7*	-0.69865	-0.9442	-1.3371	-2.337	-0.99
18489	*GINS3*	-0.6029	-1.0359	-1.3457	-2.0383	-0.99
18490	*DPP8*	0.79651	-1.855	-1.9269	-2.5153	-1.00
18491	*GFER*	-0.35972	-0.75665	-1.8692	-2.0436	-1.00
18492	*IL24*	-0.84446	-1.0231	-1.1189	-1.1685	-1.00
18493	*PCGF3*	-0.86494	-0.92425	-1.2009	-1.3353	-1.00
18494	*NDUFB9*	-0.40125	-1.1432	-1.4485	-2.1796	-1.00
18495	*TUBGCP5*	-0.59229	-0.89866	-1.502	-1.7643	-1.00
18496	*LRRC18*	-0.30496	-1.2719	-1.4168	-2.0851	-1.00
18497	*TP53INP2*	-0.6736	-0.8737	-1.45	-1.7164	-1.00
18498	*TFRC*	-0.68208	-0.96776	-1.3489	-1.7179	-1.00
18499	*MAMDC4*	-0.44353	-0.96779	-1.5876	-1.6339	-1.00
18500	*POLR2A*	-0.43634	-1.25	-1.3128	-2.6188	-1.00
18501	*TMEM132A*	-0.73051	-0.97768	-1.2968	-1.7074	-1.00
18502	*RPA3*	-0.29623	-0.97381	-1.7365	-2.1859	-1.00
18503	*SMYD2*	-0.85931	-0.90549	-1.2471	-1.6921	-1.00
18504	*SNRPD3*	-0.59886	-0.7503	-1.6635	-2.4015	-1.00
18505	*ZNRD1*	-0.76055	-0.99418	-1.2588	-1.8695	-1.00
18506	*RPL4*	-0.21123	-1.2343	-1.5684	-2.5846	-1.00
18507	*ZNF460*	-0.63341	-0.811	-1.5704	-1.8256	-1.00
18508	*SMG7*	-0.33169	-1.3057	-1.3785	-2.005	-1.01
18509	*ZNF711*	0.54647	-1.7285	-1.8341	-2.4785	-1.01
18510	*CDC45*	-0.73673	-0.97636	-1.3033	-1.4523	-1.01
18511	*SPTBN5*	-0.62613	-1.071	-1.3209	-2.2165	-1.01
18512	*TH*	-0.1042	-1.083	-1.8318	-2.2948	-1.01
18513	*FNDC5*	-0.49525	-1.13	-1.3946	-1.7658	-1.01
18514	*C7orf55*	0.26733	-1.595	-1.6926	-2.3755	-1.01
18515	*HNRNPU*	-0.43951	-1.1343	-1.4473	-1.7512	-1.01
18516	*CCDC101*	0.42584	-1.4355	-2.0123	-2.2428	-1.01
18517	*RREB1*	-0.7006	-0.79225	-1.5304	-1.8338	-1.01
18518	*RPUSD1*	0.38492	-1.507	-1.9013	-1.9341	-1.01
18519	*GMPPB*	0.043314	-1.2676	-1.8008	-2.3086	-1.01
18520	*OTOR*	-0.42401	-1.2567	-1.345	-1.4537	-1.01
18521	*PRPS2*	-0.83818	-1.0604	-1.1278	-1.8938	-1.01
18522	*YARS2*	-0.44084	-1.1978	-1.3887	-1.5831	-1.01
18523	*C8orf44*	0.25292	-1.1852	-2.0953	-2.6973	-1.01
18524	*ZP4*	-0.97393	-0.99791	-1.0565	-1.5663	-1.01
18525	*ZNF559-ZNF177*	-0.16151	-1.3457	-1.5235	-2.8327	-1.01
18526	*CCT5*	0.48172	-1.2676	-2.2449	-3.1273	-1.01
18527	*SZRD1*	-0.3785	-1.1718	-1.4811	-1.5817	-1.01
18528	*SLC18B1*	-0.20924	-0.83439	-1.988	-2.2528	-1.01
18529	*ANGPTL2*	-0.14166	-1.3148	-1.5781	-1.912	-1.01
18530	*CNOT2*	-0.57825	-1.153	-1.3068	-1.6601	-1.01
18531	*EPB41L5*	-0.33241	-1.1518	-1.5544	-1.8975	-1.01

18532	*GP2*	-0.6728	-1.1289	-1.237	-1.7149	-1.01
18533	*MROH8*	-0.26439	-1.0409	-1.7338	-2.6046	-1.01
18534	*LSM2*	-0.86358	-1.0199	-1.1564	-1.5183	-1.01
18535	*IGBP1*	0.47763	-1.632	-1.8869	-2.6779	-1.01
18536	*TRIM66*	-0.39368	-1.099	-1.5491	-1.6876	-1.01
18537	*CD80*	-0.45672	-0.79225	-1.7942	-2.2888	-1.01
18538	*TRAF2*	-0.56251	-0.78961	-1.6921	-1.8425	-1.01
18539	*TMC5*	-0.13189	-1.3343	-1.584	-2.9909	-1.02
18540	*ARID4A*	-0.18925	-1.0718	-1.7945	-2.0351	-1.02
18541	*RALGAPB*	0.06603	-1.426	-1.6964	-2.8166	-1.02
18542	*COG8*	-0.28487	-1.1739	-1.5978	-2.312	-1.02
18543	*PLCB1*	-0.04824	-1.3023	-1.7134	-2.1807	-1.02
18544	*PTRH1*	-0.50724	-0.97346	-1.5884	-2.5125	-1.02
18545	*TMCO3*	0.47329	-1.1839	-2.3585	-3.3677	-1.02
18546	*H3F3A*	-0.44353	-0.68934	-1.9375	-2.6157	-1.02
18547	*CUL4A*	-0.61358	-1.1752	-1.2867	-1.828	-1.03
18548	*MTERF2*	0.12888	-1.498	-1.7119	-2.4709	-1.03
18549	*TONSL*	0.046846	-1.4233	-1.7052	-2.8051	-1.03
18550	*NSL1*	-0.09437	-1.4123	-1.5765	-1.7349	-1.03
18551	*PTPRC*	0.40329	-1.4692	-2.0197	-2.3063	-1.03
18552	*NPPC*	-0.76055	-1.0198	-1.3053	-1.4775	-1.03
18553	*MAGED4B*	-0.2747	-0.588	-2.224	-2.3178	-1.03
18554	*ZNF671*	-0.4191	-1.0509	-1.6193	-3.2688	-1.03
18555	*HDAC2*	-0.10661	-1.4287	-1.5588	-2.5006	-1.03
18556	*RPS25*	0.37299	-1.3617	-2.1066	-2.1919	-1.03
18557	*NR2E3*	-0.30692	-1.3128	-1.4775	-2.0895	-1.03
18558	*MYOZ3*	-0.72782	-1.073	-1.3039	-1.6105	-1.03
18559	*CAPS*	-0.56926	-1.1585	-1.3795	-1.4385	-1.04
18560	*FOXA1*	0.1506	-1.6098	-1.6481	-1.72	-1.04
18561	*BOLA2*	0.09805	-1.1354	-2.071	-2.1239	-1.04
18562	*ZNF880*	0.060365	-1.1958	-1.9748	-2.0661	-1.04
18563	*ETV4*	-0.03506	-1.2118	-1.8683	-2.0632	-1.04
18564	*TRIM11*	-0.33191	-1.0256	-1.7577	-3.582	-1.04
18565	*DLGAP1*	-0.0994	-1.1214	-1.8946	-2.224	-1.04
18566	*DPF3*	-0.55513	-1.2572	-1.3033	-1.6085	-1.04
18567	*UGT3A1*	-0.78591	-0.90865	-1.4218	-1.5304	-1.04
18568	*OSBPL8*	-0.73823	-0.93878	-1.4416	-3.1728	-1.04
18569	*PABPN1*	-0.40015	-0.9532	-1.7658	-3.2023	-1.04
18570	*PDYN*	-0.44353	-1.1492	-1.5314	-1.6157	-1.04
18571	*DPM3*	0.18317	-1.4364	-1.8714	-2.2165	-1.04
18572	*CCDC59*	-0.22803	-1.2192	-1.6799	-1.7585	-1.04
18573	*MRPL20*	-0.42671	-0.87673	-1.8238	-1.8611	-1.04
18574	*CCDC74A*	-0.74086	-0.8489	-1.5393	-1.9844	-1.04
18575	*FAM231A*	-0.20775	-1.3039	-1.6182	-1.8566	-1.04
18576	*BCDIN3D*	0.73895	-1.6709	-2.2014	-4.4273	-1.04
18577	*PDGFB*	-0.6579	-0.93217	-1.545	-2.1802	-1.05

18578	*TNFSF8*	-0.50676	-1.2911	-1.3393	-1.5687	-1.05
18579	*TREM1*	-0.06833	-1.505	-1.5707	-2.5677	-1.05
18580	*ASAP3*	-0.92427	-1.0912	-1.1287	-1.7072	-1.05
18581	*GNL3L*	-0.73199	-0.89127	-1.5234	-1.7638	-1.05
18582	*HCK*	-0.33754	-1.1207	-1.6891	-1.7595	-1.05
18583	*MST1L*	-0.31011	-0.93172	-1.9073	-3.0598	-1.05
18584	*SLC6A9*	-0.01901	-1.4597	-1.6709	-2.0218	-1.05
18585	*SPDYA*	-0.00431	-1.2756	-1.8761	-2.2607	-1.05
18586	*OR6X1*	-0.59572	-1.0273	-1.5375	-1.7177	-1.05
18587	*PDCD7*	0.066478	-0.94965	-2.2804	-3.1051	-1.05
18588	*DRAP1*	-0.11265	-1.3543	-1.6968	-2.005	-1.05
18589	*KRT19*	-0.18808	-1.348	-1.6288	-1.9165	-1.05
18590	*PRPF18*	0.086847	-1.589	-1.6644	-2.0297	-1.06
18591	*ILKAP*	-0.70417	-1.2281	-1.2347	-1.301	-1.06
18592	*CD300LG*	-0.74086	-0.94052	-1.4856	-1.5121	-1.06
18593	*S1PR4*	-0.76371	-1.1904	-1.2156	-2.817	-1.06
18594	*UTP11L*	-0.7614	-1.1958	-1.2145	-1.5696	-1.06
18595	*MBLAC1*	1.2549	-1.3662	-3.0614	-3.182	-1.06
18596	*KCNN4*	-0.747	-1.1495	-1.2779	-1.4473	-1.06
18597	*CDK5*	0.5592	-0.94759	-2.7871	-2.9236	-1.06
18598	*NDFIP1*	-0.75889	-1.1739	-1.2431	-1.5874	-1.06
18599	*NIP7*	-0.76342	-1.1995	-1.2141	-2.5007	-1.06
18600	*C19orf40*	-0.81319	-1.1466	-1.2175	-1.8489	-1.06
18601	*WRB*	-0.57776	-1.0158	-1.5848	-2.0476	-1.06
18602	*PREX1*	-0.60692	-1.238	-1.3361	-1.359	-1.06
18603	*ABHD8*	0.070816	-1.371	-1.8816	-2.2264	-1.06
18604	*EIF2AK1*	-0.54688	-0.95045	-1.6877	-2.1282	-1.06
18605	*HNRNPA1*	-0.60929	-1.1297	-1.4467	-1.6255	-1.06
18606	*VCP*	-0.48654	-1.0671	-1.6323	-2.1388	-1.06
18607	*WDR6*	-0.6536	-1.1553	-1.3785	-1.3903	-1.06
18608	*RPL26L1*	-0.95893	-1.1095	-1.1214	-1.9446	-1.06
18609	*RFC5*	-0.77789	-1.0995	-1.3198	-2.8823	-1.07
18610	*CCDC88A*	1.0186	-1.6866	-2.5292	-2.7465	-1.07
18611	*CCT6A*	-0.11811	-1.3686	-1.7149	-2.072	-1.07
18612	*LOC102724862*	0.45098	-1.4504	-2.2044	-2.3287	-1.07
18613	*HS3ST3B1*	0.54628	-1.7884	-1.9622	-2.6803	-1.07
18614	*SH3BGRL2*	0.26813	-1.1637	-2.312	-2.4312	-1.07
18615	*USP17L25*	-0.47447	-1.3637	-1.3743	-1.398	-1.07
18616	*RPL24*	-0.10034	-1.2486	-1.8645	-2.3729	-1.07
18617	*IFT57*	-0.6141	-0.67691	-1.9227	-2.046	-1.07
18618	*DLD*	-1.0039	-1.0826	-1.1325	-2.1798	-1.07
18619	*NXPH3*	-0.13602	-1.4998	-1.5848	-2.3062	-1.07
18620	*GDF5*	-0.78199	-1.1027	-1.3364	-1.545	-1.07
18621	*LRRC69*	-0.44136	-0.90523	-1.8761	-2.0218	-1.07
18622	*WBP5*	0.25931	-1.6617	-1.8212	-2.1984	-1.07
18623	*NCR3LG1*	-0.35478	-1.3224	-1.547	-1.5848	-1.07

18624	*POC1B-GALNT4*	-0.48905	-0.49016	-2.2456	-2.5551	-1.07
18625	*FAM217A*	0.48077	-1.6316	-2.0752	-2.5008	-1.08
18626	*RTN4R*	-0.83436	-1.082	-1.3104	-1.4273	-1.08
18627	*PDE6B*	-0.66862	-1.0029	-1.5568	-1.8256	-1.08
18628	*RPL7A*	0.44691	-1.7412	-1.9358	-2.1198	-1.08
18629	*RERE*	0.67156	-1.9186	-1.984	-2.3534	-1.08
18630	*CHIC2*	0.046036	-1.5039	-1.7745	-2.3461	-1.08
18631	*XYLT1*	0.30066	-1.4688	-2.0653	-2.5975	-1.08
18632	*CLDN9*	-0.06441	-0.47818	-2.6923	-2.8976	-1.08
18633	*NCOR1*	-0.67702	-0.98585	-1.5744	-2.1843	-1.08
18634	*GALK2*	-0.6799	-1.1979	-1.3684	-2.1605	-1.08
18635	*RNF8*	-0.6133	-1.0671	-1.5671	-1.7595	-1.08
18636	*PSCA*	-0.57588	-0.77038	-1.9013	-2.0545	-1.08
18637	*SCYL1*	-0.73695	-1.0044	-1.5069	-1.9794	-1.08
18638	*ZFC3H1*	-0.61946	-1.2666	-1.3627	-1.5859	-1.08
18639	*OR2L2*	0.19764	-1.6763	-1.7709	-2.3696	-1.08
18640	*TP53I11*	-0.51319	-0.7436	-1.9938	-2.0817	-1.08
18641	*DCDC1*	-0.57484	-1.3282	-1.3523	-1.3666	-1.09
18642	*GTF2F1*	-0.73602	-0.89741	-1.6239	-1.8575	-1.09
18643	*APOH*	0.34764	-1.7561	-1.8557	-2.2473	-1.09
18644	*SPATA32*	-0.70057	-1.1312	-1.434	-2.1489	-1.09
18645	*ALG13*	-0.67801	-1.2814	-1.3098	-1.7512	-1.09
18646	*TAGLN2*	-0.33213	-0.97549	-1.9624	-6.2107	-1.09
18647	*MSMP*	-0.53234	-1.2935	-1.448	-1.4507	-1.09
18648	*EXOSC9*	-0.76906	-1.1028	-1.4087	-2.274	-1.09
18649	*ATR*	0.67486	-1.6526	-2.3036	-2.3871	-1.09
18650	*TBL2*	-0.94991	-1.0974	-1.2343	-1.31	-1.09
18651	*CHMP6*	-0.88281	-0.91944	-1.4801	-2.4193	-1.09
18652	*ANAPC15*	-0.97088	-1.0758	-1.2402	-1.7905	-1.10
18653	*FAM49B*	-0.44353	-0.99357	-1.85	-2.0197	-1.10
18654	*ABCA4*	-0.25893	-0.55029	-2.4784	-2.5298	-1.10
18655	*EEF1A1*	-0.54734	-1.2514	-1.4893	-1.5954	-1.10
18656	*GCNT4*	-0.56525	-1.1126	-1.6105	-2.6062	-1.10
18657	*PYHIN1*	-0.44353	-1.2277	-1.6176	-1.6268	-1.10
18658	*THOC3*	-0.24253	-1.41	-1.6371	-1.7386	-1.10
18659	*GIMAP8*	-0.83773	-0.95842	-1.4935	-2.0181	-1.10
18660	*HIST1H2AL*	0.23572	-1.4233	-2.1046	-2.8402	-1.10
18661	*TCTN3*	-0.44353	-1.1138	-1.7364	-2.1984	-1.10
18662	*GSR*	-0.52552	-0.97149	-1.8038	-1.8572	-1.10
18663	*TOMM22*	-0.03905	-1.4469	-1.8157	-2.0952	-1.10
18664	*INTS7*	-0.73577	-0.87898	-1.6952	-2.3086	-1.10
18665	*RPL15*	-0.87109	-0.94287	-1.4971	-2.0008	-1.10
18666	*SMARCAL1*	-0.38131	-1.1874	-1.7462	-2.7042	-1.10
18667	*CYP2A6*	-0.6568	-1.0099	-1.6524	-2.2951	-1.11
18668	*SQRDL*	-0.67853	-1.0793	-1.5635	-2.5243	-1.11
18669	*MRPL4*	-0.54537	-1.1387	-1.6373	-1.8404	-1.11

18670	*SNRNP200*	-0.96201	-0.97595	-1.386	-1.9041	-1.11
18671	*MFAP1*	-0.05613	-1.5294	-1.742	-2.1187	-1.11
18672	*VMP1*	-0.03972	-1.1325	-2.1572	-2.1908	-1.11
18673	*SRSF2*	-0.24984	-1.3294	-1.7512	-1.8076	-1.11
18674	*VPS33B*	-0.60754	-1.205	-1.5195	-2.537	-1.11
18675	*POLE3*	-0.55281	-0.88272	-1.8968	-1.9023	-1.11
18676	*KLRC1*	-0.63591	-1.1571	-1.5401	-2.2955	-1.11
18677	*POTEF*	-0.83656	-0.99508	-1.5105	-1.761	-1.11
18678	*C12orf75*	-0.85194	-0.91283	-1.5781	-2.796	-1.11
18679	*SACS*	-0.67573	-1.1967	-1.4741	-2.428	-1.12
18680	*MLEC*	-0.01367	-1.2071	-2.1264	-2.4696	-1.12
18681	*ARPC5*	-0.92329	-0.95775	-1.4683	-1.7266	-1.12
18682	*PI4KA*	0.058695	-1.6664	-1.7422	-2.6859	-1.12
18683	*B3GNT3*	-0.15161	-1.4233	-1.7766	-2.7277	-1.12
18684	*GALC*	-0.67458	-1.2968	-1.3818	-2.4481	-1.12
18685	*EFCC1*	-0.71717	-0.93725	-1.6989	-1.7845	-1.12
18686	*HRG*	-0.92486	-1.0699	-1.3597	-1.6561	-1.12
18687	*ST3GAL1*	0.50844	-1.5663	-2.2972	-2.4331	-1.12
18688	*ZBP1*	-1.0036	-1.1441	-1.2084	-1.6411	-1.12
18689	*NMUR1*	-0.31654	-1.4684	-1.5781	-1.9459	-1.12
18690	*RNF39*	-0.44353	-1.2977	-1.6288	-1.7994	-1.12
18691	*ABLIM1*	-1.0704	-1.0806	-1.2195	-1.3343	-1.12
18692	*METTL5*	-0.40641	-1.4361	-1.53	-1.5941	-1.12
18693	*F8A3*	-0.95321	-1.1105	-1.3111	-1.3287	-1.12
18694	*PLK1*	-0.73961	-1.1714	-1.468	-1.9624	-1.13
18695	*SPDYE2*	-0.45717	-1.3852	-1.5393	-1.6153	-1.13
18696	*SCN3A*	0.046808	-1.6731	-1.7575	-2.4709	-1.13
18697	*ATP6V1F*	-0.70442	-0.84763	-1.8341	-2.6923	-1.13
18698	*GRM4*	-1.0857	-1.1053	-1.1977	-1.2895	-1.13
18699	*ZNF792*	-0.93525	-1.065	-1.3892	-1.9686	-1.13
18700	*TMLHE*	-0.0923	-1.6158	-1.6836	-2.0146	-1.13
18701	*DPH3*	-0.60301	-0.77037	-2.0185	-2.2943	-1.13
18702	*TAB1*	0.17894	-1.7552	-1.8157	-1.9172	-1.13
18703	*POLL*	-0.95017	-1.174	-1.2691	-1.7829	-1.13
18704	*YIPF2*	-0.23176	-0.85018	-2.312	-2.5255	-1.13
18705	*HMHB1*	-0.74932	-1.0704	-1.5757	-2.0351	-1.13
18706	*USP17L18*	-0.84138	-1.2451	-1.3097	-2.1983	-1.13
18707	*MARCKS*	0.24592	-1.7865	-1.8557	-2.2866	-1.13
18708	*FAM218A*	-1.0564	-1.0782	-1.2623	-1.3878	-1.13
18709	*CEP97*	-0.85799	-1.0221	-1.517	-2.3888	-1.13
18710	*THOC2*	-1.0427	-1.1486	-1.2071	-1.565	-1.13
18711	*UFC1*	0.91834	-1.7056	-2.6113	-3.596	-1.13
18712	*PCDHB16*	-0.87128	-1.154	-1.3743	-1.855	-1.13
18713	*PPIL2*	-0.25603	-1.4689	-1.6748	-2.0061	-1.13
18714	*HBQ1*	-0.84841	-0.97595	-1.5765	-1.9708	-1.13
18715	*XRCC5*	0.030088	-1.6855	-1.7459	-1.8157	-1.13

18716	*C1orf216*	-0.53529	-1.3209	-1.547	-2.8506	-1.13
18717	*PRELP*	-0.93764	-1.0726	-1.3961	-2.1873	-1.14
18718	*ATAD2B*	0.68042	-1.4564	-2.6308	-2.7962	-1.14
18719	*RPL5*	-0.74719	-1.1479	-1.5189	-1.5258	-1.14
18720	*SF3A2*	-1.046	-1.1442	-1.226	-1.583	-1.14
18721	*PDCD11*	-0.26273	-1.1927	-1.9622	-2.0483	-1.14
18722	*H2AFB2*	-0.64076	-1.2817	-1.4969	-1.9227	-1.14
18723	*PWP2*	-0.69399	-1.1222	-1.6034	-2.2301	-1.14
18724	*WNT3*	-0.23418	-1.5524	-1.6339	-3.4438	-1.14
18725	*DONSON*	-0.7439	-1.3026	-1.3743	-1.4234	-1.14
18726	*AUP1*	-0.04646	-1.4141	-1.9622	-2.2951	-1.14
18727	*GPIHBP1*	-0.29886	-1.4016	-1.7235	-2.5184	-1.14
18728	*TMEM82*	-0.8139	-1.2973	-1.3132	-1.6635	-1.14
18729	*CMC1*	0.28263	-1.2079	-2.5007	-3.0644	-1.14
18730	*ADAM33*	-0.14736	-1.545	-1.7363	-1.7829	-1.14
18731	*PPIAL4G*	-0.45707	-1.4234	-1.5494	-2.0363	-1.14
18732	*NXN*	-1.0029	-1.1317	-1.3085	-1.8398	-1.15
18733	*C2orf47*	-0.40107	-1.4926	-1.55	-2.017	-1.15
18734	*DUSP13*	0.10318	-1.3756	-2.1739	-2.7689	-1.15
18735	*ADAMTS20*	-0.61675	-0.95545	-1.8751	-3.096	-1.15
18736	*FAM13A*	-0.64278	-0.98907	-1.8157	-2.9412	-1.15
18737	*TRRAP*	-0.73419	-1.3104	-1.4056	-1.9321	-1.15
18738	*TTK*	-0.98026	-1.0947	-1.3769	-2.1143	-1.15
18739	*MYCBP2*	-0.97499	-1.0107	-1.4684	-2.3342	-1.15
18740	*TIMELESS*	-0.37323	-1.539	-1.5425	-2.1227	-1.15
18741	*BCAM*	-0.76352	-1.274	-1.4197	-1.4213	-1.15
18742	*ALG11*	-0.32117	-1.3104	-1.8257	-2.0042	-1.15
18743	*MMP9*	-0.90635	-0.95717	-1.5959	-1.6682	-1.15
18744	*AHCY*	-1.0359	-1.1461	-1.2786	-2.203	-1.15
18745	*SOD1*	-0.74638	-0.95423	-1.7615	-2.7546	-1.15
18746	*FJX1*	-0.15148	-1.1944	-2.1224	-2.6376	-1.16
18747	*DICER1*	0.28451	-1.4275	-2.3287	-2.5934	-1.16
18748	*ME3*	-0.72882	-1.311	-1.4359	-1.7215	-1.16
18749	*RPF2*	0.03502	-1.2249	-2.2888	-2.6308	-1.16
18750	*MAF*	-0.48415	-1.1879	-1.8087	-2.4454	-1.16
18751	*MTRNR2L6*	-0.7288	-1.3399	-1.4142	-2.9834	-1.16
18752	*TBC1D5*	-0.60427	-1.2793	-1.6007	-1.8879	-1.16
18753	*NRBP1*	-0.19996	-1.2643	-2.0203	-2.4013	-1.16
18754	*APCDD1L*	0.29458	-1.7667	-2.0126	-2.095	-1.16
18755	*CATSPER4*	-0.69199	-1.0944	-1.6988	-2.5754	-1.16
18756	*TBC1D13*	-0.64571	-1.2277	-1.6128	-3.2172	-1.16
18757	*LMOD3*	-0.65435	-0.97667	-1.8561	-1.8591	-1.16
18758	*PRPF8*	0.31764	-1.8363	-1.9699	-2.5655	-1.16
18759	*FAM133B*	-0.2732	-1.5467	-1.6707	-2.2329	-1.16
18760	*NMNAT1*	-0.36057	-1.3132	-1.8212	-2.1984	-1.16
18761	*ARL10*	-0.52182	-0.93975	-2.0368	-2.0398	-1.17

18762	*COL20A1*	-1.0501	-1.2135	-1.2357	-1.7935	-1.17
18763	*NPIPB3*	-0.89828	-1.197	-1.4054	-1.6858	-1.17
18764	*RBBP4*	-0.99644	-1.0995	-1.4094	-2.1392	-1.17
18765	*PEBP1*	-0.41101	-0.92186	-2.1732	-2.7277	-1.17
18766	*MTX3*	-0.64398	-1.2958	-1.5726	-1.8975	-1.17
18767	*ATG7*	-0.49336	-1.3161	-1.7068	-1.8382	-1.17
18768	*FKBP5*	-0.59763	-1.2146	-1.7044	-3.1921	-1.17
18769	*PIK3R4*	-0.59008	-0.59886	-2.3287	-2.8702	-1.17
18770	*TMEM61*	-0.36948	-1.4852	-1.6649	-1.761	-1.17
18771	*ARAP3*	-0.43472	-1.0433	-2.0452	-2.2866	-1.17
18772	*CEP78*	-0.85296	-1.2623	-1.4103	-2.1046	-1.18
18773	*HAUS4*	-0.40646	-1.544	-1.5757	-2.2845	-1.18
18774	*SDHA*	-0.68374	-1.0475	-1.8087	-1.988	-1.18
18775	*SMIM10L1*	-0.82311	-1.2646	-1.4544	-3.8779	-1.18
18776	*DDX1*	-0.98864	-1.1437	-1.4125	-1.4856	-1.18
18777	*NDUFAF1*	-0.96661	-1.2572	-1.3249	-2.7277	-1.18
18778	*TUBA1B*	-0.27423	-1.4361	-1.8393	-2.1699	-1.18
18779	*PCSK2*	-0.46711	-1.5031	-1.5795	-1.7399	-1.18
18780	*GRB2*	-0.00331	-1.479	-2.0682	-2.4935	-1.18
18781	*MBD3L2*	-0.19423	-1.677	-1.6822	-1.7004	-1.18
18782	*SLX1B*	-0.75053	-1.3009	-1.5099	-1.7263	-1.19
18783	*GSPT1*	-0.48821	-1.4102	-1.6648	-2.6376	-1.19
18784	*GALR3*	-0.9703	-1.1898	-1.4036	-1.7235	-1.19
18785	*SF3A1*	-0.95321	-1.019	-1.5962	-2.0452	-1.19
18786	*FAM162B*	-0.92427	-1.2655	-1.379	-1.5765	-1.19
18787	*TATDN2*	-0.69502	-0.9997	-1.8792	-2.4394	-1.19
18788	*MIS18BP1*	-0.21532	-1.2961	-2.0629	-2.4696	-1.19
18789	*UCP3*	-0.5084	-1.5073	-1.5597	-2.7393	-1.19
18790	*CDRT15L2*	-0.65557	-1.1665	-1.7567	-2.9354	-1.19
18791	*RFC1*	-0.94255	-0.97768	-1.6617	-2.0995	-1.19
18792	*HDC*	-0.27961	-1.4834	-1.8199	-2.716	-1.19
18793	*NKIRAS2*	-0.19467	-1.3002	-2.0908	-2.3461	-1.20
18794	*C9orf9*	-0.73823	-1.12	-1.729	-2.1796	-1.20
18795	*CAPNS2*	-0.86047	-1.1429	-1.5856	-1.6526	-1.20
18796	*XYLT2*	-0.05348	-0.93293	-2.6075	-4.2681	-1.20
18797	*CDR2L*	-1.0134	-1.0145	-1.5661	-1.6731	-1.20
18798	*SETD8*	-0.51373	-0.92629	-2.1566	-2.5478	-1.20
18799	*DHX37*	-0.85388	-0.91807	-1.8282	-2.163	-1.20
18800	*TAF6*	-0.06667	-1.5304	-2.0042	-2.8071	-1.20
18801	*ZNF785*	-0.24887	-1.5795	-1.7745	-2.3615	-1.20
18802	*MANBAL*	-0.83184	-1.0765	-1.6964	-2.4268	-1.20
18803	*ITGB7*	-0.47107	-1.5508	-1.589	-1.6826	-1.20
18804	*CLPB*	-0.56411	-1.0019	-2.0466	-2.3729	-1.20
18805	*CCT4*	-0.74671	-1.0485	-1.8178	-1.8376	-1.20
18806	*SLMO2*	-0.31852	-1.522	-1.7734	-1.8908	-1.20
18807	*TLX1*	-0.83276	-1.2316	-1.5502	-1.5719	-1.20

18808	*SAR1B*	-0.40897	-1.4788	-1.7306	-2.412	-1.21
18809	*CLEC4F*	-0.32621	-1.4584	-1.8366	-2.5157	-1.21
18810	*JADE2*	-0.00049	-1.2935	-2.3282	-2.7546	-1.21
18811	*DDX47*	-0.57136	-1.5235	-1.5289	-2.7546	-1.21
18812	*C14orf80*	-0.79453	-1.4108	-1.4208	-1.5386	-1.21
18813	*PHC2*	-1.0564	-1.2662	-1.3053	-1.4544	-1.21
18814	*ARMCX2*	-0.25163	-1.4335	-1.9431	-2.1407	-1.21
18815	*CDK5RAP3*	-1.1169	-1.1587	-1.356	-2.712	-1.21
18816	*PPIAL4D*	-0.68518	-0.93368	-2.0146	-2.2811	-1.21
18817	*RHOD*	-0.28704	-1.3337	-2.0142	-2.0436	-1.21
18818	*IGF2*	-0.90508	-1.0922	-1.6527	-1.8038	-1.22
18819	*OR51G2*	0.32028	-1.1991	-2.7736	-2.8807	-1.22
18820	*MAP3K9*	-1.0481	-1.1944	-1.4109	-1.4467	-1.22
18821	*PIGF*	-0.85296	-1.3718	-1.4312	-1.72	-1.22
18822	*REEP2*	-0.71626	-1.4087	-1.5393	-2.0895	-1.22
18823	*MDM1*	-0.61663	-1.106	-1.9513	-3.6868	-1.22
18824	*KCNK15*	-0.73911	-1.3496	-1.5948	-1.984	-1.23
18825	*NR2C2AP*	-0.54409	-1.3836	-1.7613	-2.6635	-1.23
18826	*PSMD1*	-0.73155	-1.095	-1.8645	-1.9163	-1.23
18827	*IFIT1B*	-1.0599	-1.1756	-1.4566	-2.4312	-1.23
18828	*FPR1*	-0.21054	-0.54391	-2.9412	-3.0896	-1.23
18829	*LRTOMT*	-0.51304	-1.2163	-1.9748	-3.0028	-1.23
18830	*NSUN4*	-0.67906	-1.0096	-2.0161	-2.2117	-1.23
18831	*ATRAID*	-0.7679	-1.1747	-1.7658	-2.136	-1.24
18832	*SRSF7*	-1.0635	-1.122	-1.5264	-1.6112	-1.24
18833	*NDE1*	-0.36663	-1.4764	-1.8707	-1.8804	-1.24
18834	*C1orf56*	0.10483	-1.864	-1.9548	-2.4607	-1.24
18835	*RPAP2*	-0.38376	-1.267	-2.0653	-2.4946	-1.24
18836	*C6orf136*	-0.37929	-1.6218	-1.72	-2.0436	-1.24
18837	*C19orf52*	-0.92486	-1.1591	-1.6395	-1.773	-1.24
18838	*PSMB1*	-0.92642	-1.209	-1.5901	-2.3216	-1.24
18839	*USP8*	-0.95321	-1.2936	-1.4841	-2.2483	-1.24
18840	*ARHGEF40*	-0.83851	-1.2753	-1.6253	-2.1927	-1.25
18841	*KYNU*	-0.93064	-1.175	-1.6422	-2.4018	-1.25
18842	*ASAP2*	-0.64496	-0.70193	-2.4018	-2.4141	-1.25
18843	*HAUS3*	-0.51002	-1.4864	-1.7552	-2.0946	-1.25
18844	*PSMC4*	-0.90003	-1.4306	-1.4333	-1.6594	-1.25
18845	*KDELR1*	-0.39603	-1.1977	-2.1732	-2.337	-1.26
18846	*TBX5*	-0.9105	-0.92486	-1.9338	-2.6805	-1.26
18847	*CABP1*	-0.44353	-1.5671	-1.7591	-2.6201	-1.26
18848	*ATP6AP2*	-0.99175	-1.3148	-1.4647	-3.7232	-1.26
18849	*CA5B*	-0.64982	-0.73338	-2.391	-2.5663	-1.26
18850	*DNMT3A*	-0.7512	-1.367	-1.6589	-2.5008	-1.26
18851	*HMGN1*	-0.09079	-1.6635	-2.0249	-2.2254	-1.26
18852	*DOK1*	-0.54955	-1.4154	-1.8157	-1.8604	-1.26
18853	*HEPH*	-0.49311	-1.3465	-1.9431	-2.1108	-1.26

18854	*LSM3*	-0.56963	-1.3935	-1.8207	-1.8355	-1.26
18855	*QTRT1*	-0.29434	-1.6251	-1.8648	-2.1477	-1.26
18856	*ATN1*	-1.0999	-1.2708	-1.416	-1.5425	-1.26
18857	*RAPGEF1*	-0.61353	-1.4152	-1.7584	-1.9114	-1.26
18858	*RALGPS1*	-0.60801	-1.5302	-1.6525	-1.6934	-1.26
18859	*FBXL22*	-0.5546	-1.5603	-1.6759	-1.7911	-1.26
18860	*KPNB1*	-0.93975	-1.1752	-1.677	-2.1294	-1.26
18861	*ATP6AP1*	-1.0099	-1.2411	-1.5424	-1.9713	-1.26
18862	*PMPCA*	-1.136	-1.1596	-1.5013	-1.6989	-1.27
18863	*SEC22B*	-0.68934	-1.3127	-1.7994	-1.9763	-1.27
18864	*RNF19A*	-0.85565	-1.2399	-1.7065	-2.6635	-1.27
18865	*CYC1*	-0.73896	-0.94333	-2.1198	-2.8407	-1.27
18866	*NDUFA9*	-1.0761	-1.2529	-1.4741	-2.2254	-1.27
18867	*YPEL3*	-0.98158	-1.3453	-1.4764	-1.6065	-1.27
18868	*HS3ST4*	-0.29008	-1.5588	-1.9622	-2.0613	-1.27
18869	*RPL18*	-0.89593	-1.0688	-1.8478	-1.9938	-1.27
18870	*TRNT1*	-1.0097	-1.2934	-1.5138	-3.0502	-1.27
18871	*TMEM205*	-0.79711	-1.3627	-1.6597	-1.6648	-1.27
18872	*NANOG*	-1.1483	-1.3247	-1.3472	-2.2097	-1.27
18873	*PSRC1*	-0.87418	-1.344	-1.606	-2.0267	-1.27
18874	*HSPD1*	-0.59838	-1.4306	-1.7956	-2.1947	-1.27
18875	*LTBP3*	-1.053	-1.3393	-1.4368	-1.9375	-1.28
18876	*LENG8*	-0.95357	-1.2572	-1.62	-1.7845	-1.28
18877	*FKBP15*	-0.51847	-1.6336	-1.6804	-1.8207	-1.28
18878	*NIM1K*	-0.6373	-1.5741	-1.6241	-1.9404	-1.28
18879	*RANGAP1*	-0.86567	-1.3517	-1.6237	-1.6988	-1.28
18880	*PHF8*	-0.6714	-1.1442	-2.0303	-2.1105	-1.28
18881	*PHAX*	-1.2304	-1.2656	-1.3517	-1.6759	-1.28
18882	*DUSP9*	-0.59864	-1.2566	-1.994	-2.1094	-1.28
18883	*POLQ*	-0.72436	-0.93582	-2.1892	-3.556	-1.28
18884	*WDR3*	-1.2393	-1.2502	-1.364	-1.9162	-1.28
18885	*CTXN1*	-0.77255	-1.3836	-1.7018	-1.752	-1.29
18886	*GDF3*	-0.09403	-1.7845	-1.984	-2.0134	-1.29
18887	*C12orf77*	0.14348	-1.6963	-2.3101	-2.6593	-1.29
18888	*RBBP8*	-1.2264	-1.2837	-1.3636	-2.0398	-1.29
18889	*C1orf52*	-0.8865	-1.1653	-1.8224	-1.9582	-1.29
18890	*PSMB11*	0.43954	-1.4916	-2.8264	-2.9685	-1.29
18891	*DIS3*	-0.82208	-1.4878	-1.5715	-1.9734	-1.29
18892	*FAM69A*	-1.136	-1.3338	-1.4142	-1.988	-1.29
18893	*GHDC*	-0.04678	-1.7164	-2.1217	-3.1701	-1.29
18894	*GFM1*	-1.0004	-1.2632	-1.6332	-2.1219	-1.30
18895	*C9orf114*	-0.97138	-1.1426	-1.7836	-1.9291	-1.30
18896	*COPB2*	-1.2079	-1.3096	-1.3864	-1.7533	-1.30
18897	*ARPC3*	-0.94563	-1.217	-1.7434	-2.2794	-1.30
18898	*UQCRFS1*	-0.99833	-1.3892	-1.5194	-1.6891	-1.30
18899	*GINS2*	-0.81017	-1.4545	-1.6424	-1.8157	-1.30

18900	*USP3*	-1.0421	-1.3364	-1.5289	-1.6394	-1.30
18901	*JPH1*	-0.97252	-1.3916	-1.545	-1.5848	-1.30
18902	*PRMT5*	-0.97552	-1.2565	-1.6776	-1.6817	-1.30
18903	*SALL1*	-0.65181	-1.3291	-1.9326	-2.1532	-1.30
18904	*NAA40*	-1.0589	-1.1576	-1.7001	-2.5663	-1.31
18905	*METTL17*	0.20815	-1.626	-2.5005	-2.7884	-1.31
18906	*IL10RB*	-0.65345	-1.6105	-1.6571	-2.1113	-1.31
18907	*NOVA1*	-0.23511	-1.7908	-1.8975	-2.8718	-1.31
18908	*C1orf109*	-0.36861	-1.6401	-1.9165	-1.9722	-1.31
18909	*OGFR*	0.043666	-1.9573	-2.012	-2.0804	-1.31
18910	*EMC7*	-0.55528	-0.8703	-2.5008	-2.8229	-1.31
18911	*CTSC*	-0.71905	-1.3941	-1.8154	-1.8952	-1.31
18912	*ATHL1*	-1.0528	-1.3291	-1.5485	-2.1926	-1.31
18913	*LRRC73*	-1.2304	-1.258	-1.4448	-1.8642	-1.31
18914	*NRAS*	-0.26347	-1.7871	-1.8892	-2.2167	-1.31
18915	*MTPAP*	-1.2226	-1.3008	-1.4168	-3.0319	-1.31
18916	*MIPEP*	0.21805	-2.072	-2.0895	-5.6631	-1.31
18917	*BCL2L13*	-0.86912	-1.4218	-1.6526	-2.0804	-1.31
18918	*ZNF492*	-1.2421	-1.2646	-1.4392	-2.3488	-1.32
18919	*PPL*	0.063013	-1.7125	-2.3017	-3.447	-1.32
18920	*DMRTA2*	-0.42075	-1.3371	-2.1984	-2.3696	-1.32
18921	*RPS27*	-0.11717	-1.8792	-1.9623	-2.1498	-1.32
18922	*EBNA1BP2*	-0.12446	-1.5059	-2.3292	-2.6549	-1.32
18923	*ANP32A*	-0.41359	-1.6288	-1.9179	-2.0423	-1.32
18924	*PYGL*	-0.37215	-1.4454	-2.1478	-2.3452	-1.32
18925	*CCND3*	-1.0853	-1.3542	-1.5264	-1.6746	-1.32
18926	*SLU7*	-0.29921	-1.7662	-1.9073	-2.3025	-1.32
18927	*TUFT1*	0.19462	-1.7493	-2.4205	-3.3217	-1.33
18928	*PTF1A*	-0.73591	-1.2371	-2.0042	-2.0778	-1.33
18929	*HNF4A*	-1.0551	-1.0889	-1.8405	-2.4337	-1.33
18930	*C16orf72*	-0.99448	-1.1391	-1.8557	-2.0452	-1.33
18931	*NAA15*	-0.26057	-1.6865	-2.0466	-2.2812	-1.33
18932	*PLAT*	-0.99906	-1.3711	-1.6241	-1.6268	-1.33
18933	*NOM1*	-1.1571	-1.4061	-1.4325	-2.1419	-1.33
18934	*MRPL19*	-1.067	-1.4243	-1.5118	-2.4933	-1.33
18935	*NOL6*	-1.0211	-1.2784	-1.7052	-1.7306	-1.33
18936	*KIF4A*	-1.1034	-1.4168	-1.4846	-1.7001	-1.33
18937	*ZNF587B*	-0.97768	-1.0582	-1.9716	-2.0104	-1.34
18938	*PSMB7*	-0.98634	-1.5118	-1.5153	-1.9203	-1.34
18939	*ZFP36*	-0.73061	-1.4428	-1.8406	-4.129	-1.34
18940	*EPSTI1*	0.052562	-1.1442	-2.9236	-3.0687	-1.34
18941	*P2RY14*	0.48815	-2.0771	-2.4268	-5.8637	-1.34
18942	*BIRC5*	-1.1995	-1.305	-1.5125	-2.4946	-1.34
18943	*PGRMC2*	-0.82757	-1.4544	-1.738	-2.1334	-1.34
18944	*OSM*	-0.91823	-1.1021	-1.9997	-2.2264	-1.34
18945	*UTP14A*	-0.78444	-1.2623	-1.9738	-2.0885	-1.34

18946	*SCARB2*	-0.97549	-1.5059	-1.5404	-1.7512	-1.34
18947	*RNF113A*	-1.0822	-1.2571	-1.6852	-1.7752	-1.34
18948	*G3BP1*	-1.0204	-1.0405	-1.9656	-2.1198	-1.34
18949	*NUDT21*	-0.50911	-1.6571	-1.8604	-2.4333	-1.34
18950	*NR5A2*	-1.141	-1.4306	-1.4553	-2.1507	-1.34
18951	*OXA1L*	-0.92829	-1.3472	-1.7745	-2.8436	-1.35
18952	*PHIP*	-1.154	-1.2985	-1.6023	-2.0337	-1.35
18953	*TIMM10*	-0.85059	-1.5243	-1.6803	-2.8019	-1.35
18954	*INPP5A*	-0.70475	-1.1338	-2.2172	-2.7941	-1.35
18955	*C8orf31*	-0.6373	-1.284	-2.1351	-2.4904	-1.35
18956	*RPS26*	-0.89513	-1.279	-1.8879	-3.1568	-1.35
18957	*GPC5*	-1.1566	-1.4544	-1.4686	-2.0042	-1.36
18958	*TRAP1*	-0.771	-1.5512	-1.7577	-2.2692	-1.36
18959	*MARK2*	-0.21487	-1.649	-2.217	-2.9834	-1.36
18960	*IPO5*	-1.1317	-1.4263	-1.5235	-1.8775	-1.36
18961	*ZNF114*	-0.61599	-1.3926	-2.075	-2.7863	-1.36
18962	*PTPMT1*	-1.1249	-1.2432	-1.7234	-1.7545	-1.36
18963	*FBL*	-0.9229	-1.5824	-1.5882	-2.1669	-1.36
18964	*SETD9*	-0.91738	-1.5978	-1.6072	-1.639	-1.37
18965	*RFPL4AL1*	-1.0685	-1.445	-1.6129	-2.9736	-1.38
18966	*ACSM1*	-1.151	-1.4893	-1.4935	-1.7552	-1.38
18967	*MED30*	-1.1555	-1.2895	-1.7052	-1.8387	-1.38
18968	*DHX8*	-0.97788	-1.5225	-1.6524	-3.6181	-1.38
18969	*GEMIN5*	-0.9925	-1.2457	-1.9227	-1.9404	-1.39
18970	*SRP9*	-0.87302	-1.5568	-1.7364	-2.1589	-1.39
18971	*RPS7*	-0.68003	-1.4249	-2.0634	-2.2165	-1.39
18972	*HJURP*	-0.6603	-1.6836	-1.8264	-2.6072	-1.39
18973	*MYEOV2*	-1.1316	-1.2867	-1.7571	-2.0384	-1.39
18974	*GLYAT*	-0.18986	-1.8995	-2.0949	-2.3357	-1.39
18975	*ID2*	-0.182	-1.8742	-2.1305	-2.1794	-1.40
18976	*RPS14*	-1.1177	-1.1189	-1.9548	-1.962	-1.40
18977	*SF3B2*	-0.87647	-1.3954	-1.9196	-2.0677	-1.40
18978	*TRPC7*	-0.95579	-1.5634	-1.6776	-2.5298	-1.40
18979	*ZSCAN22*	-0.43219	-1.7829	-1.984	-2.2779	-1.40
18980	*RPS11*	-1.2665	-1.4089	-1.5284	-2.6431	-1.40
18981	*NMUR2*	-1.0402	-1.5637	-1.6014	-2.9564	-1.40
18982	*RBM24*	-1.0237	-1.183	-2.0134	-2.9296	-1.41
18983	*NDUFA13*	-0.86142	-1.6213	-1.7505	-2.1264	-1.41
18984	*NLE1*	-1.3751	-1.3973	-1.4689	-2.2895	-1.41
18985	*ADAMTS14*	-0.93188	-1.3875	-1.9375	-2.3849	-1.42
18986	*FBXO43*	-1.0139	-1.5647	-1.6975	-1.754	-1.43
18987	*TMEM238*	-0.52712	-1.7001	-2.0586	-2.1873	-1.43
18988	*SBNO1*	-0.51187	-1.7322	-2.0452	-2.1709	-1.43
18989	*VPS16*	-0.98771	-1.333	-1.9695	-2.0069	-1.43
18990	*DDAH1*	0.73626	-1.8636	-3.1665	-3.7932	-1.43
18991	*MRPL28*	-0.57484	-1.5357	-2.1888	-2.4456	-1.43

18992	*ATAD3C*	0.26822	-2.2607	-2.3086	-2.3623	-1.43
18993	*ATXN7L2*	-1.1988	-1.2975	-1.8157	-2.796	-1.44
18994	*SGMS1*	-1.2099	-1.4682	-1.6386	-1.7924	-1.44
18995	*HCCS*	-1.2296	-1.4215	-1.6703	-1.7795	-1.44
18996	*PSMD11*	-1.2686	-1.4946	-1.5635	-1.6858	-1.44
18997	*WDR1*	-0.35161	-1.828	-2.1604	-2.3017	-1.45
18998	*PABPC1L*	-1.1739	-1.3343	-1.8398	-2.0949	-1.45
18999	*PRIM1*	-1.4376	-1.4554	-1.4578	-1.7137	-1.45
19000	*PPP1R9B*	-0.56258	-1.7554	-2.0337	-2.5228	-1.45
19001	*RALB*	-1.0999	-1.5576	-1.6951	-2.2381	-1.45
19002	*PGM3*	-1.2009	-1.3878	-1.773	-1.8154	-1.45
19003	*POLD2*	-1.1619	-1.4027	-1.8157	-3.822	-1.46
19004	*CFAP99*	-0.82669	-1.5789	-1.985	-2.2859	-1.46
19005	*HIST1H3B*	-1.2118	-1.2756	-1.9041	-2.1187	-1.46
19006	*ANKRD46*	-1.0633	-1.6072	-1.7219	-2.3623	-1.46
19007	*YIF1A*	-0.69724	-1.7188	-1.9848	-2.6267	-1.47
19008	*ATP6V1B2*	-1.1124	-1.3074	-1.9811	-2.3174	-1.47
19009	*ANAPC1*	-1.2783	-1.4858	-1.6374	-1.7169	-1.47
19010	*EAF1*	-1.4036	-1.4325	-1.5687	-2.5198	-1.47
19011	*NFATC2IP*	-1.1287	-1.5744	-1.7105	-2.4518	-1.47
19012	*HIST1H2BL*	-1.1247	-1.6105	-1.6796	-1.8714	-1.47
19013	*CD300LB*	-1.2915	-1.4336	-1.6952	-3.2172	-1.47
19014	*TXNDC5*	-0.28058	-1.9938	-2.1732	-2.6652	-1.48
19015	*SBDS*	-1.1999	-1.3748	-1.8777	-2.0436	-1.48
19016	*TMTC1*	-1.2629	-1.5011	-1.6989	-2.4422	-1.49
19017	*PLA2G12B*	-0.93217	-1.6799	-1.853	-2.3349	-1.49
19018	*TMEM42*	-0.66146	-1.7364	-2.0791	-2.6377	-1.49
19019	*COPS5*	-0.66353	-1.8223	-2.0197	-2.4642	-1.50
19020	*ELMOD3*	-1.4082	-1.5249	-1.5959	-2.7379	-1.51
19021	*AGAP9*	-0.78362	-1.2193	-2.5292	-2.6122	-1.51
19022	*NDUFAB1*	-1.4633	-1.5073	-1.5634	-2.1239	-1.51
19023	*PYDC1*	-0.88438	-1.6356	-2.0351	-2.2167	-1.52
19024	*IL22RA1*	-0.94536	-1.7068	-1.9112	-2.0042	-1.52
19025	*NODAL*	0.20677	-2.3696	-2.4172	-2.6753	-1.53
19026	*NCKAP5L*	-0.94954	-1.7882	-1.843	-2.258	-1.53
19027	*TPTE2*	-1.2502	-1.5194	-1.8157	-2.1796	-1.53
19028	*HEATR3*	-0.66026	-1.8702	-2.0661	-2.2343	-1.53
19029	*SCO1*	0.1753	-2.1926	-2.6024	-3.5394	-1.54
19030	*DROSHA*	-1.3517	-1.4183	-1.8508	-2.1532	-1.54
19031	*KDM3A*	-0.44056	-1.7874	-2.3949	-4.0266	-1.54
19032	*SAP30BP*	-0.86479	-1.8613	-1.8975	-1.9811	-1.54
19033	*GLRX5*	-1.3093	-1.5954	-1.7219	-1.8157	-1.54
19034	*SRP68*	-1.1242	-1.5781	-1.9341	-2.2599	-1.55
19035	*CENPK*	-0.88094	-1.7526	-2.0042	-2.0436	-1.55
19036	*RPL36A-HNRNPH2*	-1.4258	-1.5204	-1.7257	-2.0818	-1.56
19037	*CATIP*	-1.2215	-1.6411	-1.8207	-2.0871	-1.56

19038	*HAUS8*	-1.1637	-1.416	-2.1108	-2.781	-1.56
19039	*PET117*	-1.2145	-1.6648	-1.8118	-2.0466	-1.56
19040	*CDC7*	-1.3543	-1.4523	-1.8845	-2.6208	-1.56
19041	*SUPT4H1*	-0.27587	-1.9172	-2.5057	-2.7863	-1.57
19042	*GAPDH*	-1.1587	-1.72	-1.8202	-2.2094	-1.57
19043	*SLC16A10*	-0.27453	-2.1893	-2.2413	-4.1443	-1.57
19044	*FBXO3*	-1.3554	-1.5752	-1.7766	-2.1179	-1.57
19045	*TICRR*	-1.4649	-1.5988	-1.6447	-1.9002	-1.57
19046	*TAF1*	-1.017	-1.3165	-2.3811	-3.0246	-1.57
19047	*RPL12*	-1.1524	-1.7332	-1.8297	-2.7127	-1.57
19048	*WAPAL*	-1.0024	-1.7599	-1.9713	-2.412	-1.58
19049	*PHKG2*	-1.311	-1.6613	-1.7779	-1.7905	-1.58
19050	*FCN1*	-1.5544	-1.6065	-1.6206	-3.0124	-1.59
19051	*PRKAG1*	-1.183	-1.6216	-1.9912	-2.0301	-1.60
19052	*SCTR*	-0.60658	-1.4728	-2.7221	-2.8636	-1.60
19053	*ALS2CR12*	-0.70959	-1.6707	-2.425	-3.2348	-1.60
19054	*DNM1L*	-1.5225	-1.6251	-1.6691	-1.762	-1.61
19055	*LMTK3*	-1.3814	-1.6047	-1.8366	-2.3876	-1.61
19056	*TRIM42*	-0.09284	-2.2343	-2.501	-3.4328	-1.61
19057	*MORN1*	-0.59061	-2.0249	-2.2165	-2.9308	-1.61
19058	*MSH6*	-0.72553	-0.86841	-3.2501	-3.454	-1.61
19059	*RPS8*	-1.3291	-1.622	-1.9199	-2.7991	-1.62
19060	*ADCY7*	-0.54414	-1.8952	-2.4422	-2.5146	-1.63
19061	*CHTF18*	-0.70993	-1.9673	-2.2094	-2.3936	-1.63
19062	*LRRC45*	-1.1904	-1.7068	-1.9912	-2.4322	-1.63
19063	*PGLS*	-1.1605	-1.7911	-1.9474	-1.9912	-1.63
19064	*COMMD7*	-1.1415	-1.864	-1.9013	-3.1211	-1.64
19065	*UBA52*	-1.1081	-1.7709	-2.0293	-3.051	-1.64
19066	*LOC101060321*	-0.99694	-1.9207	-1.9912	-2.6536	-1.64
19067	*DKC1*	-1.2502	-1.7507	-1.9269	-2.2964	-1.64
19068	*MCM4*	-0.68616	-1.9431	-2.3282	-2.6113	-1.65
19069	*BECN2*	-1.0809	-1.8376	-2.0466	-2.4394	-1.66
19070	*IFT46*	-1.4881	-1.7037	-1.7741	-2.2223	-1.66
19071	*CNPY2*	-0.76856	-1.2411	-2.9564	-4.0005	-1.66
19072	*AGPAT1*	-0.98574	-1.8112	-2.1848	-2.1936	-1.66
19073	*DDX49*	-1.3795	-1.7024	-1.9089	-2.6374	-1.66
19074	*TSG101*	-1.4572	-1.6565	-1.8929	-1.9968	-1.67
19075	*PKMYT1*	-1.4773	-1.7662	-1.7766	-1.8085	-1.67
19076	*XRN2*	-1.4306	-1.6785	-1.9237	-2.3729	-1.68
19077	*CFC1B*	-1.4448	-1.5552	-2.0343	-2.201	-1.68
19078	*HIST2H3D*	-0.46155	-1.4998	-3.0779	-3.7736	-1.68
19079	*TAF2*	-1.0589	-1.8908	-2.1305	-2.1802	-1.69
19080	*MRPL46*	-1.4633	-1.6072	-2.0265	-2.4472	-1.70
19081	*SPC24*	-1.3711	-1.7865	-1.94	-3.1117	-1.70
19082	*NLGN2*	0.19631	-2.5546	-2.7427	-2.8628	-1.70
19083	*TBC1D3L*	-1.2383	-1.6178	-2.2507	-2.6609	-1.70

19084	*NEUROG3*	-1.3785	-1.5588	-2.1796	-6.4493	-1.71
19085	*SLC11A1*	-1.3151	-1.8653	-1.9372	-2.1541	-1.71
19086	*RPL30*	-1.4681	-1.5715	-2.0851	-2.2771	-1.71
19087	*MRPS36*	-0.94747	-1.9846	-2.201	-2.2343	-1.71
19088	*PMS1*	-1.5082	-1.7688	-1.8623	-1.912	-1.71
19089	*RPS27L*	-1.6262	-1.7338	-1.7804	-1.855	-1.71
19090	*FGD4*	-1.2021	-1.7407	-2.2169	-2.5478	-1.72
19091	*HDLBP*	-0.69399	-1.7552	-2.7317	-2.9283	-1.73
19092	*CPNE6*	-0.84419	-1.5212	-2.8574	-2.9438	-1.74
19093	*PFDN2*	-0.36723	-2.2536	-2.6135	-3.2616	-1.74
19094	*TIPIN*	-1.3711	-1.6826	-2.1921	-2.2771	-1.75
19095	*MYL7*	-0.96173	-1.8909	-2.4112	-2.9081	-1.75
19096	*BHMT*	-1.3599	-1.8881	-2.0181	-2.3488	-1.76
19097	*UBE2R2*	-0.58212	-2.0712	-2.6157	-2.7393	-1.76
19098	*TOX2*	-1.2071	-1.7702	-2.3349	-2.6003	-1.77
19099	*NEURL1*	-1.7282	-1.7364	-1.8698	-2.1566	-1.78
19100	*WRAP53*	-0.696	-2.2955	-2.3534	-2.5477	-1.78
19101	*EGR4*	-0.51683	-2.0697	-2.7849	-3.1119	-1.79
19102	*SPDYC*	-1.88006	-1.547	-1.9686		-1.80
19103	*RPL27*	-1.0946	-2.1351	-2.1749	-2.3678	-1.80
19104	*RPE*	-1.2302	-1.7115	-2.5298	-3.5378	-1.82
19105	*TRIT1*	-1.7253	-1.7991	-1.9511	-1.9678	-1.83
19106	*URB2*	-1.2531	-1.5194	-2.7063	-4.0822	-1.83
19107	*ZNF623*	-1.4067	-1.9495	-2.1685	-2.4933	-1.84
19108	*RNF148*	-0.8514	-1.8157	-2.8922	-3.2735	-1.85
19109	*PPP6C*	-1.2318	-2.1566	-2.1798	-2.3086	-1.86
19110	*NOP56*	-1.7412	-1.8098	-2.0347	-2.7127	-1.86
19111	*GDE1*	-1.6597	-1.9251	-2.011	-3.051	-1.87
19112	*ATG12*	-1.4893	-1.9997	-2.163	-2.6267	-1.88
19113	*WTIP*	-1.4191	-1.7215	-2.6889	-4.0424	-1.94
19114	*RELL2*	-1.6926	-2.4015	-2.8995	-5.5005	-2.33

Supplementary Table 2. List of endocytosis-related genes ranked by the average of the three most enriched sgRNAs. Each gene was targeted by four single guiding RNA (sgRNA). Genes were ranked by the average of the three most enriched sgRNA.

Rank	*Gene*	Log. Fold Change of sgRNAs				*Average 3 most enriched*
		1st	2nd	3rd	4th	
29	*TAGLN*	6.237	3.6013	3.2889	-0.27744	*4.375733333*
210	*FNBP1L*	4.7845	4.4528	1.9014	-1.3291	*3.7129*
342	*DYNLT1*	4.0375	3.9498	2.5672	-0.51673	*3.518166667*
369	*WAS*	3.9411	3.684	2.8318	-1.8157	*3.485633333*
376	*ARPC1B*	4.9398	4.5636	0.9361	0.67857	*3.479833333*
703	*CLTB*	4.7656	4.4864	0.29654	0.11372	*3.182846667*
727	*CORO1A*	4.7969	3.9305	0.78273	-1.3153	*3.170043333*
1037	*EPS15L1*	4.764	2.7912	1.3561	1.1738	*2.970433333*
1276	*WIPF1*	5.3792	3.8415	-0.70193	-1.9099	*2.83959*
1482	*TCTEX1D4*	5.4376	2.7664	0.023338	-1.3139	*2.742446*
1545	*AMPH*	4.8977	1.9093	1.3351	-1.5095	*2.714033333*
1595	*CORO1B*	4.453	3.9732	-0.35367	-1.3161	*2.690843333*
2088	*SH3KBP1*	4.1292	3.0958	0.25951	-0.49028	*2.494836667*
2313	*PICALM*	4.8185	1.8296	0.6088	-1.1105	*2.418966667*
2338	*TWF2*	3.3152	2.1061	1.8107	-1.0106	*2.410666667*
2419	*CFL1*	3.0905	2.3667	1.6951	-1.5402	*2.3841*
2790	*CAP2*	3.4496	3.22	0.13093	-0.42195	*2.266843333*
3326	*PSTPIP2*	3.9571	1.7151	0.66965	-0.55699	*2.11395*
3481	*ITSN1*	3.9755	1.9877	0.25016	-0.44713	*2.07112*
4123	*TCTEX1D1*	3.4506	1.9289	0.33545	0.016531	*1.904983333*
4226	*CAPZA2*	4.4102	1.153	0.089595	-0.78588	*1.884265*
4386	*PLS3*	3.6316	1.7694	0.14607	-0.73546	*1.849023333*
4586	*GMFG*	3.0507	2.5399	-0.19984	-0.88684	*1.79692*
4615	*SNAP91*	3.9105	1.2668	0.18868	-2.0953	*1.78866*
4662	*CLTA*	3.2583	1.0428	1.0357	0.33194	*1.778933333*
4848	*MYO1F*	5.2218	0.85335	-0.84337	-1.4287	*1.743926667*
4936	*HIP1R*	4.0042	1.5834	-0.4119	-0.67608	*1.725233333*
5033	*EPN3*	5.0785	1.1417	-1.1098	-1.1793	*1.703466667*
5110	*PFN4*	2.4943	1.8352	0.73229	-0.12228	*1.687263333*
5184	*EPN1*	4.3182	0.82008	-0.11216	-0.83856	*1.675373333*
5416	*EPN2*	4.3049	1.2683	-0.68909	-2.071	*1.628036667*
5449	*TOM1L1*	2.3217	1.3165	1.2227	0.49083	*1.6203*
5682	*ACTG2*	2.4312	1.4911	0.79352	-0.61595	*1.57194*
5851	*ACTR3B*	3.6542	0.97636	-0.00822	-0.9169	*1.540779033*
5903	*PSTPIP1*	3.7016	0.70846	0.18579	-0.84136	*1.53195*
6151	*ACTG1*	3.543	1.3579	-0.4521	-0.8934	*1.482933333*
6161	*AP1B1*	3.8933	0.29458	0.25604	-0.97477	*1.481306667*
6306	*SYNJ1*	5.2149	0.5653	-1.4104	-1.5781	*1.4566*
6601	*TRIP10*	2.8578	1.4816	-0.11939	-1.4824	*1.40667*
6707	*LCP1*	3.5813	0.51261	0.059634	-0.57542	*1.384514667*

7007	*ACAT2*	1.5162	1.2603	1.2209	-2.3949	*1.332466667*
7291	*CAP1*	5.3058	-0.11854	-1.3271	-1.3464	*1.28672*
7316	*HIP1*	4.1992	0.35716	-0.7131	-0.79813	*1.281086667*
7576	*CALM1*	1.5327	1.3695	0.7883	-0.21814	*1.230166667*
7831	*CAPZA1*	2.0375	0.95152	0.57307	-0.39998	*1.187363333*
8063	*DNM1*	2.9349	0.46691	0.052818	-0.10031	*1.151542667*
8240	*ENTHD1*	3.3437	0.3533	-0.34014	-1.7495	*1.118953333*
8310	*TOM1*	1.7547	1.5397	0.02597	-0.2372	*1.10679*
8330	*WIPF2*	3.0434	0.50218	-0.23418	-0.87395	*1.1038*
8943	*AP2A1*	1.5899	1.0944	0.34843	0.12788	*1.01091*
8945	*BIN2*	3.5072	0.72975	-1.2051	-1.4141	*1.010616667*
9358	*DBNL*	3.7336	0.47677	-1.379	-1.8102	*0.94379*
9630	*BIN3*	3.3333	-0.0248	-0.60604	-1.5268	*0.900819333*
9961	*CLTCL1*	3.7542	-0.12995	-1.084	-2.7941	*0.84675*
9978	*BMP2K*	1.2865	0.7839	0.46044	-0.34451	*0.843613333*
10240	*TOM1L2*	2.498	0.07269	-0.1815	-1.5862	*0.796395*
10417	*ARPC1A*	3.9292	-0.41967	-1.2141	-1.3199	*0.765143333*
10972	*DSTN*	1.4704	0.32956	0.2058	-0.78444	*0.668586667*
11003	*SGIP1*	1.8297	1.0774	-0.92087	-1.6105	*0.662076667*
11128	*ARPC2*	1.1498	0.46726	0.30819	-0.77997	*0.64175*
11436	*CLTC*	1.1451	0.51436	0.098319	-0.49941	*0.585926333*
12532	*TWF1*	3.1933	0.51644	-2.5442	-2.9987	*0.388513333*
12537	*GMFB*	0.62056	0.29607	0.24612	-1.3635	*0.387583333*
12583	*EPS15*	1.8058	0.62639	-1.2912	-1.301	*0.38033*
12831	*CAPG*	1.1342	0.71227	-0.83276	-1.7802	*0.337903333*
13429	*MYO1E*	0.59299	0.34589	-0.2391	-0.97381	*0.23326*
13433	*AP2A2*	0.86619	0.00367	-0.17261	-1.2284	*0.2324169*
13504	*AP2B1*	1.401	0.06216	-0.80713	-0.92086	*0.218675*
13520	*CALM3*	1.2434	-0.0566	-0.53619	-1.0019	*0.21687*
13555	*FNBP1*	1.1508	-0.02433	-0.49275	-1.0863	*0.211240333*
13738	*TCTE3*	0.77639	0.63802	-0.89977	-1.7999	*0.171546667*
13846	*ARPC4*	0.44646	0.19552	-0.17979	-1.1986	*0.154063333*
13874	*CORO1C*	1.3871	-0.45339	-0.48336	-0.77227	*0.150116667*
13918	*FCHO1*	1.1713	0.56444	-1.3104	-2.0629	*0.14178*
14781	*BIN1*	1.3259	-0.21438	-1.1511	-1.1817	*-0.013193333*
14813	*SYNJ2*	1.0963	-0.25792	-0.89641	-1.6437	*-0.019343333*
14845	*FCHO2*	0.58849	0.46699	-1.1309	-1.3668	*-0.02514*
15075	*ACTR3C*	0.5122	-0.30811	-0.41596	-1.7829	*-0.070623333*
15161	*CAPZB*	0.43295	0.40361	-1.0952	-1.6239	*-0.086213333*
15162	*SH3YL1*	2.0036	-1.0707	-1.192	-1.5601	*-0.086366667*
15484	*TAGLN3*	2.1068	-1.0293	-1.5167	-1.7323	*-0.1464*
15847	*NCKIPSD*	-0.034913	-0.15626	-0.46705	-1.5306	*-0.219407667*
16022	*AAK1*	0.73215	-0.63152	-0.85536	-1.5598	*-0.251576667*
16155	*WASL*	-0.015041	-0.20103	-0.63572	-1.584	*-0.283930333*
16350	*ITSN2*	0.20545	-0.37888	-0.80946	-1.0773	*-0.32763*
16396	*AP2M1*	2.0497	-1.485	-1.5787	-1.6443	*-0.338*

16529	*ACTA1*	0.59964	0.04475	-1.7567	-1.9125	*-0.370771333*
16541	*ARPC5L*	0.30141	-0.48042	-0.94493	-1.5744	*-0.374646667*
16548	*ACTC1*	0.32666	-0.67889	-0.7771	-1.3292	*-0.376443333*
17078	*CFL2*	-0.17731	-0.50087	-0.84426	-1.4233	*-0.50748*
17113	*GRAP*	0.15694	-0.74875	-0.96144	-2.201	*-0.51775*
17266	*AP3B1*	0.098257	-0.29186	-1.4873	-1.9686	*-0.560301*
17433	*ACTR2*	0.28933	-0.70708	-1.3995	-1.9605	*-0.60575*
17639	*ACTR3*	-0.38198	-0.62969	-0.97565	-1.6208	*-0.66244*
17837	*TCTEX1D2*	-0.26714	-0.95149	-0.96718	-1.7018	*-0.728603333*
17947	*ACTB*	-0.051097	-0.91874	-1.3199	-2.0734	*-0.763245667*
18069	*ACTA2*	-0.33821	-0.92375	-1.151	-2.0059	*-0.80432*
18404	*PLS1*	0.0044681	-0.90206	-1.9474	-2.0223	*-0.948330633*
18420	*AP2S1*	-0.79367	-0.87989	-1.1973	-1.5589	*-0.956953333*
18646	*TAGLN2*	-0.33213	-0.97549	-1.9624	-6.2107	*-1.090006667*
18681	*ARPC5*	-0.92329	-0.95775	-1.4683	-1.7266	*-1.116446667*
18780	*GRB2*	-0.003309	-1.479	-2.0682	-2.4935	*-1.183503067*
18897	*ARPC3*	-0.94563	-1.217	-1.7434	-2.2794	*-1.30201*
18997	*WDR1*	-0.35161	-1.828	-2.1604	-2.3017	*-1.44667*
19054	*DNM1L*	-1.5225	-1.6251	-1.6691	-1.762	*-1.605566667*

Supplementary Table 3. List of single guiding RNAs used to generate stable knockouts for each individual candidate gene identified in the CRISPR/Cas9 screen. Targeted sequences on the gene are shown in the middle column.

Gene symbol	Target Sequence	ABM Cat. No.
Negative control	GCACTCACATCGCTACATCA	K093
LDLR	GTGGCAGCGAAACTCGTCCT	K1205924
TAGLN	CACTTCGCGGCTCATGCCAT	K2330124
DOK4	AAGATGAAGAGCAGGAAGCT	K0625224
PAQR8	GCTCAGGCGCTCCAAGATGG	K1593824
PLAA	ACGGACACAAAGGCTCCCGG	K1660724
USP44	ATGTACGTCGTAGTAACTTC	K2601324
BBS12	TCAACAACTTTCATCATTCG	K0172724
RINL	GGAAGTGGACCATCCTGGGC	K1825324
CRIM1	CGAGGGTTATGCTCCTCCTG	K0507224
SLC48A1	CTACTCCGGCATCAGCTCCG	K2192924
C4orf33	TGGATTCAGCCTGATAAATA	K0236924
PBRM1	AAAGTCCCCGCTGACACTGC	K1599924
STAB1	TGTCCATGAGCGGCTGCAGA	K2297624
SYNRG	GGAAATCCTTGTTGCTGCAT	K2809424
MUC17	TGTAGATGTTGTACCTGTGG	K16367924

Single guiding RNA were purchased from Applied Biological Materials Inc (ABM).

Supplementary Table 4: List of primer kits used for RT-qPCR

Gene	Primer ID
LDLR	Hs01092524_m1
HMGCR	Hs00168352_m1
SREBPF2	Hs01081784_m1
MVK	Hs00176077_m1
SREBF1	Hs01088679_g1
SCAP	Hs00378725_m1
SQLE	Hs01123768_m1
18S	Hs03003631_g1
